# Exploring UK medical school differences: the *MedDifs* study of selection, teaching, student and F1 perceptions, postgraduate outcomes and fitness to practise

**DOI:** 10.1186/s12916-020-01572-3

**Published:** 2020-05-14

**Authors:** I. C. McManus, Andrew Christopher Harborne, Hugo Layard Horsfall, Tobin Joseph, Daniel T. Smith, Tess Marshall-Andon, Ryan Samuels, Joshua William Kearsley, Nadine Abbas, Hassan Baig, Joseph Beecham, Natasha Benons, Charlie Caird, Ryan Clark, Thomas Cope, James Coultas, Luke Debenham, Sarah Douglas, Jack Eldridge, Thomas Hughes-Gooding, Agnieszka Jakubowska, Oliver Jones, Eve Lancaster, Calum MacMillan, Ross McAllister, Wassim Merzougui, Ben Phillips, Simon Phillips, Omar Risk, Adam Sage, Aisha Sooltangos, Robert Spencer, Roxanne Tajbakhsh, Oluseyi Adesalu, Ivan Aganin, Ammar Ahmed, Katherine Aiken, Alimatu-Sadia Akeredolu, Ibrahim Alam, Aamna Ali, Richard Anderson, Jia Jun Ang, Fady Sameh Anis, Sonam Aojula, Catherine Arthur, Alena Ashby, Ahmed Ashraf, Emma Aspinall, Mark Awad, Abdul-Muiz Azri Yahaya, Shreya Badhrinarayanan, Soham Bandyopadhyay, Sam Barnes, Daisy Bassey-Duke, Charlotte Boreham, Rebecca Braine, Joseph Brandreth, Zoe Carrington, Zoe Cashin, Shaunak Chatterjee, Mehar Chawla, Chung Shen Chean, Chris Clements, Richard Clough, Jessica Coulthurst, Liam Curry, Vinnie Christine Daniels, Simon Davies, Rebecca Davis, Hanelie De Waal, Nasreen Desai, Hannah Douglas, James Druce, Lady-Namera Ejamike, Meron Esere, Alex Eyre, Ibrahim Talal Fazmin, Sophia Fitzgerald-Smith, Verity Ford, Sarah Freeston, Katherine Garnett, Whitney General, Helen Gilbert, Zein Gowie, Ciaran Grafton-Clarke, Keshni Gudka, Leher Gumber, Rishi Gupta, Chris Harlow, Amy Harrington, Adele Heaney, Wing Hang Serene Ho, Lucy Holloway, Christina Hood, Eleanor Houghton, Saba Houshangi, Emma Howard, Benjamin Human, Harriet Hunter, Ifrah Hussain, Sami Hussain, Richard Thomas Jackson-Taylor, Bronwen Jacob-Ramsdale, Ryan Janjuha, Saleh Jawad, Muzzamil Jelani, David Johnston, Mike Jones, Sadhana Kalidindi, Savraj Kalsi, Asanish Kalyanasundaram, Anna Kane, Sahaj Kaur, Othman Khaled Al-Othman, Qaisar Khan, Sajan Khullar, Priscilla Kirkland, Hannah Lawrence-Smith, Charlotte Leeson, Julius Elisabeth Richard Lenaerts, Kerry Long, Simon Lubbock, Jamie Mac Donald Burrell, Rachel Maguire, Praveen Mahendran, Saad Majeed, Prabhjot Singh Malhotra, Vinay Mandagere, Angelos Mantelakis, Sophie McGovern, Anjola Mosuro, Adam Moxley, Sophie Mustoe, Sam Myers, Kiran Nadeem, Reza Nasseri, Tom Newman, Richard Nzewi, Rosalie Ogborne, Joyce Omatseye, Sophie Paddock, James Parkin, Mohit Patel, Sohini Pawar, Stuart Pearce, Samuel Penrice, Julian Purdy, Raisa Ramjan, Ratan Randhawa, Usman Rasul, Elliot Raymond-Taggert, Rebecca Razey, Carmel Razzaghi, Eimear Reel, Elliot John Revell, Joanna Rigbye, Oloruntobi Rotimi, Abdelrahman Said, Emma Sanders, Pranoy Sangal, Nora Sangvik Grandal, Aadam Shah, Rahul Atul Shah, Oliver Shotton, Daniel Sims, Katie Smart, Martha Amy Smith, Nick Smith, Aninditya Salma Sopian, Matthew South, Jessica Speller, Tom J. Syer, Ngan Hong Ta, Daniel Tadross, Benjamin Thompson, Jess Trevett, Matthew Tyler, Roshan Ullah, Mrudula Utukuri, Shree Vadera, Harriet Van Den Tooren, Sara Venturini, Aradhya Vijayakumar, Melanie Vine, Zoe Wellbelove, Liora Wittner, Geoffrey Hong Kiat Yong, Farris Ziyada, Oliver Patrick Devine

**Affiliations:** 1 Research Department of Medical Education, UCL Medical School, Gower Street, London, WC1E 6BT UK; 2 Good Hope Hospital, Rectory Rd, Sutton Coldfield, B75 7RR UK; 3 St George’s, University of London, Cranmer Terrace, London, SW17 0RE UK; 4 UCL Medical School, 74 Huntley Street, London, WC1E 6BT UK; 5 General Medical Council, Regent’s Place, 350 Euston Road, London, NW1 3JN UK; 6 School of Clinical Medicine, University of Cambridge, Addenbrooke’s Hospital, Hills Rd, Cambridge, CB2 0SP UK; 7 Medical Student Office, Newcastle University, Framlington Place, Newcastle upon Tyne, NE2 4HH UK; 8 Hull University Teaching Hospitals, Hull Royal Infirmary, Anlaby Road, Hull, HU3 2JZ UK; 9 Faculty of Medicine, University of Southampton, Building 85, Life Sciences Building, Highfield Campus, Southampton, SO17 1BJ UK; 10 University of Aberdeen, Suttie Centre, Foresterhill, Aberdeen, AB25 2ZD UK; 11 Norwich Medical School, Faculty of Medicine and Health Sciences, University of East Anglia, Norwich, NR4 7TJ UK; 12 Faculty of Health Sciences, University of Bristol Medical School, First Floor South, Senate House, Tyndall Avenue, Bristol, BS8 1TH UK; 13 Imperial College School of Medicine, South Kensington Campus, London, SW7 2AZ UK; 14 School of Medicine, Dentistry and Nursing, University of Glasgow, Glasgow, G12 8QQ UK; 15 University of York, John Hughlings Jackson Building, Heslington, York, YO10 5DD UK; 16 School of Medicine, Keele University, David Weatherall Building, Keele University Campus, Staffordshire, ST5 5BG UK; 17 Birmingham Medical School, Vincent Drive, Edgbaston B15 2TT, Birmingham, West Midlands UK; 18 University of Edinburgh Medical School, 47 Little France Cres, Edinburgh, EH16 4TJ UK; 19 Brighton and Sussex Medical School, BSMS Teaching Building, University of Sussex, Brighton, BN1 9PX UK; 20 The Medical School, The University of Sheffield, Beech Hill Road, Sheffield, S10 2RX UK; 21 Barts and The London Medical School, 4 Newark St, Whitechapel, London, E1 2AT UK; 22 Cambridge University Hospitals NHS Foundation Trust, Hills Road, Cambridge, CB2 0QQ UK; 23 University of Dundee School of Medicine, 4 Kirsty Semple Way, Dundee, DD2 4BF UK; 24 The University of Nottingham, Queen’s Medical Centre, Nottingham, NG7 2UH UK; 25 Whiston Hospital, Warrington Road, Prescot, L35 5DR UK; 26 Medical Sciences Divisional Office, University of Oxford, Level 3, John Radcliffe Hospital, Oxford, OX3 9DU UK; 27 Guy’s, King’s and St Thomas’ School of Medical Education, Henriette Raphael Building, Guy’s Campus, London, SE1 1UL UK; 28 Queen’s University Belfast, University Road, Belfast, BT7 1NN UK; 29 Manchester Medical School, Stopford Building, Oxford Rd, Manchester, M13 9PT UK; 30 Cardiff University School of Medicine, Cochrane Building, Heath Park Way, Cardiff, CF14 4YU UK; 31 School of Medicine, University of Leeds, Worsley Building, Leeds, LS2 9NL UK; 32 University of Liverpool Medical School, Cedar House, Ashton St, Liverpool, L69 3GE UK; 33 University of Leicester School of Medicine, George Davies Centre, Lancaster Road, Leicester, LE1 7HA UK; 34 St James’s University Hospital, Beckett Street, Leeds, West Yorkshire LS9 7TF UK; 35 Homerton University Hospital, Homerton Row E9 6SR, London, UK; 36 University Hospitals of Leicester NHS Trust, Infirmary Square, Leicester, LE1 5WW UK; 37 Nottingham University Hospitals NHS Trust, Hucknall Rd, Nottingham, NG5 1PB UK; 38 Aberdeen Royal Infirmary, Foresterhill, Aberdeen, AB25 2ZN UK

**Keywords:** Medical school differences, Teaching styles, National Student Survey, National Training Study, Postgraduate qualifications, Fitness to practise, GMC sanctions, Problem-based learning, Preparedness, Institutional histories

## Abstract

**Background:**

Medical schools differ, particularly in their teaching, but it is unclear whether such differences matter, although influential claims are often made. The Medical School Differences (*MedDifs*) study brings together a wide range of measures of UK medical schools, including postgraduate performance, fitness to practise issues, specialty choice, preparedness, satisfaction, teaching styles, entry criteria and institutional factors.

**Method:**

Aggregated data were collected for 50 measures across 29 UK medical schools. Data include *institutional history* (e.g. rate of production of hospital and GP specialists in the past), *curricular influences* (e.g. PBL schools, spend per student, staff-student ratio), s*election measures* (e.g. entry grades), *teaching and assessment* (e.g. traditional vs PBL, specialty teaching, self-regulated learning), s*tudent satisfaction, Foundation selection scores*, *Foundation satisfaction*, *postgraduate examination performa*nce and *fitness to practise* (postgraduate progression, GMC sanctions). Six specialties (General Practice, Psychiatry, Anaesthetics, Obstetrics and Gynaecology, Internal Medicine, Surgery) were examined in more detail.

**Results:**

Medical school differences are stable across time (median alpha = 0.835). The 50 measures were highly correlated, 395 (32.2%) of 1225 correlations being significant with *p* < 0.05, and 201 (16.4%) reached a Tukey-adjusted criterion of *p* < 0.0025.

Problem-based learning (PBL) schools differ on many measures, including lower performance on postgraduate assessments. While these are in part explained by lower entry grades, a surprising finding is that schools such as PBL schools which reported *greater* student satisfaction with feedback also showed *lower* performance at postgraduate examinations.

More medical school teaching of psychiatry, surgery and anaesthetics did not result in more specialist trainees. Schools that taught more general practice did have more graduates entering GP training, but those graduates performed less well in MRCGP examinations, the negative correlation resulting from numbers of GP trainees and exam outcomes being affected both by non-traditional teaching and by greater historical production of GPs.

Postgraduate exam outcomes were also higher in schools with more self-regulated learning, but lower in larger medical schools.

A path model for 29 measures found a complex causal nexus, most measures causing or being caused by other measures. Postgraduate exam performance was influenced by earlier attainment, at entry to Foundation and entry to medical school (the so-called academic backbone), and by self-regulated learning.

Foundation measures of satisfaction, including preparedness, had no subsequent influence on outcomes. Fitness to practise issues were more frequent in schools producing more male graduates and more GPs.

**Conclusions:**

Medical schools differ in large numbers of ways that are causally interconnected. Differences between schools in postgraduate examination performance, training problems and GMC sanctions have important implications for the quality of patient care and patient safety.

## Background

Medical schools differ. Whether those differences matter is however unclear. The UK General Medical Council (GMC), in a 2014 review of medical school differences in preparedness for Foundation Programme training [1], commented that perhaps,

Variation between medical schools in the interests, abilities and career progression of their graduates is inevitable and not in itself a cause for concern …

In contrast, the GMC’s 1977 Report had been more bullish, quoting the 1882 Medical Act Commission:

It would be a mistake to introduce absolute uniformity into medical education. One great merit of the present system … lies in the elasticity which is produced by the variety and number of educational bodies … Nothing should be done to weaken the individuality of the universities … [2, 3] (p.x, para 37).

Whether variation is indeed a “great merit” or potentially “cause for concern” is actually far from clear. In 2003, one of us [ICM] had asked whether medical school differences reflected, “beneficial diversity or harmful deviations”, pointing out that,

few studies have assessed the key question of the extent to which different educational environments—be they differences in philosophy, method of delivery, content, approach, attitudes, or social context—produce different sorts of doctor [4].

Five years later, an appendix to the UK Tooke Report of 2008 asked for,

answers to some fundamental questions. How does an individual student from one institution compare with another from a different institution? Where should that student be ranked nationally? … Which medical schools’ students are best prepared for the Foundation Years and, crucially, what makes the difference? [5] (p. 174)

Since those statements were written, systematic data on UK medical school differences have begun to emerge and will continue to do so in the future as the UKMED database develops [6]. Different students have long been known to apply to different medical schools for different reasons [7]. Comparing outcomes at graduation from different UK medical schools is difficult, since schools currently have no common final assessment (and indeed may set different standards [8]), although the UK Medical Licensing Assessment (UKMLA) should change that [9]. In recent years, it has become clear that graduates from different medical schools vary substantially in their performance on postgraduate assessments, including MRCP (UK) [10, 11], MRCGP [11–14], MRCOG [15] and FRCA [16], direct comparison being possible as the exams are identical for graduates of all medical schools.

The present study, Medical School Differences (*MedDifs*), which evaluates the nature of medical school differences, addresses three specific questions and one general question about how UK medical schools differ, using a database containing fifty different descriptive measures of medical schools.

1. *Preparedness*. Do medical schools differ in the preparedness of their graduates for Foundation training, and do differences in preparedness matter?
2. *Problem-based learning (PBL) schools*. Do graduates from PBL schools differ in their outcomes compared with non-PBL graduates?
3. *Specialty teaching and specialty choice*. Does more undergraduate teaching of particular specialties, such as General Practice or Psychiatry, result in more graduates choosing careers in General Practice or Psychiatry?
4. *Analysing the broad causal picture of medical school differences*. What are the causal relations between the wide set of measures of medical schools, and can one produce a map of them?

### Preparedness

The GMC has been particularly interested in the *preparedness* of medical school graduates for Foundation training, in part following on from the Tooke Report’s question on which schools’ graduates are the best prepared Foundation trainees [5]. The GMC commissioned a large-scale qualitative study in 2008 [17], which clearly described the extent of preparedness and sometimes its absence, but also reported finding no differences between three very different medical schools (one integrated, one PBL and the third graduate-entry). The UK National Training Survey (NTS), run by the GMC, has reported that “there are major differences between medical schools in the preparedness … of their graduates [for Foundation training]” [1]. The GMC explanations of the differences are sufficiently nuanced to avoid any strong conclusions, so that “there is room to debate whether the variation between schools in graduate preparedness is a problem” [1]. Nevertheless, the GMC covers well the domain of possible explanations. Preparedness measures are themselves perceptions by students and are yet to be validated against actual clinical behaviours, and the GMC report suggests that differences “may be due to subjective factors rather than real differences between medical schools or in the performance of their graduates” [1], with a suggestion that differences are perhaps related to student perceptions in the National Student Survey (NSS). The eventual conclusions of the GMC’s report are less sanguine than the suggestion that variation might “not in itself [be] a cause for concern”, as variation in preparedness “can highlight problematic issues across medical education … [which may be] tied to particular locations – perhaps *with causes that can be identified and addressed*” [1] [our emphasis]. That position has been developed by Terence Stephenson, who as Chair of the GMC in 2018 said, “The best schools show over 90% of their graduates feel well prepared, but there’s at least a 20 point spread to the lowest performing schools [ … ]. I’d be pretty troubled if I was one of those students [at a low performing school]” [18].

The GMC report considers a wide range of issues beyond preparedness itself, and together, they survey much of the terrain that any analysis of medical school differences must consider. Graduates of different schools are known to vary in their likelihood of entering particular specialties, with differences in General Practice being visible for several decades [19]. Current concerns focus particularly on General Practice and Psychiatry. The report is equivocal about:

… the substantial variations between medical schools in relation to specialisation of their graduates, whether or not this is desirable. On the one hand, the pattern can be seen as resulting from competition for places in specialty training and as reflecting the relevant and relative strengths of the graduates applying and progressing. On the other hand, the medical schools producing large numbers of GPs are helping to address a key area of concern in medical staffing. The specialties most valued by students or doctors in training may not be the most valuable to the NHS. [1]

The interpretation of “major differences between medical schools in the … subsequent careers of their graduates” [1] is seen as problematic:

Clearly, events later in a doctor’s career will tend to be less closely attributable to their undergraduate education. In any case, this information is not sufficient to demonstrate that some schools *are better than others*. That depends on the criteria you use, and not least whether it is relevant to consider the value added by the medical school taking into account the potential of the students they enrol. [1] [our emphasis]

A simple description of “better than others” is contrasted with a value-added approach, which accepts that the students enrolled at some schools may vary in their potential.

### Problem-based learning schools

Medical schools differ in the processes by which they teach and assess, and the GMC report asks “Can we draw any associations between preparedness and types of medical school?”, particularly asking about problem-based learning (PBL), which is more common in the newer medical schools. Two different reviews are cited [20, 21], but reach conflicting conclusions on the specific effects of PBL.

The *MedDifs* study aims to ask how medical school differences in teaching and other measures relate to differences in medical school outcomes. While systematic data on medical school outcomes have been rare until recently, data on the detailed processes of medical school teaching, and how they differ, have been almost non-existent. The *Analysis of Teaching of Medical Schools* (*AToMS*) study [22], which is a companion to the present paper, addresses that issue directly and provides detailed data on timetabled teaching events across the 5 years of the undergraduate course in 25 UK medical schools. PBL and non-PBL schools differed on a range of teaching measures. Overall schools could be classified in terms of two dimensions or factors, *PBL* vs *traditional* and *structured* vs *non-structured*, and those two summary measures are included in the current set of fifty measures.

Schools differ not only in how they teach but in how they assess [23] and the standards that are set [8], the GMC report commenting that “There is also the moot point about how students are assessed. There is some evidence that assessment methods and standards for passing exams vary across medical schools.” [1]. The possibility is also raised that “a national licensing examination might reduce variation in preparedness by preventing some very poor graduates from practising and possibly by encouraging more uniformity in undergraduate curricula.” [1].

### Specialty teaching and specialty choice

The GMC’s report has shown that there is little certainty about most issues concerning medical school differences, with empirical data being limited and seldom cited. In contrast, there are plenty of clear opinions about why medical schools might differ. Concerns about a shortage of GPs and psychiatrists have driven a recent discourse in medical education which concludes that it is differences between medical schools in their teaching which drive differences in outcomes. Professor Ian Cumming, the chief executive of Health Education England (HEE), put the position clearly when he said:

It’s not rocket science. If the curriculum is steeped in teaching of mental health and general practice you get a much higher percentage of graduates who work in that area in future. [24]

In October 2017, the UK Royal College of Psychiatrists also suggested that,

medical schools must do more to put mental health at the heart of the curriculum … and [thereby] encourage more medical students to consider specialising in psychiatry [25],

although there was an acknowledgment by the College’s President that,

the data we currently have to show how well a medical school is performing in terms of producing psychiatrists is limited [25]

That limitation shows a more general lack of proper evidence on differences in medical teaching, and only with such data is a serious analysis possible of the effects of medical school differences. Which measures are appropriate is unclear, as seen in a recent study claiming a relationship between GP teaching and entry into GP training [26] with “authentic” GP teaching, defined as “teaching in a practice with patient contact, in contrast to non-clinical sessions such as group tutorials in the medical school”. The political pressures for change though are seen in the conclusion of the House of Commons Health Committee that “Those medical schools that do not adequately teach primary care as a subject or fall behind in the number of graduates choosing GP training should be held to account by the General Medical Council.” [27]. The GMC however has pointed out that it has “no powers to censure or sanction medical schools that produce fewer GPs” [28], but differences between schools in pass rates for GP assessments may be within the remit of its legitimate concerns.

The processes by which experience of GP can influence career choice have been little studied. Positive experiences of general practice, undergraduate and postgraduate, may result in an interest in GP mediated via having a suitable personality, liking of the style of patient care, appreciating the intellectual challenge and an appropriate work-life balance [29], although the converse can occur, exposure to general practice clarifying that general practice is *not* an appropriate career. Unfortunately, data on these variable factors are not available broken down by medical school.

### Analysing the broad causal picture of medical school differences

Although the three specific issues mentioned so far—preparedness, PBL and specialty choice—are of importance, a greater academic challenge is to understand the relations between the wide set of ways that can characterise differences between medical schools. The set of fifty measures that we have collected will be used here to assess how medical school differences can be explained empirically, in what we think is the first systematic study of how differences between UK medical schools relate to differences in outcome across a broad range of measures.

Medical schools are social institutions embedded in complex educational systems, and there are potentially very many descriptive measures that could be included at all stages. All but a very small number of the 50 measures we have used are based on information available in the public domain. Our study uses a range of measures that potentially have impacts upon outcomes, some of which are short term (e.g. NSS evaluations, or preparedness) and some of which are historical in the life of institutions or occur later in a student’s career after leaving medical school (e.g. entry into particular career specialties, or performance on postgraduate examinations). There are many potential outcome measures that could be investigated, and for examination results, we have concentrated on six particular specialties: General Practice and Psychiatry because there is current concern about recruitment, as there is also for Obstetrics and Gynaecology (O&G) [30, 31]; Surgery, as there is a recent report on entry into Core Surgical Training [32]; and Anaesthetics and Internal Medicine, since postgraduate examination results are available (as also for General Practice and O&G). We have also considered two non-examination outcomes—problems with Annual Record of Competency Progression (ARCP) (ARCP for non-exam reasons, and fitness to practise (FtP) problems with the General Medical Council) which many indicate wider, non-academic problems with doctors.

Many of our measures are inter-related, and a challenge, as in all science, is to identify *causal* relations between measures, rather than mere correlations (although correlation is usually necessary for causation). Understanding causation is crucial in all research, and indeed in everyday life, for “Causal knowledge is what helps us predict the future, explain the past, and intervene to effect change” [33] (p. vii). The temporal ordering of events is necessary, but not sufficient, for identifying causes, since “causes are things that precede and alter the probability of their effects” [34] (p. 72). In essence, causes affect things that follow them, not things that occur before them. A priori plausibility, in the sense of putative theoretical mechanisms, and coherence, effects not being inconsistent with what is already known, are also of help in assigning causality [33]. And of course suggested causation is always a hypothesis to be tested with further data.

The details of the 50 measures will be provided in the “Method” section, in Table 1, but a conceptual overview along with some background is helpful here. Our measures can be broadly classified, in an approximate causal order as:

- *Institutional history (10 measures).* Medical schools have histories, and how they are today is in large part determined by how they were in the past, an institutional variation on the dictum that “past behaviour predicts future behaviour” [46]. We have therefore looked at the overall output of doctors; the production of specialists, including GPs, from 1990 to 2009; the proportion of female graduates; whether a school was founded after 2000; and research tradition.
- *Curricular influences (4 measures)*. A curriculum is a plan or a set of intentions for guiding teachers and students on how learning should take place, and is not merely a syllabus or a timetable, but reflects aspiration, intentions and philosophy [47]. PBL is one of several educational philosophies, albeit that it is “a seductive approach to medical education” [48] (p. 7), the implementation of which represents policy decisions which drive many other aspects of a curriculum. Implementation of a curriculum, the “curriculum in action” [47], instantiated in a timetable, is driven by external forces [49], including resources [47], such as money for teaching, staff-student ratio and numbers entering a school each year.
- *Selection (3 measures)*. Medical schools differ in the students that they admit, in academic qualifications, in the proportion of female entrants or in the proportion of students who are “non-home”. Differences in entrants reflect selection by medical schools and also self-selection by students choosing to apply to, or accept offers from, different medical schools [7], which may reflect course characteristics, institutional prestige, geography etc. Academic attainment differences may result from differences in selection methods, as well as decisions to increase diversity or accept a wider range of student attainments (“potential” as the GMC puts it [1]).
- *Teaching, learning and assessment (10 measures).* Schools differ in their teaching, as seen in the two main factors in the *AToMS* study [22], which assess a more traditional approach to teaching as opposed to newer methods such as PBL or CBL (case-based learning), and the extent to which a course is structured or unstructured. There are also differences in the teaching of particular specialties, as well as in the amount of summative assessment [23], and of self-regulated learning, based on data from two other studies [39, 50], described elsewhere [22].
- *Student satisfaction measures (NSS) (2 measures).* Medical schools are included in the NSS, and two summary measures reflect overall course perceptions, “overall satisfaction with teaching” and “overall satisfaction with feedback”. The interpretation of NSS measures can be difficult, sometimes reflecting student perceptions of course easiness [51].
- *Foundation entry scores (2 measures).* After graduation, students enter Foundation training, run by the Foundation Programme Office (UKFPO), with allocation to posts based on various measures. The Educational Performance Measure (UKFPO-EPM) is based on quartiles or deciles of performance during the undergraduate course, as well as other degrees obtained (most typically intercalated degrees), and scientific papers published. Quartiles and deciles are normed locally within medical schools, and therefore, schools show no differences in mean scores. The UKFPO Situational Judgement Test (UKFPO-SJT) is normed nationally and can be compared across medical schools [42, 43].
- *F1 perception measures (4 measures).* Four measures are available from the GMC’s National Training Survey (NTS), preparedness for Foundation training [1], and measures of overall satisfaction and satisfaction with workload and supervision during F1 training.
- *Choice of specialty training (4 measures).* The proportion of graduates applying for or appointed as trainees in specialties such as general practice.
- *Postgraduate examination performance (9 measures).* A composite measure provided by the GMC of overall pass rate at all postgraduate examinations, as well as detailed marks for larger assessments such as MRCGP, FRCA, MRCOG and MRCP (UK).
- *Fitness to practise (2 measures).* Non-exam-related problems identified during the Annual Record of Competency Progression assessments (Smith D.: ARCP outcomes by medical school. London: General Medical Council, unpublished) [45, 52] (section 4.33), as well as GMC fitness to practise (FtP) sanctions.

**Table 1:** Summary of the measures of medical school differences. Measures in bold are included in the set of 29 measures in the path model of Fig. 5

Group	Measure name	Description	Reliability	Notes on reliability
*Institutional history*	***Hist_Size***	**Historical size of medical school**. Based on GMC LRMP, with average number of graduates entering the Register who qualified from 1990 to 2014. Note that since the University of London was actually five medical schools, size is specified as an average number per London school. Note also that Oxford and Cambridge refer to the school of graduation, and not school of entry, with some Oxbridge graduates qualifying elsewhere.	**.925**	Based on numbers of graduates in years 1990–1994, 1995–1999, 2000–2004, 2005–2009 and 2010–2014
	***Hist_GP***	**Historical production of GPs by medical schools**. Based on the proportion of 1990–2009 graduates on the LRMP on the GP Register.	**.968**	Based on rates for graduates in years 1990–1994, 1995–1999, 2000–2004 and 2005–2009
	***Hist_Female***	**Historical proportion of female graduates.** Based on GMC LRMP, with average percentage of female graduates entering the Register from 1990 to 2014.	**.831**	Based on percentage of female graduates in years 1990–1994, 1995–1999, 2000–2004, 2005–2009 and 2010–2014
	*Hist_Psyc*	**Historical production of psychiatrists by medical schools**. Based on the proportion of 1990–2009 graduates on the LRMP on the Specialist Register for Psychiatry.	**.736**	Based on rates for graduates in years 1990–1994, 1995–1999 and 2000–2004. *N*s for 2005–2009 graduates were too low to be useful
	*Hist_Anaes*	**Historical production of anaesthetists by medical schools.** Based on the proportion of 1990–2009 graduates on the LRMP on the Specialist Register for Anaesthetics	**.716**	Based on rates for graduates in years 1990–1994, 1995–1999 and 2000–2004. *N*s for 2005–2009 graduates were too low to be useful
	*Hist_OG*	**Historical production of obstetricians and gynaecologists by medical schools** Based on the proportion of 1990–2009 graduates on the LRMP on the Specialist Register for O&G.	**.584**	Based on rates for graduates in years 1990–1994, 1995–1999 and 2000–2004. *N*s for 2005–2009 graduates were too low to be useful
	*Hist_IntMed*	**Historical production of internal medicine physicians by medical schools.** Based on the proportion of 1990–2009 graduates on the LRMP on the Specialist Register for Internal Medicine specialties.	**.945**	Based on rates for graduates in years 1990–1994, 1995–1999 and 2000–2004. *N*s for 2005–2009 graduates were too low to be useful
	*Hist_Surgery*	**Historical production of surgeons by medical schools.** Based on the proportion of 1990–2009 graduates on the LRMP on the Specialist Register for Surgical specialties.	**.634**	Based on rates for graduates in years 1990–1994, 1995–1999 and 2000–2004. *N*s for 2005–2009 graduates were too low to be useful
	***Post2000***	**New medical school**. A school that first took in medical students after 2000. The five London medical schools are not included as they were originally part of the University of London.	**n/a**	n/a
	***REF***	**Research Excellence Framework.** Weighted average of overall scores for the 2008 Research Assessment Exercise (RAE), based on units of assessment 1 to 9, and the 2014 Research Excellence Framework (REF) based on units of assessments 1 and 2. Notes that UoAs are not directly comparable across the 2008 and 2014 assessments. Combined results are expressed as a *Z* score.	**.691**	Based on combined estimate from RAE2008 and REF2014
*Curricular influences*	***PBL_School***	**Problem-based learning school**. School classified in the BMA guide for medical school applicants in 2017 as using problem-based or case-based learning [35], with the addition of St George’s, which is also PBL.	**n/a**	n/a
	***Spend_Student***	**Average spend per student.** The amount of money spent on each student, given as a rating out of 10. Average of values based on the Guardian guides for university applicants in 2010 [36], 2013 [37] and 2017 [38].	**.843**	Based on values for 2010, 2013 and 2017
	***Student_Staff***	**Student-staff ratio.** Expressed as the number of students per member of teaching staff. Average of values based on the Guardian guides for medical school applicants in in 2010 [36], 2013 [37] and 2017 [38].	**.835**	Based on values for 2010, 2013 and 2017
	***Entrants_N***	**Number of entrants to the medical school.** An overall measure of the size of the school based on MSC data for the number of medical students entering in 2012–16.	**.994**	Based on numbers of entrants 2012–2016
*Selection*	***Entrants_Female***	**Percent of entrants who are female.**	**.903**	Based on entrants for 2012–2016
	***Entrants_NonHome***	**Percent of entrants who are “non-home”.** Percentage of all entrants for 2012–2017 with an overseas domicile who are not paying home fees, based on HESA data. Note that proportions are higher in Scotland (16.4%), and Northern Ireland (13.3%), than in England (7.5%) or Wales (5.9%), perhaps reflecting national policy differences. This variable may therefore be confounded to some extent with geography. For English schools alone, the reliability was only .537.	**.958**	Based on entrants for 2012–2017
	***EntryGrades***	**Average entry grades.** The average UCAS scores of students currently studying at the medical school expressed as UCAS points. Average of values based on the Guardian guides for medical school applicants in 2010 [36], 2013 [37] and 2017 [38].	**.907**	Based on values for 2010, 2013 and 2017
*Teaching, learning and assessment*	***Teach_Factor1_Trad***	**Traditional vs PBL teaching**. Scores on the first factor describing differences in medical school teaching, positive scores indicating more traditional teaching rather than PBL teaching. From the *AToMS* study [22] for 2014–2015.	**n/a**	n/a
	***Teach_Factor2_Struc***	**Structured vs unstructured teaching.** Scores on the second factor describing differences in medical school teaching, positive scores indicating teaching is more structured rather than unstructured. From the *AToMS* study [22] for 2014–2015.	**n/a**	n/a
	***Teach_GP***	**Teaching in General Practice**. Total timetabled hours of GP teaching from the *AToMS* Survey [22] for 2014–2015.	**n/a**	n/a
	*Teach_Psyc*	**Teaching in Psychiatry**. Total timetabled hours of Psychiatry teaching from the *AToMS* Survey [22] for 2014–2015.	**n/a**	n/a
	*Teach_Anaes*	**Teaching in Anaesthetics**. Total timetabled hours of Anaesthetics teaching from the *AToMS* Survey [22] for 2014–2015.	**n/a**	n/a
	*Teach_OG*	**Teaching in Obstetrics and Gynaecology**. Total timetabled hours of O&G teaching from the *AToMS* Survey [22] for 2014–2015.	**n/a**	n/a
	*Teach_IntMed*	**Teaching in Internal Medicine**. Total timetabled hours of Internal Medicine teaching from the *AToMS* Survey [22] for 2014–2015.	**n/a**	n/a
	*Teach_Surgery*	**Teaching in Surgery.** Total timetabled hours of Surgery teaching from the *AToMS* Survey [22] for 2014–2015.	**n/a**	n/a
	***ExamTime***	**Total examination time.** Total assessment time in minutes for all undergraduate examinations, from the *AToMS* study [23] for 2014–2015.	**n/a**	**n/a**
	***SelfRegLearn***	**Self-regulated learning.** Overall combined estimate of hours of self-regulated learning from survey of self-regulated learning [39] and HEPI data [22].	**n/a**	**n/a**
*Student satisfaction measures*	***NSS_Satis’n***	**Course satisfaction in the NSS**. The percentage of final-year students satisfied with overall quality, based on the National Student Survey (NSS). Average of values from the Guardian guides for medical school applicants in 2010 [36], 2013 [37] and 2017 [38]. Further data are available from the Office for Students [40] with questionnaires also available [41].	**.817**	Based on values for 2010, 2013 and 2017
	***NSS_Feedback***	**Satisfaction with feedback in the NSS**. The percentage of final-year students satisfied with feedback and assessment by lecturers, based on the National Student Survey UKFPO- (NSS). Average of values from the Guardian guides for medical school applicants in 2010 [36], 2013 [37] and 2017 [38].	**.820**	Based on values for 2010, 2013 and 2017
*Foundation entry scores*	***UKFPO_EPM***	**Educational Performance Measure**. The EPM consists of a within-medical school decile measure, which cannot be compared across medical schools (“local outcomes” [42, 43]), along with additional points for additional degrees up to two peer-reviewed papers (which can be compared across medical schools and hence are “nationally comparable”). Data from the UK Foundation Programme Office [44] are summarised as the average of scores for 2012 to 2016.	**.890**	Based on values for the years 2013 to 2017
	***UKFPO_SJT***	**Situational Judgement Test**. The UKFPO-SJT score is based on a nationally standardised test so that results can be directly compared across medical schools. The UK Foundation Programme Office [44] provides total application scores (UKFPO-EPM+ UKFPO-SJT) and UKFPO-EPM scores, so that UKFPO-SJT scores are calculated by subtraction. UKFPO-SJT scores are the average of scores for the years 2012–2016.	**.937**	Based on values for the years 2013 to 2017
*F1 perception measures*	***F1_Preparedess***	**F1 preparedness**. Preparedness for F1 training has been assessed in the GMC’s National Training Survey (NTS) in 2012 to 2017 by a single question, albeit with minor changes in wording [1]. For 2013 and 2014, the question read “I was adequately prepared for my first Foundation post”, summarised as the percentage agreeing or definitely agreeing. Mean percentage agreement was used to summarise the data across years. Note that unlike the other F1 measures, F1_Prep is retrospective, looking back on undergraduate training.	**.904**	Based on values for 2012 to 2017.
	***F1_Satis’n***	**F1 overall satisfaction**. The GMC’s NTS for 2012 to 2017 contained summary measures of overall satisfaction, adequate experience, curriculum coverage, supportive environment, induction, educational supervision, teamwork, feedback, access to educational resources, clinical supervision out of hours, educational governance, clinical supervision, regional teaching, workload, local teaching and handover, although not all measures were present in all years. Factor analysis of the 16 measures at the medical school level, averaged across years, suggested perhaps three factors. The first factor, labelled **overall satisfaction,** accounted for 54% of the total variance, with overall satisfaction loading highest, and 12 measures with loadings of > .66.	**.792**	Reliability of factor scores was not available, but reliabilities of component scores were access to educational resources (alpha = .800, *n* = 5); adequate experience (alpha = .811, *n* = 6); clinical supervision (alpha = .711, *n* = 6); clinical supervision out of hours (alpha = .733, *n* = 3); educational supervision (alpha = .909, *n* = 6); feedback (alpha = .840, *n* = 6); induction (alpha = .741, *n* = 6); overall satisfaction (alpha = .883, *n* = 6); reporting systems (alpha = .846, *n* = 2); supportive environment (alpha = .669, *n* = 3); and work load (alpha = .773, *n* = 6). Reliabilities of factor scores estimated as median of component scores
	***F1_Workload***	**F1 Workload**. See F1_Sat for details. The second factor in the factor analysis accounted for 11% of total variance with positive loadings of >.73 on Workload, Regional teaching and local teaching. The factor was labelled **workload**.	**.792**	
	***F1_Superv’n***	**F1 Clinical Supervision**. See F1_Sat for details. The third factor in the factor analysis accounted for 8% of total variance with a loading of .62 on Clinical supervision and − 0.75 on Handover. The factor was labelled **workload**.	**.792**	
*Choice of specialty training*	***Trainee_GP***	**Appointed as trainee in General Practice**. UKFPO has reported the percentage of graduate by medical school who were accepted for GP training and Psychiatry training (but no other specialties) in 2012 and 2014–2016. The measure is the average of acceptances for GP in the 4 years.	**.779**	Based on rates for 2012, 2014, 2015 and 2016
	*Trainee_Psyc*	**Appointed as trainee in Psychiatry**. See Trainee_GP. UKFPO has reported the percentage of graduate by medical school who were accepted for GP training in 2012 and 2014–2016. The measure is the average of the different years.	**.470**	Based on rates for 2012, 2014, 2015 and 2016
	*TraineeApp_Surgery*	**Applied for Core Surgical Training (CST)**. Percentage of applicants to CST for the years 2013–2015 [32].	**.794**	Based on rates for 2013, 2014 and 2015
	*TraineeApp_Ans*	**Applied for training in Anaesthetics**. A single source for the number of applications in 2015 for training in anaesthetics by medical school is an analysis of UKMED data (Gale T, Lambe P, Roberts M: UKMED Project P30: demographic and educational factors associated with junior doctors' decisions to apply for general practice, psychiatry and anaesthesia training programmes in the UK, Plymouth, unpublished).	**n/a**	n/a
*Postgraduate examination performance*	***GMC_PGexams***	**Overall pass rate at postgrad examinations**. The GMC website has provided summaries of pass rates of graduates at all attempts at all UK postgraduate examinations taken between August 2013 and July 2016, broken down by medical school (https://www.gmc-uk.org/education/25496.asp). These data had been downloaded but on 18 January 2018 but were subsequently removed while the website was redeveloped, and although now available again, were unavailable for most of the time this paper was being prepared.	**n/a**	n/a
	*MRCGP_AKT*	**Average mark at MRCGP AKT**. MRCGP results at first attempt for the years 2010 to 2016 by medical school are available at http://www.rcgp.org.uk/training-exams/mrcgp-exams-overview/mrcgp-annual-reports.aspx. Marks are scaled relative to the pass mark, a just passing candidate scoring zero, and averaged across years. AKT is the Applied Knowledge Test, an MCQ assessment.	**.970**	Based on values for years 2010 to 2016
	*MRCGP_CSA*	**Average mark at MRCGP CSA**. See MRCGP-AKT. Marks are scaled relative to the pass mark, a just passing candidate scoring zero, and averaged across years. CSA is the Clinical Skills Assessment, and in an OSCE-type assessment.	**.919**	Based on values for years 2010 to 2016
	*FRCA_Pt1*	**Average mark at FRCA Part 1**. Based on results for the years 1999 to 2008 [16]. Marks are scaled relative to the pass mark, so that just passing candidates score zero.	**n/a**	n/a
	*MRCOG_Pt1*	**Average mark at MRCOG part 1**. Performance of doctors taking MRCOG between 1998 and 2008 [15]. Marks are scaled relative to the pass mark, so that just passing candidates score zero. Part 1 is a computer-based assessment.	**n/a**	n/a
	*MRCOG_Pt2*	**Average mark at MRCOG part 2 written**. Performance of doctors taking MRCOG between 1998 and 2008 [15]. Marks are scaled relative to the pass mark, so that just passing candidates score zero. Part 2 consists of a computer-based assessment and an oral, but only the oral is included here.	**n/a**	n/a
	*MRCP_Pt1*	**Average mark at MRCP (UK) part 1**. Marks were obtained for doctors taking MRCP (UK) exams at the first attempt between 2008 and 2016. Marks are scaled relative to the pass mark, so that just passing candidates score zero. Part 1 is an MCQ examination.	**.977**	Based on first attempts in the years 2010–2017
	*MRCP_Pt2*	**Average mark at MRCP (UK) part 2**. Marks were obtained for doctors taking MRCP (UK) exams at the first attempt between 2008 and 2016. Marks are scaled relative to the pass mark, so that just passing candidates score zero. Part 2 is an MCQ examination.	**.941**	Based on first attempts in the years 2010–2017
	*MRCP_PACES*	**Average mark at MRCP (UK) PACES**. Marks were obtained for doctors taking MRCP (UK) exams at the first attempt between 2008 and 2016. Marks are scaled relative to the pass mark, so that just passing candidates score zero. PACES is a clinical assessment of physical examination and communication skills.	**.857**	Based on first attempts in the years 2010–2017
*Fitness to practise issues*	***GMC_Sanctions***	**GMC sanctions**. Based on reported FtP problems (erasure, suspension, conditions, undertakings, warnings: ESCUW) from 2008 to 2016, for doctors qualifying since 1990. ESCUW events increase with time after graduation, and therefore, medical school differences were obtained from a logistic regression after including year of graduation. Differences are expressed as the log (odds) of ESCUW relative to the University of London, the largest school. Schools with fewer than 3000 graduates were excluded. Note that although rates of GMC sanctions are regarded here as causally posterior to other events, because of low rates, they mostly occur in doctors graduating before those in the majority of other measures. They do however correlate highly with ARCP-NotExam rates which do occur in more recent graduates (see above).	**.691**	Based on separate ESCUW rates calculated for graduates in the years 1990–1994, 1995–1999, 2000–2004 and 2005–2009. ESCUW rates in graduates from 2010 onwards were too low to have meaningful differences
	***ARCP_NonExam***	**Non-exam problems at ARCP (Annual Record of Competency Progression)** [45] (section 4.33). Based on ARCP and RITA assessments from 2010 to 2014 (Smith D.: ARCP outcomes by medical school. London: General Medical Council, unpublished). Doctors have multiple assessments, and the analysis considers the worst assessment of those taken. Assessments can be problematic because of exam or non-exam reasons, and only non-exam problems are included in the data. Medical specialties differ in their rates of ARCP problems, and effects are removed in a multilevel multinomial model before effects are estimated for each medical school (see Table 4 in reference Smith D.: ARCP outcomes by medical school. London: General Medical Council, unpublished). Results are expressed as the log (odds) for a poor outcome.	**n/a**	n/a

A more detailed consideration of the nature of causality and the ordering of measures is provided in Supplementary File 1.

A difficult issue in comparing medical schools is that in the UK there are inevitably relatively few of them—somewhat more than thirty, with some very new schools not yet having produced any graduates or indeed admitted any students—and the number of predictors is inevitably larger than that. The issue is discussed in detail in the “Method” section, but care has to be taken because of multiple comparisons, with a Bonferroni correction being overly conservative.

Our analysis will firstly describe the correlations between the various measures and then address three key questions: (1) the differences between PBL and non-PBL schools (which we have also assessed in the *AToMS* study in relation to the details of teaching [22]), (2) the extent to which teaching influences career choice and (3) the causal relations across measures at the ten different levels briefly in part described earlier.

## Method

### Names of medical schools

Any overall analysis of medical school differences requires medical schools to be identifiable, as otherwise identifying relationships between measures is not possible. Medical schools though, for whatever reasons, are often reluctant for schools to be named in such analyses. This means that while clear differences between schools can be found [8, 21], further research is impossible. Recently, however, concerns about school differences in postgraduate performance have led the GMC itself to publish a range of outcome data for named medical schools [53], arguing that its statutory duty of regulation requires schools to be named. Possibilities for research into medical school differences have therefore expanded greatly.

Research papers often use inconsistent names for medical schools. Here, in line with the *AToMS* study [22], we have used names based on those used by the UK Medical Schools Council (MSC) [54]. More details of all schools along with full names can be found in the World Directory of Medical Schools [55].

### Medical school histories

A problem with research on medical schools is that medical schools evolve and mutate. Descriptions of different UK medical schools show relative stability until about 2000, when for most databases there are 19 medical schools (Aberdeen, Birmingham, Bristol, Cambridge, Cardiff, Dundee, Edinburgh, Glasgow, Leeds, Leicester, Liverpool, Manchester, Newcastle, Nottingham, Oxford, Queen’s, Sheffield and Southampton). The various London schools underwent successive mergers in the 1980s and 1990s, but were then grouped by the GMC under a single awarding body, the University of London. A group of six “new” medical schools was formed in the 2000s (Brighton and Sussex (2002), Norwich (2000), Hull York (2003), Keele (2003) and Peninsula (2000))^1^ of which Warwick was a 4-year graduate-entry school only.^2^ The five London medical schools (Barts, Imperial, King’s, St.George’s and UCL) only became distinguishable within the GMC’s List of Registered Medical Practitioners (LRMP), as with many other databases, from about 2008 (Imperial), 2010 (King’s and UCL), 2014 (Barts) and 2016 (St. George’s). Peninsula medical school began to split into Exeter and Plymouth from 2013, but for most purposes here can be treated as one medical school, with averages used for the few recent datasets referring to them separately. By 2016, there are therefore 18 of the 19 older medical schools (excluding London), plus five new ones, and five London schools, making 28 schools, with Exeter and Plymouth treated as one. In terms of data records, there are 29 schools, London being included both as a single entity for data up to about 2000, and for data after about that as the five separate entities which emerged out of the University of London. Values for the University of London for more recent measures are estimated as the average of values from the current five London schools. Missing pre-2000 values for current London schools are imputed (see below). Likewise, values for Peninsula Medical School are taken as the average of Exeter and Plymouth when those values are available. Data for Durham (founded in 2001 but merged with Newcastle by 2017) are included, when available, with that of Newcastle. It should also be noted that the LRMP includes a very small number of graduates from overseas campuses for schools such as St George’s and Newcastle.

Our analysis of teaching considered only schools with 5-year courses (“standard entry medicine”; UCAS A100 codes or equivalent) [56], and therefore, schools which are entirely graduate entry only, such as Warwick and Swansea, were excluded. Graduates, in the sense of mature entrants, can enter either standard entry courses or graduate courses, but where schools offer several types of course, entrants to graduate courses are usually only a minority of entrants, although they do show some systematic differences [57]. For most datasets, it is rare to find separate information for 5-year and graduate entry or other courses, most analyses not differentiating graduates by courses (and indeed the LRMP only records the Primary Medical Qualification, and not the type of course). Our analysis is therefore restricted to a comparison of medical schools, primarily because of a lack of adequate data on courses, but we acknowledge that the ideal unit of analysis would be courses within medical schools.

### Problem-based learning schools

An important distinction is between schools that are or are not regarded as broadly problem-based learning (PBL). There is no hard classification, and for convenience, we use the classification provided on the British Medical Association (BMA) website which describes eleven UK schools as PBL or CBL (case-based learning), i.e. Barts, Cardiff, Exeter, Glasgow, Hull York, Keele, Liverpool, Manchester, Norwich, Plymouth and Sheffield [35], and in addition, we include St George’s which describes itself on its website as using PBL. For the ten schools included in the *AToMS* study, there were clear differences between PBL and non-PBL courses in teaching methods and content [22].

### The level of analysis

It must be emphasised here, and throughout this study, that all measures are aggregates at the level of medical schools and are not based on raw data at the student/doctor level, and that must be remembered when interpreting our results.

### Statistical analysis

Basic statistics are calculated using *IBM SPSS* v24, and more complex statistical calculations are carried out within *R* 3.4.2 [58].

### Missing values

Data were missing for various reasons: some medical schools only coming into existence relatively recently, some not existing when historical measures were being collected and some medical schools not responding to requests for data in previous studies [22, 23]. A particular issue is with data based on “University of London”, which exist in earlier datasets whereas later datasets have the five separate London medical schools. We have therefore used imputation to replace the missing variables, in order to keep *N* as high as possible, and to make statistical analysis more practical.

A constraint on imputation is that the number of cases (medical schools; *n* = 29) is less than the number of measures (*n* = 50), making conventional multiple imputation difficult. Missing values were therefore imputed via a single hotdeck imputation [59] based on the *k* nearest neighbours function *kNN() in R. kNN()* was chosen for imputation as from a range of methods it produced the closest match between correlation matrices based on the raw and the complete data generated by imputation, and it results in a completed matrix that is positive semi-definite despite there being more measures than cases.

### Correction for multiple testing

An *N* of 29 schools, as in the present study, means there is relatively little power for detecting a correlation, and for a two-tailed test with alpha = 0.05 and *N* = 29, a correlation of 0.37, which accounts for about 13% of variance, is required for an 80% power (beta = 0.80) for a significant result. A single test is not, however, being carried out, as in contrast to the smallish number of medical schools, there are in principle very many measures that could be collected from each school. That was clear in the *AToMS* paper [22], where in terms of teaching hours alone there are dozens of measures, and in addition, there are many other statistics available, as for instance on the GMC’s website, which has data on examination performance on individual postgraduate examinations, broken down by medical school. In terms of a frequentist approach to statistics, some form of correction is therefore needed to take type I errors into account.

A conventional Bonferroni correction is probably overly conservative, and therefore, we have used a Tukey-adjusted significance level. The standard Bonferroni correction uses an alpha value of 0.05/*N*, where *N* is the number of tests carried out, which makes sense in situations such as in genome-wide association studies where for most associations the null hypothesis is highly likely a priori. However, the Bonferroni correction is probably overly conservative for social science research where zero correlations are not a reasonable prior expectation, statistical tests are not independent and not all hypotheses are of primary interest. Tukey (reported by Mantel [60]) suggested that a better correction uses a denominator of √(*N*), so that the critical significance level is 0.05/√(*N*) [60], an approach similar to that derived using a Bayesian approach [61]. Rosenthal and Rubin [62] suggested that correlations of greater interest should be grouped together and use a less stringent criterion, and Mantel [60] also suggested a similar approach, tests which are “primary to the purposes of the investigation” having one significance level and other, secondary, tests requiring a more stringent level of significance. An additional issue for the current data is that although there are 50 measures, there are only 29 cases, so that the 50 × 50 correlation matrix is necessarily singular, with only 29 positive eigenvalues, making the effective number of measures 29, and hence, the appropriate denominator for a conventional Bonferroni correction would be 29 × 28/2 = 406 (rather than 50 × 49/2 = 1225). The denominator for the Tukey correction would then be √(406), so that a critical *p* value would be 0.05/√(406) = 0.0025. More focussed analyses will identify primary and secondary tests as suggested by Mantel, and are described separately in the “Results” section.

### Reliability of measures

Differences between medical schools are different from differences between individuals, and it is possible in principle for differences between individuals to be highly reliable, while differences in mean scores between medical schools show little or no reliability, and vice-versa. Between-school reliabilities can be estimated directly for many but not all of our measures. Reliabilities of medical school differences are shown in Table 1 and are calculated using Cronbach’s alpha across multiple occasions of measurement. Lack of reliability attenuates correlations, so that if two measures have alpha reliabilities of, say, 0.8 and 0.9, then the maximum possible empirical correlation between them is √(0.8 × 0.9) = 0.85. When, as in one case here, a measure has a reliability of 0.47, then attenuation makes it particularly difficult to find a significant relationship to other measures.

### Path modelling

Assessment of causality used path modelling which is a subset of Structural Equation Modelling (SEM) [63], which is formally related closely to Bayesian causal network analyses [64, 65]. When all variables are measured, rather than latent, path modelling can be carried out using a series of nested, ordered, regression models, following the approach of Kenny [66]. Regression analyses were mostly carried out using Bayesian Model Averaging, based on the approach of Raftery [61], which considers the 2^*k*^ possible regression models and combines them. We used the *bms*() function in the *R bms* package, with the Zellner g-prior set at a conventional level of 1 (the unit information prior, UIP). *bms()* requires at least four predictors, and for the few cases with three or fewer predictors, the *bayesglm*() function in the *arm* package in *R* was used. The criterion for inclusion was that the evidence for the alternative hypothesis was at the *moderate* or *strong* level (i.e. Bayes factors (BF) of 3–10 and 10+) [67, 68]. Likewise, evidence for the null hypothesis was considered *moderate* for a BF between 0.1 and 0.333 (1/10 and 1/3) and *strong* for a BF less than 0.1. As the number of predictors approaches the number of cases, then multicollinearity and variance inflation make it difficult to assess Bayes factors. We therefore used a compromise approach whereby for any particular dependent variable firstly the eight causally closest predictors were entered into the *bms()* model; the top five were retained, the eight next most significant predictors included, and again the top five retained, the process continuing until all predictors had been tested. The method has the advantage of reducing problems due to multicollinearity and prioritising causally closer predictors in the first instance, although more distant predictors can override better prediction if the data support that. It was decided in advance that more than five meaningful direct predictors for a measure was unlikely, particularly given the sample size, and that was supported in practice.

### Data availability

Data for the 50 summary measures for the 29 medical schools are provided as Supplementary File 2_RawAndImputedData.xlsx, which contains both the raw data and the data with imputed values for missing data.

### Ethical permission

None of the data collected as part of the present study involves personal data at the individual level. Data collected as part of the *AToMS* study were administrative data derived from medical school timetables, and other data are aggregated by medical school in other publications and databases. Ethical permission was not therefore required.

## Results

### The raw data

**Fig. 1:**
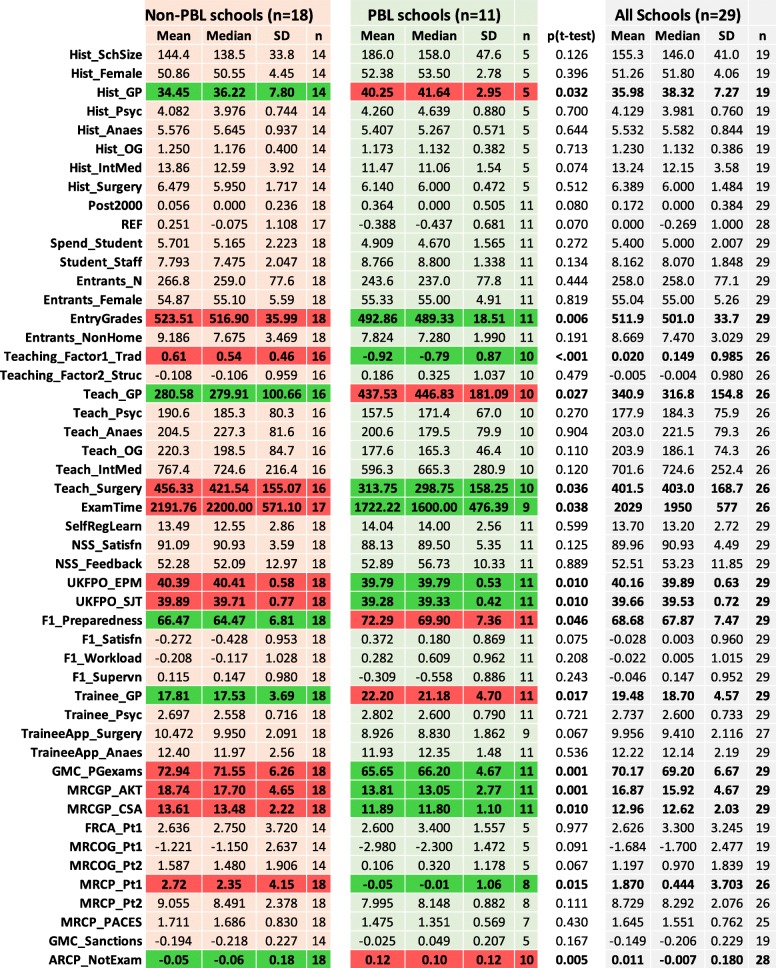
Descriptive statistics for non-PBL schools, PBL schools and all schools (mean, median, SD and *N*) for the measures used in the analyses. Means are compared using *t* tests, allowing for different variances, with significant values indicated in bold (*p* < 0.05). Significant differences are also shown in colour, red indicating the group with the numerically higher score and green the lower scores. Note that higher scores do not always mean better

Fifty measures were available for 29 institutions, with 161/1450 (11.1%) missing data points, in most cases for structural reasons, institutions being too new and measures not being available or because medical schools were not included in surveys. Descriptive statistics are shown in Fig. 1, along with the abbreviated names given in Table 1 which will be used for describing measures, with occasional exceptions for clarity, particularly on the first usage.

### The reliability of medical school differences

Alpha reliabilities could be calculated for 32 of the 50 measures and are shown in Table 1. The median reliability was 0.835 (mean = 0.824 SD = 0.123; range = 0.47 to 0.99), and all but four measures had reliabilities over 0.7. The lowest reliability of 0.47 is for *Trainee_Psyc*, the proportion of trainees entering psychiatry (and four of the six pairs of between-year correlations were close to zero).

### Correlations between the measures

**Fig. 2:**
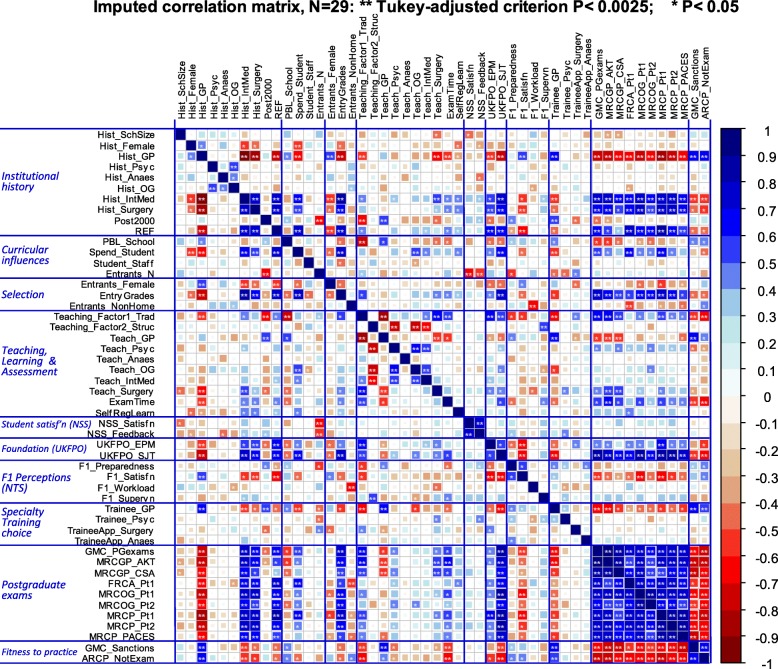
Correlogram showing correlations of the 50 measures across the 29 medical schools. Positive correlations are shown in blue and negative in red (see colour scale on the right-hand side), with the size of squares proportional to the absolute size of the correlation. Significance levels are shown in two ways: two asterisks indicate correlations significant with a Tukey-adjusted correction, and one asterisk indicates correlations significant with *p* < 0.05. For abbreviated variable names, see text. Measures are classified in an approximate causal order with clusters separated by blue lines

For all analyses, the imputed data matrix was used, for which a correlogram is shown in Fig. 2. Of the 1225 correlations, 395 (32.2%) reached a simple *p* < 0.05 criterion, and 201 (16.4%) reached the Tukey-adjusted criterion of 0.0025, making clear that there are many relationships between the measures which require exploration and explanation. As a contrast, a dataset of comparable size but filled with random numbers, whose correlogram is shown in Supplementary File 1 Fig. S1, had just 55 correlations significant at the 0.05 level (4.5%) and only two correlations (0.16%) reached the Tukey-adjusted criterion. The correlogram and equivalent descriptive statistics for the raw, non-imputed, data are available in Supplementary File 1 Fig. S2.

Although there is much of interest in the entire set of correlations in Fig. 1, and will be considered in detail below, firstly, we will consider the three specific questions that the MedDifs study set out to answer on preparedness, PBL and specialty choices, after which the general question of making causal sense of the entire set of data and mapping them will be considered.

#### Preparedness

The GMC has emphasised the importance of new doctors being prepared for the Foundation programme, the first 2 years, F1 and F2, of practice after graduation. It is therefore important to assess the extent to which preparedness relates to other measures. Correlates of *F1_Preparedness* were assessed by considering the 30 measures categorised as institutional history; curricular influences; selection; teaching, learning and assessment; student satisfaction (NSS); and foundation (UKFPO) in Table 1. Using a Tukey criterion of 0.05/sqrt (30) = 0.0091, *F1_Preparedness* correlated with lower *Entrants_N* (*r* = − 0.531, *p* = 0.003) and *Teaching_Factor1_Trad* (i.e. less traditional teaching; *r* = − 0.523, *p* = 0.0036). In terms of outcomes, *F1_Preparedness* did not correlate with any of the 15 outcome measures categorised in Table 1 as specialty training choice, postgraduate exams and fitness to practise, using a Tukey criterion of 0.05/sqrt (15) = .013. *F1_Preparedness* did correlate with *F1_Satisf’n* (*r* = 0.502, *p* = 0.006) but not with *F1_Workload or F1_Superv’n*, although it should be remembered that all four measures were assessed at the same time and there might be halo effects. Differences in self-reported preparedness do *not* therefore relate to any of the outcome measures used here, although preparedness is reported as higher in doctors from smaller medical schools and school using less traditional teaching. The causal inter-relations between the various measures will be considered below.

#### Problem-based learning schools

Figure 1 shows a comparison of mean scores of PBL and non-PBL schools, as well as basic descriptive statistics for all schools in the study. Raw significance levels with *p* < 0.05 are shown, but a Tukey-corrected level is 0.05/sqrt (48) = 0.0072. Altogether, 15/49 (30.6%) differences are significant with *p* < 0.05, and 5 differences (10.2%) reach the Tukey-corrected level. PBL schools have *higher* historical rates of producing GPs (*Hist_GP*), teach more general practice (*Teach_GP*), have higher F1 preparedness (*F1_Preparedness*), produce more trainee GPs (*Trainee_GP*), have higher rates of ARCP problems for non-exam reasons (*ARCP_NonExam*) and have *lower* entry grades (*EntryGrades*), less traditional teaching (*Teach_Factor1_Trad*), less teaching of surgery (*Teach_Surgery*), less examination time (*Exam_Time*), lower UKFPO Educational Performance Measure (*UKFPO_EPM***)** and Situational Judgement Test (*UKFPO_SJT*) scores, lower pass rates in postgraduate exams overall (*GMC_PGexams*) and lower average marks in MRCGP AKT (*MRCGP_AKT*) and CSA (*MRCGP_CSA*) exams and in MRCP (UK) Part 1 (*MRCP_Pt1*). It is clear therefore that PBL schools do differ from non-PBL schools in a range of ways. The causal inter-relationships between these measures will be considered below.

#### The relationship between specialty teaching and specialty outcomes

**Fig. 3:**
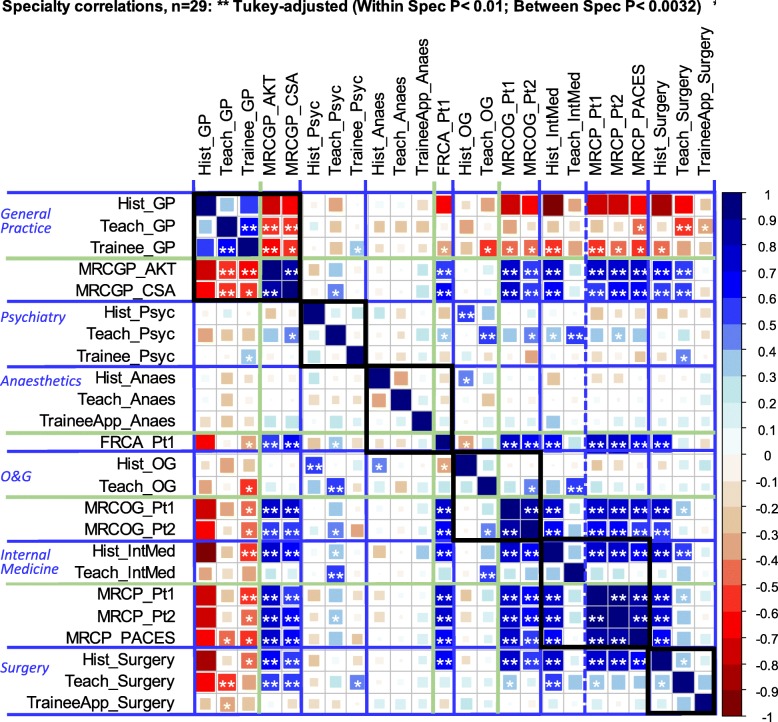
Correlogram of the 24 measures associated with particular specialties across the 29 medical schools. Correlations are the same as in Fig. 1, but re-ordered so that the different specialties can be seen more clearly. Specialties are separated by the horizontal and vertical blue lines, with examination and non-examination measures separated by solid green lines. Two asterisks indicate within- and between-specialty correlations that meet the appropriate Tukey-adjusted *p* value; one asterisk indicates correlations that meet a conventional 0.05 correlation without correction

Is it the case that a curriculum steeped in the teaching of, say, mental health or general practice produces more psychiatrists or GPs in the future [16]? We chose six specialties of interest, looking at historical production of specialists, undergraduate teaching, application or entry to specialty training, and specialty exam performance (see Table 1 for details). In Fig. 3, these measures are extracted from Fig. 2 and, to improve visibility, are reorganised by specialty, the specialties being indicated by blue lines. Overall, there are 276 correlations between the 24 measures. Only the within-specialty correlations are of real interest, of which there are 38, but 14 are relationships between examinations within specialties, which are expected to correlate highly, and therefore, the effective number of tests is 38–14 = 24, and so for within-specialty correlations, the Tukey-adjusted criterion is 0.05/√(24) = 0.010. For the remaining 238 between-specialty correlations, the Tukey-adjusted criterion is 0.05/√(238) = 0.0032.

**Fig. 4:**
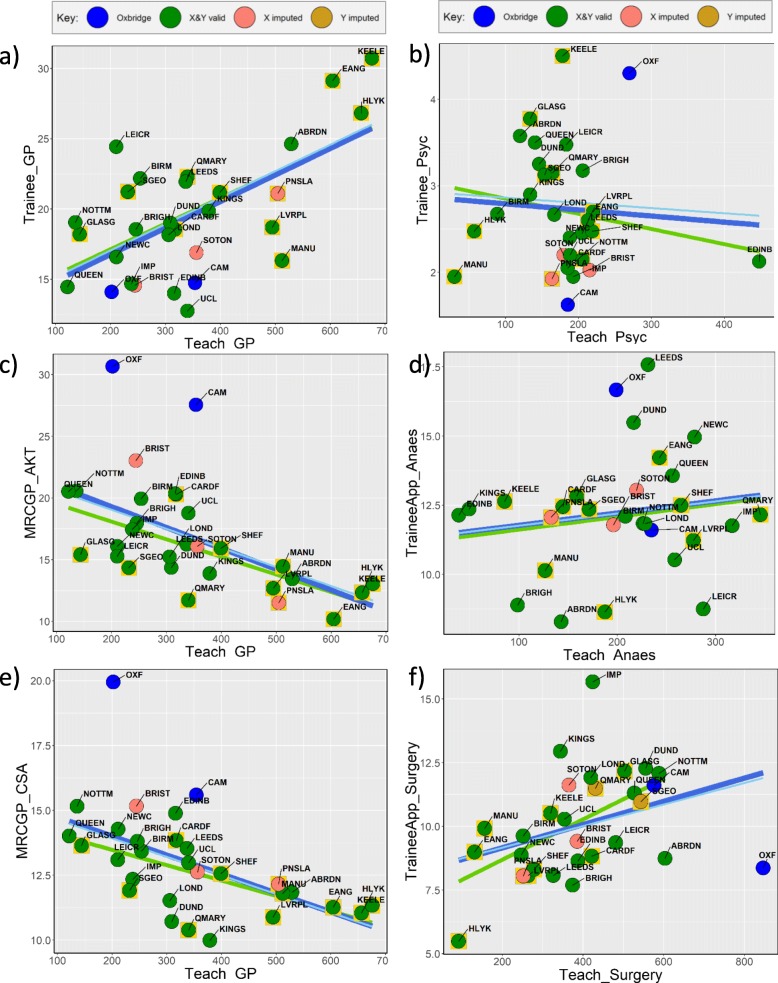
Teaching measures in relation to outcome measures. Regression lines in dark blue are for all points, pale blue for all points excluding imputed values and green for all points excluding Oxbridge (blue circles). Yellow boxes around points indicate PBL schools. See text for discussion

Hours of teaching have relatively few within-subject correlations in Fig. 3. Hours of psychiatry teaching (*Teach_Psyc*) shows no relation to numbers of psychiatry trainees (*Trainee_Psyc*) (Fig. 4b; *r* = − 0.069, *p* = 0.721), amount of anaesthetics teaching (*Teach_Anaes*) is uncorrelated with the numbers of anaesthetics trainees (*TraineeApp_Anaes*) (Fig. 4d; *r* = 0.153, *p* = 0.426), and surgery teaching (*Teach_Surgery*) is unrelated to applications for surgery training (*TraineeApp_Surgery*) (Fig. 4f; *r* = 0.357, *p* = 0.057). Although historical production of specialists shows no influences within Psychiatry (*Hist_Psyc*), O&G (*Hist_OG*) and Surgery (*Hist_Surgery*), nevertheless, *Hist_GP* does relate to *Trainee_GP*, *MRCP_AKT* and *MRCGP_CSA*, and historical production of physicians (*Hist_IntMed*) also relates to performance at MRCP (UK) (*MRCP_Pt1*, *MRCP_Pt2* and *MRCP_PACES*). Cross-specialty correlations are also apparent in Fig. 3, particularly between the different examinations, schools performing better at MRCGP also performing better at FRCA (*FRCA_Pt1*), MRCOG (*MRCOG_Pt1* and *MRCOG_Pt2*), and the three parts of MRCP (UK). Historical production rates of specialties tend to inter-correlate, *Hist_Surgery* correlating with *Hist_IntMed*, but both correlating negatively with *Hist_GP*. Schools producing more psychiatrists (*Hist_Psyc*) also produce more specialists in O&G (*Hist_OG*). Scattergrams for all of the relationships in Fig. 2 are available in Supplementary Files 3, 4, 5, 6, 7, 8 and Supplementary File 9.

An exception is the clear link between higher *Teach_GP* and higher proportion of doctors becoming GP trainees (*Trainee_GP*) (Fig. 4a; *r* = 0.621, *p* = 0.0003). However, interpreting that correlation is complicated by higher *Teach_GP* correlating with doctors performing *less* well at *MRCGP_AKT* and *MRCGP_CSA* (Fig. 4c, e; *r* = − 0.546, *p* = 0.0022 and *r* = − 0.541, *p* = 0.0024), a seemingly paradoxical result.

### Exploring the paradoxical association of greater production of GP trainees, *Trainee_GP*, with poorer performance at MRCGP exams (*MRCGP_AKT*, *MRCGP_CSA*)

A surprising, robust and seemingly paradoxical finding is that schools producing *more* GP trainees have *poorer* performance at MRCGP exams (and at postgraduate exams in general). The correlations of *Trainee_GP* with MRCGP performance are strongly negative (*MRCGP_AKT*: *r* = − 0.642, *p* = 0.00017, *n* = 29; *MRCGP_CSA*: *r* = − 0.520, *p* = 0.0038, *n* = 29), and there is also a strong negative relationship with overall postgraduate performance (*GMC_PGexams*: *r* = − 0.681, *p* = 0.000047, *n* = 29).

A correlation between two variables, *A* and *B*, *r*_*AB*_, can be spurious if both *A* and *B* are influenced by a third factor *C.* If *C* does explain the association *r*_*AB*_, then the partial correlation of *A* and *B*, taking *C* into account, *r*_*p*_ *= r*_*AB|C*_, should be zero, and *C* is the explanation of the correlation of *A* and *B*.

Partial correlations were therefore explored taking into account a range of measures thought to be causally prior to *Trainee_GP*, *MRCGP_AKT*, *MRCGP_CSA* and *GMC_PGexams*. No prior variable on its own reduced to zero the partial correlation of *Trainee_GP* with exam performance. However, *r*_*p*_ was effectively zero when both *Hist_GP* and *Teach_Factor1_Trad* were taken into account *(Trainee_GP* with *MRCGP AKT r*_*p*_ = − 0.145, *p* = 0.470, 25 df; with *MRCGP_CSA*, *r*_*p*_ = − 0.036, *p* = 0.858, 25 df; and with *GMC_PGexams r*_*p*_ = − 0.242, *p* = 0.224, 25 df).^3^

Schools producing more GP trainees perform less well in postgraduate exams in general, as well as MRCGP in particular. Such schools tend to have less traditional teaching (which predicts poorer exam performance and more GP trainees) and historically have produced more GPs (which also predicts poorer exam performance and more GP trainees). As a result, schools producing more GP trainees perform less well in examinations, with the association driven by a history of producing GPs and having less traditional teaching, there being no direct link between producing more GP trainees and overall poorer exam performance.

#### Analysing the broad causal picture of medical school differences

The final, more general, question for the *MedDifs* study concerned how teaching and other medical school measures are related to a wide range of variables, both those that are likely to be causally prior and causally posterior. To keep things relatively simple, we omitted most of the measures related to the medical specialties, and which are shown in Fig. 2, but because of the particular interest in General Practice, we retained *Hist_GP*, *Teach_GP* and *Trainee_GP*, and we also retained *GMC_PGexams*, the single overall GMC measure of postgraduate examination performance. There were therefore 29 measures in this analysis, which are shown in Table 1 in bold. The correlogram for the 29 measures is shown in Supplementary File 1 Fig. S3.

Causality is difficult to assess directly [69], but there are certain necessary constraints, which can be used to put measures into a causal ordering, with temporal ordering being important, along with any apparent absurdity of reversing causality. As an example, were historical output of doctors in a specialty to have a causal influence on, say, current student satisfaction, it would make little sense to say that increased current student satisfaction is causally responsible for the historical output of doctors in a specialty, perhaps years before the students arrived, making the converse the only plausible causal link (although of course both measures may be causally related to some third, unmeasured, variable). Supplementary File 1 has a more detailed discussion of the logic for the ordering. The various measures were broadly divided into ten broad groups (see Table 1), and the 29 measures for the present analysis were additionally ordered within the groups, somewhat more arbitrarily, in terms of plausible causal orderings.

### Path modelling

**Fig. 5:**
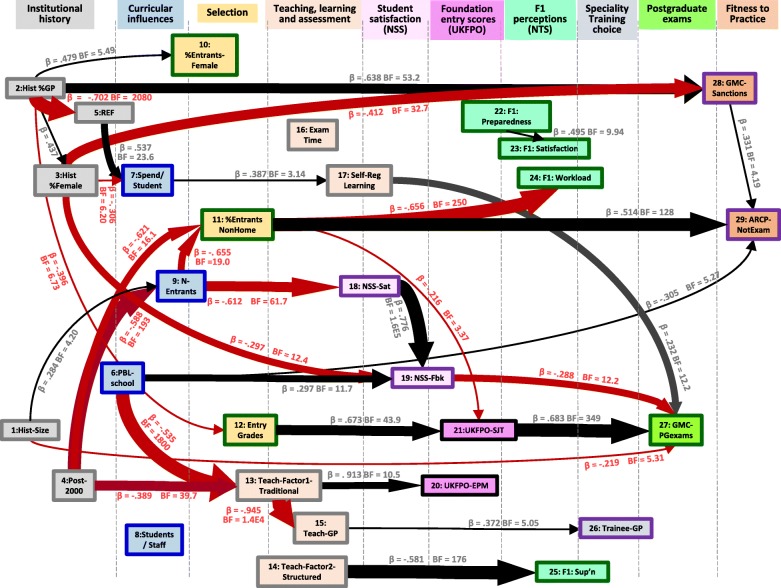
Structural model of the causal relationships of the 29 measures in Supplementary Fig. 2. Very thick lines indicate Bayes factor (BF) > 100, thick lines BF > 30, medium lines BF > 10 and thin lines BF > 3, with black and red indicating positive and negative relationships respectively. Beta and BF are shown alongside each path. For further details see text

A path model was used to assess the relationships between the 29 measures, the final model including only those paths for which sufficient evidence was present (see Fig. 5). The 29 measures were analysed using a series of successive, causally ordered, regression equations (see the “Method” section for more details). Paths were included if the posterior inclusion probability reached levels defined [67] as *moderate* (i.e. the Bayes factor was at least 3 [posterior odds = 3:1, posterior probability for a non-zero path coefficient = 75%]) or *strong* (Bayes factor = 10, posterior odds = 10:1, posterior inclusion probability = 91%). Strong paths in Fig. 5 are shown by very thick lines for BF > 100, thick lines for BF > 30 and medium lines for BF > 10, with thin lines for moderate paths with BF > 3, positive and negative path coefficients being shown by black and red lines respectively. Of 400 paths that were evaluated using the *bms()* function in *R*, there was at least moderate evidence for a non-zero association in 34 (9.0%) cases, and at least strong evidence in 21 (5.3%) cases. In addition, there was at least moderate evidence for the *null* hypothesis being true in 105 (26.3%) of paths (i.e. BF < 1/3), although no paths reached the strong level for the null hypothesis (i.e. BF < 1/10). For the few cases with fewer than four predictors, paths were evaluated using the *bayesglm()* function in *R*, with a conventional 0.05 criterion, and only a single path was included in the model on that basis. Figure 5 therefore contains 35 causal paths. Scattergrams for all combinations of the measures are available in Supplementary Files 3, 4, 5, 6, 7, 8 and 9, Supplementary File 3 containing an index of the scattergrams, and Supplementary Files 4, 5, 6, 7, 8 and 9 containing the 1225 individual graphs.

**Fig. 6:**
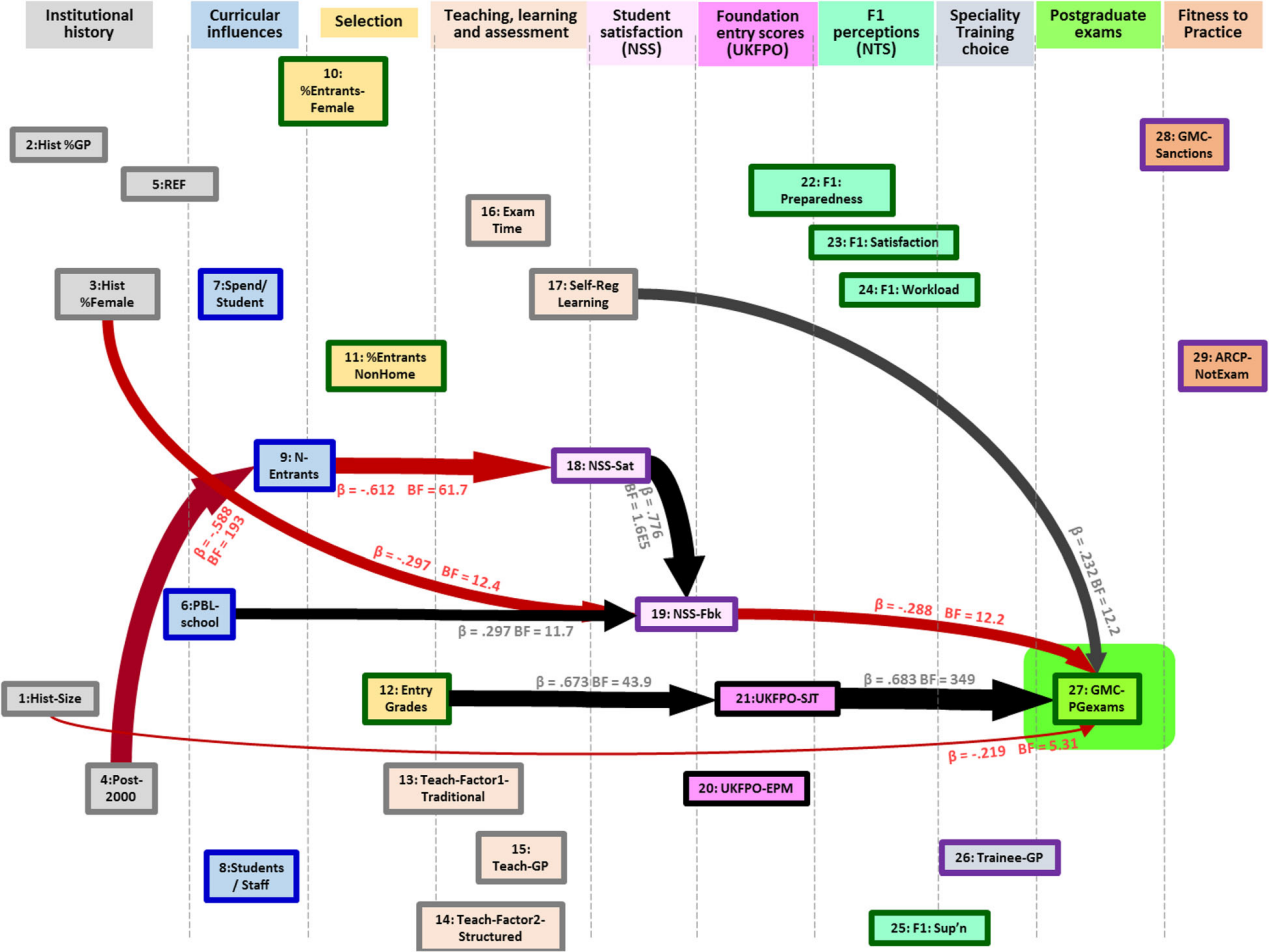
Reduced structural model for performance in postgraduate examinations indicating direct and indirect strong effects on postgraduate performance. All other paths have been removed. For further details, see text

The relations shown in Fig. 5 are generally plausible and coherent, and an overall description of them can be found in Supplementary File 1, and some are also considered in the “Discussion” section. The path model of Fig. 5 can be simplified if specific measures are of particular interest. As an example, Fig. 6 considers the various, complicated influences on postgraduate exam performance (and further examples are provided in Supplementary File 1 Figures S4 to S10). Strong or moderate paths are shown in these diagrams if they directly enter or leave the measure of interest, and indirect paths to or from the measure of interest remain if they are strong (but not moderate), other paths being removed.

### Performance on postgraduate examinations

**Fig. 7:**
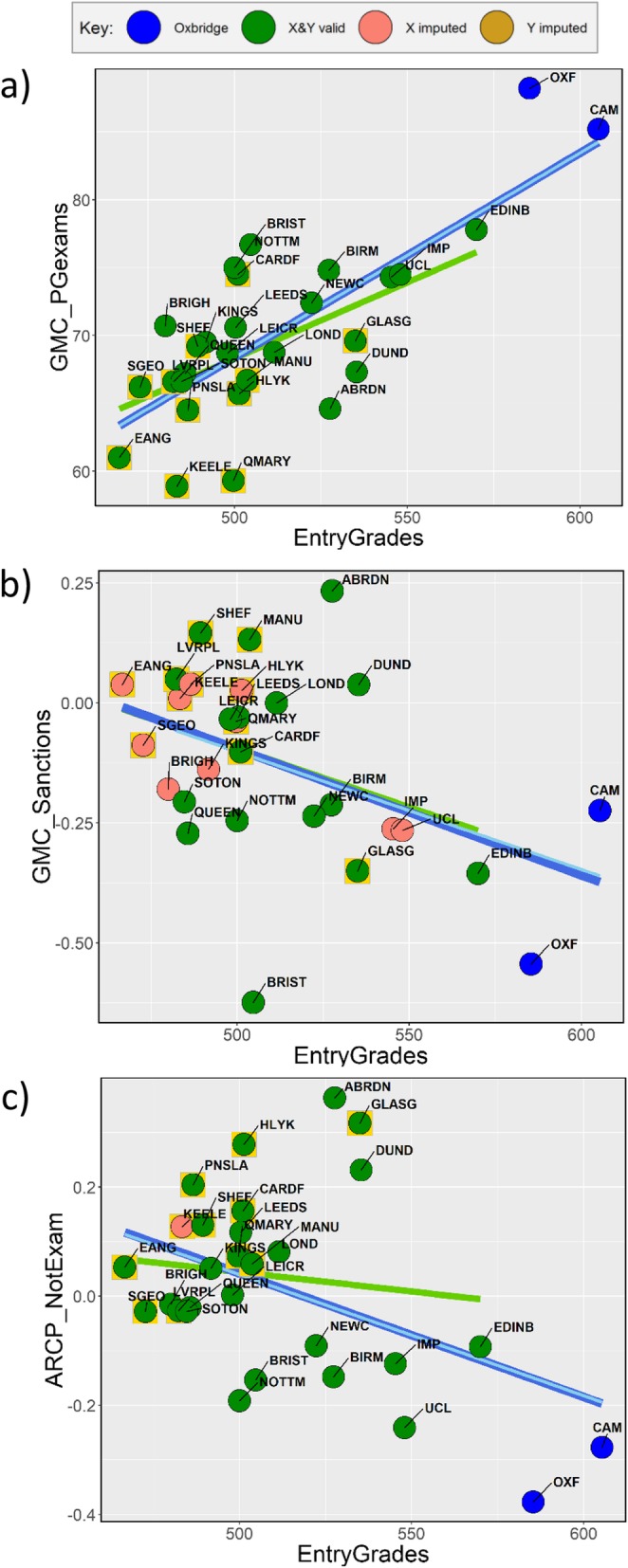
Scatterplots of relations between outcome measures and entry grades. For details of plots, see Fig. 4

*GMC_PGexams* in Fig. 6 is emphasised by a bright green box, and it can be seen that there are four direct causal influences upon postgraduate examination performance from prior measures, but no influences on subsequent measures. The largest direct effect is from *UKFPO_SJT*, which in turn is directly affected strongly by *EntryGrades*, a pattern that elsewhere we have called the “academic backbone” [70]. Entry grades are of particular interest, and Fig. 7 shows scattergrams for the relationship of *EntryGrades* to *GMC_PGexams*, as well as to GMC sanctions for fitness to practise issues (*GMC_Sanctions*) and ARCP problems not due to exam failure (*ARCP_NonExam*), the latter two being discussed in Supplementary File 1. Entry grades therefore are predictors of exam performance, but also of being sanctioned by the GMC and having ARCP problems, all of which are key outcome measures for medicine.

Self-regulated learning (*SelfRegLearn*), which is an interesting although little studied measure [71], has a strong direct effect on *GMC_PGexams*, more self-regulated learning relating to better postgraduate exam performance. Self-regulated learning may be an indicator of the independent learning which medical schools wish to inculcate in “life-long learners” [72], and may also reflect the personality measure of “conscientiousness” which meta-analyses repeatedly show is related to university-level attainment [73].

The historical size of a medical school (*Hist_Size*) relates to *GMC_PGexams*, but the effect is negative, larger schools performing less well at postgraduate assessments, the effect being moderate. The explanation for that is unclear, but it cannot be due to any of the other measures already in Fig. 6 or those effects would have mediated the effect of historical size.

The last remaining direct effect, *NSS_Feedback*, is particularly interesting and shows a strong negative effect (shown in red) on *GMC_PGexams*. *NSS_Feedback* is itself related to overall satisfaction on the National Student Survey (*NSS_Satis’n),* which is related to the number of entrants (*Entrants_N*), and which in turn is related to *Post2000*. Care has to be taken in interpreting chained effects such as these, effects in series being multiplicative, two negatives making a positive in the path algebra. As a result, the chain from *Entrants_N* to *NSS_Satis’n* to *NSS-Feedback* to *GMC_PGexams* is positive (*negative* × *positive* × *negative* = *positive*), schools with larger numbers of entrants performing better at postgraduate examinations. Similarly, *Post2000* schools do less well at postgraduate exams as the path has three negatives and one positive and hence is negative (*negative* × *negative* × *positive* × *negative* = *negative*). *NSS-Feedback* also has two other direct effects upon it, a positive effect from *PBL_School* and a negative effect from the historical proportion of females (*Hist_Female*). The three direct effects upon *NSS-Feedback* are in parallel and hence additive (although signs can mean that they cancel out by acting in different directions as with *Hist_Female* and *PBL_School*).

### Exploring NSS-Feedback scores

**Fig. 8:**
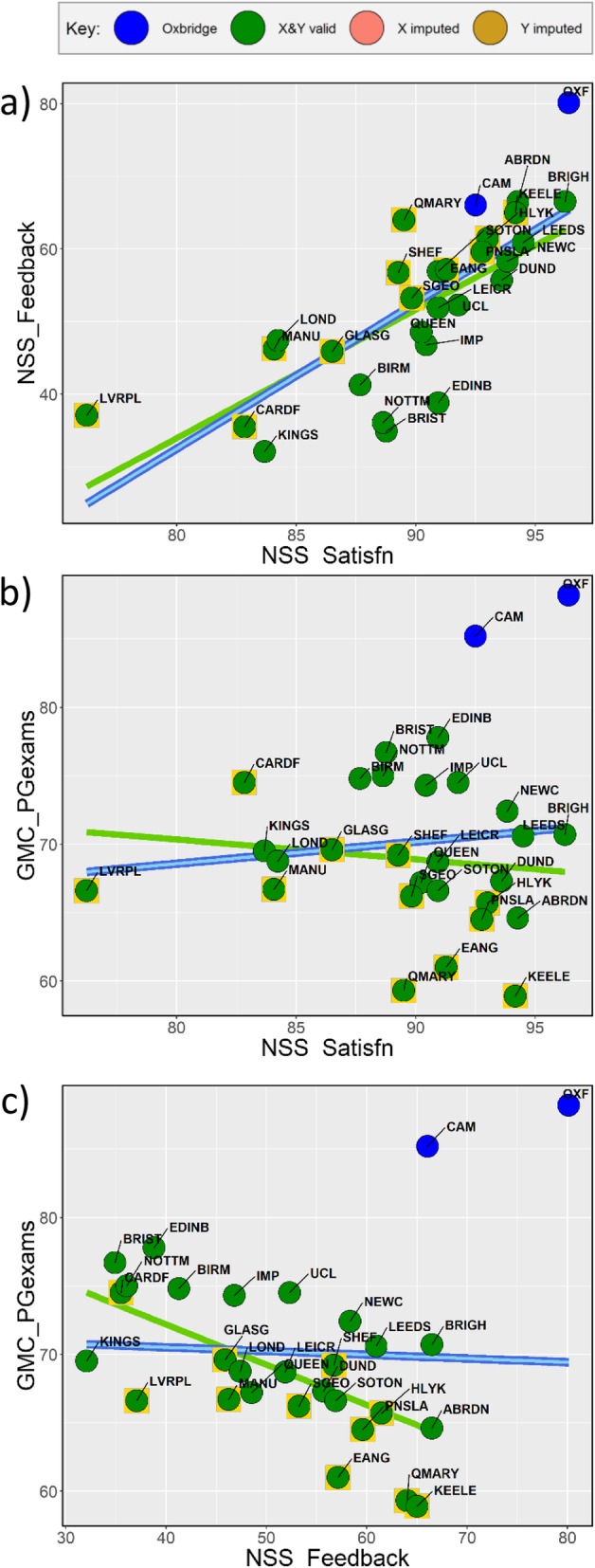
Scatterplots of relations between NSS measures and postgraduate exam performance. For details of plots, see Fig. 4

The finding that schools with higher NSS-Feedback scores have *less good* postgraduate exam results is perhaps surprising and unexpected and merits further exploration. Figure 8 shows scattergrams for the relationships between *NSS_Satis’n*, *NSS_Feedback* and *GMC_PGexams*. There is a strong overall correlation in Fig. 8a of *NSS_Satis’n* and *NSS_Feedback* of 0.762 (*p* < 0.001) showing that they share much but not all their variance (blue line). Although overall *GMC_PGexams* shows no correlation with *NSS_Satis’n* (*r* = 0.108, *p* = 0.578, blue line, Fig. 8b) or *NSS_Feedback* (*r* = − 0.049, *p* = 0.803, blue line, Fig. 8c), the scattergrams, particularly of *GMC_PGexams* with *NSS_Feedback*, strongly suggest that Oxford and Cambridge, in blue, are outliers, each having very high ratings both for *GMC_PGexams* and for *NSS_Satis’n*. Excluding the two Oxbridge schools, there is a significant correlation of *NSS_Feedback* with *GMC_PGexams* (*r* = − 0.621, *p* = 0.0005, green line, Fig. 8c) and a stronger relationship with the residual of *NSS_Feedback* after partialling out *NSS_Satis’n* (*r* = − 0.758, *p* = 0.000005). In general, it does therefore seem to be the case, excluding the important exceptions of Oxford and Cambridge, that greater satisfaction with NSS-Feedback results in poorer postgraduate examination performance. Notice also in Fig. 8c that the PBL schools, in yellow, are mostly all below the non-PBL schools on *GMC_PGexams*.

Summarising Fig. 6, higher postgraduate performance in a medical school is related to nine prior measures, higher exam performance relating to higher UKFPO-SJT marks, higher entry grades, having more self-regulated learning, less NSS overall satisfaction and satisfaction with feedback, not being a post-2000 school or a PBL school and being a school with more entrants or which is historically larger. Many of those effects are mediated via other effects, as seen in Fig. 6.

## Discussion

Although claims are still often made for differences between medical schools, sometimes on an anecdotal basis by those at particular medical schools [74], few claims are systematic, evidence-based and generalisable across the range of medical schools. The data in the present study inevitably are complex, with multiple measures from different medical schools, but they can help to answer a range of substantive questions about medical education, which are discussed below. The data do however also raise various methodological questions which should be discussed first.

### Methodological issues

#### Effect sizes

Whenever studies compare groups of individuals, as here where they are medical schools, or other groups such as countries, it is always the case that variation between groups is always much smaller than variation within groups. As an example, average income varies between the richest and poorest nations on Earth, but such variation is small compared with the variation in individual income within a country such as the USA, where poverty co-exists with large numbers of millionaires and billionaires. The equivalent is true for medical schools, between-medical-school variation invariably being much less than within-school variation.

#### Number of medical schools and statistical power

A practical problem for any comparison of medical schools is that in some countries there are relatively few of them, and comparison across countries is even harder, and we are aware of no comparable studies to the present one in the USA or elsewhere. The UK currently has over thirty medical schools, and that number is growing, but even thirty is a small number when searching for correlations between selection and teaching measures and postgraduate outcomes. With an *N* of 30, correlations need to be greater than about 0.35 to have a reasonable power of being significant, accounting for about 12% of variance, so that smaller effects will be hard to find. With many different variables in the analysis, particularly with more variables than medical schools, there is a risk of type I errors, which we have tried to minimise, using the Tukey adjustment for frequentist statistics and a Bayesian approach in fitting regression models. We have also restricted the number of variables to those of likely practical, educational or political importance, particularly for certain specialties, or for addressing key theoretical issues, as with the role of PBL and teaching styles in medical education.

#### The outcome measures

The postgraduate outcome measures in the study are inevitably limited: examination performance, specialty choice, ARCP problems and GMC sanctions, and NTS perceptions. These obviously do not reflect the very many behaviours and roles of doctors—running wards and clinics, consulting and prescribing habits, team-working, communication with patients and staff, including leadership and advocacy, etc. They should all have been included but cannot be as there are simply no systematic, large-scale measures available for students, trainees or the medical workforce. Such comparative measures are sorely needed, but without them, they cannot be analysed.

#### The reliability of aggregated data

Within UK medical schools, the average number of graduates in a single year is reasonably large, typically about 250, but that is a small *N* for estimating, say, the output for smaller specialties such as psychiatry. Small numerators and denominators inevitably result in unreliable measures. Most of our measures are therefore aggregated across a number of years, and for these, the reliabilities of between-medical school differences are reasonably high, with a median of 0.84. A clear exception was the production of psychiatry trainees, with a reliability assessed over four successive years of only 0.47. Such a low reliability probably makes unsafe claims such as “The University of Keele has produced on average more than double the percentage number of psychiatrists than the University of Cambridge since 2014” [17]. It should also be remembered that lower reliabilities inevitably attenuate correlations, making it harder to reach the levels necessary for correlations to be detected statistically (and output of psychiatrists correlates significantly with only seven of the other variables in Fig. 2 at the raw *p* < 0.05 level, and with no others at the Tukey-adjusted criterion of *p* < 0.0025). In aggregating data across years, there is also the potential problem that schools themselves may be changing, either as courses change within a single school or sometimes by the merger or fission of schools. In an ideal world, data would be modelled within single cohorts of entrants and graduates into specific courses, comparing across cohorts to assess stability, and combining within-cohort causal relationships to gain statistical power (and also additional leverage on causal mechanisms). It should also be emphasised, though, that many medical school differences are surprisingly stable, often across decades, as with the relative output of GPs [19] or performance on the MRCP (UK) examinations [10]. Institutional inertia may well result from the four decades or so that professional careers last, as well as stable differences and traditions within local healthcare provision by hospitals, general practices and public health. Together, such factors create an institutional and a regional ethos, reflected in attitudes and educational philosophies of staff, which then manifests in graduates, and may well also be perceived by applicants, with self-selection then reinforcing institutional differences.

#### Effects at the level of the medical school and the individual

All of the effects in the present study, and it cannot be emphasised sufficiently, are at the level of medical schools. Effects at the medical school level can be conceptually and statistically separate from effects at the level of individuals, but can also be similar [75]. We note especially that schools performing well in particular postgraduate examinations tend to perform well in other postgraduate examinations, which is similar to individual-level data showing that in those cases where doctors take two postgraduate examinations, such as MRCGP and MRCP (UK), better performance on one exam correlates with better performance at the other [76]. Similarly, a study at the student/doctor level has shown that doctors becoming GP trainees have lower entry qualifications than other graduates [77], an effect also shown at the medical school level (see Fig. 2). Care though should always be taken from generalising effects at one level to effects at another, and the risks of Simpson’s paradox and the ecological fallacy are always present [78].

#### Open naming of medical schools

The present study is only possible because the names of medical schools are published, as otherwise it would not have been possible to link data across sources. There is however a reluctance of many medical schools to have information published about themselves, to the extent that Freedom of Information (FoI) requests were therefore used in AToMS, as has also been the case for in another UK study which needed apparently straightforward statistics [32]. We have incorporated some data that were provided to us from other studies on the basis of non-naming of schools, and we have respected such requests, despite recognising that it potentially makes problems for future researchers. However, the data for all of the main measures used here are available in Supplementary File 2 for secondary analysis by other researchers.

#### The nature of the UKFPO situational judgement measure

A difficult theoretical and methodological issue concerns the interpretation of *UKFPO_EPM* and *UKFPO_SJT*, the latter notionally being a measure of *non-academic* attributes [79], whereas *academic* attributes are assessed by *UKFPO_EPM*. The issue is particularly crucial as the UKFPO measures are the only published assessments of academic performance of students while at medical school. In its full current form, which is the only version in the public domain, the Educational Progression Measure combines deciles, degrees and publications, making it a hybrid measure of two sorts of academic attainment: deciles which are *locally* normed *within* medical schools [42, 43] and hence should show minimal variation across medical schools, and degrees and publications which are *between* school measures of intercalated degrees and publications and therefore are *nationally* comparable. Much likely variance in educational attainment between schools is therefore not available in the UKFPO-EPM, reducing its power to correlate with other measures. Despite that, UKFPO-SJT and UKFPO-EPM do correlate highly across schools (*r* = 0.53), suggesting that UKFPO-SJT is mostly acting as if it is a measure of academic attainment, and we have interpreted it in those terms, treating it as part of the academic backbone.

A large-scale meta-analysis of SJTs [80] has shown an important moderating effect of SJT question type. The UKFPO-SJT almost entirely uses “knowledge instructions” [80] (e.g. “Rank in order the *appropriateness* of the following actions …” [our emphasis]) and only rarely uses “behavioural tendency instructions” [80] (e.g. “Rank in order the extent to which *you* agree with the following statements” [our emphasis]), behavioural tendency instructions occurring in only one of 200 example questions. Knowledge instructions are also typical of the SJTs used by the University Clinical Aptitude Test (UCAT; previously UKCAT) and HEE’s MSRA (Multi-Specialty Recruitment Assessment for selection into GP and other specialties).

In the meta-analysis [80], “knowledge” SJTs correlated highly with cognitive ability measures, with little incremental validity of their own, whereas “behavioural” SJTs correlated less with cognitive ability and had greater incremental validity. At the individual level, UKFPO-EPM and UKFPO-SJT correlate about 0.32, similar to the meta-analytic correlation with “knowledge” instructions (mean of 69 correlations = 0.32, SD = 0.17, *n* = 24,656) and higher than correlations with “behavioural” instructions (mean of 26 correlations = 0.17, SD = 0.13, *n* = 6203).

The UKFPO-SJT might be expected to ask about behavioural propensities, as it is “designed to assess for … key attributes … including commitment to professionalism, coping with pressure, effective communication, patient focus, and working effectively as part of a team” [81]. However, the knowledge instructions mean that it is does not ask what individuals *would* do, but instead is acting as a test of what doctors *should* do, as laid down in Good Medical Practice, with material studied, revised and learned as for other assessments.

The meta-analysis supports the view that SJTs are acting mainly as typical academic assessments, and the UKFPO-SJT is therefore part of the academic backbone in Fig. 5, with UKFPO-EPM not being included because of its local standardisation. The UKFPO-EPM measure across medical schools in Fig. 5 is essentially only assessing degrees and publications and shows a causal relation to a traditional approach to teaching in larger medical schools, perhaps because of the greater research opportunities at such schools.

#### Causal inter-relationships of the measures

The correlogram in Fig. 2 shows that many between-school measures are correlated, and path modelling provides a useful way of interpreting such relationships, not least as it is unlikely that measures earlier in time or within educational process can be caused by events later in time. Not all paths can be interpreted in such ways, and some are equivocal in their interpretation, but the broad picture is mostly clear. The 35 paths in Fig. 5 appear complicated, but social processes are rarely simple, and the reasons and the mechanisms for differences between institutions as complex as medical schools are unlikely to be explained by just a very small number of relationships.

The present study is essentially an exercise in epidemiology, attempting to assess causal relations by measuring correlations, using criteria similar to those of Bradford Hill [82]. Causality is certainly unlikely when correlations approach zero (and of the 400 possible causal relations assessed in constructing Fig. 5, there was moderate evidence for *the null hypothesis* being true in 105 (26.3%) of paths (i.e. BF < 1/3), the null hypothesis being three or more times more likely than the alternative hypothesis). The absence of a correlation in such cases implies the absence of a causation. The converse however is far less straightforward, and even strong correlations can be the result of unmeasured confounders or mediators. A strong method for assessing true causation is a random intervention, as in randomised controlled trials in clinical medicine. Such trials are in principle entirely possible within medical education [83], as indeed they are in many areas of education and social science more generally [84] but there has been a reluctance on the part of medical education to implement them, in some cases because of ethical and legal concerns. Nevertheless, RCTs within schools, or cluster-randomised trials across schools, are capable of answering questions of causality and may be desirable in medical education. As it is, correlational and modelling studies such as the present one are the best approach to causality that is possible, although Bayesian [causal] network models can also be of great use [65, 85, 86] in identifying properly causal relationships, and are closely related to the structural models used here [64].

#### A database of UK medical school course descriptors

There is an ever increasing demand for adequate statistics describing individual universities and courses, and the Office for Students (OfS) has recently announced that it will publish a wide range of information concerning student demographics and access statistics relevant to widening participation [87]. Similarly, a number of applications to use the UK Medical Education Database (UKMED) [6] have wished to consider medical school differences, and a systematic database of UK Medical School Course Descriptors would be useful, with data at the level of medical school courses. The unit of analysis will be medical school *courses*, so that, unlike the present study, information will be available for the various different types of medical school courses currently available (standard entry medicine, graduate entry medicine, etc.) [56]. The database of descriptors would be available for researchers to access and could include historical data on medical schools in past years.

### The specific and the general questions

The three specific questions and the general question will now be discussed.

#### The extent to which preparedness is an important predictive variable

This paper began by considering the GMC’s position paper on the specific question of differences in preparedness between medical schools [1], and it is therefore worth now returning to the various thoughts of the GMC. That there are differences in preparedness between medical schools is clear, with a reliability of 0.904 across schools (Table 1), and it seems right therefore “to debate whether the variation between schools in graduate preparedness is a problem” [1]. Preparedness correlates with attending smaller medical schools with less traditional teaching. However, it has no relationship to any of the outcome variables (see Fig. 2). In the structural model of Fig. 5, preparedness has no direct causal relations to other measures (see also Supplementary File 1 Fig. S6), and the only effect on subsequent variables is on *F1-satisfaction*, which was measured at the same time, and halo effects may well be present. It is possible that preparedness relates to important other variables that are not in the present study and not readily available to research, but at present, there would seem no major evidence that differences in preparedness, as measured, are a problem, despite medical school differences clearly being reliable. Although the GMC suggested preparedness differences may be related to NSS measures, Fig. 5 shows no evidence for that suggestion. Overall, while preparedness does differ between schools, there is no evidence of a relationship to major outcome variables. Further work is needed to explore whether preparedness matters or not, particularly in actual behaviours of F1 and F2 doctors in clinical practice, but currently, it is perhaps premature to suggest that such differences “highlight problematic issues across medical education … perhaps with causes that can be identified and addressed” [1].

Having said all of the above, it does seem unlikely that being better or less well prepared for Foundation posts is not important. The implication is therefore that the problem is with the measurement itself, which consists of agreement with a single question, “I was adequately prepared for my first Foundation post”. Foundation posts consist of a vast range of skills to be carried out, from practical procedures through communication skills to history taking, prescribing, diagnosis and so on. To summarise all of that on a four-point scale seems optimistic. Compare it with the questionnaire study of Illing et al. [17] which asked final-year medical students about preparedness in a wide range of specific areas, 22 clinical and practical skills (e.g. arterial blood sampling), 9 communication skills (dealing with difficult and violent patients), 11 teaching and learning skills (e.g. prioritising tasks effectively), 6 work environment (e.g. using knowledge of how errors can happen in practice and applying the principles of managing risk) and 7 team-working skills (e.g. handing over care of a patient e.g. at the end of a shift). Later, the same participants were asked about experience of 15 directly observed procedures (DOPS) and 16 work-place-based assessments, most of which they might reasonably have been expected to have observed during undergraduate training. A single four-point scale somehow does not address the richness and range of such topics. The idea of questionnaires listing a range of conditions, operations and procedures that students might have been seen, on in some cases performed, is not new, with questionnaires in the late 1970s containing 13 procedures [88] and 10 operations [89], in the mid and late 1980s covering 20 conditions, 18 operations, and 29 procedures [90], repeated in the mid 1990s [91], with evidence overall of a general decline in experience across the decades. The Illing et al. study fits in that tradition. If the GMC really wishes to have systematic evidence on the experiential preparation of medical students for Foundation training, then it should consider commissioning routine studies of final-year medical students to discover what is actually being done by clinical students in wards, clinics and general practices; in effect an Undergraduate National Training Survey. A key point is that merely having clinical knowledge from a textbook, as mostly is assessed by final examinations (and indeed in the future UKMLA), is likely to correlate minimally with variation in the lived experience of clinical medicine in actual practice [92]. Preparedness may well be an important way in which undergraduates differ, and probably medical schools also differ, but could be too multifaceted and too varied to be captured by a single tickbox.

#### The effects of problem-based learning

PBL schools differ in a number of ways from other medical schools, summarised in Fig. 1, with detailed differences in teaching methods and content also described in the *AToMS* paper [22].

Cavenagh, in comparing traditional and “new” (i.e. mostly problem-based learning) curricula, stated forcefully that:

The big question of medical educators, the medical profession, their regulating bodies and indeed all patients is how successful has the new curriculum been in reducing stress levels in medical students, creating a learning environment conducive to active lifelong learning and producing well-rounded and competent doctors with humanitarian attitudes towards their patients? [93] (p. 19).

Cavenagh answers that question positively, albeit with relatively little large-scale evidence, but it is also emphasised that,

… *our first concern must be that doctors are clinically competent, practise evidence-based medicine and are safe practitioners*. … If this can be delivered within the context of a supportive educational and clinical environment, where medical students are nurtured in a way that feeds their own humanity and encourages their thirst for learning and knowledge, then with effective recruitment strategies a revised curriculum should achieve the aspirations outlined for *Tomorrow’s Doctors* [93] (p. 21, our emphasis).

In term of simple comparisons of our outcome measures, PBL schools have lower scores on UKFPO-SJT and UKFPO-EPM, they report higher preparedness for F1, they are more likely to enter General Practice, they have poorer performance at postgraduate examinations, including MRCGP, and they have higher rates of non-exam problems at ARCP (Fig. 1). Several of those measures are designed to assess aspects of clinical competence, so that Cavenagh’s criterion of being “clinically competent” is seemingly not being met. However, the simple effects in Fig. 1 do not take into account the complex inter-correlations of Fig. 2, which are taken into account in a principled way in the path analysis of Fig. 5. A major predictor of many outcome measures is entry grades, the “academic backbone” [70] whereby higher attaining entrants show higher postgraduate attainment. PBL schools however have lower entry grades (Fig. 1) and therefore might be expected on that basis alone to do less well on postgraduate outcomes. However, even when entry grades and other measures are taken into account (Fig. 6), PBL schools (and post-2000 schools) tend to do less well at examinations.

Exploration of the path model in Fig. 5 suggests that the NSS-Feedback measure is an important mediating variable. The NSS-Feedback measure asks about agreement with four statements: The criteria used in marking have been clear in advance; marking and assessment has been fair; feedback on my work has been timely; and I have received helpful comments on my work. PBL schools have higher NSS-Feedback scores, even after taking NSS-Satisfaction scores into account, but higher NSS-Feedback scores are in turn related to poorer postgraduate exam outcomes. The scattergrams of Fig. 8 show a clear and significant relationship, although Oxford and Cambridge are undoubted outliers. It is possible that high satisfaction with feedback reflects a more supportive educational environment, and perhaps also one in which it is difficult to acquire realistic self-assessments of ability, which later results in problems in the less supportive, harsher, postgraduate learning environment.

Research into the role of feedback in education has been influenced by the work of Dweck and colleagues [94], who have argued that individuals differ in beliefs about their own ability, having either a “fixed mindset” or a “growth mindset”, with the former having beliefs in innate ability and the latter believing in the ability to grow and change. Crucially, Dweck has argued that different types of feedback can result in different mindsets, feedback emphasising talent or ability resulting in fixed mindsets and feedback emphasising hard work and effort reinforcing a growth mindset which is more willing to take on new and more difficult challenges [95]. It is a possibility therefore that feedback in medical schools differs according to teaching styles and induces different mindsets. More research is undoubtedly needed on the details of how teaching, learning and feedback take place in medical schools. Having said that, Dweck’s research is still controversial, one major study being unable to replicate the key claims about the large effects of different types of feedback [96], and another suggesting that the effect sizes suggested by the original studies can mostly not be replicated [97]. A further study has also included measures of the growth mindset within a wide range of attitudinal, background and other measures [98] and shows the growth mindset to be highly correlated with “grit” and in particular the Big Five measure of conscientiousness, which has repeatedly been shown to correlate with academic success [73, 99]. Feedback and responses to feedback may therefore be dependent on differences in personality, perhaps with students at different schools differing in personality as a result of self-selection. Elsewhere, we have shown that students who like PBL show higher scores on conscientiousness and openness to experience, as well as different learning styles, being higher on deep learning [100]. Clearly, there is much here on the role of feedback that needs further investigation, at the level of schools and of students, perhaps using mixed-methods, as the negative relationship between satisfaction with feedback and subsequent exam performance needs explanation.

As well as effects on postgraduate exam performance, PBL schools have a moderate direct effect on ARCP non-exam problems, which are more frequent in graduates of PBL schools and which are not mediated via NSS-Feedback. The mechanism therefore is unclear.

Cavenagh also mentions “effective recruitment strategies”, the implication being that PBL schools have sometimes found it difficult to recruit medical students, which itself may be a cause of somewhat lower entry qualifications than for more traditional schools (Fig. 1). Our data cannot take apart how applicants choose to apply to particular schools, but it may be that PBL schools specifically, or newer schools more generally, have poorer reputations amongst applicants, and hence are less likely to attract high-flying applicants.

On Cavenagh’s broader criteria, we note that PBL schools do not differ from non-PBL schools on our measure of self-regulated learning (Fig. 1), which might be expected to relate to “a learning environment conducive to active lifelong learning”. We know of no data which can ask at present about stress levels (although that is included now in NTS), or about well-roundedness or humanitarian attitudes.

#### The teaching of specific specialties, and increasing the number of GP and psychiatry trainees

An important recent claim, quoted earlier, is that curricula steeped in general practice and psychiatry produce more working GPs and psychiatrists in the future [16, 17]. For psychiatry, the relationship of teaching hours to trainee numbers is negative and non-significant (Fig. 4b), and neither do anaesthetics or surgery show significant effects (Fig. 4d, f). There is little support in general therefore for the suggestion that more teaching of specialties results in more trainees in those specialties, and specifically for the case of psychiatry. However, General Practice is a clear exception, and both this study and another [26] using a different method have found increased numbers of GP trainees from schools with more GP teaching (Fig. 4a). However, while schools teaching more GP do indeed have *more* graduates entering GP training, potentially problematic is that the graduates of those schools also perform *less well* in the MRCGP examinations (Fig. 4c, e). That apparent paradox can be explained by schools which teach a lot of GP also tending to use non-traditional teaching, which is associated with lower examination performance, and those schools also tending to have a history of producing GPs, which also is associated with lower examination performance. It is not therefore teaching a lot of general practice which makes graduates perform less well, but background factors that are correlated with performing less well in examinations.

For the present data, the mean percentage of GP trainees from a school is 19.5% (SD 4.6%), based on an average of 342 (SD 149) hours of GP teaching. Using regression analysis, an extra 100 h of GP training results in a 1.91 percentage point increase in the proportion of GP trainees. Taking schools near the extremes, with 150 or 600 h of GP teaching, the predicted proportion of GP trainees is 15.9% and 24.5%. If *all* schools were to have 600 h of GP training then, ceteris paribus, the overall proportion of GP trainees would increase from the current mean of 19.5 to 24.5% of graduates, a relative increase of 5.0 percentage points (1.25×; 25% increase). Broadly similar figures can be calculated from the other study of GP teaching [26]. Of course, nearly doubling the amount of GP teaching nationally, from 339 to 600 h, would not be easy, as GPs and GP teaching are finite resources, which are already overloaded [101]. Whether increasing the number of GP trainees by 5 percentage points (25%) would be sufficient to ameliorate the current shortage of GPs requires further modelling, particularly if other teaching were to decrease, perhaps with unintended consequences, and also were there to be more GP trainees failing MRCGP examinations or having ARCP or FtP issues [102] (although such outcomes can be modelled [19, 103]). Greater exposure to general practice could also merely confirm for many students that they definitely have no intention of a career in general practice [104].

#### Analysing the broad causal picture of medical school differences

The three specific questions which have been raised are all subsets of a much larger, broader set of questions, the answers to which are summarised in Fig. 5, and ask about how earlier measures are related to later measures, perhaps through intervening or mediating variables. Figure 5 therefore answers many possible questions in the same way as a map of the London Underground answers questions about the routes between many possible starting points and destinations. Figure 5 is in many ways the conceptual key to the entire paper and to the research enterprise, summarising a lot of information, with only paths that are likely to be important being included, with different line widths summarising the strengths of relationships. What also is of importance in Fig. 5 is what is missing—the absence of lines between measures tells one what is not happening (and there are far more absent lines than present ones). To take one example, consider student-staff ratio in the lower left-hand corner of Fig. 5. Student-staff ratios are reported by all of the student guides such as those published by the *The Times* and *The Guardian* newspapers. The implication is that they matter in some way and that some medical schools are better than others on that criterion. Certainly, medical schools differ on it and those differences are reliable. But nothing causes differences in student-staff ratio in Fig. 5, and neither does student-staff ratio cause any of the measures to the right of it. It is a difference which seems not to make a difference. One might easily have created an elaborate theoretical superstructure concerning why it might be that low student-staff ratios would be good, each student having more staff contact, which would then ripple through into a range of other measures, and might itself be caused by background factors. But in the case of medical schools, there seems to be no relationship of student-staff ratio to anything else. Of course, that claim needs hedging—this may only apply to medicine and not to other university disciplines, and it may only apply within the range of values actually found and for instance would almost inevitably become more and more important as numbers of staff fall and the ratio gets higher and higher. But for UK medical schools over the time window analysed with these data, it does not seem to be important in explaining differences between medical schools. And a similar analysis could be carried out for many of the other measures, seeing what does and what does not affect other measures.

The analyses have looked at differences between medical schools, and Figs. 2 and 5, as well as Table 1, confirm that medical schools differ in many and correlated ways. Some of those analyses were motivated by the GMC’s analysis in particular of differences in preparedness and their discussions about underlying processes and mechanisms. Just as this paper began with the GMC’s report on preparedness so it should perhaps end with a consideration of what that report says about the nature of the difference itself.

### The nature of difference

The GMC report on preparedness raises broad and deep issues about the nature of difference. The GMC correctly identifies that medical school differences undoubtedly “reflect [ … ] the relevant and relative strengths of the graduates applying and progressing” [1]. Entry grades at the medical school level in this *MedDifs* study relate to UKFPO and postgraduate exam outcomes, as well as ARCP and GMC sanctions (see Fig. 6), and at an individual level, differences in academic attainment form the academic backbone from school results through undergraduate assessments [70] lead through to better postgraduate examination performance as well as to lower rates of GMC sanctions [102].

In two complex sentences which elide several propositions, the GMC report states that,

Clearly, events later in a doctor’s career will tend to be less closely attributable to their undergraduate education. In any case, this information is not sufficient to demonstrate that some schools are better than others. [1]

Events later in a career may well be less likely to be influenced by medical schools, but such differences might still be the consequence of genuine medical school differences. If those events later in a doctor’s career are positive or negative, then it surely makes sense, though, to talk of some schools being “better than others” [1].

Mere differences between medical schools, though, do not mean that the differences are explicitly due to the schooling in those schools. In secondary education, it is recognised that most differences between secondary schools are a result of differences in intake, so that a key question concerns the value-added by secondary schools, given the ability levels of those admitted. The GMC in part also takes that line, so that it may be “relevant to consider the value added by the medical school taking into account the potential of the students they enrol” [1]. That statement recognises that entrants differ in potential, which probably is most easily considered in terms of prior academic attainment, albeit taken in its educational context [105, 106].

The previous Chair of the GMC, Sir Terence Stephenson, also recognised the important role of different entry qualifications:

When [medical schools] analyse how their students perform on … core [examination] questions they see … variability between schools, which is perhaps understandable – different schools have different entrance requirements. *People who are good at passing A levels will probably be good at passing [later assessments]* [18] (Our emphasis)

He continued, though, to ask whether medical school differences in A-level entry grades are themselves acceptable, as medical schools,

have different standards set at admission, and that’s more worrying, that people think the standard in one place should be this, and in somewhere else should be that [18].

That statement hides a radical proposal. If it is desired that average entry standards should indeed be identical for all medical schools, then that presumably could only be ensured by random or quota allocation of appropriately qualified applicants to medical schools. Without random allocation, differential application by applicants would almost inevitably result in higher qualified applicants choosing to apply to schools of perceived higher status and performance [107]. Turning the argument around, it could instead be argued that differences in average entry standards are not a problem in so far as postgraduate outcome variables also relate to those different entry standards (Fig. 7). If so, that would mean that the primary problem is that qualification rates from different medical schools are very similar, which perhaps makes little sense given differences in both entry standards and postgraduate performance. The then chair of the GMC said that there needs to be a solution to the problem of there currently being “36 different ways of qualifying as a doctor in the UK” [18], with standards at finals in effect being set locally rather than nationally. The forthcoming UK Medical Licensing Assessment (UKMLA) has been suggested to be an important part of the solution to that problem [9], but it could, and perhaps should, result in raised failure rates at some medical schools. Ultimately squaring the circle of different entry standards, different postgraduate performance and equivalent qualification rates are impossible unless one of the three changes radically.

As with other questions in medical education, assessing whether medical schools genuinely differ in the amount to which they add value over and above entry differences is complex. It might be tempting to conclude that a measure of “value added” could be derived from Fig. 7a by assessing the extent to which schools are above or below the regression line, schools above the line seemingly adding more value to equivalently qualified entrants than those below the line. That would be a start, but it cannot take into account that different students may apply to or be selected by medical schools for a host of non-academic or non-cognitive reasons (such as location, course type etc.). Neither are data on secondary school or medical school differences in selection tests, such as UCAT, BMAT and GAMSAT, available publically at present. An answer may be found in a randomised control trial, where applicants of equivalent academic attainment and who have already applied to two schools above and below the regression line in Fig. 7a are randomly allocated to one or other of those schools. Although ethical objections to such a study may be found, it is surely less problematic than randomly allocating patients to sham surgery, radiotherapy or cytotoxic drugs. At the very least, RCTs should be seriously considered, particularly given the use of RCTs for assessing an ever-widening set of social issues [84].

While considering differences, the GMC also raises another important issue when discussing whether some schools are better than others, saying “That depends on the criteria you use” [1]. Elsewhere, it comments that,

medical schools producing large numbers of GPs are helping to address a key area of concern in medical staffing. The specialties most valued by students or doctors in training may not be the most valuable to the NHS. [1]

The implication seems to be that different medical schools can be good in different ways, returning to the “the individuality of the universities … ” [2, 3] (p.x, para 37) that the GMC had earlier cited from the 1882 Commission. However, those differences may result, say, in an increased output of doctors going into a particular [needed] specialty, but also result in doctors who are less likely to pass the exams of that [needed] specialty, or to have higher rates of GMC sanctions or other problematic behaviour. It is also the case that if quality may be defined in a sufficient number of ways then the eventual likelihood is that all medical schools will achieve more highly on some criterion of quality, resulting in the philosophy espoused by the Dodo in *Alice’s Adventures in Wonderland* that “all shall have prizes”. Maybe, but then it is difficult to see how such relativism can be compatible with there being medical schools with “problematic issues … perhaps with causes that can be identified and addressed” [1]. This is not the place to consider such issues further, but they are important issues that medical education, and the GMC, the regulator of medical education, has the opportunity to be clearer about once the data from UKMLA have been incorporated into UKMED. What is clear is that any answers need to be based on data fully describing the many differences between medical schools.

#### Clarification

We have been asked to make clear, to avoid any possible doubt, that neither this nor the *AToMS* paper is stating or implying that any of the schools detailed are providing a sub-standard education or are otherwise badly run.

## Conclusions

Medical schools differ in many ways, those differences are reliable, and some of the differences matter and can be explored scientifically. Many differences show causal links with other measures, as with academic outcomes in postgraduate examinations reflecting prior attainment differences, at medical school and before (“the academic backbone” [70]). Surprisingly, schools reporting greater satisfaction on the NSS-Feedback measure performed *less* well at postgraduate outcomes; as with all such links, further research is needed to unpack the process and mechanisms underlying such differences. PBL schools differed on 15 of 49 measures. Institutional histories related to some outcomes, such as more GMC sanctions occurring for schools with higher historical proportions of male graduates and GP trainees. Measures were not available for many potentially important outcomes such as leadership, team-working, communication and advocacy, technical skills and research ability, and such data urgently need collecting. Likewise, detailed measures of undergraduate experience would be invaluable, perhaps by a GMC-initiated Undergraduate Training Survey. Preparedness, a measure of particular interest to the GMC, did not relate to our outcome measures, but might relate to detailed behaviours in F1, F2 and later posts. Confirming causality requires interventions, perhaps from time series data within medical schools, or randomised interventions within or across medical schools. As more data comes into the public domain, and numbers of medical schools increase, so the origins of medical school differences should become clearer.

## Supplementary information

**Additional file 1.** Figures S1 to S10, and additional text on the causal ordering of variables.

**Additional file 2.** Excel file of raw data and kNN-imputed data, RawAndImputedData.xlsx

**Additional file 3.** Index and notes on supplementary graphs 1 to 205 (page 1 to 205).

**Additional file 4.** Graphs 1 to 210 (pages 1 to 35).

**Additional file 5.** Graphs 211 to 420 (pages 36 to 70).

**Additional file 6.** Graphs 421 to 630 (pages 71 to 105).

**Additional file 7.** Graphs 631 to 840 (pages 106 to 140).

**Additional file 8.** Graphs 841 to 1050 (pages 141 to 175).

**Additional file 9.** Graphs 1051 to 1225 (pages 176 to 205).

## Data Availability

All of the raw and imputed data used in these analyses are openly available as an Excel file in Supplementary File 2.

## Footnotes

1 A further complication is that for a number of years some schools awarded degrees in partnership with other medical schools, such as Keele with Manchester.

2 From 2000 to 2007, Leicester was named Leicester-Warwick Medical School and statistics will include Warwick graduates. Warwick and Leicester became separate schools again in 2008.

3 Traditional teaching, *Teach_Factor1_Trad*, is negatively correlated with being a PBL school (*PBL_School*) and being post 2000 (*Post2000)*, and partial correlations taking *PBL_School* and *Post2000*, as well as *Hist_GP*, into account also gave non-significant partial correlations (*MRCGP_AKT r*_*p*_ = −.141, *p* = .492, 24 df; *MRCGP_CSA*, *r*_*p*_ = − 0.116, *p* = 0.574, 24 df; *GMC_PGexams r*_*p*_ = − 0.234, *p* = 0.250, 24 df). *Hist_GP* correlates with less good performance in postgraduate exams (*MRCGP_AKT r* = − 0.761, *p* = 0.000002; *MRCGP_CSA r* = − 0.666, *p* = 0.00008; *GMC_PGexams r* = − 0.731, *p* = 0.000007, *n* = 29), and *Teach_Factor1_Trad* correlates with better performance in exams (*MRCGP_AKT r* = 0.664, *p* = 0.000085; *MRCGP_CSA r* = 0.562, *p* = 0.0015; *GMC_PGexams r* = 0.684, *p* = 0.000043, *n* = 29 in all cases). Multiple regression showed that *Hist_GP* and *Teach_Factor1_Trad* were independent predictors of *Trainee_GP*, *MRCGP_AKT*, *MRCGP_CSA* and *GMC_PGexams*.
