# Supplementary material for: Exploring UK medical school differences: the MedDifs study of selection, teaching, student and F1 perceptions, postgraduate outcomes and fitness to practise
Source: BMC Med. 2020 May 14;18:136. doi: 10.1186/s12916-020-01572-3 (PMC7222458; doi:10.1186/s12916-020-01572-3)

Supplementary File: Causal ordering of measures

The 29 measures in main figure 5 are placed in a causal ordering which allows the path model to be fitted. Some explanation is required. It should be noted that in talking of causality it is easier to think in terms of *individuals*, as when it is said that speciality training choices are influenced by events occurring during undergraduate training, but it continually has to be remembered that the present analyses are in terms of *medical schools*, not individuals. Some influences in figure 5 can be at either level (so that individual students giving low NSS scores may also perform less well at postgraduate exams), but that is a separate question from whether students at a school with low NSS scores *as a group* perform less well at postgraduate exams as a group, the effect being institutional rather than individual (so that presumably it would manifest via low levels of morale in the institution rather than dissatisfaction in particular individuals). With sufficient data it would be possible in some cases to use multi-level modelling to analyse measures simultaneously at the individual and the group level (although that would not actually be possible for NSS measures since they are collected anonymously and cannot therefore be related to particular sets of individual examination results). In other cases, measures make no sense at the individual student level, so that the student/staff ratio, whether or not the school is PBL, or even the history of producing GPs, *only* applies at the medical school level, in effect the same student/staff ratio being applied to all students, so that within a school it is a constant[1].

Measures in figure 5 are placed across the page, so that measures to the right can be *caused by* measures to their left, but cannot *cause* measures to their right. The 29 measures are divided into ten broad groupings, for which the causal relations between groups are mostly straightforward. Within the ten categories of measure shown in figure 5 it is convenient also to order measures, so that regression analyses involve measures only being regressed on measures to their left. That is statistically convenient, and sometimes it can be justified causally, but in other cases there is an inevitable arbitrariness to it. As in all path modelling, other researchers may wish to repeat the calculations but making different assumptions about causal ordering. A few comments on specific measures are therefore also provided.

- *Institutional histories* are placed to the far left, as it makes little sense to think that any of the other measures in the system can alter the history of an institution, so that, as ever, chronological order is often a good indicator of causal ordering.
    - Within the Institutional Histories measures much of the ordering is mostly arbitrary, so that a casual story could also be told in terms of Hist-PctGPs being caused by Hist-PctFemales rather than vice-versa[2]. The key thing is not how these two measures are related, but the fact that they are probably both prior to most of the other measures to their right.
    - Mention should perhaps be made of the last two measures, Post2000 and REF. The date of creation of a medical school is inevitably fixed within chronology and there is an argument for it being placed far to the left. However in practice it makes little difference where it goes since most historical measures are structurally missing for

---

[1] It could be argued that, for many reasons, some students are actually exposed to more staff contact than others, either through student or staff choice, or because of choosing particular modules, being allocated to different teaching firms, or whatever; but obtaining data on such differences would be extremely hard, although the HEPI data might give some hints about it.

[2] Once the possibility of such changes is raised then separate research questions are raised, and more detailed analyses of year-on-year variation of the two measures might allow a causal direction to be identified, using methods such as cross-lagged panel correlations, or more sophisticated modelling.

Post2000 schools. Arbitrarily it has therefore been placed to the right. REF, the measure of research activity, is within the Institutional History group, since research activity often requires resources over long time periods, making it difficult to commence entirely new research efforts without the context of previous research. That would have been particularly true had we included ‘research strength’, the overall REF multiplied by the number of staff submitted.

- *Curricular influences* come next, and represent major decisions on the underlying philosophy on which a medical curriculum is based, including whether or not the approach is PBL, but also the underlying infrastructure and resources, represented by the size of the school, funding, and the number of staff, as well as whether the school is a new one. All of these factors will be influenced by the history of an institution, but by little else. Counter-arguments are useful in assessing causality, and for instance it is causally unlikely that entry grades, teaching methods, exam results or student opinions, etc, will change the curricular influences. Postgraduate exam results do not make schools become PBL but being PBL may cause changes in postgraduate exam results[3].
  - PBL school is placed to the left of the group since it is a philosophical decision, usually made once at a particular time, and it influences many other events within a medical school (and in principle could influence spending, staffing numbers, etc..
  - The number of entrants to a school is potentially influenced by many things, including funding, and therefore it is plausible to place it to the right of this group. Although possible it seems implausible that as numbers of entrants increases that causes a school to become PBL (although it may decide that for other reasons).
- *Selection* reflects both the sort of student that a school wishes to select, in terms of student mix and entry grades, and the sorts of school to which students wish to apply[4]. It must always be recognised that selection is a multi-faceted process, individuals choosing to apply to medical school, individuals choosing to which medical schools to apply, schools choosing to which applicants to make offers, and individuals choosing between schools for which they hold offers. Three of the four processes are driven by applicants and not by medical schools (and it is always worth remembering that no medical school can select applicants who have not applied to them). The characteristics of entrants to a medical school are therefore a composite of all four processes, only one of which is directly under the control of medical schools.
  - Entry grades are the right-most of the measures since if male or female students, or home or non-home students, differ in grades then different proportions of male or home students might influence entry grades, but the converse causation seems unlikely (higher grades cannot make an applicant a home applicant or female). Ultimately that causal analysis depends on students deciding where to apply but schools deciding to whom to make offers; if more females apply then, in the absence

[3] Of course if longitudinal data were available it may be that a causal cycle can develop, with schools altering their philosophical approach in relation to perceived causes on effects later in the chain, but that still implies that philosophy at time N affects outcomes at time N+n. Once again, cross-lagged correlations could tease apart those issues were sufficiently time-sliced data available, which they are not at present.

[4] Although it could be argued that entry measures, particularly to do with student self-selection, are influenced by factors such as previous NSS results, or similar, in practice the causality there cannot easily be disentangled. In principle it could be if, say, poor NSS results in one year alter the types of student who apply the next year, but the data are not finely-grained enough to disentangle that problem. It might be possible to analyse data collected on a year-on-year basis, looking at lagged effects to assess such year-on-year causation, but the data are probably not sufficient at present.

of discrimination, more females may receive offers, and if women on average have higher A-levels that might cause entry grades to rise (although in reality there is no evidence for that effect).

- The *Teaching, Learning and Assessment* measures cover the organisation of teaching in the school and are mostly determined by the school, caused by curricular influences, such as finance and staffing, and perhaps also by the type of student admitted.
  - Within *Teaching, Learning and Assessment* it should be noted that the strong relationship between Traditional Teaching and Teaching of GP is firstly a necessary relationship, since the amount of teaching of GP is itself one of the measures contributing to the overall measure of Traditional Teaching, and secondly GP teaching has been placed after Traditional Teaching so that any variation specific to it, rather than to the more generic description of teaching, can be allowed to influence later measures (as indeed it does).
  - Self-regulated Learning is placed to the extreme right of the group, although its placement is difficult. The logic is that while schools can influence self-regulated learning implicitly in many ways, it may well be caused by styles of teaching, etc, but is unlikely to cause changes in those styles of teaching.
- *Student Satisfaction (NSS)* measures are outcome measures representing aggregate perceptions of medical schools by students, influenced by the teaching that the school has provided.
  - Clearly NSS measures must come after teaching, learning and assessment measures, although as with all of the measures here, they might over time influence earlier measures. The responses of a particular cohort of students to NSS can only, though, be caused by the teaching etc that they have received, rather than teaching *for those particular students* changing in response to the NSS responses they will make in the future.
  - The earlier principle of generic measures coming before specific is also used for *the Student Satisfaction (NSS)* measures, where the more generic Satisfaction measure is placed before the more specific measure of Satisfaction with Feedback. That allows anything which is specifically to do with Feedback to appear in the model even after taking overall satisfaction into account.
- *Foundation Entry Scores (UKFPO)* are academic outcome measures caused by all prior measures, including the type of teaching, the types of students admitted, curricular influences and even historical, institutional, influences. Satisfaction measures may also cause lower academic scores, since it is assumed that less happy students may do less well academically.
  - The two *Foundation Entry Scores (UKFPO)* have EPM before SJT, since EPM is an aggregate of examination results throughout undergraduate training, whereas the SJT is a single assessment in the final year of undergraduate training, and therefore occurs later in time.
  - It should also be remembered, as considered extensively in the discussion section of the main paper, that EPM is a compound measure, comprising one locally standardised measure and two nationally standardised measure, meaning that much of its variance is not available for comparison across medical schools.
- *F1 Perception (NTS) scores* are the first properly postgraduate measures, and can be influenced by anything occurring before graduation.
  - The Preparedness measure is placed to the left of the group. Its wording refers to perceptions of medical school training, and in that sense it is prior to the other

    measures. However in reality Preparedness is asked about at the same time as the other three measures and there may well therefore be halo effects, and so causal ordering should be treated with care.
    - As with the NSS measures, F1_Satisfaction measure is placed to the left of Workload and Supervision, on the basis that generic measures are placed before specific measures. The relationship between Workload and Supervision is very unclear causally, but it might be that high workload results in poor perceptions of Supervision, and on that basis measures have been put in that causal order.
- *Speciality training choice* reflects decisions made at the end of Foundation training, and can be influenced by events occurring prior to that, during undergraduate or F1 training.
- *Postgraduate examinations* are largely taken after entry into specialist training and so can be considered as being caused by earlier choices, rather than causing those choices[5].
- Finally *Fitness to Practise* measures have been considered as occurring after postgraduate examinations have been taken. That is not always the case, but FtP sanctions involving the GMC (ESCUW) mostly occur later during professional careers[6]. That is not the case for non-exam-related ARCP issues, but for convenience they have been placed along with GMC sanctions.
    - The two *Fitness to Practise* measures, as already noted, are difficult to order causally. The measure of GMC sanctions, because ESCUW events are scarce (but nevertheless important), is aggregated across doctors graduating since 1990, for events occurring from 2008 to 2016. There might be an argument that they are an historical measure, but their very stability across time also means that it makes possible sense to interpret them in relation to current and recent medical school measures[7]. In contrast, non-exam related ARCP problems are indeed recent, for events from 2010 to 2014, and therefore can at least be placed to the right of GMC sanctions.

---

[5] It has to be recognised though that a number of doctors choose to take MRCP(UK) examinations during Foundation training, and their performance in those exams may itself help in deciding whether or not to apply for Core Medical Training or other specialties. That consideration does not apply to exams such as MRCGP which can only be taken after several years in GP training. Once more a more finely-grained analysis could in principle sort out the details of causality, but it is not possible given the present state of the various sets of data.

[6] A general issue is that some measures, at the medical school level, involve events happening at discrete moments of time (e.g. the proportion of graduates in a year who apply for GP training) whereas others are rarer events, and are integrated over longer periods of time, perhaps a decade or more with GMC sanctions, which are rare in the immediate years after graduation, and increase in numbers as careers progress.

[7] It can be seen, once more, that proper data would have a host of measures all assessed year-by-year with causal effects moving forward in time, and allowing the effect of measure A at time 1 (A1) on measure B at time 2 (B2) to be differentiated from the effect of measure B at time 1 (B1) on measure A at time 2 (A2.). In principle that is possible but it would be far from easy, particularly when some measures have to be integrated over time because of low rates of events (such as GMC sanctions). Once more a vision of what might be possible can be formed, but the practical realities of such analyses are (far) in the future. Nevertheless they provide a conceptual basis for thinking about the present data.

Supplementary figure S1. A correlogram for randomly generated data of the same size as Figure 1 in the main text, with 50 measures across 29 medical schools. 55 of the 1225 correlations are significant at the 0.05 level (4.5%) and only 2 correlations (0.16%) reached the Tukey-adjusted criterion of 0.0025.

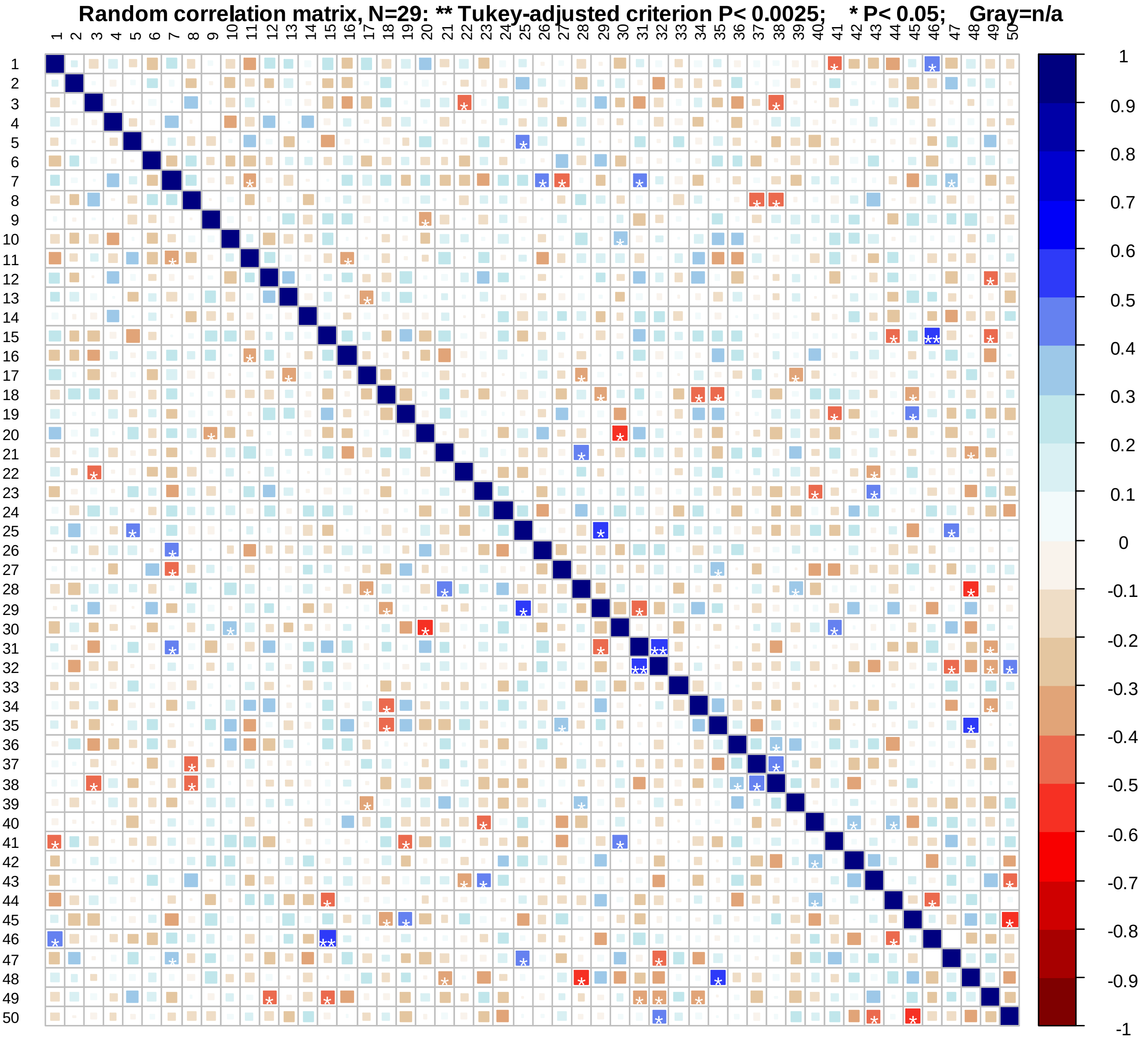

Supplementary figure S2: Correlation matrix for the raw, non-imputed, data, for 50 measures across 29 medical schools. Note that eight correlations could not be calculated and the cells are shown in gray, and not all correlations have an N of 29.

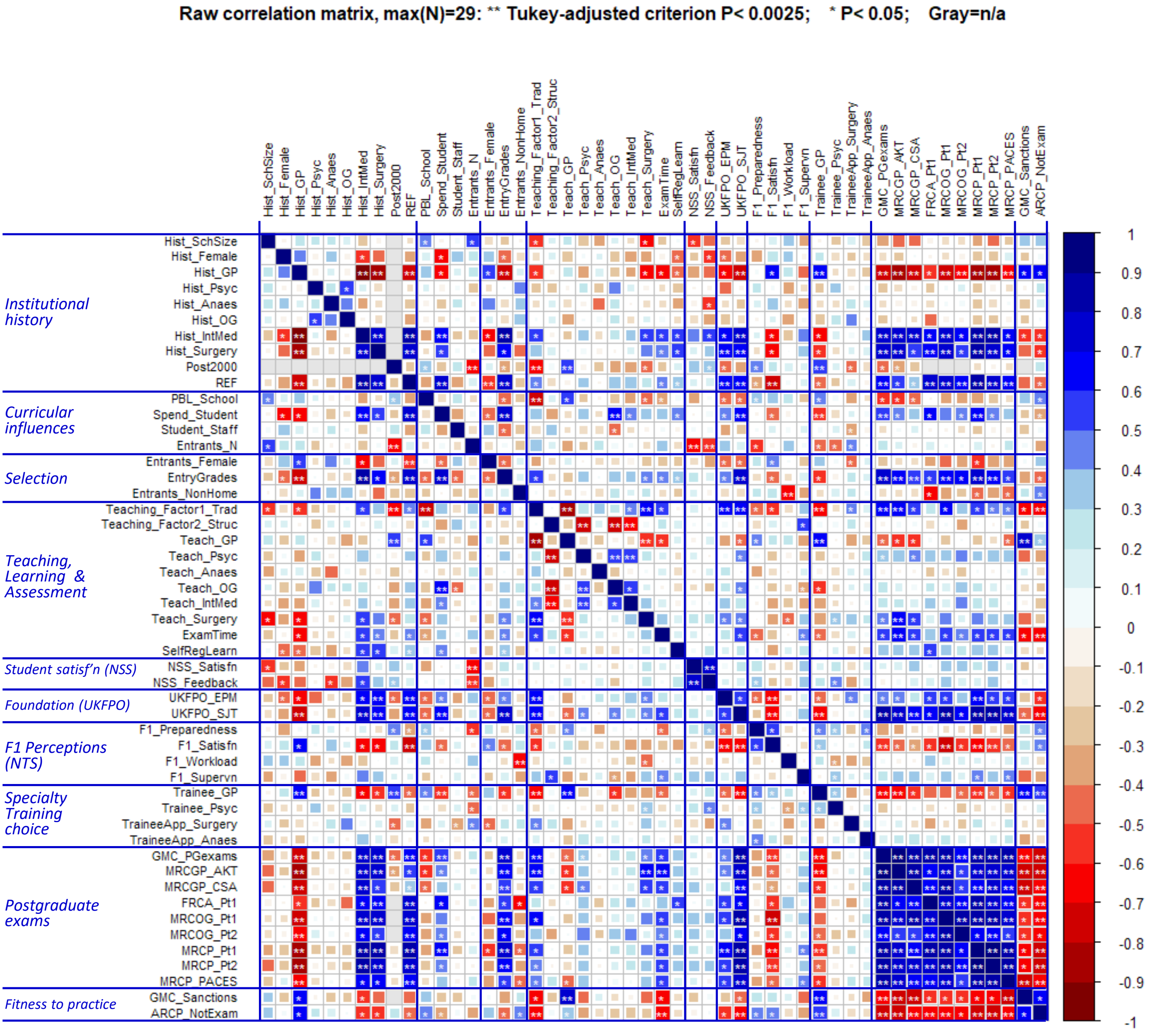

Supplementary figure S3: Correlogram for 29 medical schools across 29 measures looking at the broad influences of teaching measures on input and output measures. Correlations are the same as in main figure 1, but have been reduced in number and rearranged so that the structure of the causal levels can be seen more clearly, being separated by the horizontal and vertical blue lines.

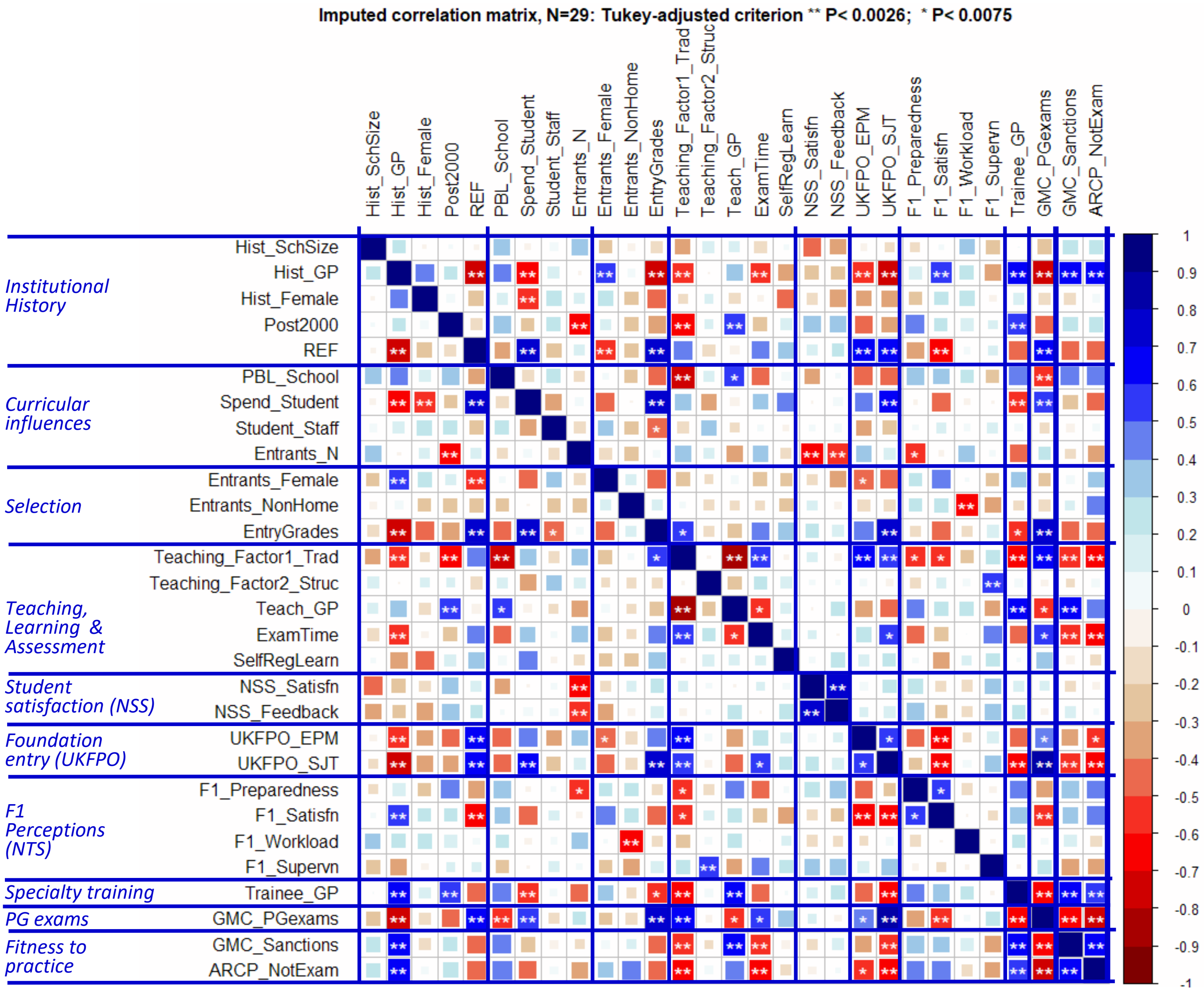

Supplementary figure S4: Reduced structural model showing direct and indirect effects on GMC sanctions and ARCP-Not Exam measures.

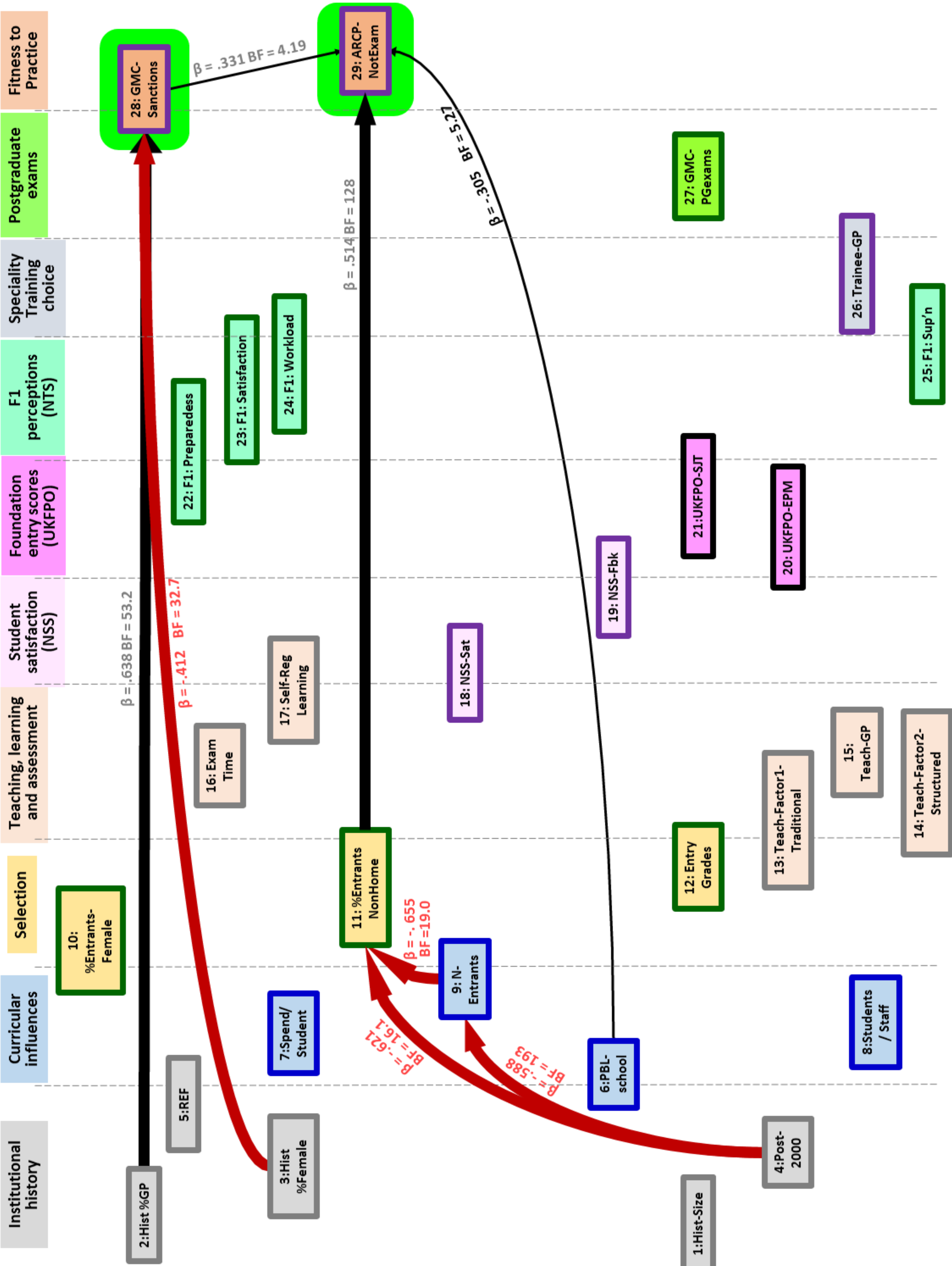

Supplementary figure S5: Reduced structural model showing direct and indirect effects on Trainee-GP.

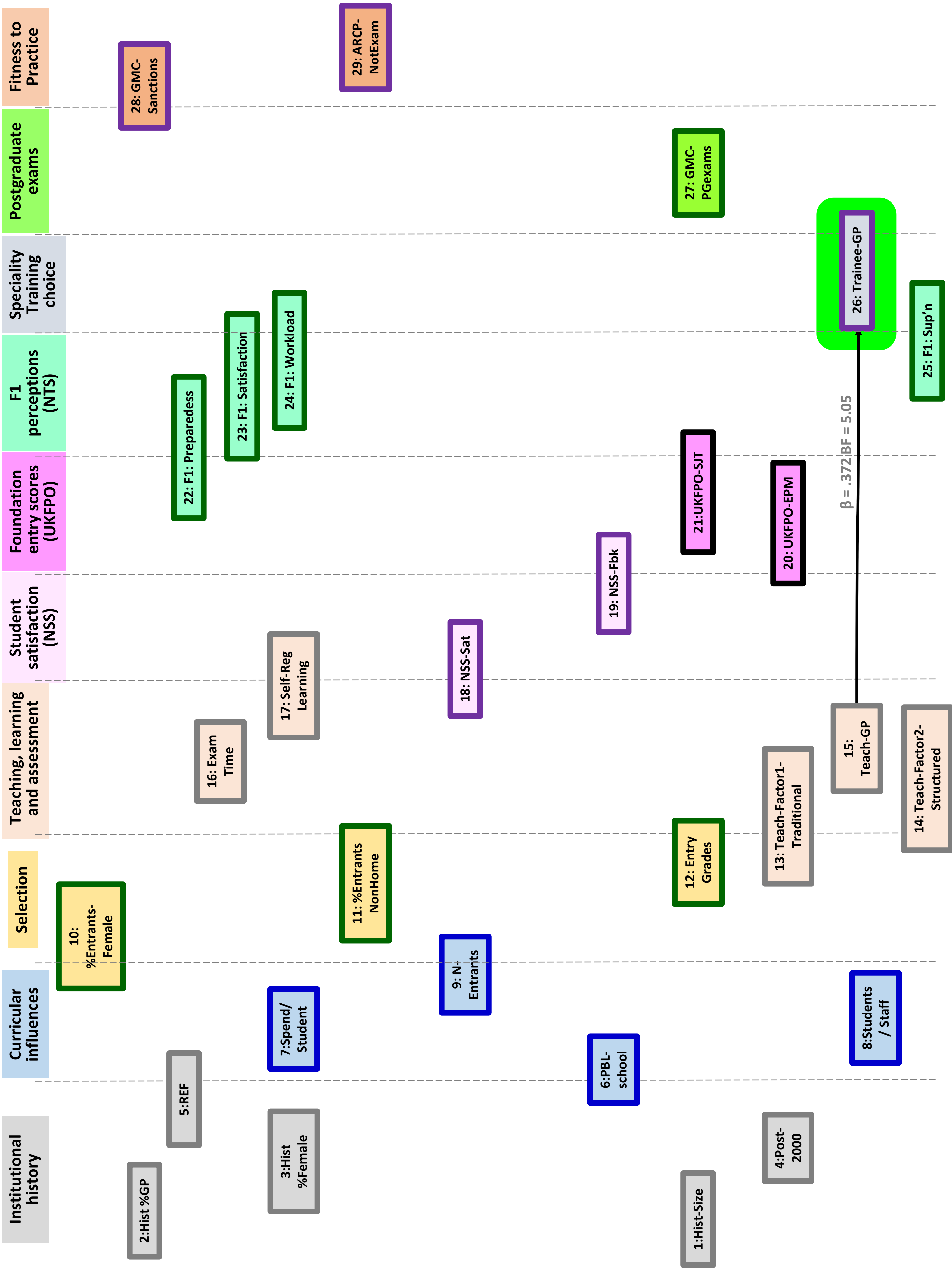

Supplementary figure S6: Reduced structural model showing direct and indirect effects on the four F1 perception measures from the NTS.

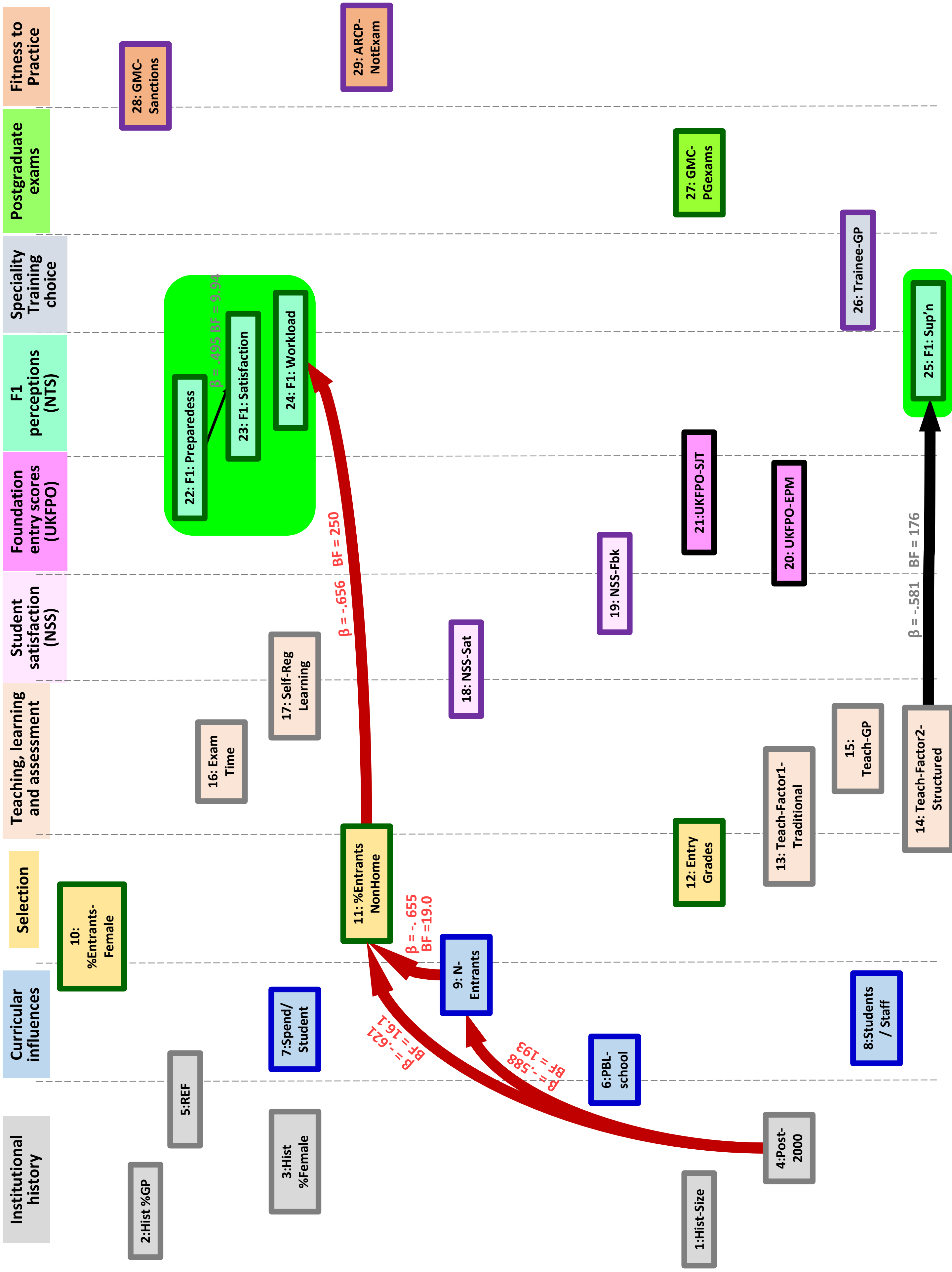

Supplementary figure S7: Reduced structural model showing direct and indirect effects for the two NSS measures of student satisfaction.

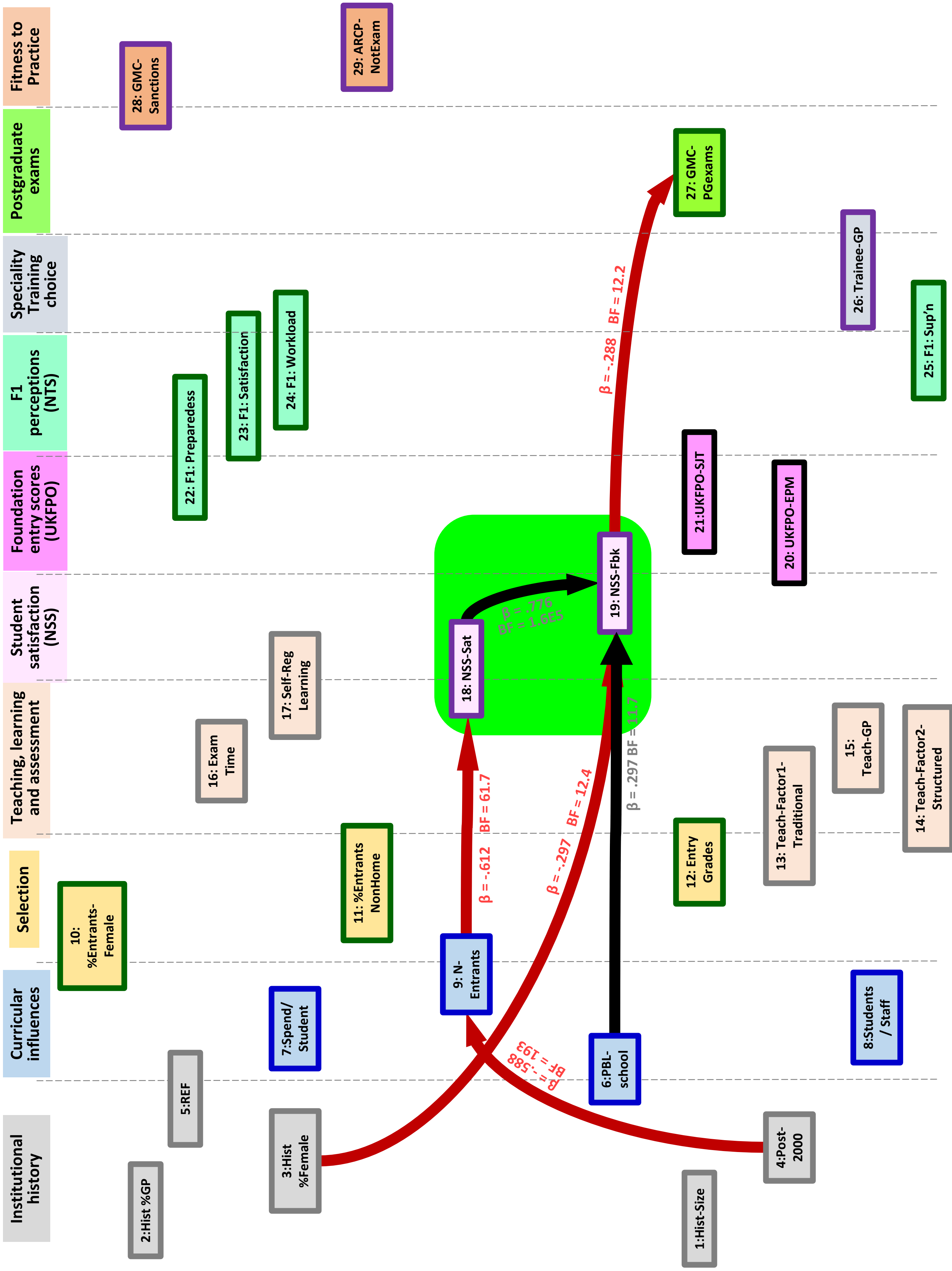

Supplementary figure S8: Reduced structural model showing direct and indirect effects for being a PBL-school.

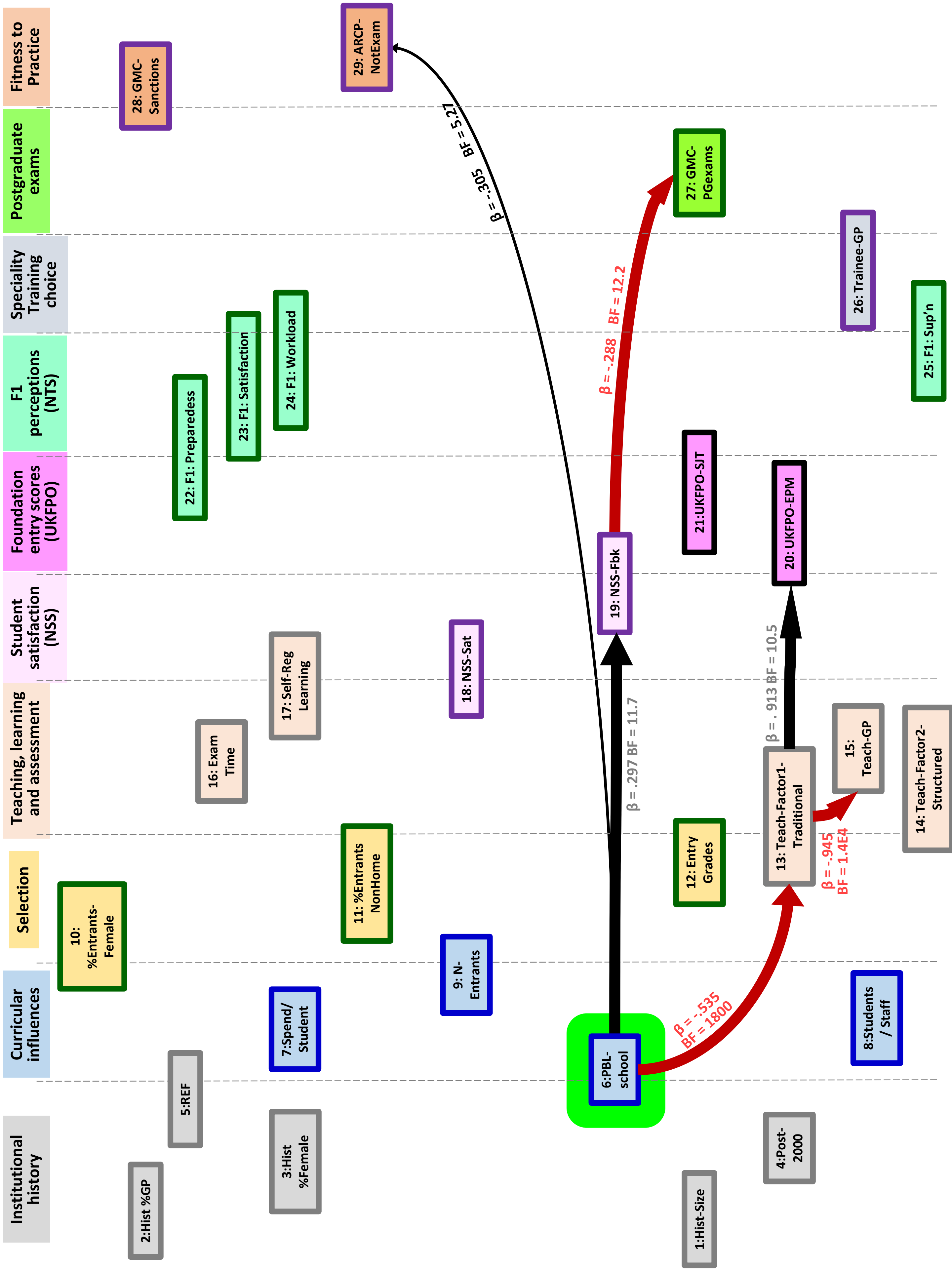

Supplementary figure S9: Reduced structural model showing direct and indirect effects for being a post-2000 medical school.

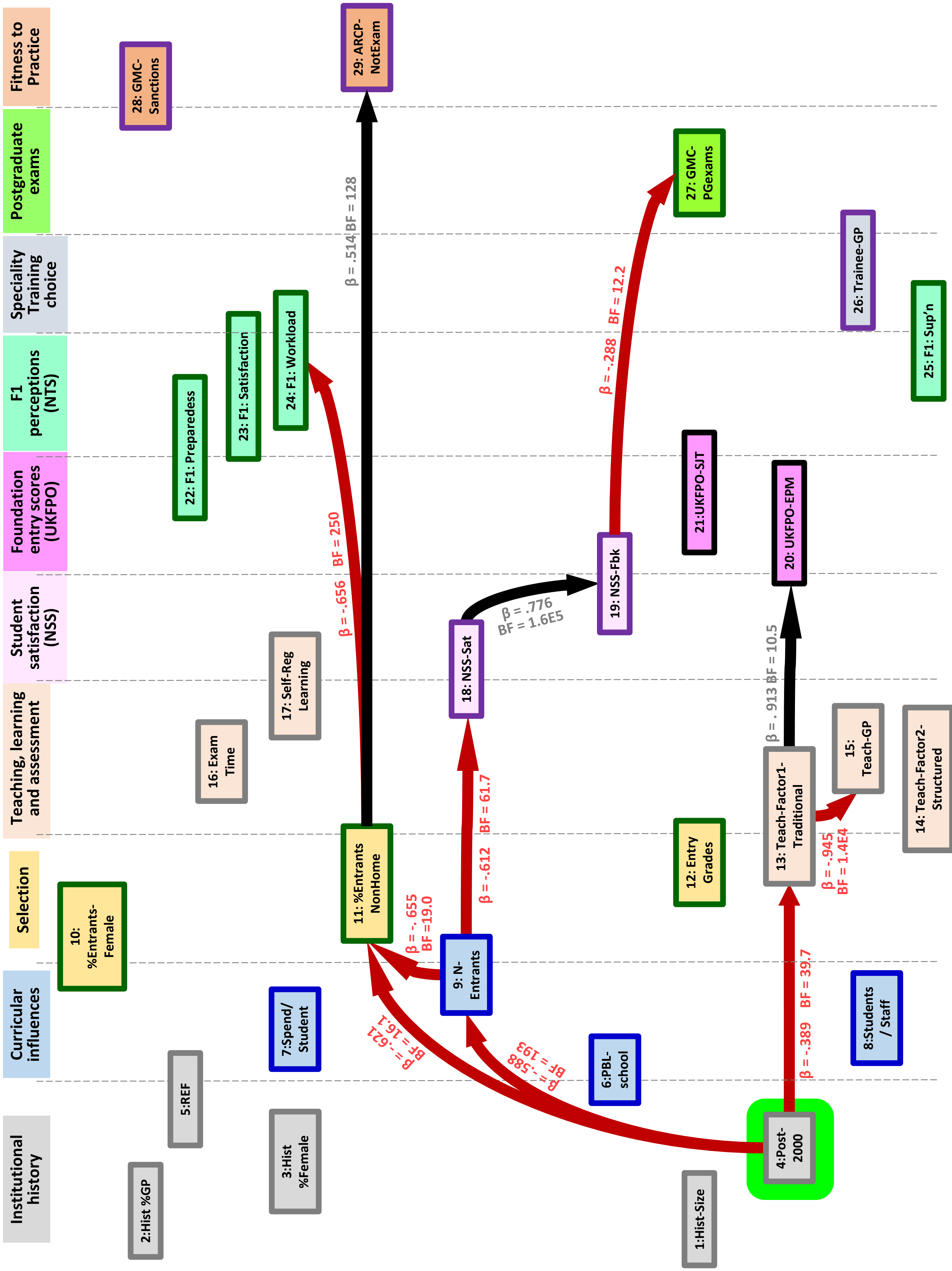

Supplementary figure S10: Reduced structural model showing direct and indirect effects for the historical measures of production of GPs and proportion of female graduates.

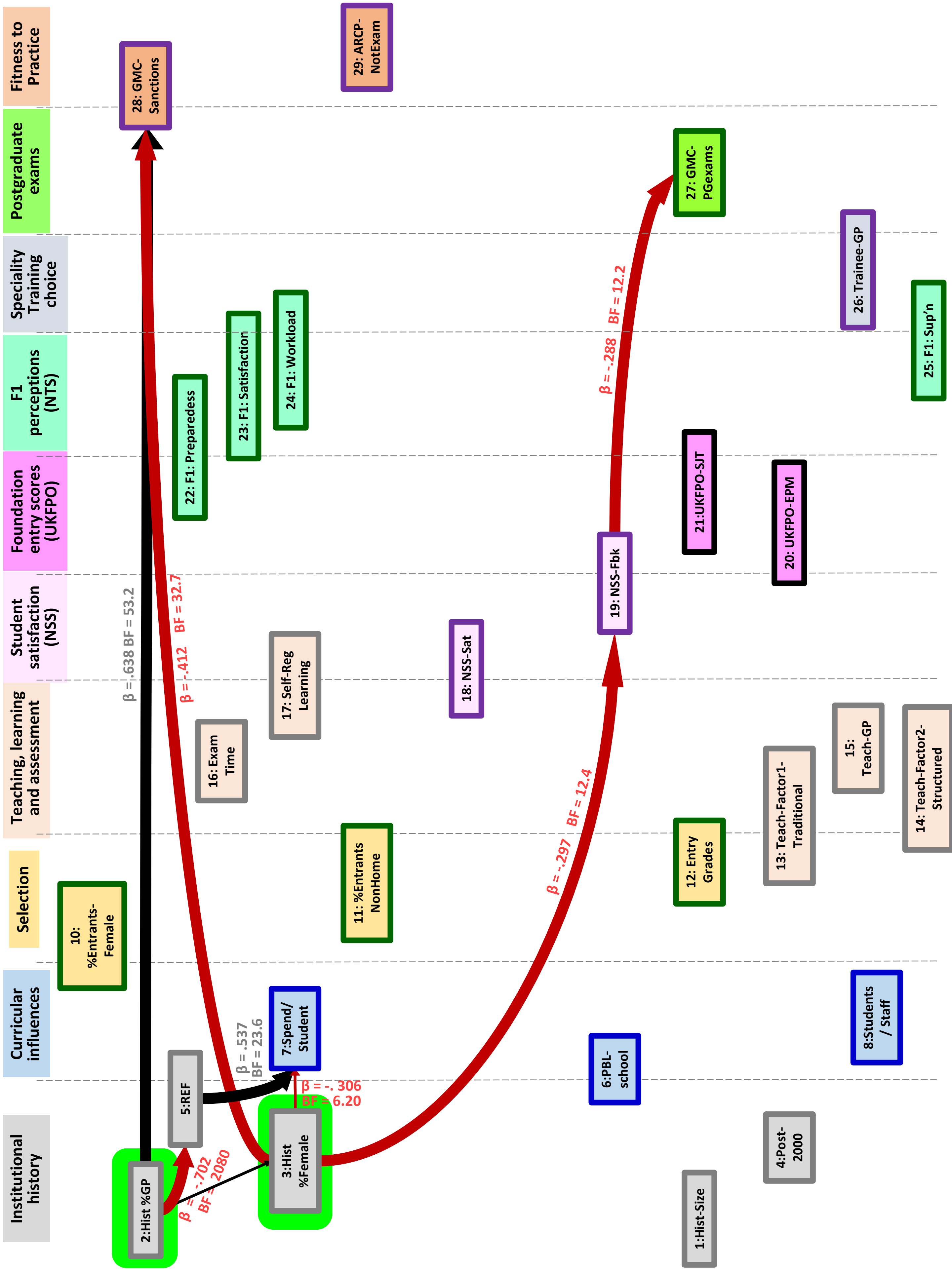

Supplement: Supplementary file 1 — Additional file 1. Figures S1 to S10, and additional text on the causal ordering of variables. [file 12916_2020_1572_MOESM1_ESM.pdf]
