# Supplementary material for: Exploring UK medical school differences: the MedDifs study of selection, teaching, student and F1 perceptions, postgraduate outcomes and fitness to practise
Source: BMC Med. 2020 May 14;18:136. doi: 10.1186/s12916-020-01572-3 (PMC7222458; doi:10.1186/s12916-020-01572-3)
Supplement: Supplementary file 3 — Additional file 3. Index and notes on supplementary graphs 1 to 205 (page 1 to 205). [file 12916_2020_1572_MOESM3_ESM.pdf]

***Supplementary File 3**: An index and notes on Supplementary Files 4 to 9*

Supplementary files 4 to 9 contain scattergrams of all the 1225 possible pairs of the 50 variables used in the analysis (main figure 2). This file contains notes on the format of the supplementary files as well as an index to them.

The original uncompressed supplementary file contained all 1225 scattergrams but was 204 Mbytes in length. *BioMedCentral* sets a limit of 20Mbytes for supplementary files and therefore the original file has been split into six files (Supplementary Files 4 to 9), each of which has also been compressed to below 20 Mbytes using *NXPowerlite* (https://www.neuxpower.com/nxpowerlite-desktop) which reduces file size without noticeably reducing image quality.

Supplementary File 3 also provides a detail listing of all of the graphs, page by page, and should be used in conjunction with Supplementary Files 4 to 9 to find appropriate graphs.

Supplementary File 4: Pages 1 to 35 (graphs 1 to 210)
Supplementary File 5: Pages 36 to 70 (graphs 211 to 420)
Supplementary File 6: Pages 71 to 105 (graphs 421 to 630)
Supplementary File 7: Pages 106 to 140 (graphs 631 to 840)
Supplementary File 8: Pages 141 to 175 (graphs 841 to 1050)
Supplementary File 9: Pages 176 to 205 (graphs 1051 to 1225)

An **index** of the 1225 plots is provided at the end of the present file. It is recommended that the index is opened in one window and a file of figures in a separate window.

Notes on the graphs.

The graphs are complicated and represent a lot of information about the measures at individual medical schools.

- The **main title** indicates:
    - The code number of the scattergram, in the form 10/56, where 56 indicates it is the 56th graph, and can be found on page 10, with six graphs per page, and 205 pages.
    - *Yn:* indicates the nth Y variable name, and *Xn:* the nth X variable. Working through the plots, the Y variable varies more slowly and the X variable more quickly. The variables in *Yn* and Xn are in the same order as the variables in main figure 2.
    - An index of the graphs is available at the end of the present file.
- The **sub-title** provides various information:
    - *Npairs* is the total number of medical school pairs
    - *NimputedPairs* is the number of data pairs with imputed values
    - *r(all)* is the Pearson correlation based on all pairs of data, raw and imputed, with *p* the statistical significance.
    - *r(NonImp)* is the correlation for just non-imputed data.
- **Circles** of various colours represent named medical schools.
    - N**ames** are shortened versions of abbreviations in the main papers
    - Data can either be raw data (i.e. an actual value is known) or imputed (the value has been imputed – see the main text).
    - Colours indicate the source of data:
        - **Green**: raw data is available for both the X and the Y axes
        - **Salmon pink**: X value only is imputed
        - **Pale brown**: Y value only is imputed
        - **Grey:** Both X and Y imputed.
        - **Blue**: Oxbridge and Cambridge (and for all cases X and Y values are raw data)
    - **Yellow Squares** behind circles indicate PBL schools
- **Regression lines** are shown by different colours
    - **Thick, dark blue:** All medical schools based on raw and imputed data
    - **Thin, pale blue line**: All medical schools based on raw, non-imputed, data.
    - **Thin green line**: All medical schools *except Oxford and Cambridge*, using raw and imputed data. Oxford and Cambridge are occasionally outliers in the UK educational system, and comparison with the dark blue line makes clear whether effects are also present when Oxbridge schools are omitted.

Notes and comments.

- Although three different regression lines are shown, mostly these don't differ, particularly when the main regression line in blue is statistically significant. Imputing and including Oxbridge schools have therefore had little effect on relationships (but see figure 8 in the main paper).
- PBL schools, which can be visually identified easily by the layout of the yellow squares, often differ on many of the measures.
- Where measures are binary e.g. PBLschool, Post-2000, etc, the scattergrams are only included for completeness, and to give a visual sense of the data.
- Later variables in main figure 1 are always plotted as the Y variable and earlier variables as the X variable, as that is usually likely to indicate the probably causal direction.

Page	Plot Number	Number On Page	X variable	Y variable	X number	Y number
1	1	1	Hist_SchSize	Hist_Female	1	2
1	2	2	Hist_SchSize	Hist_GP	1	3
1	3	3	Hist_SchSize	Hist_Psyc	1	4
1	4	4	Hist_SchSize	Hist_Anaes	1	5
1	5	5	Hist_SchSize	Hist_OG	1	6
1	6	6	Hist_SchSize	Hist_IntMed	1	7
2	7	1	Hist_SchSize	Hist_Surgery	1	8
2	8	2	Hist_SchSize	Post2000	1	9
2	9	3	Hist_SchSize	REF	1	10
2	10	4	Hist_SchSize	PBL_School	1	11
2	11	5	Hist_SchSize	Spend_Student	1	12
2	12	6	Hist_SchSize	Student_Staff	1	13
3	13	1	Hist_SchSize	Entrants_N	1	14
3	14	2	Hist_SchSize	Entrants_Female	1	15
3	15	3	Hist_SchSize	EntryGrades	1	16
3	16	4	Hist_SchSize	Entrants_NonHome	1	17
3	17	5	Hist_SchSize	Teaching_Factor1_Trad	1	18
3	18	6	Hist_SchSize	Teaching_Factor2_Struc	1	19
4	19	1	Hist_SchSize	Teach_GP	1	20
4	20	2	Hist_SchSize	Teach_Psyc	1	21
4	21	3	Hist_SchSize	Teach_Anaes	1	22
4	22	4	Hist_SchSize	Teach_OG	1	23
4	23	5	Hist_SchSize	Teach_IntMed	1	24
4	24	6	Hist_SchSize	Teach_Surgery	1	25
5	25	1	Hist_SchSize	ExamTime	1	26
5	26	2	Hist_SchSize	SelfRegLearn	1	27
5	27	3	Hist_SchSize	NSS_Satisfn	1	28
5	28	4	Hist_SchSize	NSS_Feedback	1	29
5	29	5	Hist_SchSize	UKFPO_EPM	1	30
5	30	6	Hist_SchSize	UKFPO_SJT	1	31
6	31	1	Hist_SchSize	F1_Preparedness	1	32
6	32	2	Hist_SchSize	F1_Satisfn	1	33
6	33	3	Hist_SchSize	F1_Workload	1	34
6	34	4	Hist_SchSize	F1_Supervn	1	35
6	35	5	Hist_SchSize	Trainee_GP	1	36
6	36	6	Hist_SchSize	Trainee_Psyc	1	37
7	37	1	Hist_SchSize	TraineeApp_Surgery	1	38
7	38	2	Hist_SchSize	TraineeApp_Anaes	1	39
7	39	3	Hist_SchSize	GMC_PGexams	1	40
7	40	4	Hist_SchSize	MRCGP_AKT	1	41
7	41	5	Hist_SchSize	MRCGP_CSA	1	42
7	42	6	Hist_SchSize	FRCA_Pt1	1	43
8	43	1	Hist_SchSize	MRCOG_Pt1	1	44
8	44	2	Hist_SchSize	MRCOG_Pt2	1	45
8	45	3	Hist_SchSize	MRCP_Pt1	1	46
8	46	4	Hist_SchSize	MRCP_Pt2	1	47
8	47	5	Hist_SchSize	MRCP_PACES	1	48
8	48	6	Hist_SchSize	GMC_Sanctions	1	49
9	49	1	Hist_SchSize	ARCP_NotExam	1	50

Page	Plot Number	Number On Page	X variable	Y variable	X number	Y number
9	50	2	Hist_Female	Hist_GP	2	3
9	51	3	Hist_Female	Hist_Psyc	2	4
9	52	4	Hist_Female	Hist_Anaes	2	5
9	53	5	Hist_Female	Hist_OG	2	6
9	54	6	Hist_Female	Hist_IntMed	2	7
10	55	1	Hist_Female	Hist_Surgery	2	8
10	56	2	Hist_Female	Post2000	2	9
10	57	3	Hist_Female	REF	2	10
10	58	4	Hist_Female	PBL_School	2	11
10	59	5	Hist_Female	Spend_Student	2	12
10	60	6	Hist_Female	Student_Staff	2	13
11	61	1	Hist_Female	Entrants_N	2	14
11	62	2	Hist_Female	Entrants_Female	2	15
11	63	3	Hist_Female	EntryGrades	2	16
11	64	4	Hist_Female	Entrants_NonHome	2	17
11	65	5	Hist_Female	Teaching_Factor1_Trad	2	18
11	66	6	Hist_Female	Teaching_Factor2_Struc	2	19
12	67	1	Hist_Female	Teach_GP	2	20
12	68	2	Hist_Female	Teach_Psyc	2	21
12	69	3	Hist_Female	Teach_Anaes	2	22
12	70	4	Hist_Female	Teach_OG	2	23
12	71	5	Hist_Female	Teach_IntMed	2	24
12	72	6	Hist_Female	Teach_Surgery	2	25
13	73	1	Hist_Female	ExamTime	2	26
13	74	2	Hist_Female	SelfRegLearn	2	27
13	75	3	Hist_Female	NSS_Satisfn	2	28
13	76	4	Hist_Female	NSS_Feedback	2	29
13	77	5	Hist_Female	UKFPO_EPM	2	30
13	78	6	Hist_Female	UKFPO_SJT	2	31
14	79	1	Hist_Female	F1_Preparedness	2	32
14	80	2	Hist_Female	F1_Satisfn	2	33
14	81	3	Hist_Female	F1_Workload	2	34
14	82	4	Hist_Female	F1_Supervn	2	35
14	83	5	Hist_Female	Trainee_GP	2	36
14	84	6	Hist_Female	Trainee_Psyc	2	37
15	85	1	Hist_Female	TraineeApp_Surgery	2	38
15	86	2	Hist_Female	TraineeApp_Anaes	2	39
15	87	3	Hist_Female	GMC_PGexams	2	40
15	88	4	Hist_Female	MRCGP_AKT	2	41
15	89	5	Hist_Female	MRCGP_CSA	2	42
15	90	6	Hist_Female	FRCA_Pt1	2	43
16	91	1	Hist_Female	MRCOG_Pt1	2	44
16	92	2	Hist_Female	MRCOG_Pt2	2	45
16	93	3	Hist_Female	MRCP_Pt1	2	46
16	94	4	Hist_Female	MRCP_Pt2	2	47
16	95	5	Hist_Female	MRCP_PACES	2	48
16	96	6	Hist_Female	GMC_Sanctions	2	49
17	97	1	Hist_Female	ARCP_NotExam	2	50
17	98	2	Hist_GP	Hist_Psyc	3	4

Page	Plot Number	Number On Page	X variable	Y variable	X number	Y number
17	99	3	Hist_GP	Hist_Anaes	3	5
17	100	4	Hist_GP	Hist_OG	3	6
17	101	5	Hist_GP	Hist_IntMed	3	7
17	102	6	Hist_GP	Hist_Surgery	3	8
18	103	1	Hist_GP	Post2000	3	9
18	104	2	Hist_GP	REF	3	10
18	105	3	Hist_GP	PBL_School	3	11
18	106	4	Hist_GP	Spend_Student	3	12
18	107	5	Hist_GP	Student_Staff	3	13
18	108	6	Hist_GP	Entrants_N	3	14
19	109	1	Hist_GP	Entrants_Female	3	15
19	110	2	Hist_GP	EntryGrades	3	16
19	111	3	Hist_GP	Entrants_NonHome	3	17
19	112	4	Hist_GP	Teaching_Factor1_Trad	3	18
19	113	5	Hist_GP	Teaching_Factor2_Struc	3	19
19	114	6	Hist_GP	Teach_GP	3	20
20	115	1	Hist_GP	Teach_Psyc	3	21
20	116	2	Hist_GP	Teach_Anaes	3	22
20	117	3	Hist_GP	Teach_OG	3	23
20	118	4	Hist_GP	Teach_IntMed	3	24
20	119	5	Hist_GP	Teach_Surgery	3	25
20	120	6	Hist_GP	ExamTime	3	26
21	121	1	Hist_GP	SelfRegLearn	3	27
21	122	2	Hist_GP	NSS_Satisfn	3	28
21	123	3	Hist_GP	NSS_Feedback	3	29
21	124	4	Hist_GP	UKFPO_EPM	3	30
21	125	5	Hist_GP	UKFPO_SJT	3	31
21	126	6	Hist_GP	F1_Preparedness	3	32
22	127	1	Hist_GP	F1_Satisfn	3	33
22	128	2	Hist_GP	F1_Workload	3	34
22	129	3	Hist_GP	F1_Supervn	3	35
22	130	4	Hist_GP	Trainee_GP	3	36
22	131	5	Hist_GP	Trainee_Psyc	3	37
22	132	6	Hist_GP	TraineeApp_Surgery	3	38
23	133	1	Hist_GP	TraineeApp_Anaes	3	39
23	134	2	Hist_GP	GMC_PGexams	3	40
23	135	3	Hist_GP	MRCGP_AKT	3	41
23	136	4	Hist_GP	MRCGP_CSA	3	42
23	137	5	Hist_GP	FRCA_Pt1	3	43
23	138	6	Hist_GP	MRCOG_Pt1	3	44
24	139	1	Hist_GP	MRCOG_Pt2	3	45
24	140	2	Hist_GP	MRCP_Pt1	3	46
24	141	3	Hist_GP	MRCP_Pt2	3	47
24	142	4	Hist_GP	MRCP_PACES	3	48
24	143	5	Hist_GP	GMC_Sanctions	3	49
24	144	6	Hist_GP	ARCP_NotExam	3	50
25	145	1	Hist_Psyc	Hist_Anaes	4	5
25	146	2	Hist_Psyc	Hist_OG	4	6
25	147	3	Hist_Psyc	Hist_IntMed	4	7

Page	Plot Number	Number On Page	X variable	Y variable	X number	Y number
25	148	4	Hist_Psyc	Hist_Surgery	4	8
25	149	5	Hist_Psyc	Post2000	4	9
25	150	6	Hist_Psyc	REF	4	10
26	151	1	Hist_Psyc	PBL_School	4	11
26	152	2	Hist_Psyc	Spend_Student	4	12
26	153	3	Hist_Psyc	Student_Staff	4	13
26	154	4	Hist_Psyc	Entrants_N	4	14
26	155	5	Hist_Psyc	Entrants_Female	4	15
26	156	6	Hist_Psyc	EntryGrades	4	16
27	157	1	Hist_Psyc	Entrants_NonHome	4	17
27	158	2	Hist_Psyc	Teaching_Factor1_Trad	4	18
27	159	3	Hist_Psyc	Teaching_Factor2_Struc	4	19
27	160	4	Hist_Psyc	Teach_GP	4	20
27	161	5	Hist_Psyc	Teach_Psyc	4	21
27	162	6	Hist_Psyc	Teach_Anaes	4	22
28	163	1	Hist_Psyc	Teach_OG	4	23
28	164	2	Hist_Psyc	Teach_IntMed	4	24
28	165	3	Hist_Psyc	Teach_Surgery	4	25
28	166	4	Hist_Psyc	ExamTime	4	26
28	167	5	Hist_Psyc	SelfRegLearn	4	27
28	168	6	Hist_Psyc	NSS_Satisfn	4	28
29	169	1	Hist_Psyc	NSS_Feedback	4	29
29	170	2	Hist_Psyc	UKFPO_EPM	4	30
29	171	3	Hist_Psyc	UKFPO_SJT	4	31
29	172	4	Hist_Psyc	F1_Preparedness	4	32
29	173	5	Hist_Psyc	F1_Satisfn	4	33
29	174	6	Hist_Psyc	F1_Workload	4	34
30	175	1	Hist_Psyc	F1_Supervn	4	35
30	176	2	Hist_Psyc	Trainee_GP	4	36
30	177	3	Hist_Psyc	Trainee_Psyc	4	37
30	178	4	Hist_Psyc	TraineeApp_Surgery	4	38
30	179	5	Hist_Psyc	TraineeApp_Anaes	4	39
30	180	6	Hist_Psyc	GMC_PGexams	4	40
31	181	1	Hist_Psyc	MRCGP_AKT	4	41
31	182	2	Hist_Psyc	MRCGP_CSA	4	42
31	183	3	Hist_Psyc	FRCA_Pt1	4	43
31	184	4	Hist_Psyc	MRCOG_Pt1	4	44
31	185	5	Hist_Psyc	MRCOG_Pt2	4	45
31	186	6	Hist_Psyc	MRCP_Pt1	4	46
32	187	1	Hist_Psyc	MRCP_Pt2	4	47
32	188	2	Hist_Psyc	MRCP_PACES	4	48
32	189	3	Hist_Psyc	GMC_Sanctions	4	49
32	190	4	Hist_Psyc	ARCP_NotExam	4	50
32	191	5	Hist_Anaes	Hist_OG	5	6
32	192	6	Hist_Anaes	Hist_IntMed	5	7
33	193	1	Hist_Anaes	Hist_Surgery	5	8
33	194	2	Hist_Anaes	Post2000	5	9
33	195	3	Hist_Anaes	REF	5	10
33	196	4	Hist_Anaes	PBL_School	5	11

Page	Plot Number	Number On Page	X variable	Y variable	X number	Y number
33	197	5	Hist_Anaes	Spend_Student	5	12
33	198	6	Hist_Anaes	Student_Staff	5	13
34	199	1	Hist_Anaes	Entrants_N	5	14
34	200	2	Hist_Anaes	Entrants_Female	5	15
34	201	3	Hist_Anaes	EntryGrades	5	16
34	202	4	Hist_Anaes	Entrants_NonHome	5	17
34	203	5	Hist_Anaes	Teaching_Factor1_Trad	5	18
34	204	6	Hist_Anaes	Teaching_Factor2_Struc	5	19
35	205	1	Hist_Anaes	Teach_GP	5	20
35	206	2	Hist_Anaes	Teach_Psyc	5	21
35	207	3	Hist_Anaes	Teach_Anaes	5	22
35	208	4	Hist_Anaes	Teach_OG	5	23
35	209	5	Hist_Anaes	Teach_IntMed	5	24
35	210	6	Hist_Anaes	Teach_Surgery	5	25
36	211	1	Hist_Anaes	ExamTime	5	26
36	212	2	Hist_Anaes	SelfRegLearn	5	27
36	213	3	Hist_Anaes	NSS_Satisfn	5	28
36	214	4	Hist_Anaes	NSS_Feedback	5	29
36	215	5	Hist_Anaes	UKFPO_EPM	5	30
36	216	6	Hist_Anaes	UKFPO_SJT	5	31
37	217	1	Hist_Anaes	F1_Preparedness	5	32
37	218	2	Hist_Anaes	F1_Satisfn	5	33
37	219	3	Hist_Anaes	F1_Workload	5	34
37	220	4	Hist_Anaes	F1_Supervn	5	35
37	221	5	Hist_Anaes	Trainee_GP	5	36
37	222	6	Hist_Anaes	Trainee_Psyc	5	37
38	223	1	Hist_Anaes	TraineeApp_Surgery	5	38
38	224	2	Hist_Anaes	TraineeApp_Anaes	5	39
38	225	3	Hist_Anaes	GMC_PGexams	5	40
38	226	4	Hist_Anaes	MRCGP_AKT	5	41
38	227	5	Hist_Anaes	MRCGP_CSA	5	42
38	228	6	Hist_Anaes	FRCA_Pt1	5	43
39	229	1	Hist_Anaes	MRCOG_Pt1	5	44
39	230	2	Hist_Anaes	MRCOG_Pt2	5	45
39	231	3	Hist_Anaes	MRCP_Pt1	5	46
39	232	4	Hist_Anaes	MRCP_Pt2	5	47
39	233	5	Hist_Anaes	MRCP_PACES	5	48
39	234	6	Hist_Anaes	GMC_Sanctions	5	49
40	235	1	Hist_Anaes	ARCP_NotExam	5	50
40	236	2	Hist_OG	Hist_IntMed	6	7
40	237	3	Hist_OG	Hist_Surgery	6	8
40	238	4	Hist_OG	Post2000	6	9
40	239	5	Hist_OG	REF	6	10
40	240	6	Hist_OG	PBL_School	6	11
41	241	1	Hist_OG	Spend_Student	6	12
41	242	2	Hist_OG	Student_Staff	6	13
41	243	3	Hist_OG	Entrants_N	6	14
41	244	4	Hist_OG	Entrants_Female	6	15
41	245	5	Hist_OG	EntryGrades	6	16

Page	Plot Number	Number On Page	X variable	Y variable	X number	Y number
41	246	6	Hist_OG	Entrants_NonHome	6	17
42	247	1	Hist_OG	Teaching_Factor1_Trad	6	18
42	248	2	Hist_OG	Teaching_Factor2_Struc	6	19
42	249	3	Hist_OG	Teach_GP	6	20
42	250	4	Hist_OG	Teach_Psyc	6	21
42	251	5	Hist_OG	Teach_Anaes	6	22
42	252	6	Hist_OG	Teach_OG	6	23
43	253	1	Hist_OG	Teach_IntMed	6	24
43	254	2	Hist_OG	Teach_Surgery	6	25
43	255	3	Hist_OG	ExamTime	6	26
43	256	4	Hist_OG	SelfRegLearn	6	27
43	257	5	Hist_OG	NSS_Satisfn	6	28
43	258	6	Hist_OG	NSS_Feedback	6	29
44	259	1	Hist_OG	UKFPO_EPM	6	30
44	260	2	Hist_OG	UKFPO_SJT	6	31
44	261	3	Hist_OG	F1_Preparedness	6	32
44	262	4	Hist_OG	F1_Satisfn	6	33
44	263	5	Hist_OG	F1_Workload	6	34
44	264	6	Hist_OG	F1_Supervn	6	35
45	265	1	Hist_OG	Trainee_GP	6	36
45	266	2	Hist_OG	Trainee_Psyc	6	37
45	267	3	Hist_OG	TraineeApp_Surgery	6	38
45	268	4	Hist_OG	TraineeApp_Anaes	6	39
45	269	5	Hist_OG	GMC_PGexams	6	40
45	270	6	Hist_OG	MRCGP_AKT	6	41
46	271	1	Hist_OG	MRCGP_CSA	6	42
46	272	2	Hist_OG	FRCA_Pt1	6	43
46	273	3	Hist_OG	MRCOG_Pt1	6	44
46	274	4	Hist_OG	MRCOG_Pt2	6	45
46	275	5	Hist_OG	MRCP_Pt1	6	46
46	276	6	Hist_OG	MRCP_Pt2	6	47
47	277	1	Hist_OG	MRCP_PACES	6	48
47	278	2	Hist_OG	GMC_Sanctions	6	49
47	279	3	Hist_OG	ARCP_NotExam	6	50
47	280	4	Hist_IntMed	Hist_Surgery	7	8
47	281	5	Hist_IntMed	Post2000	7	9
47	282	6	Hist_IntMed	REF	7	10
48	283	1	Hist_IntMed	PBL_School	7	11
48	284	2	Hist_IntMed	Spend_Student	7	12
48	285	3	Hist_IntMed	Student_Staff	7	13
48	286	4	Hist_IntMed	Entrants_N	7	14
48	287	5	Hist_IntMed	Entrants_Female	7	15
48	288	6	Hist_IntMed	EntryGrades	7	16
49	289	1	Hist_IntMed	Entrants_NonHome	7	17
49	290	2	Hist_IntMed	Teaching_Factor1_Trad	7	18
49	291	3	Hist_IntMed	Teaching_Factor2_Struc	7	19
49	292	4	Hist_IntMed	Teach_GP	7	20
49	293	5	Hist_IntMed	Teach_Psyc	7	21
49	294	6	Hist_IntMed	Teach_Anaes	7	22

Page	Plot Number	Number On Page	X variable	Y variable	X number	Y number
50	295	1	Hist_IntMed	Teach_OG	7	23
50	296	2	Hist_IntMed	Teach_IntMed	7	24
50	297	3	Hist_IntMed	Teach_Surgery	7	25
50	298	4	Hist_IntMed	ExamTime	7	26
50	299	5	Hist_IntMed	SelfRegLearn	7	27
50	300	6	Hist_IntMed	NSS_Satisfn	7	28
51	301	1	Hist_IntMed	NSS_Feedback	7	29
51	302	2	Hist_IntMed	UKFPO_EPM	7	30
51	303	3	Hist_IntMed	UKFPO_SJT	7	31
51	304	4	Hist_IntMed	F1_Preparedness	7	32
51	305	5	Hist_IntMed	F1_Satisfn	7	33
51	306	6	Hist_IntMed	F1_Workload	7	34
52	307	1	Hist_IntMed	F1_Supervn	7	35
52	308	2	Hist_IntMed	Trainee_GP	7	36
52	309	3	Hist_IntMed	Trainee_Psyc	7	37
52	310	4	Hist_IntMed	TraineeApp_Surgery	7	38
52	311	5	Hist_IntMed	TraineeApp_Anaes	7	39
52	312	6	Hist_IntMed	GMC_PGexams	7	40
53	313	1	Hist_IntMed	MRCGP_AKT	7	41
53	314	2	Hist_IntMed	MRCGP_CSA	7	42
53	315	3	Hist_IntMed	FRCA_Pt1	7	43
53	316	4	Hist_IntMed	MRCOG_Pt1	7	44
53	317	5	Hist_IntMed	MRCOG_Pt2	7	45
53	318	6	Hist_IntMed	MRCP_Pt1	7	46
54	319	1	Hist_IntMed	MRCP_Pt2	7	47
54	320	2	Hist_IntMed	MRCP_PACES	7	48
54	321	3	Hist_IntMed	GMC_Sanctions	7	49
54	322	4	Hist_IntMed	ARCP_NotExam	7	50
54	323	5	Hist_Surgery	Post2000	8	9
54	324	6	Hist_Surgery	REF	8	10
55	325	1	Hist_Surgery	PBL_School	8	11
55	326	2	Hist_Surgery	Spend_Student	8	12
55	327	3	Hist_Surgery	Student_Staff	8	13
55	328	4	Hist_Surgery	Entrants_N	8	14
55	329	5	Hist_Surgery	Entrants_Female	8	15
55	330	6	Hist_Surgery	EntryGrades	8	16
56	331	1	Hist_Surgery	Entrants_NonHome	8	17
56	332	2	Hist_Surgery	Teaching_Factor1_Trad	8	18
56	333	3	Hist_Surgery	Teaching_Factor2_Struc	8	19
56	334	4	Hist_Surgery	Teach_GP	8	20
56	335	5	Hist_Surgery	Teach_Psyc	8	21
56	336	6	Hist_Surgery	Teach_Anaes	8	22
57	337	1	Hist_Surgery	Teach_OG	8	23
57	338	2	Hist_Surgery	Teach_IntMed	8	24
57	339	3	Hist_Surgery	Teach_Surgery	8	25
57	340	4	Hist_Surgery	ExamTime	8	26
57	341	5	Hist_Surgery	SelfRegLearn	8	27
57	342	6	Hist_Surgery	NSS_Satisfn	8	28
58	343	1	Hist_Surgery	NSS_Feedback	8	29

Page	Plot Number	Number On Page	X variable	Y variable	X number	Y number
58	344	2	Hist_Surgery	UKFPO_EPM	8	30
58	345	3	Hist_Surgery	UKFPO_SJT	8	31
58	346	4	Hist_Surgery	F1_Preparedness	8	32
58	347	5	Hist_Surgery	F1_Satisfn	8	33
58	348	6	Hist_Surgery	F1_Workload	8	34
59	349	1	Hist_Surgery	F1_Supervn	8	35
59	350	2	Hist_Surgery	Trainee_GP	8	36
59	351	3	Hist_Surgery	Trainee_Psyc	8	37
59	352	4	Hist_Surgery	TraineeApp_Surgery	8	38
59	353	5	Hist_Surgery	TraineeApp_Anaes	8	39
59	354	6	Hist_Surgery	GMC_PGexams	8	40
60	355	1	Hist_Surgery	MRCGP_AKT	8	41
60	356	2	Hist_Surgery	MRCGP_CSA	8	42
60	357	3	Hist_Surgery	FRCA_Pt1	8	43
60	358	4	Hist_Surgery	MRCOG_Pt1	8	44
60	359	5	Hist_Surgery	MRCOG_Pt2	8	45
60	360	6	Hist_Surgery	MRCP_Pt1	8	46
61	361	1	Hist_Surgery	MRCP_Pt2	8	47
61	362	2	Hist_Surgery	MRCP_PACES	8	48
61	363	3	Hist_Surgery	GMC_Sanctions	8	49
61	364	4	Hist_Surgery	ARCP_NotExam	8	50
61	365	5	Post2000	REF	9	10
61	366	6	Post2000	PBL_School	9	11
62	367	1	Post2000	Spend_Student	9	12
62	368	2	Post2000	Student_Staff	9	13
62	369	3	Post2000	Entrants_N	9	14
62	370	4	Post2000	Entrants_Female	9	15
62	371	5	Post2000	EntryGrades	9	16
62	372	6	Post2000	Entrants_NonHome	9	17
63	373	1	Post2000	Teaching_Factor1_Trad	9	18
63	374	2	Post2000	Teaching_Factor2_Struc	9	19
63	375	3	Post2000	Teach_GP	9	20
63	376	4	Post2000	Teach_Psyc	9	21
63	377	5	Post2000	Teach_Anaes	9	22
63	378	6	Post2000	Teach_OG	9	23
64	379	1	Post2000	Teach_IntMed	9	24
64	380	2	Post2000	Teach_Surgery	9	25
64	381	3	Post2000	ExamTime	9	26
64	382	4	Post2000	SelfRegLearn	9	27
64	383	5	Post2000	NSS_Satisfn	9	28
64	384	6	Post2000	NSS_Feedback	9	29
65	385	1	Post2000	UKFPO_EPM	9	30
65	386	2	Post2000	UKFPO_SJT	9	31
65	387	3	Post2000	F1_Preparedness	9	32
65	388	4	Post2000	F1_Satisfn	9	33
65	389	5	Post2000	F1_Workload	9	34
65	390	6	Post2000	F1_Supervn	9	35
66	391	1	Post2000	Trainee_GP	9	36
66	392	2	Post2000	Trainee_Psyc	9	37

Page	Plot Number	Number On Page	X variable	Y variable	X number	Y number
66	393	3	Post2000	TraineeApp_Surgery	9	38
66	394	4	Post2000	TraineeApp_Anaes	9	39
66	395	5	Post2000	GMC_PGexams	9	40
66	396	6	Post2000	MRCGP_AKT	9	41
67	397	1	Post2000	MRCGP_CSA	9	42
67	398	2	Post2000	FRCA_Pt1	9	43
67	399	3	Post2000	MRCOG_Pt1	9	44
67	400	4	Post2000	MRCOG_Pt2	9	45
67	401	5	Post2000	MRCP_Pt1	9	46
67	402	6	Post2000	MRCP_Pt2	9	47
68	403	1	Post2000	MRCP_PACES	9	48
68	404	2	Post2000	GMC_Sanctions	9	49
68	405	3	Post2000	ARCP_NotExam	9	50
68	406	4	REF	PBL_School	10	11
68	407	5	REF	Spend_Student	10	12
68	408	6	REF	Student_Staff	10	13
69	409	1	REF	Entrants_N	10	14
69	410	2	REF	Entrants_Female	10	15
69	411	3	REF	EntryGrades	10	16
69	412	4	REF	Entrants_NonHome	10	17
69	413	5	REF	Teaching_Factor1_Trad	10	18
69	414	6	REF	Teaching_Factor2_Struc	10	19
70	415	1	REF	Teach_GP	10	20
70	416	2	REF	Teach_Psyc	10	21
70	417	3	REF	Teach_Anaes	10	22
70	418	4	REF	Teach_OG	10	23
70	419	5	REF	Teach_IntMed	10	24
70	420	6	REF	Teach_Surgery	10	25
71	421	1	REF	ExamTime	10	26
71	422	2	REF	SelfRegLearn	10	27
71	423	3	REF	NSS_Satisfn	10	28
71	424	4	REF	NSS_Feedback	10	29
71	425	5	REF	UKFPO_EPM	10	30
71	426	6	REF	UKFPO_SJT	10	31
72	427	1	REF	F1_Preparedness	10	32
72	428	2	REF	F1_Satisfn	10	33
72	429	3	REF	F1_Workload	10	34
72	430	4	REF	F1_Supervn	10	35
72	431	5	REF	Trainee_GP	10	36
72	432	6	REF	Trainee_Psyc	10	37
73	433	1	REF	TraineeApp_Surgery	10	38
73	434	2	REF	TraineeApp_Anaes	10	39
73	435	3	REF	GMC_PGexams	10	40
73	436	4	REF	MRCGP_AKT	10	41
73	437	5	REF	MRCGP_CSA	10	42
73	438	6	REF	FRCA_Pt1	10	43
74	439	1	REF	MRCOG_Pt1	10	44
74	440	2	REF	MRCOG_Pt2	10	45
74	441	3	REF	MRCP_Pt1	10	46

Page	Plot Number	Number On Page	X variable	Y variable	X number	Y number
74	442	4	REF	MRCP_Pt2	10	47
74	443	5	REF	MRCP_PACES	10	48
74	444	6	REF	GMC_Sanctions	10	49
75	445	1	REF	ARCP_NotExam	10	50
75	446	2	PBL_School	Spend_Student	11	12
75	447	3	PBL_School	Student_Staff	11	13
75	448	4	PBL_School	Entrants_N	11	14
75	449	5	PBL_School	Entrants_Female	11	15
75	450	6	PBL_School	EntryGrades	11	16
76	451	1	PBL_School	Entrants_NonHome	11	17
76	452	2	PBL_School	Teaching_Factor1_Trad	11	18
76	453	3	PBL_School	Teaching_Factor2_Struc	11	19
76	454	4	PBL_School	Teach_GP	11	20
76	455	5	PBL_School	Teach_Psyc	11	21
76	456	6	PBL_School	Teach_Anaes	11	22
77	457	1	PBL_School	Teach_OG	11	23
77	458	2	PBL_School	Teach_IntMed	11	24
77	459	3	PBL_School	Teach_Surgery	11	25
77	460	4	PBL_School	ExamTime	11	26
77	461	5	PBL_School	SelfRegLearn	11	27
77	462	6	PBL_School	NSS_Satisfn	11	28
78	463	1	PBL_School	NSS_Feedback	11	29
78	464	2	PBL_School	UKFPO_EPM	11	30
78	465	3	PBL_School	UKFPO_SJT	11	31
78	466	4	PBL_School	F1_Preparedness	11	32
78	467	5	PBL_School	F1_Satisfn	11	33
78	468	6	PBL_School	F1_Workload	11	34
79	469	1	PBL_School	F1_Supervn	11	35
79	470	2	PBL_School	Trainee_GP	11	36
79	471	3	PBL_School	Trainee_Psyc	11	37
79	472	4	PBL_School	TraineeApp_Surgery	11	38
79	473	5	PBL_School	TraineeApp_Anaes	11	39
79	474	6	PBL_School	GMC_PGexams	11	40
80	475	1	PBL_School	MRCGP_AKT	11	41
80	476	2	PBL_School	MRCGP_CSA	11	42
80	477	3	PBL_School	FRCA_Pt1	11	43
80	478	4	PBL_School	MRCOG_Pt1	11	44
80	479	5	PBL_School	MRCOG_Pt2	11	45
80	480	6	PBL_School	MRCP_Pt1	11	46
81	481	1	PBL_School	MRCP_Pt2	11	47
81	482	2	PBL_School	MRCP_PACES	11	48
81	483	3	PBL_School	GMC_Sanctions	11	49
81	484	4	PBL_School	ARCP_NotExam	11	50
81	485	5	Spend_Student	Student_Staff	12	13
81	486	6	Spend_Student	Entrants_N	12	14
82	487	1	Spend_Student	Entrants_Female	12	15
82	488	2	Spend_Student	EntryGrades	12	16
82	489	3	Spend_Student	Entrants_NonHome	12	17
82	490	4	Spend_Student	Teaching_Factor1_Trad	12	18

Page	Plot Number	Number On Page	X variable	Y variable	X number	Y number
82	491	5	Spend_Student	Teaching_Factor2_Struc	12	19
82	492	6	Spend_Student	Teach_GP	12	20
83	493	1	Spend_Student	Teach_Psyc	12	21
83	494	2	Spend_Student	Teach_Anaes	12	22
83	495	3	Spend_Student	Teach_OG	12	23
83	496	4	Spend_Student	Teach_IntMed	12	24
83	497	5	Spend_Student	Teach_Surgery	12	25
83	498	6	Spend_Student	ExamTime	12	26
84	499	1	Spend_Student	SelfRegLearn	12	27
84	500	2	Spend_Student	NSS_Satisfn	12	28
84	501	3	Spend_Student	NSS_Feedback	12	29
84	502	4	Spend_Student	UKFPO_EPM	12	30
84	503	5	Spend_Student	UKFPO_SJT	12	31
84	504	6	Spend_Student	F1_Preparedness	12	32
85	505	1	Spend_Student	F1_Satisfn	12	33
85	506	2	Spend_Student	F1_Workload	12	34
85	507	3	Spend_Student	F1_Supervn	12	35
85	508	4	Spend_Student	Trainee_GP	12	36
85	509	5	Spend_Student	Trainee_Psyc	12	37
85	510	6	Spend_Student	TraineeApp_Surgery	12	38
86	511	1	Spend_Student	TraineeApp_Anaes	12	39
86	512	2	Spend_Student	GMC_PGexams	12	40
86	513	3	Spend_Student	MRCGP_AKT	12	41
86	514	4	Spend_Student	MRCGP_CSA	12	42
86	515	5	Spend_Student	FRCA_Pt1	12	43
86	516	6	Spend_Student	MRCOG_Pt1	12	44
87	517	1	Spend_Student	MRCOG_Pt2	12	45
87	518	2	Spend_Student	MRCP_Pt1	12	46
87	519	3	Spend_Student	MRCP_Pt2	12	47
87	520	4	Spend_Student	MRCP_PACES	12	48
87	521	5	Spend_Student	GMC_Sanctions	12	49
87	522	6	Spend_Student	ARCP_NotExam	12	50
88	523	1	Student_Staff	Entrants_N	13	14
88	524	2	Student_Staff	Entrants_Female	13	15
88	525	3	Student_Staff	EntryGrades	13	16
88	526	4	Student_Staff	Entrants_NonHome	13	17
88	527	5	Student_Staff	Teaching_Factor1_Trad	13	18
88	528	6	Student_Staff	Teaching_Factor2_Struc	13	19
89	529	1	Student_Staff	Teach_GP	13	20
89	530	2	Student_Staff	Teach_Psyc	13	21
89	531	3	Student_Staff	Teach_Anaes	13	22
89	532	4	Student_Staff	Teach_OG	13	23
89	533	5	Student_Staff	Teach_IntMed	13	24
89	534	6	Student_Staff	Teach_Surgery	13	25
90	535	1	Student_Staff	ExamTime	13	26
90	536	2	Student_Staff	SelfRegLearn	13	27
90	537	3	Student_Staff	NSS_Satisfn	13	28
90	538	4	Student_Staff	NSS_Feedback	13	29
90	539	5	Student_Staff	UKFPO_EPM	13	30

Page	Plot Number	Number On Page	X variable	Y variable	X number	Y number
90	540	6	Student_Staff	UKFPO_SJT	13	31
91	541	1	Student_Staff	F1_Preparedness	13	32
91	542	2	Student_Staff	F1_Satisfn	13	33
91	543	3	Student_Staff	F1_Workload	13	34
91	544	4	Student_Staff	F1_Supervn	13	35
91	545	5	Student_Staff	Trainee_GP	13	36
91	546	6	Student_Staff	Trainee_Psyc	13	37
92	547	1	Student_Staff	TraineeApp_Surgery	13	38
92	548	2	Student_Staff	TraineeApp_Anaes	13	39
92	549	3	Student_Staff	GMC_PGexams	13	40
92	550	4	Student_Staff	MRCGP_AKT	13	41
92	551	5	Student_Staff	MRCGP_CSA	13	42
92	552	6	Student_Staff	FRCA_Pt1	13	43
93	553	1	Student_Staff	MRCOG_Pt1	13	44
93	554	2	Student_Staff	MRCOG_Pt2	13	45
93	555	3	Student_Staff	MRCP_Pt1	13	46
93	556	4	Student_Staff	MRCP_Pt2	13	47
93	557	5	Student_Staff	MRCP_PACES	13	48
93	558	6	Student_Staff	GMC_Sanctions	13	49
94	559	1	Student_Staff	ARCP_NotExam	13	50
94	560	2	Entrants_N	Entrants_Female	14	15
94	561	3	Entrants_N	EntryGrades	14	16
94	562	4	Entrants_N	Entrants_NonHome	14	17
94	563	5	Entrants_N	Teaching_Factor1_Trad	14	18
94	564	6	Entrants_N	Teaching_Factor2_Struc	14	19
95	565	1	Entrants_N	Teach_GP	14	20
95	566	2	Entrants_N	Teach_Psyc	14	21
95	567	3	Entrants_N	Teach_Anaes	14	22
95	568	4	Entrants_N	Teach_OG	14	23
95	569	5	Entrants_N	Teach_IntMed	14	24
95	570	6	Entrants_N	Teach_Surgery	14	25
96	571	1	Entrants_N	ExamTime	14	26
96	572	2	Entrants_N	SelfRegLearn	14	27
96	573	3	Entrants_N	NSS_Satisfn	14	28
96	574	4	Entrants_N	NSS_Feedback	14	29
96	575	5	Entrants_N	UKFPO_EPM	14	30
96	576	6	Entrants_N	UKFPO_SJT	14	31
97	577	1	Entrants_N	F1_Preparedness	14	32
97	578	2	Entrants_N	F1_Satisfn	14	33
97	579	3	Entrants_N	F1_Workload	14	34
97	580	4	Entrants_N	F1_Supervn	14	35
97	581	5	Entrants_N	Trainee_GP	14	36
97	582	6	Entrants_N	Trainee_Psyc	14	37
98	583	1	Entrants_N	TraineeApp_Surgery	14	38
98	584	2	Entrants_N	TraineeApp_Anaes	14	39
98	585	3	Entrants_N	GMC_PGexams	14	40
98	586	4	Entrants_N	MRCGP_AKT	14	41
98	587	5	Entrants_N	MRCGP_CSA	14	42
98	588	6	Entrants_N	FRCA_Pt1	14	43

Page	Plot Number	Number On Page	X variable	Y variable	X number	Y number
99	589	1	Entrants_N	MRCOG_Pt1	14	44
99	590	2	Entrants_N	MRCOG_Pt2	14	45
99	591	3	Entrants_N	MRCP_Pt1	14	46
99	592	4	Entrants_N	MRCP_Pt2	14	47
99	593	5	Entrants_N	MRCP_PACES	14	48
99	594	6	Entrants_N	GMC_Sanctions	14	49
100	595	1	Entrants_N	ARCP_NotExam	14	50
100	596	2	Entrants_Female	EntryGrades	15	16
100	597	3	Entrants_Female	Entrants_NonHome	15	17
100	598	4	Entrants_Female	Teaching_Factor1_Trad	15	18
100	599	5	Entrants_Female	Teaching_Factor2_Struc	15	19
100	600	6	Entrants_Female	Teach_GP	15	20
101	601	1	Entrants_Female	Teach_Psyc	15	21
101	602	2	Entrants_Female	Teach_Anaes	15	22
101	603	3	Entrants_Female	Teach_OG	15	23
101	604	4	Entrants_Female	Teach_IntMed	15	24
101	605	5	Entrants_Female	Teach_Surgery	15	25
101	606	6	Entrants_Female	ExamTime	15	26
102	607	1	Entrants_Female	SelfRegLearn	15	27
102	608	2	Entrants_Female	NSS_Satisfn	15	28
102	609	3	Entrants_Female	NSS_Feedback	15	29
102	610	4	Entrants_Female	UKFPO_EPM	15	30
102	611	5	Entrants_Female	UKFPO_SJT	15	31
102	612	6	Entrants_Female	F1_Preparedness	15	32
103	613	1	Entrants_Female	F1_Satisfn	15	33
103	614	2	Entrants_Female	F1_Workload	15	34
103	615	3	Entrants_Female	F1_Supervn	15	35
103	616	4	Entrants_Female	Trainee_GP	15	36
103	617	5	Entrants_Female	Trainee_Psyc	15	37
103	618	6	Entrants_Female	TraineeApp_Surgery	15	38
104	619	1	Entrants_Female	TraineeApp_Anaes	15	39
104	620	2	Entrants_Female	GMC_PGexams	15	40
104	621	3	Entrants_Female	MRCGP_AKT	15	41
104	622	4	Entrants_Female	MRCGP_CSA	15	42
104	623	5	Entrants_Female	FRCA_Pt1	15	43
104	624	6	Entrants_Female	MRCOG_Pt1	15	44
105	625	1	Entrants_Female	MRCOG_Pt2	15	45
105	626	2	Entrants_Female	MRCP_Pt1	15	46
105	627	3	Entrants_Female	MRCP_Pt2	15	47
105	628	4	Entrants_Female	MRCP_PACES	15	48
105	629	5	Entrants_Female	GMC_Sanctions	15	49
105	630	6	Entrants_Female	ARCP_NotExam	15	50
106	631	1	EntryGrades	Entrants_NonHome	16	17
106	632	2	EntryGrades	Teaching_Factor1_Trad	16	18
106	633	3	EntryGrades	Teaching_Factor2_Struc	16	19
106	634	4	EntryGrades	Teach_GP	16	20
106	635	5	EntryGrades	Teach_Psyc	16	21
106	636	6	EntryGrades	Teach_Anaes	16	22
107	637	1	EntryGrades	Teach_OG	16	23

Page	Plot Number	Number On Page	X variable	Y variable	X number	Y number
107	638	2	EntryGrades	Teach_IntMed	16	24
107	639	3	EntryGrades	Teach_Surgery	16	25
107	640	4	EntryGrades	ExamTime	16	26
107	641	5	EntryGrades	SelfRegLearn	16	27
107	642	6	EntryGrades	NSS_Satisfn	16	28
108	643	1	EntryGrades	NSS_Feedback	16	29
108	644	2	EntryGrades	UKFPO_EPM	16	30
108	645	3	EntryGrades	UKFPO_SJT	16	31
108	646	4	EntryGrades	F1_Preparedness	16	32
108	647	5	EntryGrades	F1_Satisfn	16	33
108	648	6	EntryGrades	F1_Workload	16	34
109	649	1	EntryGrades	F1_Supervn	16	35
109	650	2	EntryGrades	Trainee_GP	16	36
109	651	3	EntryGrades	Trainee_Psyc	16	37
109	652	4	EntryGrades	TraineeApp_Surgery	16	38
109	653	5	EntryGrades	TraineeApp_Anaes	16	39
109	654	6	EntryGrades	GMC_PGexams	16	40
110	655	1	EntryGrades	MRCGP_AKT	16	41
110	656	2	EntryGrades	MRCGP_CSA	16	42
110	657	3	EntryGrades	FRCA_Pt1	16	43
110	658	4	EntryGrades	MRCOG_Pt1	16	44
110	659	5	EntryGrades	MRCOG_Pt2	16	45
110	660	6	EntryGrades	MRCP_Pt1	16	46
111	661	1	EntryGrades	MRCP_Pt2	16	47
111	662	2	EntryGrades	MRCP_PACES	16	48
111	663	3	EntryGrades	GMC_Sanctions	16	49
111	664	4	EntryGrades	ARCP_NotExam	16	50
111	665	5	Entrants_NonHome	Teaching_Factor1_Trad	17	18
111	666	6	Entrants_NonHome	Teaching_Factor2_Struc	17	19
112	667	1	Entrants_NonHome	Teach_GP	17	20
112	668	2	Entrants_NonHome	Teach_Psyc	17	21
112	669	3	Entrants_NonHome	Teach_Anaes	17	22
112	670	4	Entrants_NonHome	Teach_OG	17	23
112	671	5	Entrants_NonHome	Teach_IntMed	17	24
112	672	6	Entrants_NonHome	Teach_Surgery	17	25
113	673	1	Entrants_NonHome	ExamTime	17	26
113	674	2	Entrants_NonHome	SelfRegLearn	17	27
113	675	3	Entrants_NonHome	NSS_Satisfn	17	28
113	676	4	Entrants_NonHome	NSS_Feedback	17	29
113	677	5	Entrants_NonHome	UKFPO_EPM	17	30
113	678	6	Entrants_NonHome	UKFPO_SJT	17	31
114	679	1	Entrants_NonHome	F1_Preparedness	17	32
114	680	2	Entrants_NonHome	F1_Satisfn	17	33
114	681	3	Entrants_NonHome	F1_Workload	17	34
114	682	4	Entrants_NonHome	F1_Supervn	17	35
114	683	5	Entrants_NonHome	Trainee_GP	17	36
114	684	6	Entrants_NonHome	Trainee_Psyc	17	37
115	685	1	Entrants_NonHome	TraineeApp_Surgery	17	38
115	686	2	Entrants_NonHome	TraineeApp_Anaes	17	39

Page	Plot Number	Number On Page	X variable	Y variable	X number	Y number
115	687	3	Entrants_NonHome	GMC_PGexams	17	40
115	688	4	Entrants_NonHome	MRCGP_AKT	17	41
115	689	5	Entrants_NonHome	MRCGP_CSA	17	42
115	690	6	Entrants_NonHome	FRCA_Pt1	17	43
116	691	1	Entrants_NonHome	MRCOG_Pt1	17	44
116	692	2	Entrants_NonHome	MRCOG_Pt2	17	45
116	693	3	Entrants_NonHome	MRCP_Pt1	17	46
116	694	4	Entrants_NonHome	MRCP_Pt2	17	47
116	695	5	Entrants_NonHome	MRCP_PACES	17	48
116	696	6	Entrants_NonHome	GMC_Sanctions	17	49
117	697	1	Entrants_NonHome	ARCP_NotExam	17	50
117	698	2	Teaching_Factor1_Trad	Teaching_Factor2_Struc	18	19
117	699	3	Teaching_Factor1_Trad	Teach_GP	18	20
117	700	4	Teaching_Factor1_Trad	Teach_Psyc	18	21
117	701	5	Teaching_Factor1_Trad	Teach_Anaes	18	22
117	702	6	Teaching_Factor1_Trad	Teach_OG	18	23
118	703	1	Teaching_Factor1_Trad	Teach_IntMed	18	24
118	704	2	Teaching_Factor1_Trad	Teach_Surgery	18	25
118	705	3	Teaching_Factor1_Trad	ExamTime	18	26
118	706	4	Teaching_Factor1_Trad	SelfRegLearn	18	27
118	707	5	Teaching_Factor1_Trad	NSS_Satisfn	18	28
118	708	6	Teaching_Factor1_Trad	NSS_Feedback	18	29
119	709	1	Teaching_Factor1_Trad	UKFPO_EPM	18	30
119	710	2	Teaching_Factor1_Trad	UKFPO_SJT	18	31
119	711	3	Teaching_Factor1_Trad	F1_Preparedness	18	32
119	712	4	Teaching_Factor1_Trad	F1_Satisfn	18	33
119	713	5	Teaching_Factor1_Trad	F1_Workload	18	34
119	714	6	Teaching_Factor1_Trad	F1_Supervn	18	35
120	715	1	Teaching_Factor1_Trad	Trainee_GP	18	36
120	716	2	Teaching_Factor1_Trad	Trainee_Psyc	18	37
120	717	3	Teaching_Factor1_Trad	TraineeApp_Surgery	18	38
120	718	4	Teaching_Factor1_Trad	TraineeApp_Anaes	18	39
120	719	5	Teaching_Factor1_Trad	GMC_PGexams	18	40
120	720	6	Teaching_Factor1_Trad	MRCGP_AKT	18	41
121	721	1	Teaching_Factor1_Trad	MRCGP_CSA	18	42
121	722	2	Teaching_Factor1_Trad	FRCA_Pt1	18	43
121	723	3	Teaching_Factor1_Trad	MRCOG_Pt1	18	44
121	724	4	Teaching_Factor1_Trad	MRCOG_Pt2	18	45
121	725	5	Teaching_Factor1_Trad	MRCP_Pt1	18	46
121	726	6	Teaching_Factor1_Trad	MRCP_Pt2	18	47
122	727	1	Teaching_Factor1_Trad	MRCP_PACES	18	48
122	728	2	Teaching_Factor1_Trad	GMC_Sanctions	18	49
122	729	3	Teaching_Factor1_Trad	ARCP_NotExam	18	50
122	730	4	Teaching_Factor2_Struc	Teach_GP	19	20
122	731	5	Teaching_Factor2_Struc	Teach_Psyc	19	21
122	732	6	Teaching_Factor2_Struc	Teach_Anaes	19	22
123	733	1	Teaching_Factor2_Struc	Teach_OG	19	23
123	734	2	Teaching_Factor2_Struc	Teach_IntMed	19	24
123	735	3	Teaching_Factor2_Struc	Teach_Surgery	19	25

Page	Plot Number	Number On Page	X variable	Y variable	X number	Y number
123	736	4	Teaching_Factor2_Struc	ExamTime	19	26
123	737	5	Teaching_Factor2_Struc	SelfRegLearn	19	27
123	738	6	Teaching_Factor2_Struc	NSS_Satisfn	19	28
124	739	1	Teaching_Factor2_Struc	NSS_Feedback	19	29
124	740	2	Teaching_Factor2_Struc	UKFPO_EPM	19	30
124	741	3	Teaching_Factor2_Struc	UKFPO_SJT	19	31
124	742	4	Teaching_Factor2_Struc	F1_Preparedness	19	32
124	743	5	Teaching_Factor2_Struc	F1_Satisfn	19	33
124	744	6	Teaching_Factor2_Struc	F1_Workload	19	34
125	745	1	Teaching_Factor2_Struc	F1_Supervn	19	35
125	746	2	Teaching_Factor2_Struc	Trainee_GP	19	36
125	747	3	Teaching_Factor2_Struc	Trainee_Psyc	19	37
125	748	4	Teaching_Factor2_Struc	TraineeApp_Surgery	19	38
125	749	5	Teaching_Factor2_Struc	TraineeApp_Anaes	19	39
125	750	6	Teaching_Factor2_Struc	GMC_PGexams	19	40
126	751	1	Teaching_Factor2_Struc	MRCGP_AKT	19	41
126	752	2	Teaching_Factor2_Struc	MRCGP_CSA	19	42
126	753	3	Teaching_Factor2_Struc	FRCA_Pt1	19	43
126	754	4	Teaching_Factor2_Struc	MRCOG_Pt1	19	44
126	755	5	Teaching_Factor2_Struc	MRCOG_Pt2	19	45
126	756	6	Teaching_Factor2_Struc	MRCP_Pt1	19	46
127	757	1	Teaching_Factor2_Struc	MRCP_Pt2	19	47
127	758	2	Teaching_Factor2_Struc	MRCP_PACES	19	48
127	759	3	Teaching_Factor2_Struc	GMC_Sanctions	19	49
127	760	4	Teaching_Factor2_Struc	ARCP_NotExam	19	50
127	761	5	Teach_GP	Teach_Psyc	20	21
127	762	6	Teach_GP	Teach_Anaes	20	22
128	763	1	Teach_GP	Teach_OG	20	23
128	764	2	Teach_GP	Teach_IntMed	20	24
128	765	3	Teach_GP	Teach_Surgery	20	25
128	766	4	Teach_GP	ExamTime	20	26
128	767	5	Teach_GP	SelfRegLearn	20	27
128	768	6	Teach_GP	NSS_Satisfn	20	28
129	769	1	Teach_GP	NSS_Feedback	20	29
129	770	2	Teach_GP	UKFPO_EPM	20	30
129	771	3	Teach_GP	UKFPO_SJT	20	31
129	772	4	Teach_GP	F1_Preparedness	20	32
129	773	5	Teach_GP	F1_Satisfn	20	33
129	774	6	Teach_GP	F1_Workload	20	34
130	775	1	Teach_GP	F1_Supervn	20	35
130	776	2	Teach_GP	Trainee_GP	20	36
130	777	3	Teach_GP	Trainee_Psyc	20	37
130	778	4	Teach_GP	TraineeApp_Surgery	20	38
130	779	5	Teach_GP	TraineeApp_Anaes	20	39
130	780	6	Teach_GP	GMC_PGexams	20	40
131	781	1	Teach_GP	MRCGP_AKT	20	41
131	782	2	Teach_GP	MRCGP_CSA	20	42
131	783	3	Teach_GP	FRCA_Pt1	20	43
131	784	4	Teach_GP	MRCOG_Pt1	20	44

Page	Plot Number	Number On Page	X variable	Y variable	X number	Y number
131	785	5	Teach_GP	MRCOG_Pt2	20	45
131	786	6	Teach_GP	MRCP_Pt1	20	46
132	787	1	Teach_GP	MRCP_Pt2	20	47
132	788	2	Teach_GP	MRCP_PACES	20	48
132	789	3	Teach_GP	GMC_Sanctions	20	49
132	790	4	Teach_GP	ARCP_NotExam	20	50
132	791	5	Teach_Psyc	Teach_Anaes	21	22
132	792	6	Teach_Psyc	Teach_OG	21	23
133	793	1	Teach_Psyc	Teach_IntMed	21	24
133	794	2	Teach_Psyc	Teach_Surgery	21	25
133	795	3	Teach_Psyc	ExamTime	21	26
133	796	4	Teach_Psyc	SelfRegLearn	21	27
133	797	5	Teach_Psyc	NSS_Satisfn	21	28
133	798	6	Teach_Psyc	NSS_Feedback	21	29
134	799	1	Teach_Psyc	UKFPO_EPM	21	30
134	800	2	Teach_Psyc	UKFPO_SJT	21	31
134	801	3	Teach_Psyc	F1_Preparedness	21	32
134	802	4	Teach_Psyc	F1_Satisfn	21	33
134	803	5	Teach_Psyc	F1_Workload	21	34
134	804	6	Teach_Psyc	F1_Supervn	21	35
135	805	1	Teach_Psyc	Trainee_GP	21	36
135	806	2	Teach_Psyc	Trainee_Psyc	21	37
135	807	3	Teach_Psyc	TraineeApp_Surgery	21	38
135	808	4	Teach_Psyc	TraineeApp_Anaes	21	39
135	809	5	Teach_Psyc	GMC_PGexams	21	40
135	810	6	Teach_Psyc	MRCGP_AKT	21	41
136	811	1	Teach_Psyc	MRCGP_CSA	21	42
136	812	2	Teach_Psyc	FRCA_Pt1	21	43
136	813	3	Teach_Psyc	MRCOG_Pt1	21	44
136	814	4	Teach_Psyc	MRCOG_Pt2	21	45
136	815	5	Teach_Psyc	MRCP_Pt1	21	46
136	816	6	Teach_Psyc	MRCP_Pt2	21	47
137	817	1	Teach_Psyc	MRCP_PACES	21	48
137	818	2	Teach_Psyc	GMC_Sanctions	21	49
137	819	3	Teach_Psyc	ARCP_NotExam	21	50
137	820	4	Teach_Anaes	Teach_OG	22	23
137	821	5	Teach_Anaes	Teach_IntMed	22	24
137	822	6	Teach_Anaes	Teach_Surgery	22	25
138	823	1	Teach_Anaes	ExamTime	22	26
138	824	2	Teach_Anaes	SelfRegLearn	22	27
138	825	3	Teach_Anaes	NSS_Satisfn	22	28
138	826	4	Teach_Anaes	NSS_Feedback	22	29
138	827	5	Teach_Anaes	UKFPO_EPM	22	30
138	828	6	Teach_Anaes	UKFPO_SJT	22	31
139	829	1	Teach_Anaes	F1_Preparedness	22	32
139	830	2	Teach_Anaes	F1_Satisfn	22	33
139	831	3	Teach_Anaes	F1_Workload	22	34
139	832	4	Teach_Anaes	F1_Supervn	22	35
139	833	5	Teach_Anaes	Trainee_GP	22	36

Page	Plot Number	Number On Page	X variable	Y variable	X number	Y number
139	834	6	Teach_Anaes	Trainee_Psyc	22	37
140	835	1	Teach_Anaes	TraineeApp_Surgery	22	38
140	836	2	Teach_Anaes	TraineeApp_Anaes	22	39
140	837	3	Teach_Anaes	GMC_PGexams	22	40
140	838	4	Teach_Anaes	MRCGP_AKT	22	41
140	839	5	Teach_Anaes	MRCGP_CSA	22	42
140	840	6	Teach_Anaes	FRCA_Pt1	22	43
141	841	1	Teach_Anaes	MRCOG_Pt1	22	44
141	842	2	Teach_Anaes	MRCOG_Pt2	22	45
141	843	3	Teach_Anaes	MRCP_Pt1	22	46
141	844	4	Teach_Anaes	MRCP_Pt2	22	47
141	845	5	Teach_Anaes	MRCP_PACES	22	48
141	846	6	Teach_Anaes	GMC_Sanctions	22	49
142	847	1	Teach_Anaes	ARCP_NotExam	22	50
142	848	2	Teach_OG	Teach_IntMed	23	24
142	849	3	Teach_OG	Teach_Surgery	23	25
142	850	4	Teach_OG	ExamTime	23	26
142	851	5	Teach_OG	SelfRegLearn	23	27
142	852	6	Teach_OG	NSS_Satisfn	23	28
143	853	1	Teach_OG	NSS_Feedback	23	29
143	854	2	Teach_OG	UKFPO_EPM	23	30
143	855	3	Teach_OG	UKFPO_SJT	23	31
143	856	4	Teach_OG	F1_Preparedness	23	32
143	857	5	Teach_OG	F1_Satisfn	23	33
143	858	6	Teach_OG	F1_Workload	23	34
144	859	1	Teach_OG	F1_Supervn	23	35
144	860	2	Teach_OG	Trainee_GP	23	36
144	861	3	Teach_OG	Trainee_Psyc	23	37
144	862	4	Teach_OG	TraineeApp_Surgery	23	38
144	863	5	Teach_OG	TraineeApp_Anaes	23	39
144	864	6	Teach_OG	GMC_PGexams	23	40
145	865	1	Teach_OG	MRCGP_AKT	23	41
145	866	2	Teach_OG	MRCGP_CSA	23	42
145	867	3	Teach_OG	FRCA_Pt1	23	43
145	868	4	Teach_OG	MRCOG_Pt1	23	44
145	869	5	Teach_OG	MRCOG_Pt2	23	45
145	870	6	Teach_OG	MRCP_Pt1	23	46
146	871	1	Teach_OG	MRCP_Pt2	23	47
146	872	2	Teach_OG	MRCP_PACES	23	48
146	873	3	Teach_OG	GMC_Sanctions	23	49
146	874	4	Teach_OG	ARCP_NotExam	23	50
146	875	5	Teach_IntMed	Teach_Surgery	24	25
146	876	6	Teach_IntMed	ExamTime	24	26
147	877	1	Teach_IntMed	SelfRegLearn	24	27
147	878	2	Teach_IntMed	NSS_Satisfn	24	28
147	879	3	Teach_IntMed	NSS_Feedback	24	29
147	880	4	Teach_IntMed	UKFPO_EPM	24	30
147	881	5	Teach_IntMed	UKFPO_SJT	24	31
147	882	6	Teach_IntMed	F1_Preparedness	24	32

Page	Plot Number	Number On Page	X variable	Y variable	X number	Y number
148	883	1	Teach_IntMed	F1_Satisfn	24	33
148	884	2	Teach_IntMed	F1_Workload	24	34
148	885	3	Teach_IntMed	F1_Supervn	24	35
148	886	4	Teach_IntMed	Trainee_GP	24	36
148	887	5	Teach_IntMed	Trainee_Psyc	24	37
148	888	6	Teach_IntMed	TraineeApp_Surgery	24	38
149	889	1	Teach_IntMed	TraineeApp_Anaes	24	39
149	890	2	Teach_IntMed	GMC_PGexams	24	40
149	891	3	Teach_IntMed	MRCGP_AKT	24	41
149	892	4	Teach_IntMed	MRCGP_CSA	24	42
149	893	5	Teach_IntMed	FRCA_Pt1	24	43
149	894	6	Teach_IntMed	MRCOG_Pt1	24	44
150	895	1	Teach_IntMed	MRCOG_Pt2	24	45
150	896	2	Teach_IntMed	MRCP_Pt1	24	46
150	897	3	Teach_IntMed	MRCP_Pt2	24	47
150	898	4	Teach_IntMed	MRCP_PACES	24	48
150	899	5	Teach_IntMed	GMC_Sanctions	24	49
150	900	6	Teach_IntMed	ARCP_NotExam	24	50
151	901	1	Teach_Surgery	ExamTime	25	26
151	902	2	Teach_Surgery	SelfRegLearn	25	27
151	903	3	Teach_Surgery	NSS_Satisfn	25	28
151	904	4	Teach_Surgery	NSS_Feedback	25	29
151	905	5	Teach_Surgery	UKFPO_EPM	25	30
151	906	6	Teach_Surgery	UKFPO_SJT	25	31
152	907	1	Teach_Surgery	F1_Preparedness	25	32
152	908	2	Teach_Surgery	F1_Satisfn	25	33
152	909	3	Teach_Surgery	F1_Workload	25	34
152	910	4	Teach_Surgery	F1_Supervn	25	35
152	911	5	Teach_Surgery	Trainee_GP	25	36
152	912	6	Teach_Surgery	Trainee_Psyc	25	37
153	913	1	Teach_Surgery	TraineeApp_Surgery	25	38
153	914	2	Teach_Surgery	TraineeApp_Anaes	25	39
153	915	3	Teach_Surgery	GMC_PGexams	25	40
153	916	4	Teach_Surgery	MRCGP_AKT	25	41
153	917	5	Teach_Surgery	MRCGP_CSA	25	42
153	918	6	Teach_Surgery	FRCA_Pt1	25	43
154	919	1	Teach_Surgery	MRCOG_Pt1	25	44
154	920	2	Teach_Surgery	MRCOG_Pt2	25	45
154	921	3	Teach_Surgery	MRCP_Pt1	25	46
154	922	4	Teach_Surgery	MRCP_Pt2	25	47
154	923	5	Teach_Surgery	MRCP_PACES	25	48
154	924	6	Teach_Surgery	GMC_Sanctions	25	49
155	925	1	Teach_Surgery	ARCP_NotExam	25	50
155	926	2	ExamTime	SelfRegLearn	26	27
155	927	3	ExamTime	NSS_Satisfn	26	28
155	928	4	ExamTime	NSS_Feedback	26	29
155	929	5	ExamTime	UKFPO_EPM	26	30
155	930	6	ExamTime	UKFPO_SJT	26	31
156	931	1	ExamTime	F1_Preparedness	26	32

Page	Plot Number	Number On Page	X variable	Y variable	X number	Y number
156	932	2	ExamTime	F1_Satisfn	26	33
156	933	3	ExamTime	F1_Workload	26	34
156	934	4	ExamTime	F1_Supervn	26	35
156	935	5	ExamTime	Trainee_GP	26	36
156	936	6	ExamTime	Trainee_Psyc	26	37
157	937	1	ExamTime	TraineeApp_Surgery	26	38
157	938	2	ExamTime	TraineeApp_Anaes	26	39
157	939	3	ExamTime	GMC_PGexams	26	40
157	940	4	ExamTime	MRCGP_AKT	26	41
157	941	5	ExamTime	MRCGP_CSA	26	42
157	942	6	ExamTime	FRCA_Pt1	26	43
158	943	1	ExamTime	MRCOG_Pt1	26	44
158	944	2	ExamTime	MRCOG_Pt2	26	45
158	945	3	ExamTime	MRCP_Pt1	26	46
158	946	4	ExamTime	MRCP_Pt2	26	47
158	947	5	ExamTime	MRCP_PACES	26	48
158	948	6	ExamTime	GMC_Sanctions	26	49
159	949	1	ExamTime	ARCP_NotExam	26	50
159	950	2	SelfRegLearn	NSS_Satisfn	27	28
159	951	3	SelfRegLearn	NSS_Feedback	27	29
159	952	4	SelfRegLearn	UKFPO_EPM	27	30
159	953	5	SelfRegLearn	UKFPO_SJT	27	31
159	954	6	SelfRegLearn	F1_Preparedness	27	32
160	955	1	SelfRegLearn	F1_Satisfn	27	33
160	956	2	SelfRegLearn	F1_Workload	27	34
160	957	3	SelfRegLearn	F1_Supervn	27	35
160	958	4	SelfRegLearn	Trainee_GP	27	36
160	959	5	SelfRegLearn	Trainee_Psyc	27	37
160	960	6	SelfRegLearn	TraineeApp_Surgery	27	38
161	961	1	SelfRegLearn	TraineeApp_Anaes	27	39
161	962	2	SelfRegLearn	GMC_PGexams	27	40
161	963	3	SelfRegLearn	MRCGP_AKT	27	41
161	964	4	SelfRegLearn	MRCGP_CSA	27	42
161	965	5	SelfRegLearn	FRCA_Pt1	27	43
161	966	6	SelfRegLearn	MRCOG_Pt1	27	44
162	967	1	SelfRegLearn	MRCOG_Pt2	27	45
162	968	2	SelfRegLearn	MRCP_Pt1	27	46
162	969	3	SelfRegLearn	MRCP_Pt2	27	47
162	970	4	SelfRegLearn	MRCP_PACES	27	48
162	971	5	SelfRegLearn	GMC_Sanctions	27	49
162	972	6	SelfRegLearn	ARCP_NotExam	27	50
163	973	1	NSS_Satisfn	NSS_Feedback	28	29
163	974	2	NSS_Satisfn	UKFPO_EPM	28	30
163	975	3	NSS_Satisfn	UKFPO_SJT	28	31
163	976	4	NSS_Satisfn	F1_Preparedness	28	32
163	977	5	NSS_Satisfn	F1_Satisfn	28	33
163	978	6	NSS_Satisfn	F1_Workload	28	34
164	979	1	NSS_Satisfn	F1_Supervn	28	35
164	980	2	NSS_Satisfn	Trainee_GP	28	36

Page	Plot Number	Number On Page	X variable	Y variable	X number	Y number
164	981	3	NSS_Satisfn	Trainee_Psyc	28	37
164	982	4	NSS_Satisfn	TraineeApp_Surgery	28	38
164	983	5	NSS_Satisfn	TraineeApp_Anaes	28	39
164	984	6	NSS_Satisfn	GMC_PGexams	28	40
165	985	1	NSS_Satisfn	MRCGP_AKT	28	41
165	986	2	NSS_Satisfn	MRCGP_CSA	28	42
165	987	3	NSS_Satisfn	FRCA_Pt1	28	43
165	988	4	NSS_Satisfn	MRCOG_Pt1	28	44
165	989	5	NSS_Satisfn	MRCOG_Pt2	28	45
165	990	6	NSS_Satisfn	MRCP_Pt1	28	46
166	991	1	NSS_Satisfn	MRCP_Pt2	28	47
166	992	2	NSS_Satisfn	MRCP_PACES	28	48
166	993	3	NSS_Satisfn	GMC_Sanctions	28	49
166	994	4	NSS_Satisfn	ARCP_NotExam	28	50
166	995	5	NSS_Feedback	UKFPO_EPM	29	30
166	996	6	NSS_Feedback	UKFPO_SJT	29	31
167	997	1	NSS_Feedback	F1_Preparedness	29	32
167	998	2	NSS_Feedback	F1_Satisfn	29	33
167	999	3	NSS_Feedback	F1_Workload	29	34
167	1000	4	NSS_Feedback	F1_Supervn	29	35
167	1001	5	NSS_Feedback	Trainee_GP	29	36
167	1002	6	NSS_Feedback	Trainee_Psyc	29	37
168	1003	1	NSS_Feedback	TraineeApp_Surgery	29	38
168	1004	2	NSS_Feedback	TraineeApp_Anaes	29	39
168	1005	3	NSS_Feedback	GMC_PGexams	29	40
168	1006	4	NSS_Feedback	MRCGP_AKT	29	41
168	1007	5	NSS_Feedback	MRCGP_CSA	29	42
168	1008	6	NSS_Feedback	FRCA_Pt1	29	43
169	1009	1	NSS_Feedback	MRCOG_Pt1	29	44
169	1010	2	NSS_Feedback	MRCOG_Pt2	29	45
169	1011	3	NSS_Feedback	MRCP_Pt1	29	46
169	1012	4	NSS_Feedback	MRCP_Pt2	29	47
169	1013	5	NSS_Feedback	MRCP_PACES	29	48
169	1014	6	NSS_Feedback	GMC_Sanctions	29	49
170	1015	1	NSS_Feedback	ARCP_NotExam	29	50
170	1016	2	UKFPO_EPM	UKFPO_SJT	30	31
170	1017	3	UKFPO_EPM	F1_Preparedness	30	32
170	1018	4	UKFPO_EPM	F1_Satisfn	30	33
170	1019	5	UKFPO_EPM	F1_Workload	30	34
170	1020	6	UKFPO_EPM	F1_Supervn	30	35
171	1021	1	UKFPO_EPM	Trainee_GP	30	36
171	1022	2	UKFPO_EPM	Trainee_Psyc	30	37
171	1023	3	UKFPO_EPM	TraineeApp_Surgery	30	38
171	1024	4	UKFPO_EPM	TraineeApp_Anaes	30	39
171	1025	5	UKFPO_EPM	GMC_PGexams	30	40
171	1026	6	UKFPO_EPM	MRCGP_AKT	30	41
172	1027	1	UKFPO_EPM	MRCGP_CSA	30	42
172	1028	2	UKFPO_EPM	FRCA_Pt1	30	43
172	1029	3	UKFPO_EPM	MRCOG_Pt1	30	44

Page	Plot Number	Number On Page	X variable	Y variable	X number	Y number
172	1030	4	UKFPO_EPM	MRCOG_Pt2	30	45
172	1031	5	UKFPO_EPM	MRCP_Pt1	30	46
172	1032	6	UKFPO_EPM	MRCP_Pt2	30	47
173	1033	1	UKFPO_EPM	MRCP_PACES	30	48
173	1034	2	UKFPO_EPM	GMC_Sanctions	30	49
173	1035	3	UKFPO_EPM	ARCP_NotExam	30	50
173	1036	4	UKFPO_SJT	F1_Preparedness	31	32
173	1037	5	UKFPO_SJT	F1_Satisfn	31	33
173	1038	6	UKFPO_SJT	F1_Workload	31	34
174	1039	1	UKFPO_SJT	F1_Supervn	31	35
174	1040	2	UKFPO_SJT	Trainee_GP	31	36
174	1041	3	UKFPO_SJT	Trainee_Psyc	31	37
174	1042	4	UKFPO_SJT	TraineeApp_Surgery	31	38
174	1043	5	UKFPO_SJT	TraineeApp_Anaes	31	39
174	1044	6	UKFPO_SJT	GMC_PGexams	31	40
175	1045	1	UKFPO_SJT	MRCGP_AKT	31	41
175	1046	2	UKFPO_SJT	MRCGP_CSA	31	42
175	1047	3	UKFPO_SJT	FRCA_Pt1	31	43
175	1048	4	UKFPO_SJT	MRCOG_Pt1	31	44
175	1049	5	UKFPO_SJT	MRCOG_Pt2	31	45
175	1050	6	UKFPO_SJT	MRCP_Pt1	31	46
176	1051	1	UKFPO_SJT	MRCP_Pt2	31	47
176	1052	2	UKFPO_SJT	MRCP_PACES	31	48
176	1053	3	UKFPO_SJT	GMC_Sanctions	31	49
176	1054	4	UKFPO_SJT	ARCP_NotExam	31	50
176	1055	5	F1_Preparedness	F1_Satisfn	32	33
176	1056	6	F1_Preparedness	F1_Workload	32	34
177	1057	1	F1_Preparedness	F1_Supervn	32	35
177	1058	2	F1_Preparedness	Trainee_GP	32	36
177	1059	3	F1_Preparedness	Trainee_Psyc	32	37
177	1060	4	F1_Preparedness	TraineeApp_Surgery	32	38
177	1061	5	F1_Preparedness	TraineeApp_Anaes	32	39
177	1062	6	F1_Preparedness	GMC_PGexams	32	40
178	1063	1	F1_Preparedness	MRCGP_AKT	32	41
178	1064	2	F1_Preparedness	MRCGP_CSA	32	42
178	1065	3	F1_Preparedness	FRCA_Pt1	32	43
178	1066	4	F1_Preparedness	MRCOG_Pt1	32	44
178	1067	5	F1_Preparedness	MRCOG_Pt2	32	45
178	1068	6	F1_Preparedness	MRCP_Pt1	32	46
179	1069	1	F1_Preparedness	MRCP_Pt2	32	47
179	1070	2	F1_Preparedness	MRCP_PACES	32	48
179	1071	3	F1_Preparedness	GMC_Sanctions	32	49
179	1072	4	F1_Preparedness	ARCP_NotExam	32	50
179	1073	5	F1_Satisfn	F1_Workload	33	34
179	1074	6	F1_Satisfn	F1_Supervn	33	35
180	1075	1	F1_Satisfn	Trainee_GP	33	36
180	1076	2	F1_Satisfn	Trainee_Psyc	33	37
180	1077	3	F1_Satisfn	TraineeApp_Surgery	33	38
180	1078	4	F1_Satisfn	TraineeApp_Anaes	33	39

Page	Plot Number	Number On Page	X variable	Y variable	X number	Y number
180	1079	5	F1_Satisfn	GMC_PGexams	33	40
180	1080	6	F1_Satisfn	MRCGP_AKT	33	41
181	1081	1	F1_Satisfn	MRCGP_CSA	33	42
181	1082	2	F1_Satisfn	FRCA_Pt1	33	43
181	1083	3	F1_Satisfn	MRCOG_Pt1	33	44
181	1084	4	F1_Satisfn	MRCOG_Pt2	33	45
181	1085	5	F1_Satisfn	MRCP_Pt1	33	46
181	1086	6	F1_Satisfn	MRCP_Pt2	33	47
182	1087	1	F1_Satisfn	MRCP_PACES	33	48
182	1088	2	F1_Satisfn	GMC_Sanctions	33	49
182	1089	3	F1_Satisfn	ARCP_NotExam	33	50
182	1090	4	F1_Workload	F1_Supervn	34	35
182	1091	5	F1_Workload	Trainee_GP	34	36
182	1092	6	F1_Workload	Trainee_Psyc	34	37
183	1093	1	F1_Workload	TraineeApp_Surgery	34	38
183	1094	2	F1_Workload	TraineeApp_Anaes	34	39
183	1095	3	F1_Workload	GMC_PGexams	34	40
183	1096	4	F1_Workload	MRCGP_AKT	34	41
183	1097	5	F1_Workload	MRCGP_CSA	34	42
183	1098	6	F1_Workload	FRCA_Pt1	34	43
184	1099	1	F1_Workload	MRCOG_Pt1	34	44
184	1100	2	F1_Workload	MRCOG_Pt2	34	45
184	1101	3	F1_Workload	MRCP_Pt1	34	46
184	1102	4	F1_Workload	MRCP_Pt2	34	47
184	1103	5	F1_Workload	MRCP_PACES	34	48
184	1104	6	F1_Workload	GMC_Sanctions	34	49
185	1105	1	F1_Workload	ARCP_NotExam	34	50
185	1106	2	F1_Supervn	Trainee_GP	35	36
185	1107	3	F1_Supervn	Trainee_Psyc	35	37
185	1108	4	F1_Supervn	TraineeApp_Surgery	35	38
185	1109	5	F1_Supervn	TraineeApp_Anaes	35	39
185	1110	6	F1_Supervn	GMC_PGexams	35	40
186	1111	1	F1_Supervn	MRCGP_AKT	35	41
186	1112	2	F1_Supervn	MRCGP_CSA	35	42
186	1113	3	F1_Supervn	FRCA_Pt1	35	43
186	1114	4	F1_Supervn	MRCOG_Pt1	35	44
186	1115	5	F1_Supervn	MRCOG_Pt2	35	45
186	1116	6	F1_Supervn	MRCP_Pt1	35	46
187	1117	1	F1_Supervn	MRCP_Pt2	35	47
187	1118	2	F1_Supervn	MRCP_PACES	35	48
187	1119	3	F1_Supervn	GMC_Sanctions	35	49
187	1120	4	F1_Supervn	ARCP_NotExam	35	50
187	1121	5	Trainee_GP	Trainee_Psyc	36	37
187	1122	6	Trainee_GP	TraineeApp_Surgery	36	38
188	1123	1	Trainee_GP	TraineeApp_Anaes	36	39
188	1124	2	Trainee_GP	GMC_PGexams	36	40
188	1125	3	Trainee_GP	MRCGP_AKT	36	41
188	1126	4	Trainee_GP	MRCGP_CSA	36	42
188	1127	5	Trainee_GP	FRCA_Pt1	36	43

Page	Plot Number	Number On Page	X variable	Y variable	X number	Y number
188	1128	6	Trainee_GP	MRCOG_Pt1	36	44
189	1129	1	Trainee_GP	MRCOG_Pt2	36	45
189	1130	2	Trainee_GP	MRCP_Pt1	36	46
189	1131	3	Trainee_GP	MRCP_Pt2	36	47
189	1132	4	Trainee_GP	MRCP_PACES	36	48
189	1133	5	Trainee_GP	GMC_Sanctions	36	49
189	1134	6	Trainee_GP	ARCP_NotExam	36	50
190	1135	1	Trainee_Psyc	TraineeApp_Surgery	37	38
190	1136	2	Trainee_Psyc	TraineeApp_Anaes	37	39
190	1137	3	Trainee_Psyc	GMC_PGexams	37	40
190	1138	4	Trainee_Psyc	MRCGP_AKT	37	41
190	1139	5	Trainee_Psyc	MRCGP_CSA	37	42
190	1140	6	Trainee_Psyc	FRCA_Pt1	37	43
191	1141	1	Trainee_Psyc	MRCOG_Pt1	37	44
191	1142	2	Trainee_Psyc	MRCOG_Pt2	37	45
191	1143	3	Trainee_Psyc	MRCP_Pt1	37	46
191	1144	4	Trainee_Psyc	MRCP_Pt2	37	47
191	1145	5	Trainee_Psyc	MRCP_PACES	37	48
191	1146	6	Trainee_Psyc	GMC_Sanctions	37	49
192	1147	1	Trainee_Psyc	ARCP_NotExam	37	50
192	1148	2	TraineeApp_Surgery	TraineeApp_Anaes	38	39
192	1149	3	TraineeApp_Surgery	GMC_PGexams	38	40
192	1150	4	TraineeApp_Surgery	MRCGP_AKT	38	41
192	1151	5	TraineeApp_Surgery	MRCGP_CSA	38	42
192	1152	6	TraineeApp_Surgery	FRCA_Pt1	38	43
193	1153	1	TraineeApp_Surgery	MRCOG_Pt1	38	44
193	1154	2	TraineeApp_Surgery	MRCOG_Pt2	38	45
193	1155	3	TraineeApp_Surgery	MRCP_Pt1	38	46
193	1156	4	TraineeApp_Surgery	MRCP_Pt2	38	47
193	1157	5	TraineeApp_Surgery	MRCP_PACES	38	48
193	1158	6	TraineeApp_Surgery	GMC_Sanctions	38	49
194	1159	1	TraineeApp_Surgery	ARCP_NotExam	38	50
194	1160	2	TraineeApp_Anaes	GMC_PGexams	39	40
194	1161	3	TraineeApp_Anaes	MRCGP_AKT	39	41
194	1162	4	TraineeApp_Anaes	MRCGP_CSA	39	42
194	1163	5	TraineeApp_Anaes	FRCA_Pt1	39	43
194	1164	6	TraineeApp_Anaes	MRCOG_Pt1	39	44
195	1165	1	TraineeApp_Anaes	MRCOG_Pt2	39	45
195	1166	2	TraineeApp_Anaes	MRCP_Pt1	39	46
195	1167	3	TraineeApp_Anaes	MRCP_Pt2	39	47
195	1168	4	TraineeApp_Anaes	MRCP_PACES	39	48
195	1169	5	TraineeApp_Anaes	GMC_Sanctions	39	49
195	1170	6	TraineeApp_Anaes	ARCP_NotExam	39	50
196	1171	1	GMC_PGexams	MRCGP_AKT	40	41
196	1172	2	GMC_PGexams	MRCGP_CSA	40	42
196	1173	3	GMC_PGexams	FRCA_Pt1	40	43
196	1174	4	GMC_PGexams	MRCOG_Pt1	40	44
196	1175	5	GMC_PGexams	MRCOG_Pt2	40	45
196	1176	6	GMC_PGexams	MRCP_Pt1	40	46

Page	Plot Number	Number On Page	X variable	Y variable	X number	Y number
197	1177	1	GMC_PGexams	MRCP_Pt2	40	47
197	1178	2	GMC_PGexams	MRCP_PACES	40	48
197	1179	3	GMC_PGexams	GMC_Sanctions	40	49
197	1180	4	GMC_PGexams	ARCP_NotExam	40	50
197	1181	5	MRCGP_AKT	MRCGP_CSA	41	42
197	1182	6	MRCGP_AKT	FRCA_Pt1	41	43
198	1183	1	MRCGP_AKT	MRCOG_Pt1	41	44
198	1184	2	MRCGP_AKT	MRCOG_Pt2	41	45
198	1185	3	MRCGP_AKT	MRCP_Pt1	41	46
198	1186	4	MRCGP_AKT	MRCP_Pt2	41	47
198	1187	5	MRCGP_AKT	MRCP_PACES	41	48
198	1188	6	MRCGP_AKT	GMC_Sanctions	41	49
199	1189	1	MRCGP_AKT	ARCP_NotExam	41	50
199	1190	2	MRCGP_CSA	FRCA_Pt1	42	43
199	1191	3	MRCGP_CSA	MRCOG_Pt1	42	44
199	1192	4	MRCGP_CSA	MRCOG_Pt2	42	45
199	1193	5	MRCGP_CSA	MRCP_Pt1	42	46
199	1194	6	MRCGP_CSA	MRCP_Pt2	42	47
200	1195	1	MRCGP_CSA	MRCP_PACES	42	48
200	1196	2	MRCGP_CSA	GMC_Sanctions	42	49
200	1197	3	MRCGP_CSA	ARCP_NotExam	42	50
200	1198	4	FRCA_Pt1	MRCOG_Pt1	43	44
200	1199	5	FRCA_Pt1	MRCOG_Pt2	43	45
200	1200	6	FRCA_Pt1	MRCP_Pt1	43	46
201	1201	1	FRCA_Pt1	MRCP_Pt2	43	47
201	1202	2	FRCA_Pt1	MRCP_PACES	43	48
201	1203	3	FRCA_Pt1	GMC_Sanctions	43	49
201	1204	4	FRCA_Pt1	ARCP_NotExam	43	50
201	1205	5	MRCOG_Pt1	MRCOG_Pt2	44	45
201	1206	6	MRCOG_Pt1	MRCP_Pt1	44	46
202	1207	1	MRCOG_Pt1	MRCP_Pt2	44	47
202	1208	2	MRCOG_Pt1	MRCP_PACES	44	48
202	1209	3	MRCOG_Pt1	GMC_Sanctions	44	49
202	1210	4	MRCOG_Pt1	ARCP_NotExam	44	50
202	1211	5	MRCOG_Pt2	MRCP_Pt1	45	46
202	1212	6	MRCOG_Pt2	MRCP_Pt2	45	47
203	1213	1	MRCOG_Pt2	MRCP_PACES	45	48
203	1214	2	MRCOG_Pt2	GMC_Sanctions	45	49
203	1215	3	MRCOG_Pt2	ARCP_NotExam	45	50
203	1216	4	MRCP_Pt1	MRCP_Pt2	46	47
203	1217	5	MRCP_Pt1	MRCP_PACES	46	48
203	1218	6	MRCP_Pt1	GMC_Sanctions	46	49
204	1219	1	MRCP_Pt1	ARCP_NotExam	46	50
204	1220	2	MRCP_Pt2	MRCP_PACES	47	48
204	1221	3	MRCP_Pt2	GMC_Sanctions	47	49
204	1222	4	MRCP_Pt2	ARCP_NotExam	47	50
204	1223	5	MRCP_PACES	GMC_Sanctions	48	49
204	1224	6	MRCP_PACES	ARCP_NotExam	48	50
205	1225	1	GMC_Sanctions	ARCP_NotExam	49	50
