# Supplementary material for: Exploring UK medical school differences: the MedDifs study of selection, teaching, student and F1 perceptions, postgraduate outcomes and fitness to practise
Source: BMC Med. 2020 May 14;18:136. doi: 10.1186/s12916-020-01572-3 (PMC7222458; doi:10.1186/s12916-020-01572-3)
Supplement: Supplementary file 4 — Additional file 4. Graphs 1 to 210 (pages 1 to 35). [file 12916_2020_1572_MOESM4_ESM.pdf]

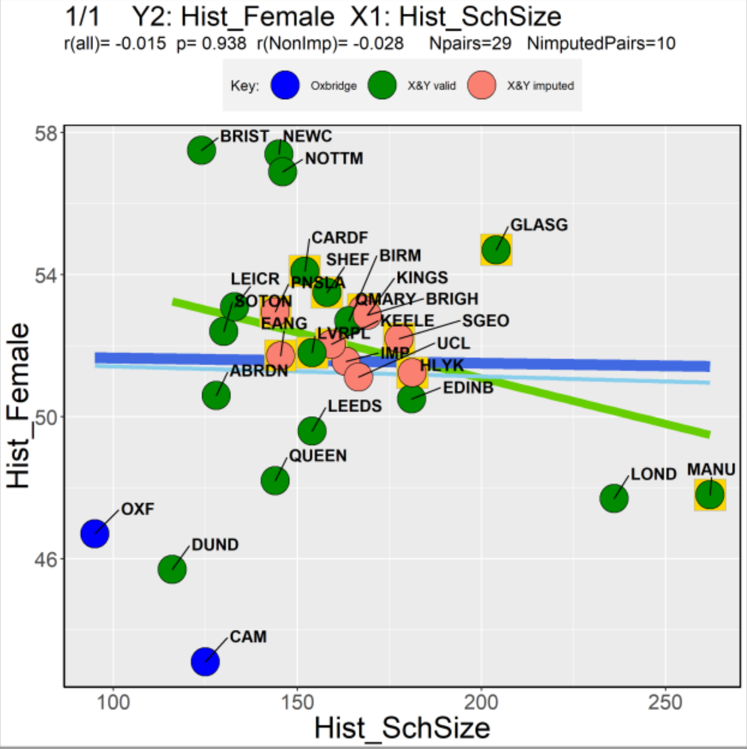
1/1 Y2: Hist_Female X1: Hist_SchSize
r(all)= -0.015 p= 0.938 r(NonImp)= -0.028 Npairs=29 NimputedPairs=10
Key: Oxbridge X&Y valid X&Y imputed
Hist_Female
Hist_SchSize
BRIST NEWC NOTTM CARDF SHEF BIRM GLASG LEICR PNSLA KINGS SOTON QMARY BRIGH EANG KEELE SGEO LVRPL UCL IMP HLYK ABRDN EDINB LEEDS QUEEN LOND MANU OXF DUND CAM

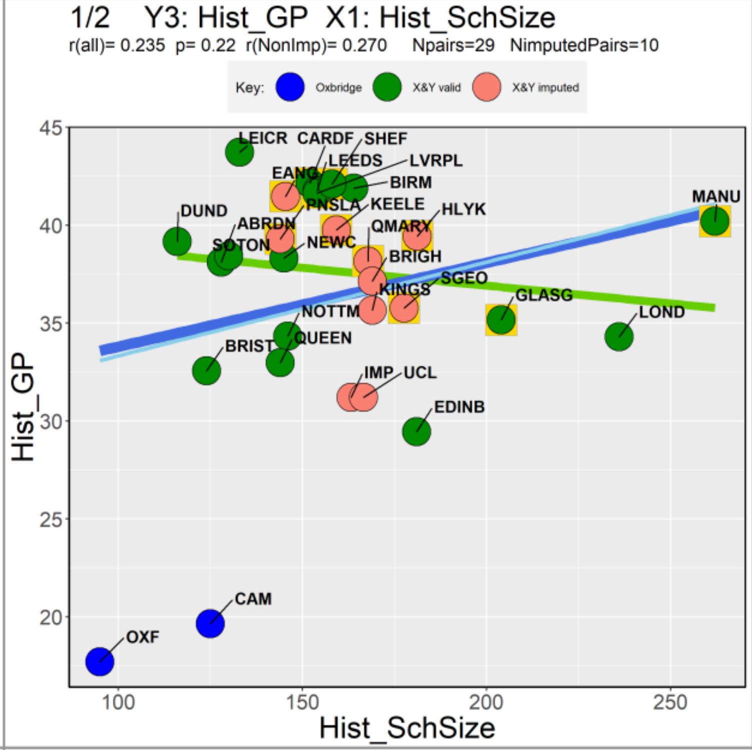
1/2 Y3: Hist_GP X1: Hist_SchSize
r(all)= 0.235 p= 0.22 r(NonImp)= 0.270 Npairs=29 NimputedPairs=10
Key: Oxbridge X&Y valid X&Y imputed
Hist_GP
Hist_SchSize
LEICR CARDF SHEF LEEDS LVRPL EANG BIRM PNSLA KEELE DUND ABRDN HLYK QMARY MANU SOTON NEWC BRIGH SGEO KINGS GLASG NOTTM LOND BRIST QUEEN IMP UCL EDINB CAM OXF

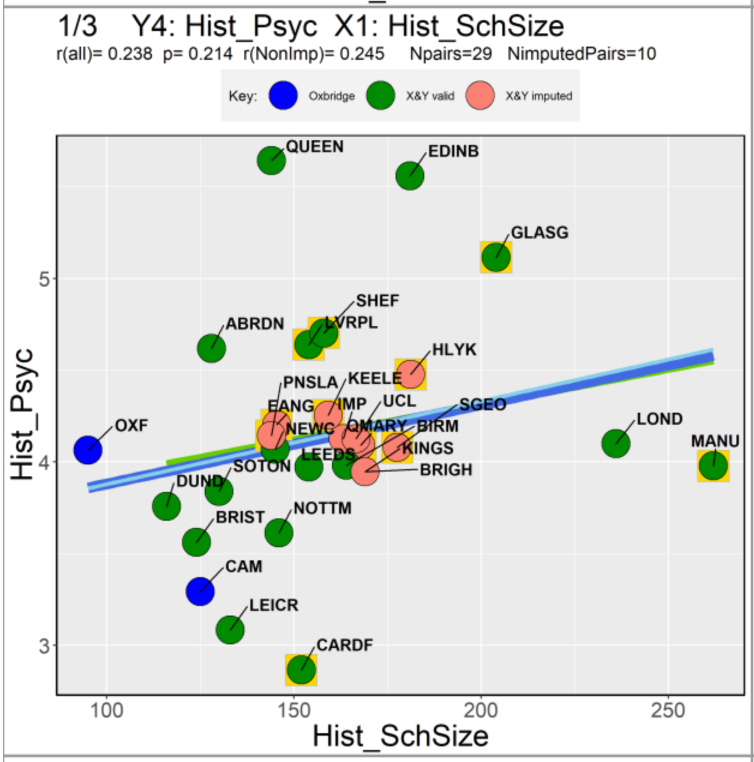
1/3 Y4: Hist_Psyc X1: Hist_SchSize
r(all)= 0.238 p= 0.214 r(NonImp)= 0.245 Npairs=29 NimputedPairs=10
Key: Oxbridge X&Y valid X&Y imputed
Hist_Psyc
Hist_SchSize
QUEEN EDINB GLASG SHEF ABRDN LVRPL HLYK PNSLA KEELE EANG IMP UCL SGEO OXF NEWC QMARY BIRM LOND KINGS MANU LEEDS SOTON BRIGH DUND BRIST NOTTM CAM LEICR CARDF

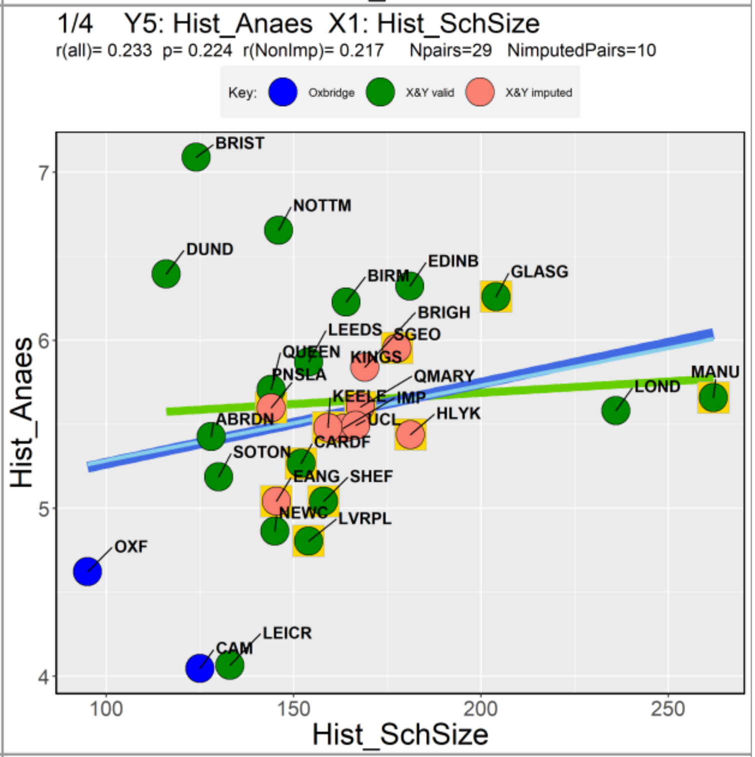
1/4 Y5: Hist_Anaes X1: Hist_SchSize
r(all)= 0.233 p= 0.224 r(NonImp)= 0.217 Npairs=29 NimputedPairs=10
Key: Oxbridge X&Y valid X&Y imputed
Hist_Anaes
Hist_SchSize
BRIST NOTTM DUND EDINB BIRM GLASG BRIGH LEEDS SGEO QUEEN KINGS PNSLA QMARY MANU LOND KEELE IMP ABRDN UCL HLYK CARDF SOTON EANG SHEF NEWC LVRPL OXF LEICR CAM

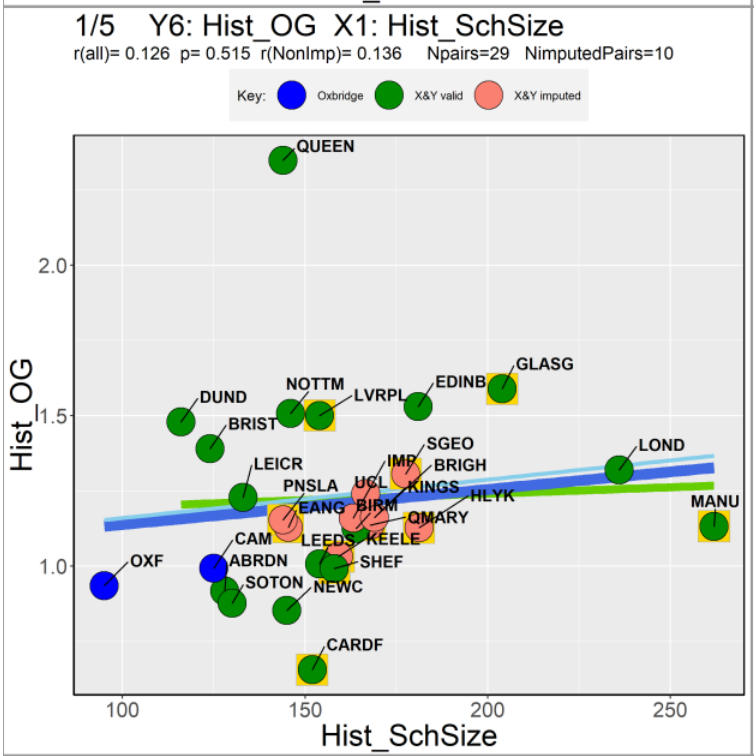
1/5 Y6: Hist_OG X1: Hist_SchSize
r(all)= 0.126 p= 0.515 r(NonImp)= 0.136 Npairs=29 NimputedPairs=10
Key: Oxbridge X&Y valid X&Y imputed
Hist_OG
Hist_SchSize
QUEEN GLASG EDINB NOTTM LVRPL DUND BRIST LOND SGEO LEICR IMP BRIGH PNSLA UCL KINGS EANG BIRM HLYK QMARY MANU CAM LEEDS KEELE ABRDN SHEF OXF SOTON NEWC CARDF

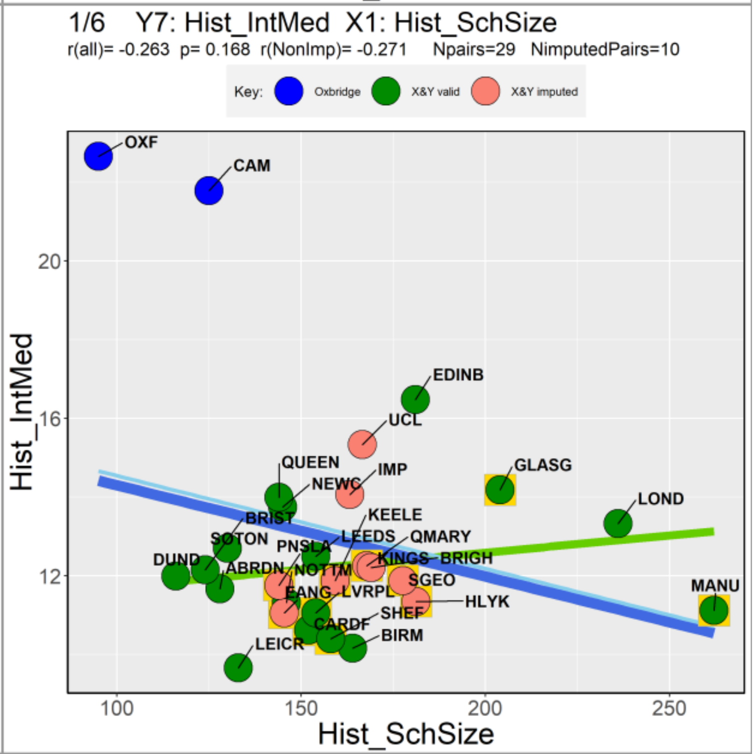
1/6 Y7: Hist_IntMed X1: Hist_SchSize
r(all)= -0.263 p= 0.168 r(NonImp)= -0.271 Npairs=29 NimputedPairs=10
Key: Oxbridge X&Y valid X&Y imputed
Hist_IntMed
Hist_SchSize
OXF CAM EDINB UCL QUEEN IMP GLASG NEWC LOND KEELE BRIST SOTON LEEDS QMARY PNSLA DUND KINGS BRIGH ABRDN NOTTM SGEO EANG LVRPL MANU SHEF HLYK CARDF BIRM LEICR

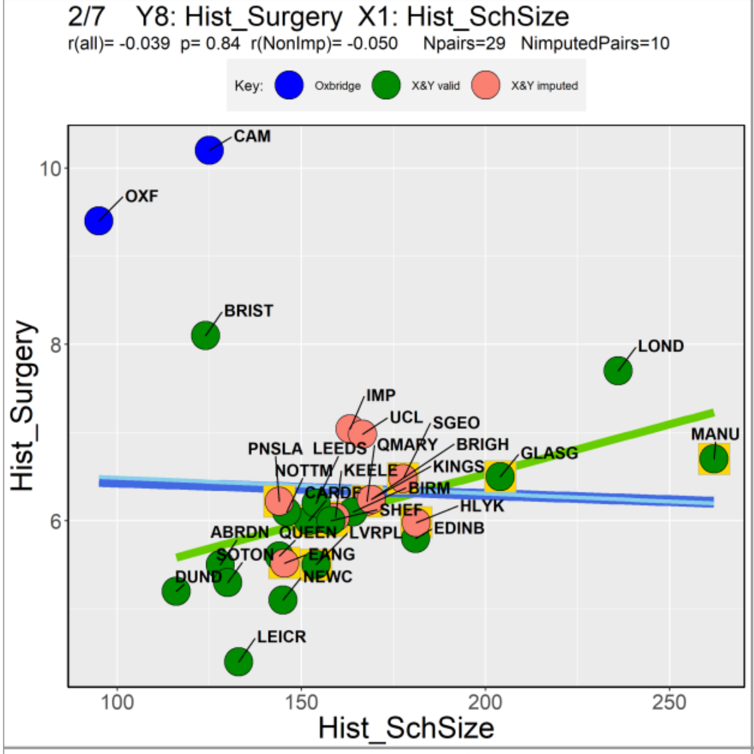
2/7 Y8: Hist_Surgery X1: Hist_SchSize
r(all)= -0.039 p= 0.84 r(NonImp)= -0.050 Npairs=29 NimputedPairs=10
Key: Oxbridge X&Y valid X&Y imputed
Hist_Surgery
Hist_SchSize

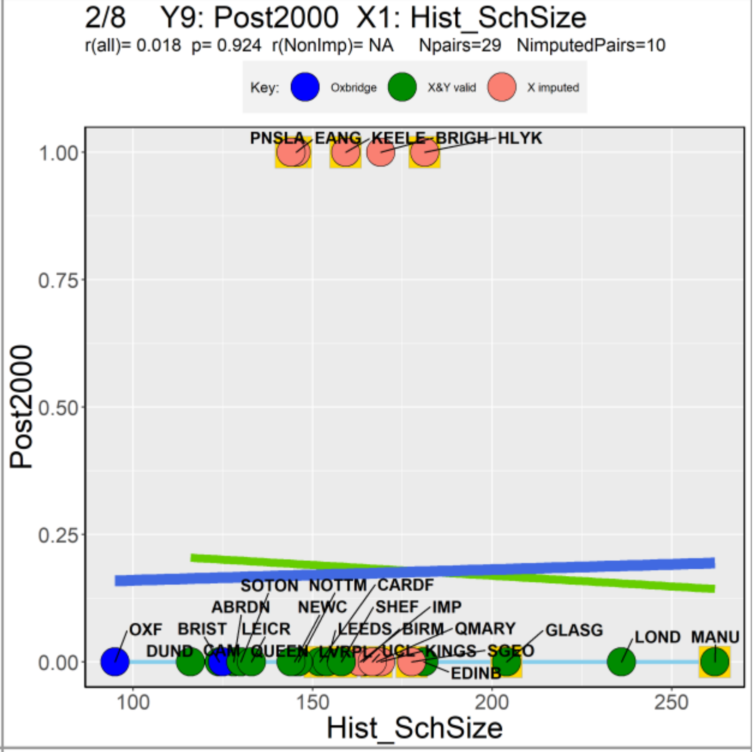
2/8 Y9: Post2000 X1: Hist_SchSize
r(all)= 0.018 p= 0.924 r(NonImp)= NA Npairs=29 NimputedPairs=10
Key: Oxbridge X&Y valid X imputed
Post2000
Hist_SchSize

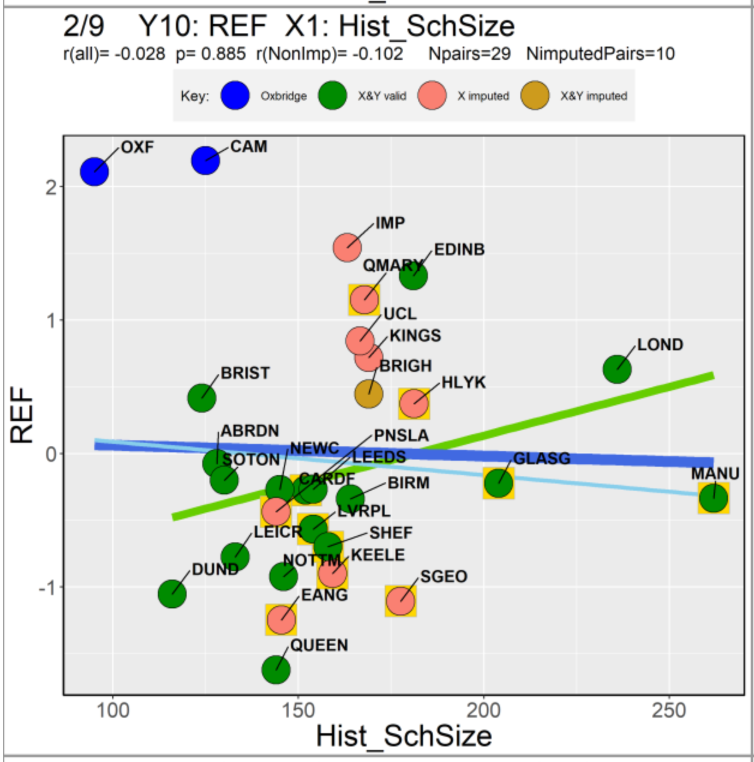
2/9 Y10: REF X1: Hist_SchSize
r(all)= -0.028 p= 0.885 r(NonImp)= -0.102 Npairs=29 NimputedPairs=10
Key: Oxbridge X&Y valid X imputed X&Y imputed
REF
Hist_SchSize

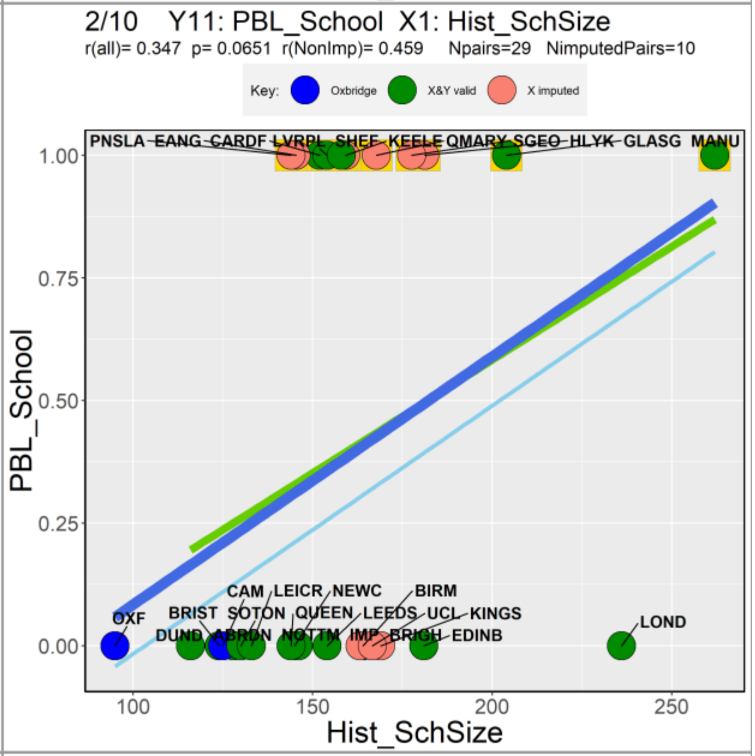
2/10 Y11: PBL_School X1: Hist_SchSize
r(all)= 0.347 p= 0.0651 r(NonImp)= 0.459 Npairs=29 NimputedPairs=10
Key: Oxbridge X&Y valid X imputed
PBL_School
Hist_SchSize

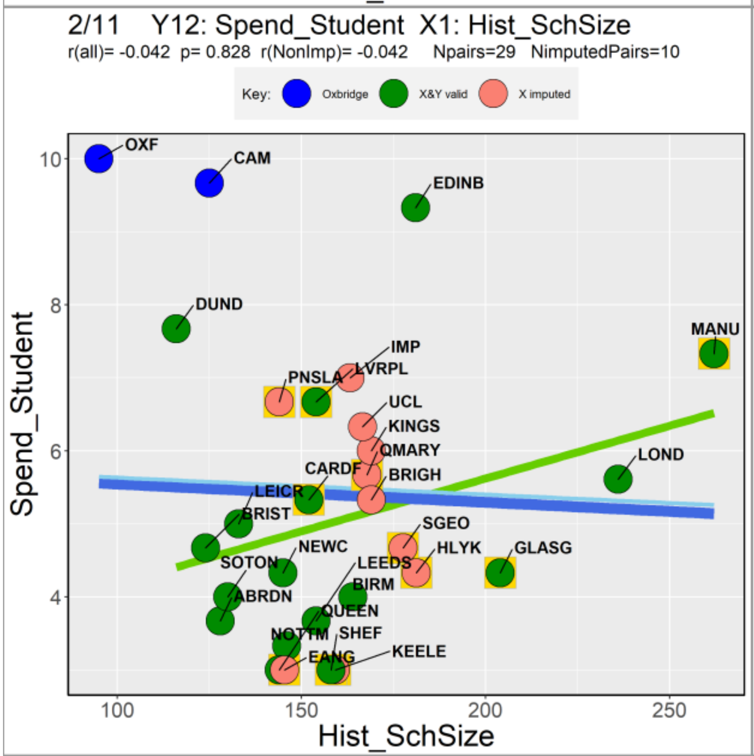
2/11 Y12: Spend_Student X1: Hist_SchSize
r(all)= -0.042 p= 0.828 r(NonImp)= -0.042 Npairs=29 NimputedPairs=10
Key: Oxbridge X&Y valid X imputed
Spend_Student
Hist_SchSize

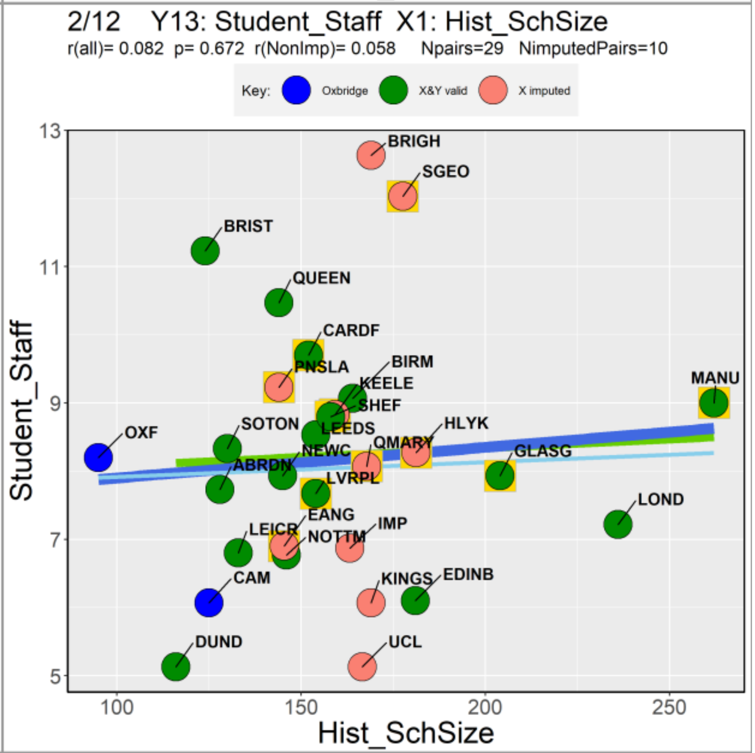
2/12 Y13: Student_Staff X1: Hist_SchSize
r(all)= 0.082 p= 0.672 r(NonImp)= 0.058 Npairs=29 NimputedPairs=10
Key: Oxbridge X&Y valid X imputed
Student_Staff
Hist_SchSize

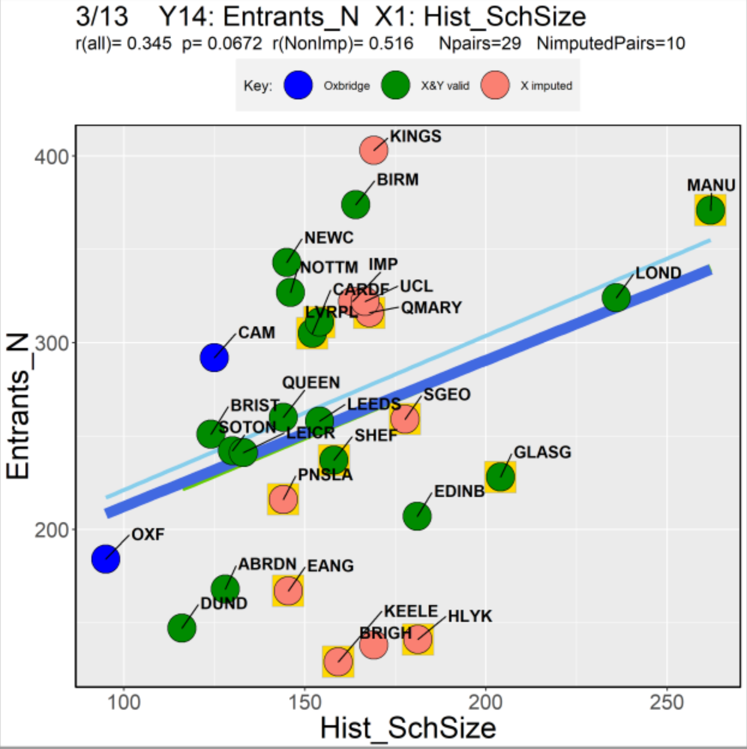
3/13 Y14: Entrants_N X1: Hist_SchSize
r(all)= 0.345 p= 0.0672 r(NonImp)= 0.516 Npairs=29 NimputedPairs=10
Key: Oxbridge X&Y valid X imputed
Entrants_N
Hist_SchSize

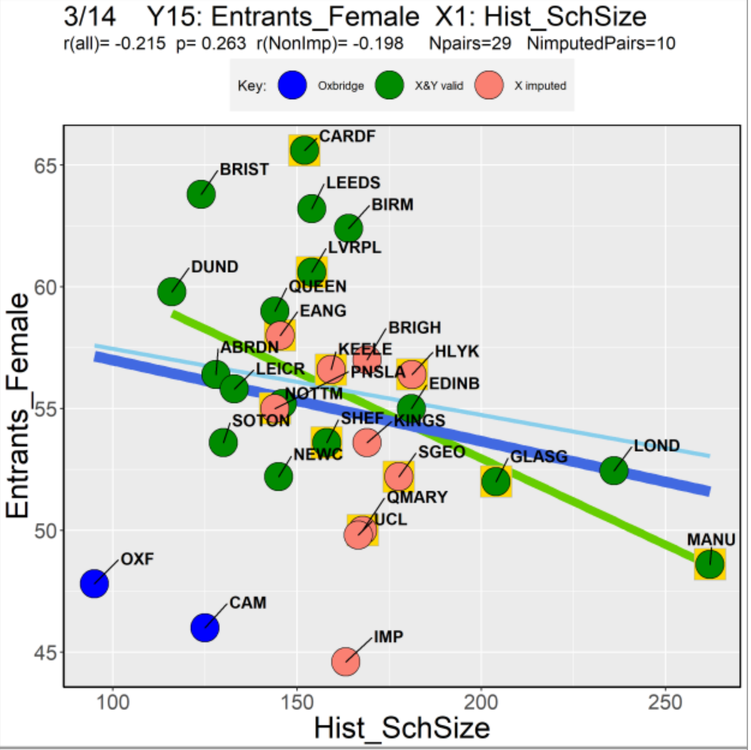
3/14 Y15: Entrants_Female X1: Hist_SchSize
r(all)= -0.215 p= 0.263 r(NonImp)= -0.198 Npairs=29 NimputedPairs=10
Key: Oxbridge X&Y valid X imputed
Entrants_Female
Hist_SchSize

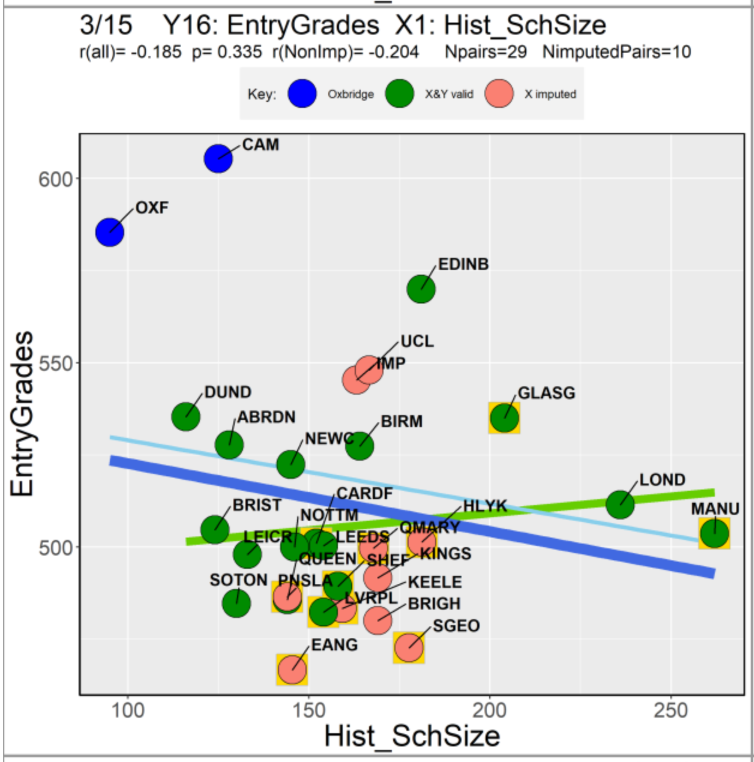
3/15 Y16: EntryGrades X1: Hist_SchSize
r(all)= -0.185 p= 0.335 r(NonImp)= -0.204 Npairs=29 NimputedPairs=10
Key: Oxbridge X&Y valid X imputed
EntryGrades
Hist_SchSize

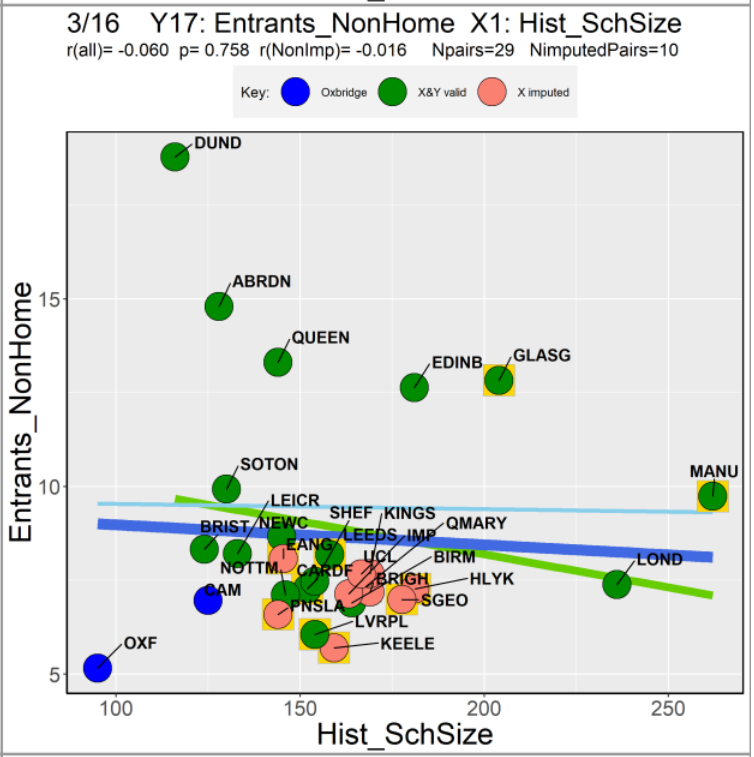
3/16 Y17: Entrants_NonHome X1: Hist_SchSize
r(all)= -0.060 p= 0.758 r(NonImp)= -0.016 Npairs=29 NimputedPairs=10
Key: Oxbridge X&Y valid X imputed
Entrants_NonHome
Hist_SchSize

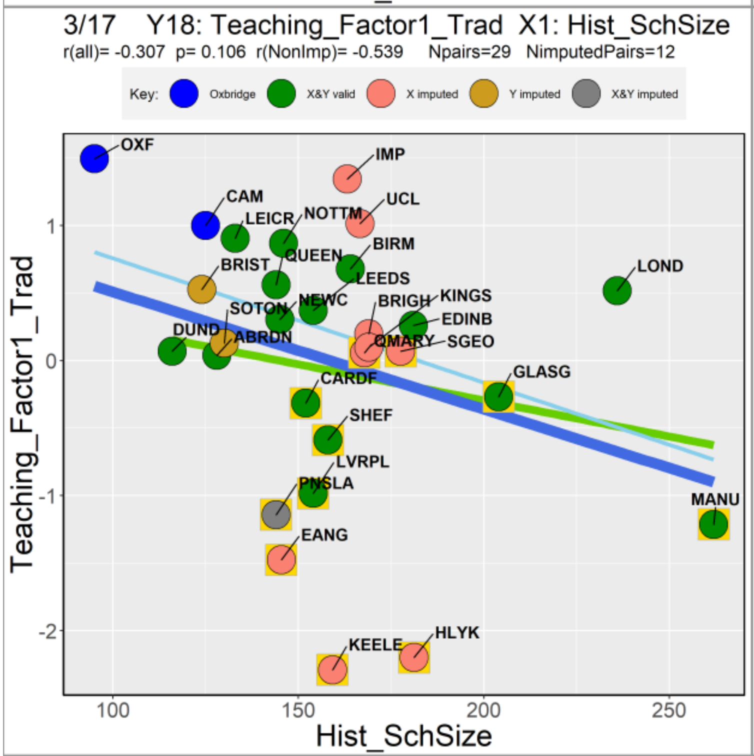
3/17 Y18: Teaching_Factor1_Trad X1: Hist_SchSize
r(all)= -0.307 p= 0.106 r(NonImp)= -0.539 Npairs=29 NimputedPairs=12
Key: Oxbridge X&Y valid X imputed Y imputed X&Y imputed
Teaching_Factor1_Trad
Hist_SchSize

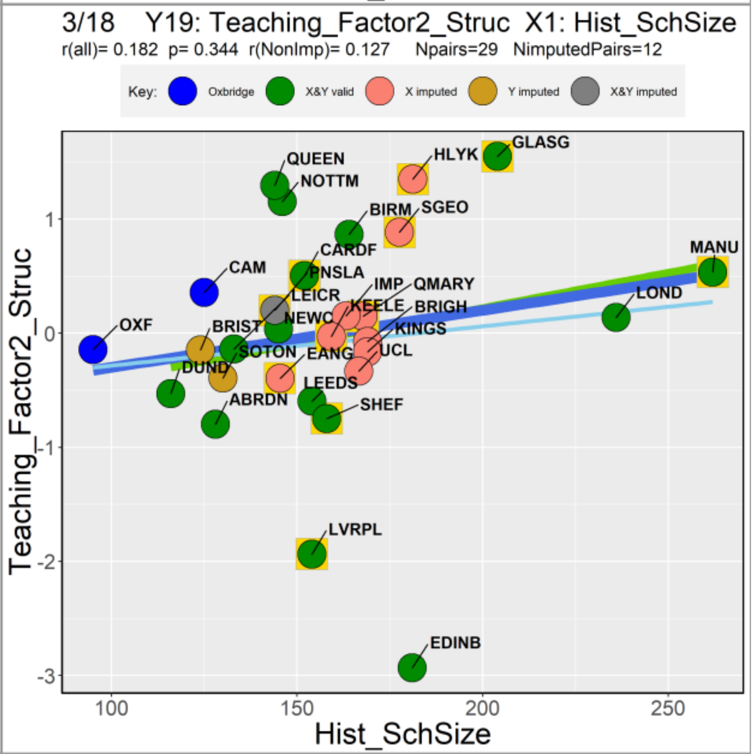
3/18 Y19: Teaching_Factor2_Struc X1: Hist_SchSize
r(all)= 0.182 p= 0.344 r(NonImp)= 0.127 Npairs=29 NimputedPairs=12
Key: Oxbridge X&Y valid X imputed Y imputed X&Y imputed
Teaching_Factor2_Struc
Hist_SchSize

4/19 Y20: Teach_GP X1: Hist_SchSize
r(all)= 0.144 p= 0.457 r(NonImp)= 0.225 Npairs=29 NimputedPairs=12

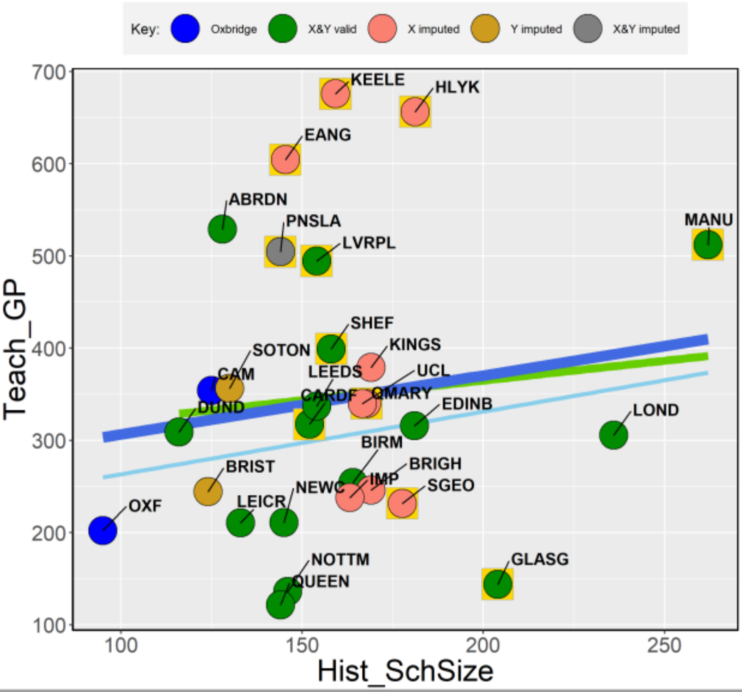

4/20 Y21: Teach_Psyc X1: Hist_SchSize
r(all)= -0.305 p= 0.108 r(NonImp)= -0.269 Npairs=29 NimputedPairs=12

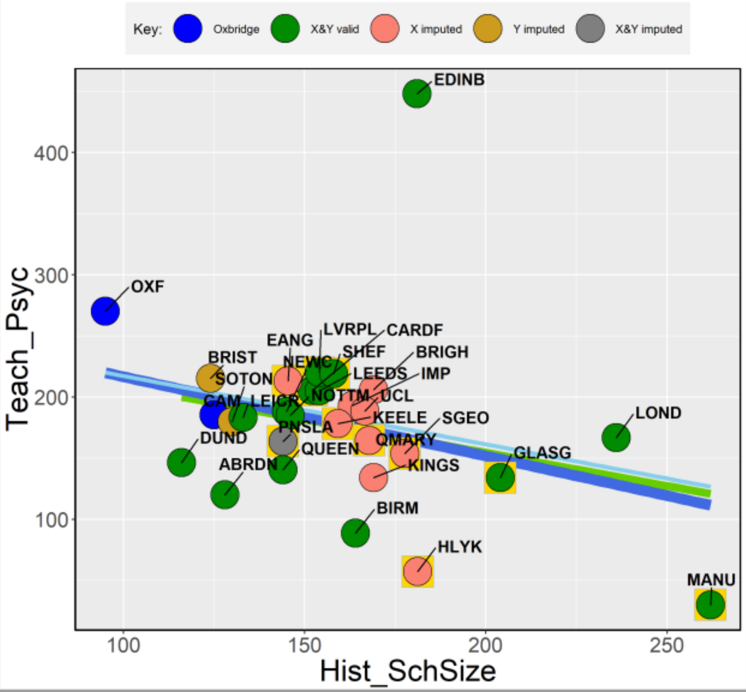

4/21 Y22: Teach_Anaes X1: Hist_SchSize
r(all)= -0.231 p= 0.228 r(NonImp)= -0.349 Npairs=29 NimputedPairs=12

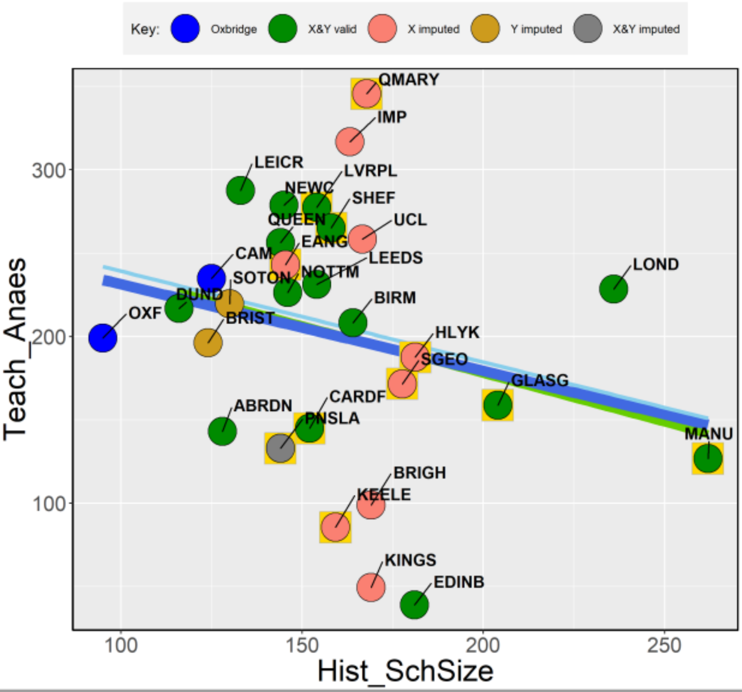

4/22 Y23: Teach_OG X1: Hist_SchSize
r(all)= 0.083 p= 0.668 r(NonImp)= 0.123 Npairs=29 NimputedPairs=12

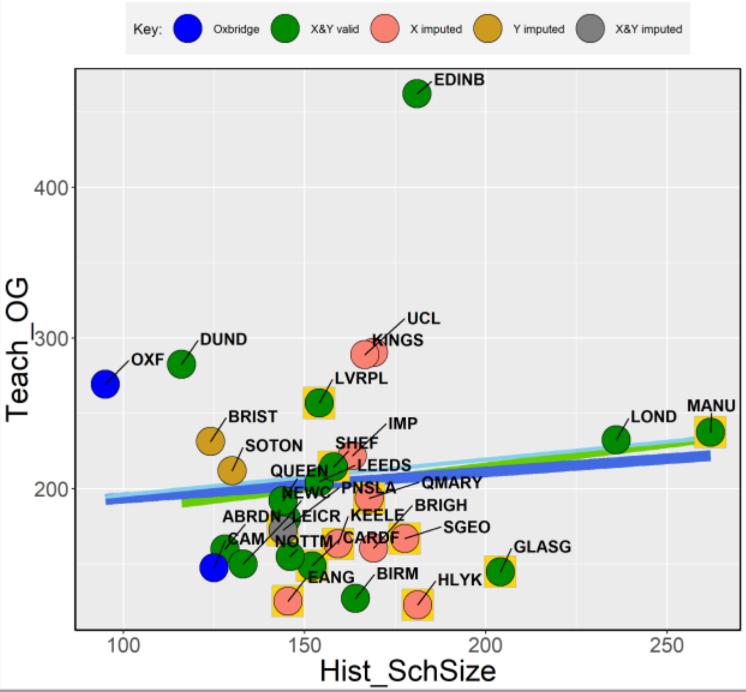

4/23 Y24: Teach_IntMed X1: Hist_SchSize
r(all)= -0.085 p= 0.661 r(NonImp)= -0.116 Npairs=29 NimputedPairs=12

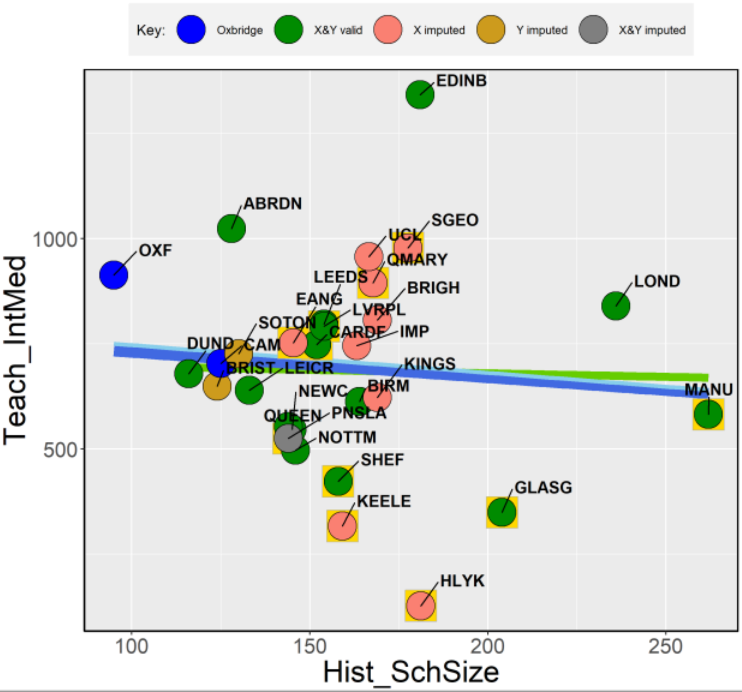

4/24 Y25: Teach_Surgery X1: Hist_SchSize
r(all)= -0.446 p= 0.0152 r(NonImp)= -0.600 Npairs=29 NimputedPairs=12

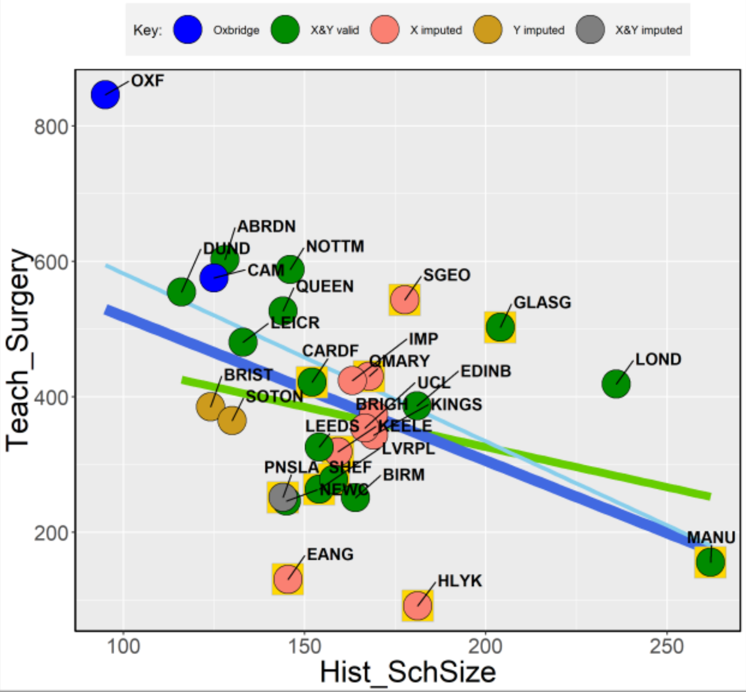

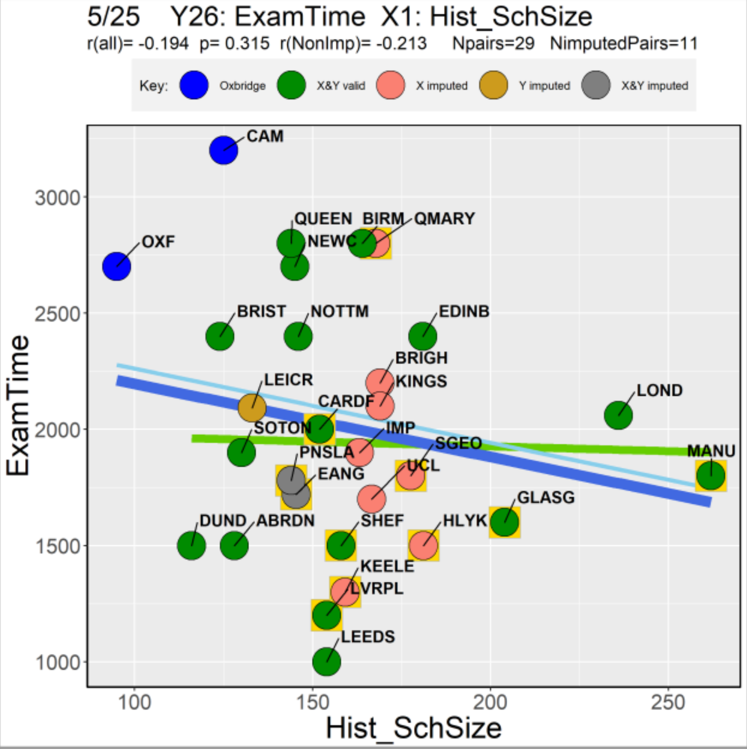
5/25 Y26: ExamTime X1: Hist_SchSize
r(all)= -0.194 p= 0.315 r(NonImp)= -0.213 Npairs=29 NimputedPairs=11
Key: Oxbridge X&Y valid X imputed Y imputed X&Y imputed
ExamTime
Hist_SchSize
CAM
OXF
QUEEN
BIRM
QMARY
NEWC
BRIST
NOTTM
EDINB
BRIGH
KINGS
LEICR
CARDF
LOND
SOTON
IMP
SGEO
PNSLA
MANU
UCL
EANG
GLASG
DUND
ABRDN
SHEF
HLYK
KEELE
LVRPL
LEEDS
3000
2500
2000
1500
1000
100
150
200
250

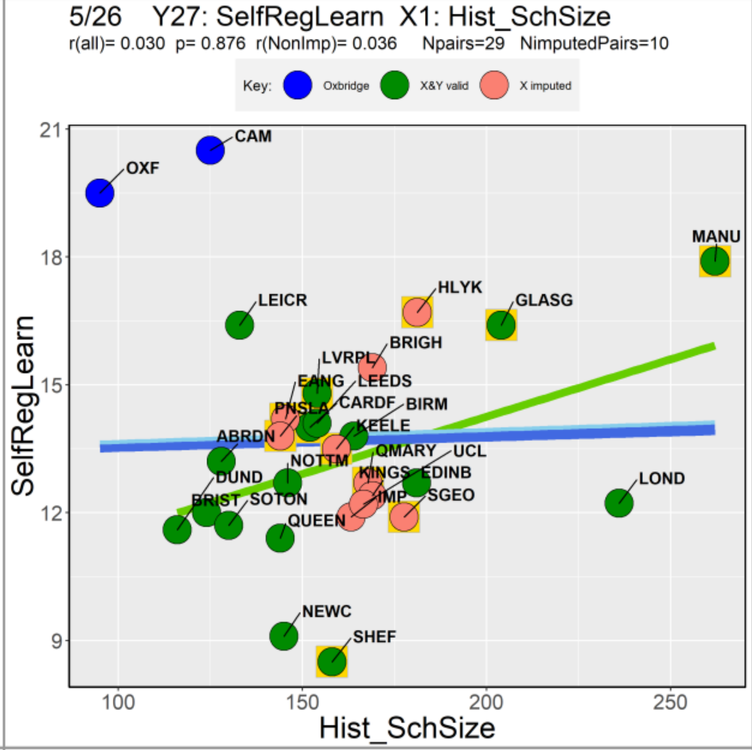
5/26 Y27: SelfRegLearn X1: Hist_SchSize
r(all)= 0.030 p= 0.876 r(NonImp)= 0.036 Npairs=29 NimputedPairs=10
Key: Oxbridge X&Y valid X imputed
SelfRegLearn
Hist_SchSize
CAM
OXF
MANU
HLYK
GLASG
LEICR
BRIGH
LVRPL
EANG
LEEDS
PNSLA
CARDF
BIRM
KEELE
ABRDN
QMARY
UCL
NOTTM
KINGS
EDINB
DUND
LOND
BRIST
SOTON
IMP
SGEO
QUEEN
NEWC
SHEF
21
18
15
12
9
100
150
200
250

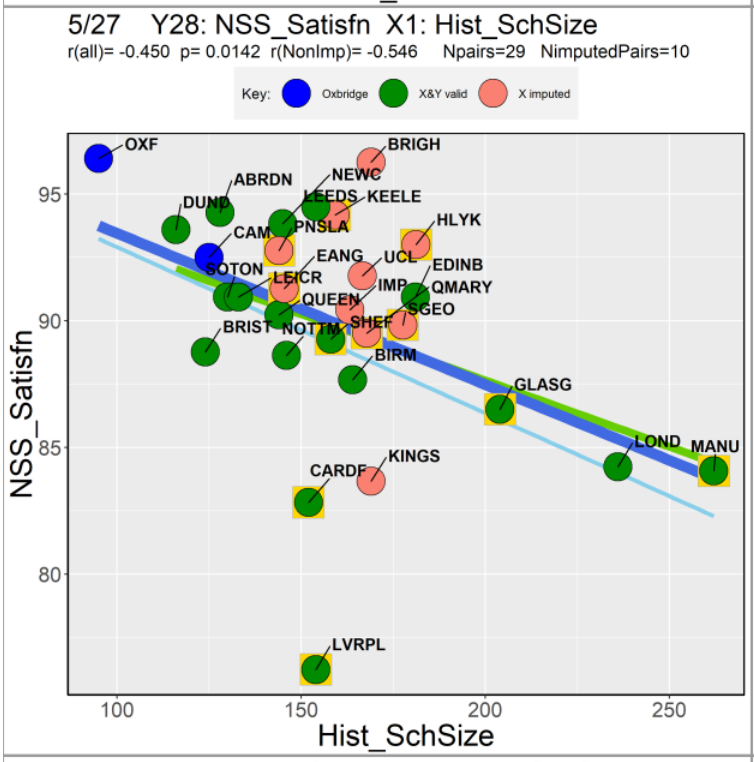
5/27 Y28: NSS_Satisfn X1: Hist_SchSize
r(all)= -0.450 p= 0.0142 r(NonImp)= -0.546 Npairs=29 NimputedPairs=10
Key: Oxbridge X&Y valid X imputed
NSS_Satisfn
Hist_SchSize
OXF
BRIGH
ABRDN
NEWC
DUND
LEEDS
KEELE
HLYK
CAM
PNSLA
EANG
UCL
EDINB
SOTON
LEICR
IMP
QMARY
QUEEN
SGEO
BRIST
NOTTM
SHEF
BIRM
GLASG
LOND
MANU
KINGS
CARDF
LVRPL
95
90
85
80
100
150
200
250

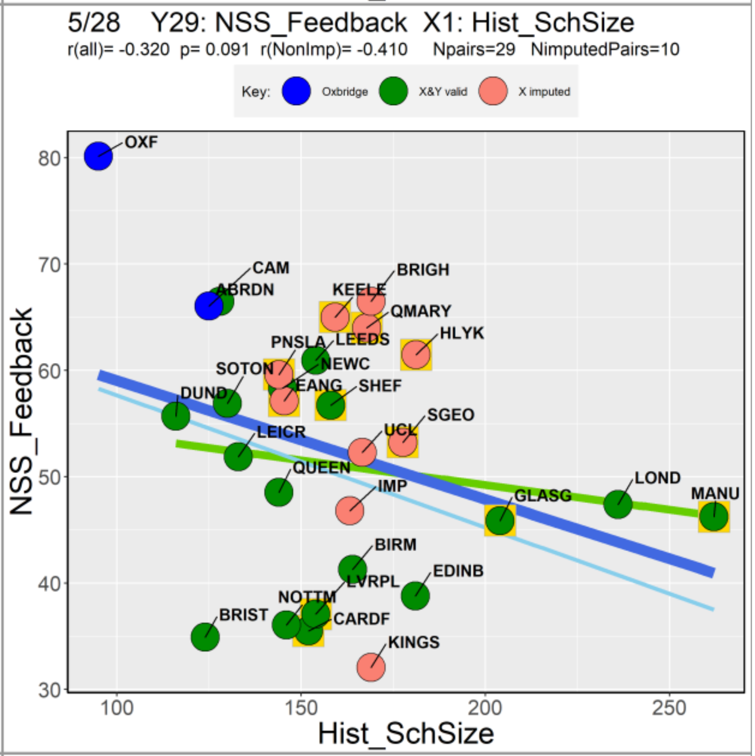
5/28 Y29: NSS_Feedback X1: Hist_SchSize
r(all)= -0.320 p= 0.091 r(NonImp)= -0.410 Npairs=29 NimputedPairs=10
Key: Oxbridge X&Y valid X imputed
NSS_Feedback
Hist_SchSize
OXF
CAM
BRIGH
ABRDN
KEELE
QMARY
HLYK
PNSLA
LEEDS
SOTON
NEWC
DUND
EANG
SHEF
SGEO
UCL
LEICR
QUEEN
IMP
LOND
GLASG
MANU
BIRM
EDINB
NOTTM
LVRPL
BRIST
CARDF
KINGS
80
70
60
50
40
30
100
150
200
250

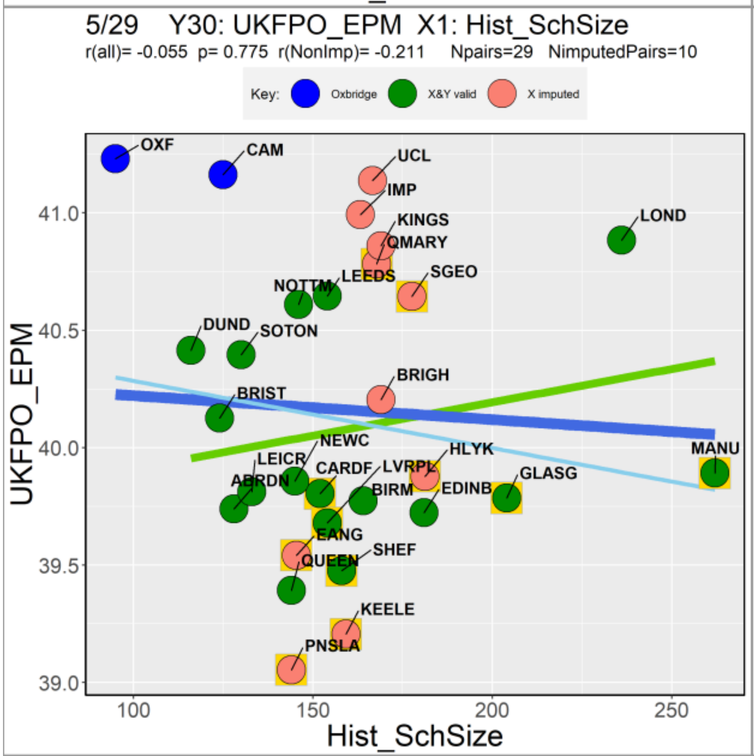
5/29 Y30: UKFPO_EPM X1: Hist_SchSize
r(all)= -0.055 p= 0.775 r(NonImp)= -0.211 Npairs=29 NimputedPairs=10
Key: Oxbridge X&Y valid X imputed
UKFPO_EPM
Hist_SchSize
OXF
CAM
UCL
IMP
LOND
KINGS
QMARY
SGEO
NOTTM
LEEDS
DUND
SOTON
BRIGH
BRIST
NEWC
MANU
HLYK
LEICR
CARDF
LVRPL
ABRDN
BIRM
EDINB
GLASG
EANG
QUEEN
SHEF
KEELE
PNSLA
41.0
40.5
40.0
39.5
39.0
100
150
200
250

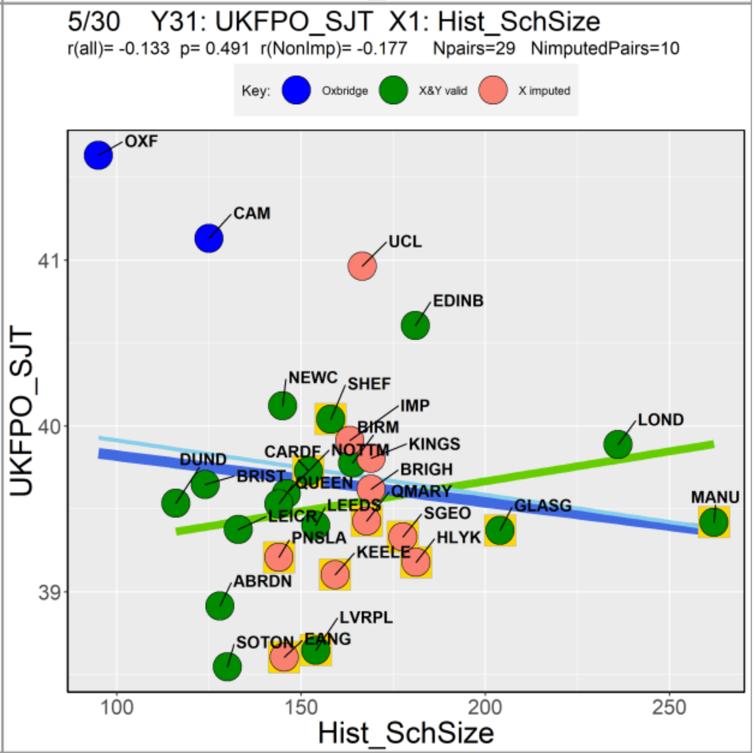
5/30 Y31: UKFPO_SJT X1: Hist_SchSize
r(all)= -0.133 p= 0.491 r(NonImp)= -0.177 Npairs=29 NimputedPairs=10
Key: Oxbridge X&Y valid X imputed
UKFPO_SJT
Hist_SchSize
OXF
CAM
UCL
EDINB
NEWC
SHEF
IMP
BIRM
LOND
CARDF
NOTTM
KINGS
DUND
BRIST
QUEEN
BRIGH
QMARY
MANU
LEEDS
GLASG
LEICR
SGEO
PNSLA
HLYK
KEELE
ABRDN
LVRPL
SOTON
EANG
41
40
39
100
150
200
250

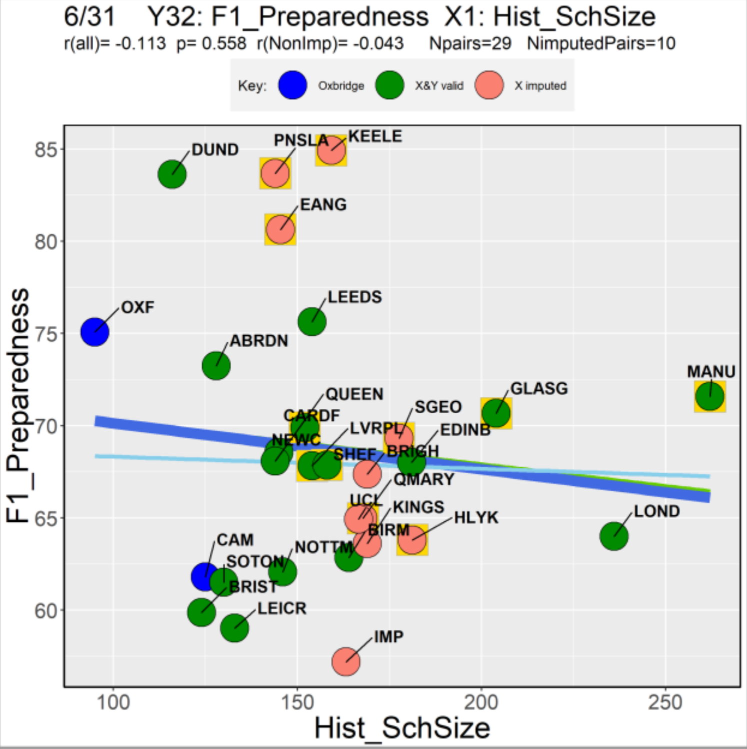
6/31 Y32: F1_Preparedness X1: Hist_SchSize
r(all)= -0.113 p= 0.558 r(NonImp)= -0.043 Npairs=29 NimputedPairs=10
Key: Oxbridge X&Y valid X imputed
F1_Preparedness
Hist_SchSize

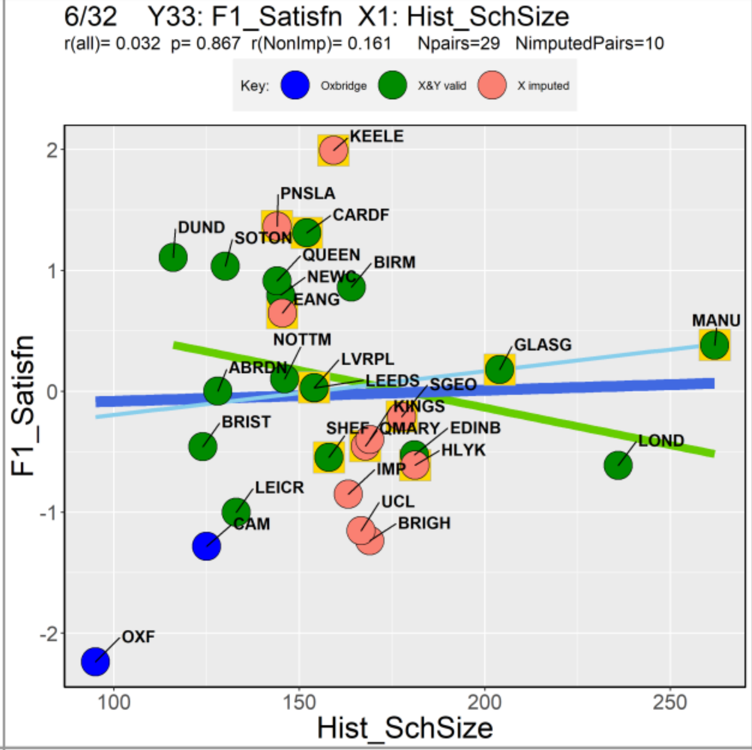
6/32 Y33: F1_Satisfn X1: Hist_SchSize
r(all)= 0.032 p= 0.867 r(NonImp)= 0.161 Npairs=29 NimputedPairs=10
Key: Oxbridge X&Y valid X imputed
F1_Satisfn
Hist_SchSize

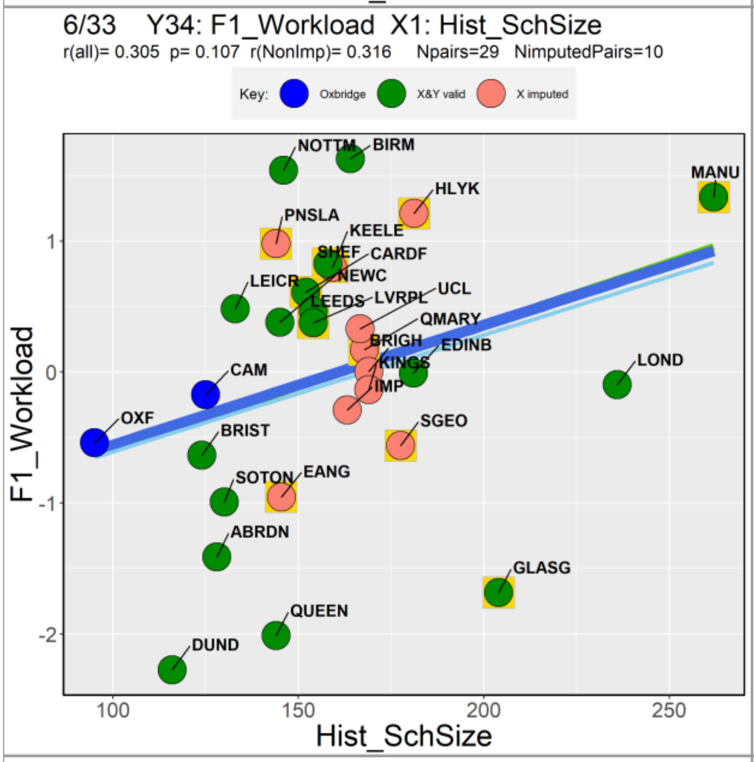
6/33 Y34: F1_Workload X1: Hist_SchSize
r(all)= 0.305 p= 0.107 r(NonImp)= 0.316 Npairs=29 NimputedPairs=10
Key: Oxbridge X&Y valid X imputed
F1_Workload
Hist_SchSize

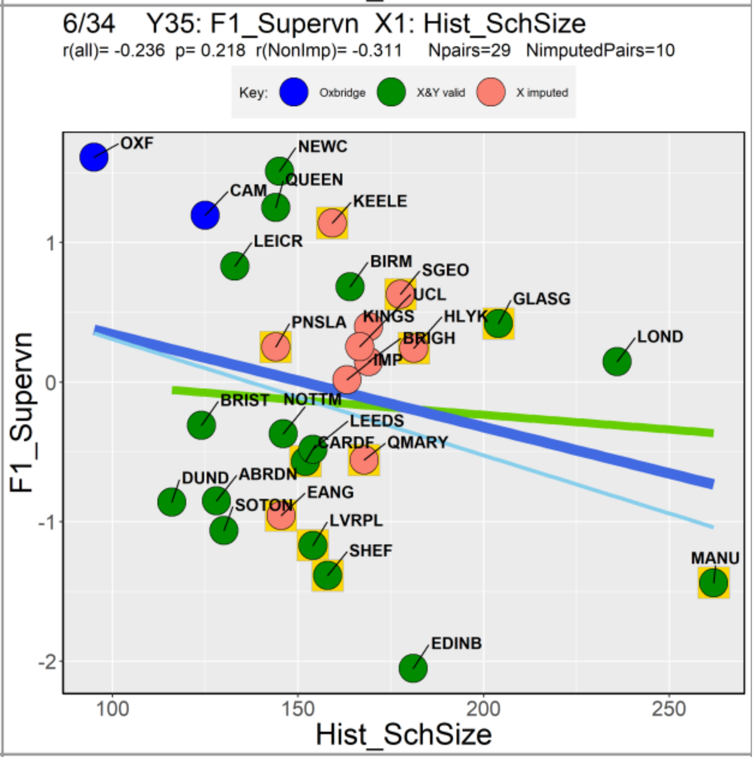
6/34 Y35: F1_Supervn X1: Hist_SchSize
r(all)= -0.236 p= 0.218 r(NonImp)= -0.311 Npairs=29 NimputedPairs=10
Key: Oxbridge X&Y valid X imputed
F1_Supervn
Hist_SchSize

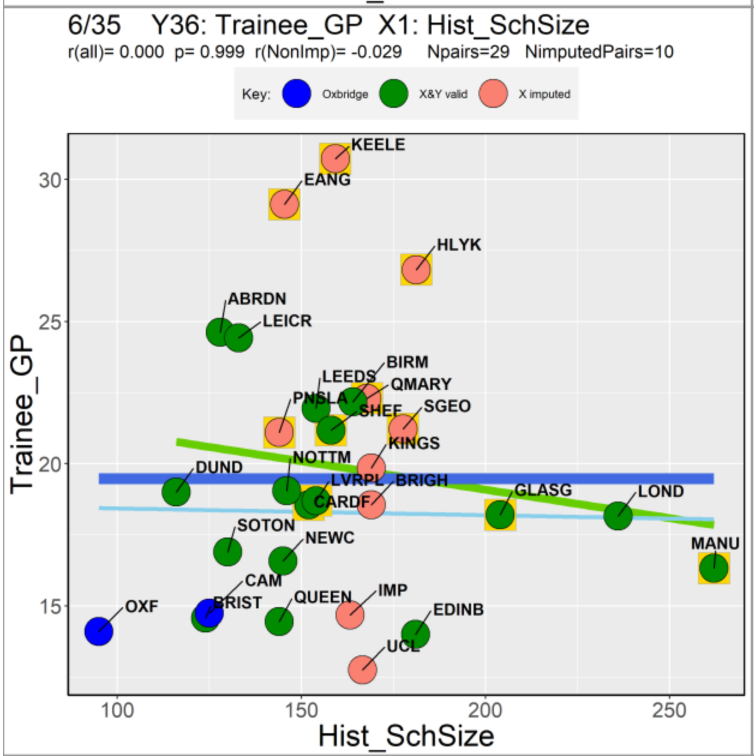
6/35 Y36: Trainee_GP X1: Hist_SchSize
r(all)= 0.000 p= 0.999 r(NonImp)= -0.029 Npairs=29 NimputedPairs=10
Key: Oxbridge X&Y valid X imputed
Trainee_GP
Hist_SchSize

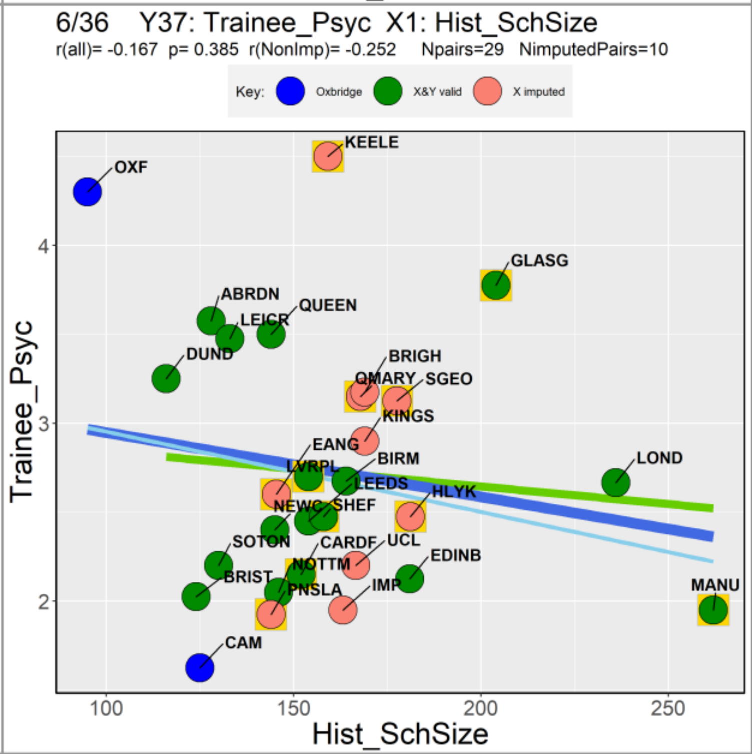
6/36 Y37: Trainee_Psyc X1: Hist_SchSize
r(all)= -0.167 p= 0.385 r(NonImp)= -0.252 Npairs=29 NimputedPairs=10
Key: Oxbridge X&Y valid X imputed
Trainee_Psyc
Hist_SchSize

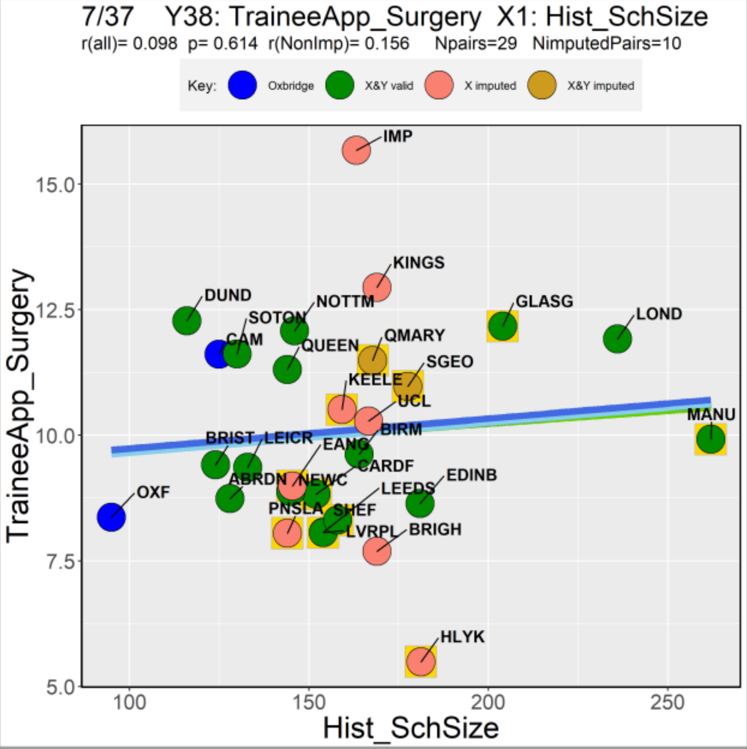
7/37 Y38: TraineeApp_Surgery X1: Hist_SchSize
r(all)= 0.098 p= 0.614 r(NonImp)= 0.156 Npairs=29 NimputedPairs=10
Key: Oxbridge X&Y valid X imputed X&Y imputed
TraineeApp_Surgery
Hist_SchSize
IMP KINGS DUND NOTTM SOTON CAM QUEEN QMARY SGEO KEELE UCL BIRM GLASG LOND MANU BRIST LEICR EANG CARDF ABRDN NEWC OXF LEEDS EDINB PNSLA SHEF LVRPL BRIGH HLYK

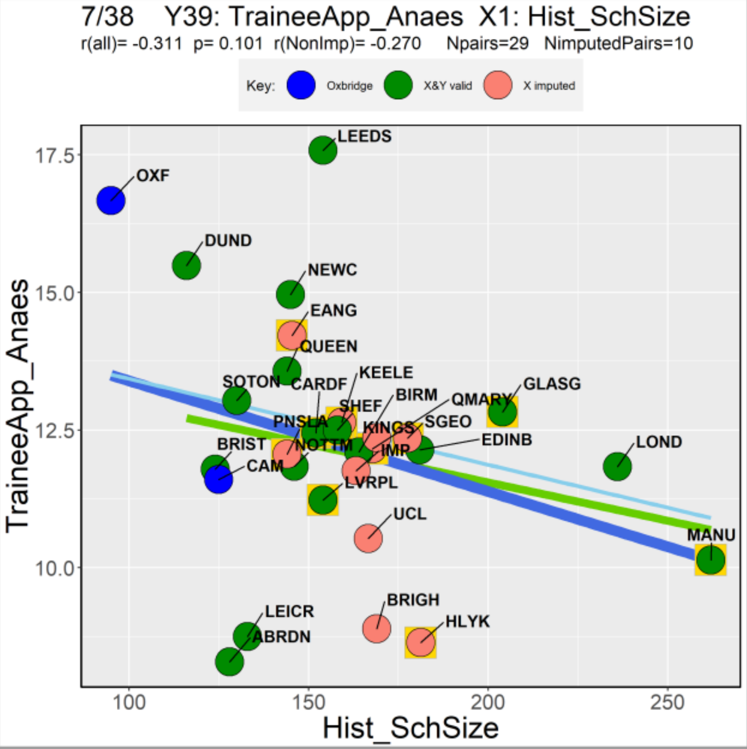
7/38 Y39: TraineeApp_Anaes X1: Hist_SchSize
r(all)= -0.311 p= 0.101 r(NonImp)= -0.270 Npairs=29 NimputedPairs=10
Key: Oxbridge X&Y valid X imputed
TraineeApp_Anaes
Hist_SchSize
LEEDS OXF DUND NEWC EANG QUEEN SOTON CARDF KEELE GLASG SHEF BIRM QMARY PNSLA KINGS SGEO EDINB BRIST NOTTM IMP LOND CAM LVRPL UCL MANU BRIGH LEICR HLYK ABRDN

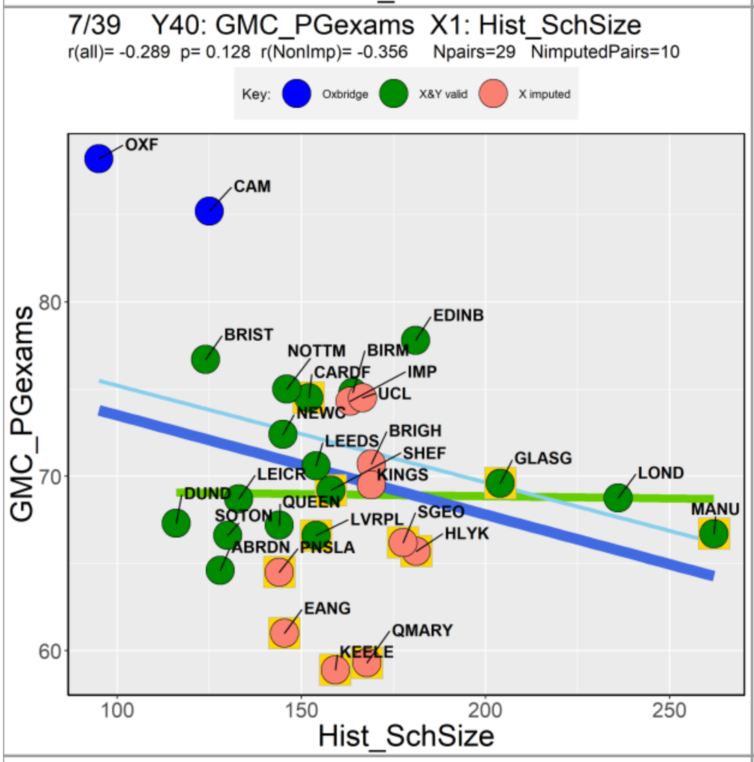
7/39 Y40: GMC_PGexams X1: Hist_SchSize
r(all)= -0.289 p= 0.128 r(NonImp)= -0.356 Npairs=29 NimputedPairs=10
Key: Oxbridge X&Y valid X imputed
GMC_PGexams
Hist_SchSize
OXF CAM EDINB BRIST NOTTM BIRM CARDF IMP UCL NEWC BRIGH LEEDS SHEF GLASG LEICR KINGS LOND DUND QUEEN SOTON LVRPL SGEO MANU HLYK ABRDN PNSLA EANG QMARY KEELE

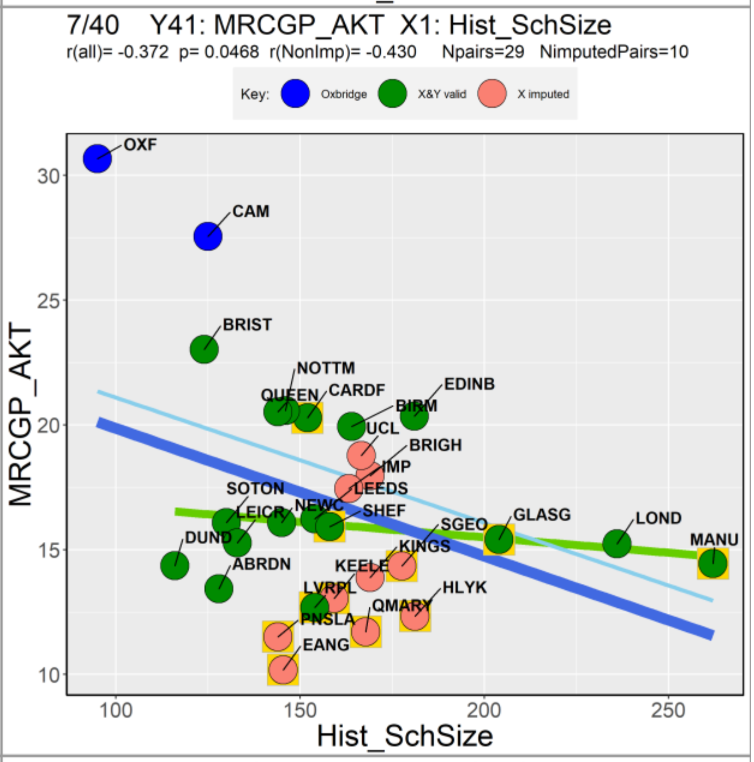
7/40 Y41: MRCGP_AKT X1: Hist_SchSize
r(all)= -0.372 p= 0.0468 r(NonImp)= -0.430 Npairs=29 NimputedPairs=10
Key: Oxbridge X&Y valid X imputed
MRCGP_AKT
Hist_SchSize
OXF CAM BRIST NOTTM EDINB QUEEN CARDF BIRM UCL BRIGH IMP LEEDS SOTON NEWC SHEF GLASG LEICR SGEO LOND DUND MANU KINGS ABRDN KEELE LVRPL HLYK QMARY PNSLA EANG

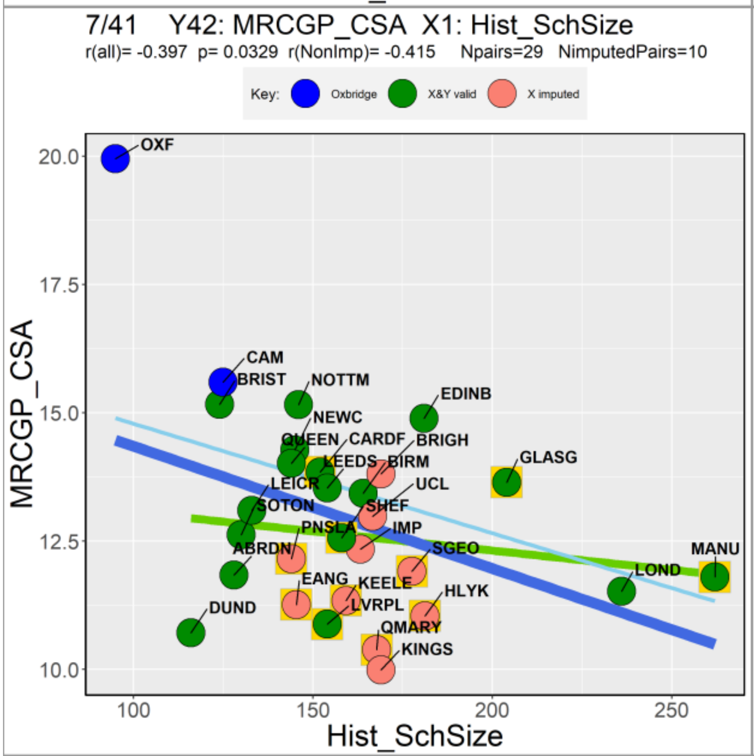
7/41 Y42: MRCGP_CSA X1: Hist_SchSize
r(all)= -0.397 p= 0.0329 r(NonImp)= -0.415 Npairs=29 NimputedPairs=10
Key: Oxbridge X&Y valid X imputed
MRCGP_CSA
Hist_SchSize
OXF CAM BRIST NOTTM EDINB NEWC QUEEN CARDF BRIGH LEEDS BIRM GLASG LEICR UCL SOTON SHEF PNSLA IMP ABRDN SGEO MANU LOND EANG KEELE HLYK DUND LVRPL QMARY KINGS

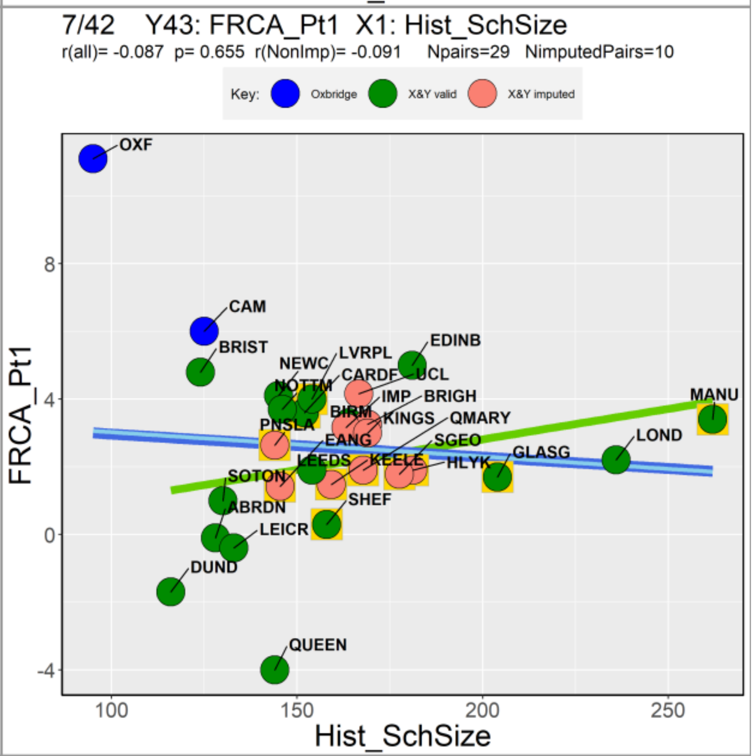
7/42 Y43: FRCA_Pt1 X1: Hist_SchSize
r(all)= -0.087 p= 0.655 r(NonImp)= -0.091 Npairs=29 NimputedPairs=10
Key: Oxbridge X&Y valid X&Y imputed
FRCA_Pt1
Hist_SchSize
OXF CAM EDINB BRIST LVRPL NEWC CARDF UCL NOTTM IMP BRIGH MANU BIRM PNSLA KINGS QMARY EANG SGEO LOND LEEDS KEELE HLYK GLASG SOTON SHEF ABRDN LEICR DUND QUEEN

8/43 Y44: MRCOG_Pt1 X1: Hist_SchSize

r(all)= -0.112 p= 0.563 r(NonImp)= -0.133 Npairs=29 NimputedPairs=10

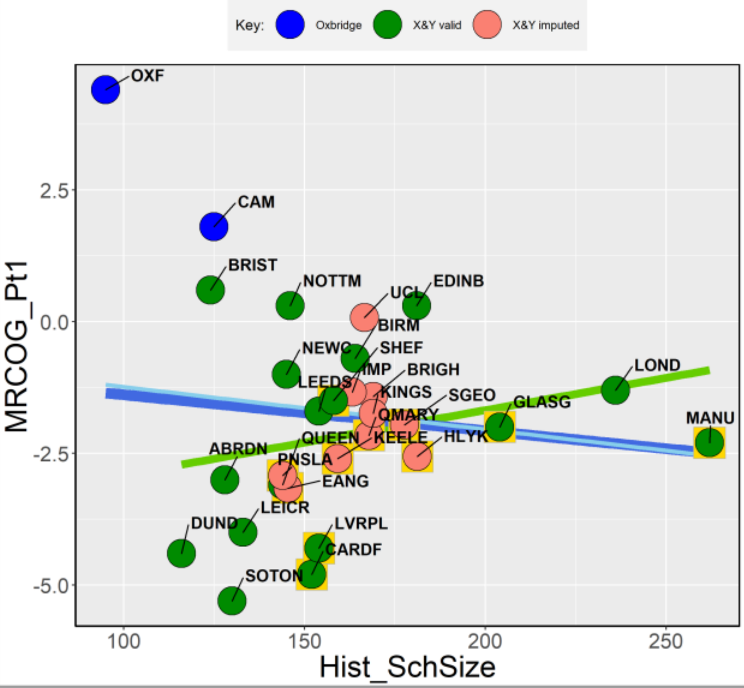

8/44 Y45: MRCOG_Pt2 X1: Hist_SchSize

r(all)= -0.145 p= 0.452 r(NonImp)= -0.144 Npairs=29 NimputedPairs=10

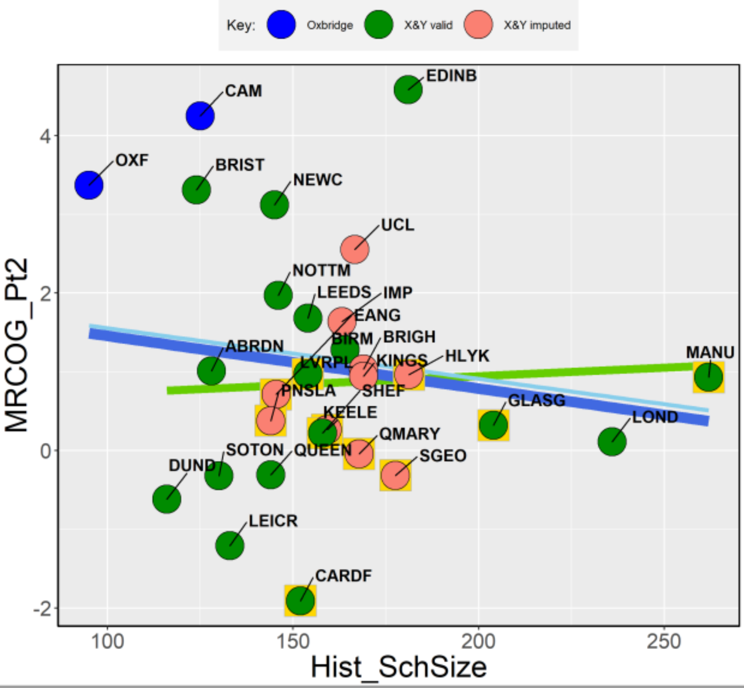

8/45 Y46: MRCP_Pt1 X1: Hist_SchSize

r(all)= -0.211 p= 0.272 r(NonImp)= -0.309 Npairs=29 NimputedPairs=10

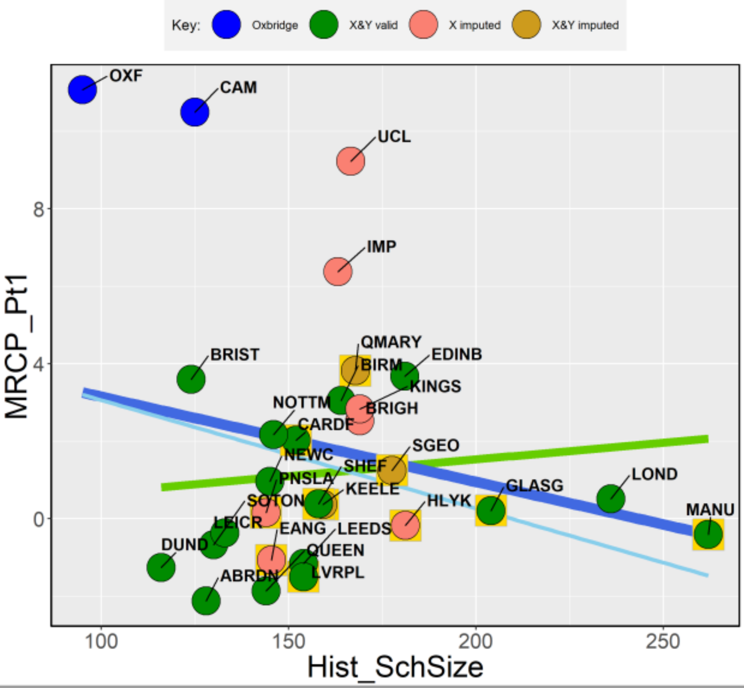

8/46 Y47: MRCP_Pt2 X1: Hist_SchSize

r(all)= -0.333 p= 0.0775 r(NonImp)= -0.415 Npairs=29 NimputedPairs=10

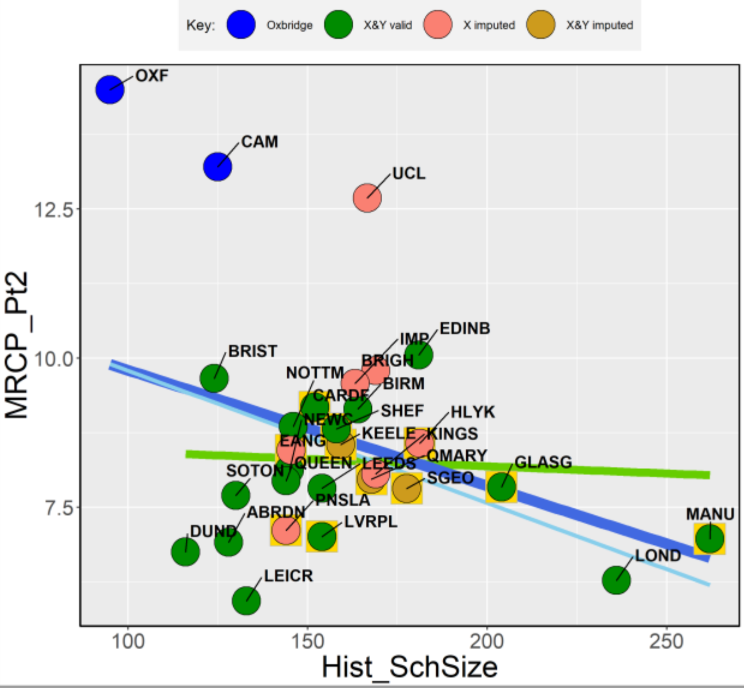

8/47 Y48: MRCP_PACES X1: Hist_SchSize

r(all)= -0.165 p= 0.393 r(NonImp)= -0.214 Npairs=29 NimputedPairs=10

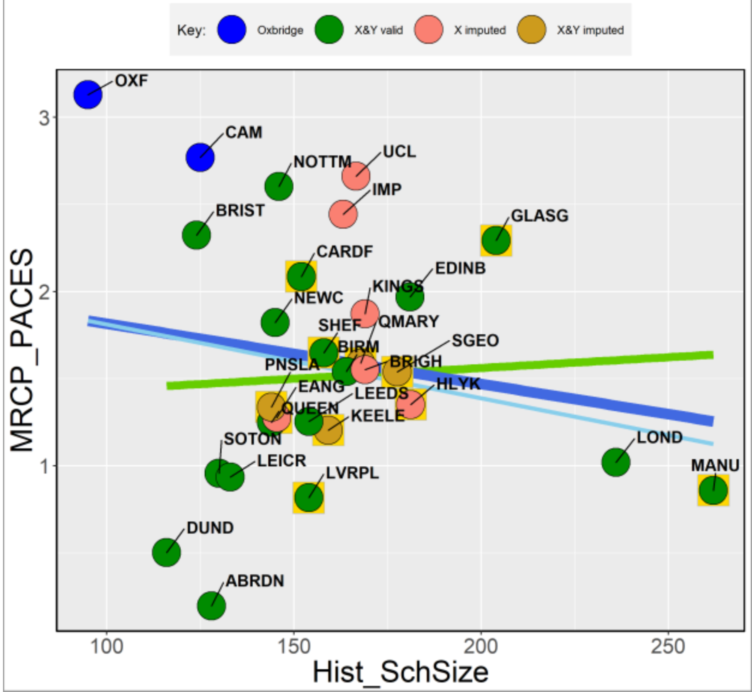

8/48 Y49: GMC_Sanctions X1: Hist_SchSize

r(all)= 0.273 p= 0.153 r(NonImp)= 0.306 Npairs=29 NimputedPairs=10

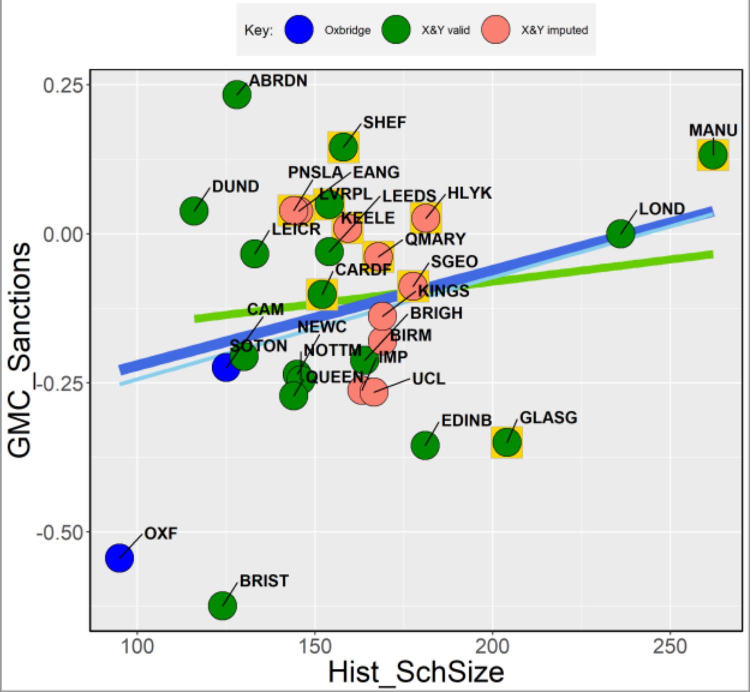

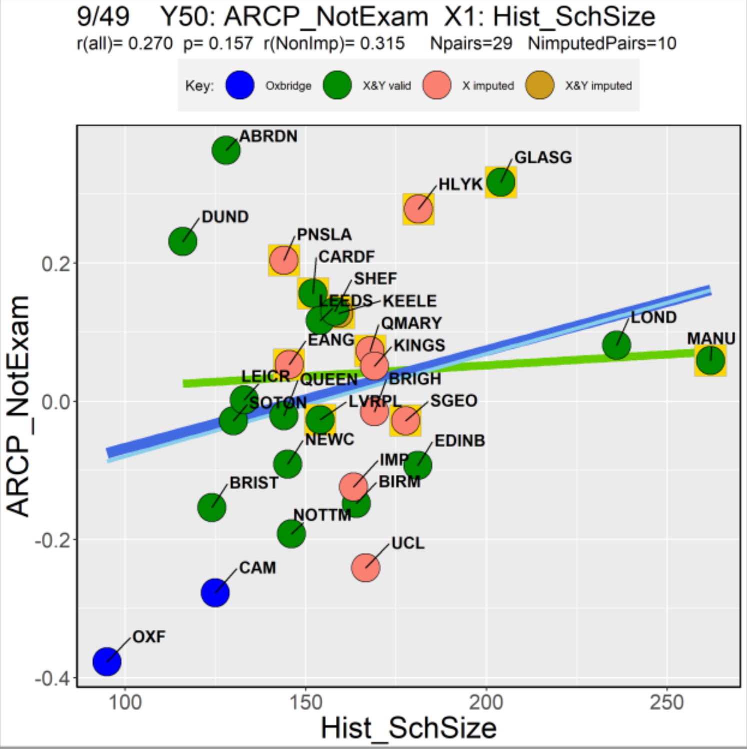
9/49 Y50: ARCP_NotExam X1: Hist_SchSize
r(all)= 0.270 p= 0.157 r(NonImp)= 0.315 Npairs=29 NimputedPairs=10
Key: Oxbridge X&Y valid X imputed X&Y imputed
ARCP_NotExam
Hist_SchSize

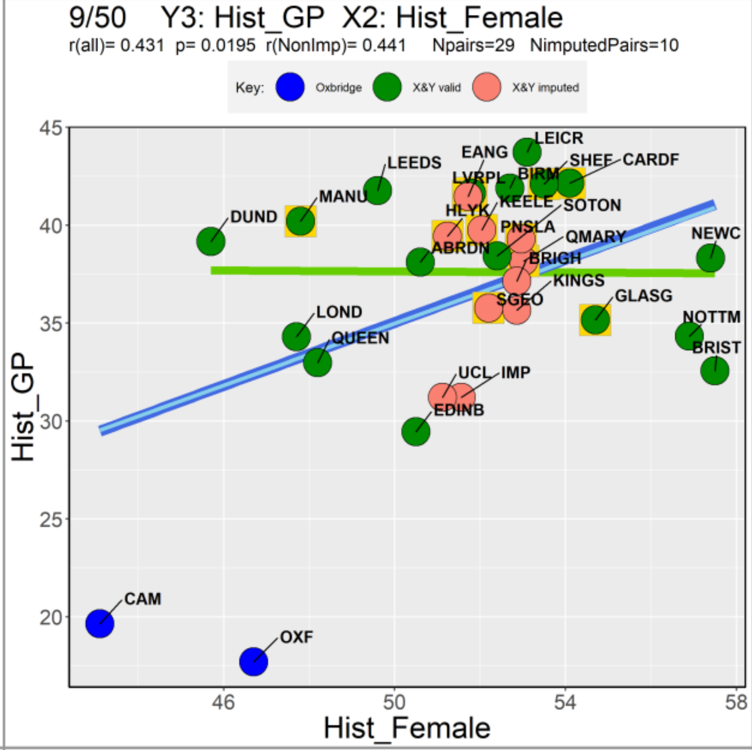
9/50 Y3: Hist_GP X2: Hist_Female
r(all)= 0.431 p= 0.0195 r(NonImp)= 0.441 Npairs=29 NimputedPairs=10
Key: Oxbridge X&Y valid X&Y imputed
Hist_GP
Hist_Female

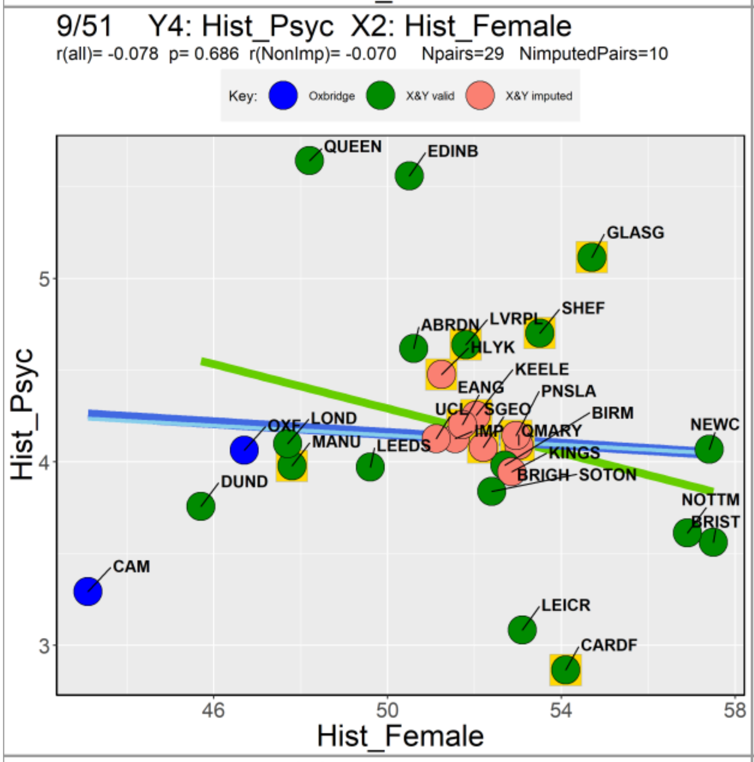
9/51 Y4: Hist_Psyc X2: Hist_Female
r(all)= -0.078 p= 0.686 r(NonImp)= -0.070 Npairs=29 NimputedPairs=10
Key: Oxbridge X&Y valid X&Y imputed
Hist_Psyc
Hist_Female

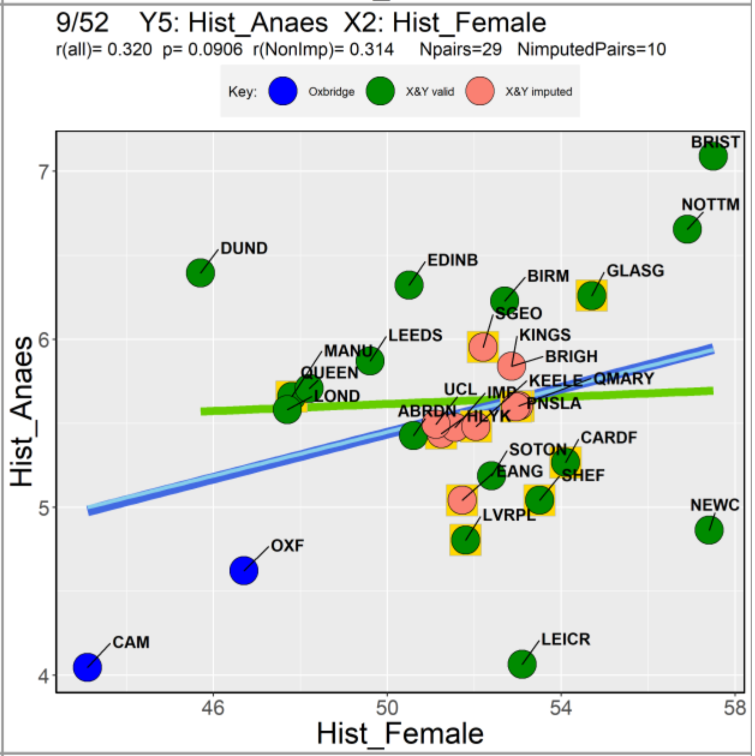
9/52 Y5: Hist_Anaes X2: Hist_Female
r(all)= 0.320 p= 0.0906 r(NonImp)= 0.314 Npairs=29 NimputedPairs=10
Key: Oxbridge X&Y valid X&Y imputed
Hist_Anaes
Hist_Female

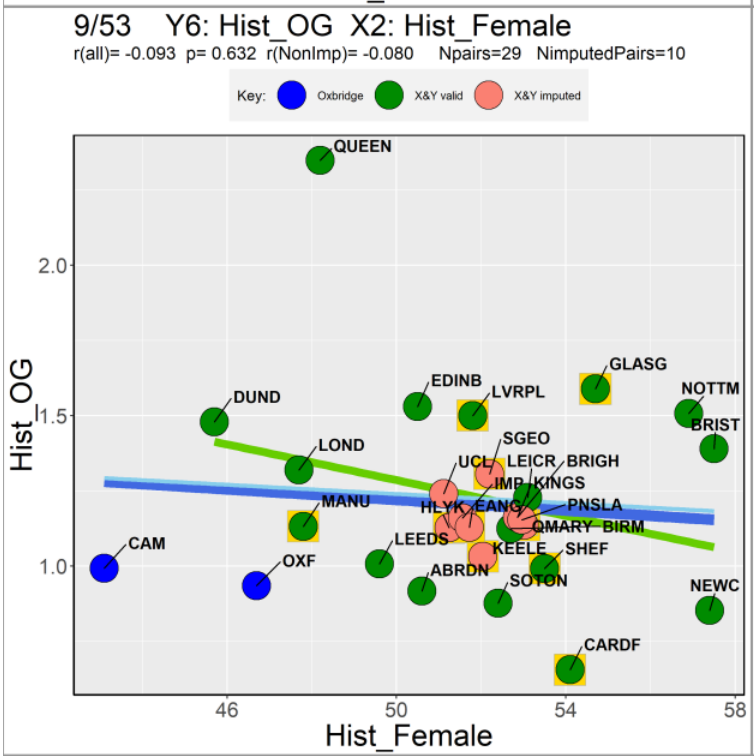
9/53 Y6: Hist_OG X2: Hist_Female
r(all)= -0.093 p= 0.632 r(NonImp)= -0.080 Npairs=29 NimputedPairs=10
Key: Oxbridge X&Y valid X&Y imputed
Hist_OG
Hist_Female

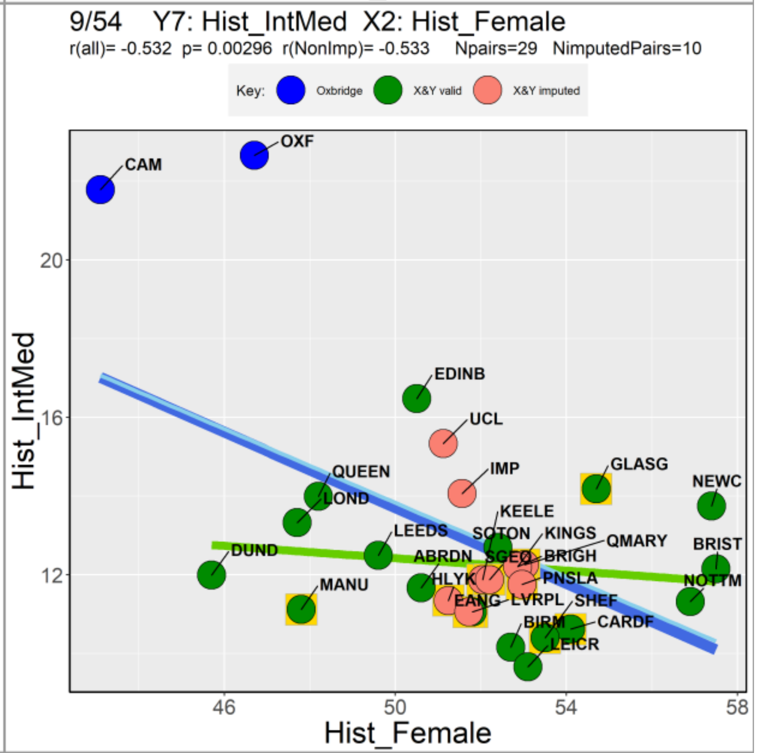
9/54 Y7: Hist_IntMed X2: Hist_Female
r(all)= -0.532 p= 0.00296 r(NonImp)= -0.533 Npairs=29 NimputedPairs=10
Key: Oxbridge X&Y valid X&Y imputed
Hist_IntMed
Hist_Female

10/55 Y8: Hist_Surgery X2: Hist_Female

r(all)= -0.423 p= 0.0223 r(NonImp)= -0.429 Npairs=29 NimputedPairs=10

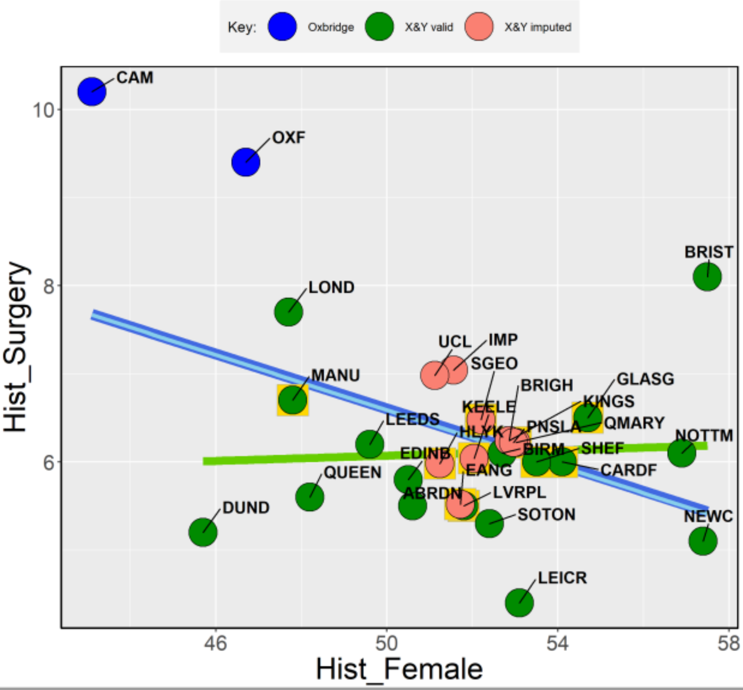

10/56 Y9: Post2000 X2: Hist_Female

r(all)= 0.083 p= 0.668 r(NonImp)= NA Npairs=29 NimputedPairs=10

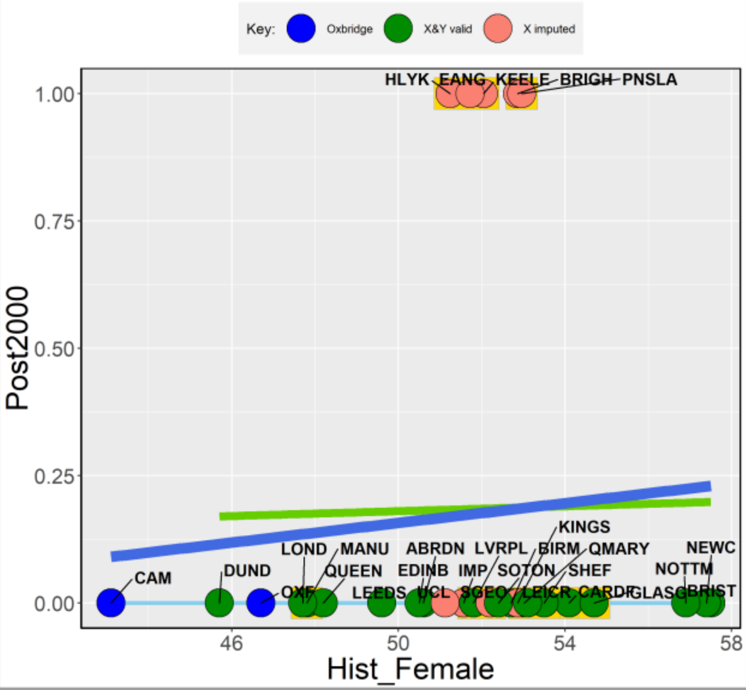

10/57 Y10: REF X2: Hist_Female

r(all)= -0.297 p= 0.118 r(NonImp)= -0.384 Npairs=29 NimputedPairs=10

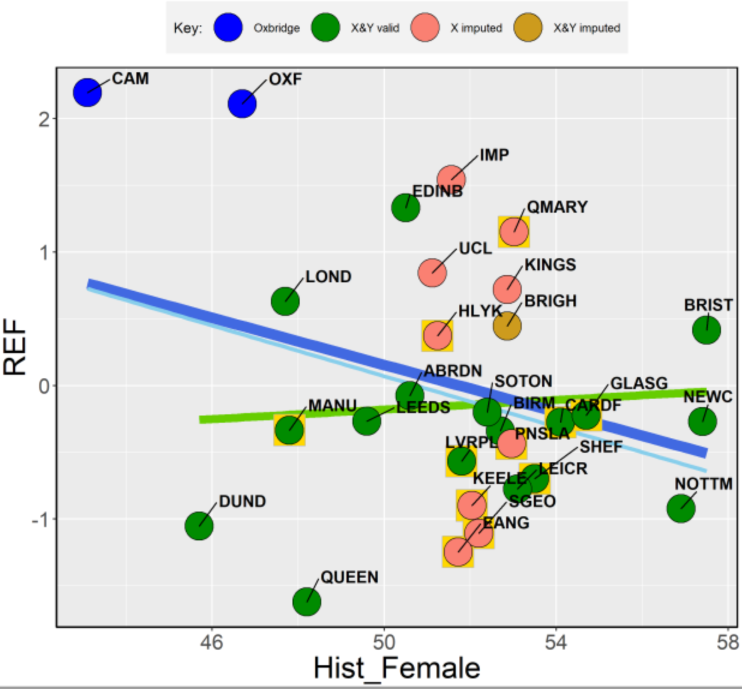

10/58 Y11: PBL_School X2: Hist_Female

r(all)= 0.170 p= 0.377 r(NonImp)= 0.169 Npairs=29 NimputedPairs=10

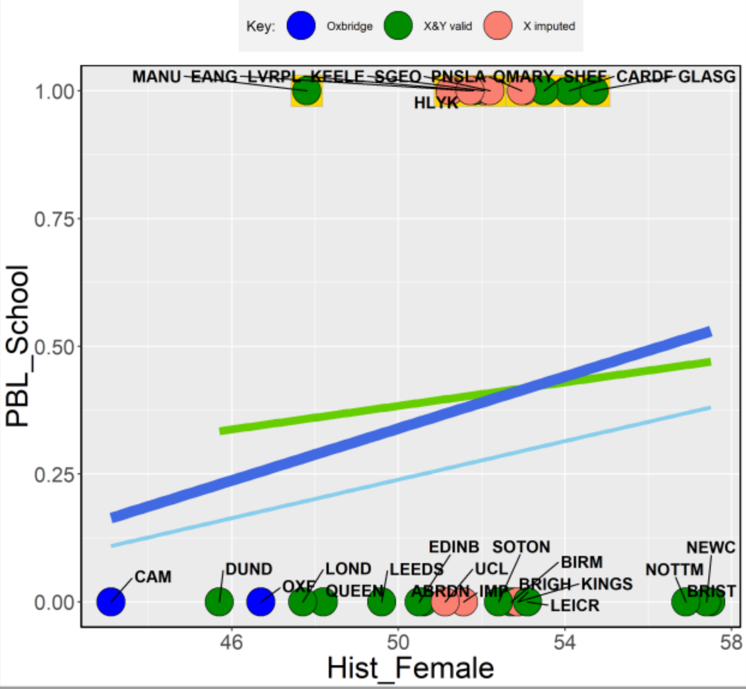

10/59 Y12: Spend_Student X2: Hist_Female

r(all)= -0.554 p= 0.00181 r(NonImp)= -0.619 Npairs=29 NimputedPairs=10

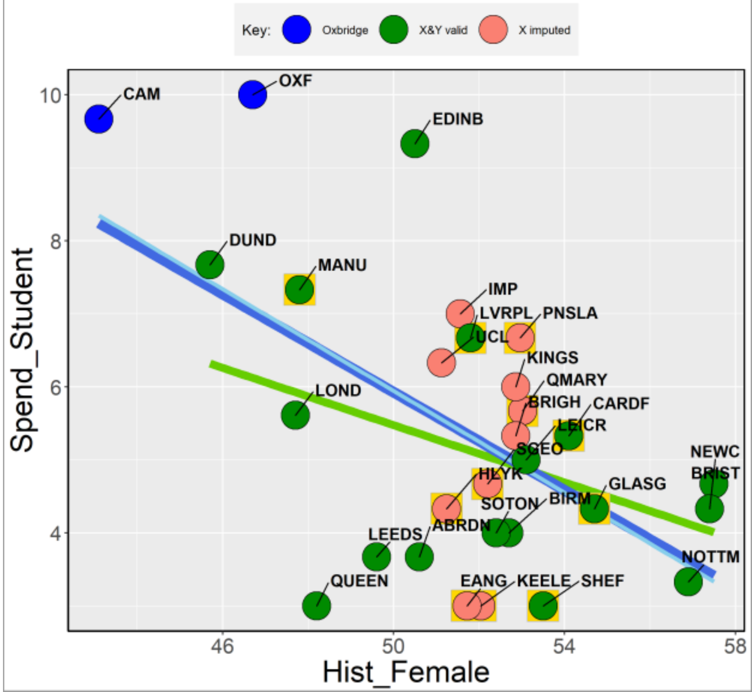

10/60 Y13: Student_Staff X2: Hist_Female

r(all)= 0.298 p= 0.116 r(NonImp)= 0.377 Npairs=29 NimputedPairs=10

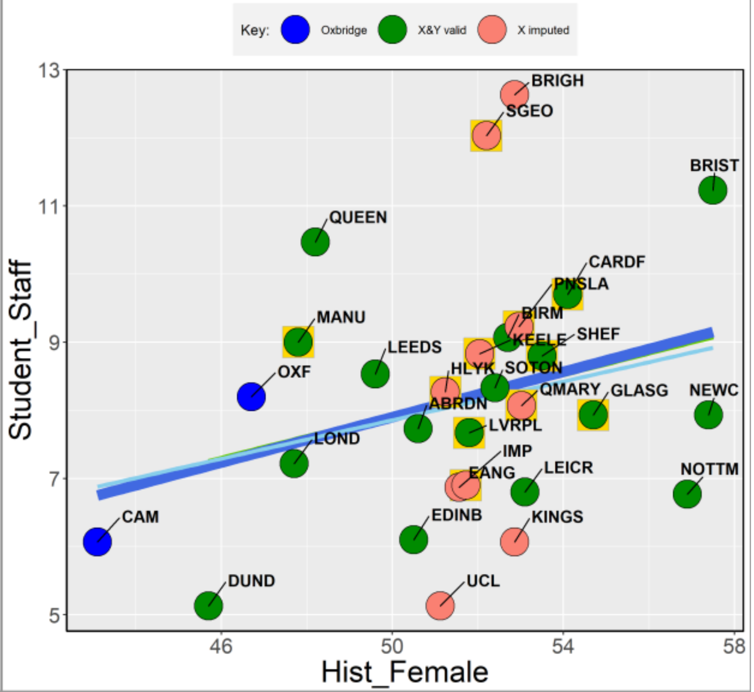

11/61 Y14: Entrants_N X2: Hist_Female

r(all)= 0.151 p= 0.433 r(NonImp)= 0.237 Npairs=29 NimputedPairs=10

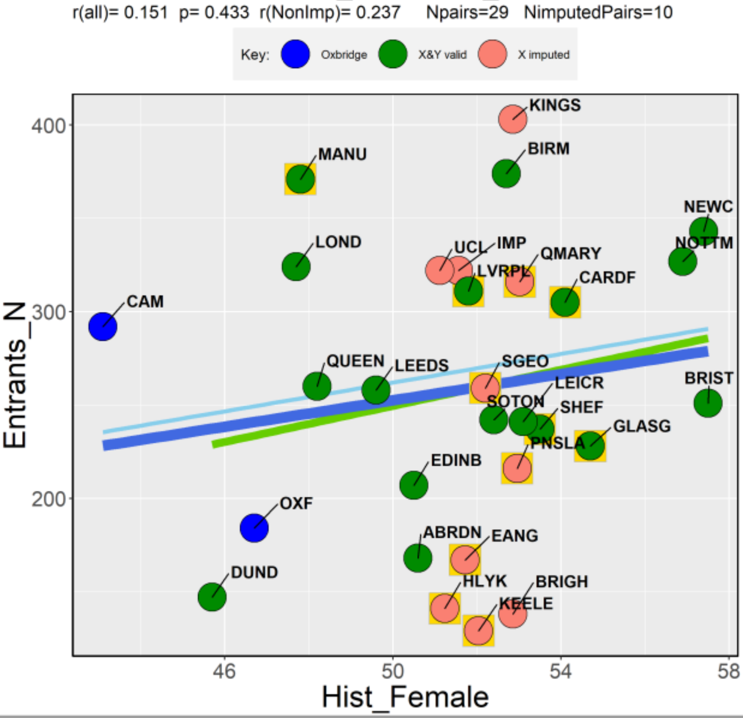

11/62 Y15: Entrants_Female X2: Hist_Female

r(all)= 0.292 p= 0.124 r(NonImp)= 0.373 Npairs=29 NimputedPairs=10

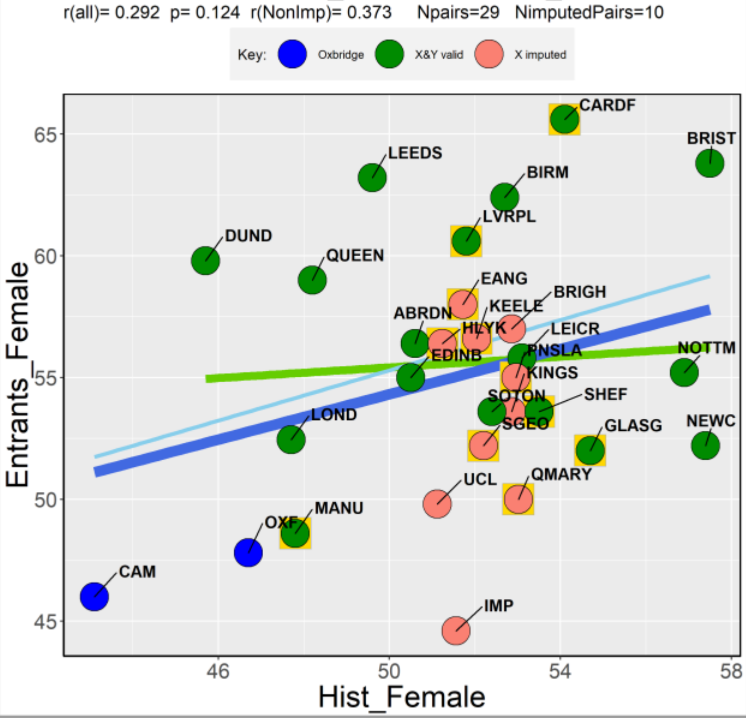

11/63 Y16: EntryGrades X2: Hist_Female

r(all)= -0.472 p= 0.00968 r(NonImp)= -0.495 Npairs=29 NimputedPairs=10

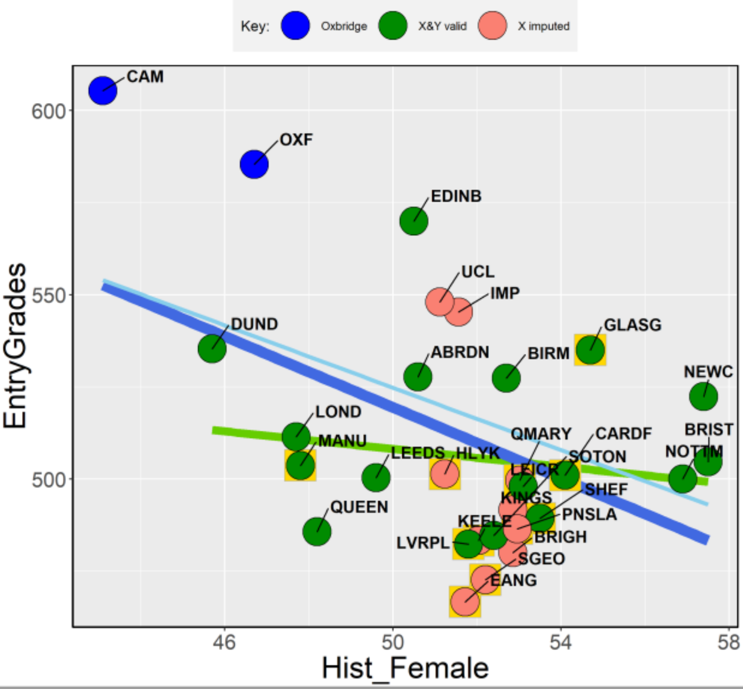

11/64 Y17: Entrants_NonHome X2: Hist_Female

r(all)= -0.228 p= 0.234 r(NonImp)= -0.195 Npairs=29 NimputedPairs=10

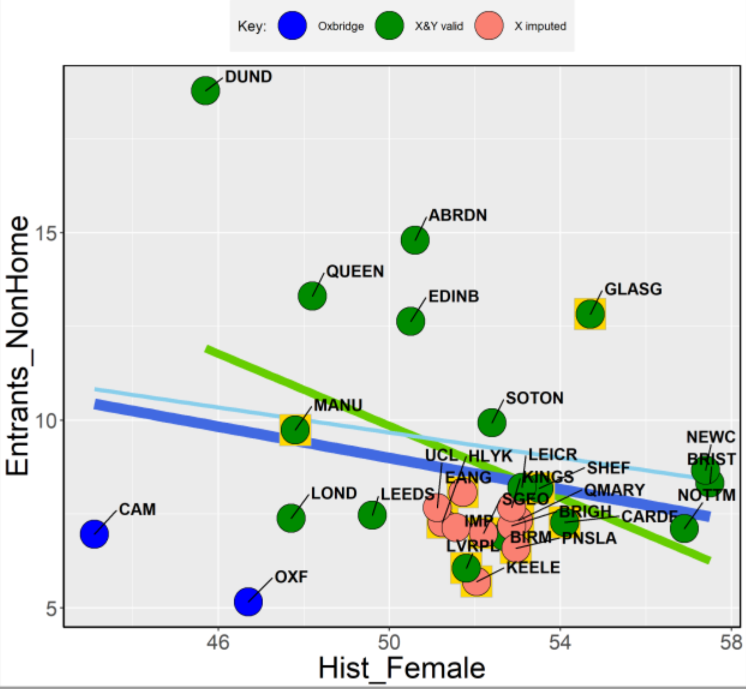

11/65 Y18: Teaching_Factor1_Trad X2: Hist_Female

r(all)= -0.111 p= 0.568 r(NonImp)= -0.179 Npairs=29 NimputedPairs=12

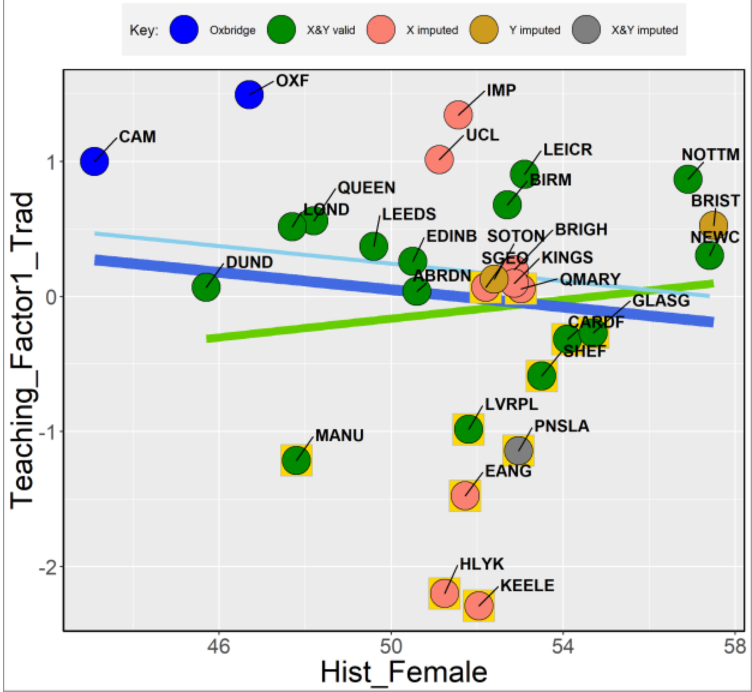

11/66 Y19: Teaching_Factor2_Struc X2: Hist_Female

r(all)= 0.110 p= 0.569 r(NonImp)= 0.128 Npairs=29 NimputedPairs=12

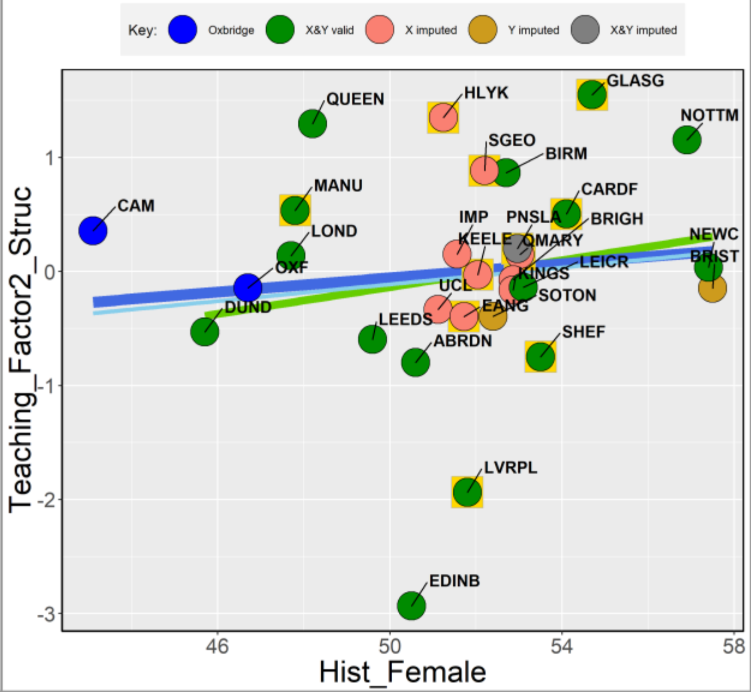

12/67 Y20: Teach_GP X2: Hist_Female

r(all)= -0.159 p= 0.409 r(NonImp)= -0.285 Npairs=29 NimputedPairs=12

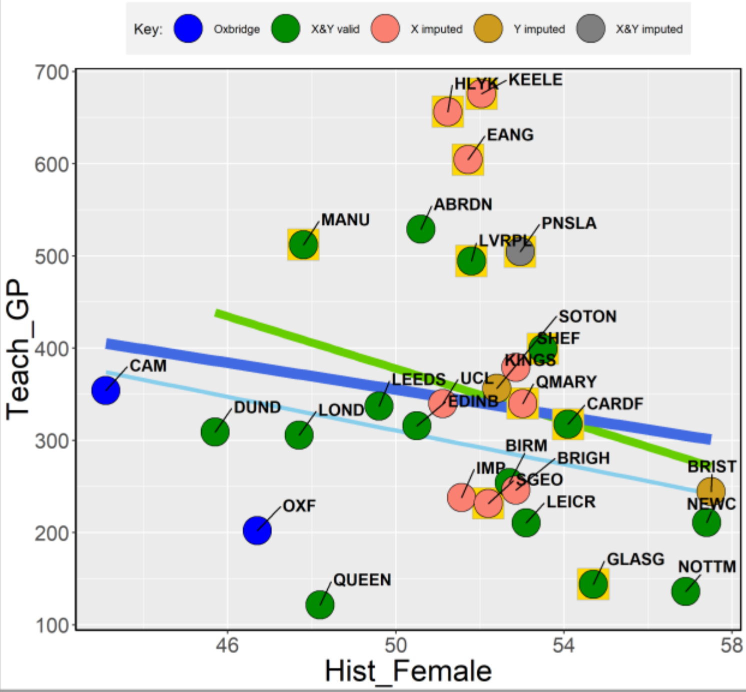

12/68 Y21: Teach_Psyc X2: Hist_Female

r(all)= 0.050 p= 0.798 r(NonImp)= 0.040 Npairs=29 NimputedPairs=12

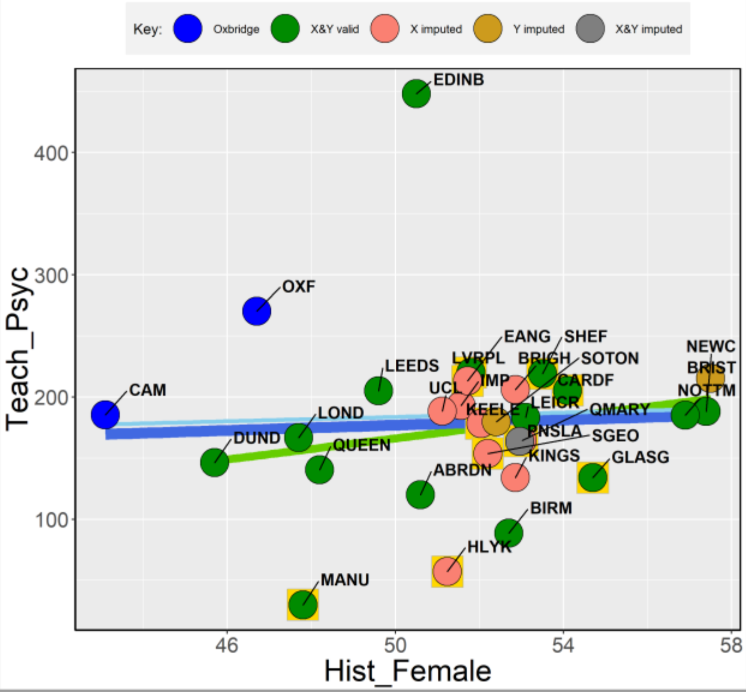

12/69 Y22: Teach_Anaes X2: Hist_Female

r(all)= 0.010 p= 0.96 r(NonImp)= 0.110 Npairs=29 NimputedPairs=12

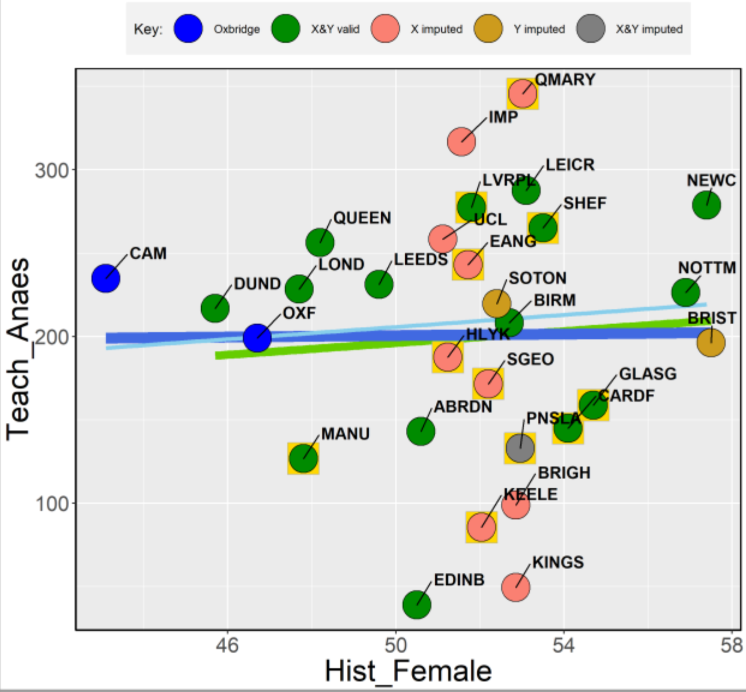

12/70 Y23: Teach_OG X2: Hist_Female

r(all)= -0.228 p= 0.234 r(NonImp)= -0.296 Npairs=29 NimputedPairs=12

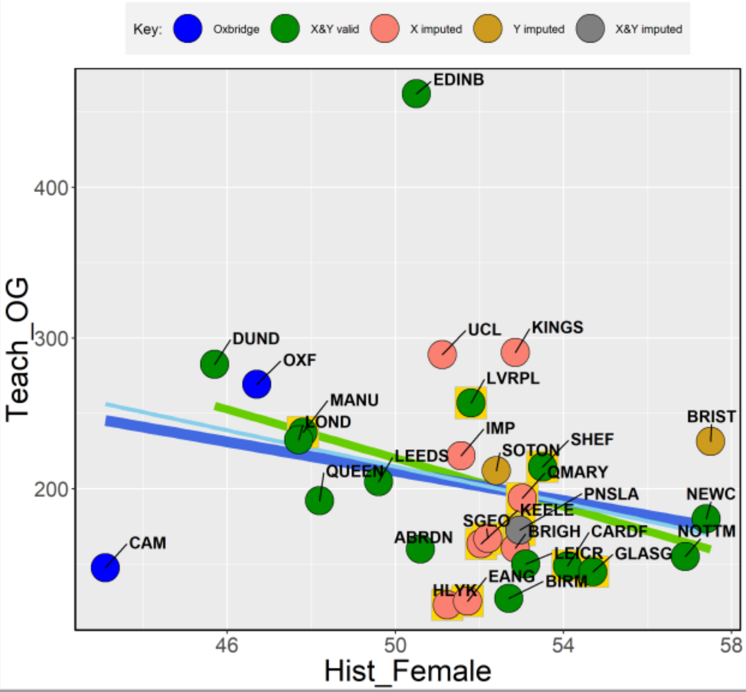

12/71 Y24: Teach_IntMed X2: Hist_Female

r(all)= -0.247 p= 0.196 r(NonImp)= -0.348 Npairs=29 NimputedPairs=12

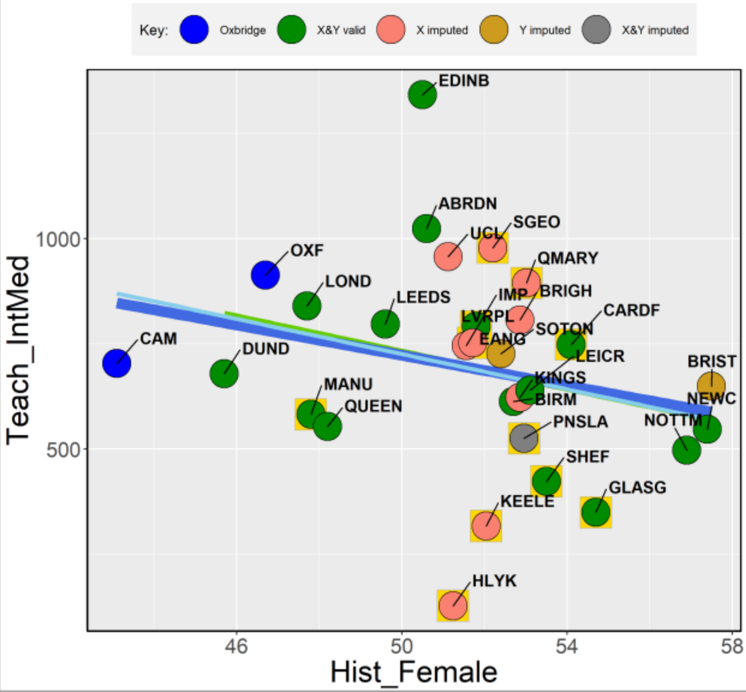

12/72 Y25: Teach_Surgery X2: Hist_Female

r(all)= -0.285 p= 0.135 r(NonImp)= -0.320 Npairs=29 NimputedPairs=12

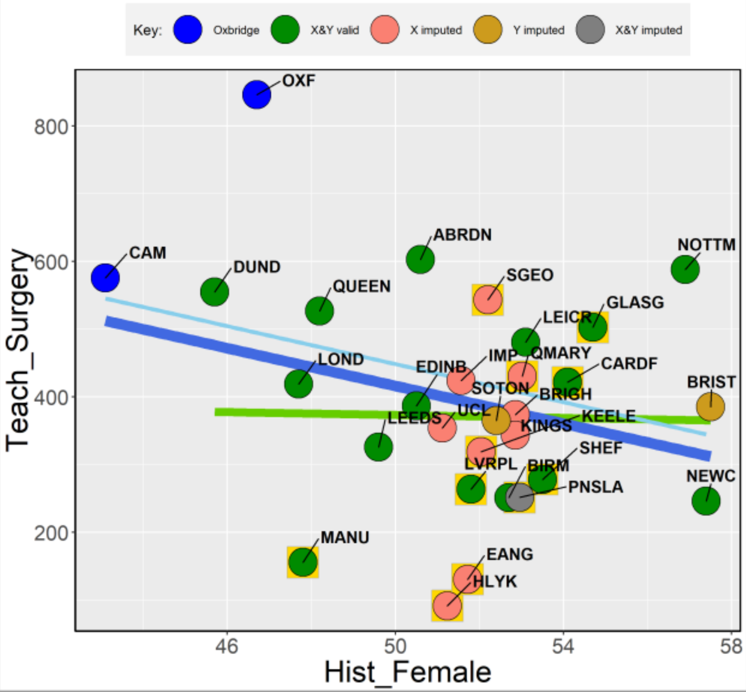

13/73 Y26: ExamTime X2: Hist_Female

r(all)= -0.051 p= 0.793 r(NonImp)= -0.072 Npairs=29 NimputedPairs=11

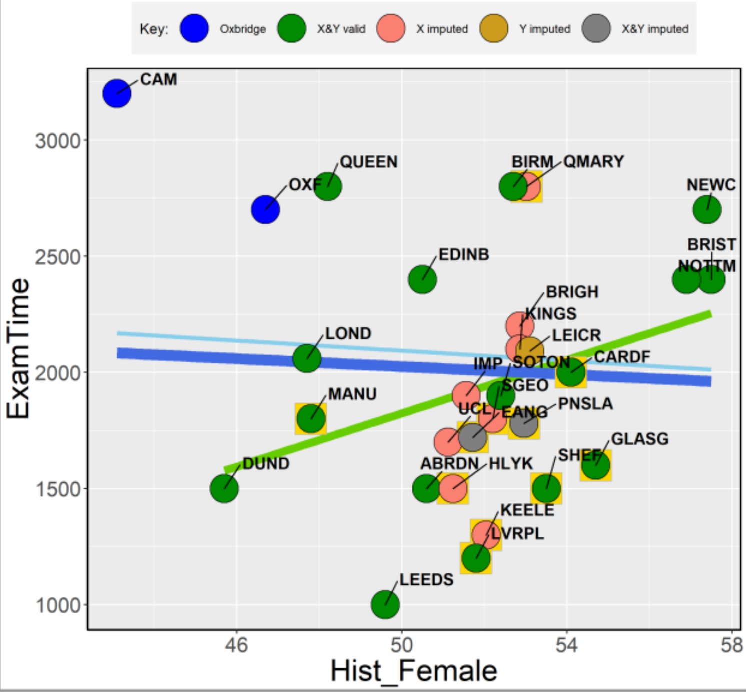

13/74 Y27: SelfRegLearn X2: Hist_Female

r(all)= -0.467 p= 0.0106 r(NonImp)= -0.493 Npairs=29 NimputedPairs=10

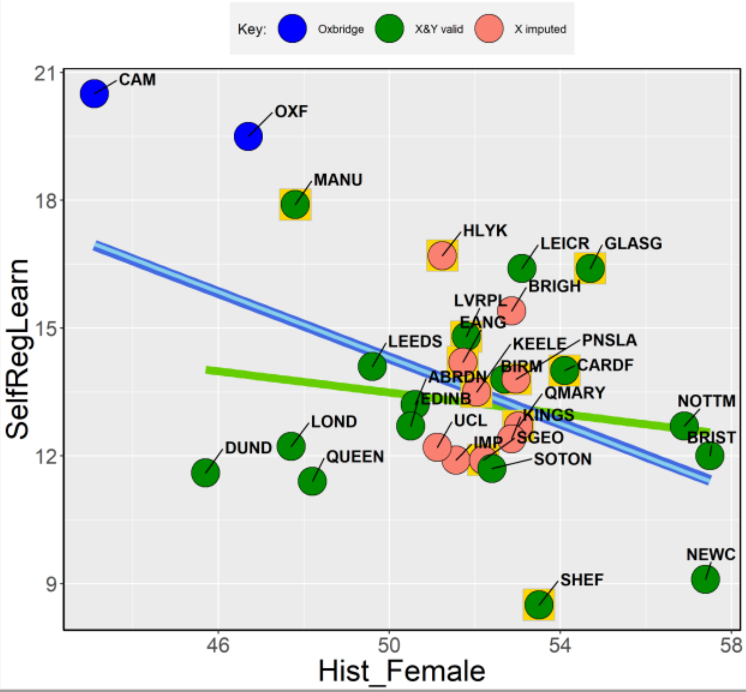

13/75 Y28: NSS_Satisfn X2: Hist_Female

r(all)= -0.160 p= 0.407 r(NonImp)= -0.206 Npairs=29 NimputedPairs=10

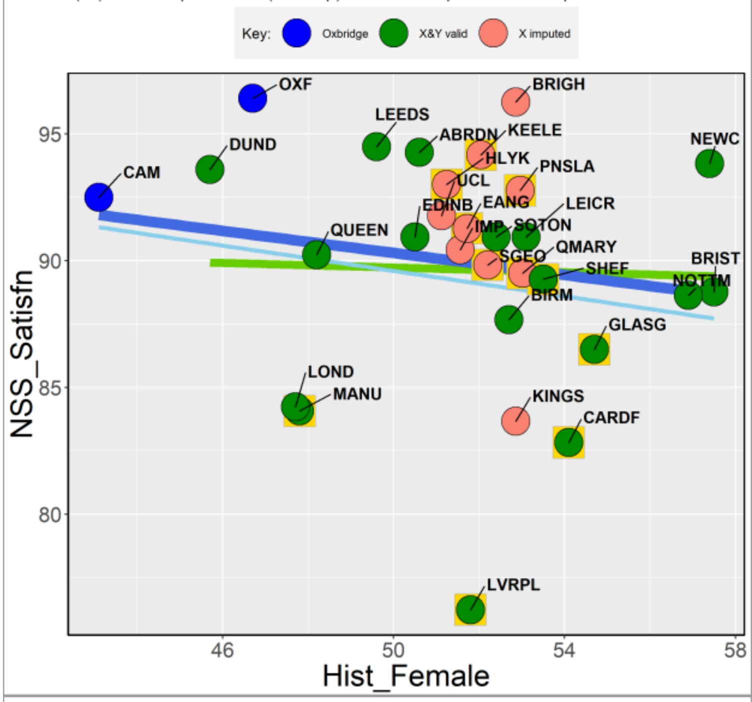

13/76 Y29: NSS_Feedback X2: Hist_Female

r(all)= -0.393 p= 0.0349 r(NonImp)= -0.511 Npairs=29 NimputedPairs=10

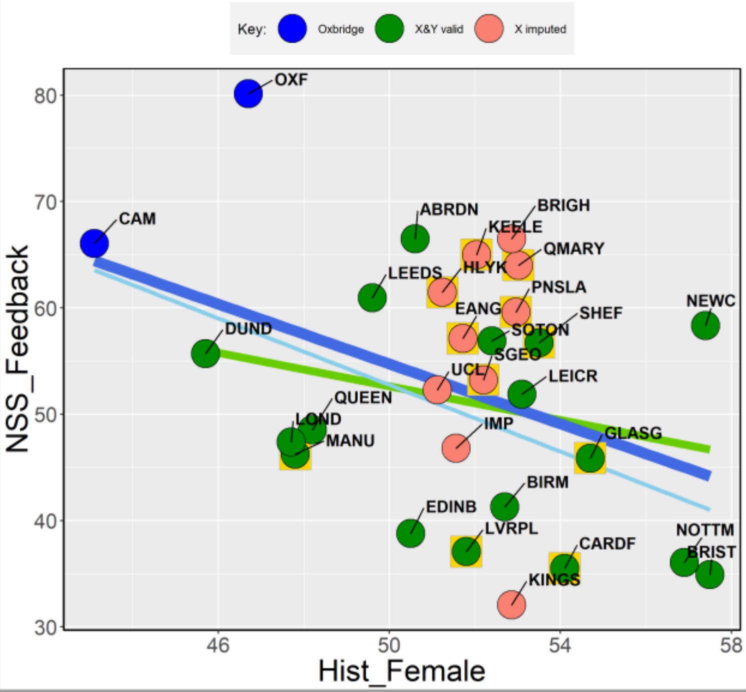

13/77 Y30: UKFPO_EPM X2: Hist_Female

r(all)= -0.321 p= 0.0898 r(NonImp)= -0.456 Npairs=29 NimputedPairs=10

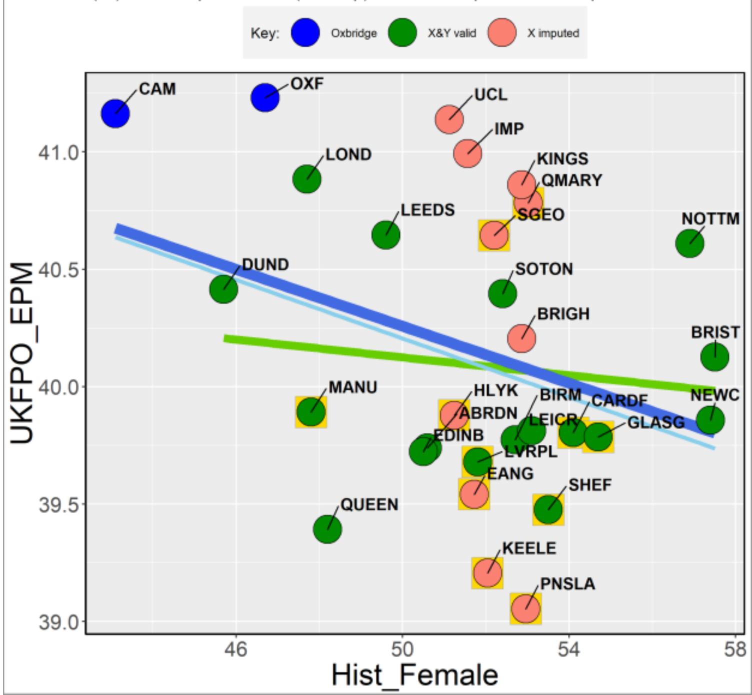

13/78 Y31: UKFPO_SJT X2: Hist_Female

r(all)= -0.328 p= 0.0825 r(NonImp)= -0.349 Npairs=29 NimputedPairs=10

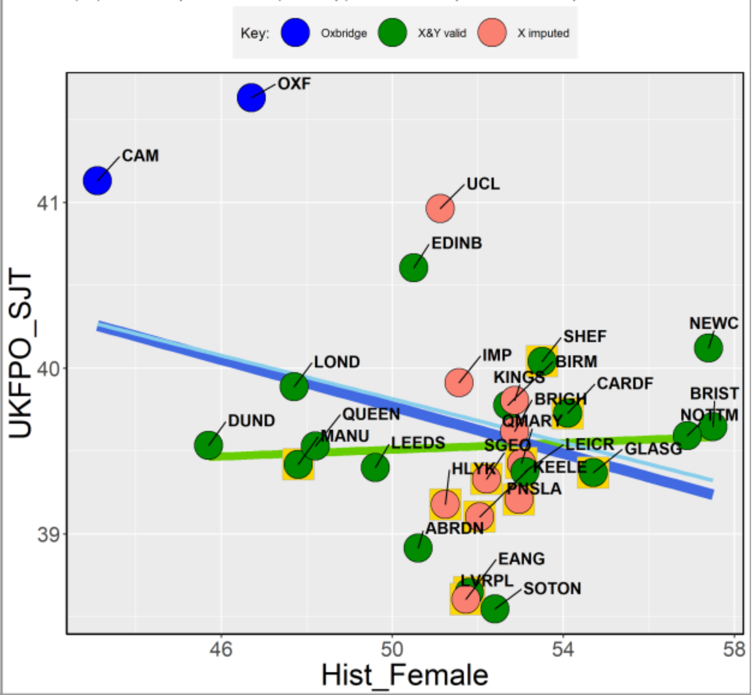

14/79 Y32: F1_Preparedness X2: Hist_Female

r(all)= -0.226 p= 0.239 r(NonImp)= -0.395 Npairs=29 NimputedPairs=10

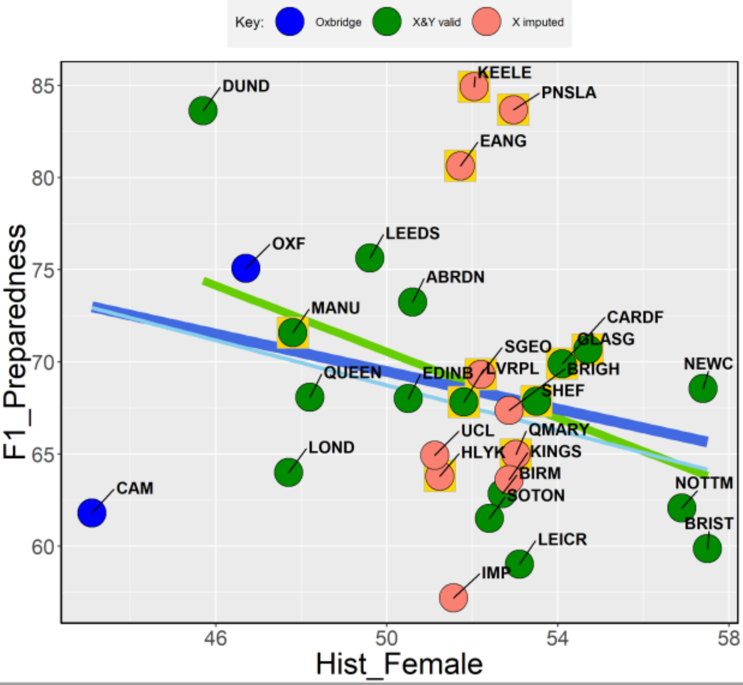

14/80 Y33: F1_Satisfn X2: Hist_Female

r(all)= 0.225 p= 0.241 r(NonImp)= 0.287 Npairs=29 NimputedPairs=10

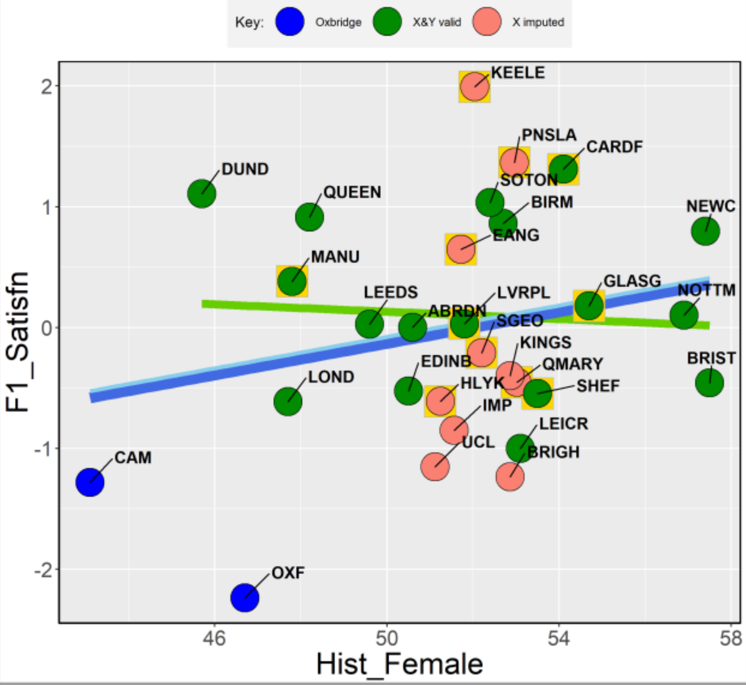

14/81 Y34: F1_Workload X2: Hist_Female

r(all)= 0.289 p= 0.129 r(NonImp)= 0.304 Npairs=29 NimputedPairs=10

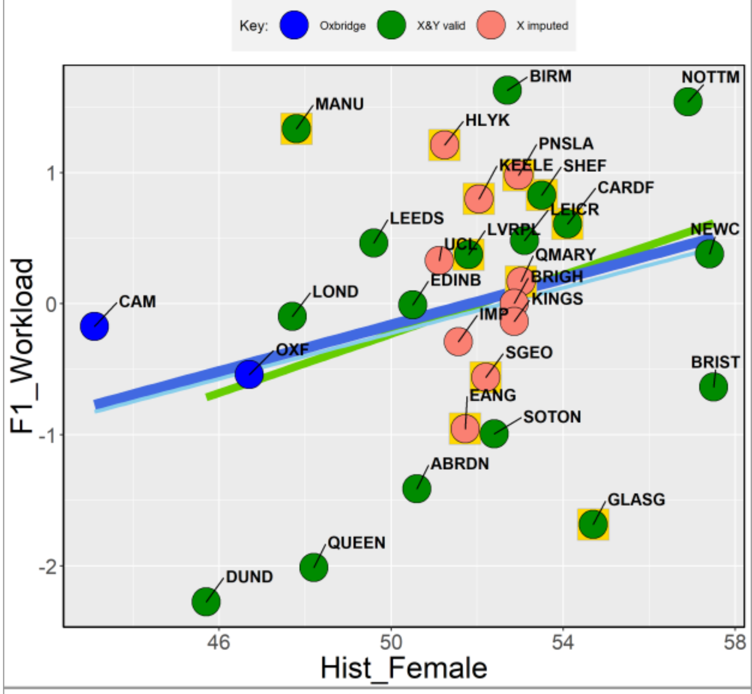

14/82 Y35: F1_Supervn X2: Hist_Female

r(all)= -0.050 p= 0.798 r(NonImp)= -0.076 Npairs=29 NimputedPairs=10

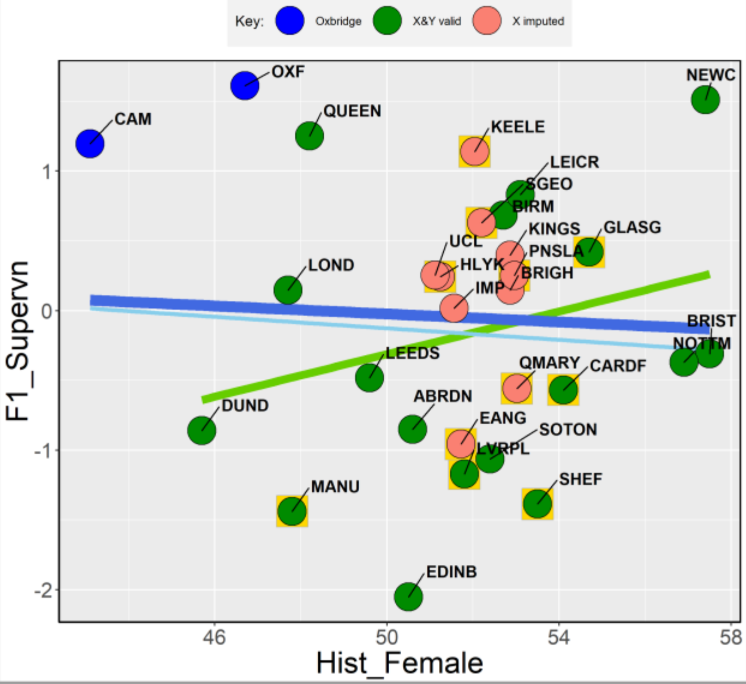

14/83 Y36: Trainee_GP X2: Hist_Female

r(all)= 0.165 p= 0.391 r(NonImp)= 0.201 Npairs=29 NimputedPairs=10

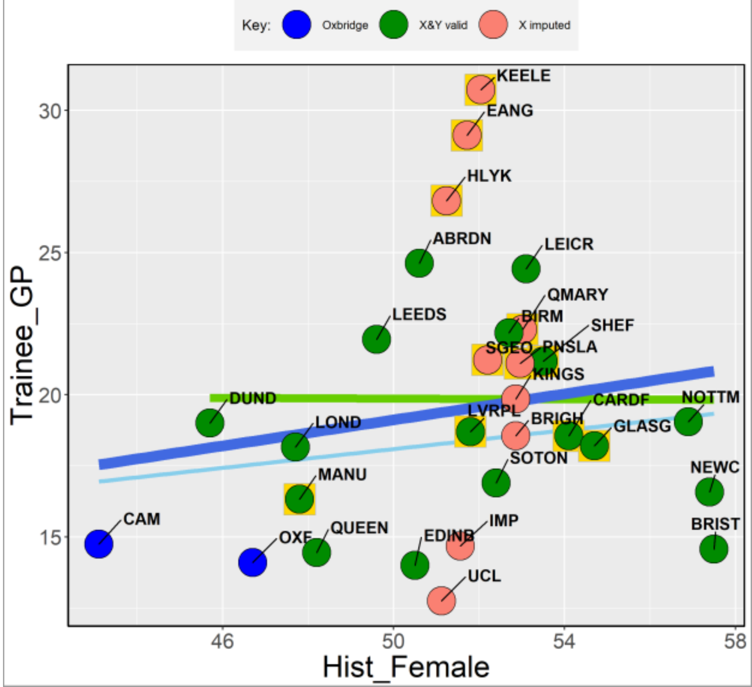

14/84 Y37: Trainee_Psyc X2: Hist_Female

r(all)= -0.095 p= 0.623 r(NonImp)= -0.155 Npairs=29 NimputedPairs=10

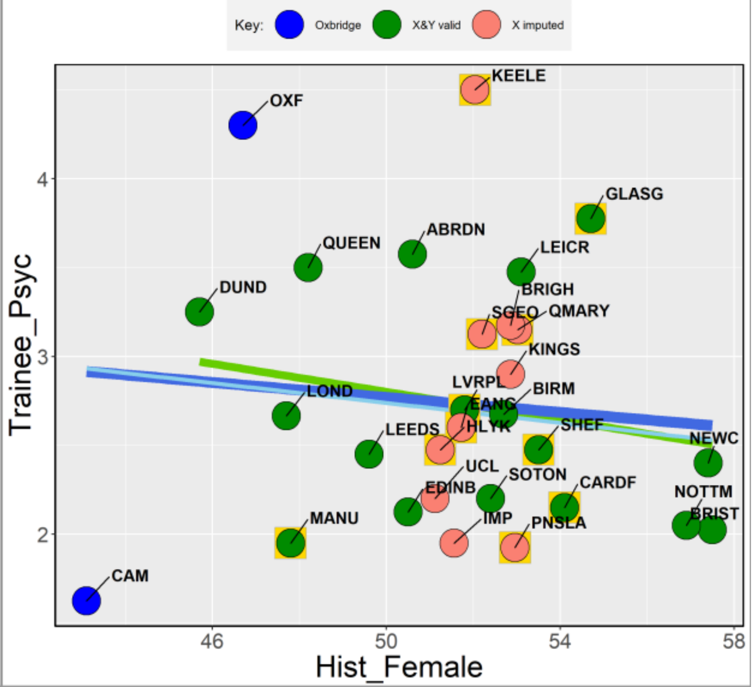

15/85 Y38: TraineeApp_Surgery X2: Hist_Female

r(all)= -0.108 p= 0.576 r(NonImp)= -0.202 Npairs=29 NimputedPairs=10

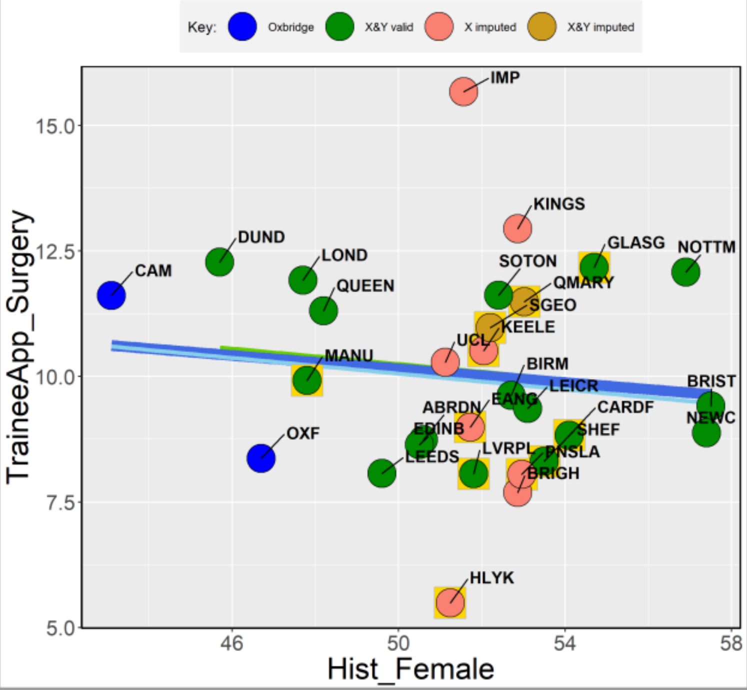

15/86 Y39: TraineeApp_Anaes X2: Hist_Female

r(all)= -0.147 p= 0.445 r(NonImp)= -0.150 Npairs=29 NimputedPairs=10

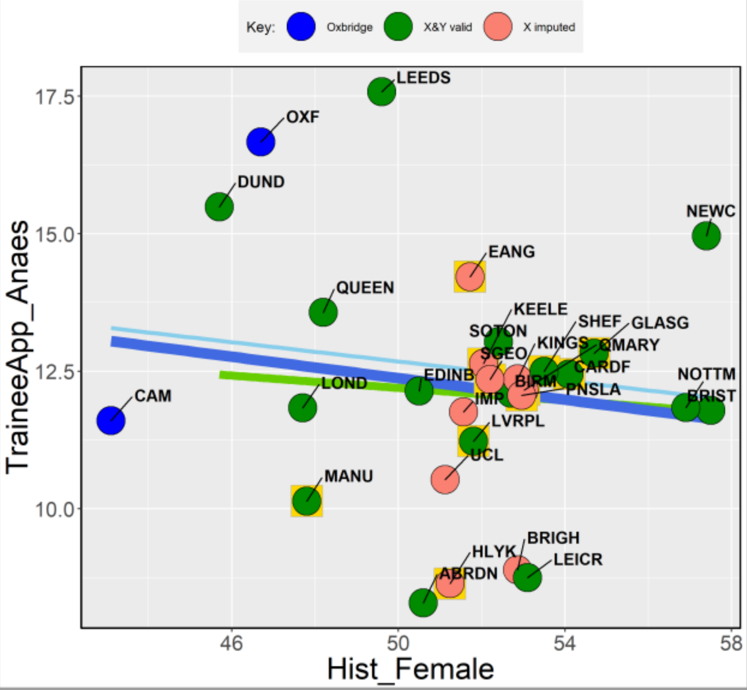

15/87 Y40: GMC_PGexams X2: Hist_Female

r(all)= -0.199 p= 0.301 r(NonImp)= -0.167 Npairs=29 NimputedPairs=10

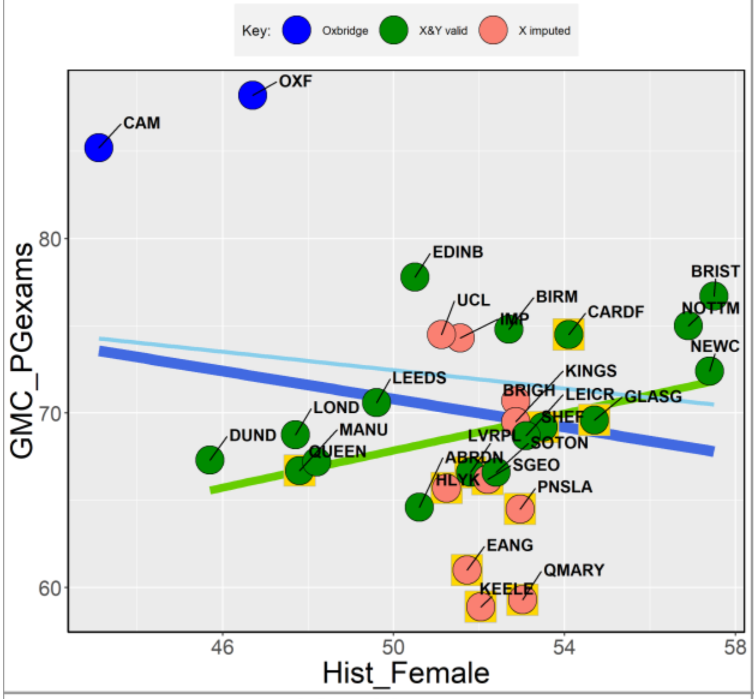

15/88 Y41: MRCGP_AKT X2: Hist_Female

r(all)= -0.247 p= 0.197 r(NonImp)= -0.220 Npairs=29 NimputedPairs=10

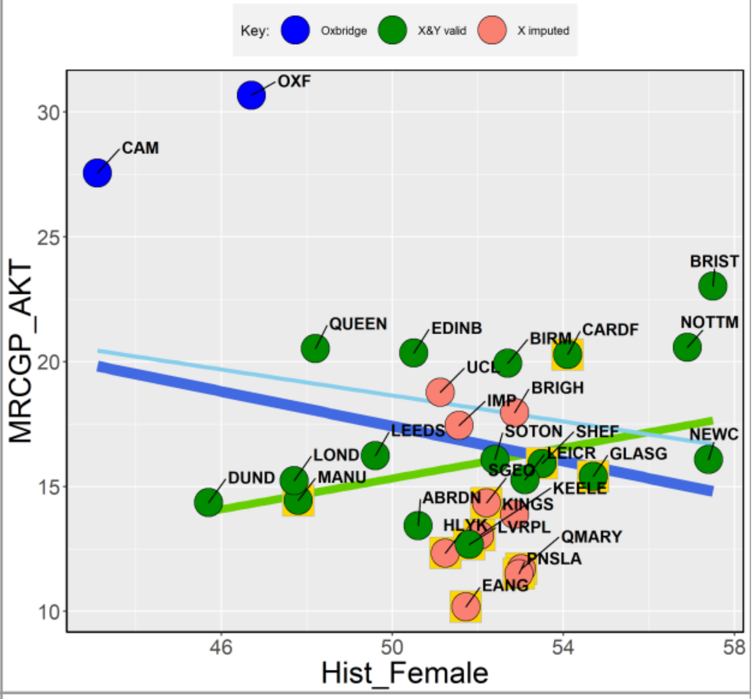

15/89 Y42: MRCGP_CSA X2: Hist_Female

r(all)= -0.061 p= 0.753 r(NonImp)= 0.006 Npairs=29 NimputedPairs=10

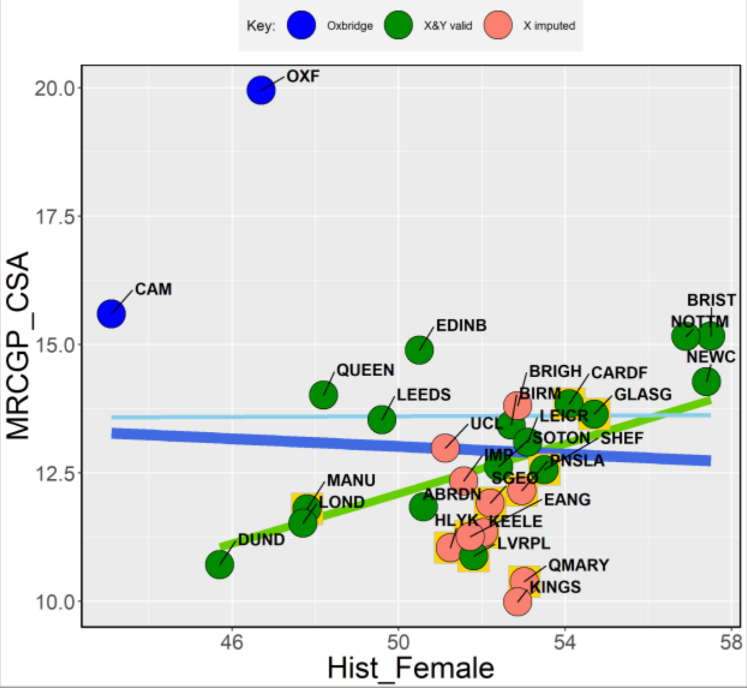

15/90 Y43: FRCA_Pt1 X2: Hist_Female

r(all)= -0.024 p= 0.901 r(NonImp)= -0.019 Npairs=29 NimputedPairs=10

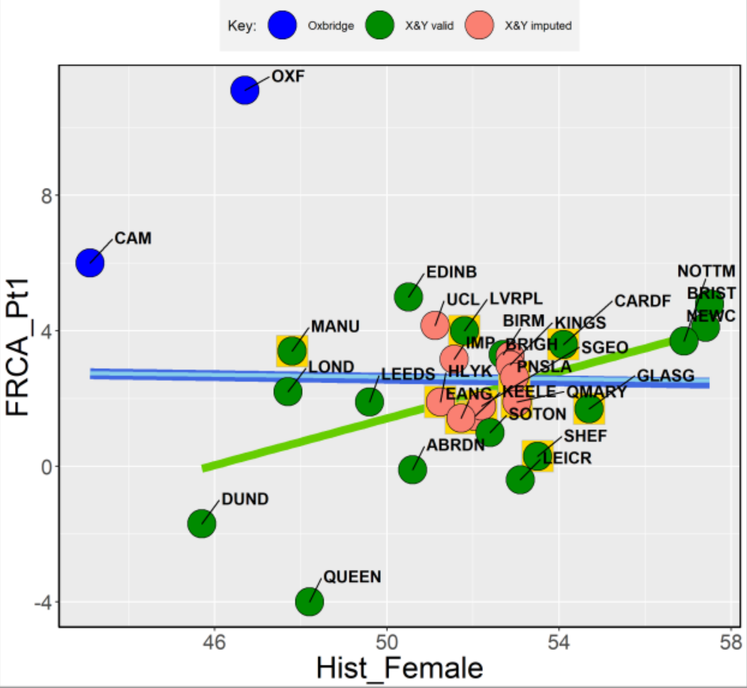

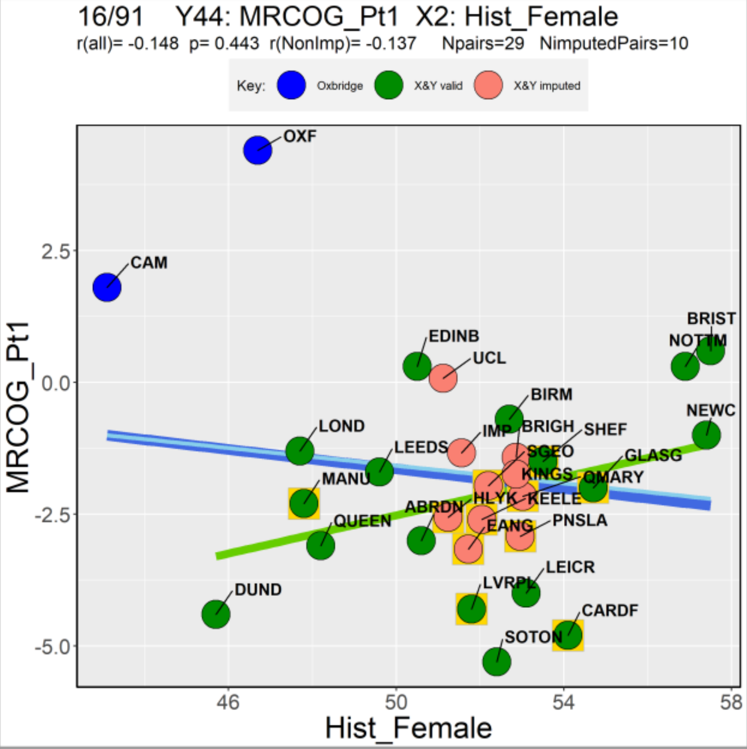
16/91 Y44: MRCOG_Pt1 X2: Hist_Female
r(all)= -0.148 p= 0.443 r(NonImp)= -0.137 Npairs=29 NimputedPairs=10
Key: Oxbridge X&Y valid X&Y imputed
MRCOG_Pt1
Hist_Female

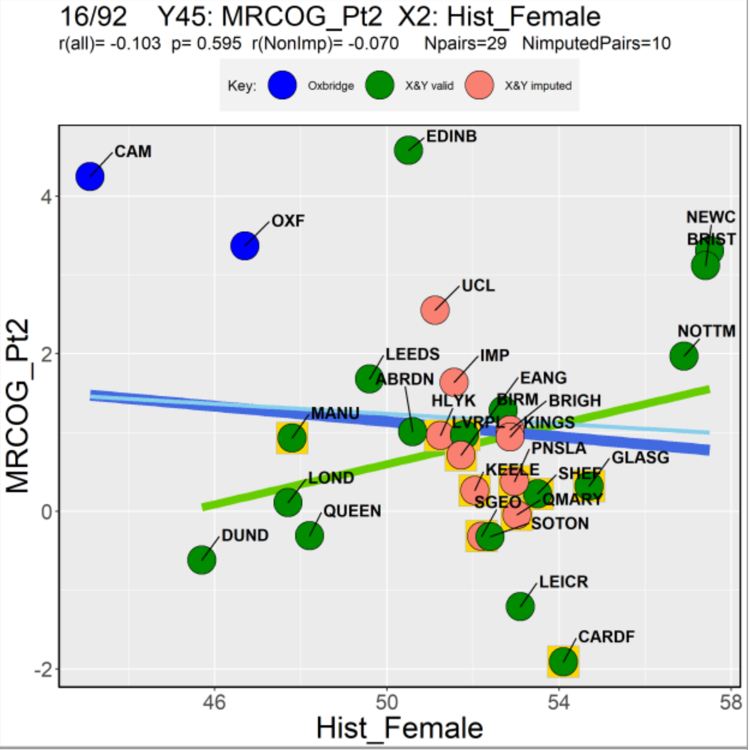
16/92 Y45: MRCOG_Pt2 X2: Hist_Female
r(all)= -0.103 p= 0.595 r(NonImp)= -0.070 Npairs=29 NimputedPairs=10
Key: Oxbridge X&Y valid X&Y imputed
MRCOG_Pt2
Hist_Female

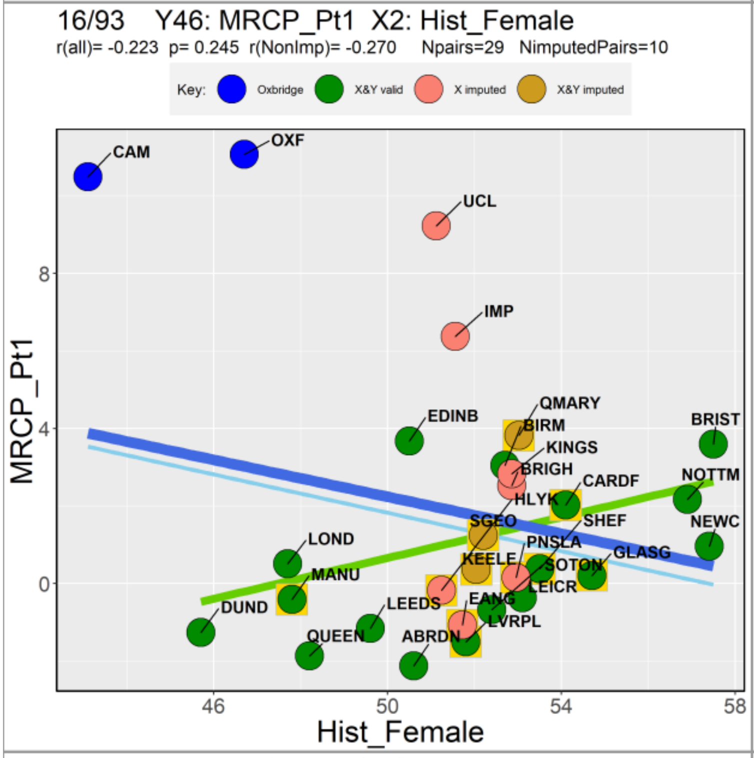
16/93 Y46: MRCP_Pt1 X2: Hist_Female
r(all)= -0.223 p= 0.245 r(NonImp)= -0.270 Npairs=29 NimputedPairs=10
Key: Oxbridge X&Y valid X imputed X&Y imputed
MRCP_Pt1
Hist_Female

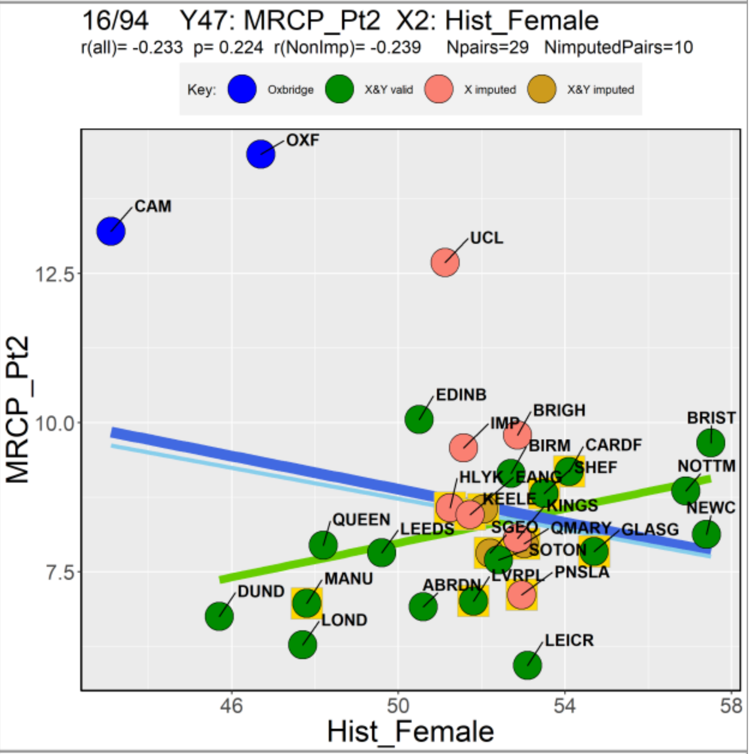
16/94 Y47: MRCP_Pt2 X2: Hist_Female
r(all)= -0.233 p= 0.224 r(NonImp)= -0.239 Npairs=29 NimputedPairs=10
Key: Oxbridge X&Y valid X imputed X&Y imputed
MRCP_Pt2
Hist_Female

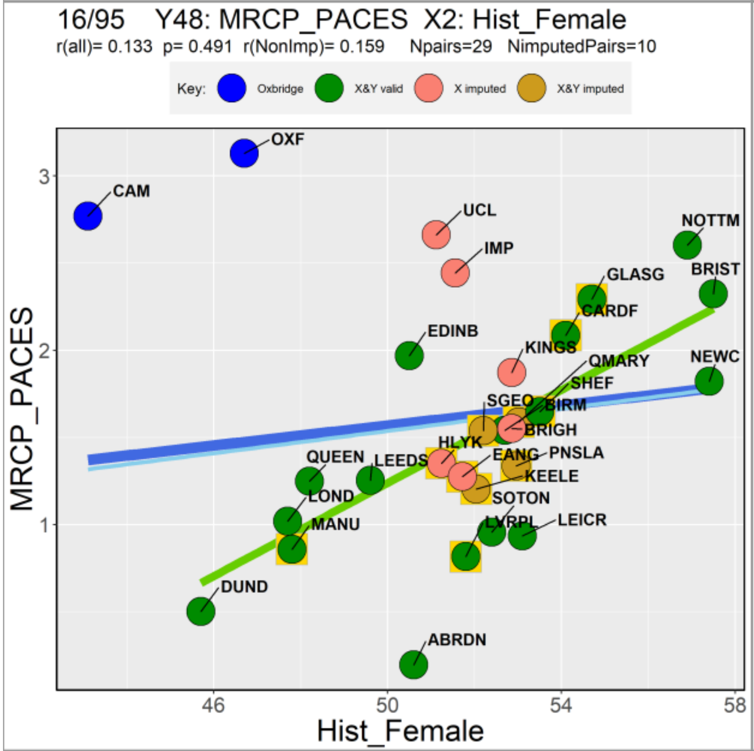
16/95 Y48: MRCP_PACES X2: Hist_Female
r(all)= 0.133 p= 0.491 r(NonImp)= 0.159 Npairs=29 NimputedPairs=10
Key: Oxbridge X&Y valid X imputed X&Y imputed
MRCP_PACES
Hist_Female

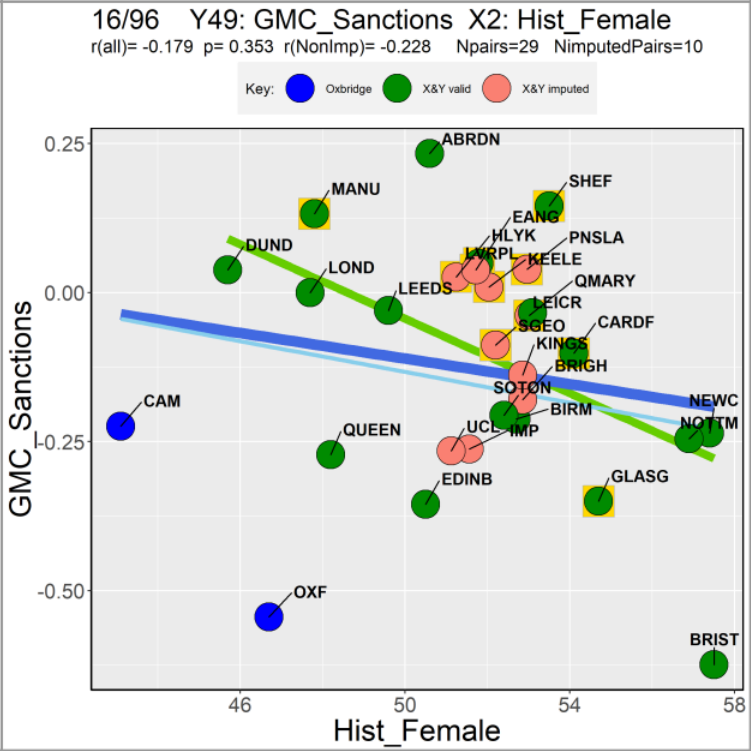
16/96 Y49: GMC_Sanctions X2: Hist_Female
r(all)= -0.179 p= 0.353 r(NonImp)= -0.228 Npairs=29 NimputedPairs=10
Key: Oxbridge X&Y valid X&Y imputed
GMC_Sanctions
Hist_Female

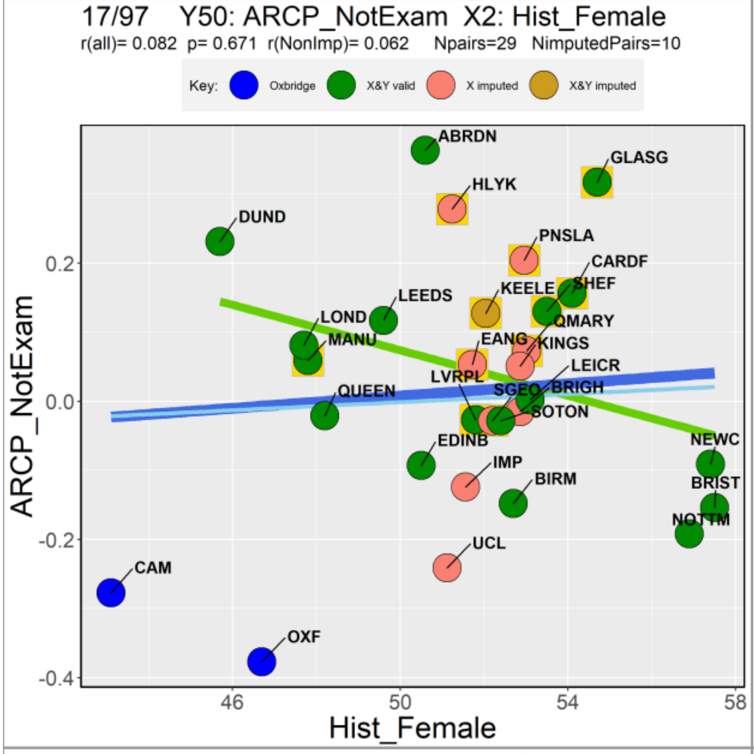
17/97 Y50: ARCP_NotExam X2: Hist_Female
r(all)= 0.082 p= 0.671 r(NonImp)= 0.062 Npairs=29 NimputedPairs=10
Key: Oxbridge X&Y valid X imputed X&Y imputed
ARCP_NotExam
Hist_Female
ABRDN GLASG HLYK DUND PNSLA CARDF LEEDS KEELE SHEF LOND QMARY MANU EANG KINGS LEICR LVRPL QUEEN SGEO BRIGH SOTON EDINB NEWC IMP BIRM BRIST NOTTM UCL CAM OXF

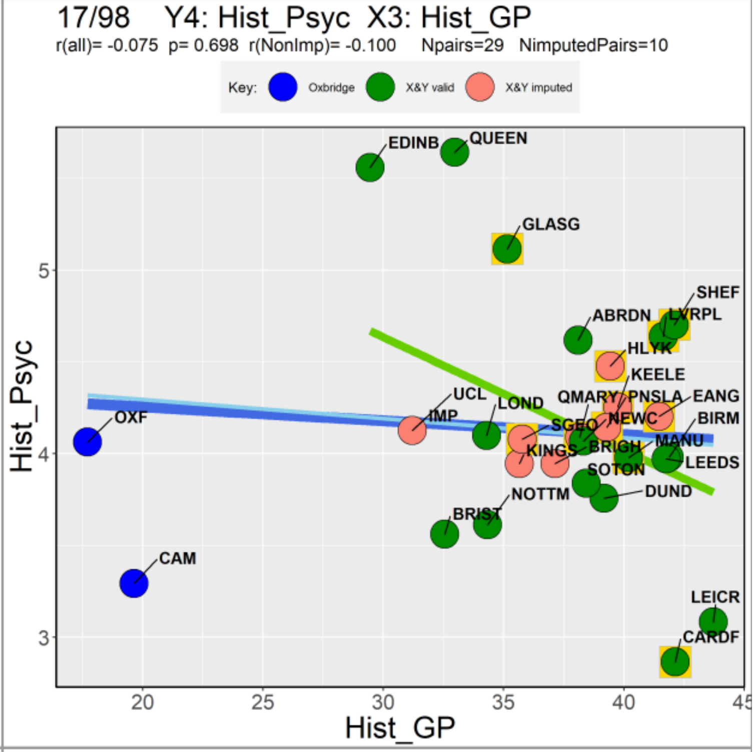
17/98 Y4: Hist_Psyc X3: Hist_GP
r(all)= -0.075 p= 0.698 r(NonImp)= -0.100 Npairs=29 NimputedPairs=10
Key: Oxbridge X&Y valid X&Y imputed
Hist_Psyc
Hist_GP
EDINB QUEEN GLASG SHEF ABRDN LVRPL HLYK KEELE UCL LOND QMARY PNSLA EANG OXF IMP SGEO NEWC BIRM KINGS BRIGH MANU LEEDS SOTON NOTTM DUND BRIST CAM LEICR CARDF

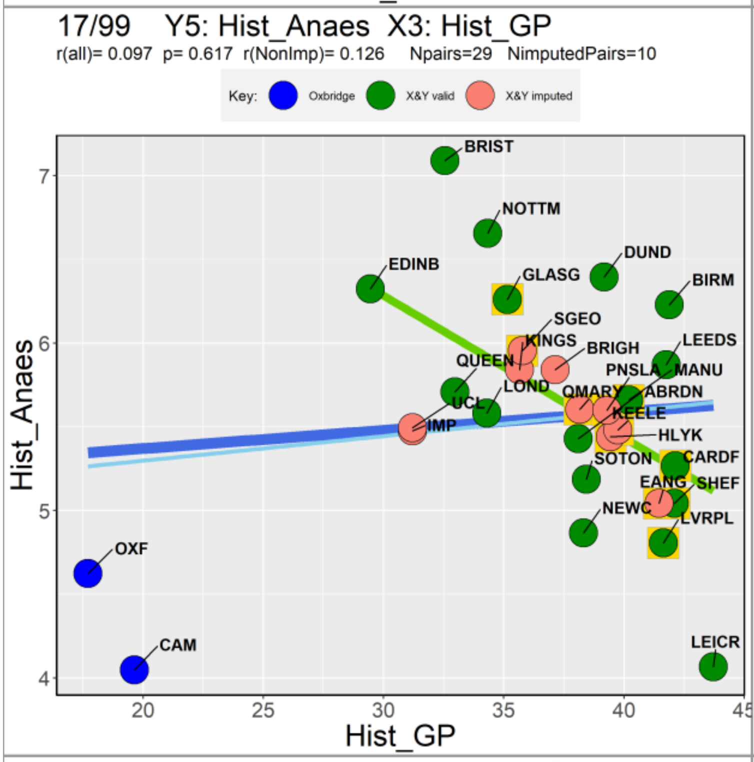
17/99 Y5: Hist_Anaes X3: Hist_GP
r(all)= 0.097 p= 0.617 r(NonImp)= 0.126 Npairs=29 NimputedPairs=10
Key: Oxbridge X&Y valid X&Y imputed
Hist_Anaes
Hist_GP
BRIST NOTTM EDINB DUND GLASG BIRM SGEO KINGS BRIGH LEEDS QUEEN PNSLA MANU LOND QMARY ABRDN UCL KEELE IMP HLYK SOTON CARDF EANG SHEF NEWC LVRPL OXF CAM LEICR

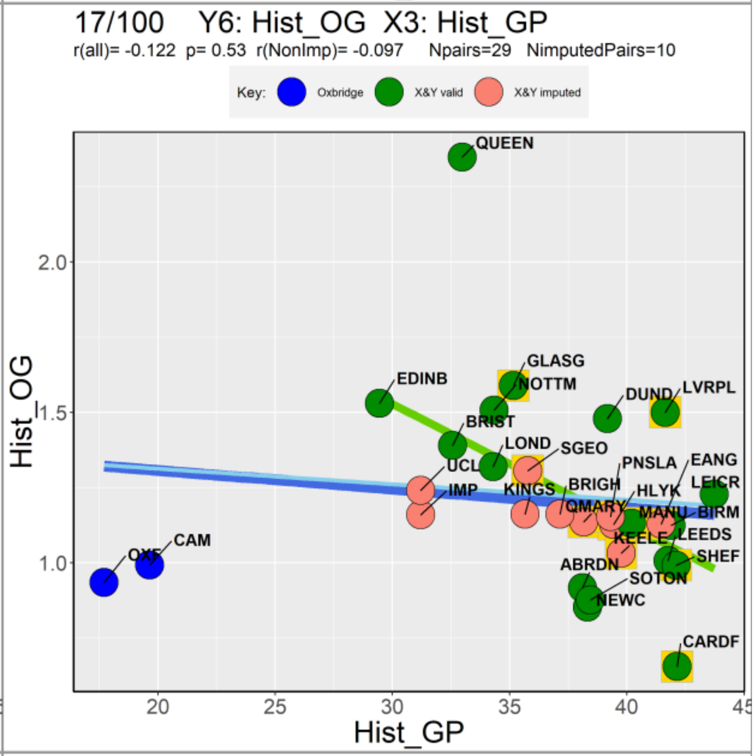
17/100 Y6: Hist_OG X3: Hist_GP
r(all)= -0.122 p= 0.53 r(NonImp)= -0.097 Npairs=29 NimputedPairs=10
Key: Oxbridge X&Y valid X&Y imputed
Hist_OG
Hist_GP
QUEEN GLASG EDINB NOTTM DUND LVRPL BRIST LOND SGEO PNSLA EANG UCL IMP KINGS BRIGH HLYK LEICR QMARY MANU BIRM KEELE LEEDS SHEF CAM OXF ABRDN SOTON NEWC CARDF

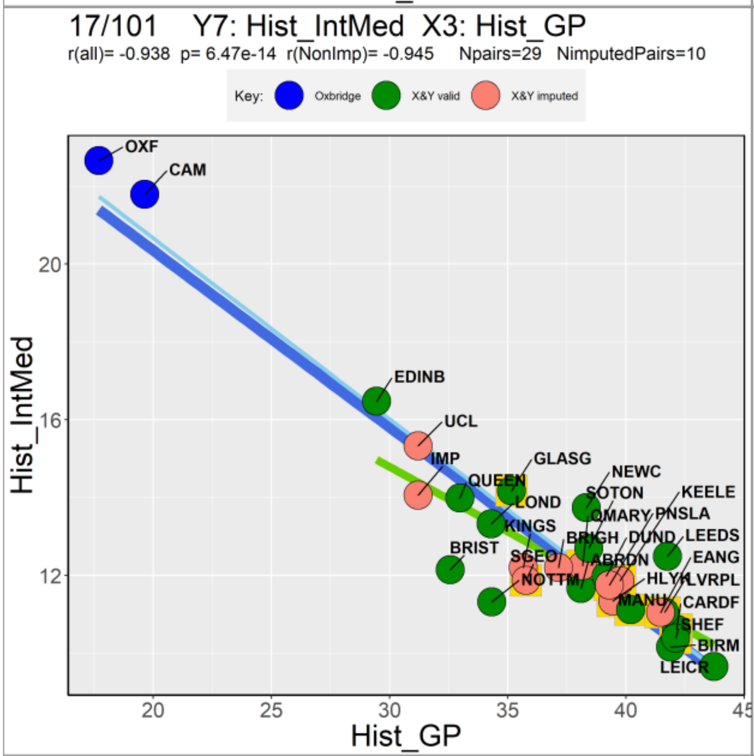
17/101 Y7: Hist_IntMed X3: Hist_GP
r(all)= -0.938 p= 6.47e-14 r(NonImp)= -0.945 Npairs=29 NimputedPairs=10
Key: Oxbridge X&Y valid X&Y imputed
Hist_IntMed
Hist_GP
OXF CAM EDINB UCL IMP GLASG NEWC QUEEN SOTON KEELE LOND QMARY PNSLA KINGS BRIGH DUND LEEDS BRIST SGEO ABRDN EANG NOTTM HLYK LVRPL MANU CARDF SHEF BIRM LEICR

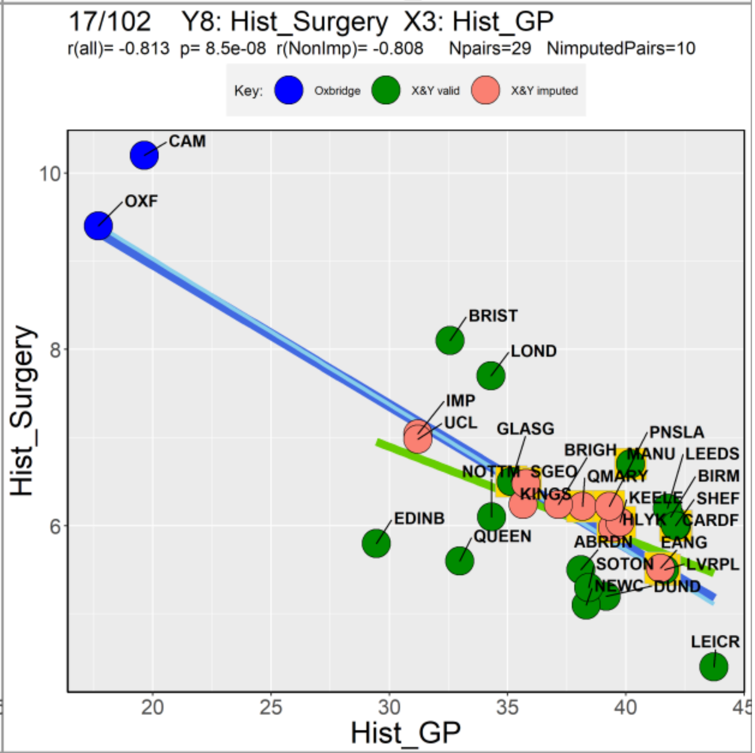
17/102 Y8: Hist_Surgery X3: Hist_GP
r(all)= -0.813 p= 8.5e-08 r(NonImp)= -0.808 Npairs=29 NimputedPairs=10
Key: Oxbridge X&Y valid X&Y imputed
Hist_Surgery
Hist_GP
CAM OXF BRIST LOND IMP UCL GLASG PNSLA BRIGH MANU LEEDS NOTTM SGEO QMARY BIRM KINGS KEELE SHEF HLYK CARDF EDINB QUEEN ABRDN EANG SOTON LVRPL NEWC DUND LEICR

18/103 Y9: Post2000 X3: Hist_GP

r(all)= 0.235 p= 0.22 r(NonImp)= NA Npairs=29 NimputedPairs=10

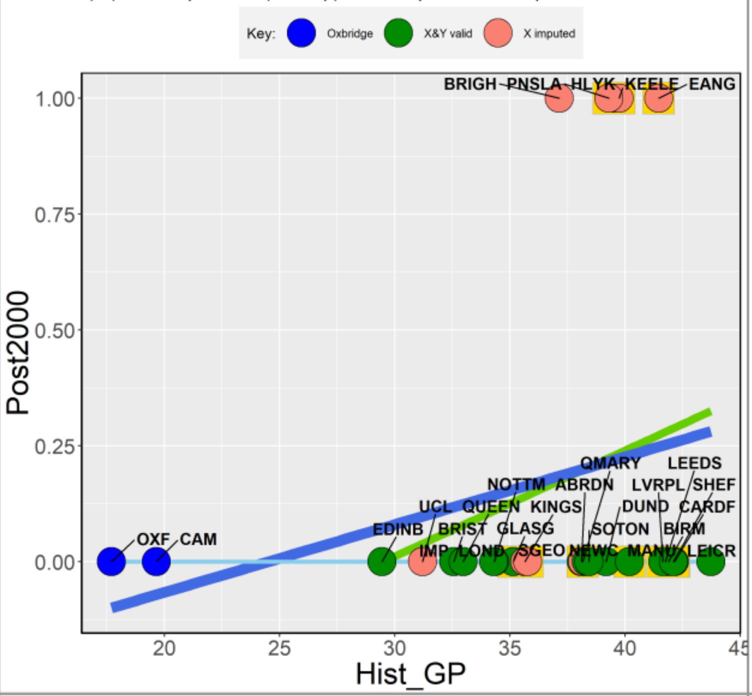

18/104 Y10: REF X3: Hist_GP

r(all)= -0.720 p= 1.05e-05 r(NonImp)= -0.791 Npairs=29 NimputedPairs=10

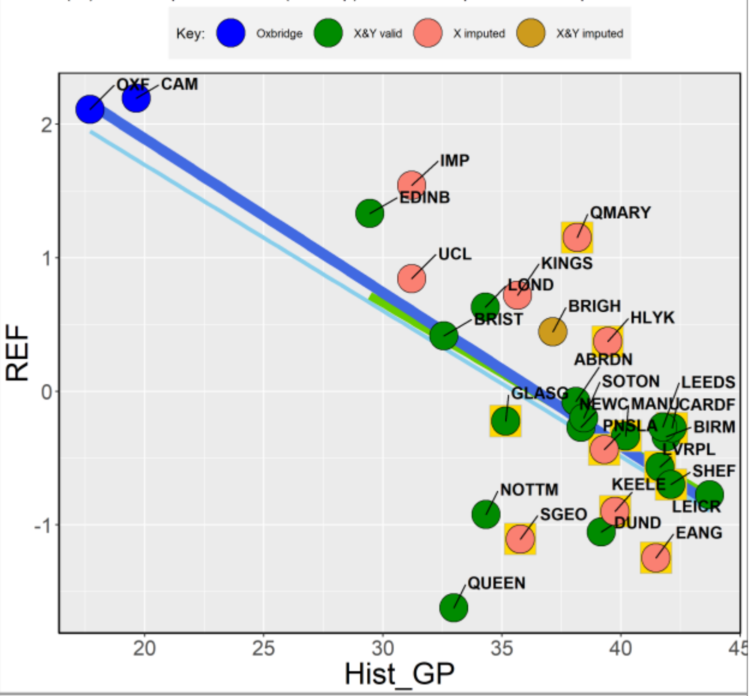

18/105 Y11: PBL_School X3: Hist_GP

r(all)= 0.420 p= 0.0234 r(NonImp)= 0.361 Npairs=29 NimputedPairs=10

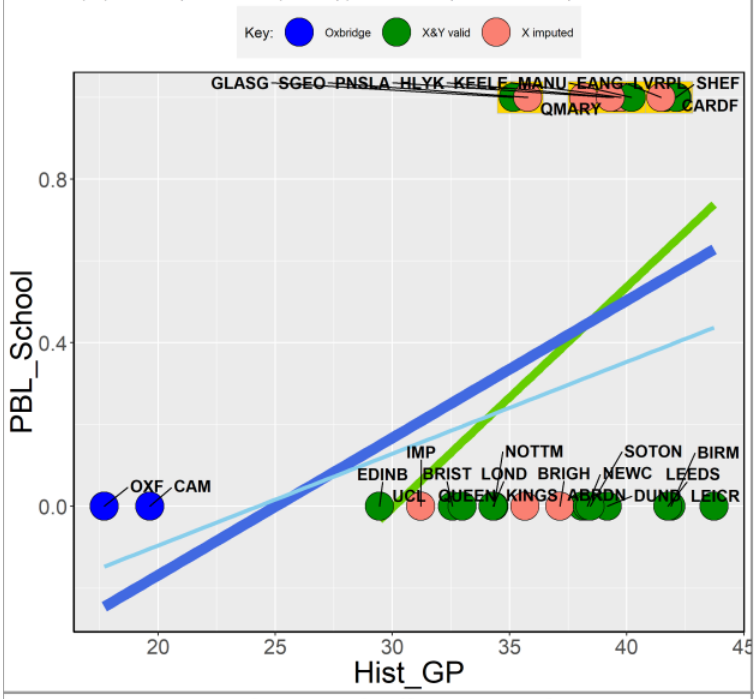

18/106 Y12: Spend_Student X3: Hist_GP

r(all)= -0.642 p= 0.000173 r(NonImp)= -0.634 Npairs=29 NimputedPairs=10

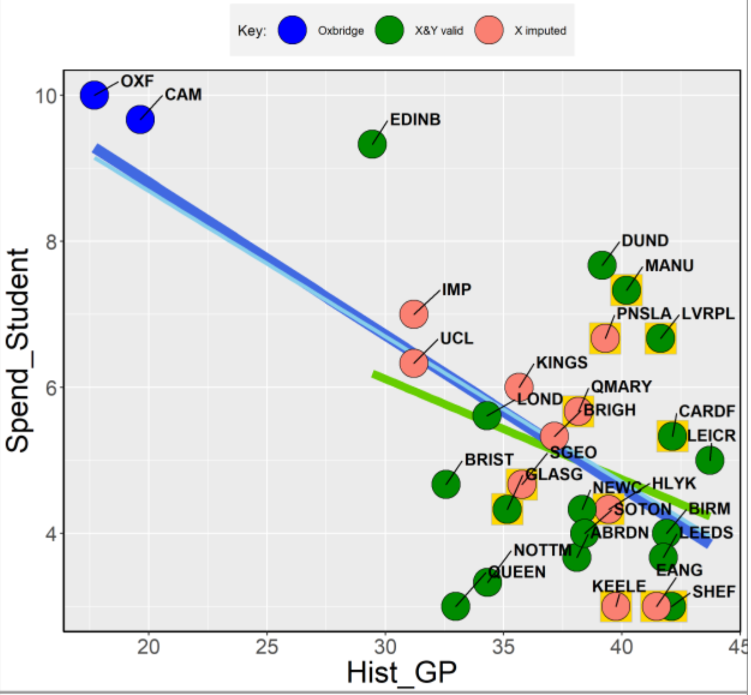

18/107 Y13: Student_Staff X3: Hist_GP

r(all)= 0.185 p= 0.338 r(NonImp)= 0.170 Npairs=29 NimputedPairs=10

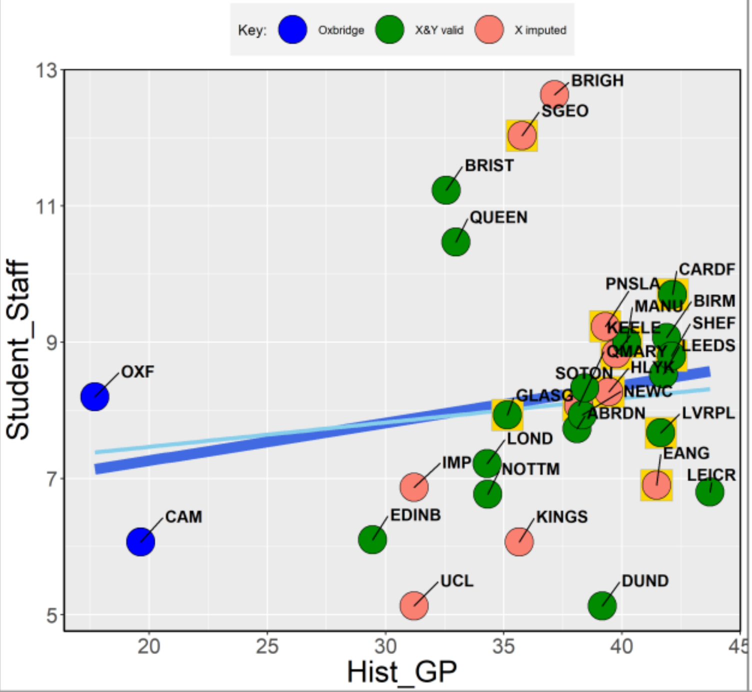

18/108 Y14: Entrants_N X3: Hist_GP

r(all)= -0.011 p= 0.955 r(NonImp)= 0.239 Npairs=29 NimputedPairs=10

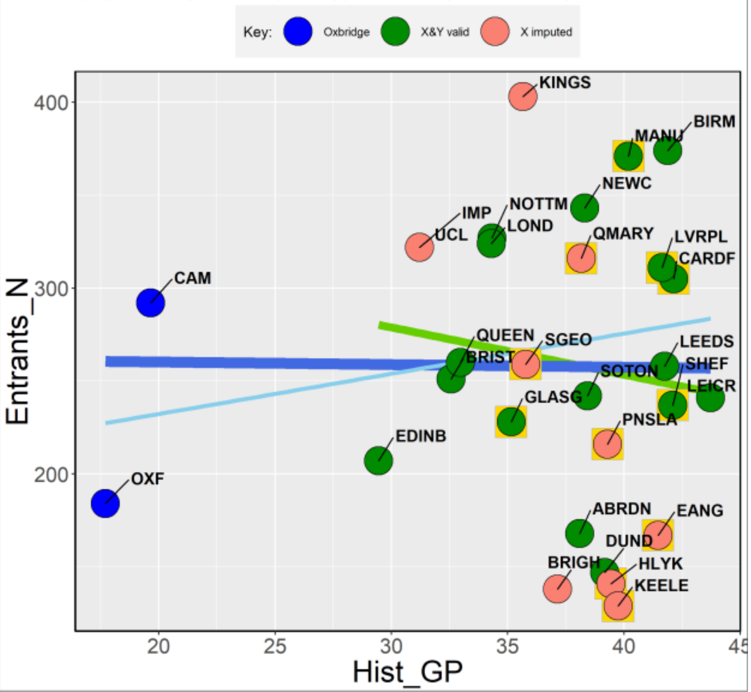

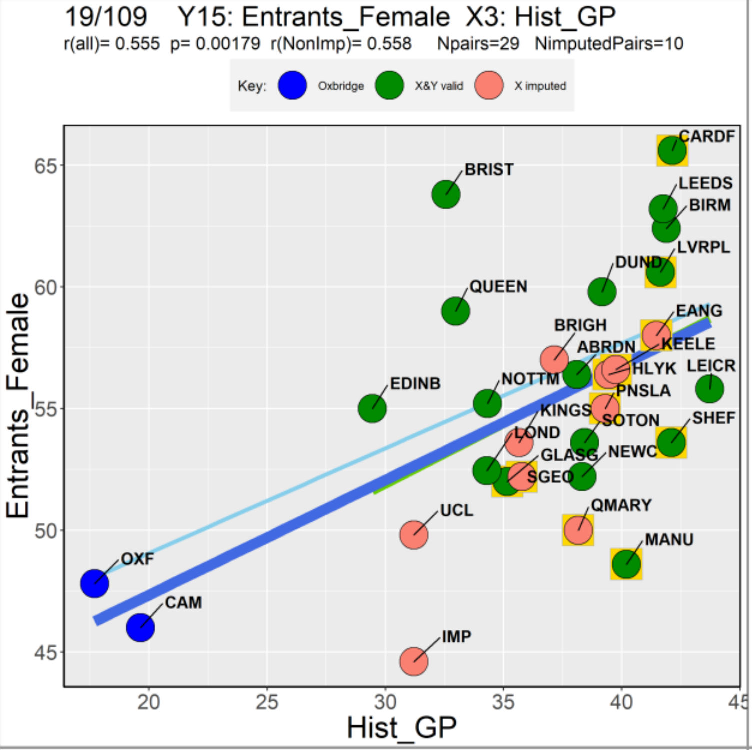
19/109 Y15: Entrants_Female X3: Hist_GP
r(all)= 0.555 p= 0.00179 r(NonImp)= 0.558 Npairs=29 NimputedPairs=10
Key: Oxbridge X&Y valid X imputed
Entrants_Female
Hist_GP
CARDF BRIST LEEDS BIRM LVRPL DUND QUEEN EANG BRIGH ABRDN KEELE HLYK LEICR EDINB NOTTM PNSLA KINGS SOTON SHEF LOND GLASG NEWC SGEO UCL QMARY MANU OXF CAM IMP

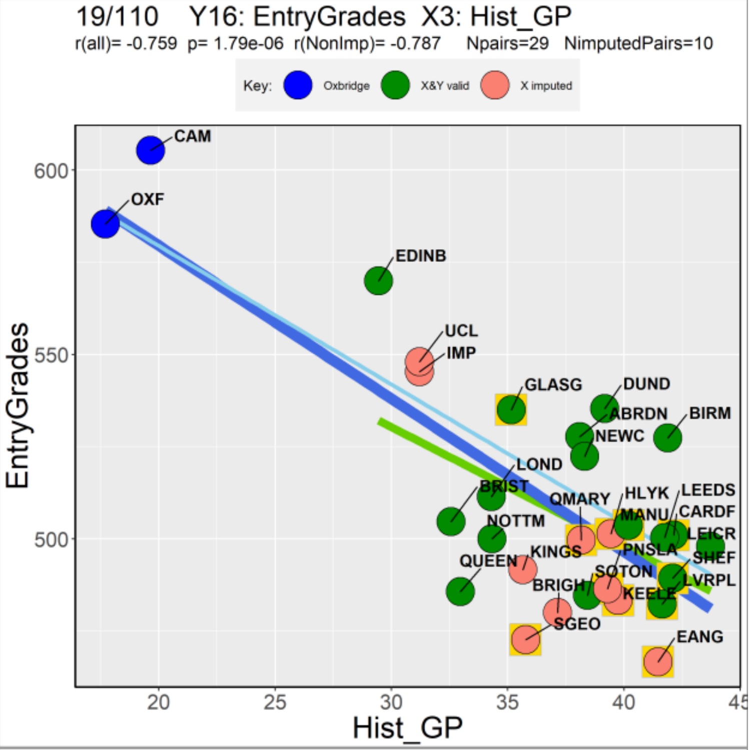
19/110 Y16: EntryGrades X3: Hist_GP
r(all)= -0.759 p= 1.79e-06 r(NonImp)= -0.787 Npairs=29 NimputedPairs=10
Key: Oxbridge X&Y valid X imputed
EntryGrades
Hist_GP
CAM OXF EDINB UCL IMP GLASG DUND ABRDN BIRM NEWC LOND BRIST HLYK LEEDS QMARY MANU CARDF NOTTM LEICR KINGS PNSLA SHEF QUEEN SOTON LVRPL BRIGH KEELE SGEO EANG

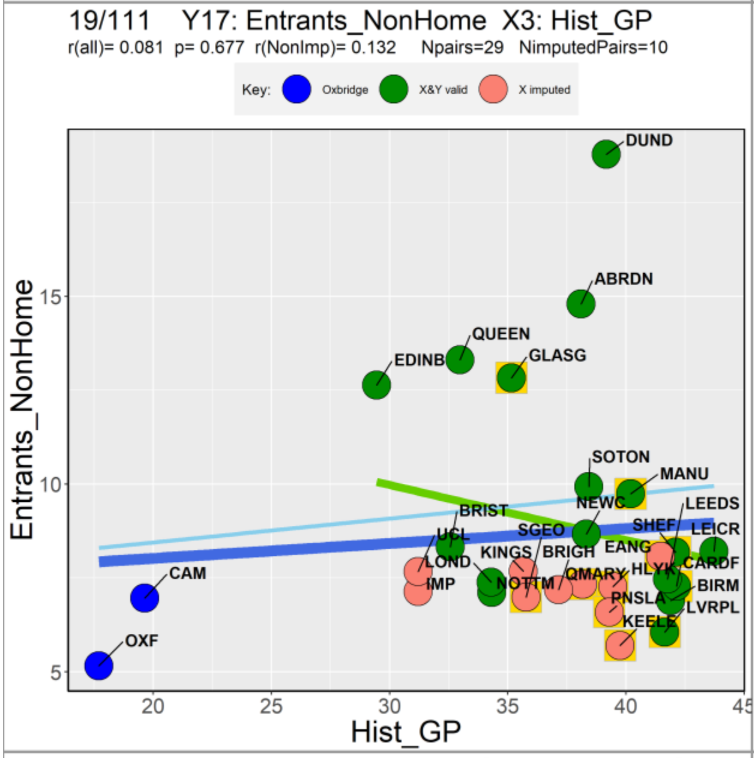
19/111 Y17: Entrants_NonHome X3: Hist_GP
r(all)= 0.081 p= 0.677 r(NonImp)= 0.132 Npairs=29 NimputedPairs=10
Key: Oxbridge X&Y valid X imputed
Entrants_NonHome
Hist_GP
DUND ABRDN QUEEN GLASG EDINB SOTON MANU NEWC LEEDS BRIST SHEF LEICR UCL SGEO EANG LOND KINGS BRIGH CARDF IMP NOTTM QMARY HLYK BIRM PNSLA LVRPL KEELE CAM OXF

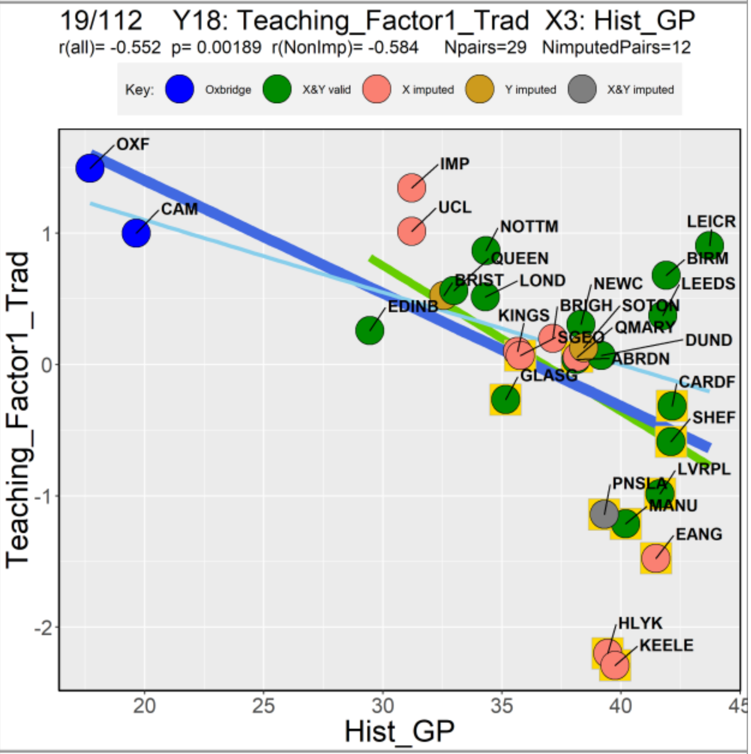
19/112 Y18: Teaching_Factor1_Trad X3: Hist_GP
r(all)= -0.552 p= 0.00189 r(NonImp)= -0.584 Npairs=29 NimputedPairs=12
Key: Oxbridge X&Y valid X imputed Y imputed X&Y imputed
Teaching_Factor1_Trad
Hist_GP
OXF IMP CAM UCL NOTTM LEICR QUEEN BIRM BRIST LOND NEWC LEEDS EDINB KINGS BRIGH SOTON SGEO QMARY DUND ABRDN GLASG CARDF SHEF LVRPL PNSLA MANU EANG HLYK KEELE

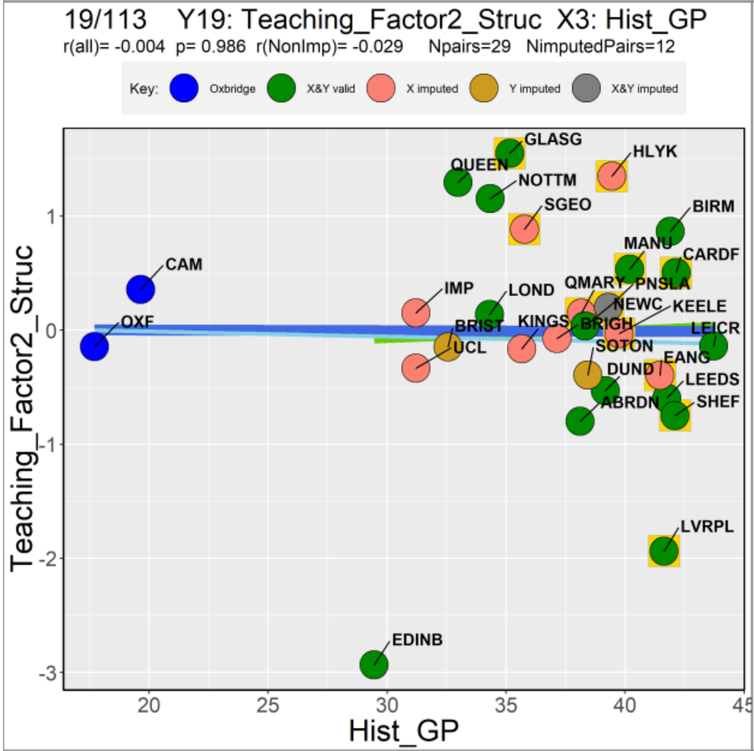
19/113 Y19: Teaching_Factor2_Struc X3: Hist_GP
r(all)= -0.004 p= 0.986 r(NonImp)= -0.029 Npairs=29 NimputedPairs=12
Key: Oxbridge X&Y valid X imputed Y imputed X&Y imputed
Teaching_Factor2_Struc
Hist_GP
GLASG HLYK QUEEN NOTTM SGEO BIRM MANU CARDF CAM IMP LOND QMARY PNSLA NEWC KEELE OXF BRIST KINGS BRIGH LEICR UCL SOTON EANG DUND LEEDS ABRDN SHEF LVRPL EDINB

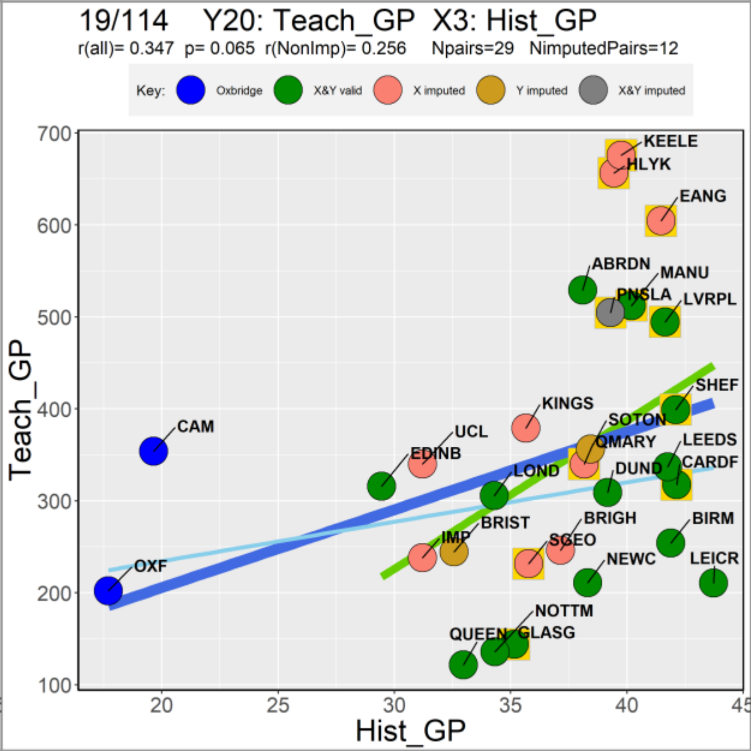
19/114 Y20: Teach_GP X3: Hist_GP
r(all)= 0.347 p= 0.065 r(NonImp)= 0.256 Npairs=29 NimputedPairs=12
Key: Oxbridge X&Y valid X imputed Y imputed X&Y imputed
Teach_GP
Hist_GP
KEELE HLYK EANG ABRDN MANU PNSLA LVRPL SHEF KINGS SOTON CAM UCL QMARY LEEDS EDINB LOND DUND CARDF BRIST BRIGH BIRM IMP SGEO NEWC LEICR OXF NOTTM QUEEN GLASG

20/115 Y21: Teach_Psyc X3: Hist_GP
r(all)= -0.354 p= 0.0595 r(NonImp)= -0.365 Npairs=29 NimputedPairs=12

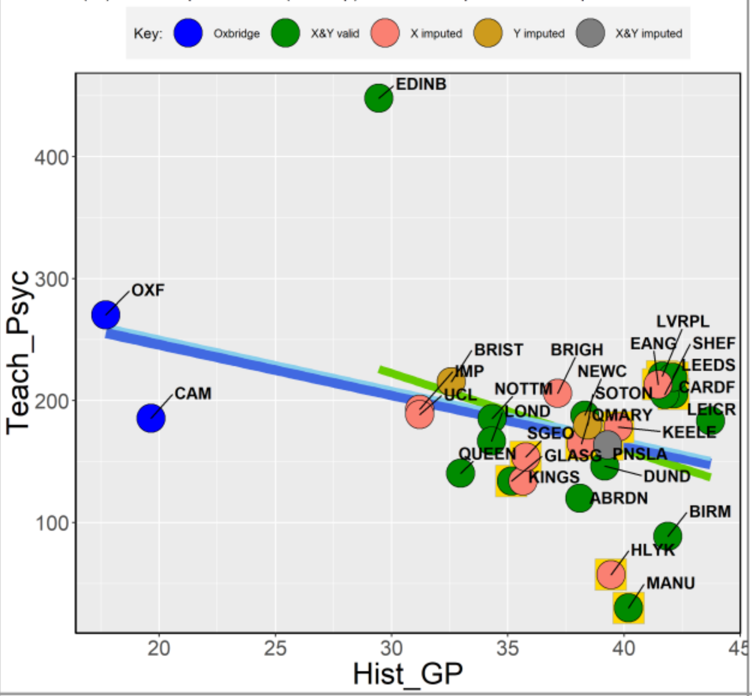

20/116 Y22: Teach_Anaes X3: Hist_GP
r(all)= 0.037 p= 0.847 r(NonImp)= 0.183 Npairs=29 NimputedPairs=12

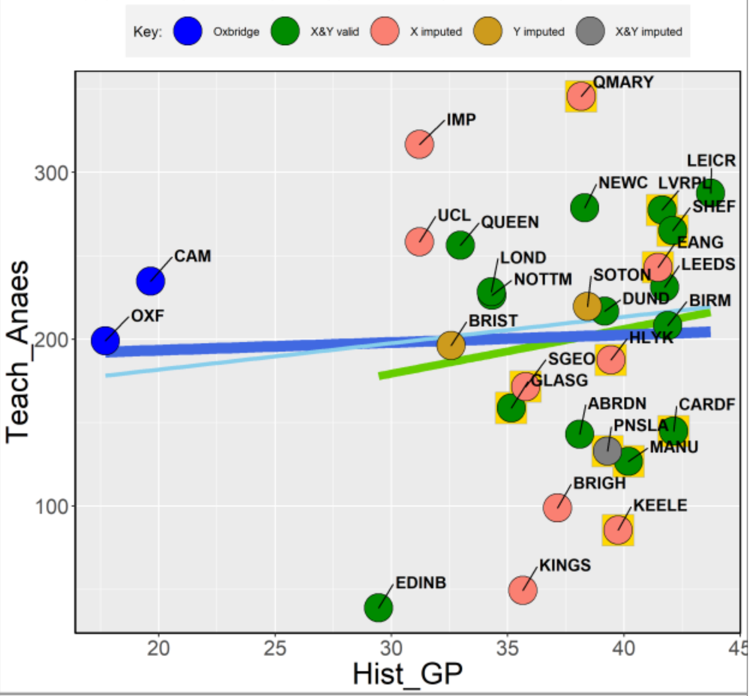

20/117 Y23: Teach_OG X3: Hist_GP
r(all)= -0.333 p= 0.0777 r(NonImp)= -0.247 Npairs=29 NimputedPairs=12

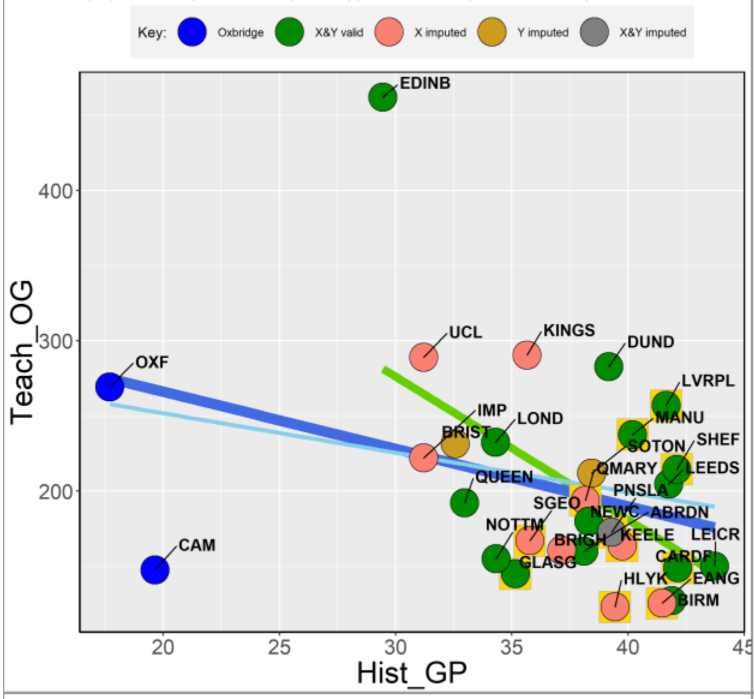

20/118 Y24: Teach_IntMed X3: Hist_GP
r(all)= -0.317 p= 0.0935 r(NonImp)= -0.304 Npairs=29 NimputedPairs=12

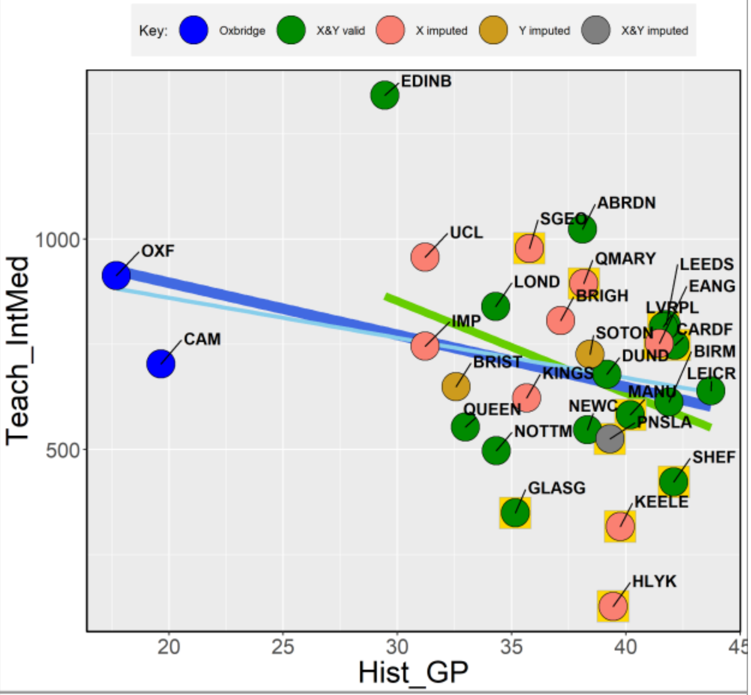

20/119 Y25: Teach_Surgery X3: Hist_GP
r(all)= -0.625 p= 0.000292 r(NonImp)= -0.672 Npairs=29 NimputedPairs=12

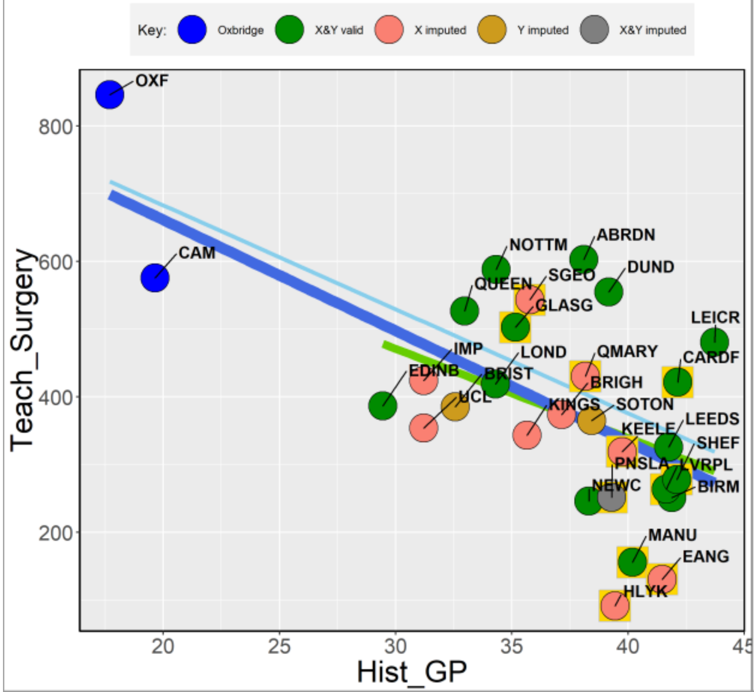

20/120 Y26: ExamTime X3: Hist_GP
r(all)= -0.548 p= 0.0021 r(NonImp)= -0.642 Npairs=29 NimputedPairs=11

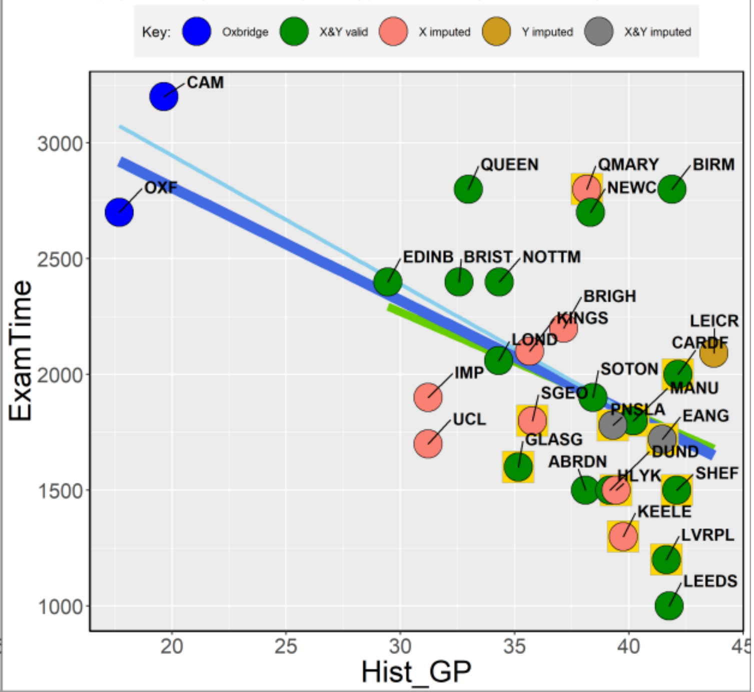

21/121 Y27: SelfRegLearn X3: Hist_GP

r(all)= -0.368 p= 0.0497 r(NonImp)= -0.484 Npairs=29 NimputedPairs=10

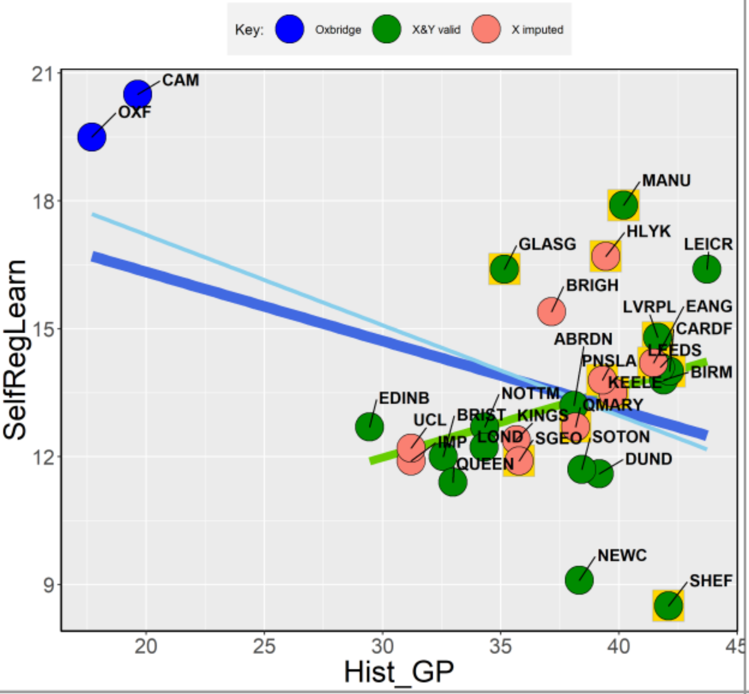

21/122 Y28: NSS_Satisfn X3: Hist_GP

r(all)= -0.265 p= 0.165 r(NonImp)= -0.382 Npairs=29 NimputedPairs=10

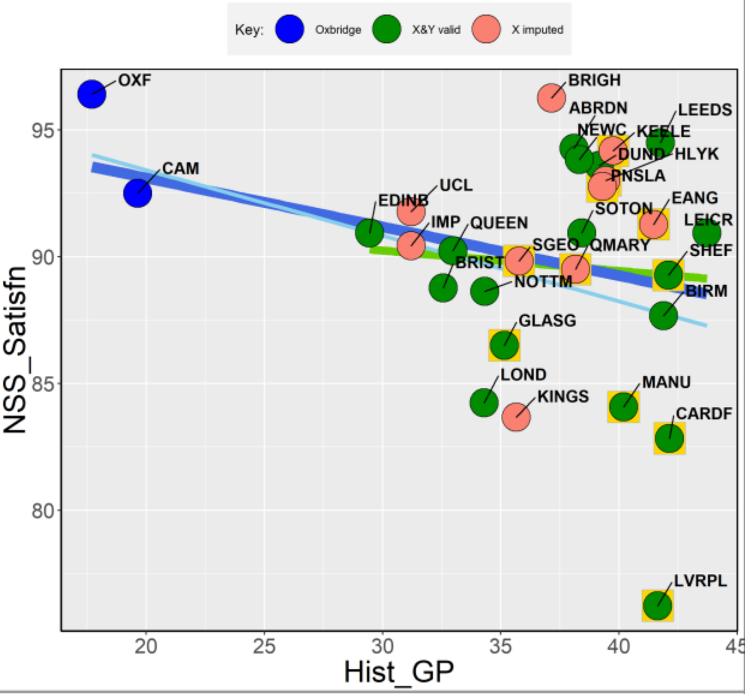

21/123 Y29: NSS_Feedback X3: Hist_GP

r(all)= -0.230 p= 0.229 r(NonImp)= -0.415 Npairs=29 NimputedPairs=10

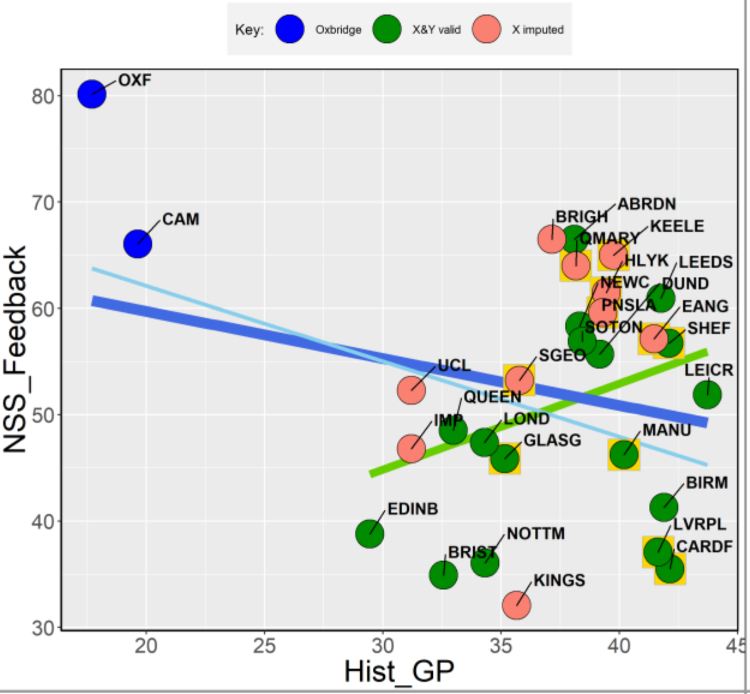

21/124 Y30: UKFPO_EPM X3: Hist_GP

r(all)= -0.594 p= 0.000688 r(NonImp)= -0.618 Npairs=29 NimputedPairs=10

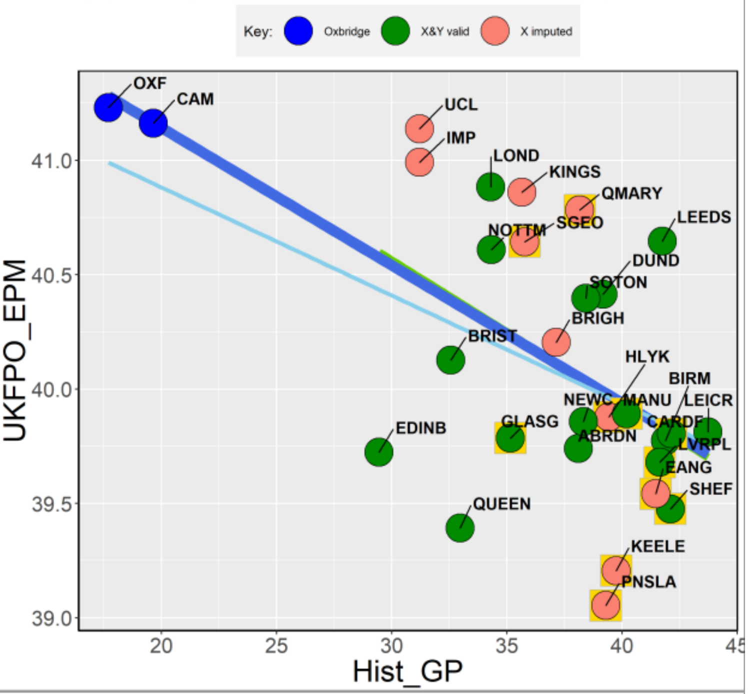

21/125 Y31: UKFPO_SJT X3: Hist_GP

r(all)= -0.772 p= 9.18e-07 r(NonImp)= -0.768 Npairs=29 NimputedPairs=10

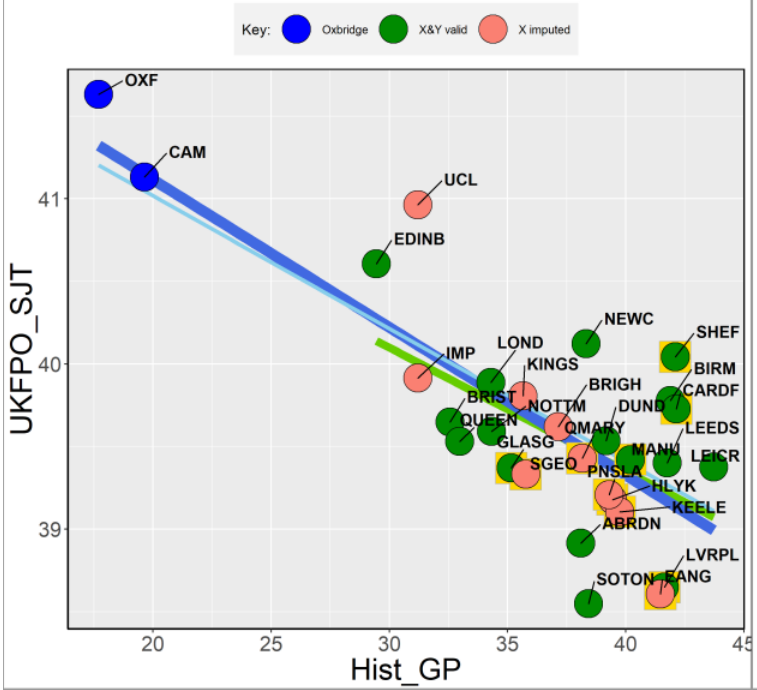

21/126 Y32: F1_Preparedness X3: Hist_GP

r(all)= 0.196 p= 0.307 r(NonImp)= 0.036 Npairs=29 NimputedPairs=10

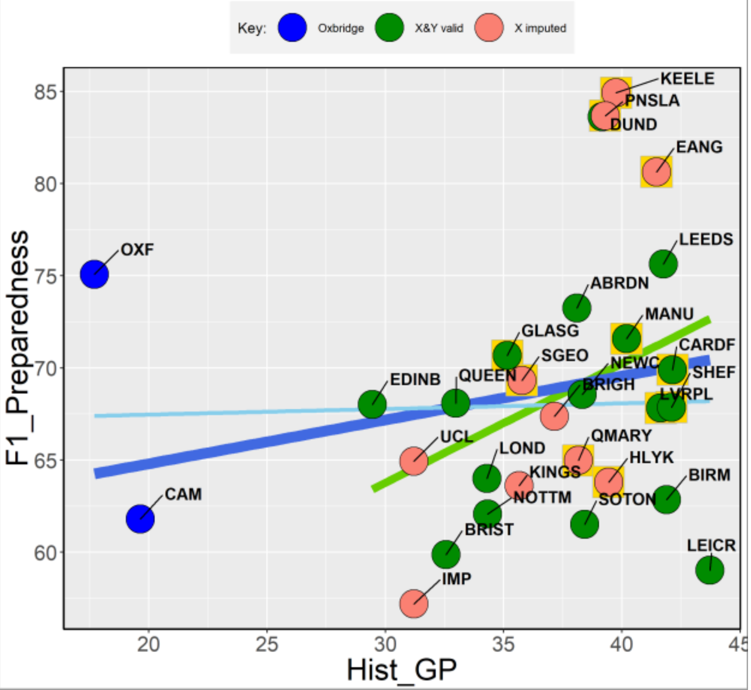

22/127 Y33: F1_Satisfn X3: Hist_GP
r(all)= 0.589 p= 0.000766 r(NonImp)= 0.638 Npairs=29 NimputedPairs=10

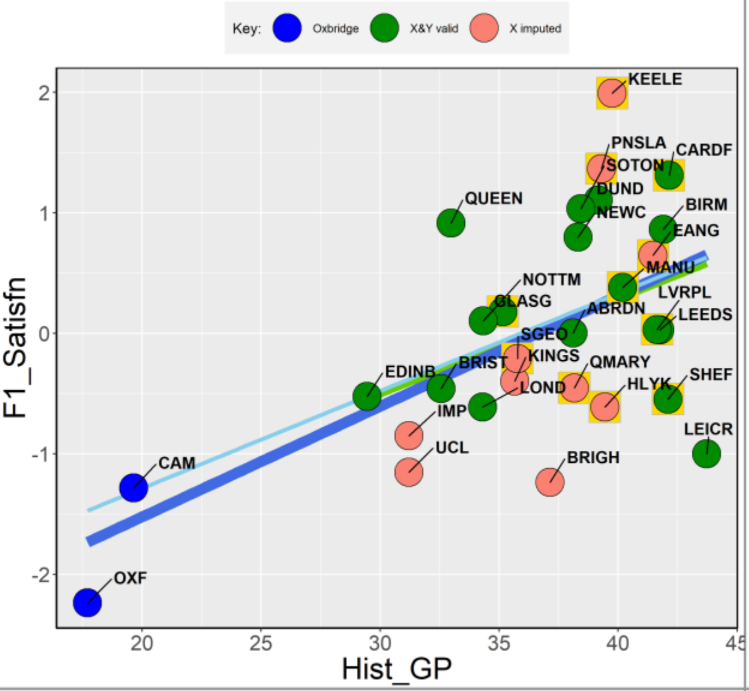

22/128 Y34: F1_Workload X3: Hist_GP
r(all)= 0.265 p= 0.164 r(NonImp)= 0.270 Npairs=29 NimputedPairs=10

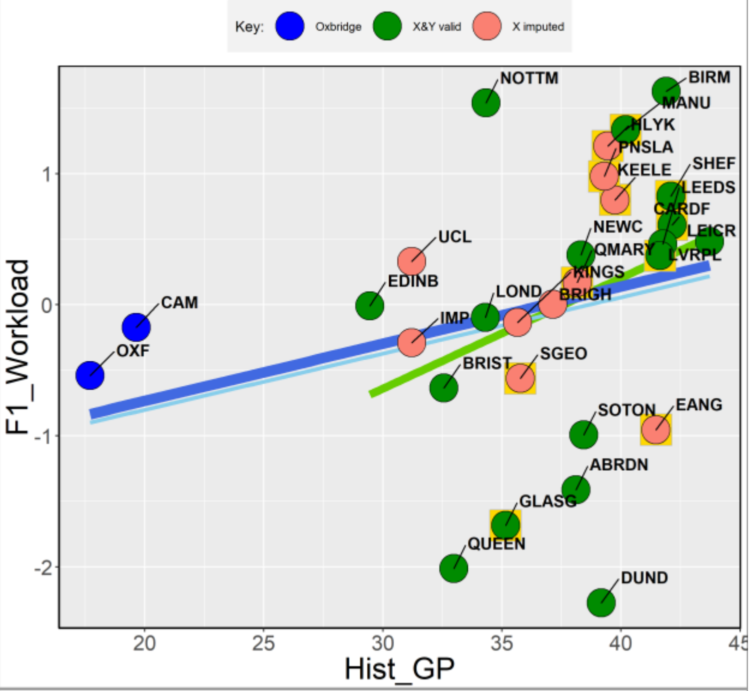

22/129 Y35: F1_Supervn X3: Hist_GP
r(all)= -0.366 p= 0.0506 r(NonImp)= -0.410 Npairs=29 NimputedPairs=10

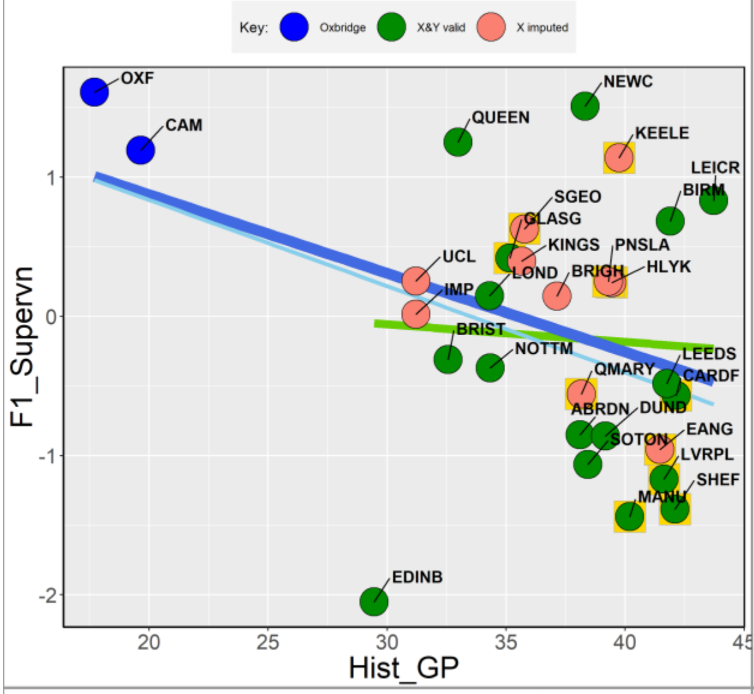

22/130 Y36: Trainee_GP X3: Hist_GP
r(all)= 0.614 p= 0.000397 r(NonImp)= 0.683 Npairs=29 NimputedPairs=10

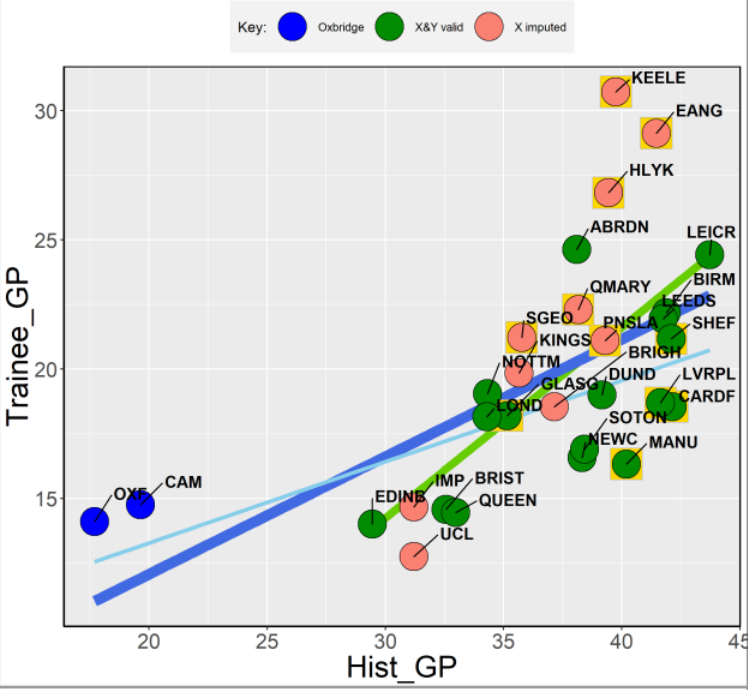

22/131 Y37: Trainee_Psyc X3: Hist_GP
r(all)= 0.002 p= 0.991 r(NonImp)= -0.100 Npairs=29 NimputedPairs=10

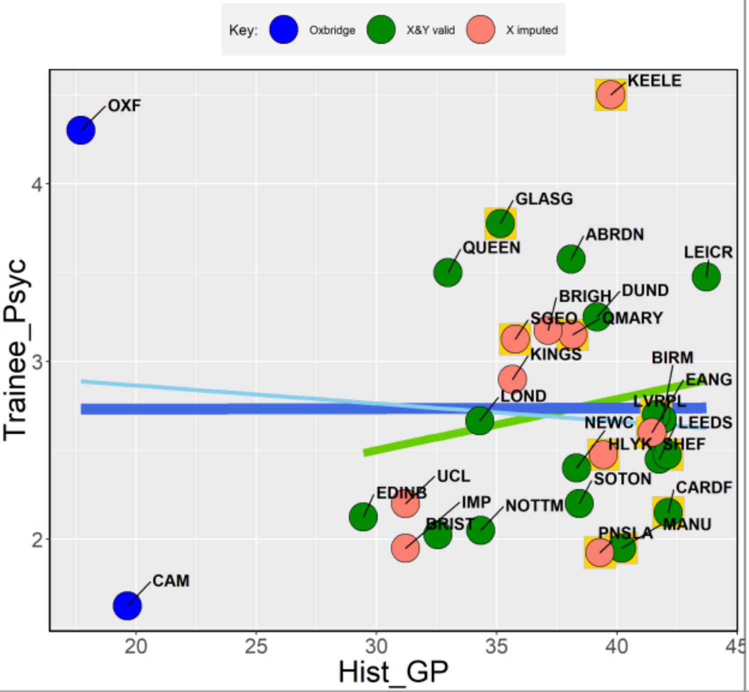

22/132 Y38: TraineeApp_Surgery X3: Hist_GP
r(all)= -0.264 p= 0.166 r(NonImp)= -0.190 Npairs=29 NimputedPairs=10

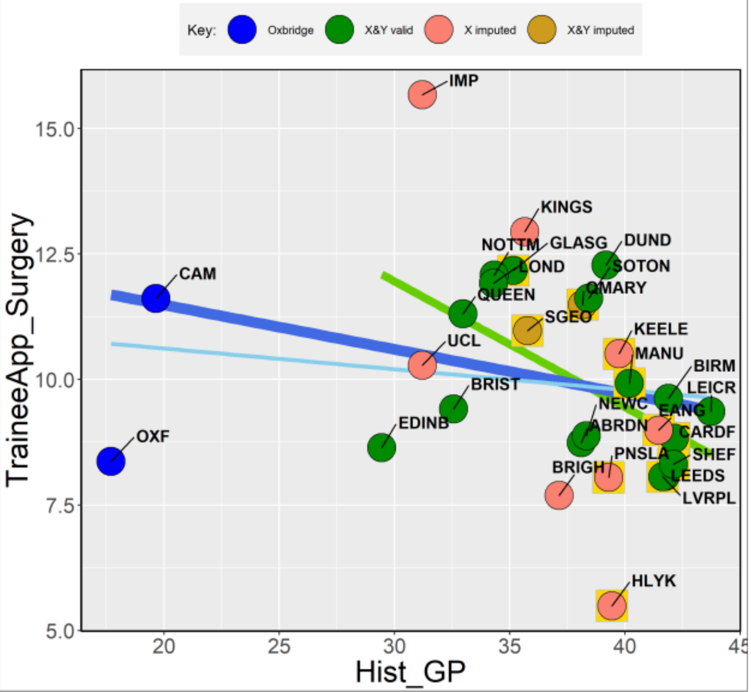

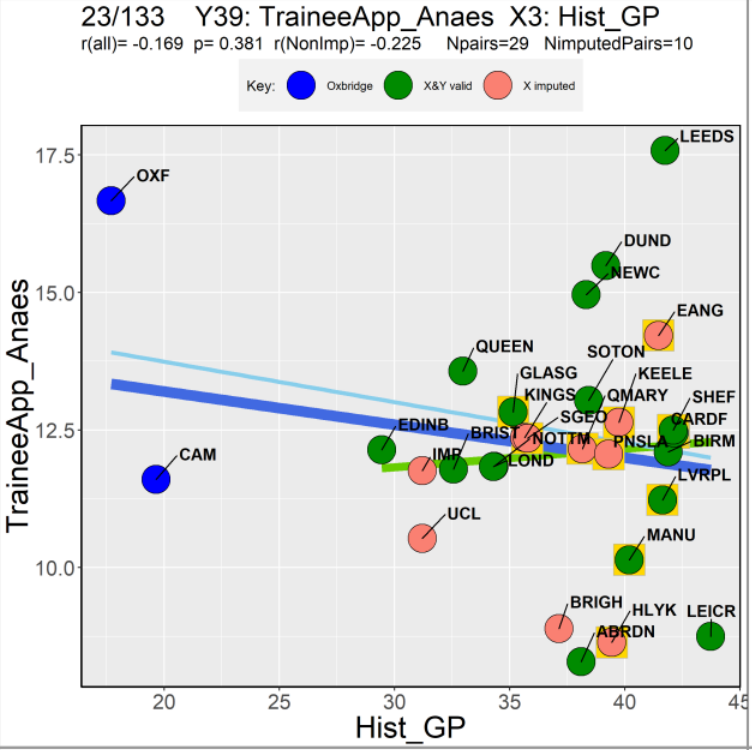
23/133 Y39: TraineeApp_Anaes X3: Hist_GP
r(all)= -0.169 p= 0.381 r(NonImp)= -0.225 Npairs=29 NimputedPairs=10
Key: Oxbridge X&Y valid X imputed
TraineeApp_Anaes
Hist_GP

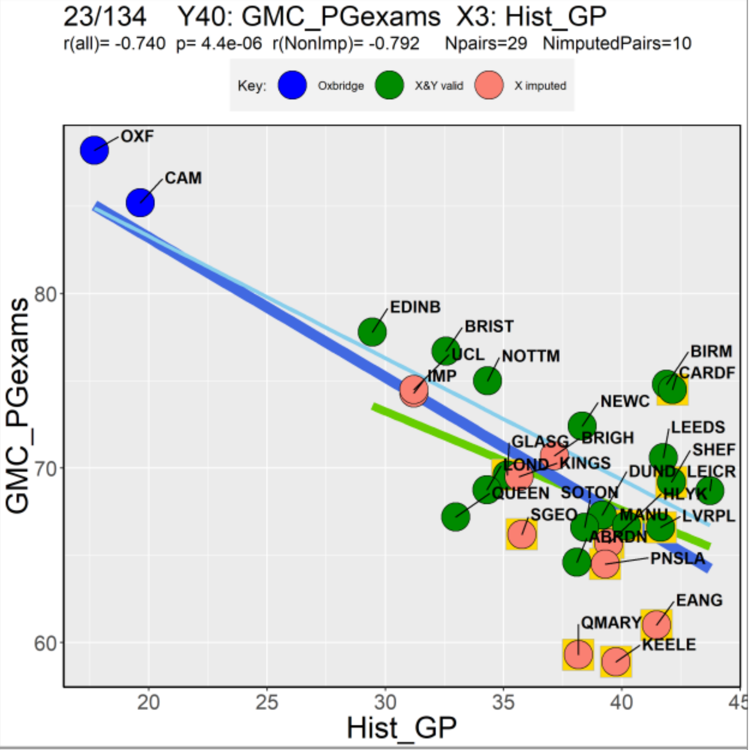
23/134 Y40: GMC_PGexams X3: Hist_GP
r(all)= -0.740 p= 4.4e-06 r(NonImp)= -0.792 Npairs=29 NimputedPairs=10
Key: Oxbridge X&Y valid X imputed
GMC_PGexams
Hist_GP

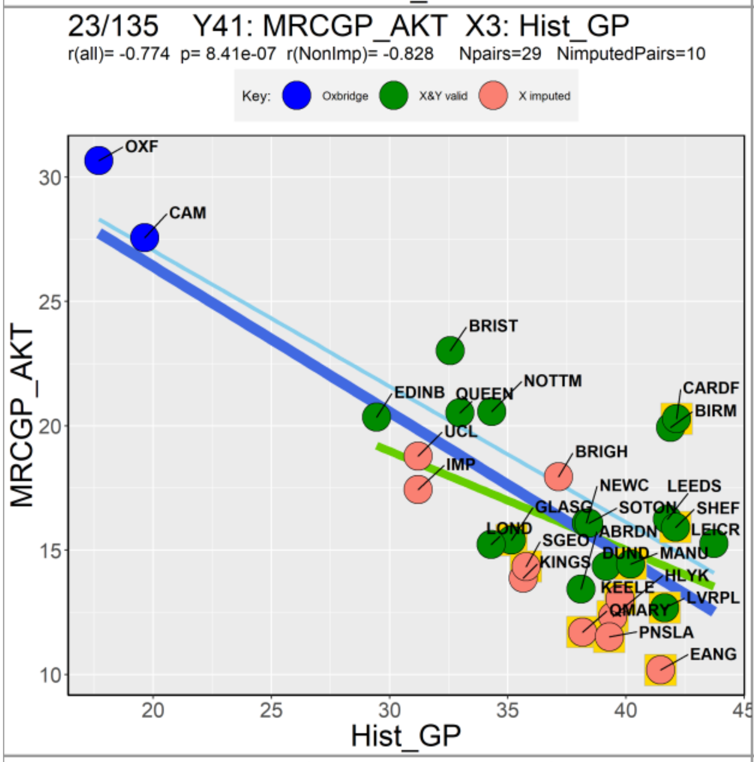
23/135 Y41: MRCGP_AKT X3: Hist_GP
r(all)= -0.774 p= 8.41e-07 r(NonImp)= -0.828 Npairs=29 NimputedPairs=10
Key: Oxbridge X&Y valid X imputed
MRCGP_AKT
Hist_GP

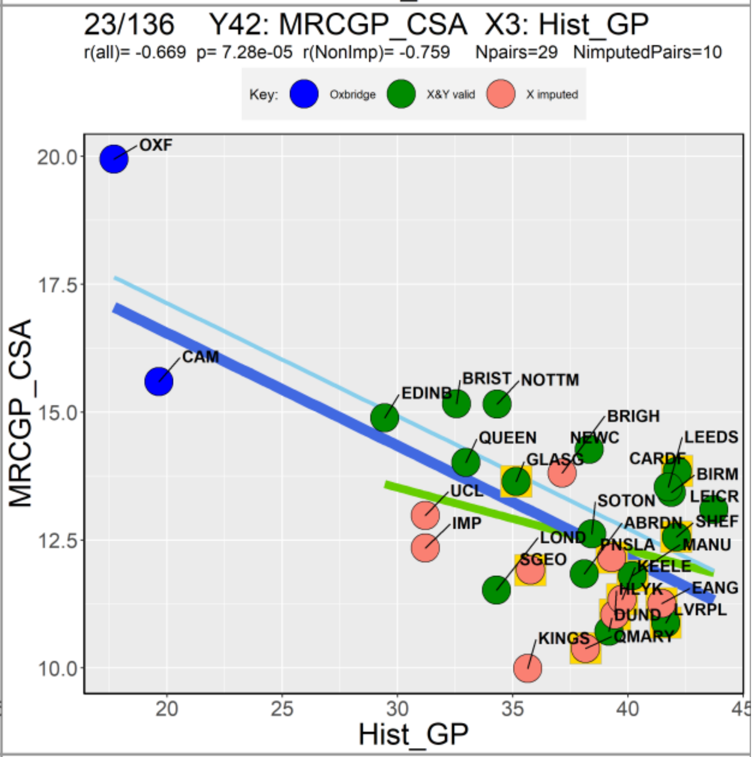
23/136 Y42: MRCGP_CSA X3: Hist_GP
r(all)= -0.669 p= 7.28e-05 r(NonImp)= -0.759 Npairs=29 NimputedPairs=10
Key: Oxbridge X&Y valid X imputed
MRCGP_CSA
Hist_GP

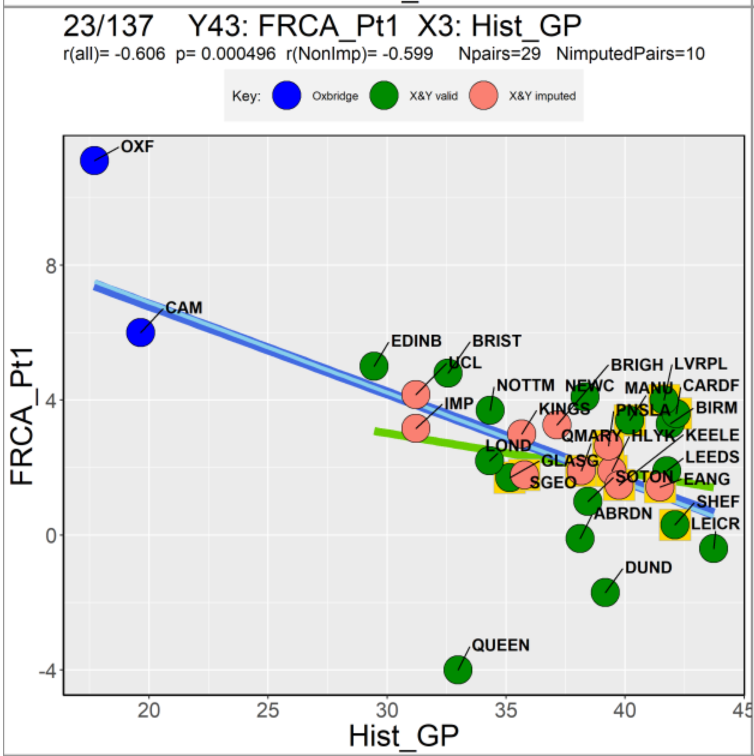
23/137 Y43: FRCA_Pt1 X3: Hist_GP
r(all)= -0.606 p= 0.000496 r(NonImp)= -0.599 Npairs=29 NimputedPairs=10
Key: Oxbridge X&Y valid X&Y imputed
FRCA_Pt1
Hist_GP

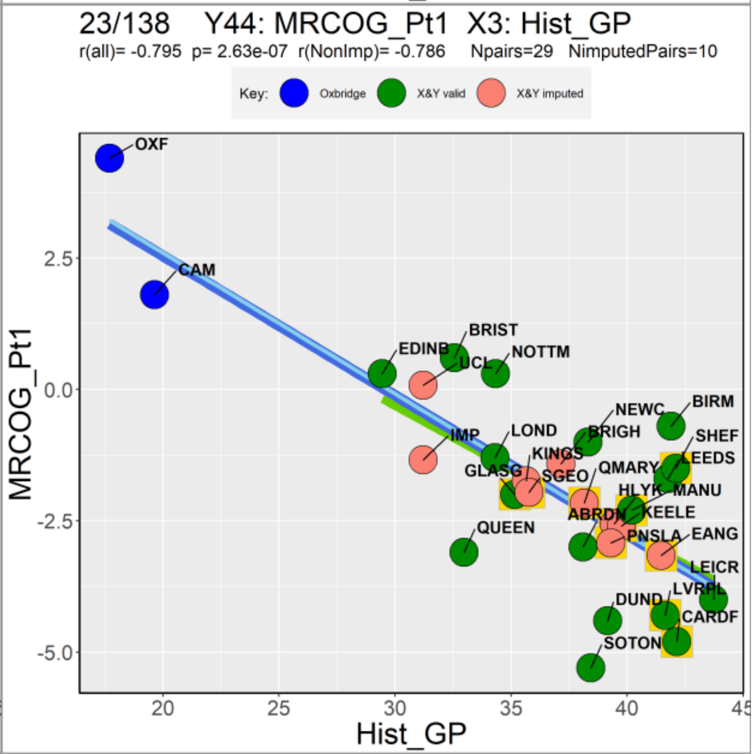
23/138 Y44: MRCOG_Pt1 X3: Hist_GP
r(all)= -0.795 p= 2.63e-07 r(NonImp)= -0.786 Npairs=29 NimputedPairs=10
Key: Oxbridge X&Y valid X&Y imputed
MRCOG_Pt1
Hist_GP

24/139 Y45: MRCOG_Pt2 X3: Hist_GP

r(all)= -0.671 p= 6.71e-05 r(NonImp)= -0.671 Npairs=29 NimputedPairs=10

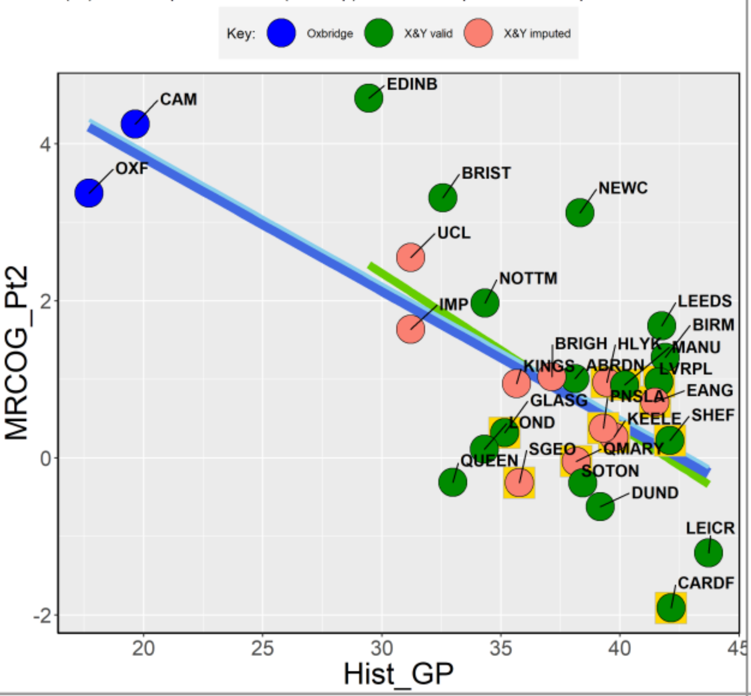

24/140 Y46: MRCP_Pt1 X3: Hist_GP

r(all)= -0.803 p= 1.56e-07 r(NonImp)= -0.831 Npairs=29 NimputedPairs=10

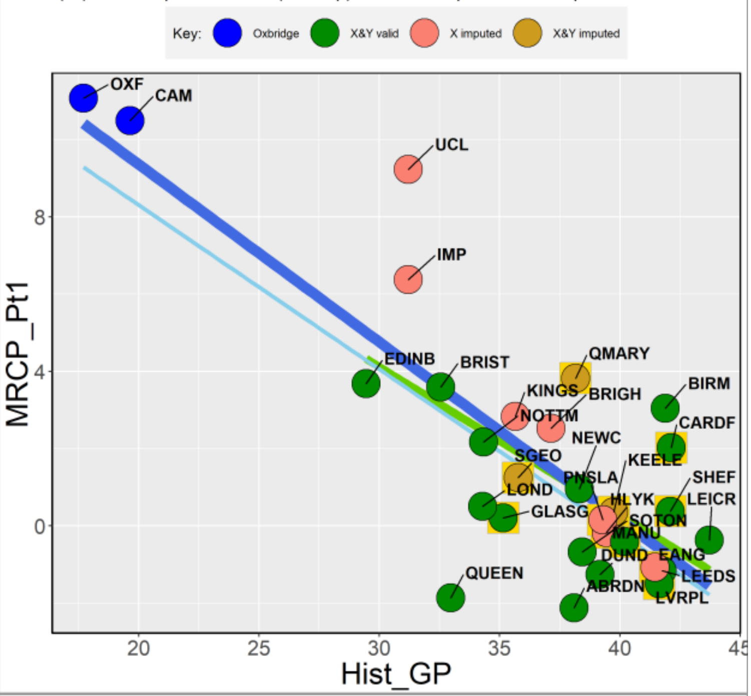

24/141 Y47: MRCP_Pt2 X3: Hist_GP

r(all)= -0.781 p= 5.91e-07 r(NonImp)= -0.822 Npairs=29 NimputedPairs=10

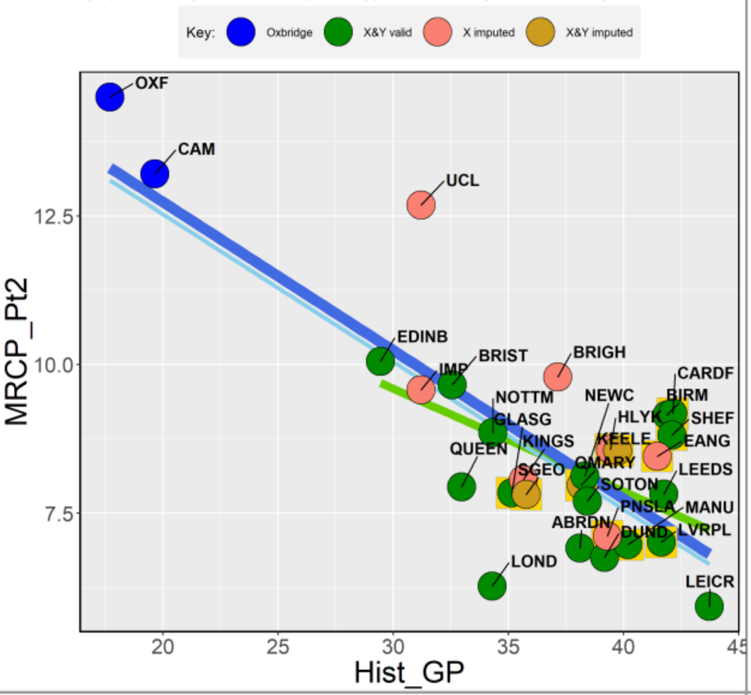

24/142 Y48: MRCP_PACES X3: Hist_GP

r(all)= -0.697 p= 2.66e-05 r(NonImp)= -0.672 Npairs=29 NimputedPairs=10

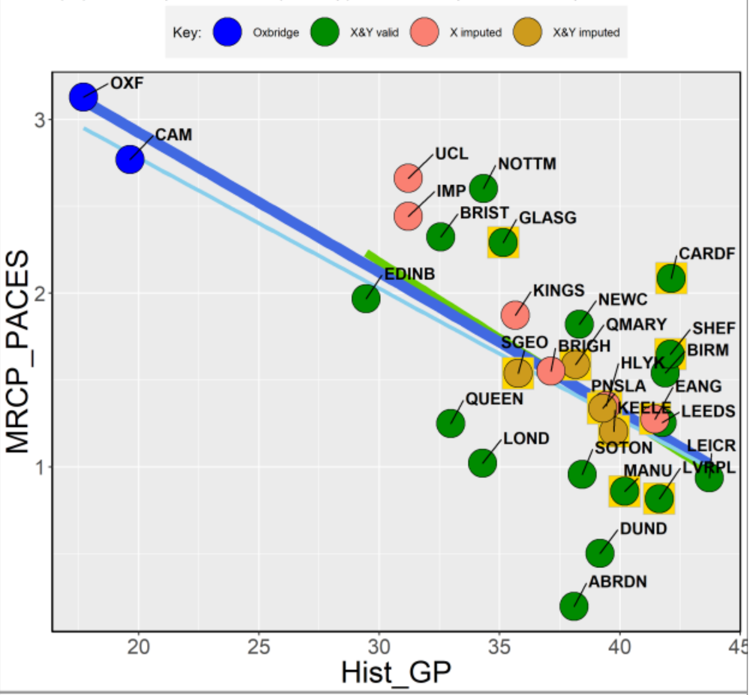

24/143 Y49: GMC_Sanctions X3: Hist_GP

r(all)= 0.655 p= 0.000115 r(NonImp)= 0.618 Npairs=29 NimputedPairs=10

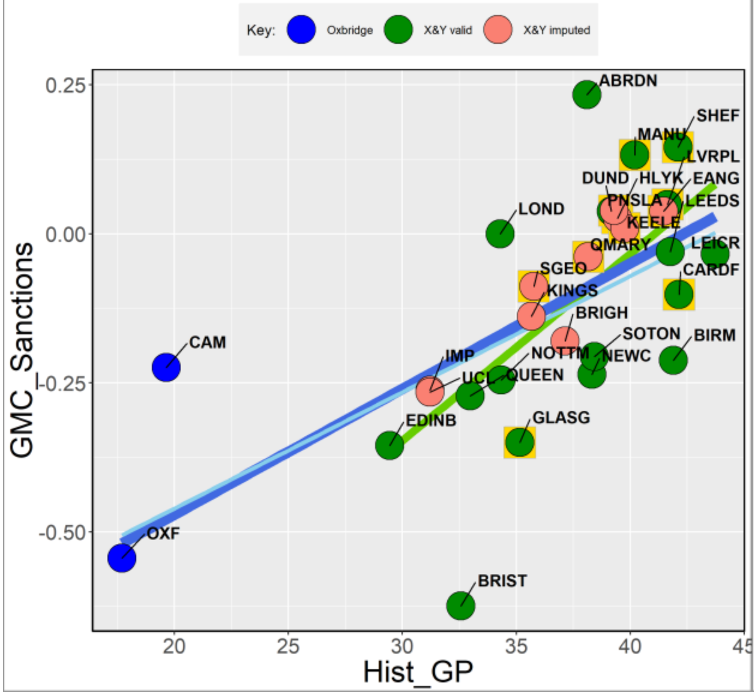

24/144 Y50: ARCP_NotExam X3: Hist_GP

r(all)= 0.637 p= 0.000201 r(NonImp)= 0.611 Npairs=29 NimputedPairs=10

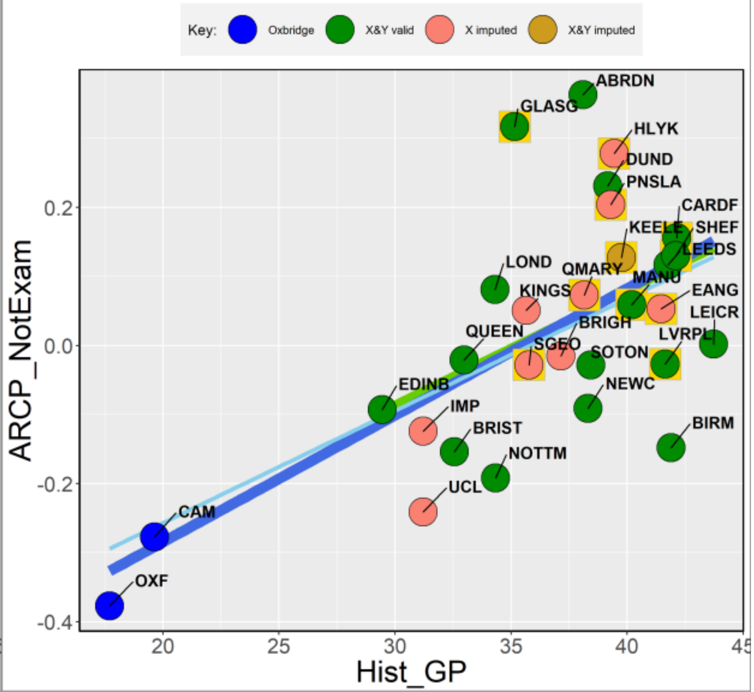

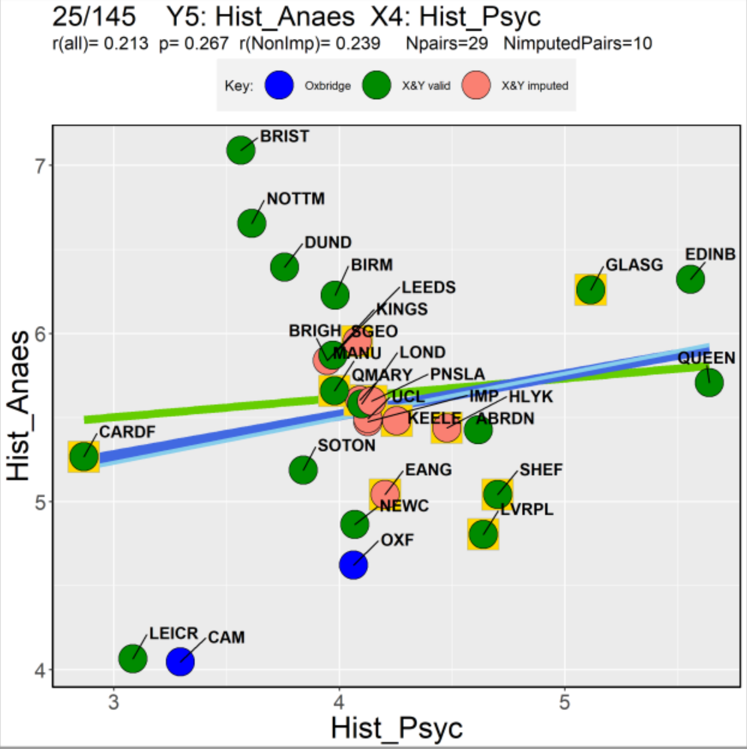
25/145 Y5: Hist_Anaes X4: Hist_Psyc
r(all)= 0.213 p= 0.267 r(NonImp)= 0.239 Npairs=29 NimputedPairs=10
Key: Oxbridge X&Y valid X&Y imputed
Hist_Anaes
Hist_Psyc

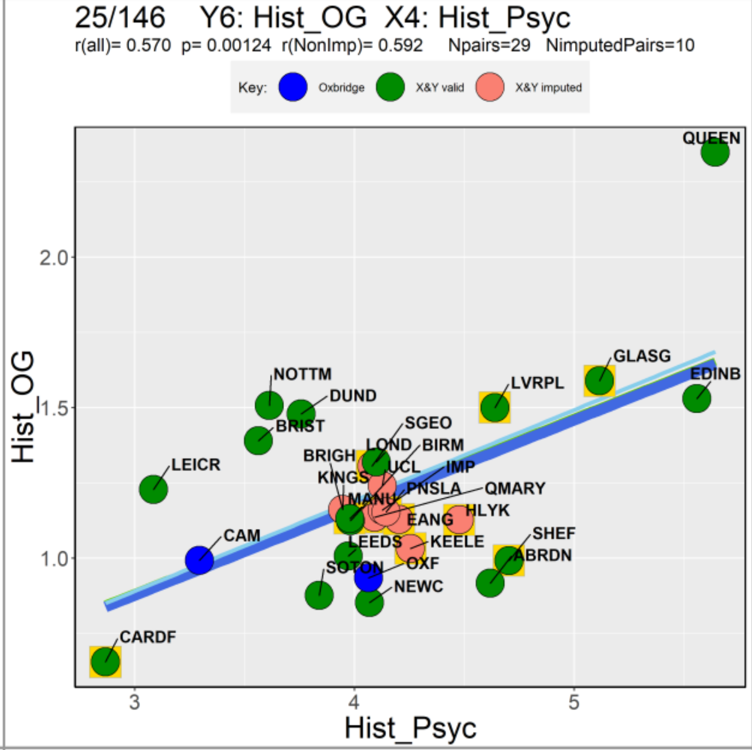
25/146 Y6: Hist_OG X4: Hist_Psyc
r(all)= 0.570 p= 0.00124 r(NonImp)= 0.592 Npairs=29 NimputedPairs=10
Key: Oxbridge X&Y valid X&Y imputed
Hist_OG
Hist_Psyc

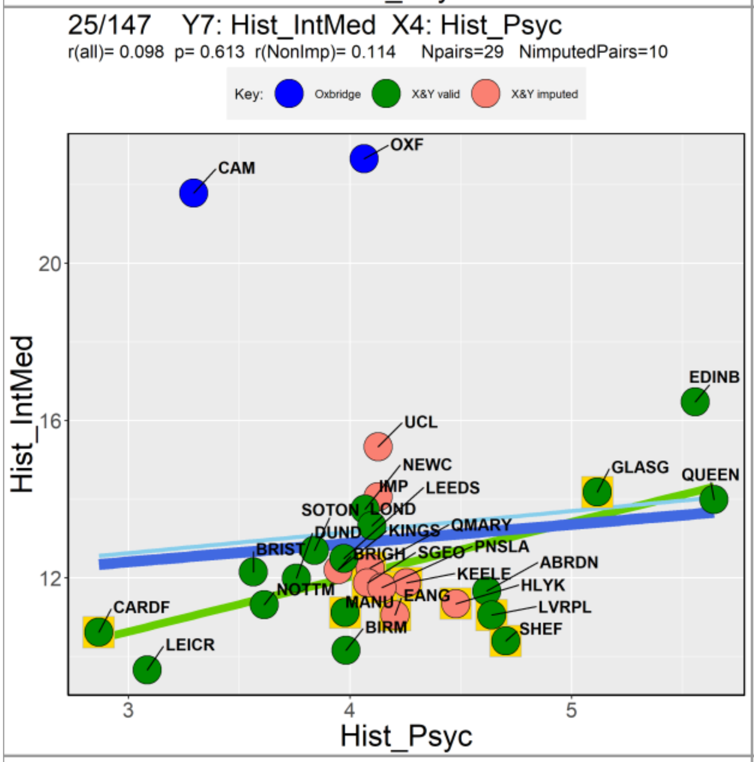
25/147 Y7: Hist_IntMed X4: Hist_Psyc
r(all)= 0.098 p= 0.613 r(NonImp)= 0.114 Npairs=29 NimputedPairs=10
Key: Oxbridge X&Y valid X&Y imputed
Hist_IntMed
Hist_Psyc

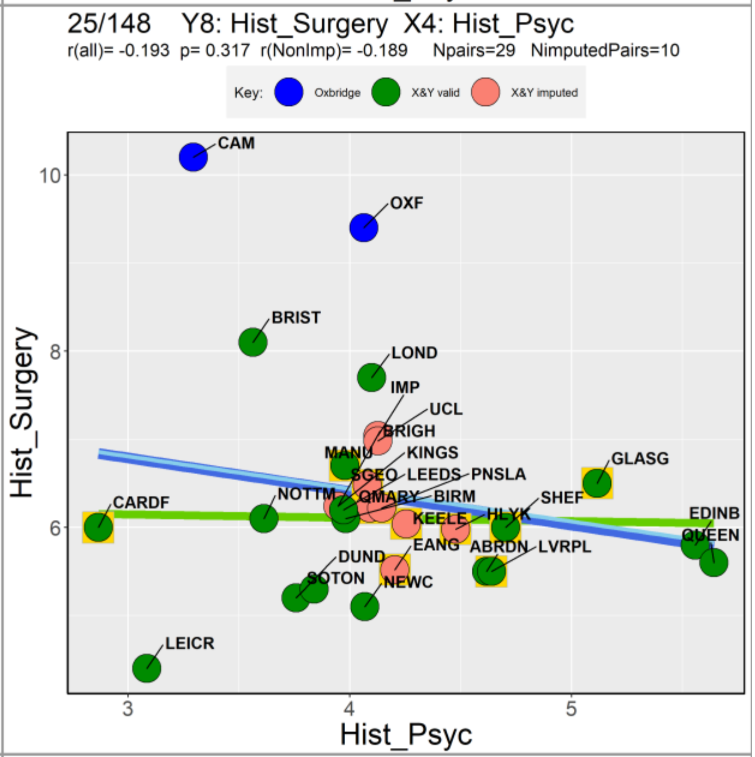
25/148 Y8: Hist_Surgery X4: Hist_Psyc
r(all)= -0.193 p= 0.317 r(NonImp)= -0.189 Npairs=29 NimputedPairs=10
Key: Oxbridge X&Y valid X&Y imputed
Hist_Surgery
Hist_Psyc

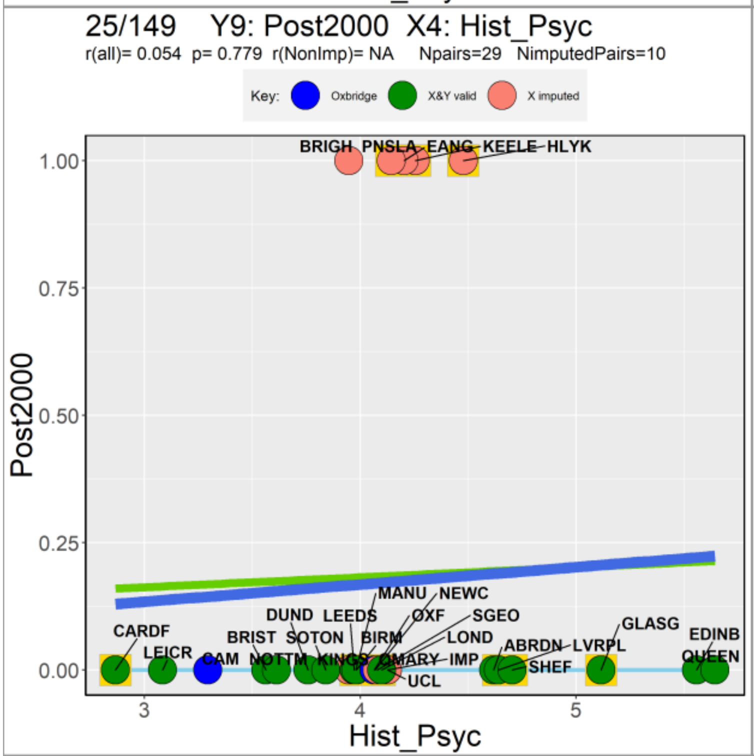
25/149 Y9: Post2000 X4: Hist_Psyc
r(all)= 0.054 p= 0.779 r(NonImp)= NA Npairs=29 NimputedPairs=10
Key: Oxbridge X&Y valid X imputed
Post2000
Hist_Psyc

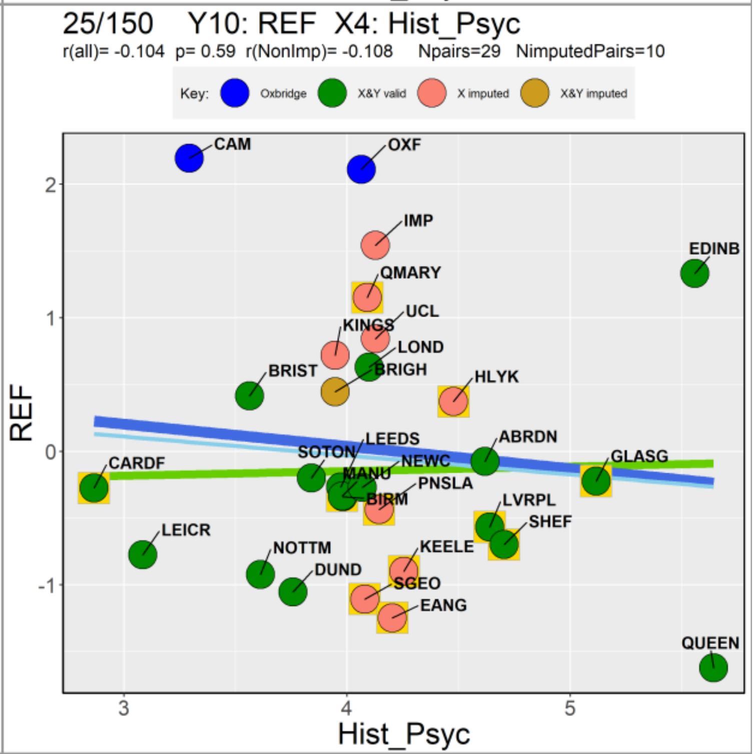
25/150 Y10: REF X4: Hist_Psyc
r(all)= -0.104 p= 0.59 r(NonImp)= -0.108 Npairs=29 NimputedPairs=10
Key: Oxbridge X&Y valid X imputed X&Y imputed
REF
Hist_Psyc

26/151 Y11: PBL_School X4: Hist_Psyc

r(all)= 0.128 p= 0.508 r(NonImp)= 0.106 Npairs=29 NimputedPairs=10

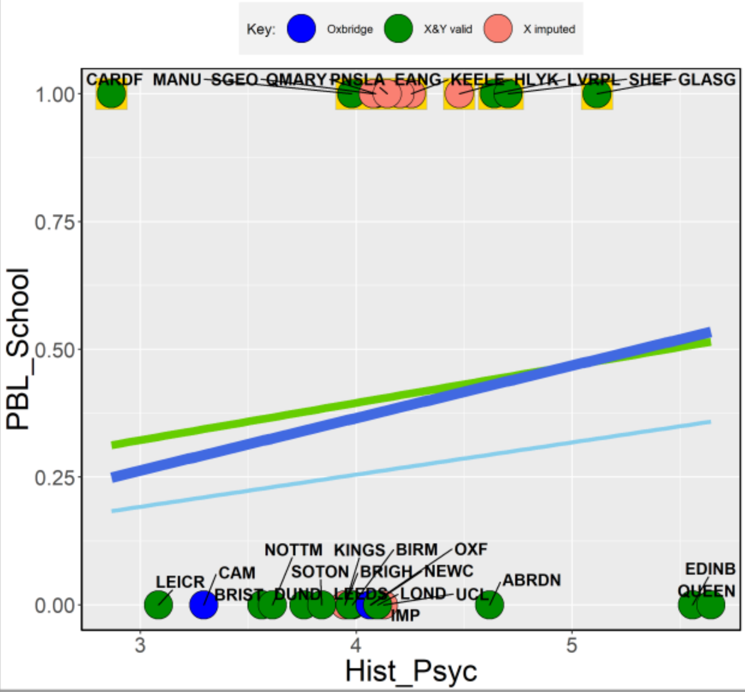

26/152 Y12: Spend_Student X4: Hist_Psyc

r(all)= -0.105 p= 0.589 r(NonImp)= -0.087 Npairs=29 NimputedPairs=10

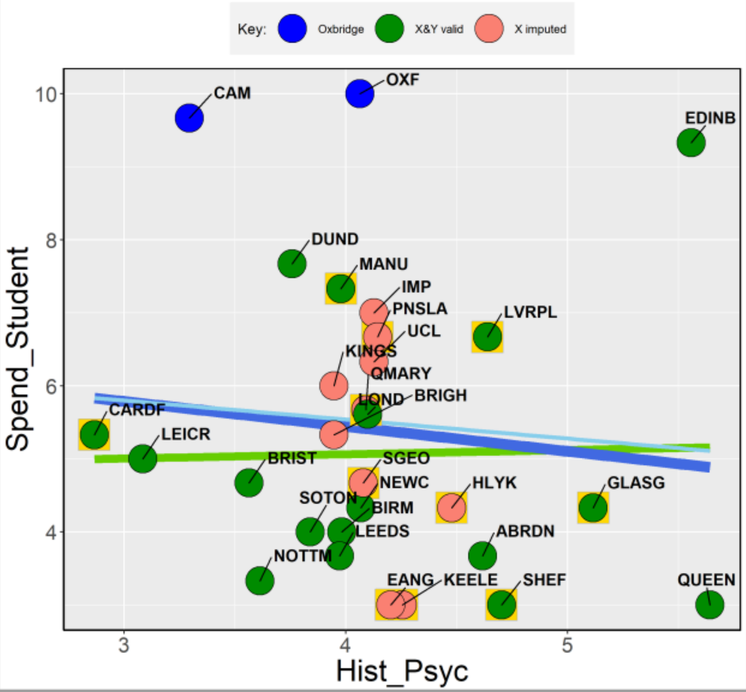

26/153 Y13: Student_Staff X4: Hist_Psyc

r(all)= 0.021 p= 0.915 r(NonImp)= 0.059 Npairs=29 NimputedPairs=10

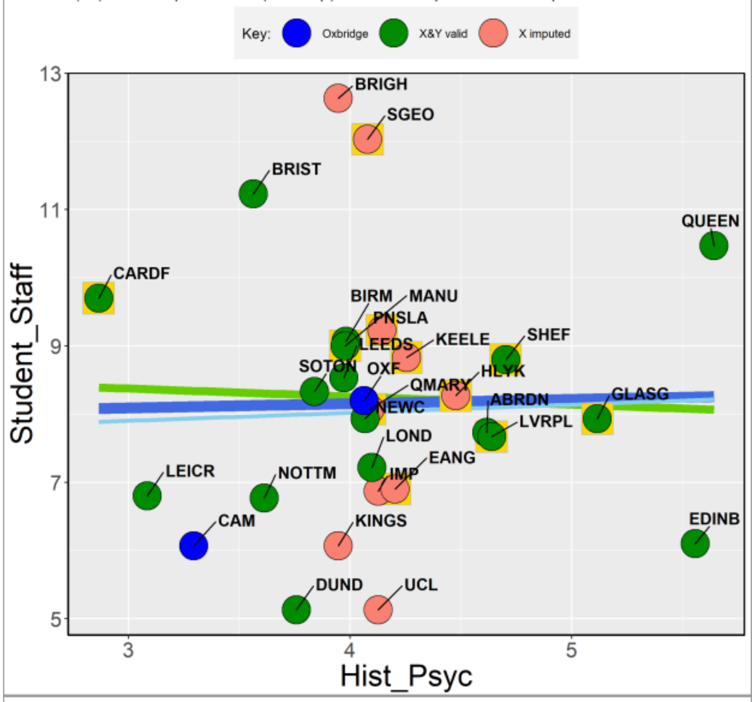

26/154 Y14: Entrants_N X4: Hist_Psyc

r(all)= -0.220 p= 0.252 r(NonImp)= -0.245 Npairs=29 NimputedPairs=10

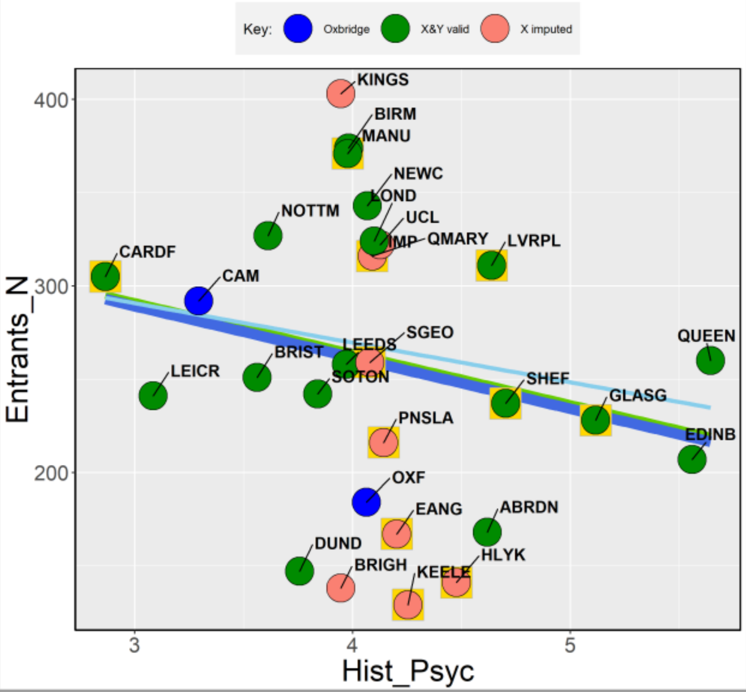

26/155 Y15: Entrants_Female X4: Hist_Psyc

r(all)= -0.076 p= 0.696 r(NonImp)= -0.105 Npairs=29 NimputedPairs=10

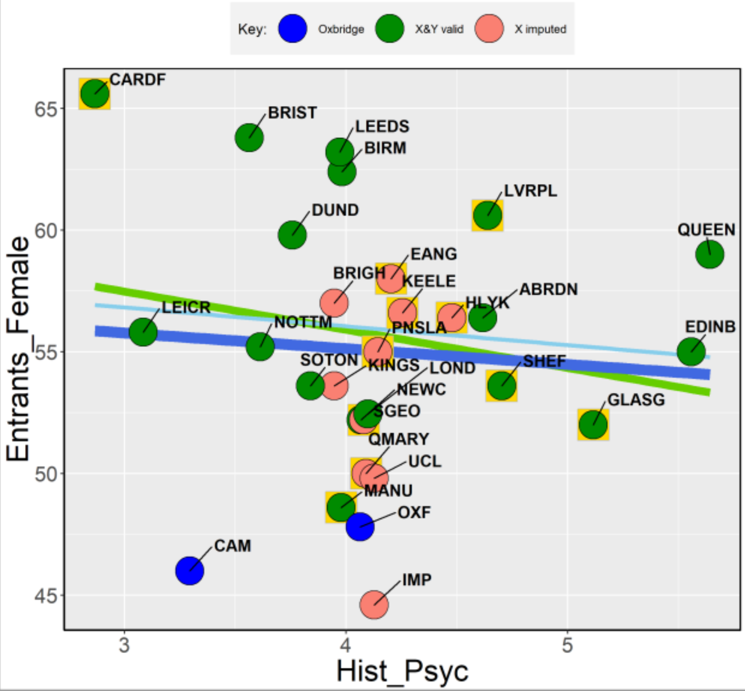

26/156 Y16: EntryGrades X4: Hist_Psyc

r(all)= 0.003 p= 0.988 r(NonImp)= 0.002 Npairs=29 NimputedPairs=10

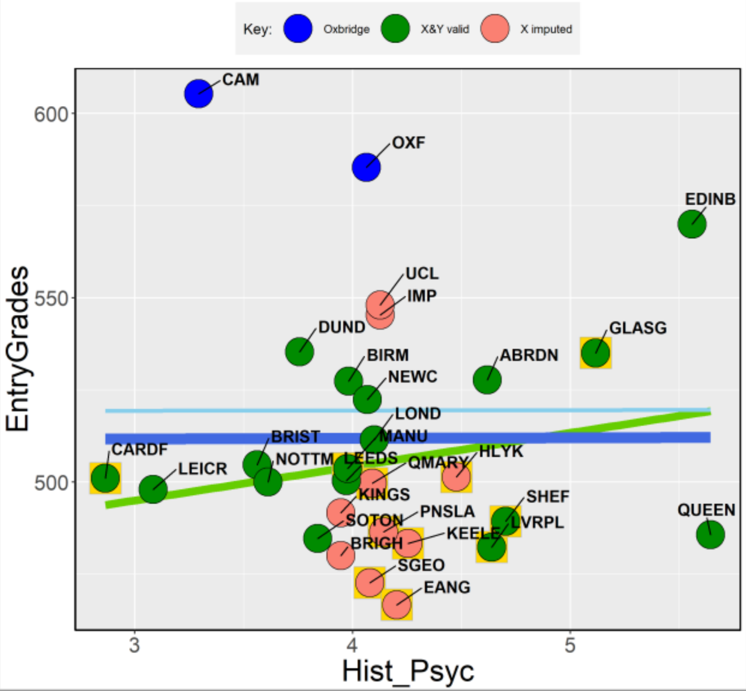

27/157 Y17: Entrants_NonHome X4: Hist_Psyc

r(all)= 0.363 p= 0.0531 r(NonImp)= 0.404 Npairs=29 NimputedPairs=10

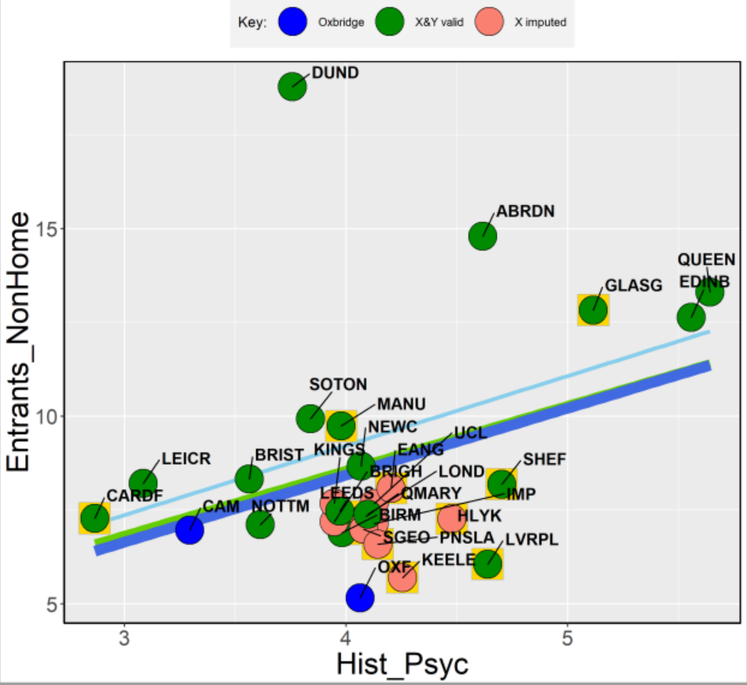

27/158 Y18: Teaching_Factor1_Trad X4: Hist_Psyc

r(all)= -0.206 p= 0.285 r(NonImp)= -0.221 Npairs=29 NimputedPairs=12

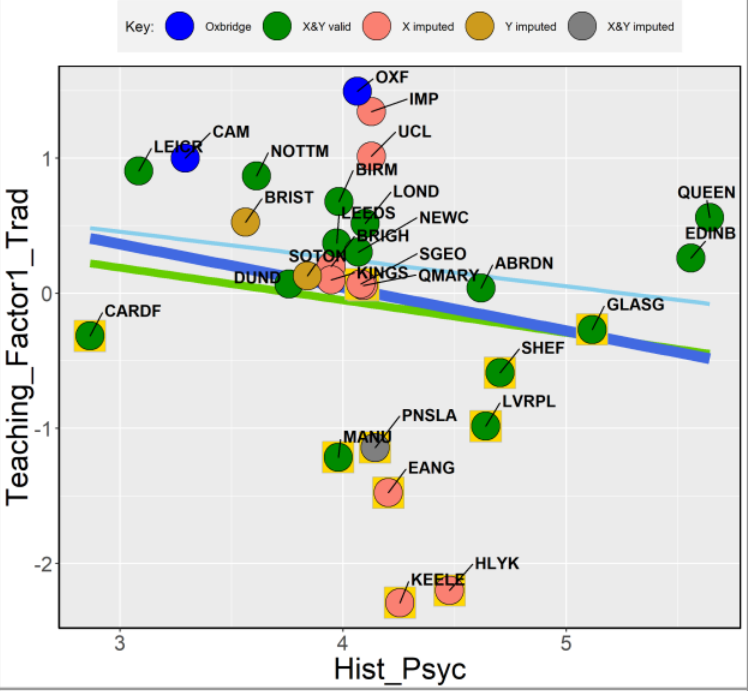

27/159 Y19: Teaching_Factor2_Struc X4: Hist_Psyc

r(all)= -0.198 p= 0.303 r(NonImp)= -0.259 Npairs=29 NimputedPairs=12

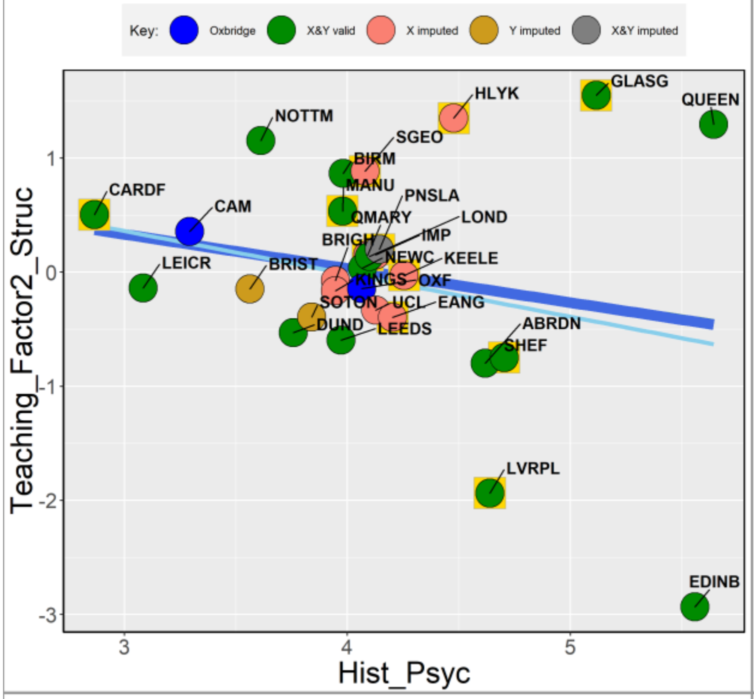

27/160 Y20: Teach_GP X4: Hist_Psyc

r(all)= 0.061 p= 0.751 r(NonImp)= -0.029 Npairs=29 NimputedPairs=12

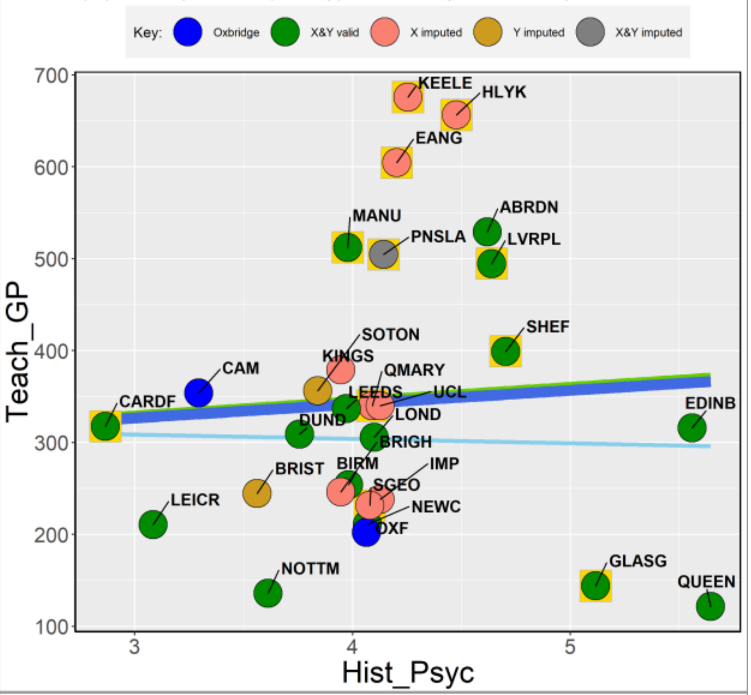

27/161 Y21: Teach_Psyc X4: Hist_Psyc

r(all)= 0.187 p= 0.332 r(NonImp)= 0.255 Npairs=29 NimputedPairs=12

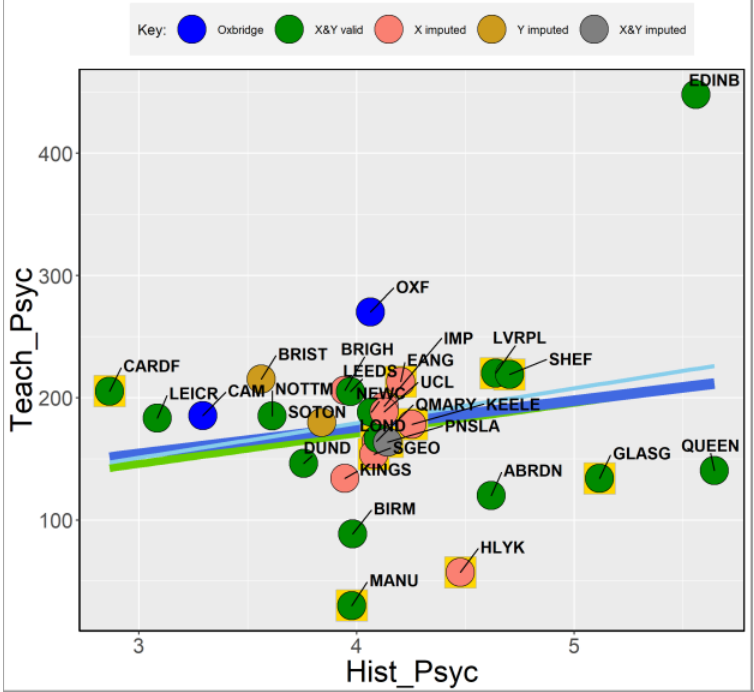

27/162 Y22: Teach_Anaes X4: Hist_Psyc

r(all)= -0.142 p= 0.462 r(NonImp)= -0.257 Npairs=29 NimputedPairs=12

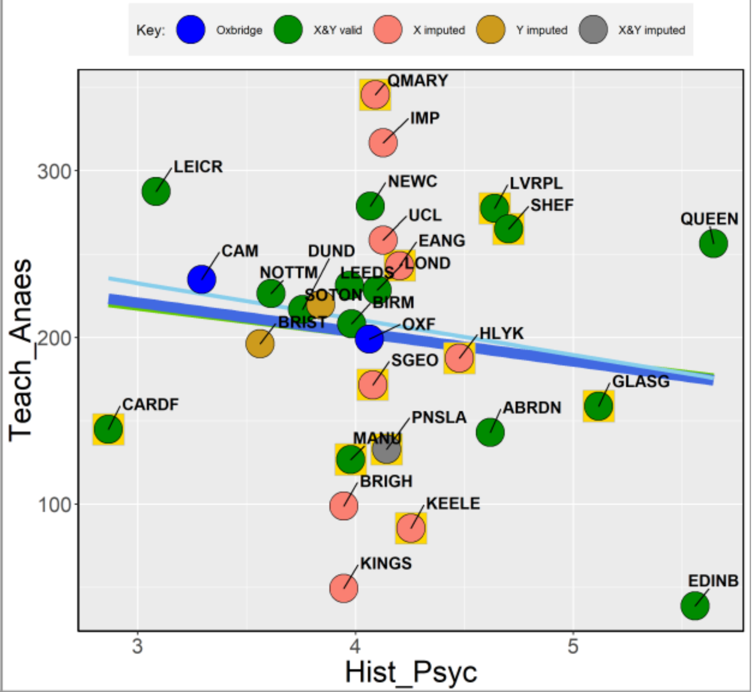

28/163 Y23: Teach_OG X4: Hist_Psyc

r(all)= 0.338 p= 0.0726 r(NonImp)= 0.461 Npairs=29 NimputedPairs=12

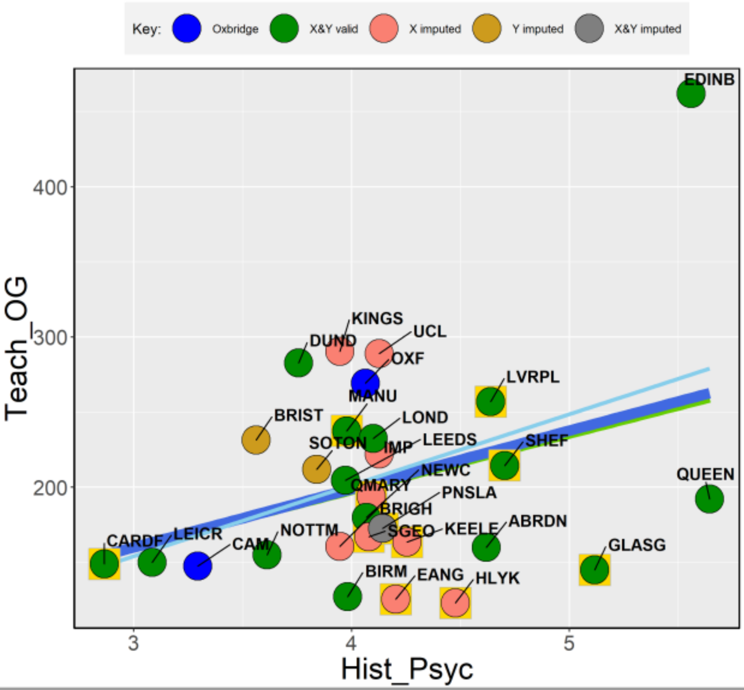

28/164 Y24: Teach_IntMed X4: Hist_Psyc

r(all)= 0.063 p= 0.744 r(NonImp)= 0.168 Npairs=29 NimputedPairs=12

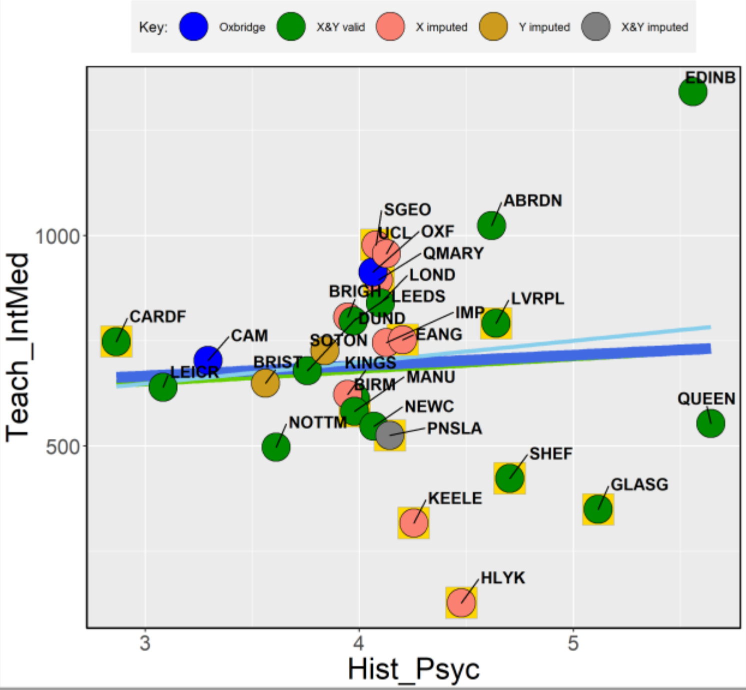

28/165 Y25: Teach_Surgery X4: Hist_Psyc

r(all)= -0.087 p= 0.654 r(NonImp)= -0.073 Npairs=29 NimputedPairs=12

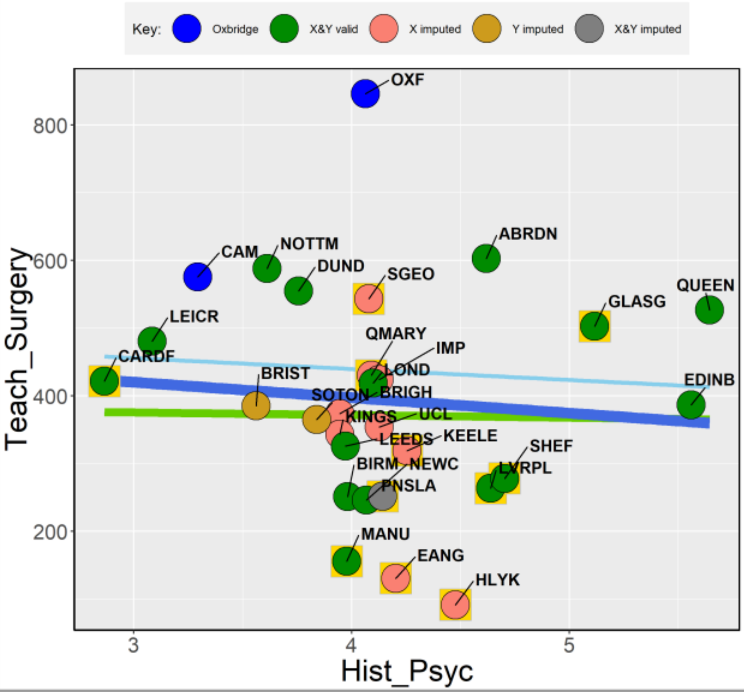

28/166 Y26: ExamTime X4: Hist_Psyc

r(all)= -0.132 p= 0.496 r(NonImp)= -0.112 Npairs=29 NimputedPairs=11

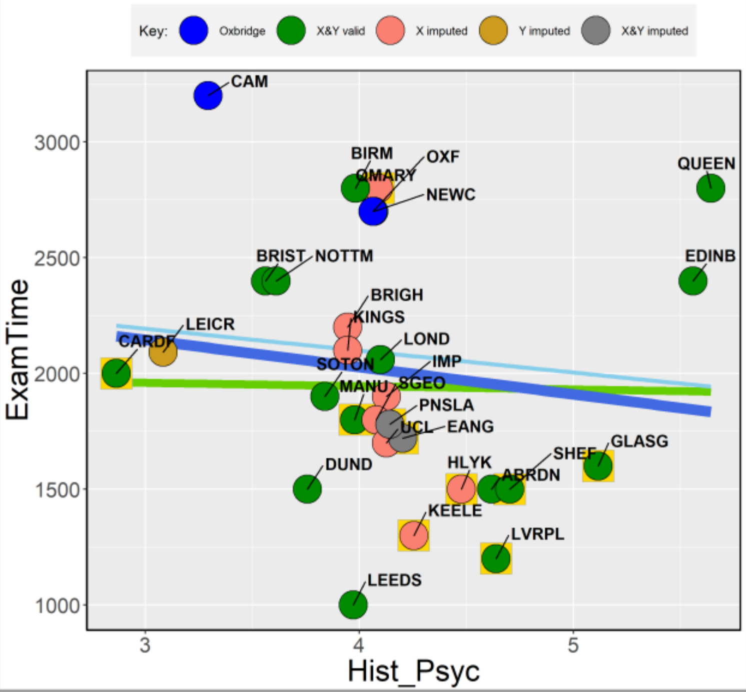

28/167 Y27: SelfRegLearn X4: Hist_Psyc

r(all)= -0.226 p= 0.238 r(NonImp)= -0.269 Npairs=29 NimputedPairs=10

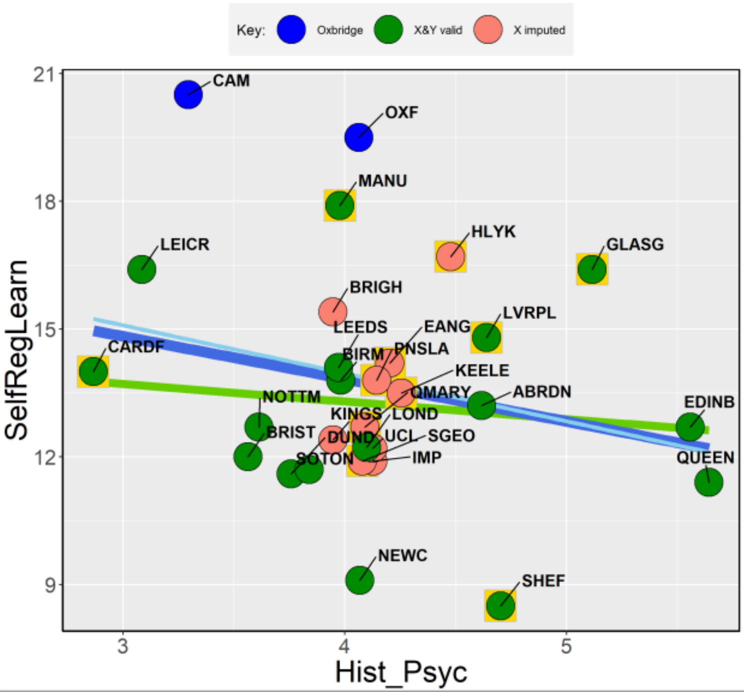

28/168 Y28: NSS_Satisfn X4: Hist_Psyc

r(all)= 0.006 p= 0.976 r(NonImp)= -0.019 Npairs=29 NimputedPairs=10

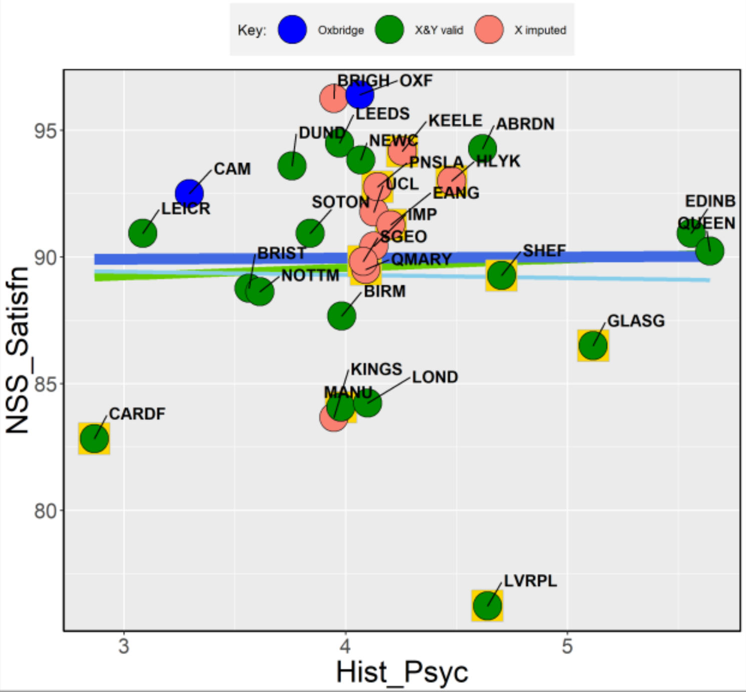

29/169 Y29: NSS_Feedback X4: Hist_Psyc
r(all)= 0.006 p= 0.976 r(NonImp)= -0.027 Npairs=29 NimputedPairs=10

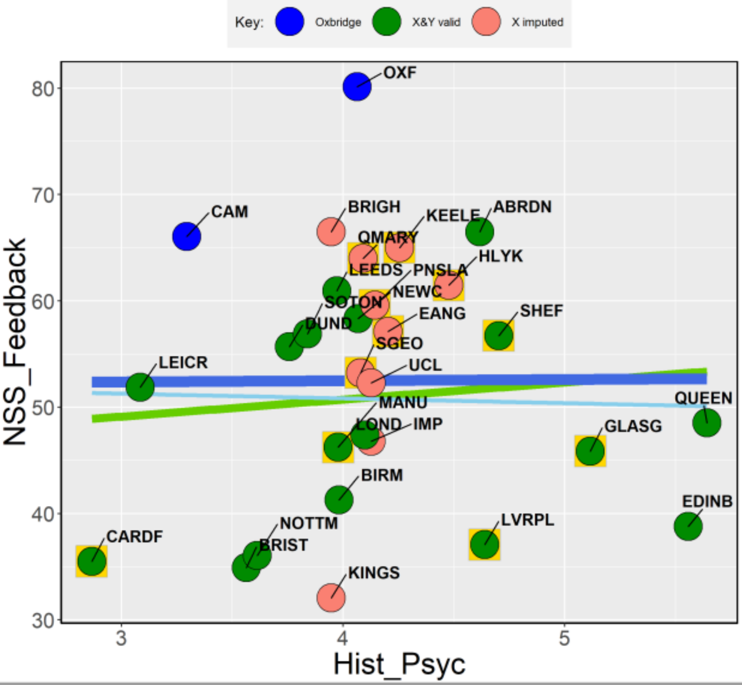

29/170 Y30: UKFPO_EPM X4: Hist_Psyc
r(all)= -0.363 p= 0.0527 r(NonImp)= -0.453 Npairs=29 NimputedPairs=10

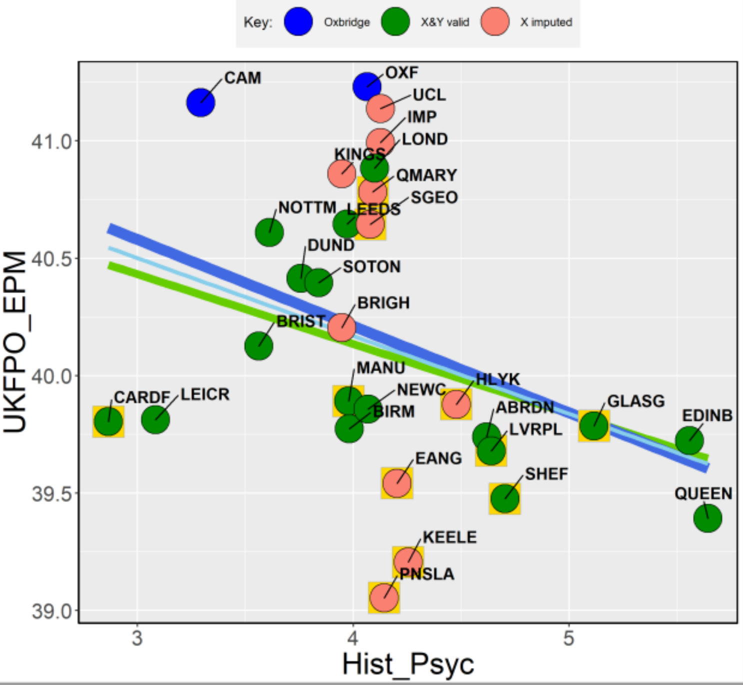

29/171 Y31: UKFPO_SJT X4: Hist_Psyc
r(all)= -0.067 p= 0.731 r(NonImp)= -0.048 Npairs=29 NimputedPairs=10

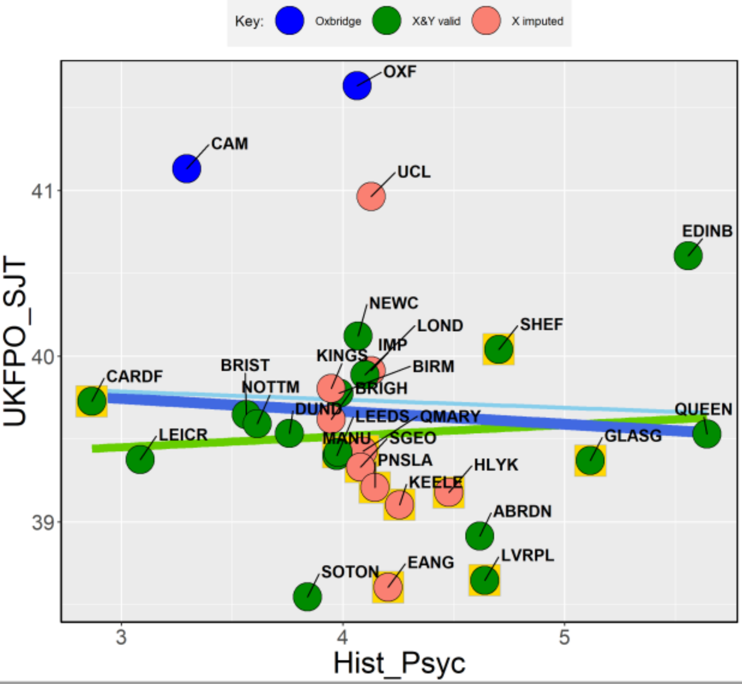

29/172 Y32: F1_Preparedness X4: Hist_Psyc
r(all)= 0.174 p= 0.366 r(NonImp)= 0.230 Npairs=29 NimputedPairs=10

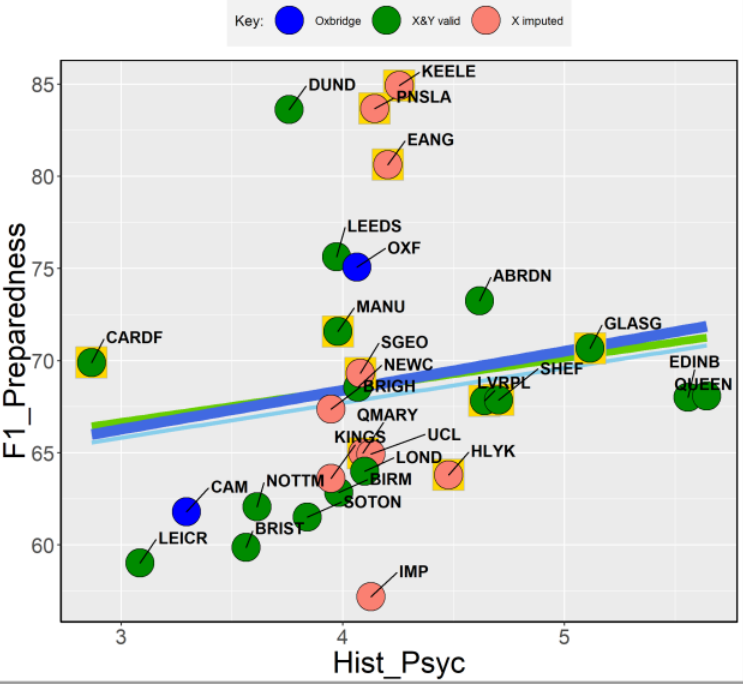

29/173 Y33: F1_Satisfn X4: Hist_Psyc
r(all)= 0.051 p= 0.794 r(NonImp)= 0.032 Npairs=29 NimputedPairs=10

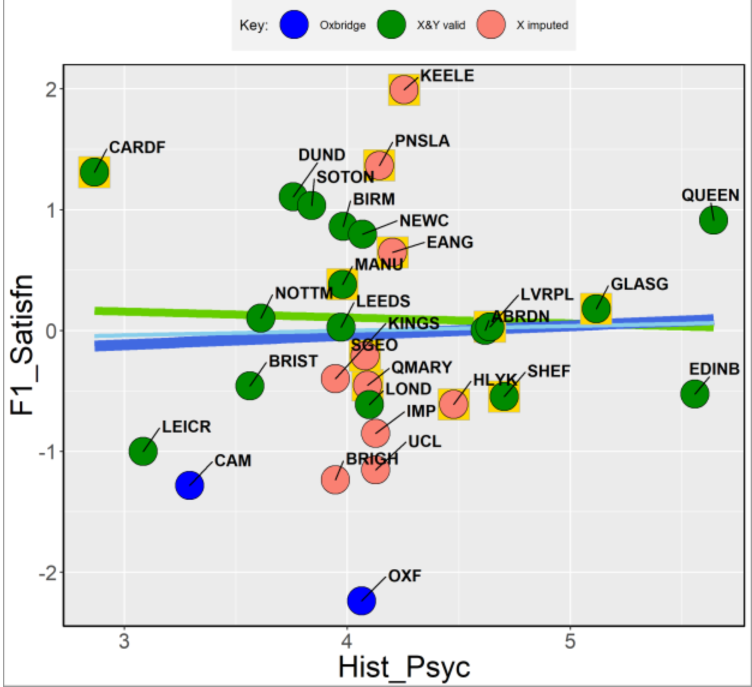

29/174 Y34: F1_Workload X4: Hist_Psyc
r(all)= -0.295 p= 0.121 r(NonImp)= -0.358 Npairs=29 NimputedPairs=10

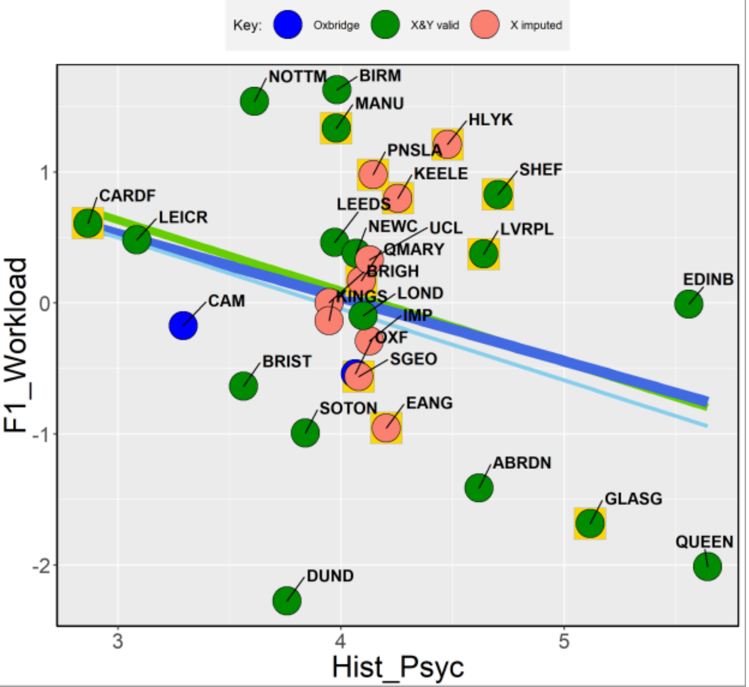

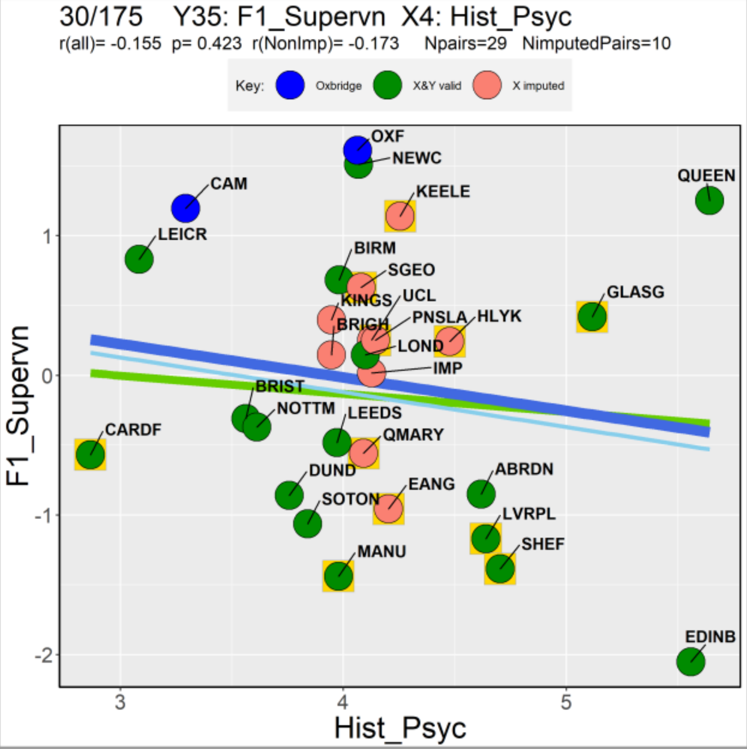
30/175 Y35: F1_Supervn X4: Hist_Psyc
r(all)= -0.155 p= 0.423 r(NonImp)= -0.173 Npairs=29 NimputedPairs=10
Key: Oxbridge X&Y valid X imputed
F1_Supervn
Hist_Psyc
-2 -1 0 1
3 4 5

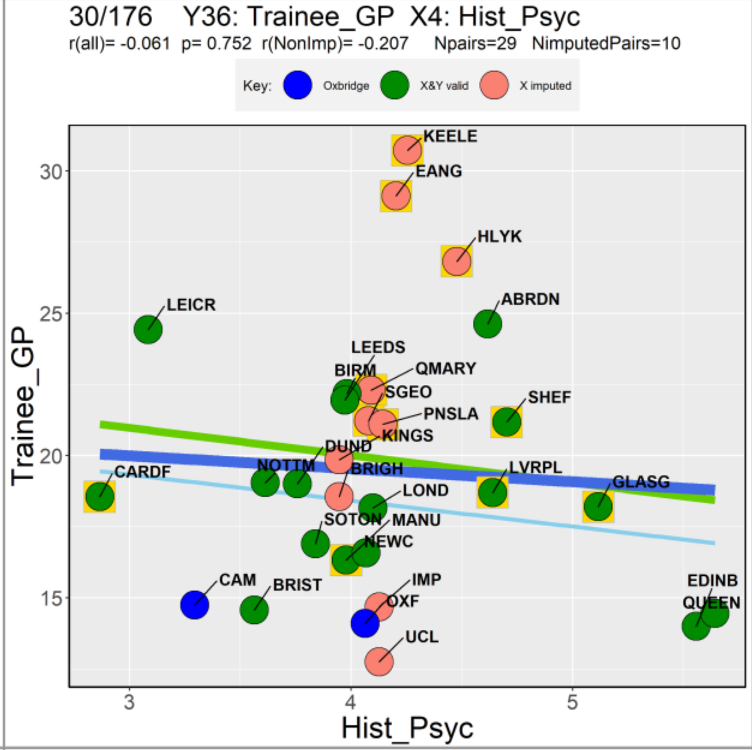
30/176 Y36: Trainee_GP X4: Hist_Psyc
r(all)= -0.061 p= 0.752 r(NonImp)= -0.207 Npairs=29 NimputedPairs=10
Key: Oxbridge X&Y valid X imputed
Trainee_GP
Hist_Psyc
15 20 25 30
3 4 5

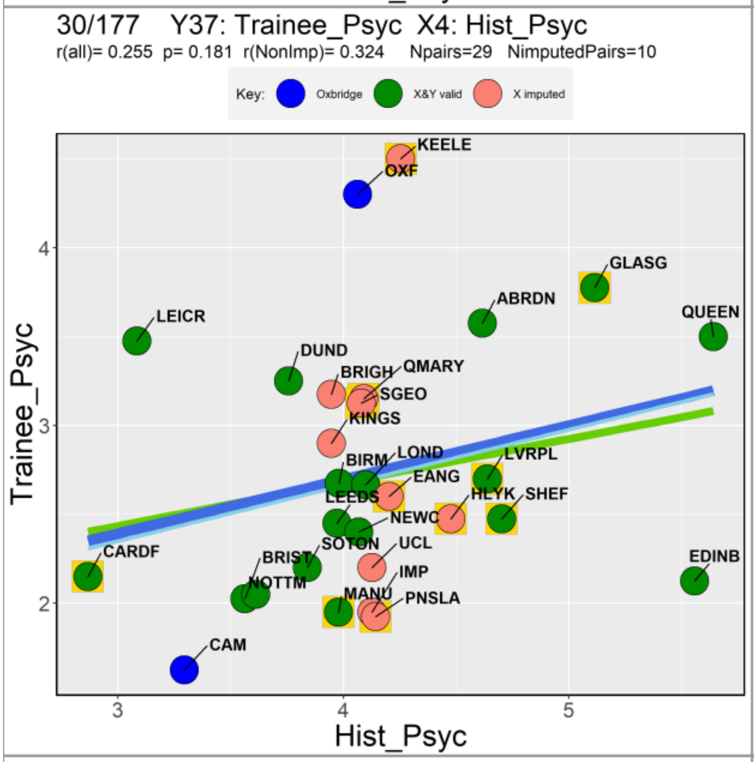
30/177 Y37: Trainee_Psyc X4: Hist_Psyc
r(all)= 0.255 p= 0.181 r(NonImp)= 0.324 Npairs=29 NimputedPairs=10
Key: Oxbridge X&Y valid X imputed
Trainee_Psyc
Hist_Psyc
2 3 4
3 4 5

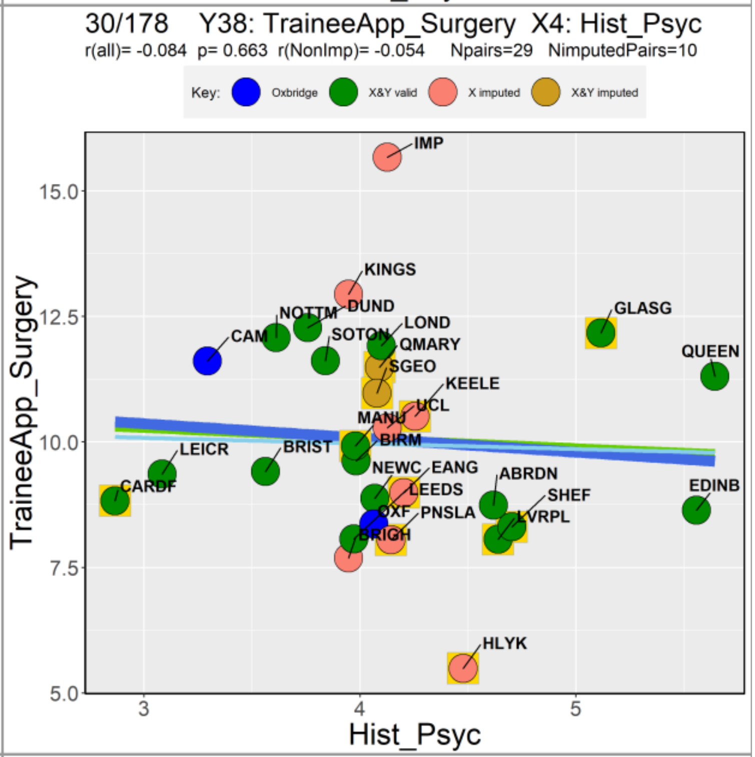
30/178 Y38: TraineeApp_Surgery X4: Hist_Psyc
r(all)= -0.084 p= 0.663 r(NonImp)= -0.054 Npairs=29 NimputedPairs=10
Key: Oxbridge X&Y valid X imputed X&Y imputed
TraineeApp_Surgery
Hist_Psyc
5.0 7.5 10.0 12.5 15.0
3 4 5

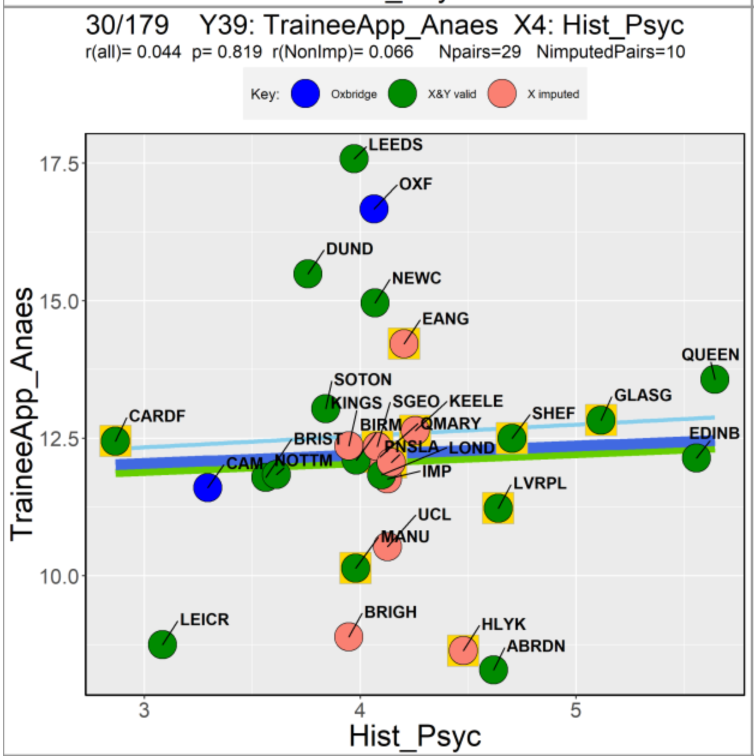
30/179 Y39: TraineeApp_Anaes X4: Hist_Psyc
r(all)= 0.044 p= 0.819 r(NonImp)= 0.066 Npairs=29 NimputedPairs=10
Key: Oxbridge X&Y valid X imputed
TraineeApp_Anaes
Hist_Psyc
10.0 12.5 15.0 17.5
3 4 5

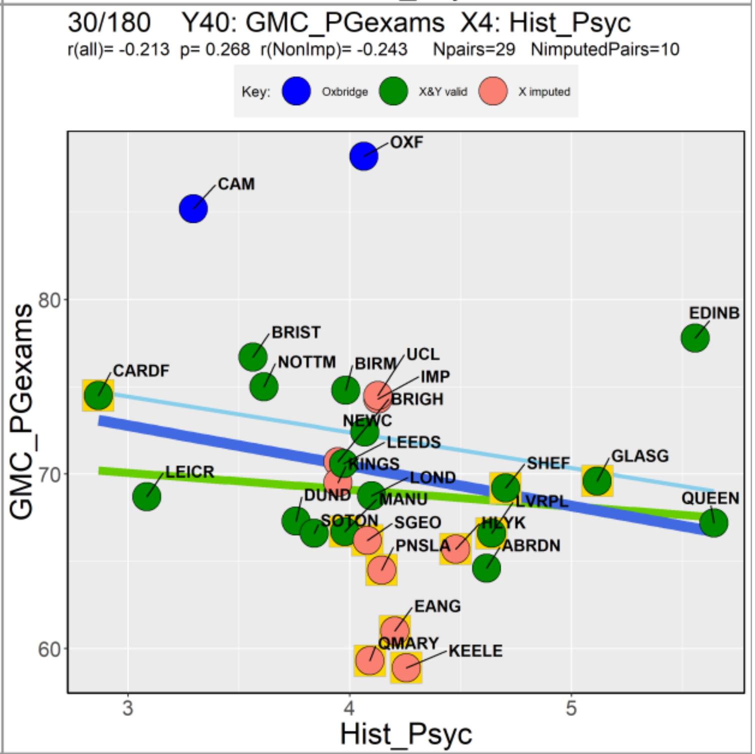
30/180 Y40: GMC_PGexams X4: Hist_Psyc
r(all)= -0.213 p= 0.268 r(NonImp)= -0.243 Npairs=29 NimputedPairs=10
Key: Oxbridge X&Y valid X imputed
GMC_PGexams
Hist_Psyc
60 70 80
3 4 5

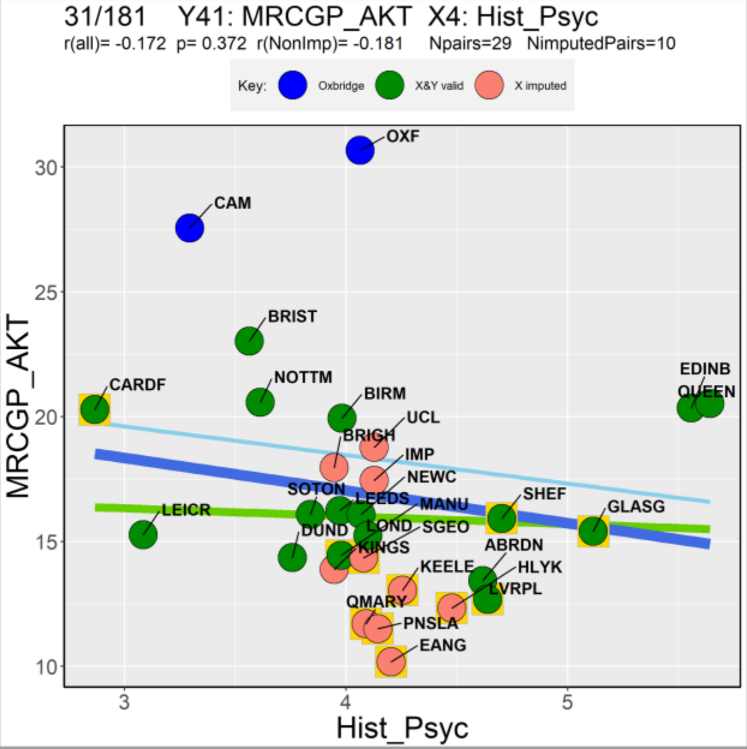
31/181 Y41: MRCGP_AKT X4: Hist_Psyc
r(all)= -0.172 p= 0.372 r(NonImp)= -0.181 Npairs=29 NimputedPairs=10
Key: Oxbridge X&Y valid X imputed
MRCGP_AKT
Hist_Psyc
10
15
20
25
30
3
4
5
OXF
CAM
BRIST
CARDF
NOTTM
BIRM
UCL
BRIGH
IMP
NEWC
SOTON
LEEDS
MANU
LEICR
DUND
LOND
SGEO
KINGS
SHEF
GLASG
ABRDN
KEELE
HLYK
LVRPL
QMARY
PNSLA
EANG
EDINB
QUEEN

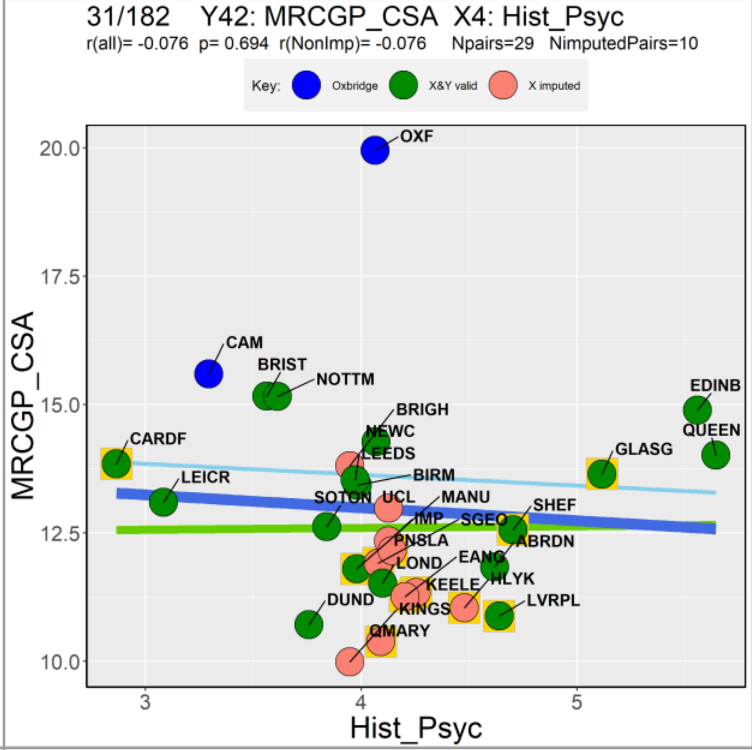
31/182 Y42: MRCGP_CSA X4: Hist_Psyc
r(all)= -0.076 p= 0.694 r(NonImp)= -0.076 Npairs=29 NimputedPairs=10
Key: Oxbridge X&Y valid X imputed
MRCGP_CSA
Hist_Psyc
10.0
12.5
15.0
17.5
20.0
3
4
5
OXF
CAM
BRIST
NOTTM
BRIGH
NEWC
LEEDS
CARDF
LEICR
BIRM
SOTON
UCL
MANU
IMP
SGEO
SHEF
PNSLA
ABRDN
LOND
EANG
HLYK
KEELE
DUND
KINGS
LVRPL
QMARY
EDINB
QUEEN
GLASG

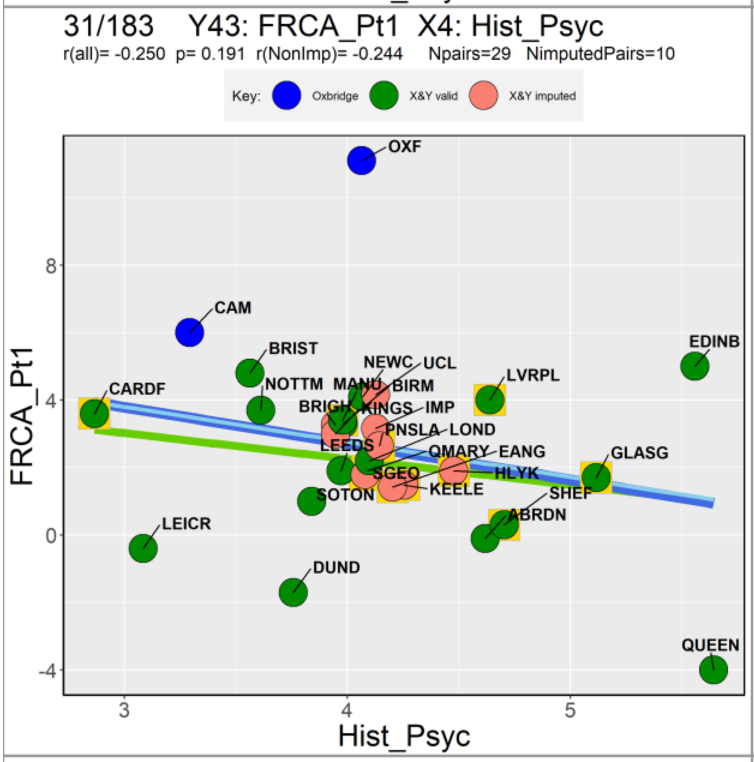
31/183 Y43: FRCA_Pt1 X4: Hist_Psyc
r(all)= -0.250 p= 0.191 r(NonImp)= -0.244 Npairs=29 NimputedPairs=10
Key: Oxbridge X&Y valid X&Y imputed
FRCA_Pt1
Hist_Psyc
-4
0
4
8
3
4
5
OXF
CAM
BRIST
NEWC
UCL
NOTTM
MANU
BIRM
LVRPL
EDINB
CARDF
BRIGH
KINGS
IMP
PNSLA
LOND
LEEDS
QMARY
EANG
GLASG
SGEO
HLYK
SOTON
KEELE
SHEF
ABRDN
LEICR
DUND
QUEEN

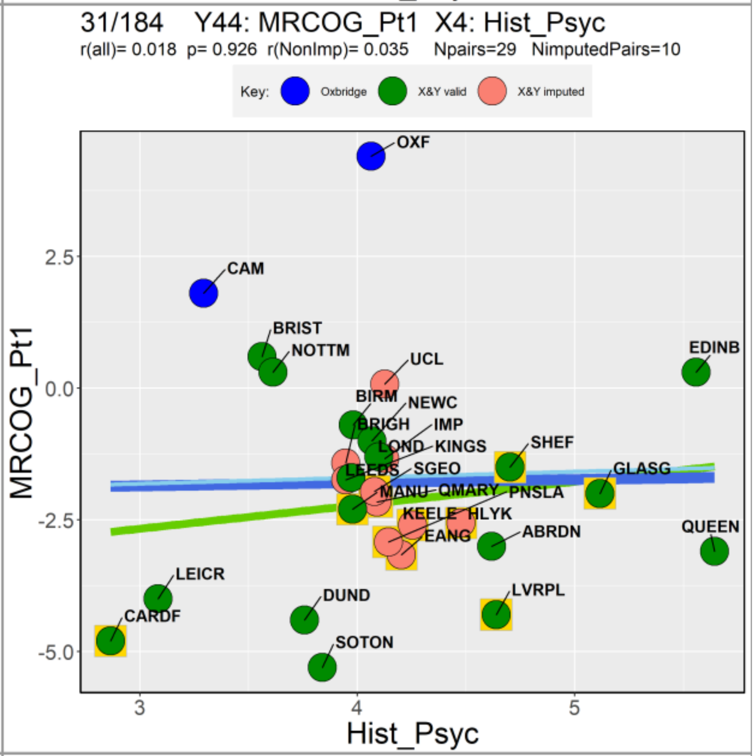
31/184 Y44: MRCOG_Pt1 X4: Hist_Psyc
r(all)= 0.018 p= 0.926 r(NonImp)= 0.035 Npairs=29 NimputedPairs=10
Key: Oxbridge X&Y valid X&Y imputed
MRCOG_Pt1
Hist_Psyc
-5.0
-2.5
0.0
2.5
3
4
5
OXF
CAM
BRIST
NOTTM
UCL
EDINB
BIRM
NEWC
BRIGH
IMP
LOND
KINGS
SHEF
LEEDS
SGEO
GLASG
MANU
QMARY
PNSLA
KEELE
HLYK
EANG
ABRDN
QUEEN
LEICR
CARDF
DUND
LVRPL
SOTON

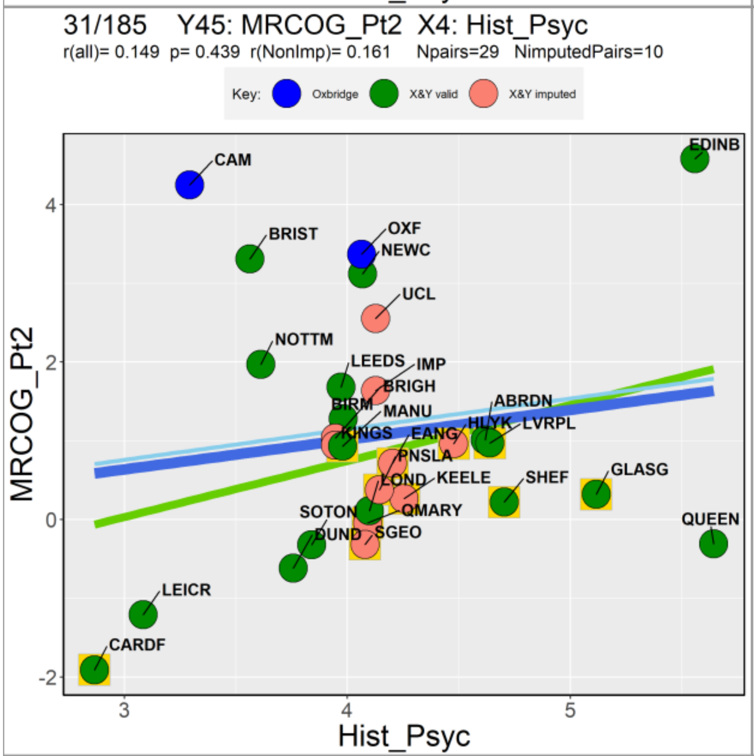
31/185 Y45: MRCOG_Pt2 X4: Hist_Psyc
r(all)= 0.149 p= 0.439 r(NonImp)= 0.161 Npairs=29 NimputedPairs=10
Key: Oxbridge X&Y valid X&Y imputed
MRCOG_Pt2
Hist_Psyc
-2
0
2
4
3
4
5
EDINB
CAM
BRIST
OXF
NEWC
UCL
NOTTM
LEEDS
IMP
BRIGH
ABRDN
BIRM
MANU
HLYK
LVRPL
KINGS
EANG
PNSLA
LOND
KEELE
SHEF
GLASG
SOTON
QMARY
DUND
SGEO
QUEEN
LEICR
CARDF

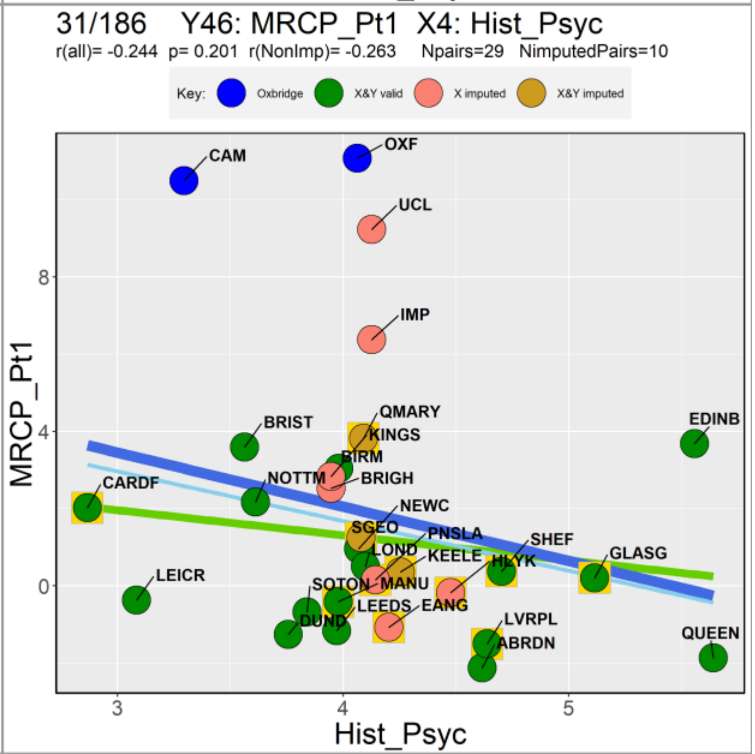
31/186 Y46: MRCP_Pt1 X4: Hist_Psyc
r(all)= -0.244 p= 0.201 r(NonImp)= -0.263 Npairs=29 NimputedPairs=10
Key: Oxbridge X&Y valid X imputed X&Y imputed
MRCP_Pt1
Hist_Psyc
0
4
8
3
4
5
OXF
CAM
UCL
IMP
QMARY
BRIST
KINGS
EDINB
BIRM
CARDF
NOTTM
BRIGH
NEWC
SGEO
PNSLA
SHEF
LOND
GLASG
KEELE
HLYK
LEICR
SOTON
MANU
LEEDS
EANG
DUND
LVRPL
ABRDN
QUEEN

32/187 Y47: MRCP_Pt2 X4: Hist_Psyc

r(all)= -0.085 p= 0.662 r(NonImp)= -0.092 Npairs=29 NimputedPairs=10

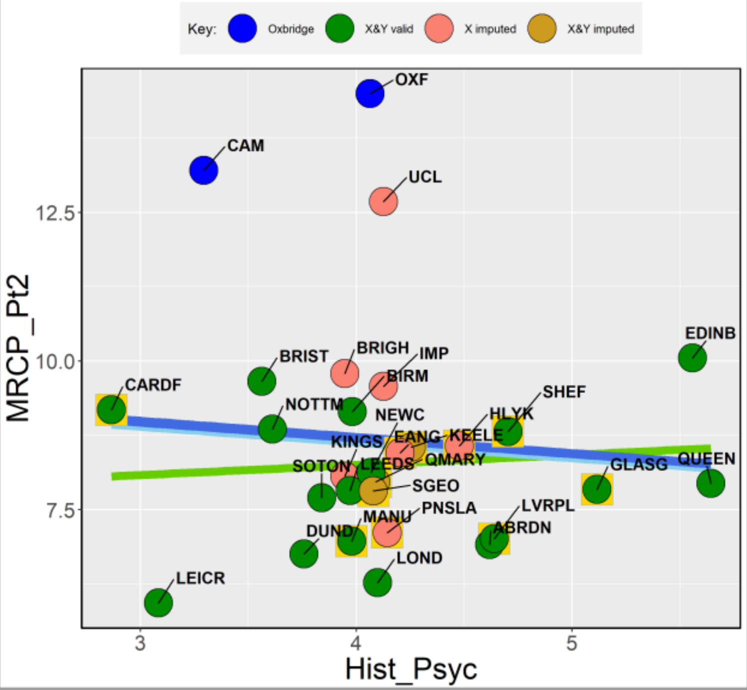

32/188 Y48: MRCP_PACES X4: Hist_Psyc

r(all)= -0.141 p= 0.465 r(NonImp)= -0.137 Npairs=29 NimputedPairs=10

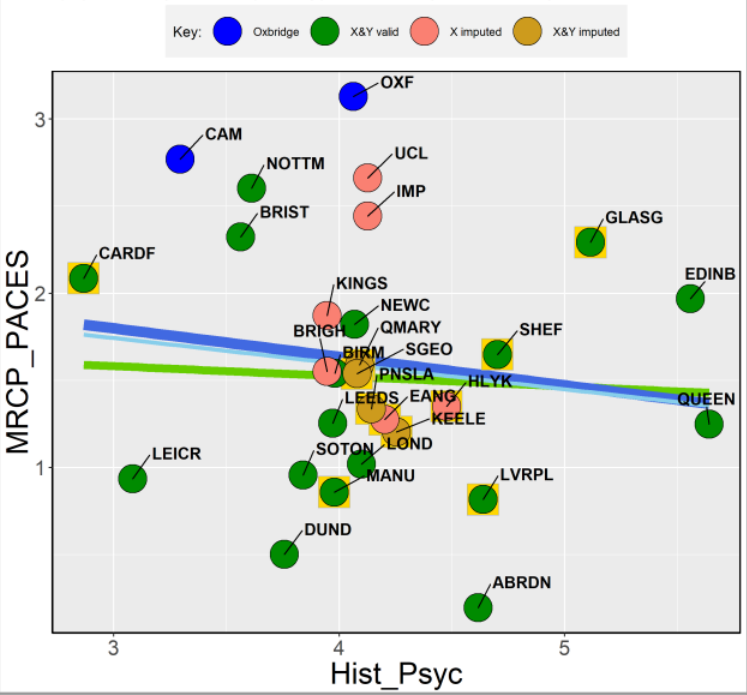

32/189 Y49: GMC_Sanctions X4: Hist_Psyc

r(all)= -0.002 p= 0.992 r(NonImp)= -0.032 Npairs=29 NimputedPairs=10

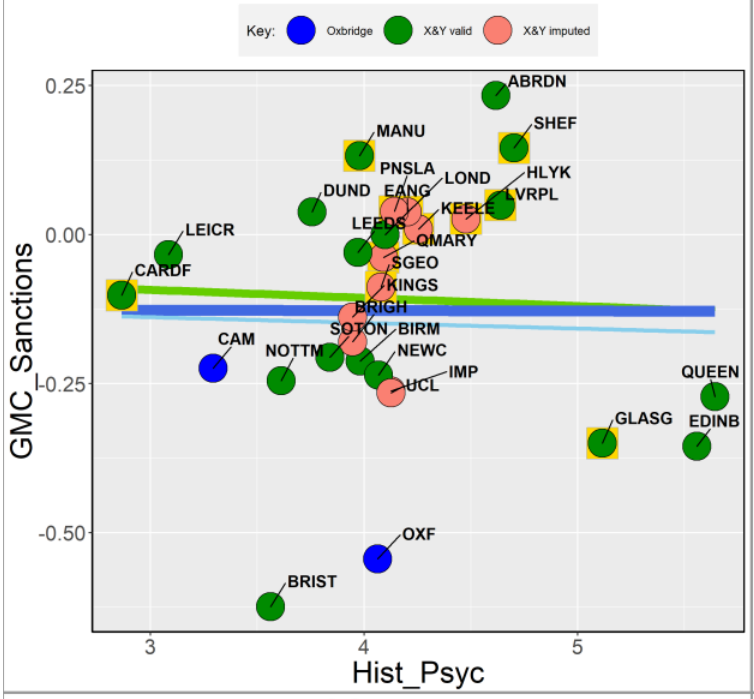

32/190 Y50: ARCP_NotExam X4: Hist_Psyc

r(all)= 0.215 p= 0.262 r(NonImp)= 0.208 Npairs=29 NimputedPairs=10

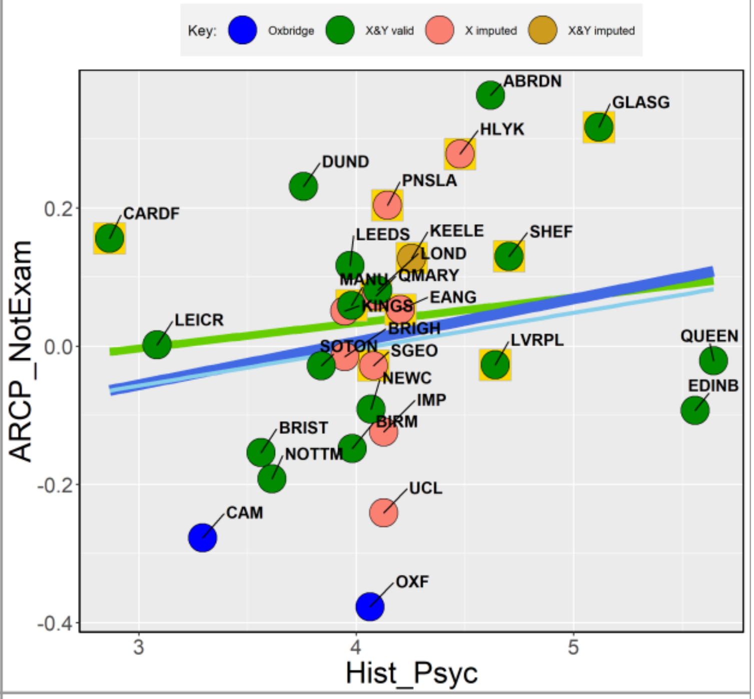

32/191 Y6: Hist_OG X5: Hist_Anaes

r(all)= 0.407 p= 0.0286 r(NonImp)= 0.412 Npairs=29 NimputedPairs=10

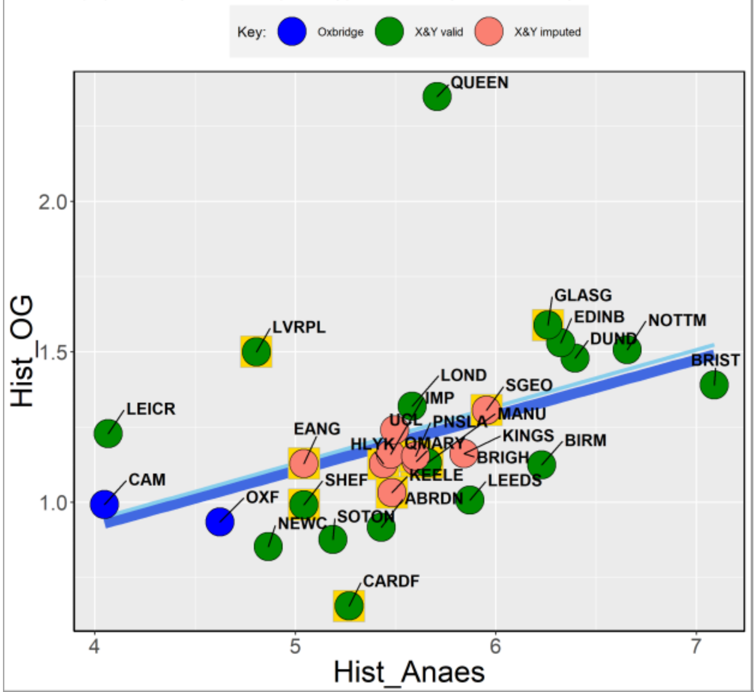

32/192 Y7: Hist_IntMed X5: Hist_Anaes

r(all)= -0.286 p= 0.133 r(NonImp)= -0.304 Npairs=29 NimputedPairs=10

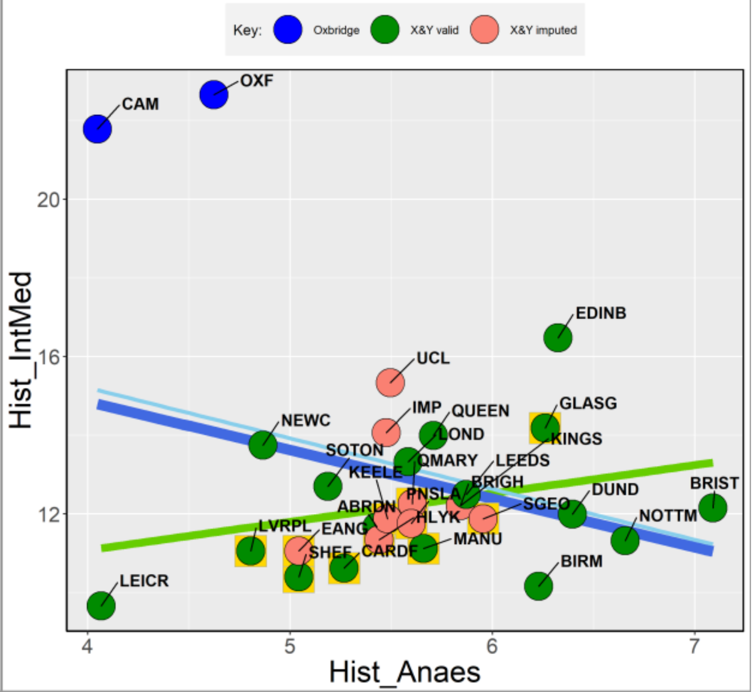

33/193 Y8: Hist_Surgery X5: Hist_Anaes

r(all)= -0.094 p= 0.628 r(NonImp)= -0.114 Npairs=29 NimputedPairs=10

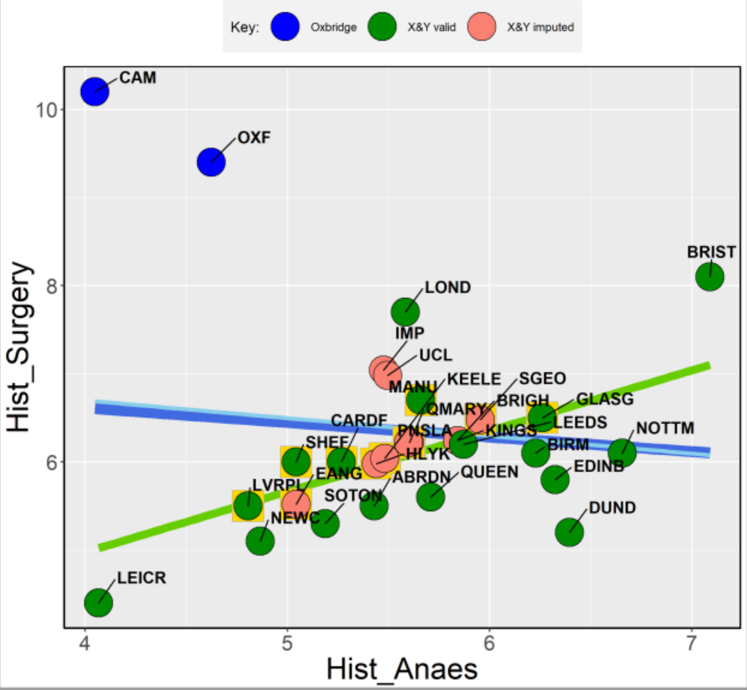

33/194 Y9: Post2000 X5: Hist_Anaes

r(all)= -0.045 p= 0.817 r(NonImp)= NA Npairs=29 NimputedPairs=10

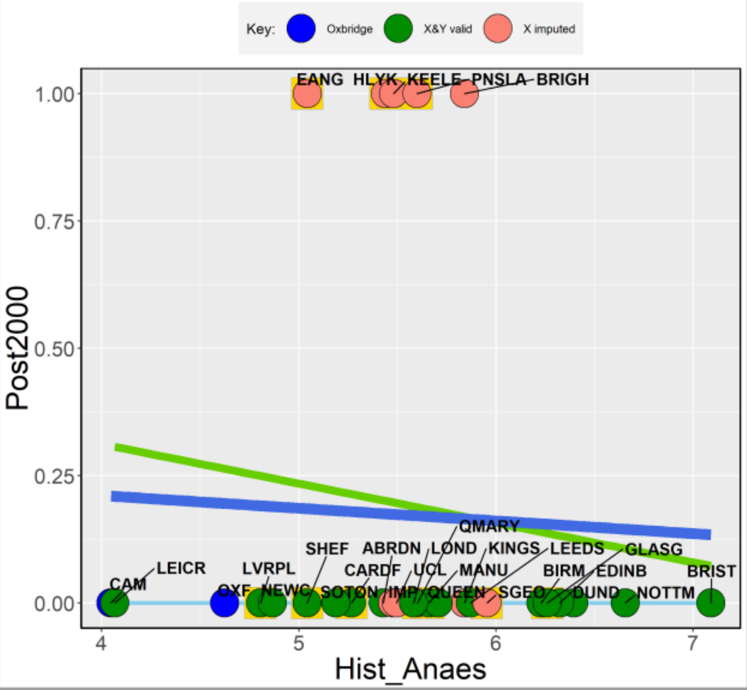

33/195 Y10: REF X5: Hist_Anaes

r(all)= -0.185 p= 0.336 r(NonImp)= -0.261 Npairs=29 NimputedPairs=10

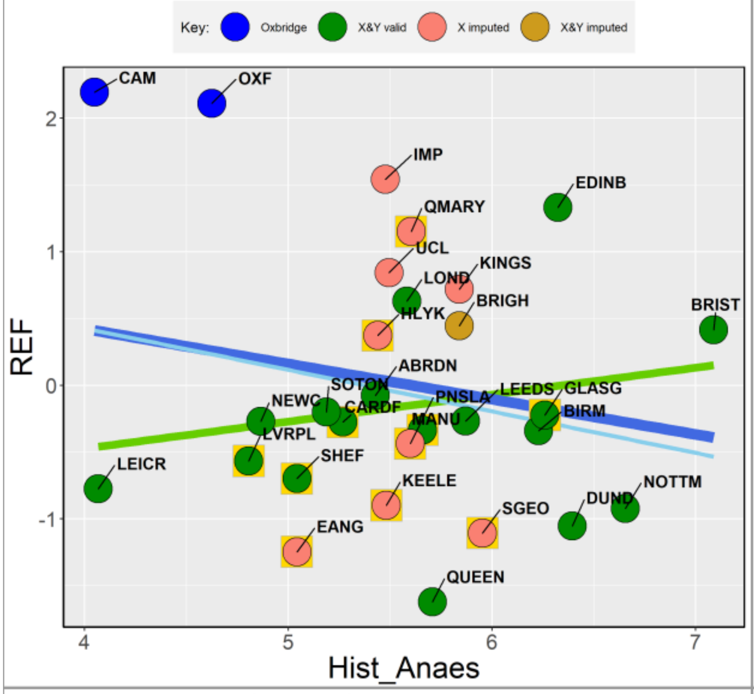

33/196 Y11: PBL_School X5: Hist_Anaes

r(all)= -0.091 p= 0.64 r(NonImp)= -0.091 Npairs=29 NimputedPairs=10

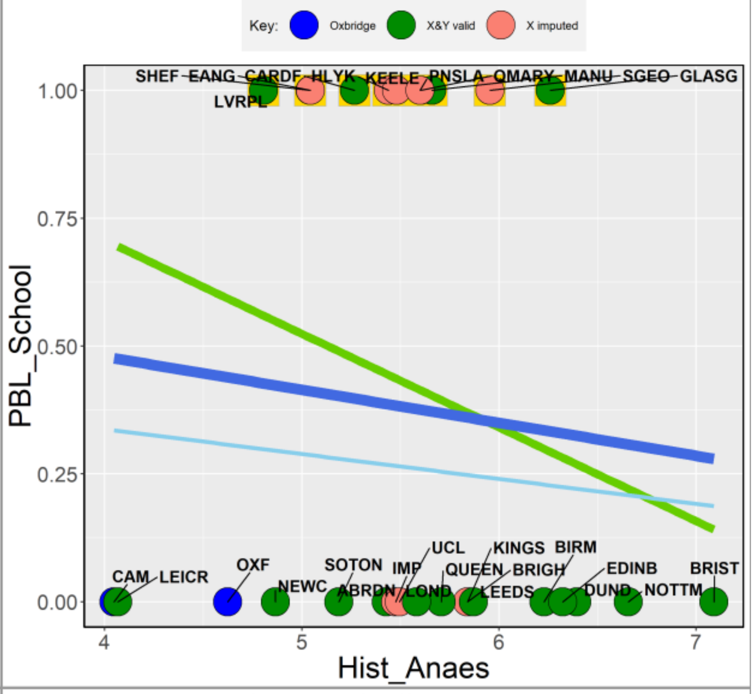

33/197 Y12: Spend_Student X5: Hist_Anaes

r(all)= -0.204 p= 0.288 r(NonImp)= -0.265 Npairs=29 NimputedPairs=10

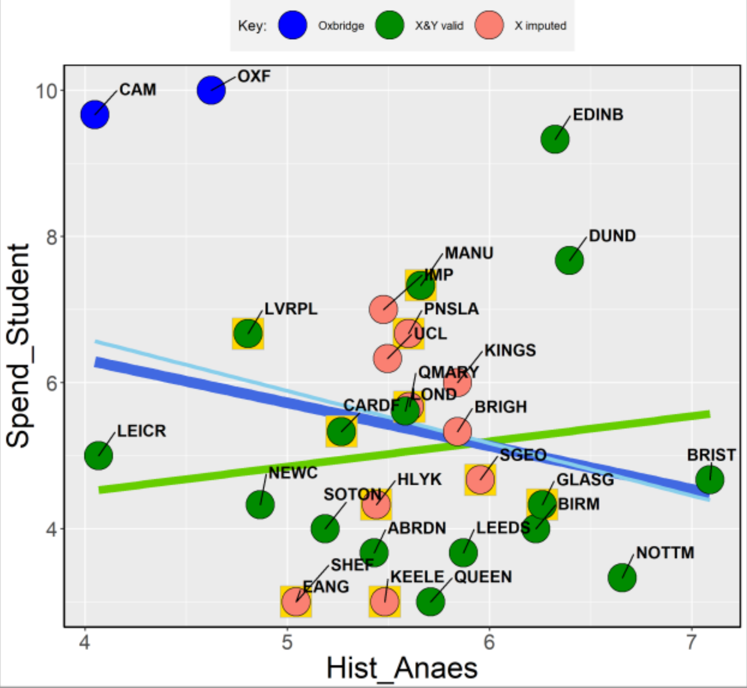

33/198 Y13: Student_Staff X5: Hist_Anaes

r(all)= 0.225 p= 0.241 r(NonImp)= 0.210 Npairs=29 NimputedPairs=10

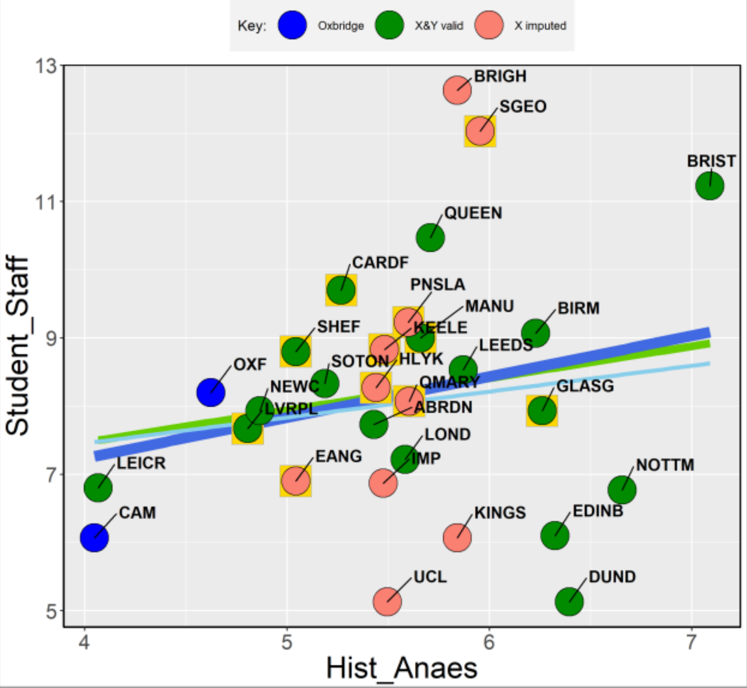

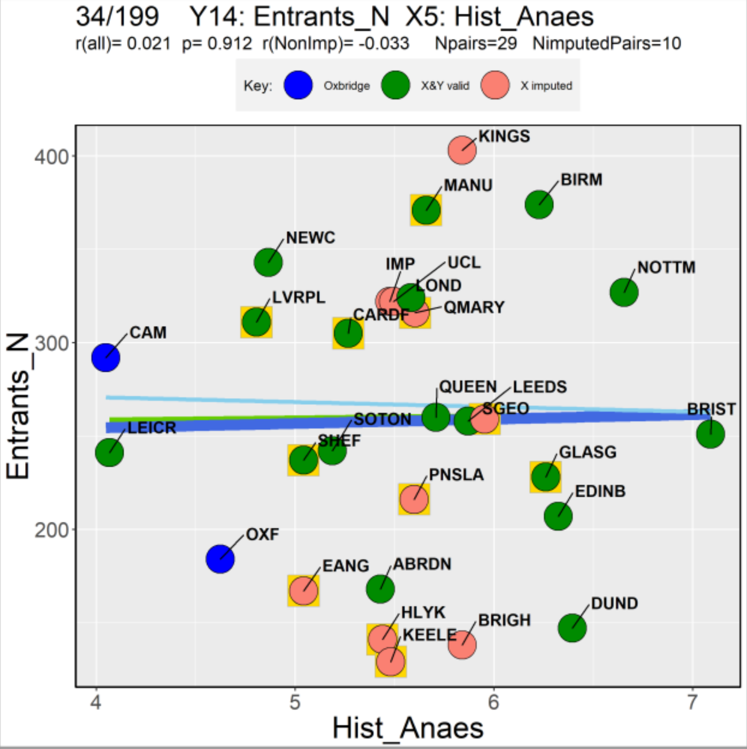
34/199 Y14: Entrants_N X5: Hist_Anaes
r(all)= 0.021 p= 0.912 r(NonImp)= -0.033 Npairs=29 NimputedPairs=10
Key: Oxbridge X&Y valid X imputed
Entrants_N
Hist_Anaes
KINGS MANU BIRM NEWC IMP UCL LOND NOTTM LVRPL CARDF QMARY CAM QUEEN LEEDS SGEO BRIST SOTON LEICR SHEF GLASG PNSLA EDINB OXF EANG ABRDN DUND HLYK KEELE BRIGH

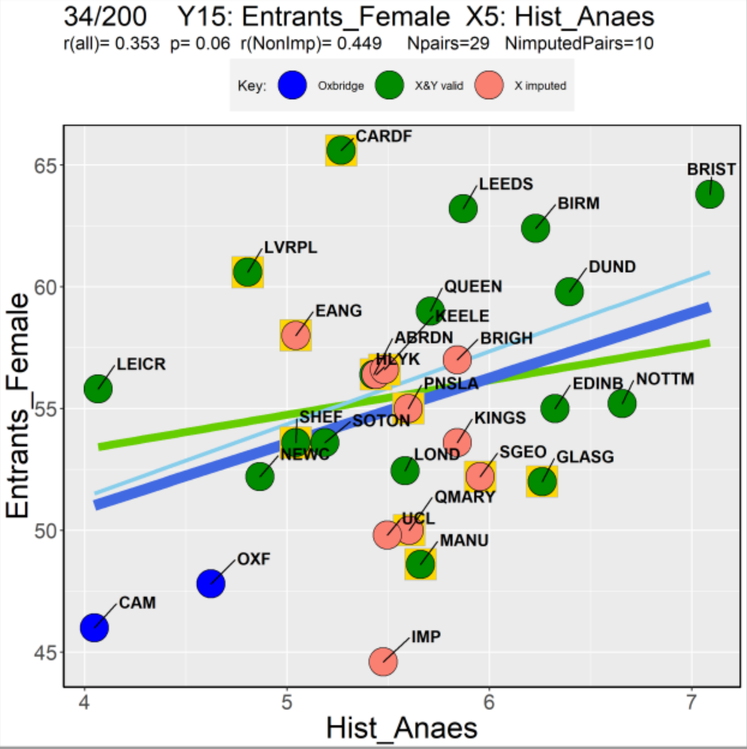
34/200 Y15: Entrants_Female X5: Hist_Anaes
r(all)= 0.353 p= 0.06 r(NonImp)= 0.449 Npairs=29 NimputedPairs=10
Key: Oxbridge X&Y valid X imputed
Entrants_Female
Hist_Anaes
CARDF BRIST LEEDS BIRM LVRPL DUND QUEEN EANG KEELE ABRDN BRIGH LEICR HLYK NOTTM PNSLA EDINB SHEF SOTON KINGS NEWC LOND SGEO GLASG QMARY UCL MANU OXF CAM IMP

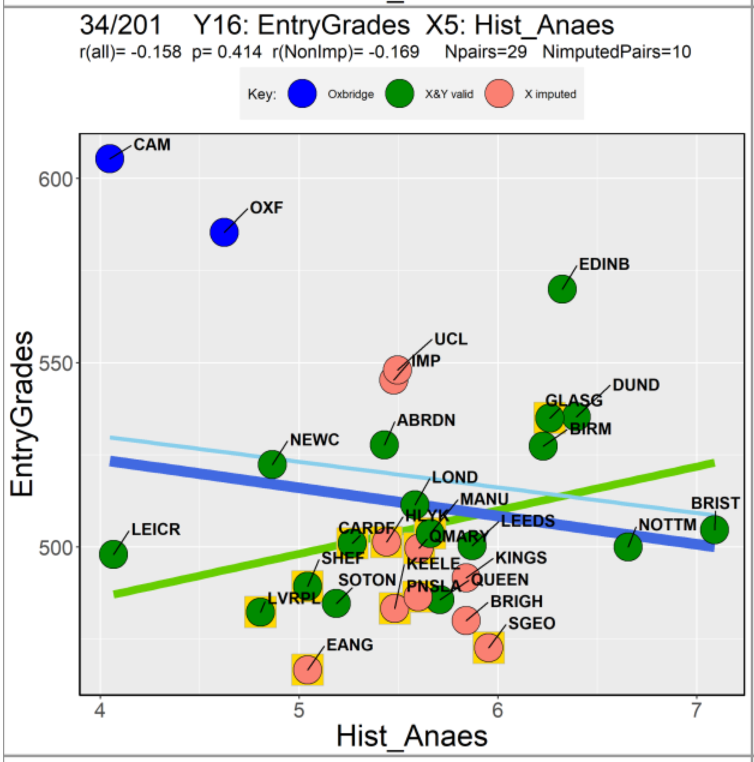
34/201 Y16: EntryGrades X5: Hist_Anaes
r(all)= -0.158 p= 0.414 r(NonImp)= -0.169 Npairs=29 NimputedPairs=10
Key: Oxbridge X&Y valid X imputed
EntryGrades
Hist_Anaes
CAM OXF EDINB UCL IMP DUND GLASG ABRDN BIRM NEWC LOND MANU HLYK LEEDS BRIST CARDF NOTTM LEICR QMARY SHEF KEELE KINGS SOTON QUEEN LVRPL PNSLA BRIGH SGEO EANG

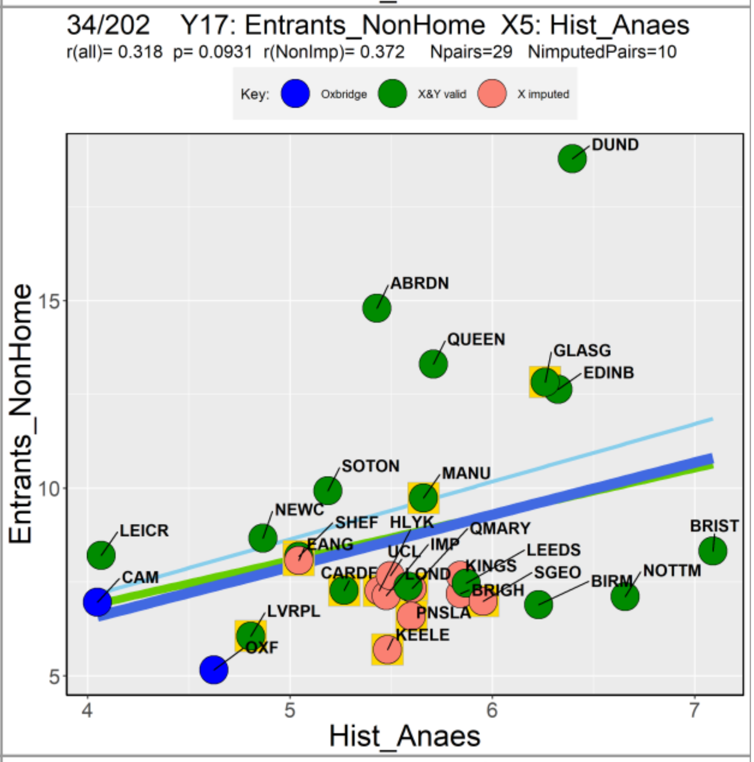
34/202 Y17: Entrants_NonHome X5: Hist_Anaes
r(all)= 0.318 p= 0.0931 r(NonImp)= 0.372 Npairs=29 NimputedPairs=10
Key: Oxbridge X&Y valid X imputed
Entrants_NonHome
Hist_Anaes
DUND ABRDN QUEEN GLASG EDINB SOTON MANU NEWC SHEF HLYK QMARY BRIST LEICR EANG IMP LEEDS CAM CARDF UCL LOND KINGS SGEO BRIGH BIRM NOTTM LVRPL PNSLA OXF KEELE

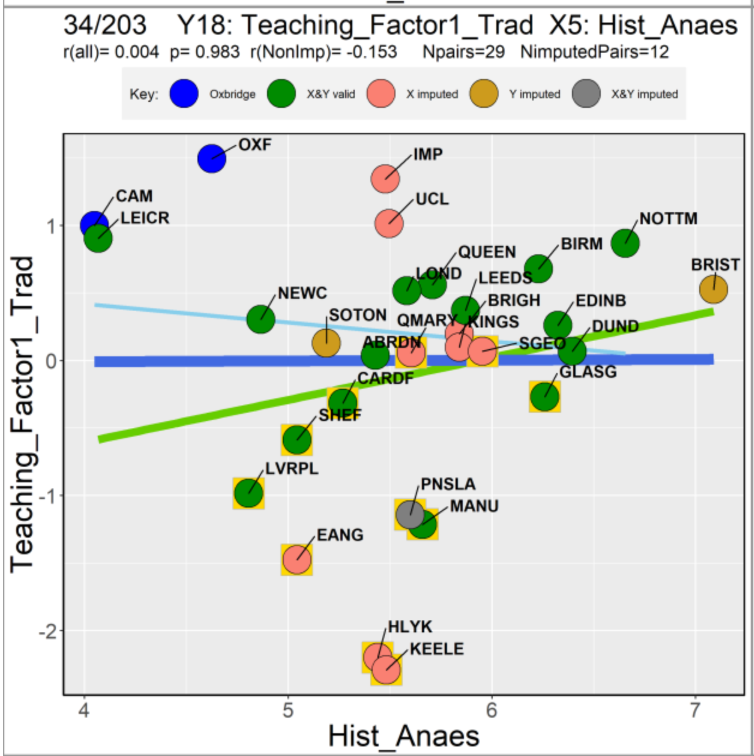
34/203 Y18: Teaching_Factor1_Trad X5: Hist_Anaes
r(all)= 0.004 p= 0.983 r(NonImp)= -0.153 Npairs=29 NimputedPairs=12
Key: Oxbridge X&Y valid X imputed Y imputed X&Y imputed
Teaching_Factor1_Trad
Hist_Anaes
OXF IMP CAM UCL LEICR NOTTM QUEEN BIRM BRIST LOND LEEDS NEWC EDINB SOTON QMARY KINGS DUND ABRDN SGEO GLASG CARDF SHEF LVRPL PNSLA MANU EANG HLYK KEELE

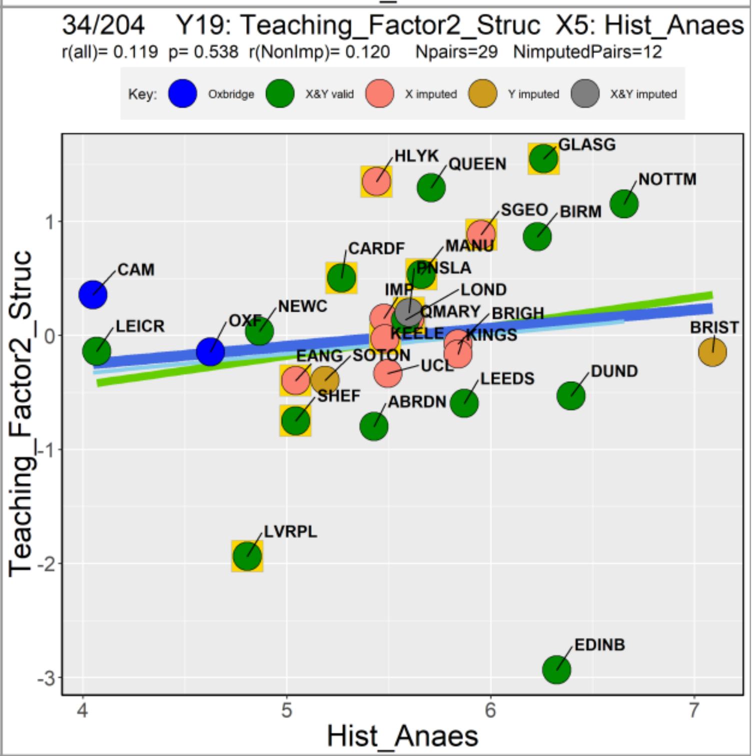
34/204 Y19: Teaching_Factor2_Struc X5: Hist_Anaes
r(all)= 0.119 p= 0.538 r(NonImp)= 0.120 Npairs=29 NimputedPairs=12
Key: Oxbridge X&Y valid X imputed Y imputed X&Y imputed
Teaching_Factor2_Struc
Hist_Anaes
GLASG HLYK QUEEN NOTTM SGEO BIRM CARDF MANU PNSLA CAM IMP LOND NEWC QMARY BRIGH LEICR OXF KEELE KINGS BRIST EANG SOTON UCL LEEDS DUND SHEF ABRDN LVRPL EDINB

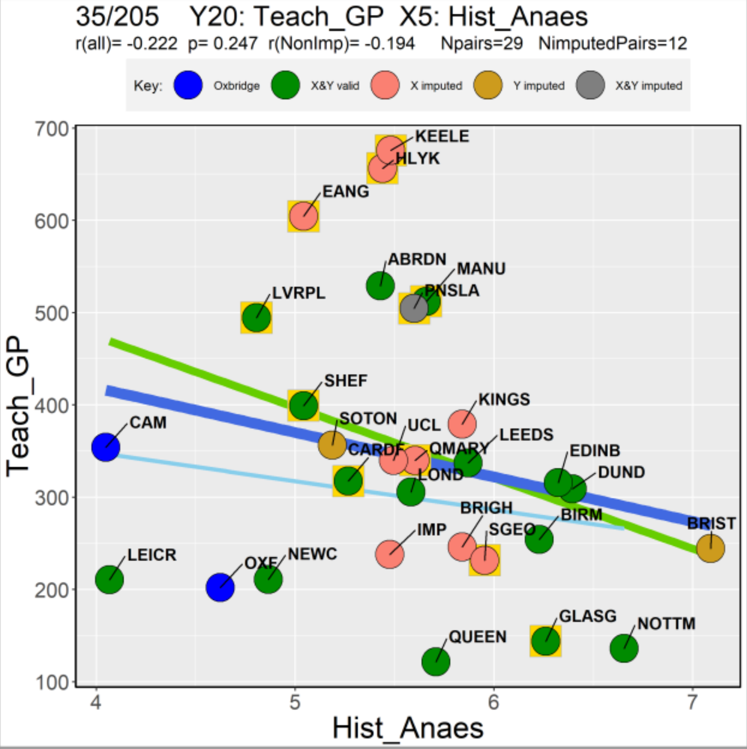
35/205 Y20: Teach_GP X5: Hist_Anaes
r(all)= -0.222 p= 0.247 r(NonImp)= -0.194 Npairs=29 NimputedPairs=12
Key: Oxbridge X&Y valid X imputed Y imputed X&Y imputed
Teach_GP
Hist_Anaes
KEELE
HLYK
EANG
ABRDN
MANU
PNSLA
LVRPL
SHEF
KINGS
CAM
SOTON
UCL
LEEDS
QMARY
CARDF
EDINB
LOND
DUND
BRIGH
BRIST
IMP
SGEO
BIRM
LEICR
OXF
NEWC
GLASG
NOTTM
QUEEN

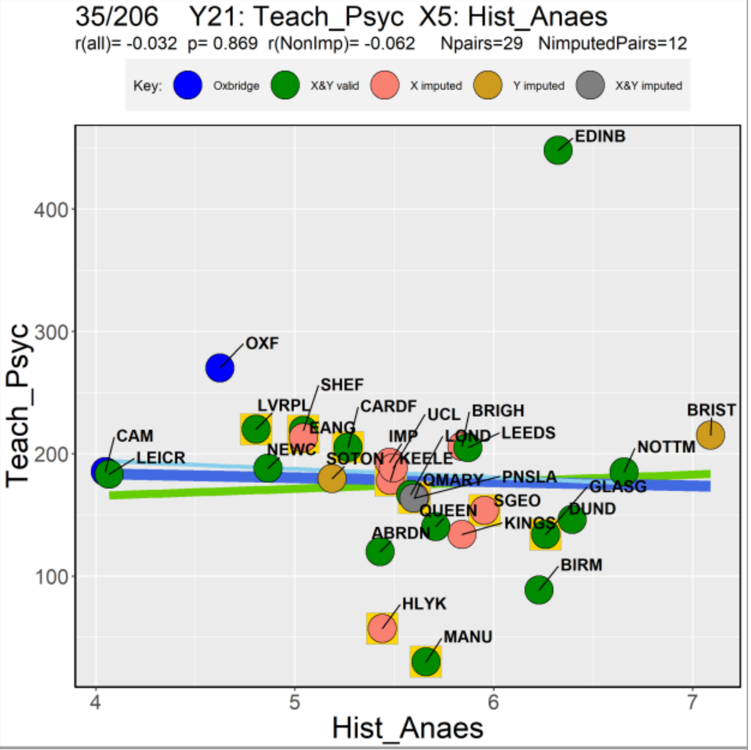
35/206 Y21: Teach_Psyc X5: Hist_Anaes
r(all)= -0.032 p= 0.869 r(NonImp)= -0.062 Npairs=29 NimputedPairs=12
Key: Oxbridge X&Y valid X imputed Y imputed X&Y imputed
Teach_Psyc
Hist_Anaes
EDINB
OXF
SHEF
LVRPL
CARDF
UCL
BRIGH
BRIST
CAM
EANG
IMP
LOND
LEEDS
NEWC
NOTTM
LEICR
SOTON
KEELE
QMARY
PNSLA
GLASG
SGEO
QUEEN
DUND
KINGS
ABRDN
BIRM
HLYK
MANU

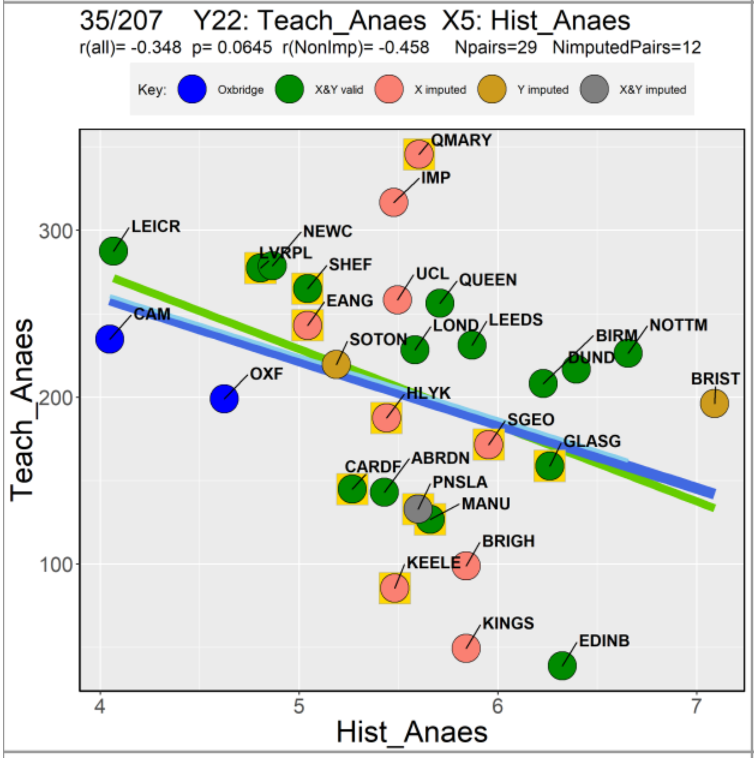
35/207 Y22: Teach_Anaes X5: Hist_Anaes
r(all)= -0.348 p= 0.0645 r(NonImp)= -0.458 Npairs=29 NimputedPairs=12
Key: Oxbridge X&Y valid X imputed Y imputed X&Y imputed
Teach_Anaes
Hist_Anaes
QMARY
IMP
LEICR
NEWC
LVRPL
SHEF
UCL
QUEEN
EANG
CAM
LOND
LEEDS
SOTON
BIRM
NOTTM
DUND
OXF
BRIST
HLYK
SGEO
GLASG
CARDF
ABRDN
PNSLA
MANU
BRIGH
KEELE
KINGS
EDINB

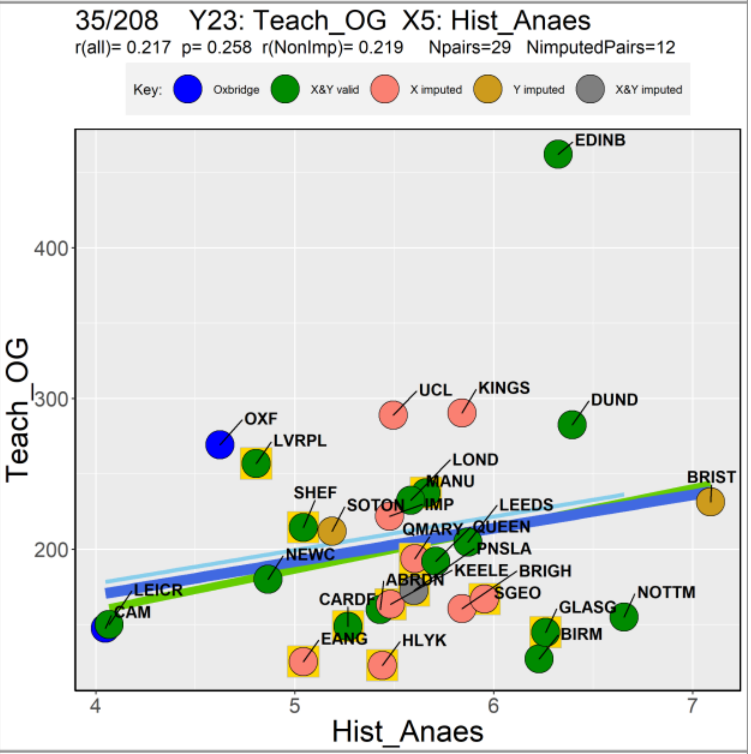
35/208 Y23: Teach_OG X5: Hist_Anaes
r(all)= 0.217 p= 0.258 r(NonImp)= 0.219 Npairs=29 NimputedPairs=12
Key: Oxbridge X&Y valid X imputed Y imputed X&Y imputed
Teach_OG
Hist_Anaes
EDINB
UCL
KINGS
DUND
OXF
LVRPL
LOND
MANU
BRIST
SHEF
SOTON
IMP
LEEDS
QMARY
QUEEN
PNSLA
NEWC
KEELE
BRIGH
ABRDN
LEICR
CARDF
SGEO
NOTTM
CAM
GLASG
BIRM
EANG
HLYK

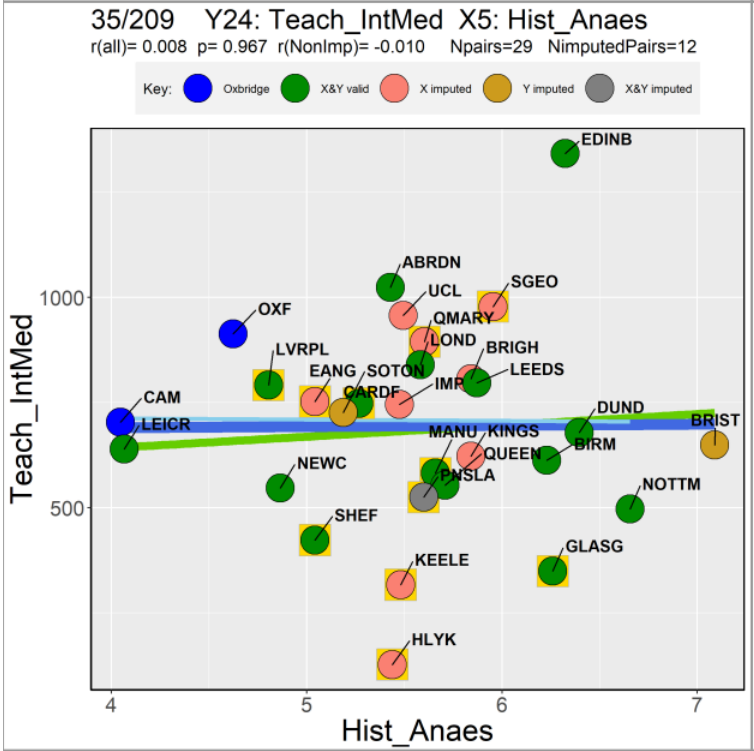
35/209 Y24: Teach_IntMed X5: Hist_Anaes
r(all)= 0.008 p= 0.967 r(NonImp)= -0.010 Npairs=29 NimputedPairs=12
Key: Oxbridge X&Y valid X imputed Y imputed X&Y imputed
Teach_IntMed
Hist_Anaes
EDINB
ABRDN
SGEO
UCL
OXF
QMARY
LOND
LVRPL
BRIGH
EANG
SOTON
LEEDS
CARDF
IMP
CAM
DUND
LEICR
BRIST
MANU
KINGS
QUEEN
BIRM
NEWC
PNSLA
NOTTM
SHEF
GLASG
KEELE
HLYK

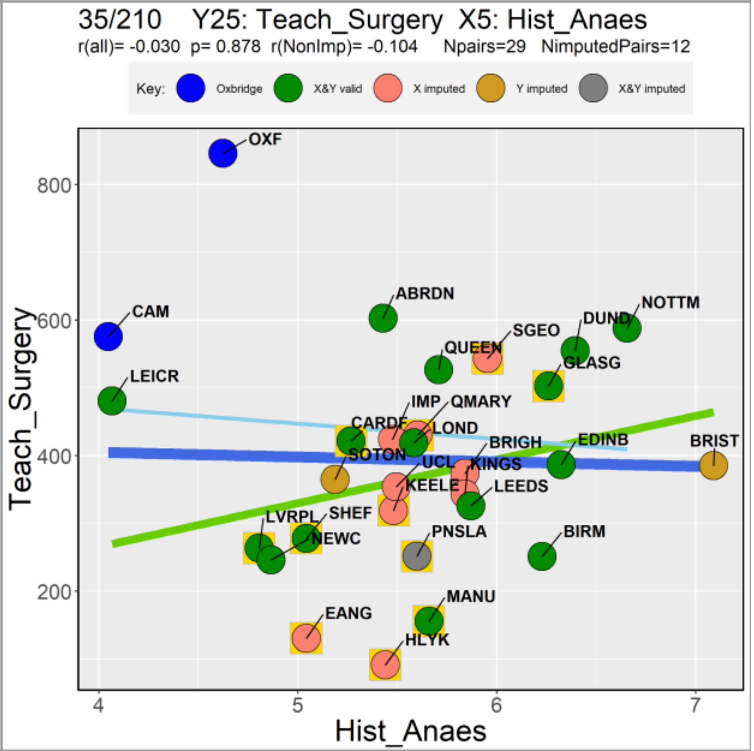
35/210 Y25: Teach_Surgery X5: Hist_Anaes
r(all)= -0.030 p= 0.878 r(NonImp)= -0.104 Npairs=29 NimputedPairs=12
Key: Oxbridge X&Y valid X imputed Y imputed X&Y imputed
Teach_Surgery
Hist_Anaes
OXF
ABRDN
CAM
NOTTM
DUND
SGEO
QUEEN
GLASG
LEICR
IMP
QMARY
CARDF
LOND
BRIGH
EDINB
BRIST
SOTON
UCL
KINGS
KEELE
LEEDS
LVRPL
SHEF
NEWC
PNSLA
BIRM
EANG
MANU
HLYK
