# Supplementary material for: Exploring UK medical school differences: the MedDifs study of selection, teaching, student and F1 perceptions, postgraduate outcomes and fitness to practise
Source: BMC Med. 2020 May 14;18:136. doi: 10.1186/s12916-020-01572-3 (PMC7222458; doi:10.1186/s12916-020-01572-3)
Supplement: Supplementary file 5 — Additional file 5. Graphs 211 to 420 (pages 36 to 70). [file 12916_2020_1572_MOESM5_ESM.pdf]

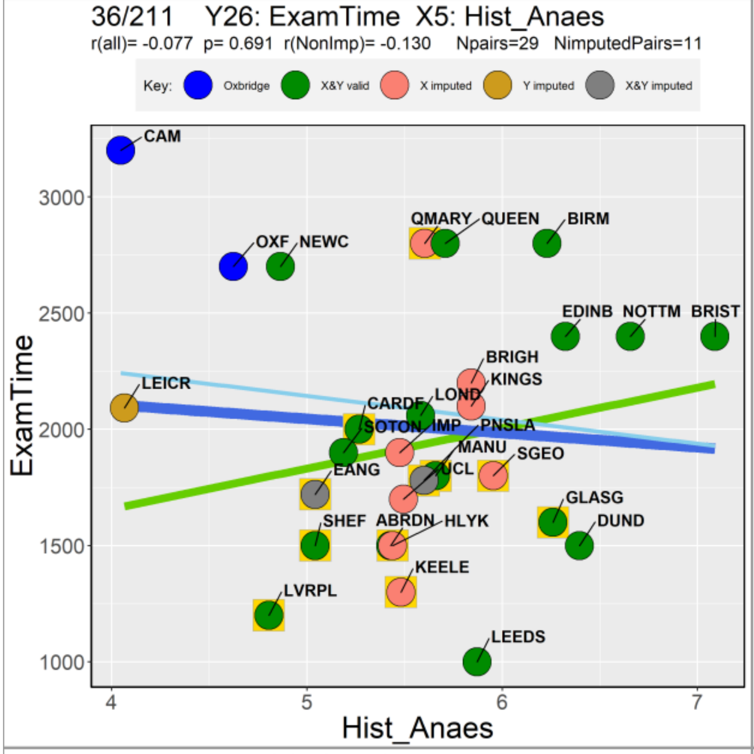
36/211 Y26: ExamTime X5: Hist_Anaes
r(all)= -0.077 p= 0.691 r(NonImp)= -0.130 Npairs=29 NimputedPairs=11
Key: Oxbridge X&Y valid X imputed Y imputed X&Y imputed
ExamTime
Hist_Anaes
1000
1500
2000
2500
3000
4
5
6
7
CAM
OXF
NEWC
QMARY
QUEEN
BIRM
EDINB
NOTTM
BRIST
BRIGH
KINGS
LEICR
LOND
CARDF
SOTON
IMP
PNSLA
MANU
SGEO
EANG
UCL
GLASG
SHEF
ABRDN
HLYK
DUND
KEELE
LVRPL
LEEDS

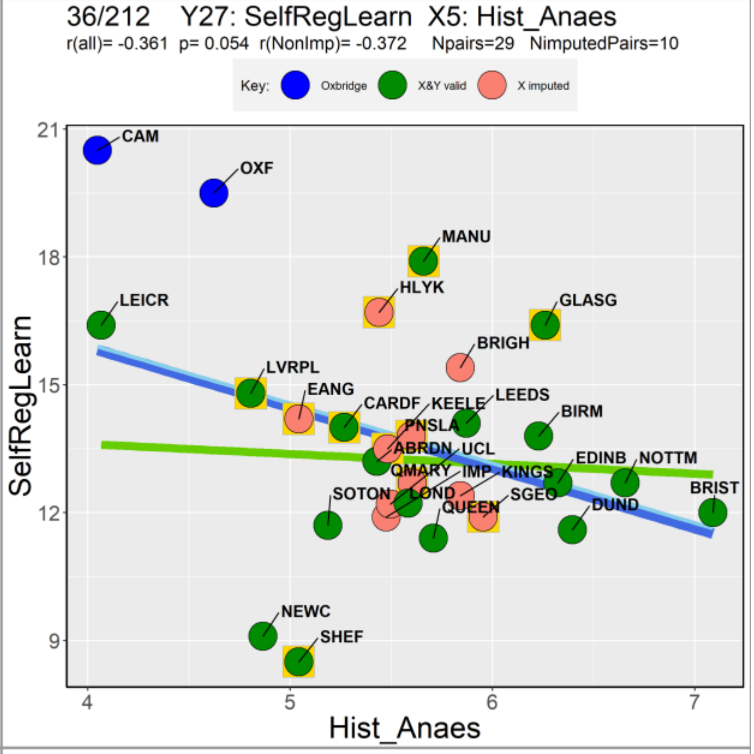
36/212 Y27: SelfRegLearn X5: Hist_Anaes
r(all)= -0.361 p= 0.054 r(NonImp)= -0.372 Npairs=29 NimputedPairs=10
Key: Oxbridge X&Y valid X imputed
SelfRegLearn
Hist_Anaes
9
12
15
18
21
4
5
6
7
CAM
OXF
MANU
HLYK
GLASG
LEICR
BRIGH
LVRPL
EANG
CARDF
KEELE
LEEDS
BIRM
PNSLA
ABRDN
UCL
EDINB
NOTTM
QMARY
IMP
KINGS
BRIST
SOTON
LOND
SGEO
DUND
QUEEN
NEWC
SHEF

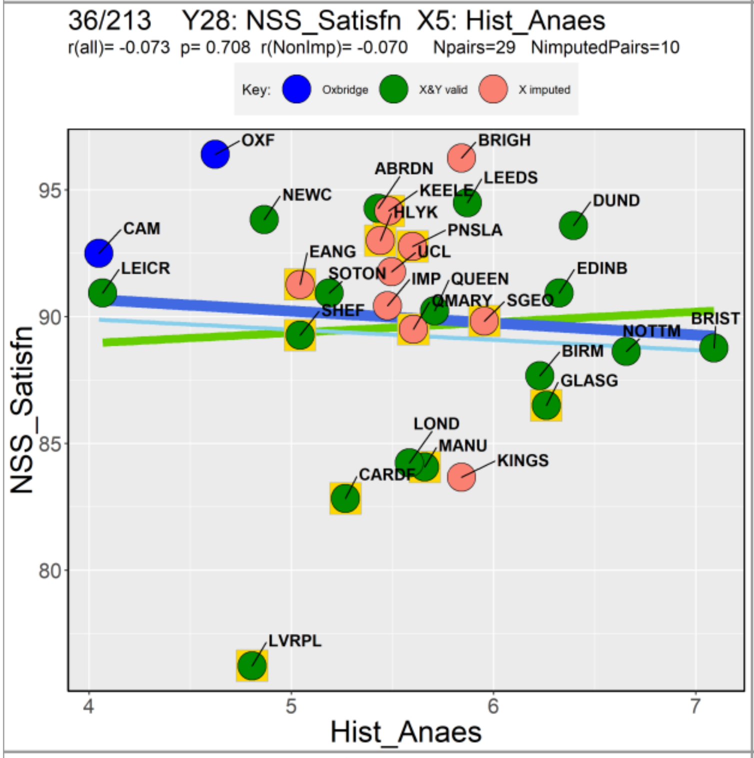
36/213 Y28: NSS_Satisfn X5: Hist_Anaes
r(all)= -0.073 p= 0.708 r(NonImp)= -0.070 Npairs=29 NimputedPairs=10
Key: Oxbridge X&Y valid X imputed
NSS_Satisfn
Hist_Anaes
80
85
90
95
4
5
6
7
OXF
BRIGH
ABRDN
LEEDS
NEWC
KEELE
DUND
CAM
HLYK
PNSLA
EANG
UCL
LEICR
SOTON
IMP
QUEEN
EDINB
SHEF
QMARY
SGEO
BRIST
NOTTM
BIRM
GLASG
LOND
MANU
KINGS
CARDF
LVRPL

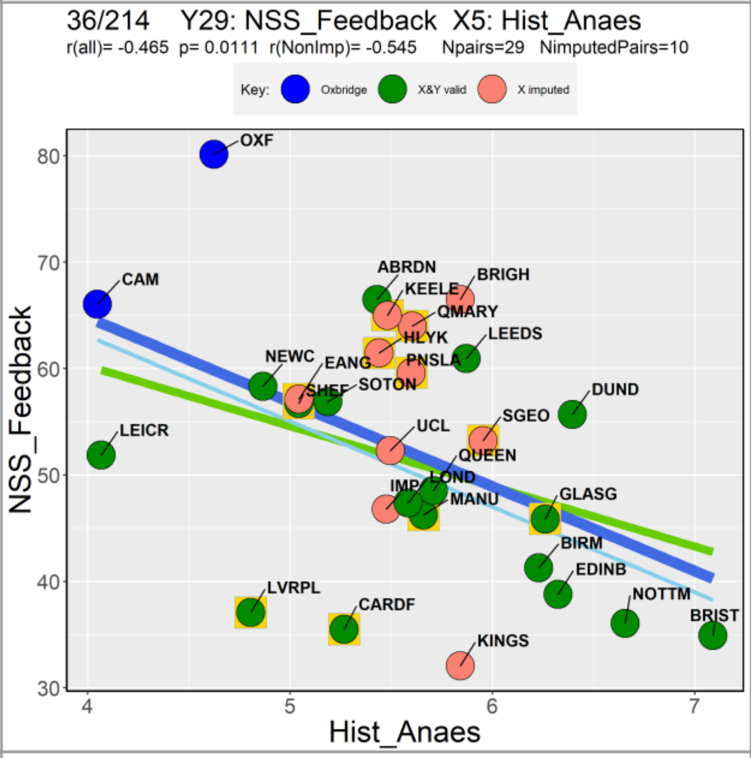
36/214 Y29: NSS_Feedback X5: Hist_Anaes
r(all)= -0.465 p= 0.0111 r(NonImp)= -0.545 Npairs=29 NimputedPairs=10
Key: Oxbridge X&Y valid X imputed
NSS_Feedback
Hist_Anaes
30
40
50
60
70
80
4
5
6
7
OXF
ABRDN
BRIGH
CAM
KEELE
QMARY
HLYK
LEEDS
NEWC
EANG
PNSLA
SHEF
SOTON
DUND
LEICR
UCL
SGEO
QUEEN
LOND
IMP
MANU
GLASG
BIRM
EDINB
LVRPL
CARDF
NOTTM
BRIST
KINGS

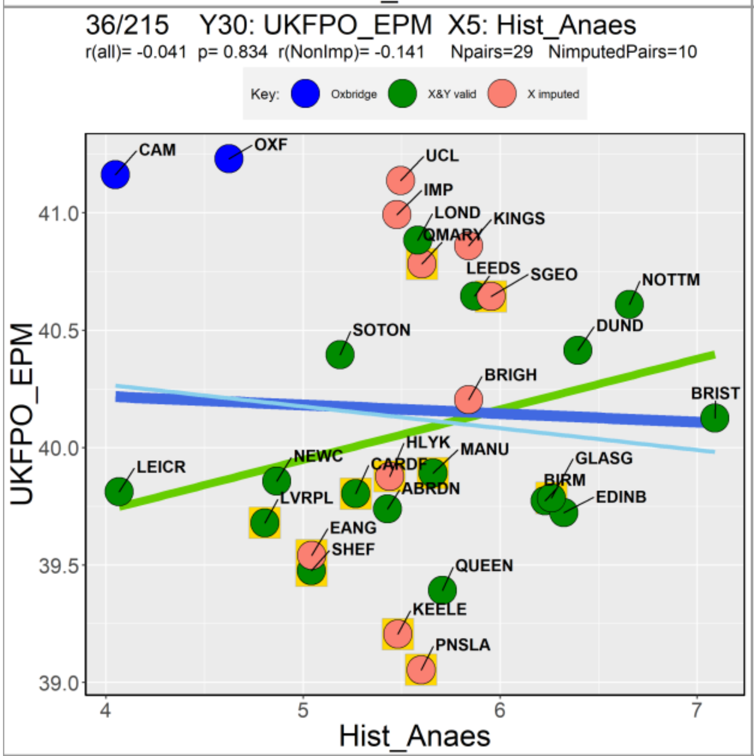
36/215 Y30: UKFPO_EPM X5: Hist_Anaes
r(all)= -0.041 p= 0.834 r(NonImp)= -0.141 Npairs=29 NimputedPairs=10
Key: Oxbridge X&Y valid X imputed
UKFPO_EPM
Hist_Anaes
39.0
39.5
40.0
40.5
41.0
4
5
6
7
CAM
OXF
UCL
IMP
LOND
KINGS
QMARY
LEEDS
SGEO
NOTTM
SOTON
DUND
BRIGH
BRIST
HLYK
MANU
NEWC
CARDF
GLASG
LEICR
LVRPL
ABRDN
BIRM
EDINB
EANG
SHEF
QUEEN
KEELE
PNSLA

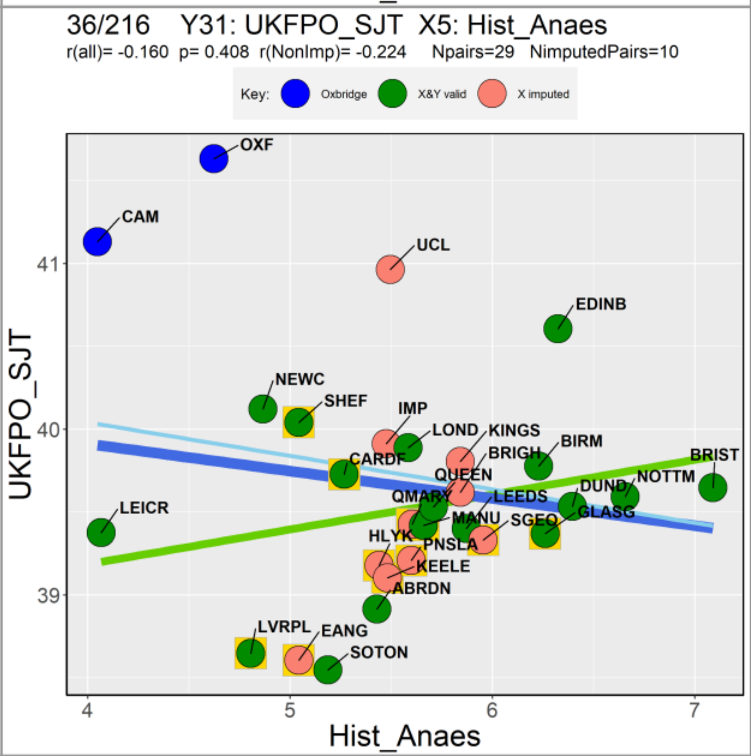
36/216 Y31: UKFPO_SJT X5: Hist_Anaes
r(all)= -0.160 p= 0.408 r(NonImp)= -0.224 Npairs=29 NimputedPairs=10
Key: Oxbridge X&Y valid X imputed
UKFPO_SJT
Hist_Anaes
39
40
41
4
5
6
7
OXF
CAM
UCL
EDINB
NEWC
SHEF
IMP
LOND
KINGS
BIRM
CARDF
BRIGH
BRIST
QUEEN
DUND
NOTTM
QMARY
LEEDS
LEICR
MANU
SGEO
GLASG
HLYK
PNSLA
KEELE
ABRDN
LVRPL
EANG
SOTON

37/217 Y32: F1_Preparedness X5: Hist_Anaes
r(all)= 0.041 p= 0.832 r(NonImp)= 0.130 Npairs=29 NimputedPairs=10

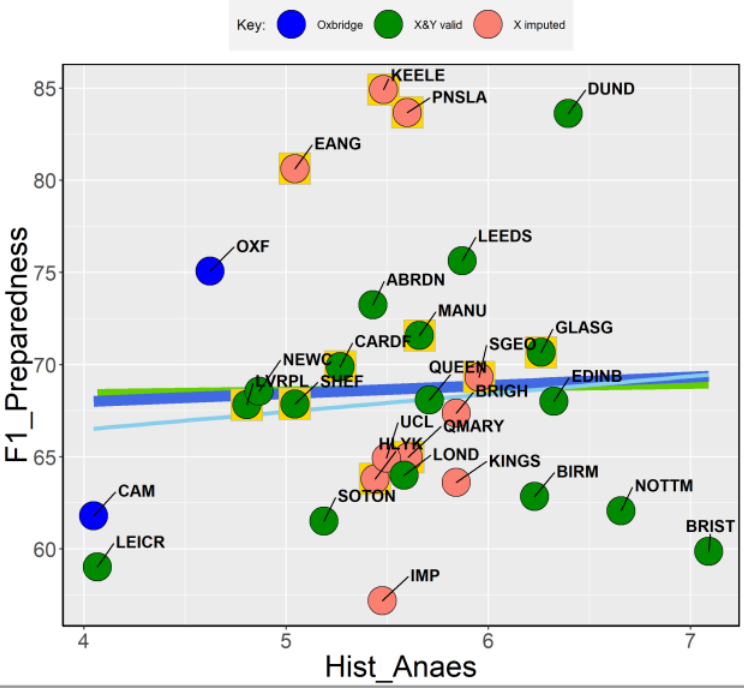

37/218 Y33: F1_Satisfn X5: Hist_Anaes
r(all)= 0.240 p= 0.209 r(NonImp)= 0.377 Npairs=29 NimputedPairs=10

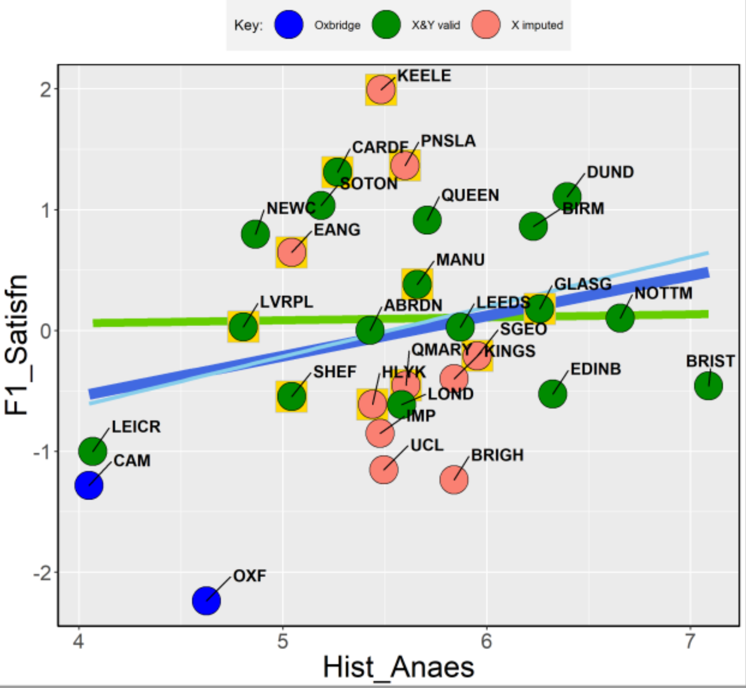

37/219 Y34: F1_Workload X5: Hist_Anaes
r(all)= -0.091 p= 0.64 r(NonImp)= -0.109 Npairs=29 NimputedPairs=10

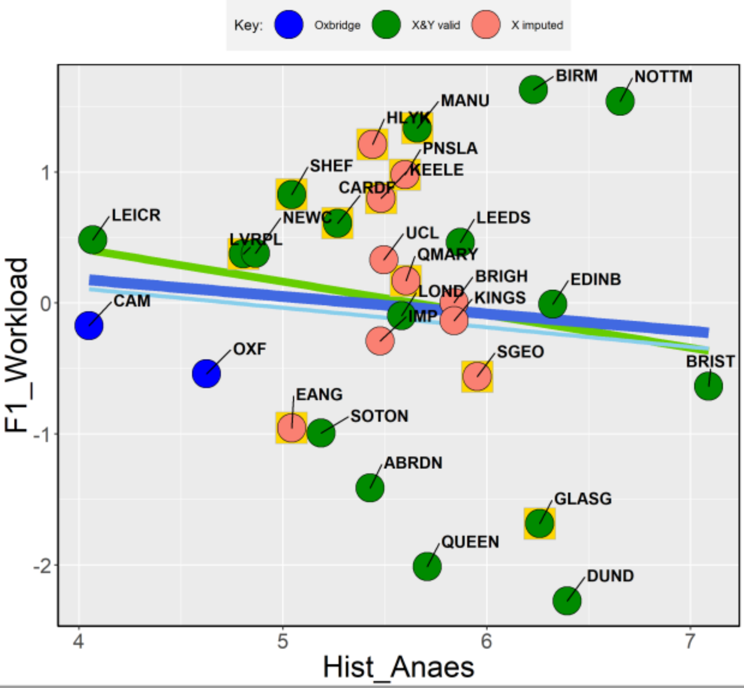

37/220 Y35: F1_Supervn X5: Hist_Anaes
r(all)= -0.277 p= 0.146 r(NonImp)= -0.355 Npairs=29 NimputedPairs=10

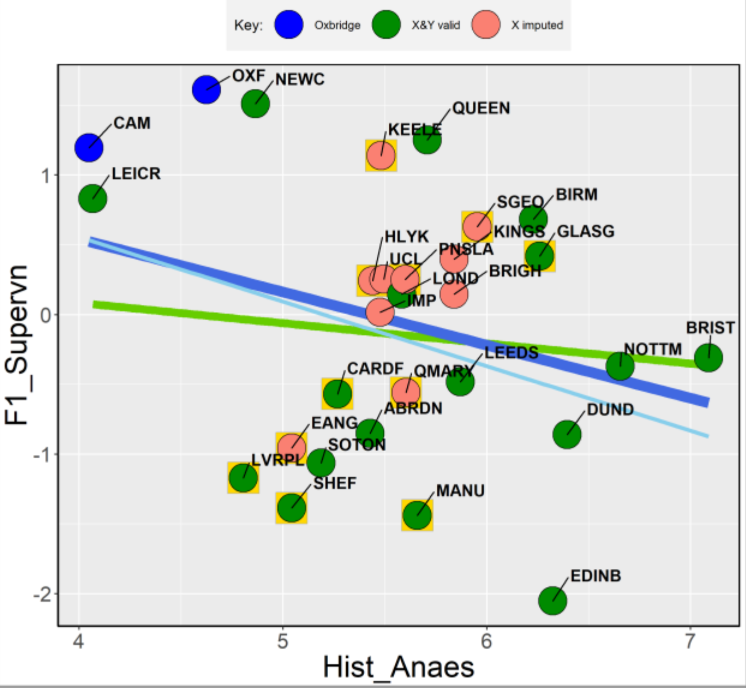

37/221 Y36: Trainee_GP X5: Hist_Anaes
r(all)= -0.114 p= 0.557 r(NonImp)= -0.108 Npairs=29 NimputedPairs=10

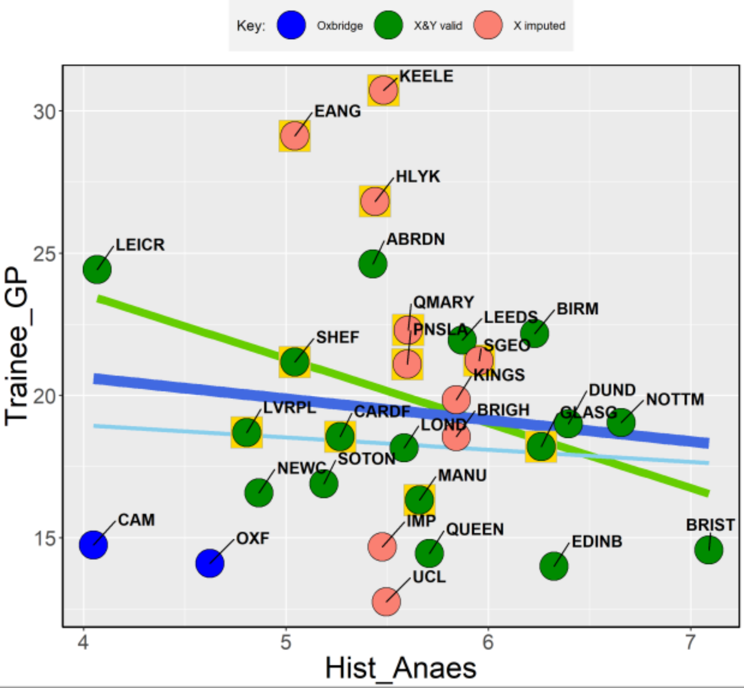

37/222 Y37: Trainee_Psyc X5: Hist_Anaes
r(all)= -0.078 p= 0.689 r(NonImp)= -0.134 Npairs=29 NimputedPairs=10

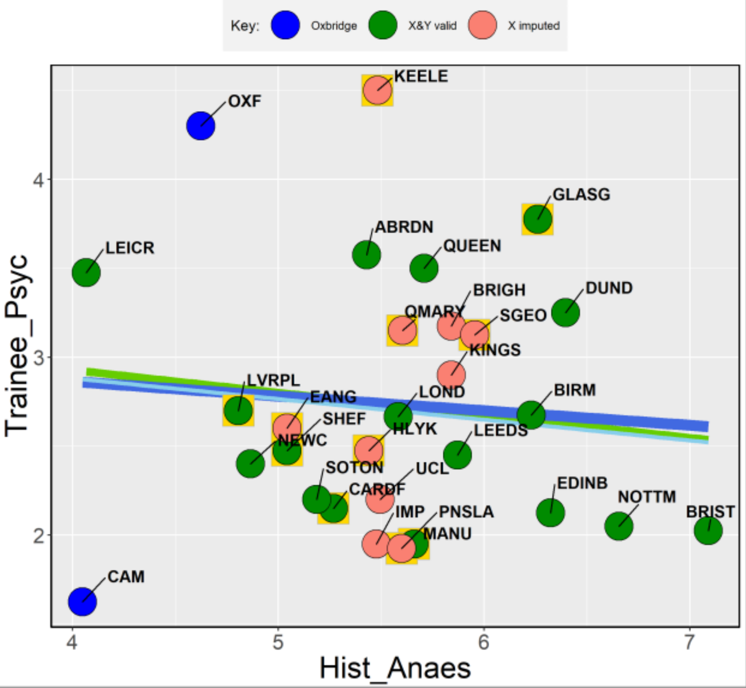

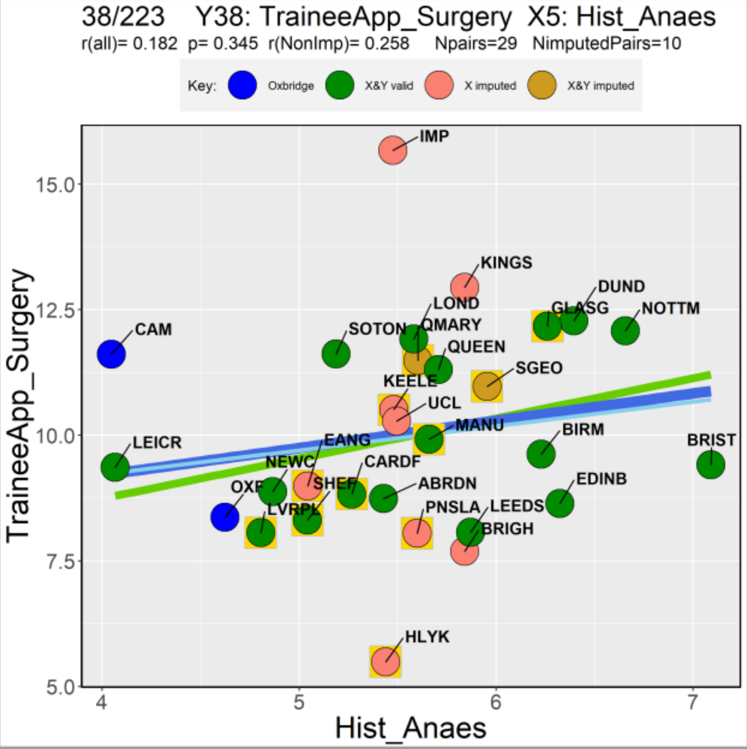
38/223 Y38: TraineeApp_Surgery X5: Hist_Anaes
r(all)= 0.182 p= 0.345 r(NonImp)= 0.258 Npairs=29 NimputedPairs=10
Key: Oxbridge X&Y valid X imputed X&Y imputed
TraineeApp_Surgery
Hist_Anaes

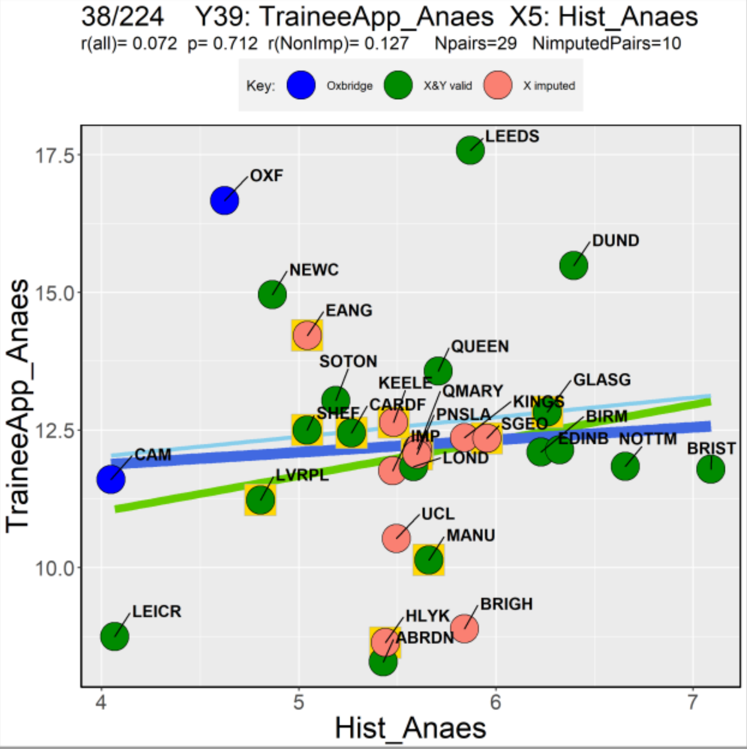
38/224 Y39: TraineeApp_Anaes X5: Hist_Anaes
r(all)= 0.072 p= 0.712 r(NonImp)= 0.127 Npairs=29 NimputedPairs=10
Key: Oxbridge X&Y valid X imputed
TraineeApp_Anaes
Hist_Anaes

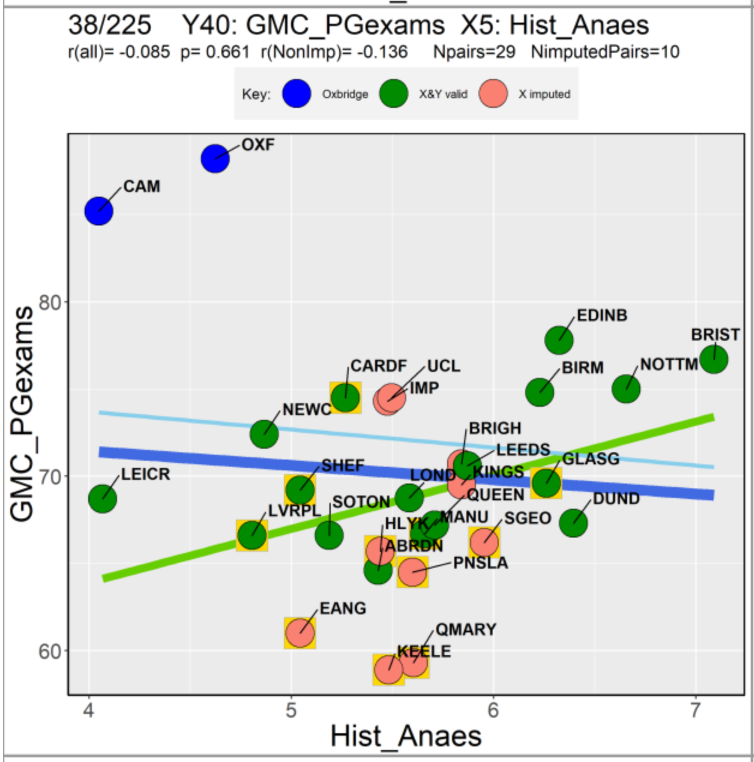
38/225 Y40: GMC_PGexams X5: Hist_Anaes
r(all)= -0.085 p= 0.661 r(NonImp)= -0.136 Npairs=29 NimputedPairs=10
Key: Oxbridge X&Y valid X imputed
GMC_PGexams
Hist_Anaes

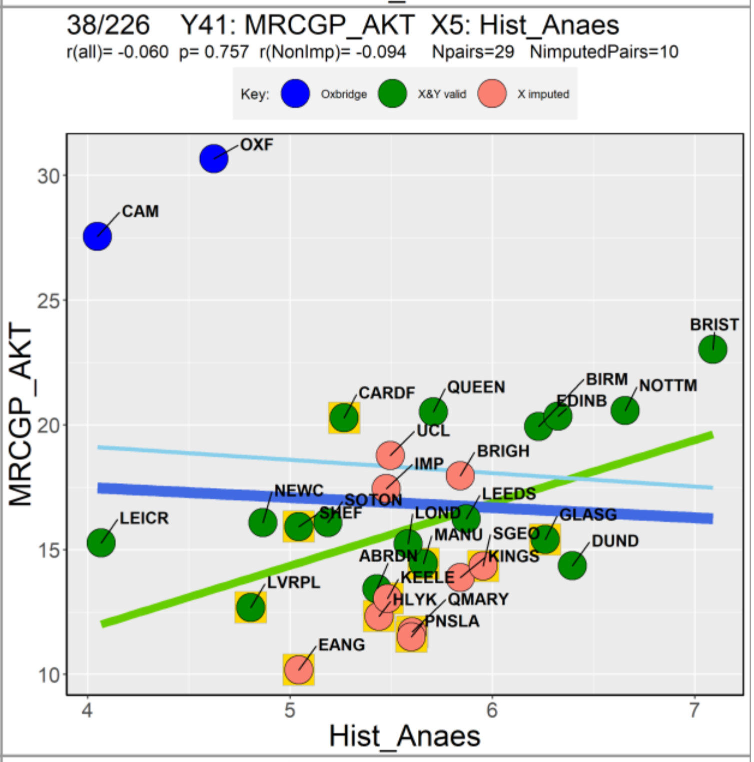
38/226 Y41: MRCGP_AKT X5: Hist_Anaes
r(all)= -0.060 p= 0.757 r(NonImp)= -0.094 Npairs=29 NimputedPairs=10
Key: Oxbridge X&Y valid X imputed
MRCGP_AKT
Hist_Anaes

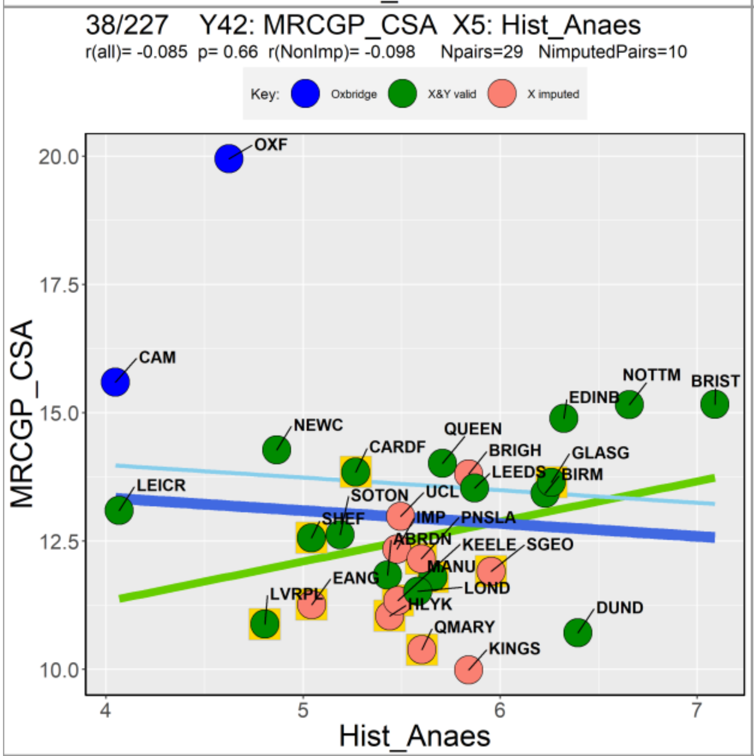
38/227 Y42: MRCGP_CSA X5: Hist_Anaes
r(all)= -0.085 p= 0.66 r(NonImp)= -0.098 Npairs=29 NimputedPairs=10
Key: Oxbridge X&Y valid X imputed
MRCGP_CSA
Hist_Anaes

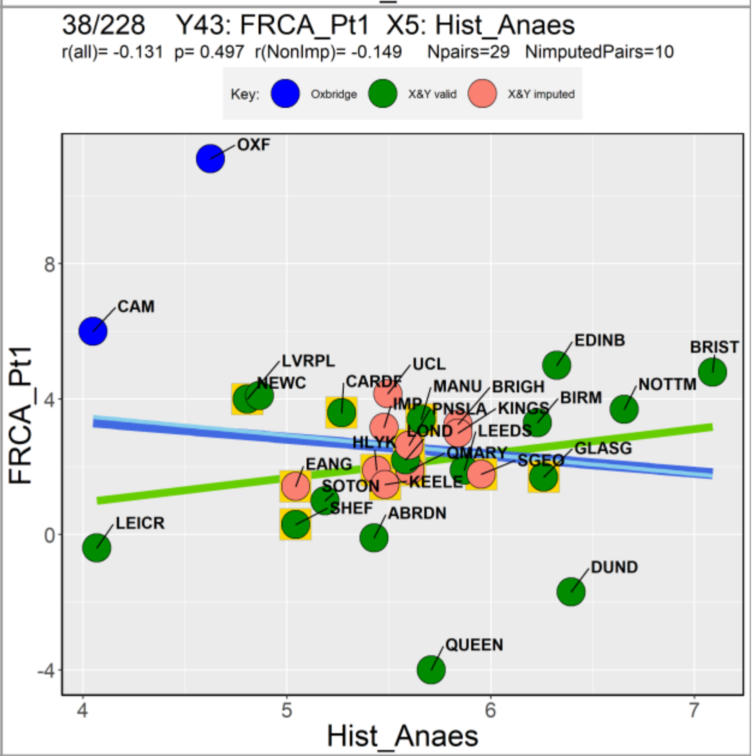
38/228 Y43: FRCA_Pt1 X5: Hist_Anaes
r(all)= -0.131 p= 0.497 r(NonImp)= -0.149 Npairs=29 NimputedPairs=10
Key: Oxbridge X&Y valid X&Y imputed
FRCA_Pt1
Hist_Anaes

39/229 Y44: MRCOG_Pt1 X5: Hist_Anaes
r(all)= 0.051 p= 0.791 r(NonImp)= 0.038 Npairs=29 NimputedPairs=10

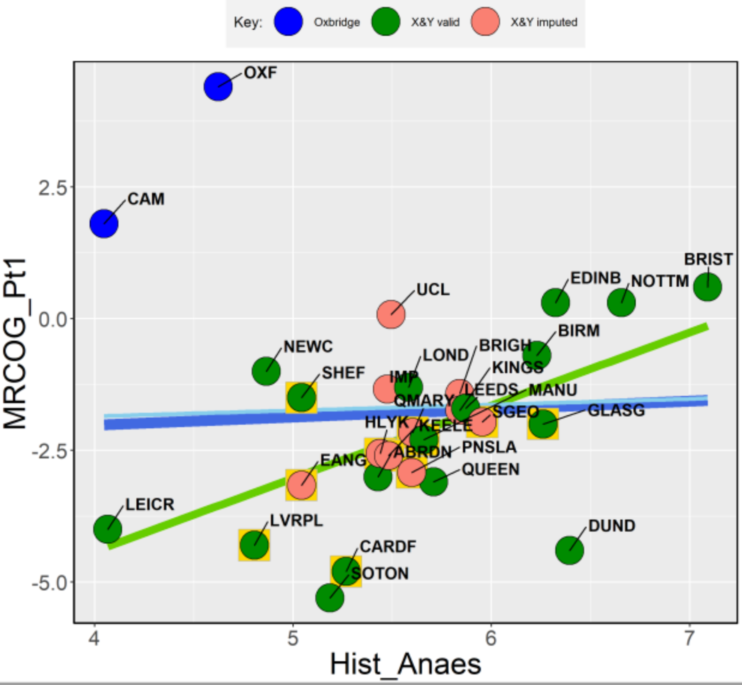

39/230 Y45: MRCOG_Pt2 X5: Hist_Anaes
r(all)= 0.054 p= 0.782 r(NonImp)= 0.080 Npairs=29 NimputedPairs=10

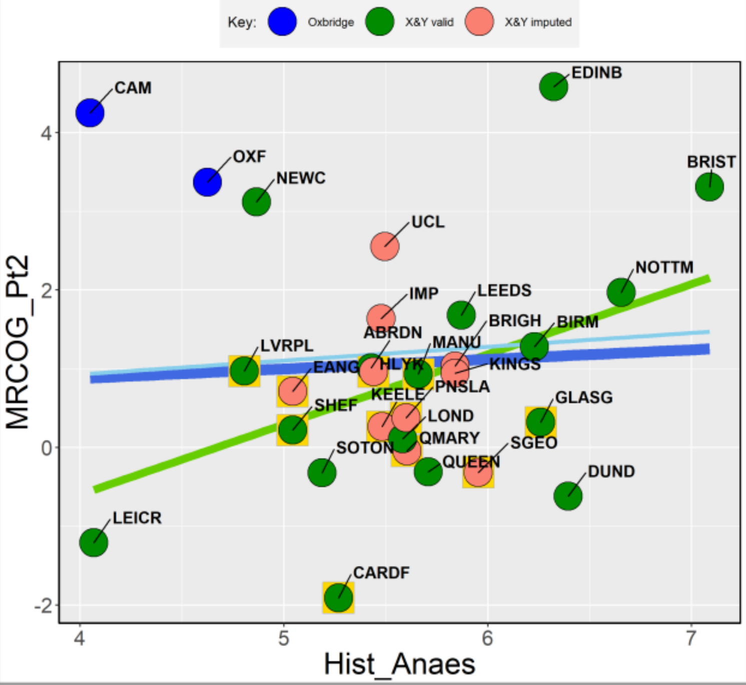

39/231 Y46: MRCP_Pt1 X5: Hist_Anaes
r(all)= -0.176 p= 0.361 r(NonImp)= -0.239 Npairs=29 NimputedPairs=10

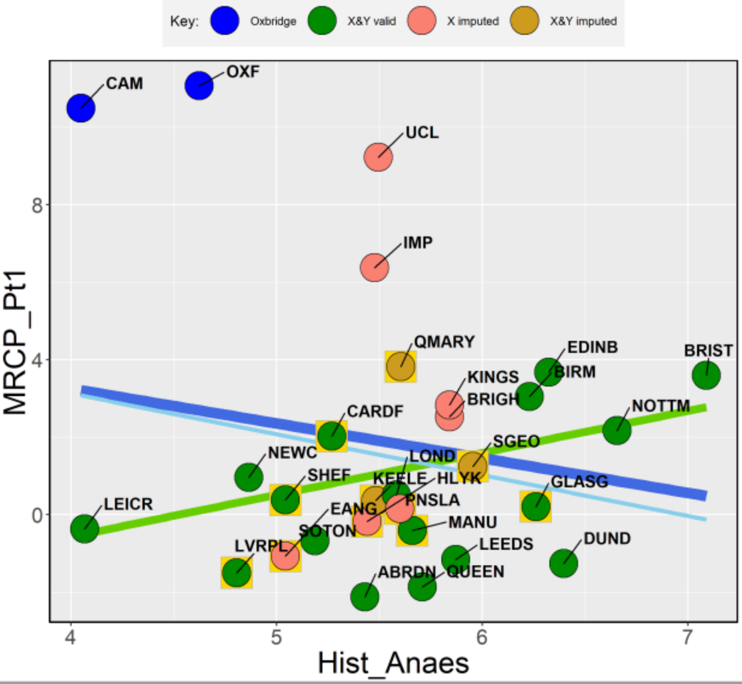

39/232 Y47: MRCP_Pt2 X5: Hist_Anaes
r(all)= -0.167 p= 0.386 r(NonImp)= -0.179 Npairs=29 NimputedPairs=10

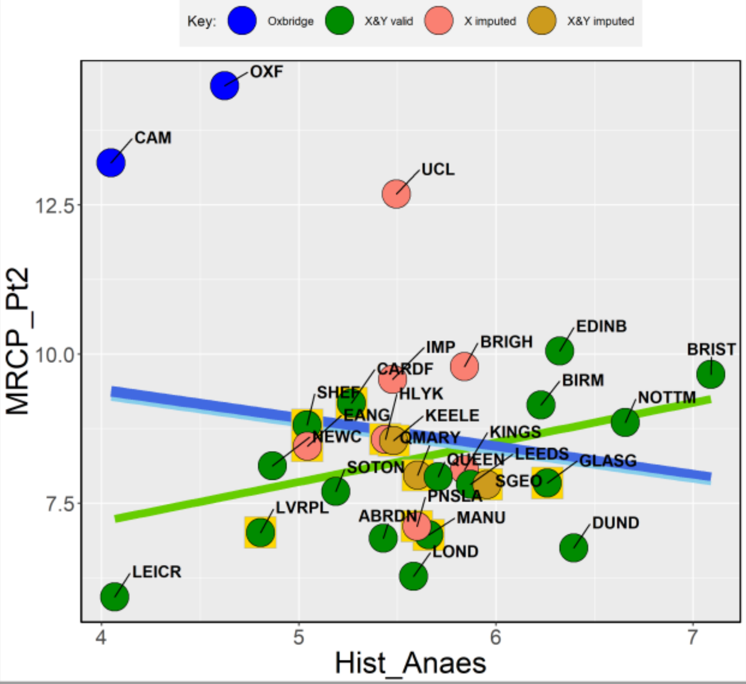

39/233 Y48: MRCP_PACES X5: Hist_Anaes
r(all)= 0.027 p= 0.889 r(NonImp)= 0.020 Npairs=29 NimputedPairs=10

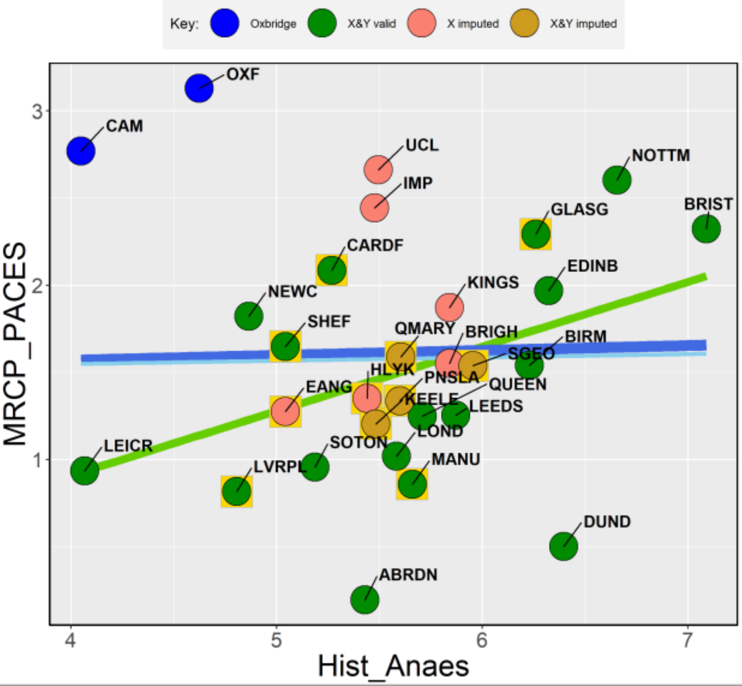

39/234 Y49: GMC_Sanctions X5: Hist_Anaes
r(all)= -0.263 p= 0.168 r(NonImp)= -0.269 Npairs=29 NimputedPairs=10

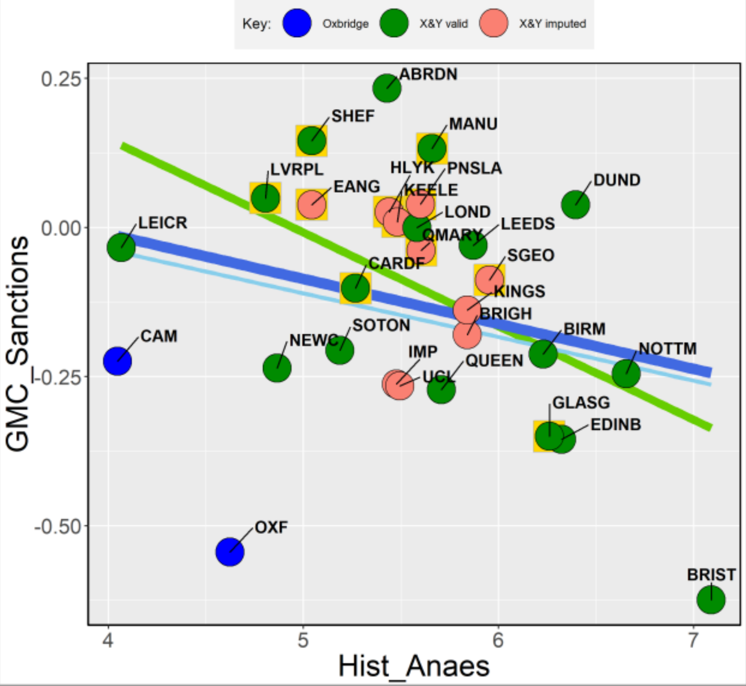

40/235 Y50: ARCP_NotExam X5: Hist_Anaes
r(all)= 0.149 p= 0.442 r(NonImp)= 0.185 Npairs=29 NimputedPairs=10

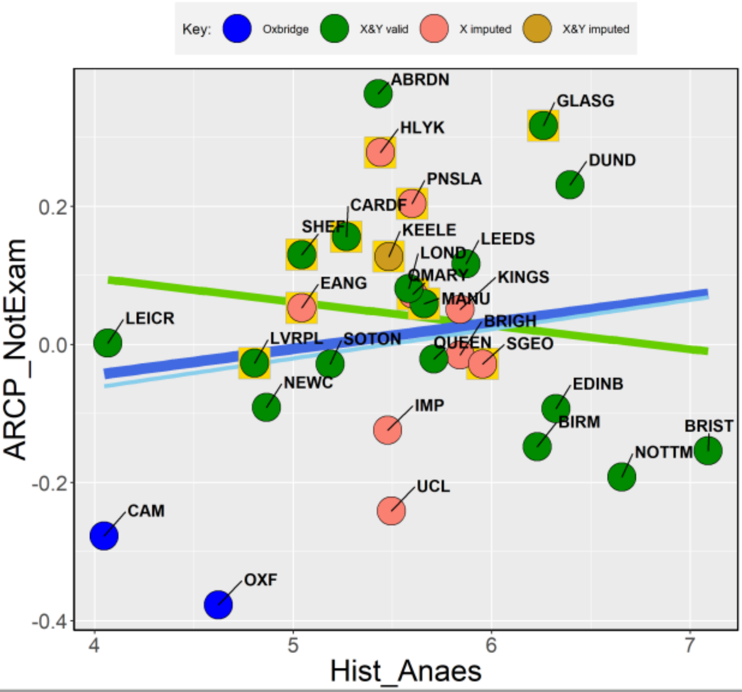

40/236 Y7: Hist_IntMed X6: Hist_OG
r(all)= -0.007 p= 0.97 r(NonImp)= -0.035 Npairs=29 NimputedPairs=10

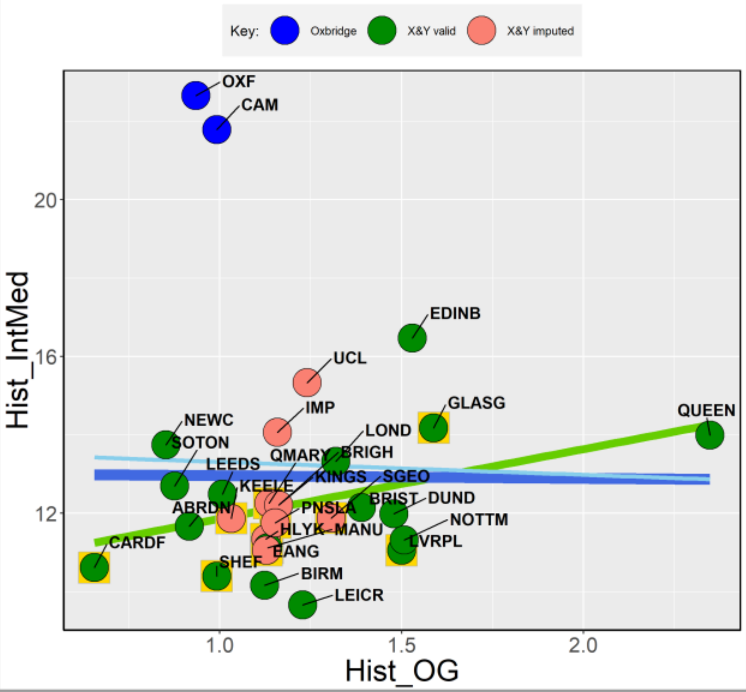

40/237 Y8: Hist_Surgery X6: Hist_OG
r(all)= -0.125 p= 0.519 r(NonImp)= -0.148 Npairs=29 NimputedPairs=10

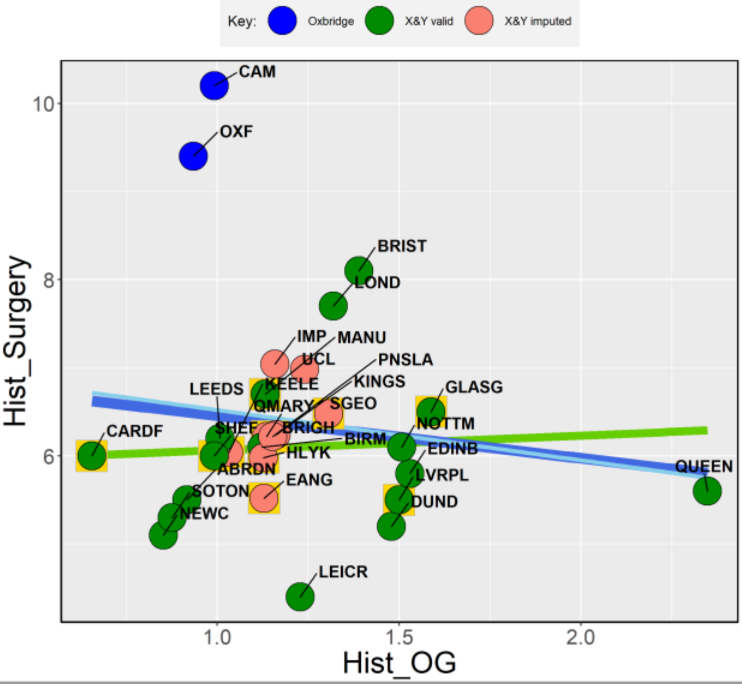

40/238 Y9: Post2000 X6: Hist_OG
r(all)= -0.127 p= 0.513 r(NonImp)= NA Npairs=29 NimputedPairs=10

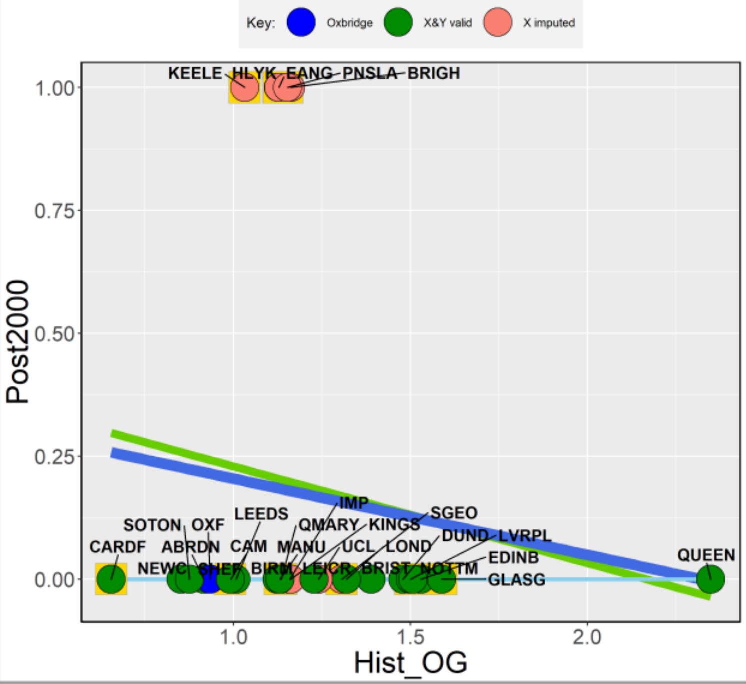

40/239 Y10: REF X6: Hist_OG
r(all)= -0.300 p= 0.114 r(NonImp)= -0.364 Npairs=29 NimputedPairs=10

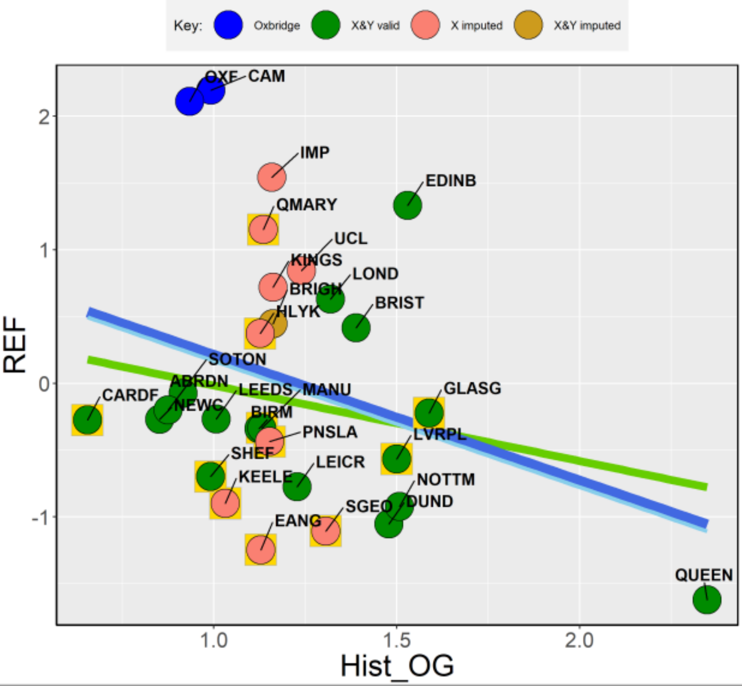

40/240 Y11: PBL_School X6: Hist_OG
r(all)= -0.119 p= 0.538 r(NonImp)= -0.090 Npairs=29 NimputedPairs=10

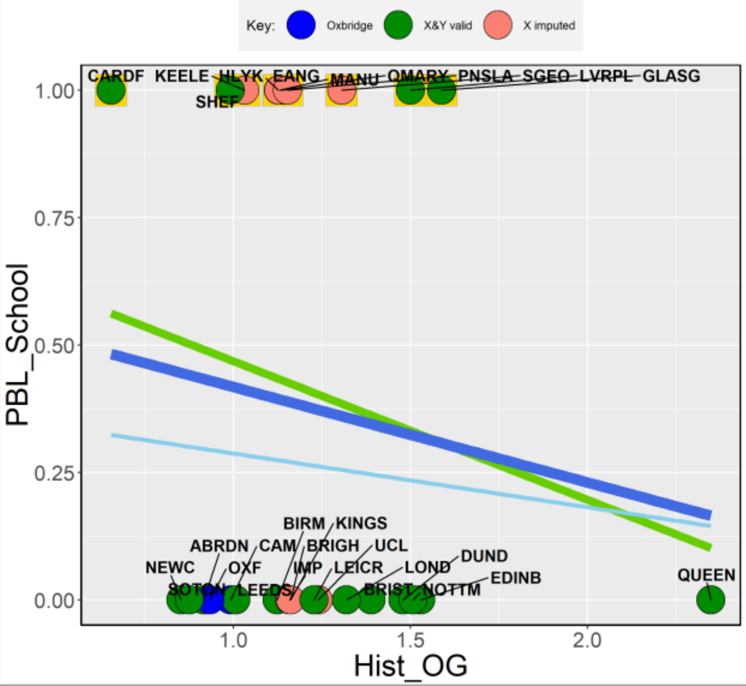

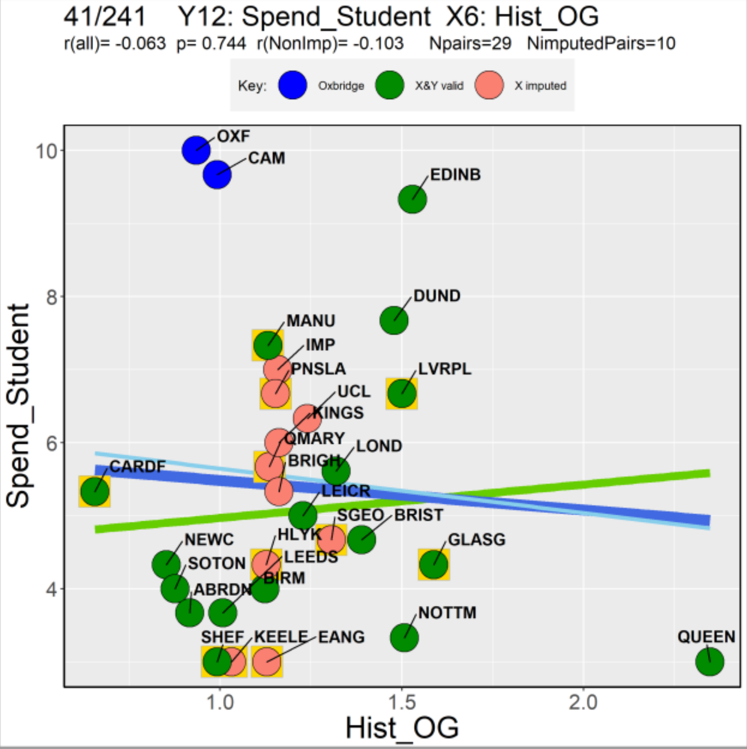
41/241 Y12: Spend_Student X6: Hist_OG
r(all)= -0.063 p= 0.744 r(NonImp)= -0.103 Npairs=29 NimputedPairs=10
Key: Oxbridge X&Y valid X imputed
Spend_Student
Hist_OG
10
8
6
4
1.0
1.5
2.0
OXF
CAM
EDINB
DUND
MANU
IMP
PNSLA
LVRPL
UCL
KINGS
QMARY
LOND
BRIGH
CARDF
LEICR
SGEO
BRIST
NEWC
HLYK
GLASG
SOTON
LEEDS
ABRDN
BIRM
NOTTM
SHEF
KEELE
EANG
QUEEN

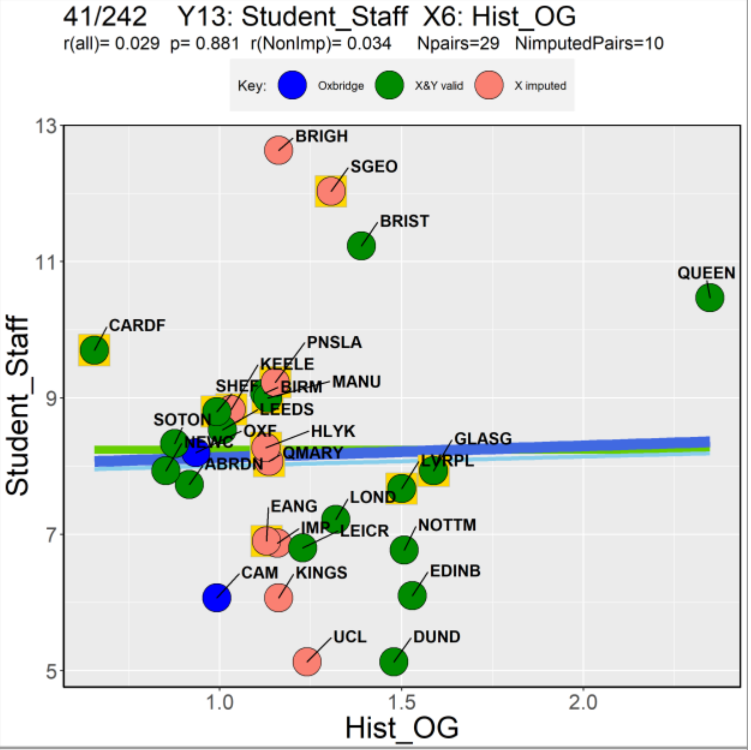
41/242 Y13: Student_Staff X6: Hist_OG
r(all)= 0.029 p= 0.881 r(NonImp)= 0.034 Npairs=29 NimputedPairs=10
Key: Oxbridge X&Y valid X imputed
Student_Staff
Hist_OG
13
11
9
7
5
1.0
1.5
2.0
BRIGH
SGEO
BRIST
QUEEN
CARDF
PNSLA
KEELE
MANU
LEEDS
SOTON
OXF
HLYK
NEWC
QMARY
GLASG
ABRDN
LVRPL
EANG
LOND
IMP
LEICR
NOTTM
CAM
KINGS
EDINB
UCL
DUND

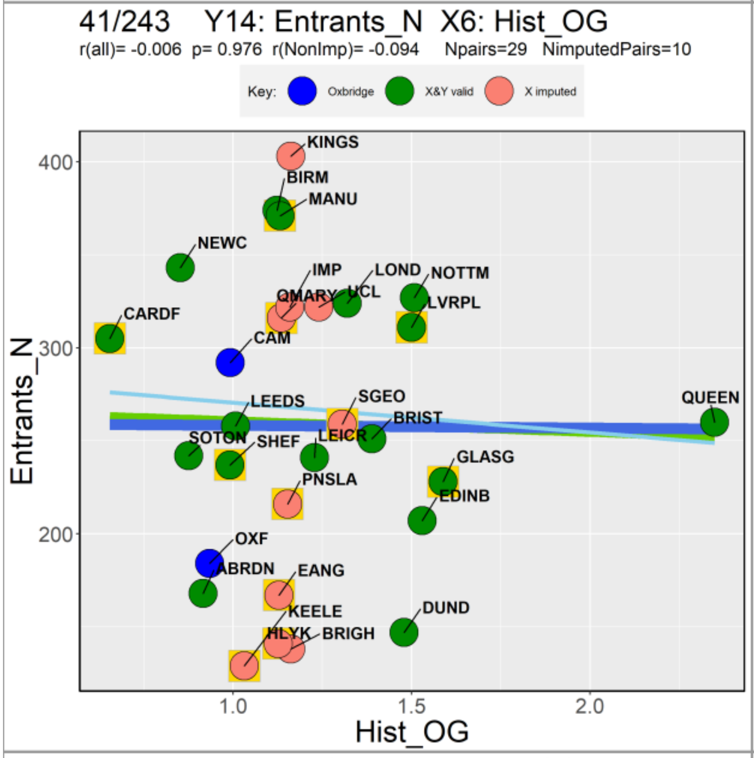
41/243 Y14: Entrants_N X6: Hist_OG
r(all)= -0.006 p= 0.976 r(NonImp)= -0.094 Npairs=29 NimputedPairs=10
Key: Oxbridge X&Y valid X imputed
Entrants_N
Hist_OG
400
300
200
1.0
1.5
2.0
KINGS
BIRM
MANU
NEWC
IMP
LOND
NOTTM
QMARY
UCL
LVRPL
CARDF
CAM
LEEDS
SGEO
QUEEN
BRIST
SOTON
SHEF
LEICR
GLASG
PNSLA
EDINB
OXF
ABRDN
EANG
KEELE
DUND
HLYK
BRIGH

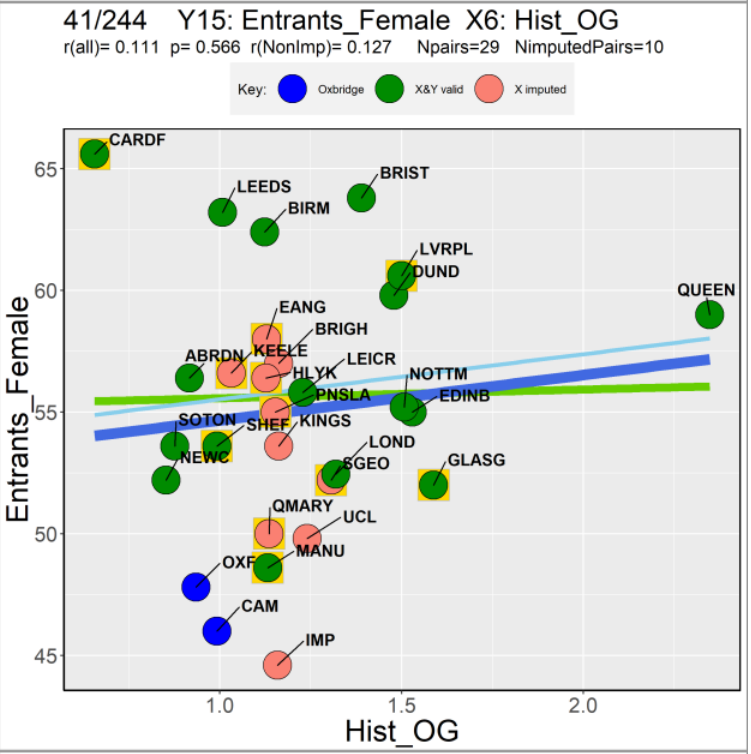
41/244 Y15: Entrants_Female X6: Hist_OG
r(all)= 0.111 p= 0.566 r(NonImp)= 0.127 Npairs=29 NimputedPairs=10
Key: Oxbridge X&Y valid X imputed
Entrants_Female
Hist_OG
65
60
55
50
45
1.0
1.5
2.0
CARDF
LEEDS
BRIST
BIRM
LVRPL
DUND
QUEEN
EANG
BRIGH
ABRDN
KEELE
LEICR
HLYK
NOTTM
PNSLA
EDINB
SOTON
SHEF
KINGS
NEWC
LOND
SGEO
GLASG
QMARY
UCL
MANU
OXF
CAM
IMP

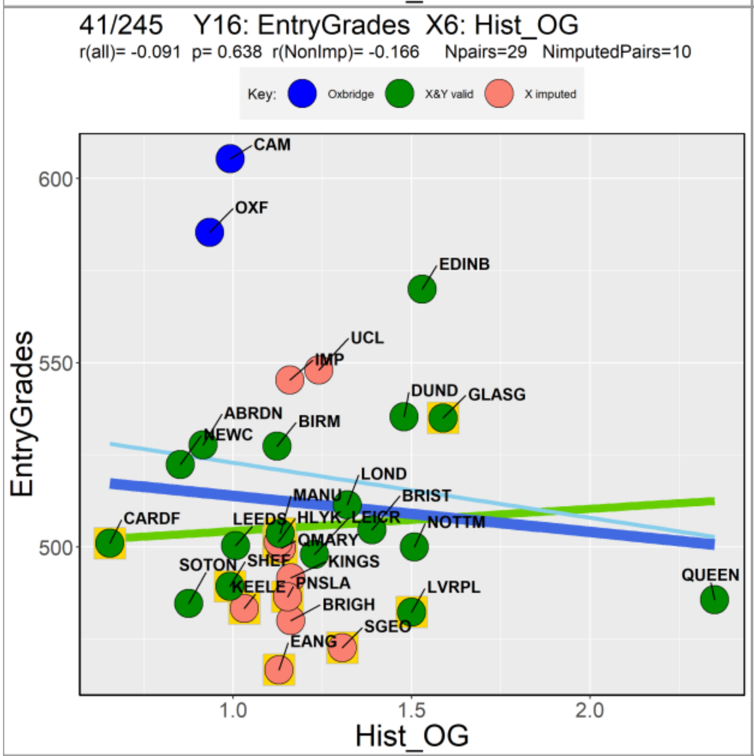
41/245 Y16: EntryGrades X6: Hist_OG
r(all)= -0.091 p= 0.638 r(NonImp)= -0.166 Npairs=29 NimputedPairs=10
Key: Oxbridge X&Y valid X imputed
EntryGrades
Hist_OG
600
550
500
1.0
1.5
2.0
CAM
OXF
EDINB
UCL
IMP
DUND
GLASG
ABRDN
BIRM
NEWC
LOND
MANU
BRIST
CARDF
LEEDS
HLYK
LEICR
NOTTM
QMARY
SOTON
SHEF
KINGS
KEELE
PNSLA
LVRPL
BRIGH
QUEEN
SGEO
EANG

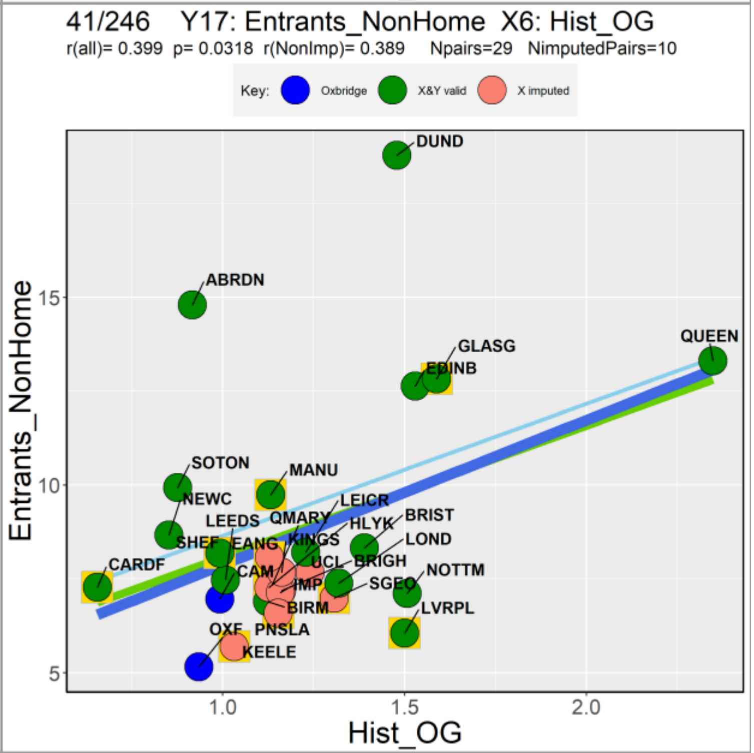
41/246 Y17: Entrants_NonHome X6: Hist_OG
r(all)= 0.399 p= 0.0318 r(NonImp)= 0.389 Npairs=29 NimputedPairs=10
Key: Oxbridge X&Y valid X imputed
Entrants_NonHome
Hist_OG
15
10
5
1.0
1.5
2.0
DUND
ABRDN
GLASG
EDINB
QUEEN
SOTON
MANU
NEWC
LEICR
LEEDS
QMARY
BRIST
HLYK
SHEF
EANG
KINGS
LOND
CARDF
CAM
UCL
BRIGH
IMP
SGEO
NOTTM
BIRM
LVRPL
OXF
PNSLA
KEELE

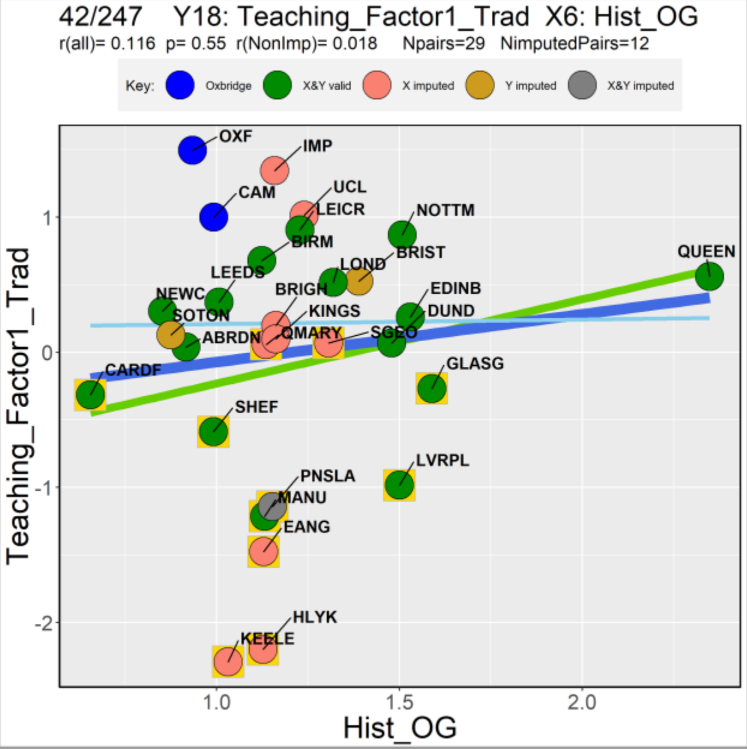
42/247 Y18: Teaching_Factor1_Trad X6: Hist_OG
r(all)= 0.116 p= 0.55 r(NonImp)= 0.018 Npairs=29 NimputedPairs=12
Key: Oxbridge X&Y valid X imputed Y imputed X&Y imputed
Teaching_Factor1_Trad
Hist_OG
OXF
IMP
CAM
UCL
LEICR
NOTTM
BIRM
LEEDS
BRIST
LOND
QUEEN
NEWC
BRIGH
EDINB
SOTON
KINGS
DUND
ABRDN
QMARY
SGEO
CARDF
GLASG
SHEF
LVRPL
PNSLA
MANU
EANG
HLYK
KEELE

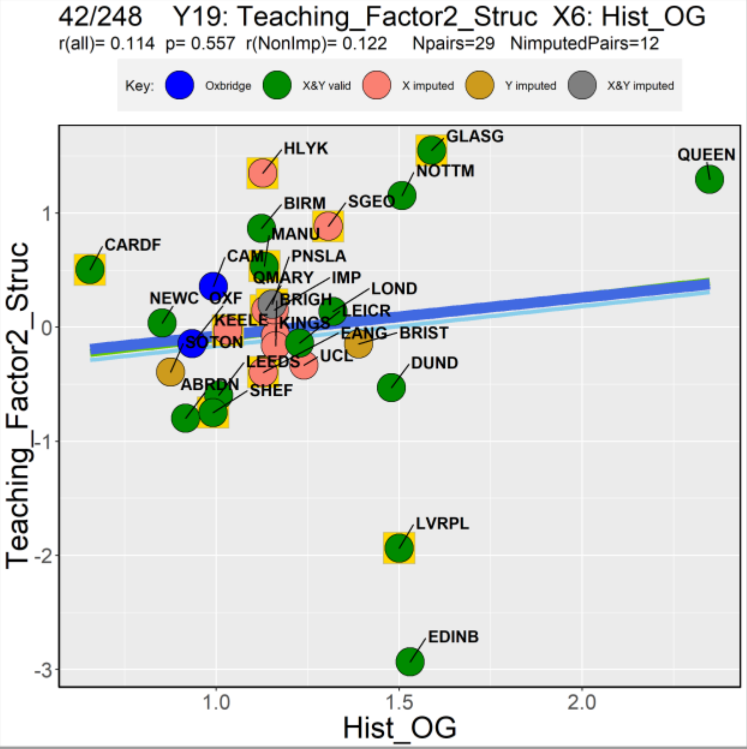
42/248 Y19: Teaching_Factor2_Struc X6: Hist_OG
r(all)= 0.114 p= 0.557 r(NonImp)= 0.122 Npairs=29 NimputedPairs=12
Key: Oxbridge X&Y valid X imputed Y imputed X&Y imputed
Teaching_Factor2_Struc
Hist_OG
GLASG
HLYK
QUEEN
NOTTM
BIRM
SGEO
CARDF
MANU
CAM
PNSLA
QMARY
IMP
LOND
NEWC
OXF
BRIGH
LEICR
KEELE
KINGS
SOTON
EANG
BRIST
UCL
LEEDS
DUND
ABRDN
SHEF
LVRPL
EDINB

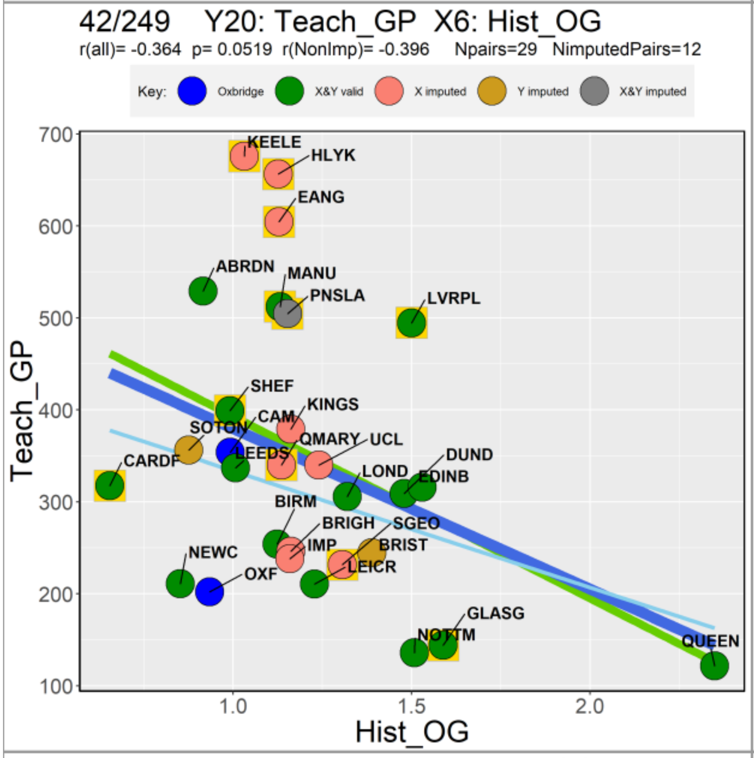
42/249 Y20: Teach_GP X6: Hist_OG
r(all)= -0.364 p= 0.0519 r(NonImp)= -0.396 Npairs=29 NimputedPairs=12
Key: Oxbridge X&Y valid X imputed Y imputed X&Y imputed
Teach_GP
Hist_OG
KEELE
HLYK
EANG
ABRDN
MANU
PNSLA
LVRPL
SHEF
KINGS
CAM
SOTON
QMARY
UCL
LEEDS
CARDF
DUND
LOND
EDINB
BIRM
BRIGH
SGEO
IMP
BRIST
NEWC
LEICR
OXF
GLASG
NOTTM
QUEEN

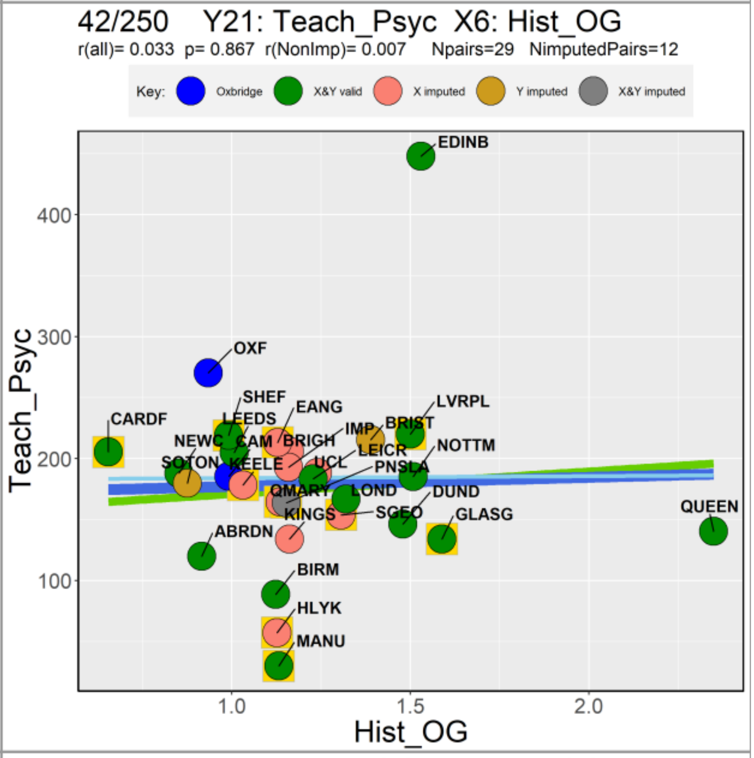
42/250 Y21: Teach_Psyc X6: Hist_OG
r(all)= 0.033 p= 0.867 r(NonImp)= 0.007 Npairs=29 NimputedPairs=12
Key: Oxbridge X&Y valid X imputed Y imputed X&Y imputed
Teach_Psyc
Hist_OG
EDINB
OXF
SHEF
EANG
LVRPL
CARDF
LEEDS
IMP
BRIST
NEWC
CAM
BRIGH
LEICR
NOTTM
SOTON
KEELE
UCL
PNSLA
QMARY
LOND
DUND
KINGS
SGEO
GLASG
QUEEN
ABRDN
BIRM
HLYK
MANU

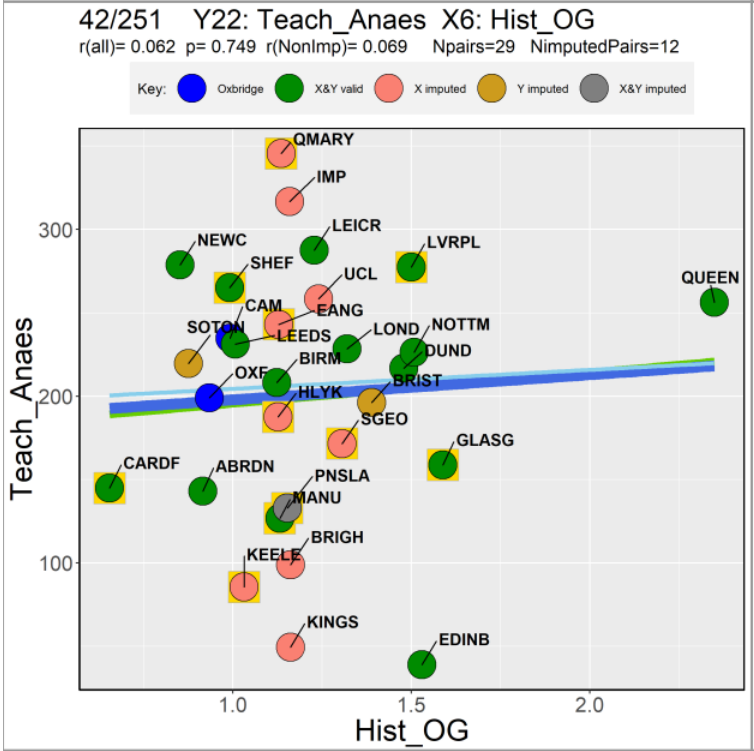
42/251 Y22: Teach_Anaes X6: Hist_OG
r(all)= 0.062 p= 0.749 r(NonImp)= 0.069 Npairs=29 NimputedPairs=12
Key: Oxbridge X&Y valid X imputed Y imputed X&Y imputed
Teach_Anaes
Hist_OG
QMARY
IMP
LEICR
NEWC
LVRPL
SHEF
UCL
QUEEN
CAM
EANG
SOTON
LEEDS
LOND
NOTTM
DUND
BIRM
OXF
BRIST
HLYK
SGEO
GLASG
CARDF
ABRDN
PNSLA
MANU
BRIGH
KEELE
KINGS
EDINB

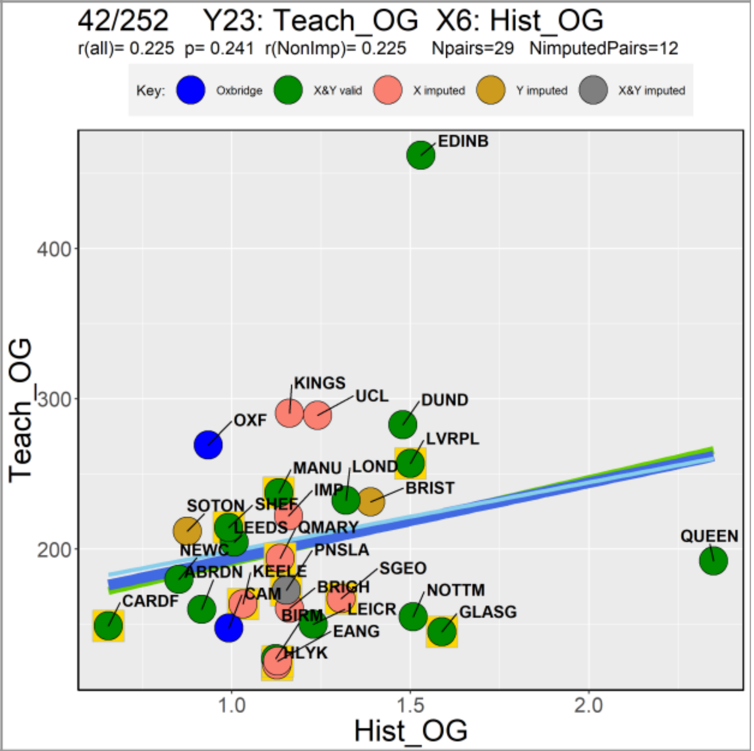
42/252 Y23: Teach_OG X6: Hist_OG
r(all)= 0.225 p= 0.241 r(NonImp)= 0.225 Npairs=29 NimputedPairs=12
Key: Oxbridge X&Y valid X imputed Y imputed X&Y imputed
Teach_OG
Hist_OG
EDINB
KINGS
UCL
DUND
OXF
LVRPL
MANU
LOND
IMP
BRIST
SOTON
SHEF
LEEDS
QMARY
QUEEN
NEWC
PNSLA
ABRDN
KEELE
SGEO
CAM
BRIGH
NOTTM
CARDF
BIRM
LEICR
GLASG
EANG
HLYK

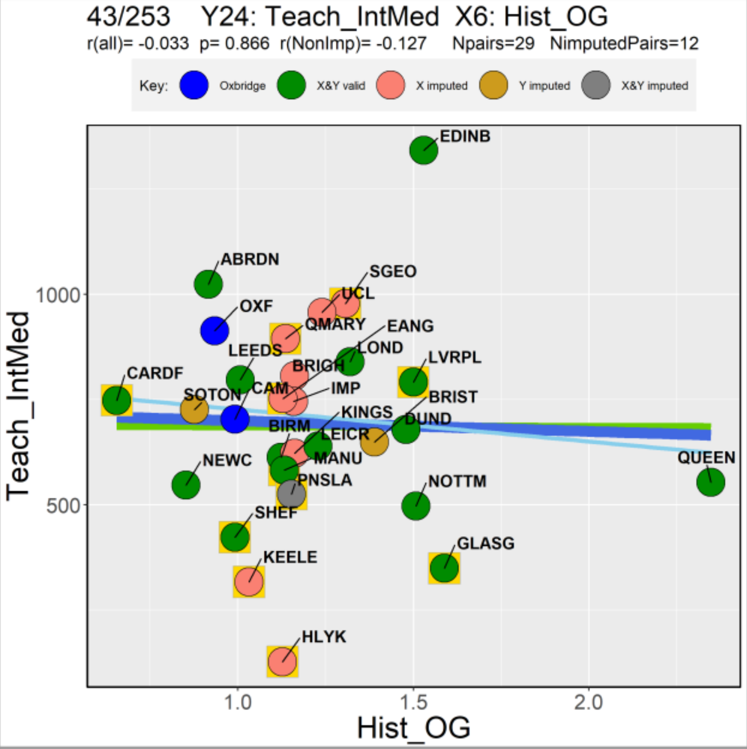
43/253 Y24: Teach_IntMed X6: Hist_OG
r(all)= -0.033 p= 0.866 r(NonImp)= -0.127 Npairs=29 NimputedPairs=12
Key: Oxbridge X&Y valid X imputed Y imputed X&Y imputed
Teach_IntMed
Hist_OG
EDINB
ABRDN
SGEO
UCL
OXF
QMARY
EANG
LEEDS
LOND
LVRPL
CARDF
BRIGH
SOTON
CAM
IMP
BRIST
KINGS
DUND
BIRM
LEICR
MANU
QUEEN
NEWC
PNSLA
NOTTM
SHEF
GLASG
KEELE
HLYK

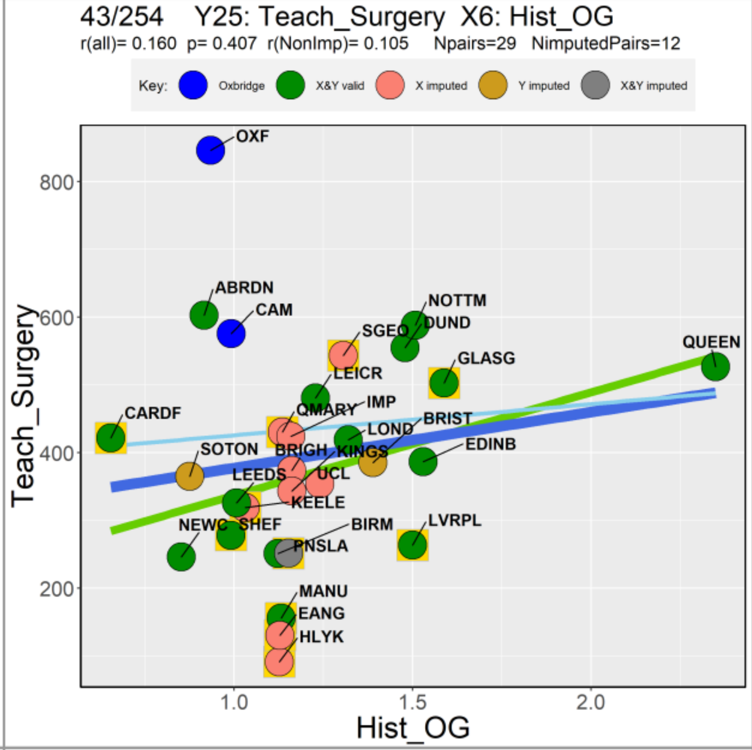
43/254 Y25: Teach_Surgery X6: Hist_OG
r(all)= 0.160 p= 0.407 r(NonImp)= 0.105 Npairs=29 NimputedPairs=12
Key: Oxbridge X&Y valid X imputed Y imputed X&Y imputed
Teach_Surgery
Hist_OG
OXF
ABRDN
CAM
NOTTM
DUND
SGEO
QUEEN
GLASG
LEICR
QMARY
IMP
CARDF
LOND
BRIST
SOTON
BRIGH
KINGS
EDINB
LEEDS
UCL
KEELE
NEWC
SHEF
BIRM
LVRPL
PNSLA
MANU
EANG
HLYK

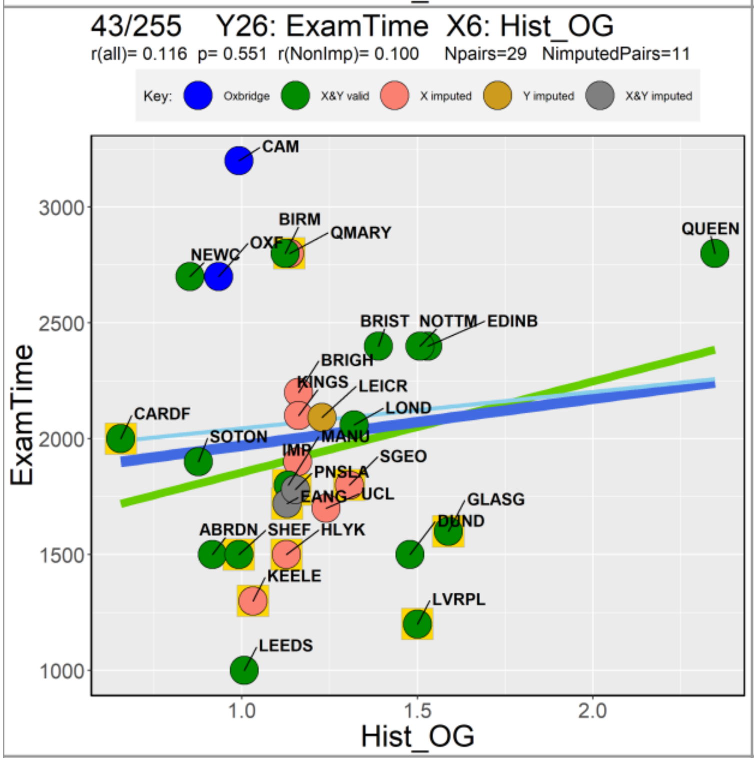
43/255 Y26: ExamTime X6: Hist_OG
r(all)= 0.116 p= 0.551 r(NonImp)= 0.100 Npairs=29 NimputedPairs=11
Key: Oxbridge X&Y valid X imputed Y imputed X&Y imputed
ExamTime
Hist_OG
CAM
BIRM
QMARY
QUEEN
NEWC
OXF
BRIST
NOTTM
EDINB
BRIGH
KINGS
LEICR
CARDF
LOND
SOTON
MANU
IMP
SGEO
PNSLA
EANG
UCL
GLASG
DUND
ABRDN
SHEF
HLYK
KEELE
LVRPL
LEEDS

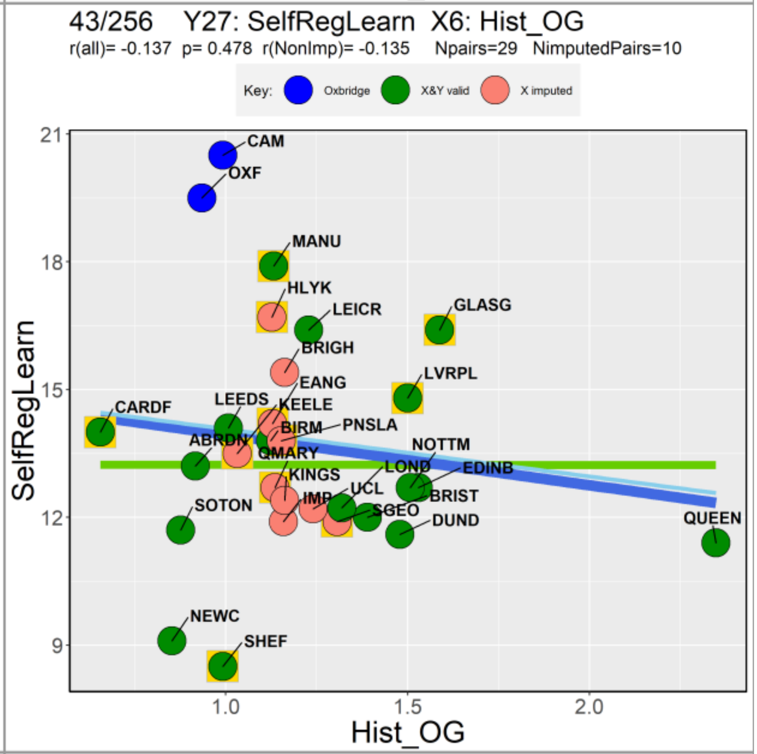
43/256 Y27: SelfRegLearn X6: Hist_OG
r(all)= -0.137 p= 0.478 r(NonImp)= -0.135 Npairs=29 NimputedPairs=10
Key: Oxbridge X&Y valid X imputed
SelfRegLearn
Hist_OG
CAM
OXF
MANU
HLYK
LEICR
GLASG
BRIGH
EANG
LVRPL
CARDF
LEEDS
KEELE
BIRM
PNSLA
ABRDN
NOTTM
QMARY
LOND
EDINB
KINGS
UCL
BRIST
IMP
SGEO
SOTON
DUND
QUEEN
NEWC
SHEF

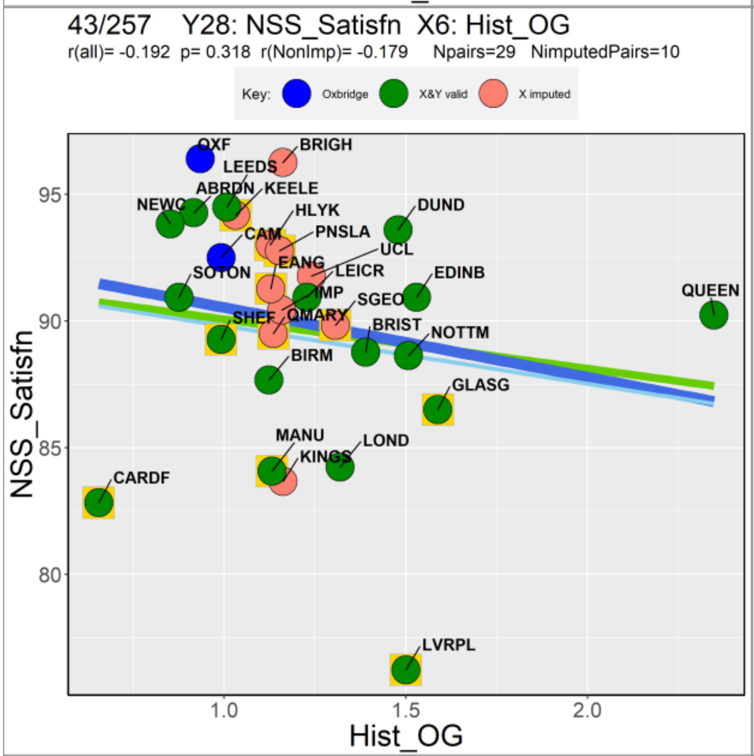
43/257 Y28: NSS_Satisfn X6: Hist_OG
r(all)= -0.192 p= 0.318 r(NonImp)= -0.179 Npairs=29 NimputedPairs=10
Key: Oxbridge X&Y valid X imputed
NSS_Satisfn
Hist_OG
OXF
BRIGH
LEEDS
ABRDN
KEELE
NEWC
HLYK
DUND
CAM
PNSLA
UCL
SOTON
EANG
LEICR
EDINB
IMP
SGEO
QUEEN
SHEF
QMARY
BRIST
NOTTM
BIRM
GLASG
MANU
LOND
KINGS
CARDF
LVRPL

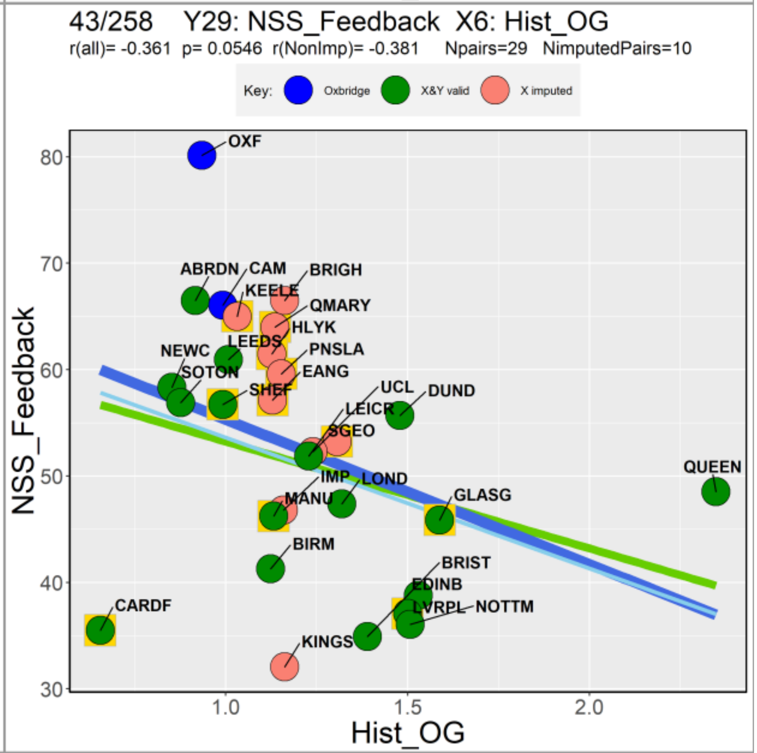
43/258 Y29: NSS_Feedback X6: Hist_OG
r(all)= -0.361 p= 0.0546 r(NonImp)= -0.381 Npairs=29 NimputedPairs=10
Key: Oxbridge X&Y valid X imputed
NSS_Feedback
Hist_OG
OXF
ABRDN
CAM
BRIGH
KEELE
QMARY
HLYK
NEWC
LEEDS
PNSLA
SOTON
EANG
SHEF
UCL
DUND
LEICR
SGEO
IMP
LOND
QUEEN
MANU
GLASG
BIRM
BRIST
EDINB
LVRPL
NOTTM
CARDF
KINGS

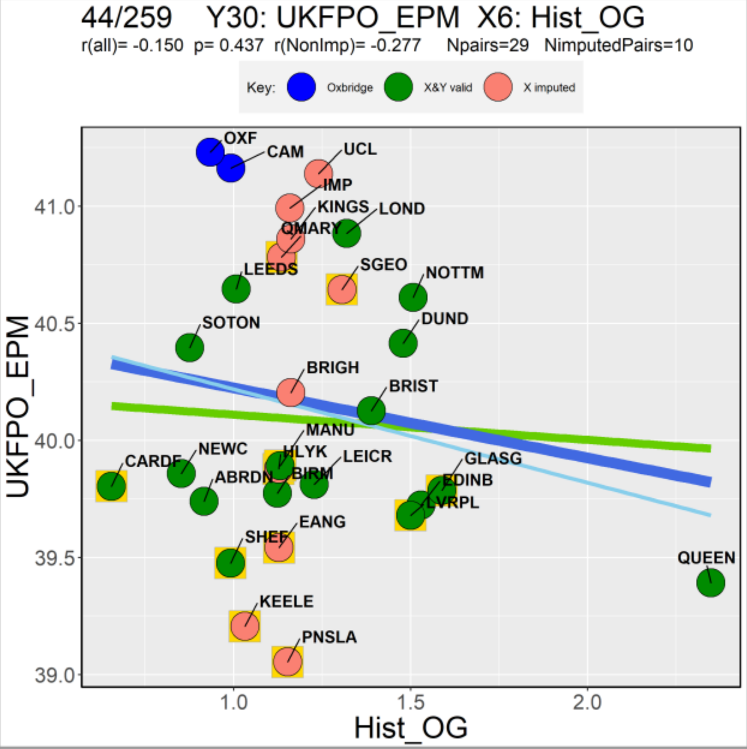
44/259 Y30: UKFPO_EPM X6: Hist_OG
r(all)= -0.150 p= 0.437 r(NonImp)= -0.277 Npairs=29 NimputedPairs=10
Key: Oxbridge X&Y valid X imputed
OXF
CAM
UCL
IMP
KINGS
LOND
QMARY
LEEDS
SGEO
NOTTM
SOTON
DUND
BRIGH
BRIST
MANU
CARDF
NEWC
HLYK
LEICR
GLASG
ABRDN
BIRM
EDINB
LVRPL
EANG
SHEF
QUEEN
KEELE
PNSLA
41.0
40.5
40.0
39.5
39.0
1.0
1.5
2.0
UKFPO_EPM
Hist_OG

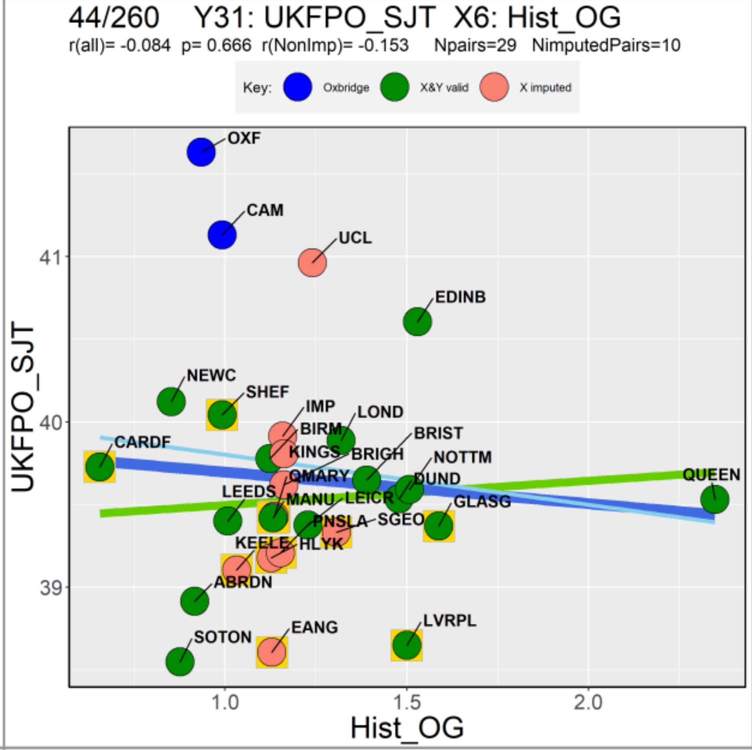
44/260 Y31: UKFPO_SJT X6: Hist_OG
r(all)= -0.084 p= 0.666 r(NonImp)= -0.153 Npairs=29 NimputedPairs=10
Key: Oxbridge X&Y valid X imputed
OXF
CAM
UCL
EDINB
NEWC
SHEF
IMP
LOND
BIRM
BRIST
CARDF
KINGS
BRIGH
NOTTM
QMARY
LEICR
QUEEN
DUND
LEEDS
MANU
GLASG
PNSLA
SGEO
KEELE
HLYK
ABRDN
EANG
LVRPL
SOTON
41
40
39
1.0
1.5
2.0
UKFPO_SJT
Hist_OG

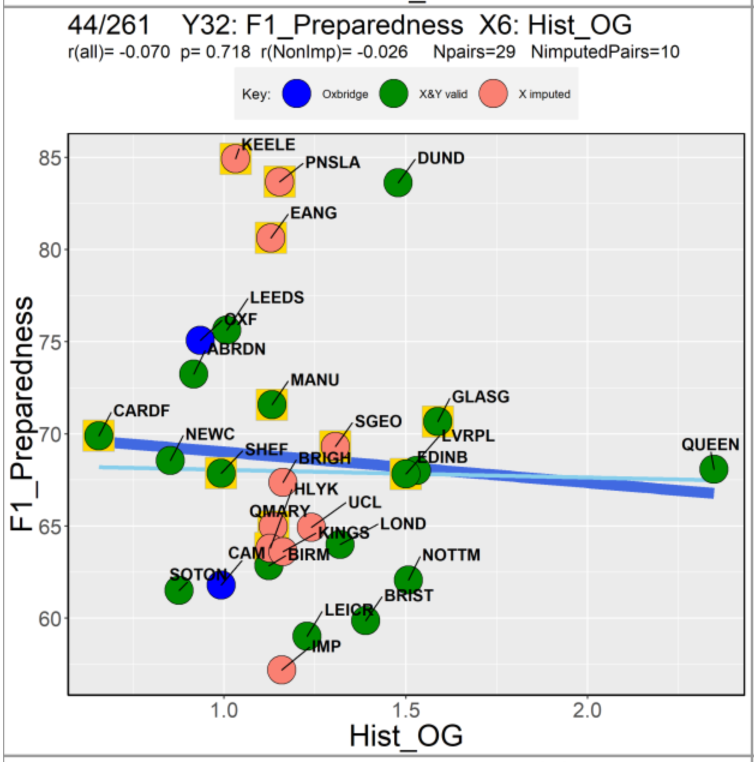
44/261 Y32: F1_Preparedness X6: Hist_OG
r(all)= -0.070 p= 0.718 r(NonImp)= -0.026 Npairs=29 NimputedPairs=10
Key: Oxbridge X&Y valid X imputed
KEELE
PNSLA
DUND
EANG
LEEDS
OXF
ABRDN
MANU
GLASG
CARDF
SGEO
LVRPL
NEWC
SHEF
BRIGH
EDINB
QUEEN
HLYK
QMARY
UCL
LOND
KINGS
CAM
BIRM
NOTTM
SOTON
BRIST
LEICR
IMP
85
80
75
70
65
60
1.0
1.5
2.0
F1_Preparedness
Hist_OG

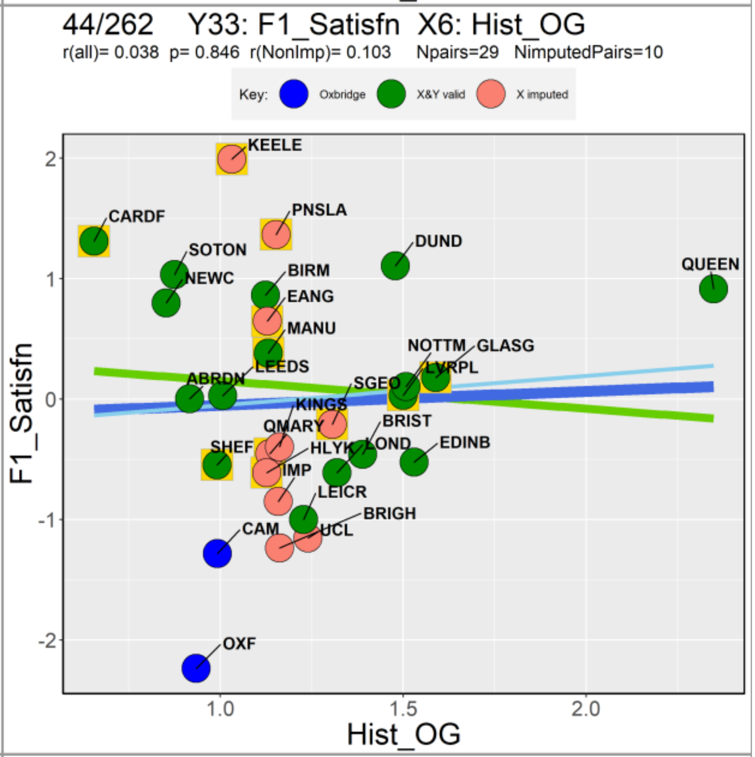
44/262 Y33: F1_Satisfn X6: Hist_OG
r(all)= 0.038 p= 0.846 r(NonImp)= 0.103 Npairs=29 NimputedPairs=10
Key: Oxbridge X&Y valid X imputed
KEELE
CARDF
PNSLA
SOTON
DUND
NEWC
BIRM
QUEEN
EANG
MANU
NOTTM
GLASG
LEEDS
LVRPL
ABRDN
SGEO
KINGS
QMARY
BRIST
SHEF
HLYK
LOND
EDINB
IMP
LEICR
BRIGH
CAM
UCL
OXF
2
1
0
-1
-2
1.0
1.5
2.0
F1_Satisfn
Hist_OG

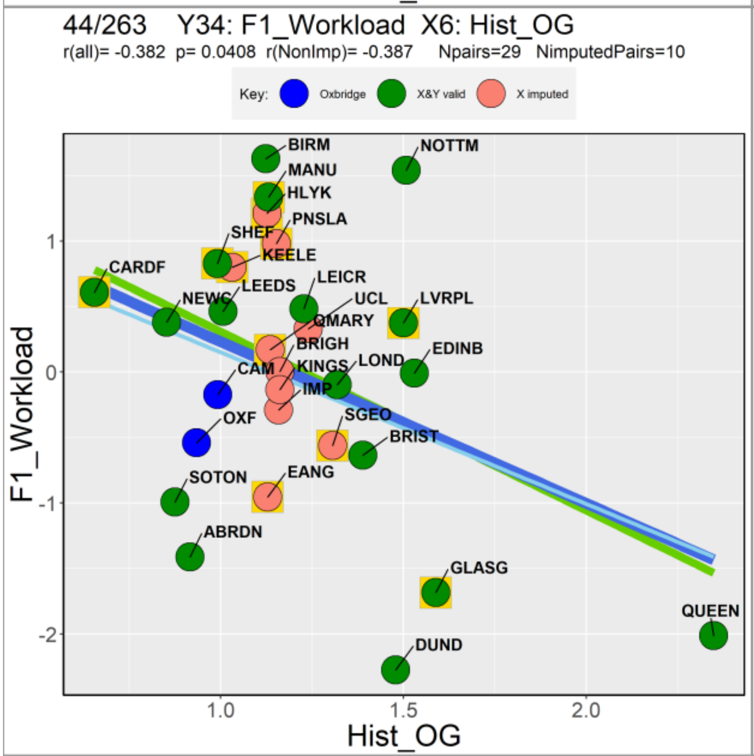
44/263 Y34: F1_Workload X6: Hist_OG
r(all)= -0.382 p= 0.0408 r(NonImp)= -0.387 Npairs=29 NimputedPairs=10
Key: Oxbridge X&Y valid X imputed
BIRM
NOTTM
MANU
HLYK
PNSLA
SHEF
KEELE
CARDF
LEEDS
LEICR
NEWC
UCL
LVRPL
QMARY
BRIGH
EDINB
CAM
KINGS
LOND
IMP
OXF
SGEO
BRIST
SOTON
EANG
ABRDN
GLASG
QUEEN
DUND
1
0
-1
-2
1.0
1.5
2.0
F1_Workload
Hist_OG

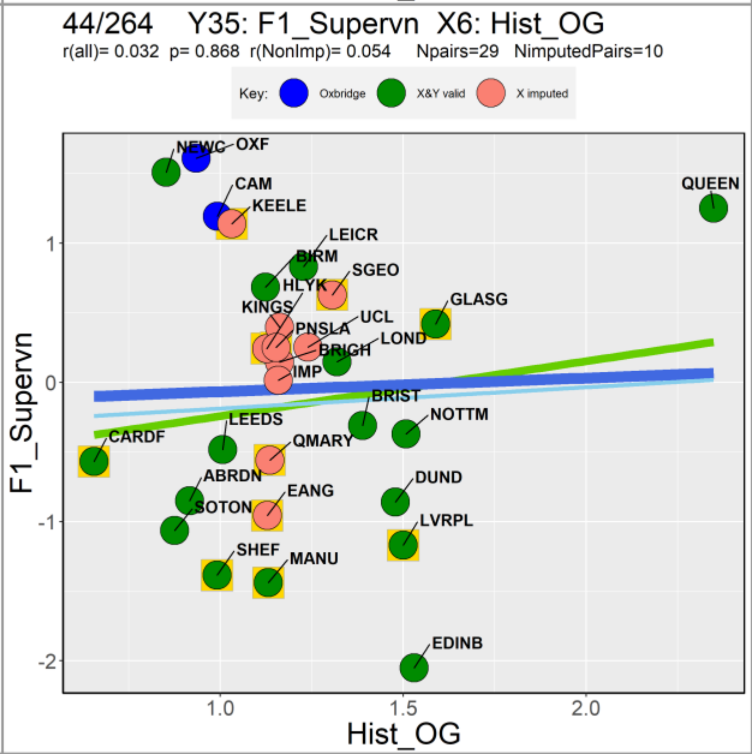
44/264 Y35: F1_Supervn X6: Hist_OG
r(all)= 0.032 p= 0.868 r(NonImp)= 0.054 Npairs=29 NimputedPairs=10
Key: Oxbridge X&Y valid X imputed
NEWC
OXF
CAM
QUEEN
KEELE
LEICR
BIRM
SGEO
HLYK
GLASG
KINGS
UCL
PNSLA
LOND
BRIGH
IMP
BRIST
NOTTM
LEEDS
CARDF
QMARY
ABRDN
DUND
SOTON
EANG
LVRPL
SHEF
MANU
EDINB
1
0
-1
-2
1.0
1.5
2.0
F1_Supervn
Hist_OG

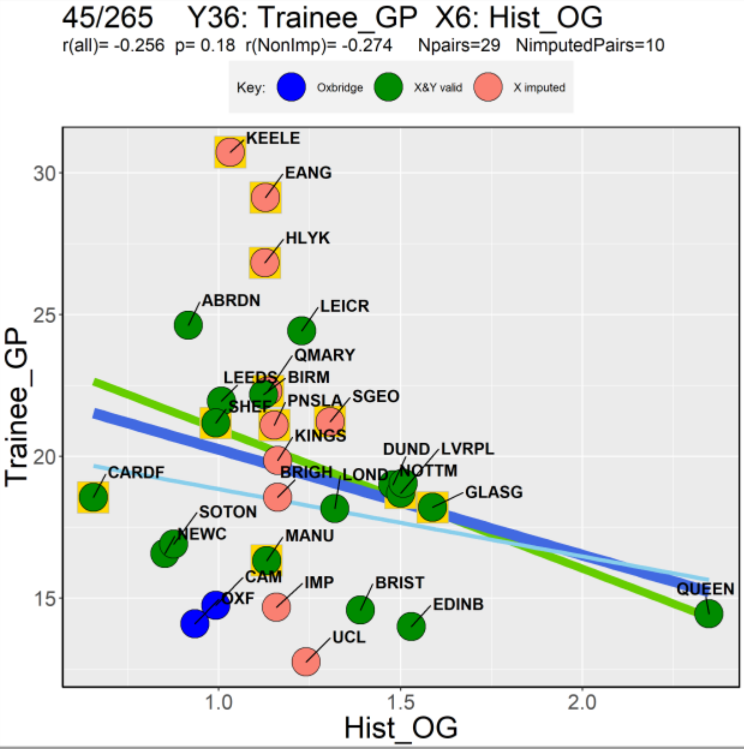
45/265 Y36: Trainee_GP X6: Hist_OG
r(all)= -0.256 p= 0.18 r(NonImp)= -0.274 Npairs=29 NimputedPairs=10
Key: Oxbridge X&Y valid X imputed
Trainee_GP
Hist_OG

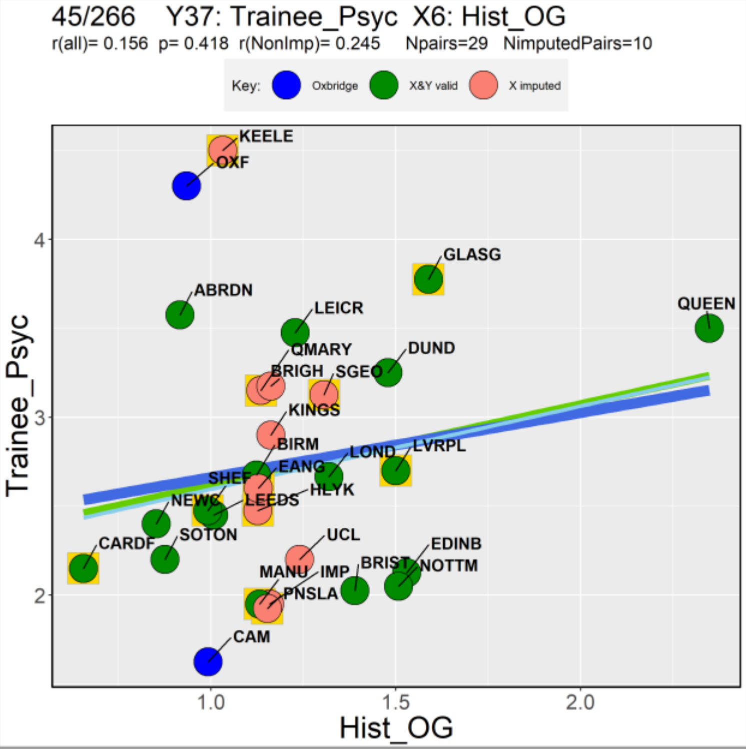
45/266 Y37: Trainee_Psyc X6: Hist_OG
r(all)= 0.156 p= 0.418 r(NonImp)= 0.245 Npairs=29 NimputedPairs=10
Key: Oxbridge X&Y valid X imputed
Trainee_Psyc
Hist_OG

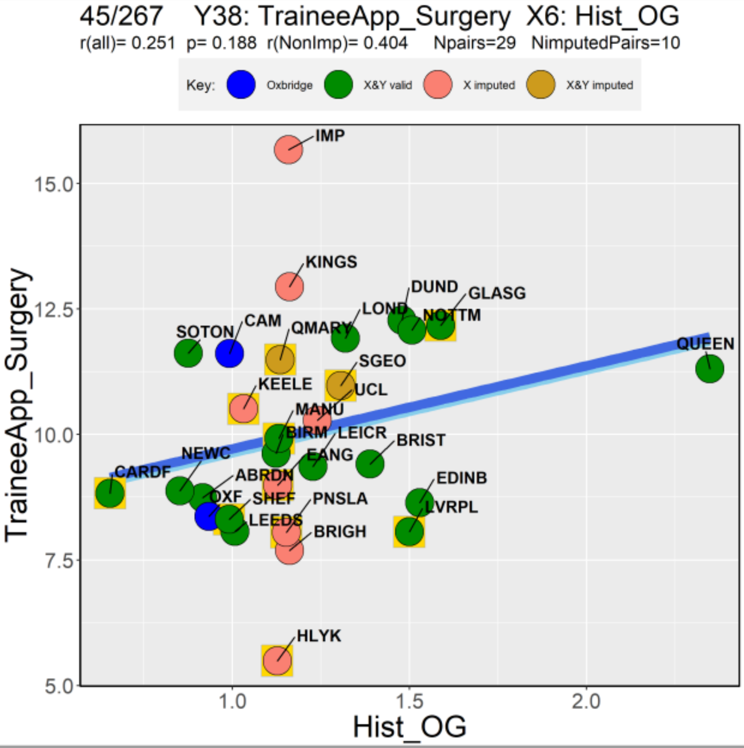
45/267 Y38: TraineeApp_Surgery X6: Hist_OG
r(all)= 0.251 p= 0.188 r(NonImp)= 0.404 Npairs=29 NimputedPairs=10
Key: Oxbridge X&Y valid X imputed X&Y imputed
TraineeApp_Surgery
Hist_OG

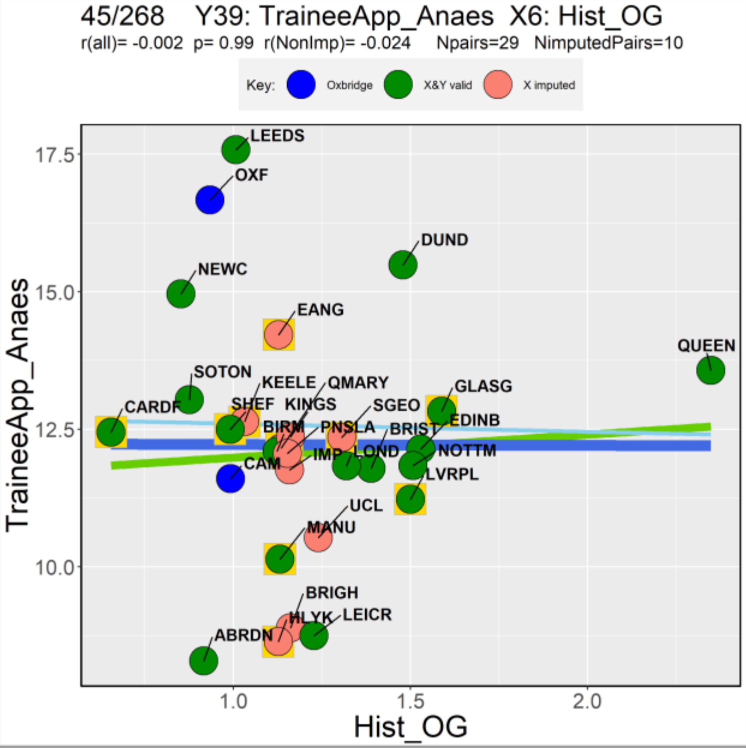
45/268 Y39: TraineeApp_Anaes X6: Hist_OG
r(all)= -0.002 p= 0.99 r(NonImp)= -0.024 Npairs=29 NimputedPairs=10
Key: Oxbridge X&Y valid X imputed
TraineeApp_Anaes
Hist_OG

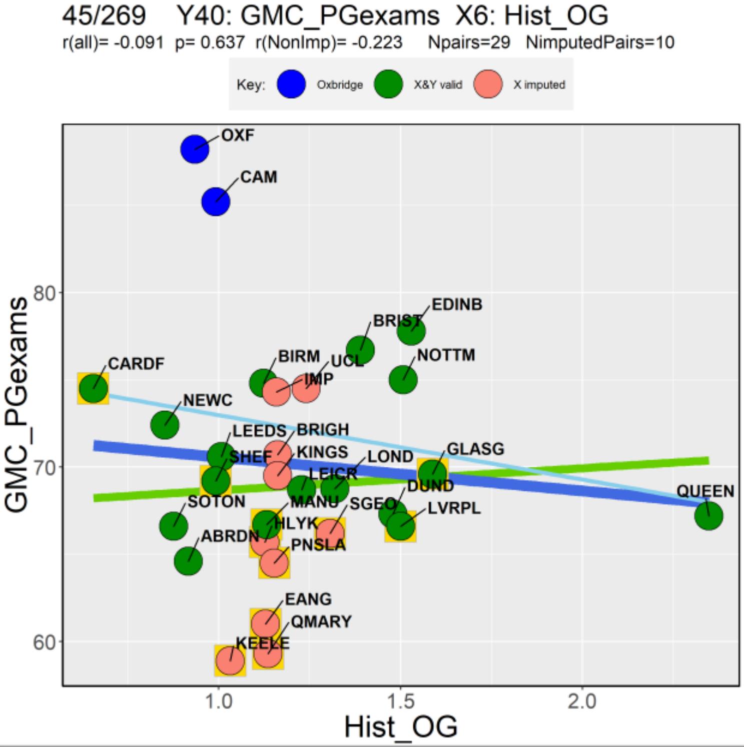
45/269 Y40: GMC_PGexams X6: Hist_OG
r(all)= -0.091 p= 0.637 r(NonImp)= -0.223 Npairs=29 NimputedPairs=10
Key: Oxbridge X&Y valid X imputed
GMC_PGexams
Hist_OG

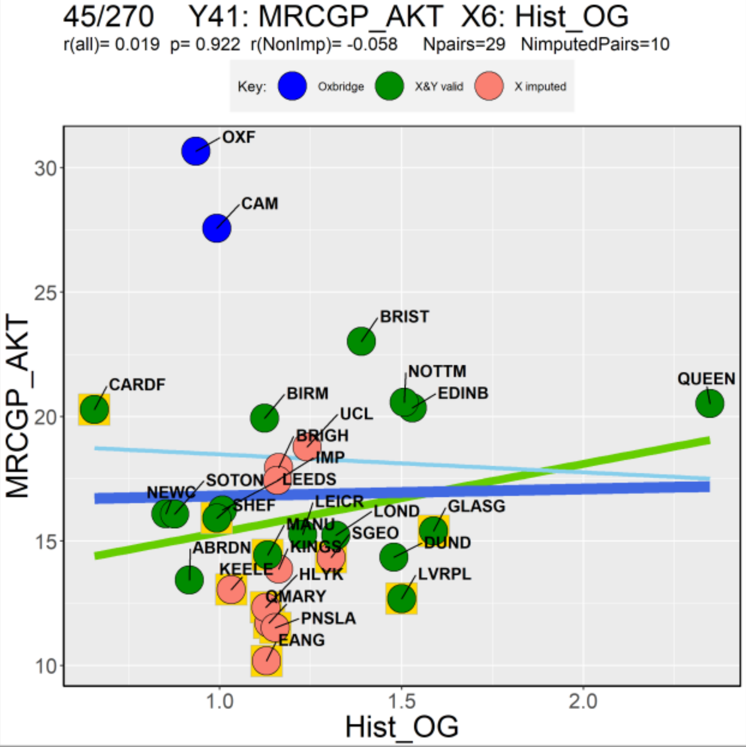
45/270 Y41: MRCGP_AKT X6: Hist_OG
r(all)= 0.019 p= 0.922 r(NonImp)= -0.058 Npairs=29 NimputedPairs=10
Key: Oxbridge X&Y valid X imputed
MRCGP_AKT
Hist_OG

46/271 Y42: MRCGP_CSA X6: Hist_OG

r(all)= -0.021 p= 0.913 r(NonImp)= -0.101 Npairs=29 NimputedPairs=10

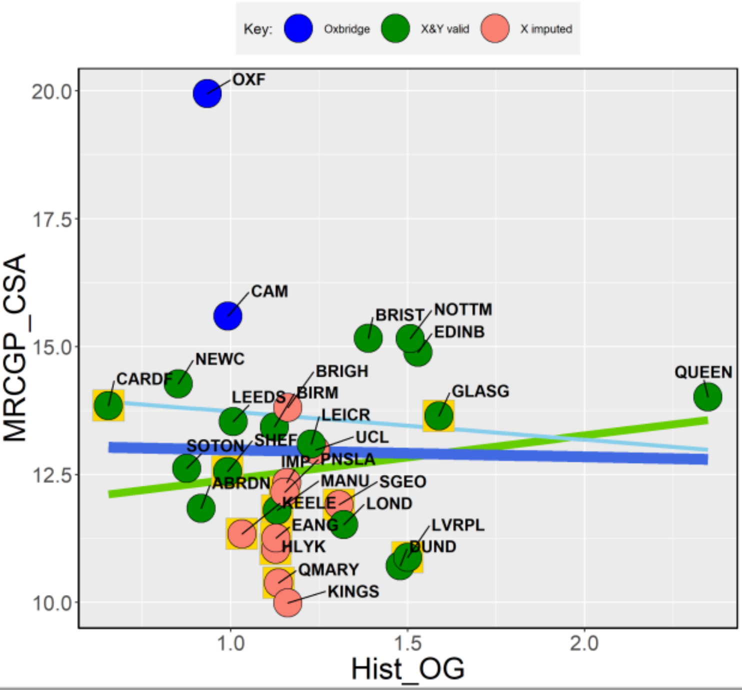

46/272 Y43: FRCA_Pt1 X6: Hist_OG

r(all)= -0.396 p= 0.0333 r(NonImp)= -0.423 Npairs=29 NimputedPairs=10

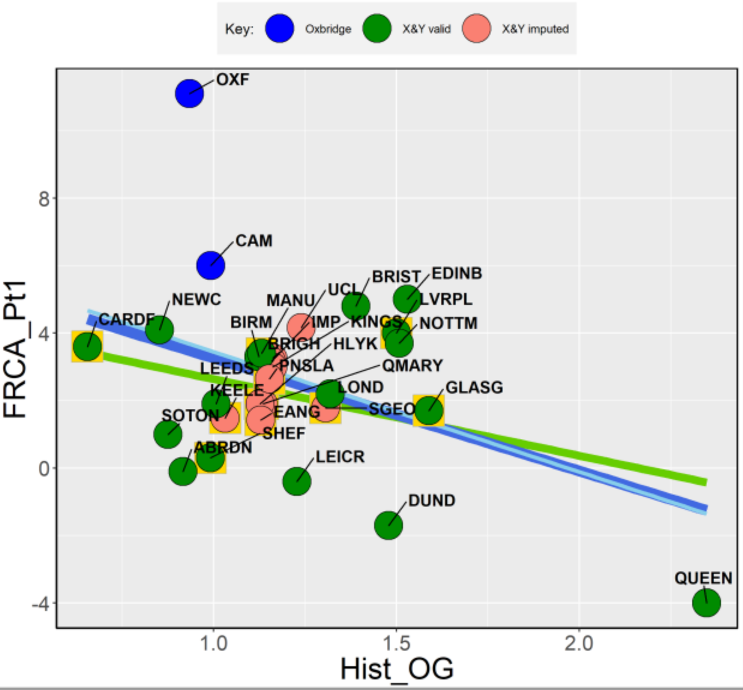

46/273 Y44: MRCOG_Pt1 X6: Hist_OG

r(all)= -0.031 p= 0.872 r(NonImp)= -0.059 Npairs=29 NimputedPairs=10

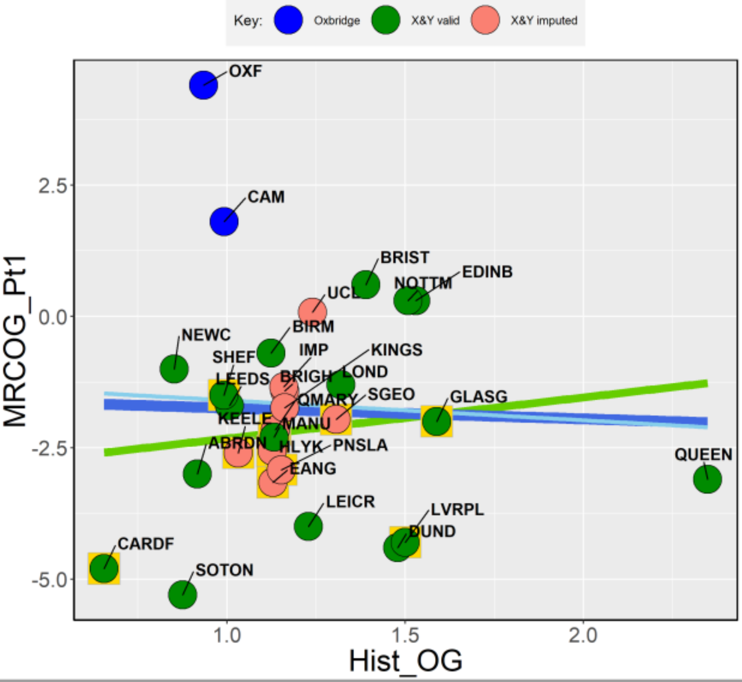

46/274 Y45: MRCOG_Pt2 X6: Hist_OG

r(all)= -0.027 p= 0.89 r(NonImp)= -0.048 Npairs=29 NimputedPairs=10

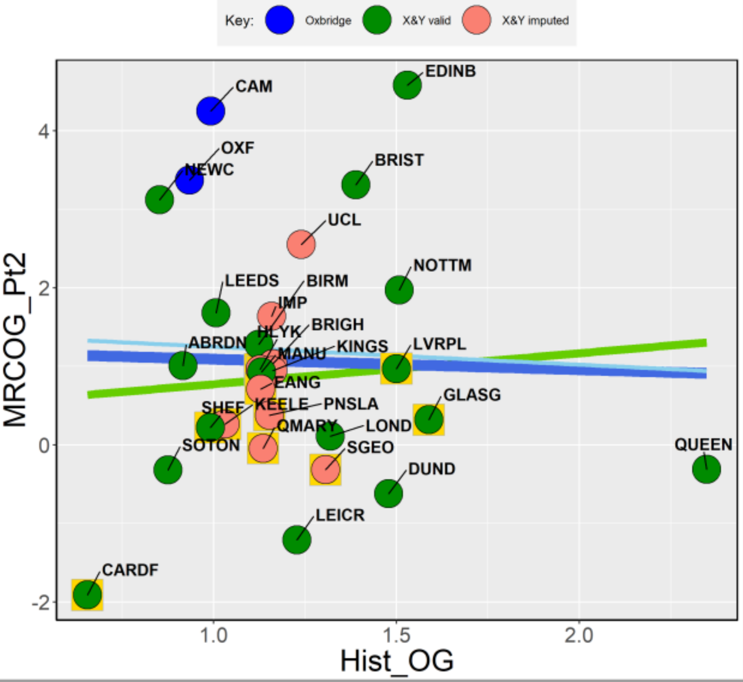

46/275 Y46: MRCP_Pt1 X6: Hist_OG

r(all)= -0.211 p= 0.271 r(NonImp)= -0.267 Npairs=29 NimputedPairs=10

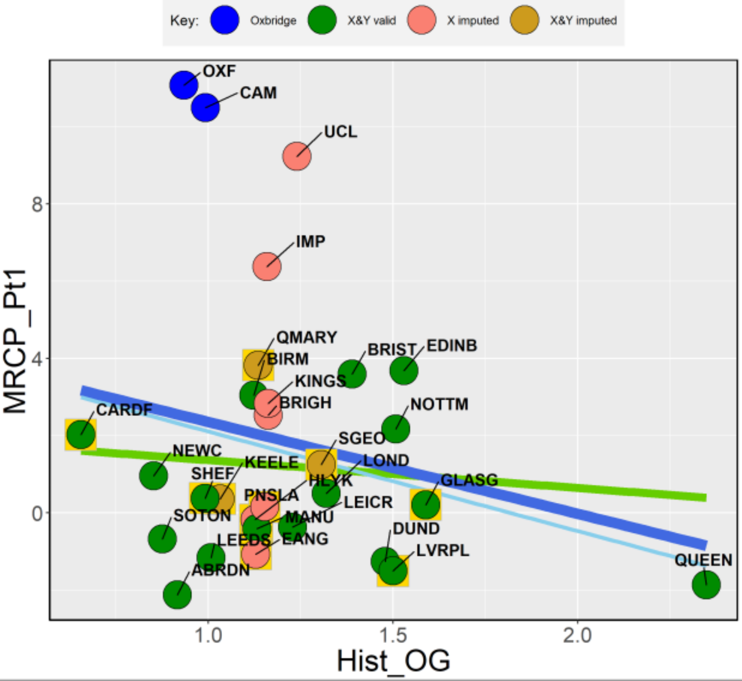

46/276 Y47: MRCP_Pt2 X6: Hist_OG

r(all)= -0.186 p= 0.333 r(NonImp)= -0.220 Npairs=29 NimputedPairs=10

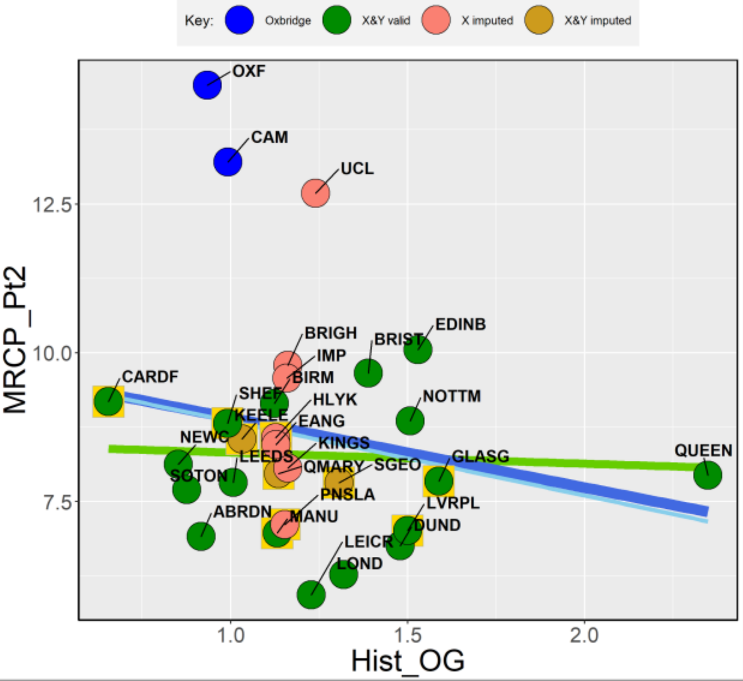

47/277 Y48: MRCP_PACES X6: Hist_OG

r(all)= -0.057 p= 0.769 r(NonImp)= -0.081 Npairs=29 NimputedPairs=10

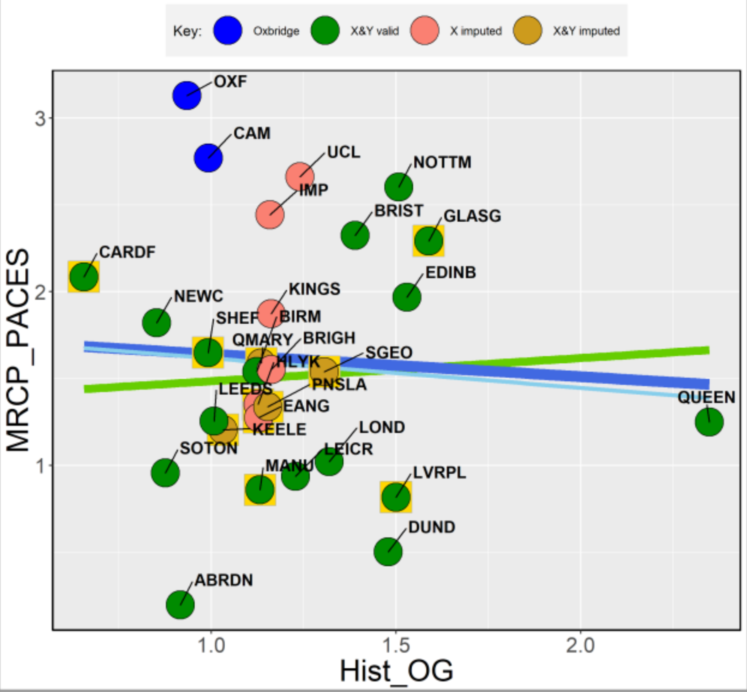

47/278 Y49: GMC_Sanctions X6: Hist_OG

r(all)= -0.231 p= 0.229 r(NonImp)= -0.212 Npairs=29 NimputedPairs=10

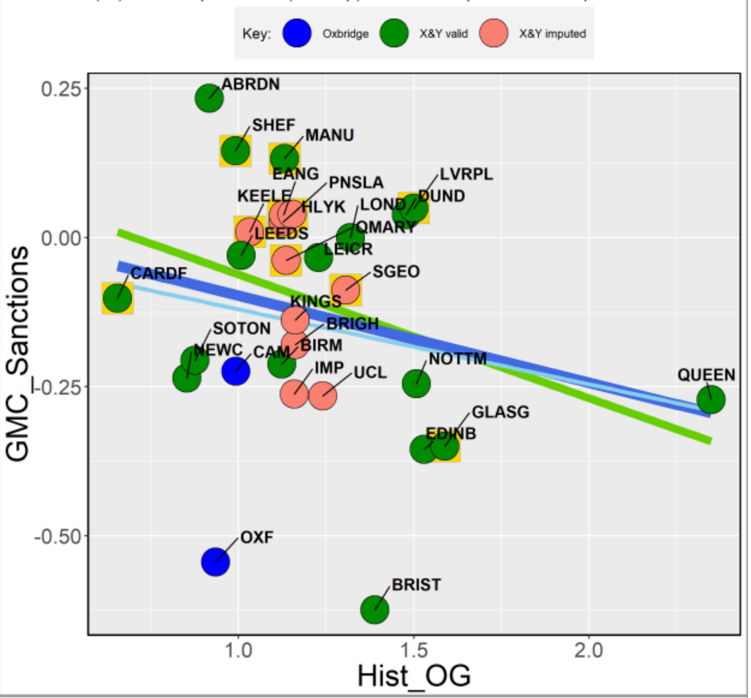

47/279 Y50: ARCP_NotExam X6: Hist_OG

r(all)= -0.036 p= 0.851 r(NonImp)= 0.010 Npairs=29 NimputedPairs=10

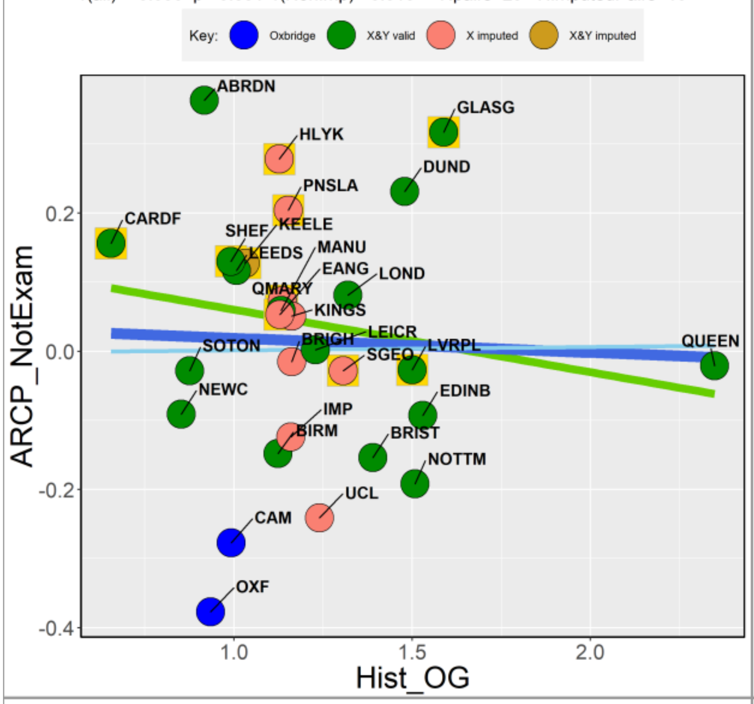

47/280 Y8: Hist_Surgery X7: Hist_IntMed

r(all)= 0.755 p= 2.18e-06 r(NonImp)= 0.751 Npairs=29 NimputedPairs=10

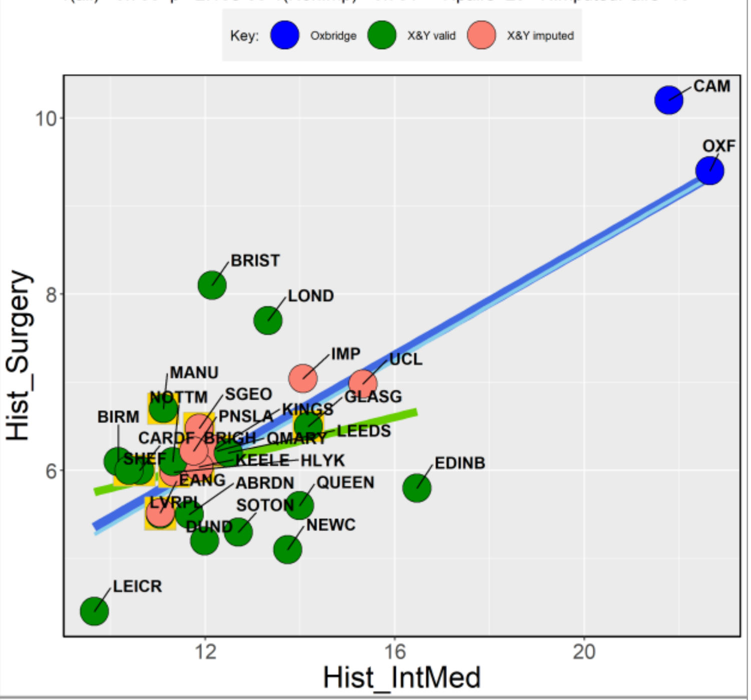

47/281 Y9: Post2000 X7: Hist_IntMed

r(all)= -0.202 p= 0.293 r(NonImp)= NA Npairs=29 NimputedPairs=10

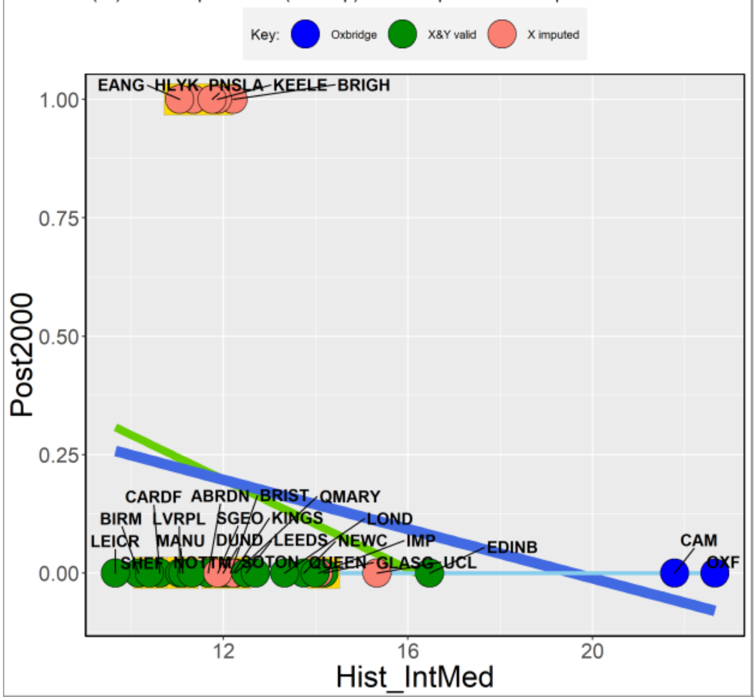

47/282 Y10: REF X7: Hist_IntMed

r(all)= 0.718 p= 1.18e-05 r(NonImp)= 0.820 Npairs=29 NimputedPairs=10

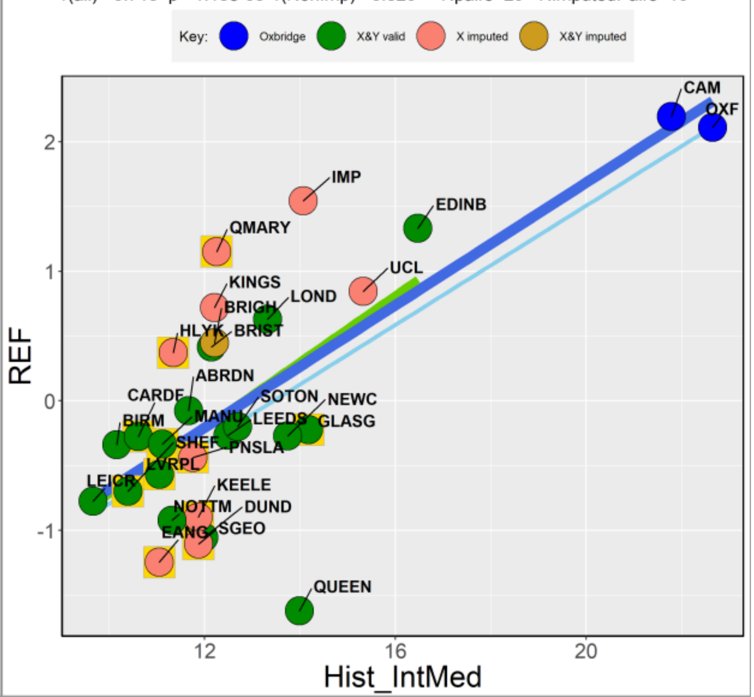

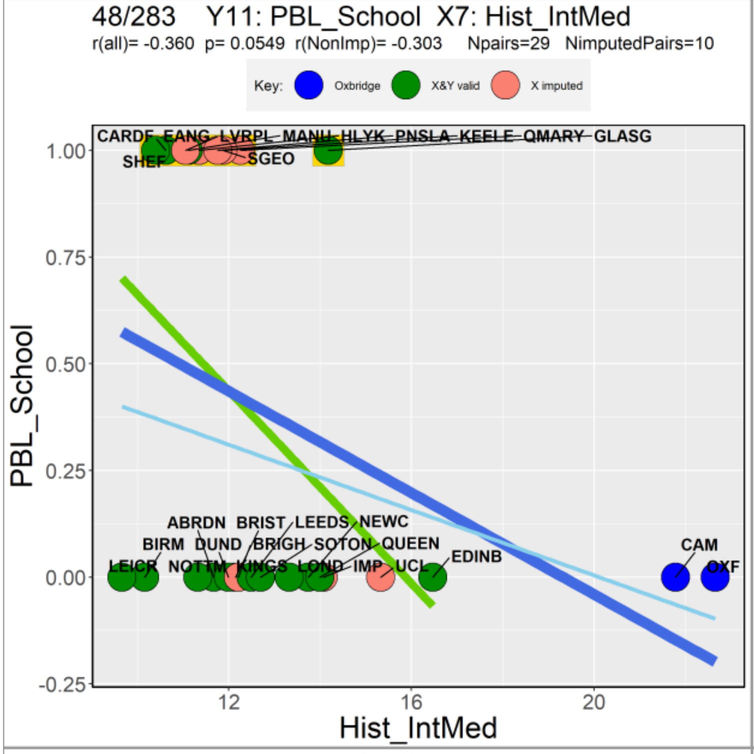
48/283 Y11: PBL_School X7: Hist_IntMed
r(all)= -0.360 p= 0.0549 r(NonImp)= -0.303 Npairs=29 NimputedPairs=10
Key: Oxbridge X&Y valid X imputed
PBL_School
1.00
0.75
0.50
0.25
0.00
-0.25
12
16
20
Hist_IntMed

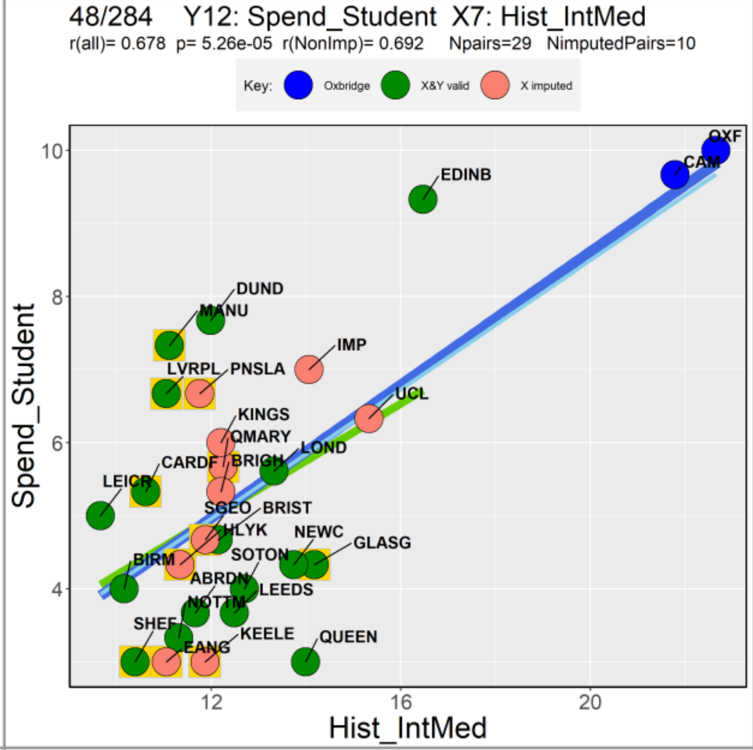
48/284 Y12: Spend_Student X7: Hist_IntMed
r(all)= 0.678 p= 5.26e-05 r(NonImp)= 0.692 Npairs=29 NimputedPairs=10
Key: Oxbridge X&Y valid X imputed
Spend_Student
10
8
6
4
12
16
20
Hist_IntMed

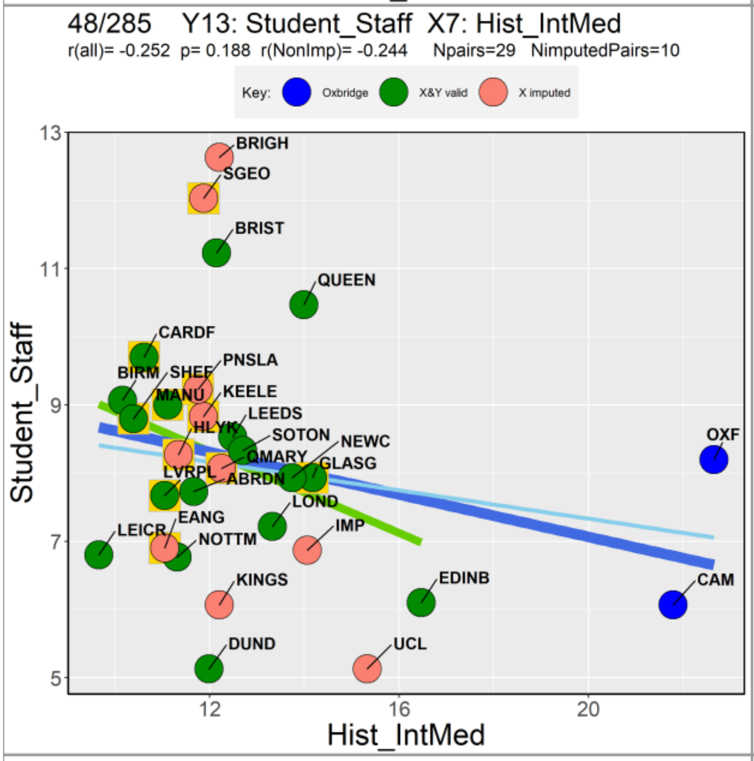
48/285 Y13: Student_Staff X7: Hist_IntMed
r(all)= -0.252 p= 0.188 r(NonImp)= -0.244 Npairs=29 NimputedPairs=10
Key: Oxbridge X&Y valid X imputed
Student_Staff
13
11
9
7
5
12
16
20
Hist_IntMed

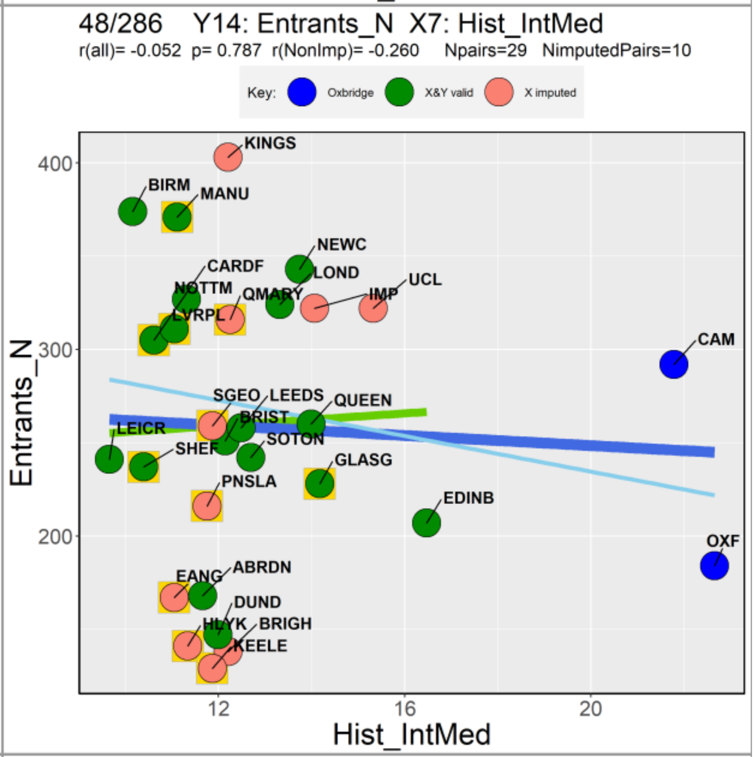
48/286 Y14: Entrants_N X7: Hist_IntMed
r(all)= -0.052 p= 0.787 r(NonImp)= -0.260 Npairs=29 NimputedPairs=10
Key: Oxbridge X&Y valid X imputed
Entrants_N
400
300
200
12
16
20
Hist_IntMed

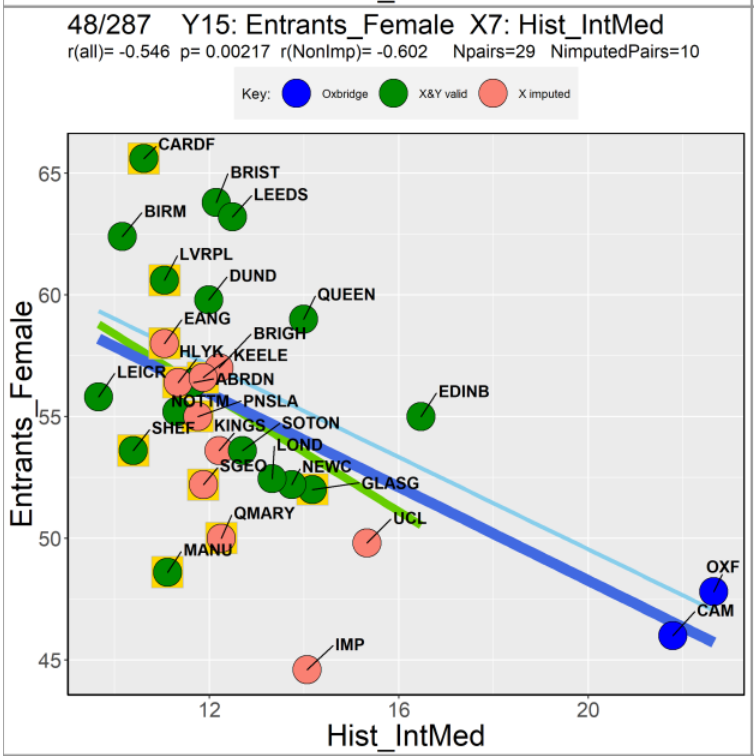
48/287 Y15: Entrants_Female X7: Hist_IntMed
r(all)= -0.546 p= 0.00217 r(NonImp)= -0.602 Npairs=29 NimputedPairs=10
Key: Oxbridge X&Y valid X imputed
Entrants_Female
65
60
55
50
45
12
16
20
Hist_IntMed

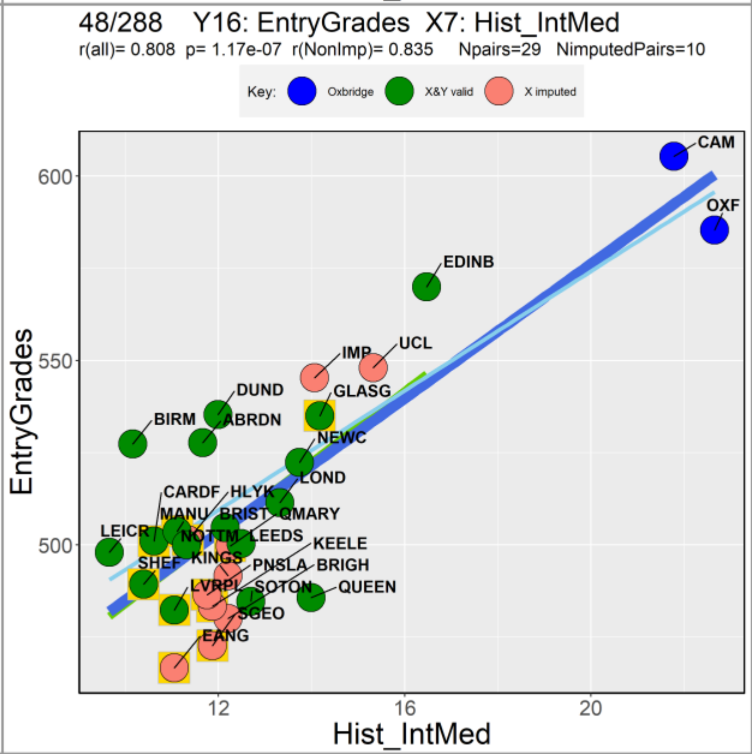
48/288 Y16: EntryGrades X7: Hist_IntMed
r(all)= 0.808 p= 1.17e-07 r(NonImp)= 0.835 Npairs=29 NimputedPairs=10
Key: Oxbridge X&Y valid X imputed
EntryGrades
600
550
500
12
16
20
Hist_IntMed

49/289 Y17: Entrants_NonHome X7: Hist_IntMed
r(all)= -0.066 p= 0.733 r(NonImp)= -0.137 Npairs=29 NimputedPairs=10

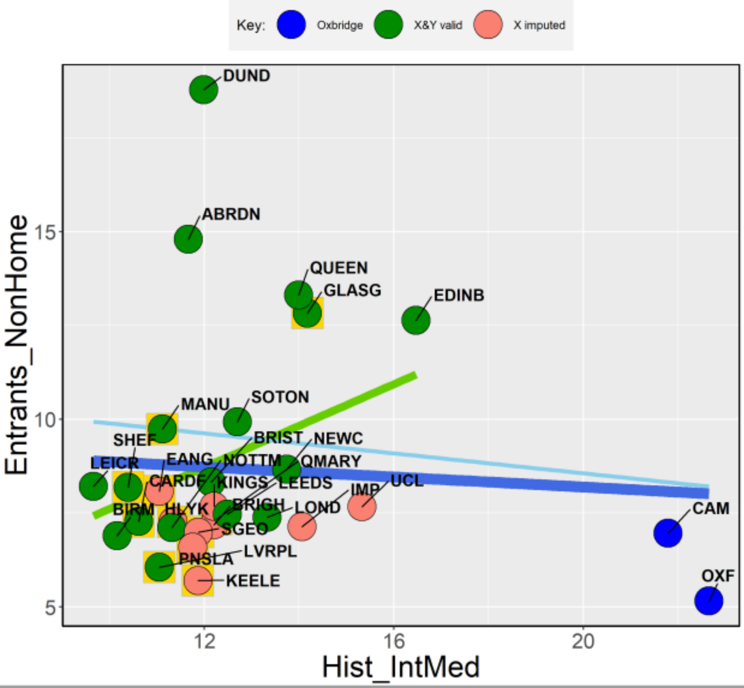

49/290 Y18: Teaching_Factor1_Trad X7: Hist_IntMed
r(all)= 0.472 p= 0.00967 r(NonImp)= 0.535 Npairs=29 NimputedPairs=12

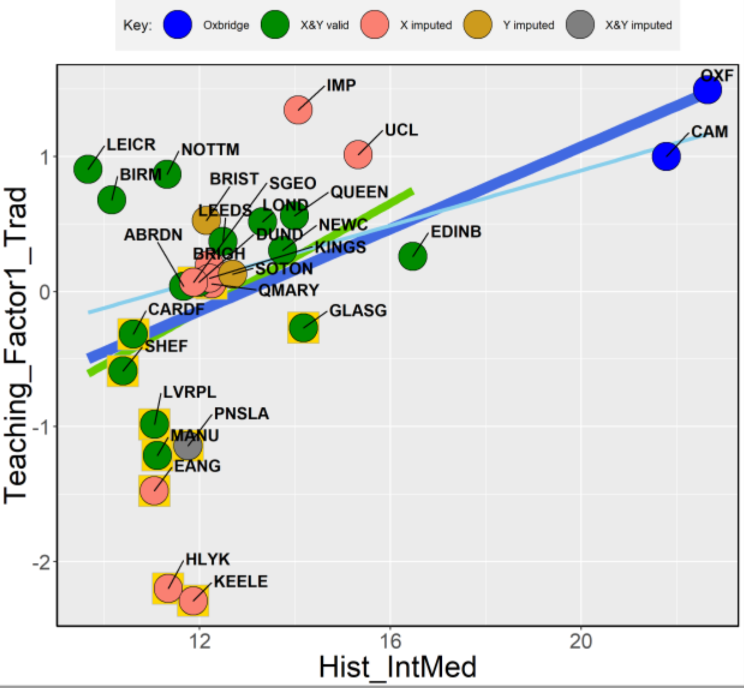

49/291 Y19: Teaching_Factor2_Struc X7: Hist_IntMed
r(all)= -0.087 p= 0.654 r(NonImp)= -0.048 Npairs=29 NimputedPairs=12

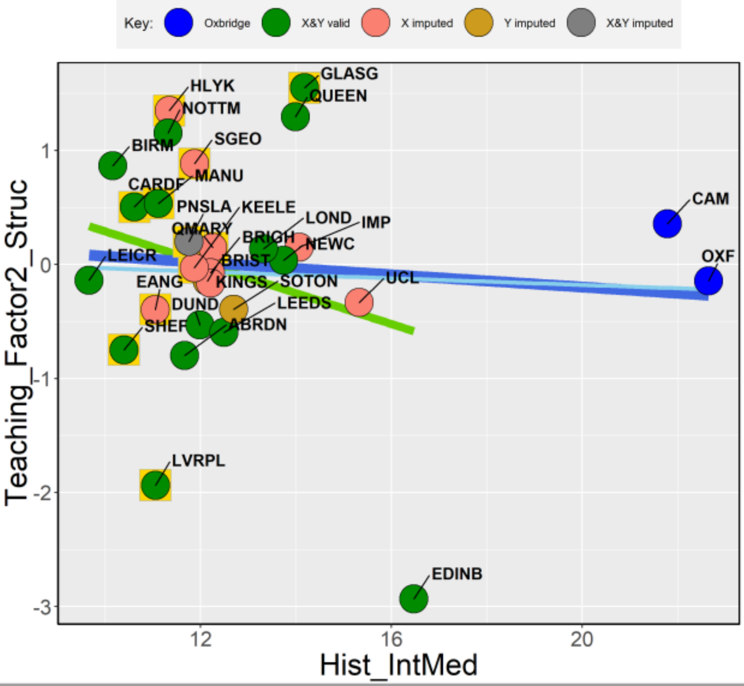

49/292 Y20: Teach_GP X7: Hist_IntMed
r(all)= -0.258 p= 0.176 r(NonImp)= -0.203 Npairs=29 NimputedPairs=12

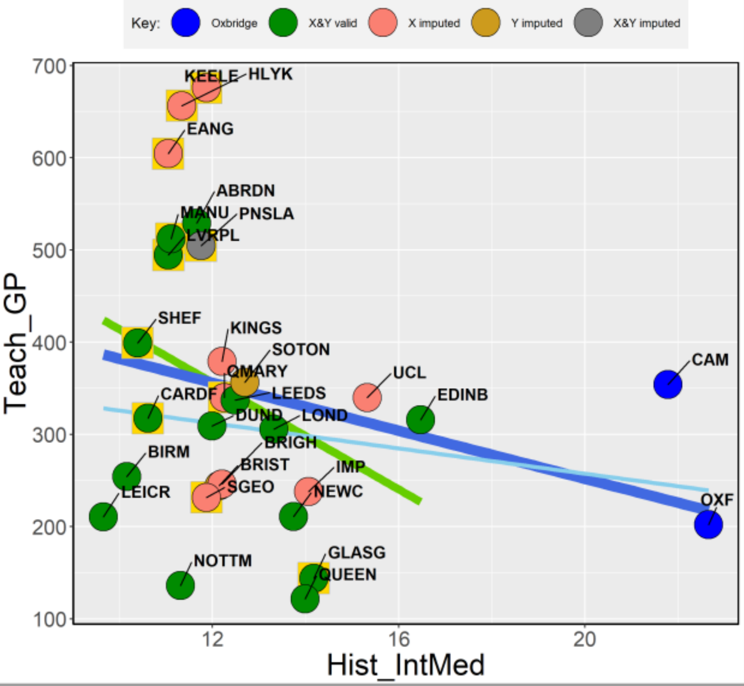

49/293 Y21: Teach_Psyc X7: Hist_IntMed
r(all)= 0.391 p= 0.036 r(NonImp)= 0.398 Npairs=29 NimputedPairs=12

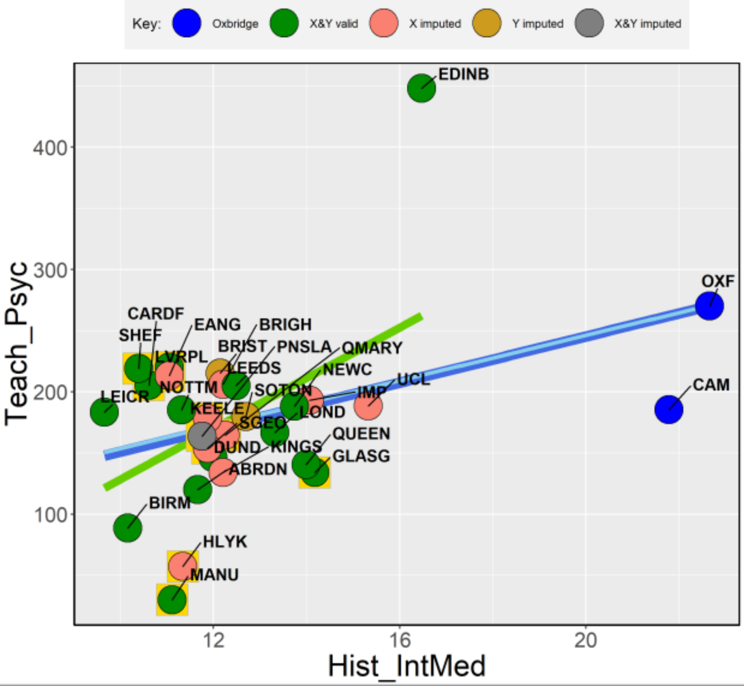

49/294 Y22: Teach_Anaes X7: Hist_IntMed
r(all)= -0.003 p= 0.986 r(NonImp)= -0.149 Npairs=29 NimputedPairs=12

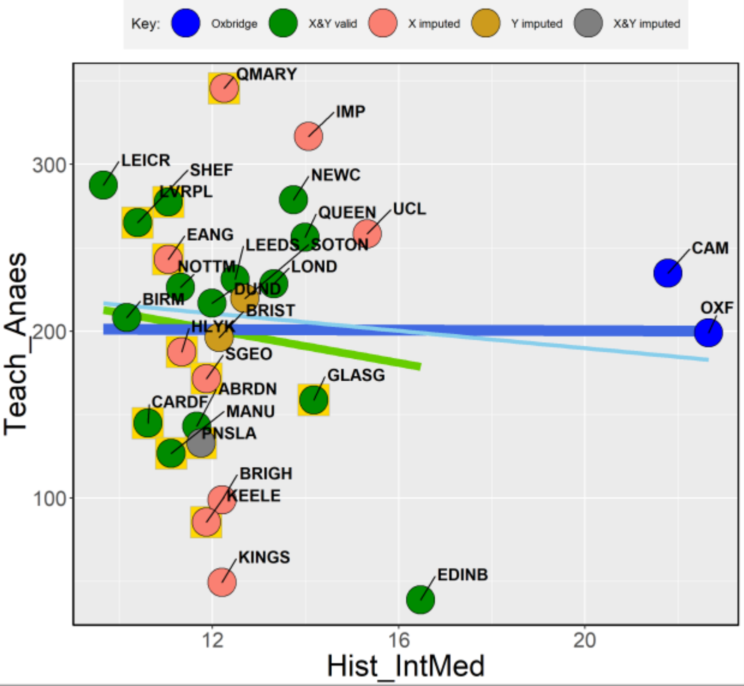

50/295 Y23: Teach_OG X7: Hist_IntMed
r(all)= 0.324 p= 0.0866 r(NonImp)= 0.267 Npairs=29 NimputedPairs=12

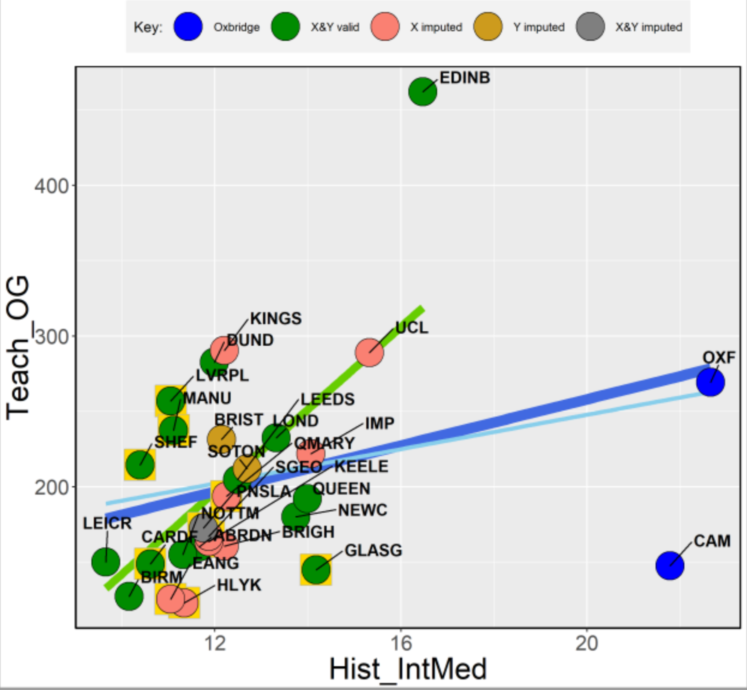

50/296 Y24: Teach_IntMed X7: Hist_IntMed
r(all)= 0.315 p= 0.0955 r(NonImp)= 0.320 Npairs=29 NimputedPairs=12

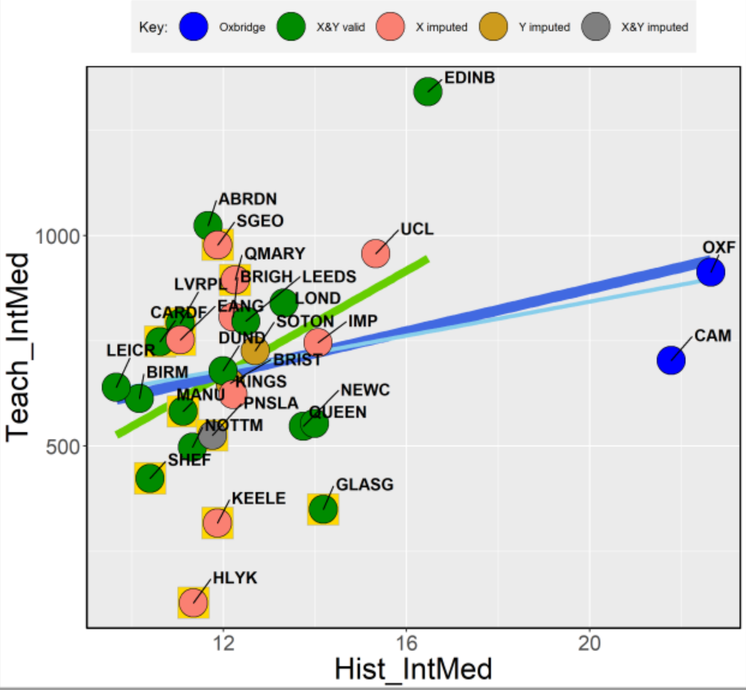

50/297 Y25: Teach_Surgery X7: Hist_IntMed
r(all)= 0.554 p= 0.00181 r(NonImp)= 0.586 Npairs=29 NimputedPairs=12

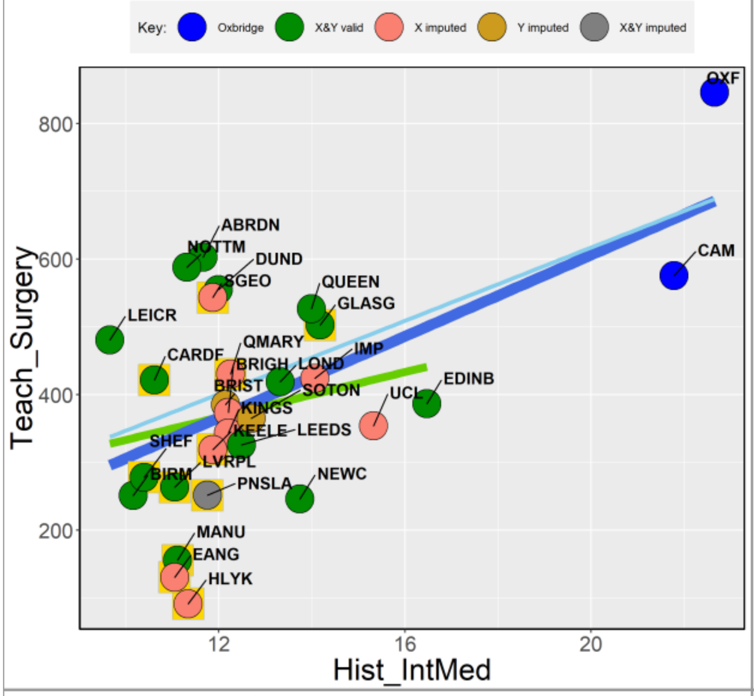

50/298 Y26: ExamTime X7: Hist_IntMed
r(all)= 0.476 p= 0.00897 r(NonImp)= 0.542 Npairs=29 NimputedPairs=11

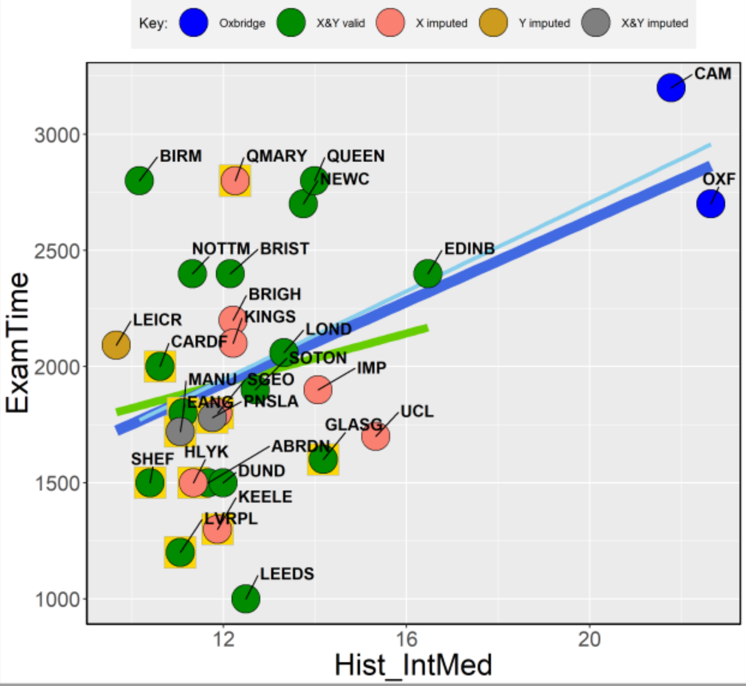

50/299 Y27: SelfRegLearn X7: Hist_IntMed
r(all)= 0.441 p= 0.0166 r(NonImp)= 0.530 Npairs=29 NimputedPairs=10

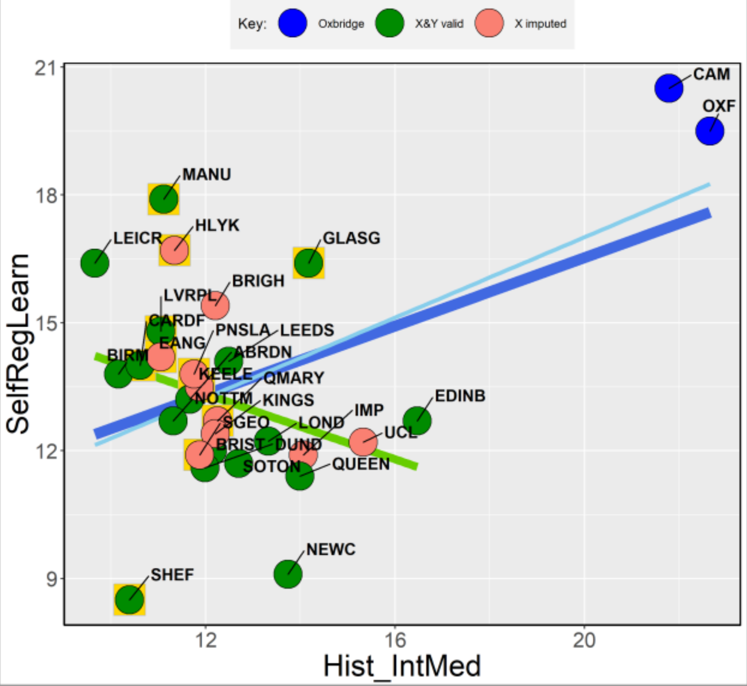

50/300 Y28: NSS_Satisfn X7: Hist_IntMed
r(all)= 0.334 p= 0.0768 r(NonImp)= 0.439 Npairs=29 NimputedPairs=10

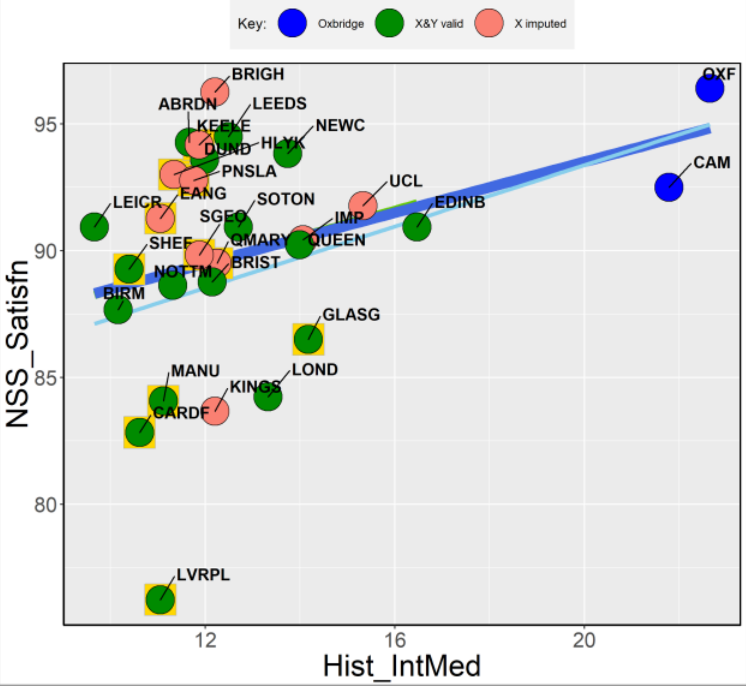

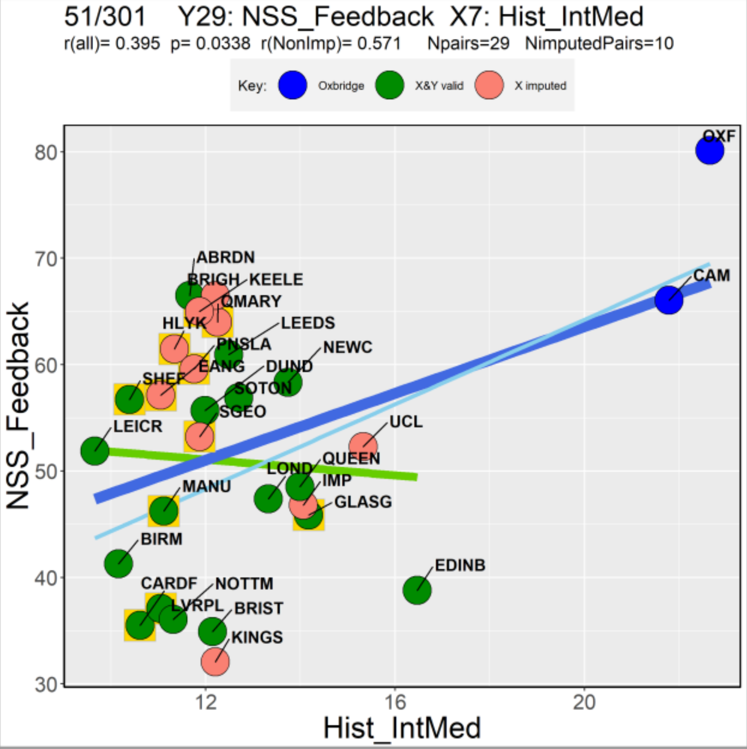
51/301 Y29: NSS_Feedback X7: Hist_IntMed
r(all)= 0.395 p= 0.0338 r(NonImp)= 0.571 Npairs=29 NimputedPairs=10
Key: Oxbridge X&Y valid X imputed
NSS_Feedback
Hist_IntMed

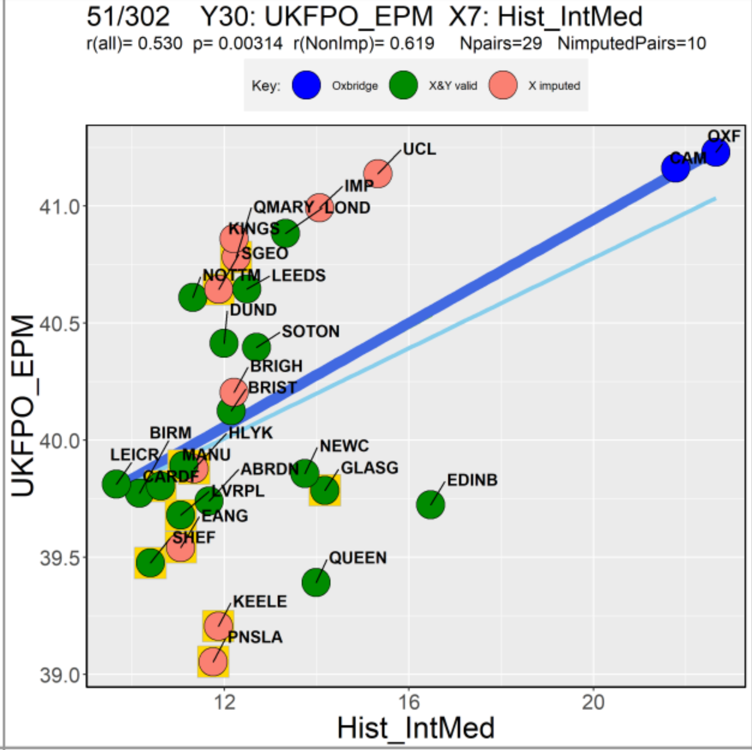
51/302 Y30: UKFPO_EPM X7: Hist_IntMed
r(all)= 0.530 p= 0.00314 r(NonImp)= 0.619 Npairs=29 NimputedPairs=10
Key: Oxbridge X&Y valid X imputed
UKFPO_EPM
Hist_IntMed

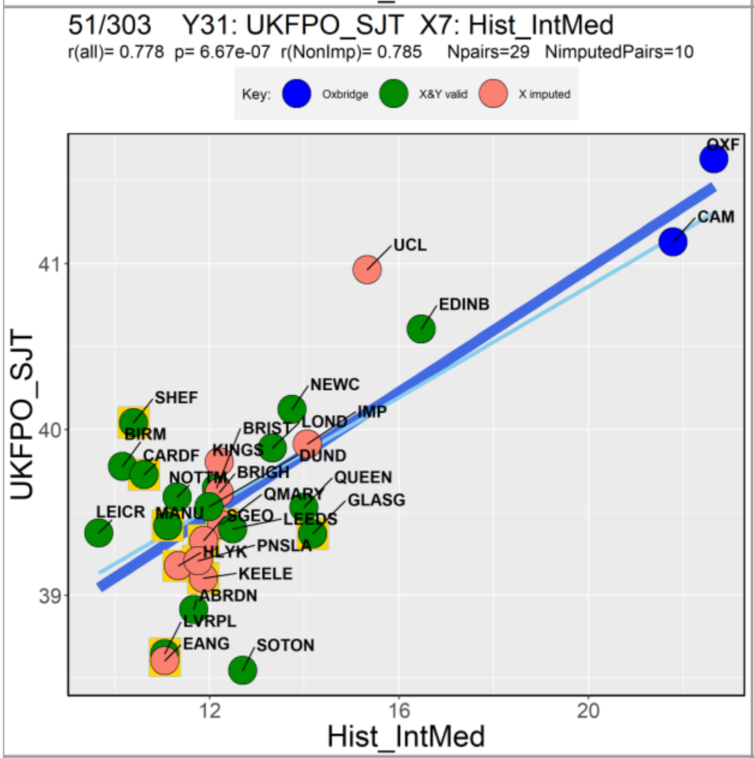
51/303 Y31: UKFPO_SJT X7: Hist_IntMed
r(all)= 0.778 p= 6.67e-07 r(NonImp)= 0.785 Npairs=29 NimputedPairs=10
Key: Oxbridge X&Y valid X imputed
UKFPO_SJT
Hist_IntMed

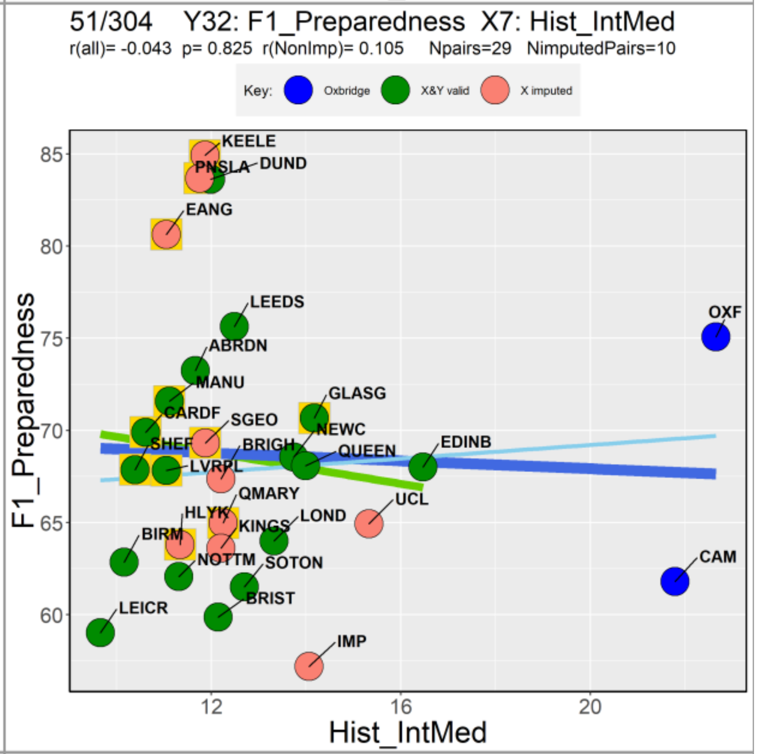
51/304 Y32: F1_Preparedness X7: Hist_IntMed
r(all)= -0.043 p= 0.825 r(NonImp)= 0.105 Npairs=29 NimputedPairs=10
Key: Oxbridge X&Y valid X imputed
F1_Preparedness
Hist_IntMed

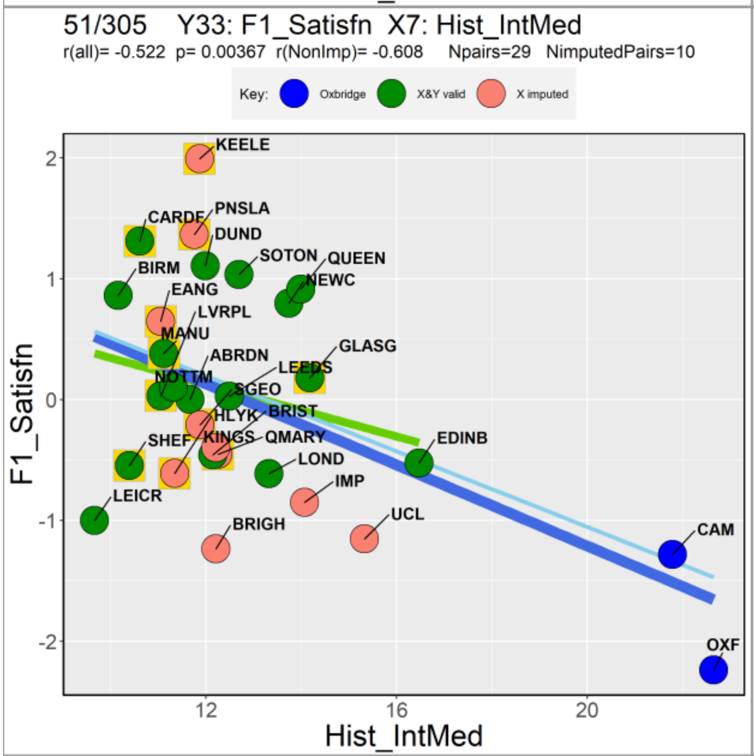
51/305 Y33: F1_Satisfn X7: Hist_IntMed
r(all)= -0.522 p= 0.00367 r(NonImp)= -0.608 Npairs=29 NimputedPairs=10
Key: Oxbridge X&Y valid X imputed
F1_Satisfn
Hist_IntMed

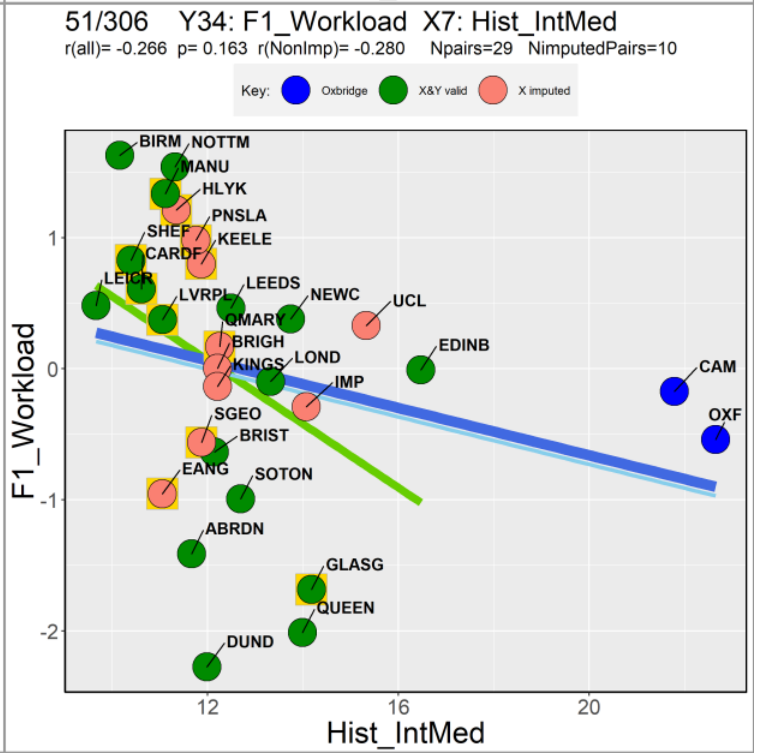
51/306 Y34: F1_Workload X7: Hist_IntMed
r(all)= -0.266 p= 0.163 r(NonImp)= -0.280 Npairs=29 NimputedPairs=10
Key: Oxbridge X&Y valid X imputed
F1_Workload
Hist_IntMed

52/307 Y35: F1_Supervn X7: Hist_IntMed
r(all)= 0.379 p= 0.0427 r(NonImp)= 0.441 Npairs=29 NimputedPairs=10

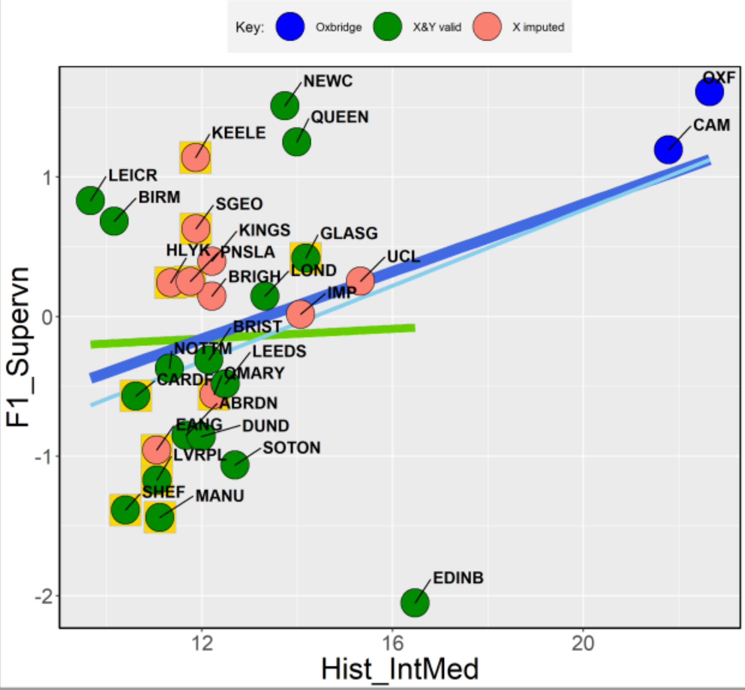

52/308 Y36: Trainee_GP X7: Hist_IntMed
r(all)= -0.556 p= 0.00174 r(NonImp)= -0.637 Npairs=29 NimputedPairs=10

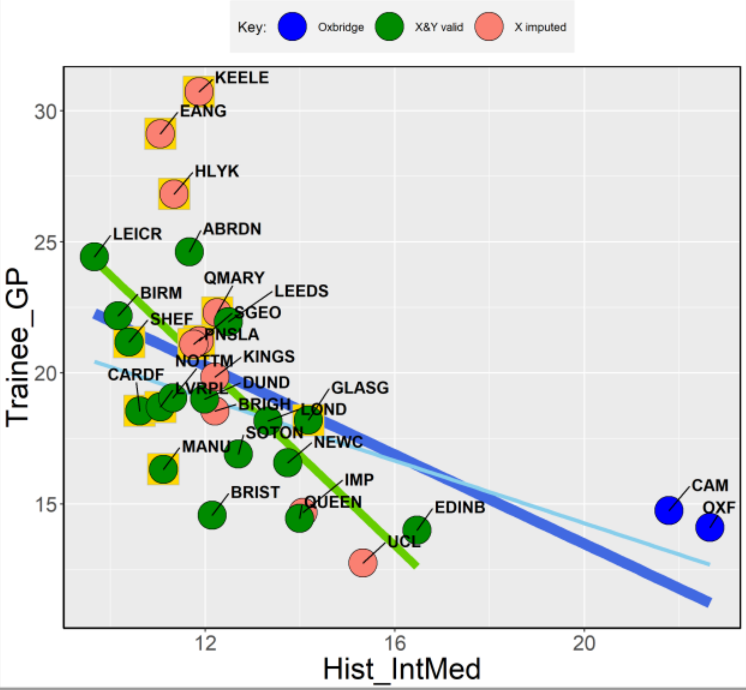

52/309 Y37: Trainee_Psyc X7: Hist_IntMed
r(all)= 0.050 p= 0.795 r(NonImp)= 0.145 Npairs=29 NimputedPairs=10

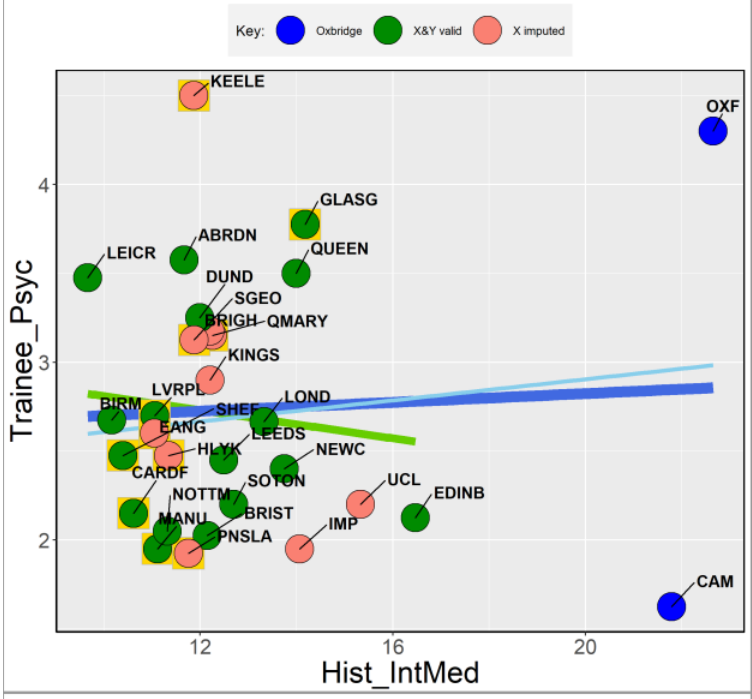

52/310 Y38: TraineeApp_Surgery X7: Hist_IntMed
r(all)= 0.138 p= 0.475 r(NonImp)= 0.086 Npairs=29 NimputedPairs=10

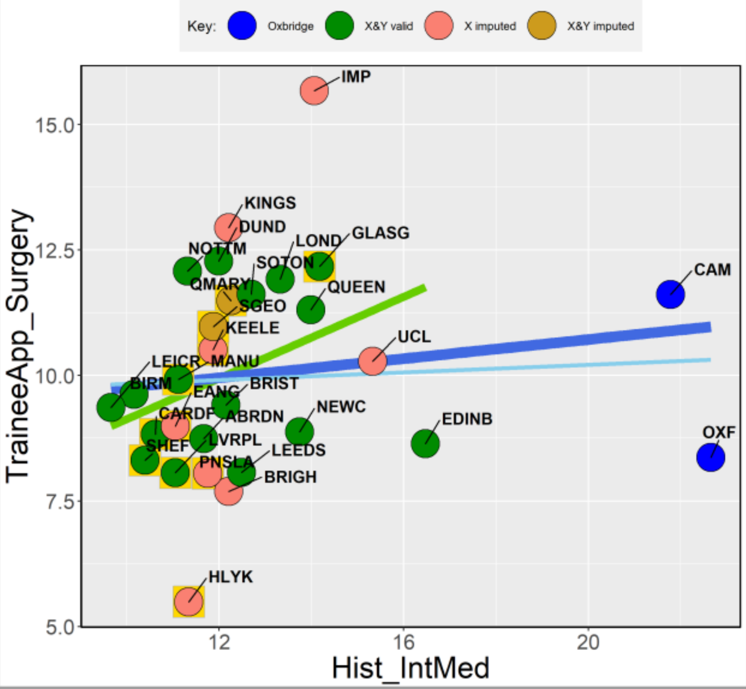

52/311 Y39: TraineeApp_Anaes X7: Hist_IntMed
r(all)= 0.313 p= 0.0984 r(NonImp)= 0.368 Npairs=29 NimputedPairs=10

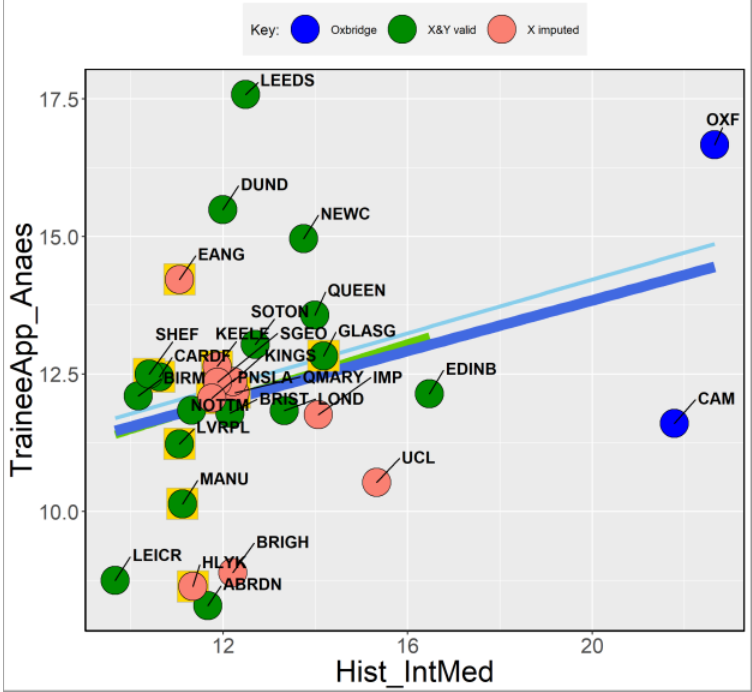

52/312 Y40: GMC_PGexams X7: Hist_IntMed
r(all)= 0.712 p= 1.48e-05 r(NonImp)= 0.767 Npairs=29 NimputedPairs=10

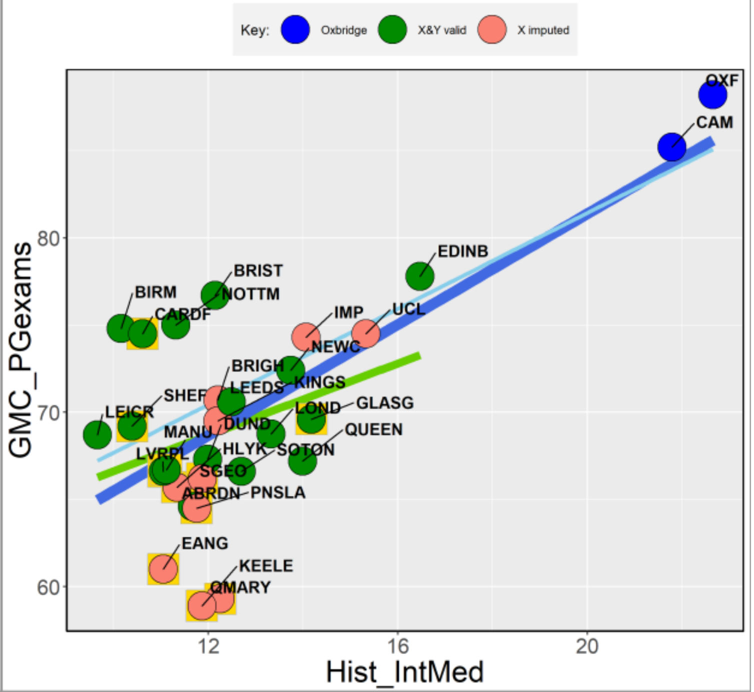

53/313 Y41: MRCGP_AKT X7: Hist_IntMed

r(all)= 0.729 p= 7.39e-06 r(NonImp)= 0.756 Npairs=29 NimputedPairs=10

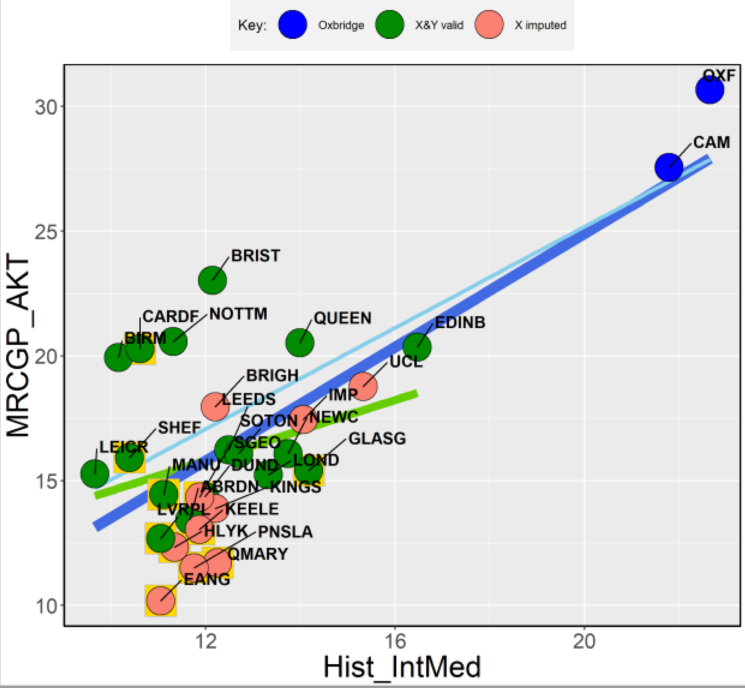

53/314 Y42: MRCGP_CSA X7: Hist_IntMed

r(all)= 0.673 p= 6.26e-05 r(NonImp)= 0.722 Npairs=29 NimputedPairs=10

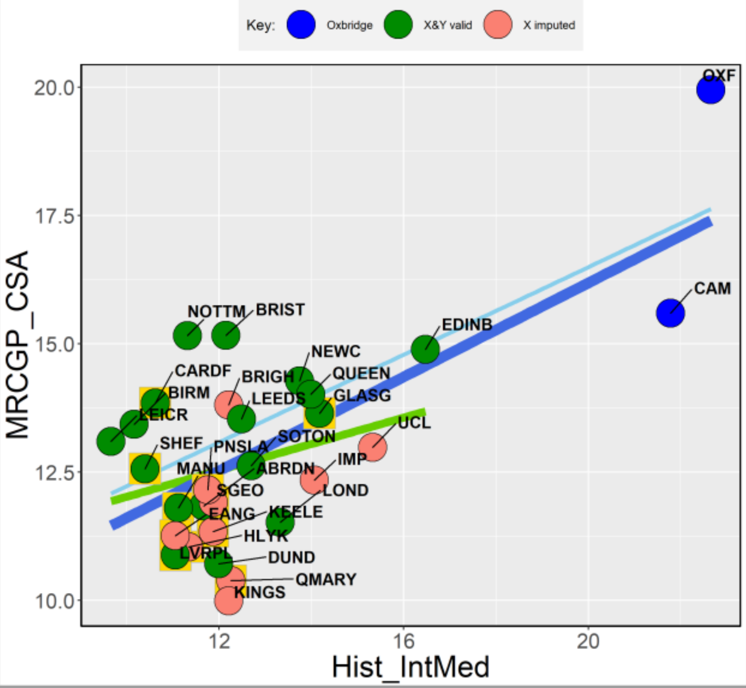

53/315 Y43: FRCA_Pt1 X7: Hist_IntMed

r(all)= 0.610 p= 0.000437 r(NonImp)= 0.603 Npairs=29 NimputedPairs=10

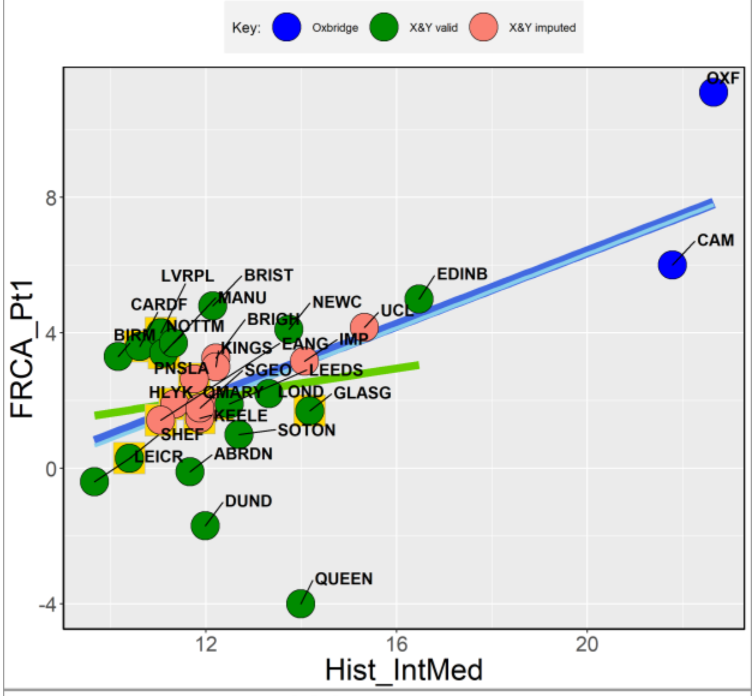

53/316 Y44: MRCOG_Pt1 X7: Hist_IntMed

r(all)= 0.726 p= 8.34e-06 r(NonImp)= 0.712 Npairs=29 NimputedPairs=10

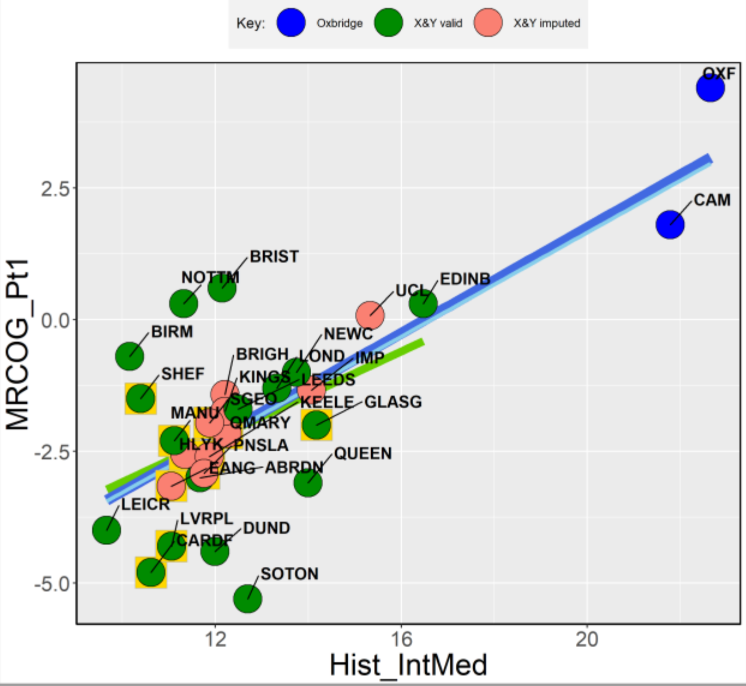

53/317 Y45: MRCOG_Pt2 X7: Hist_IntMed

r(all)= 0.657 p= 0.000108 r(NonImp)= 0.642 Npairs=29 NimputedPairs=10

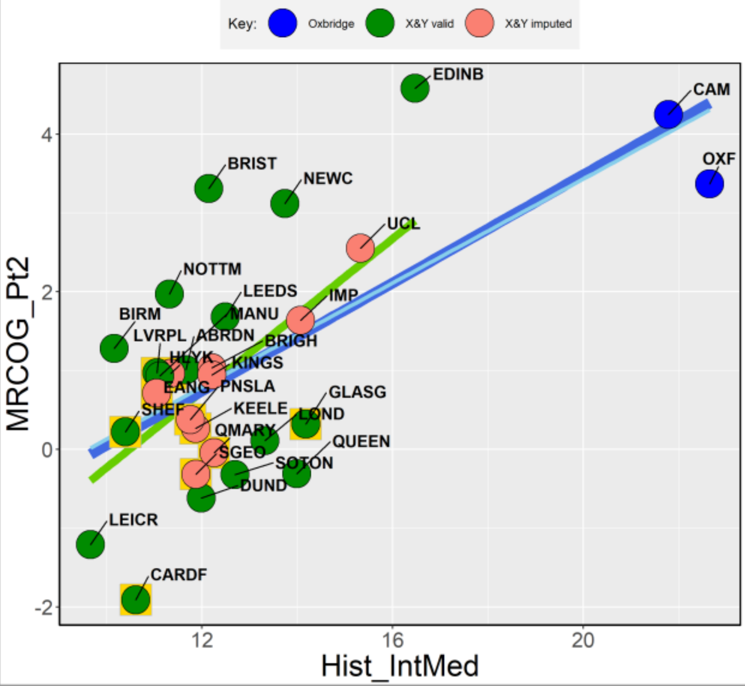

53/318 Y46: MRCP_Pt1 X7: Hist_IntMed

r(all)= 0.759 p= 1.84e-06 r(NonImp)= 0.806 Npairs=29 NimputedPairs=10

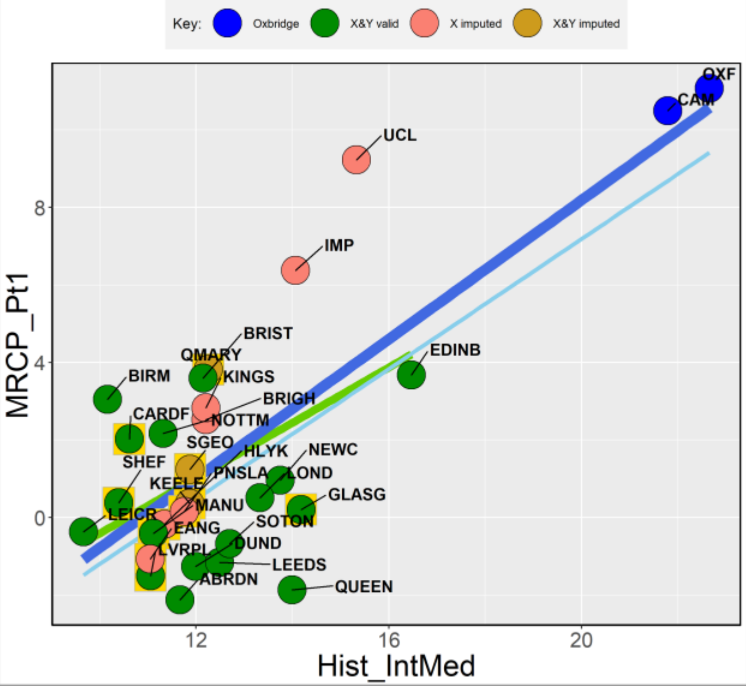

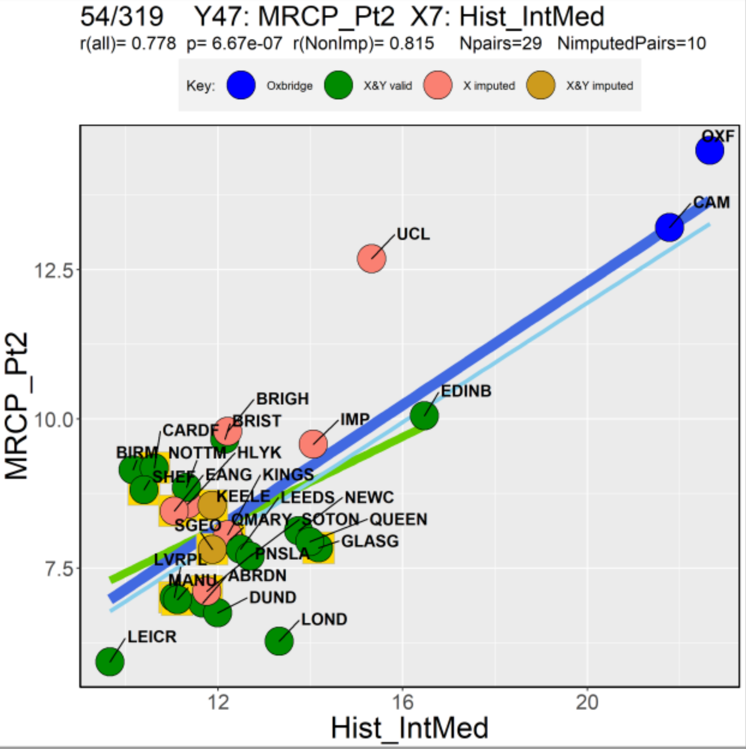
54/319 Y47: MRCP_Pt2 X7: Hist_IntMed
r(all)= 0.778 p= 6.67e-07 r(NonImp)= 0.815 Npairs=29 NimputedPairs=10
Key: Oxbridge X&Y valid X imputed X&Y imputed
MRCP_Pt2
Hist_IntMed

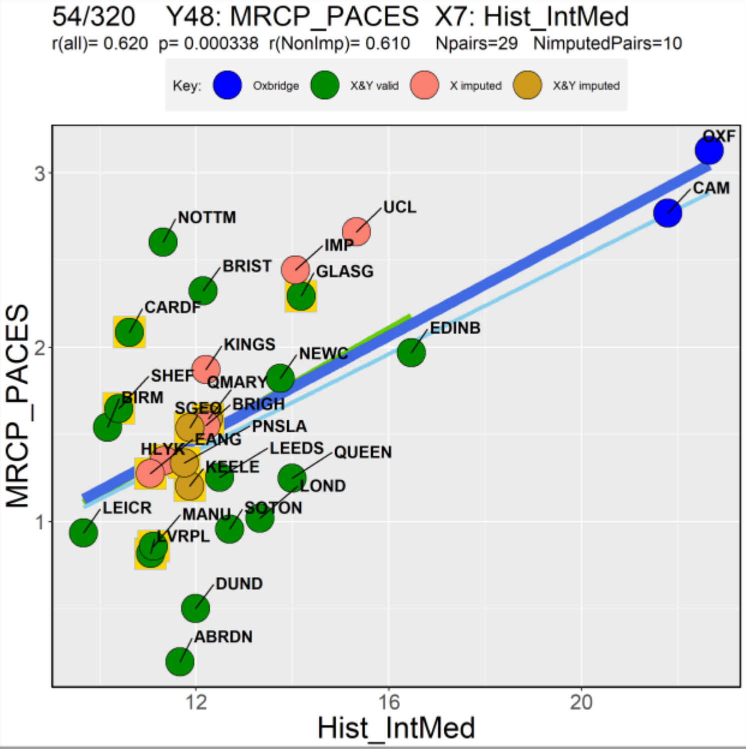
54/320 Y48: MRCP_PACES X7: Hist_IntMed
r(all)= 0.620 p= 0.000338 r(NonImp)= 0.610 Npairs=29 NimputedPairs=10
Key: Oxbridge X&Y valid X imputed X&Y imputed
MRCP_PACES
Hist_IntMed

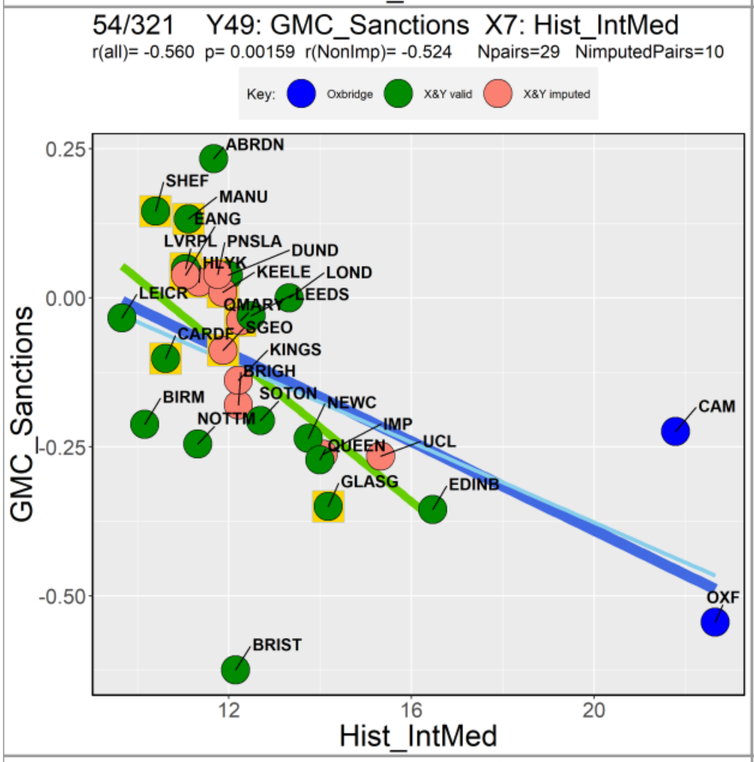
54/321 Y49: GMC_Sanctions X7: Hist_IntMed
r(all)= -0.560 p= 0.00159 r(NonImp)= -0.524 Npairs=29 NimputedPairs=10
Key: Oxbridge X&Y valid X&Y imputed
GMC_Sanctions
Hist_IntMed

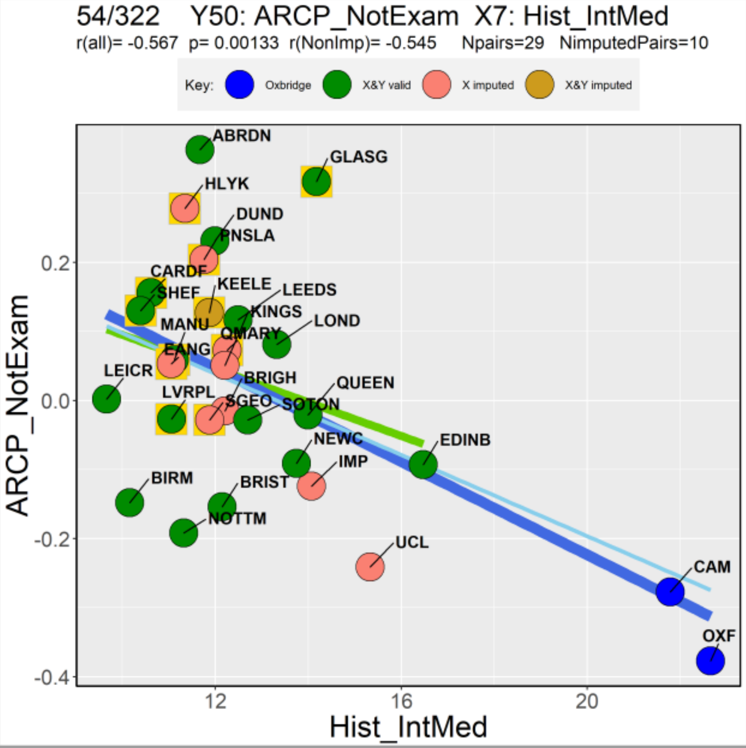
54/322 Y50: ARCP_NotExam X7: Hist_IntMed
r(all)= -0.567 p= 0.00133 r(NonImp)= -0.545 Npairs=29 NimputedPairs=10
Key: Oxbridge X&Y valid X imputed X&Y imputed
ARCP_NotExam
Hist_IntMed

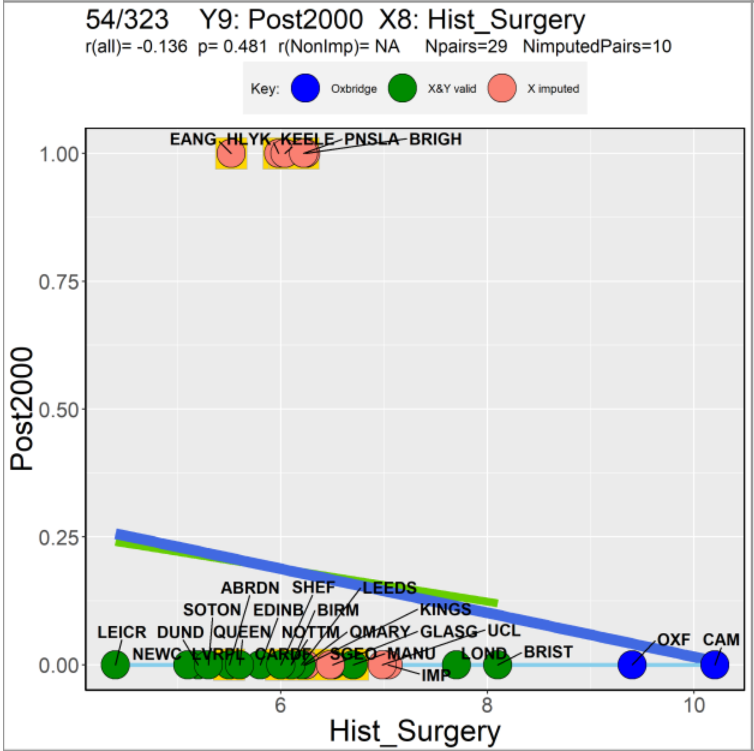
54/323 Y9: Post2000 X8: Hist_Surgery
r(all)= -0.136 p= 0.481 r(NonImp)= NA Npairs=29 NimputedPairs=10
Key: Oxbridge X&Y valid X imputed
Post2000
Hist_Surgery

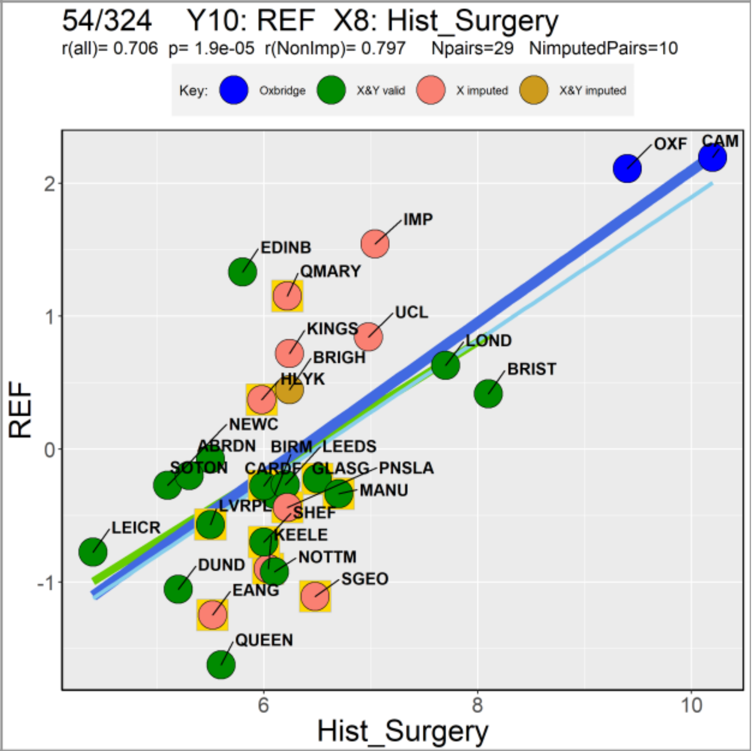
54/324 Y10: REF X8: Hist_Surgery
r(all)= 0.706 p= 1.9e-05 r(NonImp)= 0.797 Npairs=29 NimputedPairs=10
Key: Oxbridge X&Y valid X imputed X&Y imputed
REF
Hist_Surgery

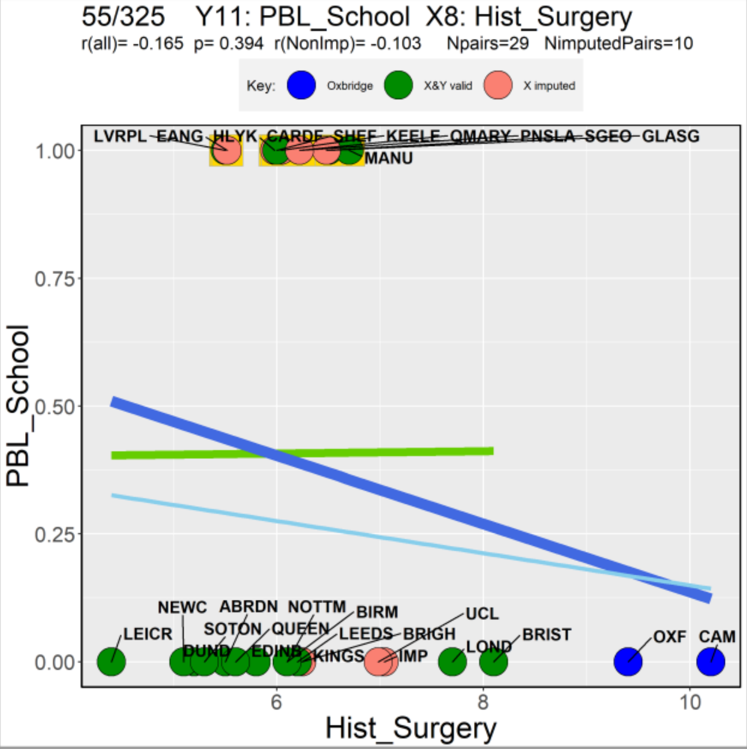
55/325 Y11: PBL_School X8: Hist_Surgery
r(all)= -0.165 p= 0.394 r(NonImp)= -0.103 Npairs=29 NimputedPairs=10
Key: Oxbridge X&Y valid X imputed
PBL_School
Hist_Surgery
LVRPL EANG HLYK CARDF SHEF KEELE QMARY PNSLA SGEO GLASG
MANU
NEWC ABRDN NOTTM BIRM UCL
LEICR SOTON QUEEN LEEDS BRIGH BRIST OXF CAM
DUND EDINB KINGS IMP LOND
1.00
0.75
0.50
0.25
0.00
6
8
10

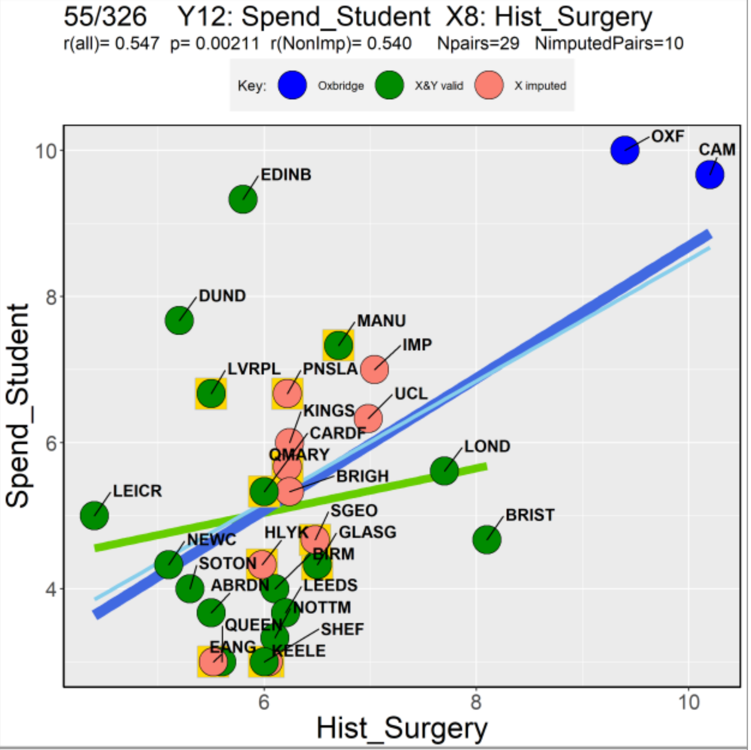
55/326 Y12: Spend_Student X8: Hist_Surgery
r(all)= 0.547 p= 0.00211 r(NonImp)= 0.540 Npairs=29 NimputedPairs=10
Key: Oxbridge X&Y valid X imputed
Spend_Student
Hist_Surgery
OXF
CAM
EDINB
DUND
MANU
IMP
LVRPL
PNSLA
UCL
KINGS
CARDF
QMARY
LOND
BRIGH
LEICR
SGEO
BRIST
NEWC
HLYK
GLASG
SOTON
BIRM
ABRDN
LEEDS
NOTTM
QUEEN
SHEF
EANG
KEELE
10
8
6
4
6
8
10

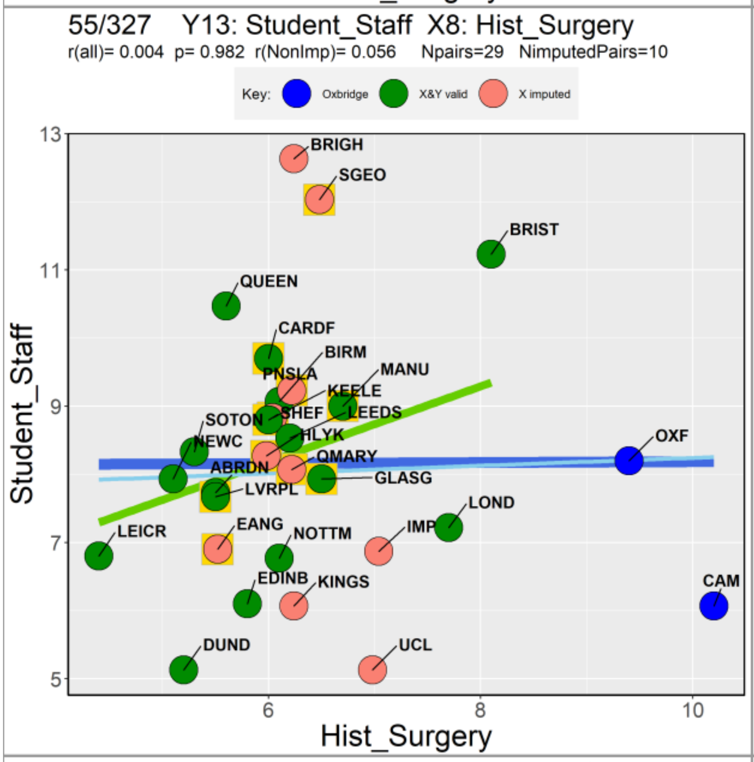
55/327 Y13: Student_Staff X8: Hist_Surgery
r(all)= 0.004 p= 0.982 r(NonImp)= 0.056 Npairs=29 NimputedPairs=10
Key: Oxbridge X&Y valid X imputed
Student_Staff
Hist_Surgery
BRIGH
SGEO
BRIST
QUEEN
CARDF
BIRM
MANU
PNSLA
KEELE
SOTON
SHEF
LEEDS
HLYK
NEWC
OXF
QMARY
ABRDN
GLASG
LVRPL
LOND
LEICR
EANG
NOTTM
IMP
CAM
EDINB
KINGS
DUND
UCL
13
11
9
7
5
6
8
10

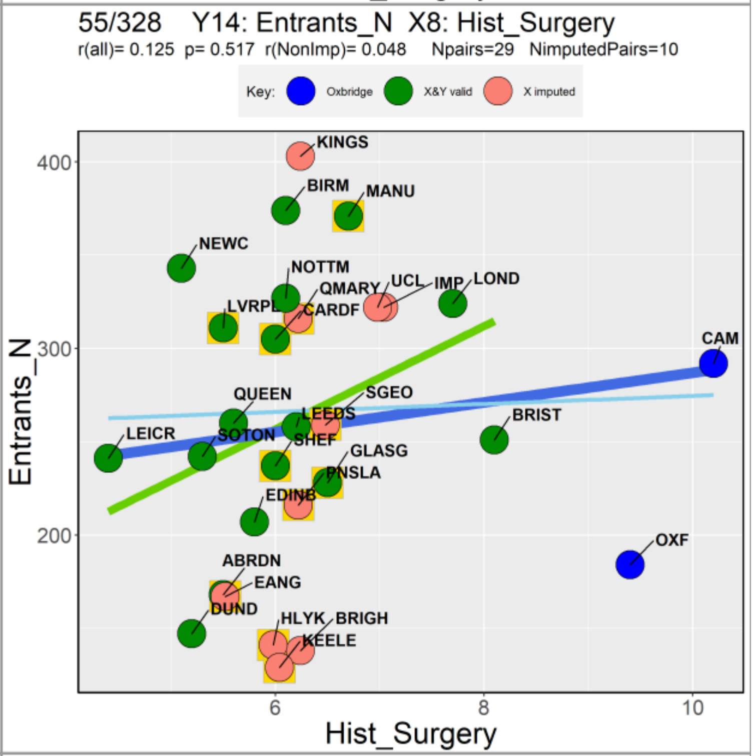
55/328 Y14: Entrants_N X8: Hist_Surgery
r(all)= 0.125 p= 0.517 r(NonImp)= 0.048 Npairs=29 NimputedPairs=10
Key: Oxbridge X&Y valid X imputed
Entrants_N
Hist_Surgery
KINGS
BIRM
MANU
NEWC
NOTTM
QMARY
UCL
IMP
LOND
LVRPL
CARDF
CAM
QUEEN
SGEO
LEEDS
BRIST
LEICR
SOTON
SHEF
GLASG
PNSLA
EDINB
OXF
ABRDN
EANG
DUND
HLYK
BRIGH
KEELE
400
300
200
6
8
10

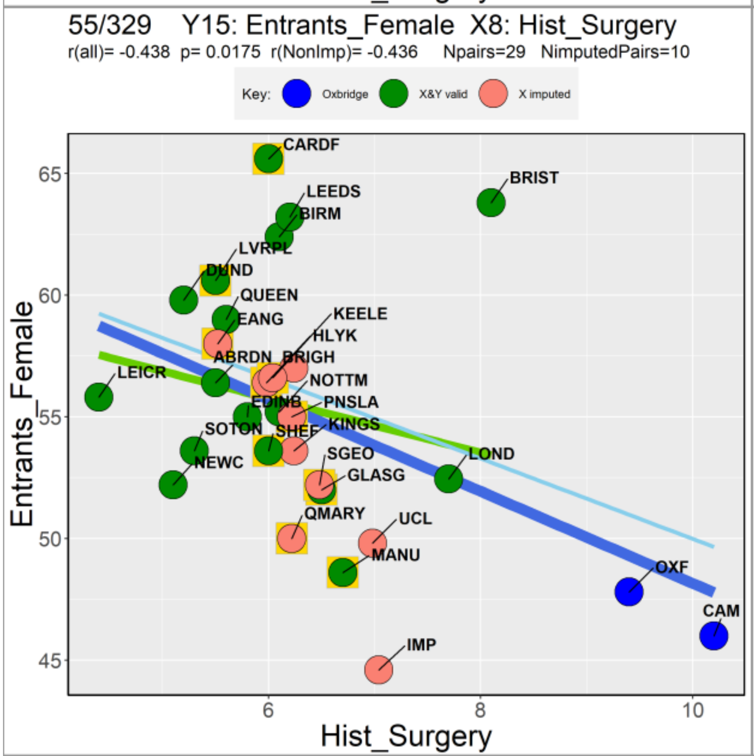
55/329 Y15: Entrants_Female X8: Hist_Surgery
r(all)= -0.438 p= 0.0175 r(NonImp)= -0.436 Npairs=29 NimputedPairs=10
Key: Oxbridge X&Y valid X imputed
Entrants_Female
Hist_Surgery
CARDF
BRIST
LEEDS
BIRM
LVRPL
DUND
QUEEN
KEELE
EANG
HLYK
LEICR
ABRDN
BRIGH
NOTTM
EDINB
PNSLA
SOTON
SHEF
KINGS
SGEO
LOND
NEWC
GLASG
QMARY
UCL
MANU
OXF
CAM
IMP
65
60
55
50
45
6
8
10

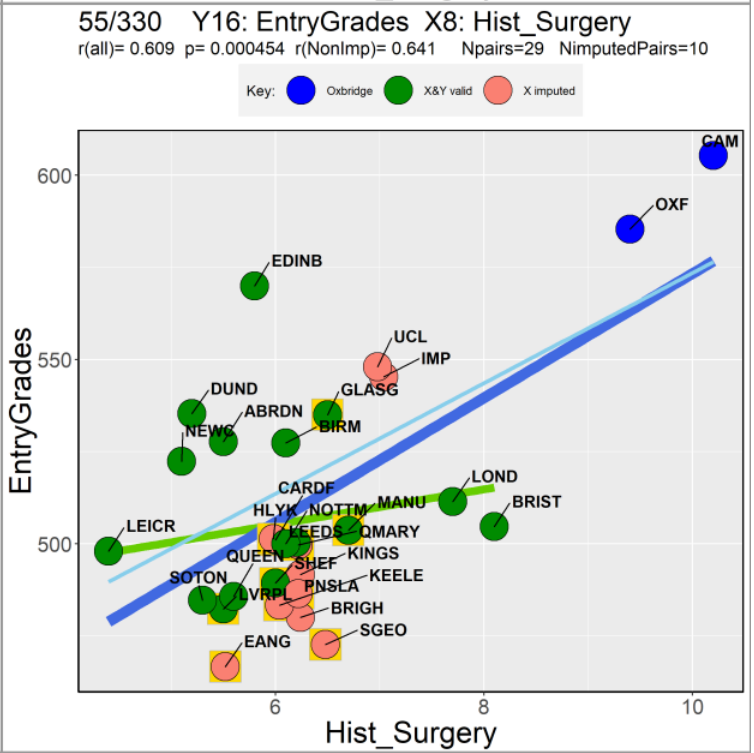
55/330 Y16: EntryGrades X8: Hist_Surgery
r(all)= 0.609 p= 0.000454 r(NonImp)= 0.641 Npairs=29 NimputedPairs=10
Key: Oxbridge X&Y valid X imputed
EntryGrades
Hist_Surgery
CAM
OXF
EDINB
UCL
IMP
DUND
GLASG
ABRDN
NEWC
BIRM
CARDF
LOND
HLYK
NOTTM
MANU
BRIST
LEICR
LEEDS
QMARY
QUEEN
KINGS
SHEF
SOTON
PNSLA
KEELE
LVRPL
BRIGH
SGEO
EANG
600
550
500
6
8
10

56/331 Y17: Entrants_NonHome X8: Hist_Surgery
r(all)= -0.357 p= 0.057 r(NonImp)= -0.411 Npairs=29 NimputedPairs=10

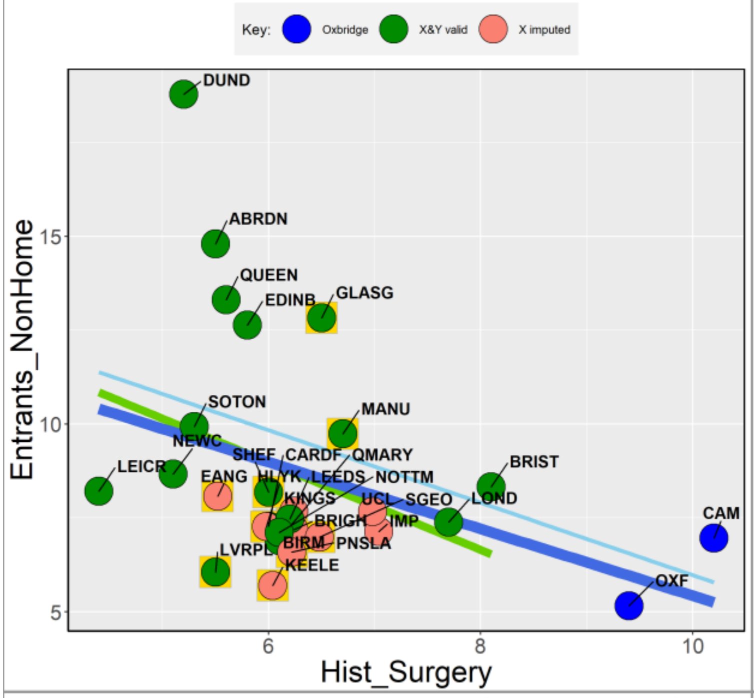

56/332 Y18: Teaching_Factor1_Trad X8: Hist_Surgery
r(all)= 0.358 p= 0.0562 r(NonImp)= 0.380 Npairs=29 NimputedPairs=12

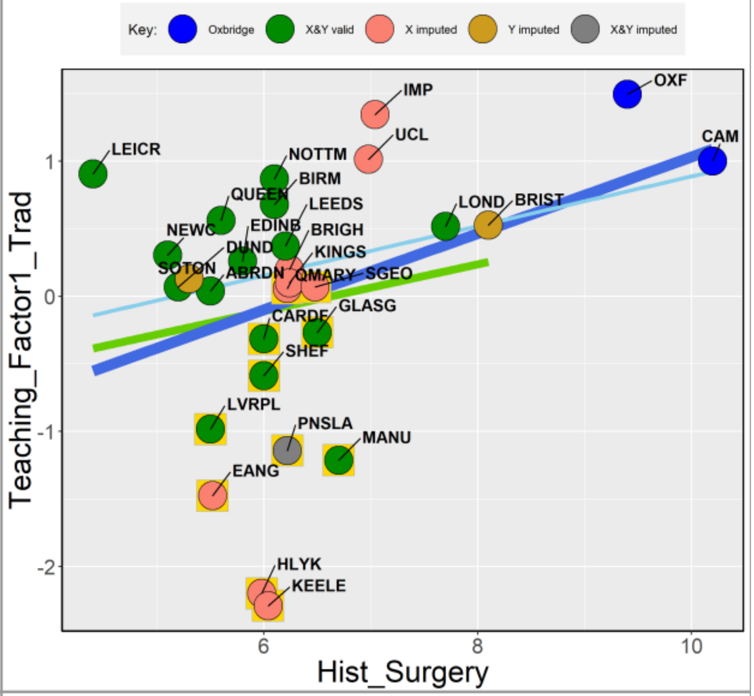

56/333 Y19: Teaching_Factor2_Struc X8: Hist_Surger
r(all)= 0.157 p= 0.415 r(NonImp)= 0.184 Npairs=29 NimputedPairs=12

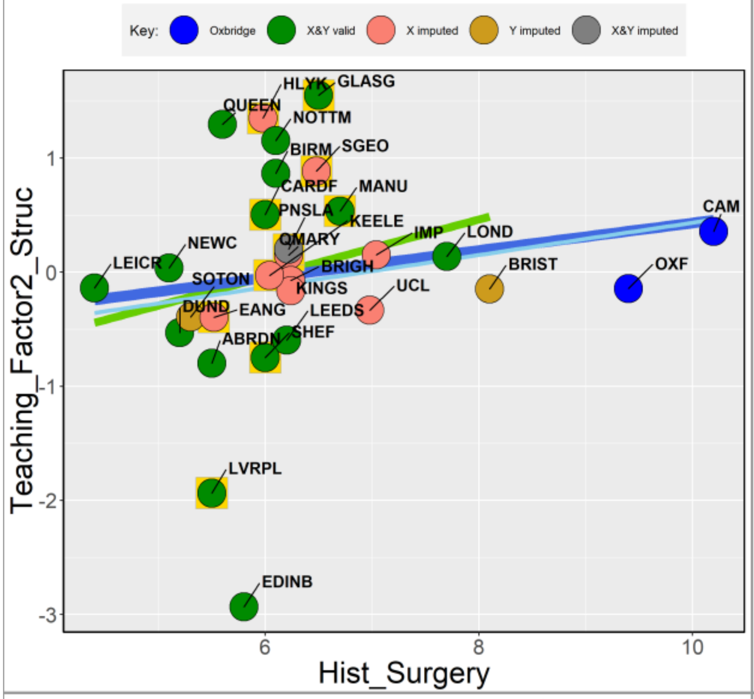

56/334 Y20: Teach_GP X8: Hist_Surgery
r(all)= -0.139 p= 0.472 r(NonImp)= 0.005 Npairs=29 NimputedPairs=12

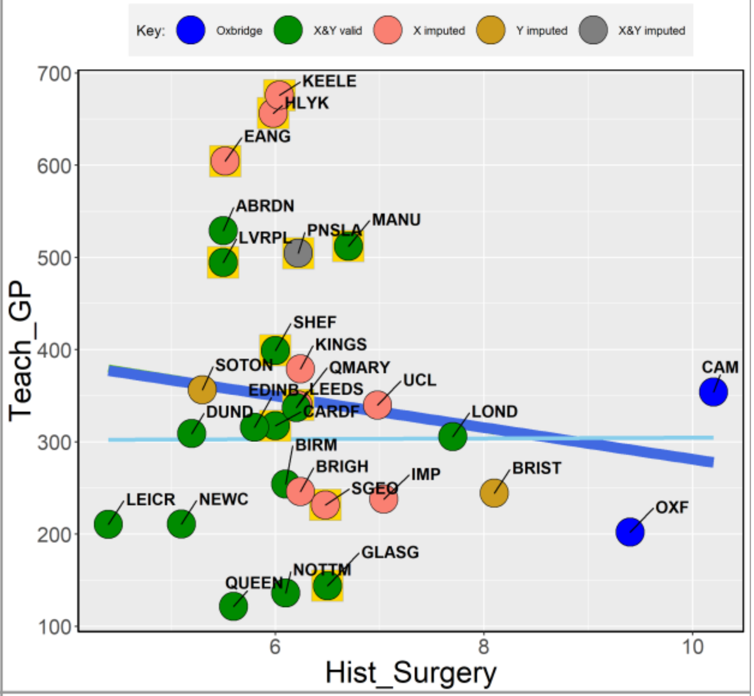

56/335 Y21: Teach_Psyc X8: Hist_Surgery
r(all)= 0.095 p= 0.624 r(NonImp)= 0.064 Npairs=29 NimputedPairs=12

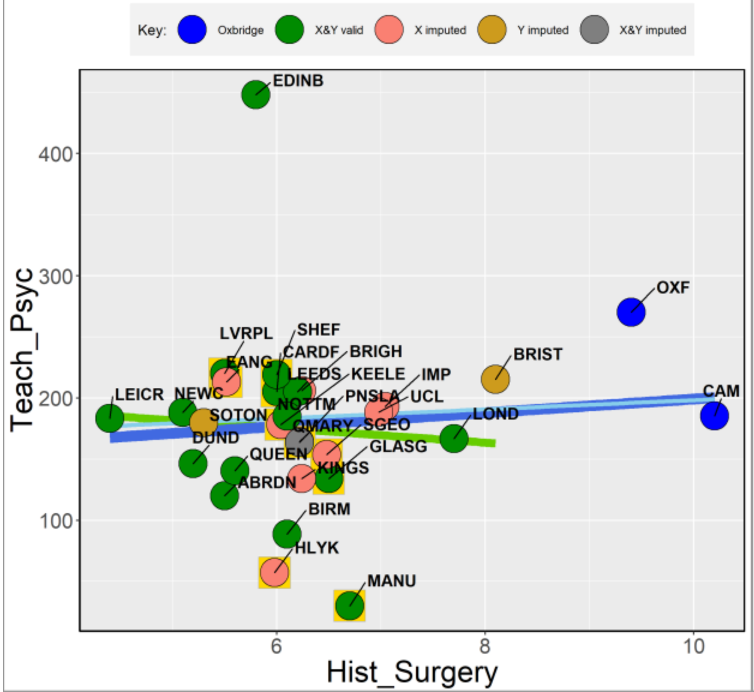

56/336 Y22: Teach_Anaes X8: Hist_Surgery
r(all)= -0.012 p= 0.952 r(NonImp)= -0.088 Npairs=29 NimputedPairs=12

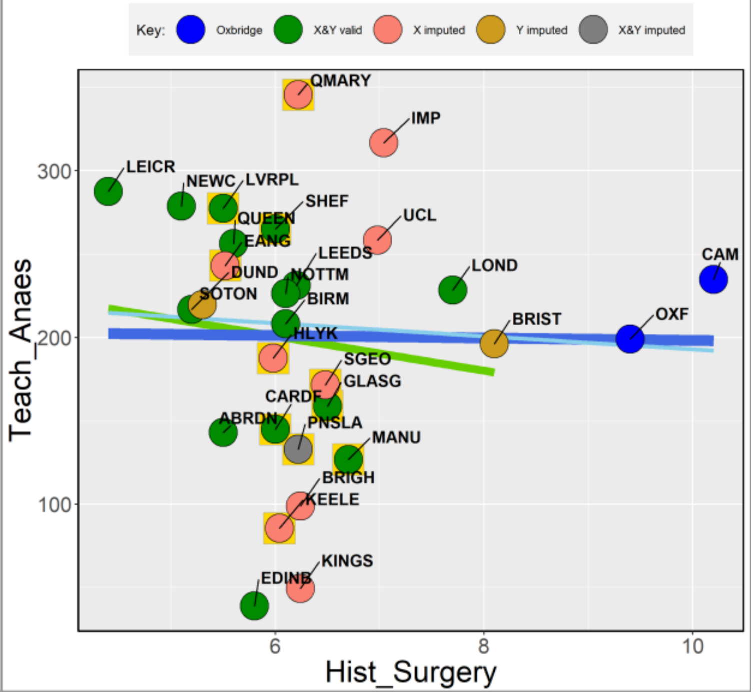

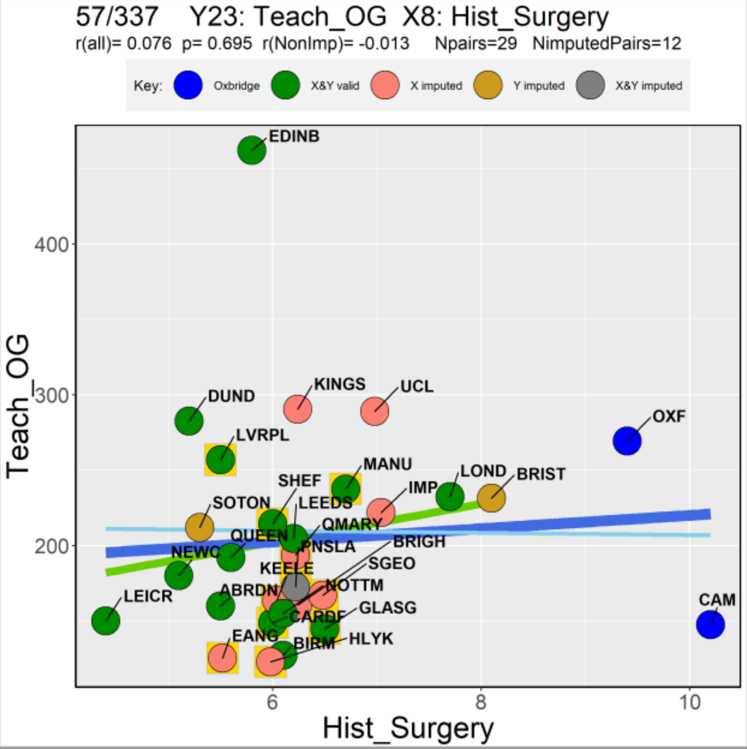
57/337 Y23: Teach_OG X8: Hist_Surgery
r(all)= 0.076 p= 0.695 r(NonImp)= -0.013 Npairs=29 NimputedPairs=12
Key: Oxbridge X&Y valid X imputed Y imputed X&Y imputed
Teach_OG
Hist_Surgery
EDINB
KINGS
UCL
DUND
LVRPL
OXF
MANU
SHEF
IMP
LOND
BRIST
SOTON
LEEDS
QMARY
QUEEN
PNSLA
BRIGH
NEWC
KEELE
SGEO
LEICR
ABRDN
NOTTM
GLASG
CARDF
EANG
BIRM
HLYK
CAM

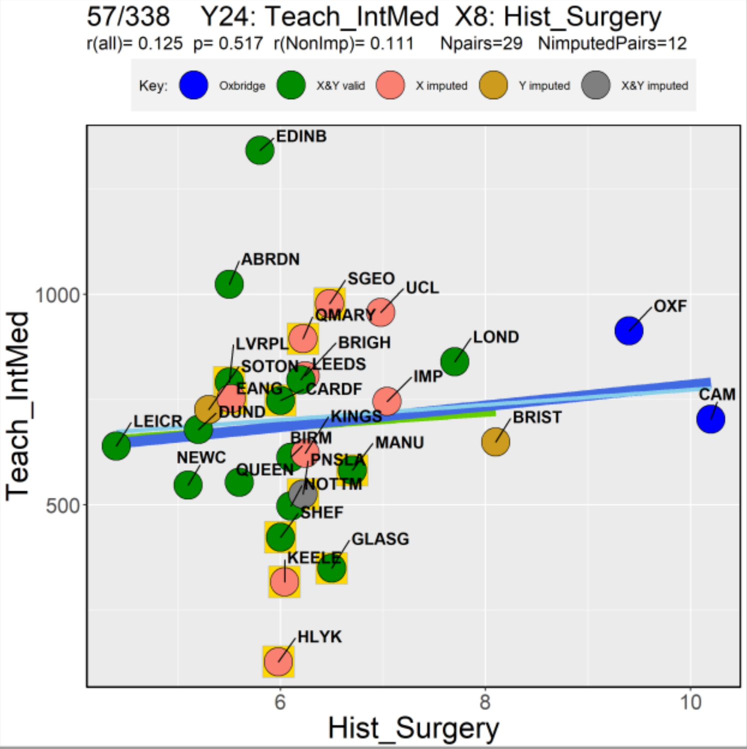
57/338 Y24: Teach_IntMed X8: Hist_Surgery
r(all)= 0.125 p= 0.517 r(NonImp)= 0.111 Npairs=29 NimputedPairs=12
Key: Oxbridge X&Y valid X imputed Y imputed X&Y imputed
Teach_IntMed
Hist_Surgery
EDINB
ABRDN
SGEO
UCL
QMARY
OXF
LVRPL
BRIGH
LOND
SOTON
LEEDS
IMP
EANG
CARDF
DUND
CAM
LEICR
KINGS
BRIST
BIRM
MANU
NEWC
PNSLA
QUEEN
NOTTM
SHEF
GLASG
KEELE
HLYK

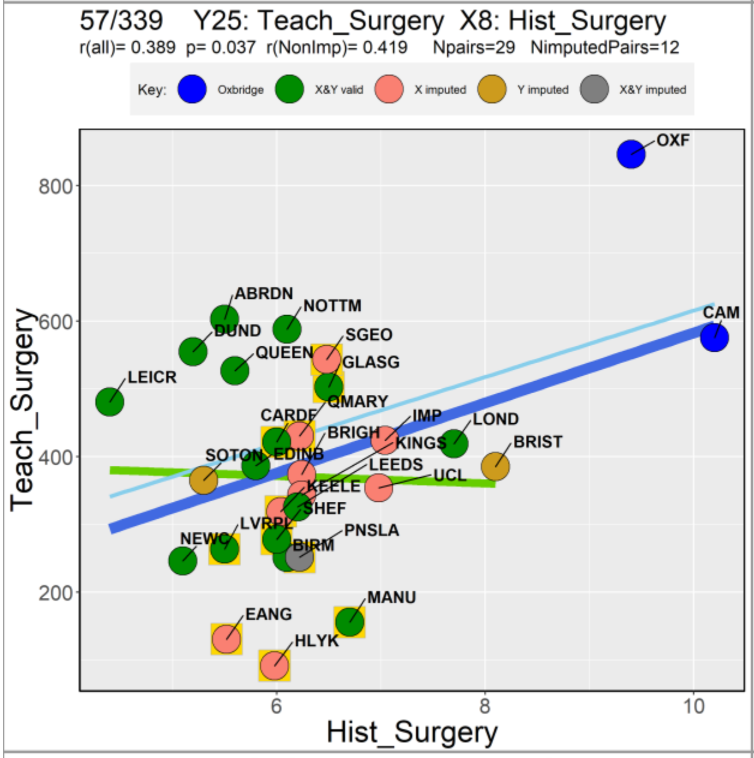
57/339 Y25: Teach_Surgery X8: Hist_Surgery
r(all)= 0.389 p= 0.037 r(NonImp)= 0.419 Npairs=29 NimputedPairs=12
Key: Oxbridge X&Y valid X imputed Y imputed X&Y imputed
Teach_Surgery
Hist_Surgery
OXF
ABRDN
NOTTM
DUND
CAM
SGEO
QUEEN
LEICR
GLASG
QMARY
CARDF
IMP
LOND
BRIGH
KINGS
BRIST
SOTON
EDINB
LEEDS
UCL
KEELE
SHEF
LVRPL
NEWC
BIRM
PNSLA
MANU
EANG
HLYK

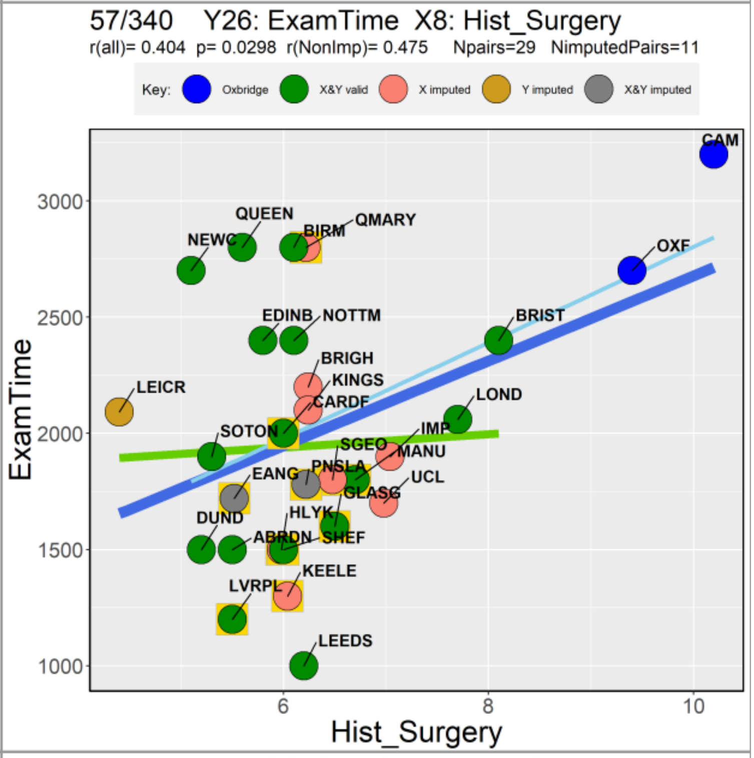
57/340 Y26: ExamTime X8: Hist_Surgery
r(all)= 0.404 p= 0.0298 r(NonImp)= 0.475 Npairs=29 NimputedPairs=11
Key: Oxbridge X&Y valid X imputed Y imputed X&Y imputed
ExamTime
Hist_Surgery
CAM
QUEEN
QMARY
NEWC
BIRM
OXF
EDINB
NOTTM
BRIST
BRIGH
KINGS
LEICR
LOND
CARDF
SOTON
IMP
SGEO
MANU
EANG
PNSLA
UCL
GLASG
DUND
HLYK
ABRDN
SHEF
KEELE
LVRPL
LEEDS

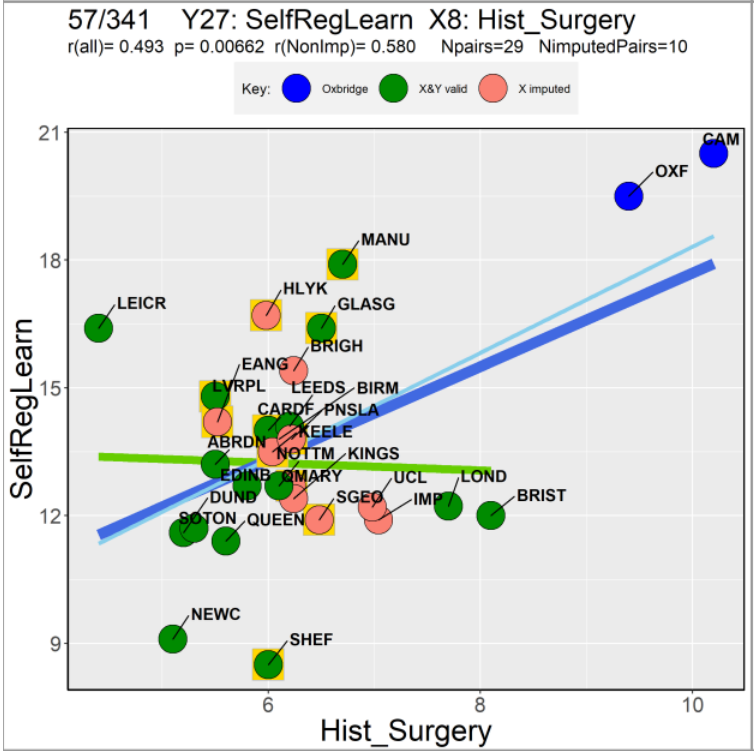
57/341 Y27: SelfRegLearn X8: Hist_Surgery
r(all)= 0.493 p= 0.00662 r(NonImp)= 0.580 Npairs=29 NimputedPairs=10
Key: Oxbridge X&Y valid X imputed
SelfRegLearn
Hist_Surgery
CAM
OXF
MANU
LEICR
HLYK
GLASG
BRIGH
EANG
LVRPL
LEEDS
BIRM
CARDF
PNSLA
KEELE
ABRDN
NOTTM
KINGS
EDINB
QMARY
UCL
LOND
DUND
SGEO
IMP
BRIST
SOTON
QUEEN
NEWC
SHEF

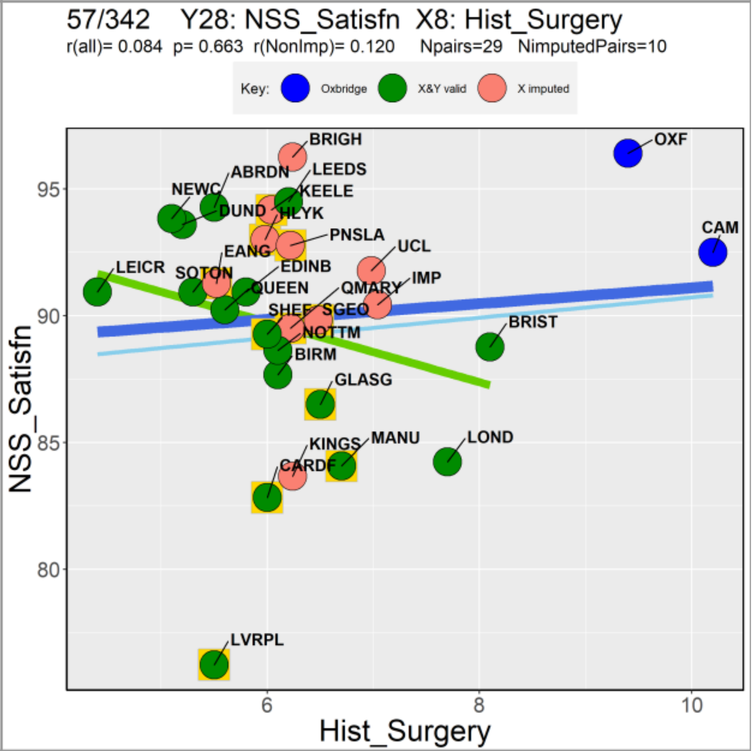
57/342 Y28: NSS_Satisfn X8: Hist_Surgery
r(all)= 0.084 p= 0.663 r(NonImp)= 0.120 Npairs=29 NimputedPairs=10
Key: Oxbridge X&Y valid X imputed
NSS_Satisfn
Hist_Surgery
BRIGH
OXF
ABRDN
LEEDS
NEWC
KEELE
DUND
HLYK
CAM
PNSLA
EANG
UCL
LEICR
SOTON
EDINB
QUEEN
QMARY
IMP
SHEF
SGEO
NOTTM
BRIST
BIRM
GLASG
KINGS
MANU
LOND
CARDF
LVRPL

58/343 Y29: NSS_Feedback X8: Hist_Surgery
r(all)= 0.214 p= 0.266 r(NonImp)= 0.314 Npairs=29 NimputedPairs=10

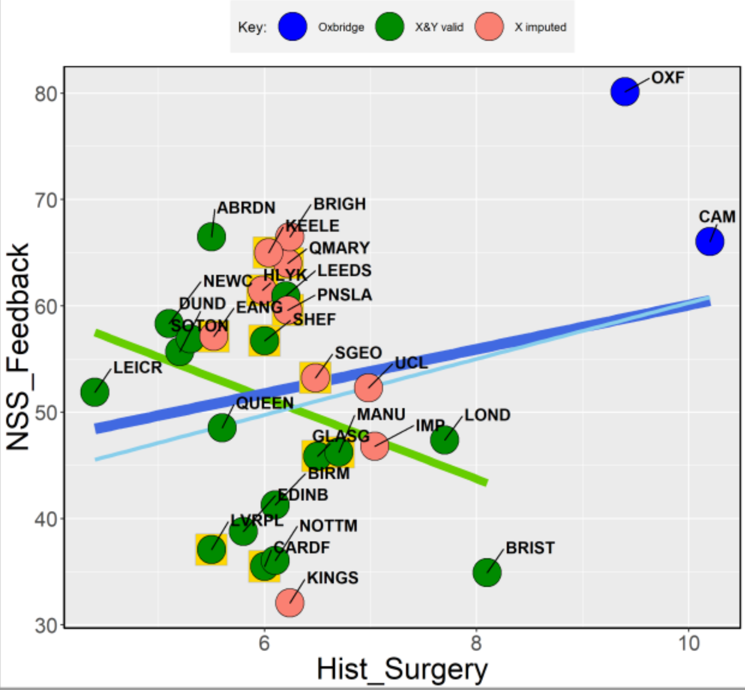

58/344 Y30: UKFPO_EPM X8: Hist_Surgery
r(all)= 0.582 p= 0.000924 r(NonImp)= 0.693 Npairs=29 NimputedPairs=10

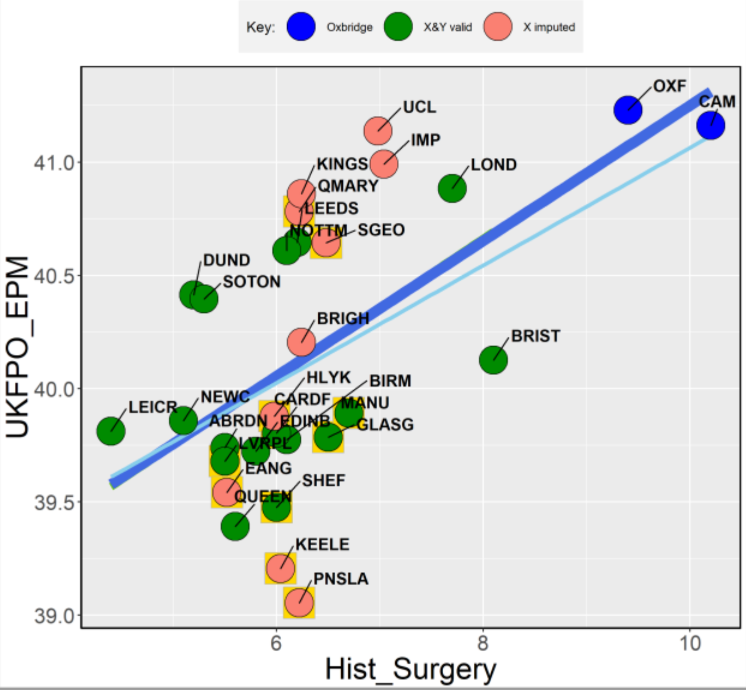

58/345 Y31: UKFPO_SJT X8: Hist_Surgery
r(all)= 0.669 p= 7.15e-05 r(NonImp)= 0.690 Npairs=29 NimputedPairs=10

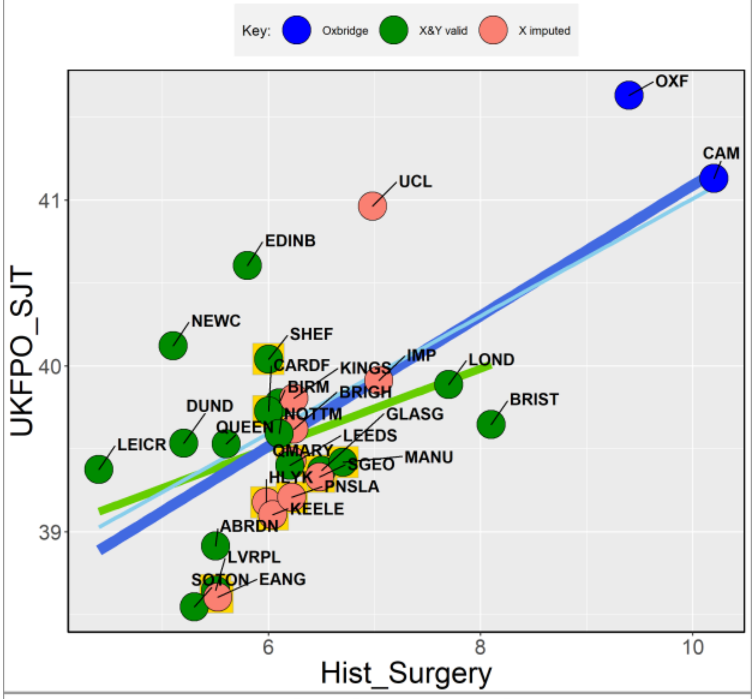

58/346 Y32: F1_Preparedness X8: Hist_Surgery
r(all)= -0.167 p= 0.386 r(NonImp)= -0.107 Npairs=29 NimputedPairs=10

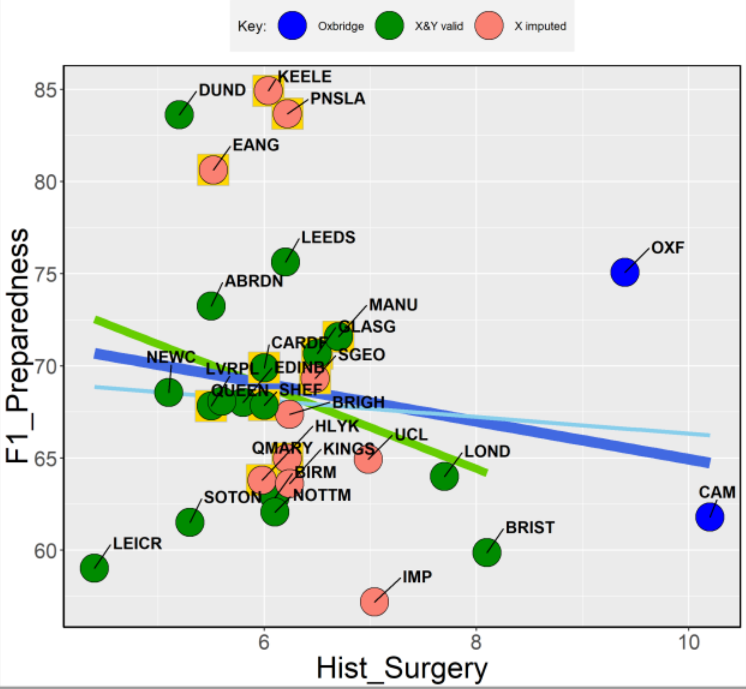

58/347 Y33: F1_Satisfn X8: Hist_Surgery
r(all)= -0.543 p= 0.00234 r(NonImp)= -0.634 Npairs=29 NimputedPairs=10

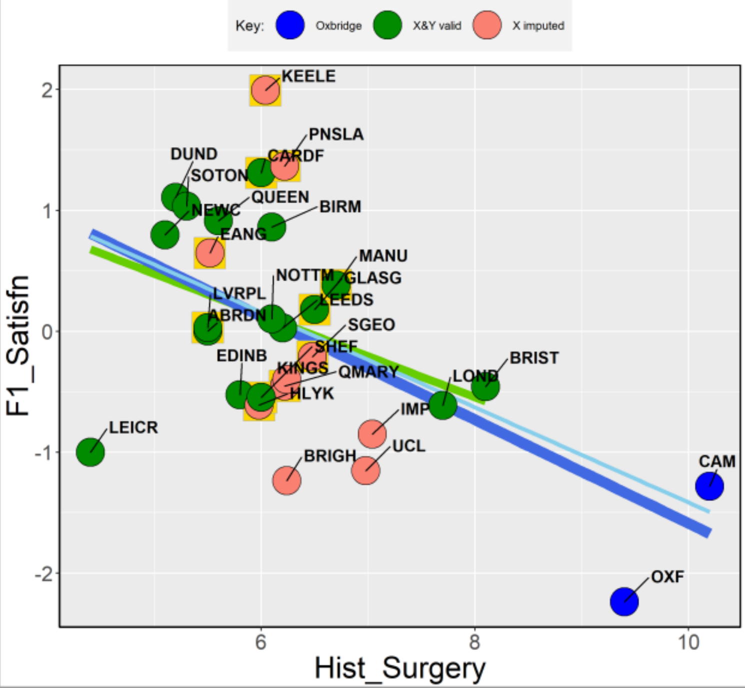

58/348 Y34: F1_Workload X8: Hist_Surgery
r(all)= -0.005 p= 0.98 r(NonImp)= 0.000 Npairs=29 NimputedPairs=10

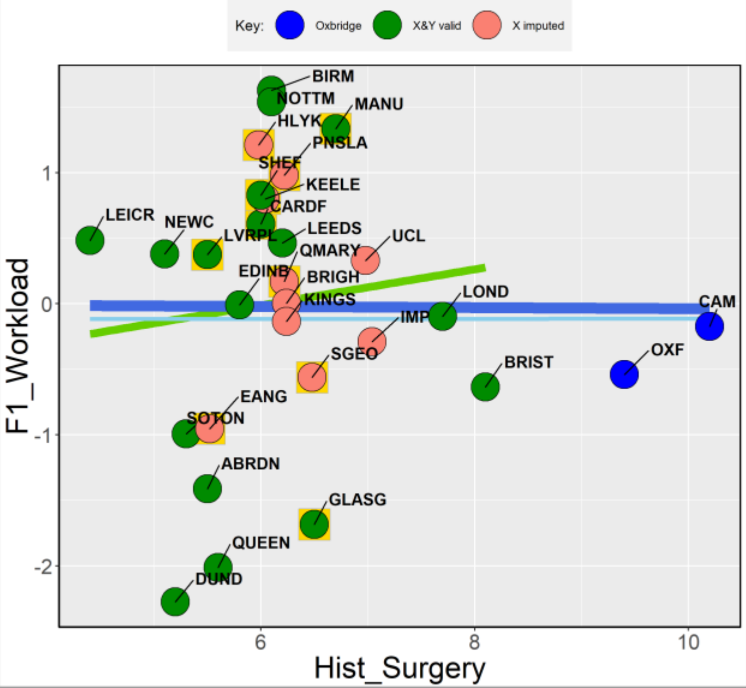

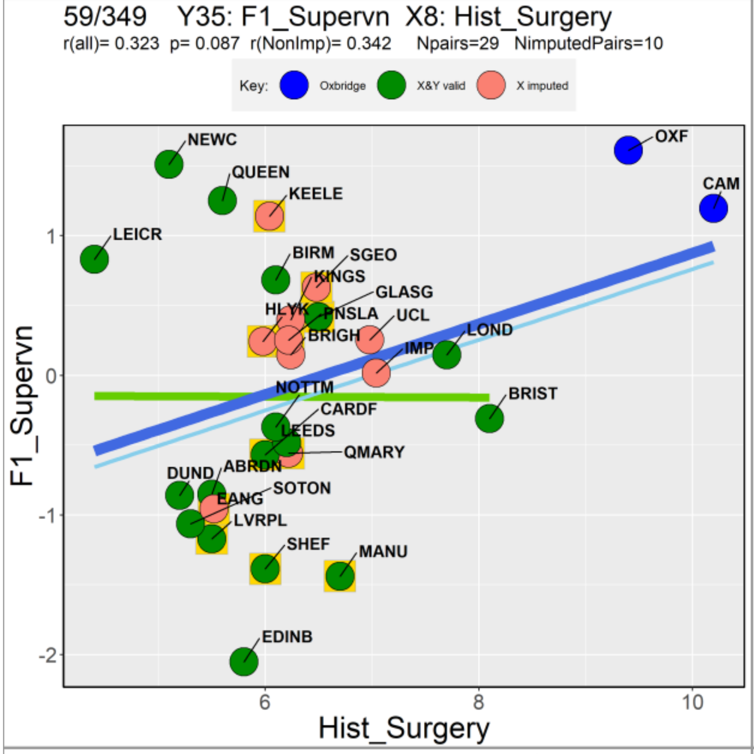

59/349 Y35: F1_Supervn X8: Hist_Surgery
r(all)= 0.323 p= 0.087 r(NonImp)= 0.342 Npairs=29 NimputedPairs=10
Key: Oxbridge X&Y valid X imputed
F1_Supervn
Hist_Surgery
NEWC
QUEEN
KEELE
LEICR
BIRM
SGEO
KINGS
GLASG
HLYK
PNSLA
UCL
BRIGH
IMP
LOND
OXF
CAM
NOTTM
BRIST
CARDF
LEEDS
QMARY
DUND
ABRDN
SOTON
EANG
LVRPL
SHEF
MANU
EDINB

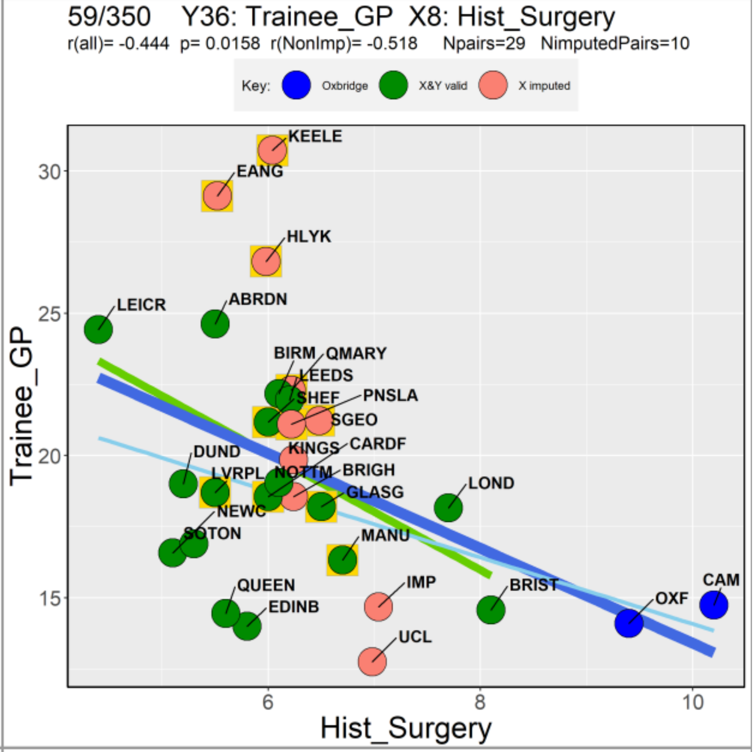

59/350 Y36: Trainee_GP X8: Hist_Surgery
r(all)= -0.444 p= 0.0158 r(NonImp)= -0.518 Npairs=29 NimputedPairs=10
Key: Oxbridge X&Y valid X imputed
Trainee_GP
Hist_Surgery
KEELE
EANG
HLYK
LEICR
ABRDN
BIRM
QMARY
LEEDS
SHEF
PNSLA
SGEO
KINGS
CARDF
DUND
LVRPL
NOTTM
BRIGH
GLASG
LOND
NEWC
SOTON
MANU
QUEEN
EDINB
IMP
BRIST
UCL
OXF
CAM

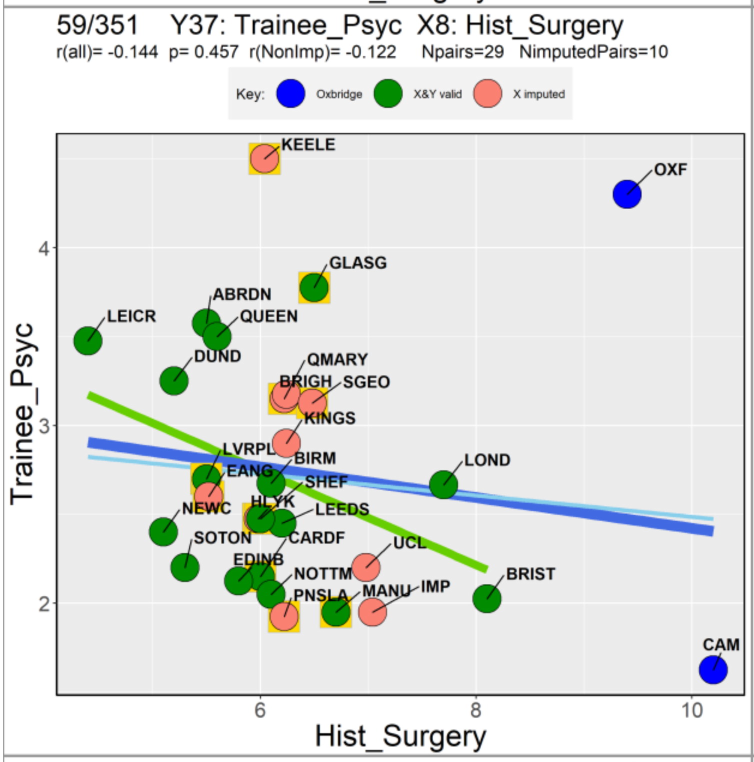

59/351 Y37: Trainee_Psyc X8: Hist_Surgery
r(all)= -0.144 p= 0.457 r(NonImp)= -0.122 Npairs=29 NimputedPairs=10
Key: Oxbridge X&Y valid X imputed
Trainee_Psyc
Hist_Surgery
KEELE
OXF
GLASG
ABRDN
LEICR
QUEEN
DUND
QMARY
BRIGH
SGEO
KINGS
LVRPL
BIRM
EANG
LOND
SHEF
NEWC
HLYK
LEEDS
SOTON
CARDF
UCL
EDINB
NOTTM
BRIST
PNSLA
MANU
IMP
CAM

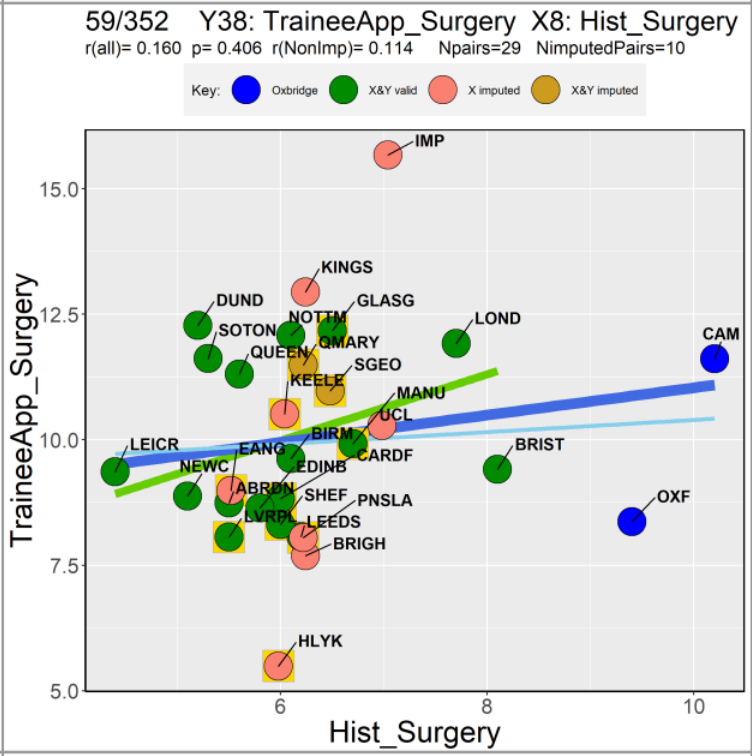

59/352 Y38: TraineeApp_Surgery X8: Hist_Surgery
r(all)= 0.160 p= 0.406 r(NonImp)= 0.114 Npairs=29 NimputedPairs=10
Key: Oxbridge X&Y valid X imputed X&Y imputed
TraineeApp_Surgery
Hist_Surgery
IMP
KINGS
DUND
GLASG
NOTTM
LOND
SOTON
QUEEN
QMARY
CAM
SGEO
KEELE
MANU
UCL
BIRM
LEICR
EANG
BRIST
CARDF
NEWC
EDINB
ABRDN
SHEF
OXF
LVRPL
PNSLA
LEEDS
BRIGH
HLYK

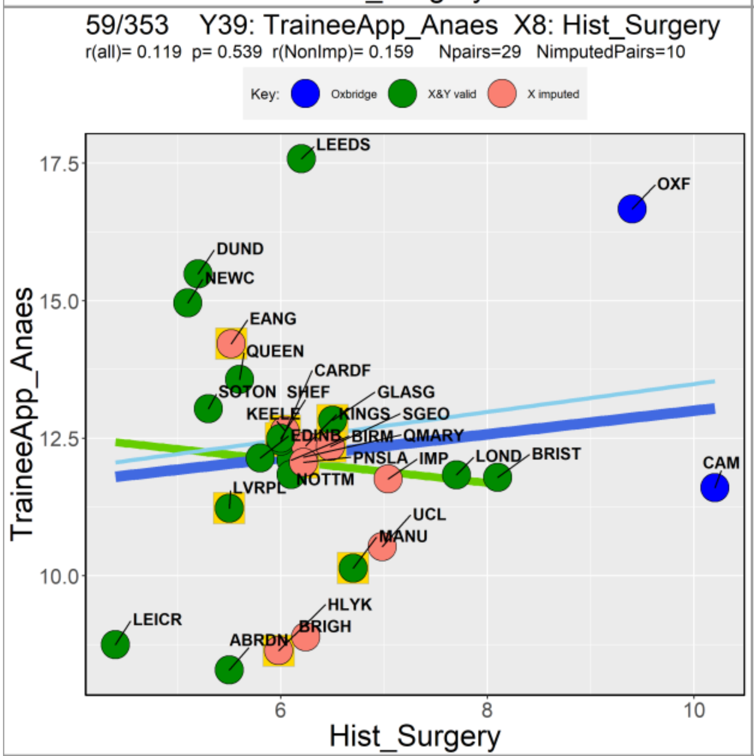

59/353 Y39: TraineeApp_Anaes X8: Hist_Surgery
r(all)= 0.119 p= 0.539 r(NonImp)= 0.159 Npairs=29 NimputedPairs=10
Key: Oxbridge X&Y valid X imputed
TraineeApp_Anaes
Hist_Surgery
LEEDS
OXF
DUND
NEWC
EANG
QUEEN
CARDF
SOTON
SHEF
GLASG
KEELE
KINGS
SGEO
EDINB
BIRM
QMARY
PNSLA
IMP
LOND
BRIST
CAM
LVRPL
NOTTM
UCL
MANU
HLYK
LEICR
BRIGH
ABRDN

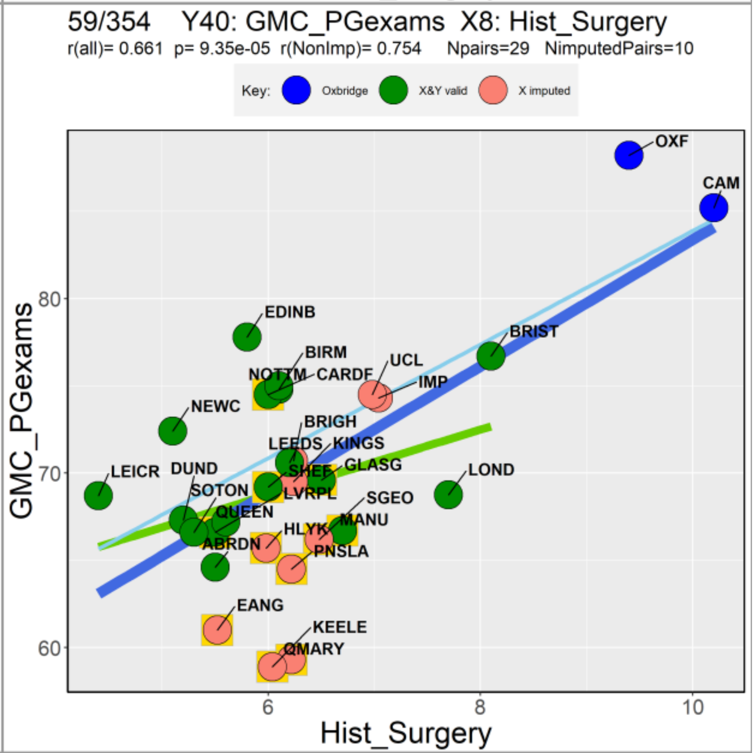

59/354 Y40: GMC_PGexams X8: Hist_Surgery
r(all)= 0.661 p= 9.35e-05 r(NonImp)= 0.754 Npairs=29 NimputedPairs=10
Key: Oxbridge X&Y valid X imputed
GMC_PGexams
Hist_Surgery
OXF
CAM
EDINB
BRIST
BIRM
UCL
NOTTM
CARDF
IMP
NEWC
BRIGH
LEEDS
KINGS
LEICR
DUND
SHEF
GLASG
LOND
SOTON
LVRPL
SGEO
QUEEN
HLYK
MANU
ABRDN
PNSLA
EANG
KEELE
QMARY

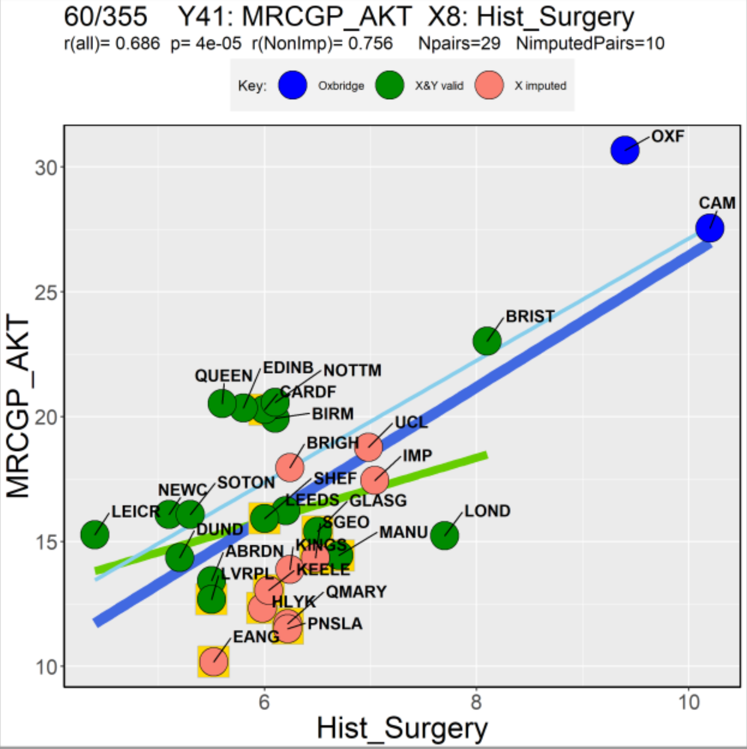
60/355 Y41: MRCGP_AKT X8: Hist_Surgery
r(all)= 0.686 p= 4e-05 r(NonImp)= 0.756 Npairs=29 NimputedPairs=10
Key: Oxbridge X&Y valid X imputed
MRCGP_AKT
Hist_Surgery
OXF
CAM
BRIST
QUEEN
EDINB
NOTTM
CARDF
BIRM
UCL
BRIGH
IMP
NEWC
SOTON
SHEF
LEEDS
GLASG
LEICR
DUND
SGEO
LOND
ABRDN
KINGS
MANU
LVRPL
KEELE
HLYK
QMARY
PNSLA
EANG

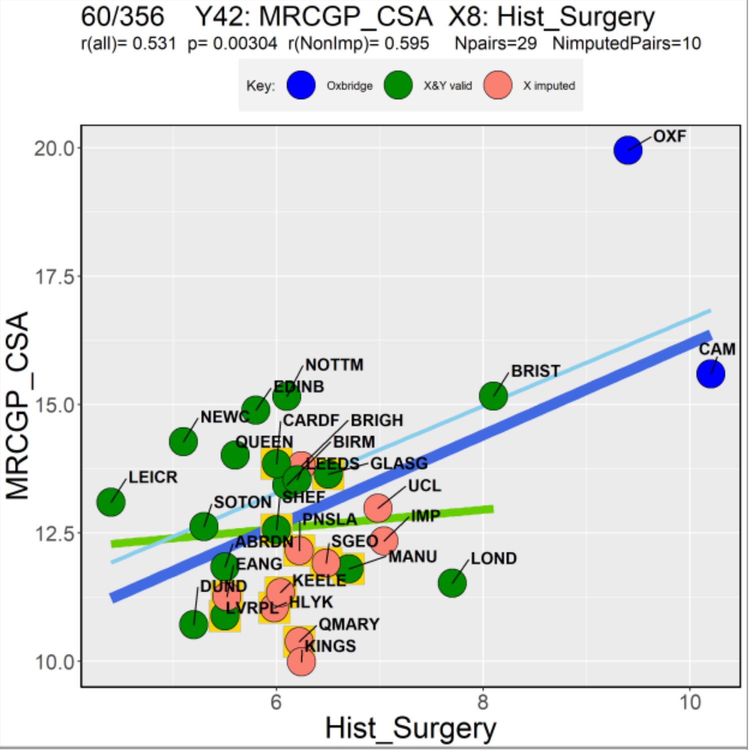
60/356 Y42: MRCGP_CSA X8: Hist_Surgery
r(all)= 0.531 p= 0.00304 r(NonImp)= 0.595 Npairs=29 NimputedPairs=10
Key: Oxbridge X&Y valid X imputed
MRCGP_CSA
Hist_Surgery
OXF
CAM
BRIST
NOTTM
EDINB
NEWC
CARDF
BRIGH
QUEEN
BIRM
LEEDS
GLASG
LEICR
SHEF
UCL
SOTON
IMP
PNSLA
ABRDN
SGEO
MANU
LOND
EANG
KEELE
DUND
LVRPL
HLYK
QMARY
KINGS

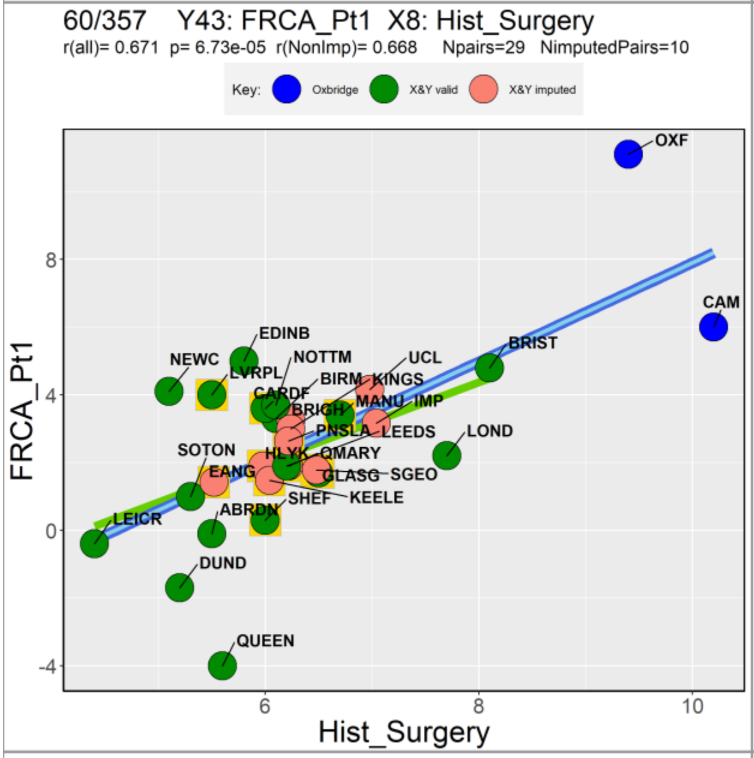
60/357 Y43: FRCA_Pt1 X8: Hist_Surgery
r(all)= 0.671 p= 6.73e-05 r(NonImp)= 0.668 Npairs=29 NimputedPairs=10
Key: Oxbridge X&Y valid X&Y imputed
FRCA_Pt1
Hist_Surgery
OXF
CAM
BRIST
EDINB
NEWC
LVRPL
NOTTM
UCL
BIRM
KINGS
CARDF
MANU
IMP
BRIGH
PNSLA
LEEDS
LOND
SOTON
HLYK
QMARY
EANG
GLASG
SGEO
SHEF
KEELE
LEICR
ABRDN
DUND
QUEEN

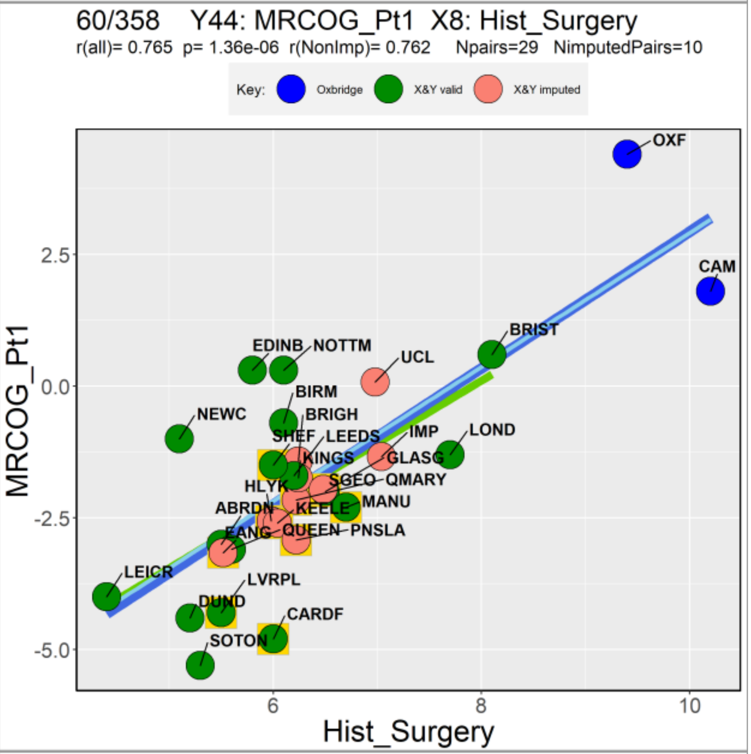
60/358 Y44: MRCOG_Pt1 X8: Hist_Surgery
r(all)= 0.765 p= 1.36e-06 r(NonImp)= 0.762 Npairs=29 NimputedPairs=10
Key: Oxbridge X&Y valid X&Y imputed
MRCOG_Pt1
Hist_Surgery
OXF
CAM
BRIST
EDINB
NOTTM
UCL
BIRM
NEWC
BRIGH
SHEF
LEEDS
IMP
LOND
KINGS
GLASG
HLYK
SGEO
QMARY
ABRDN
KEELE
MANU
EANG
QUEEN
PNSLA
LEICR
LVRPL
DUND
CARDF
SOTON

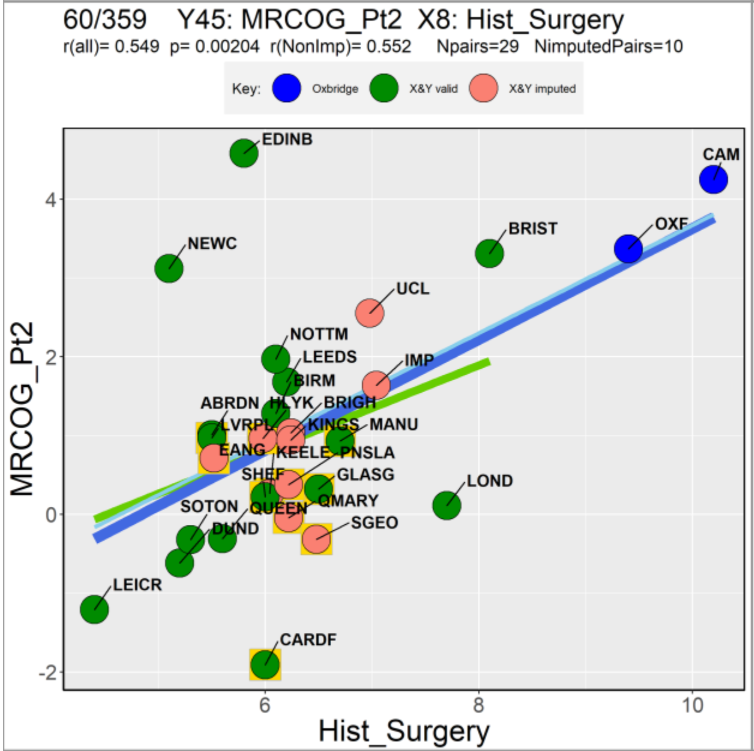
60/359 Y45: MRCOG_Pt2 X8: Hist_Surgery
r(all)= 0.549 p= 0.00204 r(NonImp)= 0.552 Npairs=29 NimputedPairs=10
Key: Oxbridge X&Y valid X&Y imputed
MRCOG_Pt2
Hist_Surgery
EDINB
CAM
NEWC
BRIST
OXF
UCL
NOTTM
LEEDS
IMP
BIRM
ABRDN
HLYK
BRIGH
LVRPL
KINGS
MANU
EANG
KEELE
PNSLA
SHEF
GLASG
LOND
SOTON
QUEEN
QMARY
DUND
SGEO
LEICR
CARDF

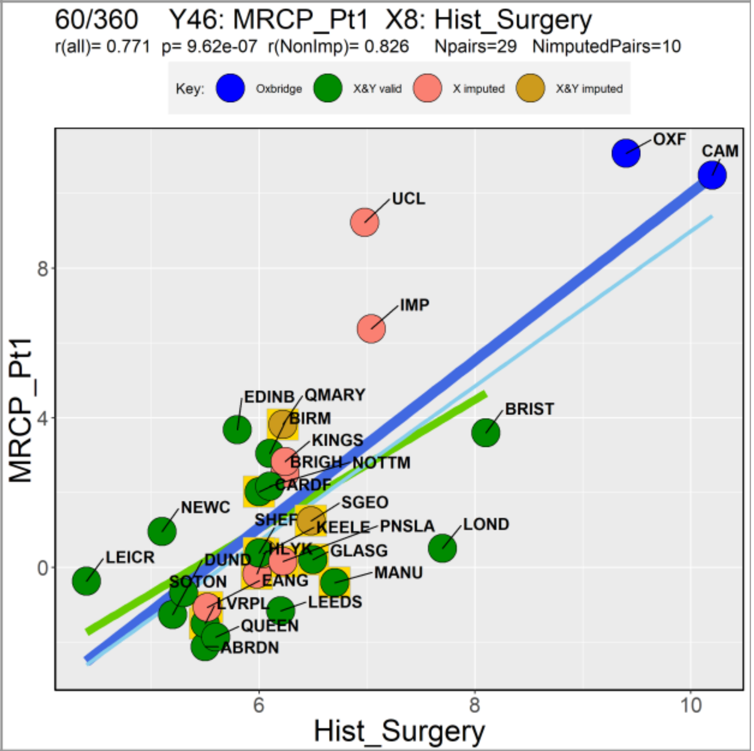
60/360 Y46: MRCP_Pt1 X8: Hist_Surgery
r(all)= 0.771 p= 9.62e-07 r(NonImp)= 0.826 Npairs=29 NimputedPairs=10
Key: Oxbridge X&Y valid X imputed X&Y imputed
MRCP_Pt1
Hist_Surgery
OXF
CAM
UCL
IMP
EDINB
QMARY
BRIST
BIRM
KINGS
BRIGH
NOTTM
CARDF
NEWC
SGEO
SHEF
KEELE
PNSLA
LOND
HLYK
GLASG
LEICR
DUND
SOTON
EANG
MANU
LVRPL
LEEDS
QUEEN
ABRDN

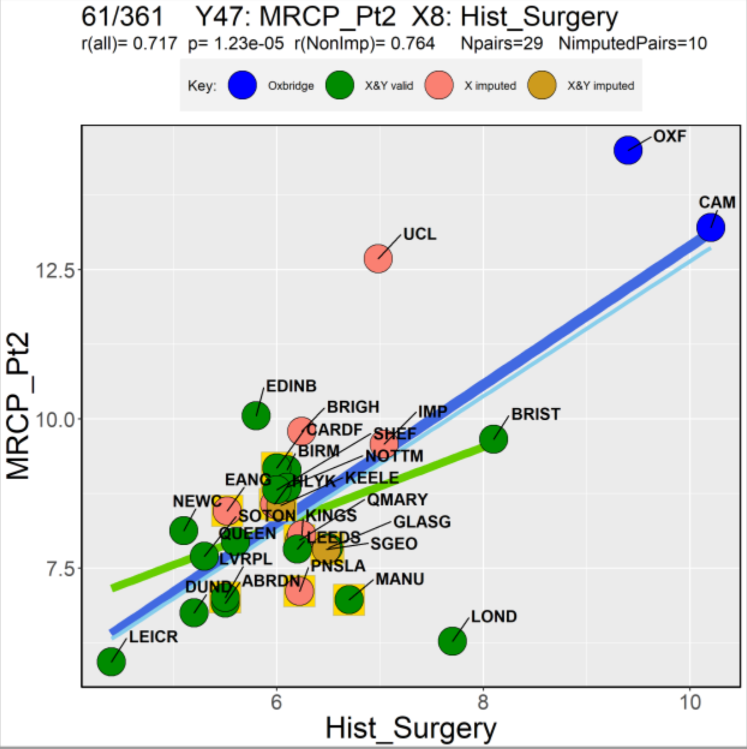
61/361 Y47: MRCP_Pt2 X8: Hist_Surgery
r(all)= 0.717 p= 1.23e-05 r(NonImp)= 0.764 Npairs=29 NimputedPairs=10
Key: Oxbridge X&Y valid X imputed X&Y imputed
MRCP_Pt2
Hist_Surgery

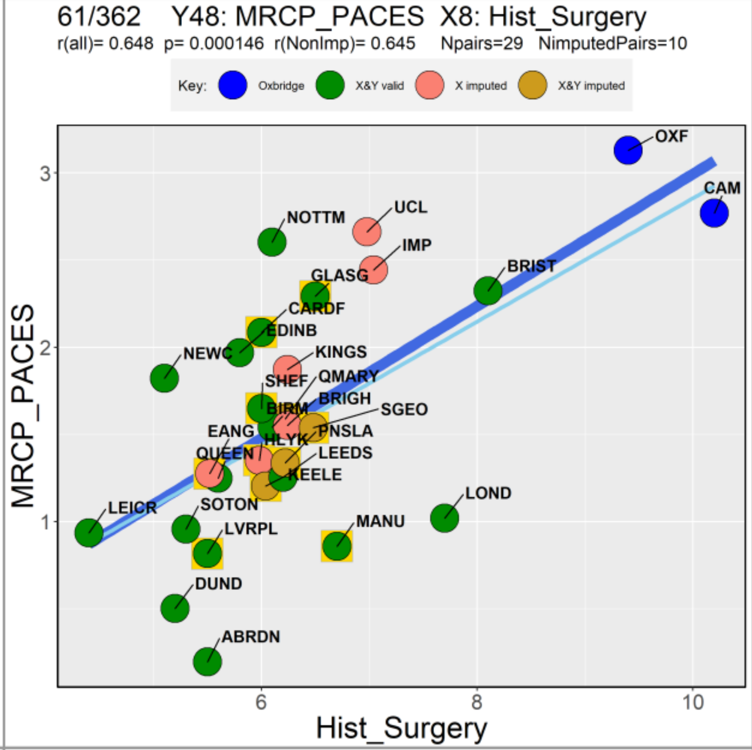
61/362 Y48: MRCP_PACES X8: Hist_Surgery
r(all)= 0.648 p= 0.000146 r(NonImp)= 0.645 Npairs=29 NimputedPairs=10
Key: Oxbridge X&Y valid X imputed X&Y imputed
MRCP_PACES
Hist_Surgery

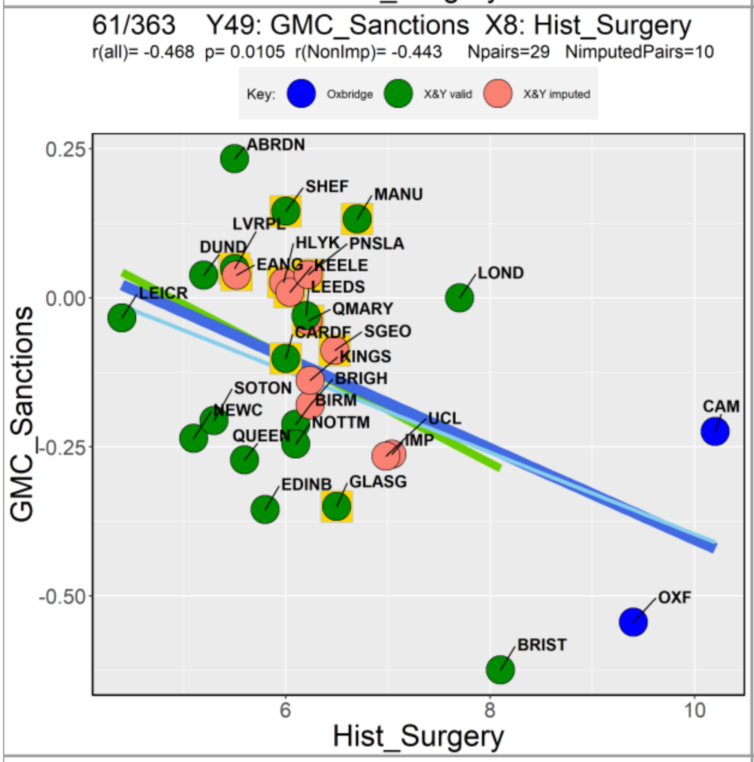
61/363 Y49: GMC_Sanctions X8: Hist_Surgery
r(all)= -0.468 p= 0.0105 r(NonImp)= -0.443 Npairs=29 NimputedPairs=10
Key: Oxbridge X&Y valid X&Y imputed
GMC_Sanctions
Hist_Surgery

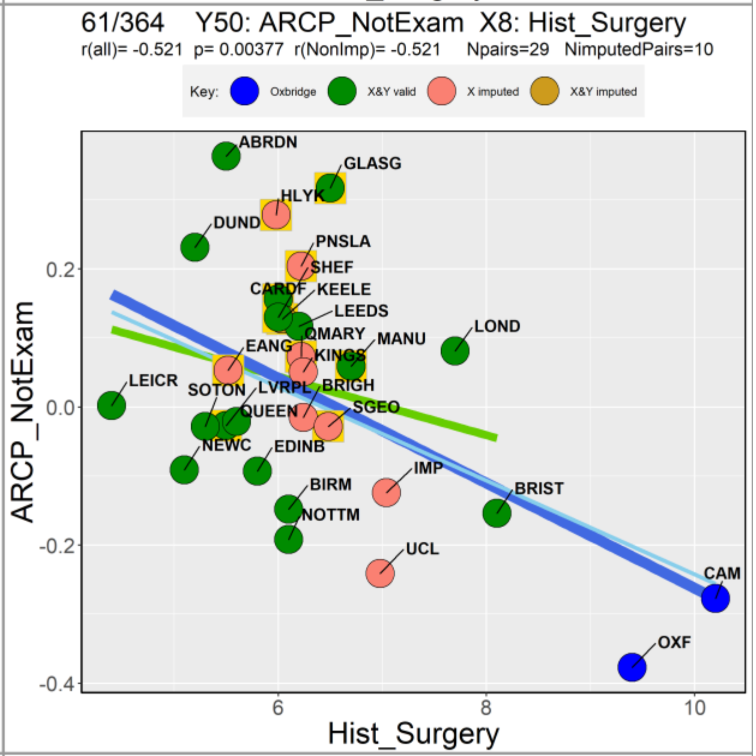
61/364 Y50: ARCP_NotExam X8: Hist_Surgery
r(all)= -0.521 p= 0.00377 r(NonImp)= -0.521 Npairs=29 NimputedPairs=10
Key: Oxbridge X&Y valid X imputed X&Y imputed
ARCP_NotExam
Hist_Surgery

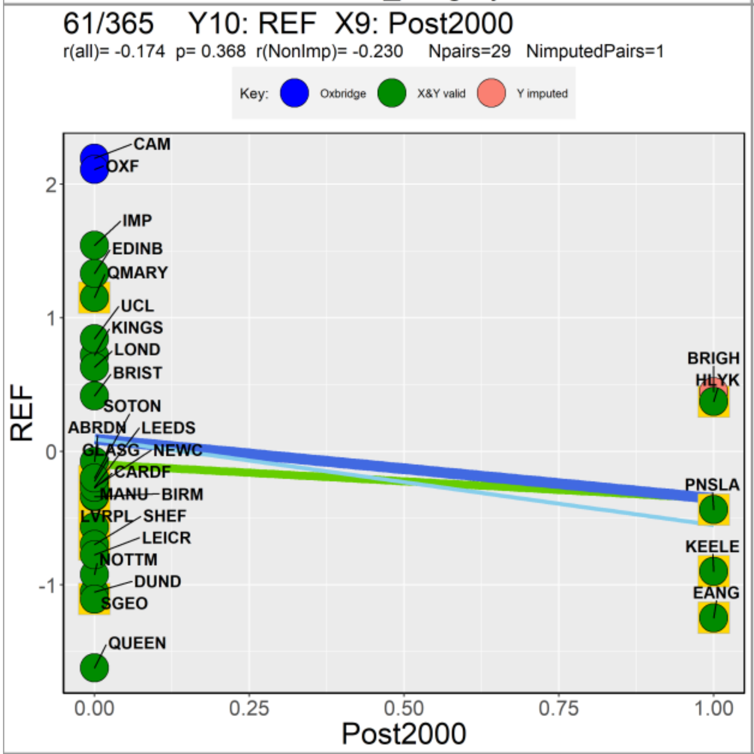
61/365 Y10: REF X9: Post2000
r(all)= -0.174 p= 0.368 r(NonImp)= -0.230 Npairs=29 NimputedPairs=1
Key: Oxbridge X&Y valid Y imputed
REF
Post2000

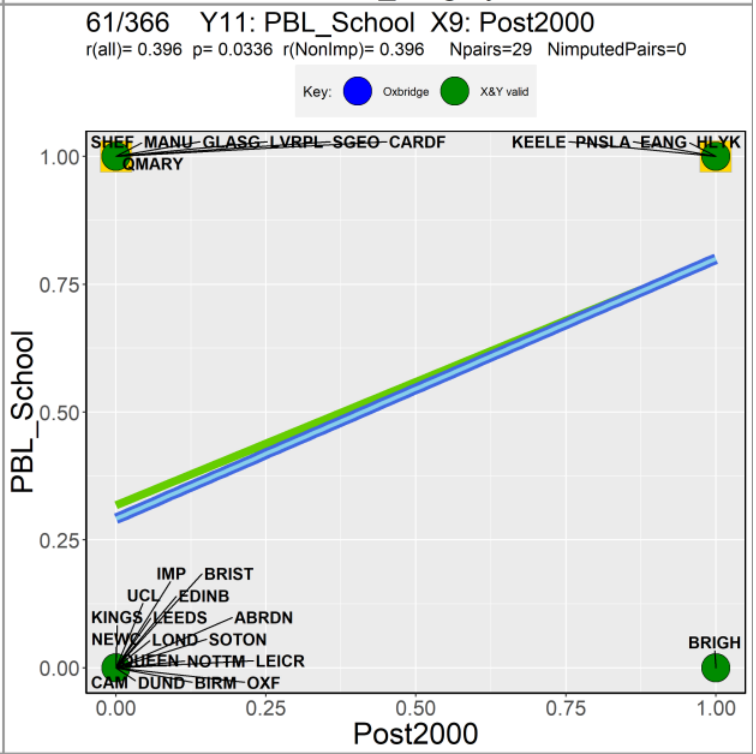
61/366 Y11: PBL_School X9: Post2000
r(all)= 0.396 p= 0.0336 r(NonImp)= 0.396 Npairs=29 NimputedPairs=0
Key: Oxbridge X&Y valid
PBL_School
Post2000

62/367 Y12: Spend_Student X9: Post2000
r(all)= -0.216 p= 0.26 r(NonImp)= -0.216 Npairs=29 NimputedPairs=0

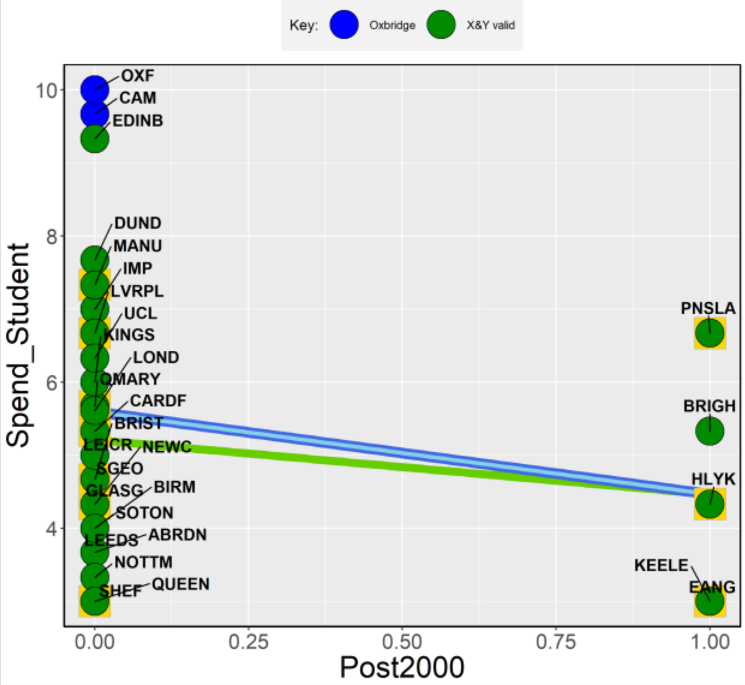

62/368 Y13: Student_Staff X9: Post2000
r(all)= 0.254 p= 0.184 r(NonImp)= 0.254 Npairs=29 NimputedPairs=0

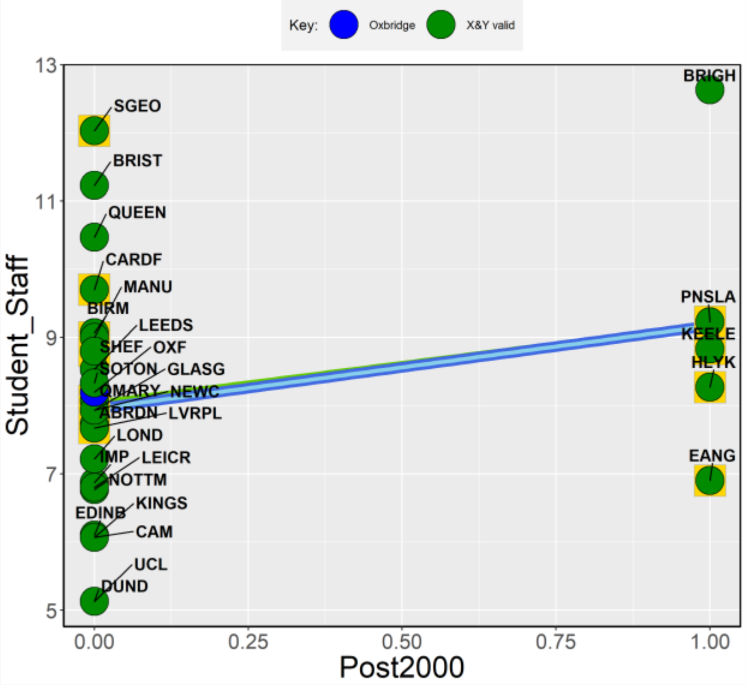

62/369 Y14: Entrants_N X9: Post2000
r(all)= -0.601 p= 0.00056 r(NonImp)= -0.601 Npairs=29 NimputedPairs=0

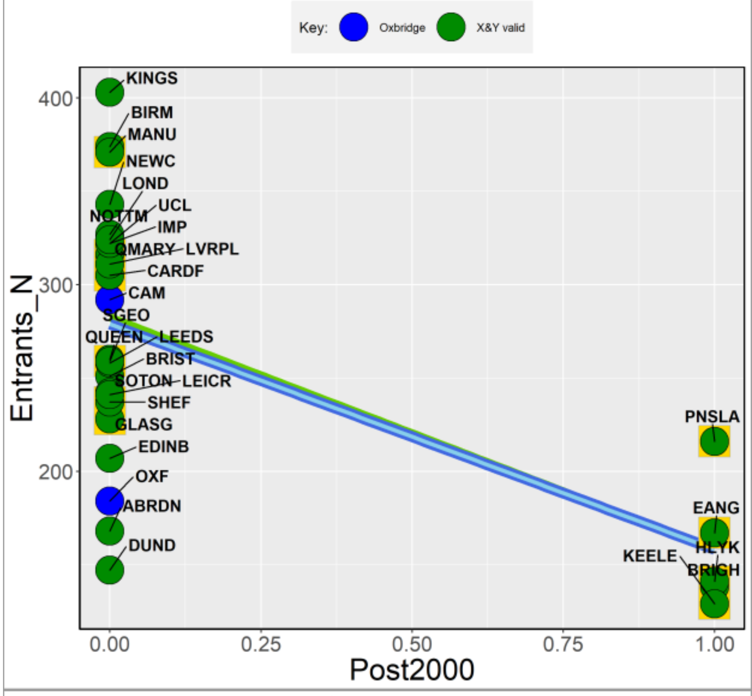

62/370 Y15: Entrants_Female X9: Post2000
r(all)= 0.138 p= 0.477 r(NonImp)= 0.138 Npairs=29 NimputedPairs=0

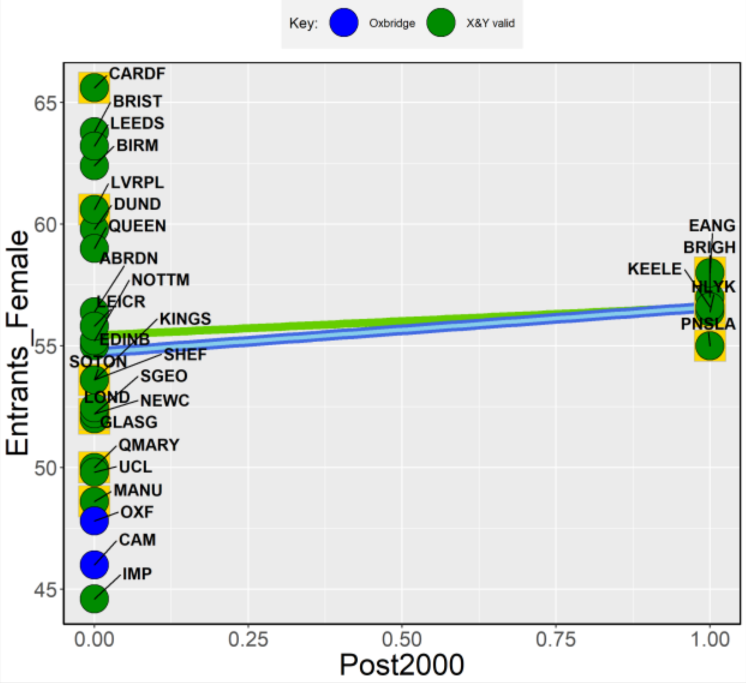

62/371 Y16: EntryGrades X9: Post2000
r(all)= -0.390 p= 0.0365 r(NonImp)= -0.390 Npairs=29 NimputedPairs=0

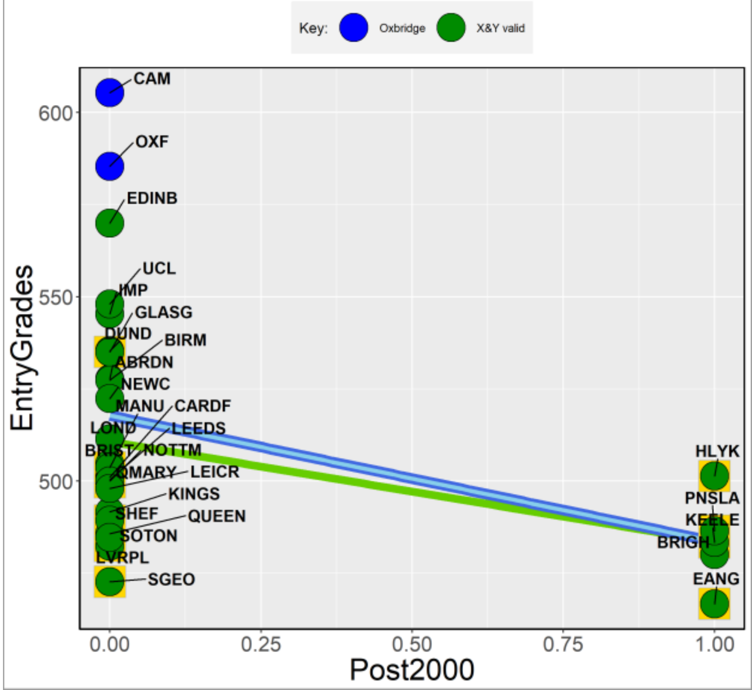

62/372 Y17: Entrants_NonHome X9: Post2000
r(all)= -0.261 p= 0.171 r(NonImp)= -0.261 Npairs=29 NimputedPairs=0

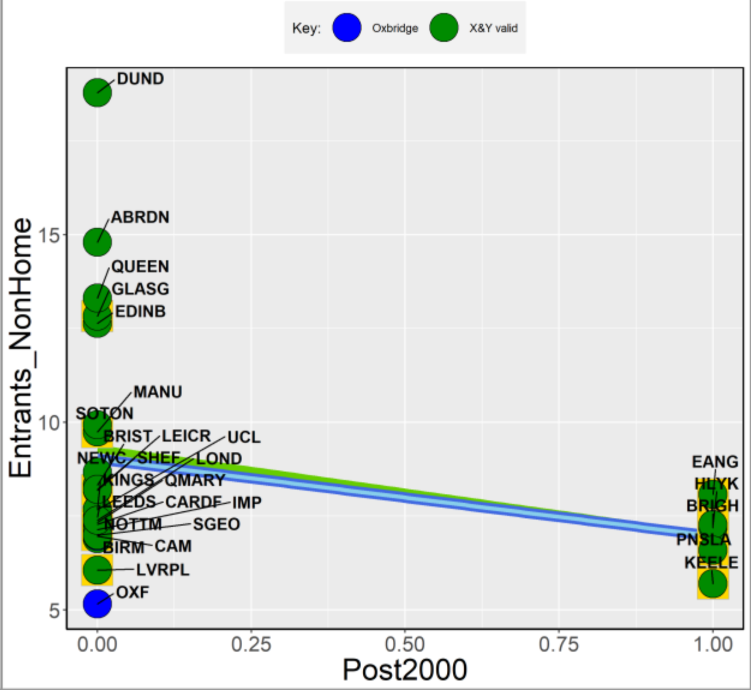

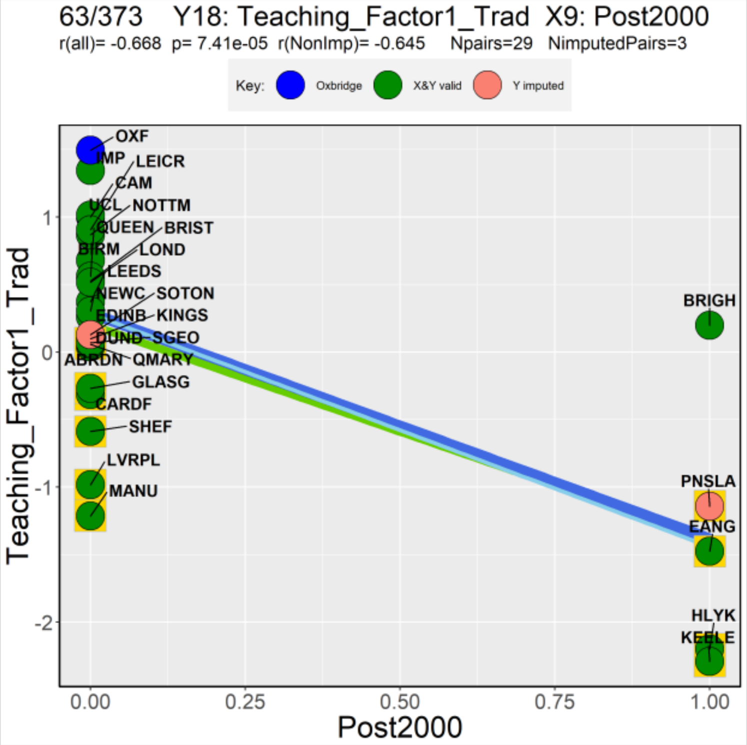
63/373 Y18: Teaching_Factor1_Trad X9: Post2000
r(all)= -0.668 p= 7.41e-05 r(NonImp)= -0.645 Npairs=29 NimputedPairs=3
Key: Oxbridge X&Y valid Y imputed
Teaching_Factor1_Trad
Post2000
0.00
0.25
0.50
0.75
1.00
1
0
-1
-2
OXF
IMP
LEICR
CAM
UCL
NOTTM
QUEEN
BRIST
BIRM
LOND
LEEDS
NEWC
SOTON
EDINB
KINGS
DUND
SGEO
ABRDN
QMARY
GLASG
CARDF
SHEF
LVRPL
MANU
BRIGH
PNSLA
EANG
HLYK
KEELE

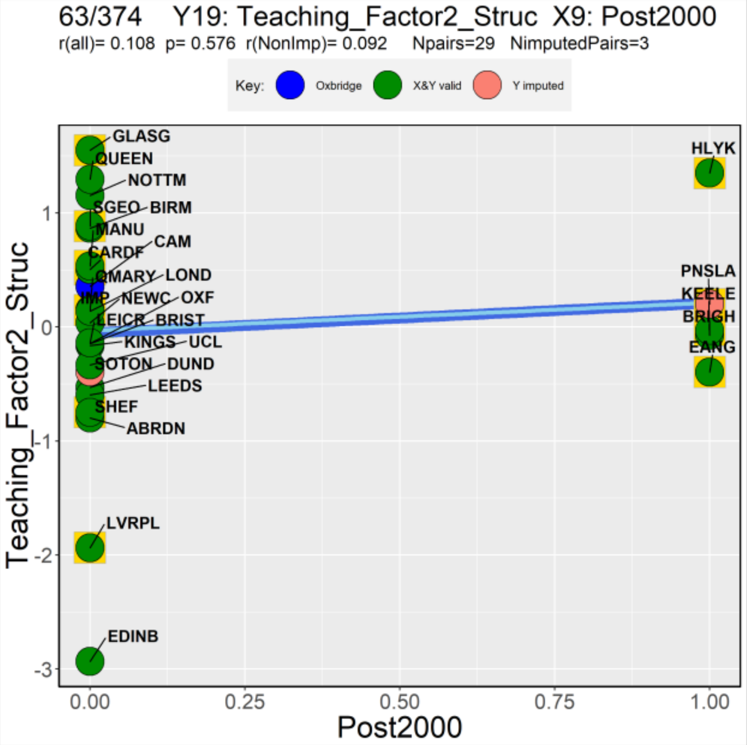
63/374 Y19: Teaching_Factor2_Struc X9: Post2000
r(all)= 0.108 p= 0.576 r(NonImp)= 0.092 Npairs=29 NimputedPairs=3
Key: Oxbridge X&Y valid Y imputed
Teaching_Factor2_Struc
Post2000
0.00
0.25
0.50
0.75
1.00
1
0
-1
-2
-3
GLASG
QUEEN
NOTTM
SGEO
BIRM
MANU
CAM
CARDF
QMARY
LOND
IMP
NEWC
OXF
LEICR
BRIST
KINGS
UCL
SOTON
DUND
LEEDS
SHEF
ABRDN
LVRPL
EDINB
HLYK
PNSLA
KEELE
BRIGH
EANG

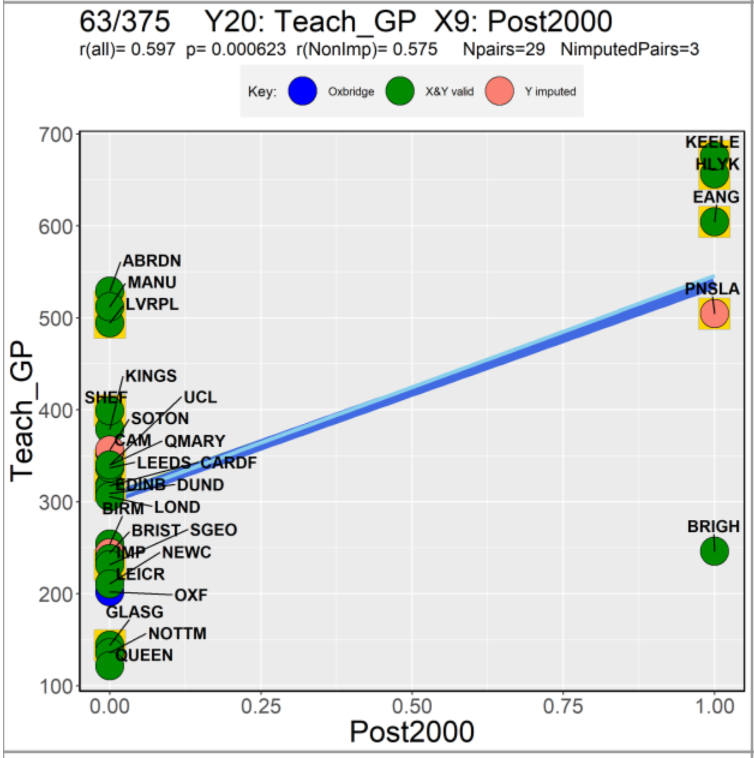
63/375 Y20: Teach_GP X9: Post2000
r(all)= 0.597 p= 0.000623 r(NonImp)= 0.575 Npairs=29 NimputedPairs=3
Key: Oxbridge X&Y valid Y imputed
Teach_GP
Post2000
0.00
0.25
0.50
0.75
1.00
700
600
500
400
300
200
100
ABRDN
MANU
LVRPL
KINGS
SHEF
UCL
SOTON
CAM
QMARY
LEEDS
CARDF
EDINB
DUND
BIRM
LOND
BRIST
SGEO
IMP
NEWC
LEICR
OXF
GLASG
NOTTM
QUEEN
KEELE
HLYK
EANG
PNSLA
BRIGH

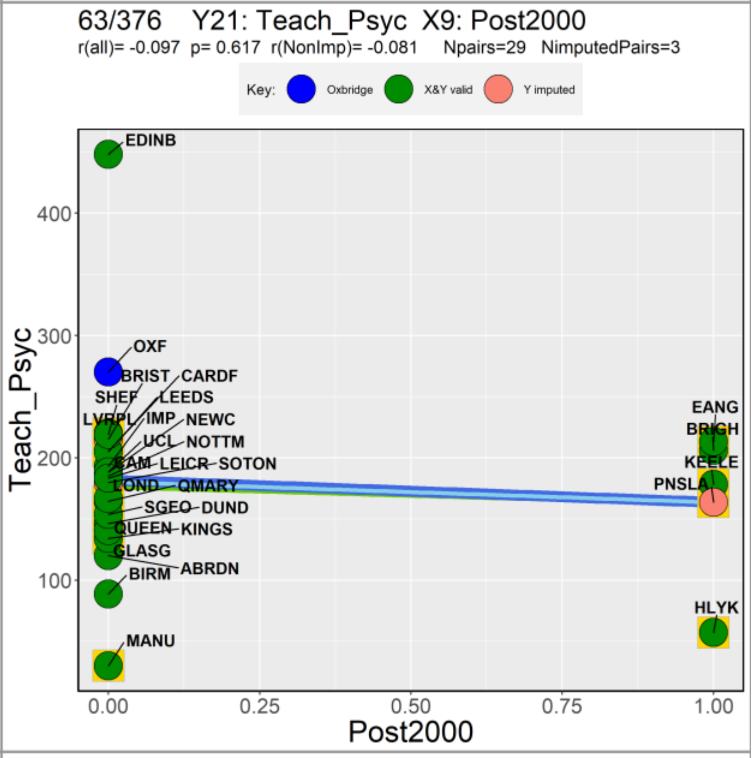
63/376 Y21: Teach_Psyc X9: Post2000
r(all)= -0.097 p= 0.617 r(NonImp)= -0.081 Npairs=29 NimputedPairs=3
Key: Oxbridge X&Y valid Y imputed
Teach_Psyc
Post2000
0.00
0.25
0.50
0.75
1.00
400
300
200
100
EDINB
OXF
BRIST
CARDF
SHEF
LEEDS
LVRPL
IMP
NEWC
UCL
NOTTM
CAM
LEICR
SOTON
LOND
QMARY
SGEO
DUND
QUEEN
KINGS
GLASG
ABRDN
BIRM
MANU
EANG
BRIGH
KEELE
PNSLA
HLYK

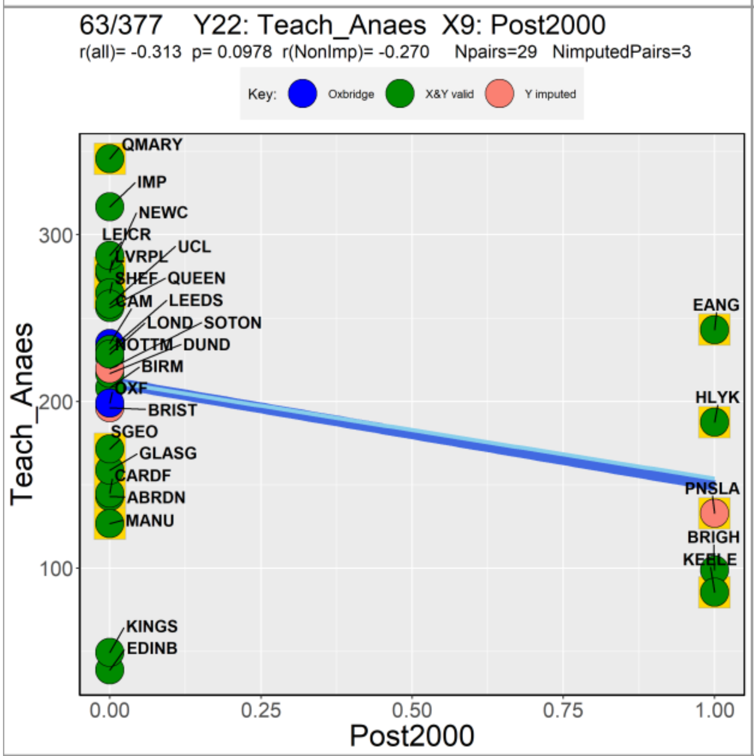
63/377 Y22: Teach_Anaes X9: Post2000
r(all)= -0.313 p= 0.0978 r(NonImp)= -0.270 Npairs=29 NimputedPairs=3
Key: Oxbridge X&Y valid Y imputed
Teach_Anaes
Post2000
0.00
0.25
0.50
0.75
1.00
300
200
100
QMARY
IMP
NEWC
LEICR
LVRPL
UCL
SHEF
QUEEN
CAM
LEEDS
LOND
SOTON
NOTTM
DUND
BIRM
OXF
BRIST
SGEO
GLASG
CARDF
ABRDN
MANU
KINGS
EDINB
EANG
HLYK
PNSLA
BRIGH
KEELE

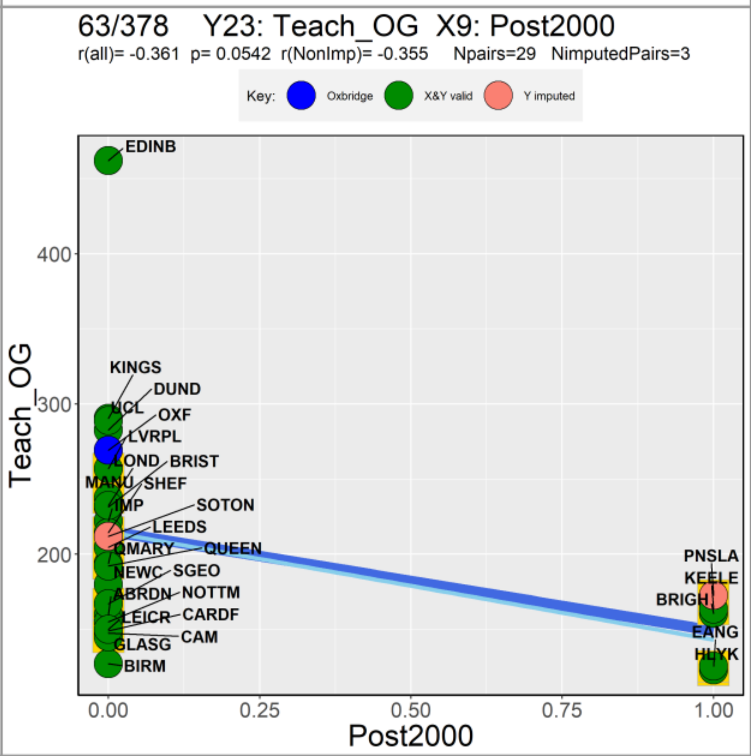
63/378 Y23: Teach_OG X9: Post2000
r(all)= -0.361 p= 0.0542 r(NonImp)= -0.355 Npairs=29 NimputedPairs=3
Key: Oxbridge X&Y valid Y imputed
Teach_OG
Post2000
0.00
0.25
0.50
0.75
1.00
400
300
200
EDINB
KINGS
DUND
UCL
OXF
LVRPL
LOND
BRIST
MANU
SHEF
IMP
SOTON
LEEDS
QMARY
QUEEN
NEWC
SGEO
ABRDN
NOTTM
LEICR
CARDF
GLASG
CAM
BIRM
PNSLA
KEELE
BRIGH
EANG
HLYK

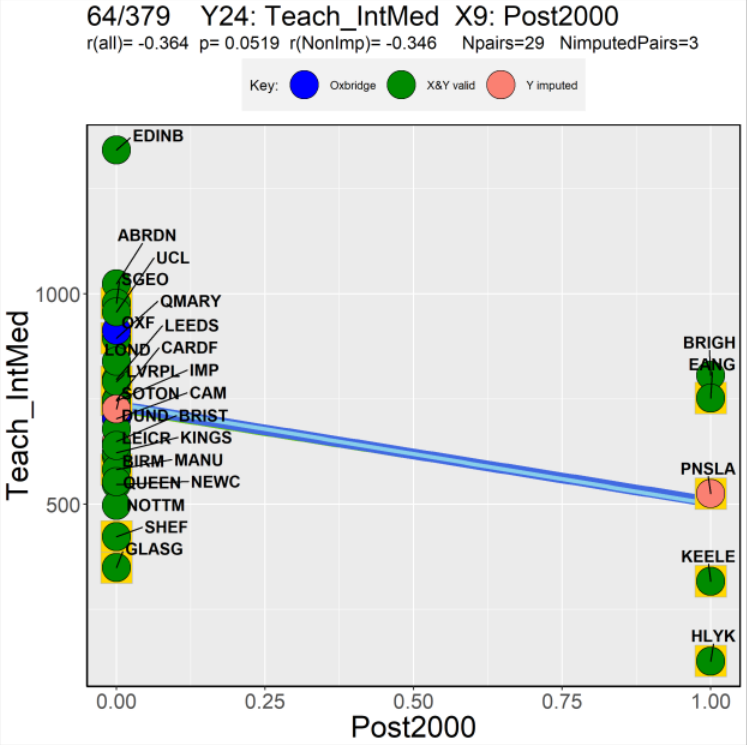
64/379 Y24: Teach_IntMed X9: Post2000
r(all)= -0.364 p= 0.0519 r(NonImp)= -0.346 Npairs=29 NimputedPairs=3
Key: Oxbridge X&Y valid Y imputed
Teach_IntMed
1000
500
0.00 0.25 0.50 0.75 1.00
Post2000
EDINB
ABRDN
UCL
SGEO
QMARY
OXF
LEEDS
LOND
CARDF
LVRPL
IMP
SOTON
CAM
DUND
BRIST
LEICR
KINGS
BIRM
MANU
QUEEN
NEWC
NOTTM
SHEF
GLASG
BRIGH
EANG
PNSLA
KEELE
HLYK

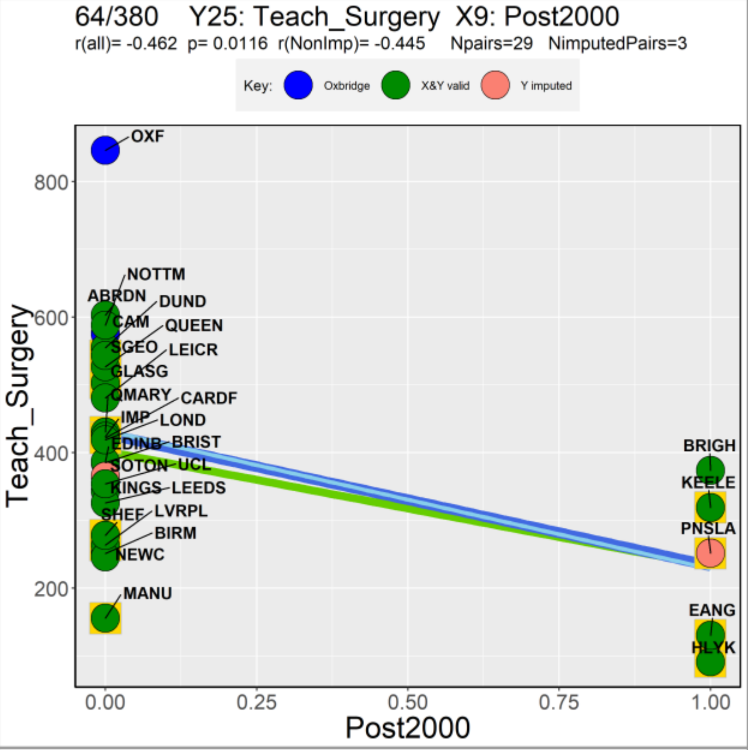
64/380 Y25: Teach_Surgery X9: Post2000
r(all)= -0.462 p= 0.0116 r(NonImp)= -0.445 Npairs=29 NimputedPairs=3
Key: Oxbridge X&Y valid Y imputed
Teach_Surgery
800
600
400
200
0.00 0.25 0.50 0.75 1.00
Post2000
OXF
NOTTM
ABRDN
DUND
CAM
QUEEN
SGEO
LEICR
GLASG
QMARY
CARDF
IMP
LOND
EDINB
BRIST
SOTON
UCL
KINGS
LEEDS
SHEF
LVRPL
BIRM
NEWC
MANU
BRIGH
KEELE
PNSLA
EANG
HLYK

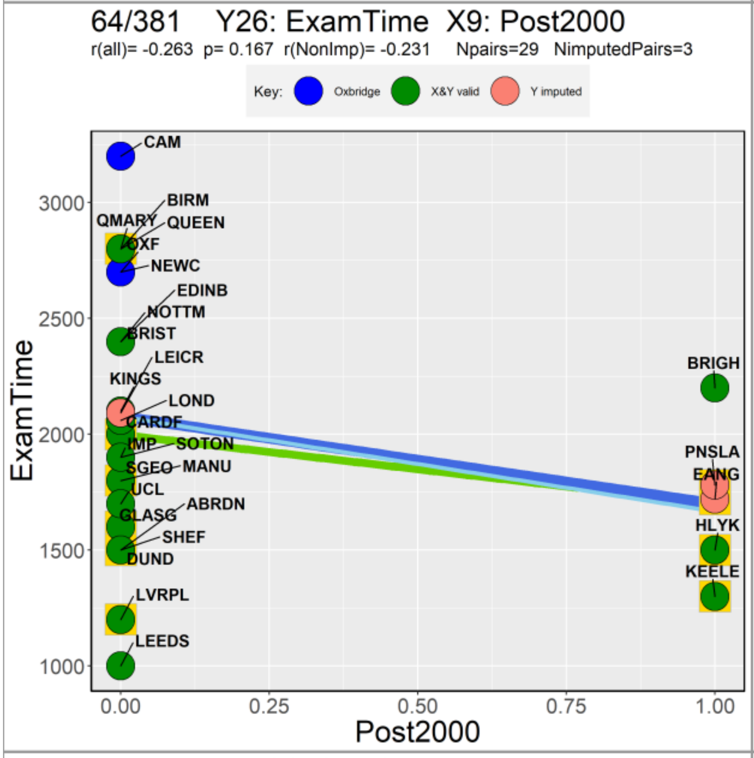
64/381 Y26: ExamTime X9: Post2000
r(all)= -0.263 p= 0.167 r(NonImp)= -0.231 Npairs=29 NimputedPairs=3
Key: Oxbridge X&Y valid Y imputed
ExamTime
3000
2500
2000
1500
1000
0.00 0.25 0.50 0.75 1.00
Post2000
CAM
BIRM
QMARY
QUEEN
OXF
NEWC
EDINB
NOTTM
BRIST
LEICR
KINGS
LOND
CARDF
IMP
SOTON
SGEO
MANU
UCL
GLASG
ABRDN
SHEF
DUND
LVRPL
LEEDS
BRIGH
PNSLA
EANG
HLYK
KEELE

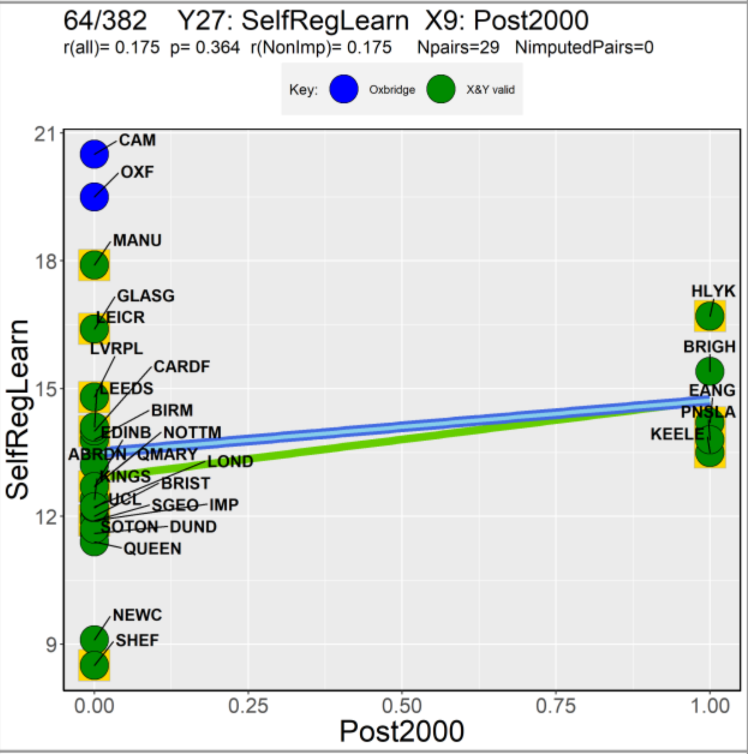
64/382 Y27: SelfRegLearn X9: Post2000
r(all)= 0.175 p= 0.364 r(NonImp)= 0.175 Npairs=29 NimputedPairs=0
Key: Oxbridge X&Y valid
SelfRegLearn
21
18
15
12
9
0.00 0.25 0.50 0.75 1.00
Post2000
CAM
OXF
MANU
GLASG
LEICR
LVRPL
CARDF
LEEDS
BIRM
EDINB
NOTTM
ABRDN
QMARY
LOND
KINGS
BRIST
UCL
SGEO
IMP
SOTON
DUND
QUEEN
NEWC
SHEF
HLYK
BRIGH
EANG
PNSLA
KEELE

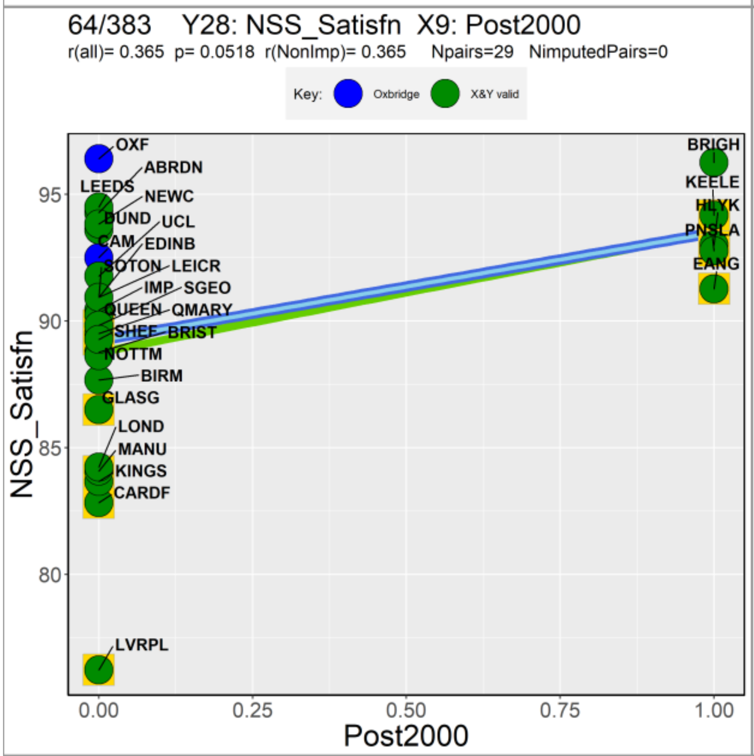
64/383 Y28: NSS_Satisfn X9: Post2000
r(all)= 0.365 p= 0.0518 r(NonImp)= 0.365 Npairs=29 NimputedPairs=0
Key: Oxbridge X&Y valid
NSS_Satisfn
95
90
85
80
0.00 0.25 0.50 0.75 1.00
Post2000
OXF
ABRDN
LEEDS
NEWC
DUND
UCL
CAM
EDINB
SOTON
LEICR
IMP
SGEO
QUEEN
QMARY
SHEF
BRIST
NOTTM
BIRM
GLASG
LOND
MANU
KINGS
CARDF
LVRPL
BRIGH
KEELE
HLYK
PNSLA
EANG

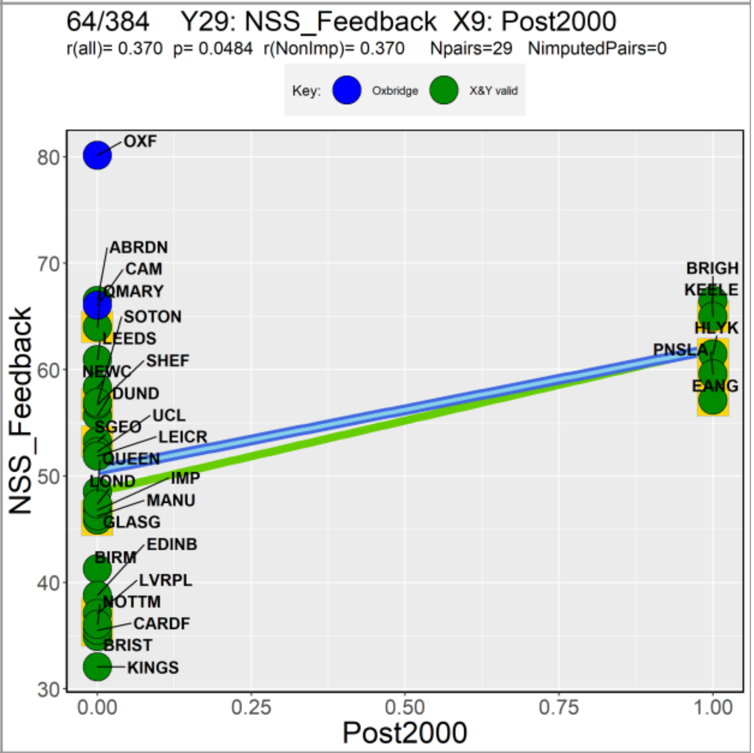
64/384 Y29: NSS_Feedback X9: Post2000
r(all)= 0.370 p= 0.0484 r(NonImp)= 0.370 Npairs=29 NimputedPairs=0
Key: Oxbridge X&Y valid
NSS_Feedback
80
70
60
50
40
30
0.00 0.25 0.50 0.75 1.00
Post2000
OXF
ABRDN
CAM
QMARY
SOTON
LEEDS
NEWC
SHEF
DUND
UCL
SGEO
LEICR
QUEEN
LOND
IMP
MANU
GLASG
EDINB
BIRM
LVRPL
NOTTM
CARDF
BRIST
KINGS
BRIGH
KEELE
HLYK
PNSLA
EANG

65/385 Y30: UKFPO_EPM X9: Post2000

r(all)= -0.435 p= 0.0184 r(NonImp)= -0.435 Npairs=29 NimputedPairs=0

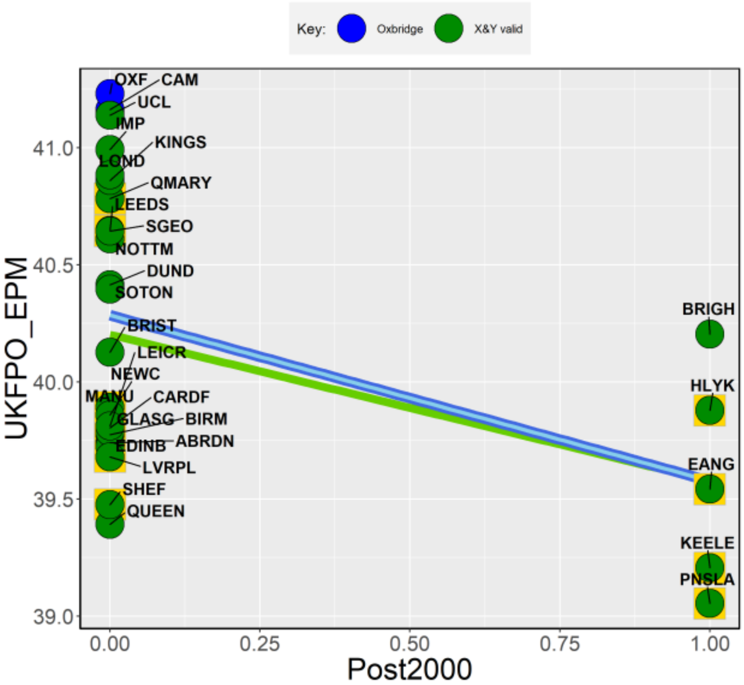

65/386 Y31: UKFPO_SJT X9: Post2000

r(all)= -0.334 p= 0.0766 r(NonImp)= -0.334 Npairs=29 NimputedPairs=0

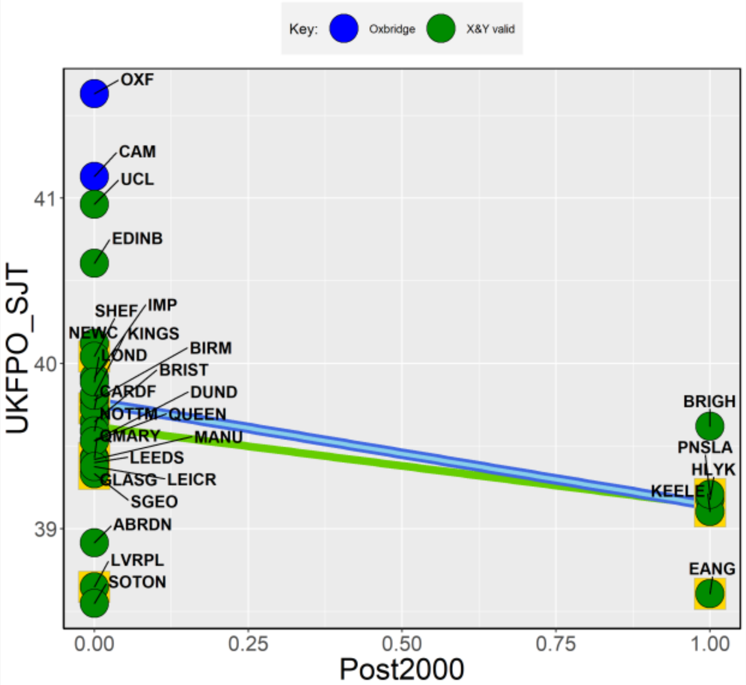

65/387 Y32: F1_Preparedness X9: Post2000

r(all)= 0.461 p= 0.0119 r(NonImp)= 0.461 Npairs=29 NimputedPairs=0

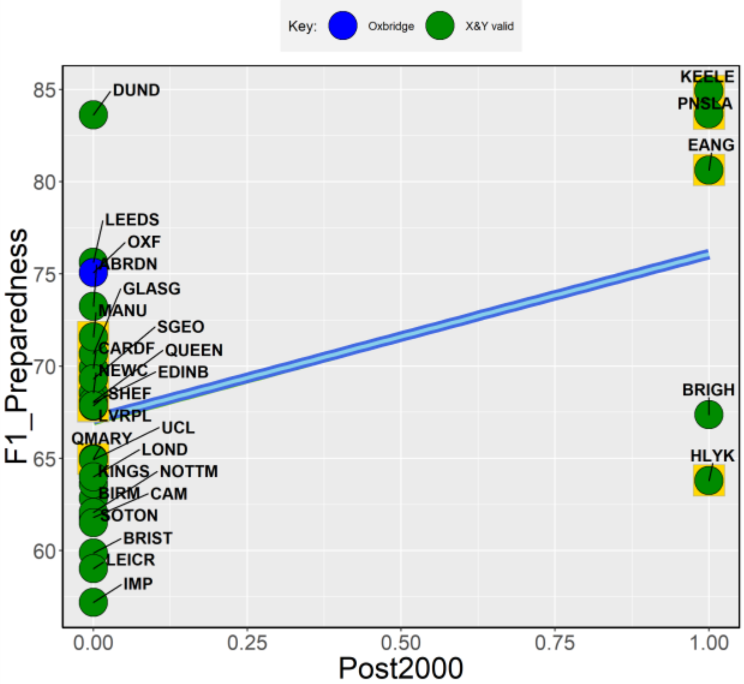

65/388 Y33: F1_Satisfn X9: Post2000

r(all)= 0.222 p= 0.246 r(NonImp)= 0.222 Npairs=29 NimputedPairs=0

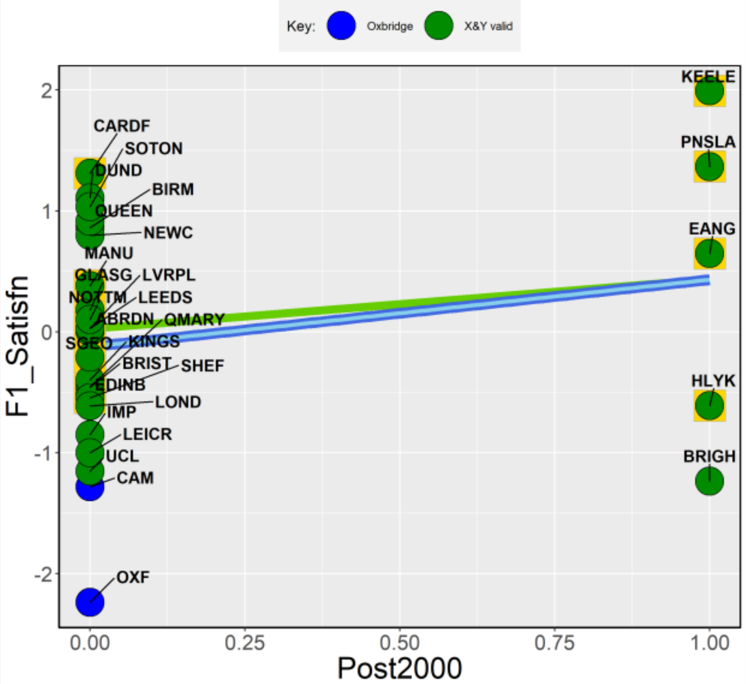

65/389 Y34: F1_Workload X9: Post2000

r(all)= 0.197 p= 0.306 r(NonImp)= 0.197 Npairs=29 NimputedPairs=0

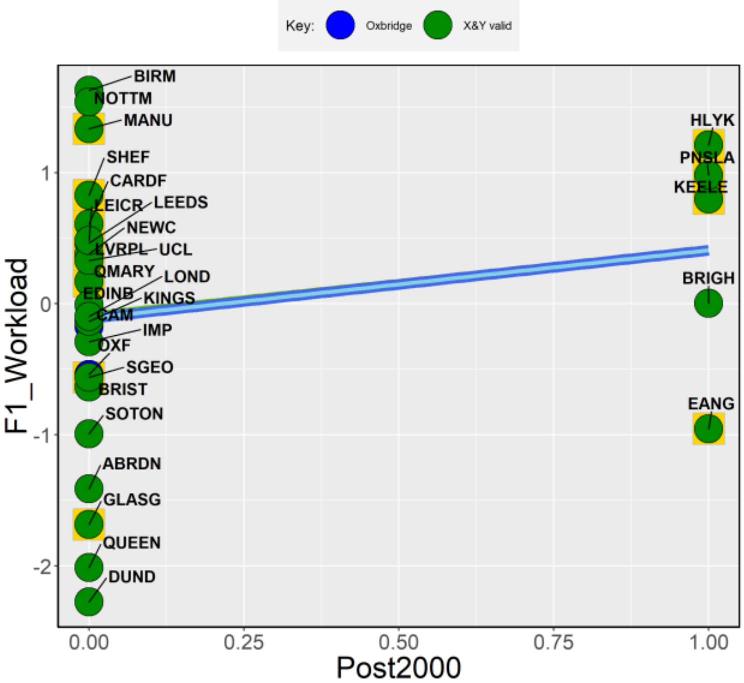

65/390 Y35: F1_Supervn X9: Post2000

r(all)= 0.103 p= 0.596 r(NonImp)= 0.103 Npairs=29 NimputedPairs=0

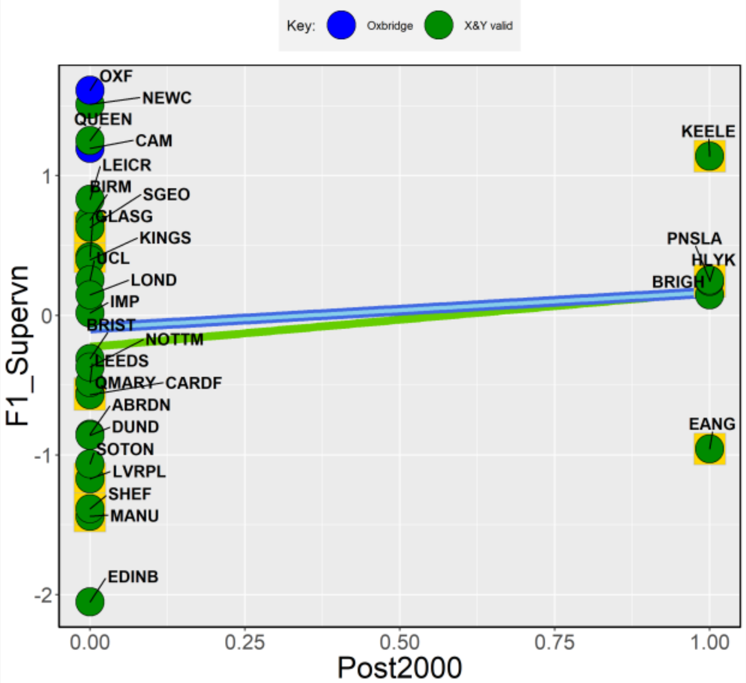

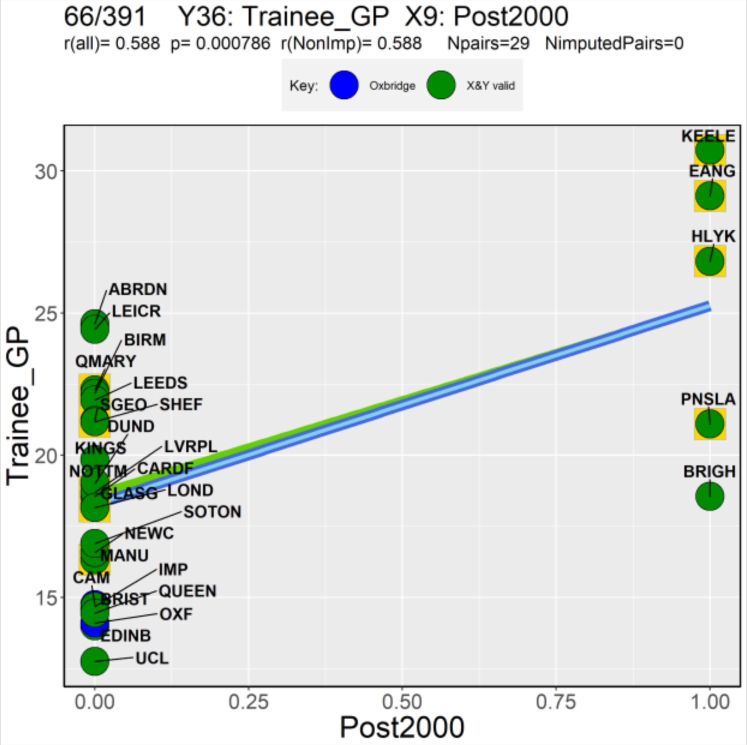
66/391 Y36: Trainee_GP X9: Post2000
r(all)= 0.588 p= 0.000786 r(NonImp)= 0.588 Npairs=29 NimputedPairs=0
Key: Oxbridge X&Y valid
Trainee_GP
Post2000
KEELE
EANG
HLYK
PNSLA
BRIGH
ABRDN
LEICR
BIRM
QMARY
LEEDS
SGEO
SHEF
DUND
KINGS
LVRPL
NOTTM
CARDF
GLASG
LOND
SOTON
NEWC
MANU
CAM
IMP
BRIST
QUEEN
OXF
EDINB
UCL

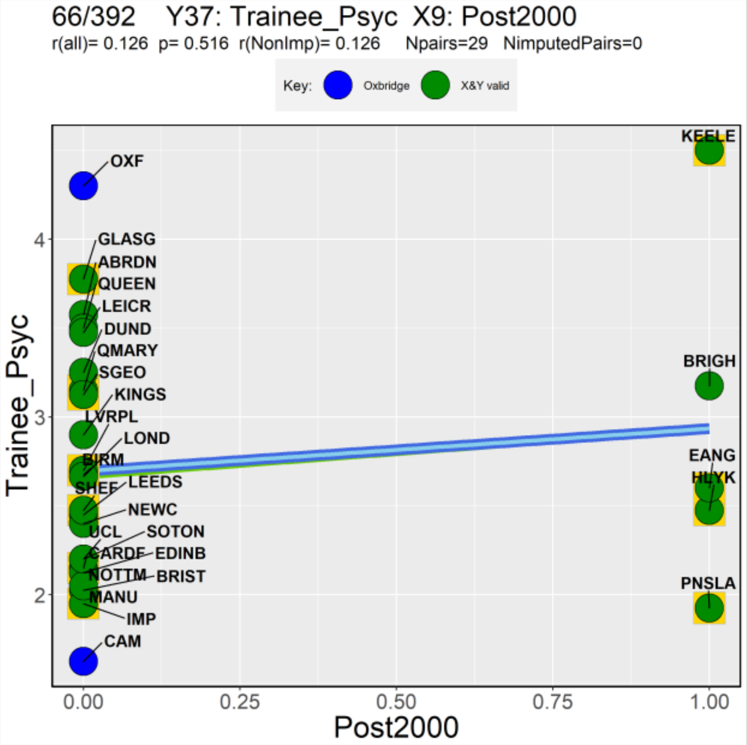
66/392 Y37: Trainee_Psyc X9: Post2000
r(all)= 0.126 p= 0.516 r(NonImp)= 0.126 Npairs=29 NimputedPairs=0
Key: Oxbridge X&Y valid
Trainee_Psyc
Post2000
KEELE
OXF
GLASG
ABRDN
QUEEN
LEICR
DUND
QMARY
SGEO
KINGS
LVRPL
LOND
BIRM
SHEF
LEEDS
NEWC
UCL
SOTON
CARDF
EDINB
NOTTM
BRIST
MANU
IMP
CAM
BRIGH
EANG
HLYK
PNSLA

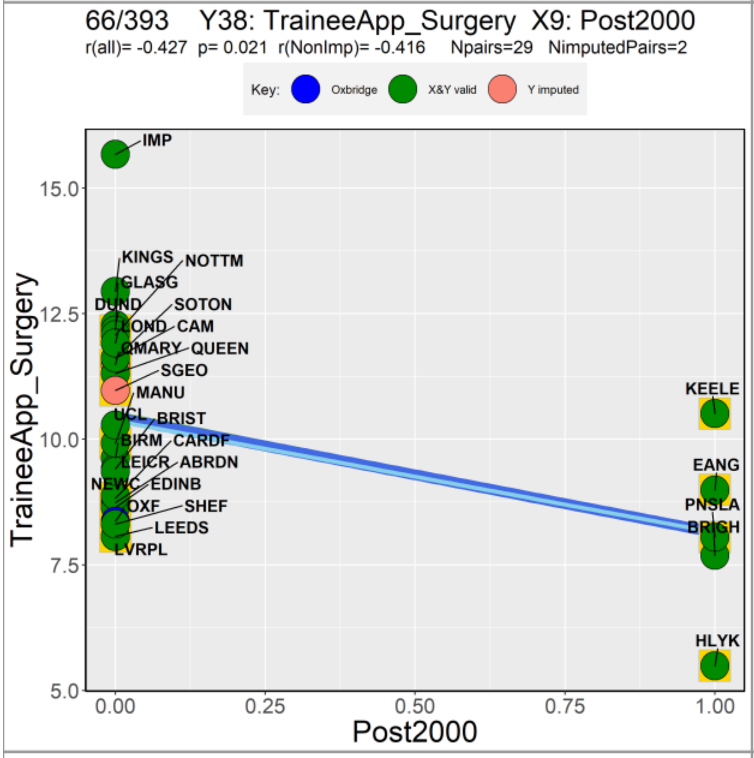
66/393 Y38: TraineeApp_Surgery X9: Post2000
r(all)= -0.427 p= 0.021 r(NonImp)= -0.416 Npairs=29 NimputedPairs=2
Key: Oxbridge X&Y valid Y imputed
TraineeApp_Surgery
Post2000
IMP
KINGS
NOTTM
GLASG
DUND
SOTON
LOND
CAM
QMARY
QUEEN
SGEO
MANU
UCL
BRIST
BIRM
CARDF
LEICR
ABRDN
NEWC
EDINB
OXF
SHEF
LEEDS
LVRPL
KEELE
EANG
PNSLA
BRIGH
HLYK

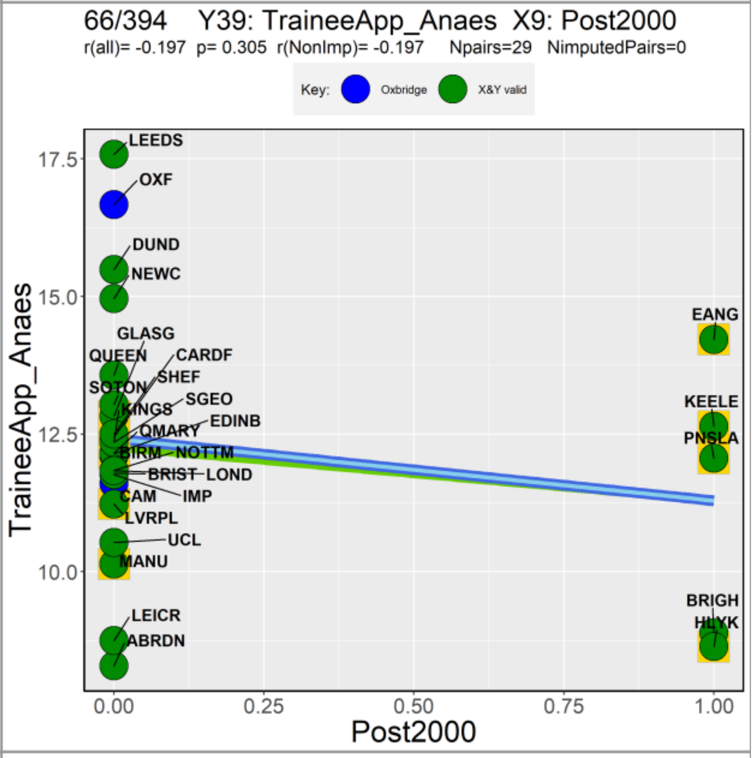
66/394 Y39: TraineeApp_Anaes X9: Post2000
r(all)= -0.197 p= 0.305 r(NonImp)= -0.197 Npairs=29 NimputedPairs=0
Key: Oxbridge X&Y valid
TraineeApp_Anaes
Post2000
LEEDS
OXF
DUND
NEWC
GLASG
QUEEN
CARDF
SHEF
SOTON
KINGS
SGEO
QMARY
EDINB
BIRM
NOTTM
BRIST
LOND
CAM
IMP
LVRPL
UCL
MANU
LEICR
ABRDN
EANG
KEELE
PNSLA
BRIGH
HLYK

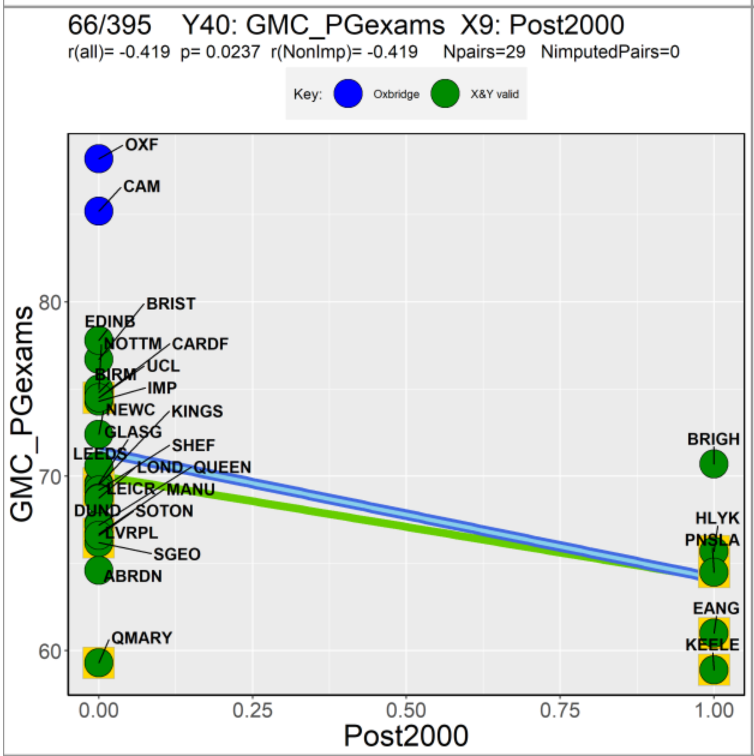
66/395 Y40: GMC_PGexams X9: Post2000
r(all)= -0.419 p= 0.0237 r(NonImp)= -0.419 Npairs=29 NimputedPairs=0
Key: Oxbridge X&Y valid
GMC_PGexams
Post2000
OXF
CAM
BRIST
EDINB
NOTTM
CARDF
BIRM
UCL
IMP
NEWC
KINGS
GLASG
LEEDS
SHEF
LOND
QUEEN
LEICR
MANU
DUND
SOTON
LVRPL
SGEO
ABRDN
QMARY
BRIGH
HLYK
PNSLA
EANG
KEELE

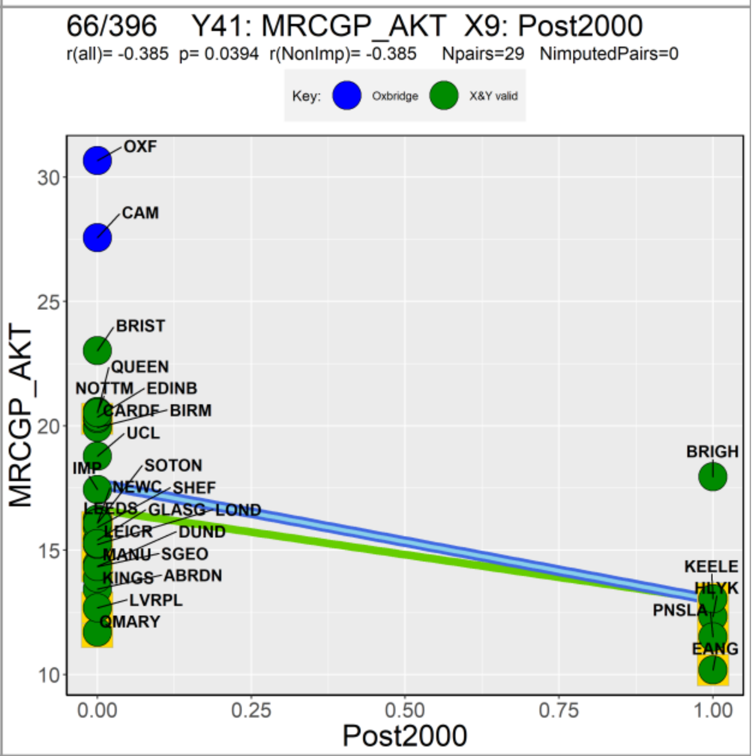
66/396 Y41: MRCGP_AKT X9: Post2000
r(all)= -0.385 p= 0.0394 r(NonImp)= -0.385 Npairs=29 NimputedPairs=0
Key: Oxbridge X&Y valid
MRCGP_AKT
Post2000
OXF
CAM
BRIST
QUEEN
NOTTM
EDINB
CARDF
BIRM
UCL
IMP
SOTON
NEWC
SHEF
LEEDS
GLASG
LOND
LEICR
DUND
MANU
SGEO
KINGS
ABRDN
LVRPL
QMARY
BRIGH
KEELE
HLYK
PNSLA
EANG

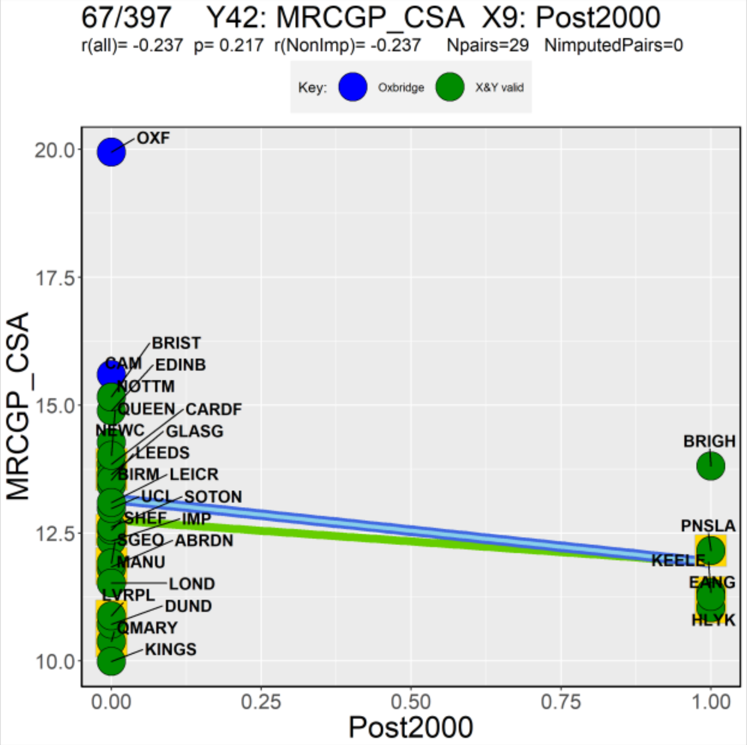
67/397 Y42: MRCGP_CSA X9: Post2000
r(all)= -0.237 p= 0.217 r(NonImp)= -0.237 Npairs=29 NimputedPairs=0
Key: Oxbridge X&Y valid
OXF
BRIST
CAM
EDINB
NOTTM
QUEEN
CARDF
NEWC
GLASG
LEEDS
BIRM
LEICR
UCL
SOTON
SHEF
IMP
SGEO
ABRDN
MANU
LOND
LVRPL
DUND
QMARY
KINGS
BRIGH
PNSLA
KEELE
EANG
HLYK
MRCGP_CSA
20.0
17.5
15.0
12.5
10.0
0.00
0.25
0.50
0.75
1.00
Post2000

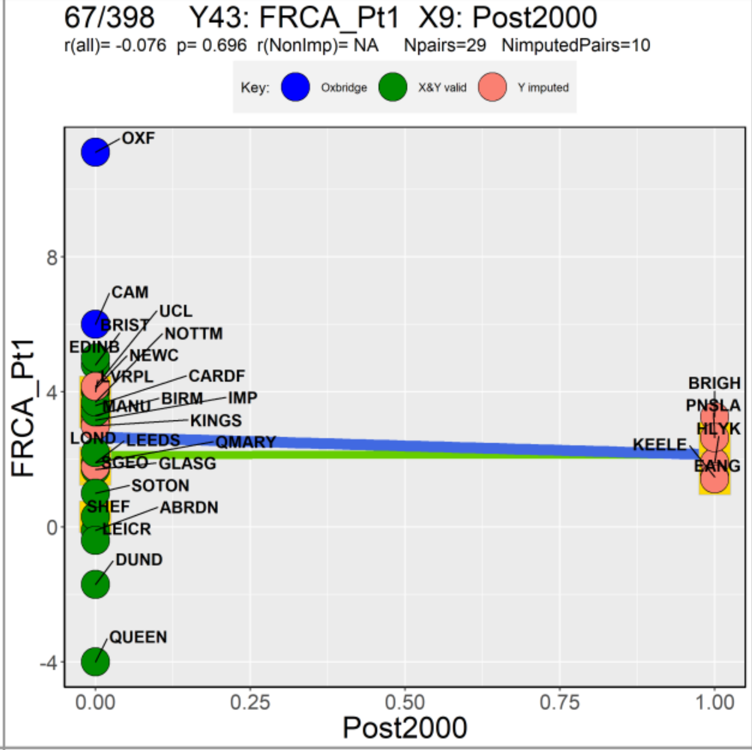
67/398 Y43: FRCA_Pt1 X9: Post2000
r(all)= -0.076 p= 0.696 r(NonImp)= NA Npairs=29 NimputedPairs=10
Key: Oxbridge X&Y valid Y imputed
OXF
CAM
UCL
BRIST
NOTTM
EDINB
NEWC
LVRPL
CARDF
MANU
BIRM
IMP
KINGS
LOND
LEEDS
QMARY
SGEO
GLASG
SOTON
SHEF
ABRDN
LEICR
DUND
QUEEN
BRIGH
PNSLA
HLYK
KEELE
EANG
FRCA_Pt1
8
4
0
-4
0.00
0.25
0.50
0.75
1.00
Post2000

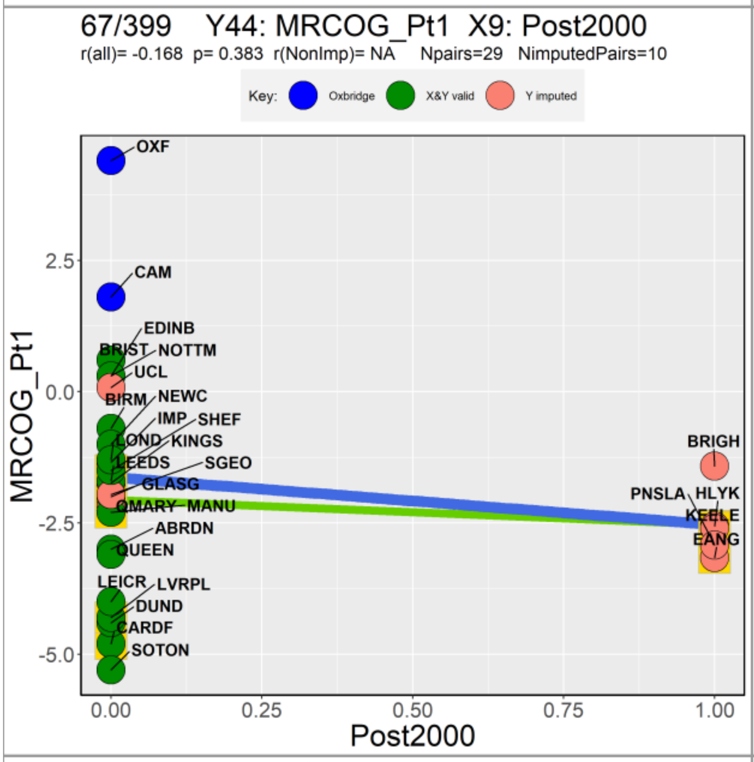
67/399 Y44: MRCOG_Pt1 X9: Post2000
r(all)= -0.168 p= 0.383 r(NonImp)= NA Npairs=29 NimputedPairs=10
Key: Oxbridge X&Y valid Y imputed
OXF
CAM
EDINB
BRIST
NOTTM
UCL
BIRM
NEWC
IMP
SHEF
LOND
KINGS
LEEDS
SGEO
GLASG
QMARY
MANU
ABRDN
QUEEN
LEICR
LVRPL
DUND
CARDF
SOTON
BRIGH
PNSLA
HLYK
KEELE
EANG
MRCOG_Pt1
2.5
0.0
-2.5
-5.0
0.00
0.25
0.50
0.75
1.00
Post2000

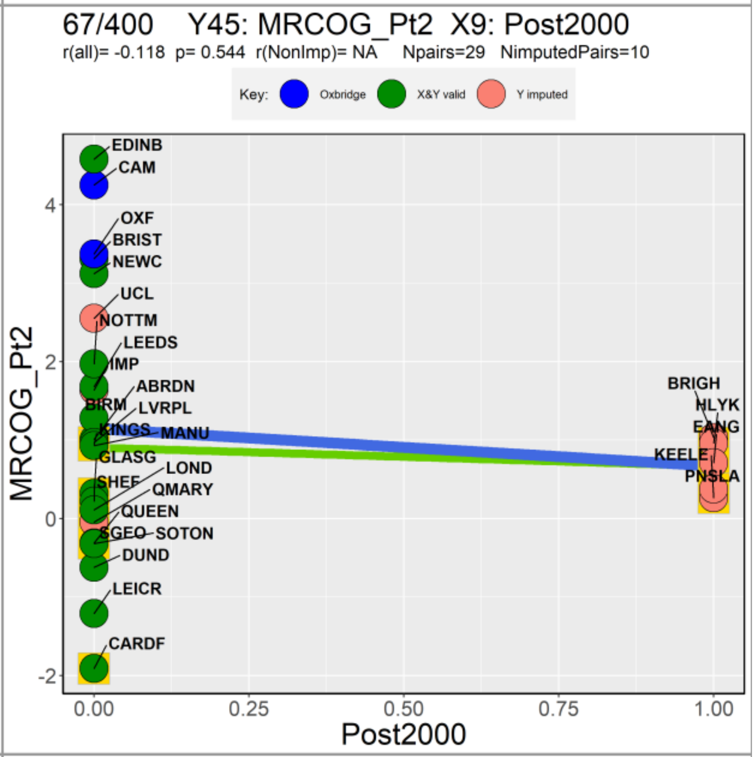
67/400 Y45: MRCOG_Pt2 X9: Post2000
r(all)= -0.118 p= 0.544 r(NonImp)= NA Npairs=29 NimputedPairs=10
Key: Oxbridge X&Y valid Y imputed
EDINB
CAM
OXF
BRIST
NEWC
UCL
NOTTM
LEEDS
IMP
ABRDN
BIRM
LVRPL
KINGS
MANU
GLASG
LOND
SHEF
QMARY
QUEEN
SGEO
SOTON
DUND
LEICR
CARDF
BRIGH
HLYK
EANG
KEELE
PNSLA
MRCOG_Pt2
4
2
0
-2
0.00
0.25
0.50
0.75
1.00
Post2000

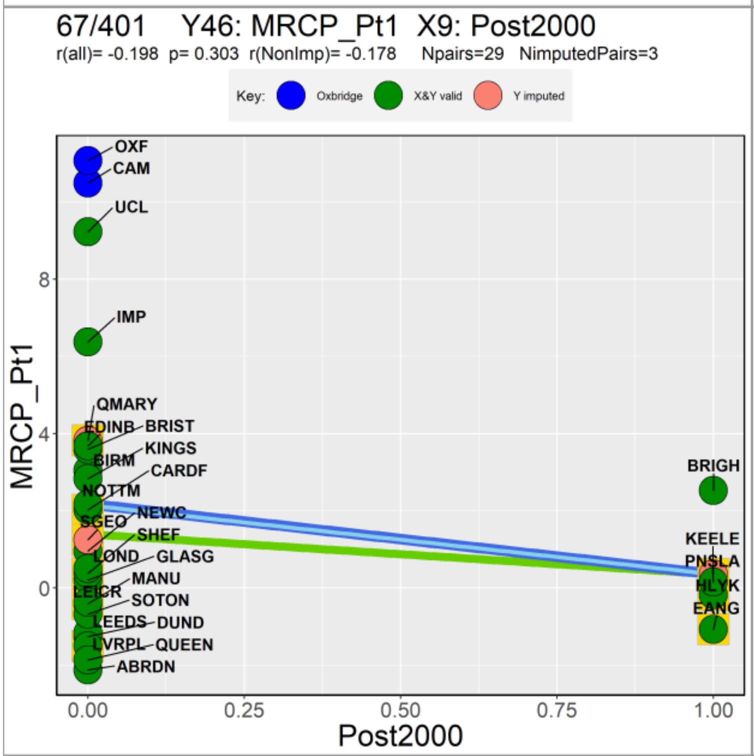
67/401 Y46: MRCP_Pt1 X9: Post2000
r(all)= -0.198 p= 0.303 r(NonImp)= -0.178 Npairs=29 NimputedPairs=3
Key: Oxbridge X&Y valid Y imputed
OXF
CAM
UCL
IMP
QMARY
EDINB
BRIST
KINGS
BIRM
CARDF
NOTTM
NEWC
SGEO
SHEF
LOND
GLASG
MANU
LEICR
SOTON
LEEDS
DUND
LVRPL
QUEEN
ABRDN
BRIGH
KEELE
PNSLA
HLYK
EANG
MRCP_Pt1
8
4
0
0.00
0.25
0.50
0.75
1.00
Post2000

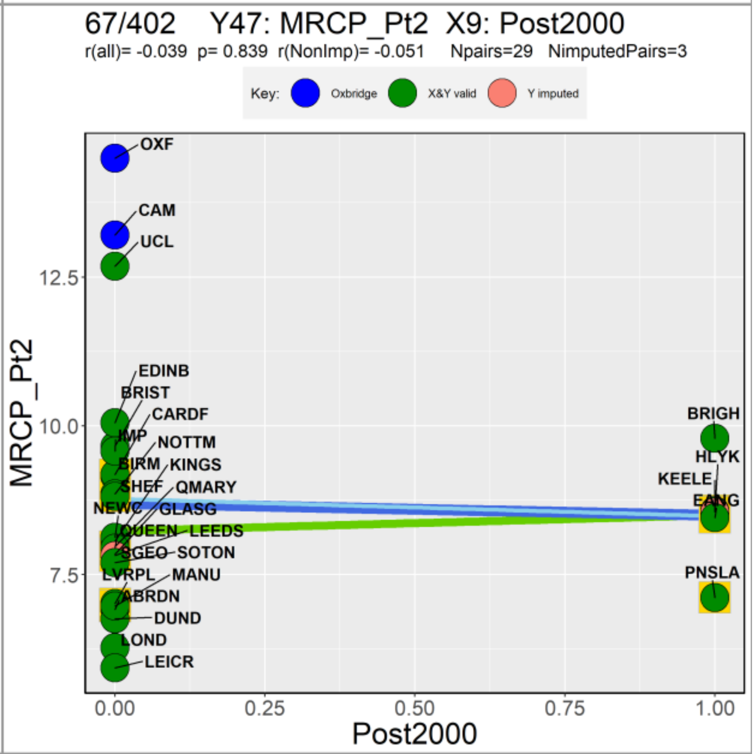
67/402 Y47: MRCP_Pt2 X9: Post2000
r(all)= -0.039 p= 0.839 r(NonImp)= -0.051 Npairs=29 NimputedPairs=3
Key: Oxbridge X&Y valid Y imputed
OXF
CAM
UCL
EDINB
BRIST
CARDF
IMP
NOTTM
BIRM
KINGS
SHEF
QMARY
NEWC
GLASG
QUEEN
LEEDS
SGEO
SOTON
LVRPL
MANU
ABRDN
DUND
LOND
LEICR
BRIGH
HLYK
KEELE
EANG
PNSLA
MRCP_Pt2
12.5
10.0
7.5
0.00
0.25
0.50
0.75
1.00
Post2000

68/403 Y48: MRCP_PACES X9: Post2000

r(all)= -0.176 p= 0.361 r(NonImp)= -0.125 Npairs=29 NimputedPairs=4

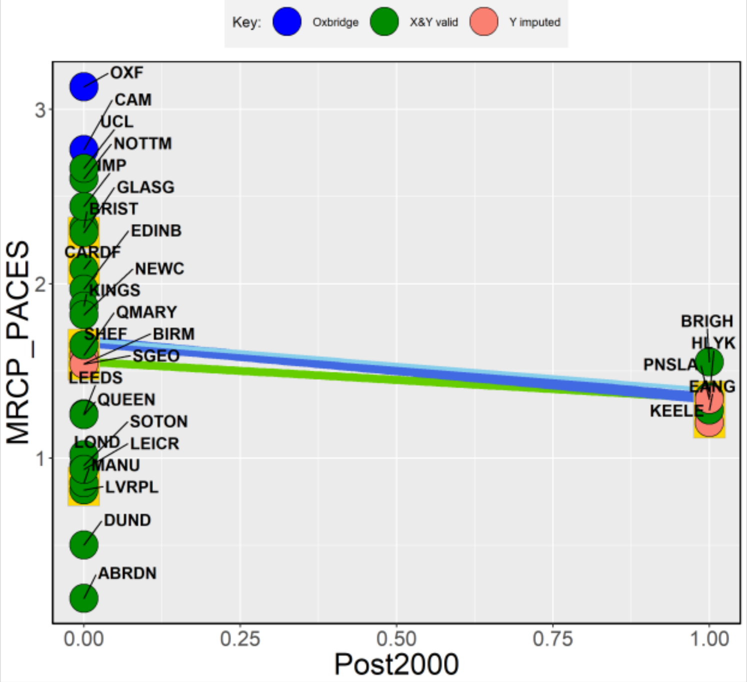

68/404 Y49: GMC_Sanctions X9: Post2000

r(all)= 0.267 p= 0.162 r(NonImp)= NA Npairs=29 NimputedPairs=10

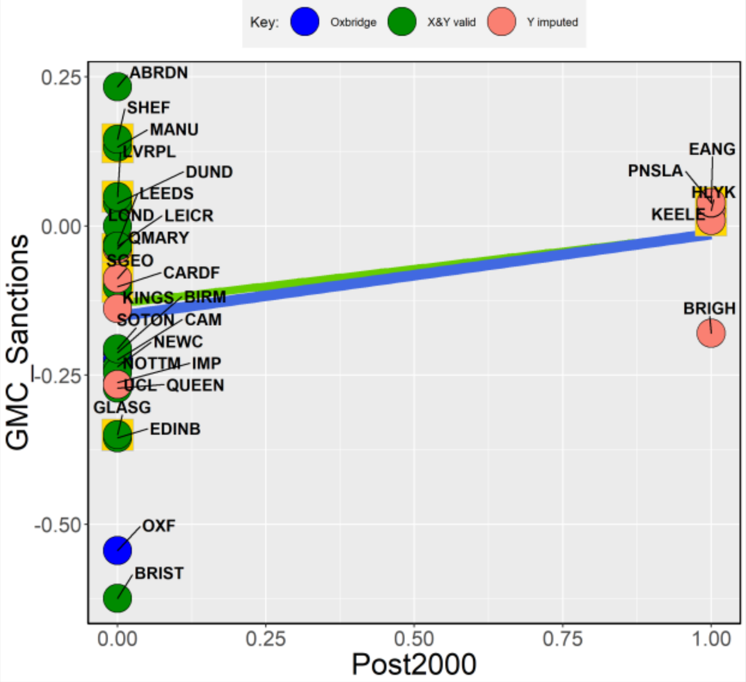

68/405 Y50: ARCP_NotExam X9: Post2000

r(all)= 0.300 p= 0.114 r(NonImp)= 0.276 Npairs=29 NimputedPairs=1

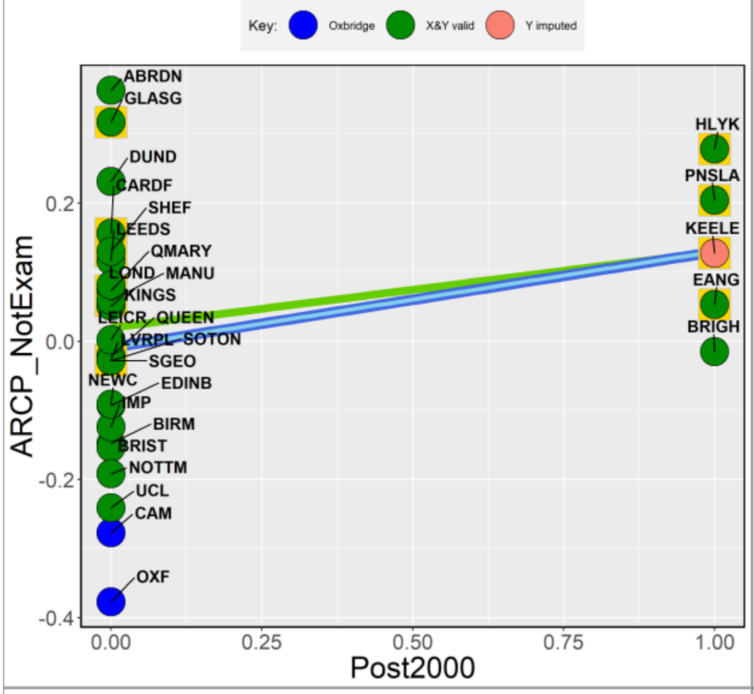

68/406 Y11: PBL_School X10: REF

r(all)= -0.325 p= 0.0851 r(NonImp)= -0.317 Npairs=29 NimputedPairs=1

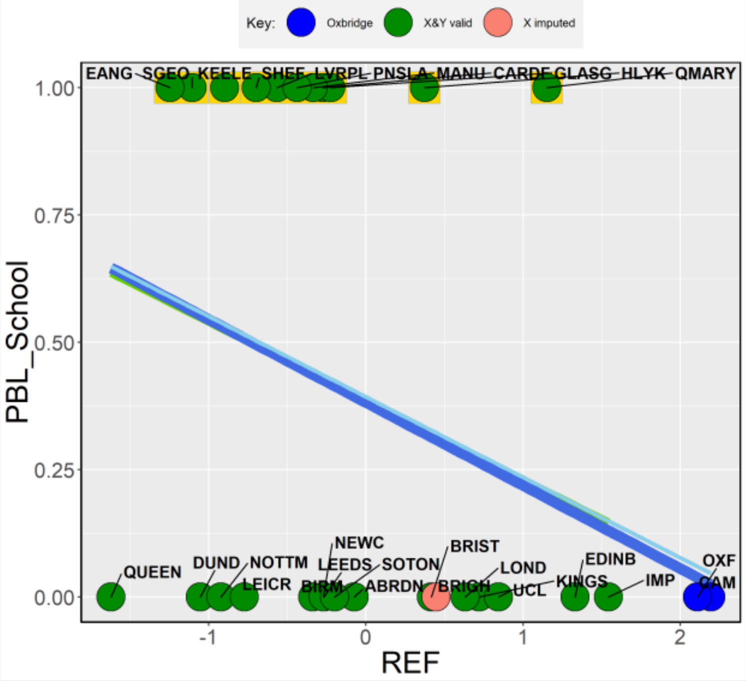

68/407 Y12: Spend_Student X10: REF

r(all)= 0.705 p= 1.97e-05 r(NonImp)= 0.708 Npairs=29 NimputedPairs=1

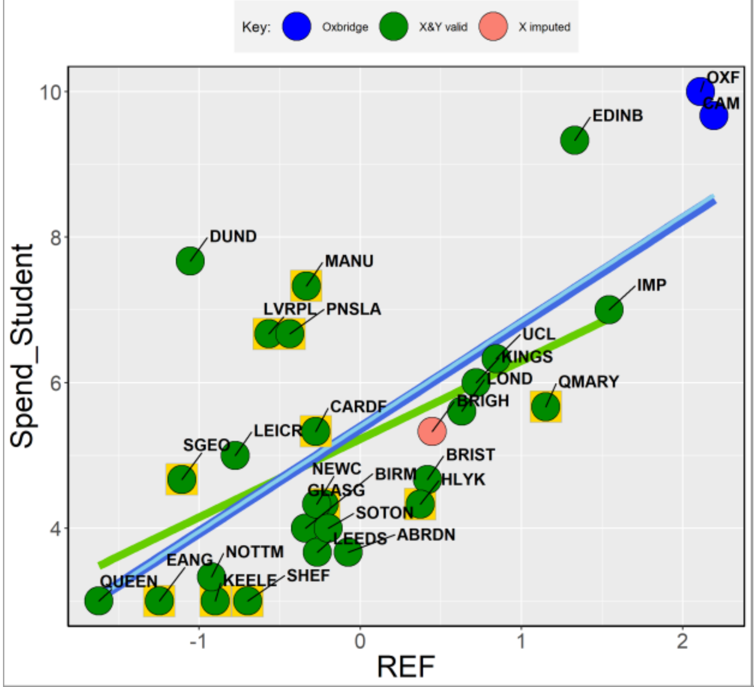

68/408 Y13: Student_Staff X10: REF

r(all)= -0.272 p= 0.154 r(NonImp)= -0.352 Npairs=29 NimputedPairs=1

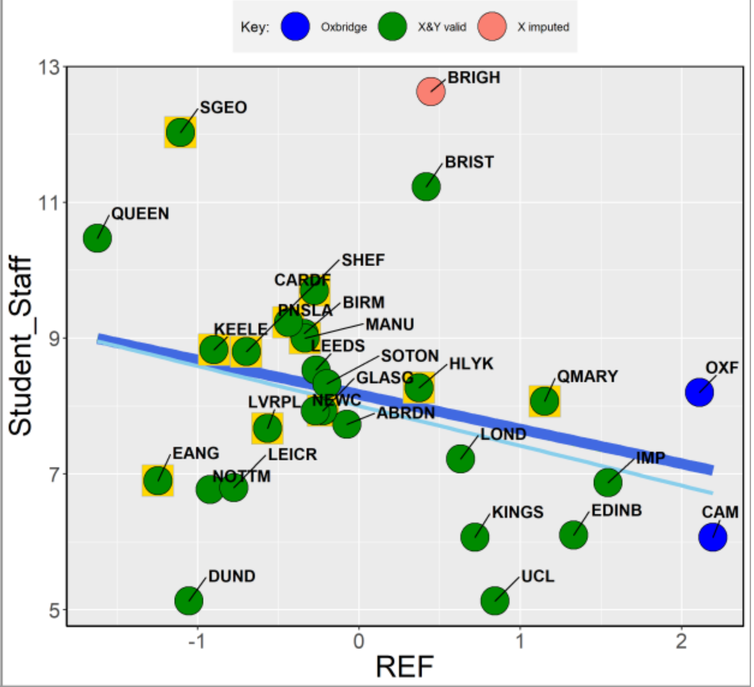

69/409 Y14: Entrants_N X10: REF

r(all)= 0.148 p= 0.445 r(NonImp)= 0.182 Npairs=29 NimputedPairs=1

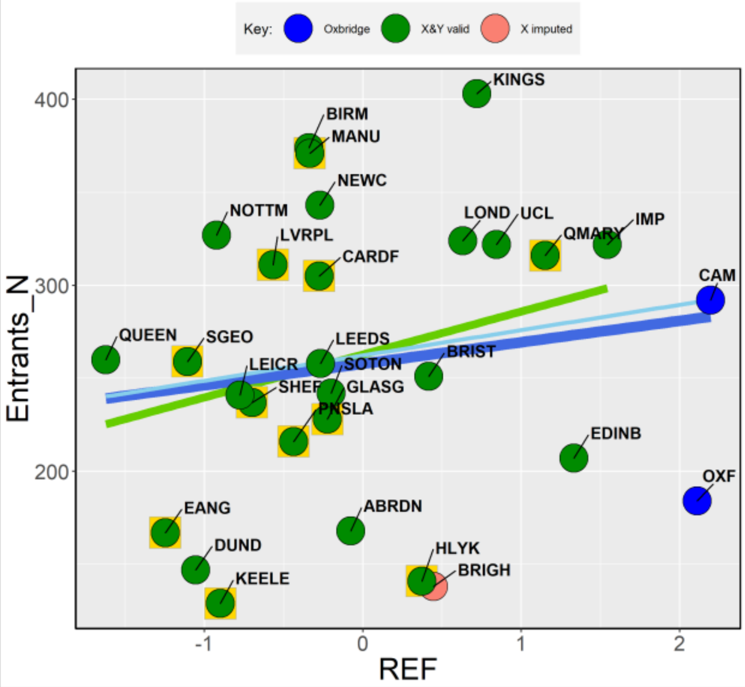

69/410 Y15: Entrants_Female X10: REF

r(all)= -0.546 p= 0.00218 r(NonImp)= -0.555 Npairs=29 NimputedPairs=1

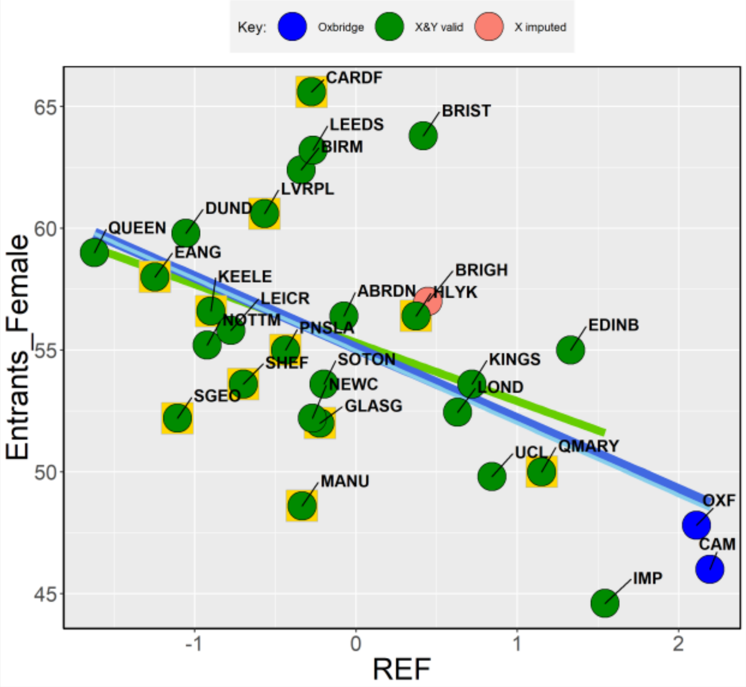

69/411 Y16: EntryGrades X10: REF

r(all)= 0.728 p= 7.53e-06 r(NonImp)= 0.759 Npairs=29 NimputedPairs=1

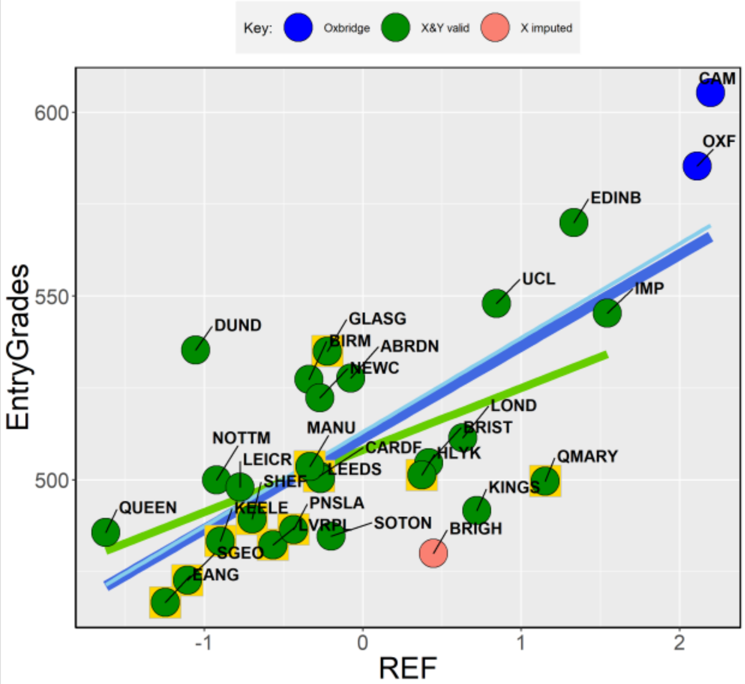

69/412 Y17: Entrants_NonHome X10: REF

r(all)= -0.270 p= 0.157 r(NonImp)= -0.264 Npairs=29 NimputedPairs=1

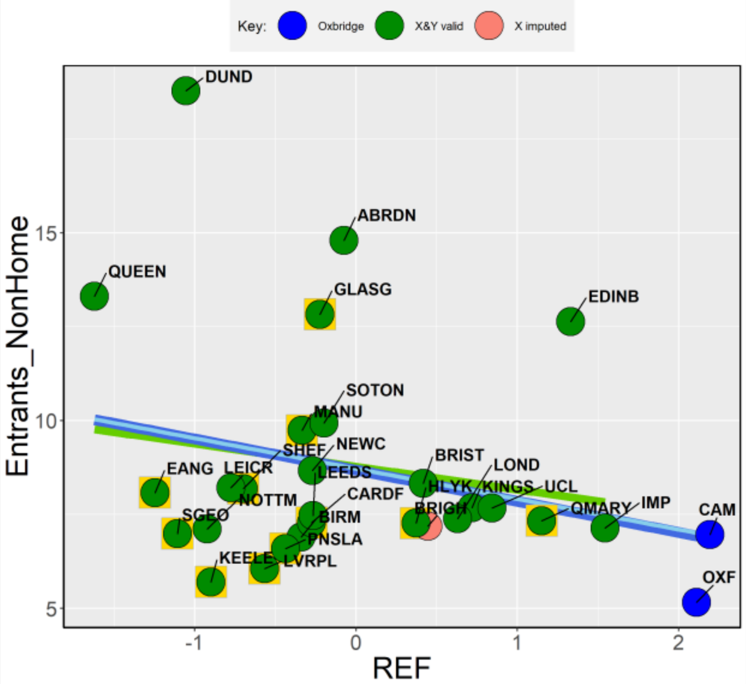

69/413 Y18: Teaching_Factor1_Trad X10: REF

r(all)= 0.433 p= 0.0189 r(NonImp)= 0.422 Npairs=29 NimputedPairs=4

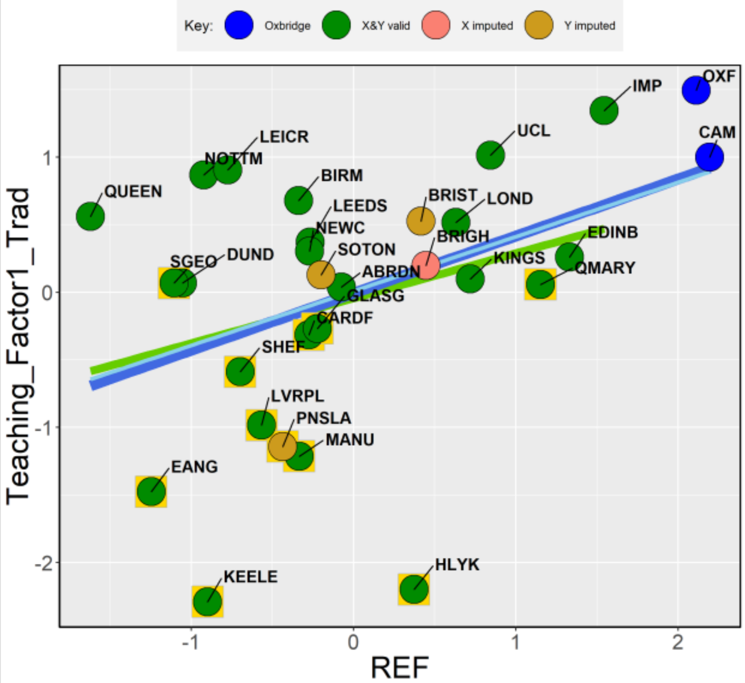

69/414 Y19: Teaching_Factor2_Struc X10: REF

r(all)= -0.195 p= 0.31 r(NonImp)= -0.195 Npairs=29 NimputedPairs=4

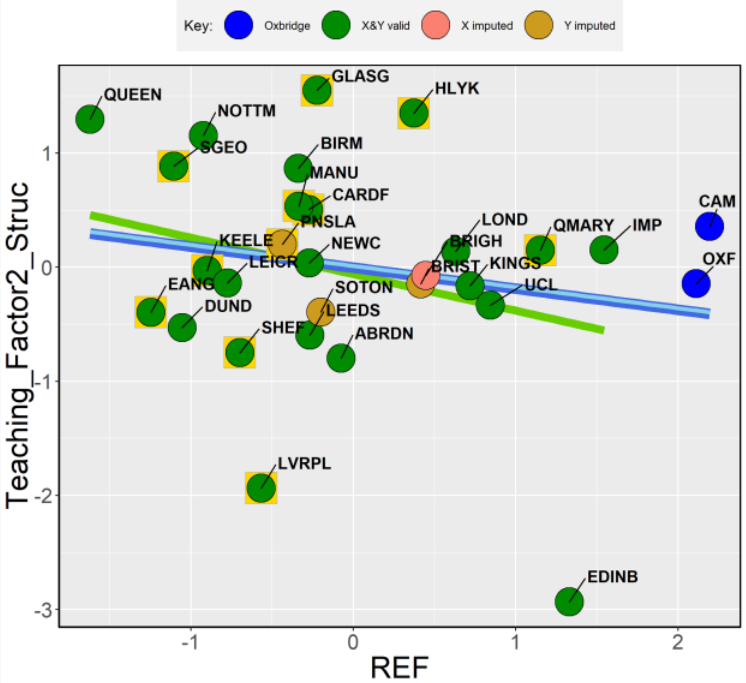

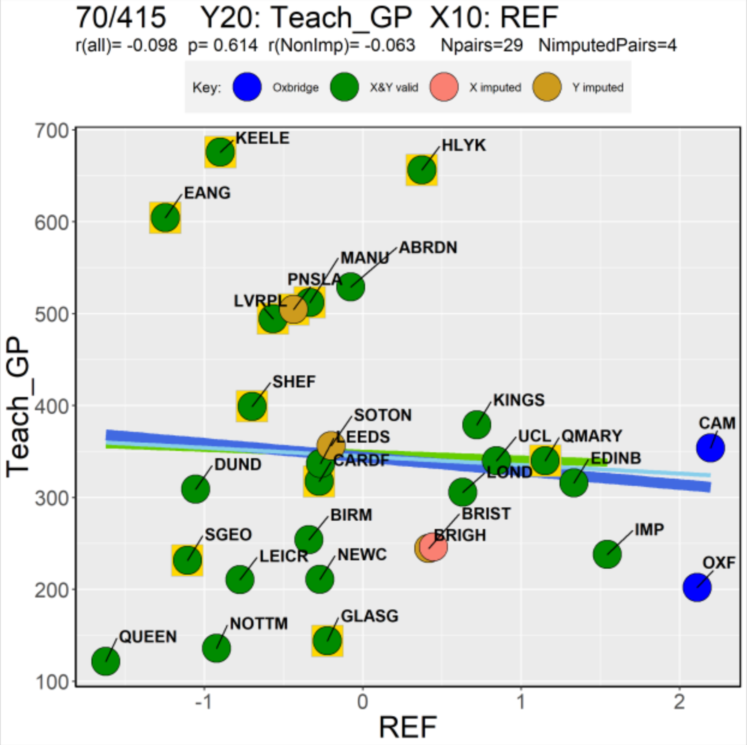

70/415 Y20: Teach_GP X10: REF
r(all)= -0.098 p= 0.614 r(NonImp)= -0.063 Npairs=29 NimputedPairs=4
Key: Oxbridge X&Y valid X imputed Y imputed
Teach_GP
REF

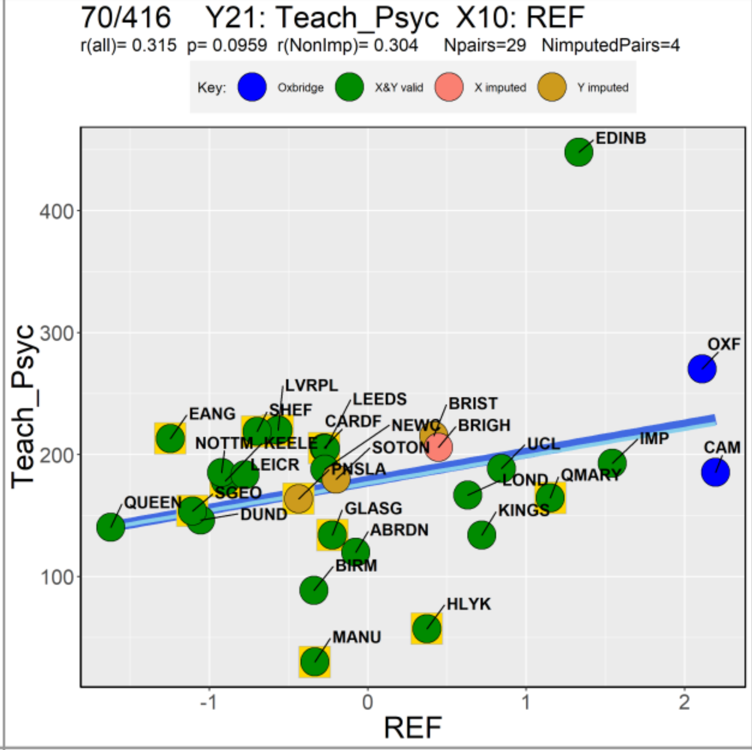

70/416 Y21: Teach_Psyc X10: REF
r(all)= 0.315 p= 0.0959 r(NonImp)= 0.304 Npairs=29 NimputedPairs=4
Key: Oxbridge X&Y valid X imputed Y imputed
Teach_Psyc
REF

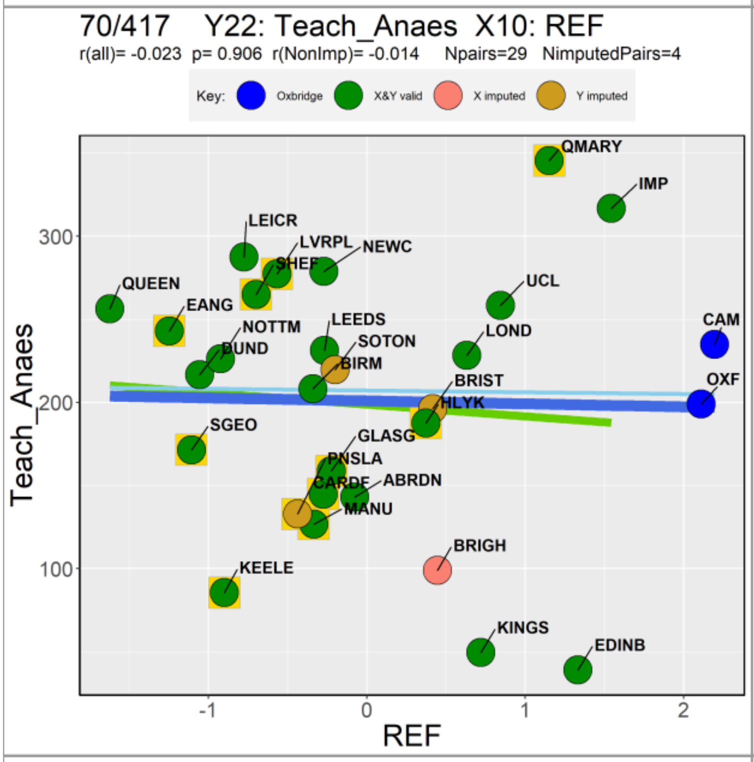

70/417 Y22: Teach_Anaes X10: REF
r(all)= -0.023 p= 0.906 r(NonImp)= -0.014 Npairs=29 NimputedPairs=4
Key: Oxbridge X&Y valid X imputed Y imputed
Teach_Anaes
REF

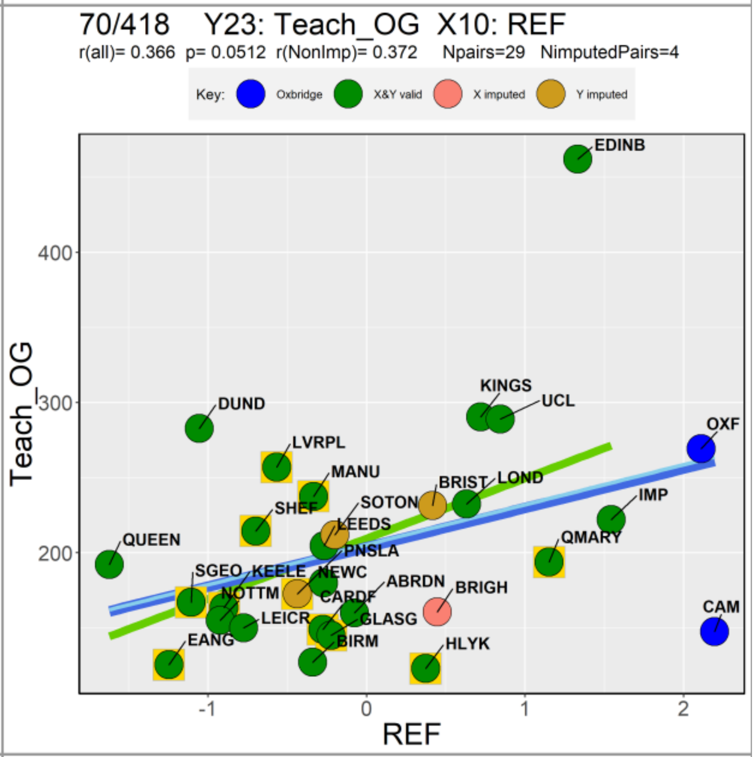

70/418 Y23: Teach_OG X10: REF
r(all)= 0.366 p= 0.0512 r(NonImp)= 0.372 Npairs=29 NimputedPairs=4
Key: Oxbridge X&Y valid X imputed Y imputed
Teach_OG
REF

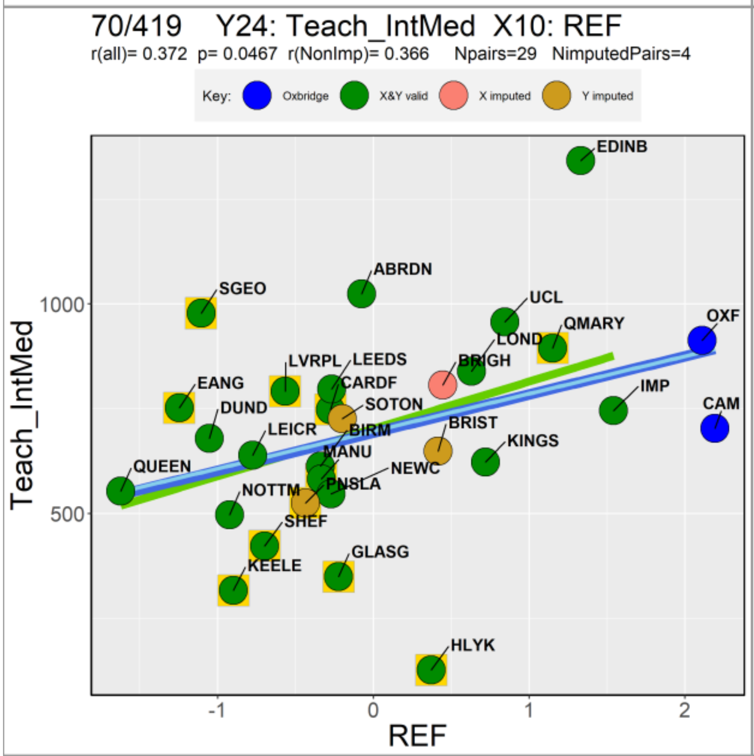

70/419 Y24: Teach_IntMed X10: REF
r(all)= 0.372 p= 0.0467 r(NonImp)= 0.366 Npairs=29 NimputedPairs=4
Key: Oxbridge X&Y valid X imputed Y imputed
Teach_IntMed
REF

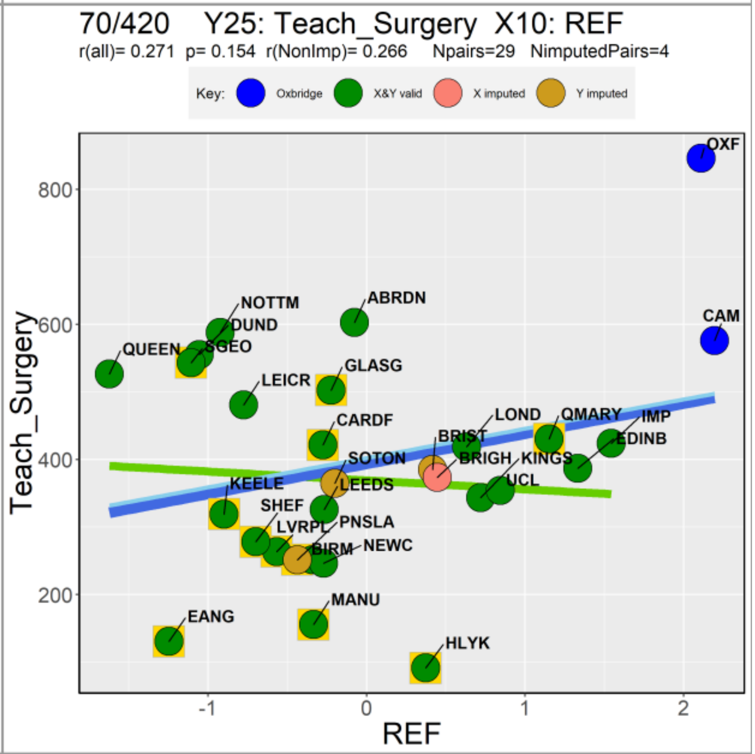

70/420 Y25: Teach_Surgery X10: REF
r(all)= 0.271 p= 0.154 r(NonImp)= 0.266 Npairs=29 NimputedPairs=4
Key: Oxbridge X&Y valid X imputed Y imputed
Teach_Surgery
REF
