# Supplementary material for: Exploring UK medical school differences: the MedDifs study of selection, teaching, student and F1 perceptions, postgraduate outcomes and fitness to practise
Source: BMC Med. 2020 May 14;18:136. doi: 10.1186/s12916-020-01572-3 (PMC7222458; doi:10.1186/s12916-020-01572-3)

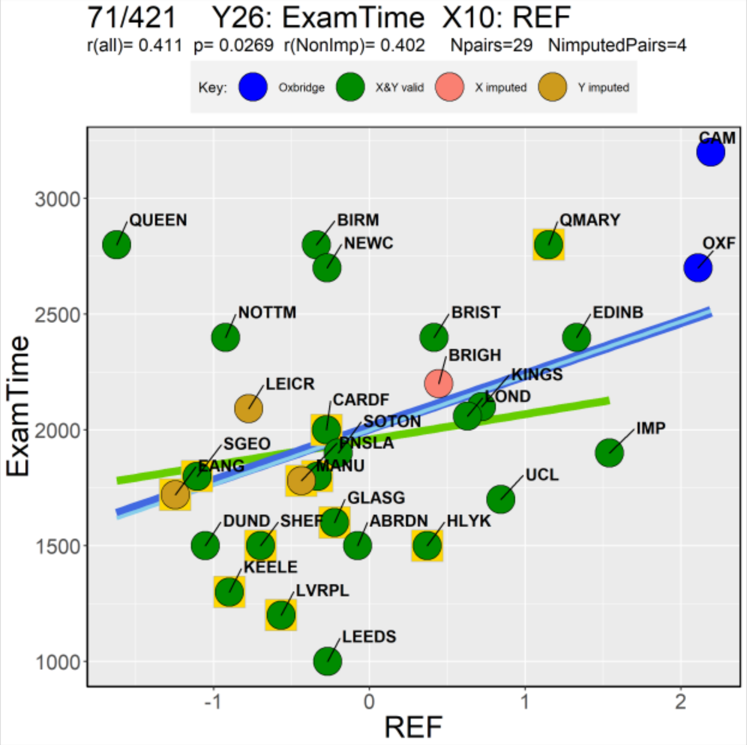
71/421 Y26: ExamTime X10: REF
r(all)= 0.411 p= 0.0269 r(NonImp)= 0.402 Npairs=29 NimputedPairs=4
Key: Oxbridge X&Y valid X imputed Y imputed
ExamTime
REF

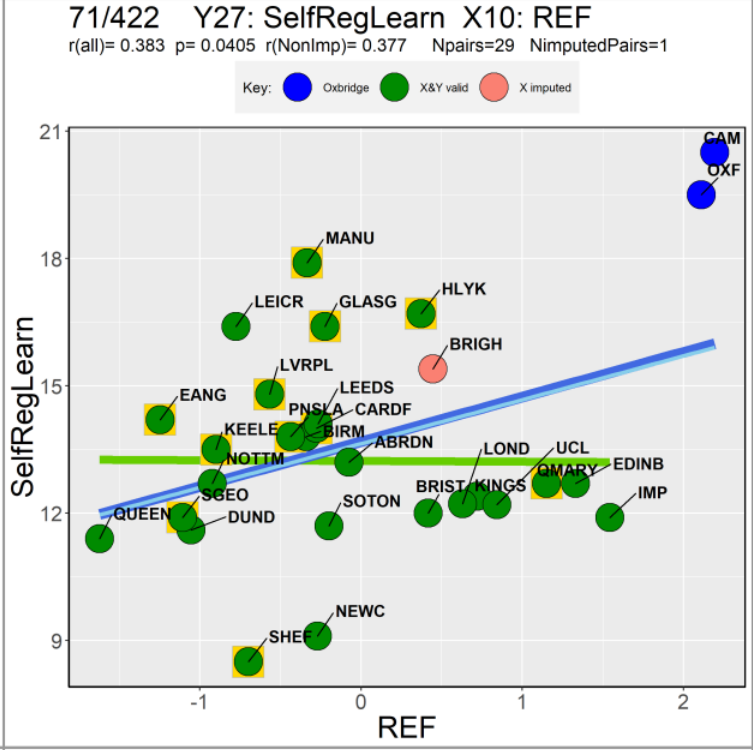
71/422 Y27: SelfRegLearn X10: REF
r(all)= 0.383 p= 0.0405 r(NonImp)= 0.377 Npairs=29 NimputedPairs=1
Key: Oxbridge X&Y valid X imputed
SelfRegLearn
REF

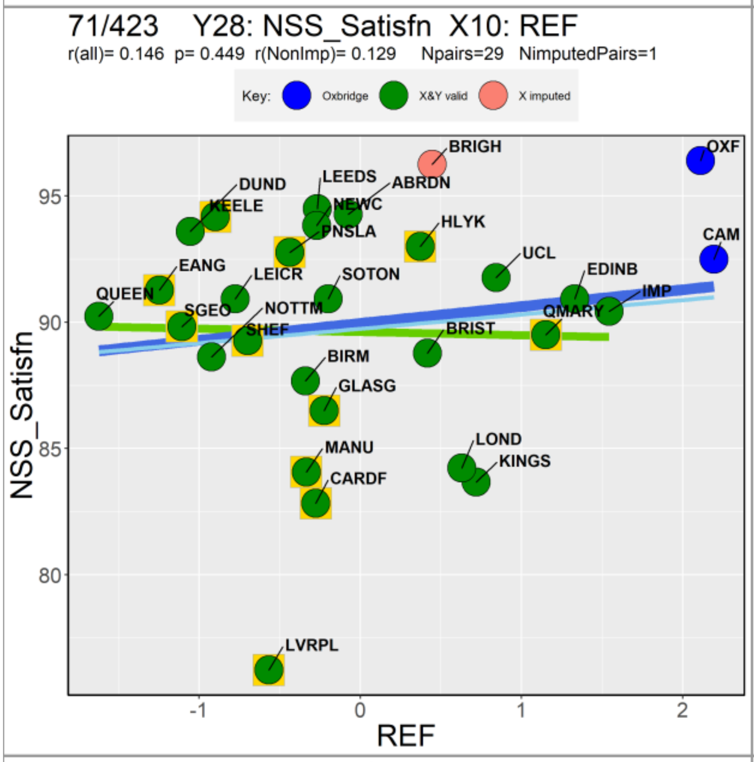
71/423 Y28: NSS_Satisfn X10: REF
r(all)= 0.146 p= 0.449 r(NonImp)= 0.129 Npairs=29 NimputedPairs=1
Key: Oxbridge X&Y valid X imputed
NSS_Satisfn
REF

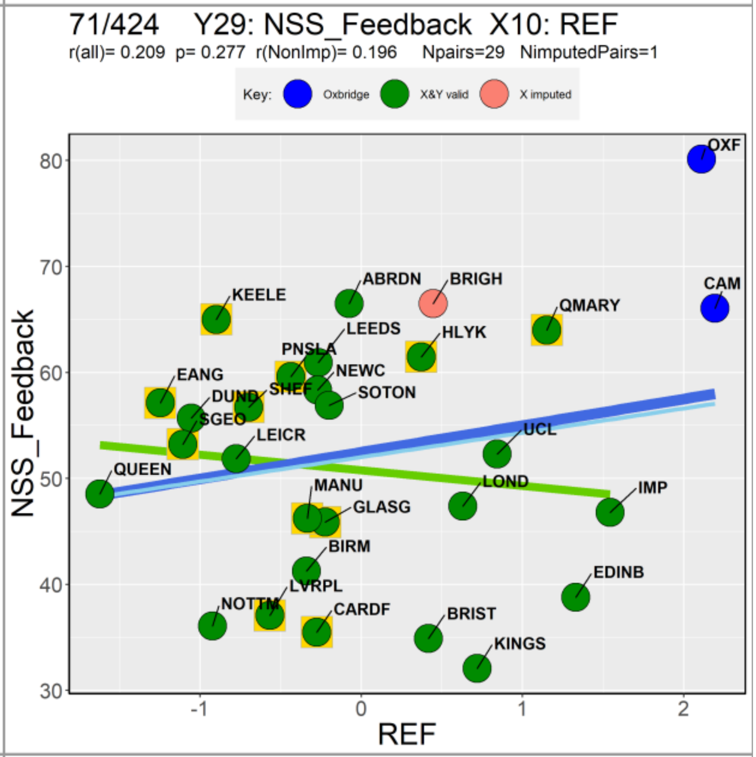
71/424 Y29: NSS_Feedback X10: REF
r(all)= 0.209 p= 0.277 r(NonImp)= 0.196 Npairs=29 NimputedPairs=1
Key: Oxbridge X&Y valid X imputed
NSS_Feedback
REF

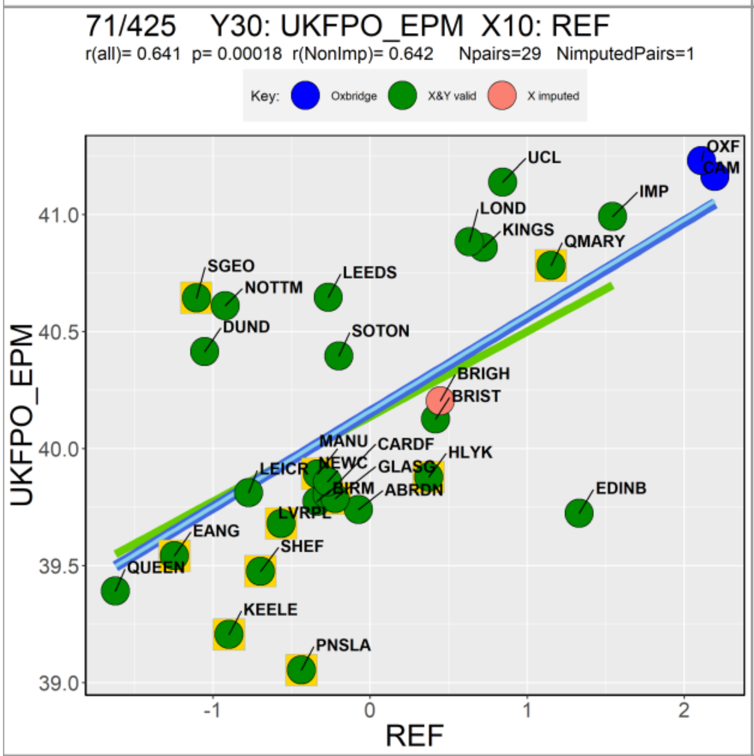
71/425 Y30: UKFPO_EPM X10: REF
r(all)= 0.641 p= 0.00018 r(NonImp)= 0.642 Npairs=29 NimputedPairs=1
Key: Oxbridge X&Y valid X imputed
UKFPO_EPM
REF

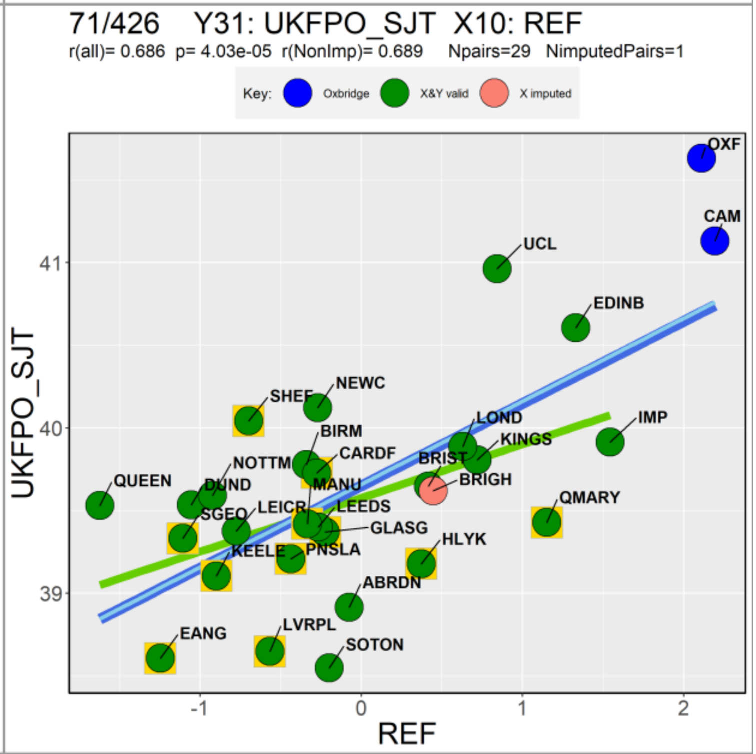
71/426 Y31: UKFPO_SJT X10: REF
r(all)= 0.686 p= 4.03e-05 r(NonImp)= 0.689 Npairs=29 NimputedPairs=1
Key: Oxbridge X&Y valid X imputed
UKFPO_SJT
REF

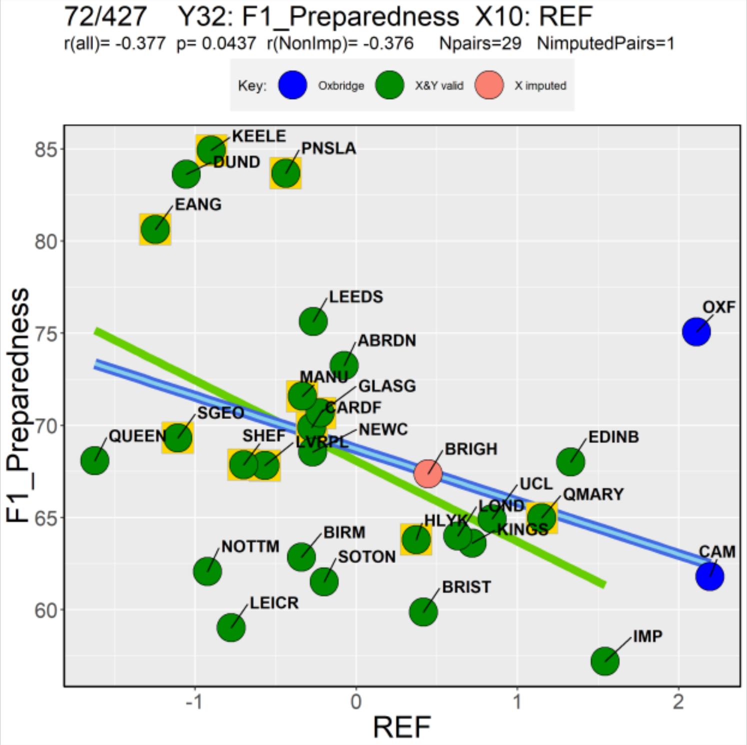
72/427 Y32: F1_Preparedness X10: REF
r(all)= -0.377 p= 0.0437 r(NonImp)= -0.376 Npairs=29 NimputedPairs=1
Key: Oxbridge X&Y valid X imputed
F1_Preparedness
REF

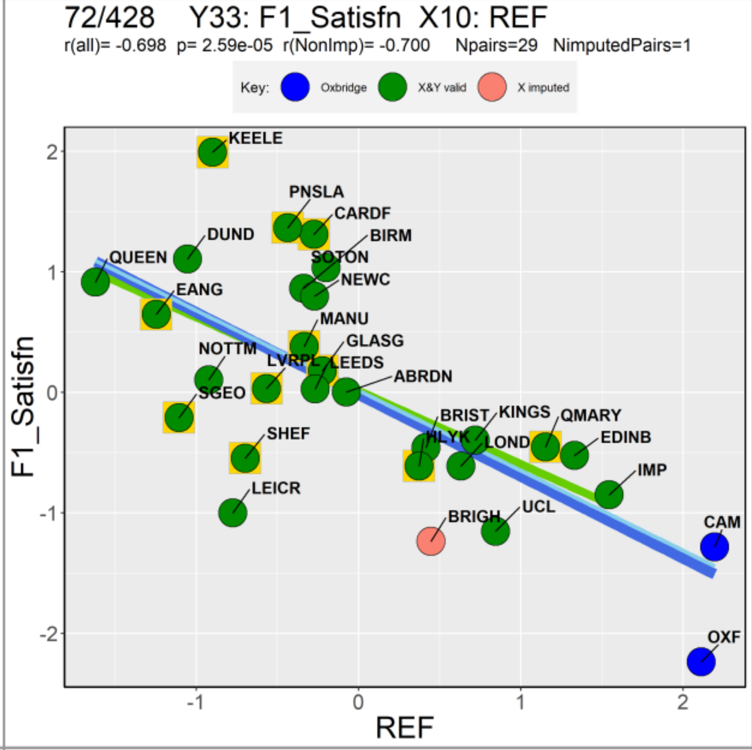
72/428 Y33: F1_Satisfn X10: REF
r(all)= -0.698 p= 2.59e-05 r(NonImp)= -0.700 Npairs=29 NimputedPairs=1
Key: Oxbridge X&Y valid X imputed
F1_Satisfn
REF

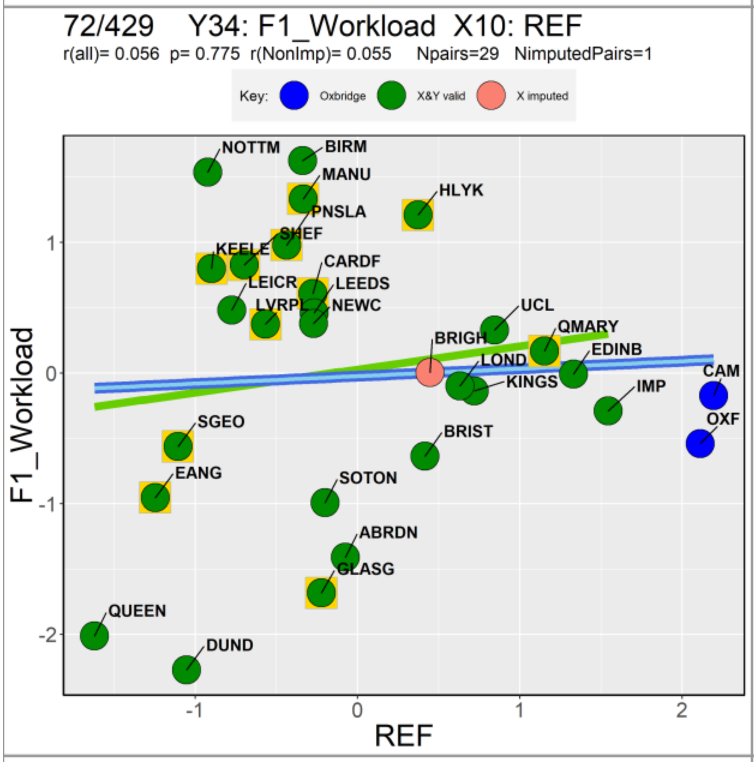
72/429 Y34: F1_Workload X10: REF
r(all)= 0.056 p= 0.775 r(NonImp)= 0.055 Npairs=29 NimputedPairs=1
Key: Oxbridge X&Y valid X imputed
F1_Workload
REF

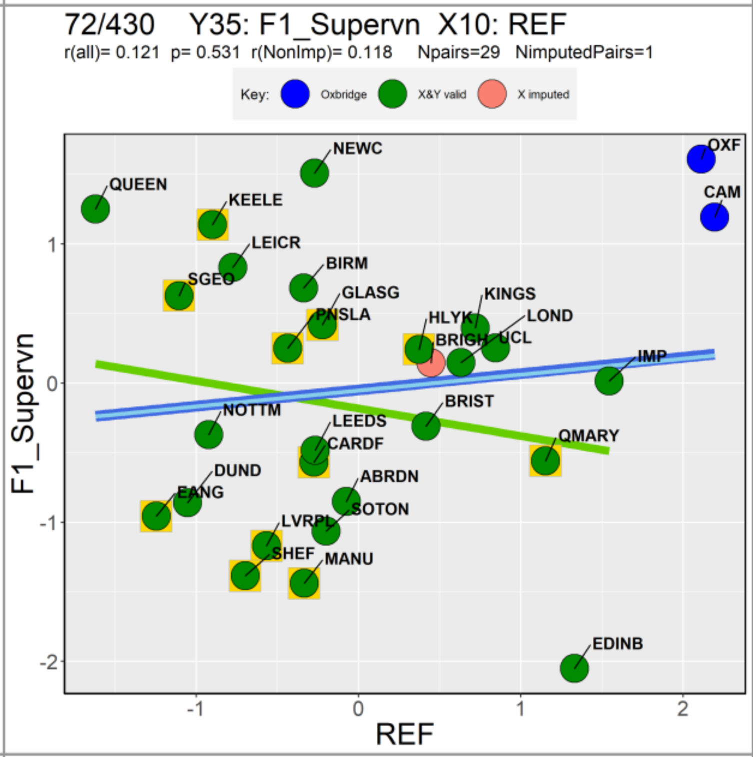
72/430 Y35: F1_Supervn X10: REF
r(all)= 0.121 p= 0.531 r(NonImp)= 0.118 Npairs=29 NimputedPairs=1
Key: Oxbridge X&Y valid X imputed
F1_Supervn
REF

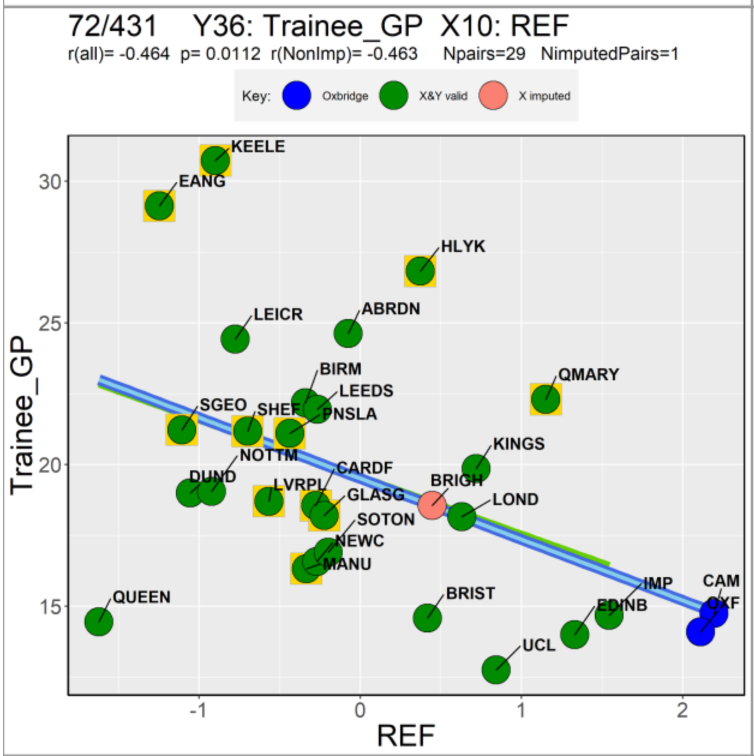
72/431 Y36: Trainee_GP X10: REF
r(all)= -0.464 p= 0.0112 r(NonImp)= -0.463 Npairs=29 NimputedPairs=1
Key: Oxbridge X&Y valid X imputed
Trainee_GP
REF

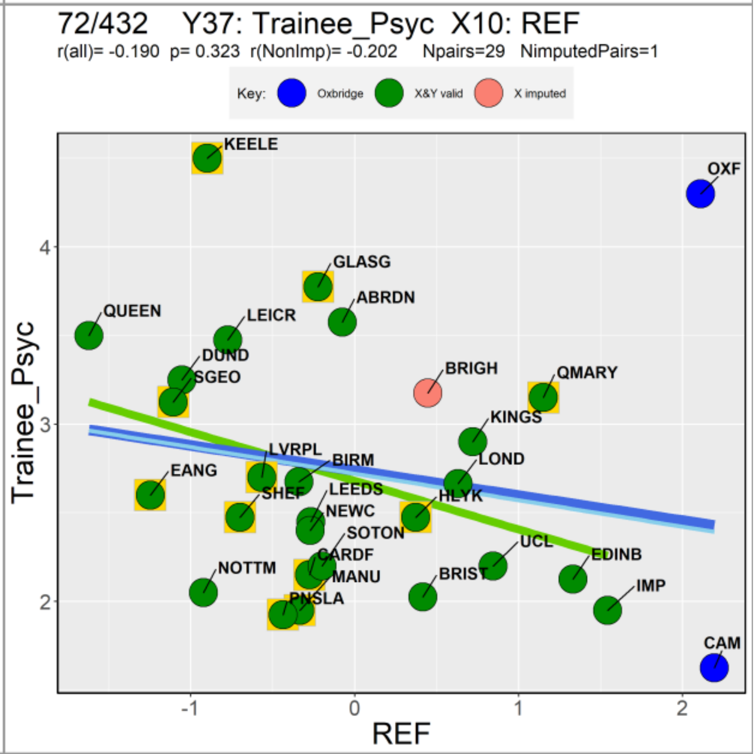
72/432 Y37: Trainee_Psyc X10: REF
r(all)= -0.190 p= 0.323 r(NonImp)= -0.202 Npairs=29 NimputedPairs=1
Key: Oxbridge X&Y valid X imputed
Trainee_Psyc
REF

73/433 Y38: TraineeApp_Surgery X10: REF
r(all)= 0.120 p= 0.536 r(NonImp)= 0.139 Npairs=29 NimputedPairs=3

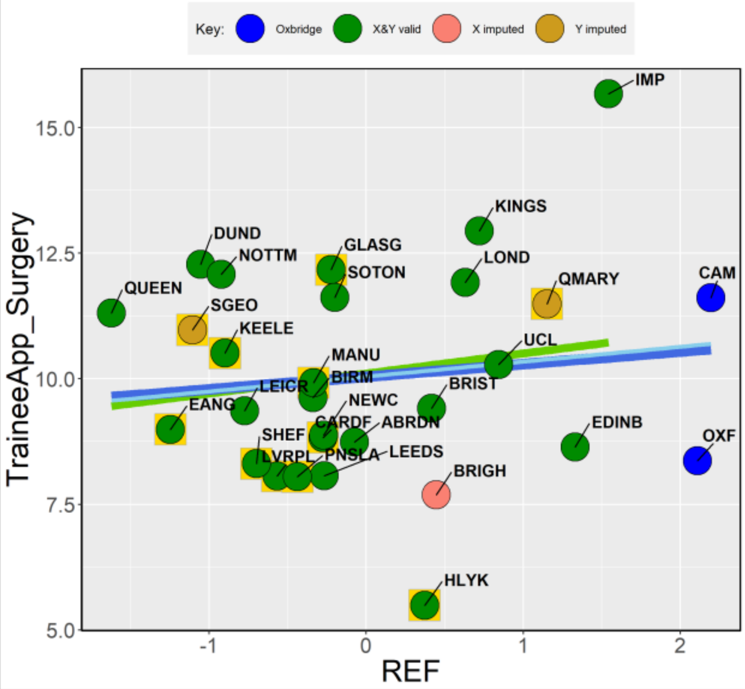

73/434 Y39: TraineeApp_Anaes X10: REF
r(all)= -0.068 p= 0.727 r(NonImp)= -0.045 Npairs=29 NimputedPairs=1

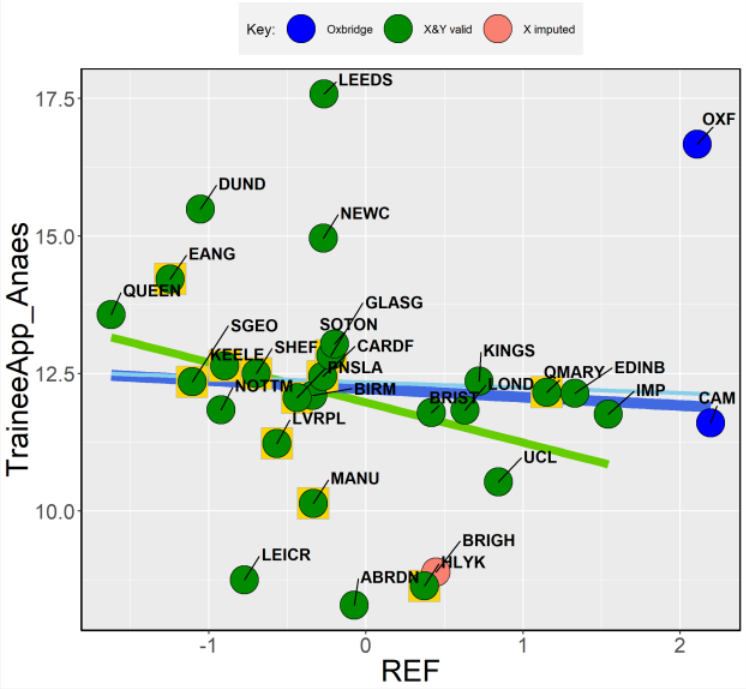

73/435 Y40: GMC_PGexams X10: REF
r(all)= 0.615 p= 0.000384 r(NonImp)= 0.616 Npairs=29 NimputedPairs=1

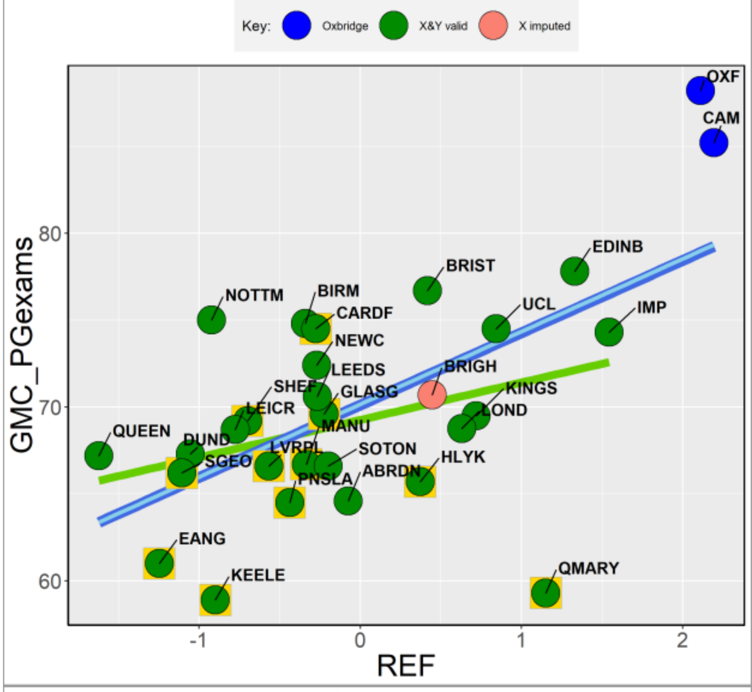

73/436 Y41: MRCGP_AKT X10: REF
r(all)= 0.515 p= 0.00423 r(NonImp)= 0.514 Npairs=29 NimputedPairs=1

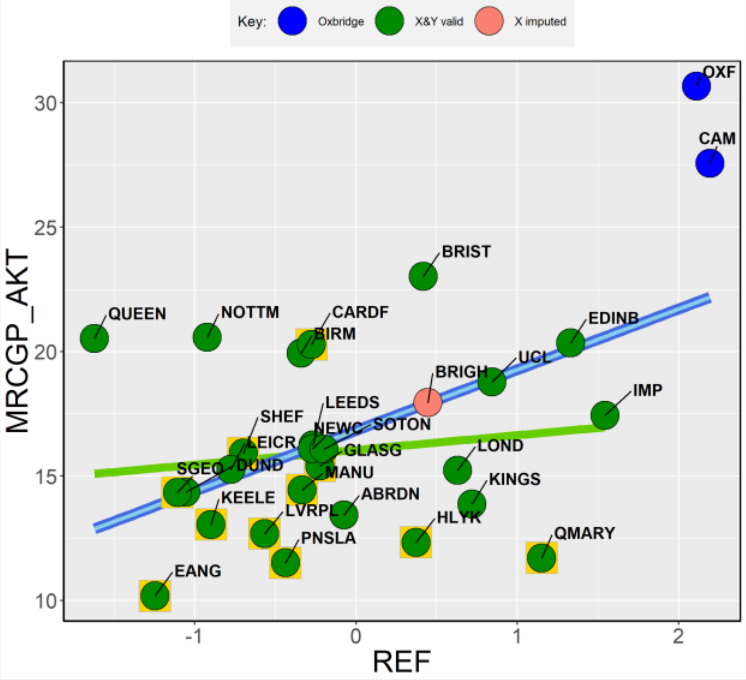

73/437 Y42: MRCGP_CSA X10: REF
r(all)= 0.379 p= 0.0424 r(NonImp)= 0.375 Npairs=29 NimputedPairs=1

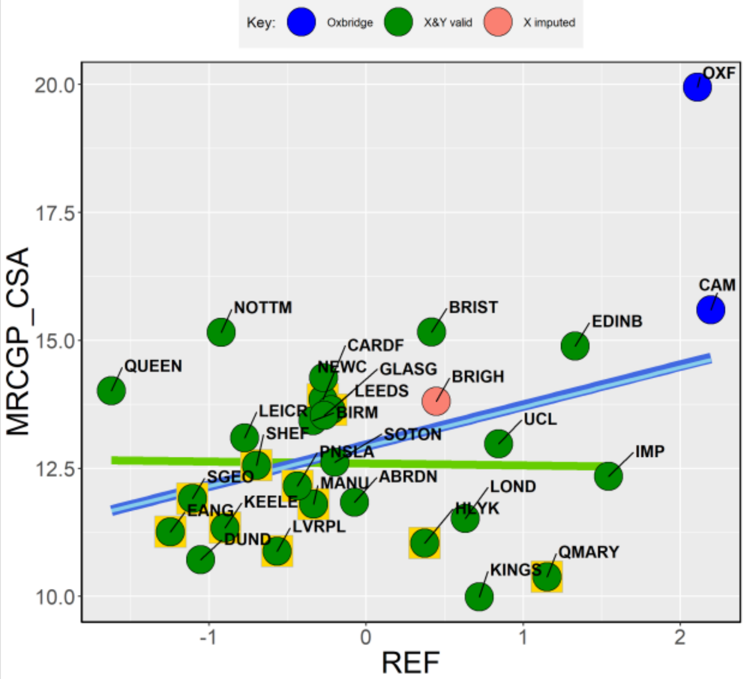

73/438 Y43: FRCA_Pt1 X10: REF
r(all)= 0.702 p= 2.2e-05 r(NonImp)= 0.791 Npairs=29 NimputedPairs=10

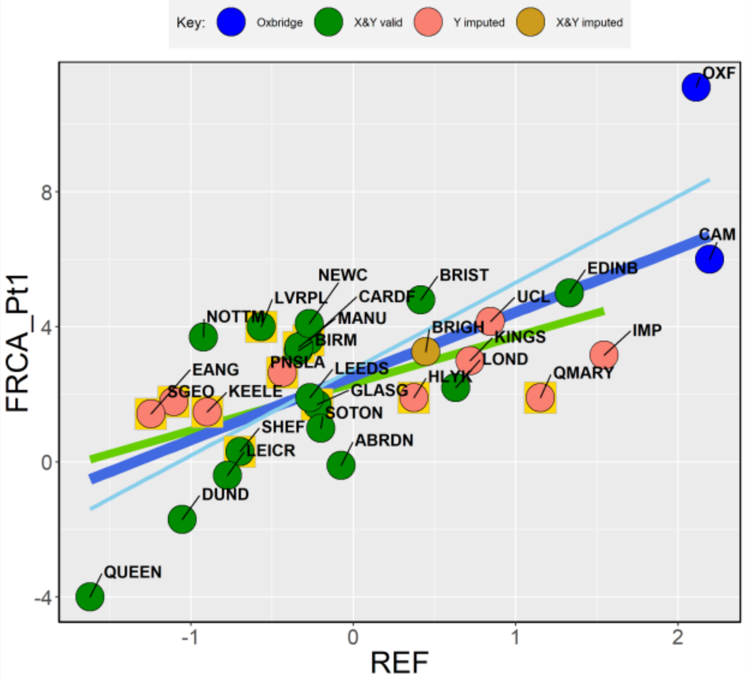

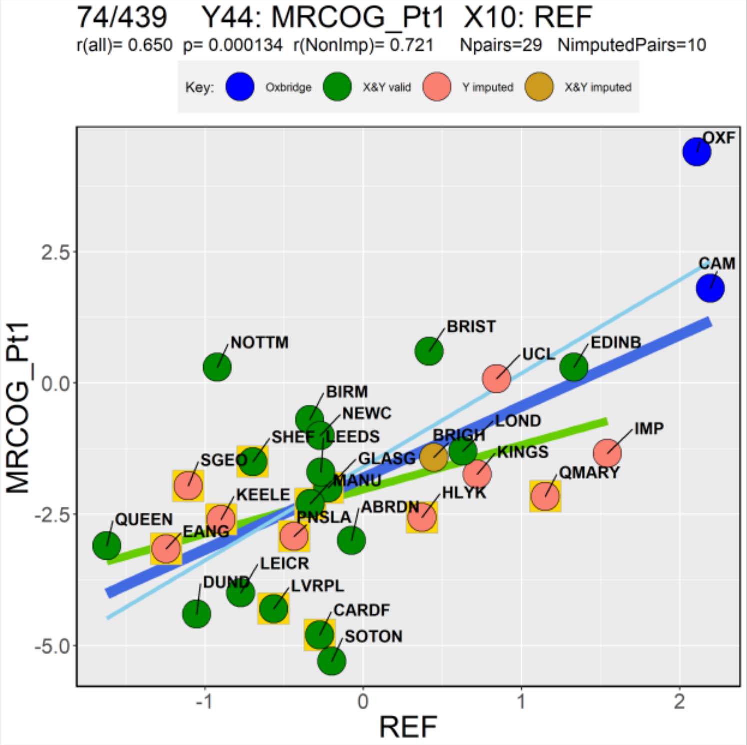
74/439 Y44: MRCOG_Pt1 X10: REF
r(all)= 0.650 p= 0.000134 r(NonImp)= 0.721 Npairs=29 NimputedPairs=10
Key: Oxbridge X&Y valid Y imputed X&Y imputed
MRCOG_Pt1
2.5
0.0
-2.5
-5.0
-1
0
1
2
REF
OXF
CAM
BRIST
NOTTM
UCL
EDINB
BIRM
NEWC
SHEF
LEEDS
LOND
BRIGH
IMP
SGEO
GLASG
KINGS
MANU
QMARY
KEELE
HLYK
ABRDN
QUEEN
PNSLA
EANG
LEICR
DUND
LVRPL
CARDF
SOTON

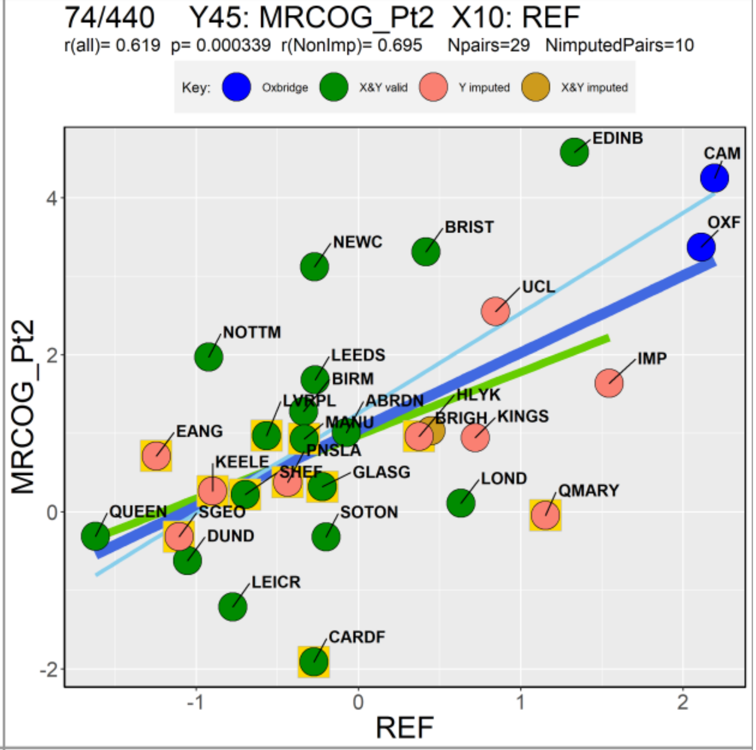
74/440 Y45: MRCOG_Pt2 X10: REF
r(all)= 0.619 p= 0.000339 r(NonImp)= 0.695 Npairs=29 NimputedPairs=10
Key: Oxbridge X&Y valid Y imputed X&Y imputed
MRCOG_Pt2
4
2
0
-2
-1
0
1
2
REF
EDINB
CAM
OXF
BRIST
NEWC
UCL
NOTTM
LEEDS
BIRM
IMP
LVRPL
ABRDN
HLYK
MANU
BRIGH
KINGS
EANG
PNSLA
KEELE
SHEF
GLASG
LOND
QMARY
QUEEN
SGEO
SOTON
DUND
LEICR
CARDF

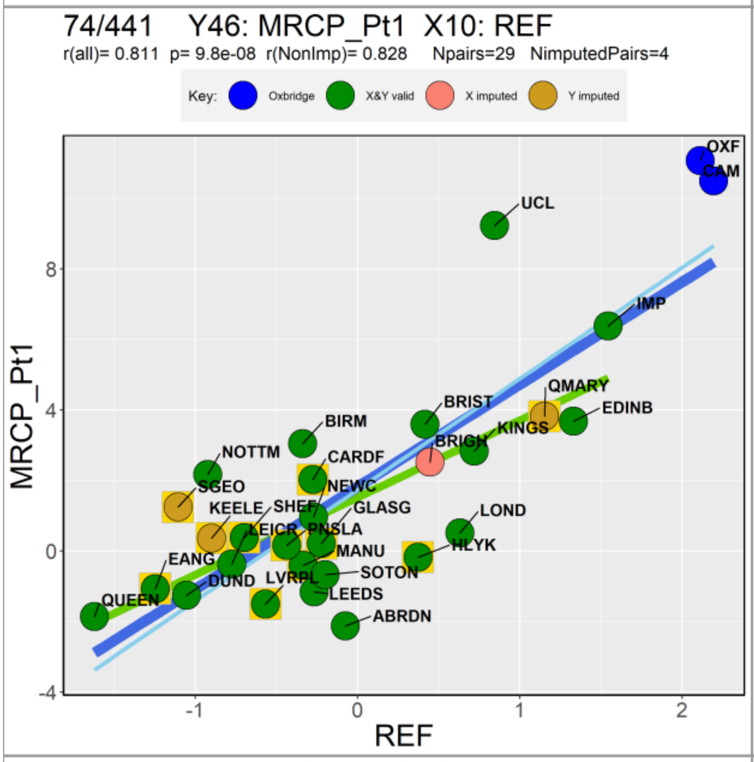
74/441 Y46: MRCP_Pt1 X10: REF
r(all)= 0.811 p= 9.8e-08 r(NonImp)= 0.828 Npairs=29 NimputedPairs=4
Key: Oxbridge X&Y valid X imputed Y imputed
MRCP_Pt1
8
4
0
-4
-1
0
1
2
REF
OXF
CAM
UCL
IMP
QMARY
BRIST
EDINB
BIRM
KINGS
BRIGH
NOTTM
CARDF
SGEO
NEWC
KEELE
SHEF
GLASG
LOND
LEICR
PNSLA
HLYK
MANU
EANG
SOTON
DUND
LVRPL
LEEDS
QUEEN
ABRDN

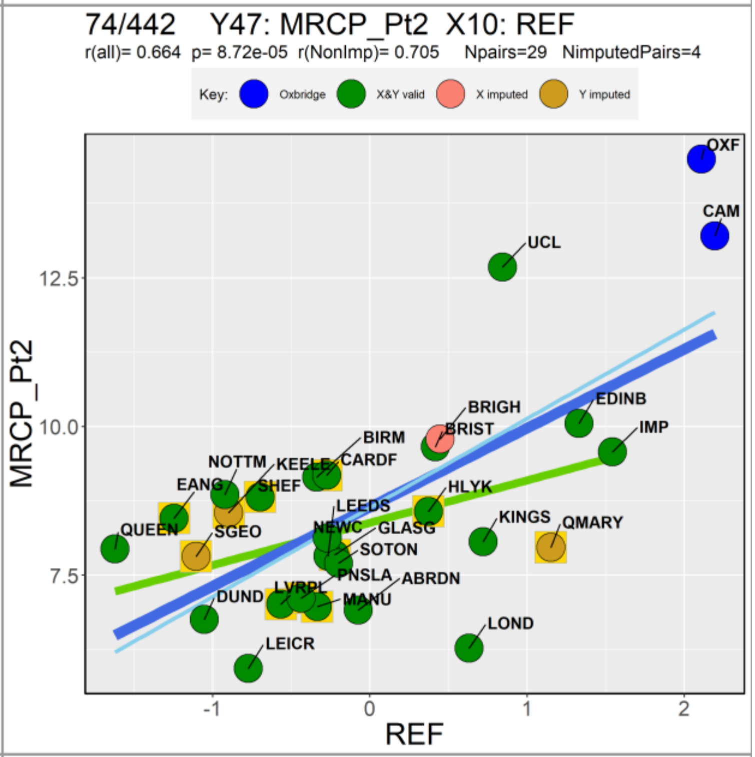
74/442 Y47: MRCP_Pt2 X10: REF
r(all)= 0.664 p= 8.72e-05 r(NonImp)= 0.705 Npairs=29 NimputedPairs=4
Key: Oxbridge X&Y valid X imputed Y imputed
MRCP_Pt2
12.5
10.0
7.5
-1
0
1
2
REF
OXF
CAM
UCL
EDINB
BRIGH
IMP
BRIST
BIRM
NOTTM
KEELE
CARDF
EANG
SHEF
HLYK
LEEDS
KINGS
QUEEN
SGEO
QMARY
NEWC
GLASG
SOTON
PNSLA
ABRDN
DUND
LVRPL
MANU
LOND
LEICR

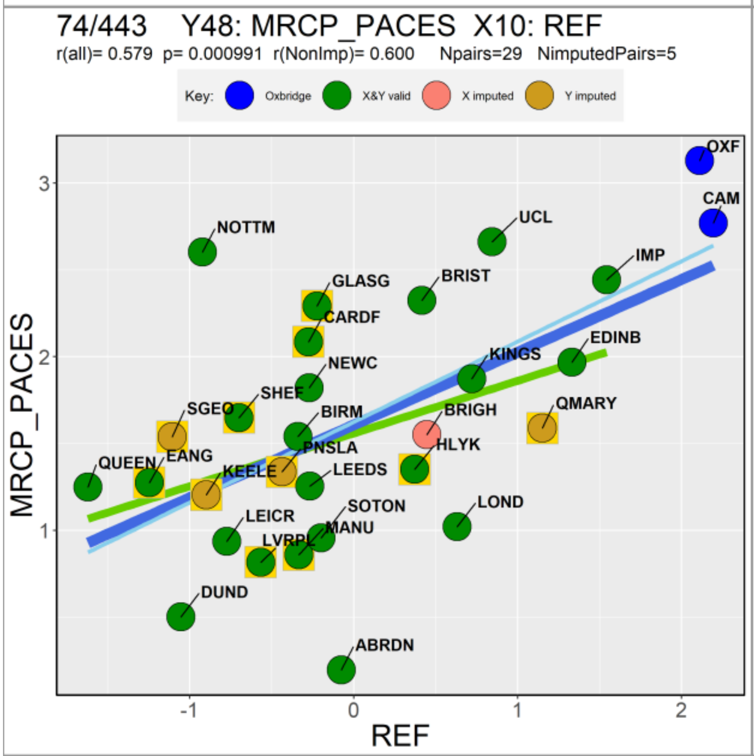
74/443 Y48: MRCP_PACES X10: REF
r(all)= 0.579 p= 0.000991 r(NonImp)= 0.600 Npairs=29 NimputedPairs=5
Key: Oxbridge X&Y valid X imputed Y imputed
MRCP_PACES
3
2
1
-1
0
1
2
REF
OXF
CAM
NOTTM
UCL
IMP
GLASG
BRIST
CARDF
EDINB
KINGS
NEWC
SHEF
SGEO
BIRM
QMARY
BRIGH
PNSLA
HLYK
QUEEN
EANG
KEELE
LEEDS
SOTON
LOND
LEICR
MANU
LVRPL
DUND
ABRDN

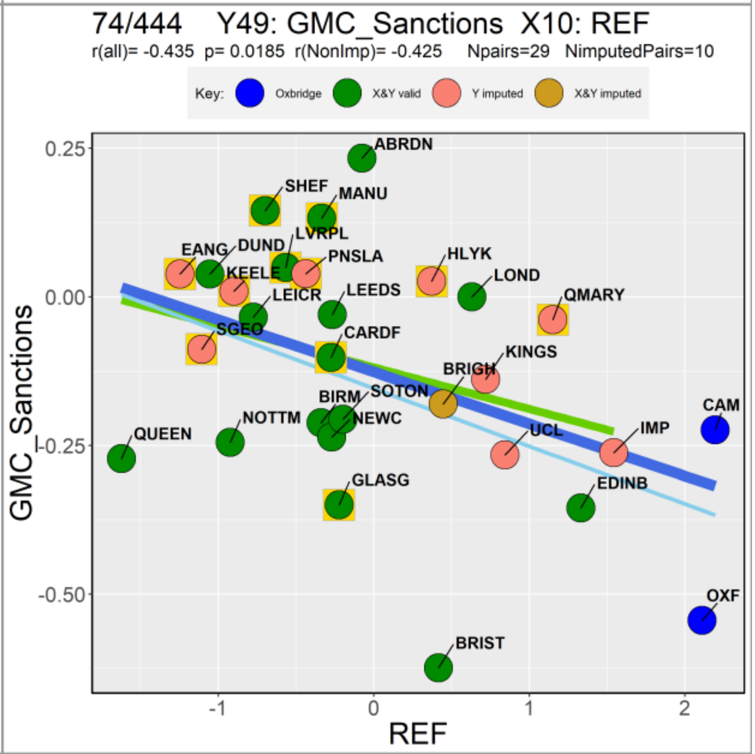
74/444 Y49: GMC_Sanctions X10: REF
r(all)= -0.435 p= 0.0185 r(NonImp)= -0.425 Npairs=29 NimputedPairs=10
Key: Oxbridge X&Y valid Y imputed X&Y imputed
GMC_Sanctions
0.25
0.00
-0.25
-0.50
-1
0
1
2
REF
ABRDN
SHEF
MANU
LVRPL
EANG
DUND
PNSLA
HLYK
KEELE
LOND
LEICR
LEEDS
QMARY
SGEO
CARDF
KINGS
BRIGH
BIRM
SOTON
NEWC
CAM
NOTTM
QUEEN
UCL
IMP
GLASG
EDINB
OXF
BRIST

75/445 Y50: ARCP_NotExam X10: REF

r(all)= -0.445 p= 0.0155 r(NonImp)= -0.433 Npairs=29 NimputedPairs=2

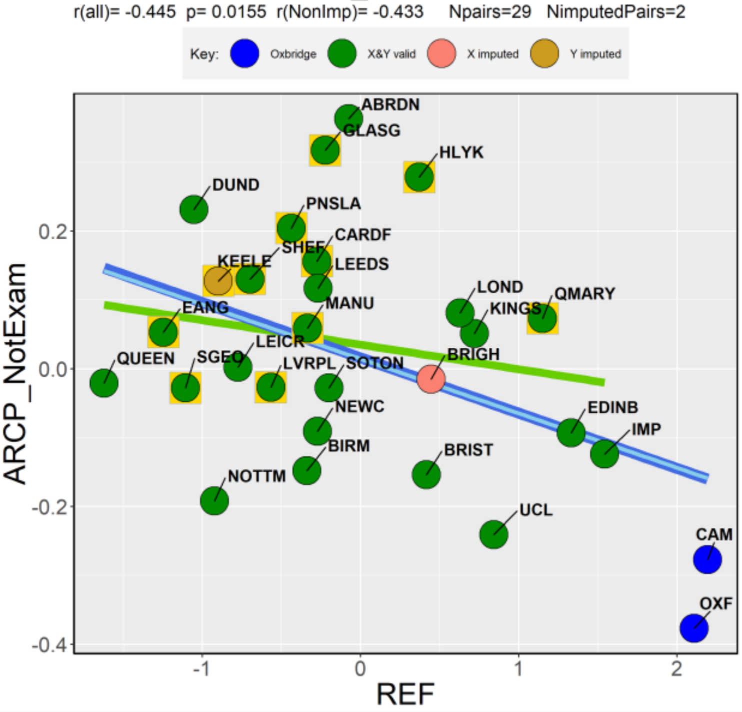

75/446 Y12: Spend_Student X11: PBL_School

r(all)= -0.195 p= 0.311 r(NonImp)= -0.195 Npairs=29 NimputedPairs=0

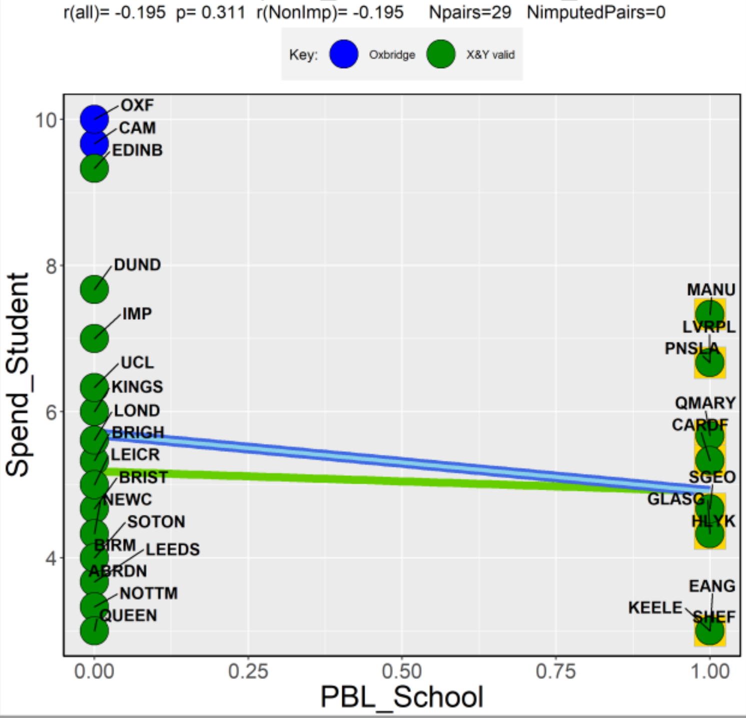

75/447 Y13: Student_Staff X11: PBL_School

r(all)= 0.260 p= 0.173 r(NonImp)= 0.260 Npairs=29 NimputedPairs=0

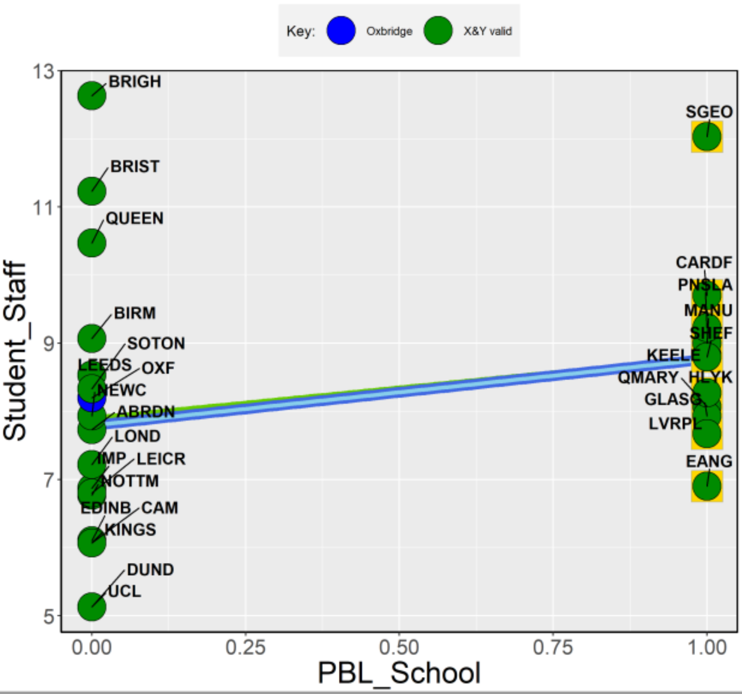

75/448 Y14: Entrants_N X11: PBL_School

r(all)= -0.149 p= 0.442 r(NonImp)= -0.149 Npairs=29 NimputedPairs=0

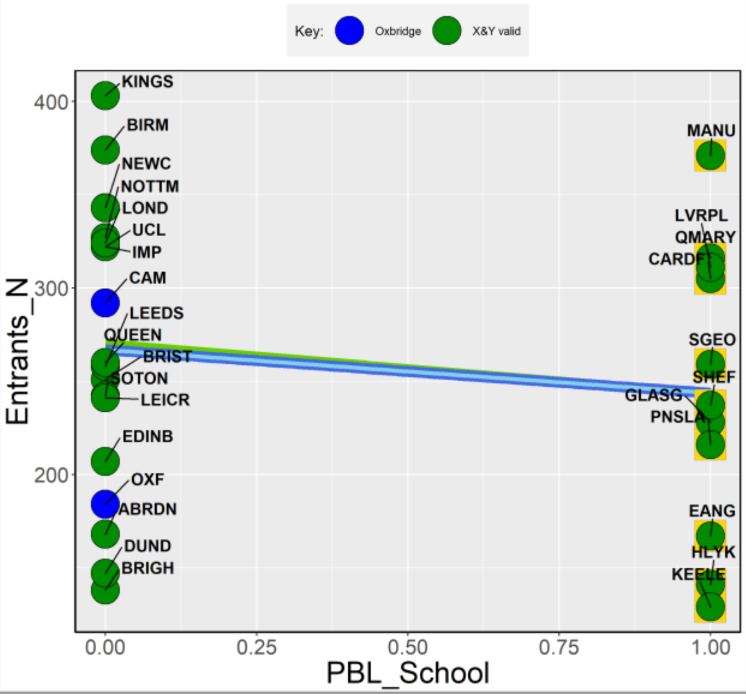

75/449 Y15: Entrants_Female X11: PBL_School

r(all)= 0.043 p= 0.825 r(NonImp)= 0.043 Npairs=29 NimputedPairs=0

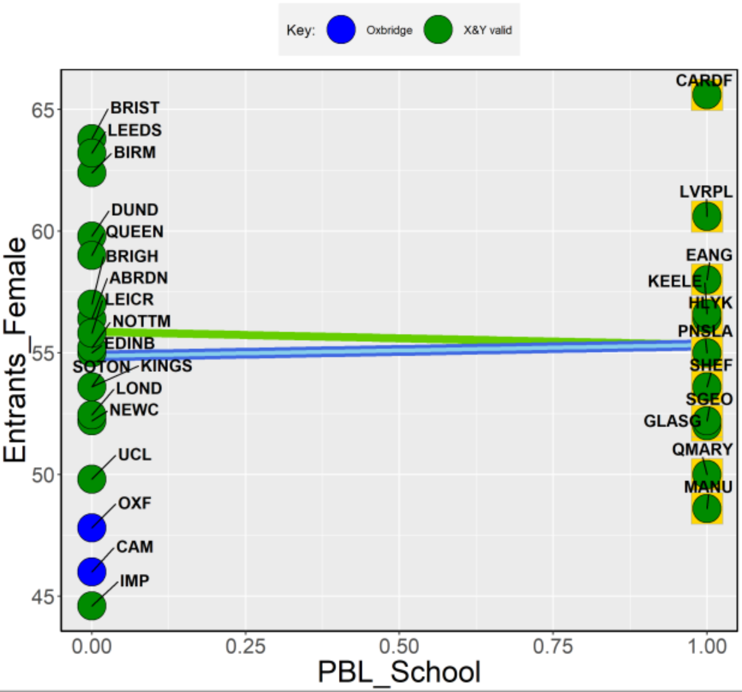

75/450 Y16: EntryGrades X11: PBL_School

r(all)= -0.449 p= 0.0146 r(NonImp)= -0.449 Npairs=29 NimputedPairs=0

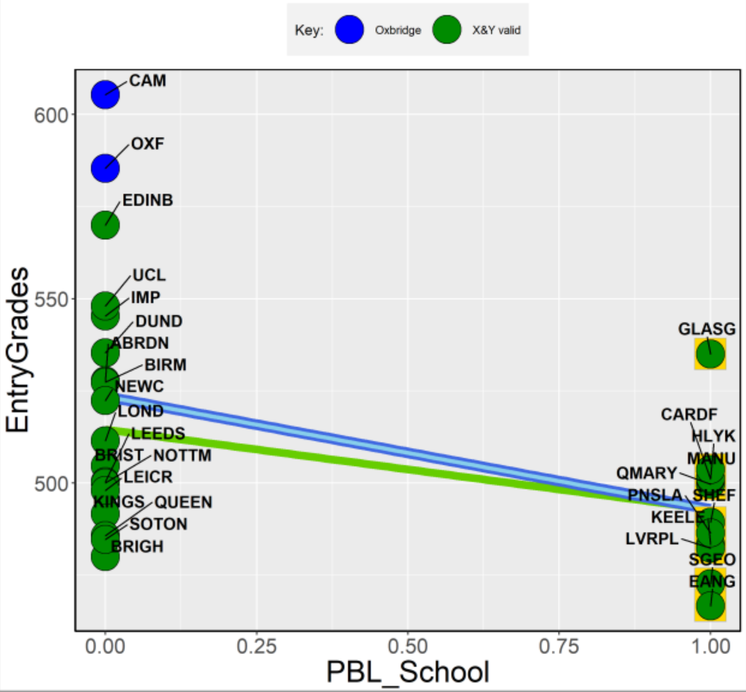

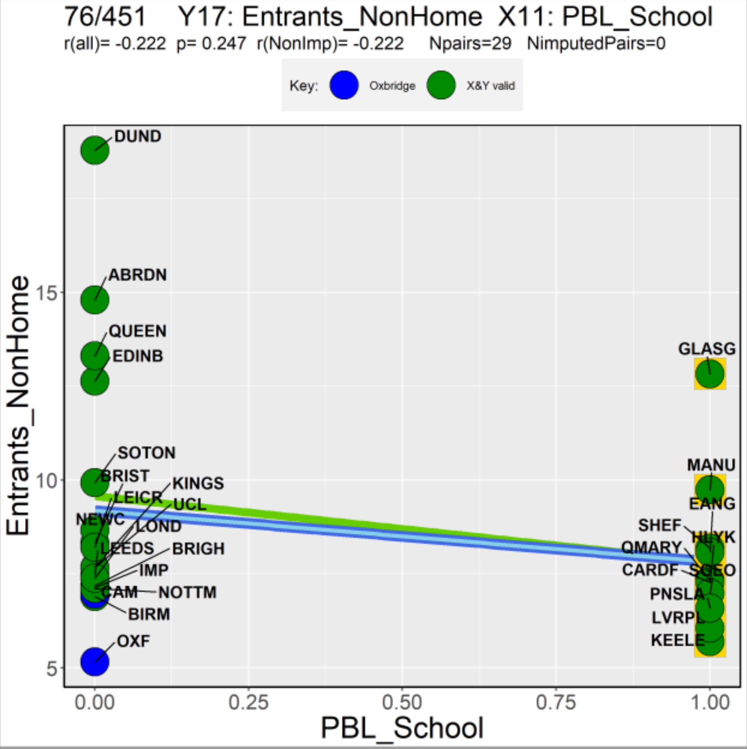
76/451 Y17: Entrants_NonHome X11: PBL_School
r(all)= -0.222 p= 0.247 r(NonImp)= -0.222 Npairs=29 NimputedPairs=0
Key: Oxbridge X&Y valid
Entrants_NonHome
PBL_School
0.00 0.25 0.50 0.75 1.00
5 10 15
DUND
ABRDN
QUEEN
EDINB
SOTON
BRIST
KINGS
LEICR
UCL
NEWC
LOND
LEEDS
BRIGH
IMP
CAM
NOTTM
BIRM
OXF
GLASG
MANU
EANG
SHEF
HLYK
QMARY
CARDF
SGEO
PNSLA
LVRPL
KEELE
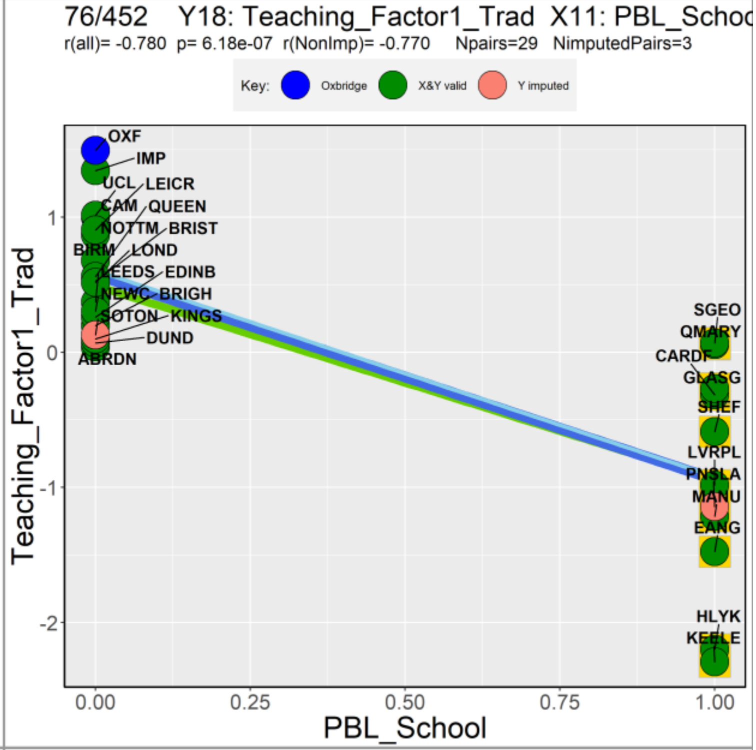
76/452 Y18: Teaching_Factor1_Trad X11: PBL_Schoo
r(all)= -0.780 p= 6.18e-07 r(NonImp)= -0.770 Npairs=29 NimputedPairs=3
Key: Oxbridge X&Y valid Y imputed
Teaching_Factor1_Trad
PBL_School
0.00 0.25 0.50 0.75 1.00
-2 -1 0 1
OXF
IMP
UCL
LEICR
CAM
QUEEN
NOTTM
BRIST
BIRM
LOND
LEEDS
EDINB
NEWC
BRIGH
SOTON
KINGS
DUND
ABRDN
SGEO
QMARY
CARDF
GLASG
SHEF
LVRPL
PNSLA
MANU
EANG
HLYK
KEELE
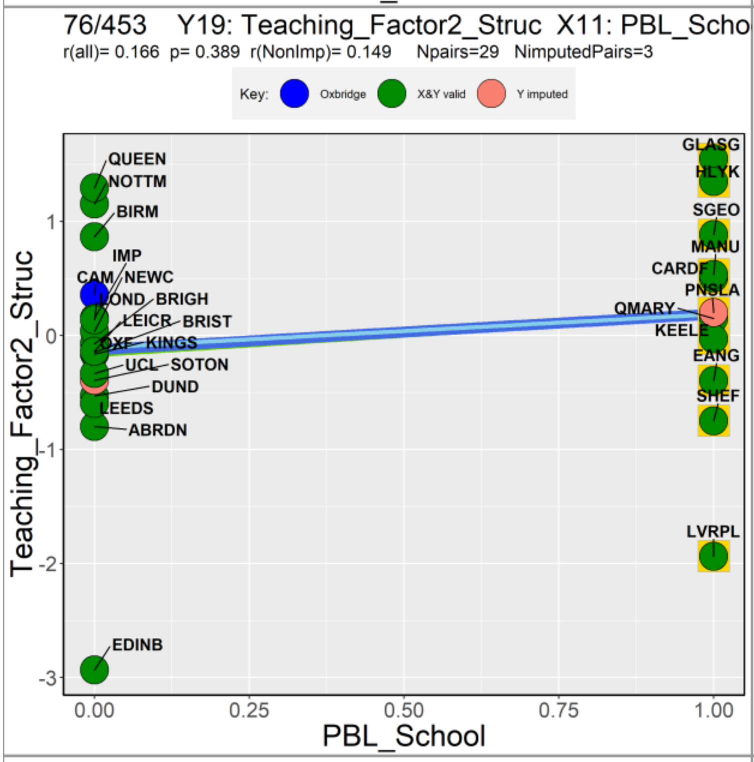
76/453 Y19: Teaching_Factor2_Struc X11: PBL_Scho
r(all)= 0.166 p= 0.389 r(NonImp)= 0.149 Npairs=29 NimputedPairs=3
Key: Oxbridge X&Y valid Y imputed
Teaching_Factor2_Struc
PBL_School
0.00 0.25 0.50 0.75 1.00
-3 -2 -1 0 1
QUEEN
NOTTM
BIRM
IMP
CAM
NEWC
LOND
BRIGH
LEICR
BRIST
OXF
KINGS
UCL
SOTON
DUND
LEEDS
ABRDN
EDINB
GLASG
HLYK
SGEO
MANU
CARDF
PNSLA
QMARY
KEELE
EANG
SHEF
LVRPL
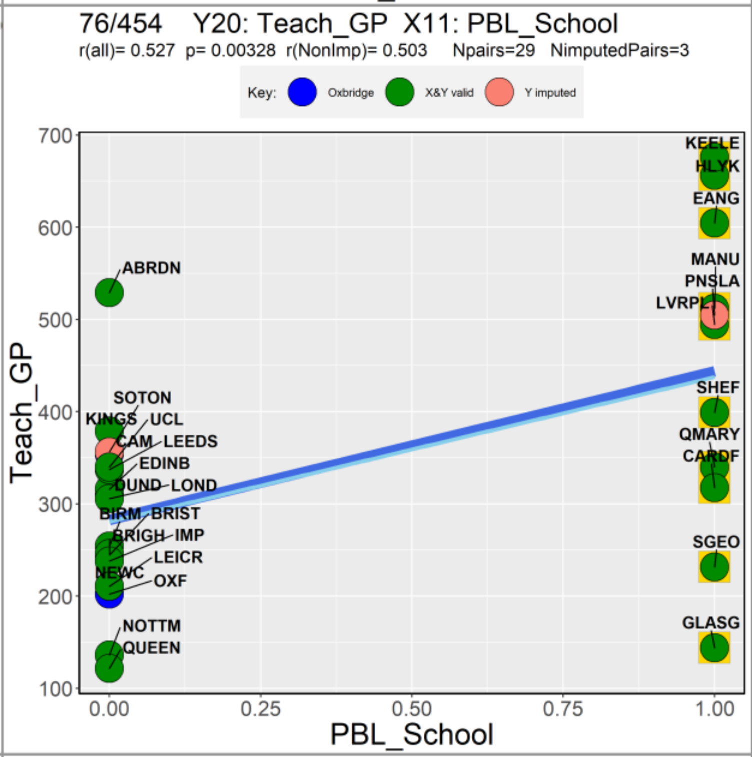
76/454 Y20: Teach_GP X11: PBL_School
r(all)= 0.527 p= 0.00328 r(NonImp)= 0.503 Npairs=29 NimputedPairs=3
Key: Oxbridge X&Y valid Y imputed
Teach_GP
PBL_School
0.00 0.25 0.50 0.75 1.00
100 200 300 400 500 600 700
ABRDN
SOTON
KINGS
UCL
CAM
LEEDS
EDINB
DUND
LOND
BIRM
BRIST
BRIGH
IMP
LEICR
NEWC
OXF
NOTTM
QUEEN
KEELE
HLYK
EANG
MANU
PNSLA
LVRPL
SHEF
QMARY
CARDF
SGEO
GLASG
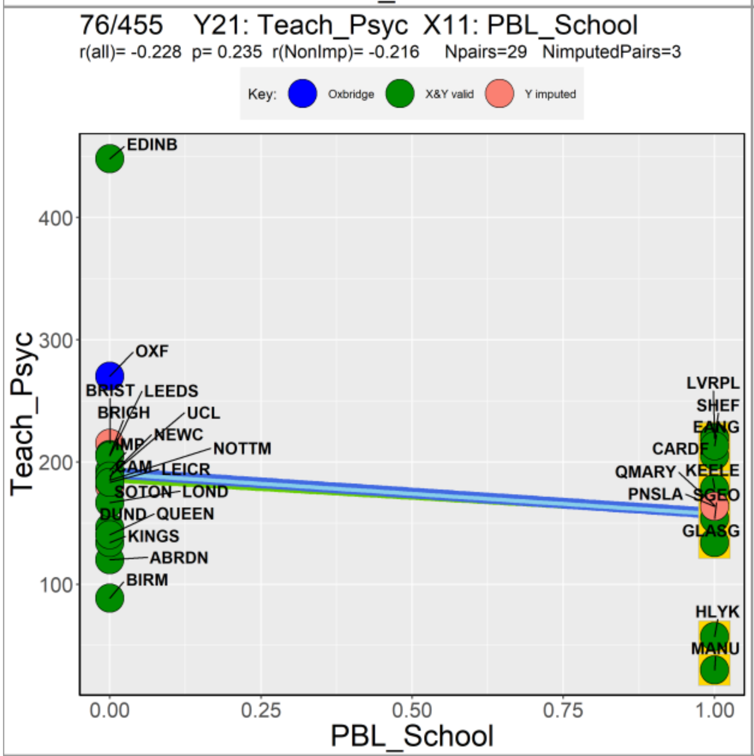
76/455 Y21: Teach_Psyc X11: PBL_School
r(all)= -0.228 p= 0.235 r(NonImp)= -0.216 Npairs=29 NimputedPairs=3
Key: Oxbridge X&Y valid Y imputed
Teach_Psyc
PBL_School
0.00 0.25 0.50 0.75 1.00
100 200 300 400
EDINB
OXF
BRIST
LEEDS
BRIGH
UCL
IMP
NEWC
NOTTM
CAM
LEICR
SOTON
LOND
DUND
QUEEN
KINGS
ABRDN
BIRM
LVRPL
SHEF
EANG
CARDF
QMARY
KEELE
PNSLA
SGEO
GLASG
HLYK
MANU
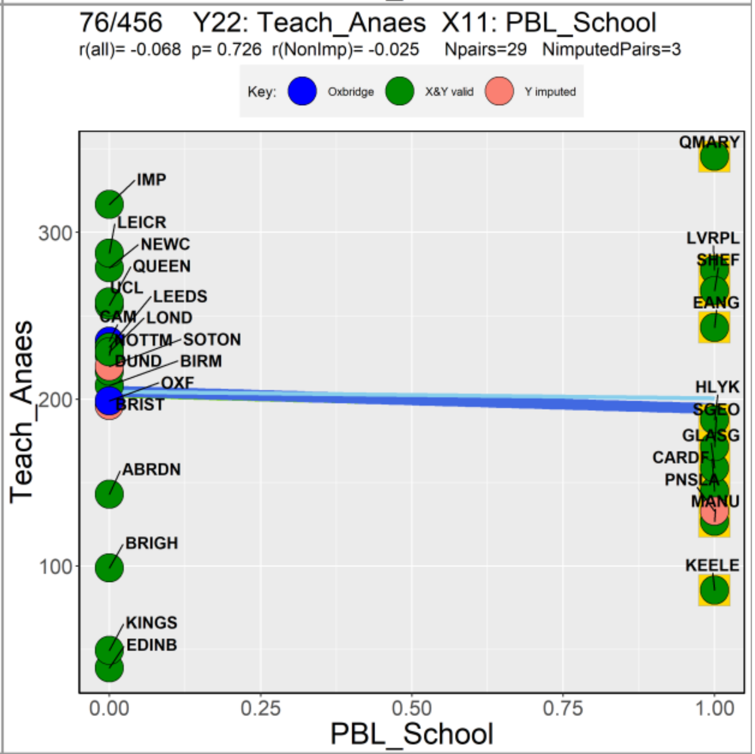
76/456 Y22: Teach_Anaes X11: PBL_School
r(all)= -0.068 p= 0.726 r(NonImp)= -0.025 Npairs=29 NimputedPairs=3
Key: Oxbridge X&Y valid Y imputed
Teach_Anaes
PBL_School
0.00 0.25 0.50 0.75 1.00
100 200 300
IMP
LEICR
NEWC
QUEEN
UCL
LEEDS
CAM
LOND
NOTTM
SOTON
DUND
BIRM
OXF
BRIST
ABRDN
BRIGH
KINGS
EDINB
QMARY
LVRPL
SHEF
EANG
HLYK
SGEO
GLASG
CARDF
PNSLA
MANU
KEELE

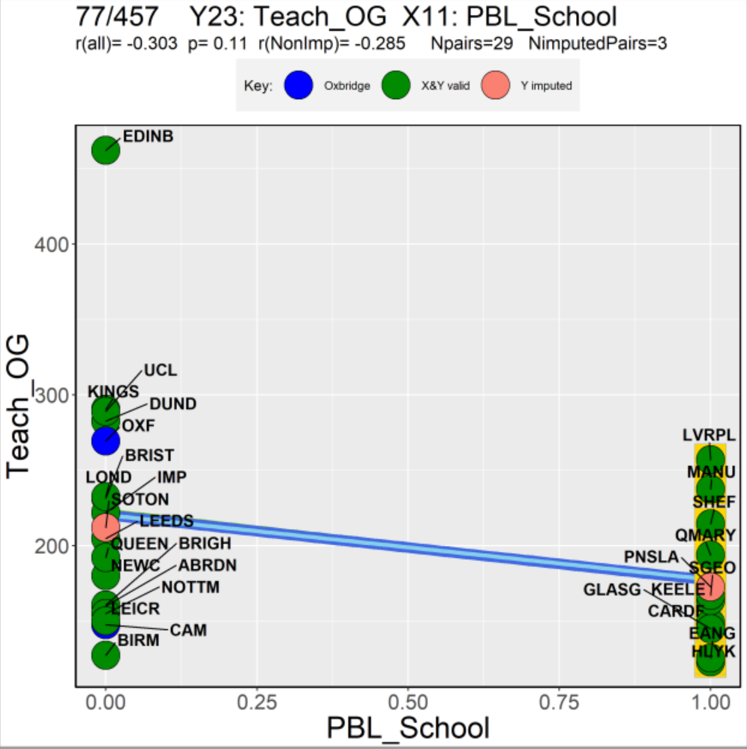
77/457 Y23: Teach_OG X11: PBL_School
r(all)= -0.303 p= 0.11 r(NonImp)= -0.285 Npairs=29 NimputedPairs=3
Key: Oxbridge X&Y valid Y imputed
Teach_OG
PBL_School
400
300
200
0.00
0.25
0.50
0.75
1.00

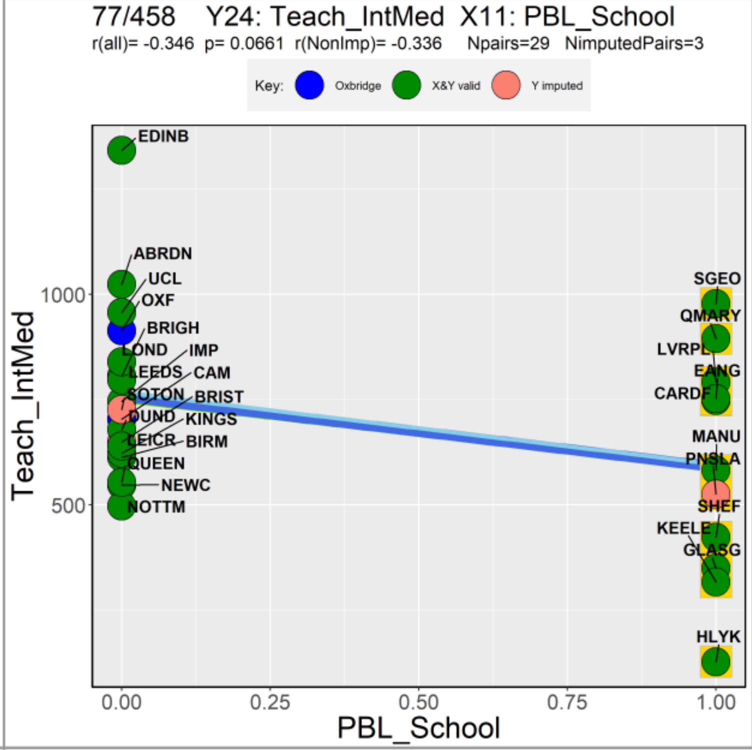
77/458 Y24: Teach_IntMed X11: PBL_School
r(all)= -0.346 p= 0.0661 r(NonImp)= -0.336 Npairs=29 NimputedPairs=3
Key: Oxbridge X&Y valid Y imputed
Teach_IntMed
PBL_School
1000
500
0.00
0.25
0.50
0.75
1.00

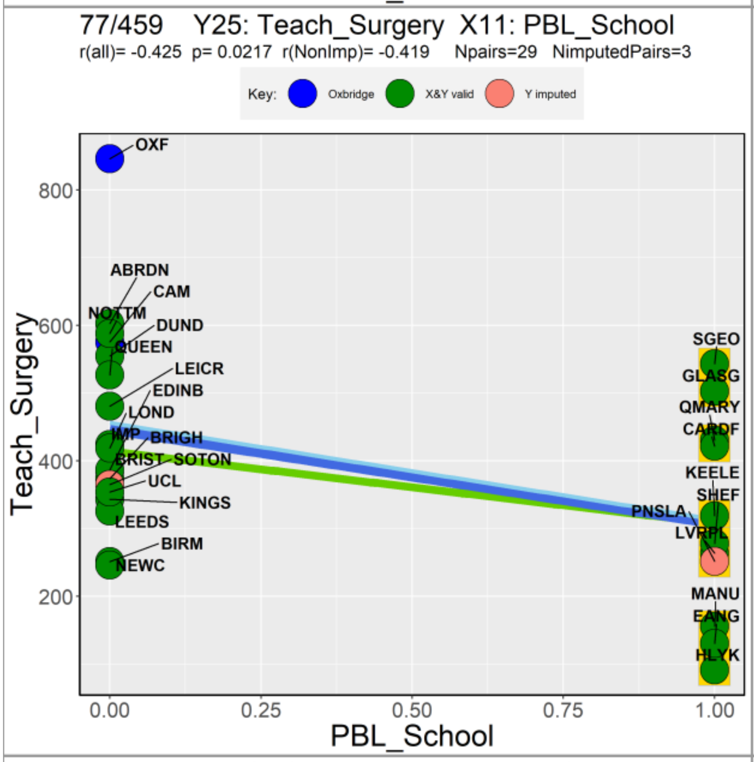
77/459 Y25: Teach_Surgery X11: PBL_School
r(all)= -0.425 p= 0.0217 r(NonImp)= -0.419 Npairs=29 NimputedPairs=3
Key: Oxbridge X&Y valid Y imputed
Teach_Surgery
PBL_School
800
600
400
200
0.00
0.25
0.50
0.75
1.00

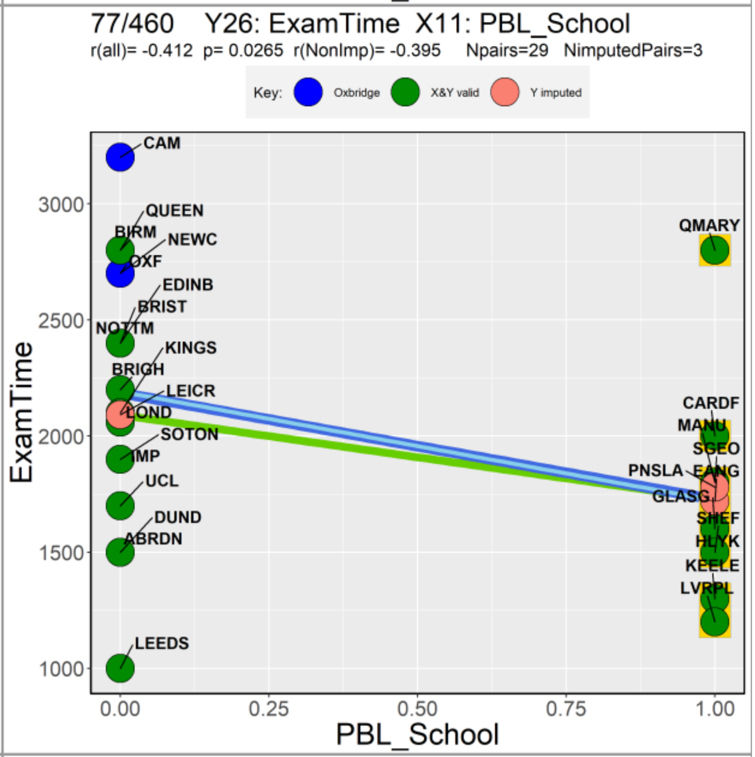
77/460 Y26: ExamTime X11: PBL_School
r(all)= -0.412 p= 0.0265 r(NonImp)= -0.395 Npairs=29 NimputedPairs=3
Key: Oxbridge X&Y valid Y imputed
ExamTime
PBL_School
3000
2500
2000
1500
1000
0.00
0.25
0.50
0.75
1.00

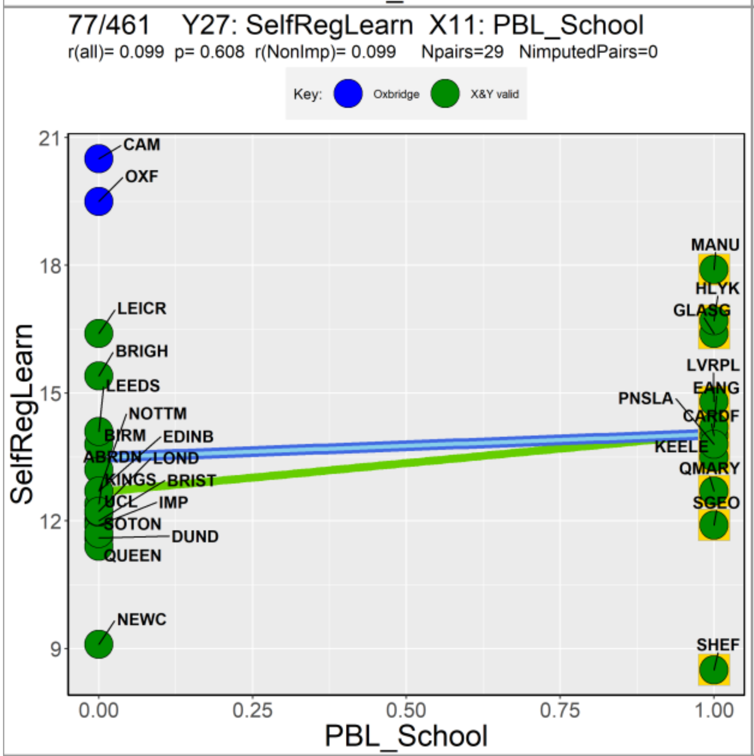
77/461 Y27: SelfRegLearn X11: PBL_School
r(all)= 0.099 p= 0.608 r(NonImp)= 0.099 Npairs=29 NimputedPairs=0
Key: Oxbridge X&Y valid
SelfRegLearn
PBL_School
21
18
15
12
9
0.00
0.25
0.50
0.75
1.00

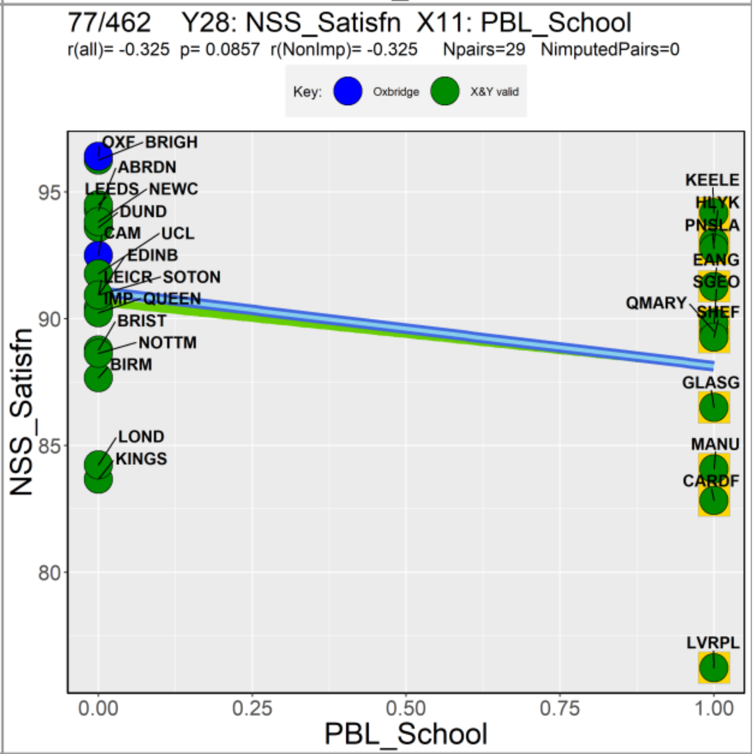
77/462 Y28: NSS_Satisfn X11: PBL_School
r(all)= -0.325 p= 0.0857 r(NonImp)= -0.325 Npairs=29 NimputedPairs=0
Key: Oxbridge X&Y valid
NSS_Satisfn
PBL_School
95
90
85
80
0.00
0.25
0.50
0.75
1.00

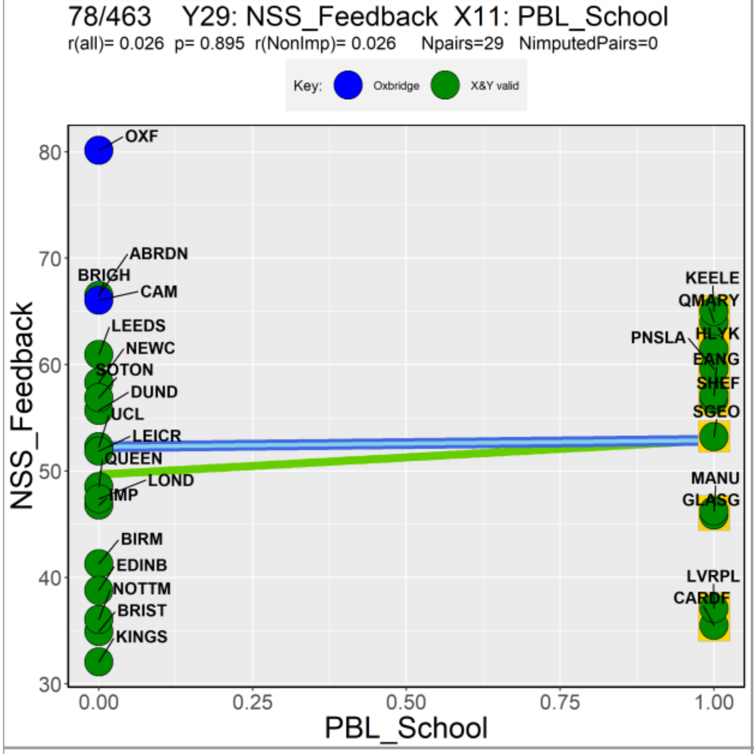
78/463 Y29: NSS_Feedback X11: PBL_School
r(all)= 0.026 p= 0.895 r(NonImp)= 0.026 Npairs=29 NimputedPairs=0
Key: Oxbridge X&Y valid
NSS_Feedback
PBL_School
30 40 50 60 70 80
0.00 0.25 0.50 0.75 1.00
OXF ABRDN BRIGH CAM LEEDS NEWC SOTON DUND UCL LEICR QUEEN LOND IMP BIRM EDINB NOTTM BRIST KINGS
KEELE QMARY PNSLA HLYK EANG SHEF SGEO MANU GLASG LVRPL CARDF

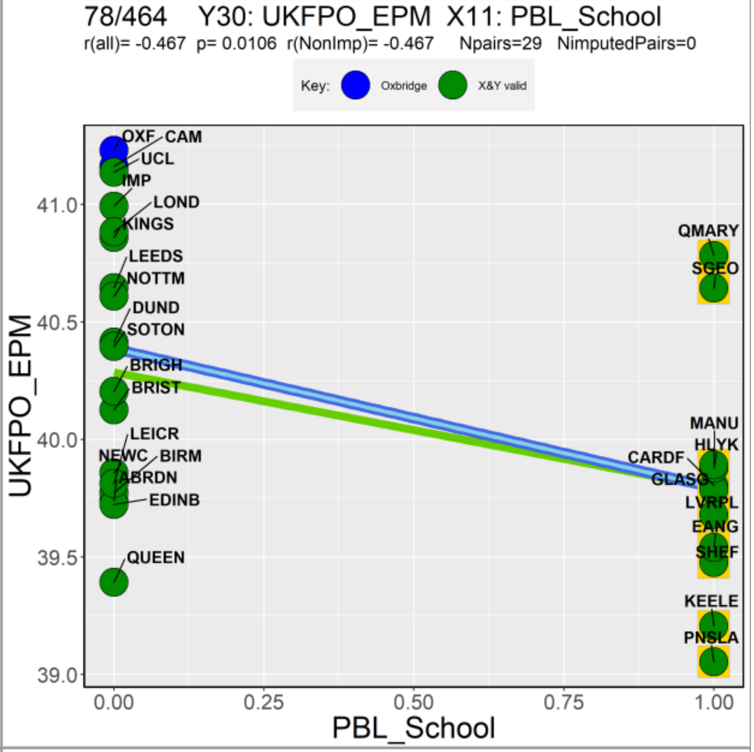
78/464 Y30: UKFPO_EPM X11: PBL_School
r(all)= -0.467 p= 0.0106 r(NonImp)= -0.467 Npairs=29 NimputedPairs=0
Key: Oxbridge X&Y valid
UKFPO_EPM
PBL_School
39.0 39.5 40.0 40.5 41.0
0.00 0.25 0.50 0.75 1.00
OXF CAM UCL IMP LOND KINGS LEEDS NOTTM DUND SOTON BRIGH BRIST LEICR NEWC BIRM ABRDN EDINB QUEEN
QMARY SGEO MANU HLYK CARDF GLASG LVRPL EANG SHEF KEELE PNSLA

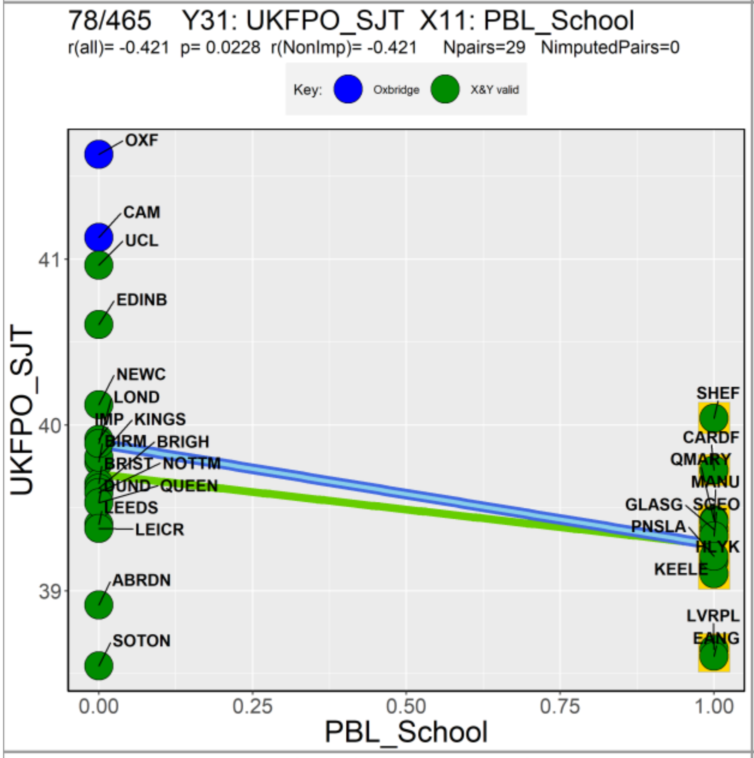
78/465 Y31: UKFPO_SJT X11: PBL_School
r(all)= -0.421 p= 0.0228 r(NonImp)= -0.421 Npairs=29 NimputedPairs=0
Key: Oxbridge X&Y valid
UKFPO_SJT
PBL_School
39 40 41
0.00 0.25 0.50 0.75 1.00
OXF CAM UCL EDINB NEWC LOND IMP KINGS BIRM BRIGH BRIST NOTTM DUND QUEEN LEEDS LEICR ABRDN SOTON
SHEF CARDF QMARY MANU GLASG SGEO PNSLA HLYK KEELE LVRPL EANG

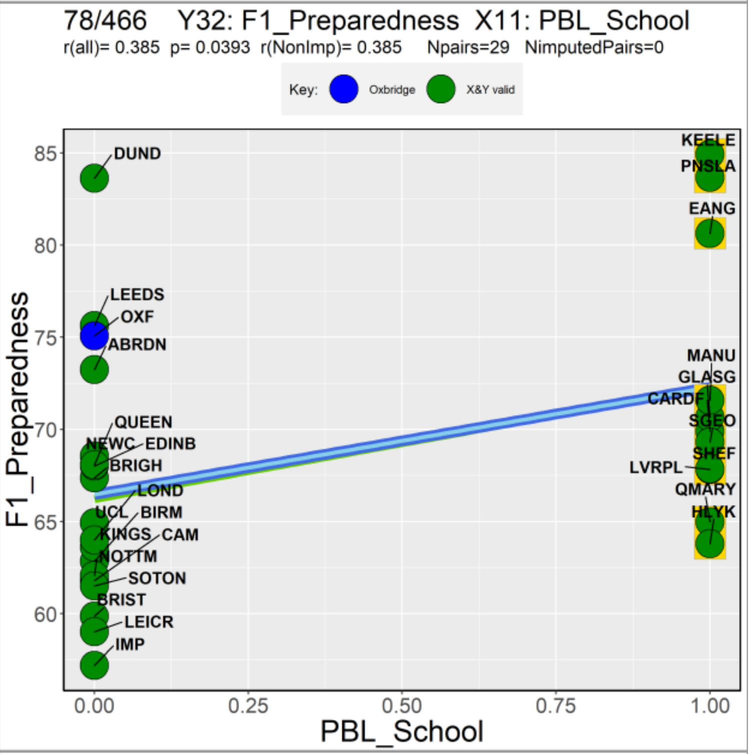
78/466 Y32: F1_Preparedness X11: PBL_School
r(all)= 0.385 p= 0.0393 r(NonImp)= 0.385 Npairs=29 NimputedPairs=0
Key: Oxbridge X&Y valid
F1_Preparedness
PBL_School
60 65 70 75 80 85
0.00 0.25 0.50 0.75 1.00
DUND LEEDS OXF ABRDN QUEEN NEWC EDINB BRIGH LOND UCL BIRM KINGS CAM NOTTM SOTON BRIST LEICR IMP
KEELE PNSLA EANG MANU GLASG CARDF SGEO SHEF LVRPL QMARY HLYK

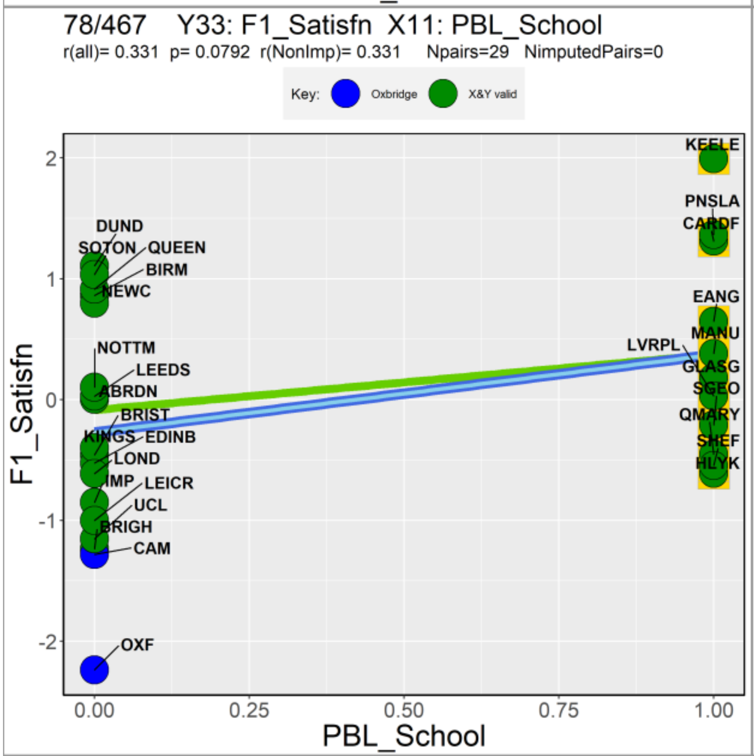
78/467 Y33: F1_Satisfn X11: PBL_School
r(all)= 0.331 p= 0.0792 r(NonImp)= 0.331 Npairs=29 NimputedPairs=0
Key: Oxbridge X&Y valid
F1_Satisfn
PBL_School
-2 -1 0 1 2
0.00 0.25 0.50 0.75 1.00
DUND SOTON QUEEN BIRM NEWC NOTTM LEEDS ABRDN BRIST KINGS EDINB LOND IMP LEICR UCL BRIGH CAM OXF
KEELE PNSLA CARDF EANG LVRPL MANU GLASG SGEO QMARY SHEF HLYK

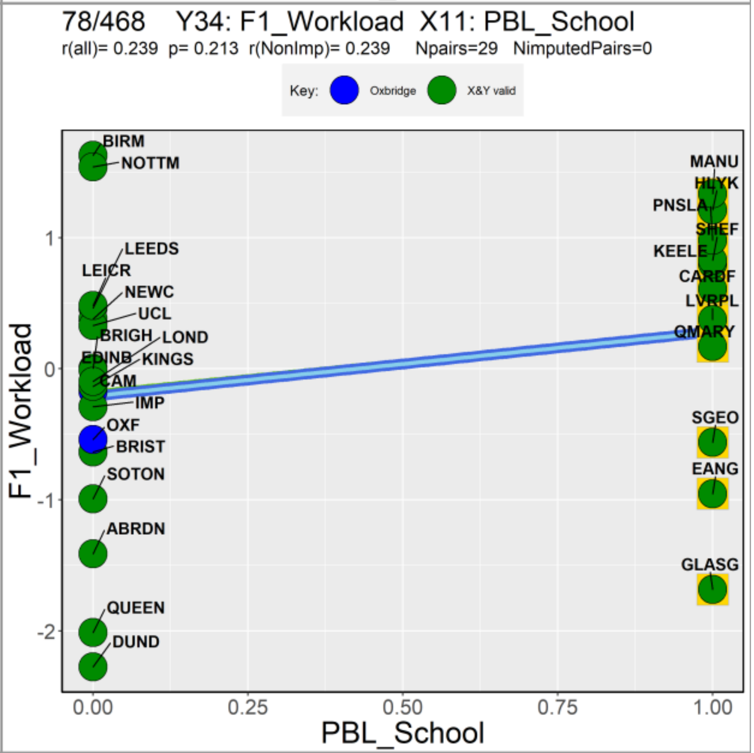
78/468 Y34: F1_Workload X11: PBL_School
r(all)= 0.239 p= 0.213 r(NonImp)= 0.239 Npairs=29 NimputedPairs=0
Key: Oxbridge X&Y valid
F1_Workload
PBL_School
-2 -1 0 1
0.00 0.25 0.50 0.75 1.00
BIRM NOTTM LEEDS LEICR NEWC UCL BRIGH LOND EDINB KINGS CAM IMP OXF BRIST SOTON ABRDN QUEEN DUND
MANU HLYK PNSLA SHEF KEELE CARDF LVRPL QMARY SGEO EANG GLASG

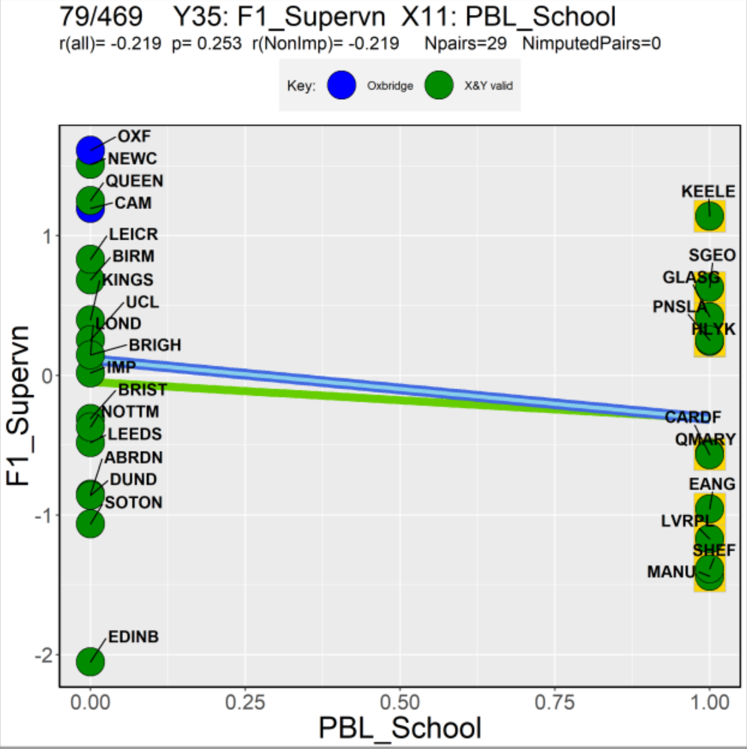
79/469 Y35: F1_Supervn X11: PBL_School
r(all)= -0.219 p= 0.253 r(NonImp)= -0.219 Npairs=29 NimputedPairs=0
Key: Oxbridge X&Y valid
OXF
NEWC
QUEEN
CAM
LEICR
BIRM
KINGS
UCL
LOND
BRIGH
IMP
BRIST
NOTTM
LEEDS
ABRDN
DUND
SOTON
EDINB
KEELE
SGEO
GLASG
PNSLA
HLYK
CARDF
QMARY
EANG
LVRPL
SHEF
MANU
F1_Supervn
PBL_School
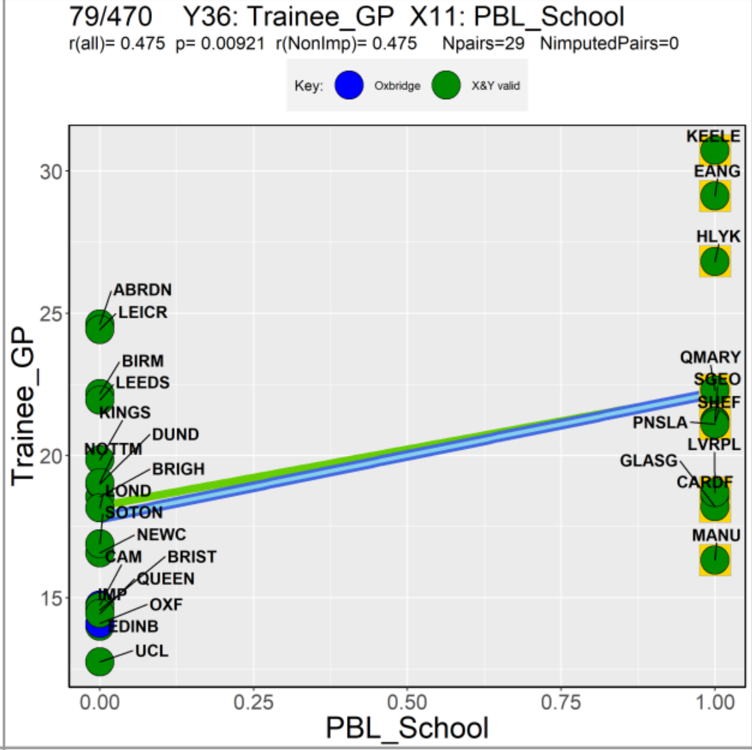
79/470 Y36: Trainee_GP X11: PBL_School
r(all)= 0.475 p= 0.00921 r(NonImp)= 0.475 Npairs=29 NimputedPairs=0
Key: Oxbridge X&Y valid
ABRDN
LEICR
BIRM
LEEDS
KINGS
DUND
NOTTM
BRIGH
LOND
SOTON
NEWC
CAM
BRIST
QUEEN
IMP
OXF
EDINB
UCL
KEELE
EANG
HLYK
QMARY
SGEO
SHEF
PNSLA
LVRPL
GLASG
CARDF
MANU
Trainee_GP
PBL_School
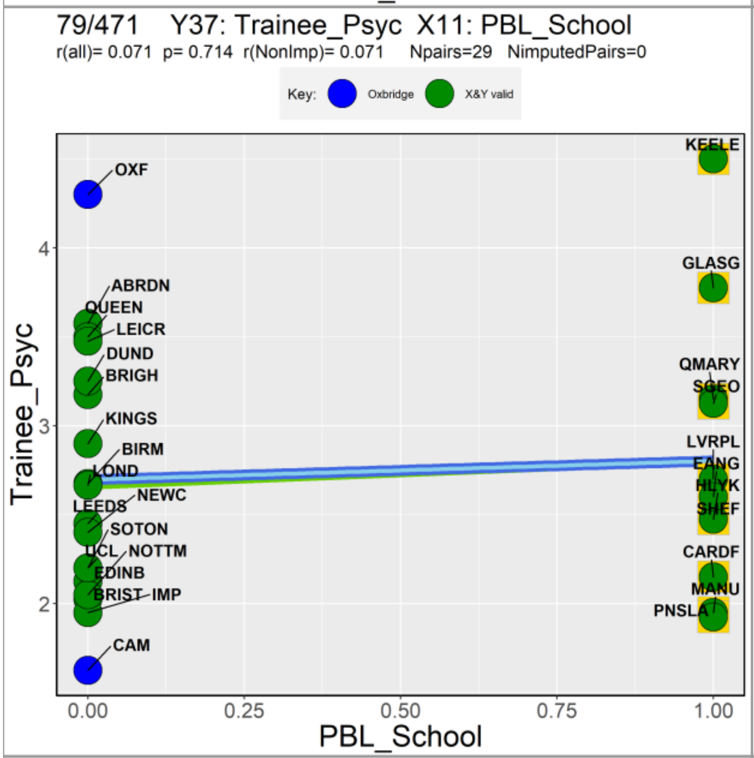
79/471 Y37: Trainee_Psyc X11: PBL_School
r(all)= 0.071 p= 0.714 r(NonImp)= 0.071 Npairs=29 NimputedPairs=0
Key: Oxbridge X&Y valid
OXF
ABRDN
QUEEN
LEICR
DUND
BRIGH
KINGS
BIRM
LOND
NEWC
LEEDS
SOTON
UCL
NOTTM
EDINB
BRIST
IMP
CAM
KEELE
GLASG
QMARY
SGEO
LVRPL
EANG
HLYK
SHEF
CARDF
MANU
PNSLA
Trainee_Psyc
PBL_School
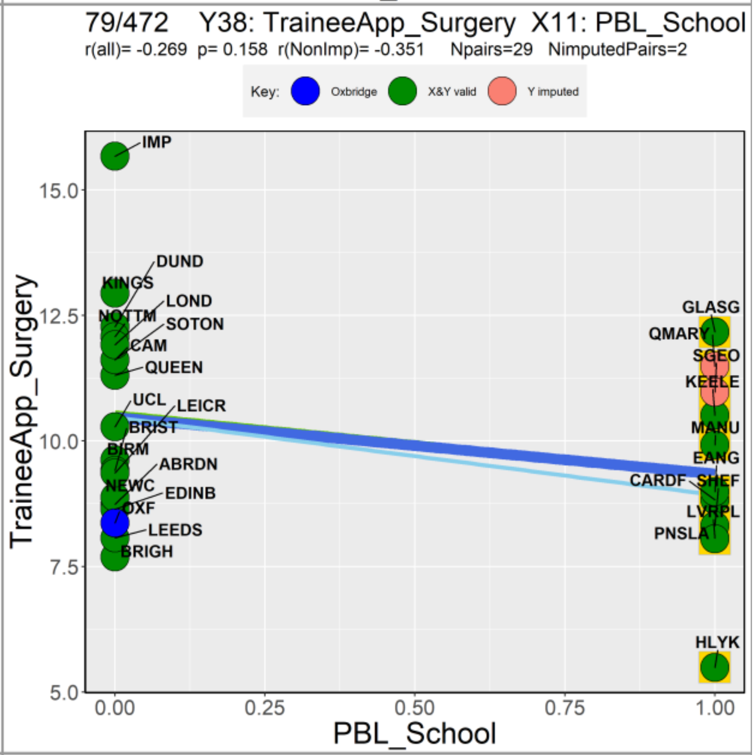
79/472 Y38: TraineeApp_Surgery X11: PBL_School
r(all)= -0.269 p= 0.158 r(NonImp)= -0.351 Npairs=29 NimputedPairs=2
Key: Oxbridge X&Y valid Y imputed
IMP
DUND
KINGS
LOND
NOTTM
SOTON
CAM
QUEEN
UCL
LEICR
BRIST
BIRM
ABRDN
NEWC
EDINB
OXF
LEEDS
BRIGH
GLASG
QMARY
SGEO
KEELE
MANU
EANG
CARDF
SHEF
LVRPL
PNSLA
HLYK
TraineeApp_Surgery
PBL_School
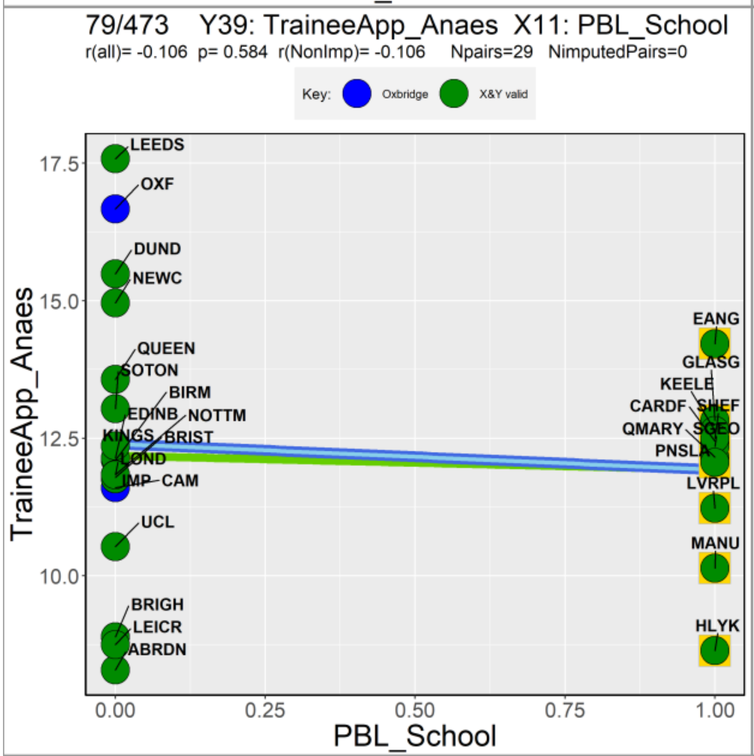
79/473 Y39: TraineeApp_Anaes X11: PBL_School
r(all)= -0.106 p= 0.584 r(NonImp)= -0.106 Npairs=29 NimputedPairs=0
Key: Oxbridge X&Y valid
LEEDS
OXF
DUND
NEWC
QUEEN
SOTON
BIRM
EDINB
NOTTM
KINGS
BRIST
LOND
IMP
CAM
UCL
BRIGH
LEICR
ABRDN
EANG
GLASG
KEELE
CARDF
SHEF
QMARY
SGEO
PNSLA
LVRPL
MANU
HLYK
TraineeApp_Anaes
PBL_School
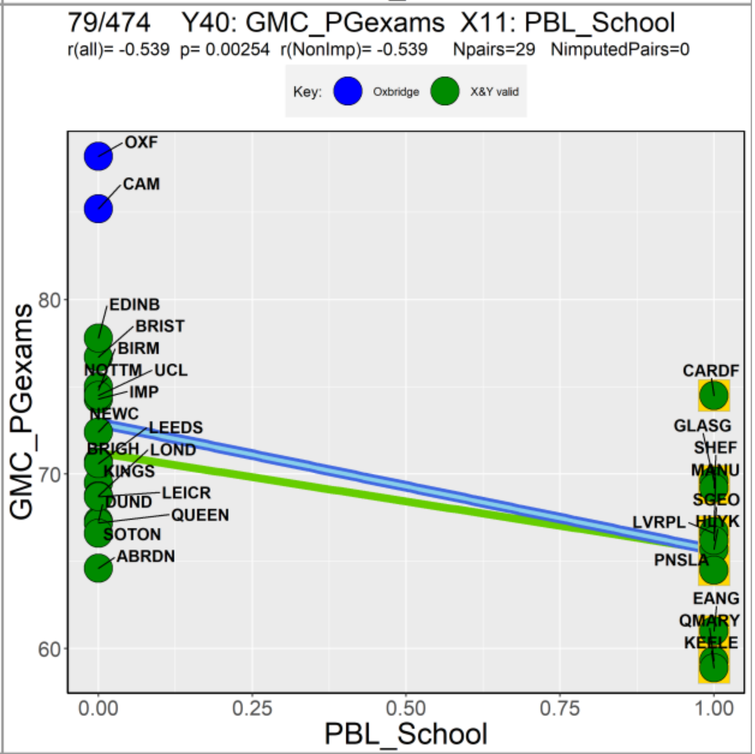
79/474 Y40: GMC_PGexams X11: PBL_School
r(all)= -0.539 p= 0.00254 r(NonImp)= -0.539 Npairs=29 NimputedPairs=0
Key: Oxbridge X&Y valid
OXF
CAM
EDINB
BRIST
BIRM
NOTTM
UCL
IMP
NEWC
LEEDS
BRIGH
LOND
KINGS
LEICR
DUND
QUEEN
SOTON
ABRDN
CARDF
GLASG
SHEF
MANU
SGEO
LVRPL
HLYK
PNSLA
EANG
QMARY
KEELE
GMC_PGexams
PBL_School

80/475 Y41: MRCGP_AKT X11: PBL_School

r(all)= -0.522 p= 0.00365 r(NonImp)= -0.522 Npairs=29 NimputedPairs=0

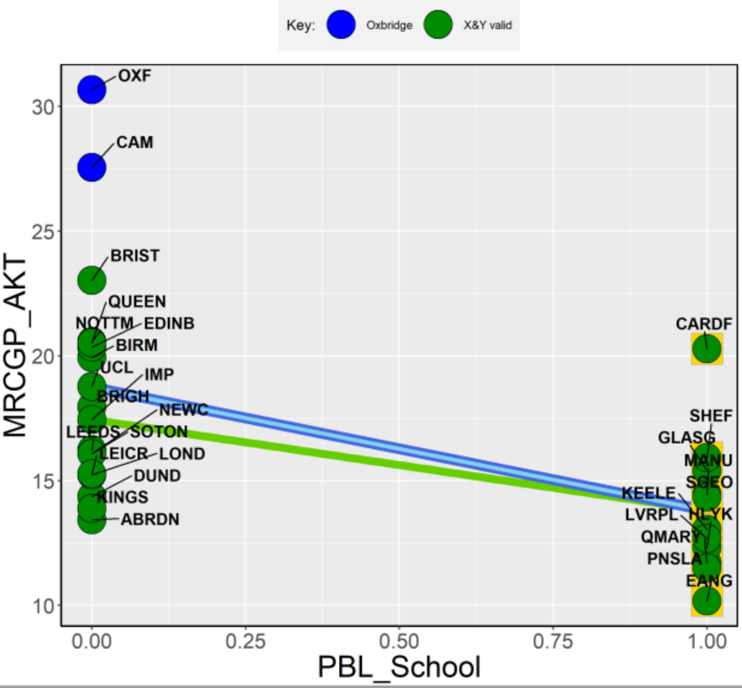

80/476 Y42: MRCGP_CSA X11: PBL_School

r(all)= -0.416 p= 0.0247 r(NonImp)= -0.416 Npairs=29 NimputedPairs=0

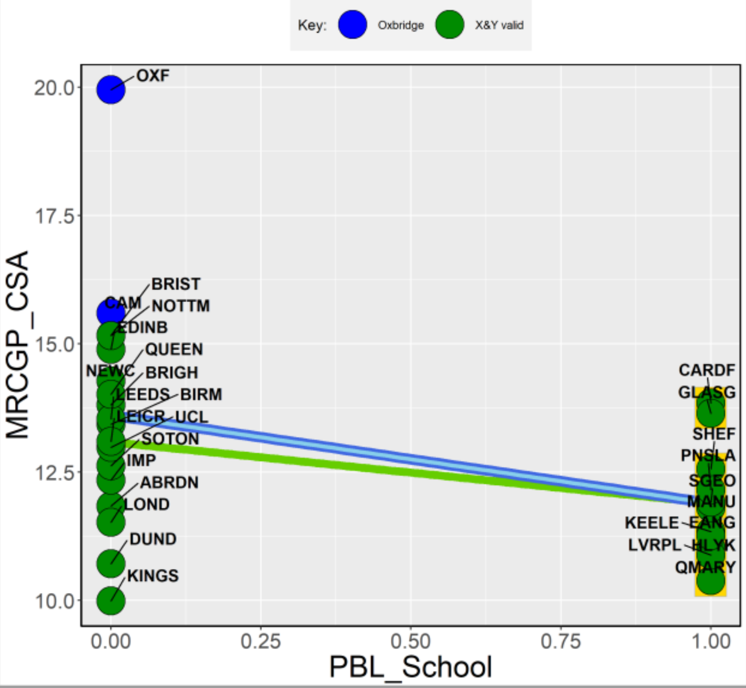

80/477 Y43: FRCA_Pt1 X11: PBL_School

r(all)= -0.114 p= 0.557 r(NonImp)= -0.005 Npairs=29 NimputedPairs=10

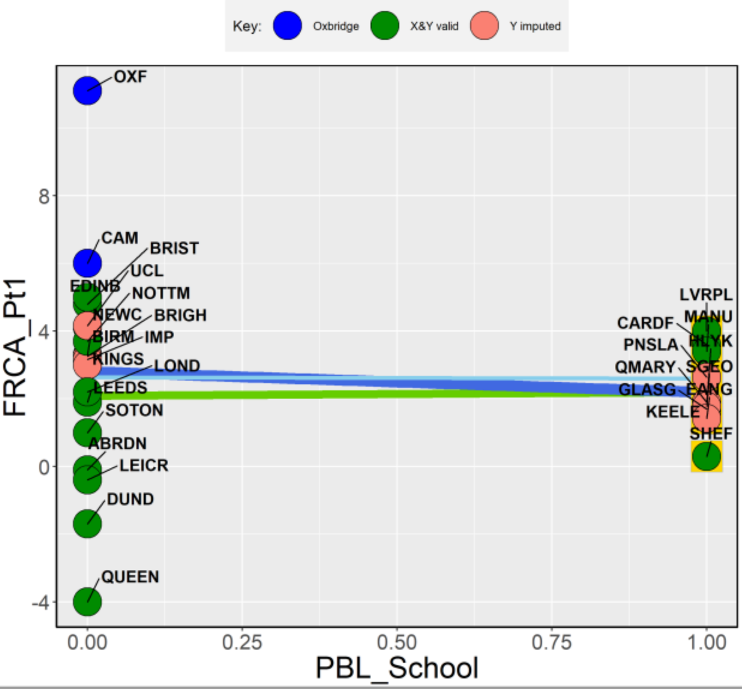

80/478 Y44: MRCOG_Pt1 X11: PBL_School

r(all)= -0.372 p= 0.0466 r(NonImp)= -0.321 Npairs=29 NimputedPairs=10

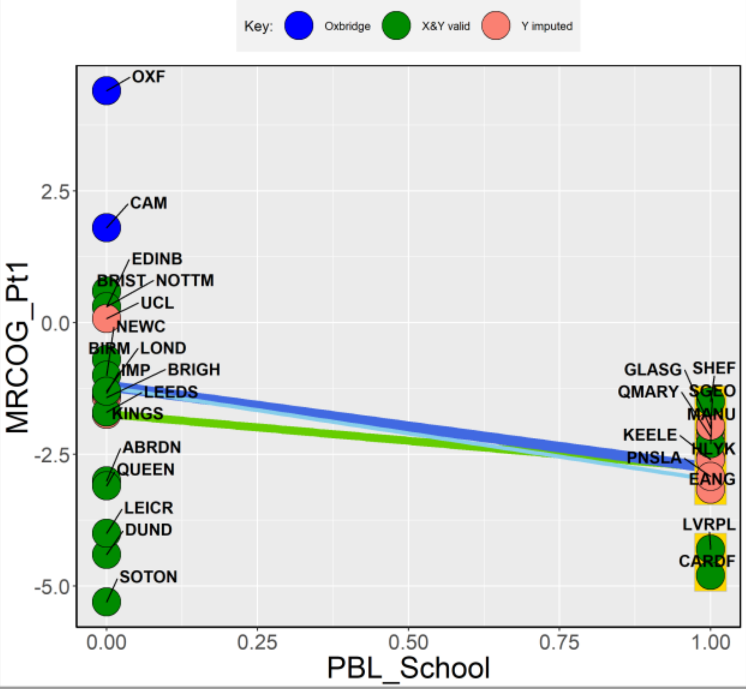

80/479 Y45: MRCOG_Pt2 X11: PBL_School

r(all)= -0.428 p= 0.0207 r(NonImp)= -0.364 Npairs=29 NimputedPairs=10

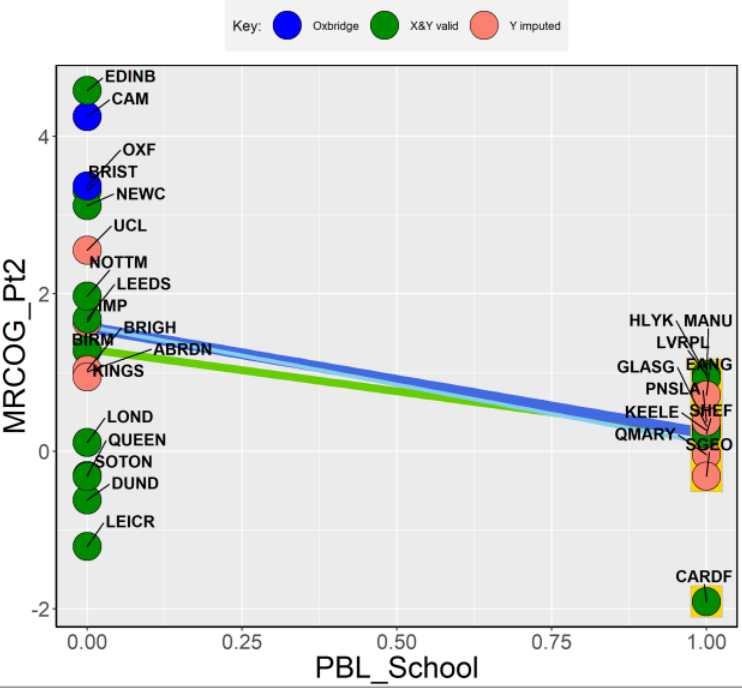

80/480 Y46: MRCP_Pt1 X11: PBL_School

r(all)= -0.317 p= 0.0941 r(NonImp)= -0.352 Npairs=29 NimputedPairs=3

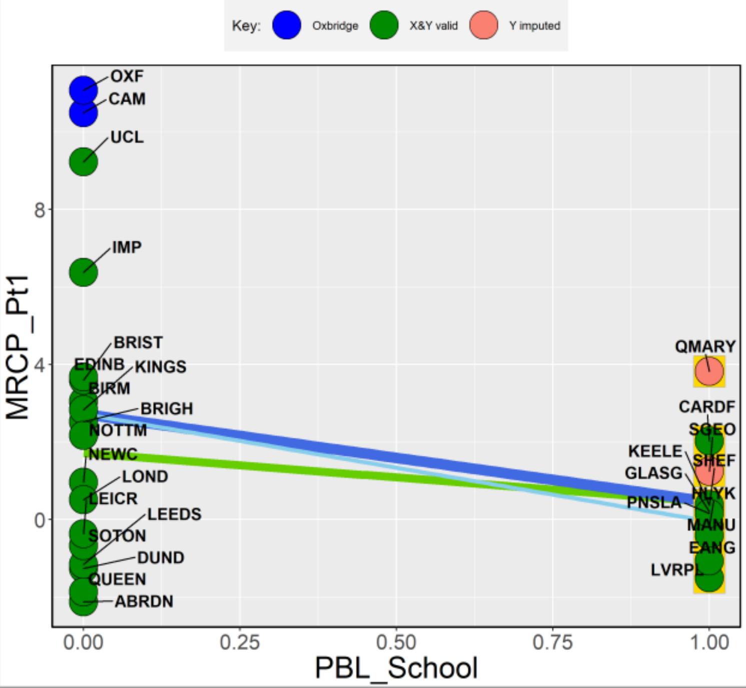

81/481 Y47: MRCP_Pt2 X11: PBL_School
r(all)= -0.257 p= 0.178 r(NonImp)= -0.240 Npairs=29 NimputedPairs=3

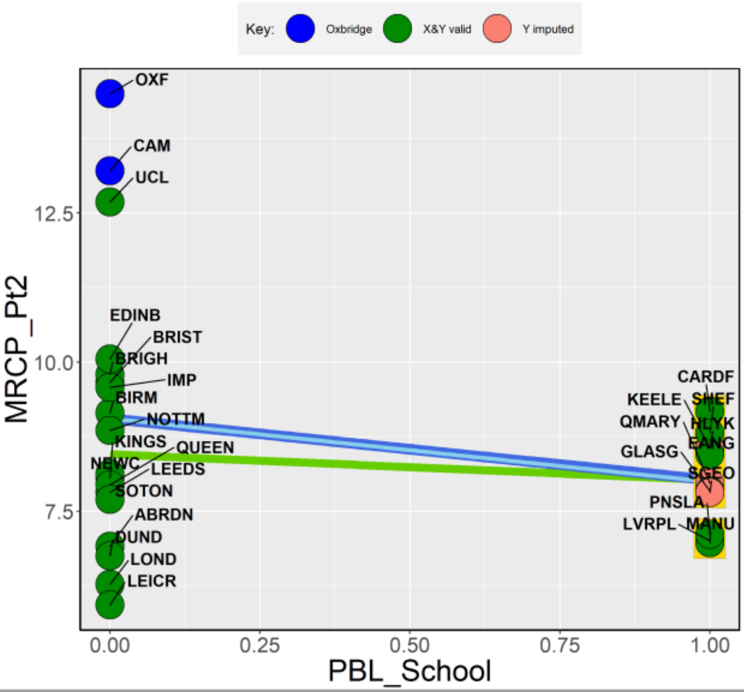

81/482 Y48: MRCP_PACES X11: PBL_School
r(all)= -0.178 p= 0.356 r(NonImp)= -0.142 Npairs=29 NimputedPairs=4

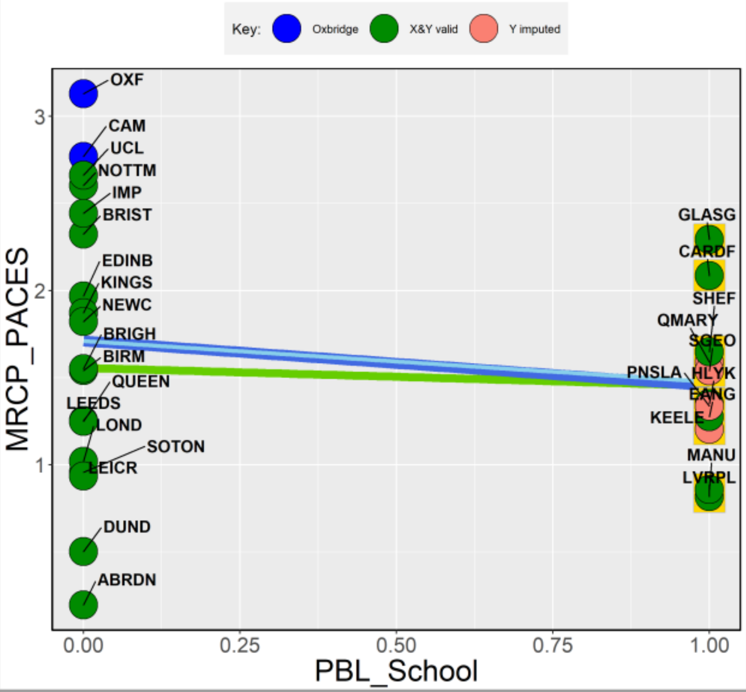

81/483 Y49: GMC_Sanctions X11: PBL_School
r(all)= 0.460 p= 0.012 r(NonImp)= 0.332 Npairs=29 NimputedPairs=10

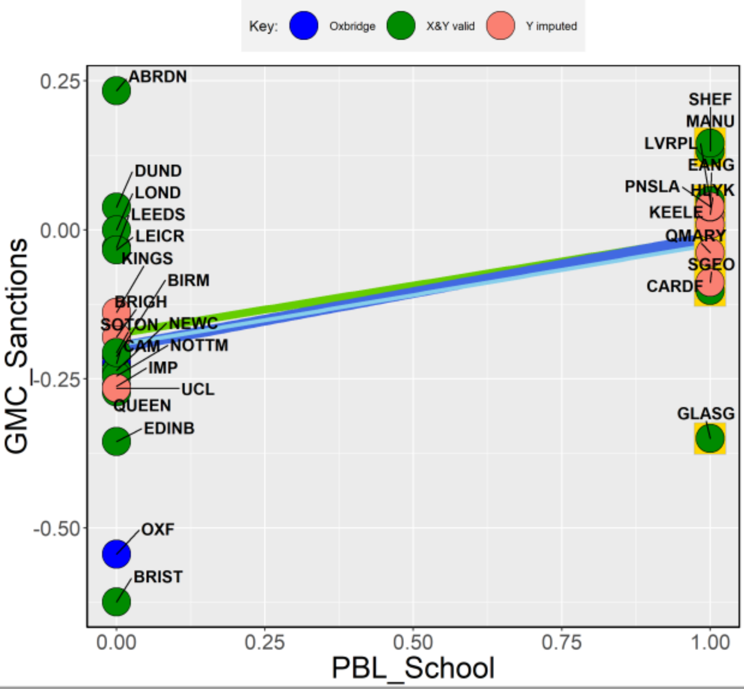

81/484 Y50: ARCP_NotExam X11: PBL_School
r(all)= 0.480 p= 0.00836 r(NonImp)= 0.468 Npairs=29 NimputedPairs=1

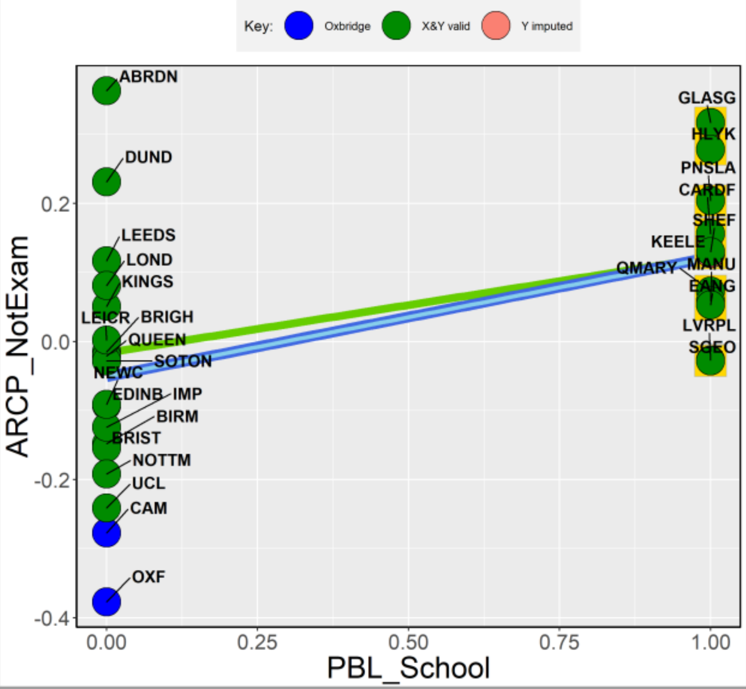

81/485 Y13: Student_Staff X12: Spend_Student
r(all)= -0.351 p= 0.0616 r(NonImp)= -0.351 Npairs=29 NimputedPairs=0

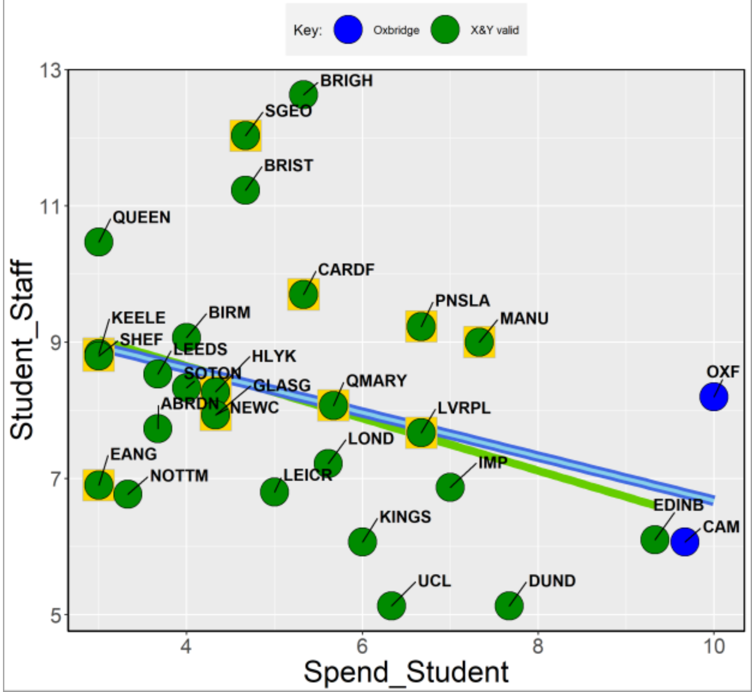

81/486 Y14: Entrants_N X12: Spend_Student
r(all)= 0.090 p= 0.641 r(NonImp)= 0.090 Npairs=29 NimputedPairs=0

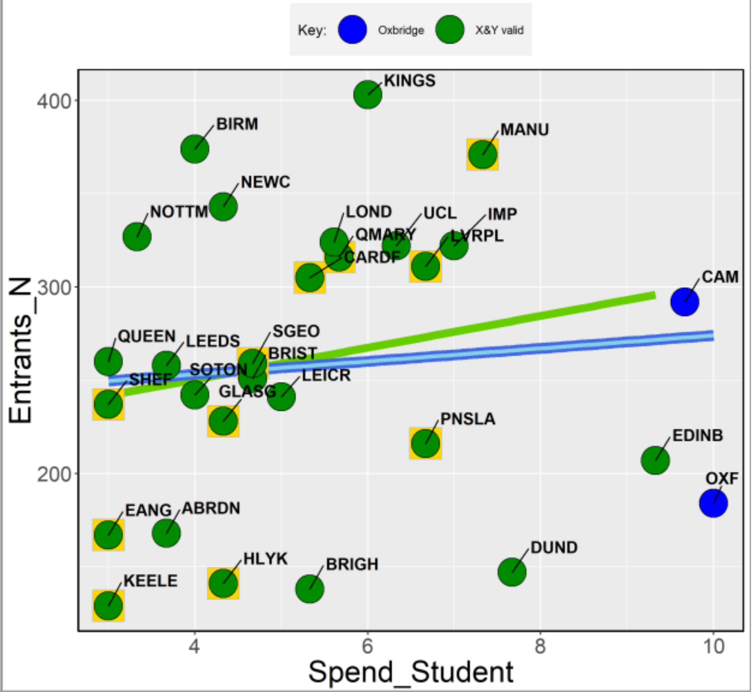

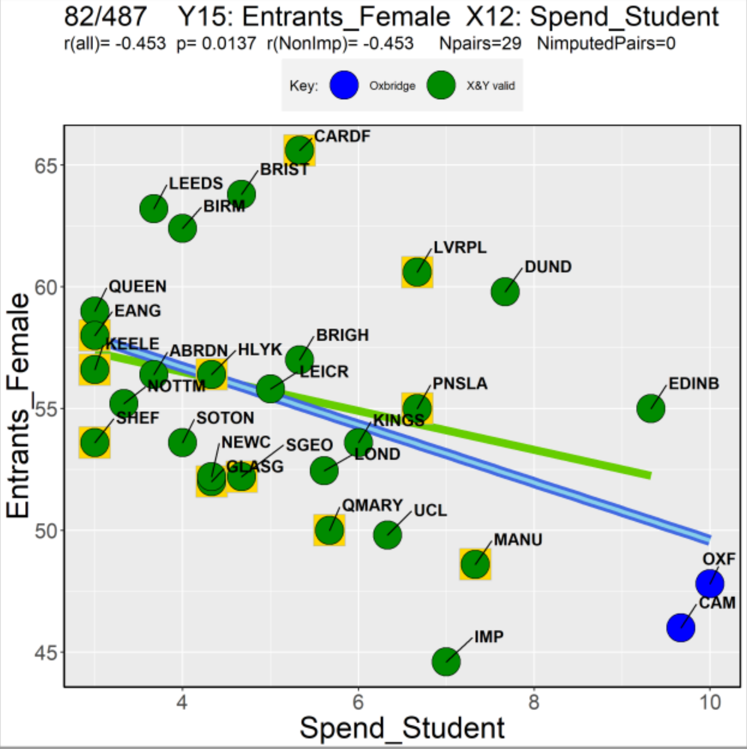
82/487 Y15: Entrants_Female X12: Spend_Student
r(all)= -0.453 p= 0.0137 r(NonImp)= -0.453 Npairs=29 NimputedPairs=0
Key: Oxbridge X&Y valid
Entrants_Female
Spend_Student

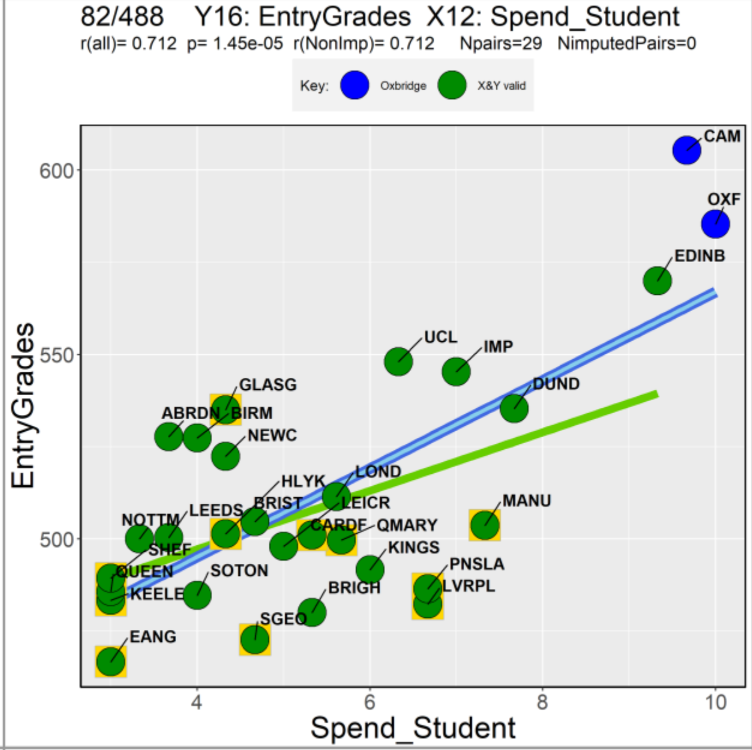
82/488 Y16: EntryGrades X12: Spend_Student
r(all)= 0.712 p= 1.45e-05 r(NonImp)= 0.712 Npairs=29 NimputedPairs=0
Key: Oxbridge X&Y valid
EntryGrades
Spend_Student

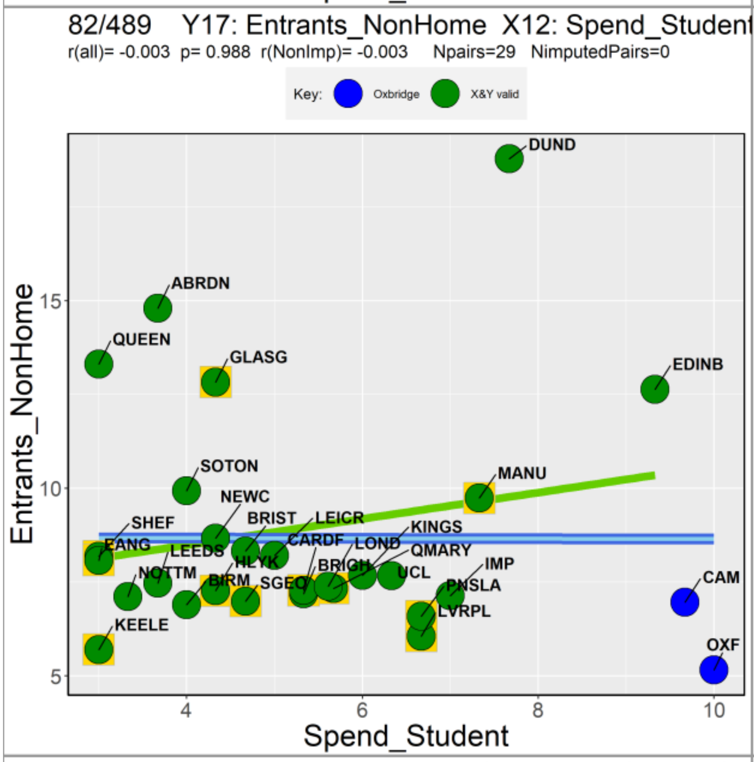
82/489 Y17: Entrants_NonHome X12: Spend_Student
r(all)= -0.003 p= 0.988 r(NonImp)= -0.003 Npairs=29 NimputedPairs=0
Key: Oxbridge X&Y valid
Entrants_NonHome
Spend_Student

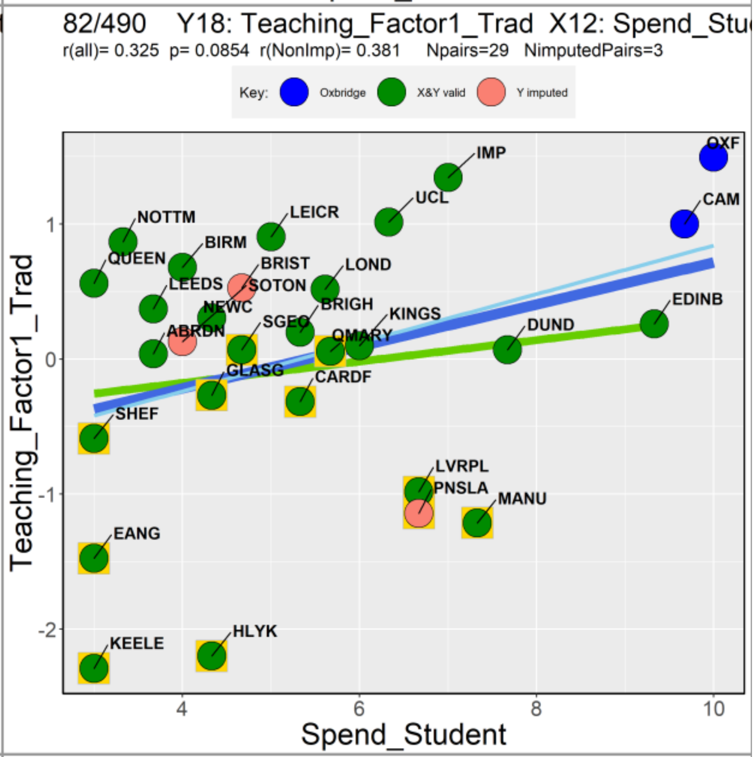
82/490 Y18: Teaching_Factor1_Trad X12: Spend_Stu
r(all)= 0.325 p= 0.0854 r(NonImp)= 0.381 Npairs=29 NimputedPairs=3
Key: Oxbridge X&Y valid Y imputed
Teaching_Factor1_Trad
Spend_Student

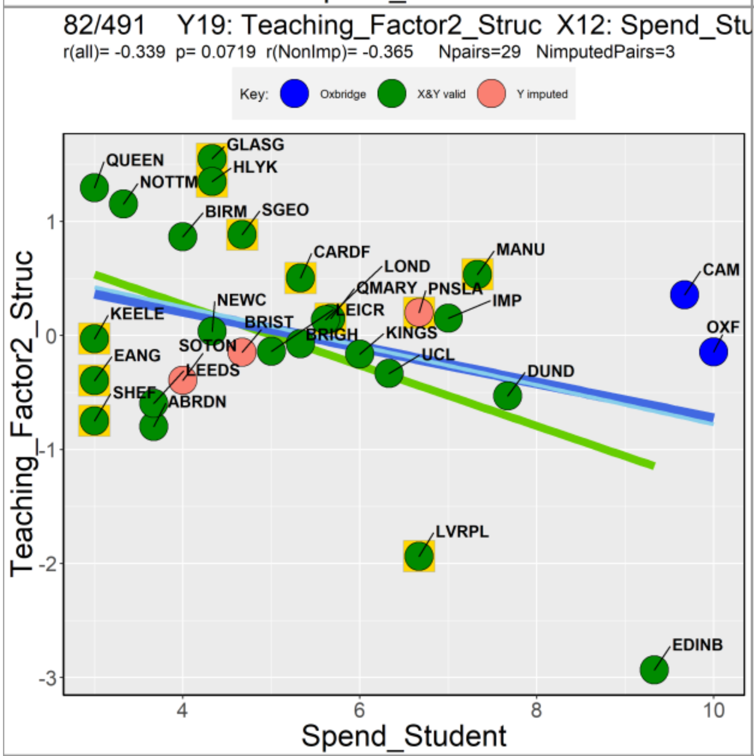
82/491 Y19: Teaching_Factor2_Struc X12: Spend_Stu
r(all)= -0.339 p= 0.0719 r(NonImp)= -0.365 Npairs=29 NimputedPairs=3
Key: Oxbridge X&Y valid Y imputed
Teaching_Factor2_Struc
Spend_Student

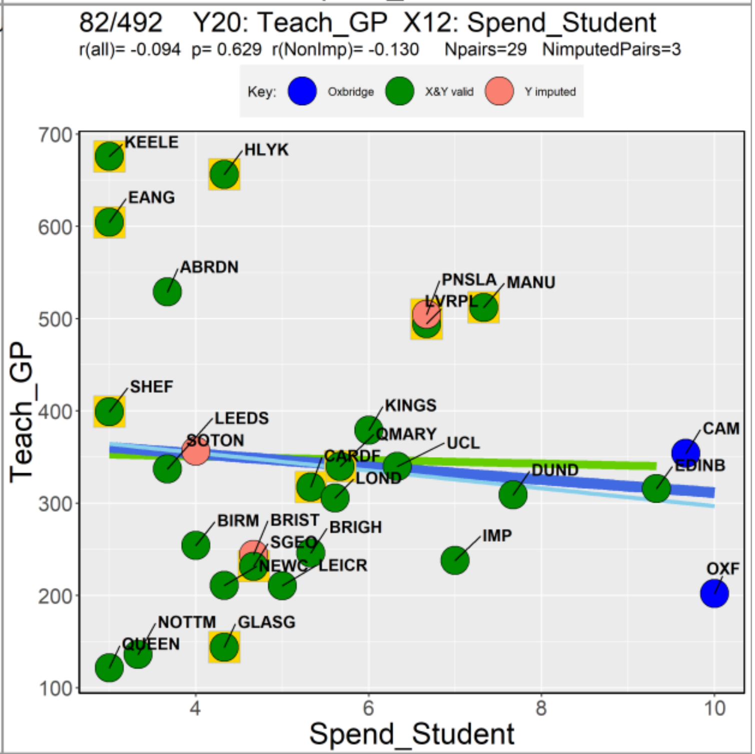
82/492 Y20: Teach_GP X12: Spend_Student
r(all)= -0.094 p= 0.629 r(NonImp)= -0.130 Npairs=29 NimputedPairs=3
Key: Oxbridge X&Y valid Y imputed
Teach_GP
Spend_Student

83/493 Y21: Teach_Psyc X12: Spend_Student

r(all)= 0.349 p= 0.0635 r(NonImp)= 0.370 Npairs=29 NimputedPairs=3

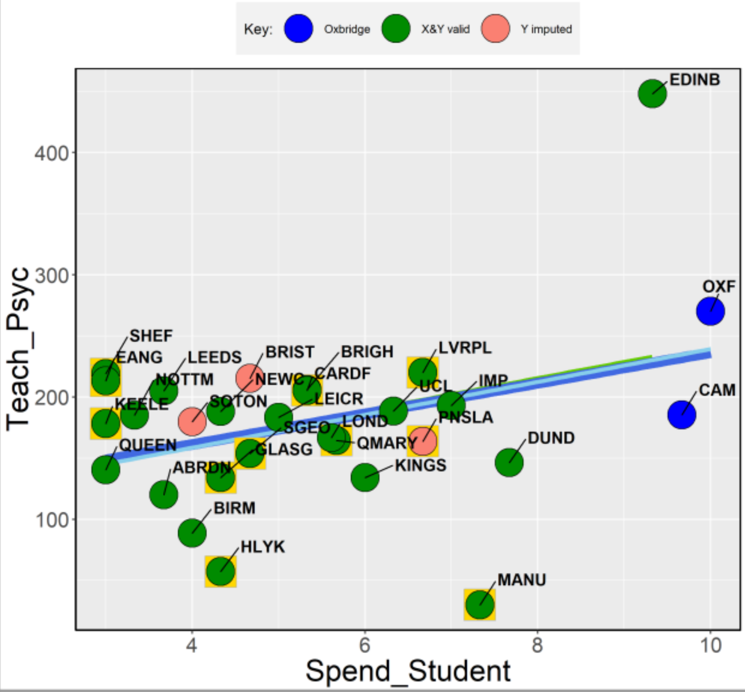

83/494 Y22: Teach_Anaes X12: Spend_Student

r(all)= -0.135 p= 0.485 r(NonImp)= -0.114 Npairs=29 NimputedPairs=3

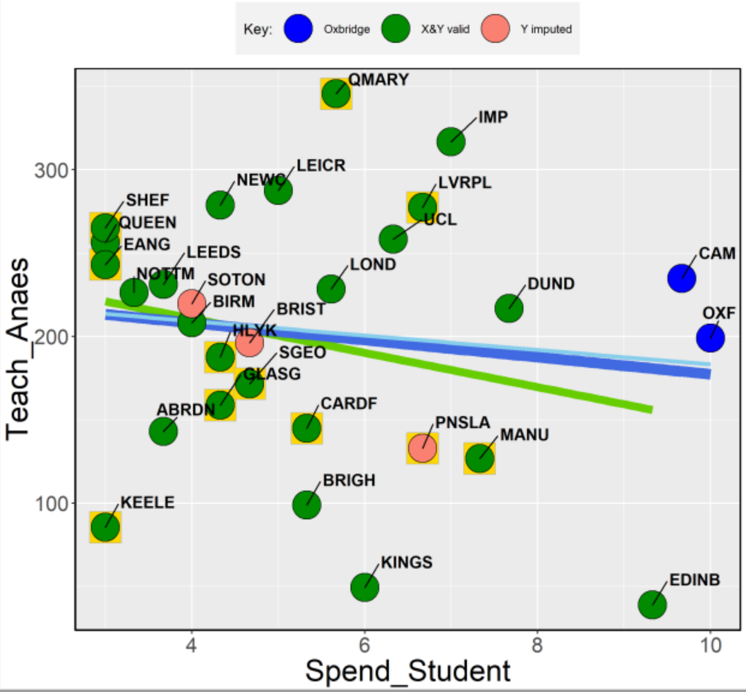

83/495 Y23: Teach_OG X12: Spend_Student

r(all)= 0.574 p= 0.00114 r(NonImp)= 0.607 Npairs=29 NimputedPairs=3

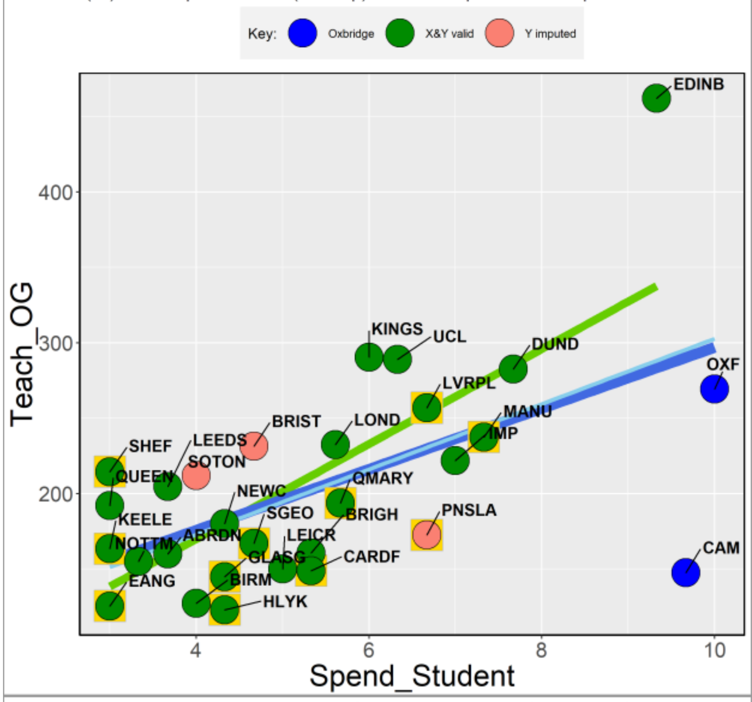

83/496 Y24: Teach_IntMed X12: Spend_Student

r(all)= 0.449 p= 0.0146 r(NonImp)= 0.479 Npairs=29 NimputedPairs=3

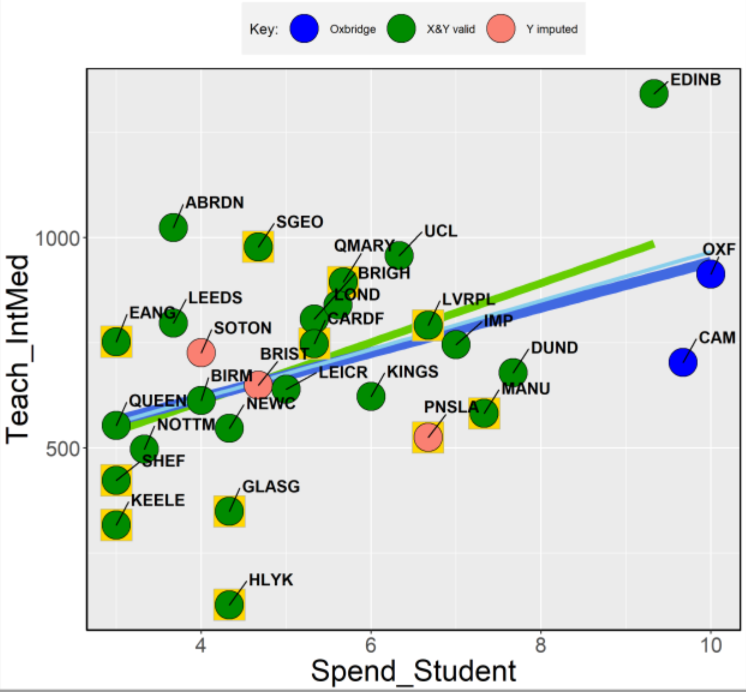

83/497 Y25: Teach_Surgery X12: Spend_Student

r(all)= 0.327 p= 0.0831 r(NonImp)= 0.353 Npairs=29 NimputedPairs=3

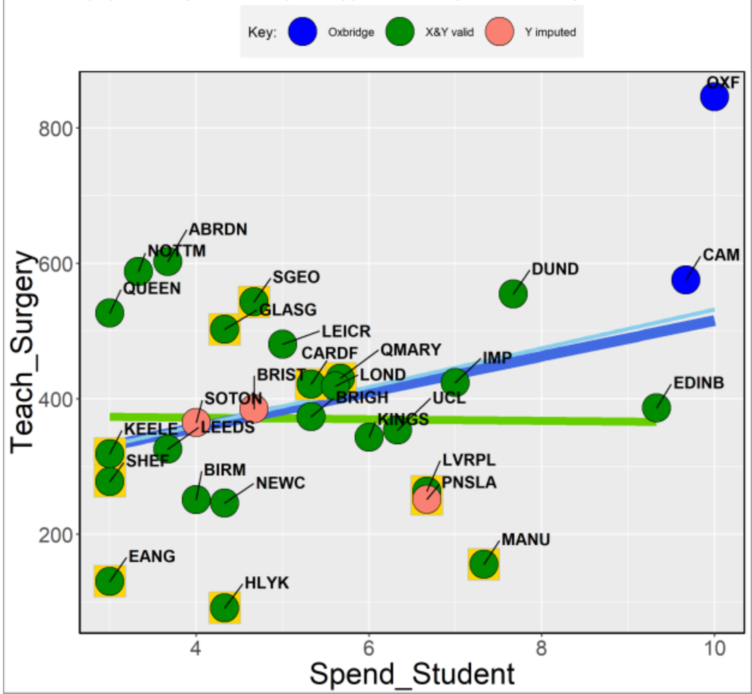

83/498 Y26: ExamTime X12: Spend_Student

r(all)= 0.303 p= 0.11 r(NonImp)= 0.304 Npairs=29 NimputedPairs=3

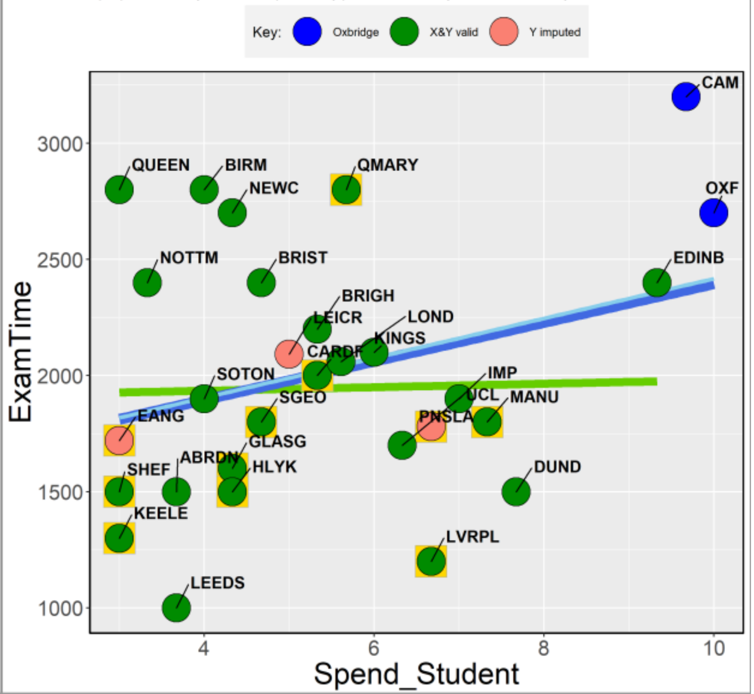

84/499 Y27: SelfRegLearn X12: Spend_Student
r(all)= 0.481 p= 0.00828 r(NonImp)= 0.481 Npairs=29 NimputedPairs=0

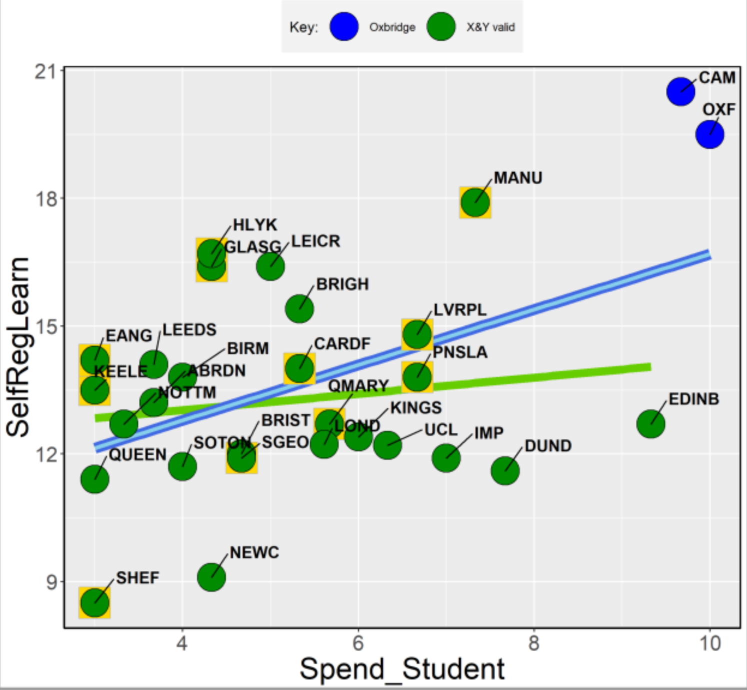

84/500 Y28: NSS_Satisfn X12: Spend_Student
r(all)= -0.005 p= 0.978 r(NonImp)= -0.005 Npairs=29 NimputedPairs=0

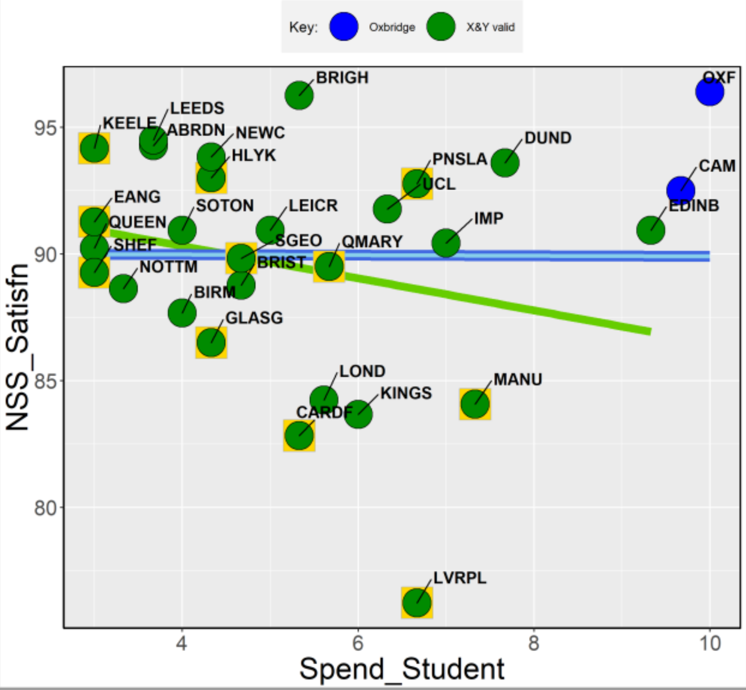

84/501 Y29: NSS_Feedback X12: Spend_Student
r(all)= 0.095 p= 0.624 r(NonImp)= 0.095 Npairs=29 NimputedPairs=0

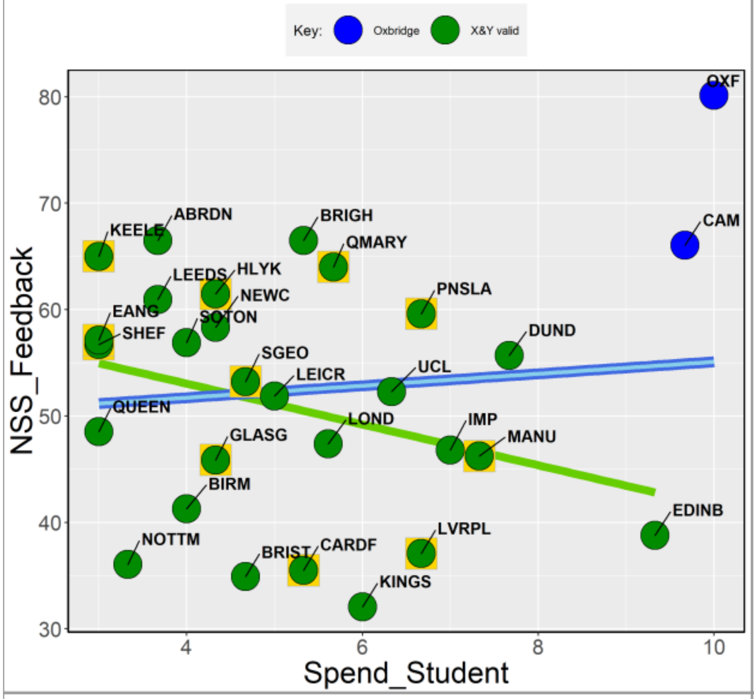

84/502 Y30: UKFPO_EPM X12: Spend_Student
r(all)= 0.450 p= 0.0144 r(NonImp)= 0.450 Npairs=29 NimputedPairs=0

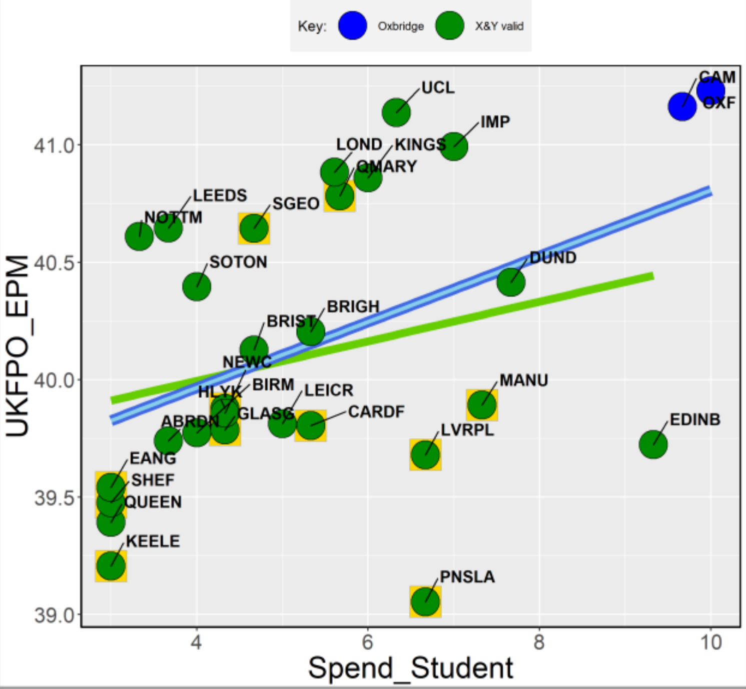

84/503 Y31: UKFPO_SJT X12: Spend_Student
r(all)= 0.631 p= 0.000239 r(NonImp)= 0.631 Npairs=29 NimputedPairs=0

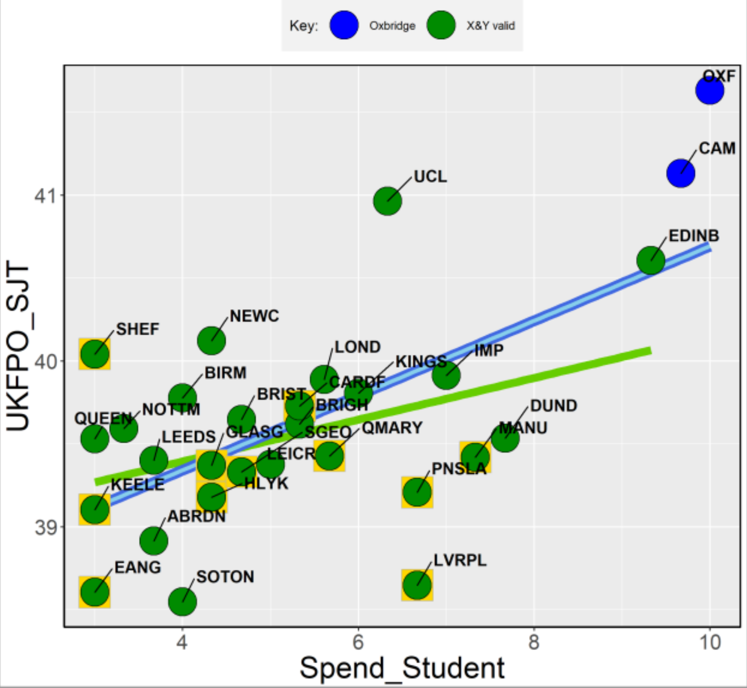

84/504 Y32: F1_Preparedness X12: Spend_Student
r(all)= -0.027 p= 0.89 r(NonImp)= -0.027 Npairs=29 NimputedPairs=0

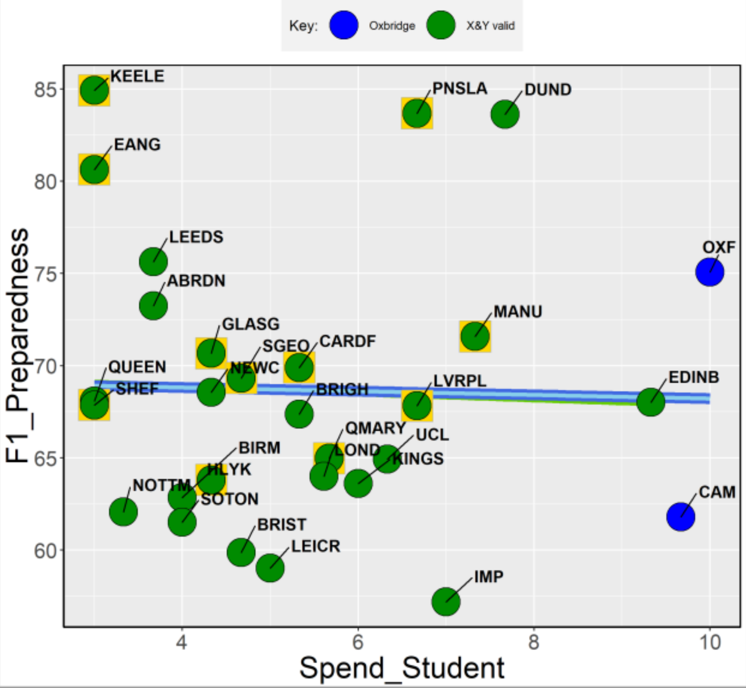

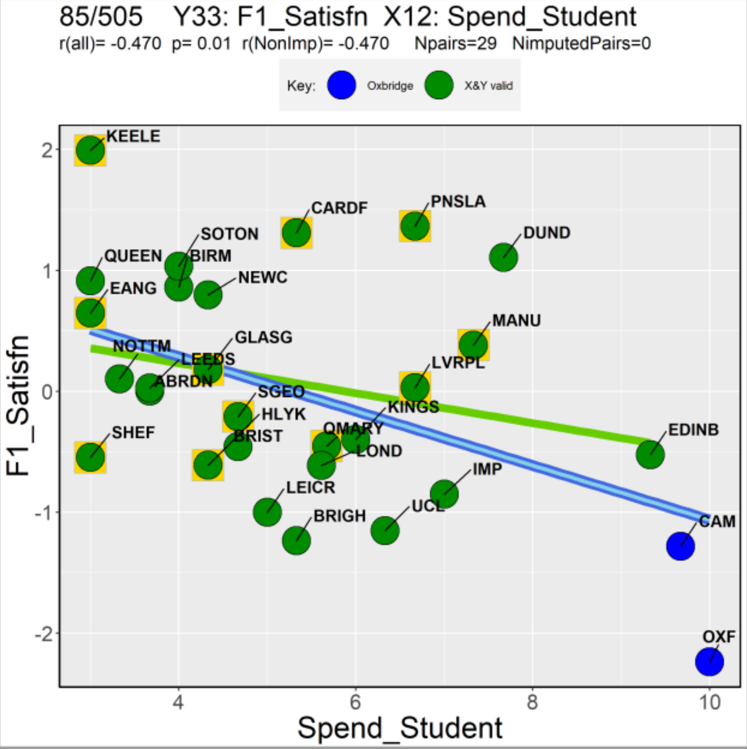
85/505 Y33: F1_Satisfn X12: Spend_Student
r(all)= -0.470 p= 0.01 r(NonImp)= -0.470 Npairs=29 NimputedPairs=0
Key: Oxbridge X&Y valid
F1_Satisfn
Spend_Student
KEELE
CARDF
PNSLA
SOTON
DUND
QUEEN
BIRM
EANG
NEWC
MANU
GLASG
NOTTM
LEEDS
ABRDN
LVRPL
SGEO
KINGS
HLYK
SHEF
QMARY
BRIST
LOND
EDINB
IMP
LEICR
UCL
BRIGH
CAM
OXF

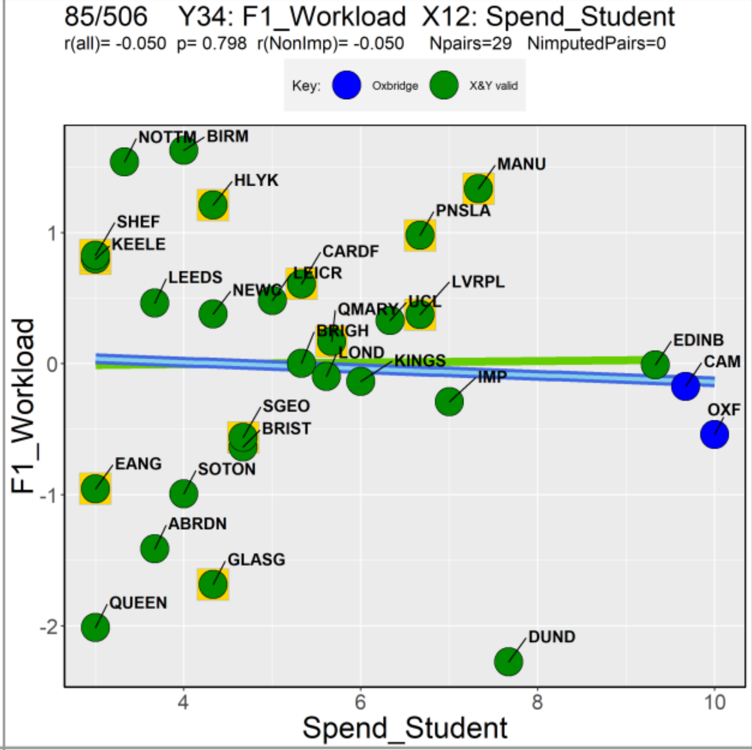
85/506 Y34: F1_Workload X12: Spend_Student
r(all)= -0.050 p= 0.798 r(NonImp)= -0.050 Npairs=29 NimputedPairs=0
Key: Oxbridge X&Y valid
F1_Workload
Spend_Student
NOTTM
BIRM
MANU
HLYK
PNSLA
SHEF
KEELE
CARDF
LEEDS
LEICR
LVRPL
NEWC
QMARY
UCL
BRIGH
LOND
EDINB
KINGS
CAM
IMP
OXF
SGEO
BRIST
EANG
SOTON
ABRDN
GLASG
QUEEN
DUND

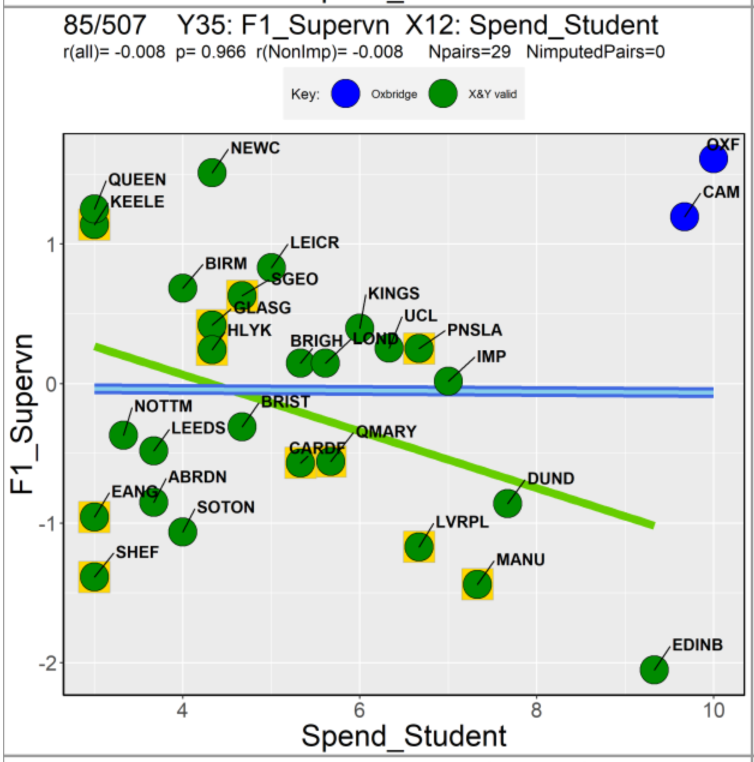
85/507 Y35: F1_Supervn X12: Spend_Student
r(all)= -0.008 p= 0.966 r(NonImp)= -0.008 Npairs=29 NimputedPairs=0
Key: Oxbridge X&Y valid
F1_Supervn
Spend_Student
NEWC
OXF
QUEEN
CAM
KEELE
LEICR
BIRM
SGEO
KINGS
GLASG
UCL
HLYK
PNSLA
BRIGH
LOND
IMP
NOTTM
BRIST
LEEDS
QMARY
CARDF
DUND
EANG
ABRDN
SOTON
LVRPL
SHEF
MANU
EDINB

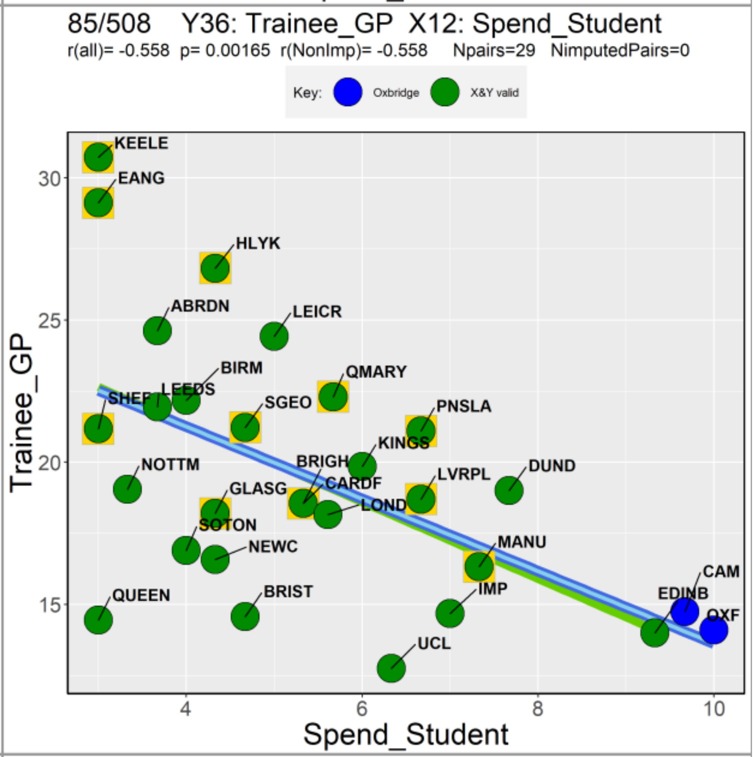
85/508 Y36: Trainee_GP X12: Spend_Student
r(all)= -0.558 p= 0.00165 r(NonImp)= -0.558 Npairs=29 NimputedPairs=0
Key: Oxbridge X&Y valid
Trainee_GP
Spend_Student
KEELE
EANG
HLYK
ABRDN
LEICR
BIRM
QMARY
SHEF
LEEDS
SGEO
PNSLA
KINGS
NOTTM
BRIGH
DUND
LVRPL
GLASG
CARDF
LOND
SOTON
NEWC
MANU
CAM
EDINB
QUEEN
BRIST
IMP
OXF
UCL

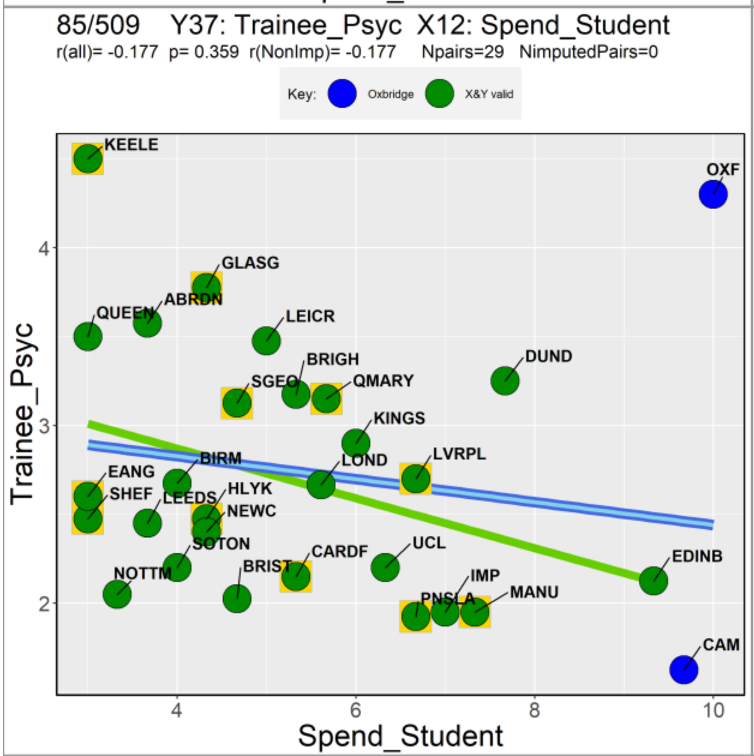
85/509 Y37: Trainee_Psyc X12: Spend_Student
r(all)= -0.177 p= 0.359 r(NonImp)= -0.177 Npairs=29 NimputedPairs=0
Key: Oxbridge X&Y valid
Trainee_Psyc
Spend_Student
KEELE
OXF
GLASG
QUEEN
ABRDN
LEICR
BRIGH
DUND
SGEO
QMARY
KINGS
BIRM
LOND
LVRPL
EANG
SHEF
LEEDS
HLYK
NEWC
SOTON
UCL
NOTTM
BRIST
CARDF
IMP
EDINB
PNSLA
MANU
CAM

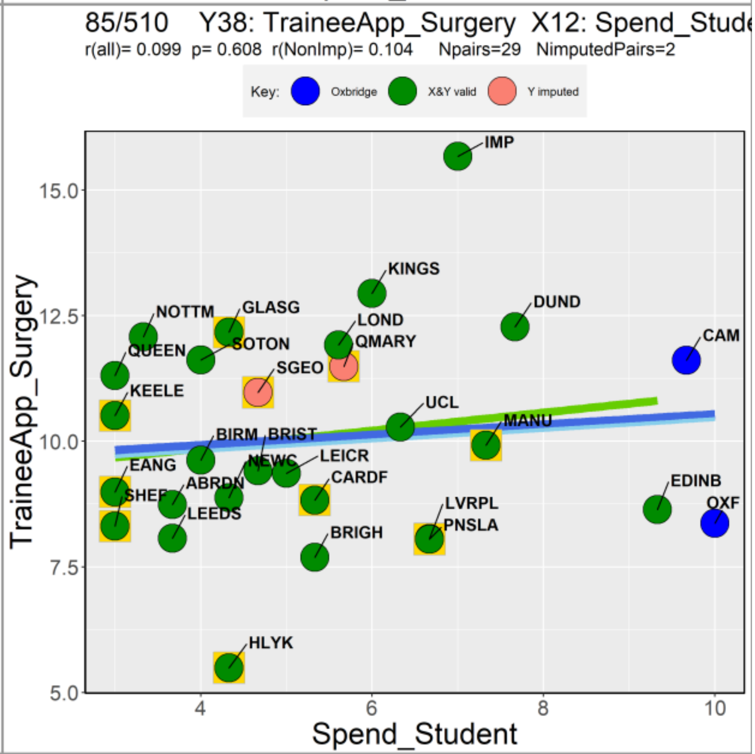
85/510 Y38: TraineeApp_Surgery X12: Spend_Stude
r(all)= 0.099 p= 0.608 r(NonImp)= 0.104 Npairs=29 NimputedPairs=2
Key: Oxbridge X&Y valid Y imputed
TraineeApp_Surgery
Spend_Student
IMP
KINGS
DUND
NOTTM
GLASG
LOND
CAM
QUEEN
SOTON
QMARY
SGEO
KEELE
UCL
BIRM
BRIST
MANU
EANG
NEWC
LEICR
ABRDN
CARDF
EDINB
SHEF
LVRPL
OXF
LEEDS
PNSLA
BRIGH
HLYK

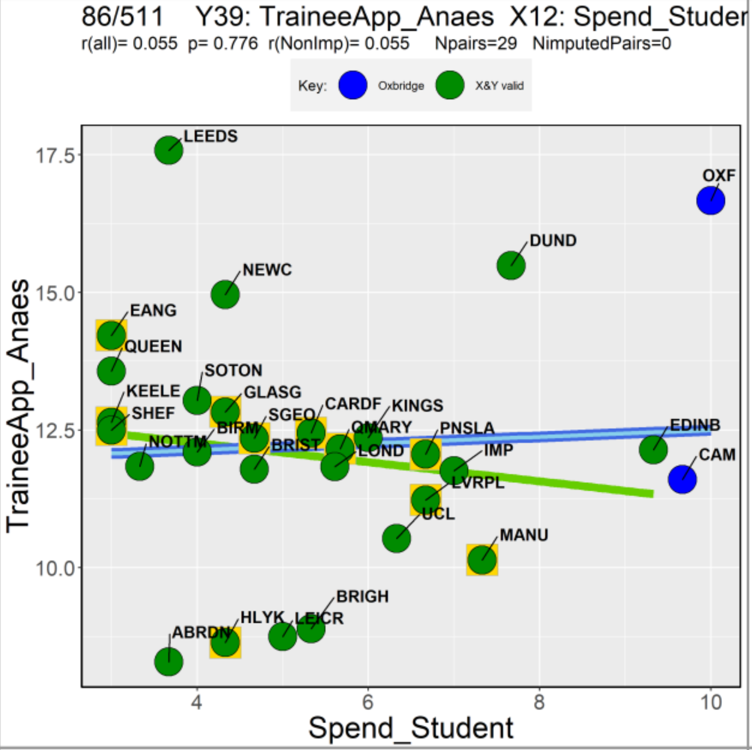
86/511 Y39: TraineeApp_Anaes X12: Spend_Studer
r(all)= 0.055 p= 0.776 r(NonImp)= 0.055 Npairs=29 NimputedPairs=0
Key: Oxbridge X&Y valid
TraineeApp_Anaes
Spend_Student
LEEDS
OXF
DUND
NEWC
EANG
QUEEN
SOTON
KEELE
GLASG
SHEF
SGEO
CARDF
KINGS
BIRM
QMARY
PNSLA
EDINB
NOTTM
BRIST
LOND
IMP
CAM
LVRPL
UCL
MANU
BRIGH
HLYK
LEICR
ABRDN

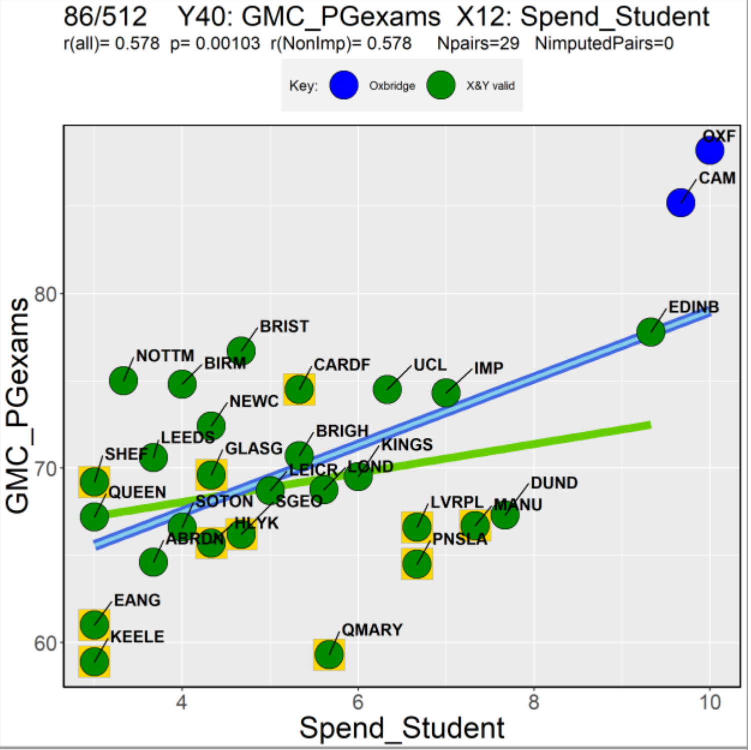
86/512 Y40: GMC_PGexams X12: Spend_Student
r(all)= 0.578 p= 0.00103 r(NonImp)= 0.578 Npairs=29 NimputedPairs=0
Key: Oxbridge X&Y valid
GMC_PGexams
Spend_Student
OXF
CAM
EDINB
BRIST
NOTTM
BIRM
CARDF
UCL
IMP
NEWC
LEEDS
BRIGH
SHEF
GLASG
KINGS
LEICR
LOND
QUEEN
SOTON
SGEO
DUND
LVRPL
MANU
HLYK
ABRDN
PNSLA
EANG
KEELE
QMARY

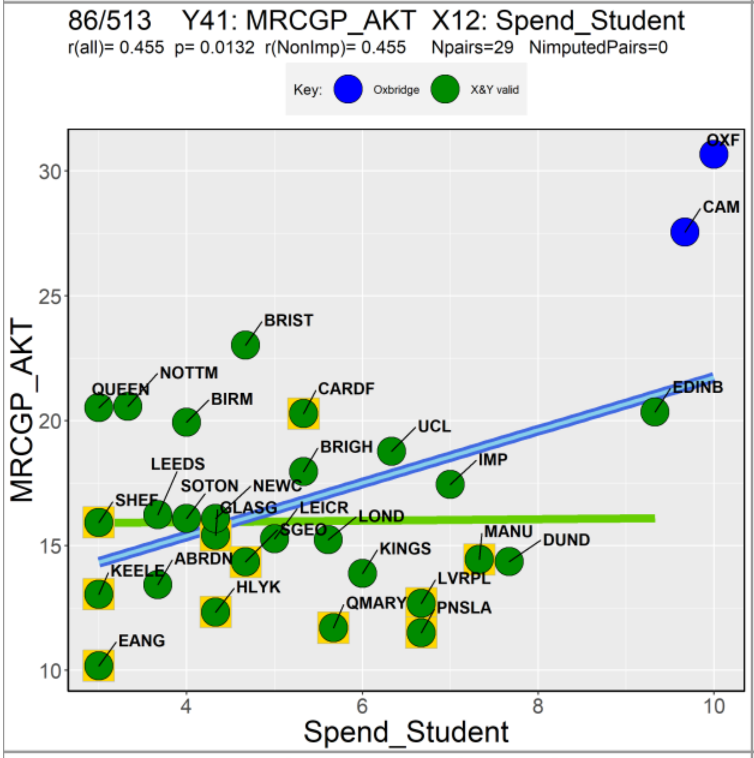
86/513 Y41: MRCGP_AKT X12: Spend_Student
r(all)= 0.455 p= 0.0132 r(NonImp)= 0.455 Npairs=29 NimputedPairs=0
Key: Oxbridge X&Y valid
MRCGP_AKT
Spend_Student
OXF
CAM
BRIST
NOTTM
QUEEN
BIRM
CARDF
EDINB
UCL
LEEDS
BRIGH
IMP
SHEF
SOTON
NEWC
GLASG
LEICR
LOND
SGEO
MANU
DUND
KINGS
KEELE
ABRDN
HLYK
LVRPL
QMARY
PNSLA
EANG

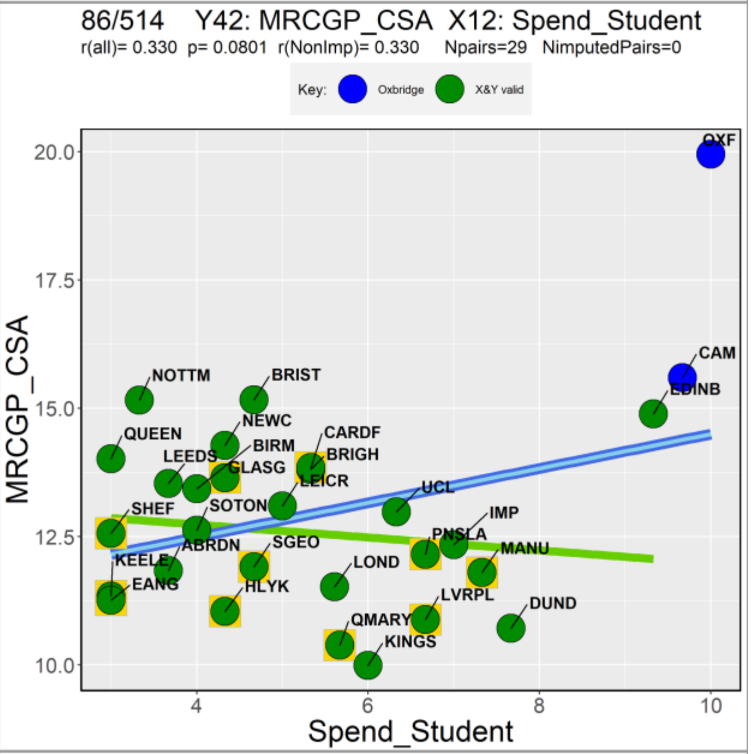
86/514 Y42: MRCGP_CSA X12: Spend_Student
r(all)= 0.330 p= 0.0801 r(NonImp)= 0.330 Npairs=29 NimputedPairs=0
Key: Oxbridge X&Y valid
MRCGP_CSA
Spend_Student
OXF
CAM
EDINB
NOTTM
BRIST
QUEEN
NEWC
CARDF
LEEDS
BIRM
BRIGH
GLASG
LEICR
SHEF
UCL
SOTON
IMP
PNSLA
ABRDN
SGEO
MANU
KEELE
LOND
EANG
HLYK
LVRPL
DUND
QMARY
KINGS

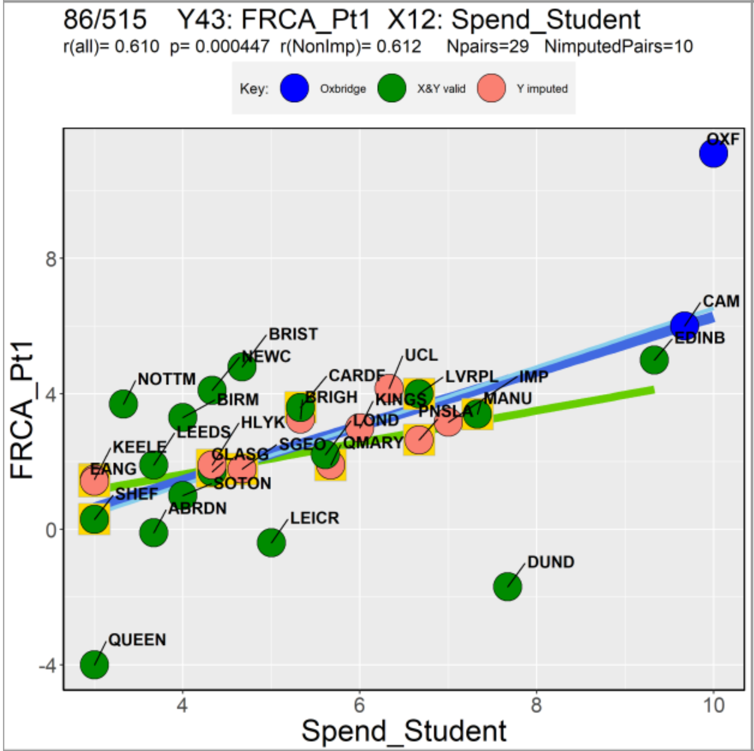
86/515 Y43: FRCA_Pt1 X12: Spend_Student
r(all)= 0.610 p= 0.000447 r(NonImp)= 0.612 Npairs=29 NimputedPairs=10
Key: Oxbridge X&Y valid Y imputed
FRCA_Pt1
Spend_Student
OXF
CAM
EDINB
BRIST
NEWC
CARDF
UCL
LVRPL
IMP
NOTTM
BIRM
BRIGH
KINGS
MANU
HLYK
LOND
PNSLA
LEEDS
KEELE
GLASG
SGEO
QMARY
EANG
SOTON
SHEF
ABRDN
LEICR
DUND
QUEEN

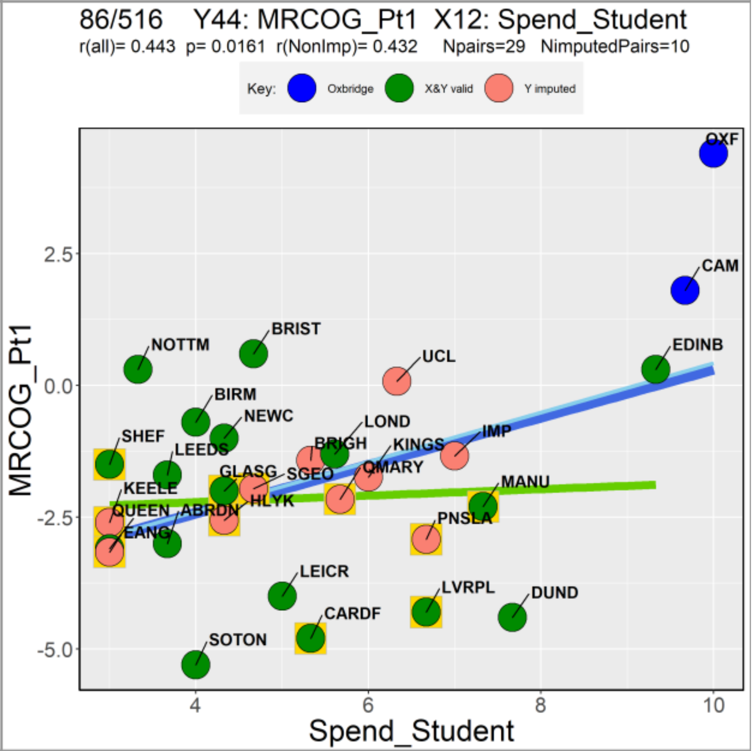
86/516 Y44: MRCOG_Pt1 X12: Spend_Student
r(all)= 0.443 p= 0.0161 r(NonImp)= 0.432 Npairs=29 NimputedPairs=10
Key: Oxbridge X&Y valid Y imputed
MRCOG_Pt1
Spend_Student
OXF
CAM
EDINB
NOTTM
BRIST
UCL
BIRM
NEWC
LOND
SHEF
LEEDS
BRIGH
KINGS
IMP
QMARY
GLASG
SGEO
MANU
KEELE
HLYK
QUEEN
ABRDN
PNSLA
EANG
LEICR
LVRPL
DUND
CARDF
SOTON

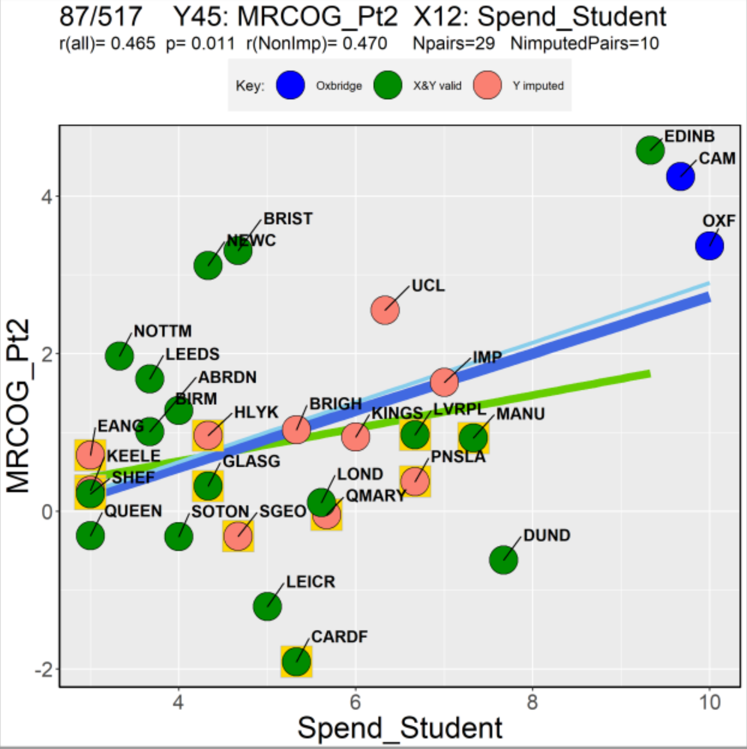
87/517 Y45: MRCOG_Pt2 X12: Spend_Student
r(all)= 0.465 p= 0.011 r(NonImp)= 0.470 Npairs=29 NimputedPairs=10
Key: Oxbridge X&Y valid Y imputed
MRCOG_Pt2
Spend_Student

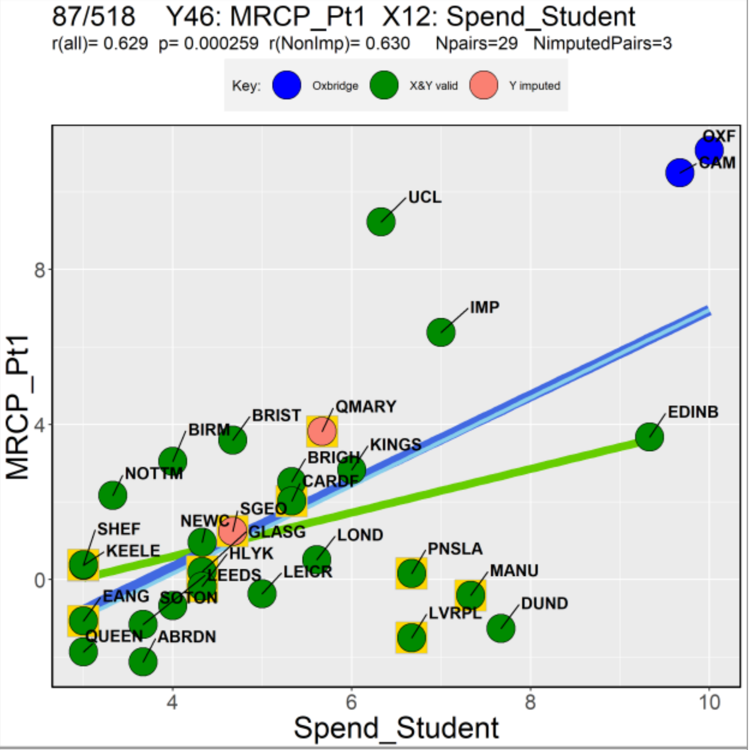
87/518 Y46: MRCP_Pt1 X12: Spend_Student
r(all)= 0.629 p= 0.000259 r(NonImp)= 0.630 Npairs=29 NimputedPairs=3
Key: Oxbridge X&Y valid Y imputed
MRCP_Pt1
Spend_Student

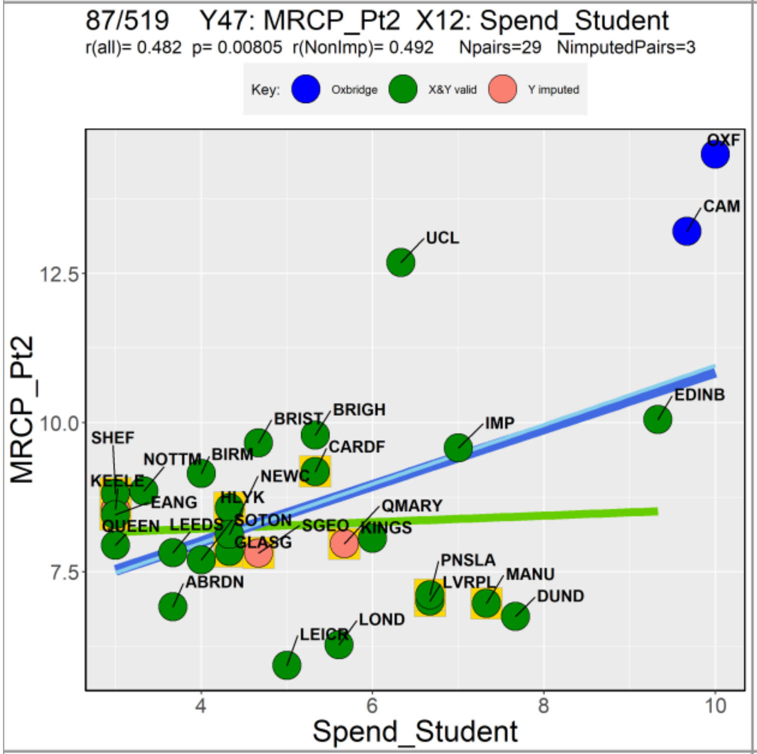
87/519 Y47: MRCP_Pt2 X12: Spend_Student
r(all)= 0.482 p= 0.00805 r(NonImp)= 0.492 Npairs=29 NimputedPairs=3
Key: Oxbridge X&Y valid Y imputed
MRCP_Pt2
Spend_Student

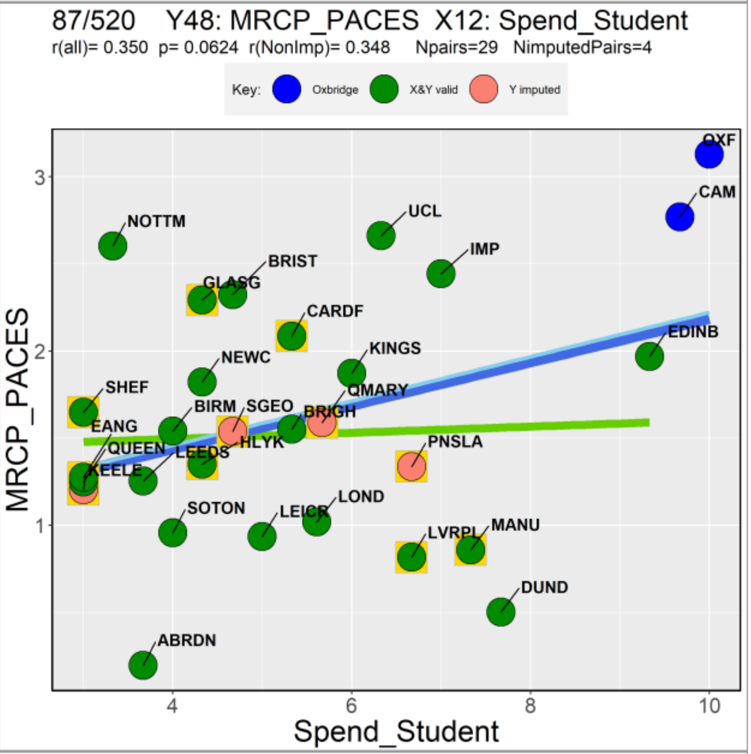
87/520 Y48: MRCP_PACES X12: Spend_Student
r(all)= 0.350 p= 0.0624 r(NonImp)= 0.348 Npairs=29 NimputedPairs=4
Key: Oxbridge X&Y valid Y imputed
MRCP_PACES
Spend_Student

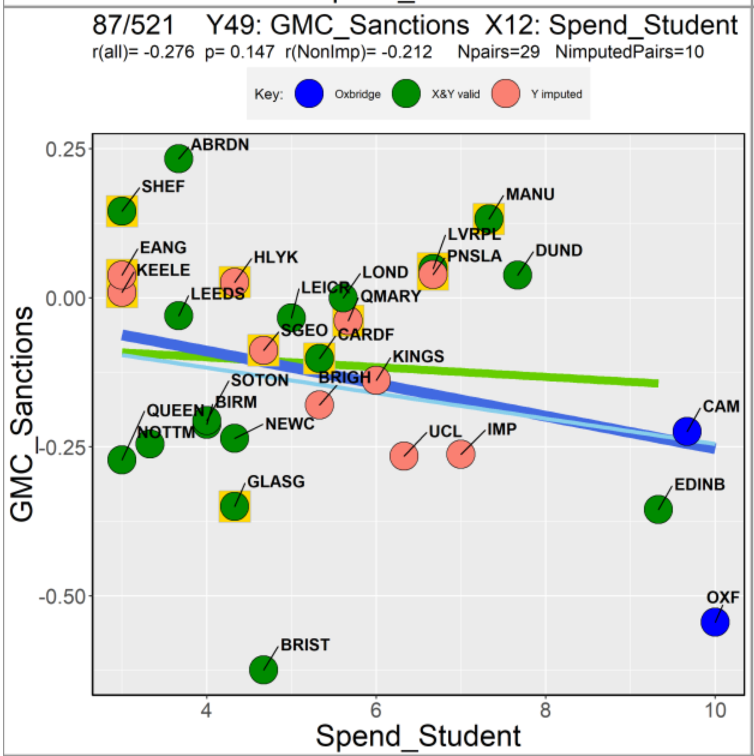
87/521 Y49: GMC_Sanctions X12: Spend_Student
r(all)= -0.276 p= 0.147 r(NonImp)= -0.212 Npairs=29 NimputedPairs=10
Key: Oxbridge X&Y valid Y imputed
GMC_Sanctions
Spend_Student

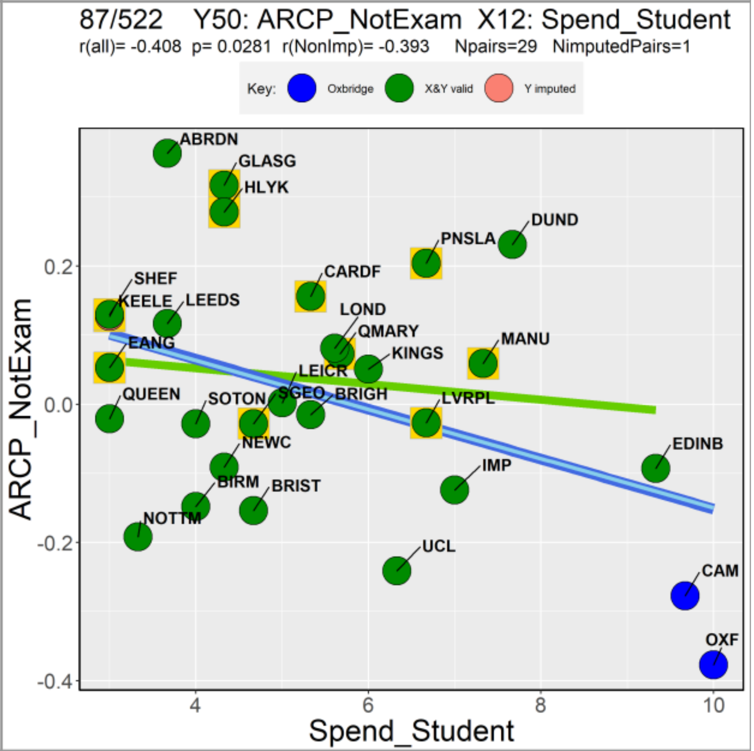
87/522 Y50: ARCP_NotExam X12: Spend_Student
r(all)= -0.408 p= 0.0281 r(NonImp)= -0.393 Npairs=29 NimputedPairs=1
Key: Oxbridge X&Y valid Y imputed
ARCP_NotExam
Spend_Student

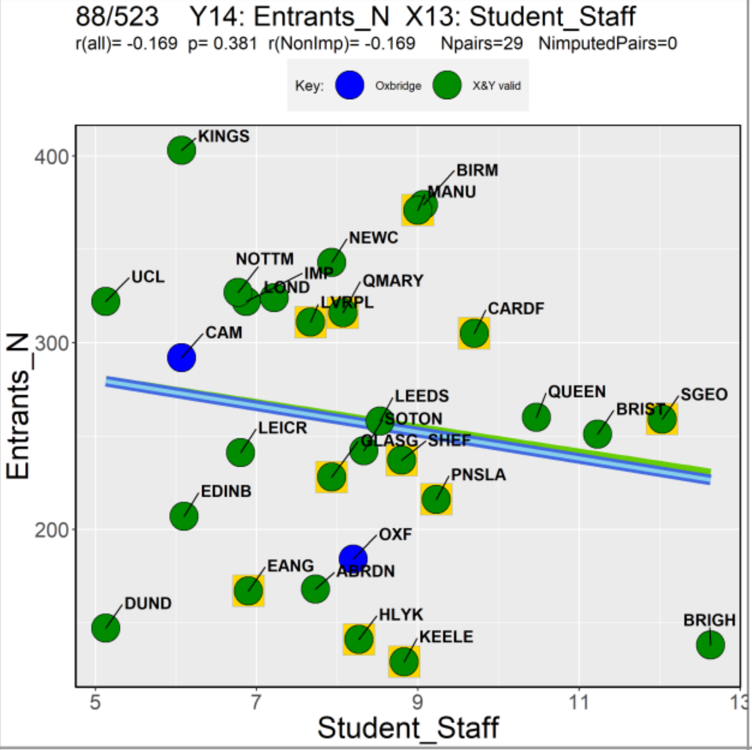
88/523 Y14: Entrants_N X13: Student_Staff
r(all)= -0.169 p= 0.381 r(NonImp)= -0.169 Npairs=29 NimputedPairs=0
Key: Oxbridge X&Y valid
Entrants_N
Student_Staff

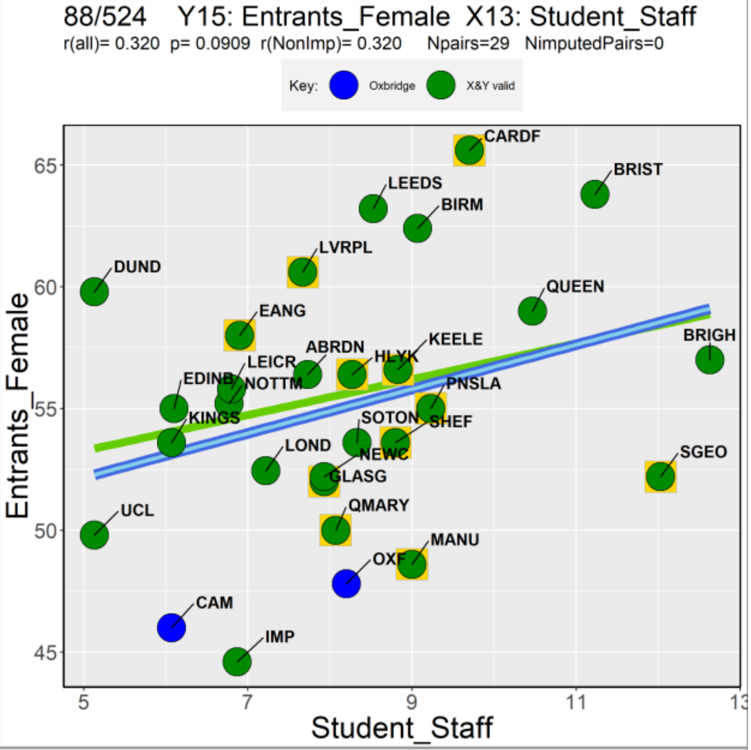
88/524 Y15: Entrants_Female X13: Student_Staff
r(all)= 0.320 p= 0.0909 r(NonImp)= 0.320 Npairs=29 NimputedPairs=0
Key: Oxbridge X&Y valid
Entrants_Female
Student_Staff

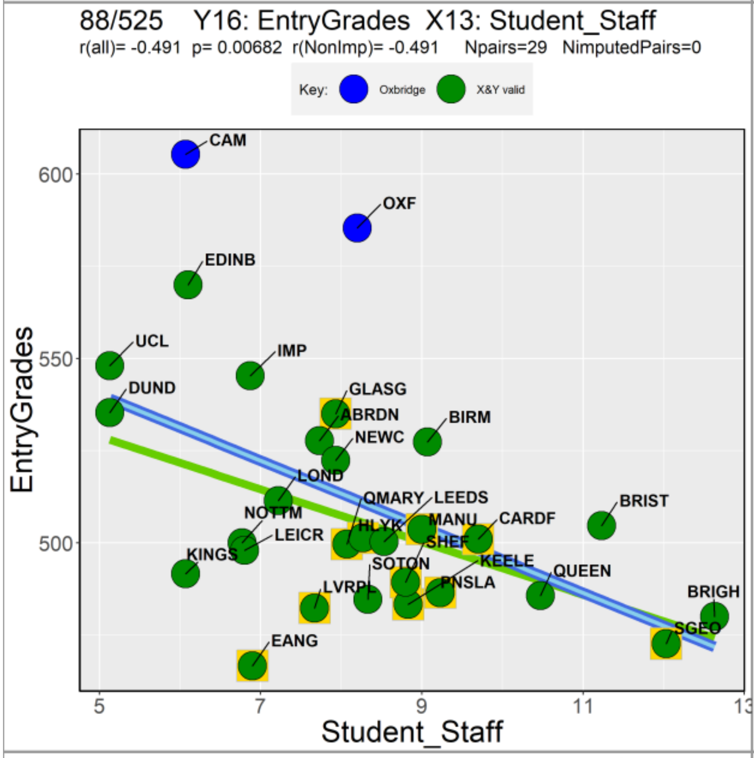
88/525 Y16: EntryGrades X13: Student_Staff
r(all)= -0.491 p= 0.00682 r(NonImp)= -0.491 Npairs=29 NimputedPairs=0
Key: Oxbridge X&Y valid
EntryGrades
Student_Staff

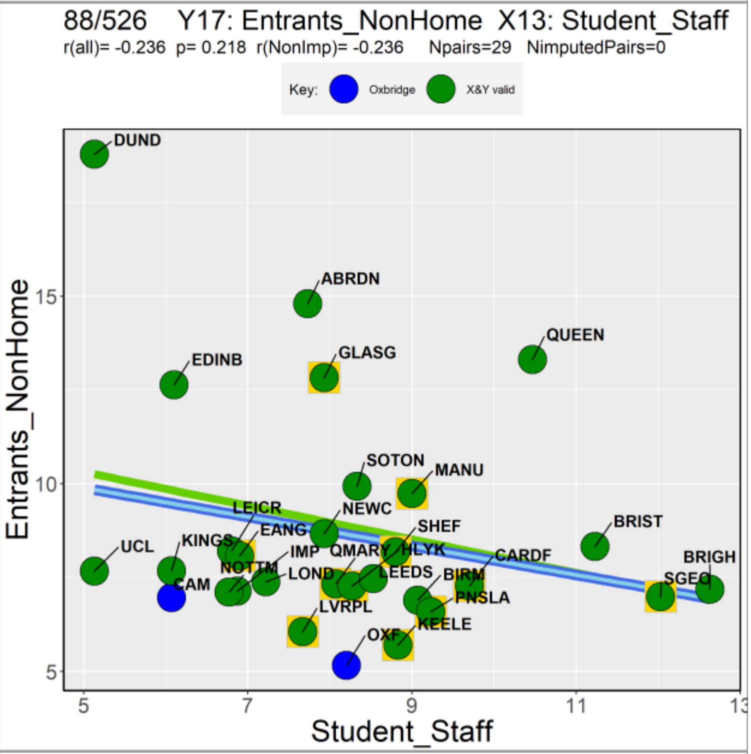
88/526 Y17: Entrants_NonHome X13: Student_Staff
r(all)= -0.236 p= 0.218 r(NonImp)= -0.236 Npairs=29 NimputedPairs=0
Key: Oxbridge X&Y valid
Entrants_NonHome
Student_Staff

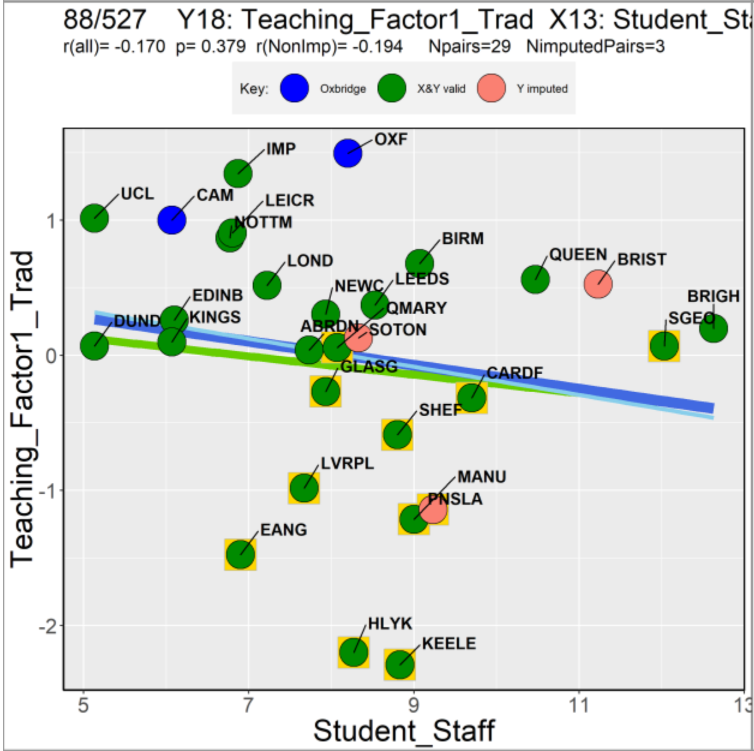
88/527 Y18: Teaching_Factor1_Trad X13: Student_Sta
r(all)= -0.170 p= 0.379 r(NonImp)= -0.194 Npairs=29 NimputedPairs=3
Key: Oxbridge X&Y valid Y imputed
Teaching_Factor1_Trad
Student_Staff

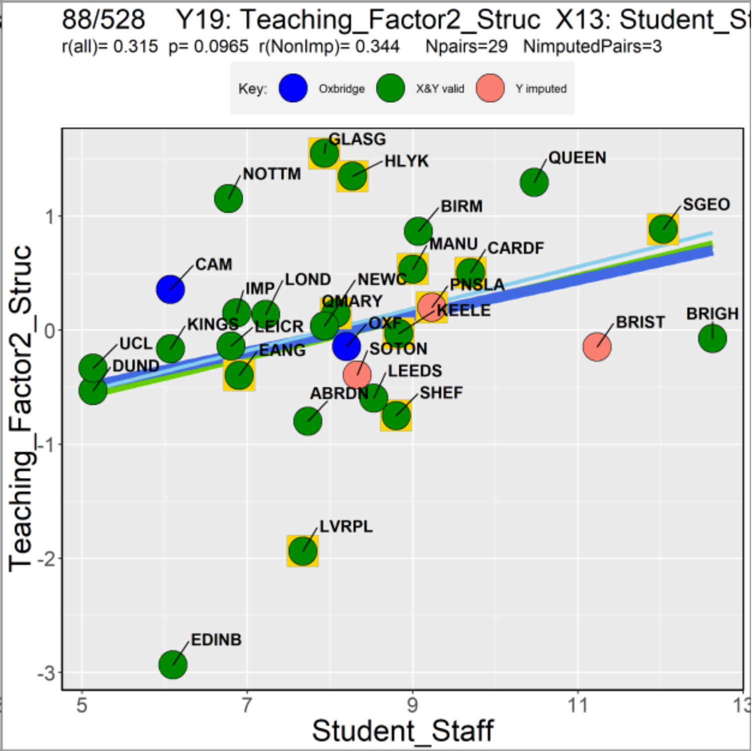
88/528 Y19: Teaching_Factor2_Struc X13: Student_St
r(all)= 0.315 p= 0.0965 r(NonImp)= 0.344 Npairs=29 NimputedPairs=3
Key: Oxbridge X&Y valid Y imputed
Teaching_Factor2_Struc
Student_Staff

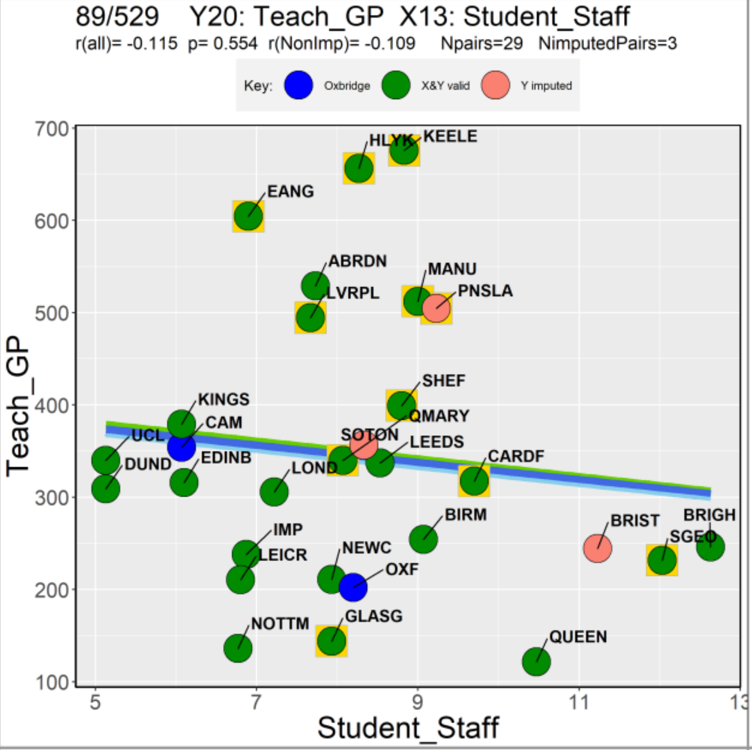
89/529 Y20: Teach_GP X13: Student_Staff
r(all)= -0.115 p= 0.554 r(NonImp)= -0.109 Npairs=29 NimputedPairs=3
Key: Oxbridge X&Y valid Y imputed
Teach_GP
Student_Staff

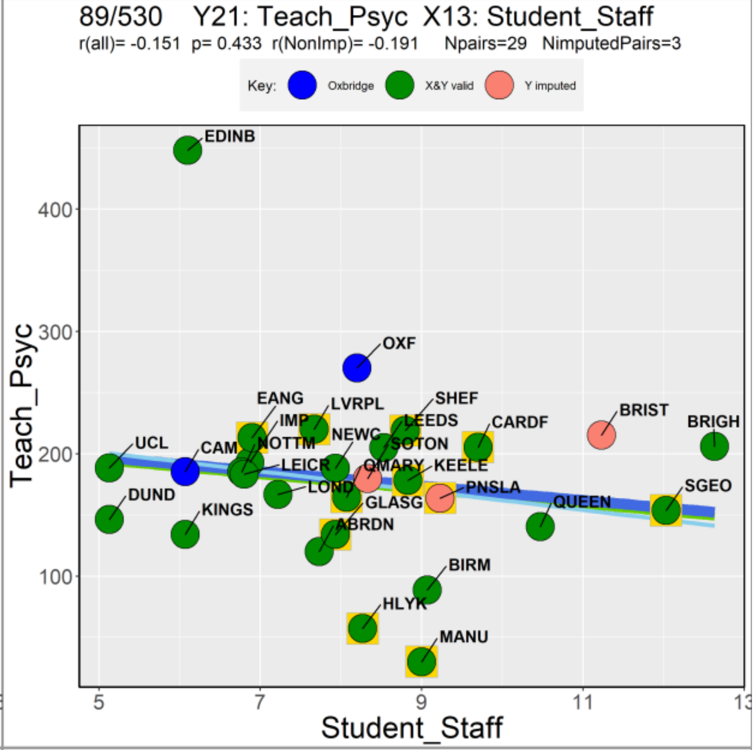
89/530 Y21: Teach_Psyc X13: Student_Staff
r(all)= -0.151 p= 0.433 r(NonImp)= -0.191 Npairs=29 NimputedPairs=3
Key: Oxbridge X&Y valid Y imputed
Teach_Psyc
Student_Staff

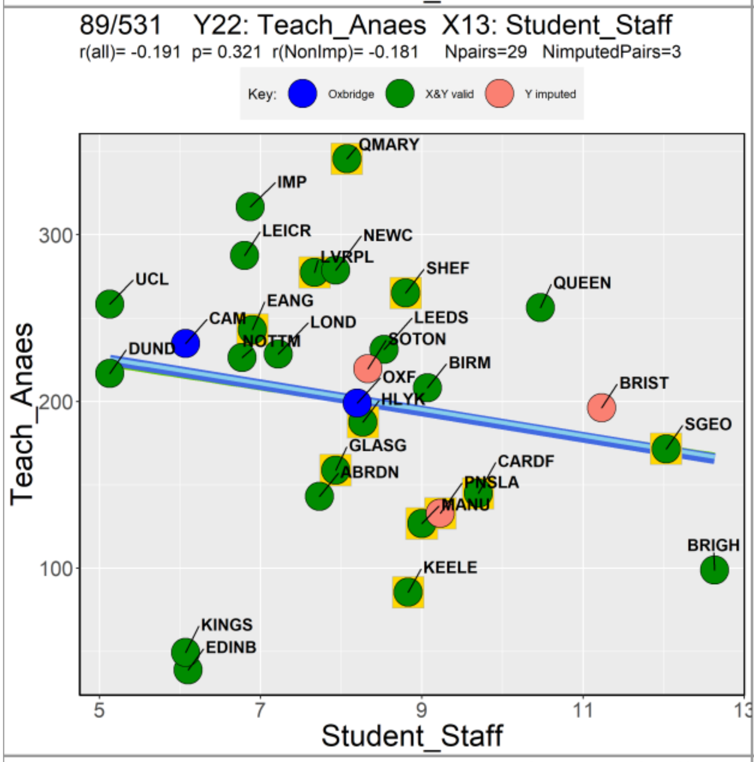
89/531 Y22: Teach_Anaes X13: Student_Staff
r(all)= -0.191 p= 0.321 r(NonImp)= -0.181 Npairs=29 NimputedPairs=3
Key: Oxbridge X&Y valid Y imputed
Teach_Anaes
Student_Staff

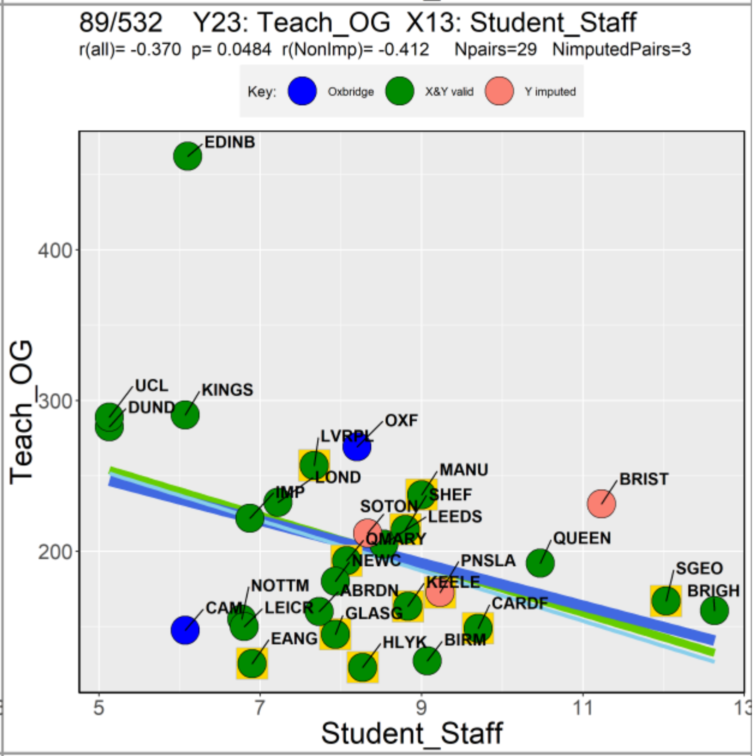
89/532 Y23: Teach_OG X13: Student_Staff
r(all)= -0.370 p= 0.0484 r(NonImp)= -0.412 Npairs=29 NimputedPairs=3
Key: Oxbridge X&Y valid Y imputed
Teach_OG
Student_Staff

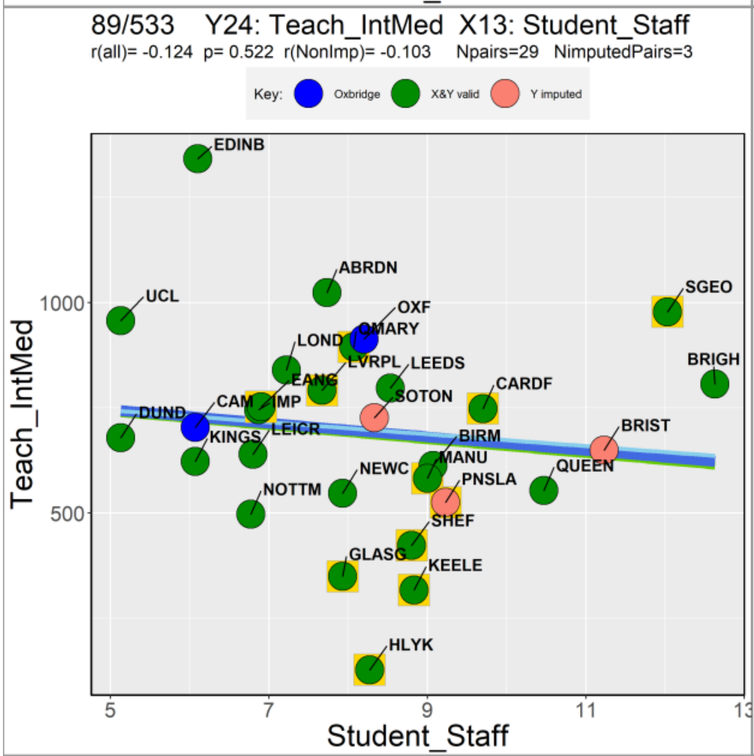
89/533 Y24: Teach_IntMed X13: Student_Staff
r(all)= -0.124 p= 0.522 r(NonImp)= -0.103 Npairs=29 NimputedPairs=3
Key: Oxbridge X&Y valid Y imputed
Teach_IntMed
Student_Staff

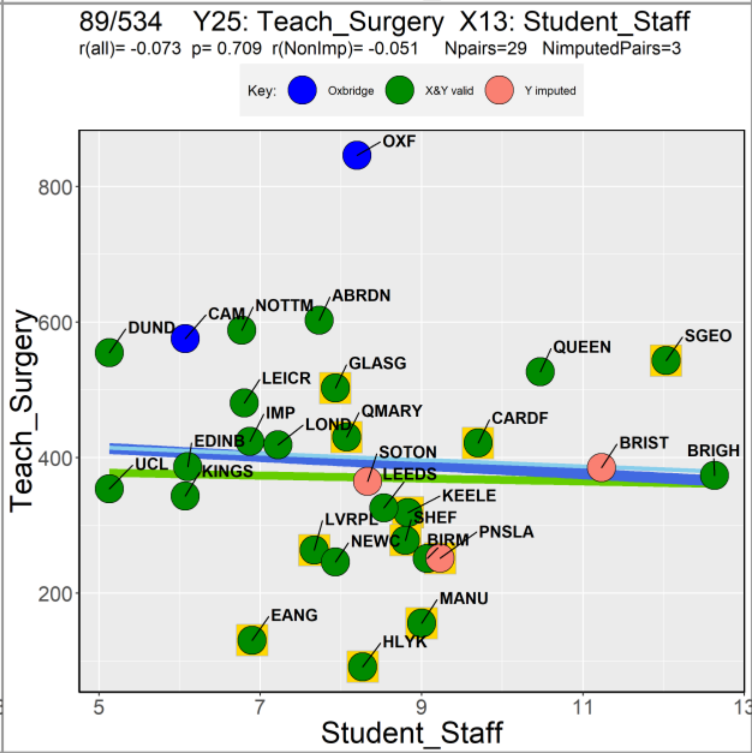
89/534 Y25: Teach_Surgery X13: Student_Staff
r(all)= -0.073 p= 0.709 r(NonImp)= -0.051 Npairs=29 NimputedPairs=3
Key: Oxbridge X&Y valid Y imputed
Teach_Surgery
Student_Staff

90/535 Y26: ExamTime X13: Student_Staff
r(all)= 0.050 p= 0.799 r(NonImp)= 0.050 Npairs=29 NimputedPairs=3

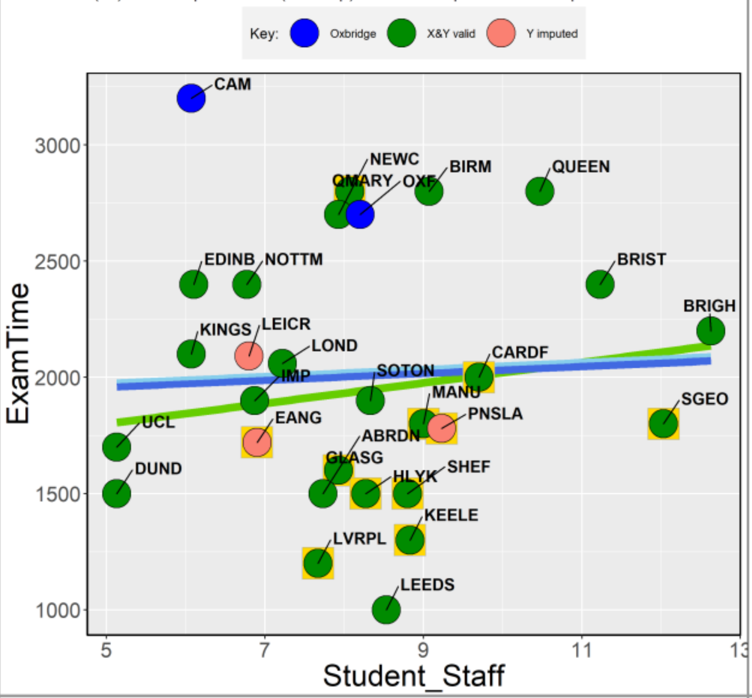

90/536 Y27: SelfRegLearn X13: Student_Staff
r(all)= -0.045 p= 0.817 r(NonImp)= -0.045 Npairs=29 NimputedPairs=0

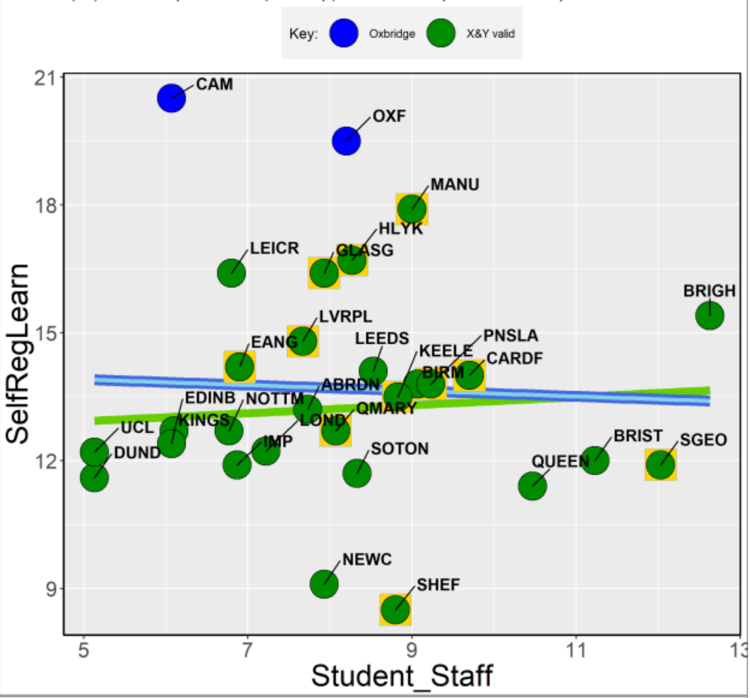

90/537 Y28: NSS_Satisfn X13: Student_Staff
r(all)= 0.052 p= 0.787 r(NonImp)= 0.052 Npairs=29 NimputedPairs=0

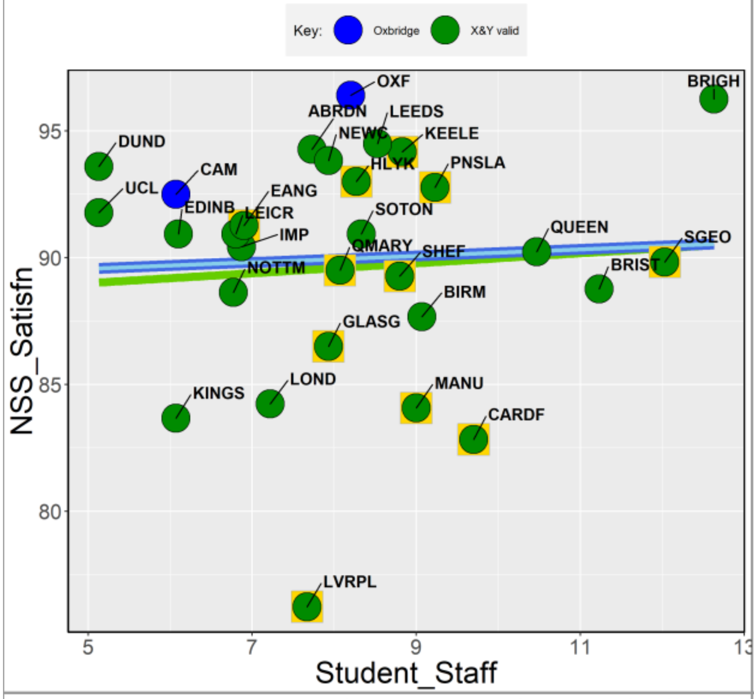

90/538 Y29: NSS_Feedback X13: Student_Staff
r(all)= 0.081 p= 0.678 r(NonImp)= 0.081 Npairs=29 NimputedPairs=0

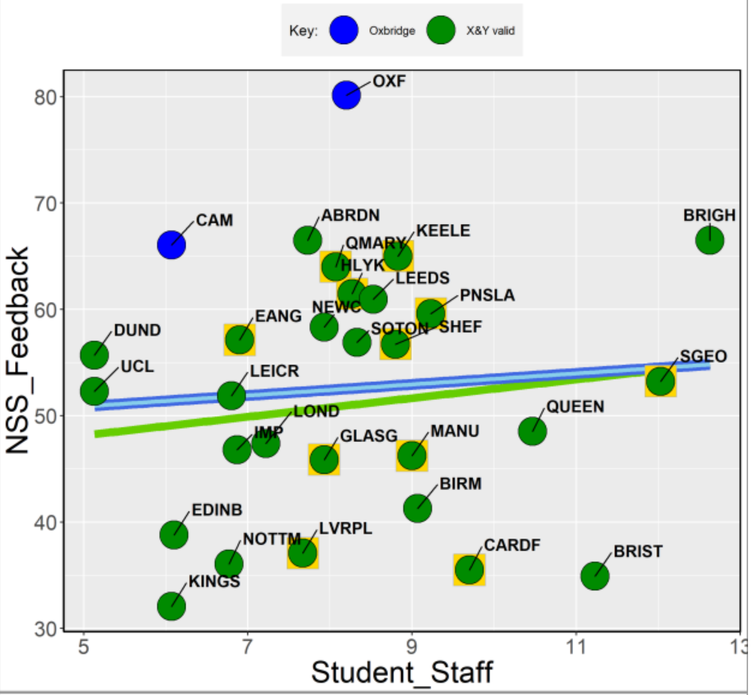

90/539 Y30: UKFPO_EPM X13: Student_Staff
r(all)= -0.308 p= 0.104 r(NonImp)= -0.308 Npairs=29 NimputedPairs=0

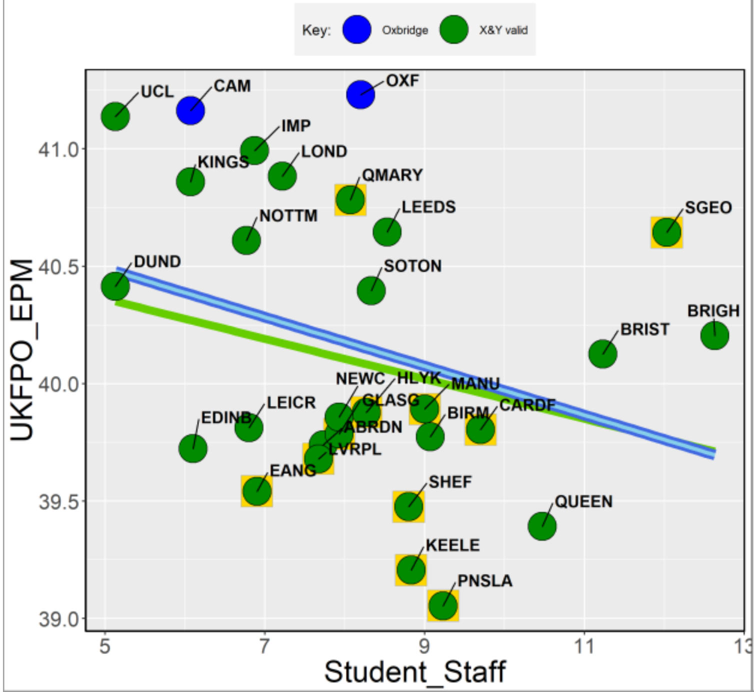

90/540 Y31: UKFPO_SJT X13: Student_Staff
r(all)= -0.256 p= 0.18 r(NonImp)= -0.256 Npairs=29 NimputedPairs=0

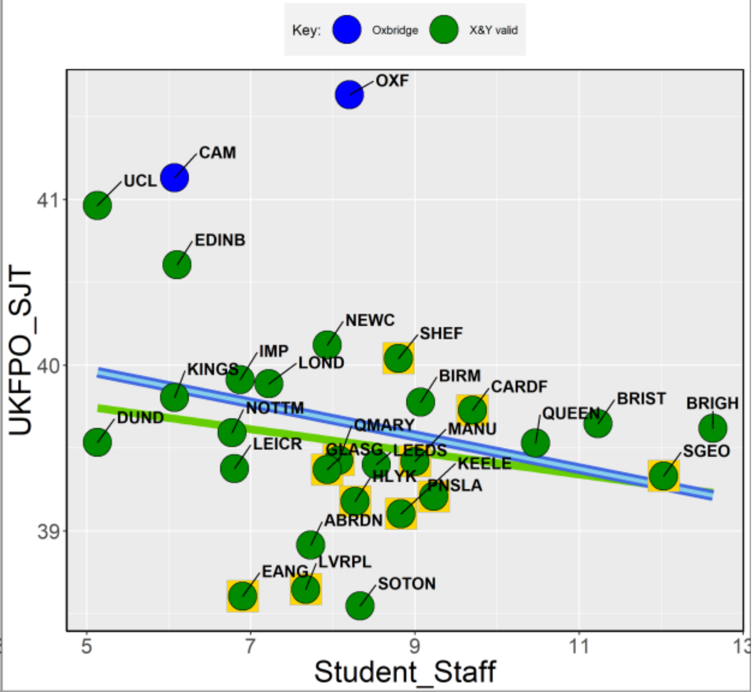

91/541 Y32: F1_Preparedness X13: Student_Staff
r(all)= 0.031 p= 0.874 r(NonImp)= 0.031 Npairs=29 NimputedPairs=0

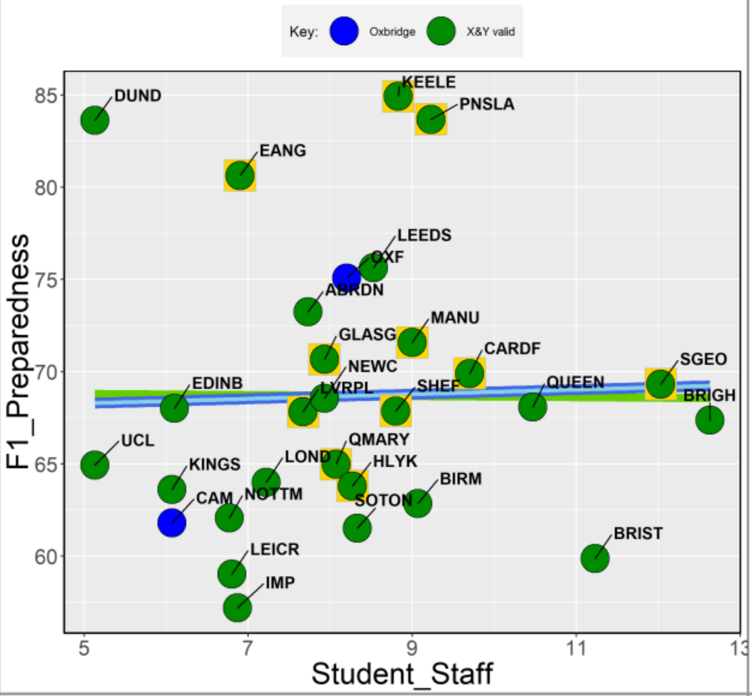

91/542 Y33: F1_Satisfn X13: Student_Staff
r(all)= 0.132 p= 0.493 r(NonImp)= 0.132 Npairs=29 NimputedPairs=0

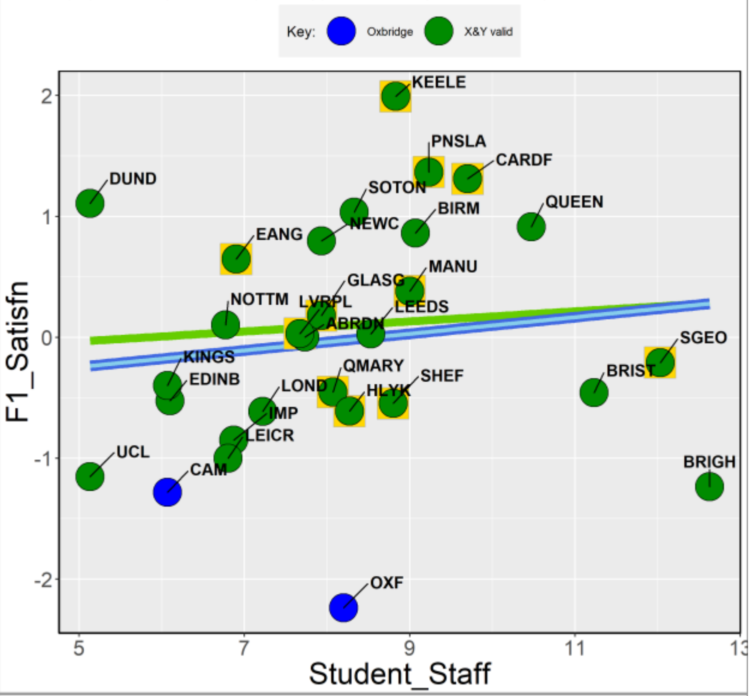

91/543 Y34: F1_Workload X13: Student_Staff
r(all)= 0.060 p= 0.757 r(NonImp)= 0.060 Npairs=29 NimputedPairs=0

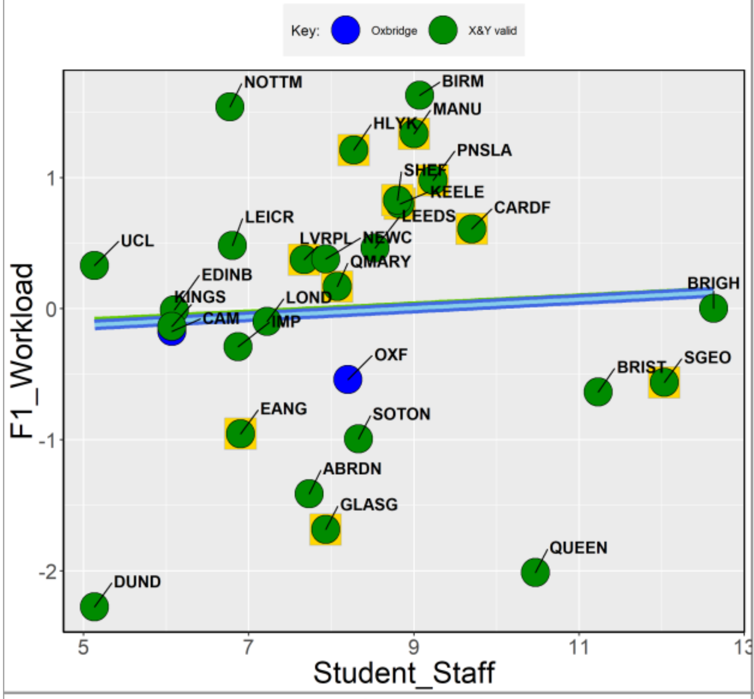

91/544 Y35: F1_Supervn X13: Student_Staff
r(all)= 0.145 p= 0.452 r(NonImp)= 0.145 Npairs=29 NimputedPairs=0

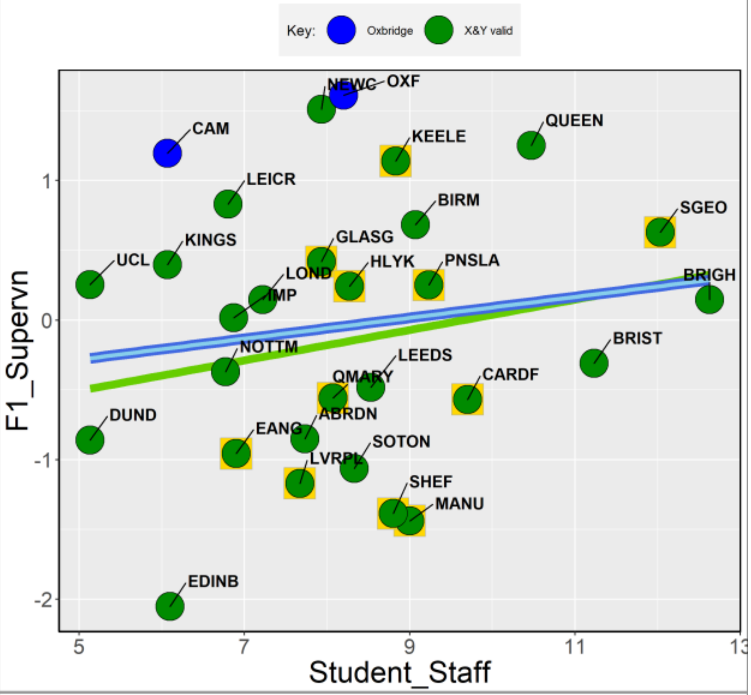

91/545 Y36: Trainee_GP X13: Student_Staff
r(all)= 0.068 p= 0.727 r(NonImp)= 0.068 Npairs=29 NimputedPairs=0

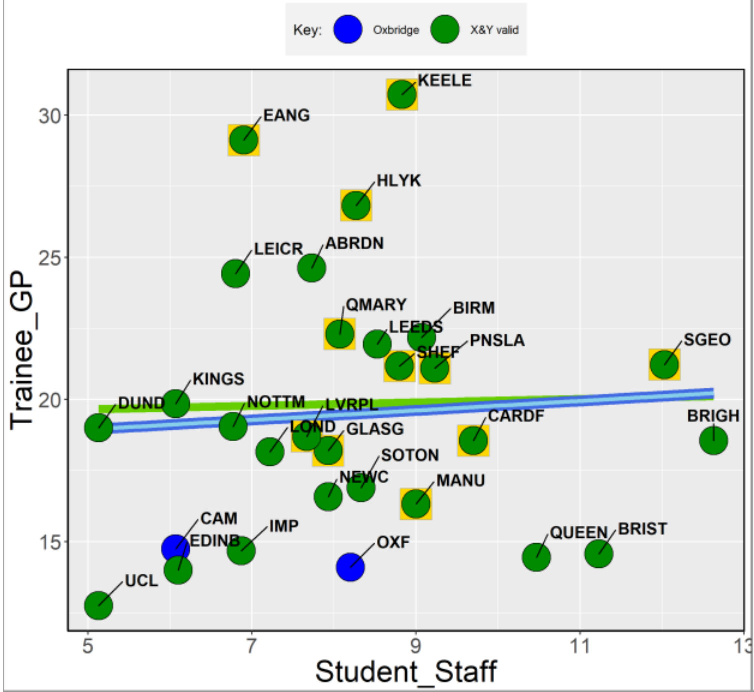

91/546 Y37: Trainee_Psyc X13: Student_Staff
r(all)= 0.142 p= 0.463 r(NonImp)= 0.142 Npairs=29 NimputedPairs=0

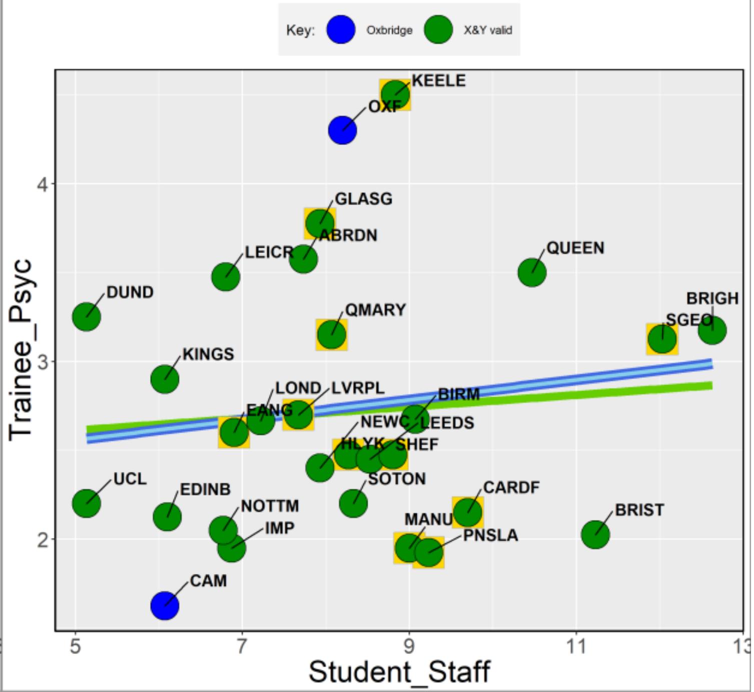

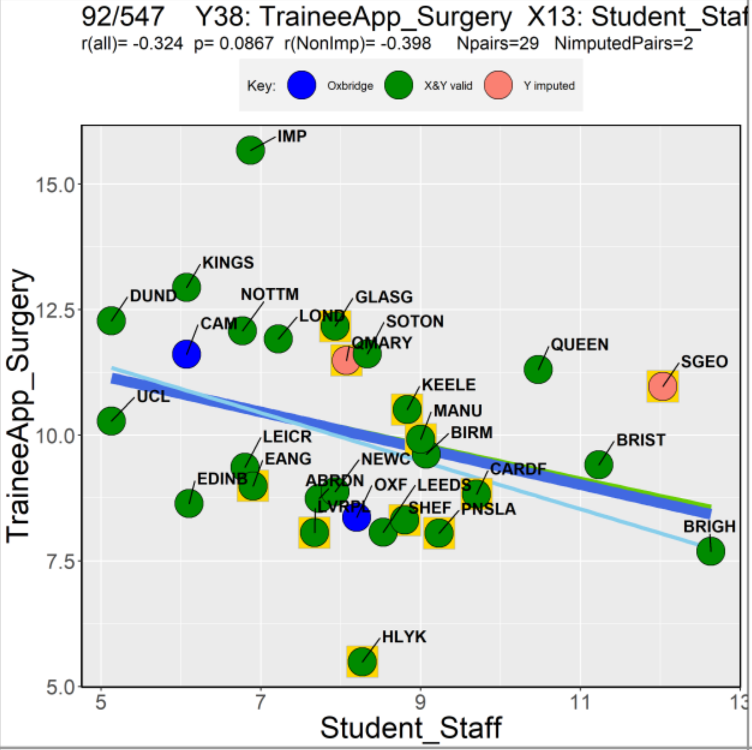
92/547 Y38: TraineeApp_Surgery X13: Student_Staf
r(all)= -0.324 p= 0.0867 r(NonImp)= -0.398 Npairs=29 NimputedPairs=2
Key: Oxbridge X&Y valid Y imputed
TraineeApp_Surgery
Student_Staff

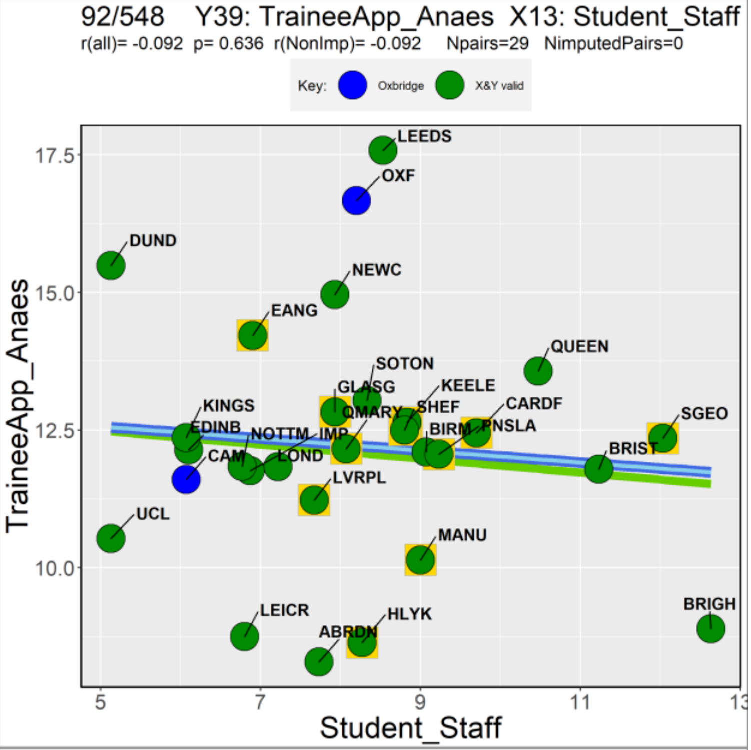
92/548 Y39: TraineeApp_Anaes X13: Student_Staff
r(all)= -0.092 p= 0.636 r(NonImp)= -0.092 Npairs=29 NimputedPairs=0
Key: Oxbridge X&Y valid
TraineeApp_Anaes
Student_Staff

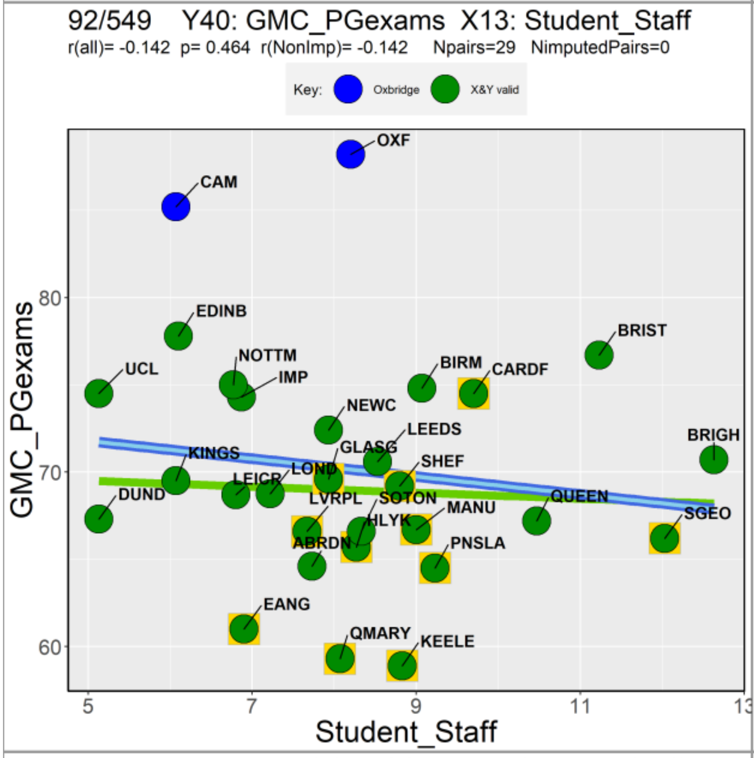
92/549 Y40: GMC_PGexams X13: Student_Staff
r(all)= -0.142 p= 0.464 r(NonImp)= -0.142 Npairs=29 NimputedPairs=0
Key: Oxbridge X&Y valid
GMC_PGexams
Student_Staff

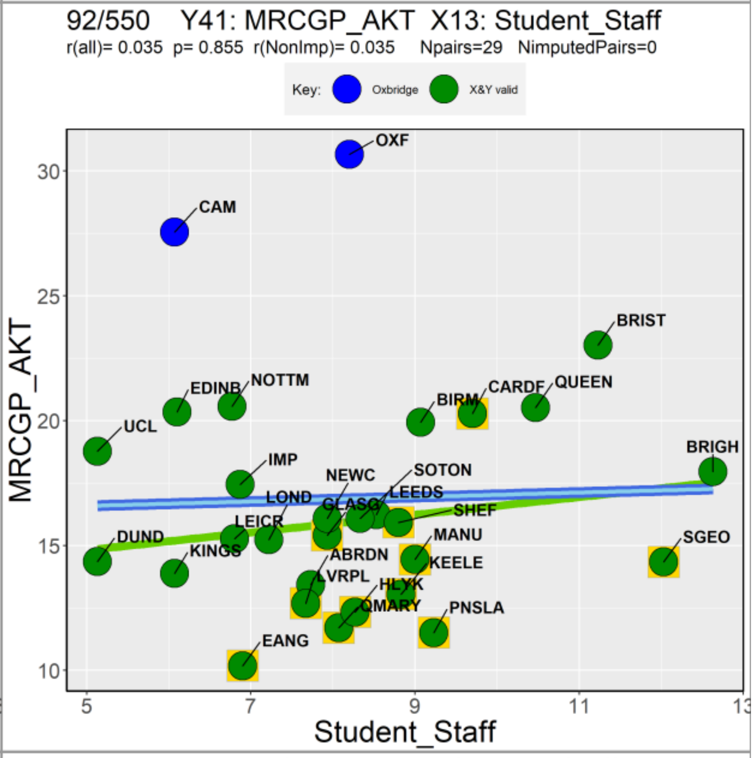
92/550 Y41: MRCGP_AKT X13: Student_Staff
r(all)= 0.035 p= 0.855 r(NonImp)= 0.035 Npairs=29 NimputedPairs=0
Key: Oxbridge X&Y valid
MRCGP_AKT
Student_Staff

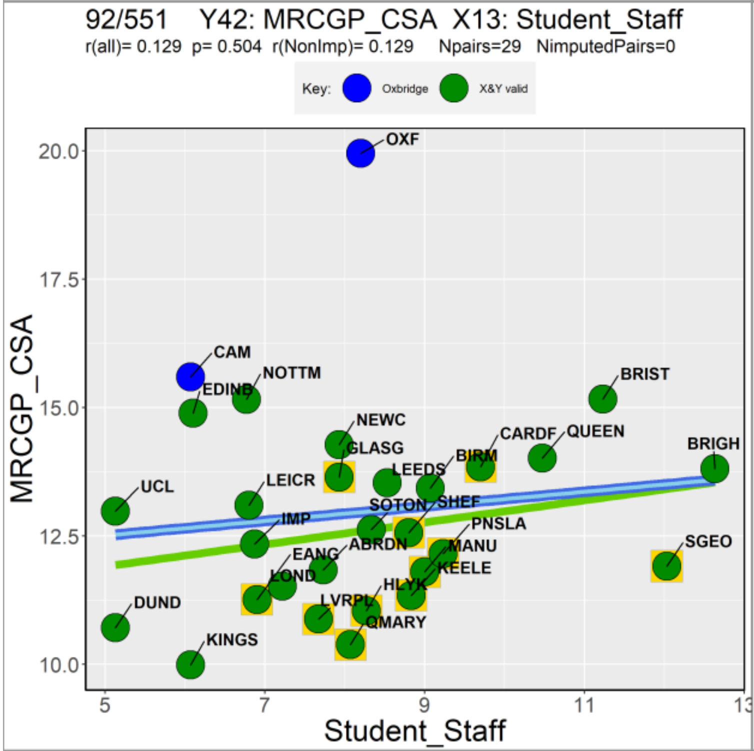
92/551 Y42: MRCGP_CSA X13: Student_Staff
r(all)= 0.129 p= 0.504 r(NonImp)= 0.129 Npairs=29 NimputedPairs=0
Key: Oxbridge X&Y valid
MRCGP_CSA
Student_Staff

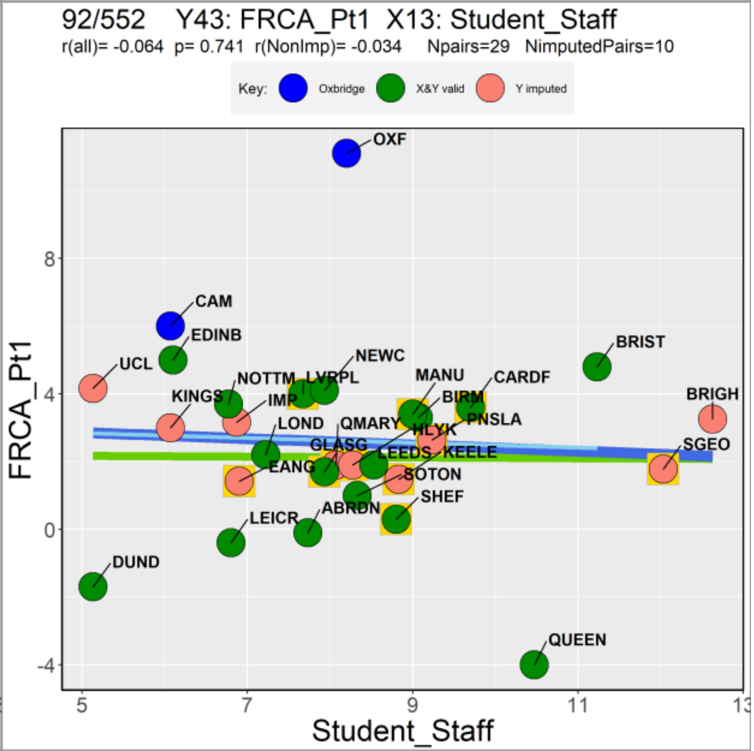
92/552 Y43: FRCA_Pt1 X13: Student_Staff
r(all)= -0.064 p= 0.741 r(NonImp)= -0.034 Npairs=29 NimputedPairs=10
Key: Oxbridge X&Y valid Y imputed
FRCA_Pt1
Student_Staff

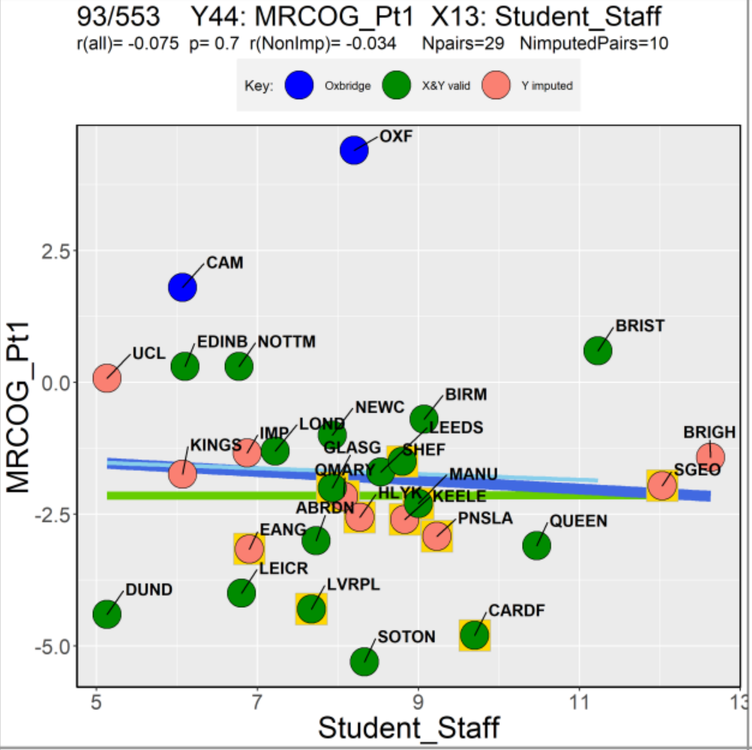
93/553 Y44: MRCOG_Pt1 X13: Student_Staff
r(all)= -0.075 p= 0.7 r(NonImp)= -0.034 Npairs=29 NimputedPairs=10
Key: Oxbridge X&Y valid Y imputed
MRCOG_Pt1
Student_Staff
2.5
0.0
-2.5
-5.0
5
7
9
11
13
OXF
CAM
UCL
EDINB
NOTTM
BRIST
BIRM
NEWC
LOND
LEEDS
IMP
KINGS
GLASG
SHEF
BRIGH
QMARY
MANU
SGEO
HLYK
KEELE
ABRDN
PNSLA
QUEEN
EANG
LEICR
DUND
LVRPL
CARDF
SOTON

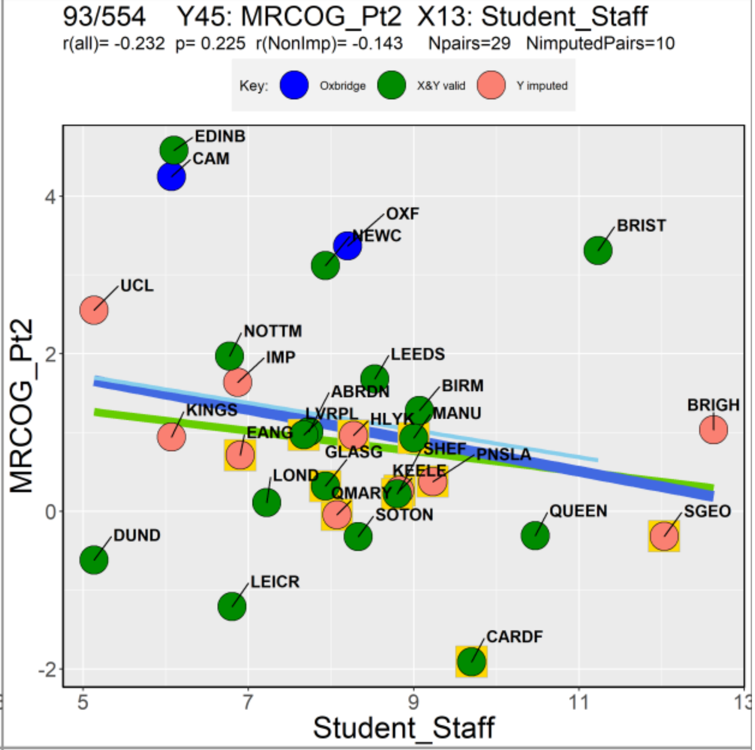
93/554 Y45: MRCOG_Pt2 X13: Student_Staff
r(all)= -0.232 p= 0.225 r(NonImp)= -0.143 Npairs=29 NimputedPairs=10
Key: Oxbridge X&Y valid Y imputed
MRCOG_Pt2
Student_Staff
4
2
0
-2
5
7
9
11
13
EDINB
CAM
OXF
NEWC
BRIST
UCL
NOTTM
IMP
LEEDS
BIRM
ABRDN
BRIGH
KINGS
LVRPL
HLYK
MANU
EANG
GLASG
SHEF
PNSLA
KEELE
LOND
QMARY
SOTON
QUEEN
SGEO
DUND
LEICR
CARDF

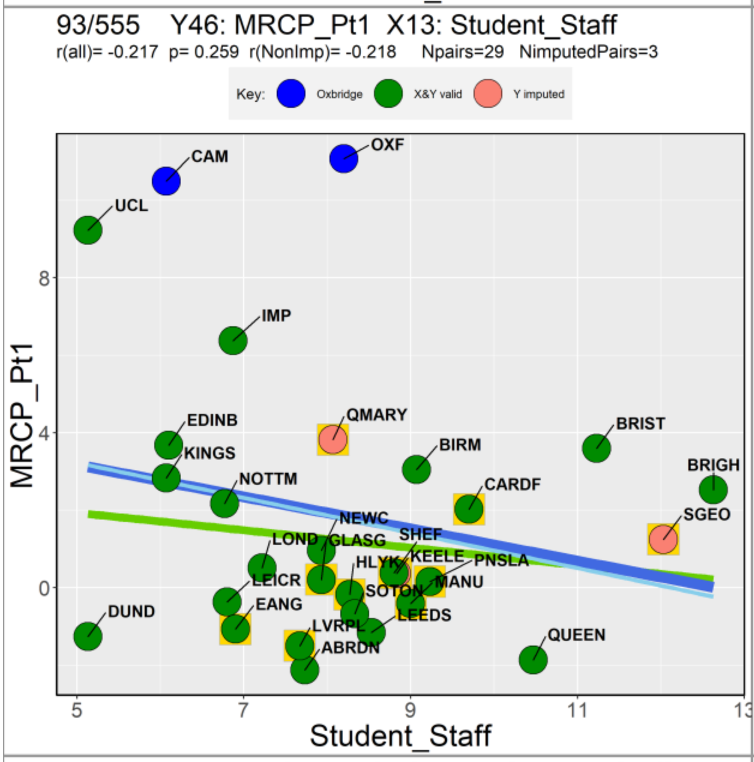
93/555 Y46: MRCP_Pt1 X13: Student_Staff
r(all)= -0.217 p= 0.259 r(NonImp)= -0.218 Npairs=29 NimputedPairs=3
Key: Oxbridge X&Y valid Y imputed
MRCP_Pt1
Student_Staff
8
4
0
5
7
9
11
13
OXF
CAM
UCL
IMP
QMARY
EDINB
BRIST
KINGS
BIRM
NOTTM
CARDF
BRIGH
NEWC
SGEO
LOND
GLASG
SHEF
HLYK
KEELE
PNSLA
LEICR
SOTON
MANU
EANG
DUND
LVRPL
LEEDS
ABRDN
QUEEN

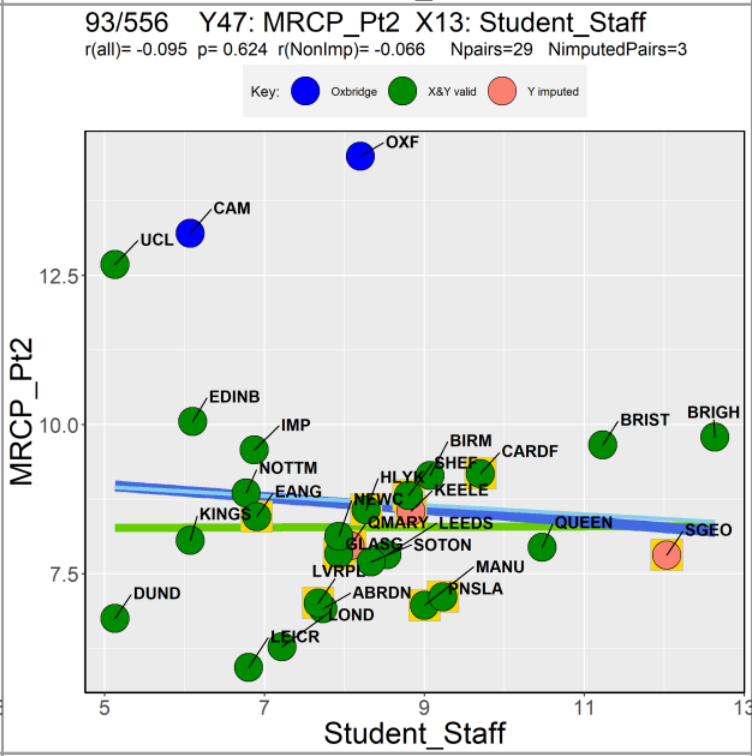
93/556 Y47: MRCP_Pt2 X13: Student_Staff
r(all)= -0.095 p= 0.624 r(NonImp)= -0.066 Npairs=29 NimputedPairs=3
Key: Oxbridge X&Y valid Y imputed
MRCP_Pt2
Student_Staff
12.5
10.0
7.5
5
7
9
11
13
OXF
CAM
UCL
EDINB
IMP
BRIST
BRIGH
BIRM
NOTTM
SHEF
CARDF
HLYK
EANG
NEWC
KEELE
KINGS
QMARY
LEEDS
QUEEN
SGEO
GLASG
SOTON
LVRPL
MANU
DUND
ABRDN
PNSLA
LOND
LEICR

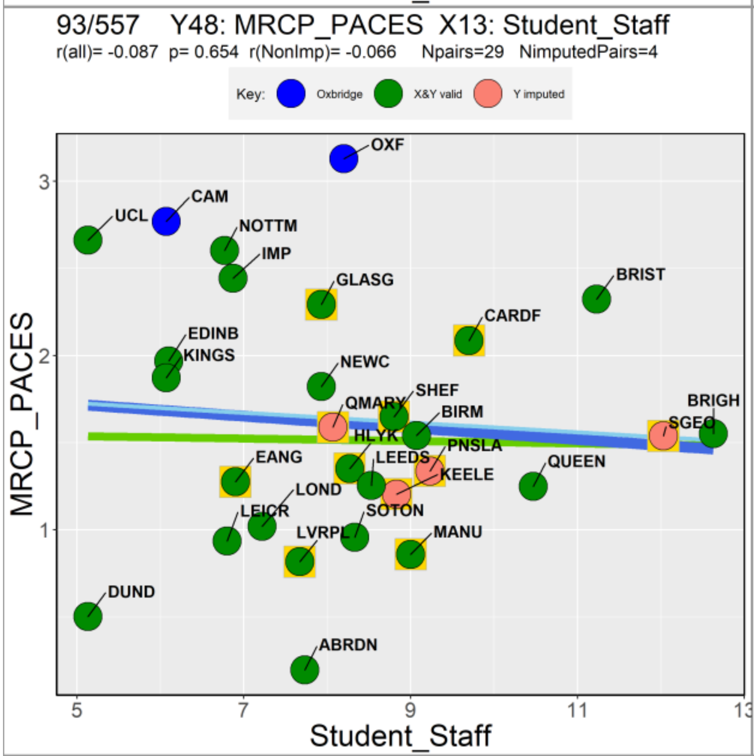
93/557 Y48: MRCP_PACES X13: Student_Staff
r(all)= -0.087 p= 0.654 r(NonImp)= -0.066 Npairs=29 NimputedPairs=4
Key: Oxbridge X&Y valid Y imputed
MRCP_PACES
Student_Staff
3
2
1
5
7
9
11
13
OXF
CAM
UCL
NOTTM
IMP
GLASG
BRIST
CARDF
EDINB
KINGS
NEWC
QMARY
SHEF
BIRM
BRIGH
SGEO
HLYK
PNSLA
EANG
LEEDS
KEELE
QUEEN
LOND
LEICR
SOTON
LVRPL
MANU
DUND
ABRDN

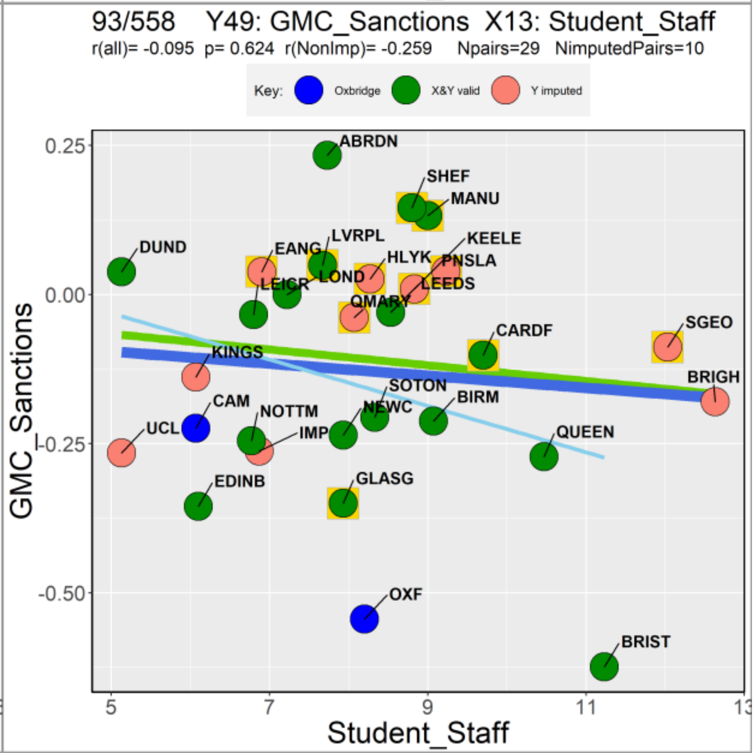
93/558 Y49: GMC_Sanctions X13: Student_Staff
r(all)= -0.095 p= 0.624 r(NonImp)= -0.259 Npairs=29 NimputedPairs=10
Key: Oxbridge X&Y valid Y imputed
GMC_Sanctions
Student_Staff
0.25
0.00
-0.25
-0.50
5
7
9
11
13
ABRDN
SHEF
MANU
DUND
EANG
LVRPL
KEELE
HLYK
PNSLA
LEICR
LOND
LEEDS
QMARY
CARDF
SGEO
KINGS
SOTON
BRIGH
CAM
NOTTM
NEWC
BIRM
UCL
IMP
QUEEN
EDINB
GLASG
OXF
BRIST

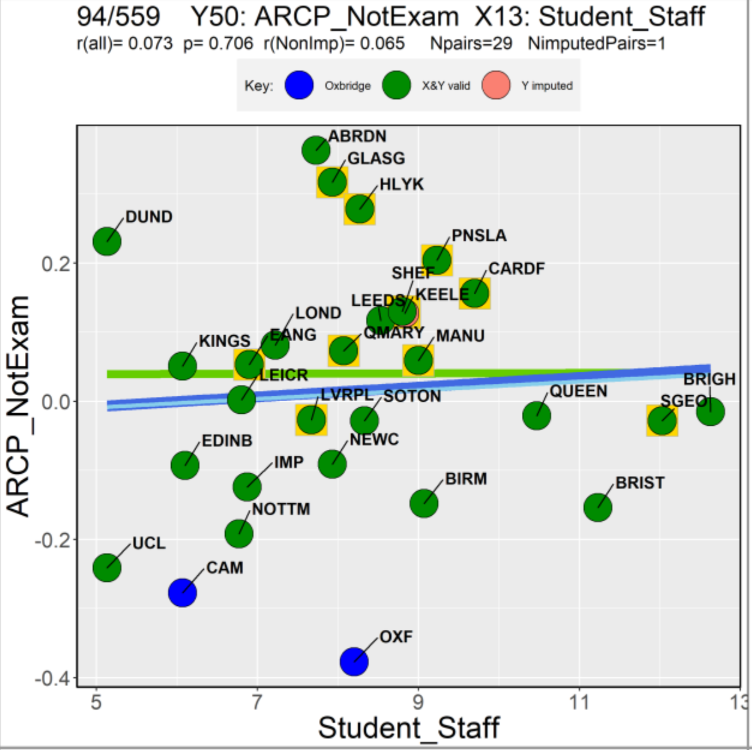

94/559 Y50: ARCP_NotExam X13: Student_Staff
r(all)= 0.073 p= 0.706 r(NonImp)= 0.065 Npairs=29 NimputedPairs=1
Key: Oxbridge X&Y valid Y imputed
ABRDN
GLASG
HLYK
DUND
PNSLA
SHEF
CARDF
LEEDS
KEELE
LOND
KINGS
EANG
QMARY
MANU
LEICR
BRIGH
LVRPL
SOTON
QUEEN
SGEO
EDINB
NEWC
IMP
BIRM
BRIST
NOTTM
UCL
CAM
OXF
ARCP_NotExam
Student_Staff

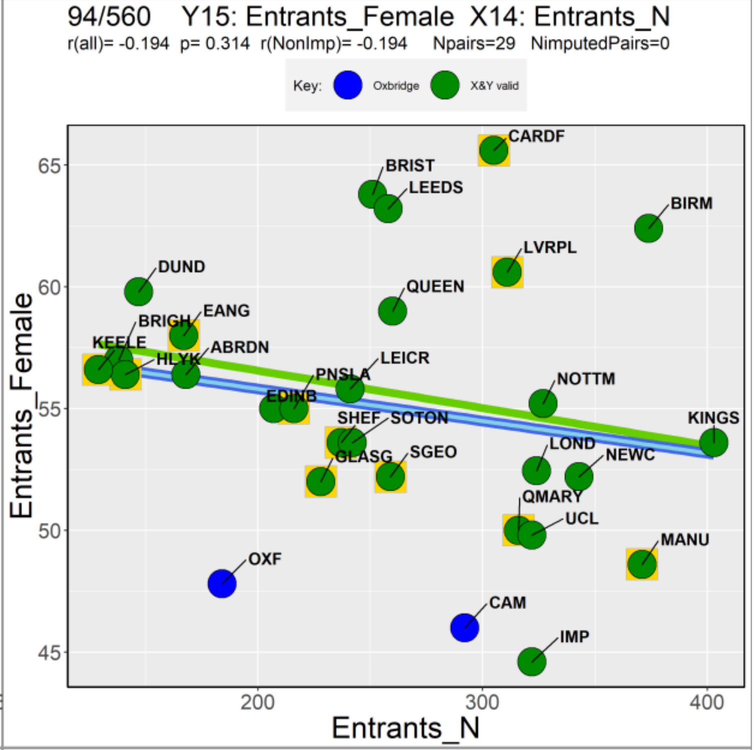

94/560 Y15: Entrants_Female X14: Entrants_N
r(all)= -0.194 p= 0.314 r(NonImp)= -0.194 Npairs=29 NimputedPairs=0
Key: Oxbridge X&Y valid
CARDF
BRIST
LEEDS
BIRM
LVRPL
DUND
QUEEN
BRIGH
EANG
KEELE
ABRDN
HLYK
LEICR
PNSLA
NOTTM
EDINB
SHEF
SOTON
KINGS
LOND
NEWC
GLASG
SGEO
QMARY
UCL
MANU
OXF
CAM
IMP
Entrants_Female
Entrants_N

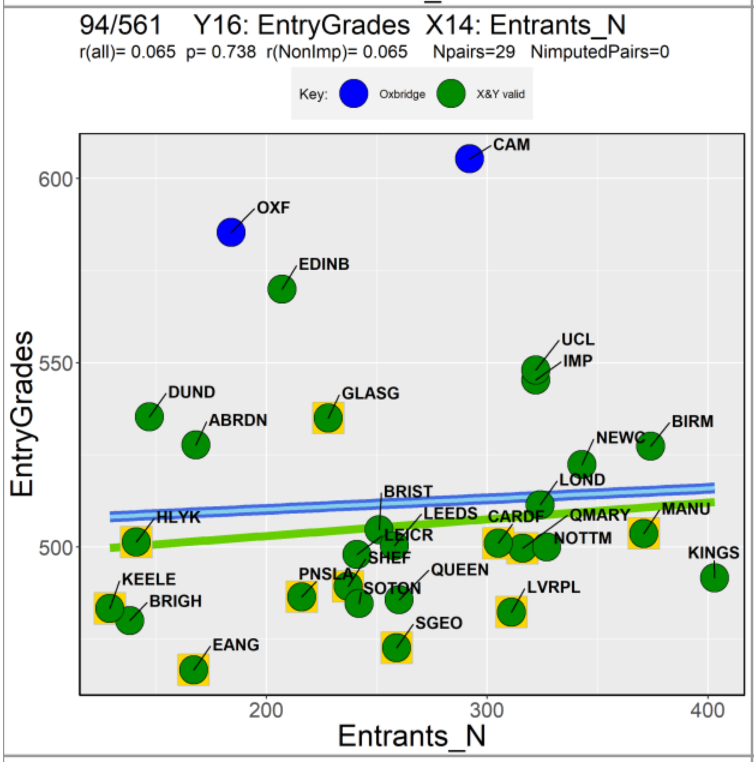

94/561 Y16: EntryGrades X14: Entrants_N
r(all)= 0.065 p= 0.738 r(NonImp)= 0.065 Npairs=29 NimputedPairs=0
Key: Oxbridge X&Y valid
CAM
OXF
EDINB
UCL
IMP
DUND
GLASG
ABRDN
BIRM
NEWC
LOND
BRIST
HLYK
LEEDS
CARDF
QMARY
MANU
LEICR
NOTTM
SHEF
KINGS
PNSLA
QUEEN
KEELE
SOTON
BRIGH
LVRPL
SGEO
EANG
EntryGrades
Entrants_N

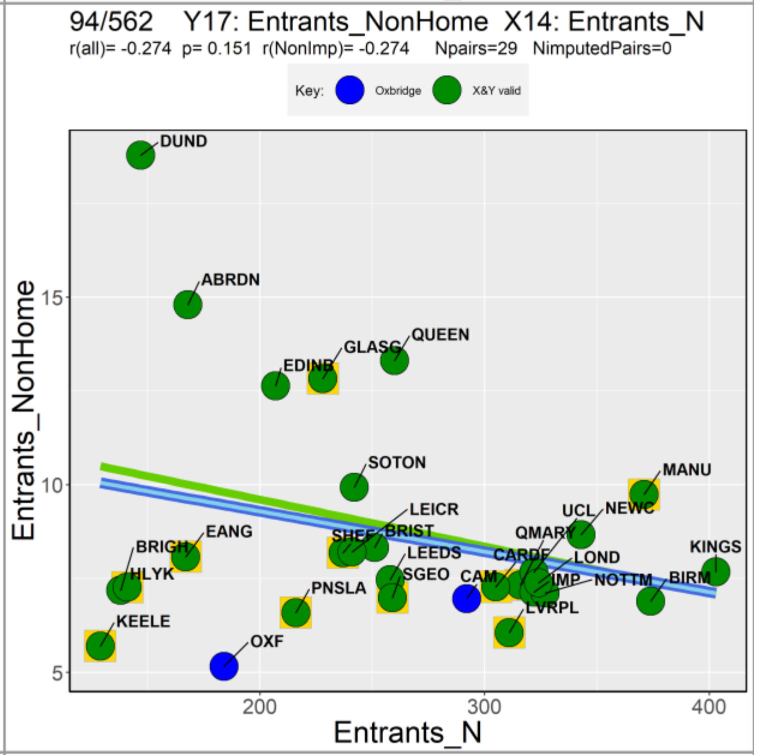

94/562 Y17: Entrants_NonHome X14: Entrants_N
r(all)= -0.274 p= 0.151 r(NonImp)= -0.274 Npairs=29 NimputedPairs=0
Key: Oxbridge X&Y valid
DUND
ABRDN
GLASG
QUEEN
EDINB
SOTON
MANU
LEICR
UCL
NEWC
EANG
BRIST
QMARY
BRIGH
SHEF
KINGS
LEEDS
CARDF
LOND
HLYK
SGEO
CAM
IMP
NOTTM
BIRM
PNSLA
LVRPL
KEELE
OXF
Entrants_NonHome
Entrants_N

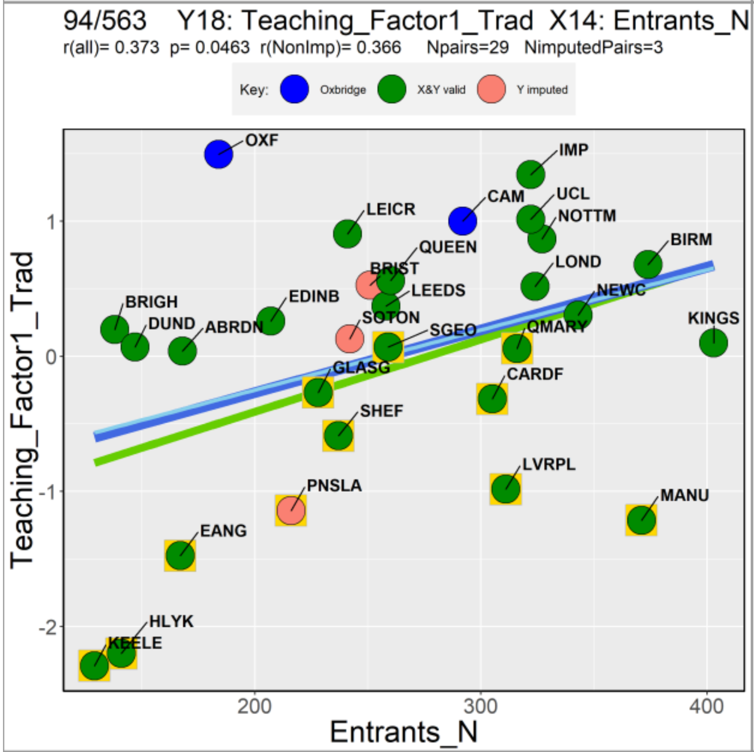

94/563 Y18: Teaching_Factor1_Trad X14: Entrants_N
r(all)= 0.373 p= 0.0463 r(NonImp)= 0.366 Npairs=29 NimputedPairs=3
Key: Oxbridge X&Y valid Y imputed
OXF
IMP
CAM
UCL
LEICR
NOTTM
QUEEN
BIRM
BRIST
LOND
LEEDS
NEWC
BRIGH
EDINB
DUND
ABRDN
SOTON
SGEO
QMARY
KINGS
GLASG
CARDF
SHEF
LVRPL
PNSLA
MANU
EANG
HLYK
KEELE
Teaching_Factor1_Trad
Entrants_N

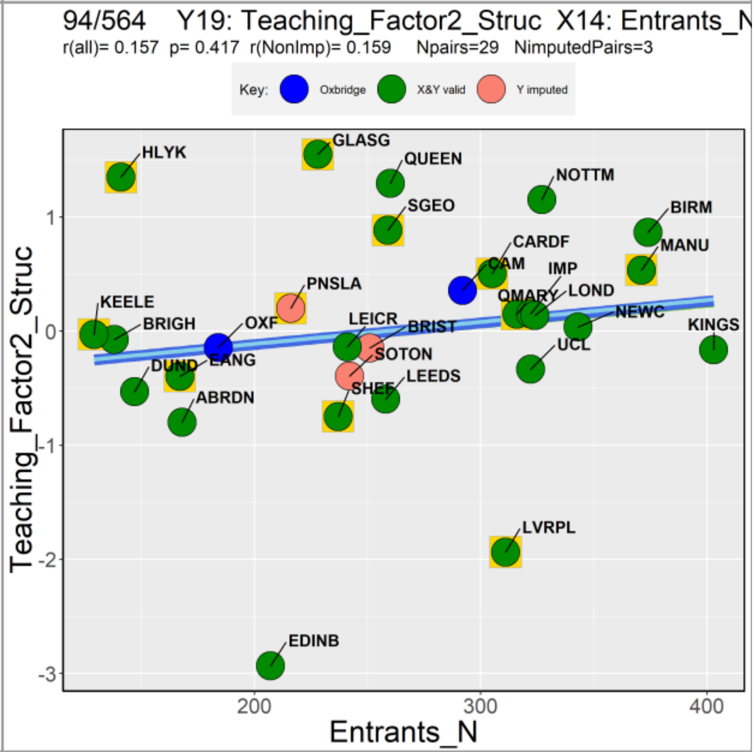

94/564 Y19: Teaching_Factor2_Struc X14: Entrants_N
r(all)= 0.157 p= 0.417 r(NonImp)= 0.159 Npairs=29 NimputedPairs=3
Key: Oxbridge X&Y valid Y imputed
HLYK
GLASG
QUEEN
NOTTM
SGEO
BIRM
CARDF
MANU
CAM
IMP
PNSLA
QMARY
LOND
KEELE
NEWC
BRIGH
OXF
LEICR
BRIST
KINGS
UCL
DUND
EANG
SOTON
LEEDS
SHEF
ABRDN
LVRPL
EDINB
Teaching_Factor2_Struc
Entrants_N

95/565 Y20: Teach_GP X14: Entrants_N
r(all)= -0.328 p= 0.0826 r(NonImp)= -0.319 Npairs=29 NimputedPairs=3

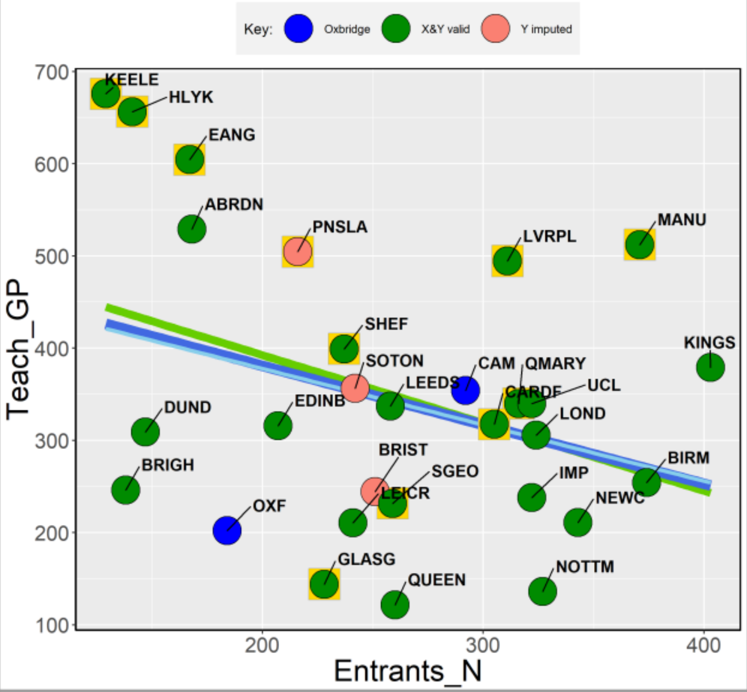

95/566 Y21: Teach_Psyc X14: Entrants_N
r(all)= -0.205 p= 0.285 r(NonImp)= -0.210 Npairs=29 NimputedPairs=3

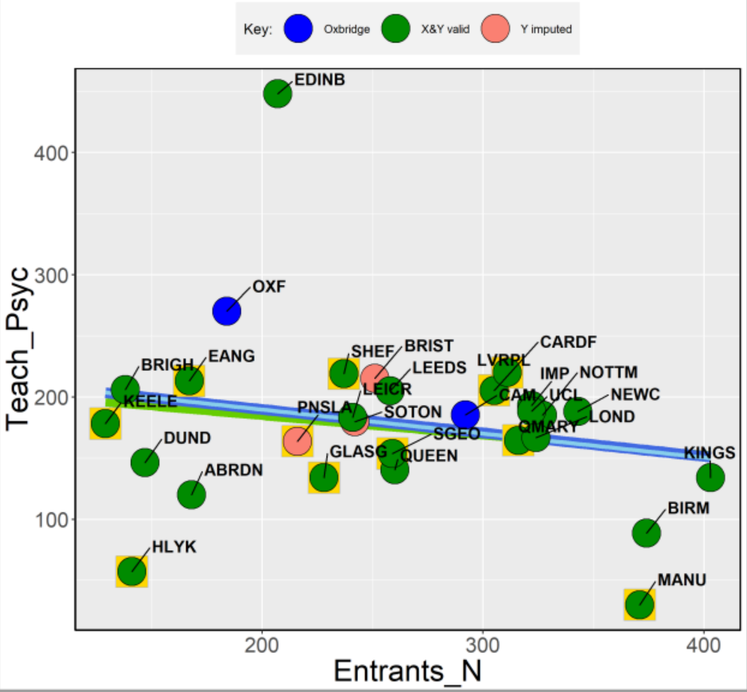

95/567 Y22: Teach_Anaes X14: Entrants_N
r(all)= 0.247 p= 0.197 r(NonImp)= 0.235 Npairs=29 NimputedPairs=3

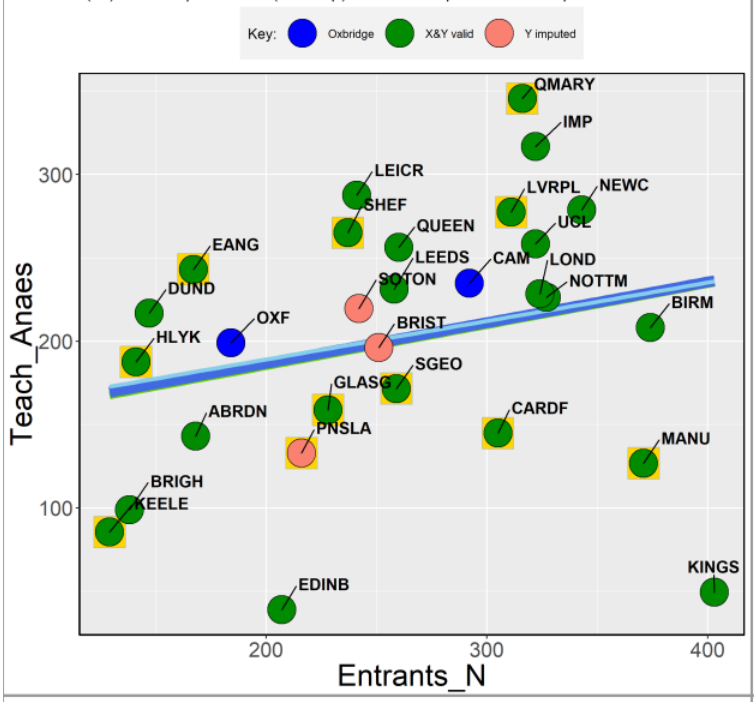

95/568 Y23: Teach_OG X14: Entrants_N
r(all)= 0.104 p= 0.591 r(NonImp)= 0.099 Npairs=29 NimputedPairs=3

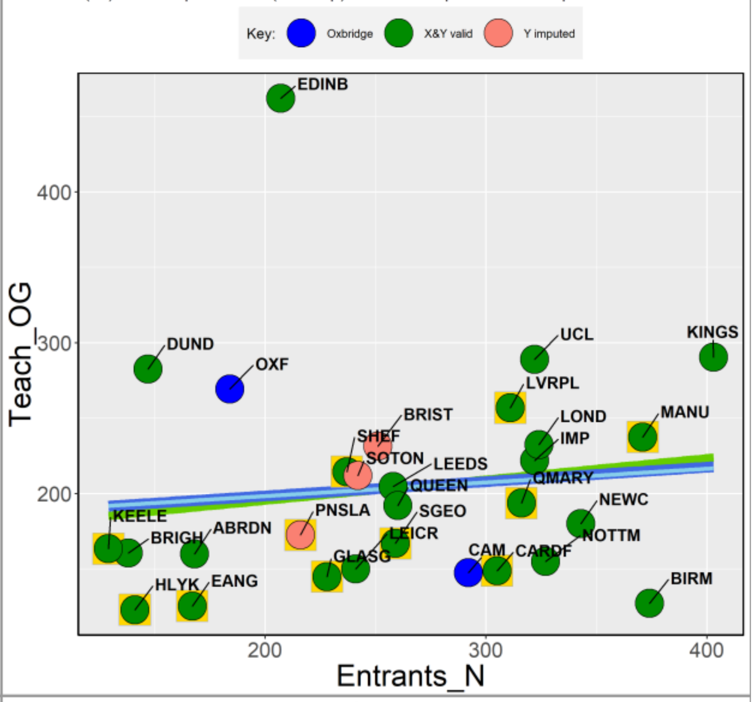

95/569 Y24: Teach_IntMed X14: Entrants_N
r(all)= 0.065 p= 0.739 r(NonImp)= 0.051 Npairs=29 NimputedPairs=3

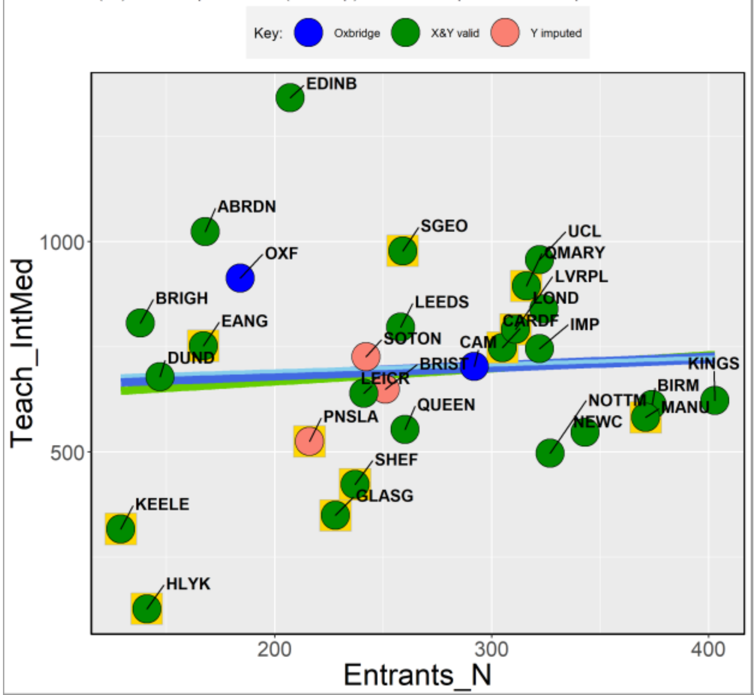

95/570 Y25: Teach_Surgery X14: Entrants_N
r(all)= -0.114 p= 0.554 r(NonImp)= -0.137 Npairs=29 NimputedPairs=3

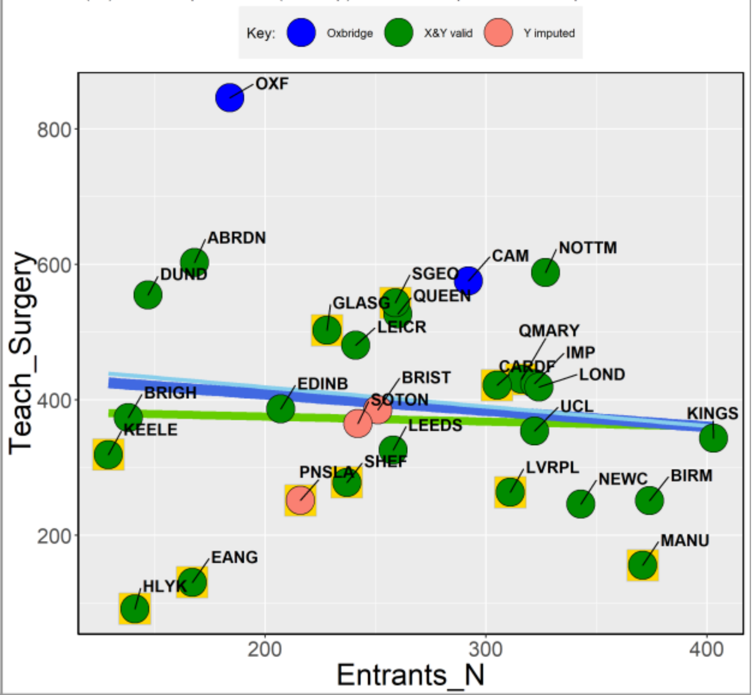

96/571 Y26: ExamTime X14: Entrants_N
r(all)= 0.340 p= 0.0713 r(NonImp)= 0.322 Npairs=29 NimputedPairs=3

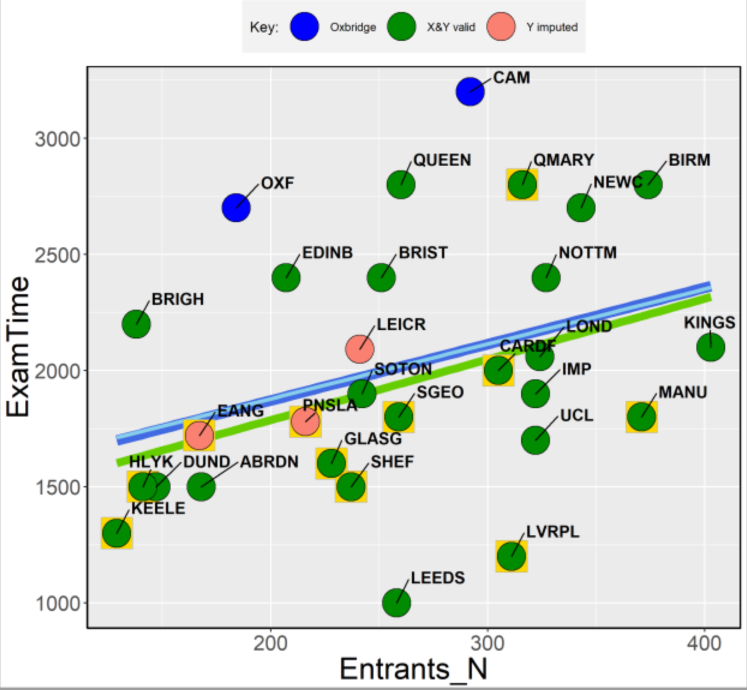

96/572 Y27: SelfRegLearn X14: Entrants_N
r(all)= -0.151 p= 0.435 r(NonImp)= -0.151 Npairs=29 NimputedPairs=0

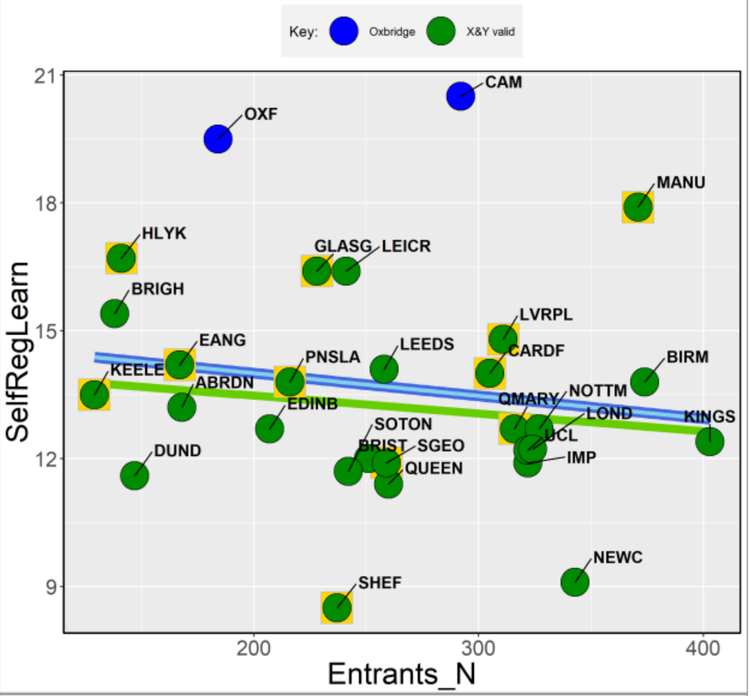

96/573 Y28: NSS_Satisfn X14: Entrants_N
r(all)= -0.614 p= 0.000399 r(NonImp)= -0.614 Npairs=29 NimputedPairs=0

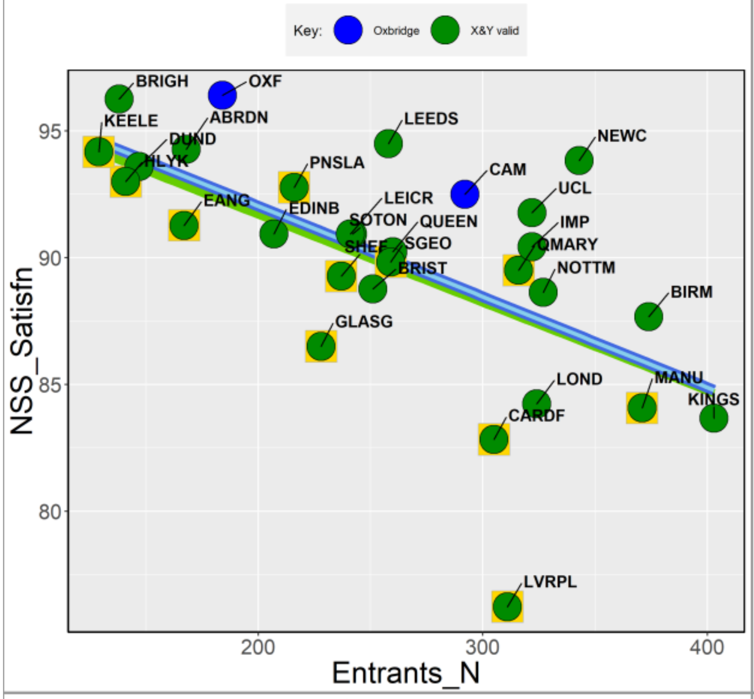

96/574 Y29: NSS_Feedback X14: Entrants_N
r(all)= -0.574 p= 0.00114 r(NonImp)= -0.574 Npairs=29 NimputedPairs=0

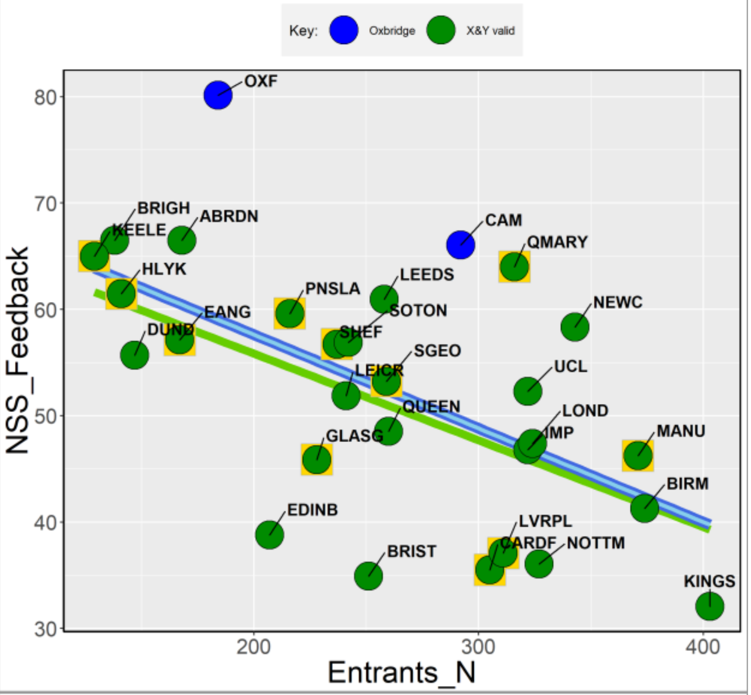

96/575 Y30: UKFPO_EPM X14: Entrants_N
r(all)= 0.338 p= 0.0728 r(NonImp)= 0.338 Npairs=29 NimputedPairs=0

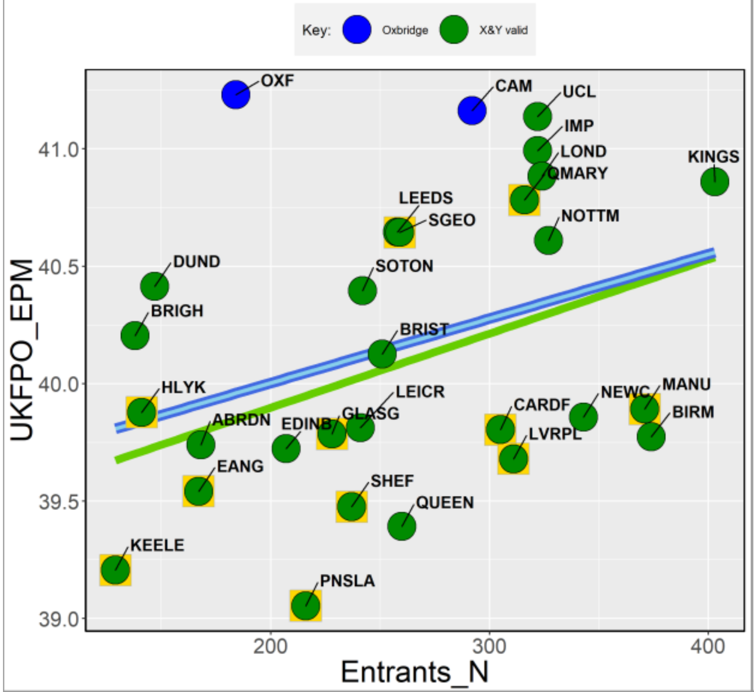

96/576 Y31: UKFPO_SJT X14: Entrants_N
r(all)= 0.195 p= 0.311 r(NonImp)= 0.195 Npairs=29 NimputedPairs=0

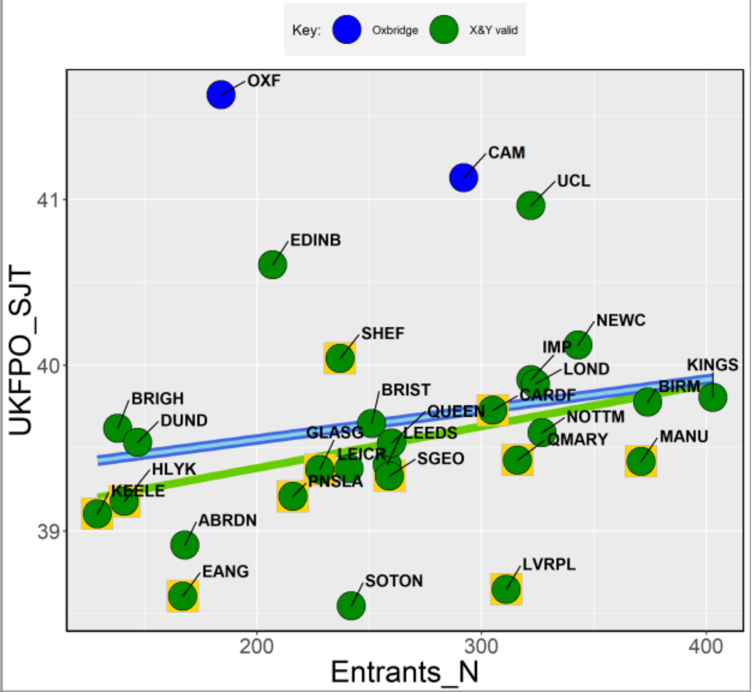

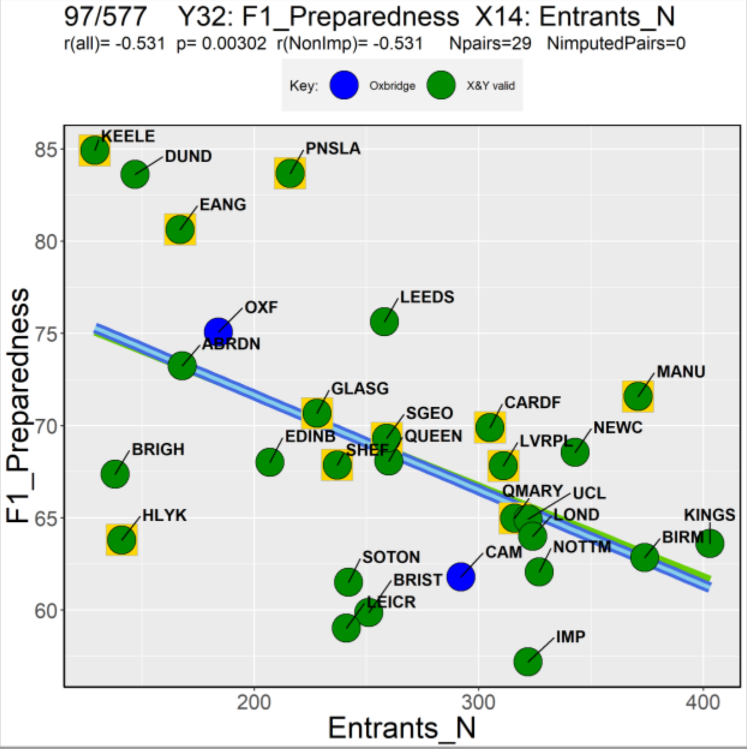
97/577 Y32: F1_Preparedness X14: Entrants_N
r(all)= -0.531 p= 0.00302 r(NonImp)= -0.531 Npairs=29 NimputedPairs=0
Key: Oxbridge X&Y valid
F1_Preparedness
Entrants_N

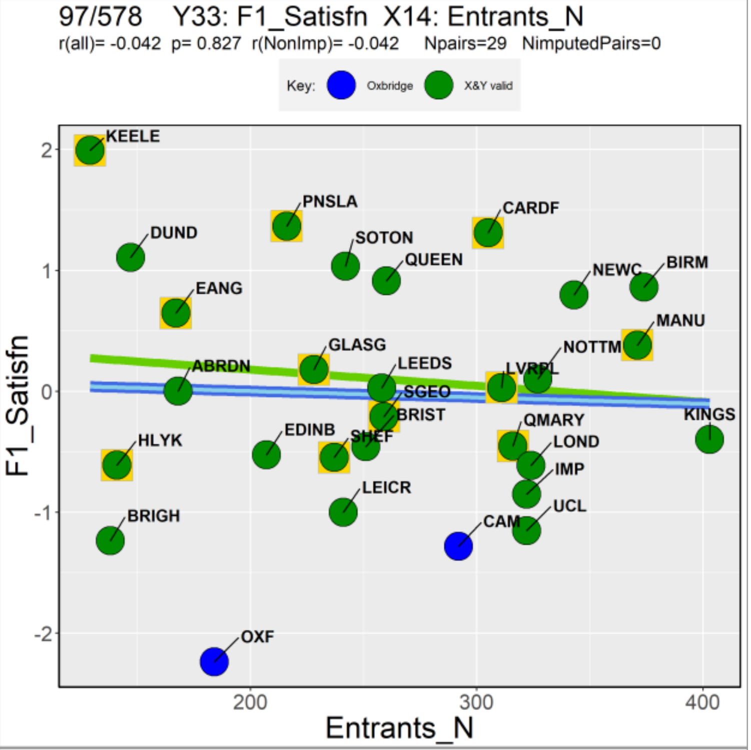
97/578 Y33: F1_Satisfn X14: Entrants_N
r(all)= -0.042 p= 0.827 r(NonImp)= -0.042 Npairs=29 NimputedPairs=0
Key: Oxbridge X&Y valid
F1_Satisfn
Entrants_N

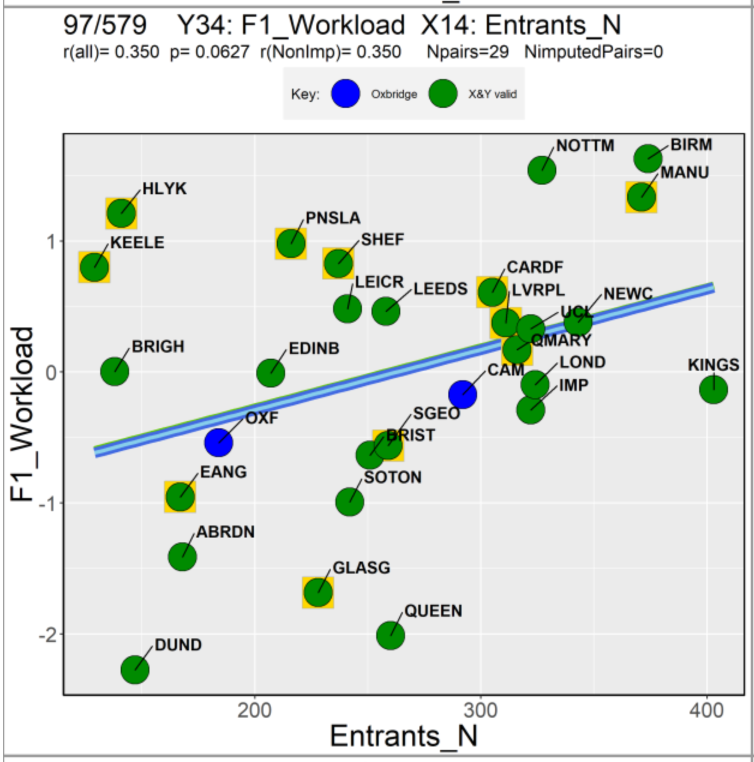
97/579 Y34: F1_Workload X14: Entrants_N
r(all)= 0.350 p= 0.0627 r(NonImp)= 0.350 Npairs=29 NimputedPairs=0
Key: Oxbridge X&Y valid
F1_Workload
Entrants_N

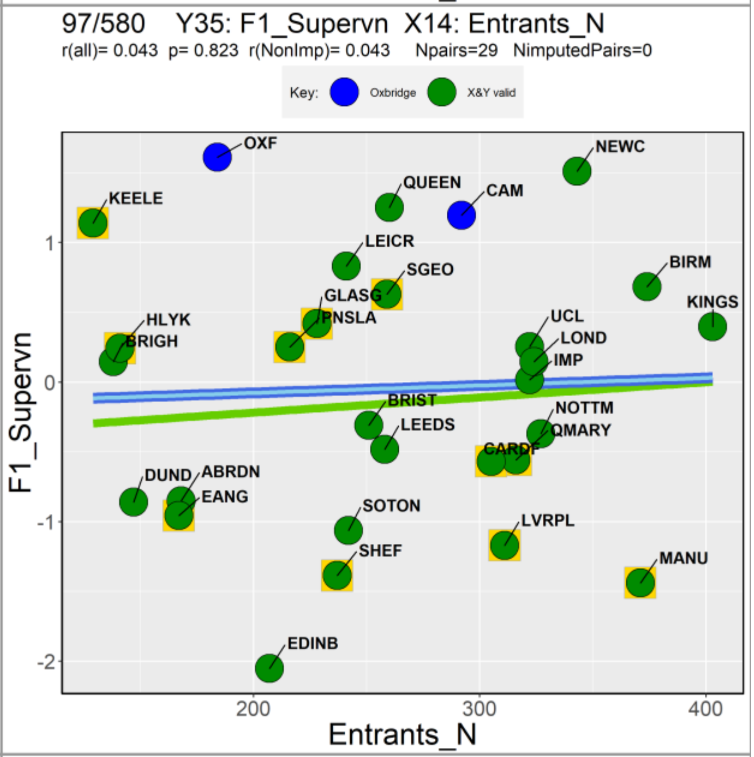
97/580 Y35: F1_Supervn X14: Entrants_N
r(all)= 0.043 p= 0.823 r(NonImp)= 0.043 Npairs=29 NimputedPairs=0
Key: Oxbridge X&Y valid
F1_Supervn
Entrants_N

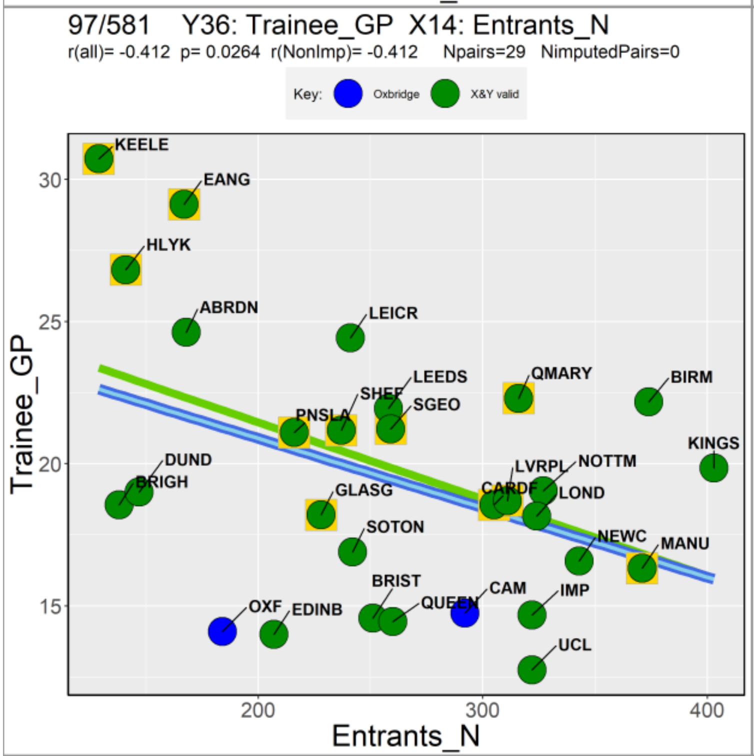
97/581 Y36: Trainee_GP X14: Entrants_N
r(all)= -0.412 p= 0.0264 r(NonImp)= -0.412 Npairs=29 NimputedPairs=0
Key: Oxbridge X&Y valid
Trainee_GP
Entrants_N

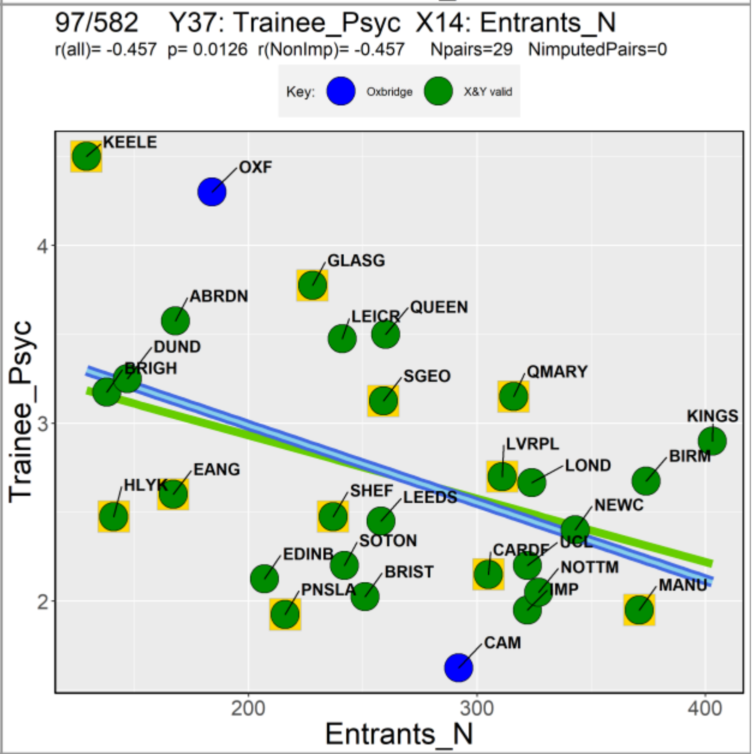
97/582 Y37: Trainee_Psyc X14: Entrants_N
r(all)= -0.457 p= 0.0126 r(NonImp)= -0.457 Npairs=29 NimputedPairs=0
Key: Oxbridge X&Y valid
Trainee_Psyc
Entrants_N

98/583 Y38: TraineeApp_Surgery X14: Entrants_N
r(all)= 0.412 p= 0.0264 r(NonImp)= 0.401 Npairs=29 NimputedPairs=2

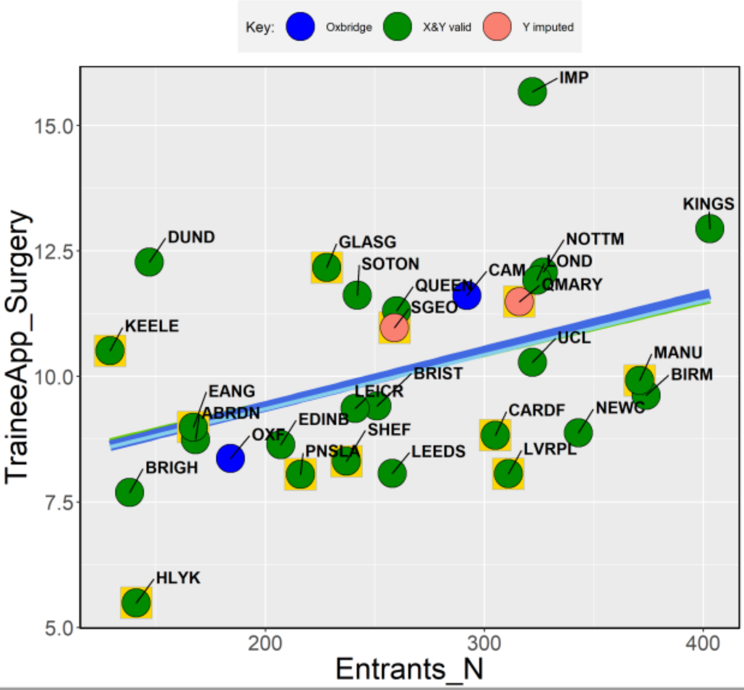

98/584 Y39: TraineeApp_Anaes X14: Entrants_N
r(all)= 0.006 p= 0.973 r(NonImp)= 0.006 Npairs=29 NimputedPairs=0

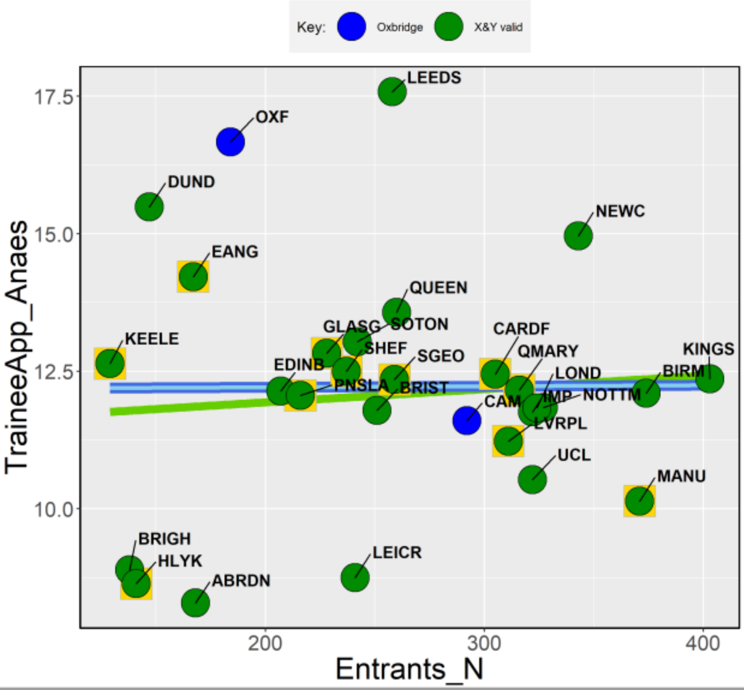

98/585 Y40: GMC_PGexams X14: Entrants_N
r(all)= 0.213 p= 0.268 r(NonImp)= 0.213 Npairs=29 NimputedPairs=0

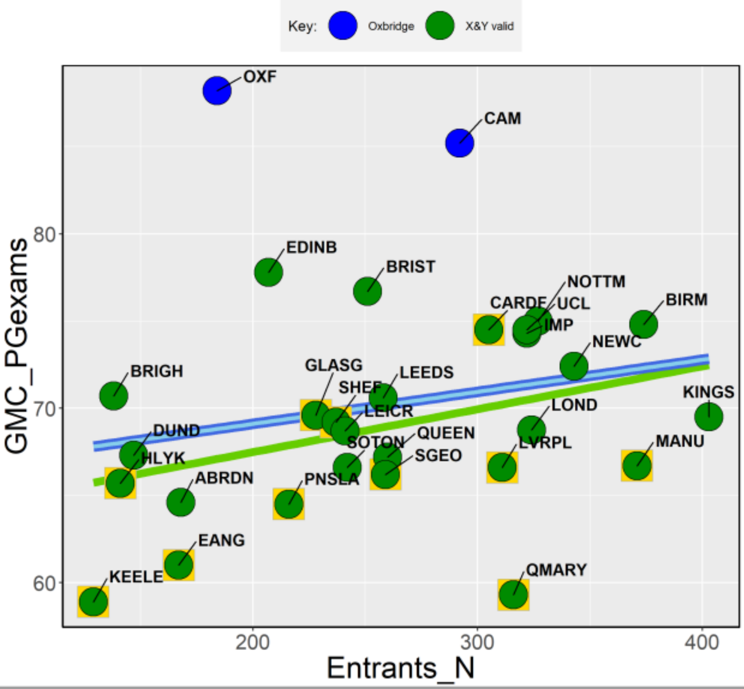

98/586 Y41: MRCGP_AKT X14: Entrants_N
r(all)= 0.106 p= 0.583 r(NonImp)= 0.106 Npairs=29 NimputedPairs=0

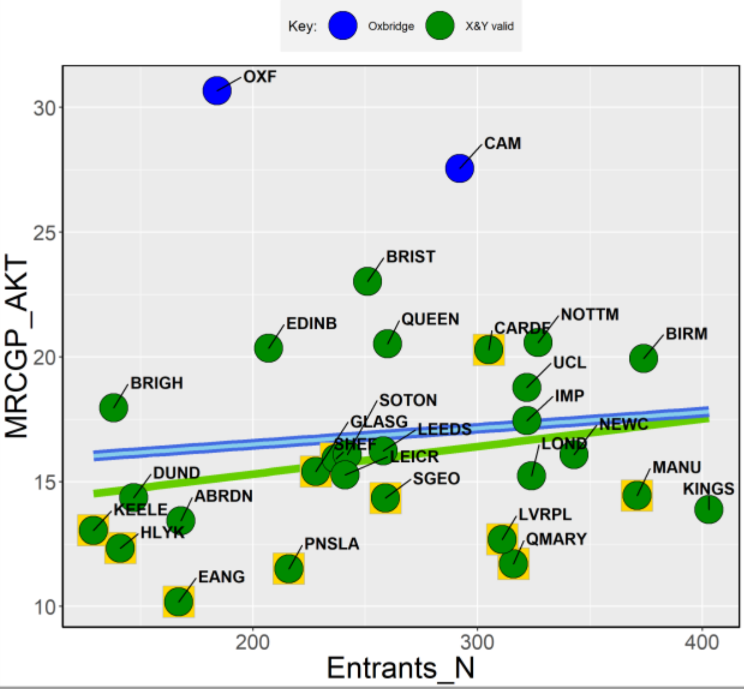

98/587 Y42: MRCGP_CSA X14: Entrants_N
r(all)= -0.063 p= 0.747 r(NonImp)= -0.063 Npairs=29 NimputedPairs=0

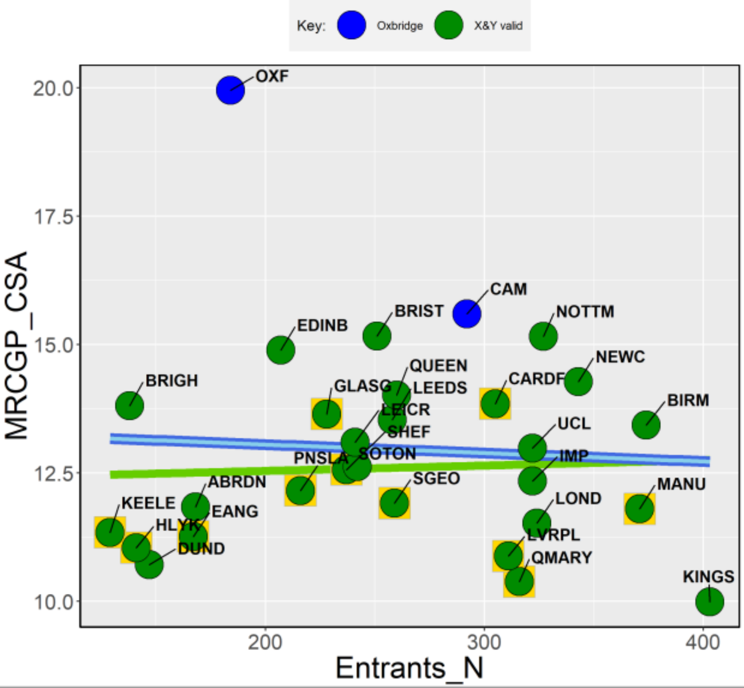

98/588 Y43: FRCA_Pt1 X14: Entrants_N
r(all)= 0.189 p= 0.326 r(NonImp)= 0.175 Npairs=29 NimputedPairs=10

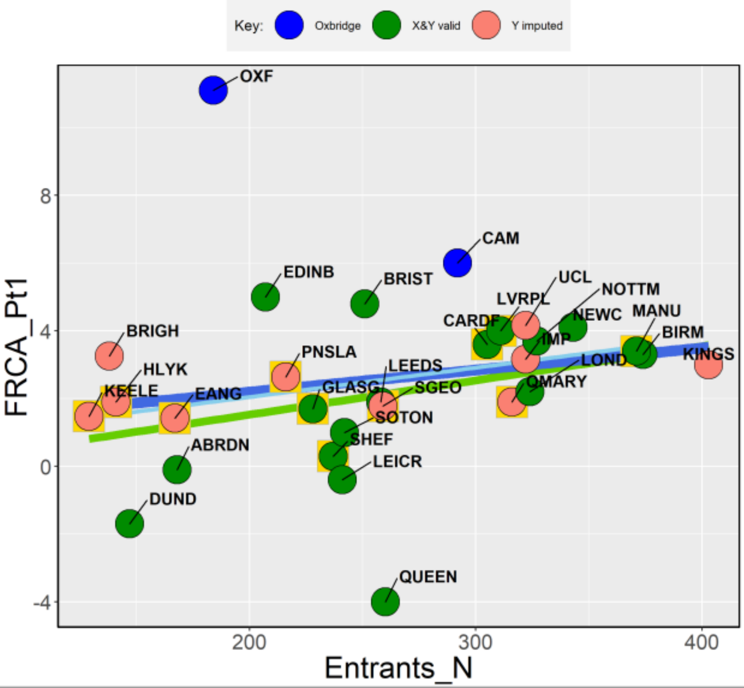

99/589 Y44: MRCOG_Pt1 X14: Entrants_N

r(all)= 0.118 p= 0.542 r(NonImp)= 0.017 Npairs=29 NimputedPairs=10

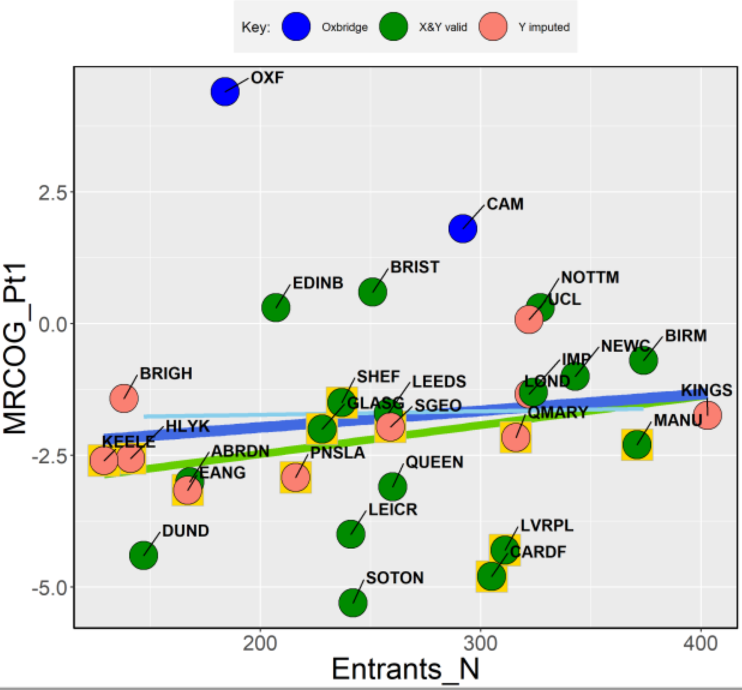

99/590 Y45: MRCOG_Pt2 X14: Entrants_N

r(all)= 0.089 p= 0.645 r(NonImp)= 0.023 Npairs=29 NimputedPairs=10

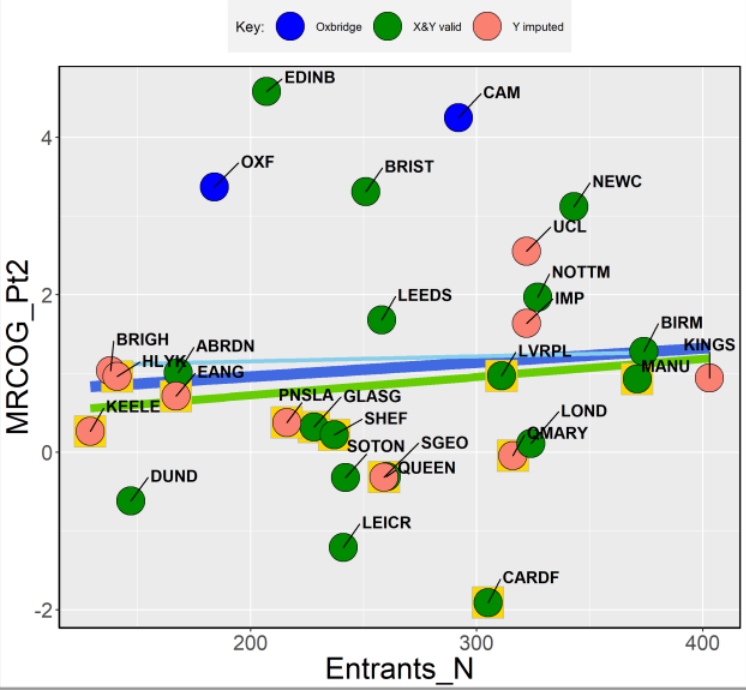

99/591 Y46: MRCP_Pt1 X14: Entrants_N

r(all)= 0.214 p= 0.264 r(NonImp)= 0.187 Npairs=29 NimputedPairs=3

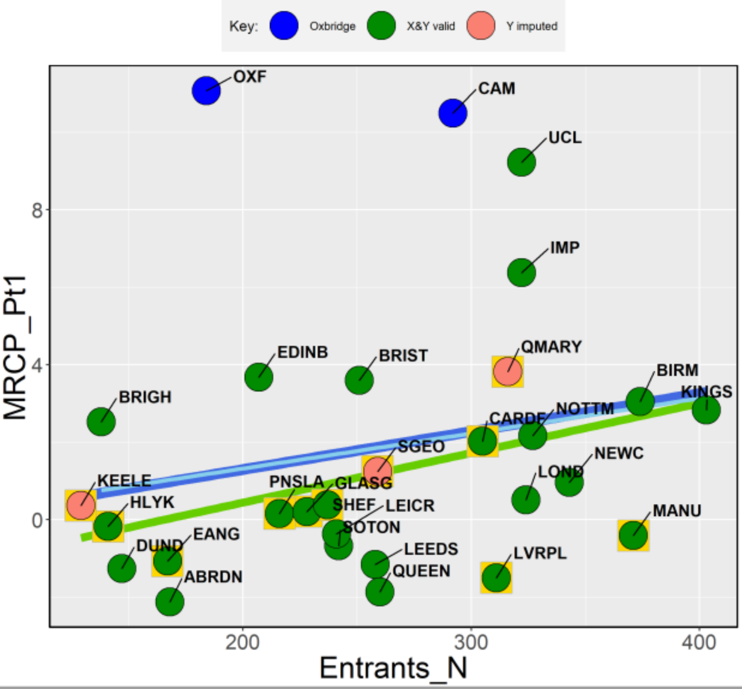

99/592 Y47: MRCP_Pt2 X14: Entrants_N

r(all)= -0.031 p= 0.874 r(NonImp)= -0.028 Npairs=29 NimputedPairs=3

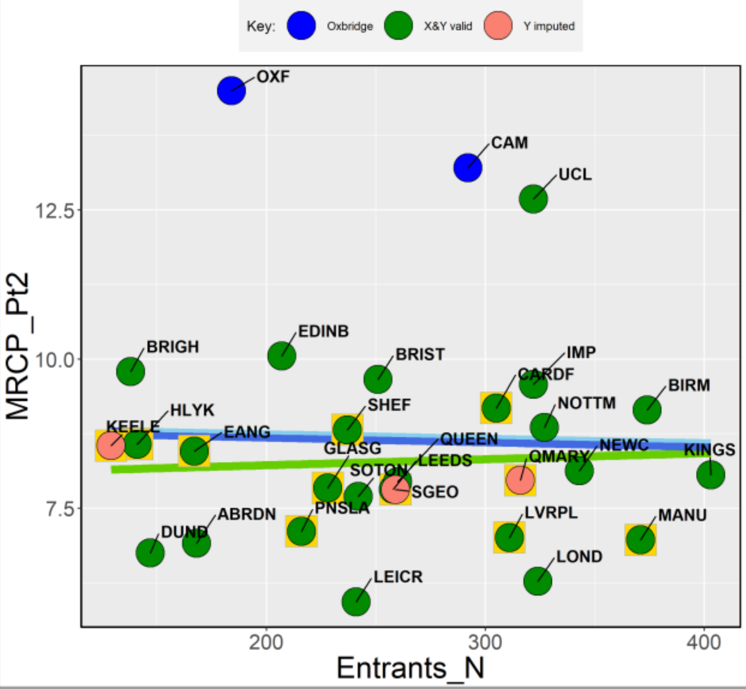

99/593 Y48: MRCP_PACES X14: Entrants_N

r(all)= 0.245 p= 0.199 r(NonImp)= 0.220 Npairs=29 NimputedPairs=4

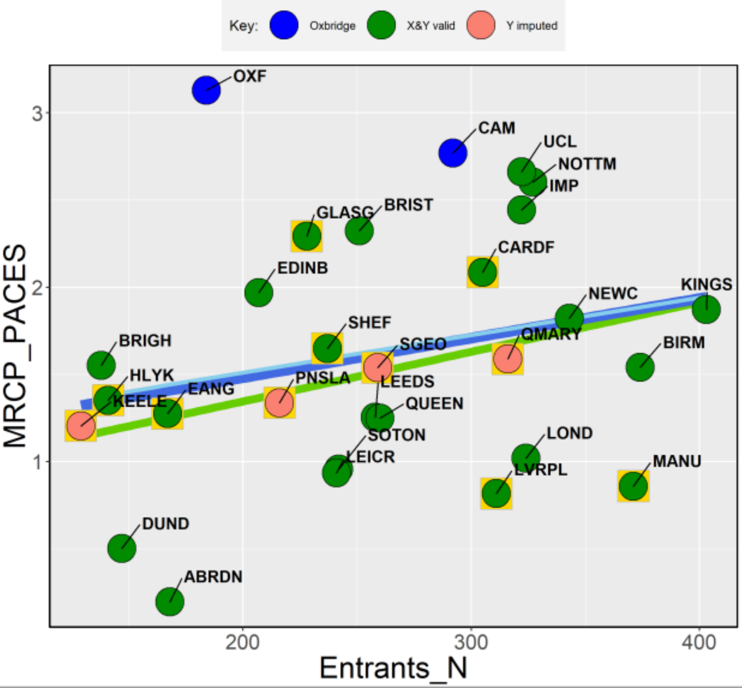

99/594 Y49: GMC_Sanctions X14: Entrants_N

r(all)= -0.121 p= 0.531 r(NonImp)= 0.062 Npairs=29 NimputedPairs=10

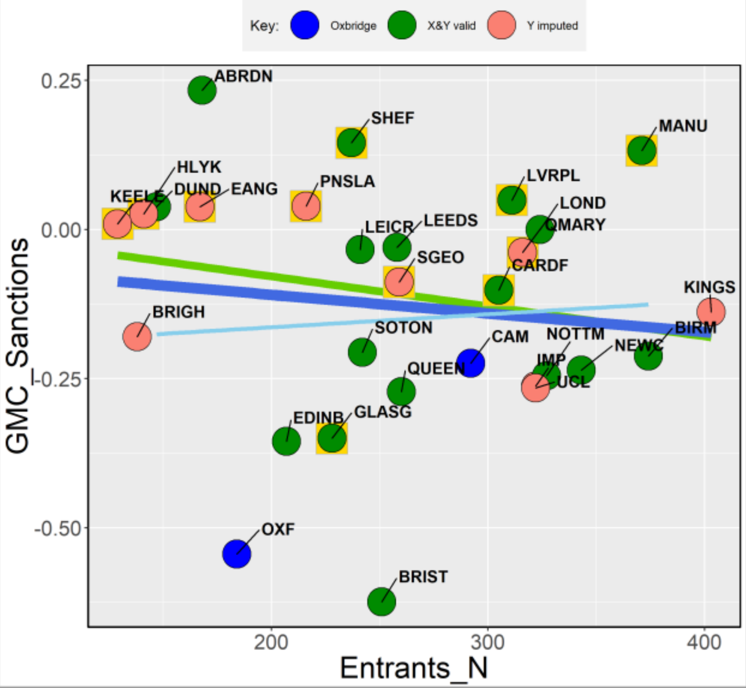

100/595 Y50: ARCP_NotExam X14: Entrants_N
r(all)= -0.361 p= 0.0546 r(NonImp)= -0.342 Npairs=29 NimputedPairs=1

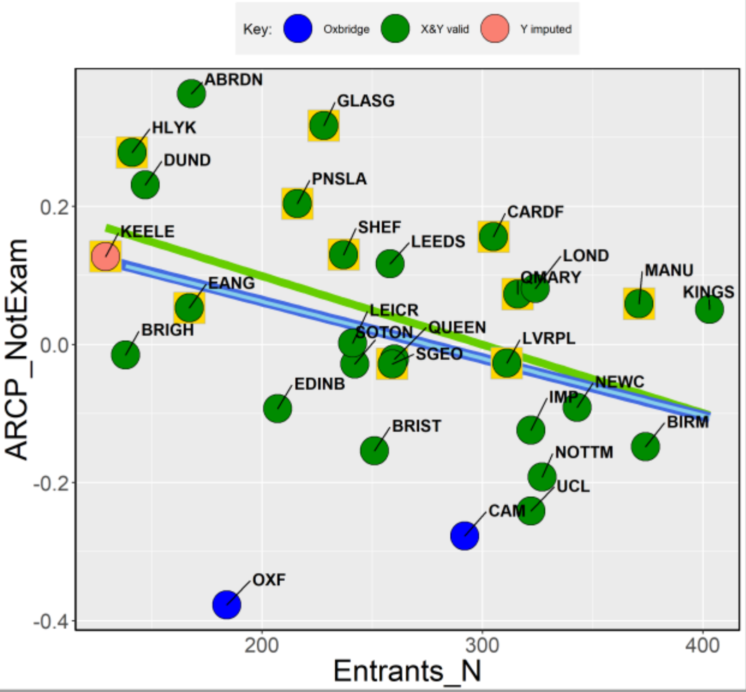

100/596 Y16: EntryGrades X15: Entrants_Female
r(all)= -0.453 p= 0.0135 r(NonImp)= -0.453 Npairs=29 NimputedPairs=0

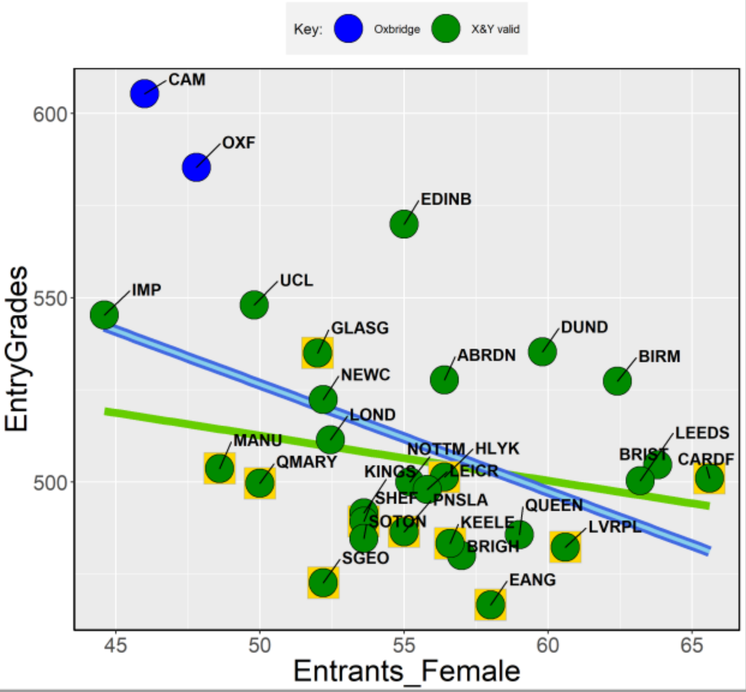

100/597 Y17: Entrants_NonHome X15: Entrants_Fem
r(all)= 0.147 p= 0.446 r(NonImp)= 0.147 Npairs=29 NimputedPairs=0

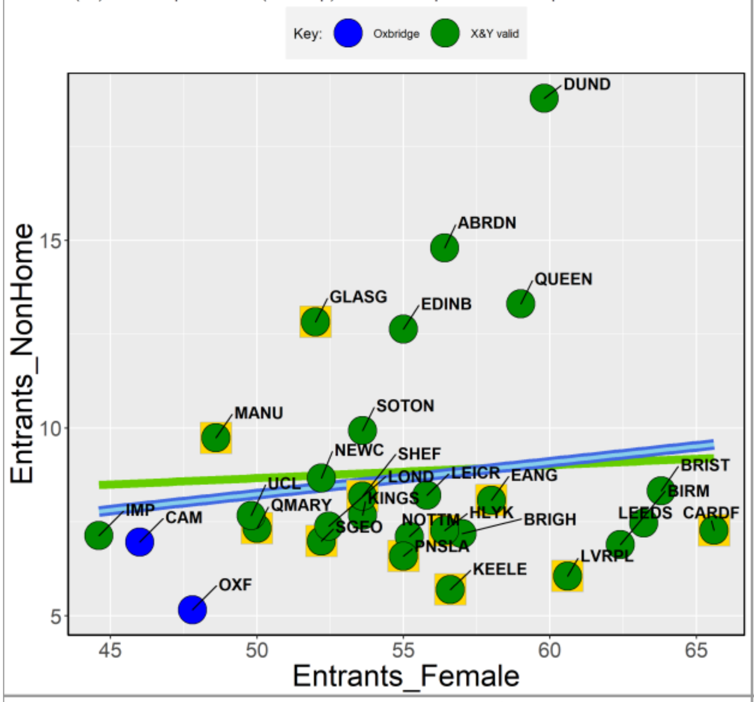

100/598 Y18: Teaching_Factor1_Trad X15: Entrants_F
r(all)= -0.254 p= 0.183 r(NonImp)= -0.311 Npairs=29 NimputedPairs=3

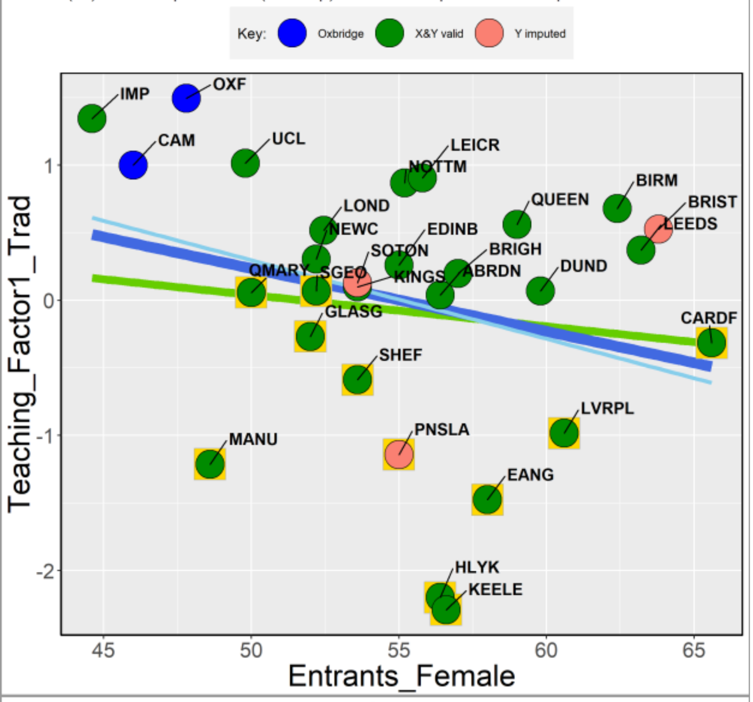

100/599 Y19: Teaching_Factor2_Struc X15: Entrants_
r(all)= -0.110 p= 0.57 r(NonImp)= -0.111 Npairs=29 NimputedPairs=3

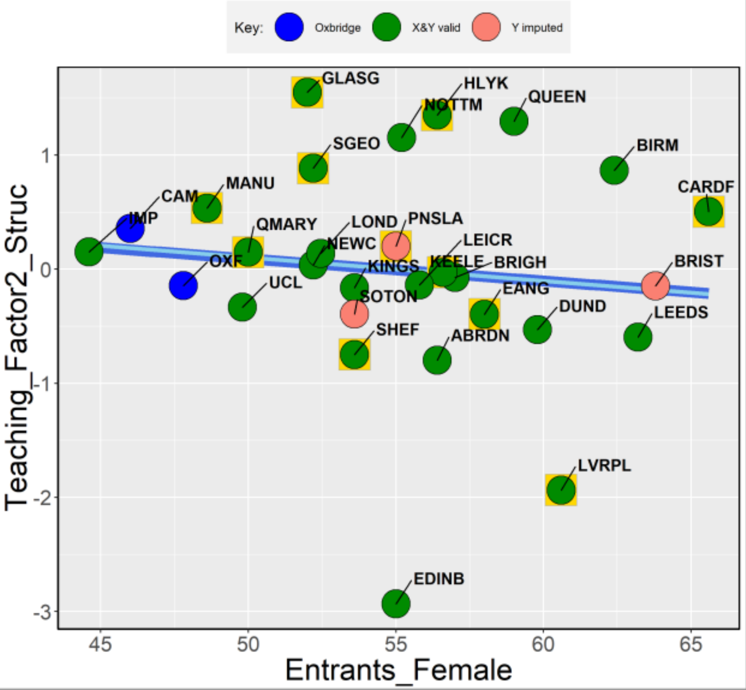

100/600 Y20: Teach_GP X15: Entrants_Female
r(all)= 0.082 p= 0.673 r(NonImp)= 0.132 Npairs=29 NimputedPairs=3

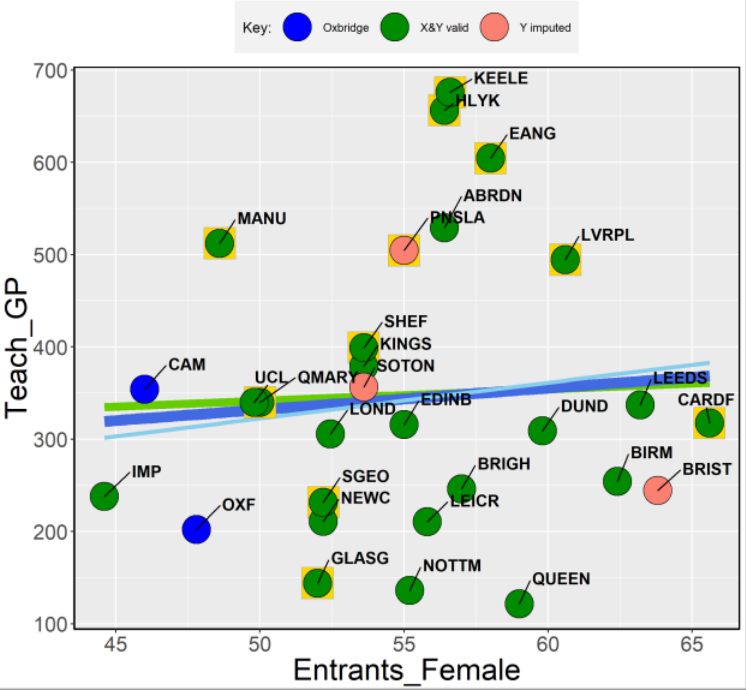

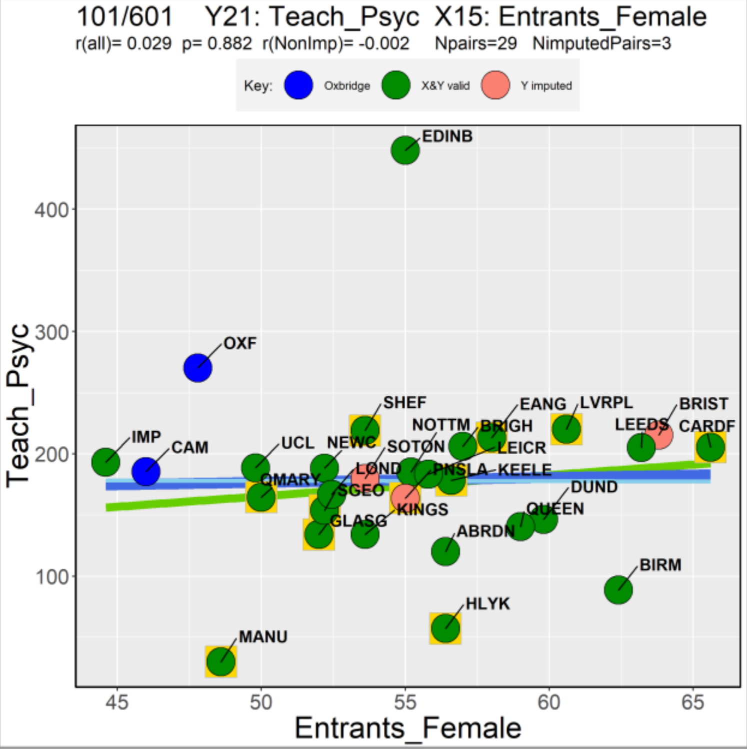
101/601 Y21: Teach_Psyc X15: Entrants_Female
r(all)= 0.029 p= 0.882 r(NonImp)= -0.002 Npairs=29 NimputedPairs=3
Key: Oxbridge X&Y valid Y imputed
Teach_Psyc
Entrants_Female

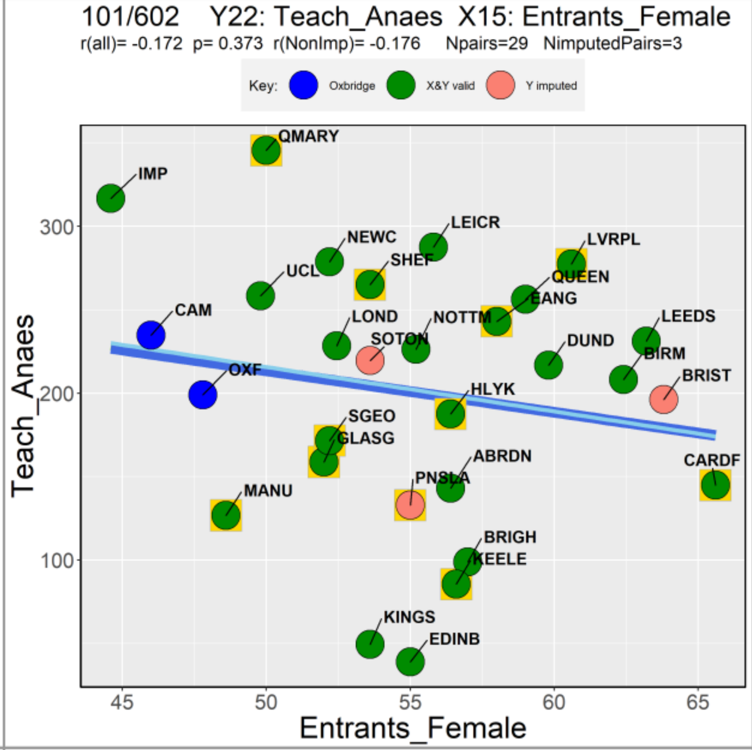
101/602 Y22: Teach_Anaes X15: Entrants_Female
r(all)= -0.172 p= 0.373 r(NonImp)= -0.176 Npairs=29 NimputedPairs=3
Key: Oxbridge X&Y valid Y imputed
Teach_Anaes
Entrants_Female

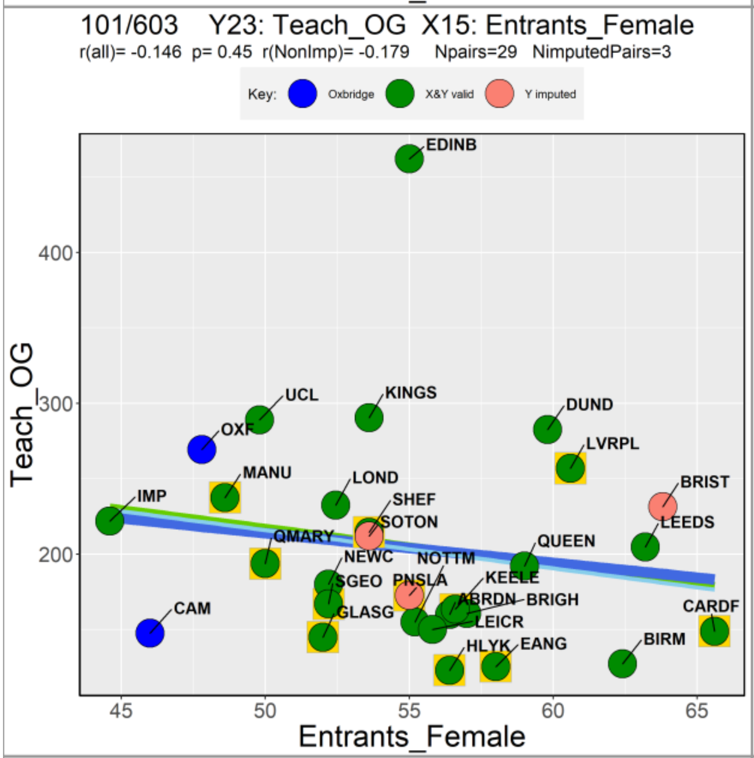
101/603 Y23: Teach_OG X15: Entrants_Female
r(all)= -0.146 p= 0.45 r(NonImp)= -0.179 Npairs=29 NimputedPairs=3
Key: Oxbridge X&Y valid Y imputed
Teach_OG
Entrants_Female

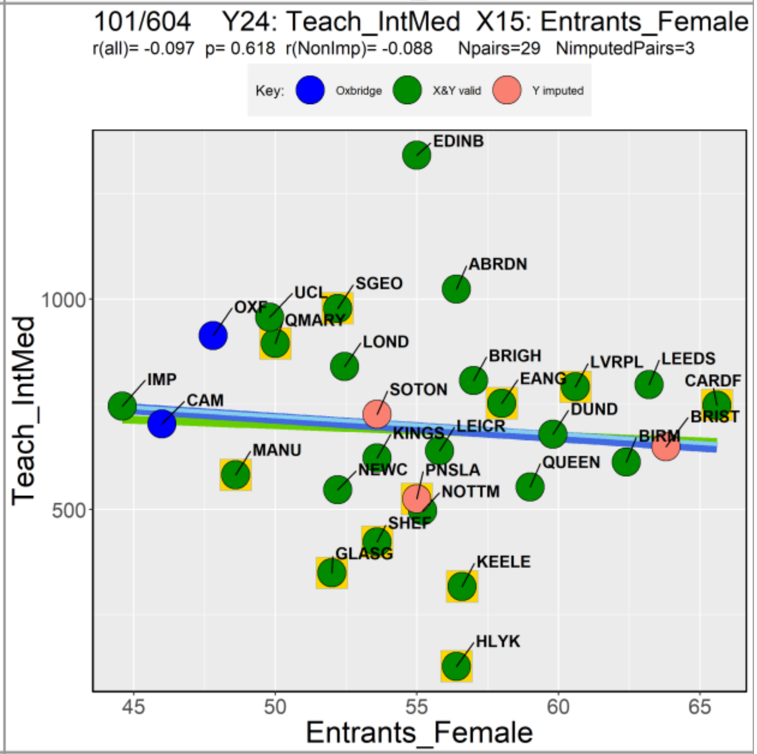
101/604 Y24: Teach_IntMed X15: Entrants_Female
r(all)= -0.097 p= 0.618 r(NonImp)= -0.088 Npairs=29 NimputedPairs=3
Key: Oxbridge X&Y valid Y imputed
Teach_IntMed
Entrants_Female

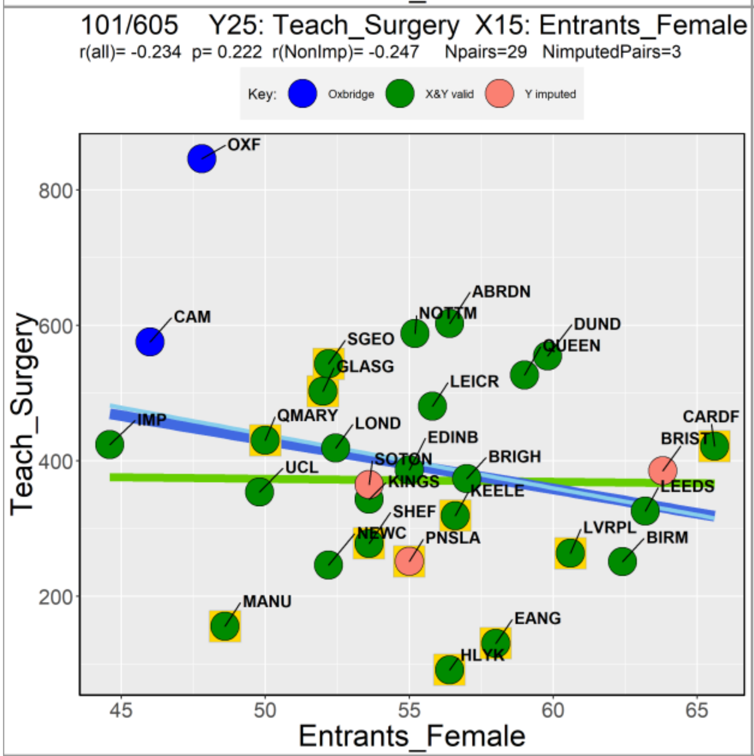
101/605 Y25: Teach_Surgery X15: Entrants_Female
r(all)= -0.234 p= 0.222 r(NonImp)= -0.247 Npairs=29 NimputedPairs=3
Key: Oxbridge X&Y valid Y imputed
Teach_Surgery
Entrants_Female

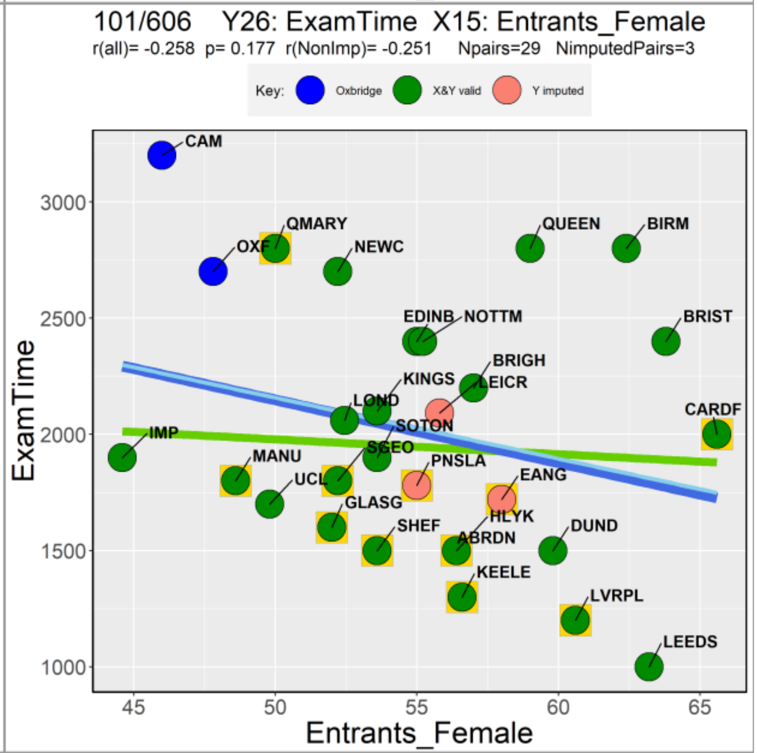
101/606 Y26: ExamTime X15: Entrants_Female
r(all)= -0.258 p= 0.177 r(NonImp)= -0.251 Npairs=29 NimputedPairs=3
Key: Oxbridge X&Y valid Y imputed
ExamTime
Entrants_Female

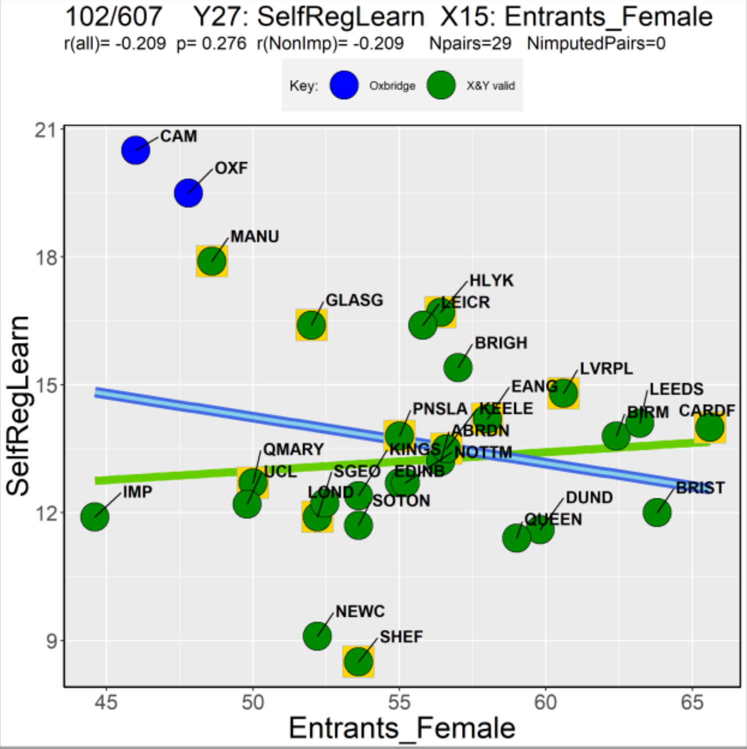
102/607 Y27: SelfRegLearn X15: Entrants_Female
r(all)= -0.209 p= 0.276 r(NonImp)= -0.209 Npairs=29 NimputedPairs=0
Key: Oxbridge X&Y valid
SelfRegLearn
Entrants_Female
CAM
OXF
MANU
GLASG
HLYK
LEICR
BRIGH
LVRPL
EANG
LEEDS
PNSLA
KEELE
BIRM
CARDF
ABRDN
QMARY
KINGS
NOTTM
UCL
SGEO
EDINB
IMP
LOND
SOTON
DUND
BRIST
QUEEN
NEWC
SHEF

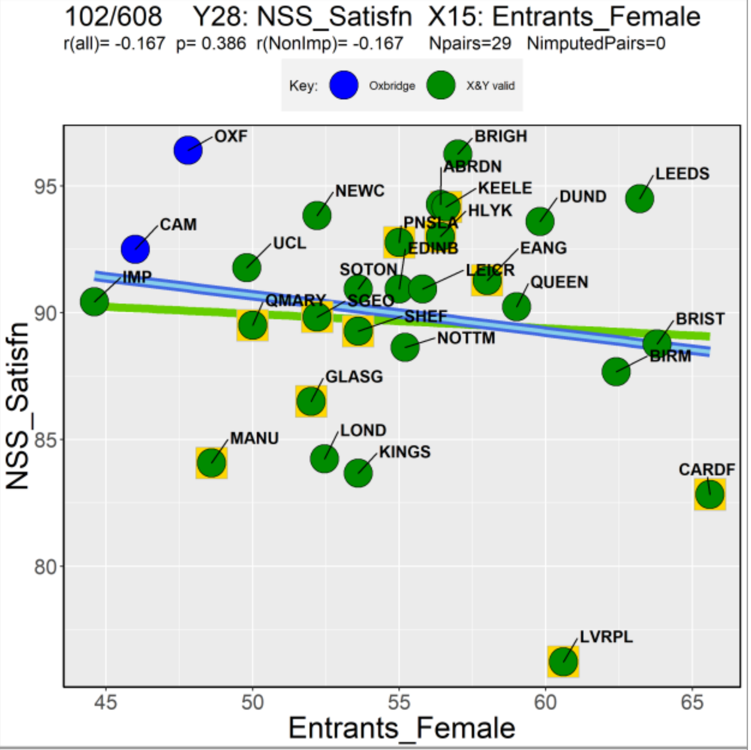
102/608 Y28: NSS_Satisfn X15: Entrants_Female
r(all)= -0.167 p= 0.386 r(NonImp)= -0.167 Npairs=29 NimputedPairs=0
Key: Oxbridge X&Y valid
NSS_Satisfn
Entrants_Female
OXF
BRIGH
ABRDN
LEEDS
NEWC
KEELE
DUND
HLYK
CAM
PNSLA
UCL
EDINB
EANG
SOTON
LEICR
QUEEN
IMP
QMARY
SGEO
SHEF
BRIST
NOTTM
BIRM
GLASG
MANU
LOND
KINGS
CARDF
LVRPL

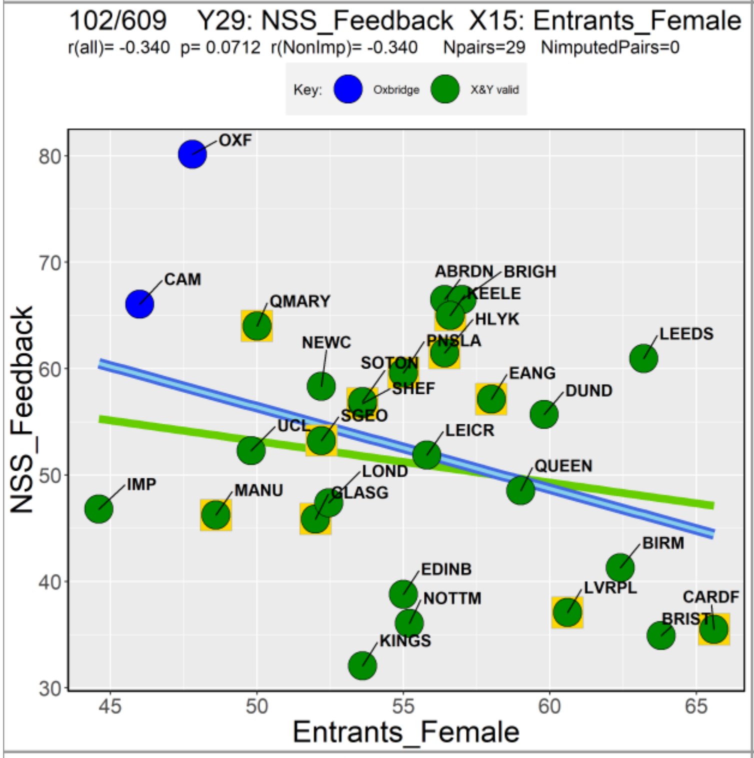
102/609 Y29: NSS_Feedback X15: Entrants_Female
r(all)= -0.340 p= 0.0712 r(NonImp)= -0.340 Npairs=29 NimputedPairs=0
Key: Oxbridge X&Y valid
NSS_Feedback
Entrants_Female
OXF
ABRDN
BRIGH
CAM
KEELE
QMARY
HLYK
NEWC
PNSLA
LEEDS
SOTON
EANG
SHEF
DUND
SGEO
UCL
LEICR
QUEEN
IMP
LOND
MANU
GLASG
BIRM
EDINB
NOTTM
LVRPL
CARDF
BRIST
KINGS

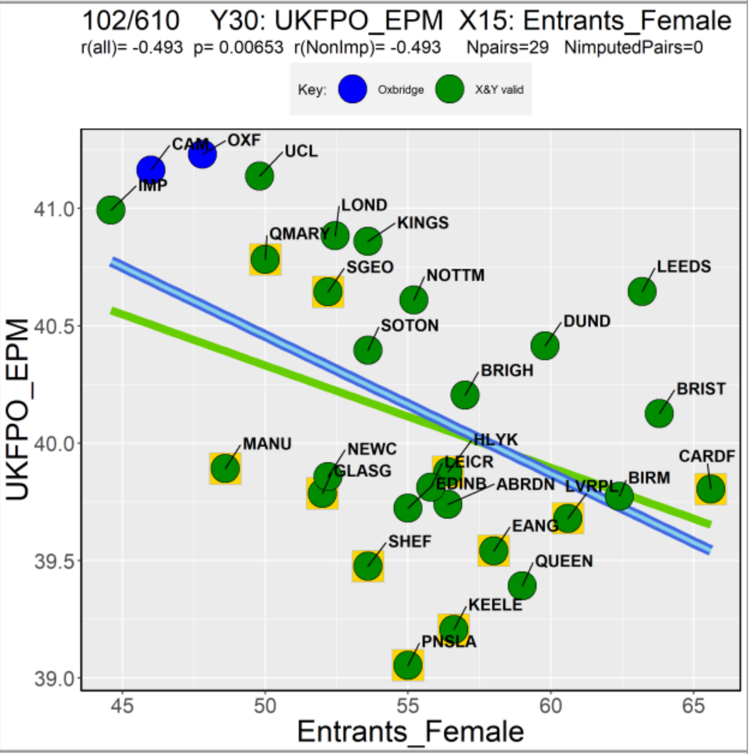
102/610 Y30: UKFPO_EPM X15: Entrants_Female
r(all)= -0.493 p= 0.00653 r(NonImp)= -0.493 Npairs=29 NimputedPairs=0
Key: Oxbridge X&Y valid
UKFPO_EPM
Entrants_Female
CAM
OXF
UCL
IMP
LOND
QMARY
KINGS
SGEO
NOTTM
LEEDS
DUND
SOTON
BRIGH
BRIST
HLYK
MANU
NEWC
CARDF
LEICR
GLASG
EDINB
ABRDN
LVRPL
BIRM
EANG
SHEF
QUEEN
KEELE
PNSLA

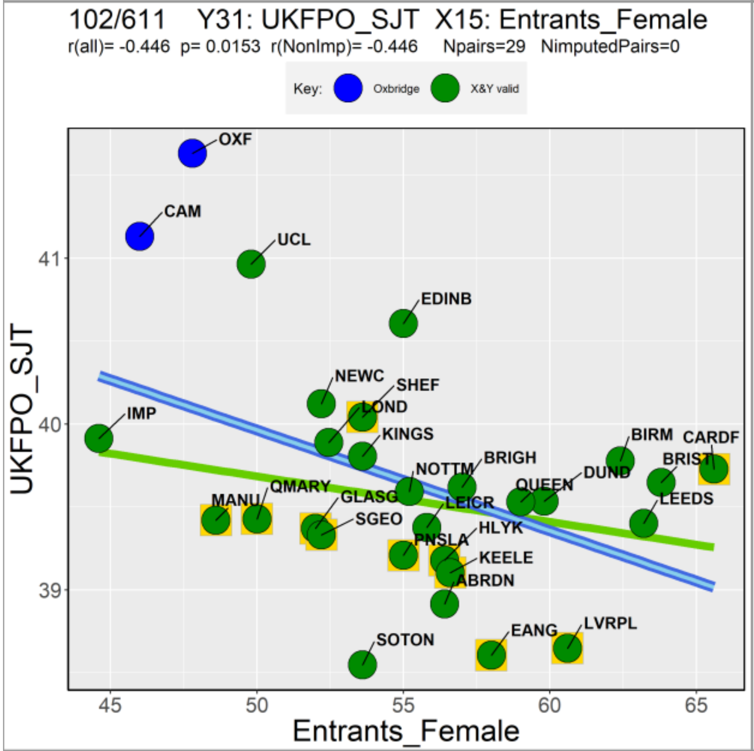
102/611 Y31: UKFPO_SJT X15: Entrants_Female
r(all)= -0.446 p= 0.0153 r(NonImp)= -0.446 Npairs=29 NimputedPairs=0
Key: Oxbridge X&Y valid
UKFPO_SJT
Entrants_Female
OXF
CAM
UCL
EDINB
NEWC
SHEF
IMP
LOND
KINGS
BIRM
CARDF
NOTTM
BRIGH
BRIST
QUEEN
DUND
MANU
QMARY
GLASG
LEICR
LEEDS
SGEO
HLYK
PNSLA
KEELE
ABRDN
SOTON
EANG
LVRPL

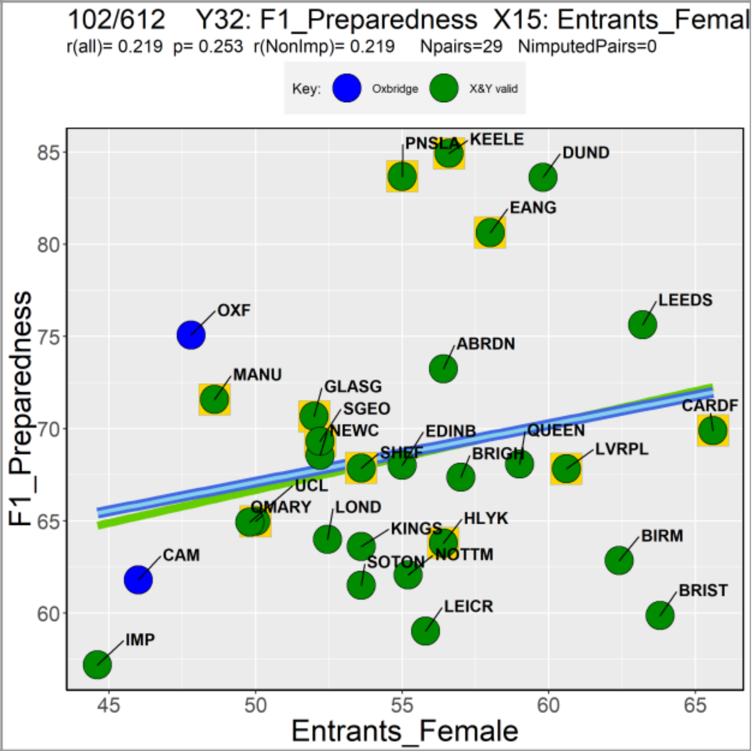
102/612 Y32: F1_Preparedness X15: Entrants_Femal
r(all)= 0.219 p= 0.253 r(NonImp)= 0.219 Npairs=29 NimputedPairs=0
Key: Oxbridge X&Y valid
F1_Preparedness
Entrants_Female
PNSLA
KEELE
DUND
EANG
OXF
LEEDS
ABRDN
MANU
GLASG
SGEO
NEWC
EDINB
QUEEN
CARDF
SHEF
BRIGH
LVRPL
UCL
QMARY
LOND
KINGS
HLYK
CAM
SOTON
NOTTM
BIRM
LEICR
BRIST
IMP

103/613 Y33: F1_Satisfn X15: Entrants_Female
r(all)= 0.483 p= 0.00798 r(NonImp)= 0.483 Npairs=29 NimputedPairs=0

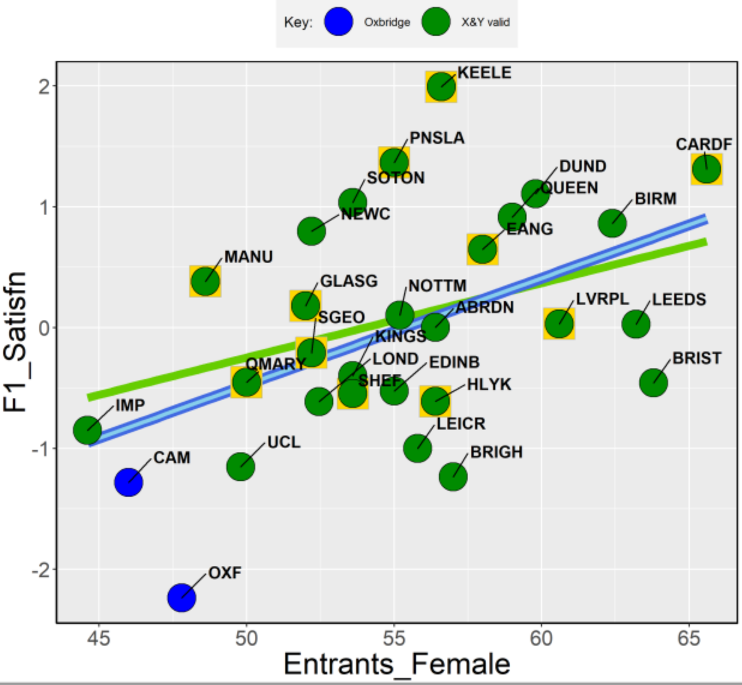

103/614 Y34: F1_Workload X15: Entrants_Female
r(all)= 0.015 p= 0.937 r(NonImp)= 0.015 Npairs=29 NimputedPairs=0

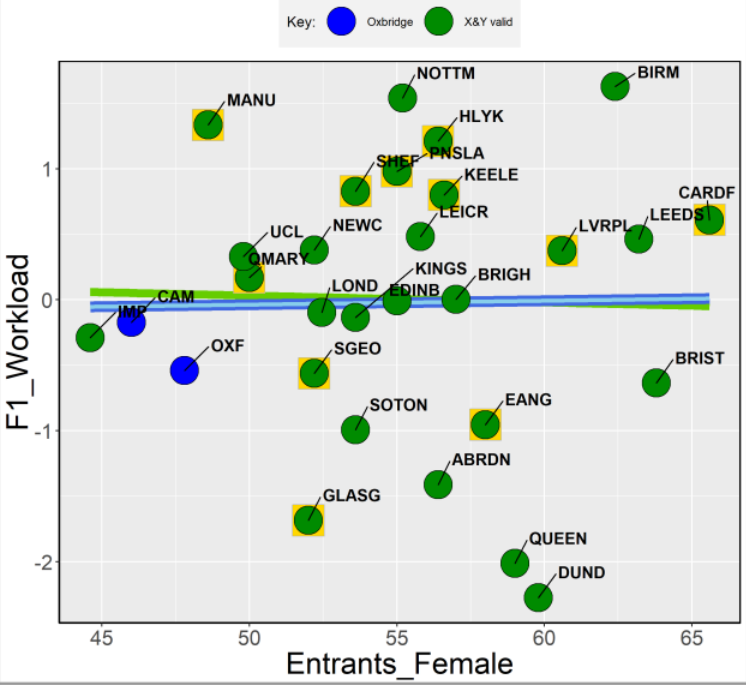

103/615 Y35: F1_Supervn X15: Entrants_Female
r(all)= -0.220 p= 0.251 r(NonImp)= -0.220 Npairs=29 NimputedPairs=0

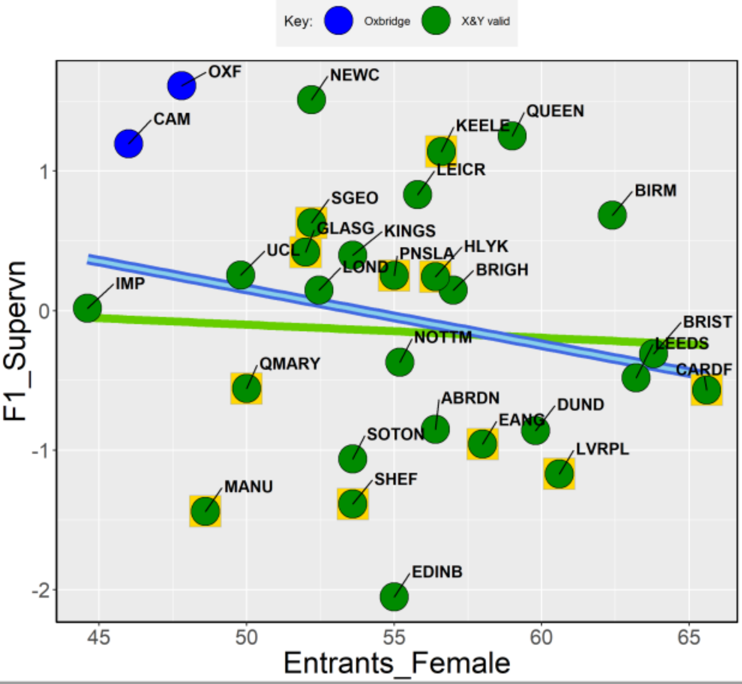

103/616 Y36: Trainee_GP X15: Entrants_Female
r(all)= 0.313 p= 0.0985 r(NonImp)= 0.313 Npairs=29 NimputedPairs=0

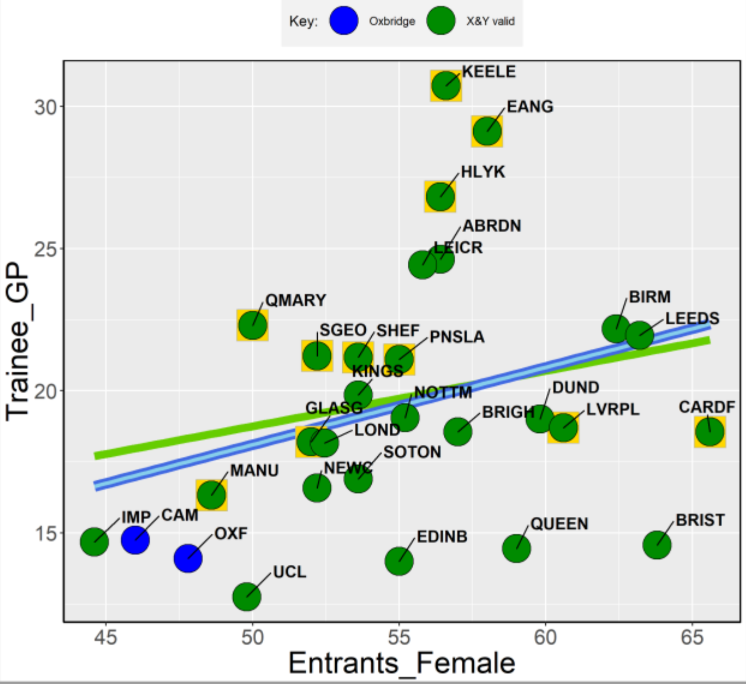

103/617 Y37: Trainee_Psyc X15: Entrants_Female
r(all)= 0.047 p= 0.808 r(NonImp)= 0.047 Npairs=29 NimputedPairs=0

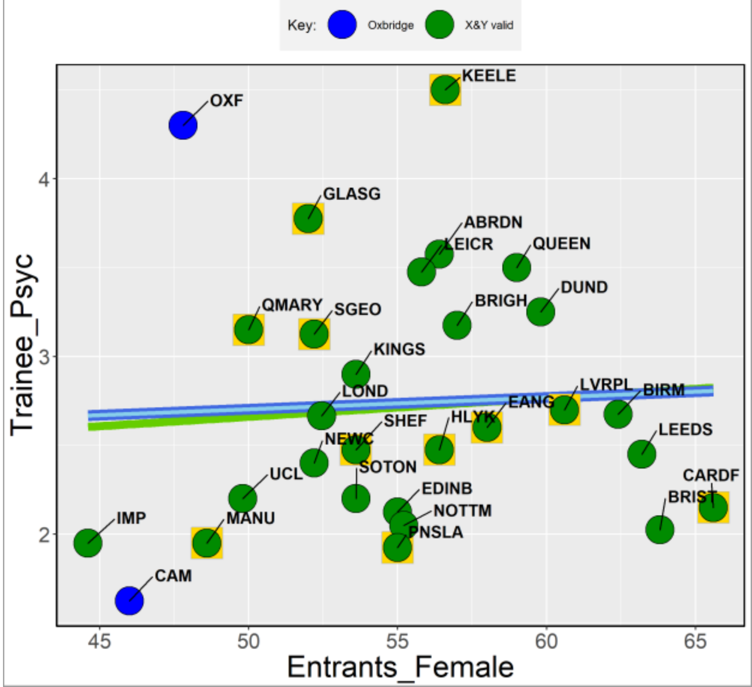

103/618 Y38: TraineeApp_Surgery X15: Entrants_Fe
r(all)= -0.431 p= 0.0195 r(NonImp)= -0.411 Npairs=29 NimputedPairs=2

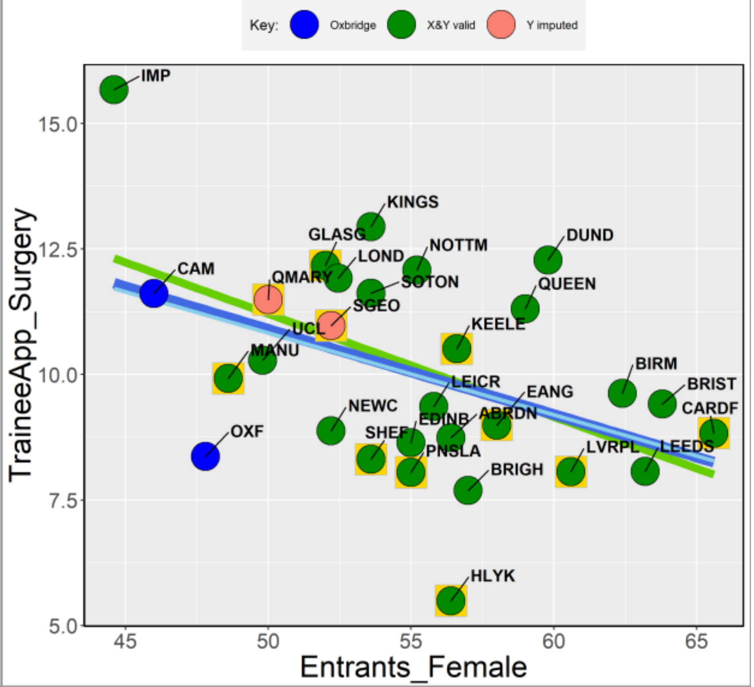

104/619 Y39: TraineeApp_Anaes X15: Entrants_Fe[illegible]
r(all)= 0.105 p= 0.586 r(NonImp)= 0.105 Npairs=29 NimputedPairs=0

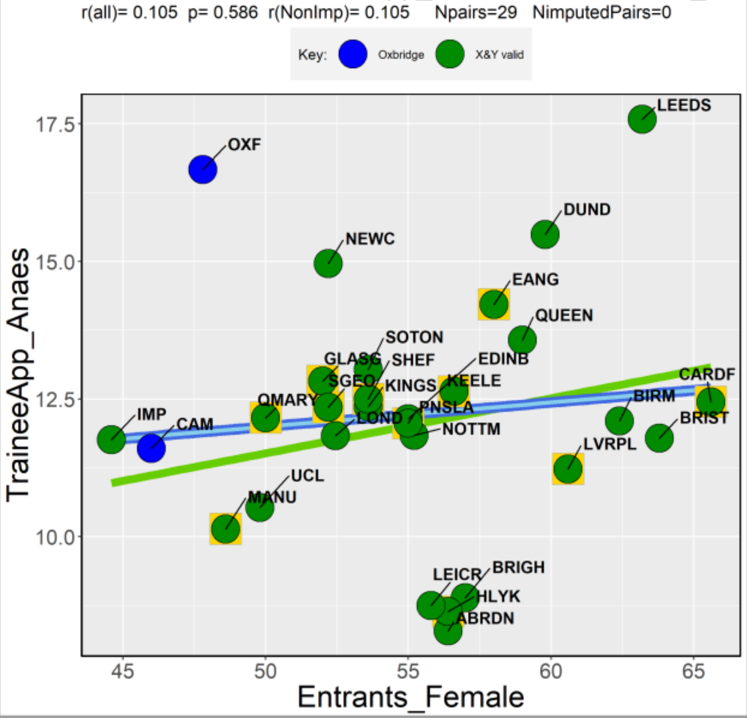

104/620 Y40: GMC_PGexams X15: Entrants_Female
r(all)= -0.204 p= 0.29 r(NonImp)= -0.204 Npairs=29 NimputedPairs=0

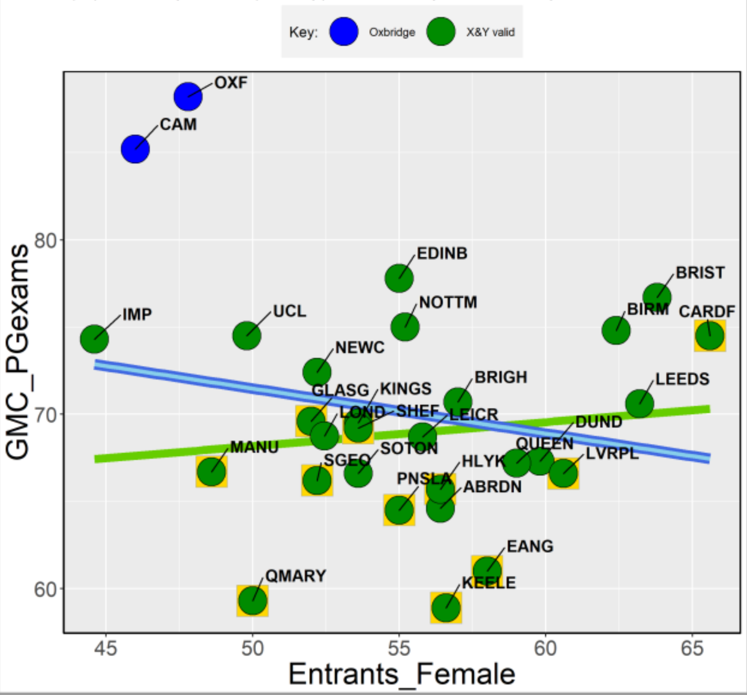

104/621 Y41: MRCGP_AKT X15: Entrants_Female
r(all)= -0.137 p= 0.478 r(NonImp)= -0.137 Npairs=29 NimputedPairs=0

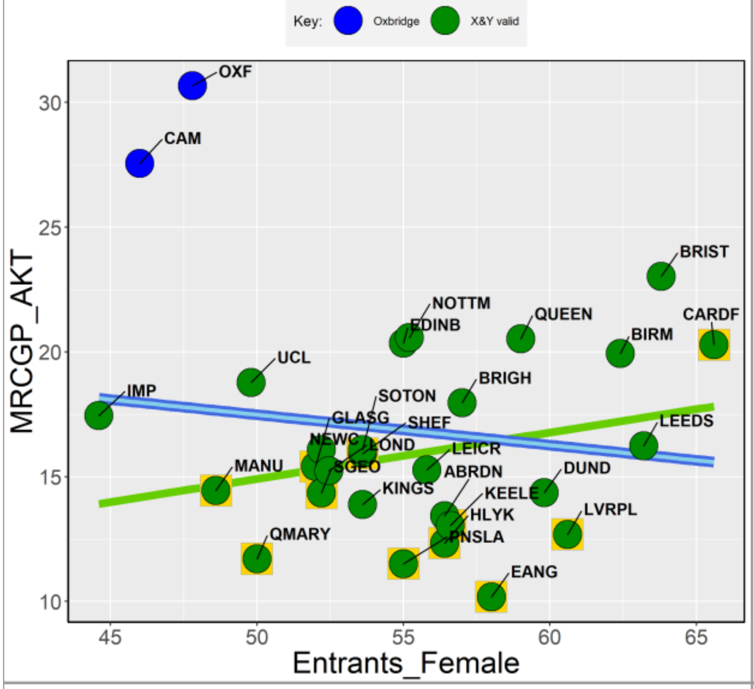

104/622 Y42: MRCGP_CSA X15: Entrants_Female
r(all)= -0.108 p= 0.578 r(NonImp)= -0.108 Npairs=29 NimputedPairs=0

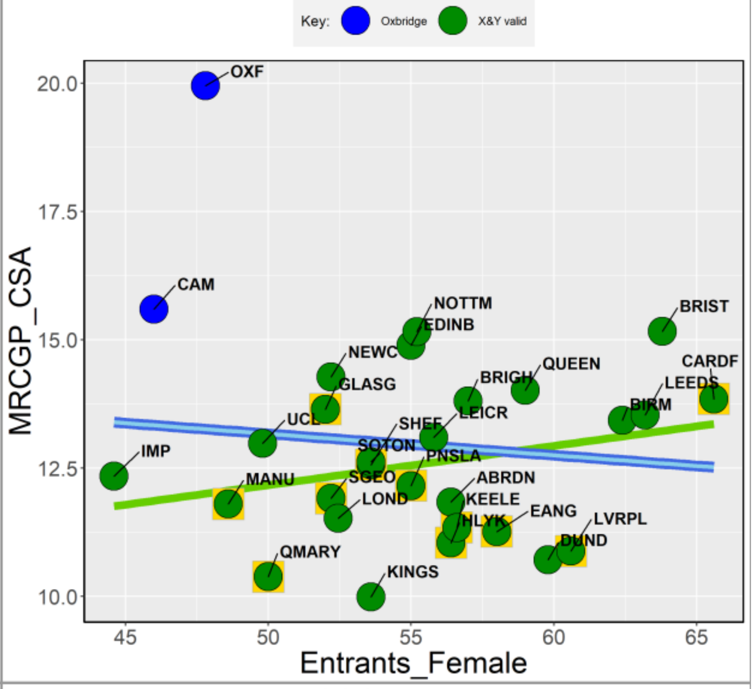

104/623 Y43: FRCA_Pt1 X15: Entrants_Female
r(all)= -0.311 p= 0.101 r(NonImp)= -0.329 Npairs=29 NimputedPairs=10

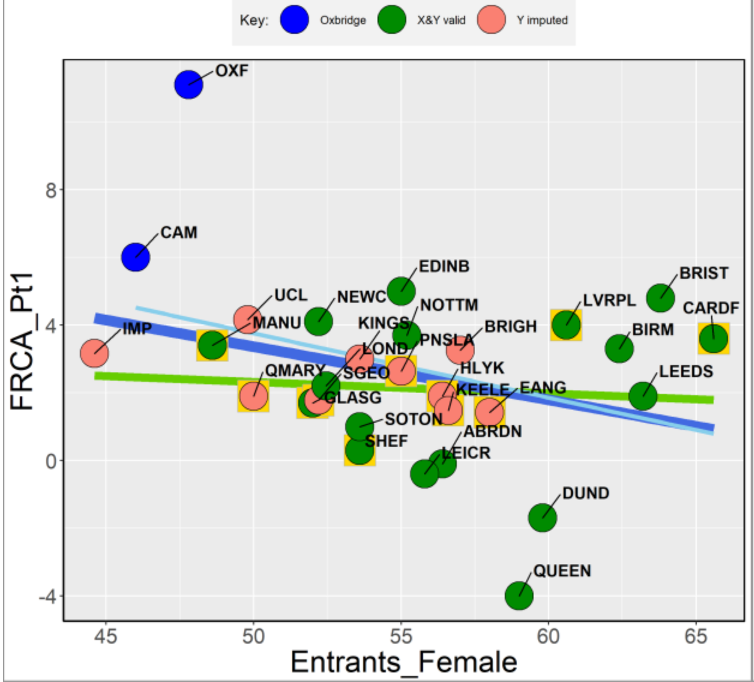

104/624 Y44: MRCOG_Pt1 X15: Entrants_Female
r(all)= -0.418 p= 0.0241 r(NonImp)= -0.442 Npairs=29 NimputedPairs=10

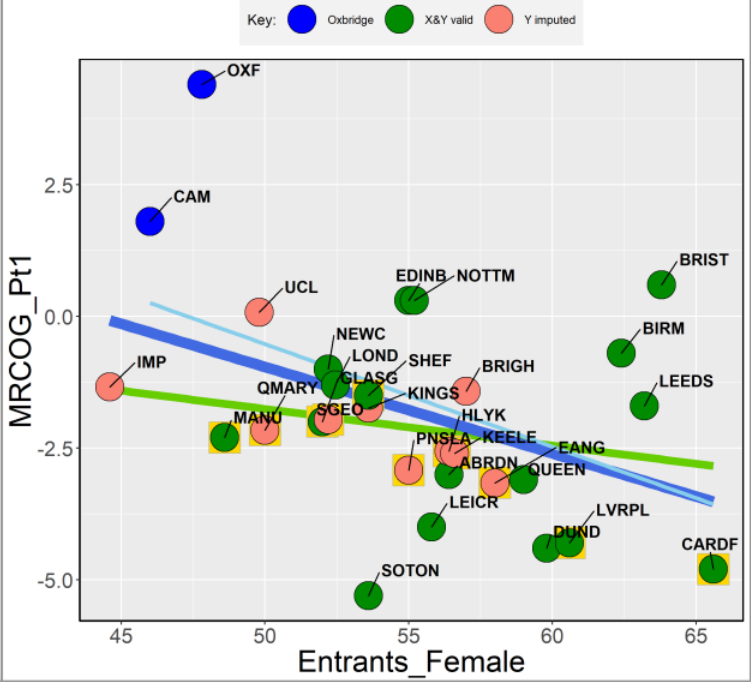

105/625 Y45: MRCOG_Pt2 X15: Entrants_Female
r(all)= -0.305 p= 0.108 r(NonImp)= -0.353 Npairs=29 NimputedPairs=10

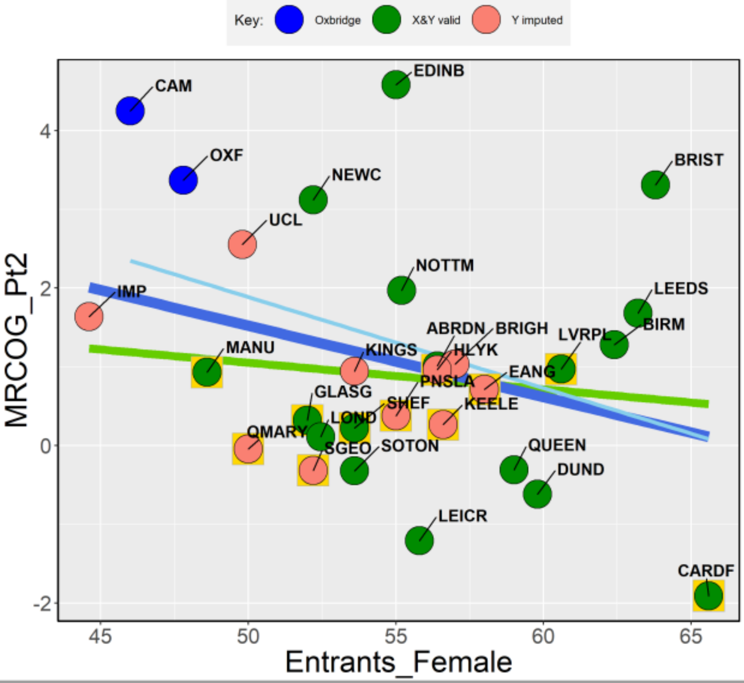

105/626 Y46: MRCP_Pt1 X15: Entrants_Female
r(all)= -0.530 p= 0.00312 r(NonImp)= -0.527 Npairs=29 NimputedPairs=3

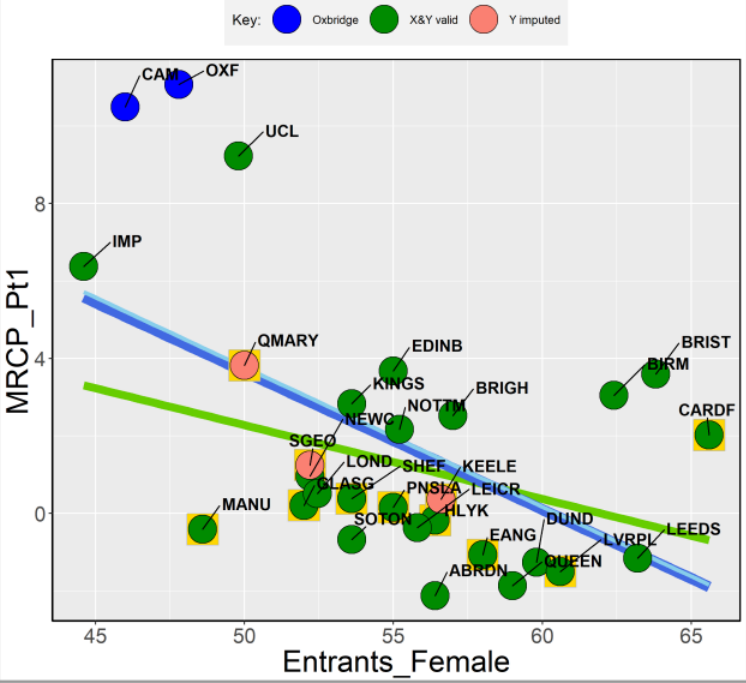

105/627 Y47: MRCP_Pt2 X15: Entrants_Female
r(all)= -0.338 p= 0.0727 r(NonImp)= -0.371 Npairs=29 NimputedPairs=3

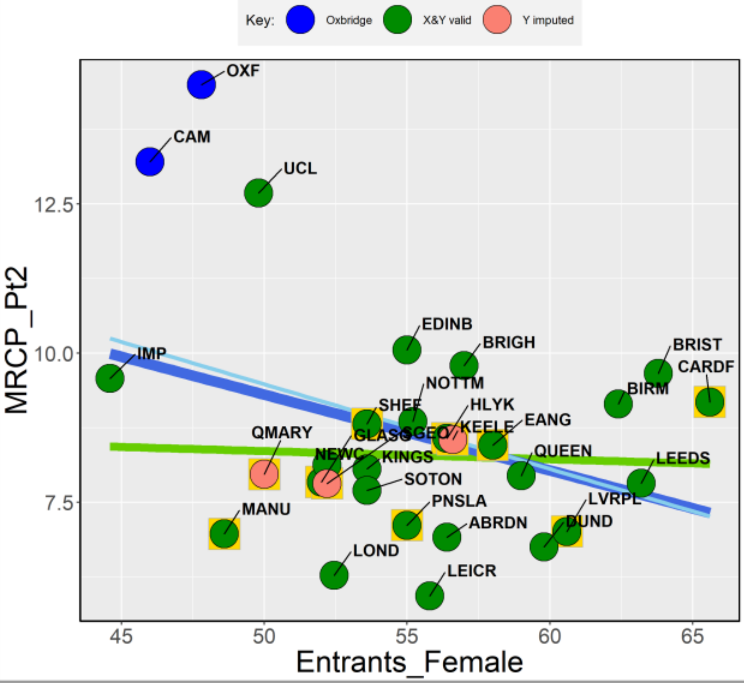

105/628 Y48: MRCP_PACES X15: Entrants_Female
r(all)= -0.370 p= 0.0482 r(NonImp)= -0.382 Npairs=29 NimputedPairs=4

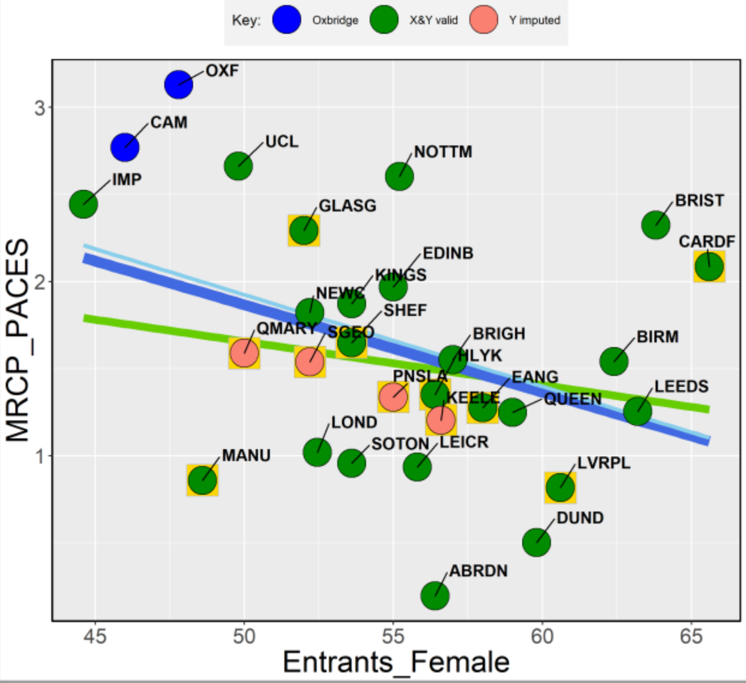

105/629 Y49: GMC_Sanctions X15: Entrants_Fema

r(all)= 0.097 p= 0.618 r(NonImp)= 0.035 Npairs=29 NimputedPairs=10

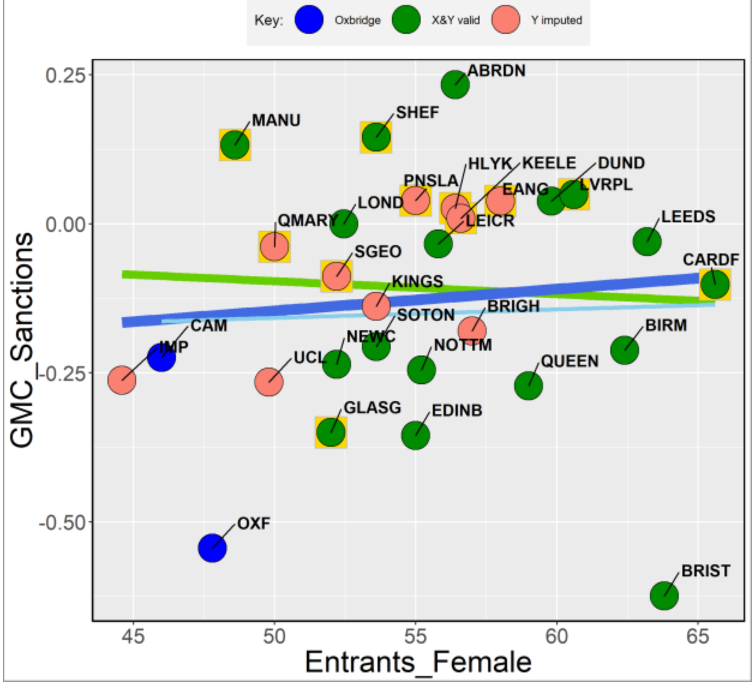

105/630 Y50: ARCP_NotExam X15: Entrants_Femal
r(all)= 0.313 p= 0.0986 r(NonImp)= 0.309 Npairs=29 NimputedPairs=1

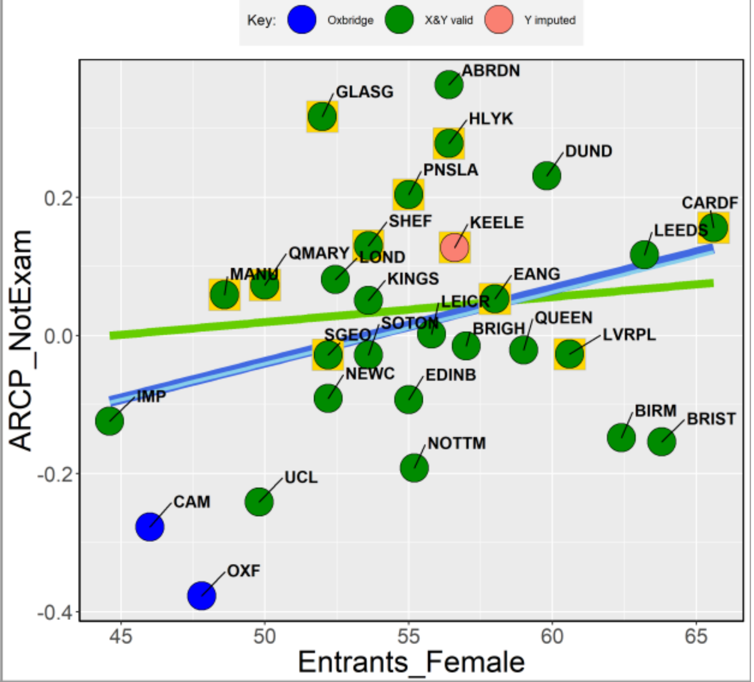

Supplement: Supplementary file 6 — Additional file 6. Graphs 421 to 630 (pages 71 to 105). [file 12916_2020_1572_MOESM6_ESM.pdf]
