# Supplementary material for: Exploring UK medical school differences: the MedDifs study of selection, teaching, student and F1 perceptions, postgraduate outcomes and fitness to practise
Source: BMC Med. 2020 May 14;18:136. doi: 10.1186/s12916-020-01572-3 (PMC7222458; doi:10.1186/s12916-020-01572-3)

106/631 Y17: Entrants_NonHome X16: EntryGrades
r(all)= 0.153 p= 0.429 r(NonImp)= 0.153 Npairs=29 NimputedPairs=0

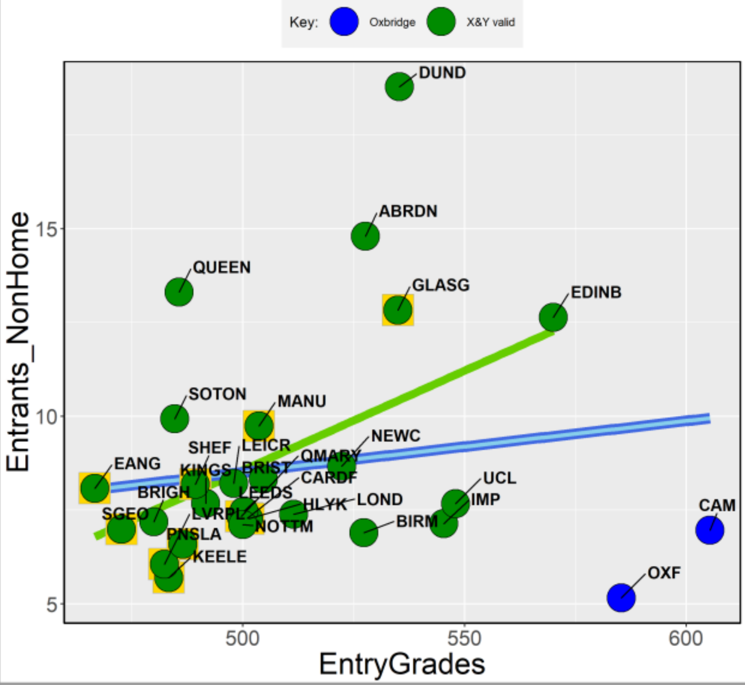

106/632 Y18: Teaching_Factor1_Trad X16: EntryGrad
r(all)= 0.538 p= 0.00259 r(NonImp)= 0.543 Npairs=29 NimputedPairs=3

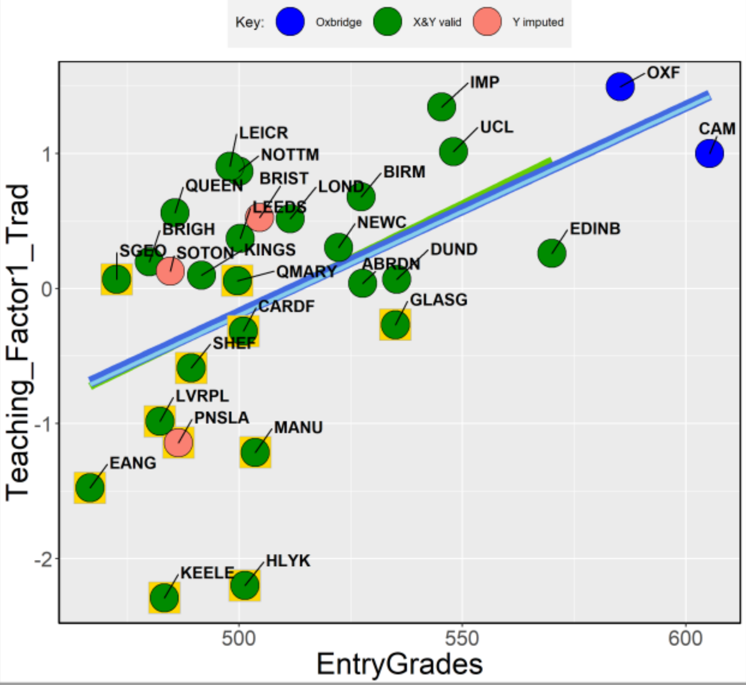

106/633 Y19: Teaching_Factor2_Struc X16: EntryGrad
r(all)= -0.142 p= 0.463 r(NonImp)= -0.154 Npairs=29 NimputedPairs=3

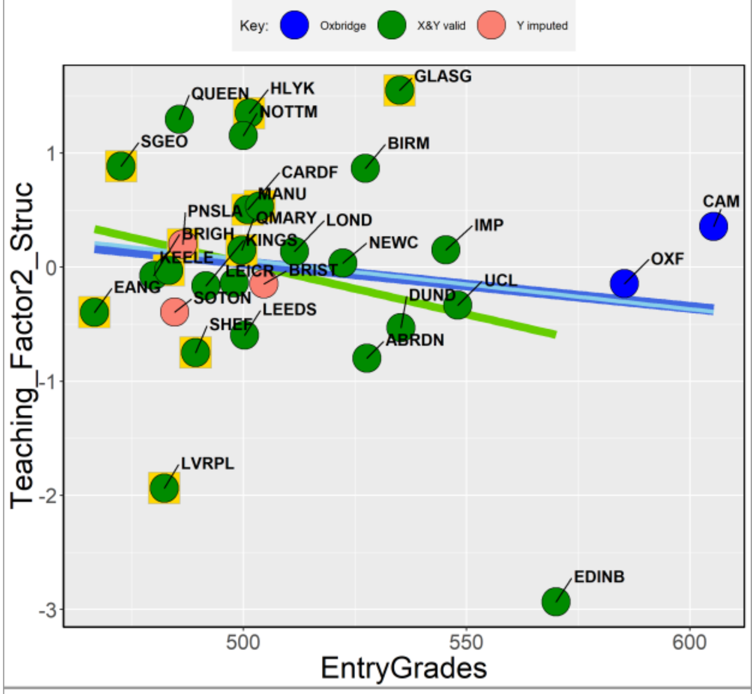

106/634 Y20: Teach_GP X16: EntryGrades
r(all)= -0.267 p= 0.161 r(NonImp)= -0.254 Npairs=29 NimputedPairs=3

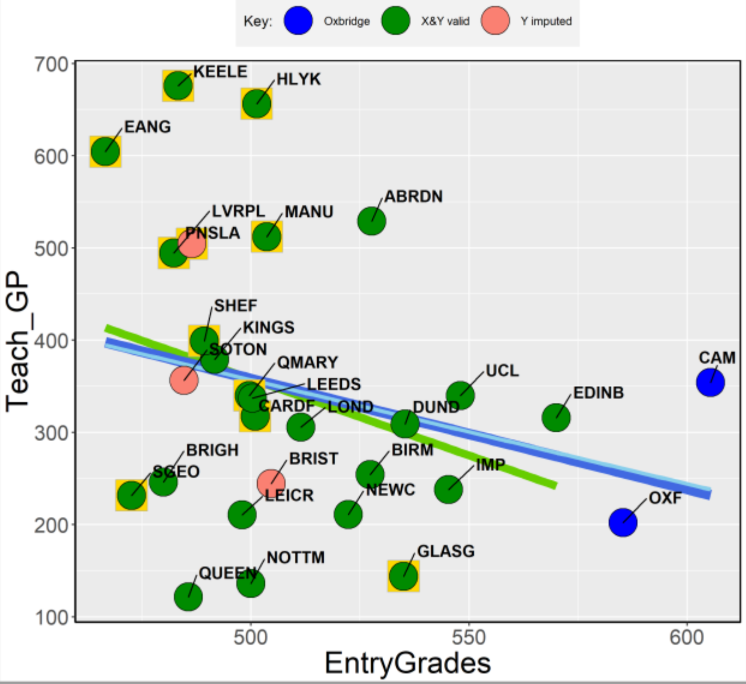

106/635 Y21: Teach_Psyc X16: EntryGrades
r(all)= 0.297 p= 0.118 r(NonImp)= 0.305 Npairs=29 NimputedPairs=3

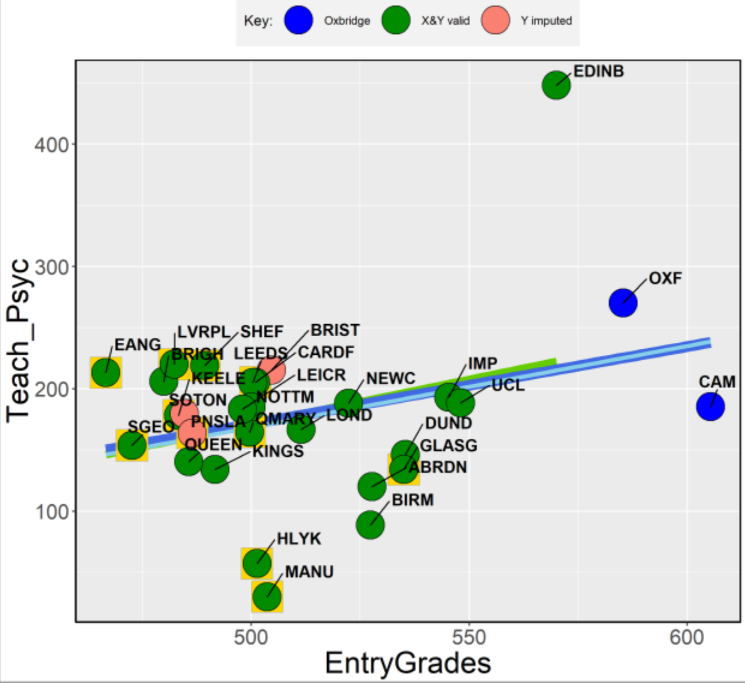

106/636 Y22: Teach_Anaes X16: EntryGrades
r(all)= 0.023 p= 0.904 r(NonImp)= 0.004 Npairs=29 NimputedPairs=3

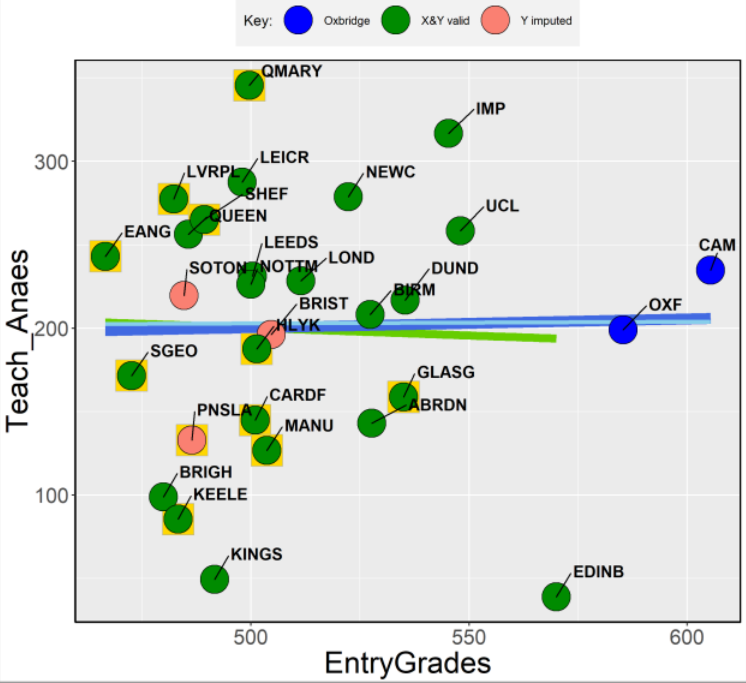

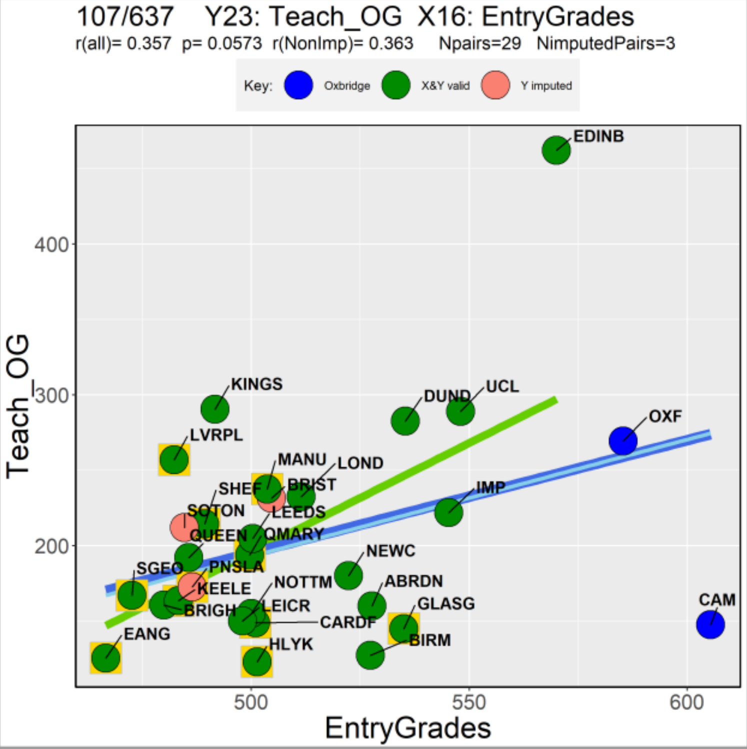
107/637 Y23: Teach_OG X16: EntryGrades
r(all)= 0.357 p= 0.0573 r(NonImp)= 0.363 Npairs=29 NimputedPairs=3
Key: Oxbridge X&Y valid Y imputed
Teach_OG
EntryGrades
EDINB
KINGS
DUND
UCL
OXF
LVRPL
MANU
LOND
SHEF
BRIST
SOTON
LEEDS
IMP
QUEEN
QMARY
NEWC
SGEO
PNSLA
NOTTM
ABRDN
KEELE
LEICR
GLASG
BRIGH
CARDF
CAM
EANG
HLYK
BIRM
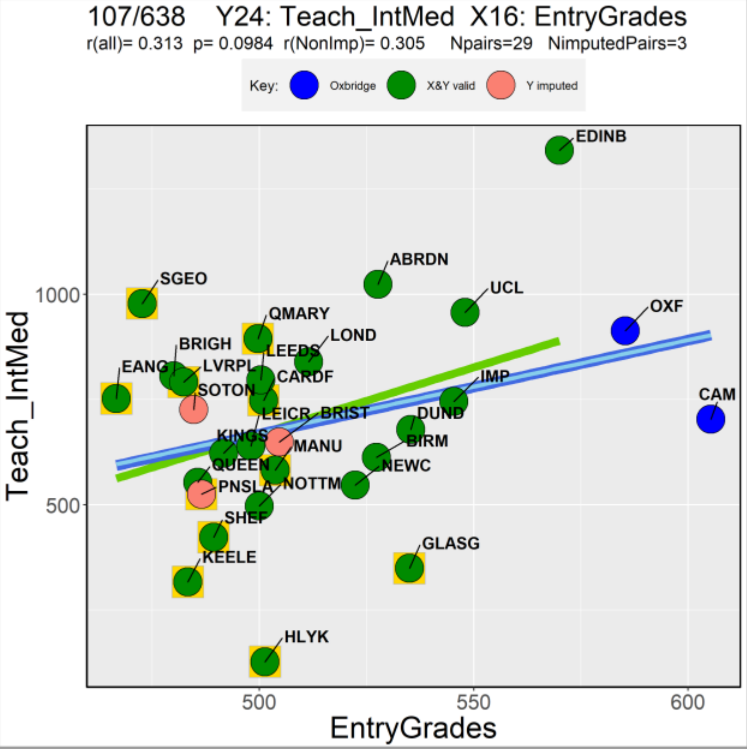
107/638 Y24: Teach_IntMed X16: EntryGrades
r(all)= 0.313 p= 0.0984 r(NonImp)= 0.305 Npairs=29 NimputedPairs=3
Key: Oxbridge X&Y valid Y imputed
Teach_IntMed
EntryGrades
EDINB
ABRDN
SGEO
UCL
OXF
QMARY
BRIGH
LOND
LEEDS
EANG
LVRPL
CARDF
SOTON
IMP
LEICR
BRIST
DUND
CAM
KINGS
BIRM
MANU
QUEEN
NEWC
PNSLA
NOTTM
SHEF
GLASG
KEELE
HLYK
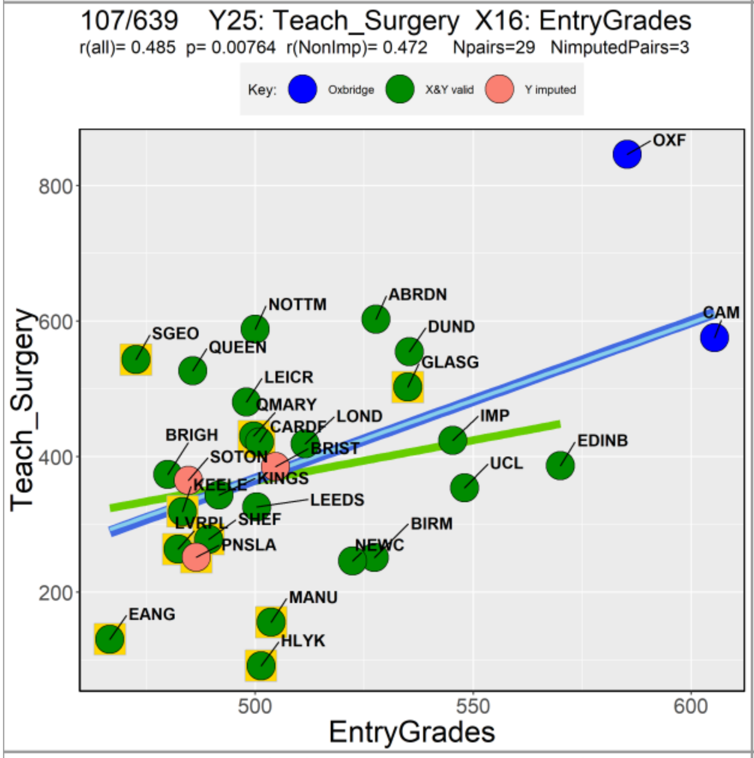
107/639 Y25: Teach_Surgery X16: EntryGrades
r(all)= 0.485 p= 0.00764 r(NonImp)= 0.472 Npairs=29 NimputedPairs=3
Key: Oxbridge X&Y valid Y imputed
Teach_Surgery
EntryGrades
OXF
NOTTM
ABRDN
CAM
SGEO
DUND
QUEEN
GLASG
LEICR
QMARY
LOND
CARDF
IMP
BRIGH
EDINB
BRIST
SOTON
UCL
KEELE
KINGS
LEEDS
LVRPL
SHEF
BIRM
PNSLA
NEWC
MANU
EANG
HLYK
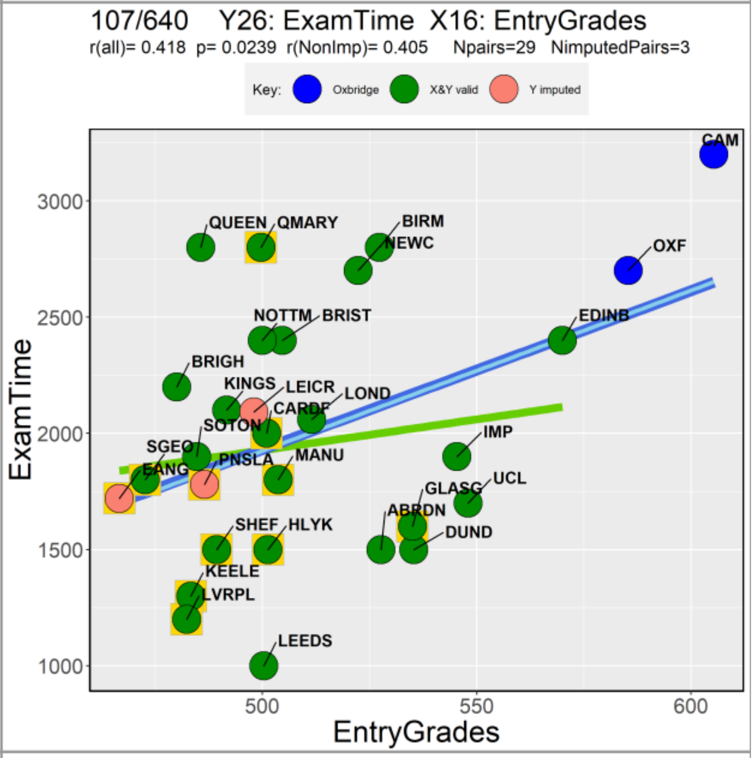
107/640 Y26: ExamTime X16: EntryGrades
r(all)= 0.418 p= 0.0239 r(NonImp)= 0.405 Npairs=29 NimputedPairs=3
Key: Oxbridge X&Y valid Y imputed
ExamTime
EntryGrades
CAM
QUEEN
QMARY
BIRM
NEWC
OXF
NOTTM
BRIST
EDINB
BRIGH
KINGS
LEICR
LOND
CARDF
SOTON
IMP
SGEO
PNSLA
MANU
EANG
GLASG
UCL
SHEF
HLYK
ABRDN
DUND
KEELE
LVRPL
LEEDS
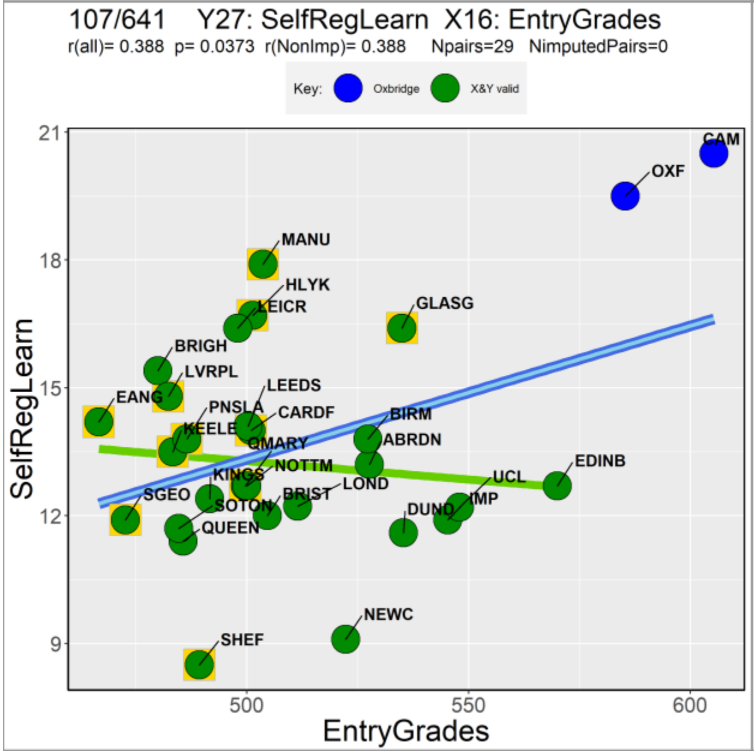
107/641 Y27: SelfRegLearn X16: EntryGrades
r(all)= 0.388 p= 0.0373 r(NonImp)= 0.388 Npairs=29 NimputedPairs=0
Key: Oxbridge X&Y valid
SelfRegLearn
EntryGrades
CAM
OXF
MANU
HLYK
LEICR
GLASG
BRIGH
LVRPL
LEEDS
EANG
PNSLA
CARDF
BIRM
KEELE
ABRDN
QMARY
EDINB
KINGS
NOTTM
UCL
SGEO
BRIST
LOND
SOTON
DUND
IMP
QUEEN
NEWC
SHEF
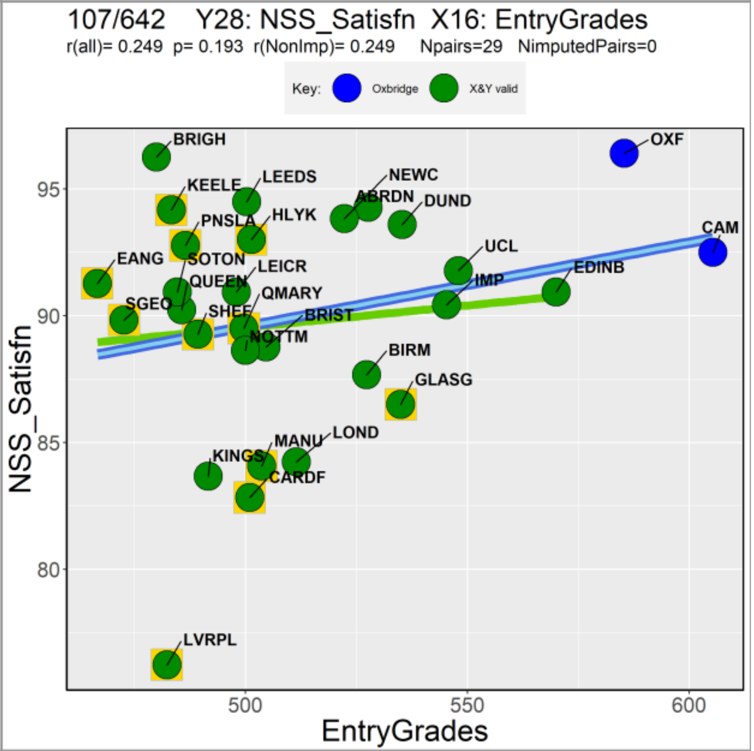
107/642 Y28: NSS_Satisfn X16: EntryGrades
r(all)= 0.249 p= 0.193 r(NonImp)= 0.249 Npairs=29 NimputedPairs=0
Key: Oxbridge X&Y valid
NSS_Satisfn
EntryGrades
BRIGH
OXF
KEELE
LEEDS
NEWC
ABRDN
DUND
PNSLA
HLYK
CAM
EANG
SOTON
UCL
LEICR
QUEEN
IMP
EDINB
QMARY
SGEO
SHEF
BRIST
NOTTM
BIRM
GLASG
MANU
LOND
KINGS
CARDF
LVRPL

108/643 Y29: NSS_Feedback X16: EntryGrades
r(all)= 0.188 p= 0.328 r(NonImp)= 0.188 Npairs=29 NimputedPairs=0

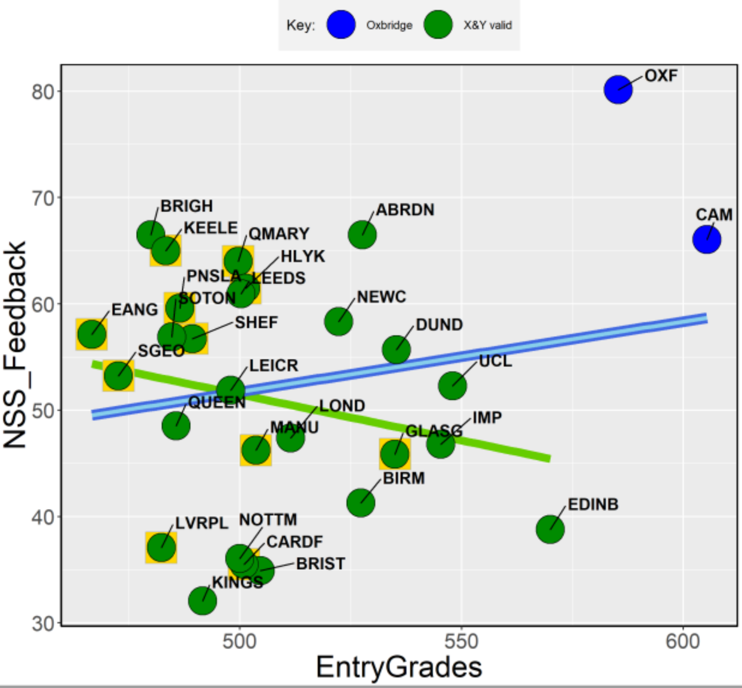

108/644 Y30: UKFPO_EPM X16: EntryGrades
r(all)= 0.472 p= 0.00966 r(NonImp)= 0.472 Npairs=29 NimputedPairs=0

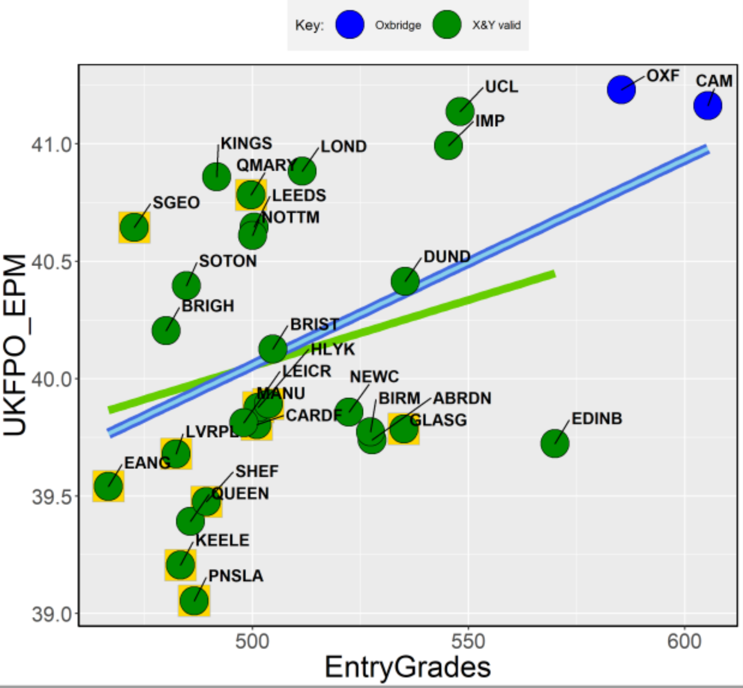

108/645 Y31: UKFPO_SJT X16: EntryGrades
r(all)= 0.794 p= 2.79e-07 r(NonImp)= 0.794 Npairs=29 NimputedPairs=0

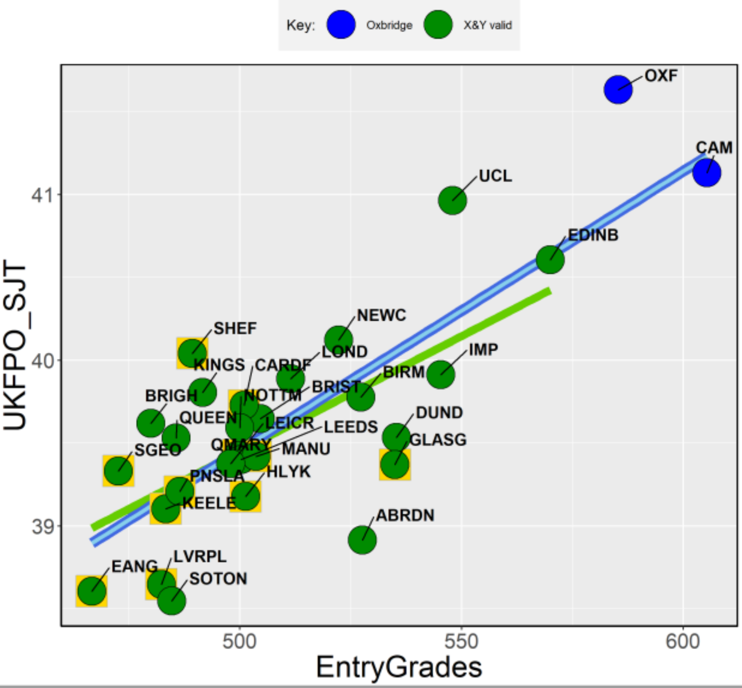

108/646 Y32: F1_Preparedness X16: EntryGrades
r(all)= -0.157 p= 0.415 r(NonImp)= -0.157 Npairs=29 NimputedPairs=0

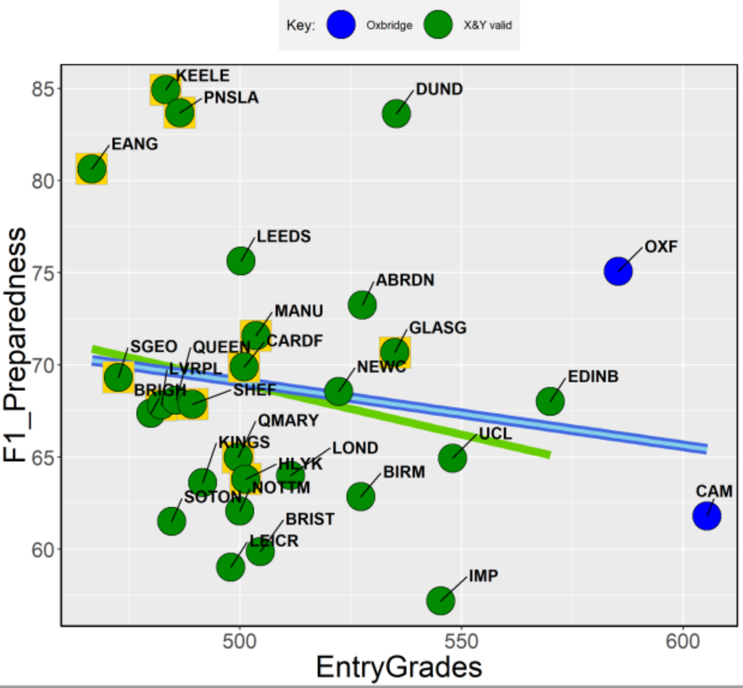

108/647 Y33: F1_Satisfn X16: EntryGrades
r(all)= -0.472 p= 0.00968 r(NonImp)= -0.472 Npairs=29 NimputedPairs=0

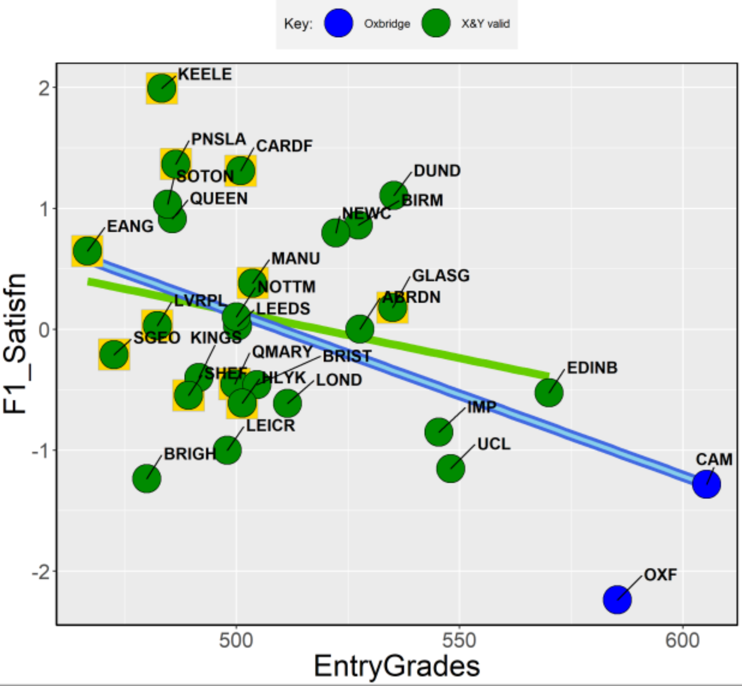

108/648 Y34: F1_Workload X16: EntryGrades
r(all)= -0.133 p= 0.491 r(NonImp)= -0.133 Npairs=29 NimputedPairs=0

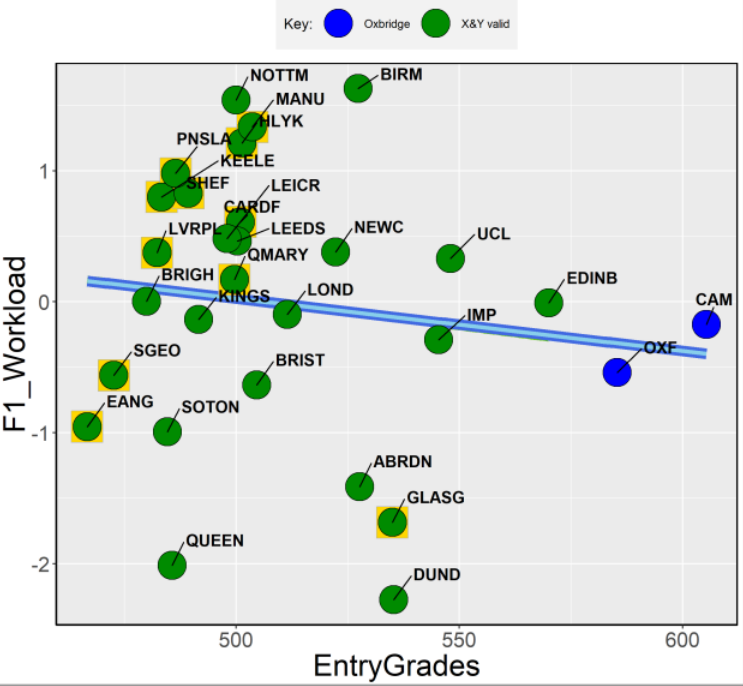

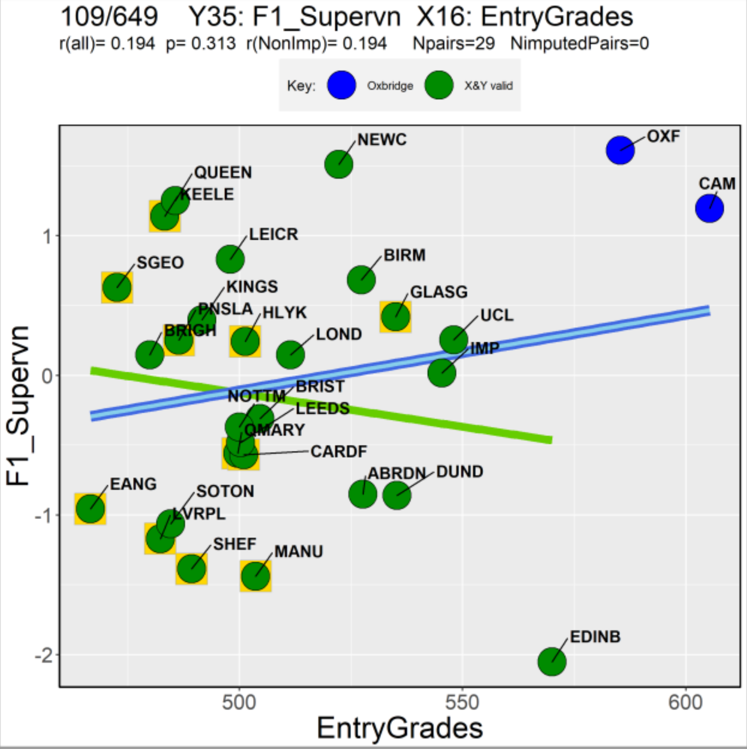
109/649 Y35: F1_Supervn X16: EntryGrades
r(all)= 0.194 p= 0.313 r(NonImp)= 0.194 Npairs=29 NimputedPairs=0
Key: Oxbridge X&Y valid
F1_Supervn
EntryGrades

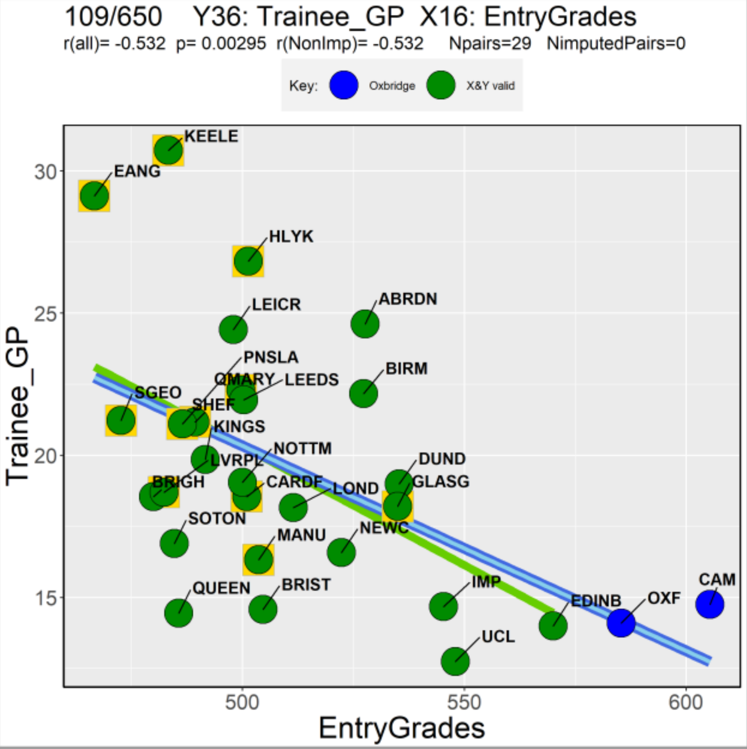
109/650 Y36: Trainee_GP X16: EntryGrades
r(all)= -0.532 p= 0.00295 r(NonImp)= -0.532 Npairs=29 NimputedPairs=0
Key: Oxbridge X&Y valid
Trainee_GP
EntryGrades

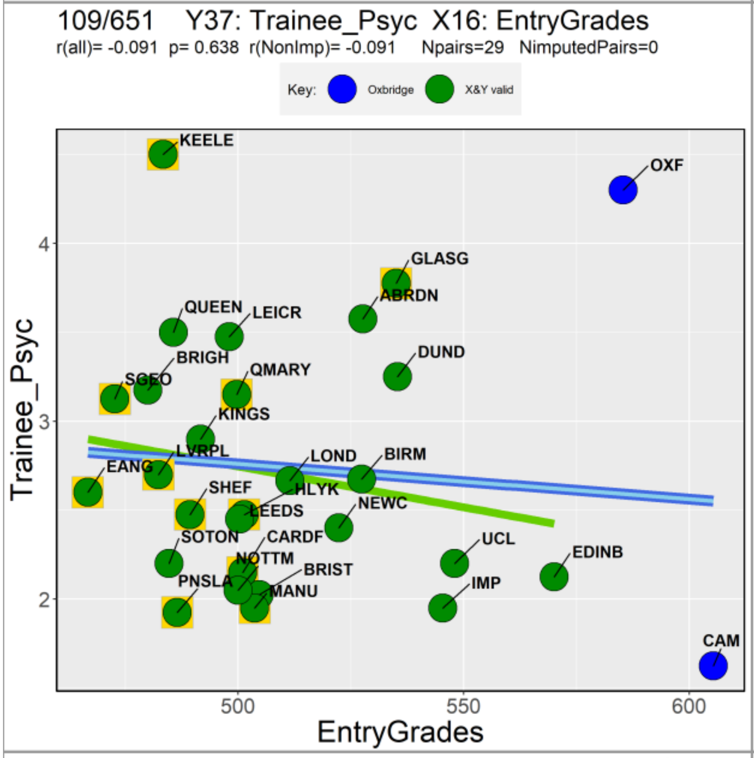
109/651 Y37: Trainee_Psyc X16: EntryGrades
r(all)= -0.091 p= 0.638 r(NonImp)= -0.091 Npairs=29 NimputedPairs=0
Key: Oxbridge X&Y valid
Trainee_Psyc
EntryGrades

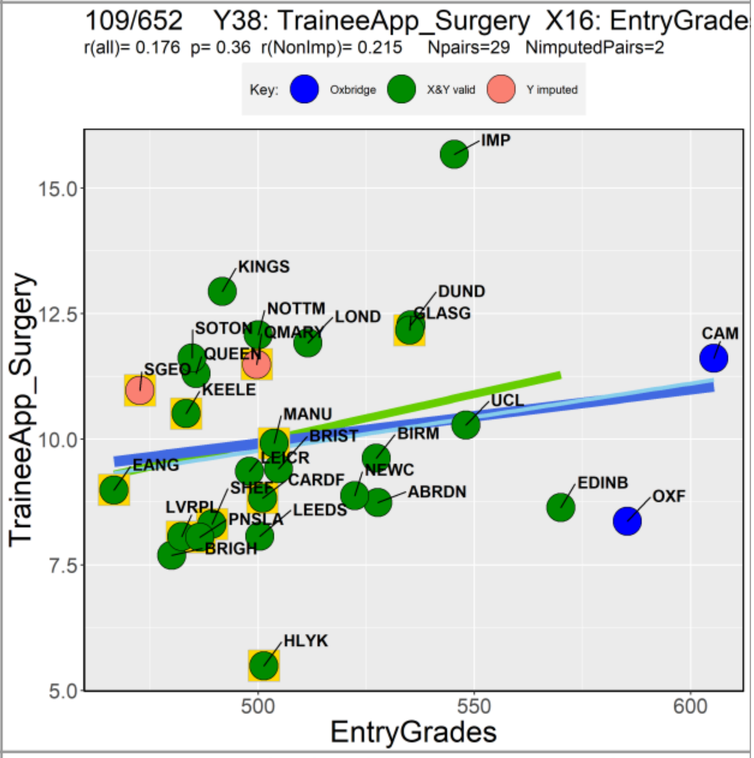
109/652 Y38: TraineeApp_Surgery X16: EntryGrade
r(all)= 0.176 p= 0.36 r(NonImp)= 0.215 Npairs=29 NimputedPairs=2
Key: Oxbridge X&Y valid Y imputed
TraineeApp_Surgery
EntryGrades

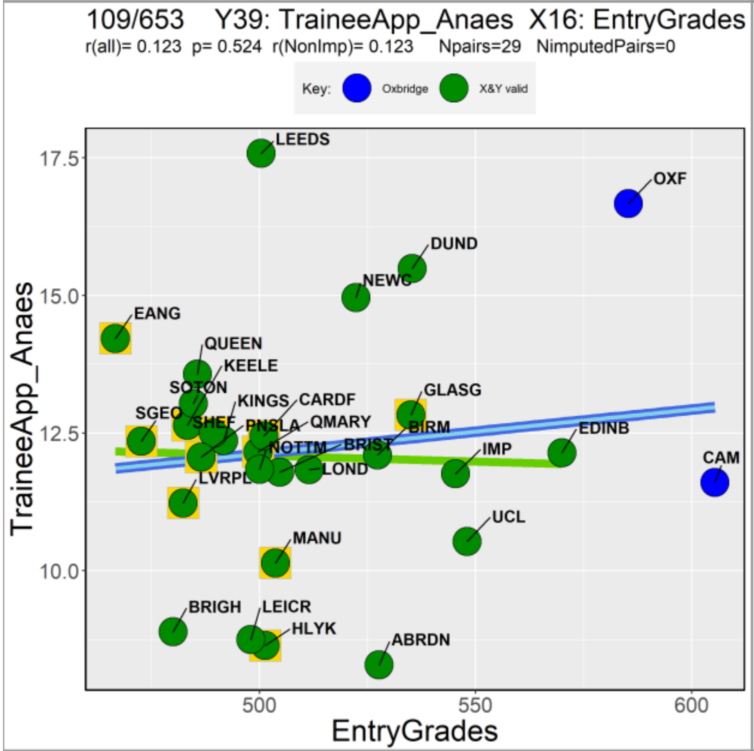
109/653 Y39: TraineeApp_Anaes X16: EntryGrades
r(all)= 0.123 p= 0.524 r(NonImp)= 0.123 Npairs=29 NimputedPairs=0
Key: Oxbridge X&Y valid
TraineeApp_Anaes
EntryGrades

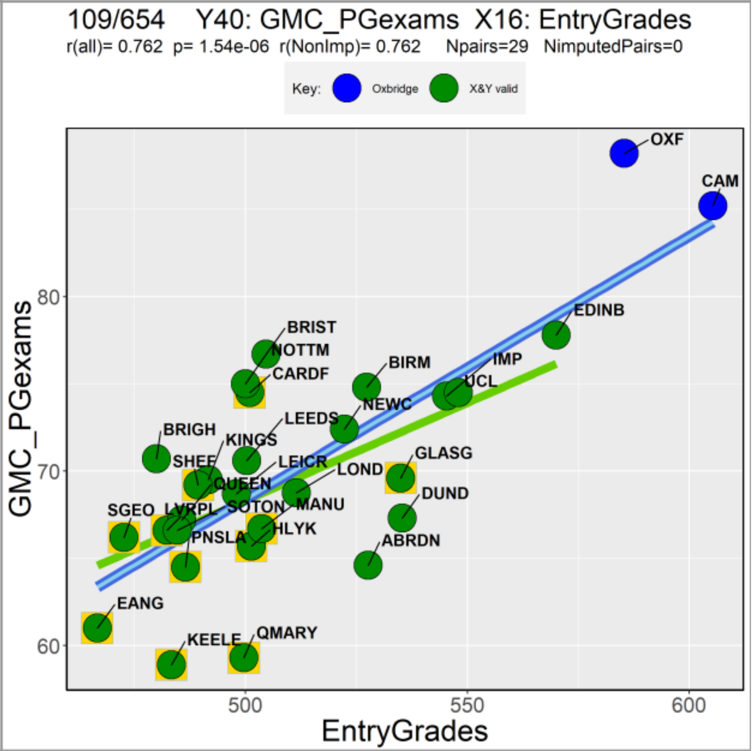
109/654 Y40: GMC_PGexams X16: EntryGrades
r(all)= 0.762 p= 1.54e-06 r(NonImp)= 0.762 Npairs=29 NimputedPairs=0
Key: Oxbridge X&Y valid
GMC_PGexams
EntryGrades

110/655 Y41: MRCGP_AKT X16: EntryGrades

r(all)= 0.675 p= 6e-05 r(NonImp)= 0.675 Npairs=29 NimputedPairs=0

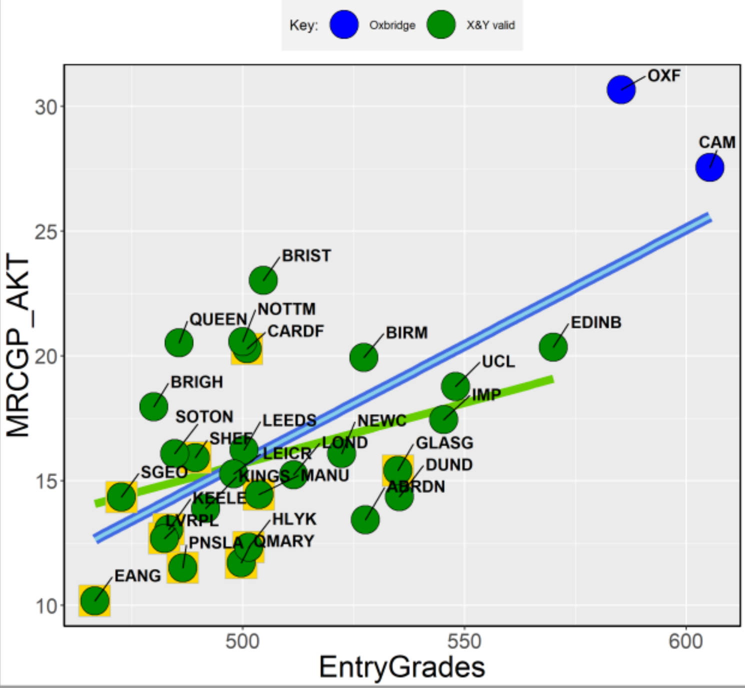

110/656 Y42: MRCGP_CSA X16: EntryGrades

r(all)= 0.567 p= 0.00133 r(NonImp)= 0.567 Npairs=29 NimputedPairs=0

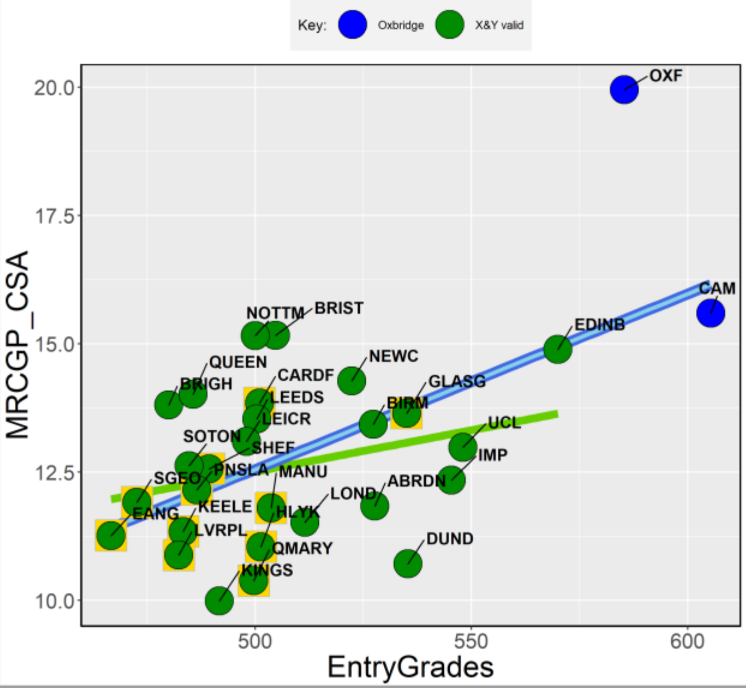

110/657 Y43: FRCA_Pt1 X16: EntryGrades

r(all)= 0.540 p= 0.00252 r(NonImp)= 0.578 Npairs=29 NimputedPairs=10

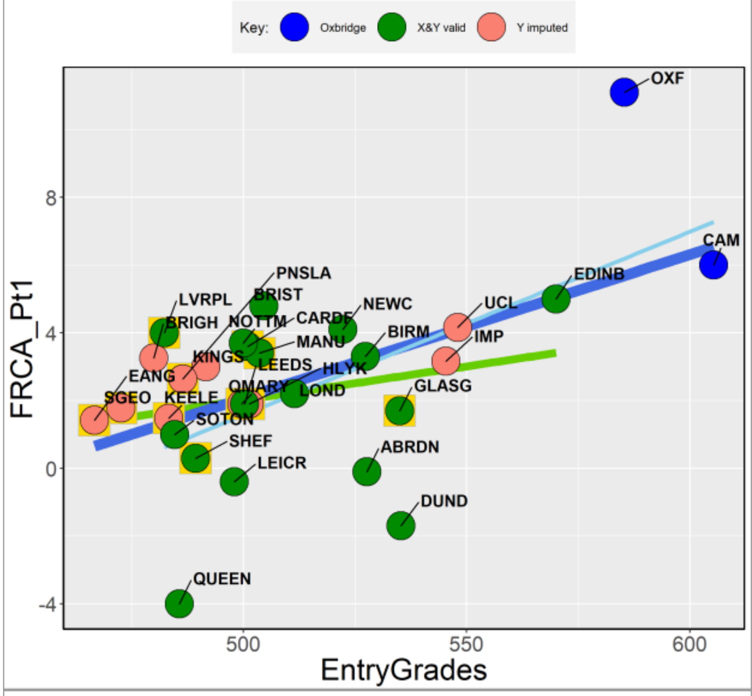

110/658 Y44: MRCOG_Pt1 X16: EntryGrades

r(all)= 0.655 p= 0.000114 r(NonImp)= 0.685 Npairs=29 NimputedPairs=10

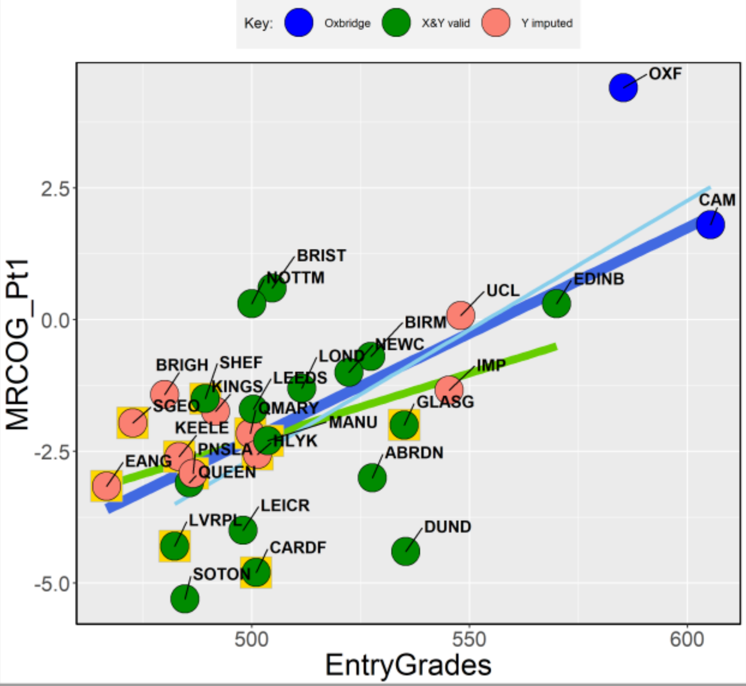

110/659 Y45: MRCOG_Pt2 X16: EntryGrades

r(all)= 0.656 p= 0.000111 r(NonImp)= 0.651 Npairs=29 NimputedPairs=10

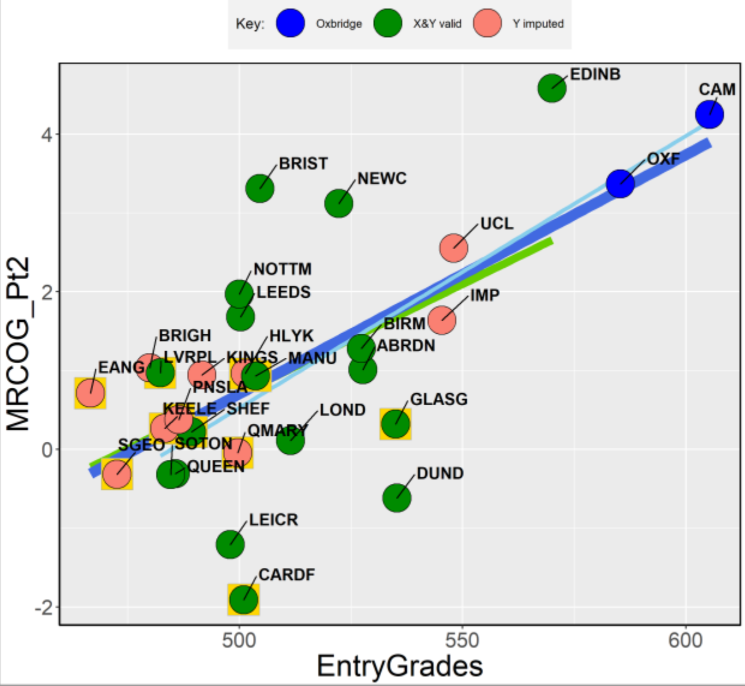

110/660 Y46: MRCP_Pt1 X16: EntryGrades

r(all)= 0.721 p= 1.01e-05 r(NonImp)= 0.748 Npairs=29 NimputedPairs=3

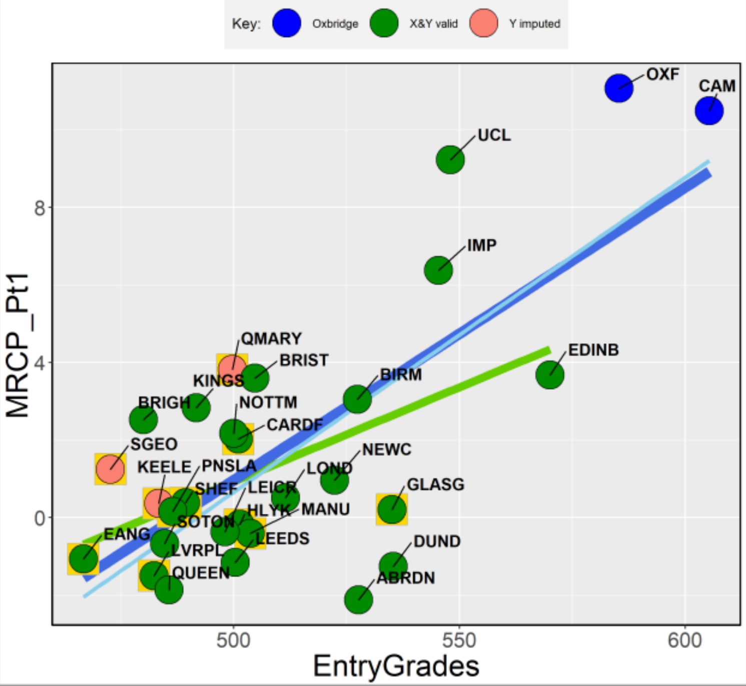

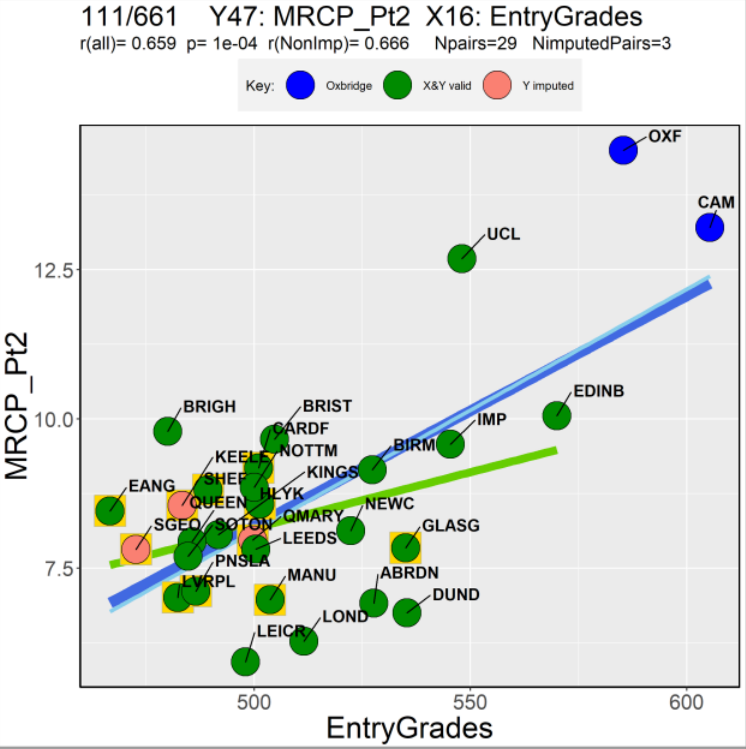
111/661 Y47: MRCP_Pt2 X16: EntryGrades
r(all)= 0.659 p= 1e-04 r(NonImp)= 0.666 Npairs=29 NimputedPairs=3
Key: Oxbridge X&Y valid Y imputed
MRCP_Pt2
EntryGrades
12.5
10.0
7.5
500
550
600
OXF
CAM
UCL
EDINB
IMP
BIRM
BRIGH
BRIST
CARDF
NOTTM
KEELE
KINGS
SHEF
HLYK
EANG
QUEEN
QMARY
NEWC
SGEO
SOTON
LEEDS
GLASG
PNSLA
LVRPL
MANU
ABRDN
DUND
LOND
LEICR

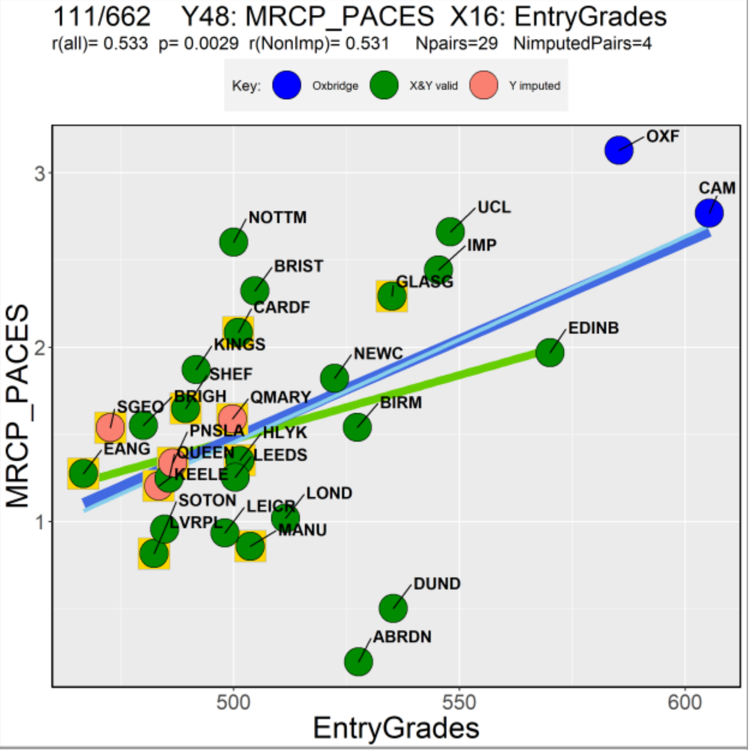
111/662 Y48: MRCP_PACES X16: EntryGrades
r(all)= 0.533 p= 0.0029 r(NonImp)= 0.531 Npairs=29 NimputedPairs=4
Key: Oxbridge X&Y valid Y imputed
MRCP_PACES
EntryGrades
3
2
1
500
550
600
OXF
CAM
UCL
NOTTM
IMP
BRIST
GLASG
CARDF
EDINB
KINGS
NEWC
SHEF
SGEO
BRIGH
QMARY
BIRM
PNSLA
HLYK
EANG
QUEEN
LEEDS
KEELE
SOTON
LEICR
LOND
LVRPL
MANU
DUND
ABRDN

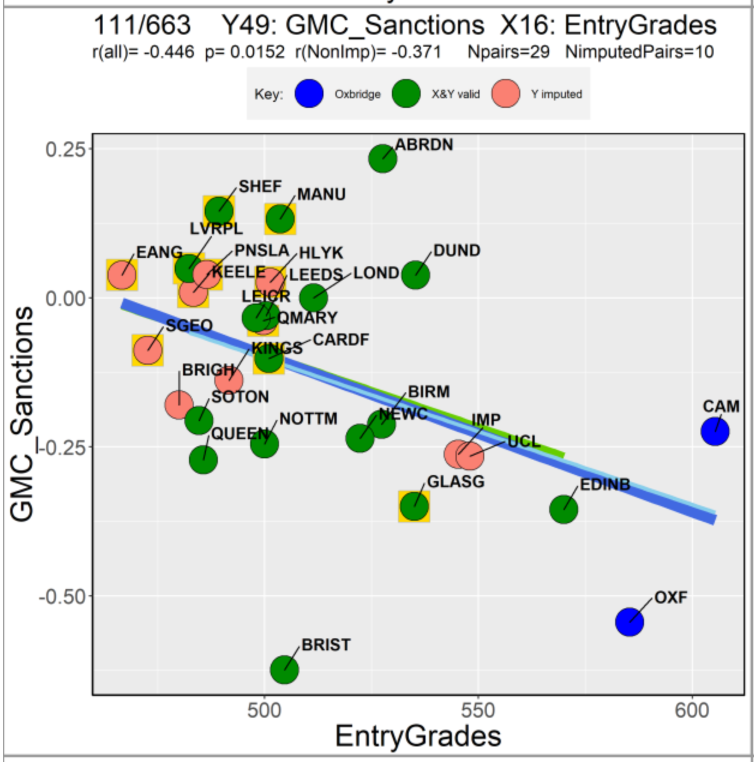
111/663 Y49: GMC_Sanctions X16: EntryGrades
r(all)= -0.446 p= 0.0152 r(NonImp)= -0.371 Npairs=29 NimputedPairs=10
Key: Oxbridge X&Y valid Y imputed
GMC_Sanctions
EntryGrades
0.25
0.00
-0.25
-0.50
500
550
600
ABRDN
SHEF
MANU
LVRPL
EANG
PNSLA
HLYK
DUND
KEELE
LEEDS
LOND
LEICR
QMARY
SGEO
KINGS
CARDF
BRIGH
SOTON
BIRM
CAM
NOTTM
NEWC
IMP
QUEEN
UCL
GLASG
EDINB
OXF
BRIST

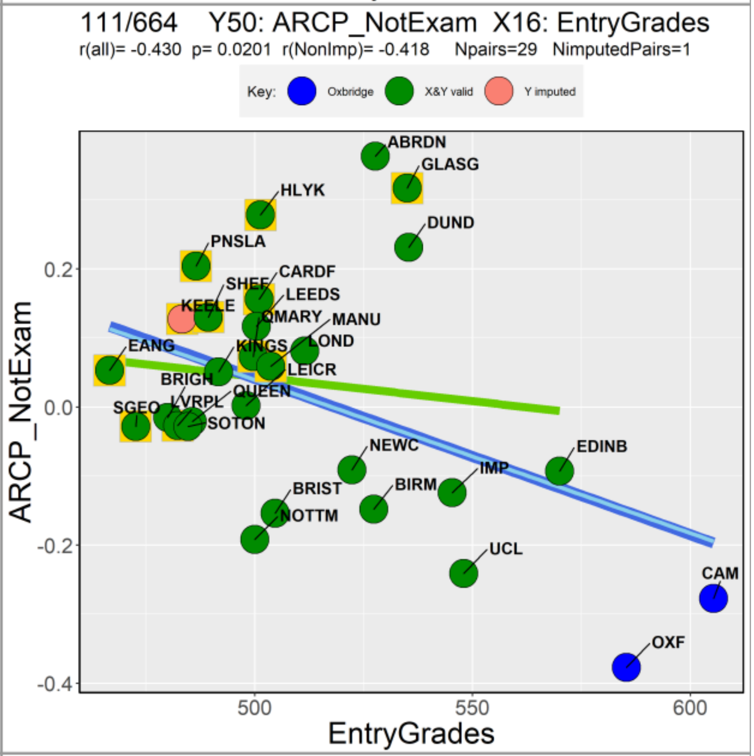
111/664 Y50: ARCP_NotExam X16: EntryGrades
r(all)= -0.430 p= 0.0201 r(NonImp)= -0.418 Npairs=29 NimputedPairs=1
Key: Oxbridge X&Y valid Y imputed
ARCP_NotExam
EntryGrades
0.2
0.0
-0.2
-0.4
500
550
600
ABRDN
GLASG
HLYK
DUND
PNSLA
CARDF
SHEF
LEEDS
KEELE
QMARY
MANU
EANG
KINGS
LOND
LEICR
BRIGH
QUEEN
SGEO
LVRPL
SOTON
NEWC
EDINB
BRIST
BIRM
IMP
NOTTM
UCL
CAM
OXF

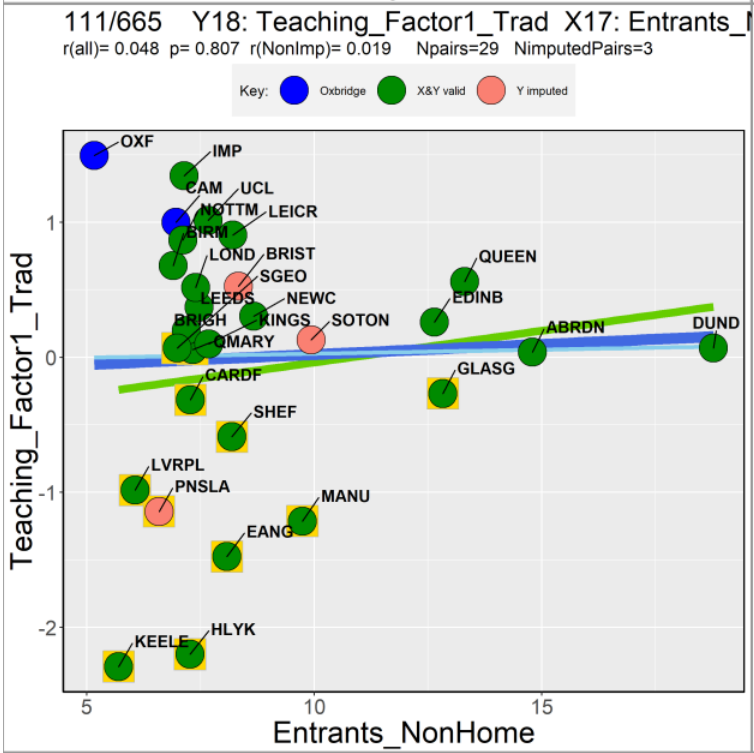
111/665 Y18: Teaching_Factor1_Trad X17: Entrants_N
r(all)= 0.048 p= 0.807 r(NonImp)= 0.019 Npairs=29 NimputedPairs=3
Key: Oxbridge X&Y valid Y imputed
Teaching_Factor1_Trad
Entrants_NonHome
1
0
-1
-2
5
10
15
OXF
IMP
CAM
UCL
NOTTM
LEICR
BIRM
LOND
BRIST
SGEO
QUEEN
LEEDS
NEWC
EDINB
BRIGH
KINGS
SOTON
QMARY
ABRDN
DUND
CARDF
GLASG
SHEF
LVRPL
PNSLA
MANU
EANG
KEELE
HLYK

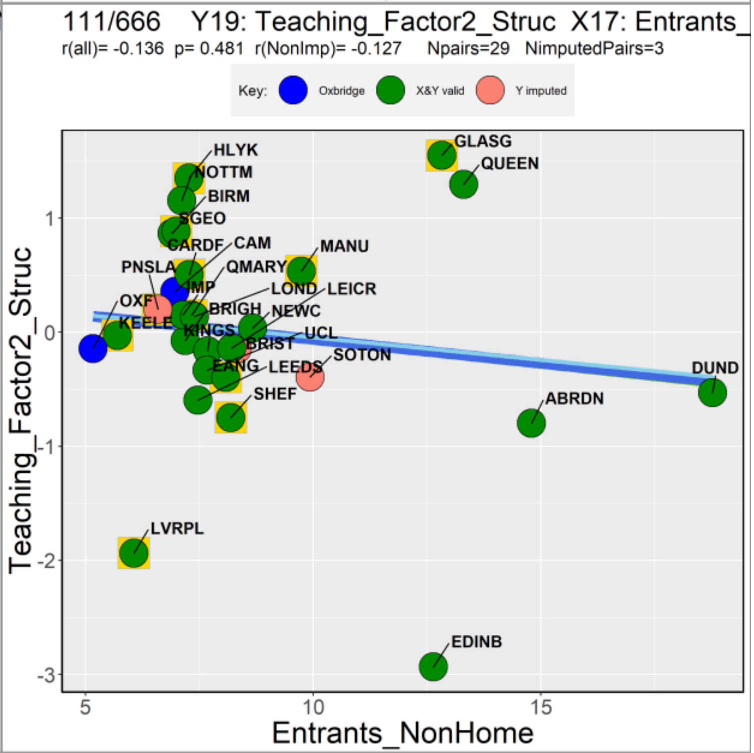
111/666 Y19: Teaching_Factor2_Struc X17: Entrants_
r(all)= -0.136 p= 0.481 r(NonImp)= -0.127 Npairs=29 NimputedPairs=3
Key: Oxbridge X&Y valid Y imputed
Teaching_Factor2_Struc
Entrants_NonHome
1
0
-1
-2
-3
5
10
15
HLYK
GLASG
NOTTM
QUEEN
BIRM
SGEO
CARDF
CAM
MANU
PNSLA
QMARY
IMP
LOND
LEICR
OXF
BRIGH
NEWC
KEELE
KINGS
UCL
BRIST
SOTON
EANG
LEEDS
DUND
SHEF
ABRDN
LVRPL
EDINB

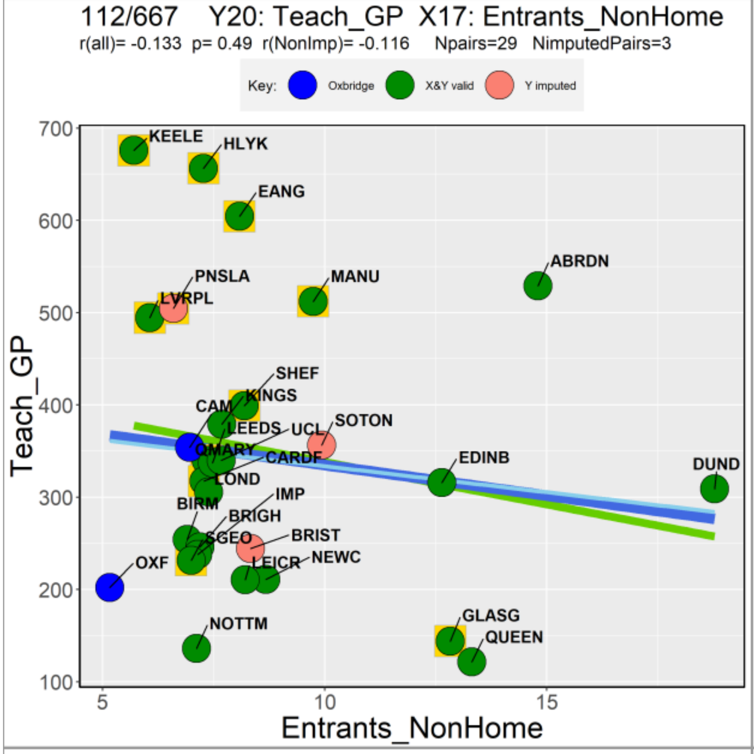
112/667 Y20: Teach_GP X17: Entrants_NonHome
r(all)= -0.133 p= 0.49 r(NonImp)= -0.116 Npairs=29 NimputedPairs=3
Key: Oxbridge X&Y valid Y imputed
Teach_GP
Entrants_NonHome
KEELE
HLYK
EANG
PNSLA
MANU
ABRDN
LVRPL
SHEF
KINGS
CAM
LEEDS
UCL
SOTON
QMARY
CARDF
EDINB
DUND
LOND
IMP
BIRM
BRIGH
SGEO
BRIST
OXF
LEICR
NEWC
NOTTM
GLASG
QUEEN

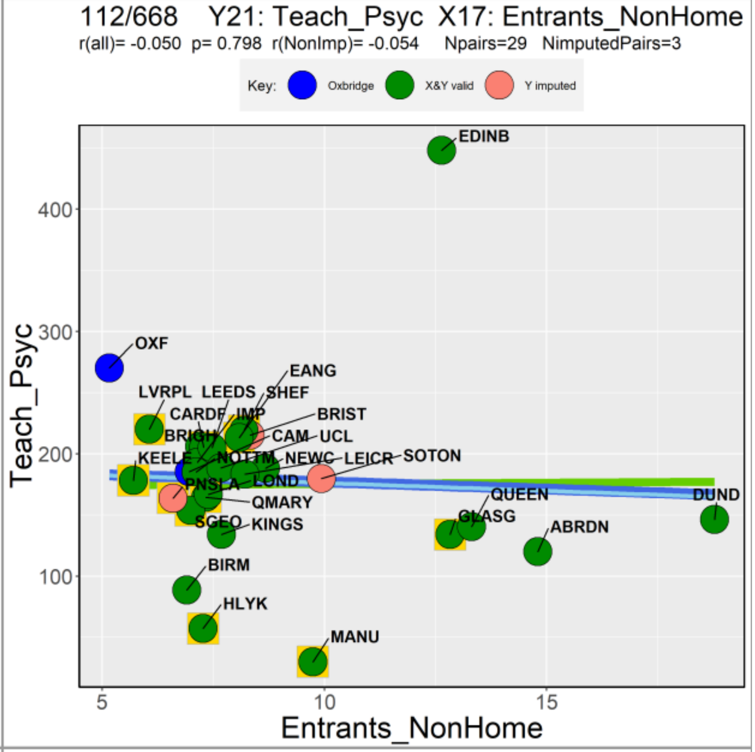
112/668 Y21: Teach_Psyc X17: Entrants_NonHome
r(all)= -0.050 p= 0.798 r(NonImp)= -0.054 Npairs=29 NimputedPairs=3
Key: Oxbridge X&Y valid Y imputed
Teach_Psyc
Entrants_NonHome
EDINB
OXF
EANG
LVRPL
LEEDS
SHEF
CARDF
IMP
BRIST
BRIGH
CAM
UCL
KEELE
NOTTM
NEWC
LEICR
SOTON
PNSLA
LOND
QMARY
QUEEN
GLASG
DUND
SGEO
KINGS
ABRDN
BIRM
HLYK
MANU

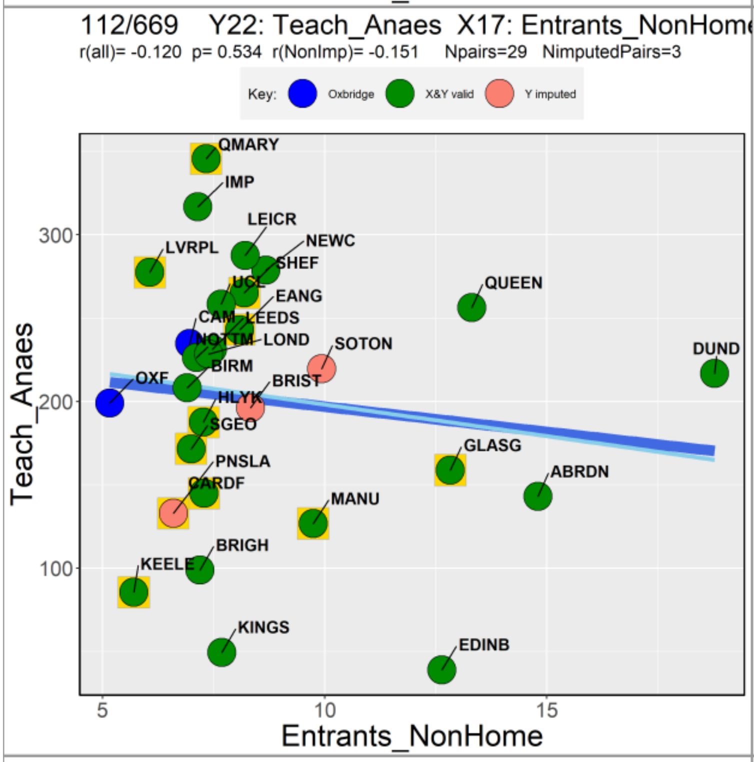
112/669 Y22: Teach_Anaes X17: Entrants_NonHome
r(all)= -0.120 p= 0.534 r(NonImp)= -0.151 Npairs=29 NimputedPairs=3
Key: Oxbridge X&Y valid Y imputed
Teach_Anaes
Entrants_NonHome
QMARY
IMP
LEICR
LVRPL
NEWC
SHEF
QUEEN
UCL
EANG
CAM
LEEDS
NOTTM
LOND
SOTON
DUND
BIRM
OXF
BRIST
HLYK
SGEO
GLASG
PNSLA
ABRDN
CARDF
MANU
BRIGH
KEELE
KINGS
EDINB

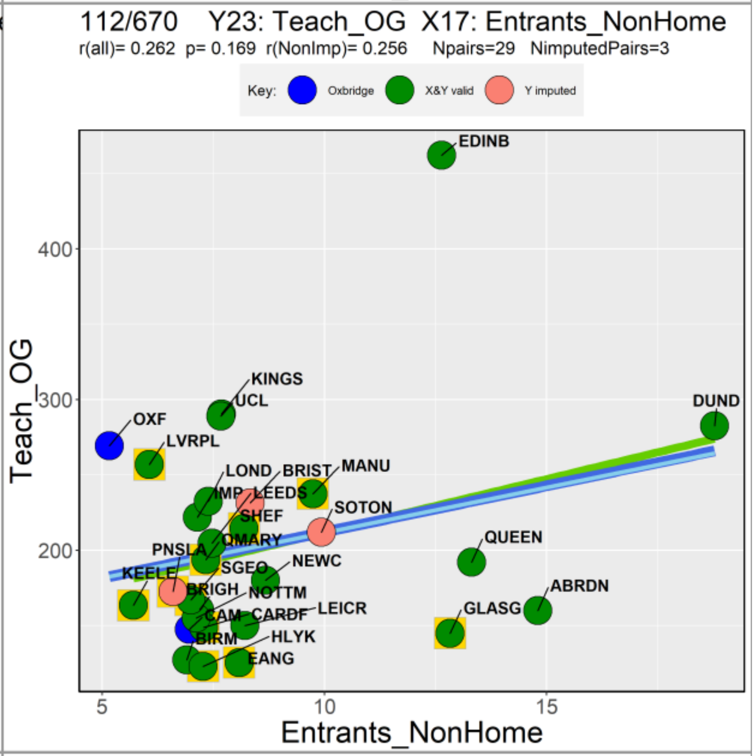
112/670 Y23: Teach_OG X17: Entrants_NonHome
r(all)= 0.262 p= 0.169 r(NonImp)= 0.256 Npairs=29 NimputedPairs=3
Key: Oxbridge X&Y valid Y imputed
Teach_OG
Entrants_NonHome
EDINB
KINGS
UCL
DUND
OXF
LVRPL
LOND
BRIST
MANU
IMP
LEEDS
SHEF
SOTON
QMARY
QUEEN
PNSLA
SGEO
NEWC
KEELE
BRIGH
NOTTM
ABRDN
CAM
CARDF
LEICR
GLASG
BIRM
HLYK
EANG

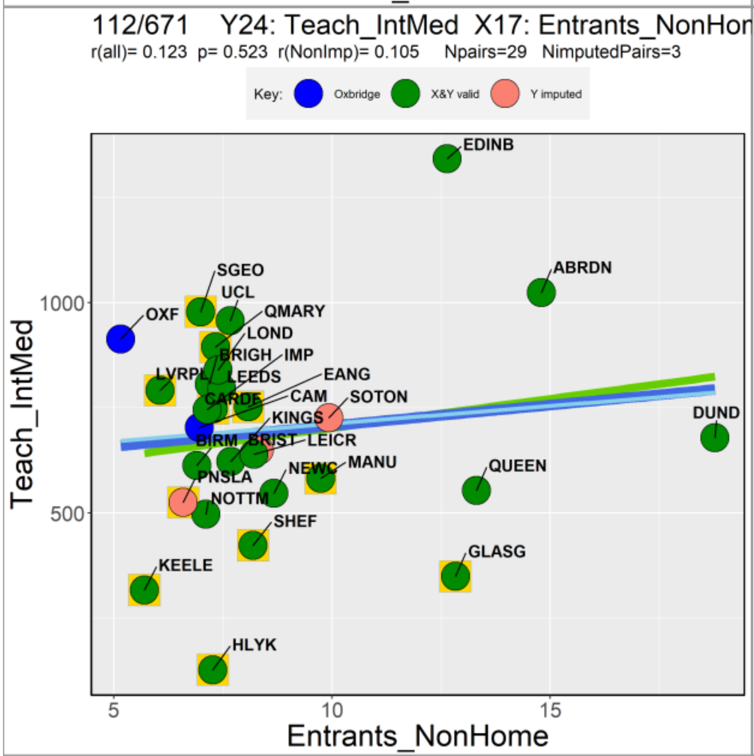
112/671 Y24: Teach_IntMed X17: Entrants_NonHor
r(all)= 0.123 p= 0.523 r(NonImp)= 0.105 Npairs=29 NimputedPairs=3
Key: Oxbridge X&Y valid Y imputed
Teach_IntMed
Entrants_NonHome
EDINB
ABRDN
SGEO
UCL
OXF
QMARY
LOND
BRIGH
IMP
LVRPL
LEEDS
EANG
CARDF
CAM
SOTON
KINGS
DUND
BIRM
BRIST
LEICR
NEWC
MANU
QUEEN
PNSLA
NOTTM
SHEF
GLASG
KEELE
HLYK

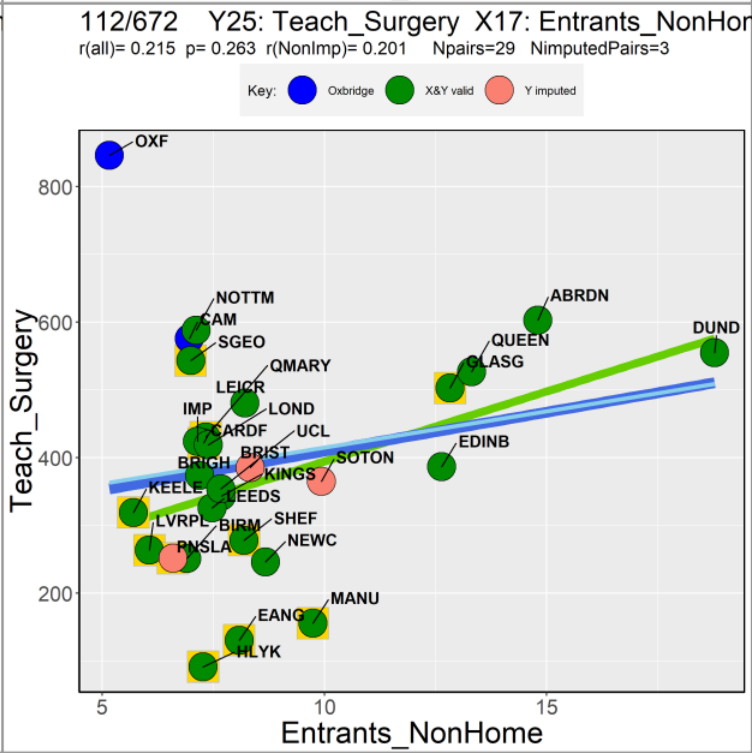
112/672 Y25: Teach_Surgery X17: Entrants_NonHor
r(all)= 0.215 p= 0.263 r(NonImp)= 0.201 Npairs=29 NimputedPairs=3
Key: Oxbridge X&Y valid Y imputed
Teach_Surgery
Entrants_NonHome
OXF
NOTTM
CAM
SGEO
ABRDN
QUEEN
GLASG
DUND
QMARY
LEICR
IMP
LOND
CARDF
UCL
EDINB
BRIST
BRIGH
SOTON
KINGS
KEELE
LEEDS
LVRPL
BIRM
SHEF
PNSLA
NEWC
MANU
EANG
HLYK

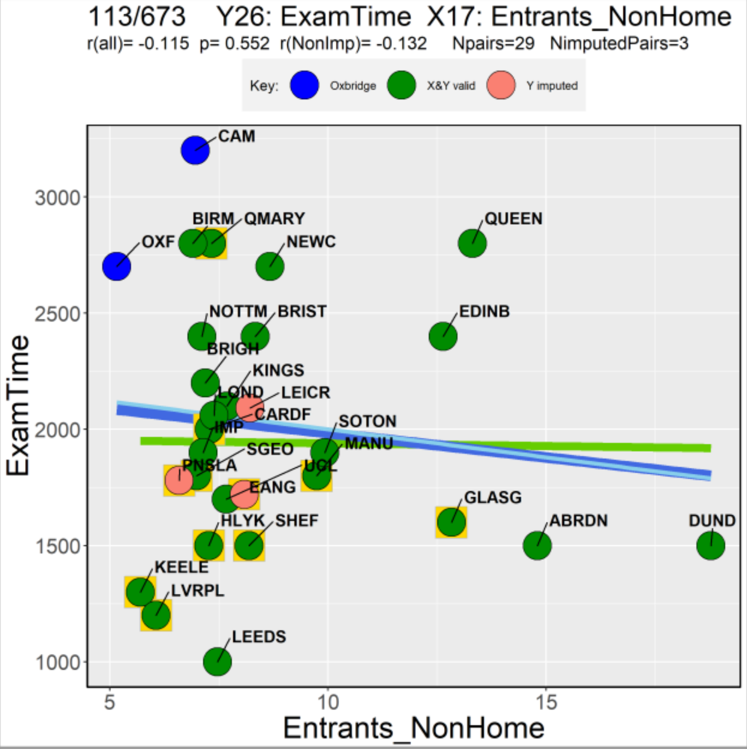
113/673 Y26: ExamTime X17: Entrants_NonHome
r(all)= -0.115 p= 0.552 r(NonImp)= -0.132 Npairs=29 NimputedPairs=3
Key: Oxbridge X&Y valid Y imputed
ExamTime
Entrants_NonHome
CAM
OXF
BIRM
QMARY
NEWC
QUEEN
NOTTM
BRIST
EDINB
BRIGH
KINGS
LOND
LEICR
CARDF
IMP
SOTON
MANU
SGEO
PNSLA
UCL
EANG
GLASG
HLYK
SHEF
ABRDN
DUND
KEELE
LVRPL
LEEDS

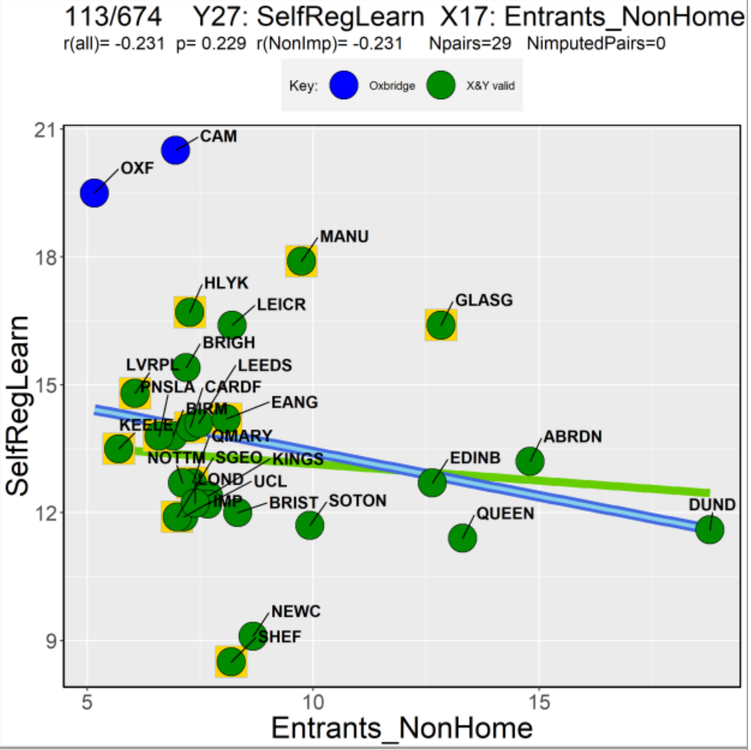
113/674 Y27: SelfRegLearn X17: Entrants_NonHome
r(all)= -0.231 p= 0.229 r(NonImp)= -0.231 Npairs=29 NimputedPairs=0
Key: Oxbridge X&Y valid
SelfRegLearn
Entrants_NonHome
CAM
OXF
MANU
HLYK
LEICR
GLASG
BRIGH
LVRPL
LEEDS
PNSLA
CARDF
EANG
BIRM
KEELE
QMARY
ABRDN
NOTTM
SGEO
KINGS
EDINB
LOND
UCL
IMP
BRIST
SOTON
QUEEN
DUND
NEWC
SHEF

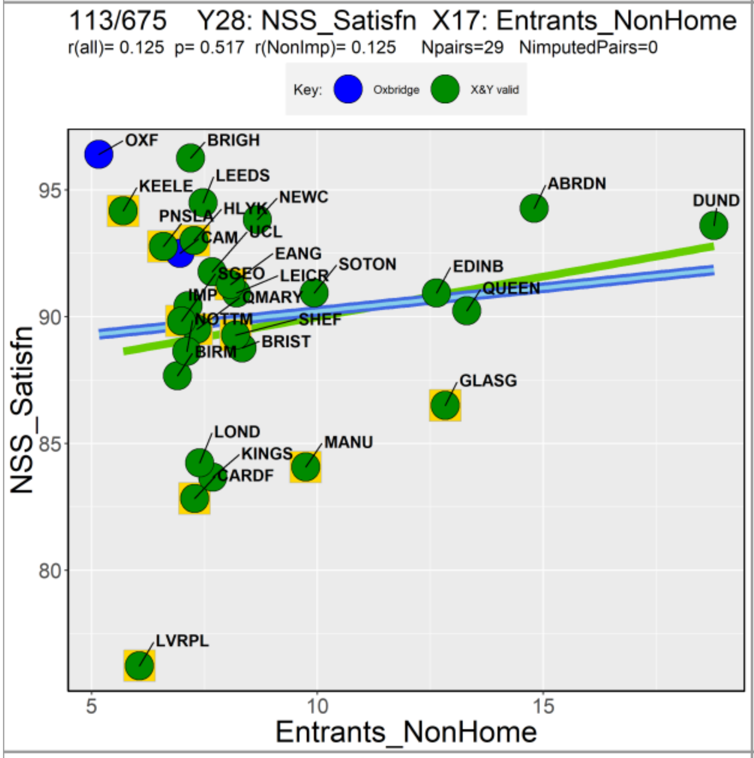
113/675 Y28: NSS_Satisfn X17: Entrants_NonHome
r(all)= 0.125 p= 0.517 r(NonImp)= 0.125 Npairs=29 NimputedPairs=0
Key: Oxbridge X&Y valid
NSS_Satisfn
Entrants_NonHome
OXF
BRIGH
KEELE
LEEDS
ABRDN
NEWC
PNSLA
HLYK
DUND
CAM
UCL
EANG
SOTON
SGEO
LEICR
EDINB
IMP
QUEEN
QMARY
NOTTM
SHEF
BRIST
BIRM
GLASG
LOND
KINGS
MANU
CARDF
LVRPL

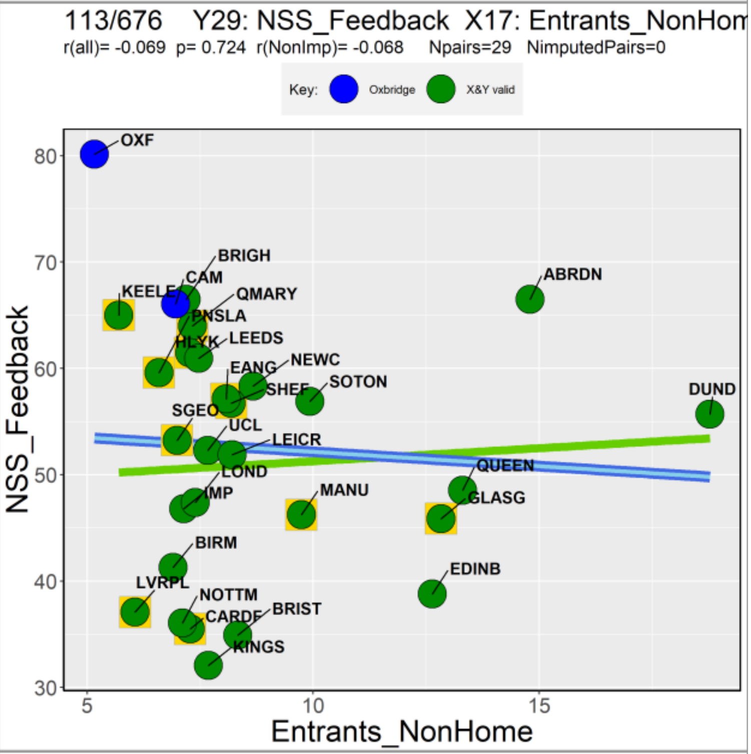
113/676 Y29: NSS_Feedback X17: Entrants_NonHom
r(all)= -0.069 p= 0.724 r(NonImp)= -0.068 Npairs=29 NimputedPairs=0
Key: Oxbridge X&Y valid
NSS_Feedback
Entrants_NonHome
OXF
BRIGH
ABRDN
CAM
KEELE
QMARY
PNSLA
HLYK
LEEDS
NEWC
EANG
SHEF
SOTON
DUND
SGEO
UCL
LEICR
QUEEN
LOND
IMP
MANU
GLASG
BIRM
EDINB
LVRPL
NOTTM
CARDF
BRIST
KINGS

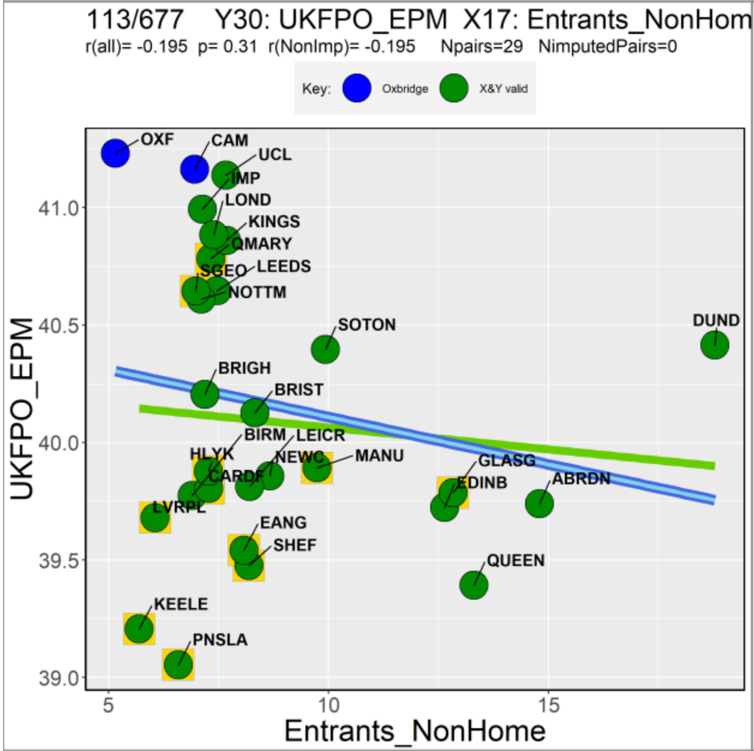
113/677 Y30: UKFPO_EPM X17: Entrants_NonHom
r(all)= -0.195 p= 0.31 r(NonImp)= -0.195 Npairs=29 NimputedPairs=0
Key: Oxbridge X&Y valid
UKFPO_EPM
Entrants_NonHome
OXF
CAM
UCL
IMP
LOND
KINGS
QMARY
SGEO
LEEDS
NOTTM
SOTON
DUND
BRIGH
BRIST
BIRM
LEICR
HLYK
NEWC
MANU
CARDF
GLASG
EDINB
ABRDN
LVRPL
EANG
SHEF
QUEEN
KEELE
PNSLA

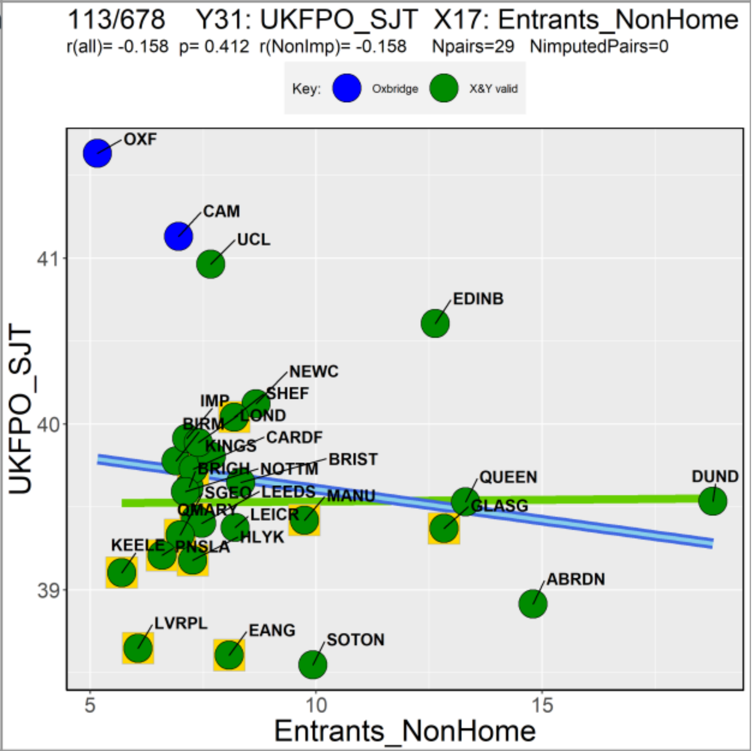
113/678 Y31: UKFPO_SJT X17: Entrants_NonHome
r(all)= -0.158 p= 0.412 r(NonImp)= -0.158 Npairs=29 NimputedPairs=0
Key: Oxbridge X&Y valid
UKFPO_SJT
Entrants_NonHome
OXF
CAM
UCL
EDINB
NEWC
IMP
SHEF
LOND
BIRM
KINGS
CARDF
BRIGH
NOTTM
BRIST
QUEEN
DUND
SGEO
LEEDS
MANU
GLASG
QMARY
LEICR
HLYK
KEELE
PNSLA
ABRDN
LVRPL
EANG
SOTON

114/679 Y32: F1_Preparedness X17: Entrants_NonH

r(all)= 0.228 p= 0.234 r(NonImp)= 0.228 Npairs=29 NimputedPairs=0

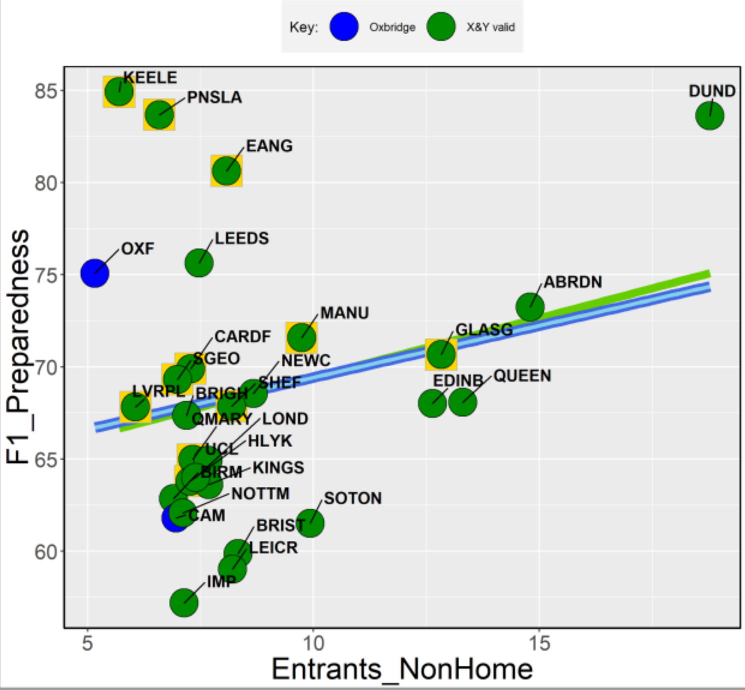

114/680 Y33: F1_Satisfn X17: Entrants_NonHome

r(all)= 0.261 p= 0.171 r(NonImp)= 0.261 Npairs=29 NimputedPairs=0

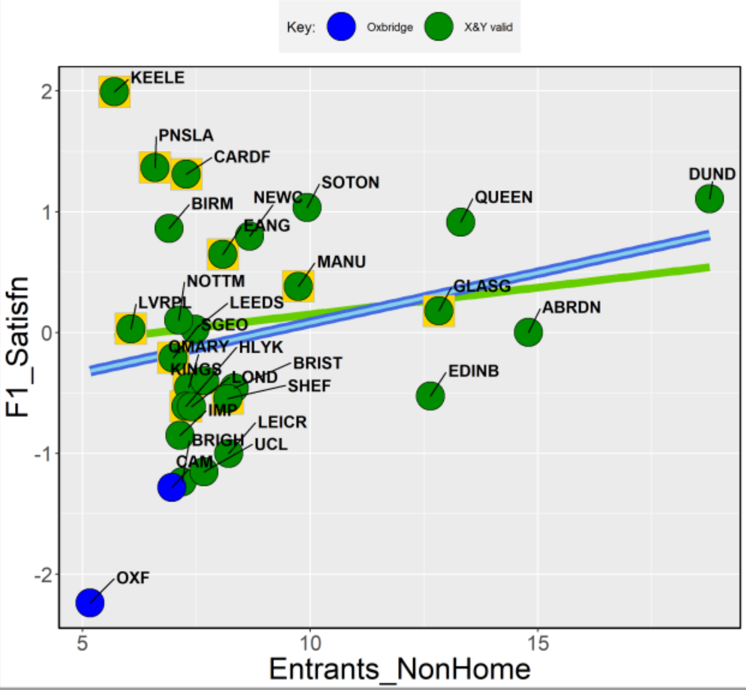

114/681 Y34: F1_Workload X17: Entrants_NonHome

r(all)= -0.675 p= 5.82e-05 r(NonImp)= -0.675 Npairs=29 NimputedPairs=0

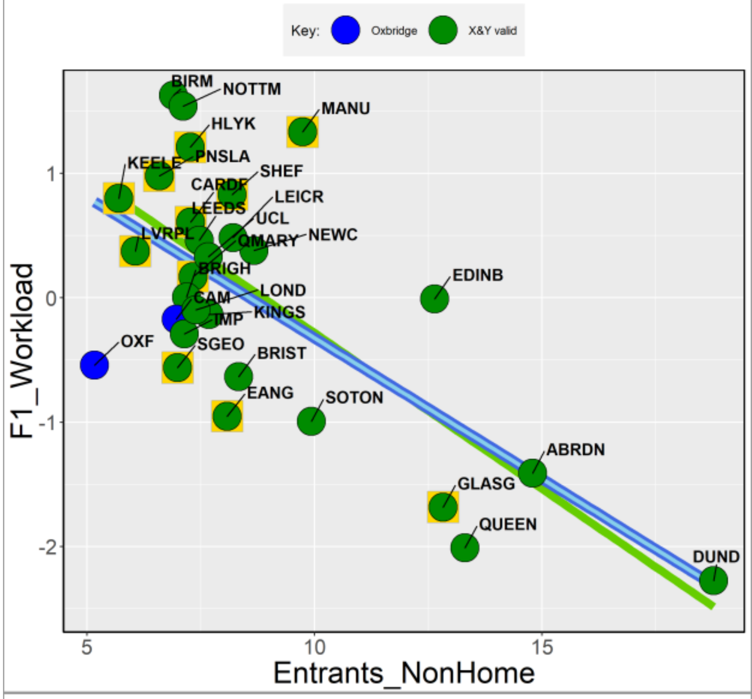

114/682 Y35: F1_Supervn X17: Entrants_NonHome

r(all)= -0.321 p= 0.0892 r(NonImp)= -0.321 Npairs=29 NimputedPairs=0

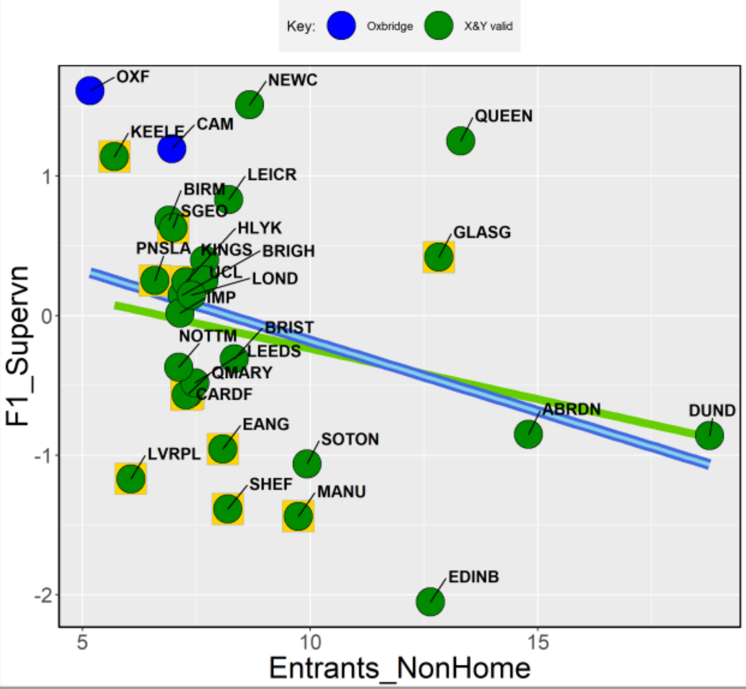

114/683 Y36: Trainee_GP X17: Entrants_NonHome

r(all)= -0.132 p= 0.494 r(NonImp)= -0.132 Npairs=29 NimputedPairs=0

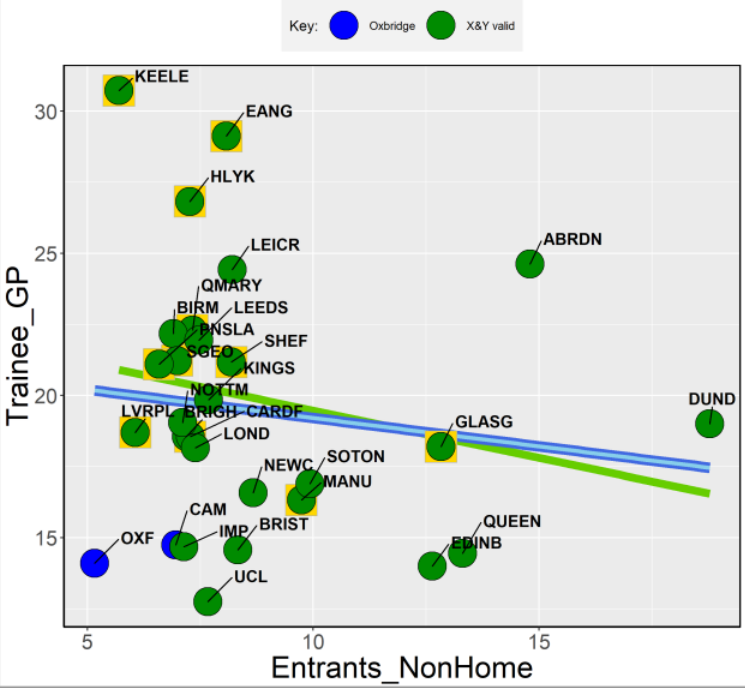

114/684 Y37: Trainee_Psyc X17: Entrants_NonHome

r(all)= 0.158 p= 0.412 r(NonImp)= 0.158 Npairs=29 NimputedPairs=0

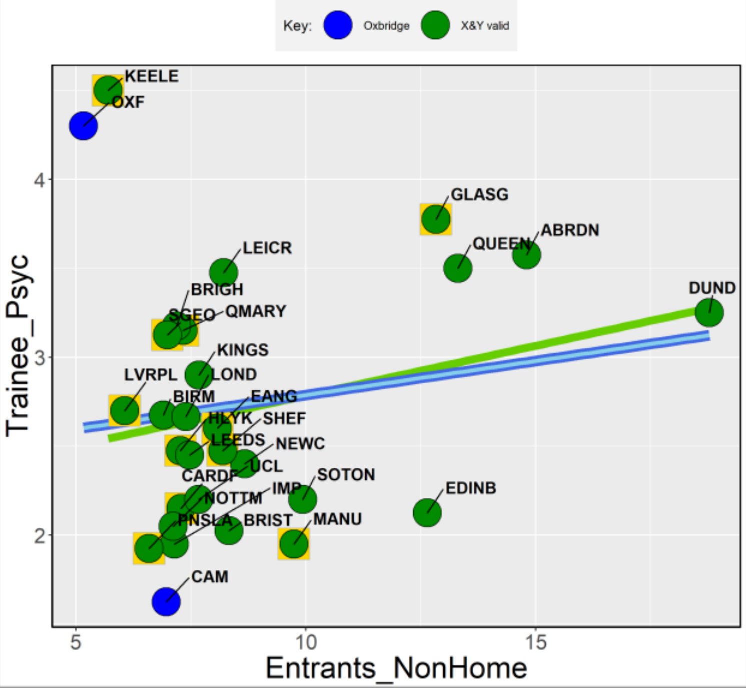

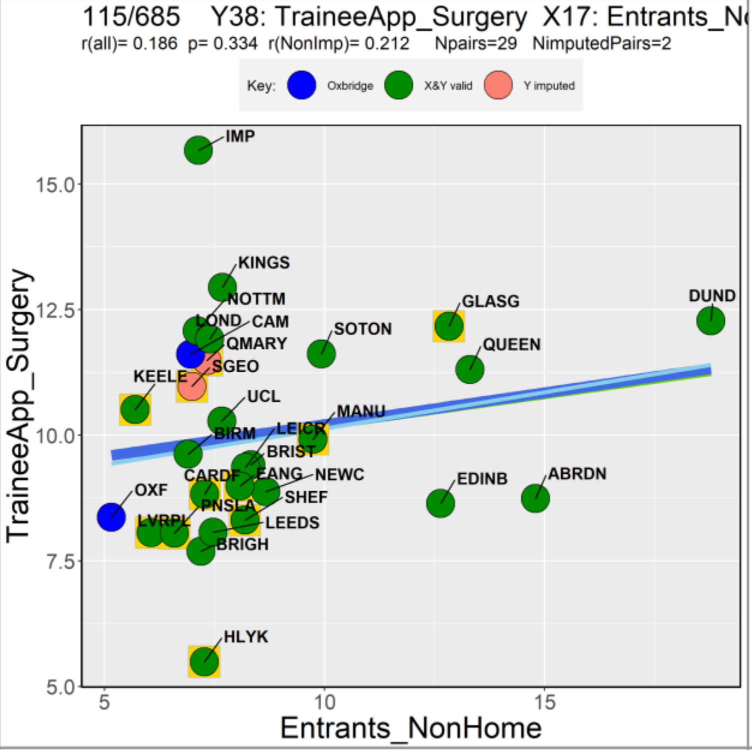
115/685 Y38: TraineeApp_Surgery X17: Entrants_N
r(all)= 0.186 p= 0.334 r(NonImp)= 0.212 Npairs=29 NimputedPairs=2
Key: Oxbridge X&Y valid Y imputed
TraineeApp_Surgery
Entrants_NonHome
IMP KINGS NOTTM LOND CAM QMARY SGEO KEELE UCL BIRM LEICR BRIST MANU CARDF EANG NEWC SHEF PNSLA LEEDS LVRPL BRIGH HLYK OXF SOTON GLASG QUEEN EDINB ABRDN DUND

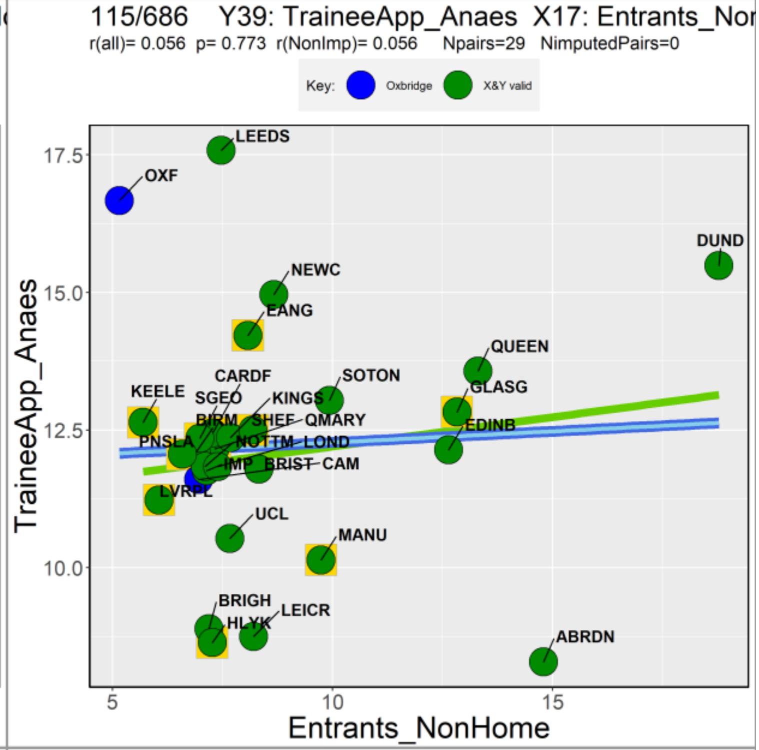
115/686 Y39: TraineeApp_Anaes X17: Entrants_Nor
r(all)= 0.056 p= 0.773 r(NonImp)= 0.056 Npairs=29 NimputedPairs=0
Key: Oxbridge X&Y valid
TraineeApp_Anaes
Entrants_NonHome
LEEDS OXF NEWC EANG CARDF KEELE SGEO KINGS SOTON QUEEN GLASG BIRM SHEF QMARY PNSLA NOTTM LOND IMP BRIST CAM EDINB LVRPL UCL MANU BRIGH HLYK LEICR ABRDN DUND

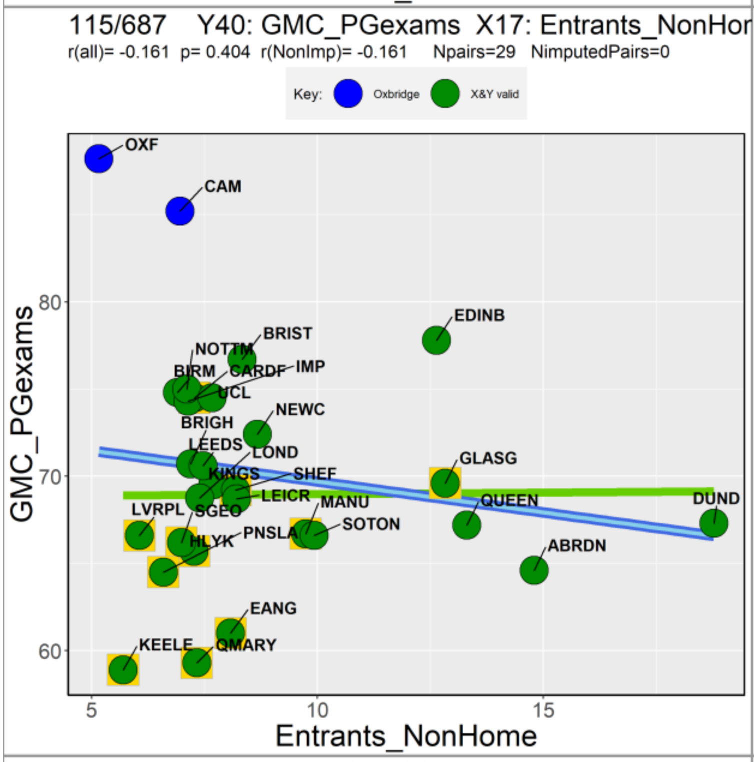
115/687 Y40: GMC_PGexams X17: Entrants_NonHor
r(all)= -0.161 p= 0.404 r(NonImp)= -0.161 Npairs=29 NimputedPairs=0
Key: Oxbridge X&Y valid
GMC_PGexams
Entrants_NonHome
OXF CAM EDINB BRIST NOTTM BIRM CARDF IMP UCL NEWC BRIGH LEEDS LOND KINGS SHEF LEICR LVRPL SGEO MANU SOTON HLYK PNSLA GLASG QUEEN DUND ABRDN EANG KEELE QMARY

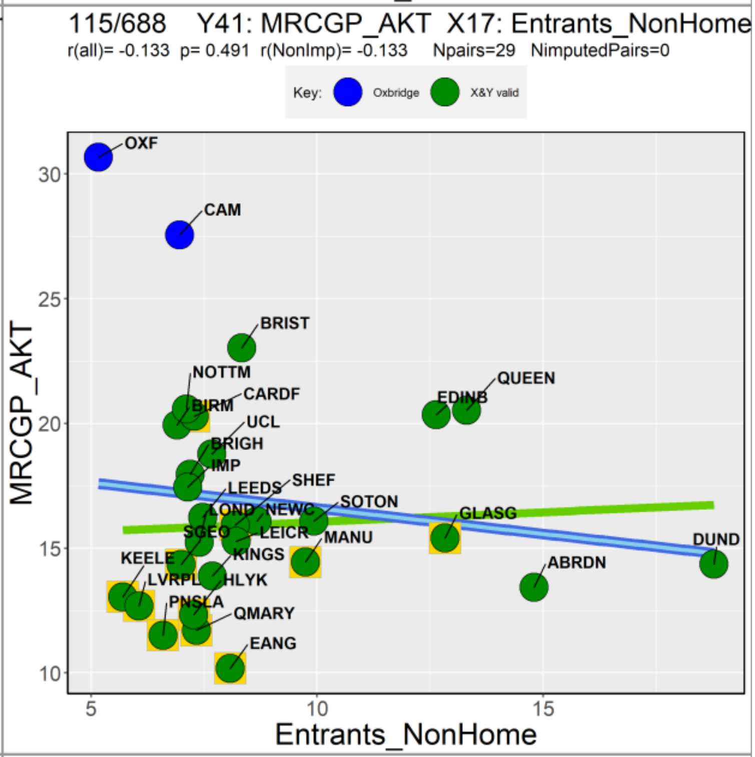
115/688 Y41: MRCGP_AKT X17: Entrants_NonHome
r(all)= -0.133 p= 0.491 r(NonImp)= -0.133 Npairs=29 NimputedPairs=0
Key: Oxbridge X&Y valid
MRCGP_AKT
Entrants_NonHome
OXF CAM BRIST NOTTM CARDF BIRM UCL BRIGH IMP LEEDS SHEF LOND NEWC SOTON SGEO LEICR MANU KEELE KINGS LVRPL HLYK PNSLA QMARY EANG EDINB QUEEN GLASG DUND ABRDN

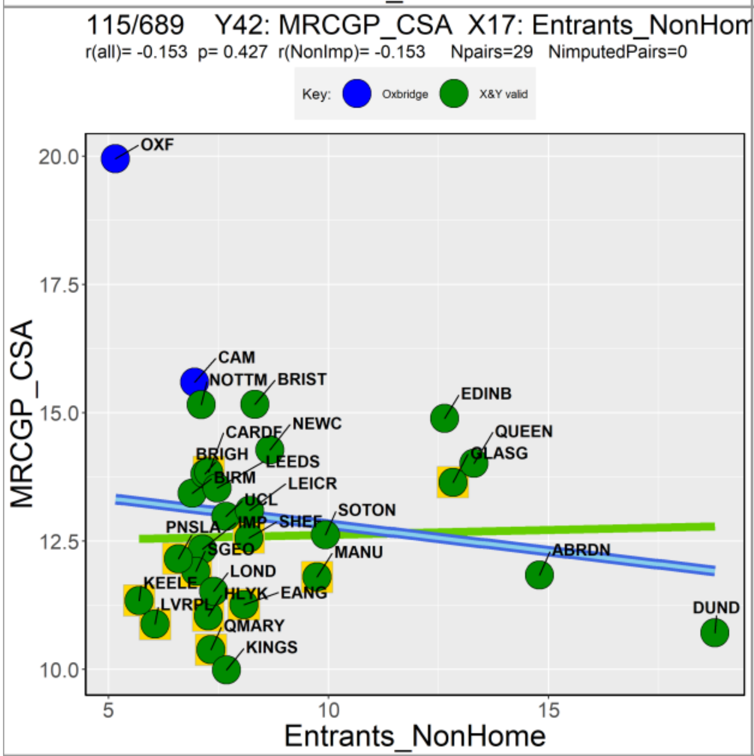
115/689 Y42: MRCGP_CSA X17: Entrants_NonHom
r(all)= -0.153 p= 0.427 r(NonImp)= -0.153 Npairs=29 NimputedPairs=0
Key: Oxbridge X&Y valid
MRCGP_CSA
Entrants_NonHome
OXF CAM NOTTM BRIST EDINB CARDF NEWC QUEEN BRIGH LEEDS GLASG BIRM LEICR UCL SOTON PNSLA IMP SHEF SGEO MANU LOND ABRDN KEELE HLYK EANG LVRPL QMARY KINGS DUND

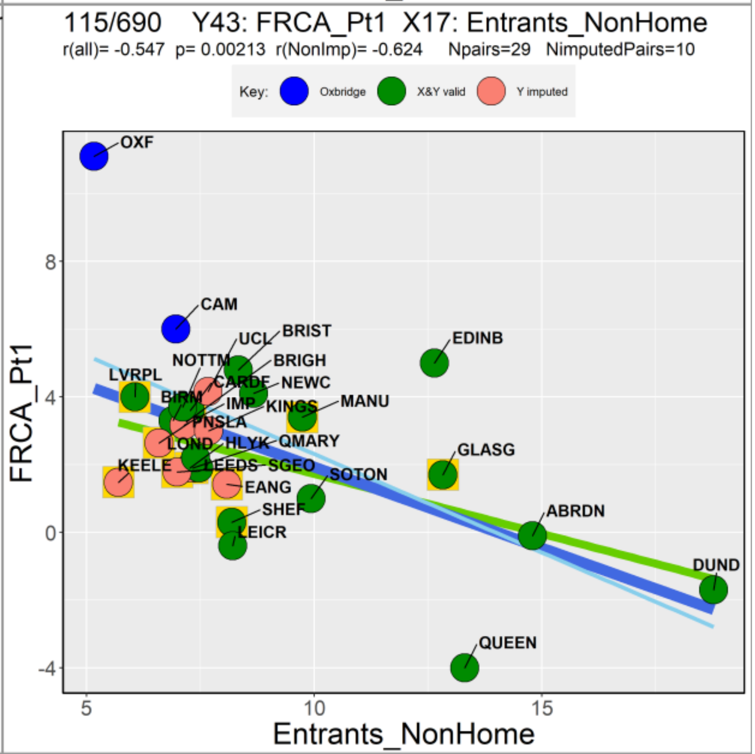
115/690 Y43: FRCA_Pt1 X17: Entrants_NonHome
r(all)= -0.547 p= 0.00213 r(NonImp)= -0.624 Npairs=29 NimputedPairs=10
Key: Oxbridge X&Y valid Y imputed
FRCA_Pt1
Entrants_NonHome
OXF CAM UCL BRIST EDINB NOTTM BRIGH LVRPL CARDF NEWC BIRM IMP KINGS MANU PNSLA LOND HLYK QMARY GLASG KEELE LEEDS SGEO SOTON EANG SHEF LEICR ABRDN DUND QUEEN

116/691 Y44: MRCOG_Pt1 X17: Entrants_NonHome
r(all)= -0.321 p= 0.0895 r(NonImp)= -0.399 Npairs=29 NimputedPairs=10

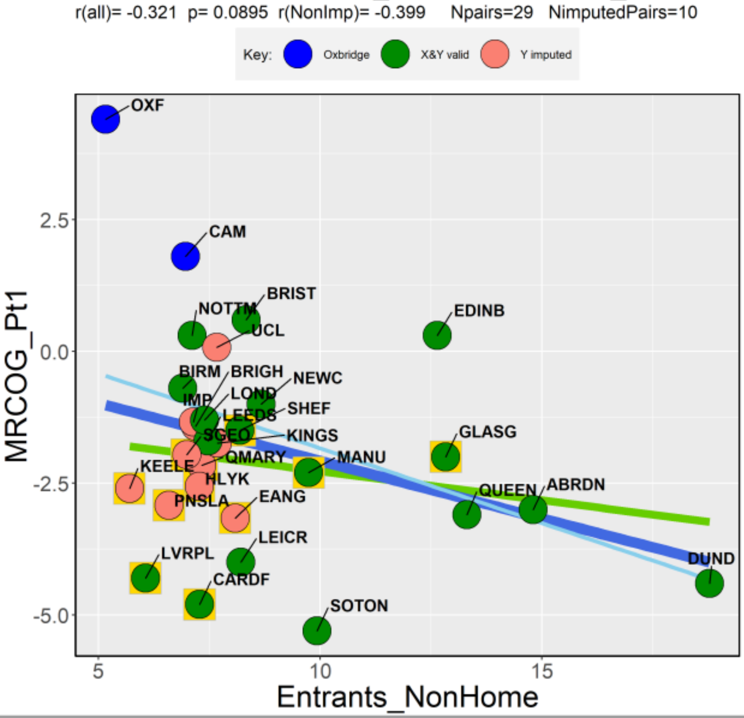

116/692 Y45: MRCOG_Pt2 X17: Entrants_NonHome
r(all)= -0.142 p= 0.462 r(NonImp)= -0.230 Npairs=29 NimputedPairs=10

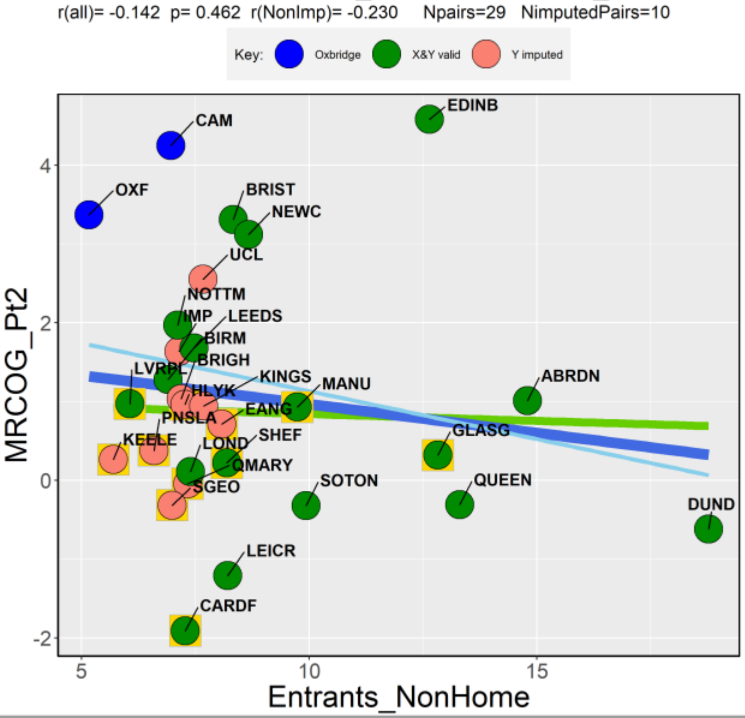

116/693 Y46: MRCP_Pt1 X17: Entrants_NonHome
r(all)= -0.395 p= 0.0339 r(NonImp)= -0.421 Npairs=29 NimputedPairs=3

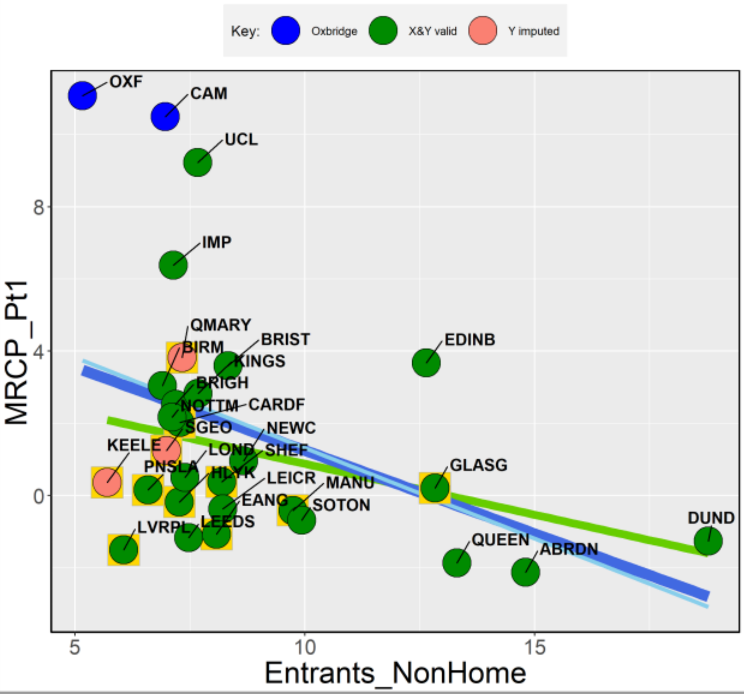

116/694 Y47: MRCP_Pt2 X17: Entrants_NonHome
r(all)= -0.332 p= 0.0787 r(NonImp)= -0.363 Npairs=29 NimputedPairs=3

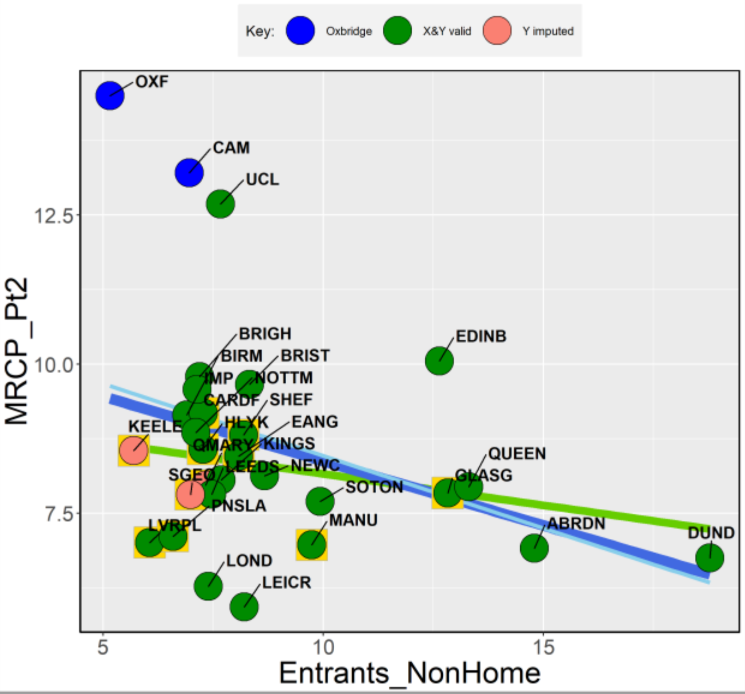

116/695 Y48: MRCP_PACES X17: Entrants_NonHome
r(all)= -0.414 p= 0.0256 r(NonImp)= -0.474 Npairs=29 NimputedPairs=4

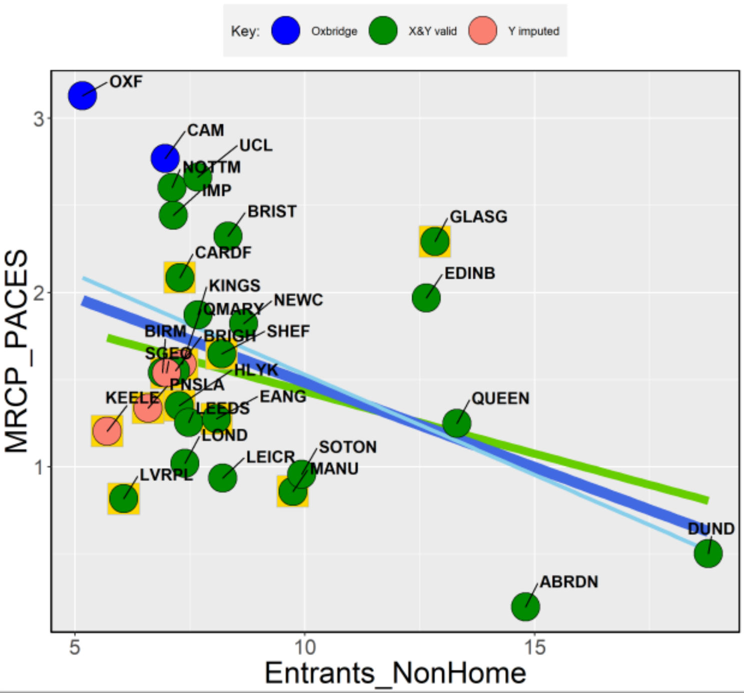

116/696 Y49: GMC_Sanctions X17: Entrants_NonH

r(all)= 0.114 p= 0.555 r(NonImp)= 0.213 Npairs=29 NimputedPairs=10

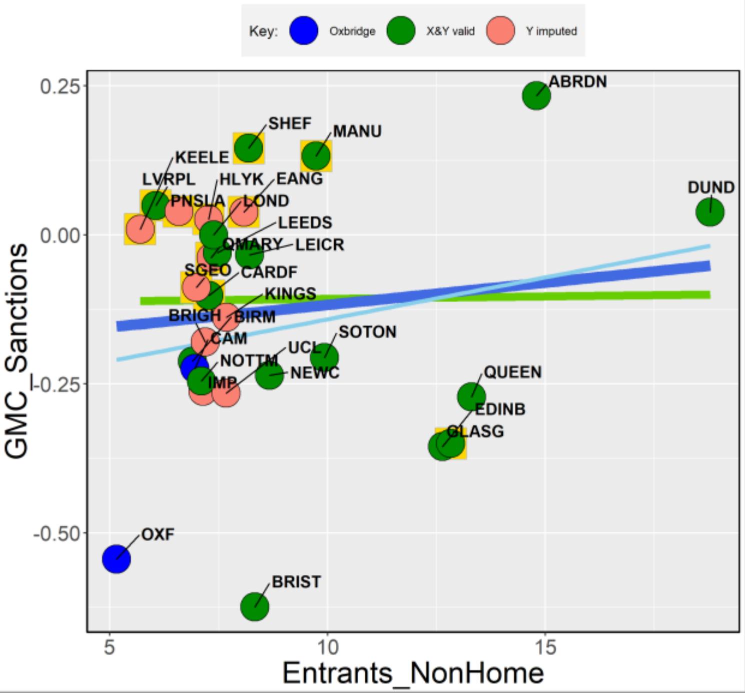

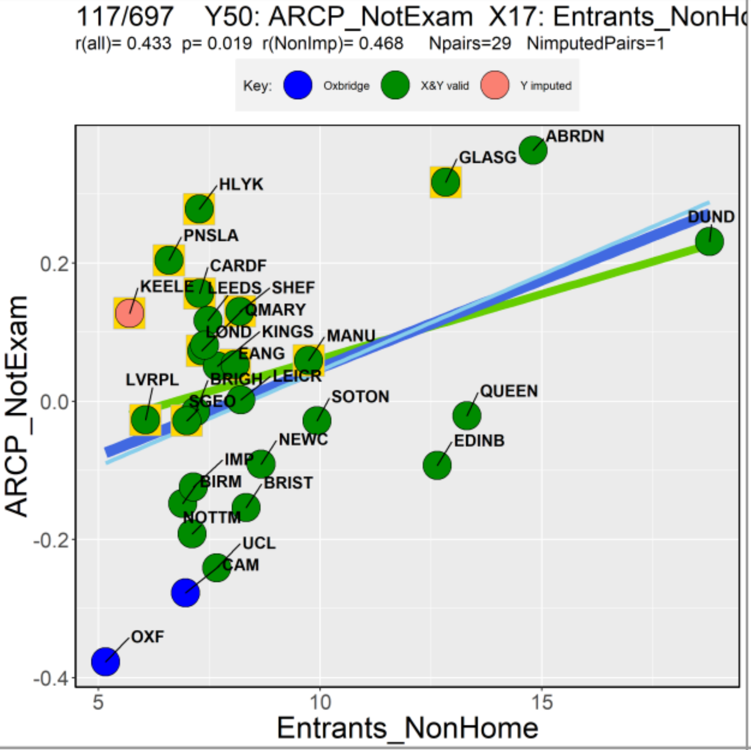
117/697 Y50: ARCP_NotExam X17: Entrants_NonHo
r(all)= 0.433 p= 0.019 r(NonImp)= 0.468 Npairs=29 NimputedPairs=1
Key: Oxbridge X&Y valid Y imputed
ARCP_NotExam
Entrants_NonHome
ABRDN
GLASG
HLYK
DUND
PNSLA
CARDF
KEELE
LEEDS
SHEF
QMARY
LOND
KINGS
MANU
EANG
LVRPL
BRIGH
LEICR
SGEO
SOTON
QUEEN
NEWC
EDINB
IMP
BIRM
BRIST
NOTTM
UCL
CAM
OXF

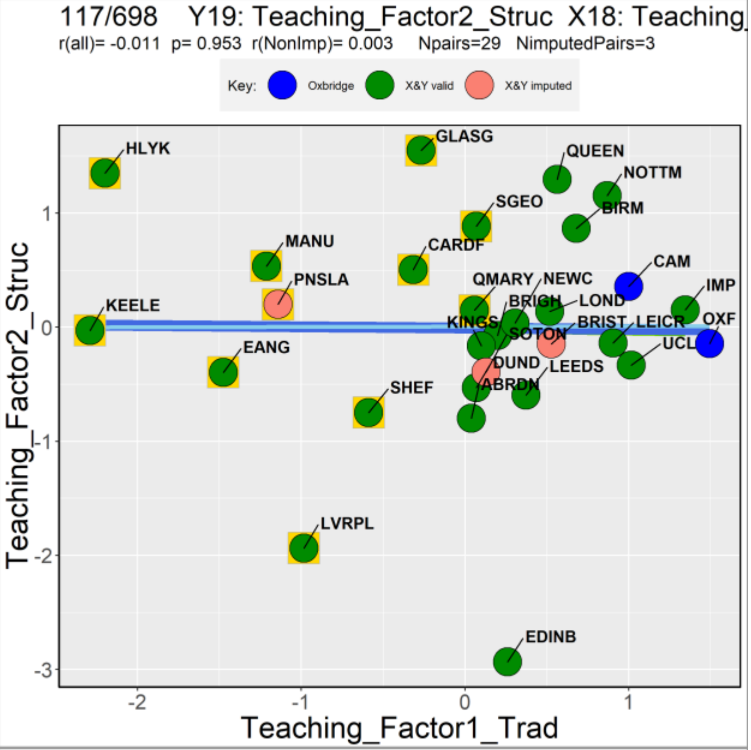
117/698 Y19: Teaching_Factor2_Struc X18: Teaching
r(all)= -0.011 p= 0.953 r(NonImp)= 0.003 Npairs=29 NimputedPairs=3
Key: Oxbridge X&Y valid X&Y imputed
Teaching_Factor2_Struc
Teaching_Factor1_Trad
HLYK
GLASG
QUEEN
NOTTM
SGEO
BIRM
MANU
CARDF
CAM
PNSLA
QMARY
NEWC
IMP
KEELE
BRIGH
LOND
KINGS
BRIST
LEICR
OXF
SOTON
UCL
EANG
DUND
LEEDS
SHEF
ABRDN
LVRPL
EDINB

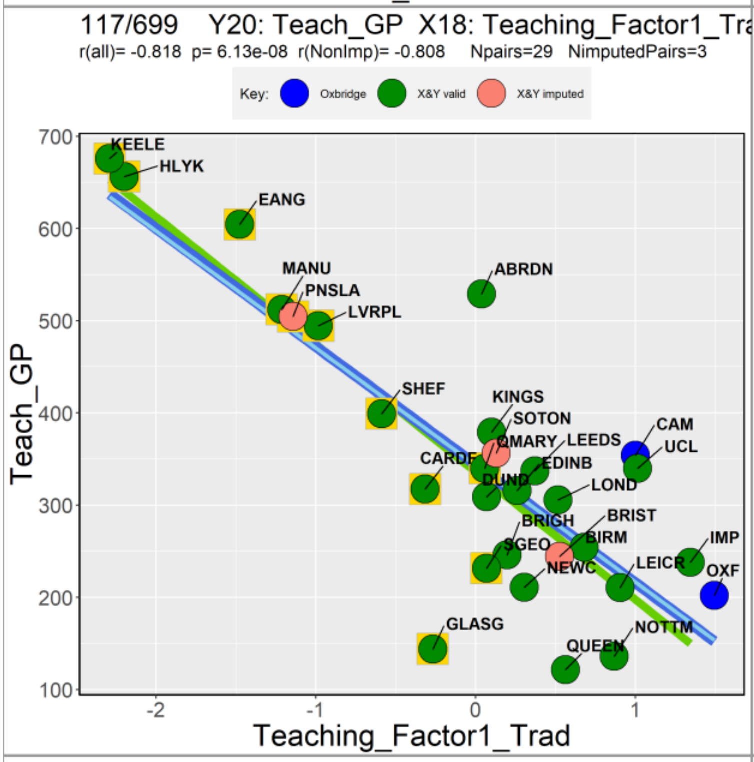
117/699 Y20: Teach_GP X18: Teaching_Factor1_Tra
r(all)= -0.818 p= 6.13e-08 r(NonImp)= -0.808 Npairs=29 NimputedPairs=3
Key: Oxbridge X&Y valid X&Y imputed
Teach_GP
Teaching_Factor1_Trad
KEELE
HLYK
EANG
MANU
ABRDN
PNSLA
LVRPL
SHEF
KINGS
SOTON
CAM
QMARY
LEEDS
UCL
CARDF
EDINB
DUND
LOND
BRIGH
BRIST
SGEO
BIRM
IMP
NEWC
LEICR
OXF
GLASG
NOTTM
QUEEN

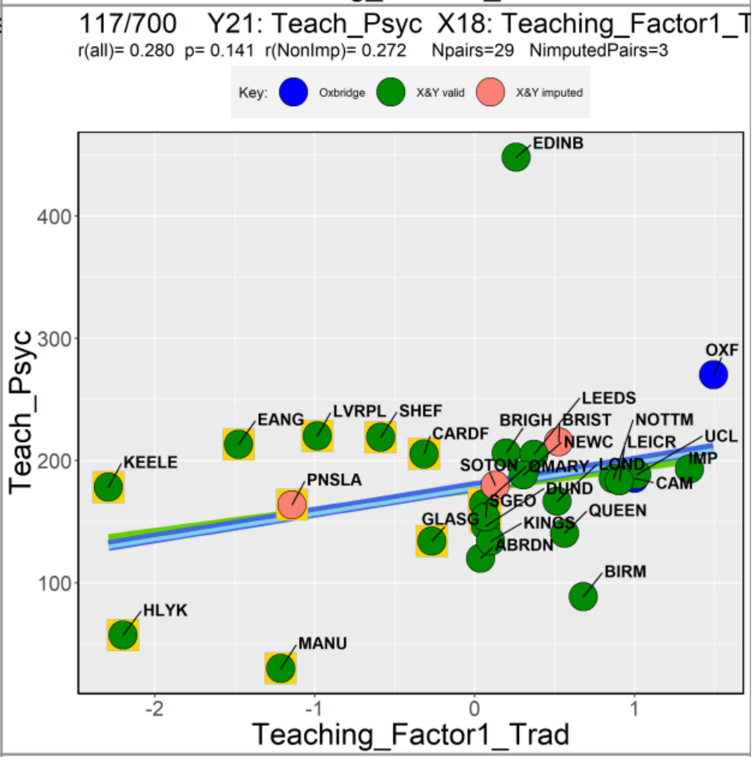
117/700 Y21: Teach_Psyc X18: Teaching_Factor1_T
r(all)= 0.280 p= 0.141 r(NonImp)= 0.272 Npairs=29 NimputedPairs=3
Key: Oxbridge X&Y valid X&Y imputed
Teach_Psyc
Teaching_Factor1_Trad
EDINB
OXF
LEEDS
EANG
LVRPL
SHEF
BRIGH
BRIST
NOTTM
CARDF
NEWC
LEICR
UCL
KEELE
SOTON
QMARY
LOND
IMP
PNSLA
CAM
DUND
SGEO
GLASG
KINGS
QUEEN
ABRDN
BIRM
HLYK
MANU

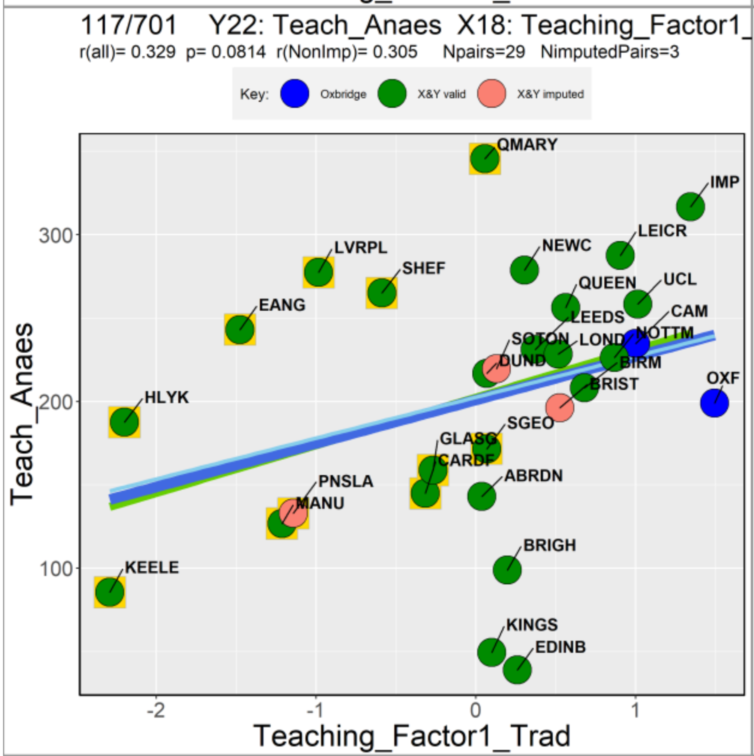
117/701 Y22: Teach_Anaes X18: Teaching_Factor1_
r(all)= 0.329 p= 0.0814 r(NonImp)= 0.305 Npairs=29 NimputedPairs=3
Key: Oxbridge X&Y valid X&Y imputed
Teach_Anaes
Teaching_Factor1_Trad
QMARY
IMP
LEICR
LVRPL
NEWC
SHEF
QUEEN
UCL
EANG
LEEDS
CAM
SOTON
LOND
NOTTM
DUND
BIRM
BRIST
OXF
HLYK
SGEO
GLASG
CARDF
PNSLA
ABRDN
MANU
BRIGH
KEELE
KINGS
EDINB

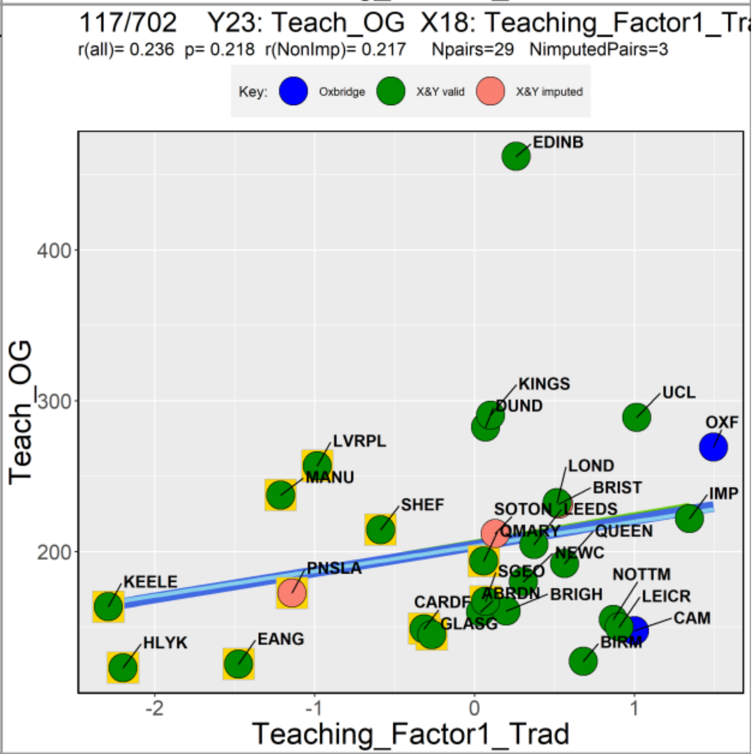
117/702 Y23: Teach_OG X18: Teaching_Factor1_Tra
r(all)= 0.236 p= 0.218 r(NonImp)= 0.217 Npairs=29 NimputedPairs=3
Key: Oxbridge X&Y valid X&Y imputed
Teach_OG
Teaching_Factor1_Trad
EDINB
KINGS
UCL
DUND
OXF
LVRPL
MANU
LOND
BRIST
SHEF
SOTON
LEEDS
IMP
QMARY
QUEEN
NEWC
SGEO
PNSLA
KEELE
NOTTM
ABRDN
BRIGH
LEICR
CARDF
CAM
GLASG
HLYK
EANG
BIRM

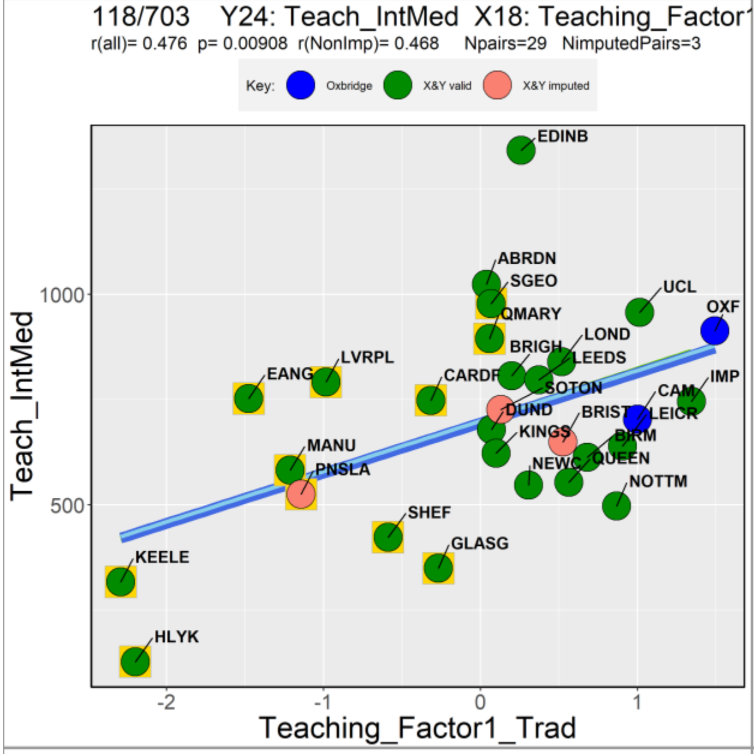
118/703 Y24: Teach_IntMed X18: Teaching_Factor1
r(all)= 0.476 p= 0.00908 r(NonImp)= 0.468 Npairs=29 NimputedPairs=3
Key: Oxbridge X&Y valid X&Y imputed
Teach_IntMed
Teaching_Factor1_Trad
1000
500
-2
-1
0
1
EDINB
ABRDN
SGEO
QMARY
UCL
OXF
LOND
BRIGH
LEEDS
EANG
LVRPL
CARDF
SOTON
IMP
CAM
DUND
BRIST
LEICR
KINGS
BIRM
MANU
NEWC
QUEEN
PNSLA
NOTTM
SHEF
GLASG
KEELE
HLYK

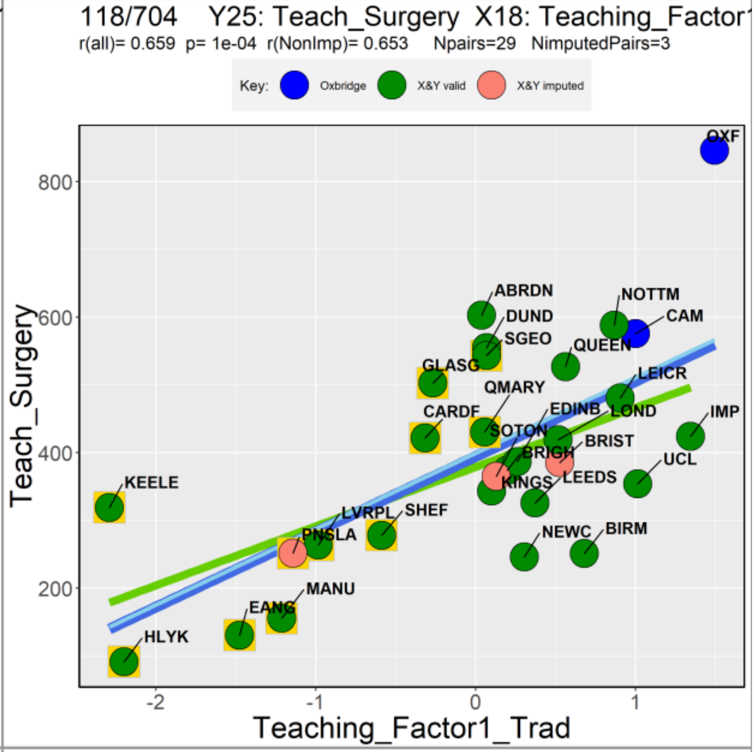
118/704 Y25: Teach_Surgery X18: Teaching_Factor1
r(all)= 0.659 p= 1e-04 r(NonImp)= 0.653 Npairs=29 NimputedPairs=3
Key: Oxbridge X&Y valid X&Y imputed
Teach_Surgery
Teaching_Factor1_Trad
800
600
400
200
-2
-1
0
1
OXF
ABRDN
NOTTM
DUND
CAM
SGEO
QUEEN
GLASG
LEICR
QMARY
CARDF
EDINB
LOND
IMP
SOTON
BRIST
BRIGH
UCL
KINGS
LEEDS
KEELE
LVRPL
SHEF
PNSLA
NEWC
BIRM
MANU
EANG
HLYK

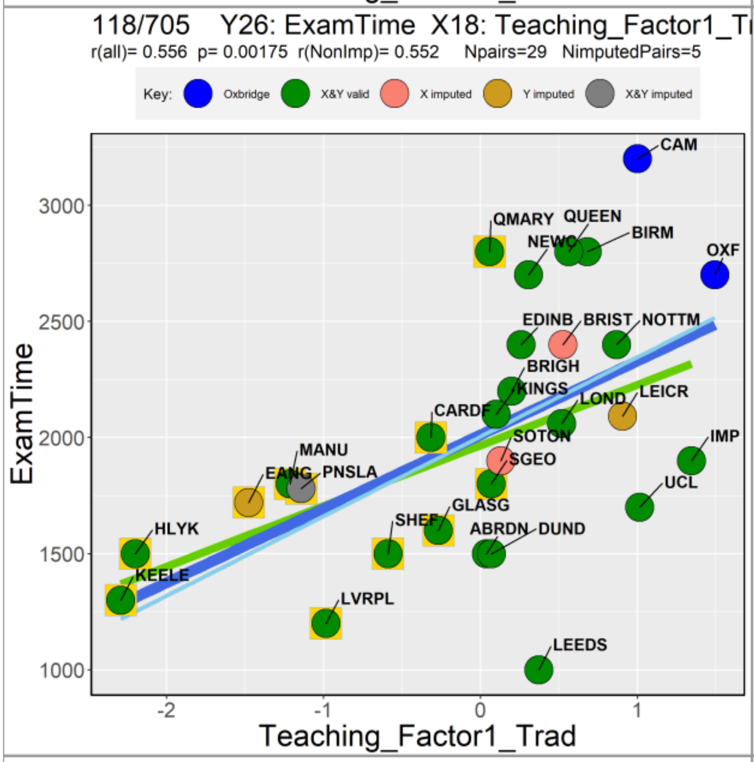
118/705 Y26: ExamTime X18: Teaching_Factor1_Tr
r(all)= 0.556 p= 0.00175 r(NonImp)= 0.552 Npairs=29 NimputedPairs=5
Key: Oxbridge X&Y valid X imputed Y imputed X&Y imputed
ExamTime
Teaching_Factor1_Trad
3000
2500
2000
1500
1000
-2
-1
0
1
CAM
QMARY
QUEEN
NEWC
BIRM
OXF
EDINB
BRIST
NOTTM
BRIGH
KINGS
CARDF
LOND
LEICR
SOTON
IMP
MANU
SGEO
EANG
PNSLA
UCL
GLASG
SHEF
HLYK
ABRDN
DUND
KEELE
LVRPL
LEEDS

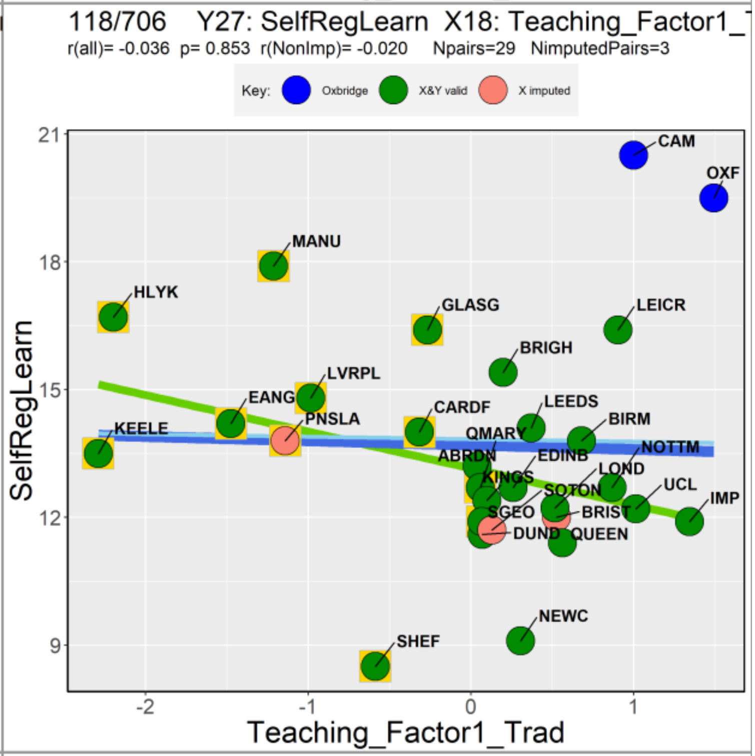
118/706 Y27: SelfRegLearn X18: Teaching_Factor1_
r(all)= -0.036 p= 0.853 r(NonImp)= -0.020 Npairs=29 NimputedPairs=3
Key: Oxbridge X&Y valid X imputed
SelfRegLearn
Teaching_Factor1_Trad
21
18
15
12
9
-2
-1
0
1
CAM
OXF
MANU
HLYK
GLASG
LEICR
BRIGH
LVRPL
EANG
CARDF
LEEDS
PNSLA
KEELE
BIRM
QMARY
NOTTM
ABRDN
EDINB
LOND
KINGS
UCL
SOTON
IMP
SGEO
BRIST
DUND
QUEEN
NEWC
SHEF

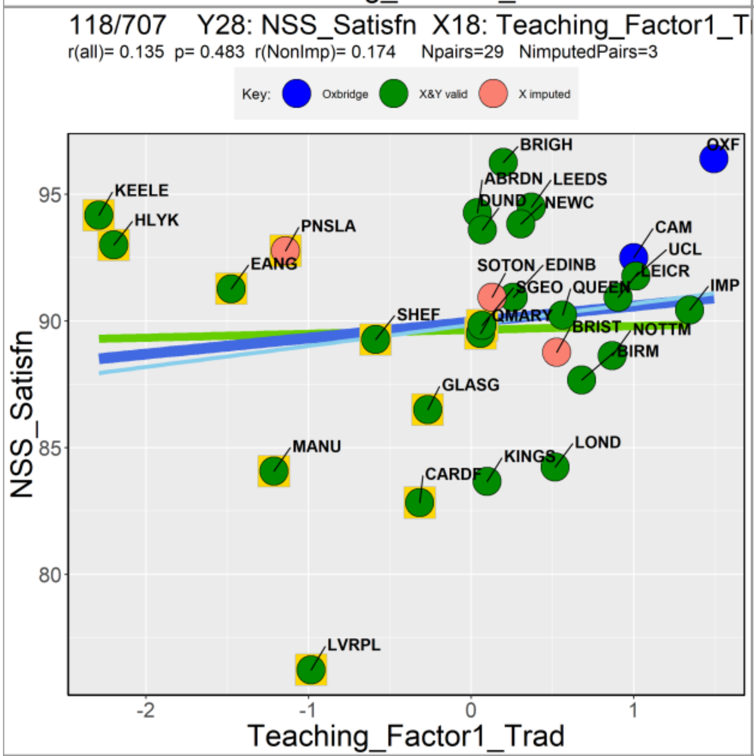
118/707 Y28: NSS_Satisfn X18: Teaching_Factor1_Tr
r(all)= 0.135 p= 0.483 r(NonImp)= 0.174 Npairs=29 NimputedPairs=3
Key: Oxbridge X&Y valid X imputed
NSS_Satisfn
Teaching_Factor1_Trad
95
90
85
80
-2
-1
0
1
BRIGH
OXF
KEELE
ABRDN
LEEDS
DUND
NEWC
HLYK
PNSLA
CAM
UCL
EANG
SOTON
EDINB
LEICR
SGEO
QUEEN
IMP
SHEF
QMARY
BRIST
NOTTM
BIRM
GLASG
MANU
KINGS
LOND
CARDF
LVRPL

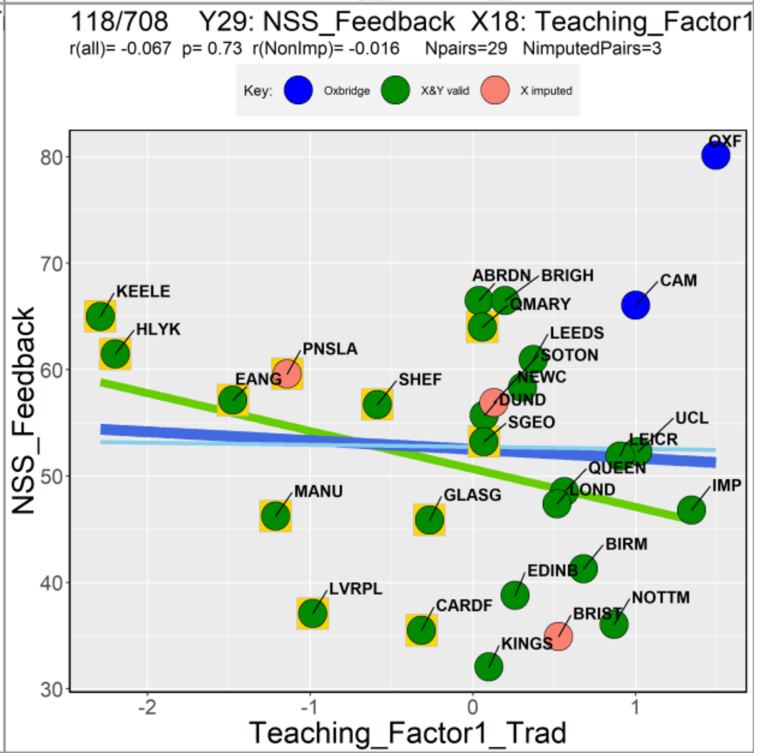
118/708 Y29: NSS_Feedback X18: Teaching_Factor1
r(all)= -0.067 p= 0.73 r(NonImp)= -0.016 Npairs=29 NimputedPairs=3
Key: Oxbridge X&Y valid X imputed
NSS_Feedback
Teaching_Factor1_Trad
80
70
60
50
40
30
-2
-1
0
1
OXF
ABRDN
BRIGH
CAM
KEELE
QMARY
HLYK
LEEDS
PNSLA
SOTON
EANG
SHEF
NEWC
DUND
SGEO
UCL
LEICR
QUEEN
MANU
GLASG
LOND
IMP
BIRM
EDINB
LVRPL
CARDF
BRIST
NOTTM
KINGS

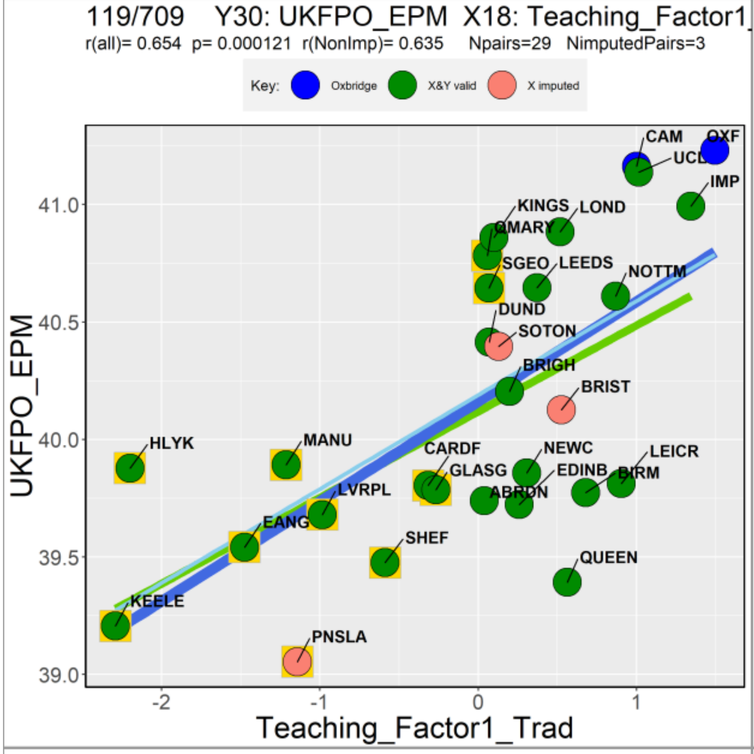
119/709 Y30: UKFPO_EPM X18: Teaching_Factor1_
r(all)= 0.654 p= 0.000121 r(NonImp)= 0.635 Npairs=29 NimputedPairs=3
Key: Oxbridge X&Y valid X imputed
UKFPO_EPM
Teaching_Factor1_Trad
CAM OXF UCL IMP KINGS LOND QMARY SGEO LEEDS NOTTM DUND SOTON BRIGH BRIST HLYK MANU CARDF NEWC LEICR GLASG EDINB BIRM LVRPL ABRDN EANG SHEF QUEEN KEELE PNSLA

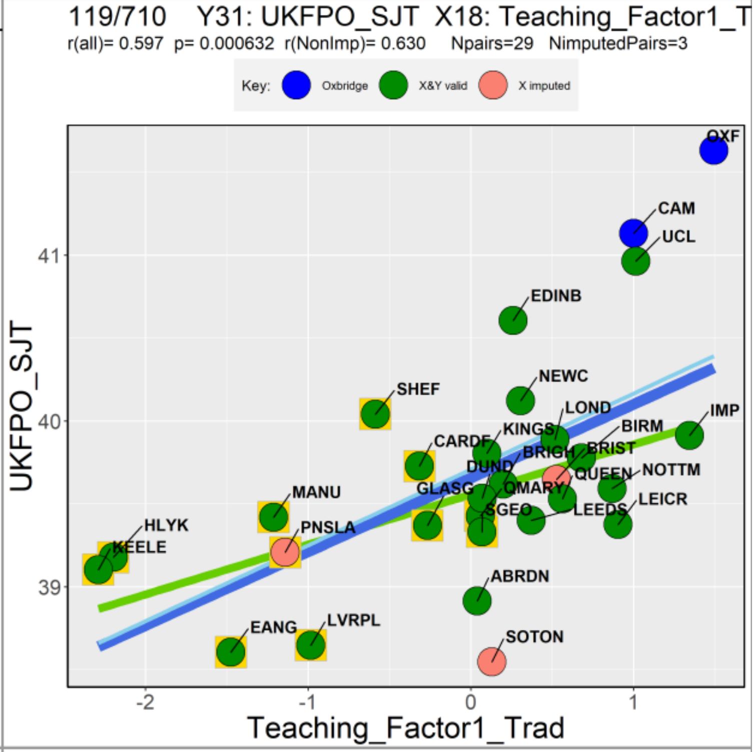
119/710 Y31: UKFPO_SJT X18: Teaching_Factor1_T
r(all)= 0.597 p= 0.000632 r(NonImp)= 0.630 Npairs=29 NimputedPairs=3
Key: Oxbridge X&Y valid X imputed
UKFPO_SJT
Teaching_Factor1_Trad
OXF CAM UCL EDINB NEWC SHEF LOND IMP KINGS BIRM CARDF BRIGH BRIST DUND QUEEN NOTTM GLASG QMARY MANU SGEO LEEDS LEICR HLYK PNSLA KEELE ABRDN EANG LVRPL SOTON

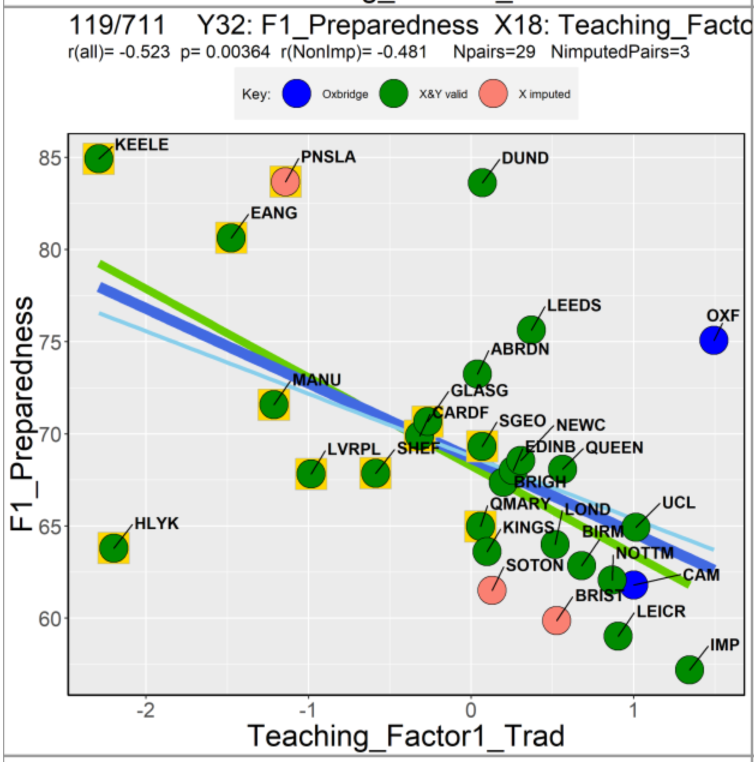
119/711 Y32: F1_Preparedness X18: Teaching_Facto
r(all)= -0.523 p= 0.00364 r(NonImp)= -0.481 Npairs=29 NimputedPairs=3
Key: Oxbridge X&Y valid X imputed
F1_Preparedness
Teaching_Factor1_Trad
KEELE PNSLA DUND EANG LEEDS OXF ABRDN MANU GLASG CARDF SGEO NEWC LVRPL SHEF EDINB QUEEN BRIGH QMARY LOND UCL HLYK KINGS BIRM NOTTM SOTON CAM BRIST LEICR IMP

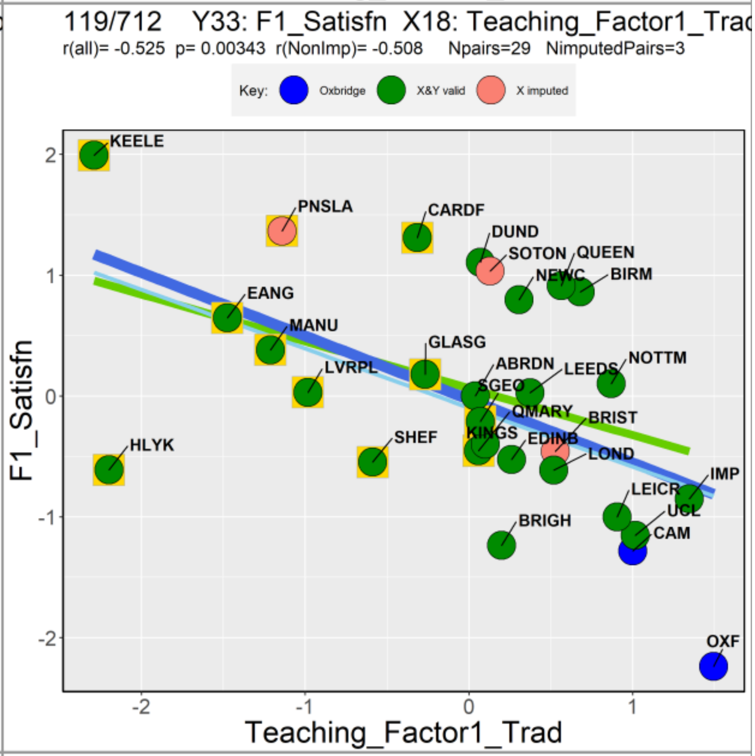
119/712 Y33: F1_Satisfn X18: Teaching_Factor1_Trad
r(all)= -0.525 p= 0.00343 r(NonImp)= -0.508 Npairs=29 NimputedPairs=3
Key: Oxbridge X&Y valid X imputed
F1_Satisfn
Teaching_Factor1_Trad
KEELE PNSLA CARDF DUND SOTON QUEEN NEWC BIRM EANG MANU GLASG LVRPL ABRDN LEEDS NOTTM SGEO QMARY BRIST SHEF KINGS EDINB HLYK LOND IMP LEICR UCL CAM BRIGH OXF

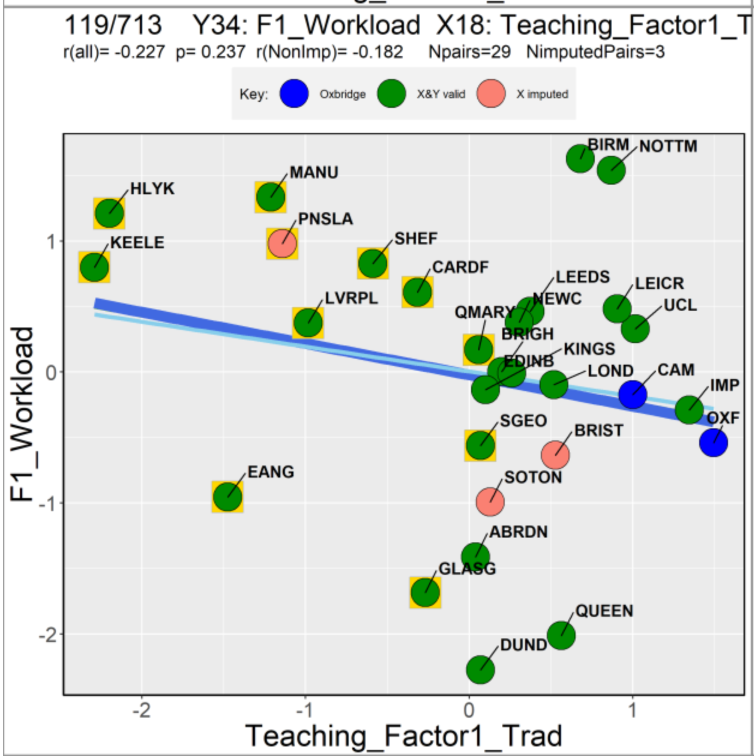
119/713 Y34: F1_Workload X18: Teaching_Factor1_T
r(all)= -0.227 p= 0.237 r(NonImp)= -0.182 Npairs=29 NimputedPairs=3
Key: Oxbridge X&Y valid X imputed
F1_Workload
Teaching_Factor1_Trad
BIRM NOTTM MANU HLYK PNSLA KEELE SHEF CARDF LEEDS LEICR LVRPL QMARY NEWC UCL BRIGH KINGS EDINB LOND CAM IMP OXF SGEO BRIST EANG SOTON ABRDN GLASG QUEEN DUND

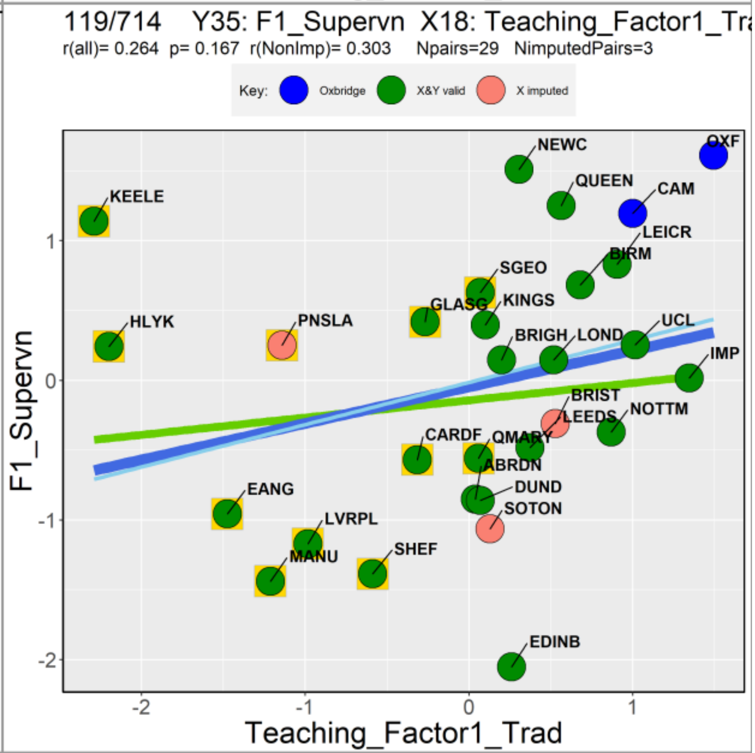
119/714 Y35: F1_Supervn X18: Teaching_Factor1_Tra
r(all)= 0.264 p= 0.167 r(NonImp)= 0.303 Npairs=29 NimputedPairs=3
Key: Oxbridge X&Y valid X imputed
F1_Supervn
Teaching_Factor1_Trad
NEWC OXF KEELE QUEEN CAM LEICR BIRM SGEO GLASG KINGS HLYK PNSLA UCL BRIGH LOND IMP BRIST LEEDS NOTTM CARDF QMARY ABRDN EANG DUND SOTON LVRPL MANU SHEF EDINB

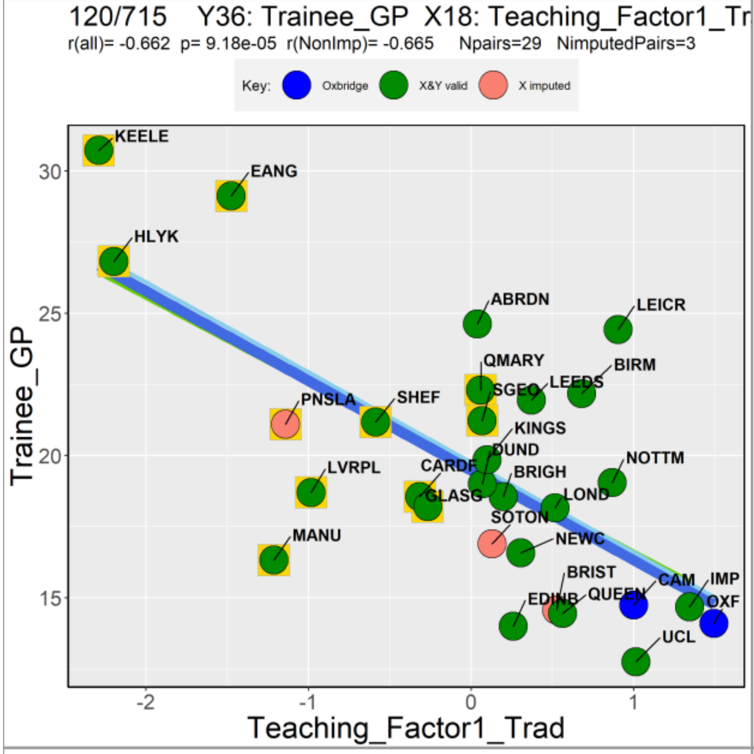
120/715 Y36: Trainee_GP X18: Teaching_Factor1_Tr
r(all)= -0.662 p= 9.18e-05 r(NonImp)= -0.665 Npairs=29 NimputedPairs=3
Key: Oxbridge X&Y valid X imputed
Trainee_GP
Teaching_Factor1_Trad
30
25
20
15
-2
-1
0
1
KEELE
EANG
HLYK
ABRDN
LEICR
QMARY
BIRM
PNSLA
SHEF
SGEO
LEEDS
KINGS
DUND
LVRPL
CARDF
BRIGH
NOTTM
GLASG
LOND
SOTON
MANU
NEWC
BRIST
CAM
IMP
EDINB
QUEEN
OXF
UCL

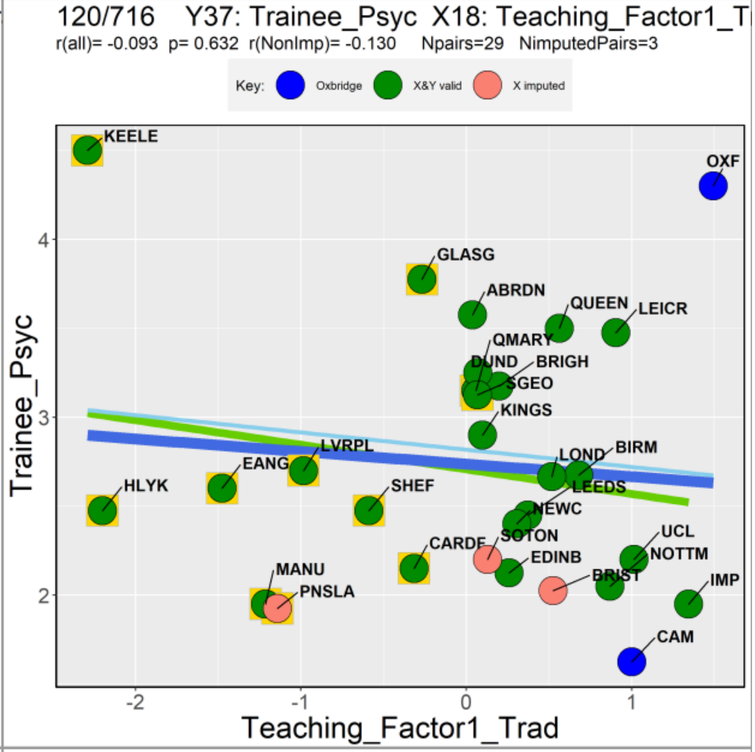
120/716 Y37: Trainee_Psyc X18: Teaching_Factor1_T
r(all)= -0.093 p= 0.632 r(NonImp)= -0.130 Npairs=29 NimputedPairs=3
Key: Oxbridge X&Y valid X imputed
Trainee_Psyc
Teaching_Factor1_Trad
4
3
2
-2
-1
0
1
KEELE
OXF
GLASG
ABRDN
QUEEN
LEICR
QMARY
DUND
BRIGH
SGEO
KINGS
LVRPL
LOND
BIRM
EANG
HLYK
SHEF
LEEDS
NEWC
SOTON
CARDF
UCL
EDINB
NOTTM
MANU
BRIST
PNSLA
IMP
CAM

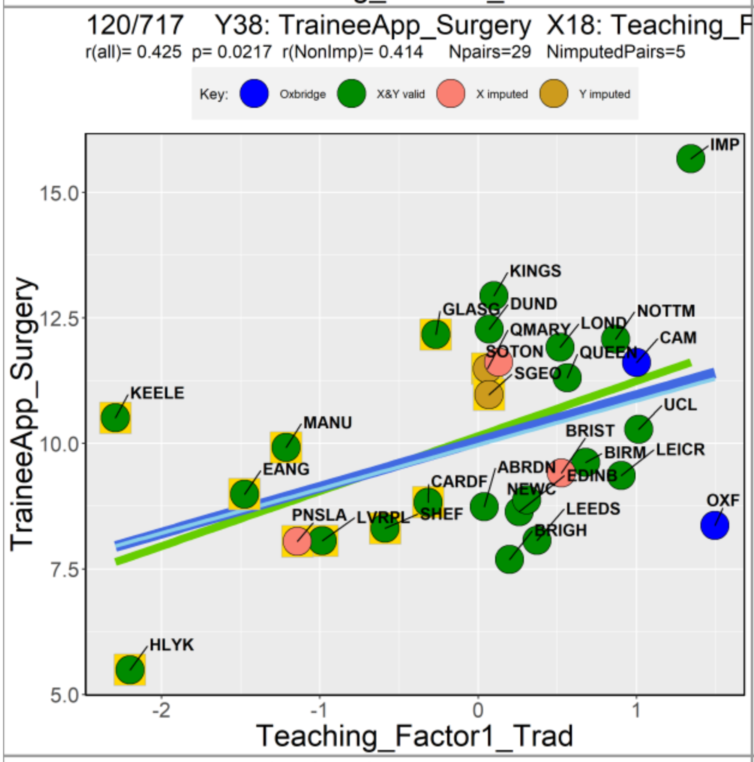
120/717 Y38: TraineeApp_Surgery X18: Teaching_F
r(all)= 0.425 p= 0.0217 r(NonImp)= 0.414 Npairs=29 NimputedPairs=5
Key: Oxbridge X&Y valid X imputed Y imputed
TraineeApp_Surgery
Teaching_Factor1_Trad
15.0
12.5
10.0
7.5
5.0
-2
-1
0
1
IMP
KINGS
GLASG
DUND
NOTTM
QMARY
LOND
CAM
SOTON
QUEEN
SGEO
KEELE
UCL
MANU
BRIST
BIRM
LEICR
ABRDN
EANG
EDINB
CARDF
NEWC
OXF
PNSLA
LVRPL
SHEF
LEEDS
BRIGH
HLYK

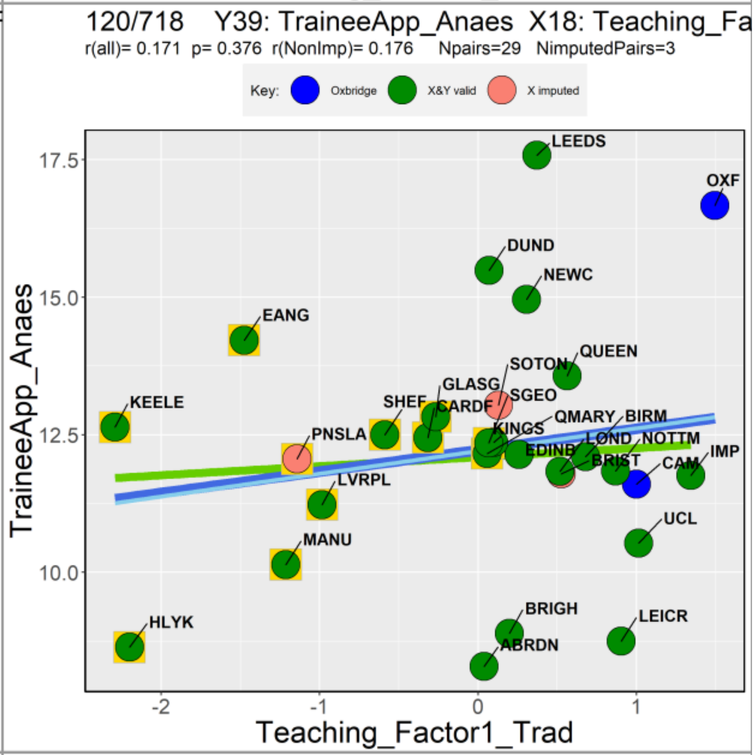
120/718 Y39: TraineeApp_Anaes X18: Teaching_Fa
r(all)= 0.171 p= 0.376 r(NonImp)= 0.176 Npairs=29 NimputedPairs=3
Key: Oxbridge X&Y valid X imputed
TraineeApp_Anaes
Teaching_Factor1_Trad
17.5
15.0
12.5
10.0
-2
-1
0
1
LEEDS
OXF
DUND
NEWC
EANG
SOTON
QUEEN
GLASG
SGEO
KEELE
SHEF
CARDF
QMARY
BIRM
KINGS
PNSLA
LOND
NOTTM
EDINB
BRIST
IMP
CAM
LVRPL
UCL
MANU
HLYK
BRIGH
LEICR
ABRDN

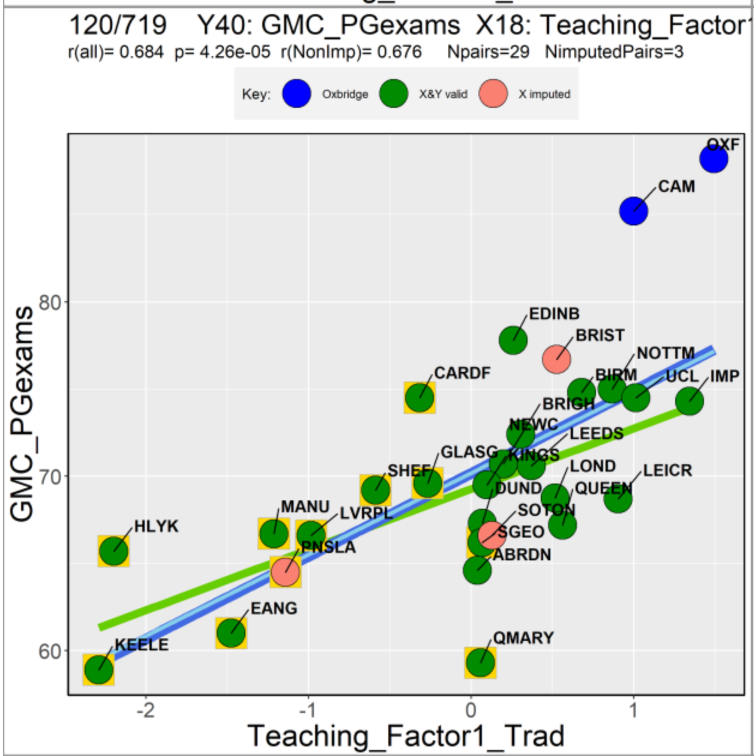
120/719 Y40: GMC_PGexams X18: Teaching_Factor
r(all)= 0.684 p= 4.26e-05 r(NonImp)= 0.676 Npairs=29 NimputedPairs=3
Key: Oxbridge X&Y valid X imputed
GMC_PGexams
Teaching_Factor1_Trad
80
70
60
-2
-1
0
1
OXF
CAM
EDINB
BRIST
NOTTM
CARDF
BIRM
UCL
IMP
BRIGH
NEWC
LEEDS
GLASG
KINGS
SHEF
LOND
LEICR
DUND
QUEEN
MANU
LVRPL
SOTON
HLYK
PNSLA
SGEO
ABRDN
EANG
QMARY
KEELE

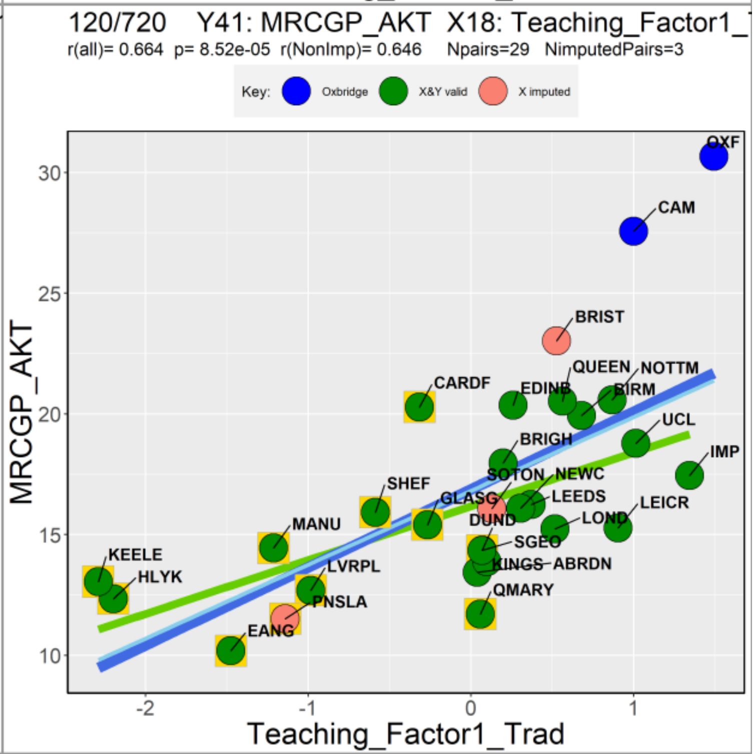
120/720 Y41: MRCGP_AKT X18: Teaching_Factor1_
r(all)= 0.664 p= 8.52e-05 r(NonImp)= 0.646 Npairs=29 NimputedPairs=3
Key: Oxbridge X&Y valid X imputed
MRCGP_AKT
Teaching_Factor1_Trad
30
25
20
15
10
-2
-1
0
1
OXF
CAM
BRIST
QUEEN
NOTTM
CARDF
EDINB
BIRM
UCL
BRIGH
IMP
SOTON
NEWC
SHEF
GLASG
LEEDS
LEICR
MANU
DUND
LOND
SGEO
KEELE
KINGS
ABRDN
HLYK
LVRPL
QMARY
PNSLA
EANG

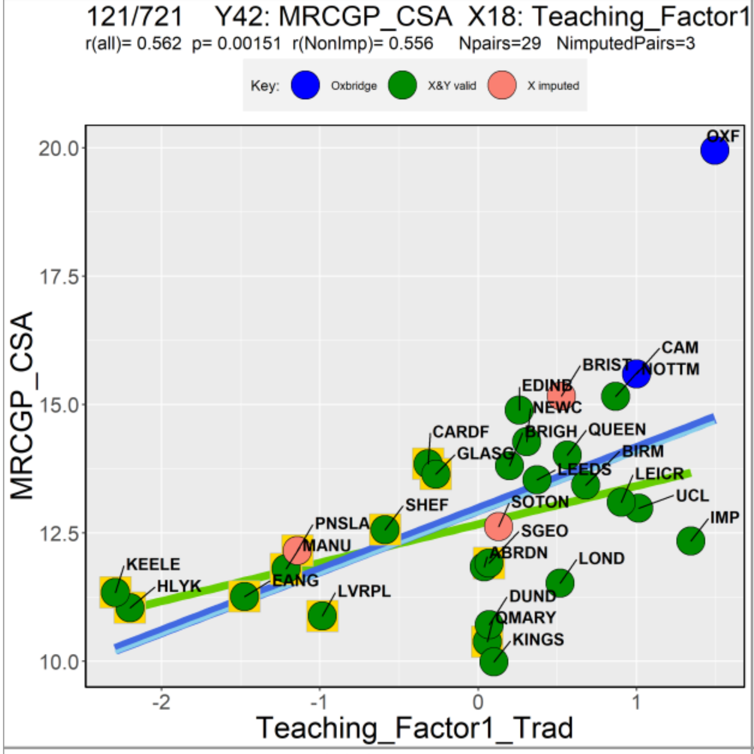
121/721 Y42: MRCGP_CSA X18: Teaching_Factor1
r(all)= 0.562 p= 0.00151 r(NonImp)= 0.556 Npairs=29 NimputedPairs=3
Key: Oxbridge X&Y valid X imputed
MRCGP_CSA
Teaching_Factor1_Trad
OXF
CAM
BRIST
NOTTM
EDINB
NEWC
CARDF
BRIGH
QUEEN
GLASG
BIRM
LEEDS
LEICR
UCL
SHEF
SOTON
IMP
PNSLA
MANU
SGEO
ABRDN
KEELE
LOND
HLYK
EANG
LVRPL
DUND
QMARY
KINGS

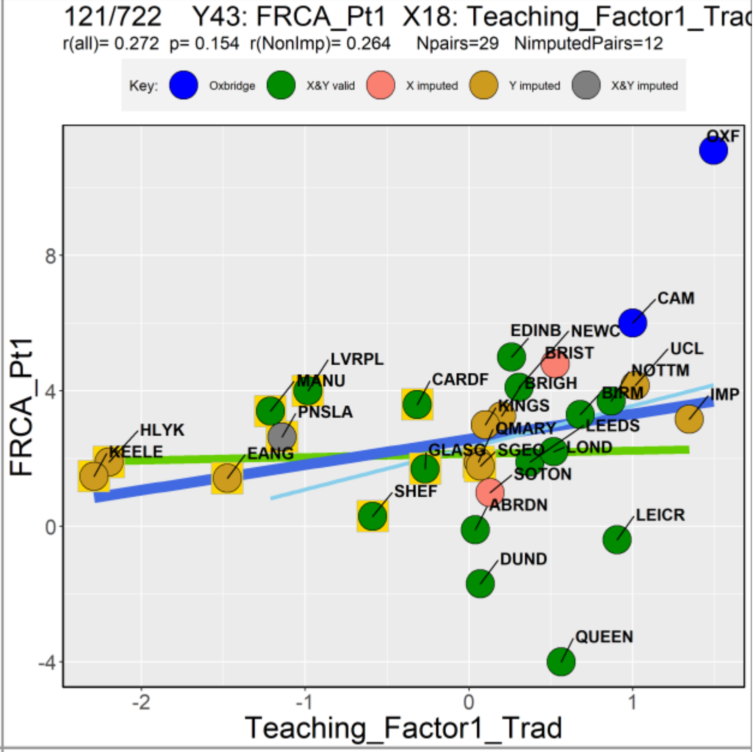
121/722 Y43: FRCA_Pt1 X18: Teaching_Factor1_Trac
r(all)= 0.272 p= 0.154 r(NonImp)= 0.264 Npairs=29 NimputedPairs=12
Key: Oxbridge X&Y valid X imputed Y imputed X&Y imputed
FRCA_Pt1
Teaching_Factor1_Trad
OXF
CAM
EDINB
NEWC
UCL
LVRPL
BRIST
NOTTM
MANU
CARDF
BRIGH
BIRM
IMP
PNSLA
KINGS
LEEDS
HLYK
QMARY
KEELE
EANG
GLASG
SGEO
LOND
SOTON
SHEF
ABRDN
LEICR
DUND
QUEEN

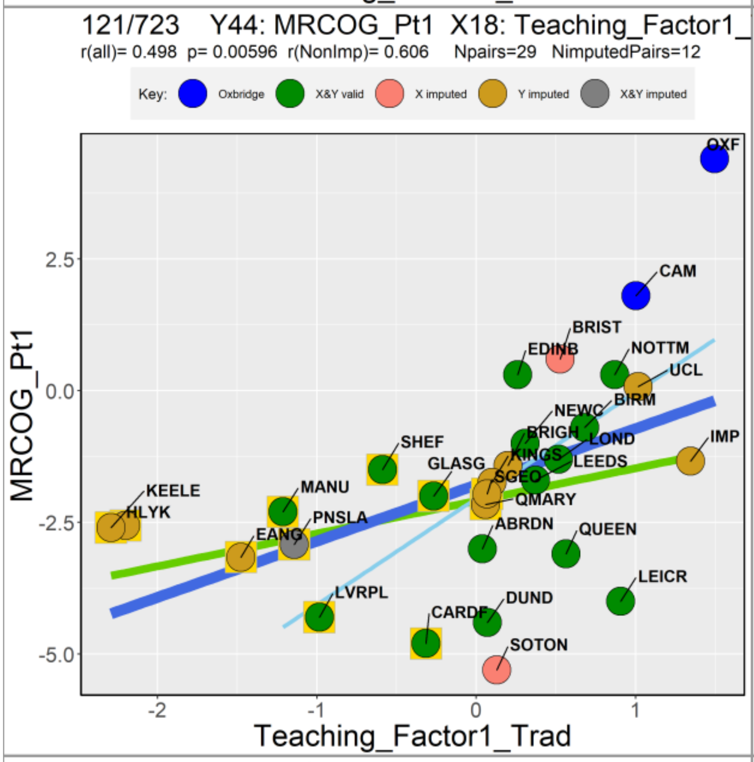
121/723 Y44: MRCOG_Pt1 X18: Teaching_Factor1_
r(all)= 0.498 p= 0.00596 r(NonImp)= 0.606 Npairs=29 NimputedPairs=12
Key: Oxbridge X&Y valid X imputed Y imputed X&Y imputed
MRCOG_Pt1
Teaching_Factor1_Trad
OXF
CAM
BRIST
EDINB
NOTTM
UCL
NEWC
BIRM
BRIGH
LOND
SHEF
KINGS
LEEDS
GLASG
SGEO
IMP
KEELE
MANU
QMARY
HLYK
PNSLA
ABRDN
EANG
QUEEN
LEICR
LVRPL
DUND
CARDF
SOTON

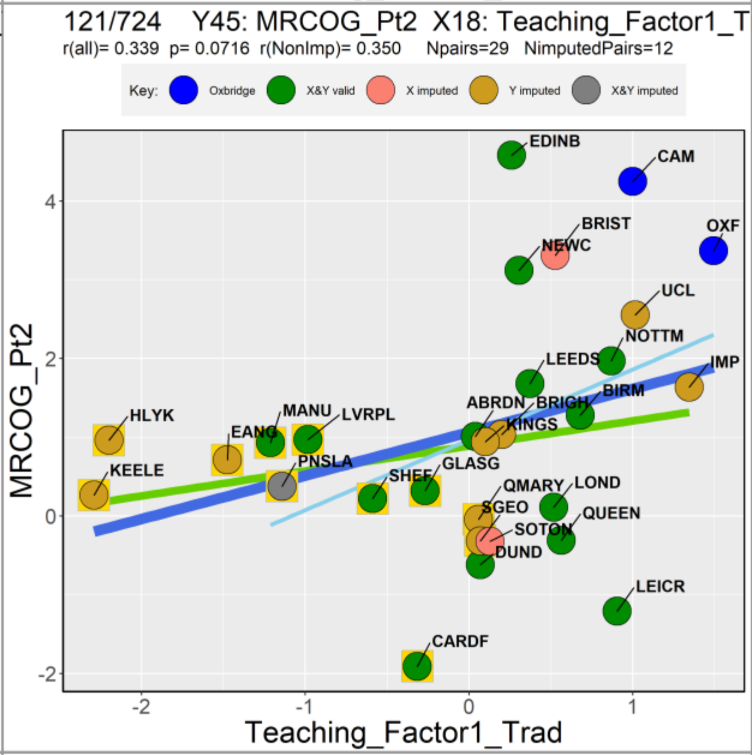
121/724 Y45: MRCOG_Pt2 X18: Teaching_Factor1_T
r(all)= 0.339 p= 0.0716 r(NonImp)= 0.350 Npairs=29 NimputedPairs=12
Key: Oxbridge X&Y valid X imputed Y imputed X&Y imputed
MRCOG_Pt2
Teaching_Factor1_Trad
EDINB
CAM
BRIST
OXF
NEWC
UCL
NOTTM
LEEDS
IMP
HLYK
MANU
LVRPL
ABRDN
BRIGH
BIRM
EANG
KINGS
PNSLA
GLASG
KEELE
SHEF
QMARY
LOND
SGEO
QUEEN
SOTON
DUND
LEICR
CARDF

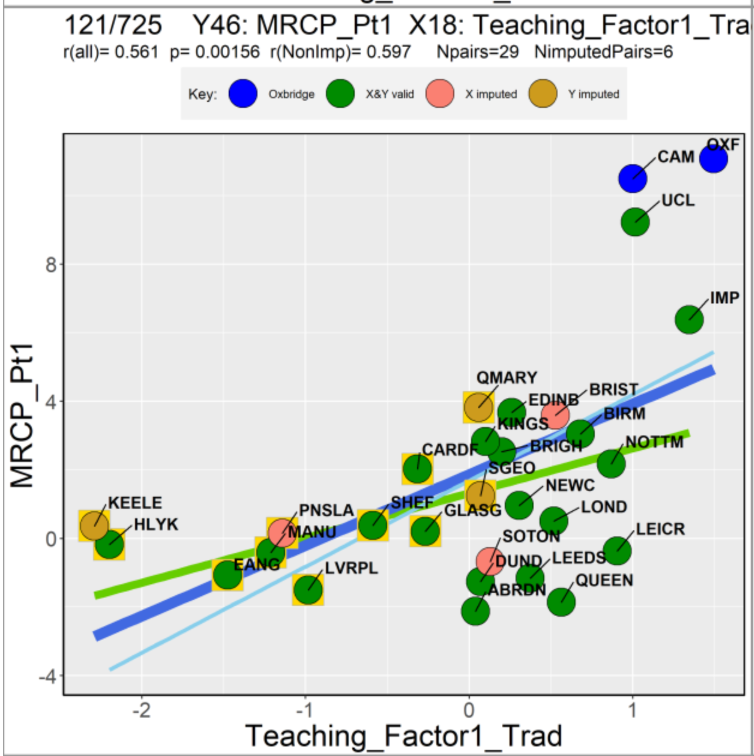
121/725 Y46: MRCP_Pt1 X18: Teaching_Factor1_Tra
r(all)= 0.561 p= 0.00156 r(NonImp)= 0.597 Npairs=29 NimputedPairs=6
Key: Oxbridge X&Y valid X imputed Y imputed
MRCP_Pt1
Teaching_Factor1_Trad
OXF
CAM
UCL
IMP
QMARY
BRIST
EDINB
BIRM
KINGS
CARDF
BRIGH
NOTTM
SGEO
NEWC
KEELE
PNSLA
SHEF
GLASG
LOND
HLYK
MANU
SOTON
LEICR
EANG
DUND
LEEDS
LVRPL
ABRDN
QUEEN

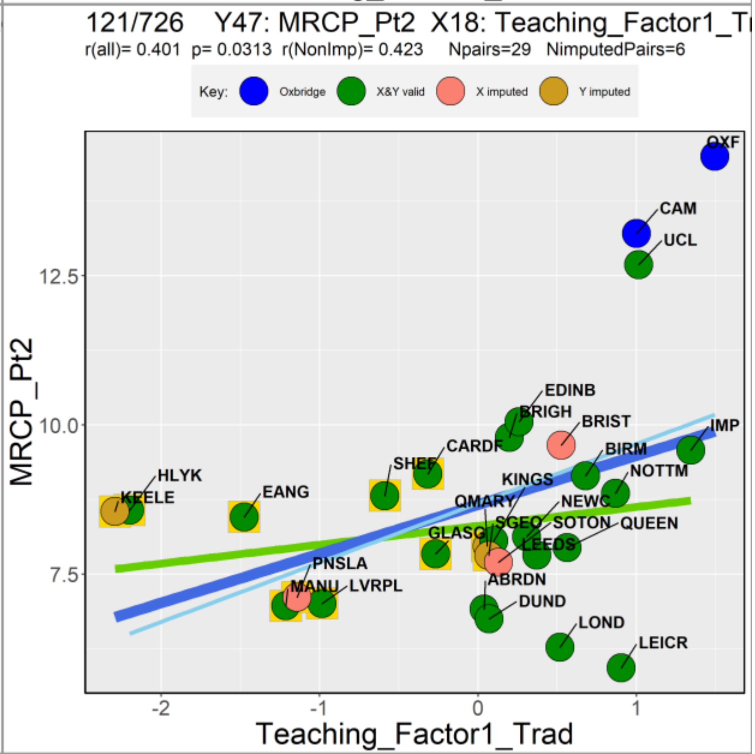
121/726 Y47: MRCP_Pt2 X18: Teaching_Factor1_Ti
r(all)= 0.401 p= 0.0313 r(NonImp)= 0.423 Npairs=29 NimputedPairs=6
Key: Oxbridge X&Y valid X imputed Y imputed
MRCP_Pt2
Teaching_Factor1_Trad
OXF
CAM
UCL
EDINB
BRIGH
BRIST
IMP
CARDF
SHEF
BIRM
HLYK
EANG
KINGS
NOTTM
KEELE
QMARY
NEWC
GLASG
SGEO
SOTON
QUEEN
LEEDS
PNSLA
ABRDN
MANU
LVRPL
DUND
LOND
LEICR

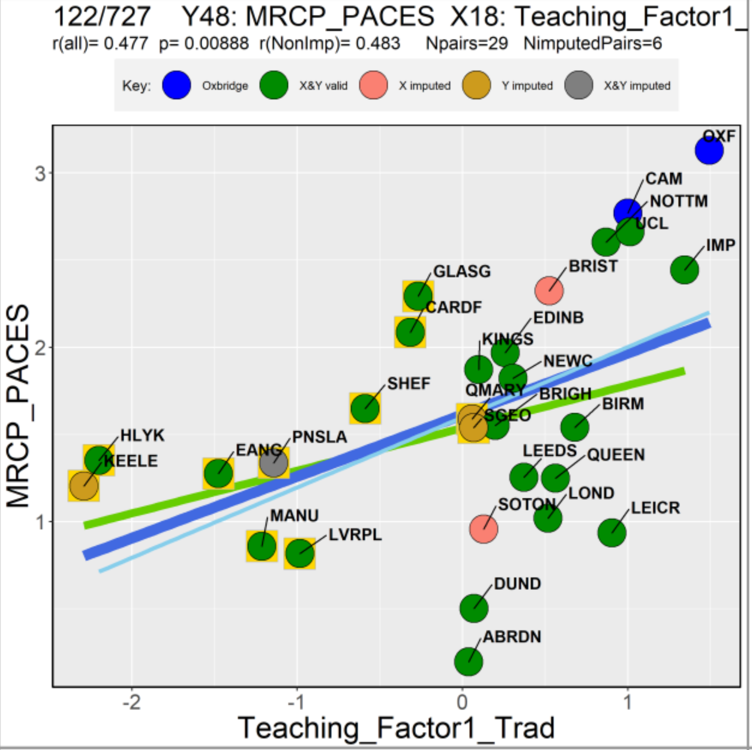
122/727 Y48: MRCP_PACES X18: Teaching_Factor1_
r(all)= 0.477 p= 0.00888 r(NonImp)= 0.483 Npairs=29 NimputedPairs=6
Key: Oxbridge X&Y valid X imputed Y imputed X&Y imputed
MRCP_PACES
Teaching_Factor1_Trad

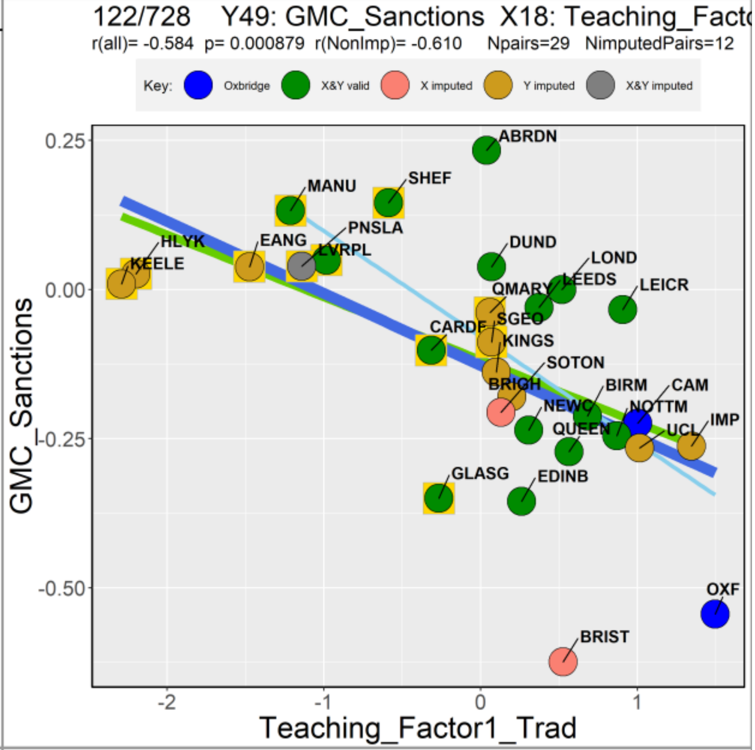
122/728 Y49: GMC_Sanctions X18: Teaching_Facto
r(all)= -0.584 p= 0.000879 r(NonImp)= -0.610 Npairs=29 NimputedPairs=12
Key: Oxbridge X&Y valid X imputed Y imputed X&Y imputed
GMC_Sanctions
Teaching_Factor1_Trad

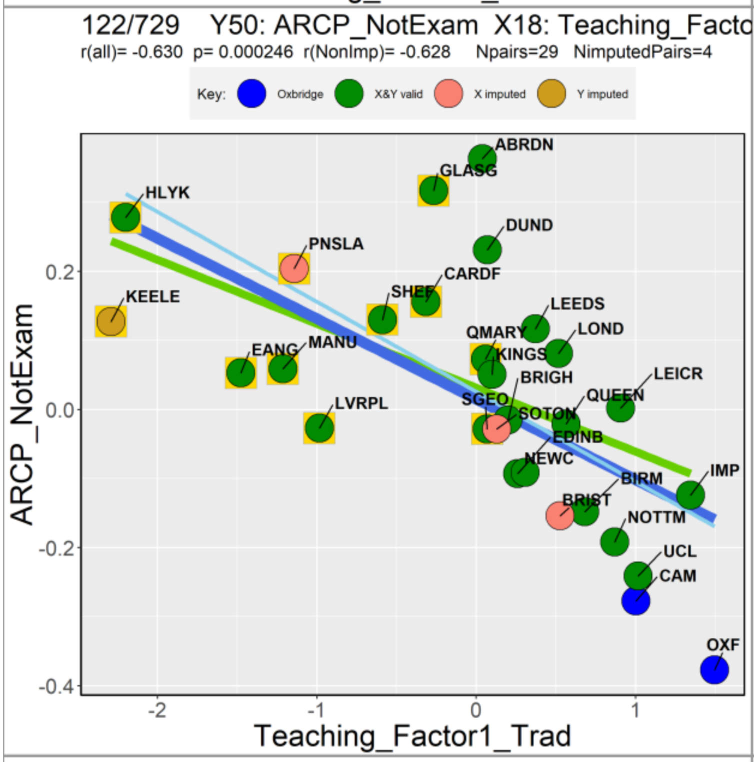
122/729 Y50: ARCP_NotExam X18: Teaching_Facto
r(all)= -0.630 p= 0.000246 r(NonImp)= -0.628 Npairs=29 NimputedPairs=4
Key: Oxbridge X&Y valid X imputed Y imputed
ARCP_NotExam
Teaching_Factor1_Trad

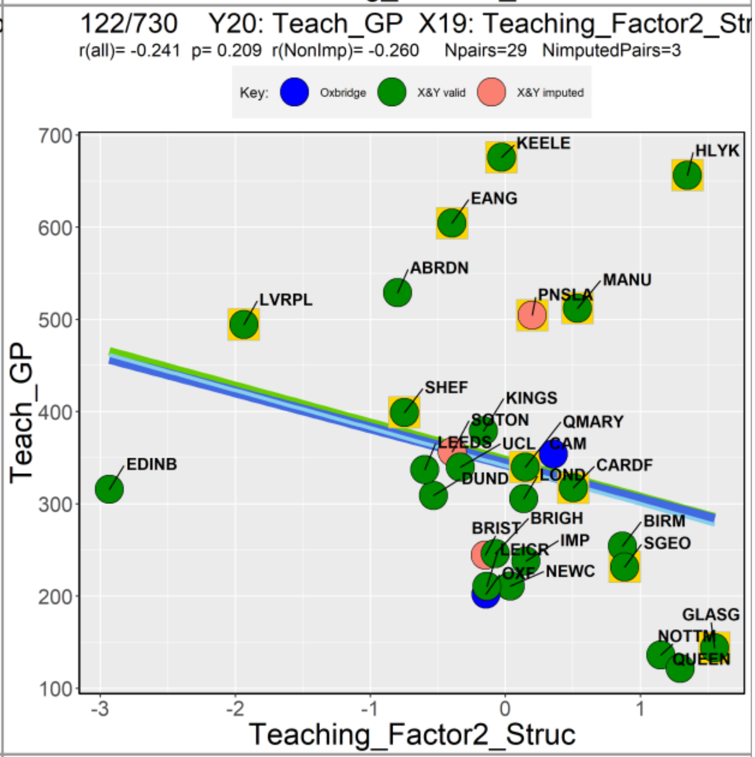
122/730 Y20: Teach_GP X19: Teaching_Factor2_Str
r(all)= -0.241 p= 0.209 r(NonImp)= -0.260 Npairs=29 NimputedPairs=3
Key: Oxbridge X&Y valid X&Y imputed
Teach_GP
Teaching_Factor2_Struc

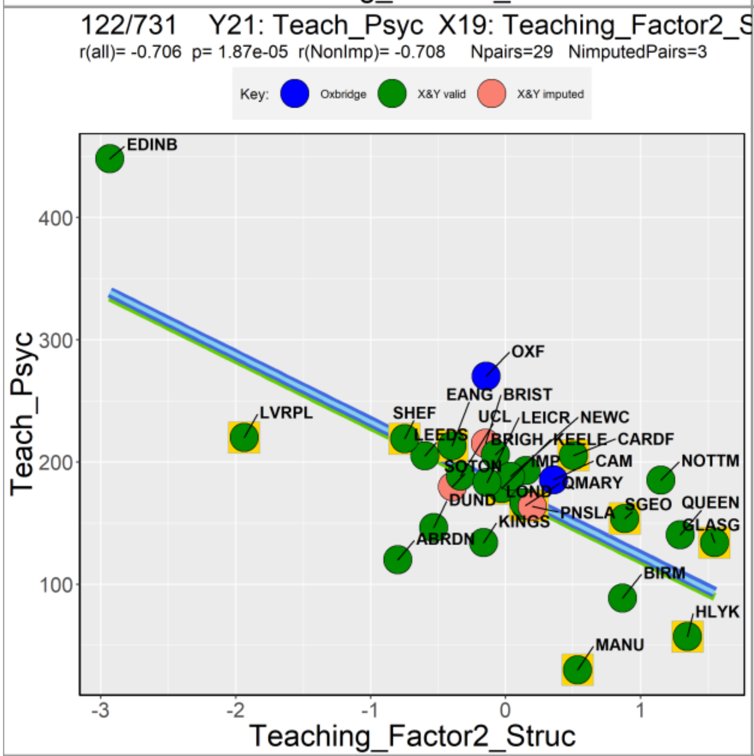
122/731 Y21: Teach_Psyc X19: Teaching_Factor2_S
r(all)= -0.706 p= 1.87e-05 r(NonImp)= -0.708 Npairs=29 NimputedPairs=3
Key: Oxbridge X&Y valid X&Y imputed
Teach_Psyc
Teaching_Factor2_Struc

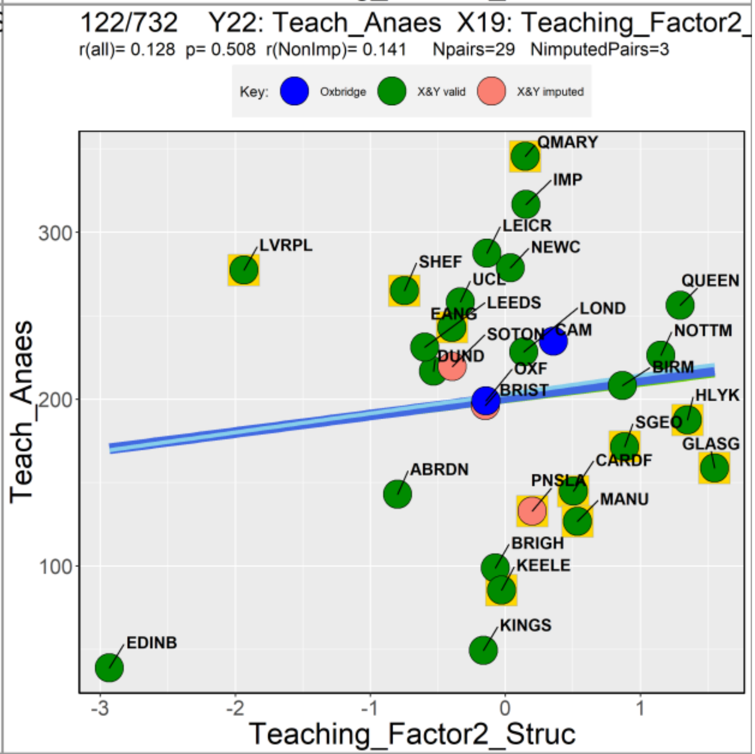
122/732 Y22: Teach_Anaes X19: Teaching_Factor2_
r(all)= 0.128 p= 0.508 r(NonImp)= 0.141 Npairs=29 NimputedPairs=3
Key: Oxbridge X&Y valid X&Y imputed
Teach_Anaes
Teaching_Factor2_Struc

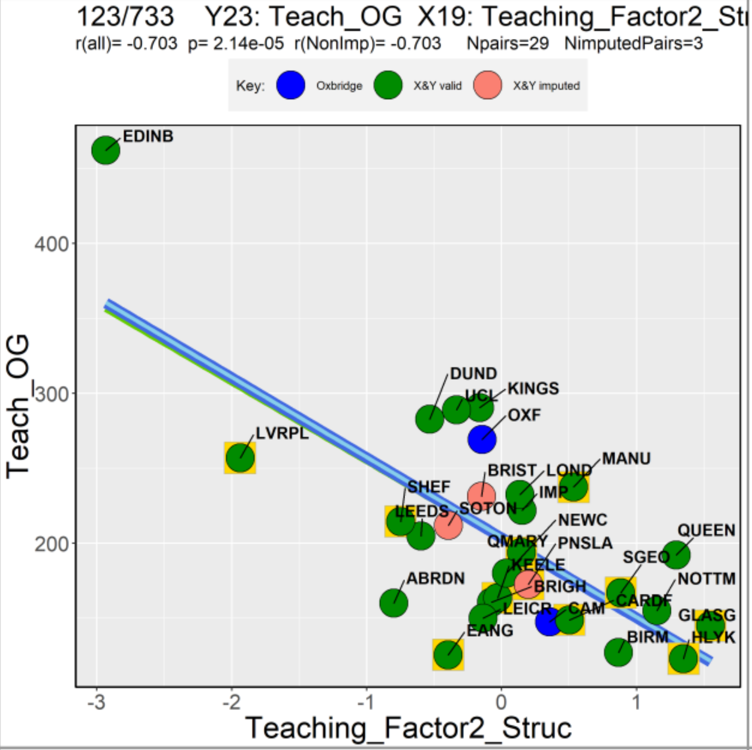
123/733 Y23: Teach_OG X19: Teaching_Factor2_Str
r(all)= -0.703 p= 2.14e-05 r(NonImp)= -0.703 Npairs=29 NimputedPairs=3
Key: Oxbridge X&Y valid X&Y imputed
Teach_OG
Teaching_Factor2_Struc
EDINB
DUND
UCL
KINGS
OXF
LVRPL
BRIST
LOND
MANU
SHEF
IMP
LEEDS
SOTON
NEWC
QUEEN
QMARY
PNSLA
KEELE
SGEO
ABRDN
BRIGH
NOTTM
CARDF
LEICR
CAM
GLASG
EANG
BIRM
HLYK

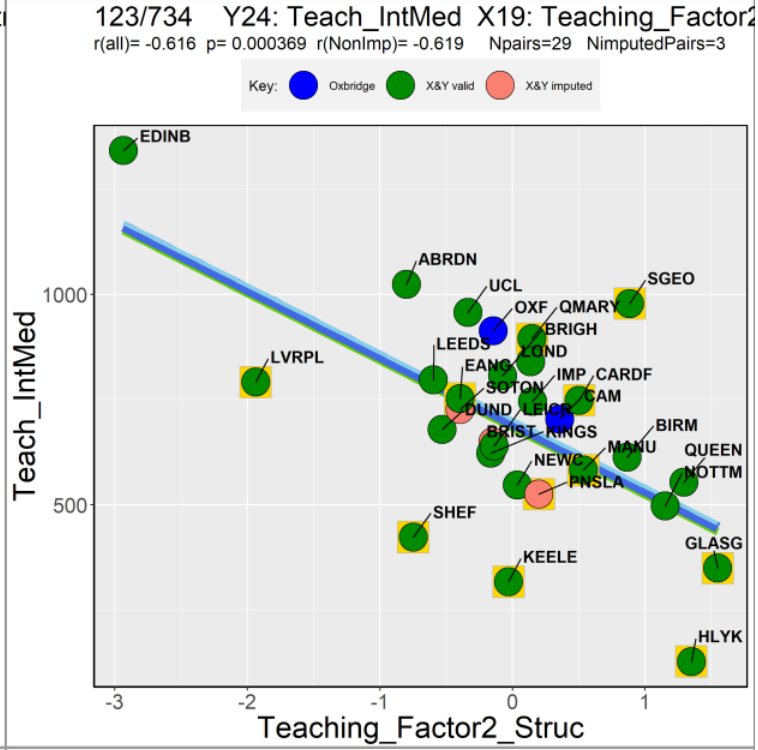
123/734 Y24: Teach_IntMed X19: Teaching_Factor2
r(all)= -0.616 p= 0.000369 r(NonImp)= -0.619 Npairs=29 NimputedPairs=3
Key: Oxbridge X&Y valid X&Y imputed
Teach_IntMed
Teaching_Factor2_Struc
EDINB
ABRDN
SGEO
UCL
OXF
QMARY
BRIGH
LVRPL
LEEDS
LOND
EANG
IMP
CARDF
SOTON
CAM
DUND
LEICR
BIRM
BRIST
KINGS
MANU
QUEEN
NEWC
NOTTM
PNSLA
SHEF
GLASG
KEELE
HLYK

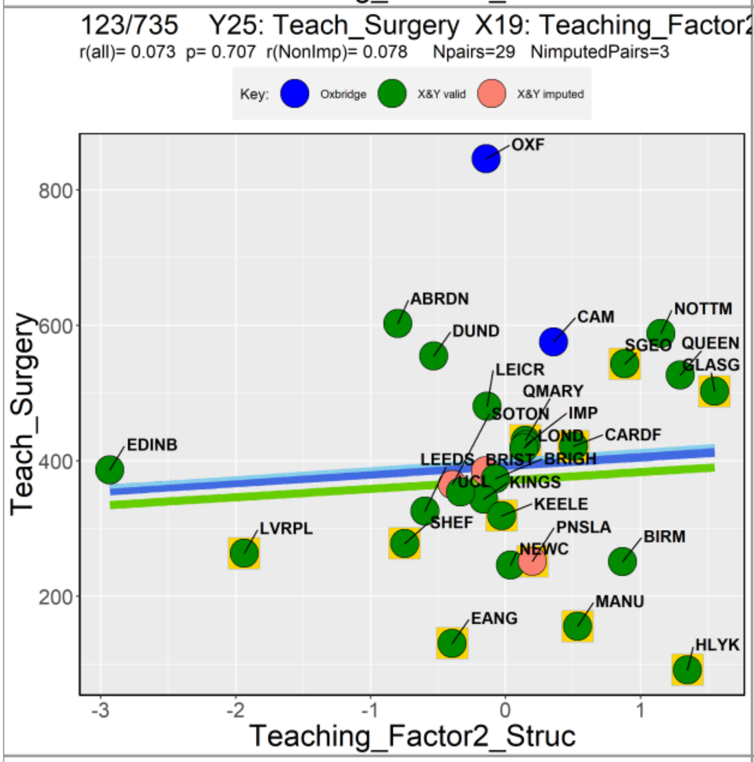
123/735 Y25: Teach_Surgery X19: Teaching_Factor2
r(all)= 0.073 p= 0.707 r(NonImp)= 0.078 Npairs=29 NimputedPairs=3
Key: Oxbridge X&Y valid X&Y imputed
Teach_Surgery
Teaching_Factor2_Struc
OXF
ABRDN
CAM
NOTTM
DUND
SGEO
QUEEN
LEICR
GLASG
QMARY
EDINB
SOTON
IMP
LOND
CARDF
LEEDS
BRIST
BRIGH
UCL
KINGS
KEELE
SHEF
LVRPL
PNSLA
BIRM
NEWC
MANU
EANG
HLYK

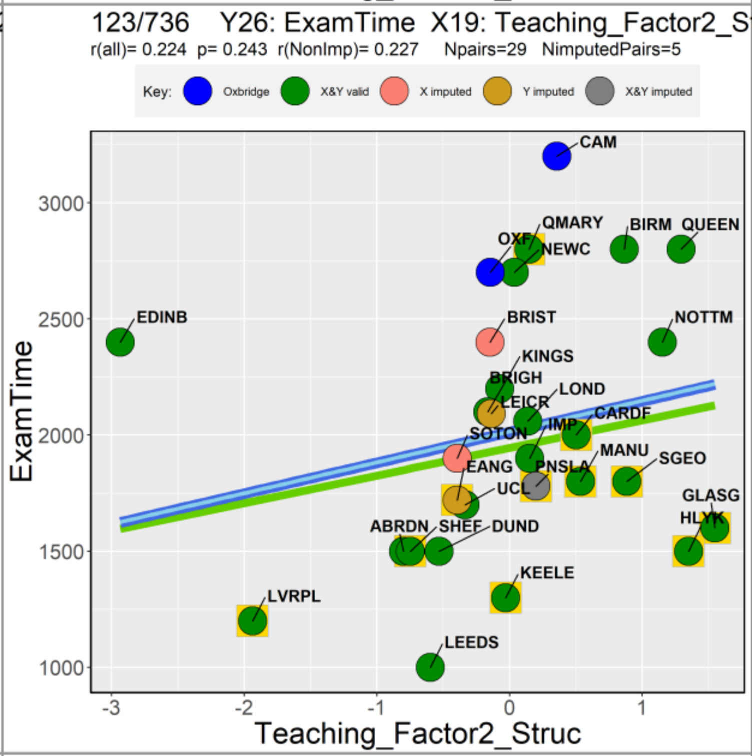
123/736 Y26: ExamTime X19: Teaching_Factor2_S
r(all)= 0.224 p= 0.243 r(NonImp)= 0.227 Npairs=29 NimputedPairs=5
Key: Oxbridge X&Y valid X imputed Y imputed X&Y imputed
ExamTime
Teaching_Factor2_Struc
CAM
QMARY
OXF
BIRM
QUEEN
NEWC
EDINB
BRIST
NOTTM
KINGS
BRIGH
LOND
LEICR
CARDF
IMP
SOTON
MANU
SGEO
EANG
PNSLA
UCL
GLASG
HLYK
ABRDN
SHEF
DUND
KEELE
LVRPL
LEEDS

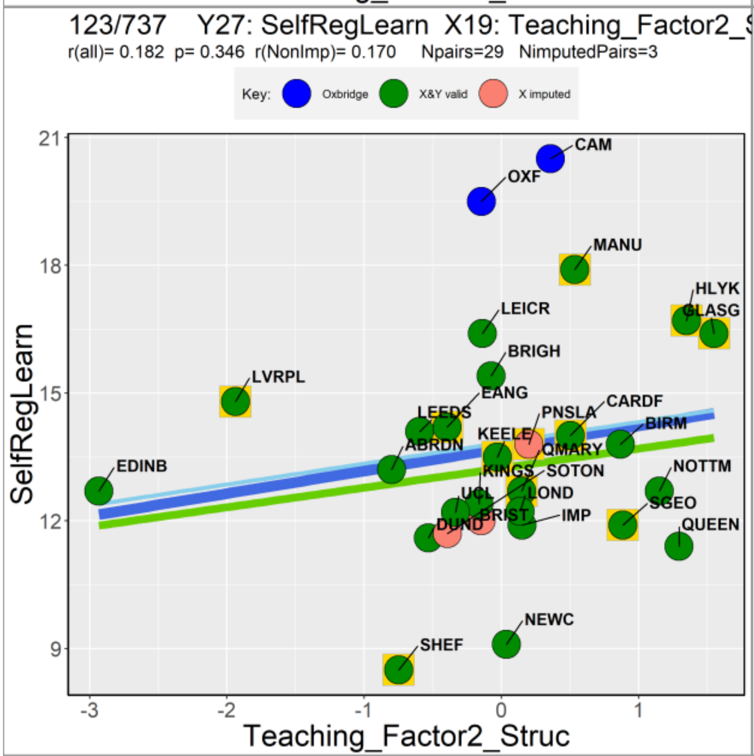
123/737 Y27: SelfRegLearn X19: Teaching_Factor2_S
r(all)= 0.182 p= 0.346 r(NonImp)= 0.170 Npairs=29 NimputedPairs=3
Key: Oxbridge X&Y valid X imputed
SelfRegLearn
Teaching_Factor2_Struc
CAM
OXF
MANU
HLYK
LEICR
GLASG
BRIGH
LVRPL
EANG
CARDF
LEEDS
PNSLA
BIRM
ABRDN
KEELE
QMARY
EDINB
KINGS
SOTON
NOTTM
UCL
LOND
SGEO
DUND
BRIST
IMP
QUEEN
NEWC
SHEF

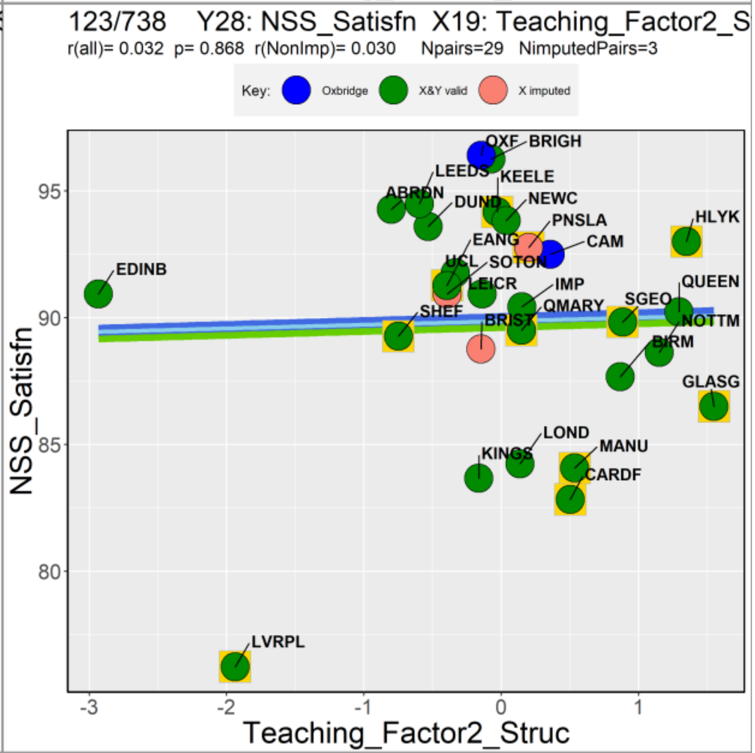
123/738 Y28: NSS_Satisfn X19: Teaching_Factor2_S
r(all)= 0.032 p= 0.868 r(NonImp)= 0.030 Npairs=29 NimputedPairs=3
Key: Oxbridge X&Y valid X imputed
NSS_Satisfn
Teaching_Factor2_Struc
OXF
BRIGH
LEEDS
KEELE
ABRDN
DUND
NEWC
PNSLA
HLYK
EANG
CAM
EDINB
UCL
SOTON
LEICR
IMP
QUEEN
SGEO
SHEF
QMARY
BRIST
NOTTM
BIRM
GLASG
LOND
MANU
KINGS
CARDF
LVRPL

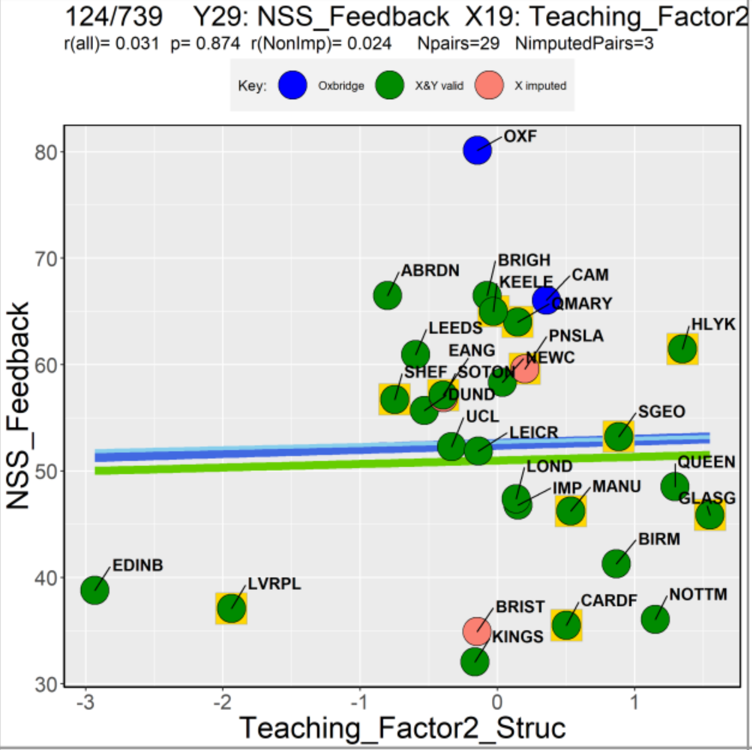

124/739 Y29: NSS_Feedback X19: Teaching_Factor2
r(all)= 0.031 p= 0.874 r(NonImp)= 0.024 Npairs=29 NimputedPairs=3
Key: Oxbridge X&Y valid X imputed
NSS_Feedback
Teaching_Factor2_Struc
OXF
ABRDN
BRIGH
KEELE
CAM
QMARY
LEEDS
PNSLA
HLYK
EANG
NEWC
SHEF
SOTON
DUND
UCL
SGEO
LEICR
QUEEN
LOND
IMP
MANU
GLASG
BIRM
EDINB
LVRPL
BRIST
CARDF
NOTTM
KINGS

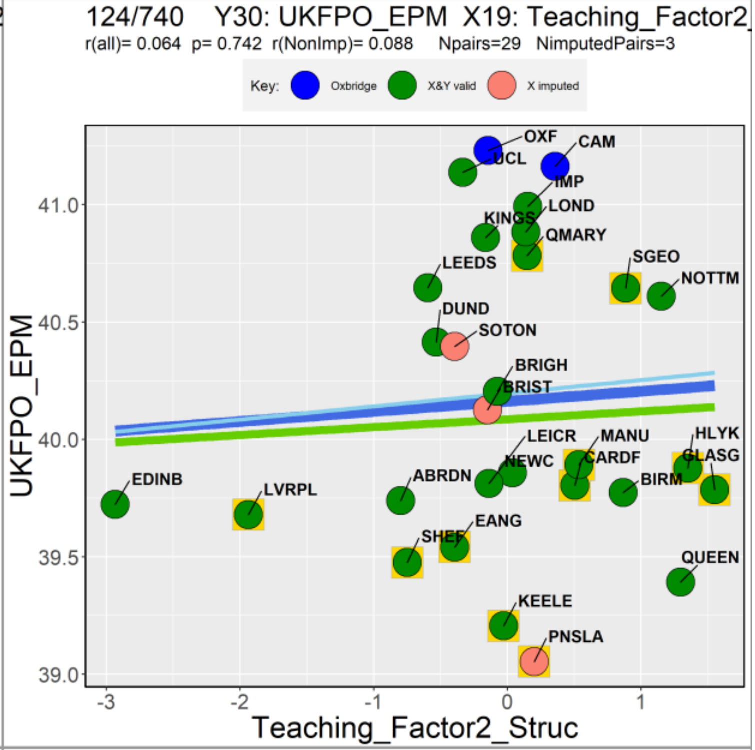

124/740 Y30: UKFPO_EPM X19: Teaching_Factor2
r(all)= 0.064 p= 0.742 r(NonImp)= 0.088 Npairs=29 NimputedPairs=3
Key: Oxbridge X&Y valid X imputed
UKFPO_EPM
Teaching_Factor2_Struc
OXF
CAM
UCL
IMP
LOND
KINGS
QMARY
LEEDS
SGEO
NOTTM
DUND
SOTON
BRIGH
BRIST
LEICR
MANU
HLYK
NEWC
CARDF
GLASG
EDINB
ABRDN
BIRM
LVRPL
EANG
SHEF
QUEEN
KEELE
PNSLA

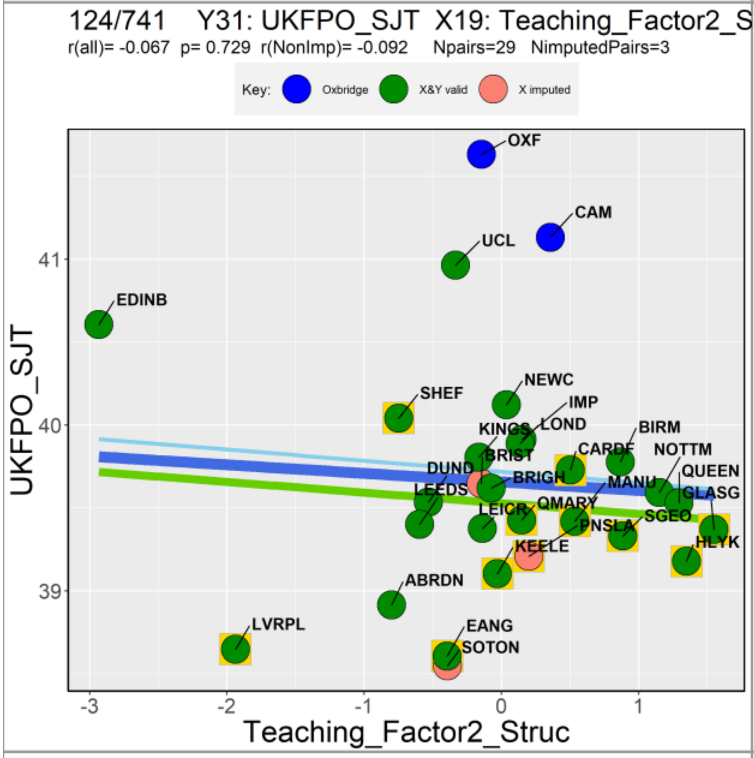

124/741 Y31: UKFPO_SJT X19: Teaching_Factor2_S
r(all)= -0.067 p= 0.729 r(NonImp)= -0.092 Npairs=29 NimputedPairs=3
Key: Oxbridge X&Y valid X imputed
UKFPO_SJT
Teaching_Factor2_Struc
OXF
CAM
UCL
EDINB
NEWC
SHEF
IMP
KINGS
LOND
BIRM
BRIST
CARDF
NOTTM
DUND
BRIGH
QUEEN
LEEDS
MANU
GLASG
LEICR
QMARY
SGEO
PNSLA
HLYK
KEELE
ABRDN
LVRPL
EANG
SOTON

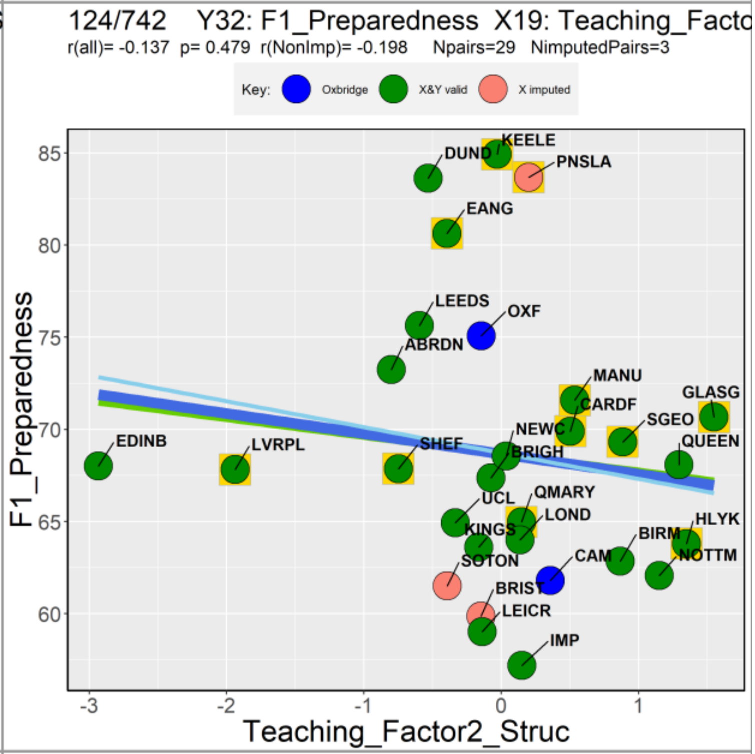

124/742 Y32: F1_Preparedness X19: Teaching_Facto
r(all)= -0.137 p= 0.479 r(NonImp)= -0.198 Npairs=29 NimputedPairs=3
Key: Oxbridge X&Y valid X imputed
F1_Preparedness
Teaching_Factor2_Struc
DUND
KEELE
PNSLA
EANG
LEEDS
OXF
ABRDN
MANU
GLASG
CARDF
SGEO
EDINB
LVRPL
SHEF
NEWC
BRIGH
QUEEN
UCL
QMARY
LOND
KINGS
HLYK
BIRM
SOTON
CAM
NOTTM
BRIST
LEICR
IMP

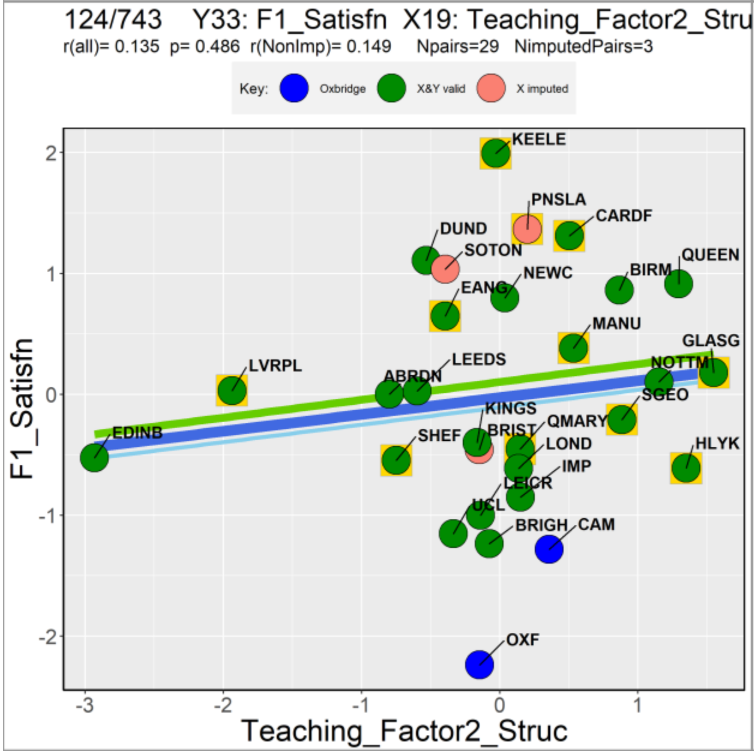

124/743 Y33: F1_Satisfn X19: Teaching_Factor2_Stru
r(all)= 0.135 p= 0.486 r(NonImp)= 0.149 Npairs=29 NimputedPairs=3
Key: Oxbridge X&Y valid X imputed
F1_Satisfn
Teaching_Factor2_Struc
KEELE
PNSLA
CARDF
DUND
SOTON
QUEEN
BIRM
EANG
NEWC
MANU
GLASG
LVRPL
ABRDN
LEEDS
NOTTM
SGEO
EDINB
KINGS
SHEF
BRIST
QMARY
LOND
HLYK
IMP
LEICR
UCL
BRIGH
CAM
OXF

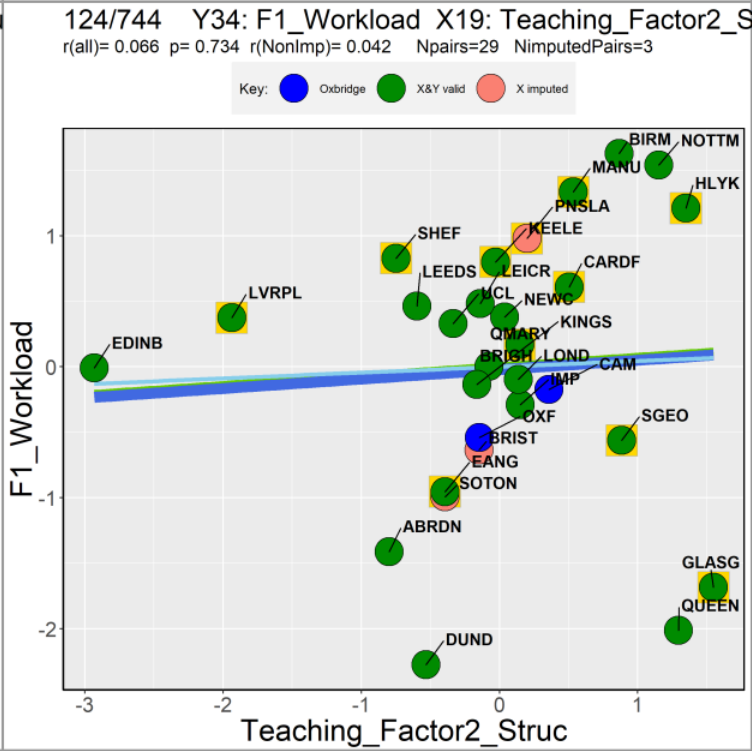

124/744 Y34: F1_Workload X19: Teaching_Factor2_S
r(all)= 0.066 p= 0.734 r(NonImp)= 0.042 Npairs=29 NimputedPairs=3
Key: Oxbridge X&Y valid X imputed
F1_Workload
Teaching_Factor2_Struc
BIRM
NOTTM
MANU
HLYK
PNSLA
SHEF
KEELE
CARDF
LEEDS
LEICR
UCL
NEWC
LVRPL
QMARY
KINGS
EDINB
BRIGH
LOND
CAM
IMP
OXF
SGEO
BRIST
EANG
SOTON
ABRDN
GLASG
QUEEN
DUND

125/745 Y35: F1_Supervn X19: Teaching_Factor2_Str

r(all)= 0.555 p= 0.00178 r(NonImp)= 0.551 Npairs=29 NimputedPairs=3

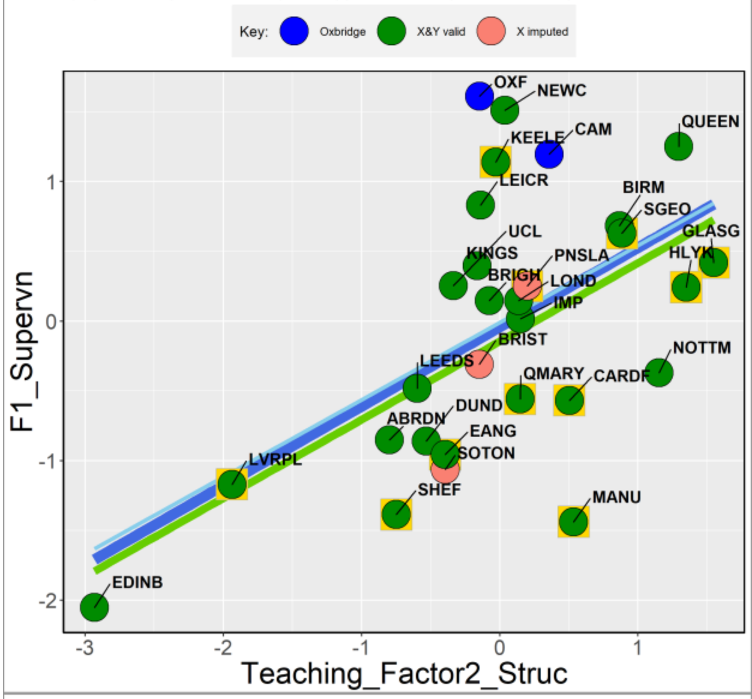

125/746 Y36: Trainee_GP X19: Teaching_Factor2_St

r(all)= 0.098 p= 0.614 r(NonImp)= 0.083 Npairs=29 NimputedPairs=3

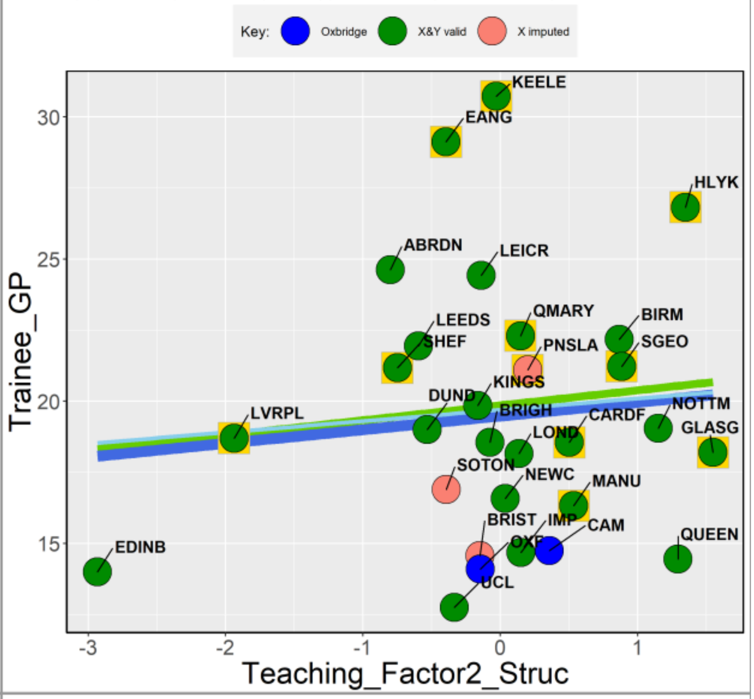

125/747 Y37: Trainee_Psyc X19: Teaching_Factor2_S

r(all)= 0.094 p= 0.629 r(NonImp)= 0.090 Npairs=29 NimputedPairs=3

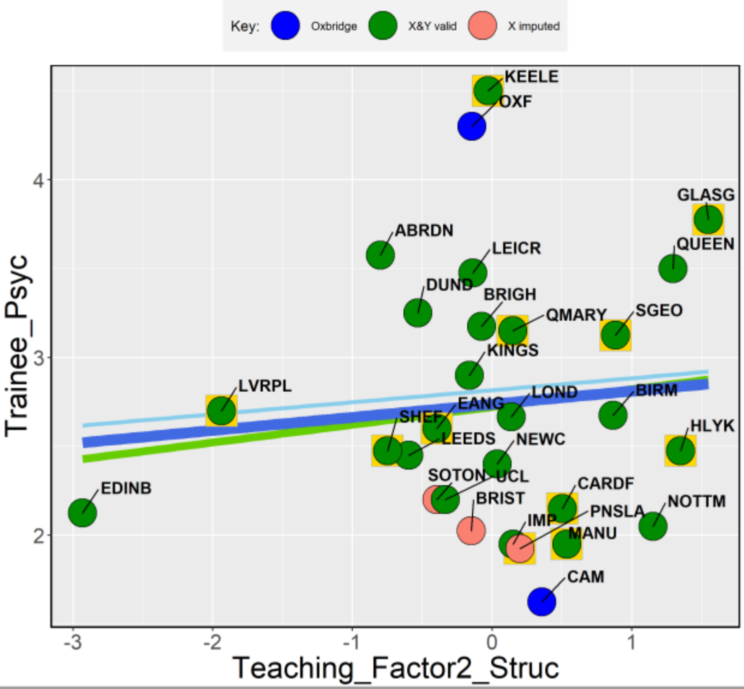

125/748 Y38: TraineeApp_Surgery X19: Teaching_F

r(all)= 0.232 p= 0.226 r(NonImp)= 0.244 Npairs=29 NimputedPairs=5

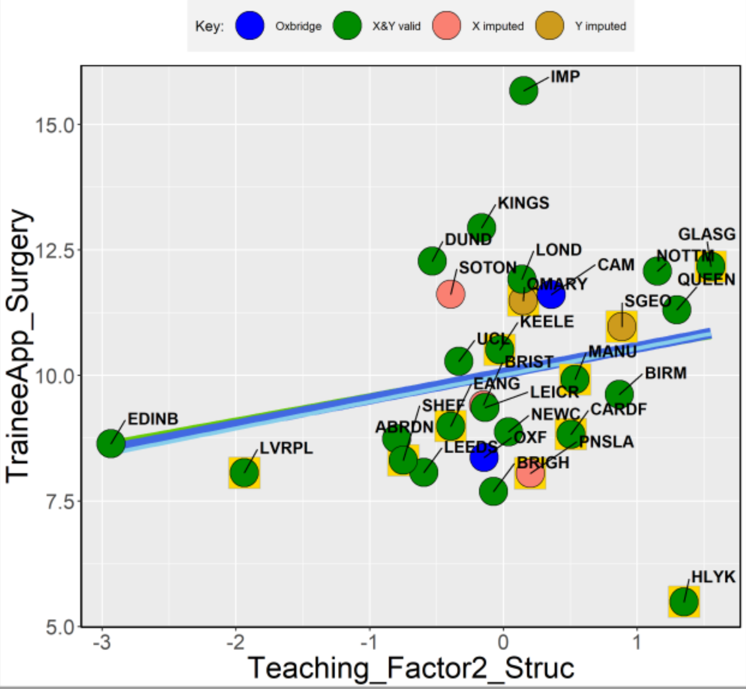

125/749 Y39: TraineeApp_Anaes X19: Teaching_Fa

r(all)= -0.072 p= 0.709 r(NonImp)= -0.068 Npairs=29 NimputedPairs=3

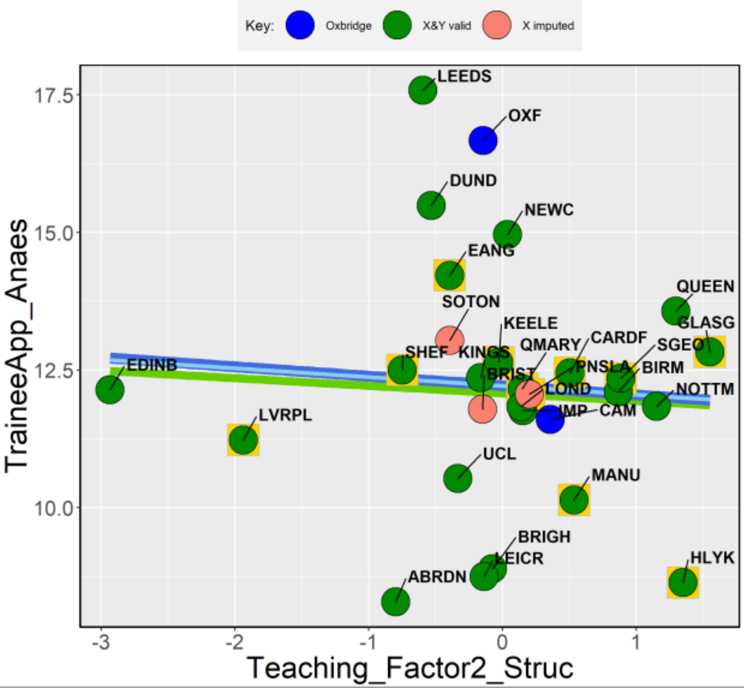

125/750 Y40: GMC_PGexams X19: Teaching_Factor2

r(all)= -0.056 p= 0.775 r(NonImp)= -0.054 Npairs=29 NimputedPairs=3

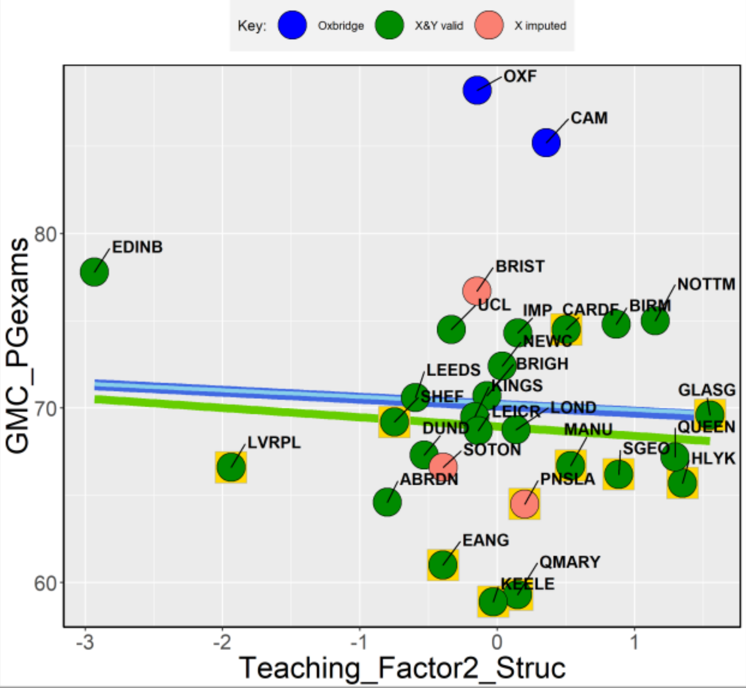

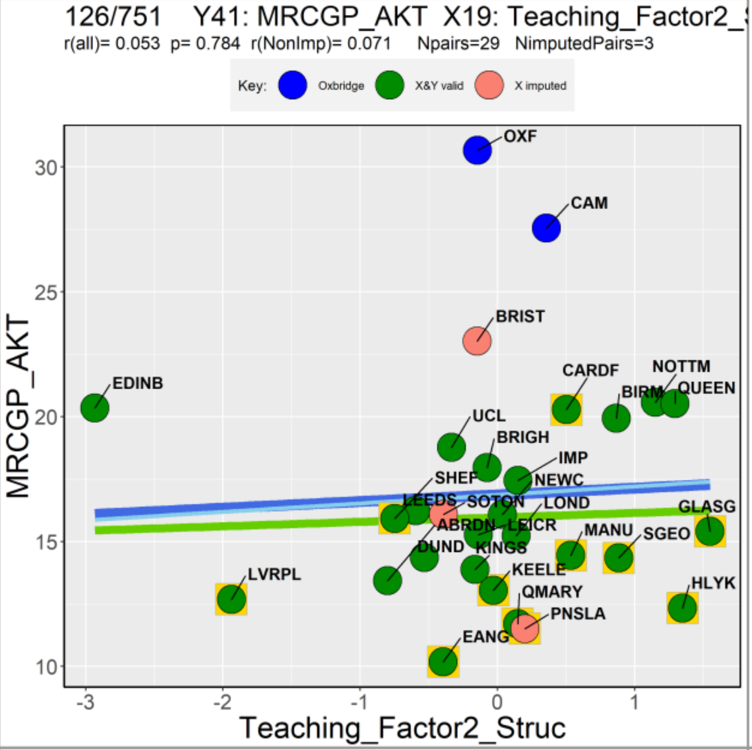
126/751 Y41: MRCGP_AKT X19: Teaching_Factor2_
r(all)= 0.053 p= 0.784 r(NonImp)= 0.071 Npairs=29 NimputedPairs=3
Key: Oxbridge X&Y valid X imputed
MRCGP_AKT
Teaching_Factor2_Struc
OXF
CAM
BRIST
EDINB
CARDF
NOTTM
QUEEN
BIRM
UCL
BRIGH
IMP
SHEF
NEWC
LEEDS
SOTON
LOND
ABRDN
LEICR
GLASG
DUND
KINGS
MANU
SGEO
LVRPL
KEELE
QMARY
HLYK
PNSLA
EANG

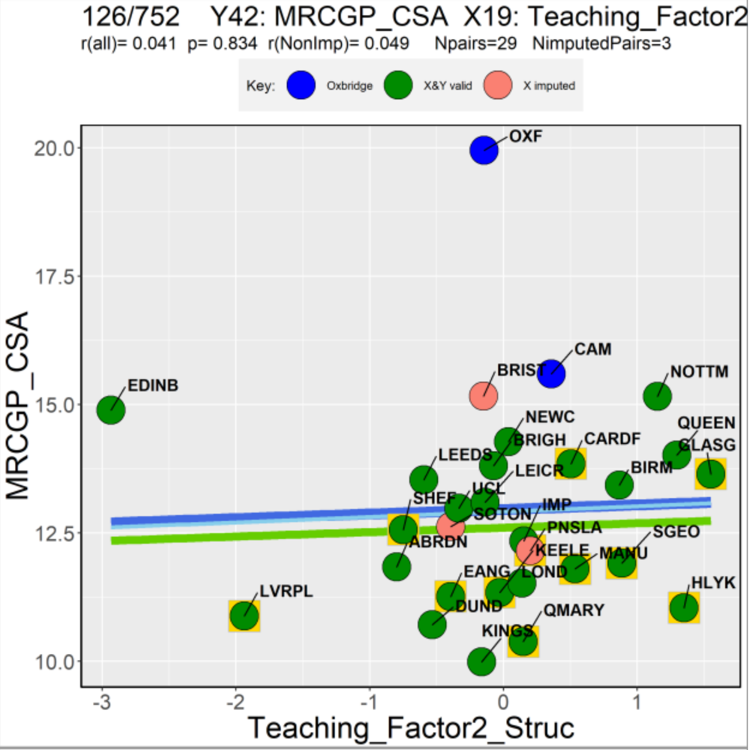
126/752 Y42: MRCGP_CSA X19: Teaching_Factor2
r(all)= 0.041 p= 0.834 r(NonImp)= 0.049 Npairs=29 NimputedPairs=3
Key: Oxbridge X&Y valid X imputed
MRCGP_CSA
Teaching_Factor2_Struc

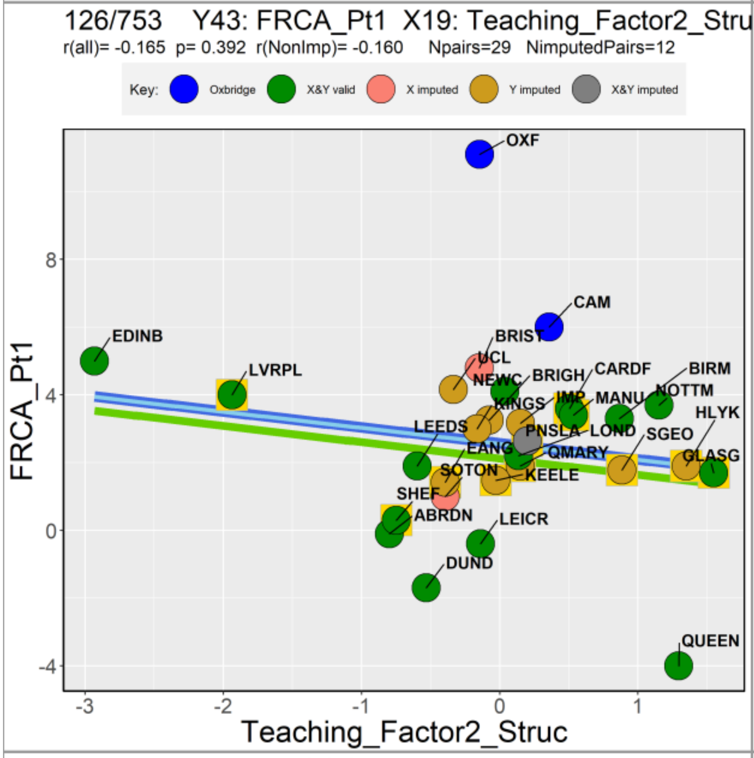
126/753 Y43: FRCA_Pt1 X19: Teaching_Factor2_Stru
r(all)= -0.165 p= 0.392 r(NonImp)= -0.160 Npairs=29 NimputedPairs=12
Key: Oxbridge X&Y valid X imputed Y imputed X&Y imputed
FRCA_Pt1
Teaching_Factor2_Struc

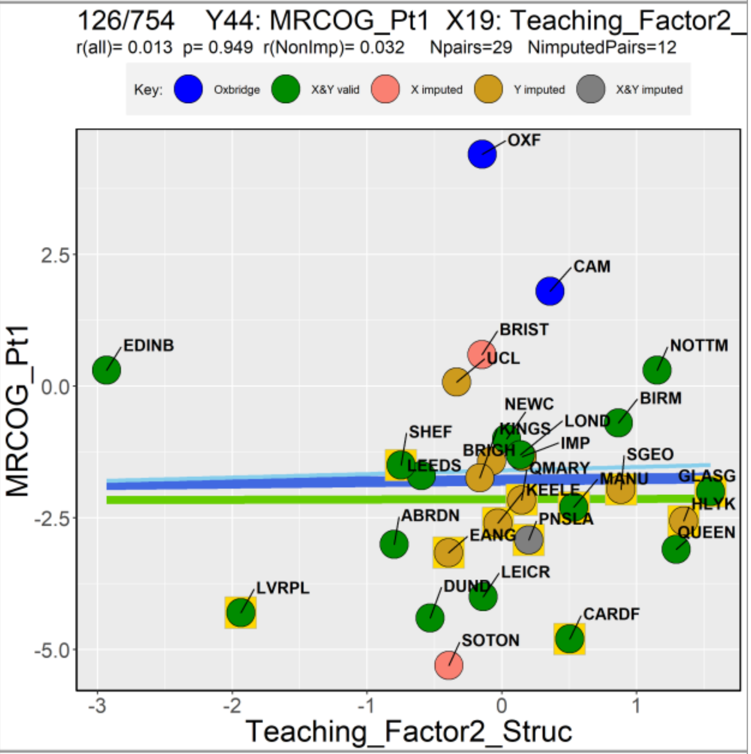
126/754 Y44: MRCOG_Pt1 X19: Teaching_Factor2_
r(all)= 0.013 p= 0.949 r(NonImp)= 0.032 Npairs=29 NimputedPairs=12
Key: Oxbridge X&Y valid X imputed Y imputed X&Y imputed
MRCOG_Pt1
Teaching_Factor2_Struc

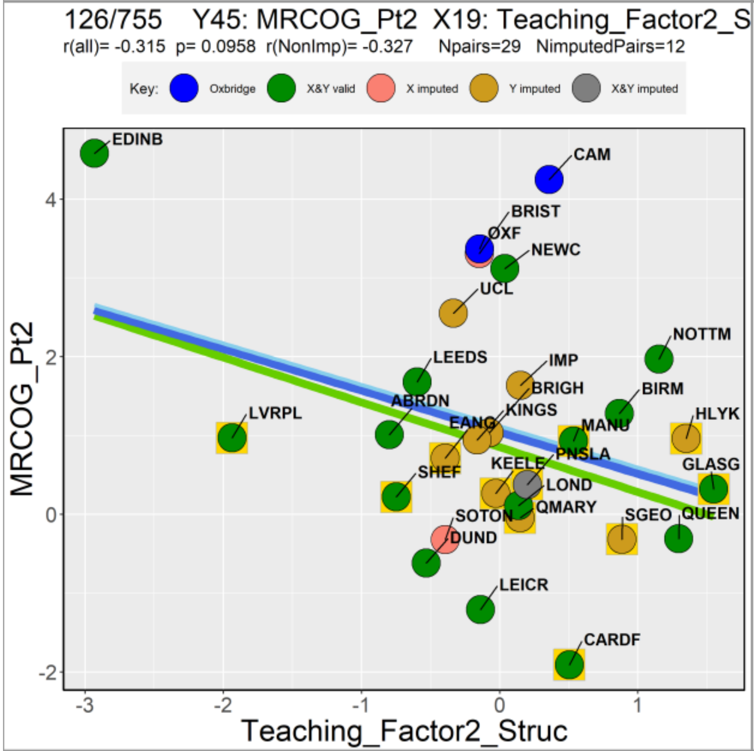
126/755 Y45: MRCOG_Pt2 X19: Teaching_Factor2_S
r(all)= -0.315 p= 0.0958 r(NonImp)= -0.327 Npairs=29 NimputedPairs=12
Key: Oxbridge X&Y valid X imputed Y imputed X&Y imputed
MRCOG_Pt2
Teaching_Factor2_Struc

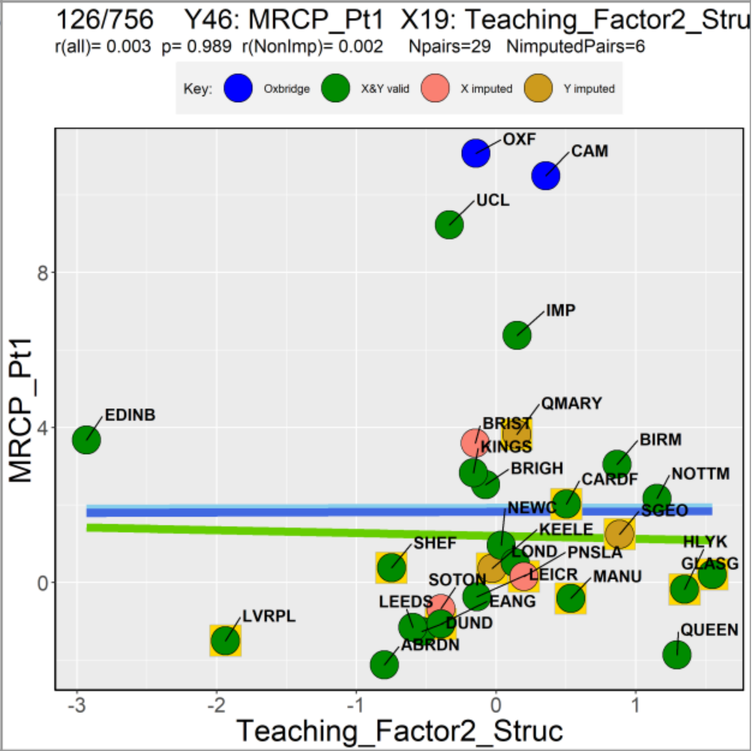
126/756 Y46: MRCP_Pt1 X19: Teaching_Factor2_Stru
r(all)= 0.003 p= 0.989 r(NonImp)= 0.002 Npairs=29 NimputedPairs=6
Key: Oxbridge X&Y valid X imputed Y imputed
MRCP_Pt1
Teaching_Factor2_Struc

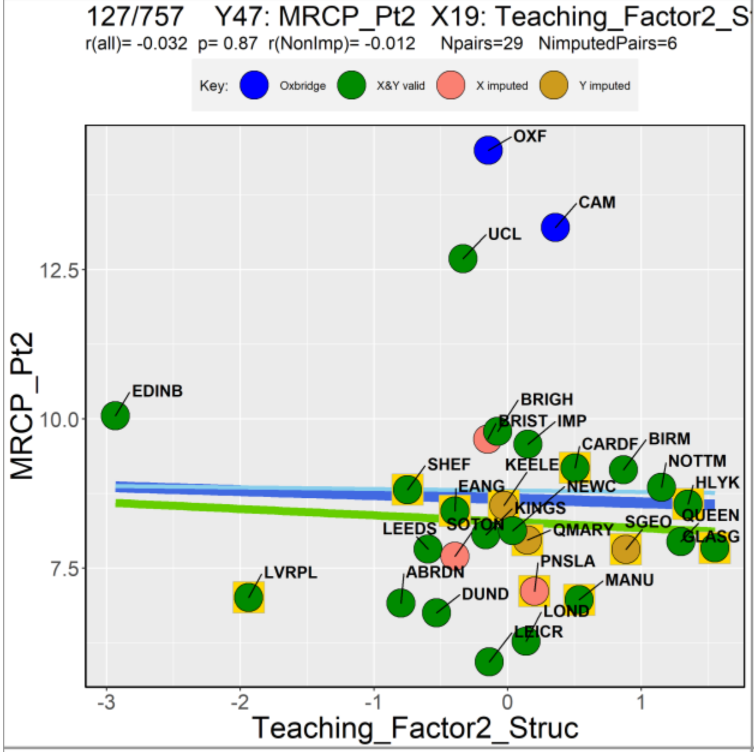
127/757 Y47: MRCP_Pt2 X19: Teaching_Factor2_S
r(all)= -0.032 p= 0.87 r(NonImp)= -0.012 Npairs=29 NimputedPairs=6
Key: Oxbridge X&Y valid X imputed Y imputed
MRCP_Pt2
Teaching_Factor2_Struc

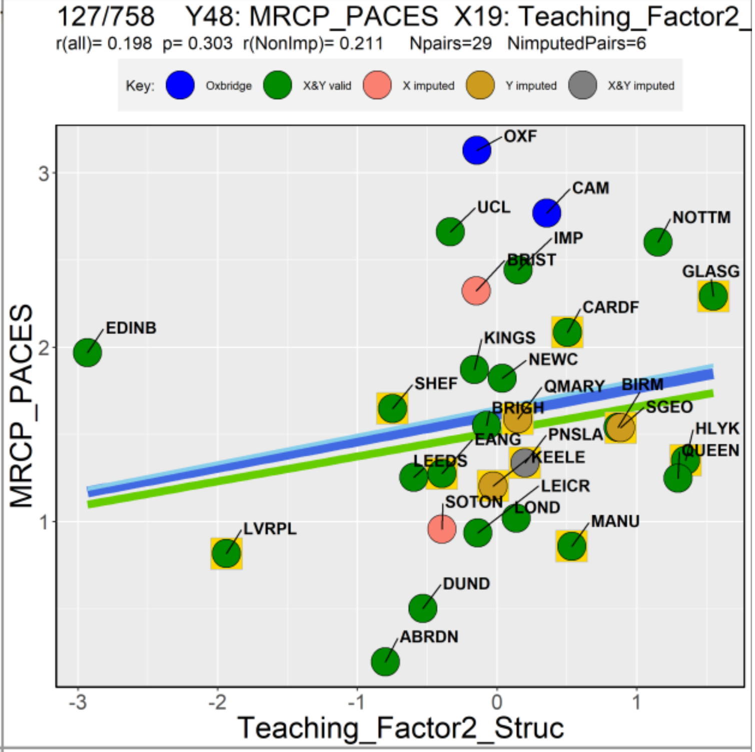
127/758 Y48: MRCP_PACES X19: Teaching_Factor2_
r(all)= 0.198 p= 0.303 r(NonImp)= 0.211 Npairs=29 NimputedPairs=6
Key: Oxbridge X&Y valid X imputed Y imputed X&Y imputed
MRCP_PACES
Teaching_Factor2_Struc

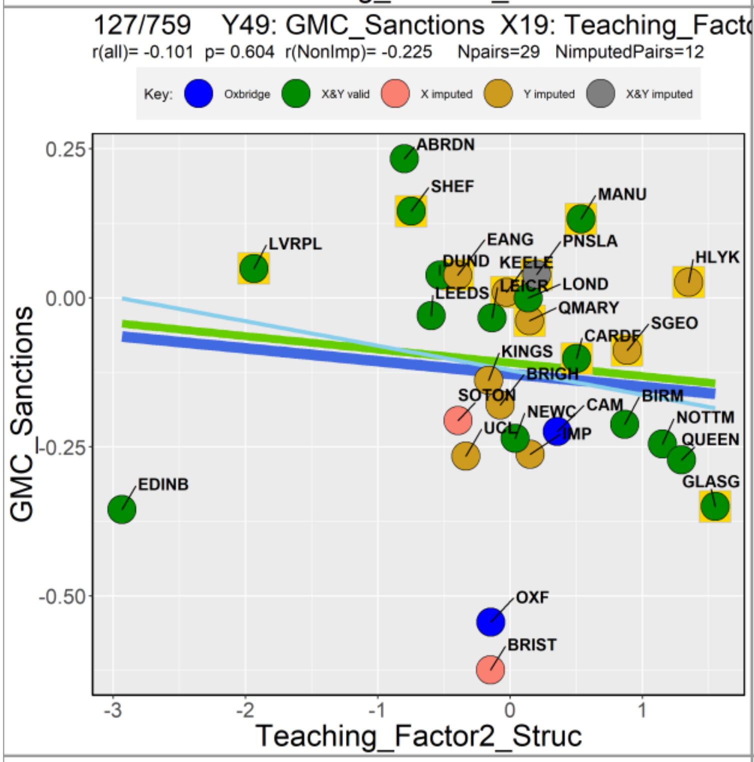
127/759 Y49: GMC_Sanctions X19: Teaching_Facto
r(all)= -0.101 p= 0.604 r(NonImp)= -0.225 Npairs=29 NimputedPairs=12
Key: Oxbridge X&Y valid X imputed Y imputed X&Y imputed
GMC_Sanctions
Teaching_Factor2_Struc

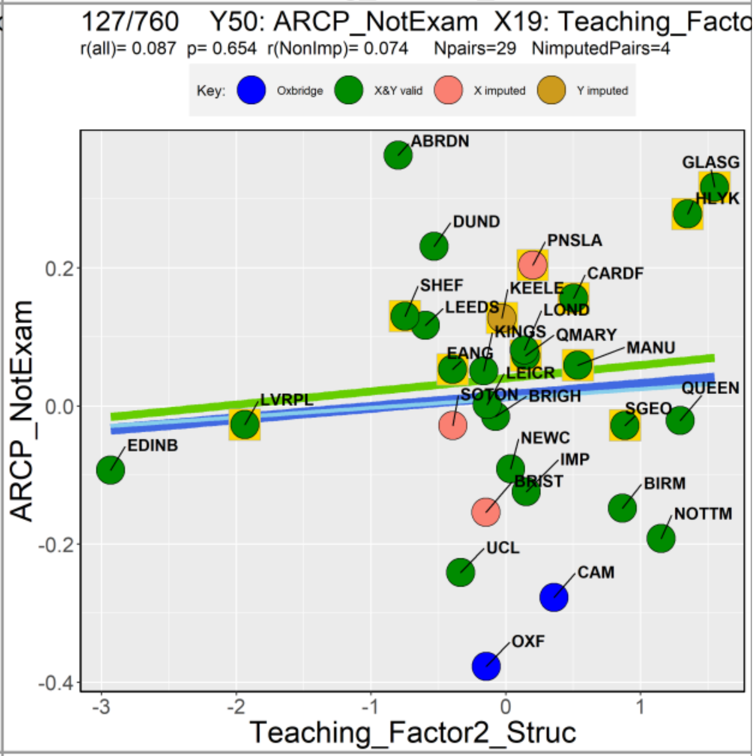
127/760 Y50: ARCP_NotExam X19: Teaching_Facto
r(all)= 0.087 p= 0.654 r(NonImp)= 0.074 Npairs=29 NimputedPairs=4
Key: Oxbridge X&Y valid X imputed Y imputed
ARCP_NotExam
Teaching_Factor2_Struc

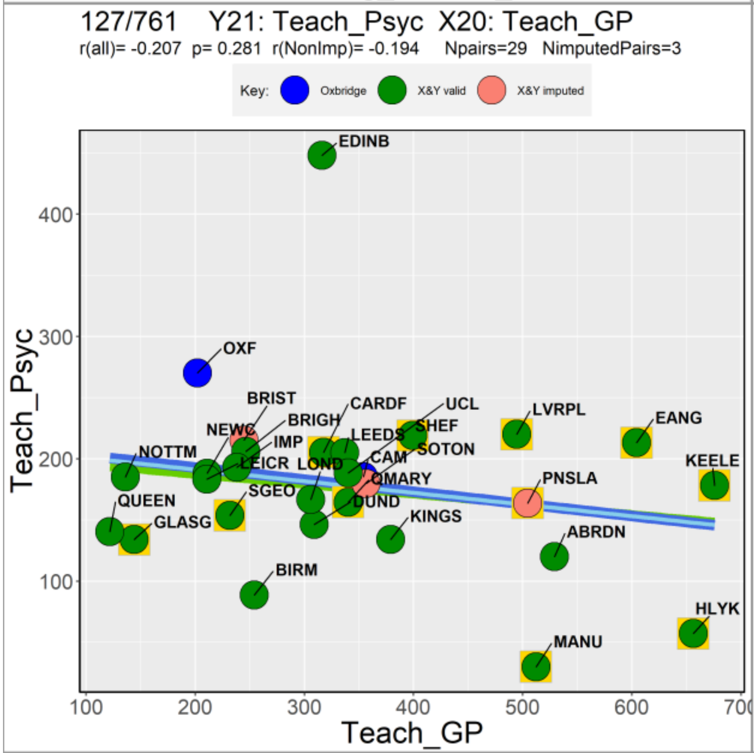
127/761 Y21: Teach_Psyc X20: Teach_GP
r(all)= -0.207 p= 0.281 r(NonImp)= -0.194 Npairs=29 NimputedPairs=3
Key: Oxbridge X&Y valid X&Y imputed
Teach_Psyc
Teach_GP

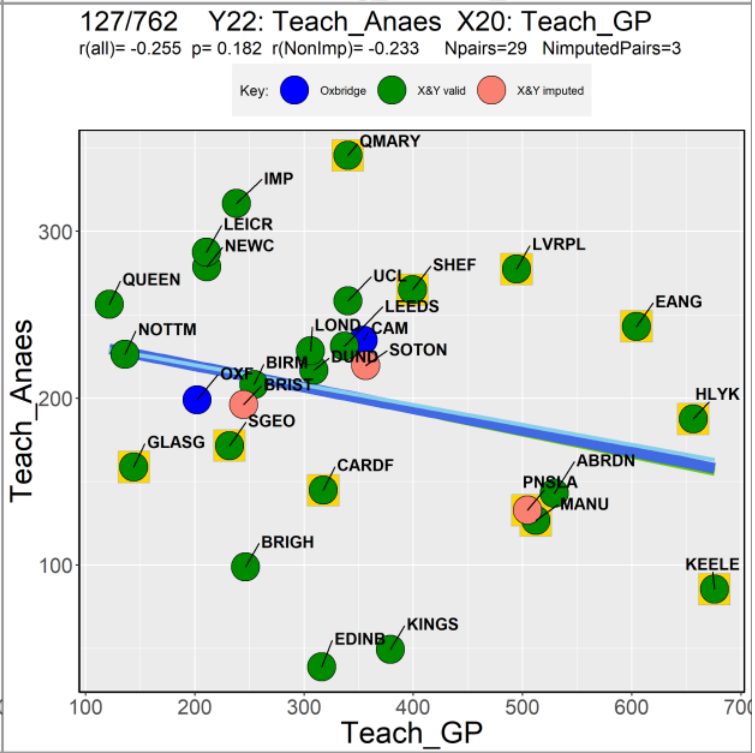
127/762 Y22: Teach_Anaes X20: Teach_GP
r(all)= -0.255 p= 0.182 r(NonImp)= -0.233 Npairs=29 NimputedPairs=3
Key: Oxbridge X&Y valid X&Y imputed
Teach_Anaes
Teach_GP

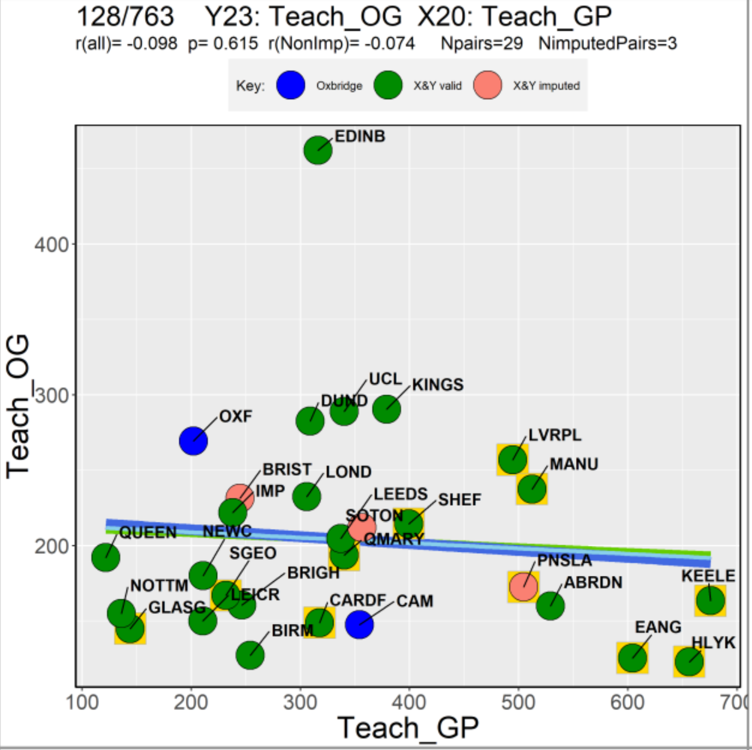
128/763 Y23: Teach_OG X20: Teach_GP
r(all)= -0.098 p= 0.615 r(NonImp)= -0.074 Npairs=29 NimputedPairs=3
Key: Oxbridge X&Y valid X&Y imputed
Teach_OG
Teach_GP
EDINB
OXF
UCL
KINGS
DUND
LVRPL
MANU
BRIST
LOND
IMP
LEEDS
SHEF
SOTON
QMARY
QUEEN
NEWC
SGEO
BRIGH
PNSLA
ABRDN
KEELE
NOTTM
GLASG
LEICR
CARDF
CAM
BIRM
EANG
HLYK

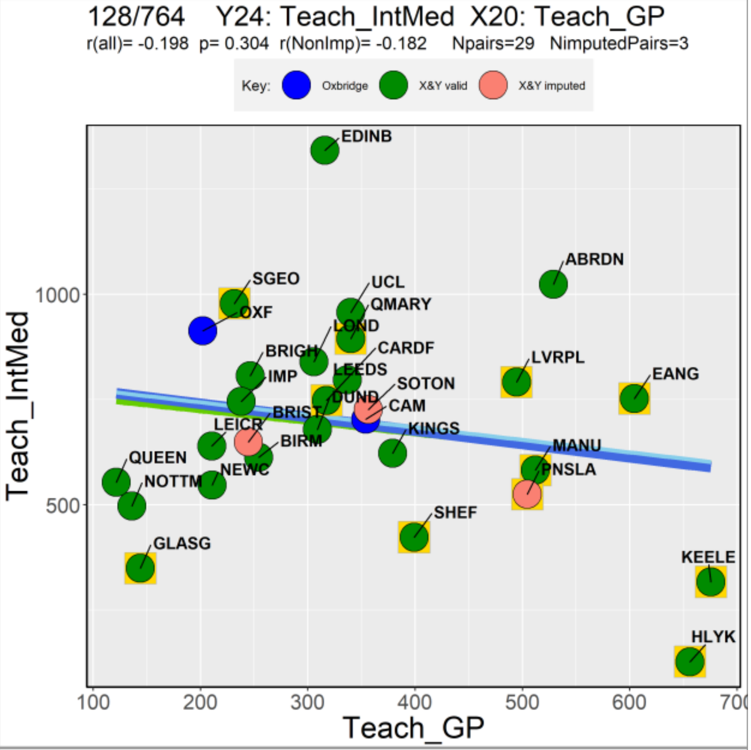
128/764 Y24: Teach_IntMed X20: Teach_GP
r(all)= -0.198 p= 0.304 r(NonImp)= -0.182 Npairs=29 NimputedPairs=3
Key: Oxbridge X&Y valid X&Y imputed
Teach_IntMed
Teach_GP
EDINB
ABRDN
SGEO
UCL
QMARY
OXF
LOND
CARDF
BRIGH
LVRPL
EANG
IMP
LEEDS
SOTON
DUND
CAM
BRIST
LEICR
KINGS
BIRM
MANU
QUEEN
NEWC
PNSLA
NOTTM
SHEF
GLASG
KEELE
HLYK

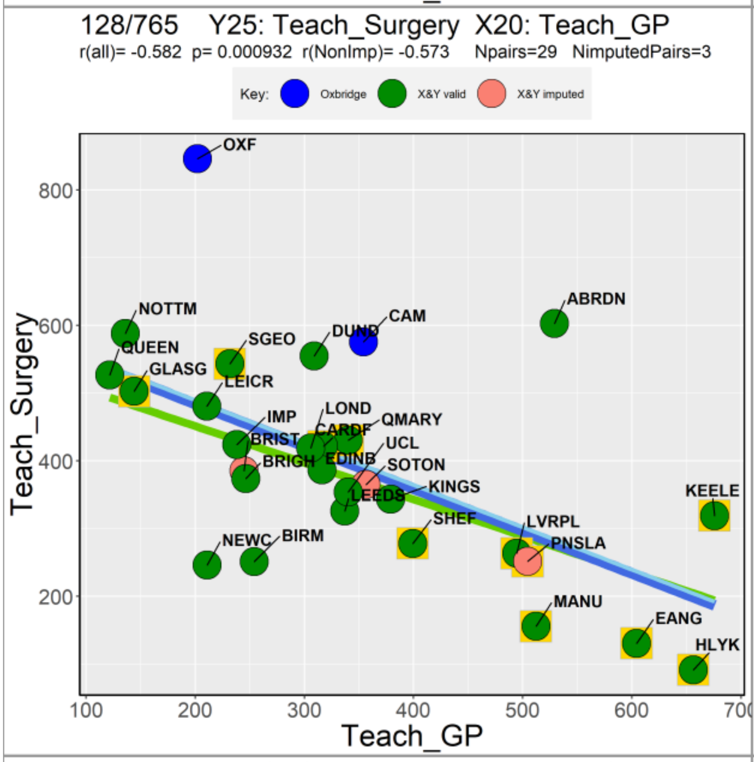
128/765 Y25: Teach_Surgery X20: Teach_GP
r(all)= -0.582 p= 0.000932 r(NonImp)= -0.573 Npairs=29 NimputedPairs=3
Key: Oxbridge X&Y valid X&Y imputed
Teach_Surgery
Teach_GP
OXF
ABRDN
NOTTM
CAM
SGEO
DUND
QUEEN
GLASG
LEICR
LOND
IMP
QMARY
CARDF
BRIST
UCL
BRIGH
EDINB
SOTON
LEEDS
KINGS
KEELE
SHEF
LVRPL
NEWC
BIRM
PNSLA
MANU
EANG
HLYK

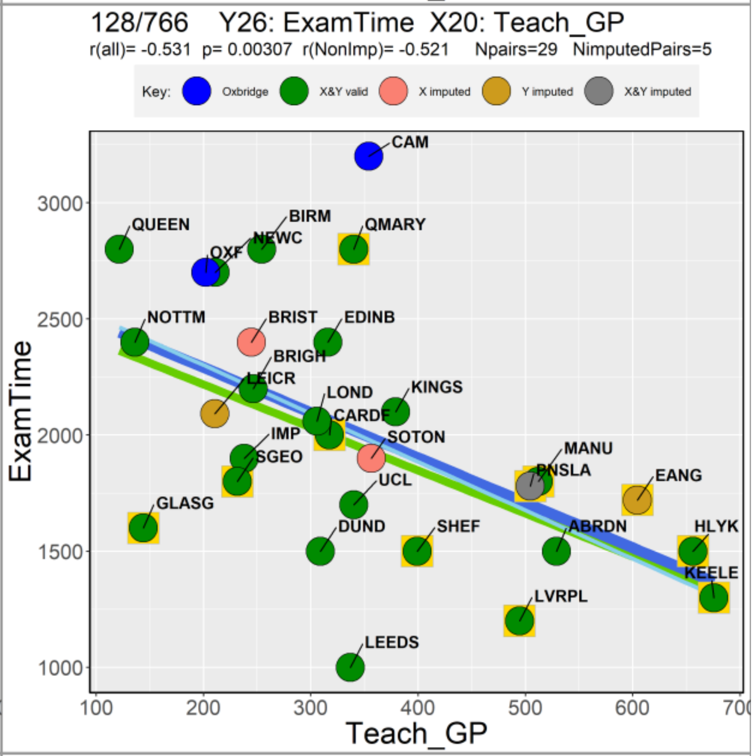
128/766 Y26: ExamTime X20: Teach_GP
r(all)= -0.531 p= 0.00307 r(NonImp)= -0.521 Npairs=29 NimputedPairs=5
Key: Oxbridge X&Y valid X imputed Y imputed X&Y imputed
ExamTime
Teach_GP
CAM
QUEEN
BIRM
QMARY
NEWC
OXF
NOTTM
BRIST
EDINB
BRIGH
LEICR
KINGS
LOND
CARDF
IMP
SOTON
SGEO
MANU
PNSLA
UCL
EANG
GLASG
DUND
SHEF
ABRDN
HLYK
KEELE
LVRPL
LEEDS

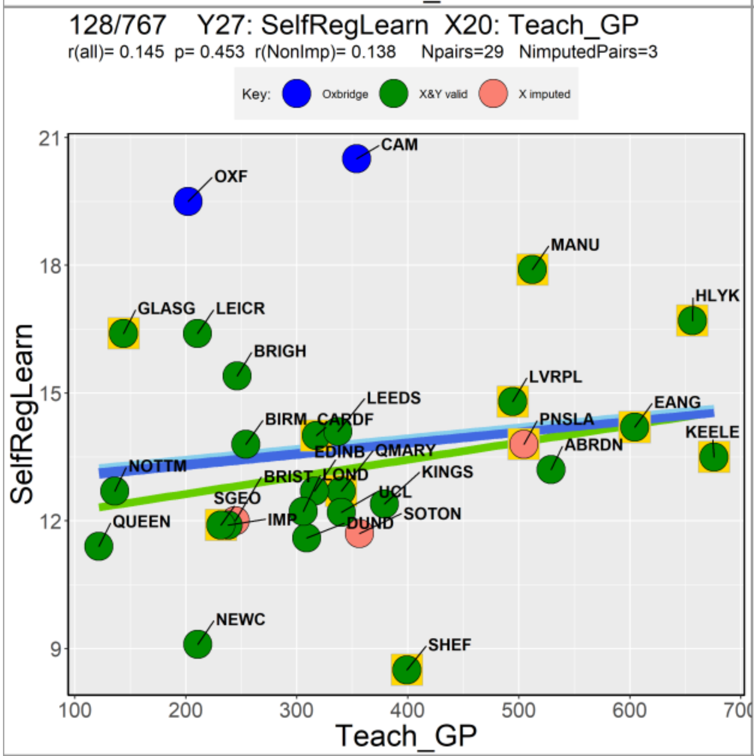
128/767 Y27: SelfRegLearn X20: Teach_GP
r(all)= 0.145 p= 0.453 r(NonImp)= 0.138 Npairs=29 NimputedPairs=3
Key: Oxbridge X&Y valid X imputed
SelfRegLearn
Teach_GP
CAM
OXF
MANU
GLASG
LEICR
HLYK
BRIGH
LVRPL
LEEDS
EANG
BIRM
CARDF
PNSLA
EDINB
QMARY
ABRDN
KEELE
NOTTM
BRIST
LOND
KINGS
UCL
SGEO
QUEEN
IMP
DUND
SOTON
NEWC
SHEF

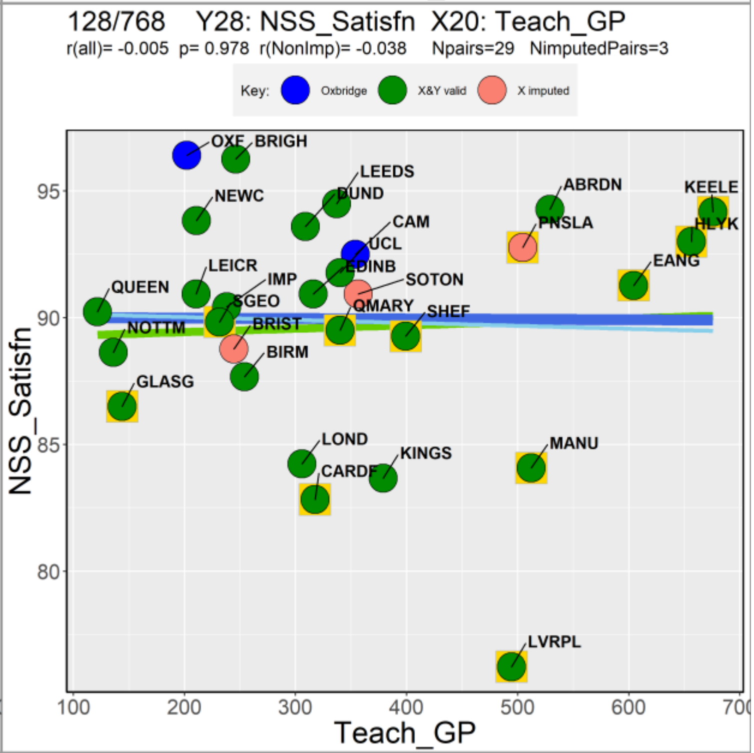
128/768 Y28: NSS_Satisfn X20: Teach_GP
r(all)= -0.005 p= 0.978 r(NonImp)= -0.038 Npairs=29 NimputedPairs=3
Key: Oxbridge X&Y valid X imputed
NSS_Satisfn
Teach_GP
OXF
BRIGH
LEEDS
ABRDN
KEELE
NEWC
DUND
CAM
PNSLA
HLYK
UCL
EANG
LEICR
EDINB
IMP
SOTON
QUEEN
SGEO
QMARY
SHEF
NOTTM
BRIST
BIRM
GLASG
LOND
KINGS
CARDF
MANU
LVRPL

129/769 Y29: NSS_Feedback X20: Teach_GP
r(all)= 0.239 p= 0.211 r(NonImp)= 0.196 Npairs=29 NimputedPairs=3

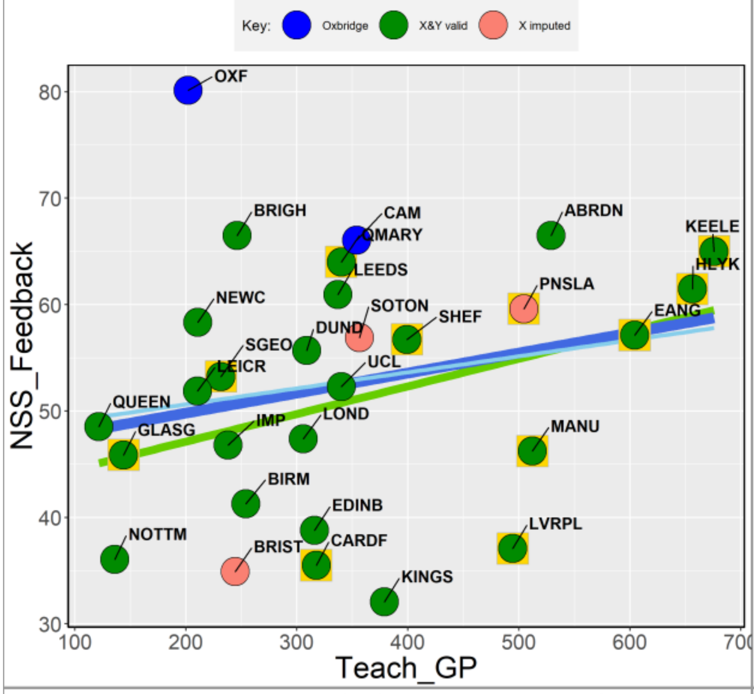

129/770 Y30: UKFPO_EPM X20: Teach_GP
r(all)= -0.374 p= 0.0458 r(NonImp)= -0.338 Npairs=29 NimputedPairs=3

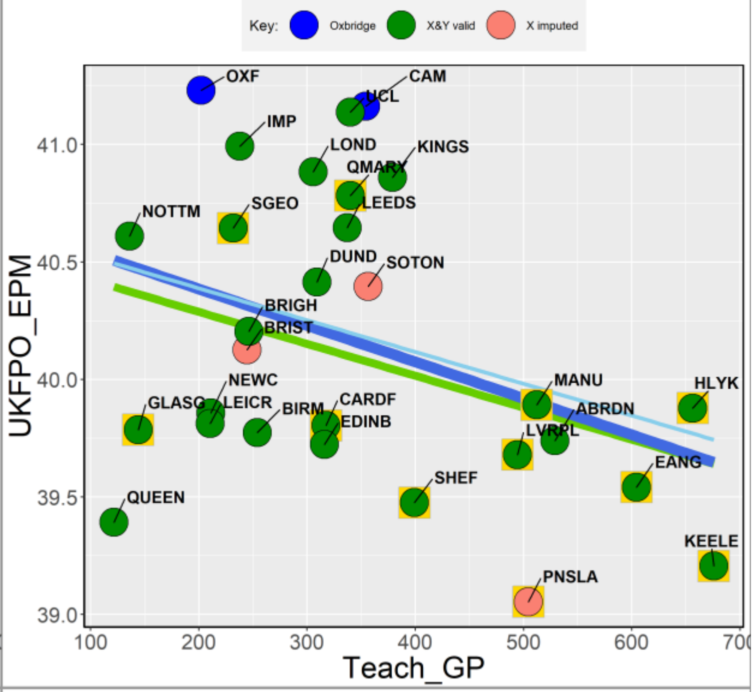

129/771 Y31: UKFPO_SJT X20: Teach_GP
r(all)= -0.404 p= 0.0297 r(NonImp)= -0.408 Npairs=29 NimputedPairs=3

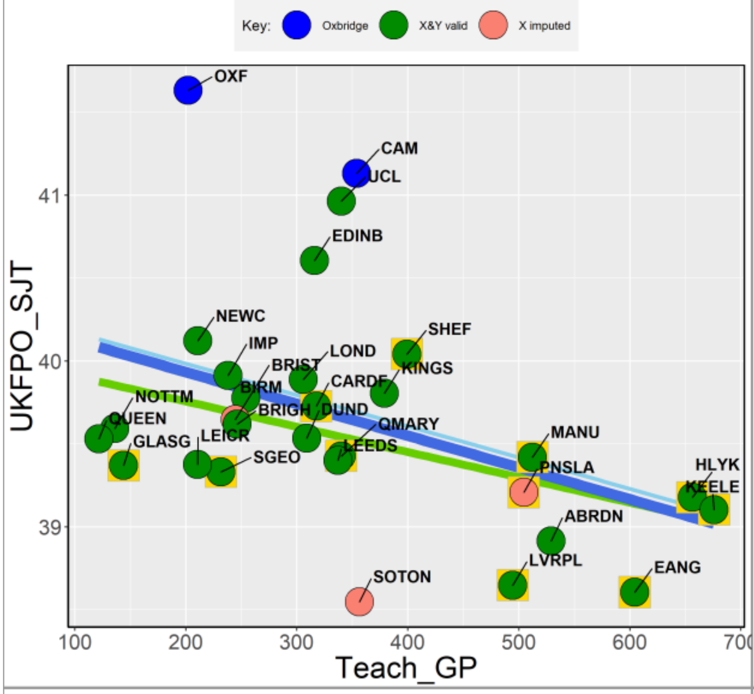

129/772 Y32: F1_Preparedness X20: Teach_GP
r(all)= 0.445 p= 0.0155 r(NonImp)= 0.402 Npairs=29 NimputedPairs=3

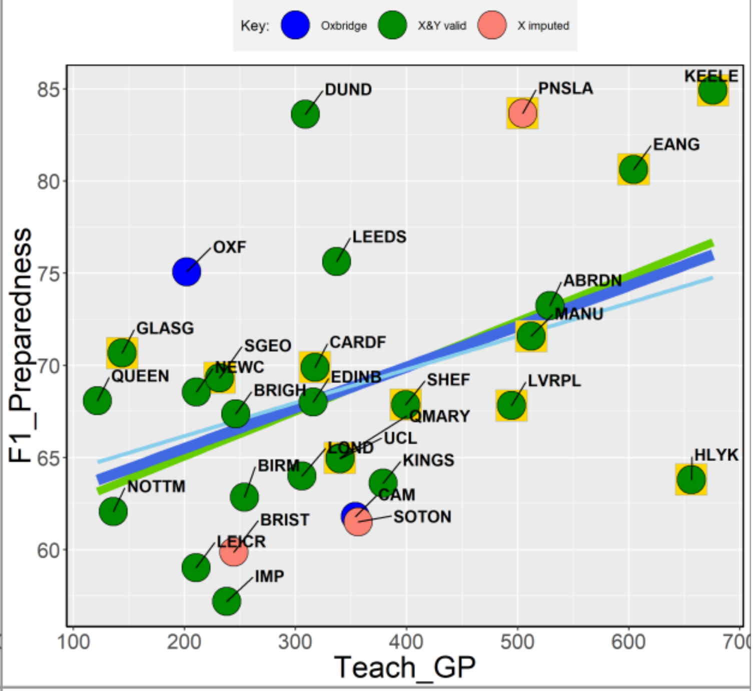

129/773 Y33: F1_Satisfn X20: Teach_GP
r(all)= 0.282 p= 0.139 r(NonImp)= 0.233 Npairs=29 NimputedPairs=3

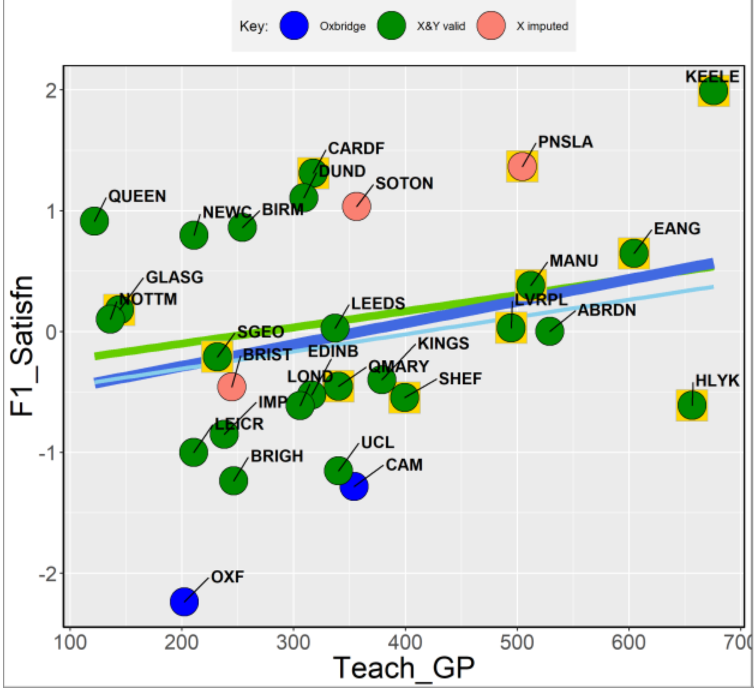

129/774 Y34: F1_Workload X20: Teach_GP
r(all)= 0.256 p= 0.18 r(NonImp)= 0.223 Npairs=29 NimputedPairs=3

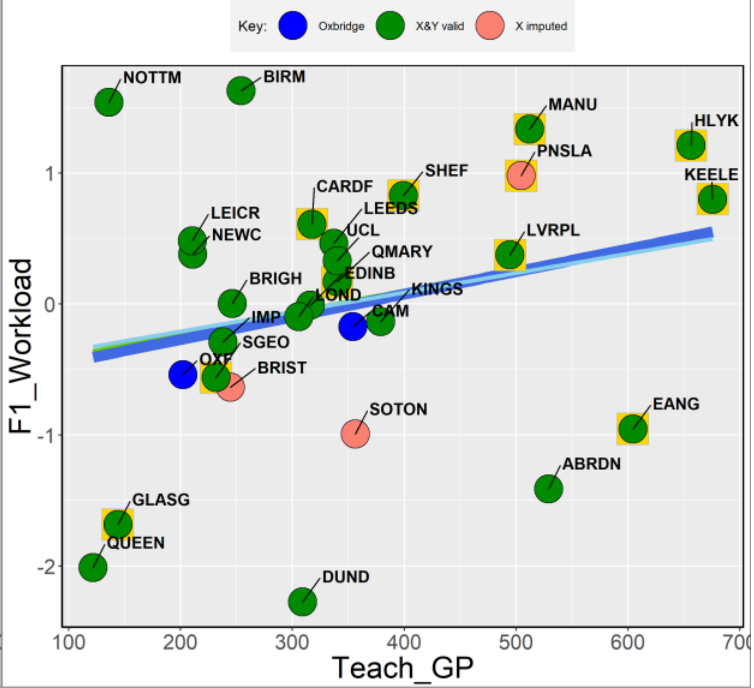

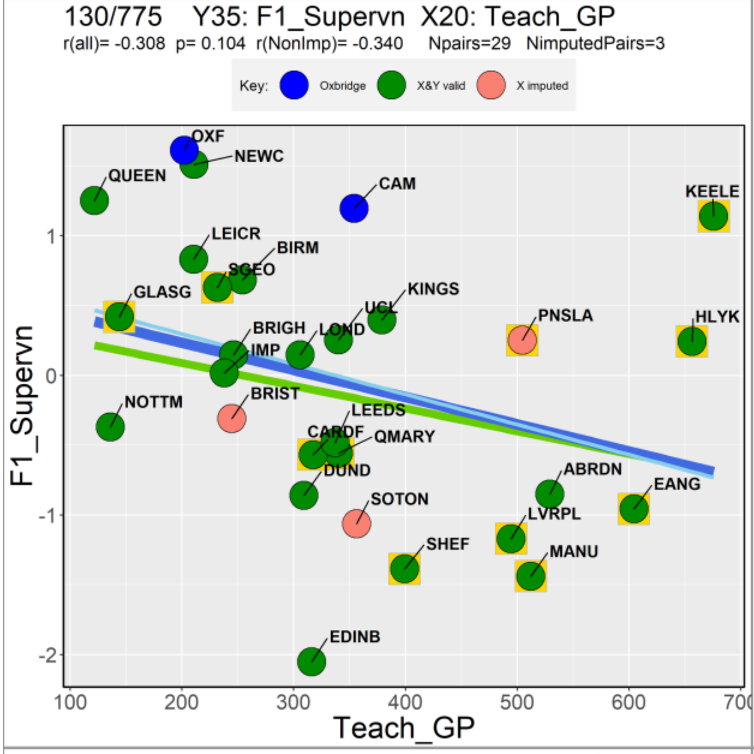
130/775 Y35: F1_Supervn X20: Teach_GP
r(all)= -0.308 p= 0.104 r(NonImp)= -0.340 Npairs=29 NimputedPairs=3
Key: Oxbridge X&Y valid X imputed
F1_Supervn
Teach_GP
1 0 -1 -2
100 200 300 400 500 600 700
OXF NEWC QUEEN CAM KEELE LEICR BIRM SGEO GLASG KINGS UCL PNSLA HLYK BRIGH LOND IMP NOTTM BRIST LEEDS CARDF QMARY DUND ABRDN EANG SOTON LVRPL SHEF MANU EDINB

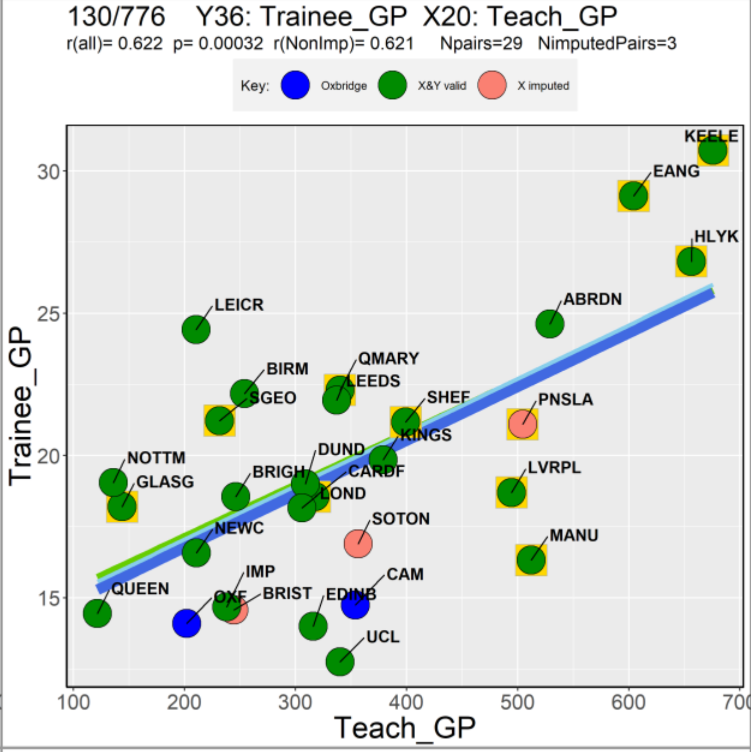
130/776 Y36: Trainee_GP X20: Teach_GP
r(all)= 0.622 p= 0.00032 r(NonImp)= 0.621 Npairs=29 NimputedPairs=3
Key: Oxbridge X&Y valid X imputed
Trainee_GP
Teach_GP
30 25 20 15
100 200 300 400 500 600 700
KEELE EANG HLYK ABRDN LEICR QMARY BIRM LEEDS SGEO SHEF PNSLA KINGS NOTTM DUND GLASG BRIGH CARDF LOND LVRPL SOTON NEWC MANU CAM QUEEN OXF BRIST EDINB UCL

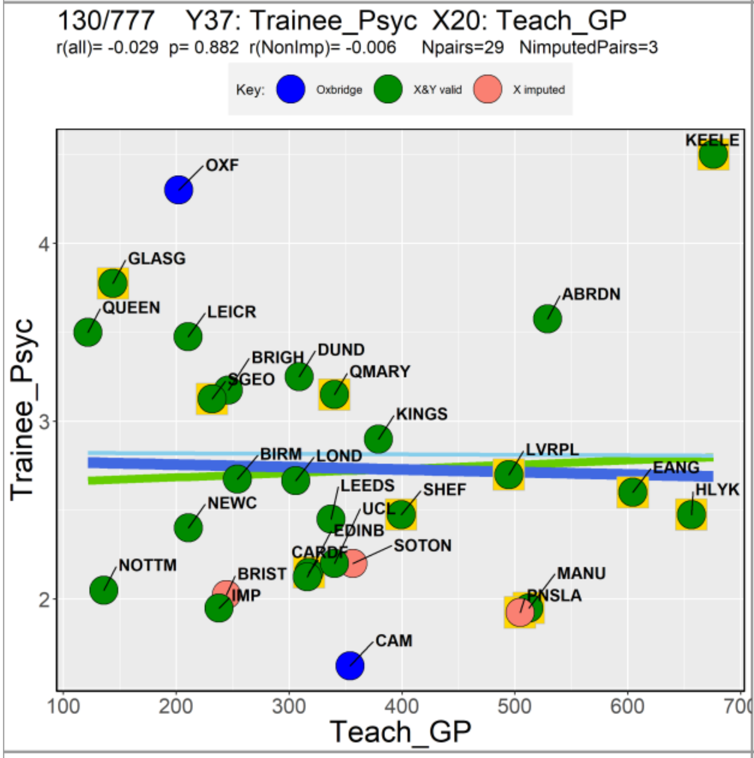
130/777 Y37: Trainee_Psyc X20: Teach_GP
r(all)= -0.029 p= 0.882 r(NonImp)= -0.006 Npairs=29 NimputedPairs=3
Key: Oxbridge X&Y valid X imputed
Trainee_Psyc
Teach_GP
4 3 2
100 200 300 400 500 600 700
KEELE OXF GLASG ABRDN QUEEN LEICR BRIGH DUND SGEO QMARY KINGS BIRM LOND LVRPL EANG LEEDS SHEF HLYK NEWC UCL EDINB CARDF SOTON NOTTM BRIST MANU IMP PNSLA CAM

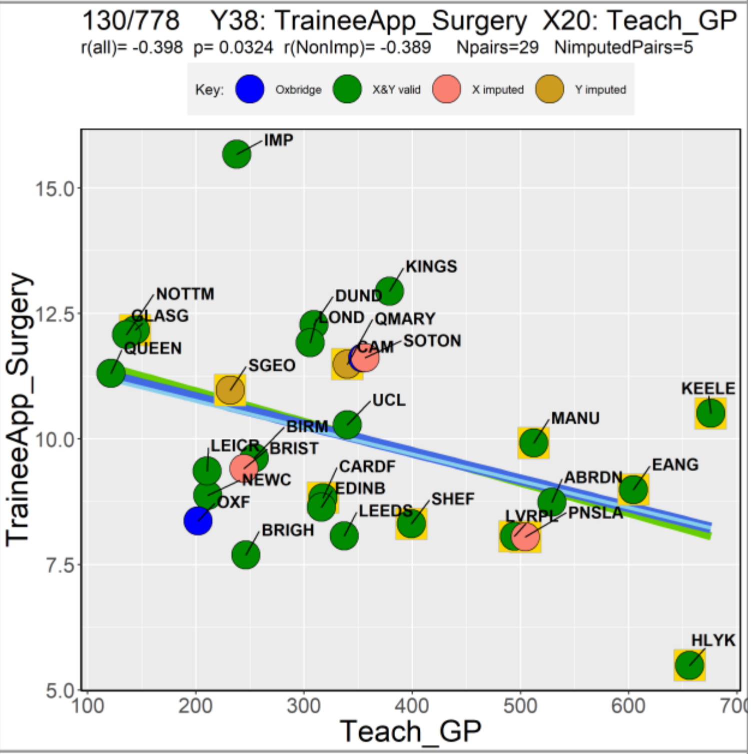
130/778 Y38: TraineeApp_Surgery X20: Teach_GP
r(all)= -0.398 p= 0.0324 r(NonImp)= -0.389 Npairs=29 NimputedPairs=5
Key: Oxbridge X&Y valid X imputed Y imputed
TraineeApp_Surgery
Teach_GP
15.0 12.5 10.0 7.5 5.0
100 200 300 400 500 600 700
IMP KINGS NOTTM DUND GLASG LOND QMARY CAM SOTON QUEEN SGEO UCL KEELE BIRM MANU LEICR BRIST CARDF EANG ABRDN NEWC EDINB OXF LEEDS SHEF LVRPL PNSLA BRIGH HLYK

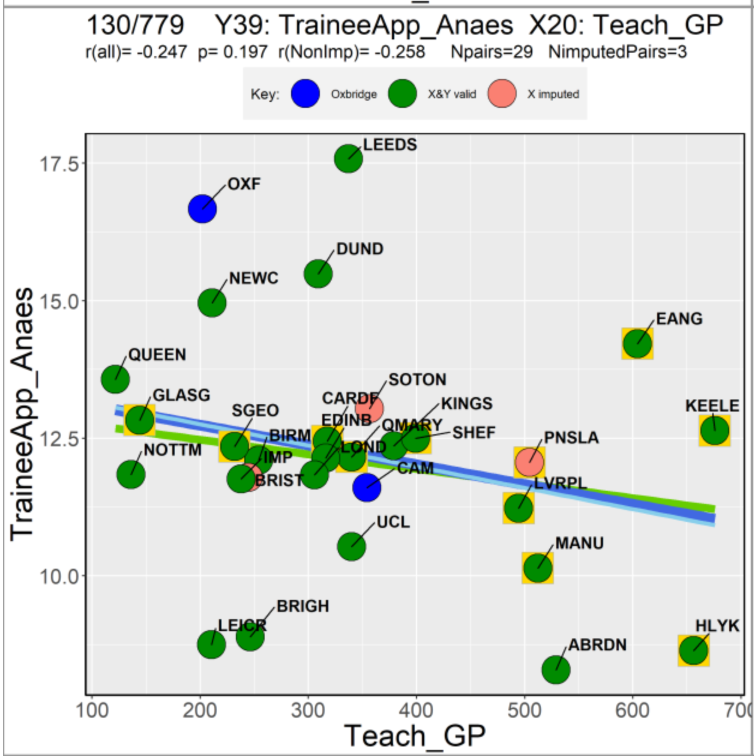
130/779 Y39: TraineeApp_Anaes X20: Teach_GP
r(all)= -0.247 p= 0.197 r(NonImp)= -0.258 Npairs=29 NimputedPairs=3
Key: Oxbridge X&Y valid X imputed
TraineeApp_Anaes
Teach_GP
17.5 15.0 12.5 10.0
100 200 300 400 500 600 700
LEEDS OXF DUND NEWC EANG QUEEN SOTON CARDF KINGS GLASG SGEO EDINB QMARY SHEF KEELE BIRM PNSLA NOTTM IMP LOND CAM BRIST LVRPL UCL MANU BRIGH LEICR ABRDN HLYK

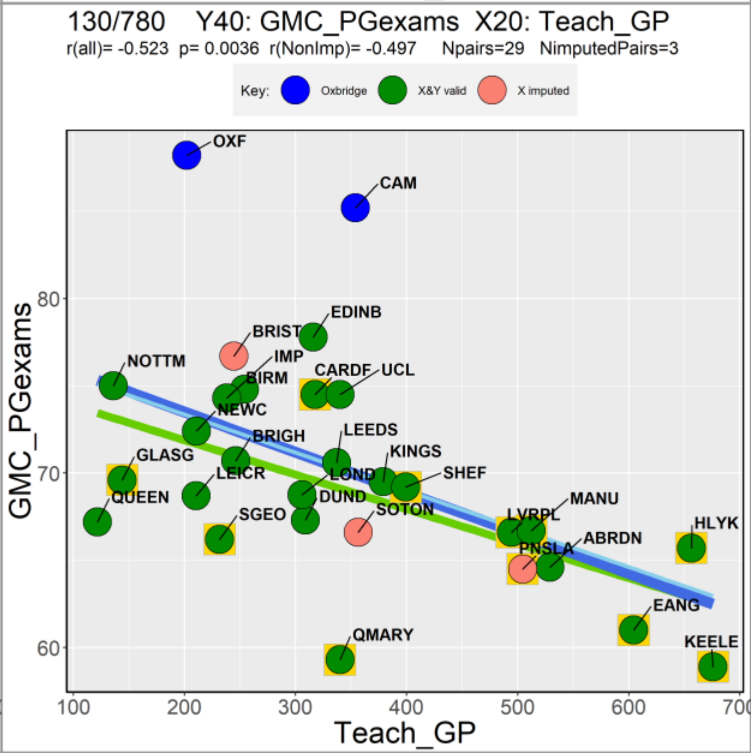
130/780 Y40: GMC_PGexams X20: Teach_GP
r(all)= -0.523 p= 0.0036 r(NonImp)= -0.497 Npairs=29 NimputedPairs=3
Key: Oxbridge X&Y valid X imputed
GMC_PGexams
Teach_GP
80 70 60
100 200 300 400 500 600 700
OXF CAM EDINB BRIST NOTTM IMP CARDF UCL BIRM NEWC BRIGH LEEDS GLASG KINGS LEICR LOND SHEF QUEEN DUND SOTON MANU SGEO LVRPL ABRDN HLYK PNSLA EANG QMARY KEELE

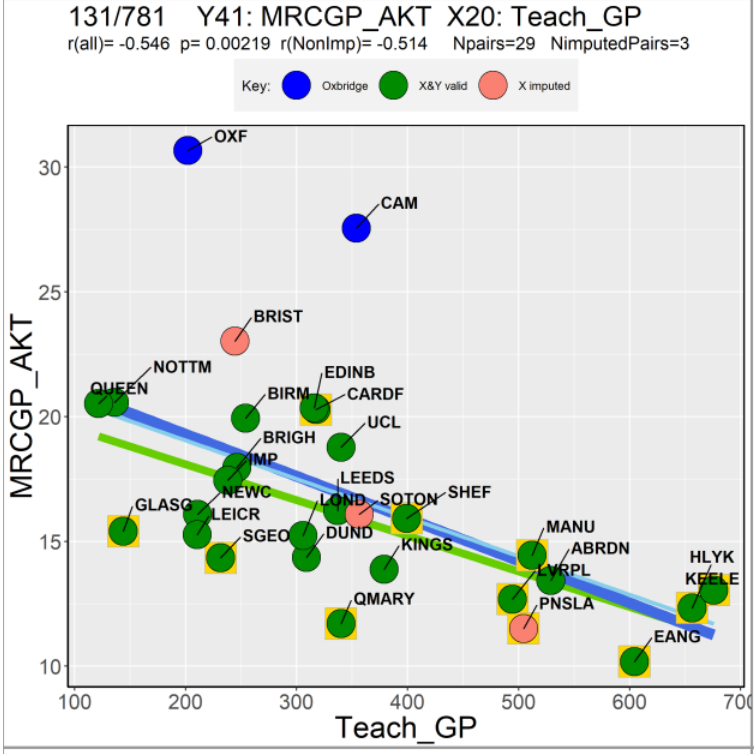
131/781 Y41: MRCGP_AKT X20: Teach_GP
r(all)= -0.546 p= 0.00219 r(NonImp)= -0.514 Npairs=29 NimputedPairs=3
Key: Oxbridge X&Y valid X imputed
MRCGP_AKT
Teach_GP

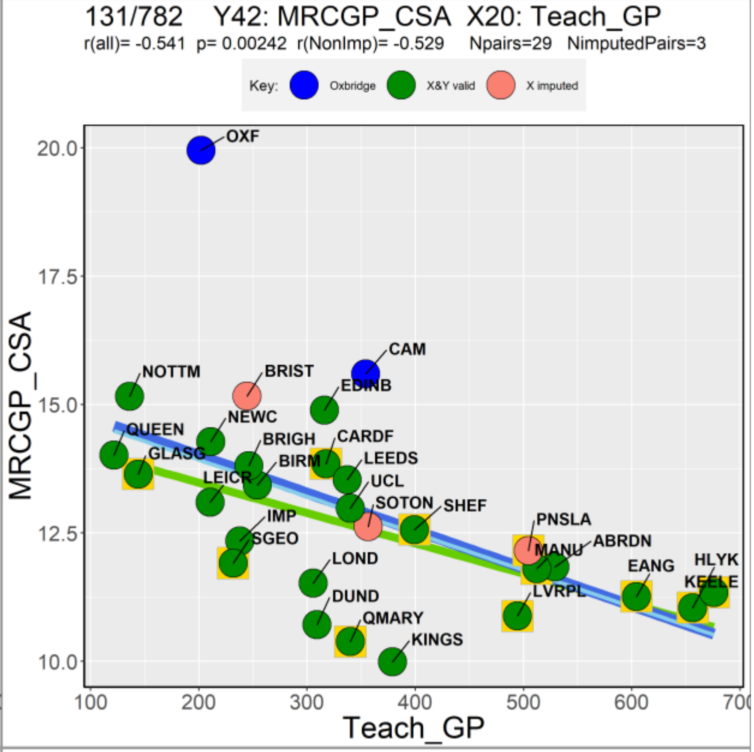
131/782 Y42: MRCGP_CSA X20: Teach_GP
r(all)= -0.541 p= 0.00242 r(NonImp)= -0.529 Npairs=29 NimputedPairs=3
Key: Oxbridge X&Y valid X imputed
MRCGP_CSA
Teach_GP

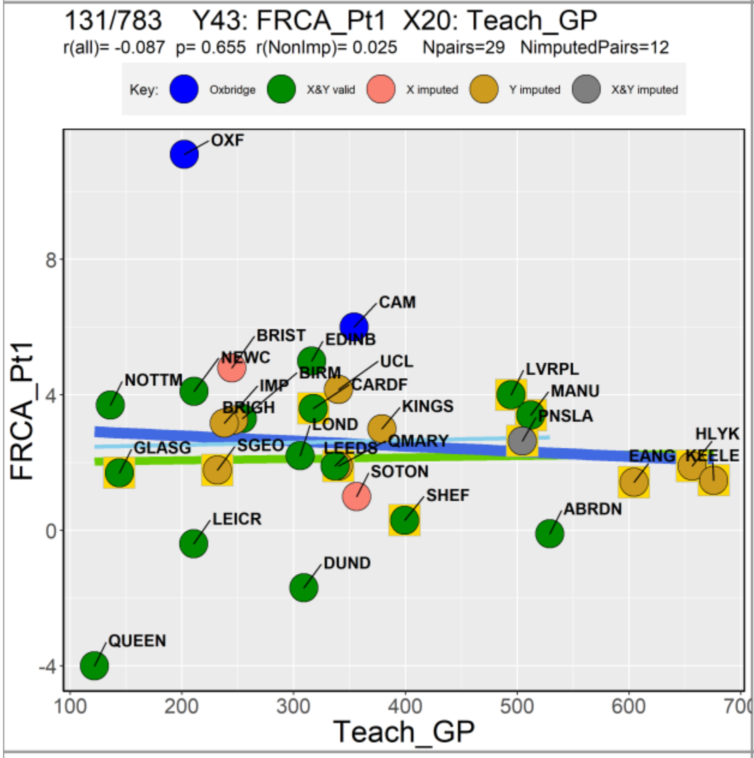
131/783 Y43: FRCA_Pt1 X20: Teach_GP
r(all)= -0.087 p= 0.655 r(NonImp)= 0.025 Npairs=29 NimputedPairs=12
Key: Oxbridge X&Y valid X imputed Y imputed X&Y imputed
FRCA_Pt1
Teach_GP

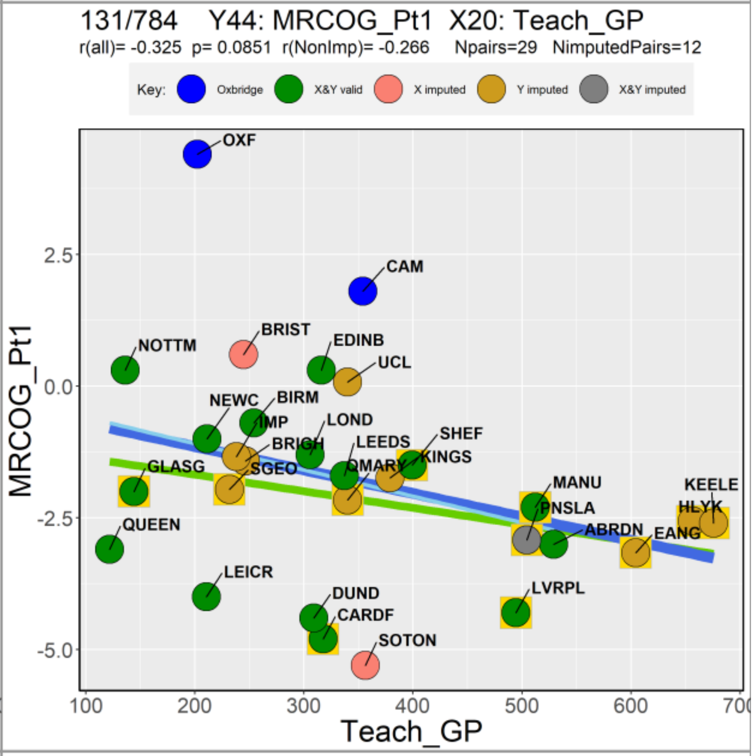
131/784 Y44: MRCOG_Pt1 X20: Teach_GP
r(all)= -0.325 p= 0.0851 r(NonImp)= -0.266 Npairs=29 NimputedPairs=12
Key: Oxbridge X&Y valid X imputed Y imputed X&Y imputed
MRCOG_Pt1
Teach_GP

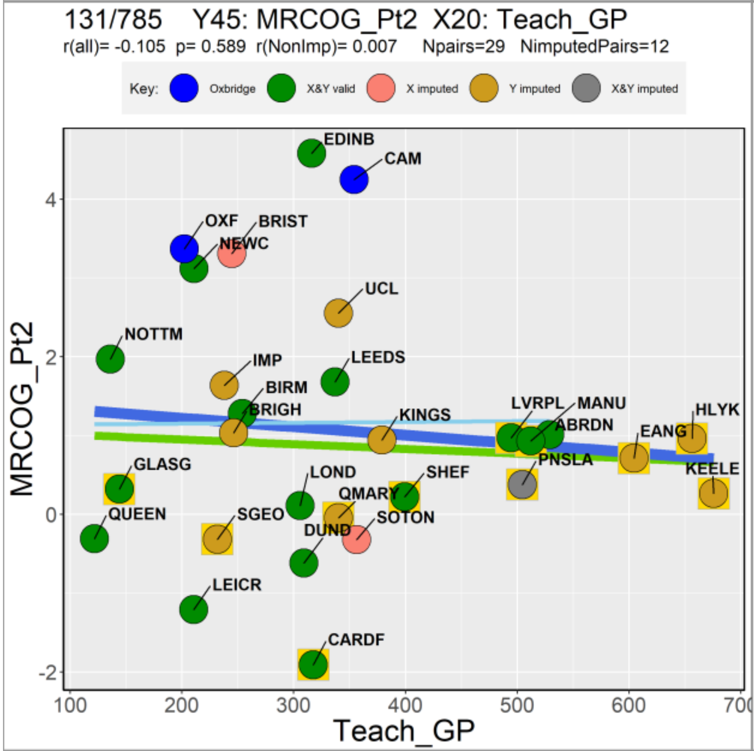
131/785 Y45: MRCOG_Pt2 X20: Teach_GP
r(all)= -0.105 p= 0.589 r(NonImp)= 0.007 Npairs=29 NimputedPairs=12
Key: Oxbridge X&Y valid X imputed Y imputed X&Y imputed
MRCOG_Pt2
Teach_GP

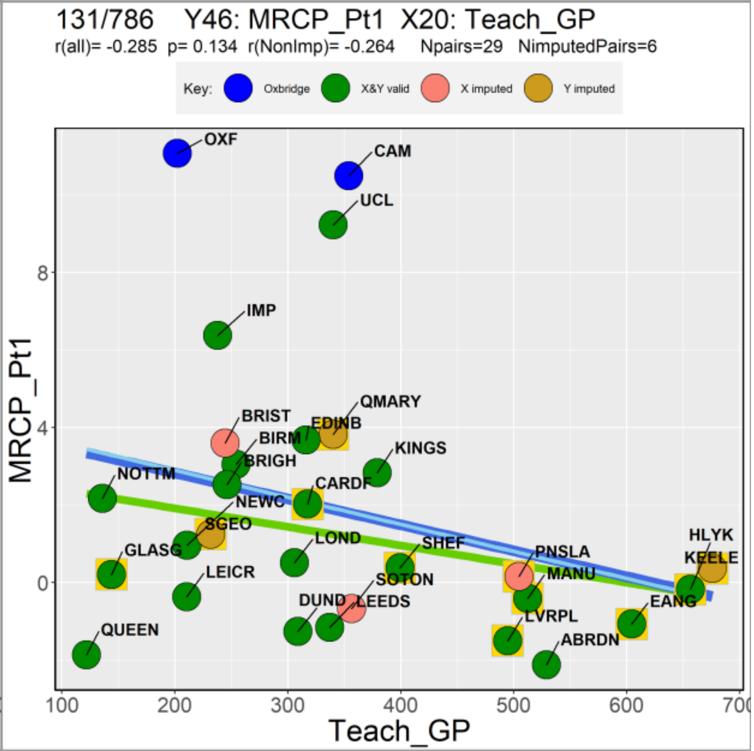
131/786 Y46: MRCP_Pt1 X20: Teach_GP
r(all)= -0.285 p= 0.134 r(NonImp)= -0.264 Npairs=29 NimputedPairs=6
Key: Oxbridge X&Y valid X imputed Y imputed
MRCP_Pt1
Teach_GP

132/787 Y47: MRCP_Pt2 X20: Teach_GP

r(all)= -0.176 p= 0.36 r(NonImp)= -0.161 Npairs=29 NimputedPairs=6

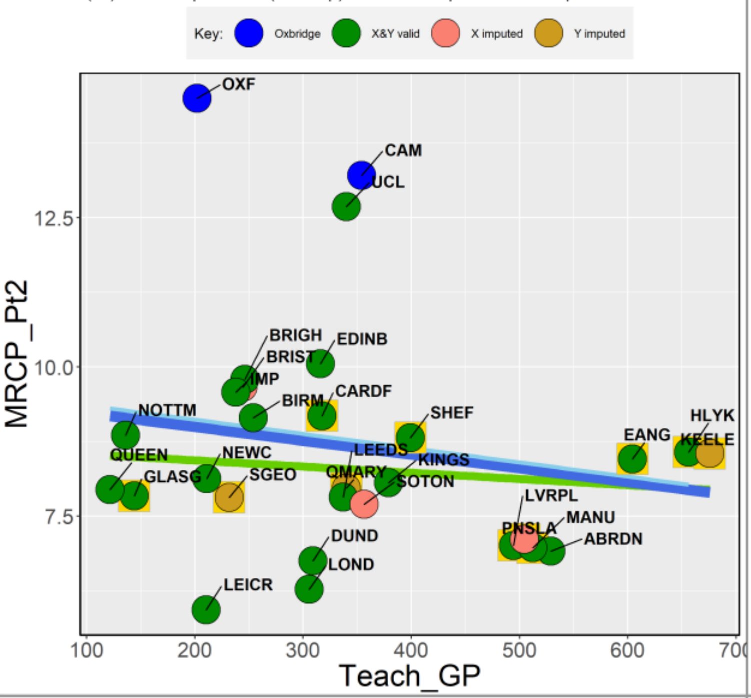

132/788 Y48: MRCP_PACES X20: Teach_GP

r(all)= -0.452 p= 0.0139 r(NonImp)= -0.443 Npairs=29 NimputedPairs=6

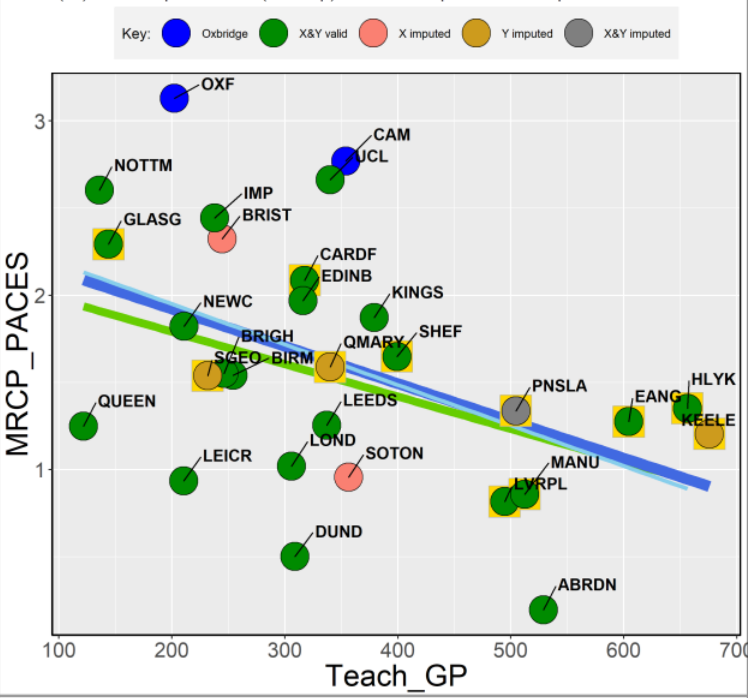

132/789 Y49: GMC_Sanctions X20: Teach_GP

r(all)= 0.647 p= 0.000148 r(NonImp)= 0.752 Npairs=29 NimputedPairs=12

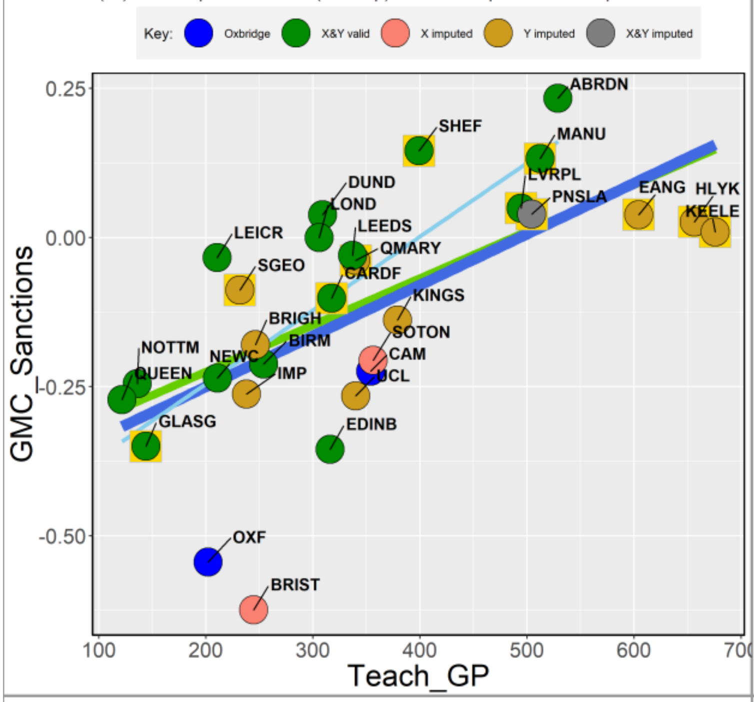

132/790 Y50: ARCP_NotExam X20: Teach_GP

r(all)= 0.446 p= 0.0152 r(NonImp)= 0.400 Npairs=29 NimputedPairs=4

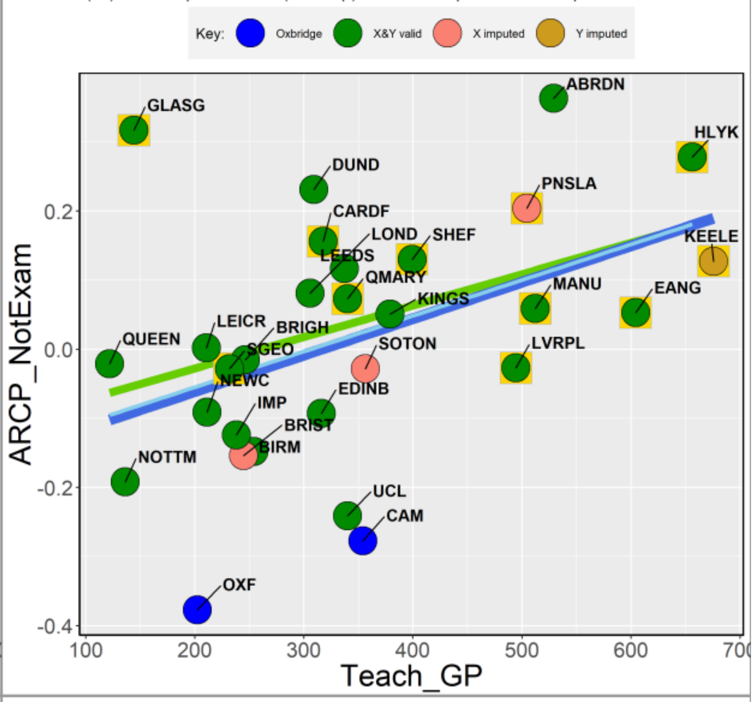

132/791 Y22: Teach_Anaes X21: Teach_Psyc

r(all)= -0.102 p= 0.6 r(NonImp)= -0.109 Npairs=29 NimputedPairs=3

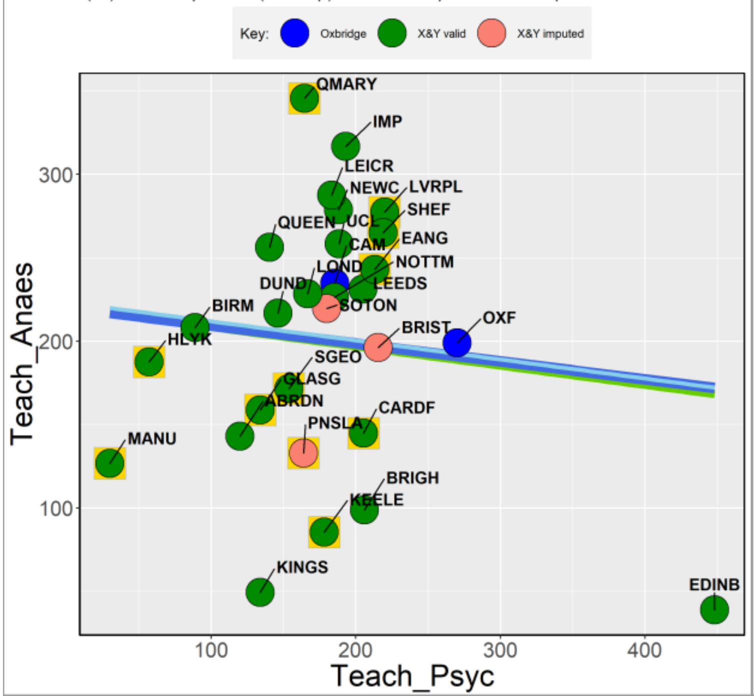

132/792 Y23: Teach_OG X21: Teach_Psyc

r(all)= 0.602 p= 0.000555 r(NonImp)= 0.598 Npairs=29 NimputedPairs=3

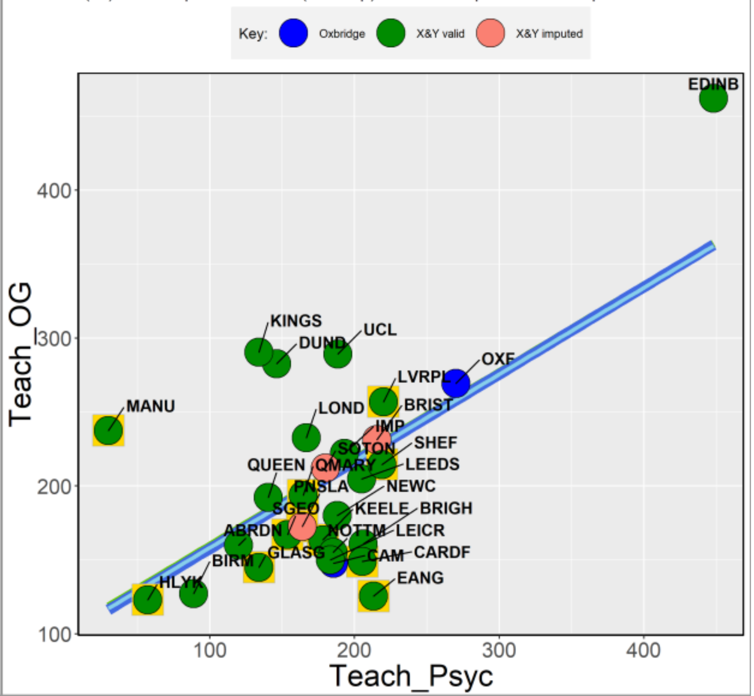

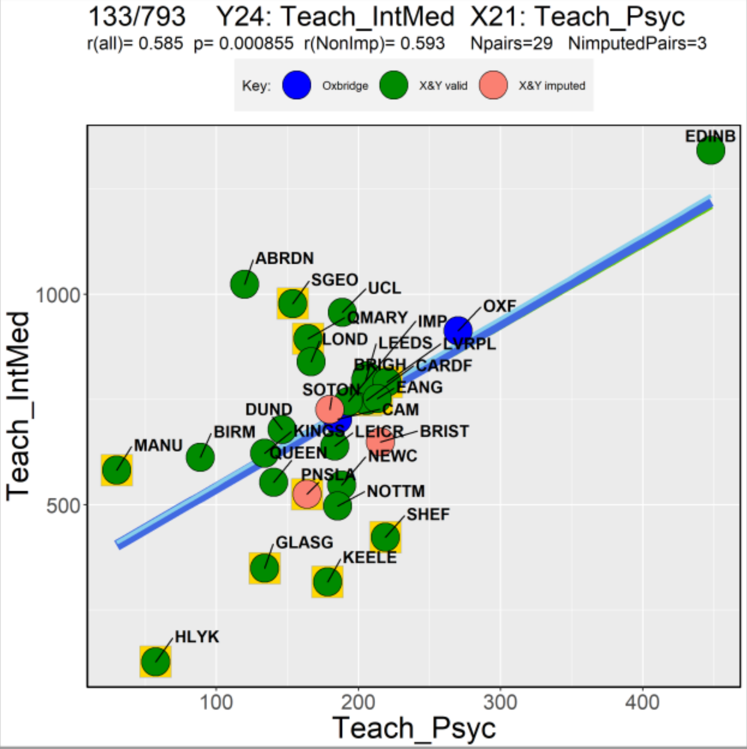
133/793 Y24: Teach_IntMed X21: Teach_Psyc
r(all)= 0.585 p= 0.000855 r(NonImp)= 0.593 Npairs=29 NimputedPairs=3
Key: Oxbridge X&Y valid X&Y imputed
Teach_IntMed
Teach_Psyc
1000
500
100
200
300
400
EDINB
ABRDN
SGEO
UCL
OXF
QMARY
IMP
LOND
LEEDS
LVRPL
BRIGH
CARDF
SOTON
EANG
DUND
CAM
MANU
BIRM
KINGS
LEICR
BRIST
QUEEN
NEWC
PNSLA
NOTTM
SHEF
GLASG
KEELE
HLYK

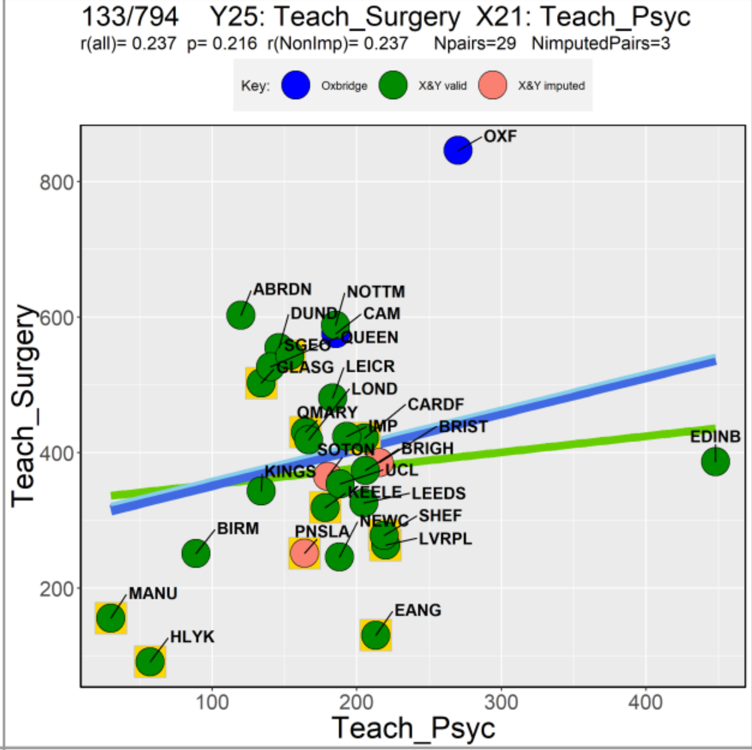
133/794 Y25: Teach_Surgery X21: Teach_Psyc
r(all)= 0.237 p= 0.216 r(NonImp)= 0.237 Npairs=29 NimputedPairs=3
Key: Oxbridge X&Y valid X&Y imputed
Teach_Surgery
Teach_Psyc
800
600
400
200
100
200
300
400
OXF
ABRDN
NOTTM
DUND
CAM
SGEO
QUEEN
GLASG
LEICR
LOND
CARDF
QMARY
IMP
BRIST
EDINB
SOTON
BRIGH
KINGS
UCL
KEELE
LEEDS
NEWC
SHEF
BIRM
PNSLA
LVRPL
MANU
EANG
HLYK

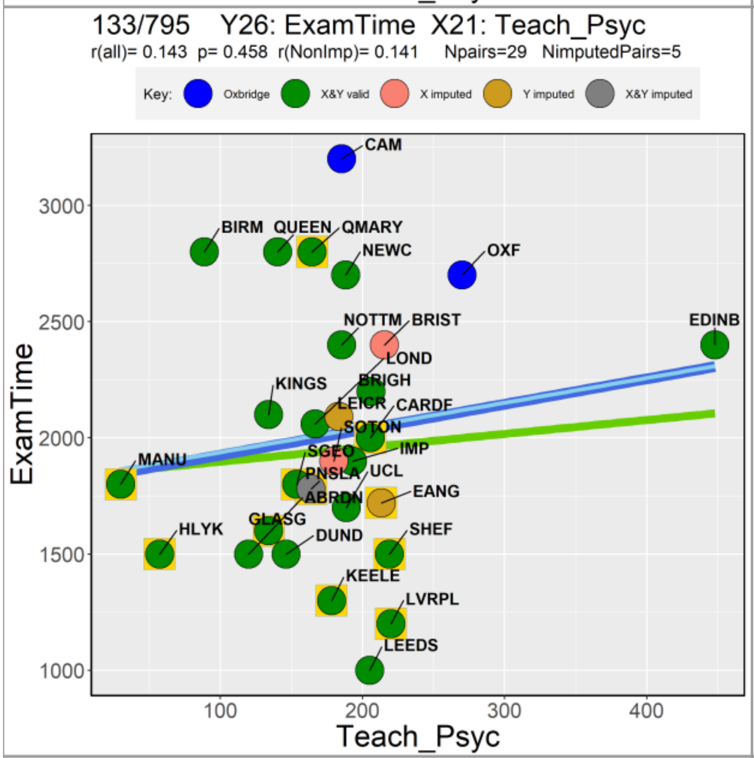
133/795 Y26: ExamTime X21: Teach_Psyc
r(all)= 0.143 p= 0.458 r(NonImp)= 0.141 Npairs=29 NimputedPairs=5
Key: Oxbridge X&Y valid X imputed Y imputed X&Y imputed
ExamTime
Teach_Psyc
3000
2500
2000
1500
1000
100
200
300
400
CAM
BIRM
QUEEN
QMARY
NEWC
OXF
NOTTM
BRIST
EDINB
LOND
KINGS
BRIGH
LEICR
CARDF
SOTON
MANU
SGEO
IMP
PNSLA
UCL
ABRDN
EANG
GLASG
HLYK
DUND
SHEF
KEELE
LVRPL
LEEDS

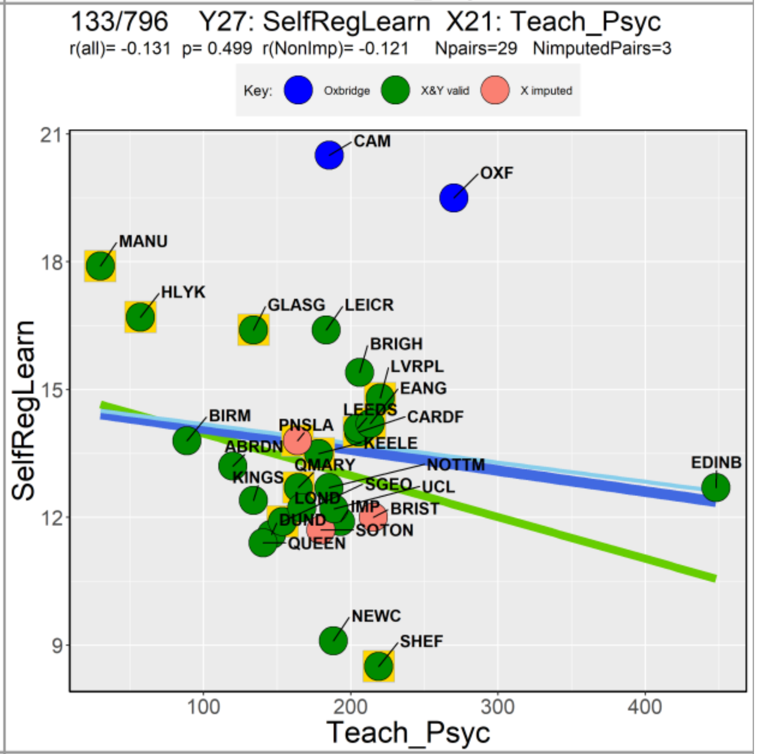
133/796 Y27: SelfRegLearn X21: Teach_Psyc
r(all)= -0.131 p= 0.499 r(NonImp)= -0.121 Npairs=29 NimputedPairs=3
Key: Oxbridge X&Y valid X imputed
SelfRegLearn
Teach_Psyc
21
18
15
12
9
100
200
300
400
CAM
OXF
MANU
HLYK
GLASG
LEICR
BRIGH
LVRPL
EANG
BIRM
PNSLA
LEEDS
CARDF
KEELE
ABRDN
QMARY
NOTTM
EDINB
KINGS
SGEO
UCL
LOND
IMP
BRIST
DUND
SOTON
QUEEN
NEWC
SHEF

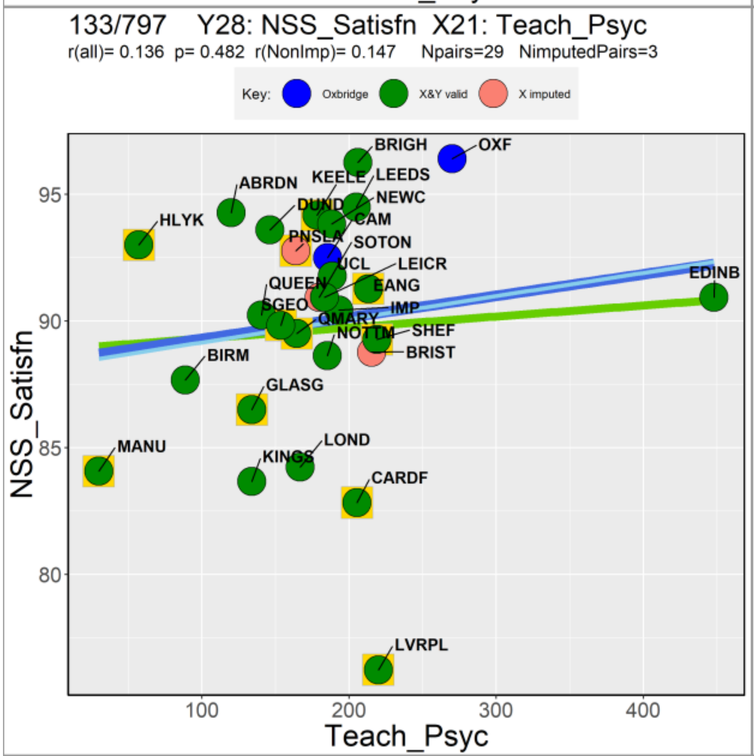
133/797 Y28: NSS_Satisfn X21: Teach_Psyc
r(all)= 0.136 p= 0.482 r(NonImp)= 0.147 Npairs=29 NimputedPairs=3
Key: Oxbridge X&Y valid X imputed
NSS_Satisfn
Teach_Psyc
95
90
85
80
100
200
300
400
BRIGH
OXF
ABRDN
KEELE
LEEDS
DUND
NEWC
HLYK
CAM
PNSLA
SOTON
UCL
LEICR
QUEEN
EANG
EDINB
SGEO
IMP
QMARY
SHEF
NOTTM
BIRM
BRIST
GLASG
MANU
LOND
KINGS
CARDF
LVRPL

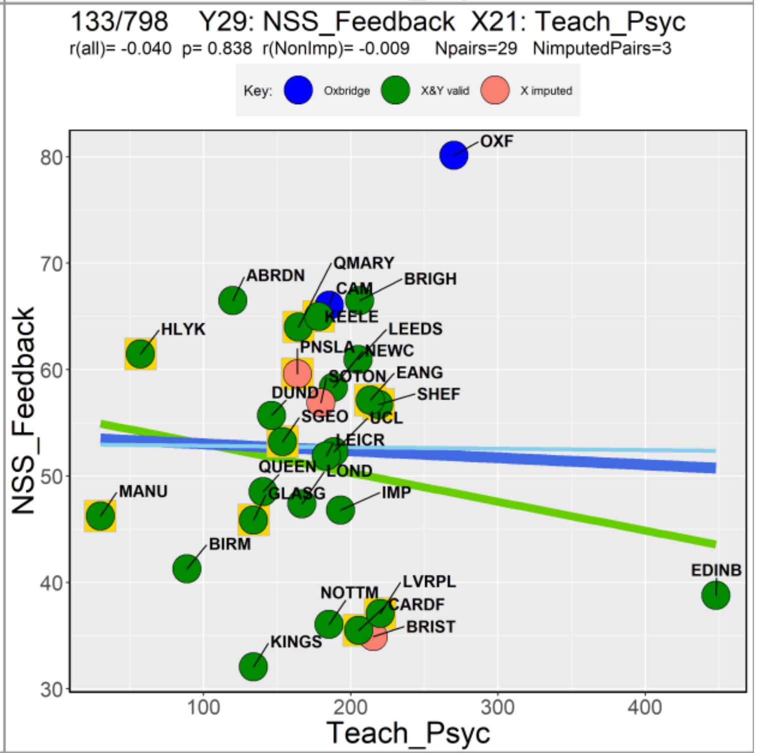
133/798 Y29: NSS_Feedback X21: Teach_Psyc
r(all)= -0.040 p= 0.838 r(NonImp)= -0.009 Npairs=29 NimputedPairs=3
Key: Oxbridge X&Y valid X imputed
NSS_Feedback
Teach_Psyc
80
70
60
50
40
30
100
200
300
400
OXF
QMARY
ABRDN
BRIGH
CAM
KEELE
HLYK
LEEDS
PNSLA
NEWC
SOTON
EANG
DUND
SHEF
SGEO
UCL
LEICR
QUEEN
LOND
MANU
GLASG
IMP
BIRM
EDINB
LVRPL
NOTTM
CARDF
BRIST
KINGS

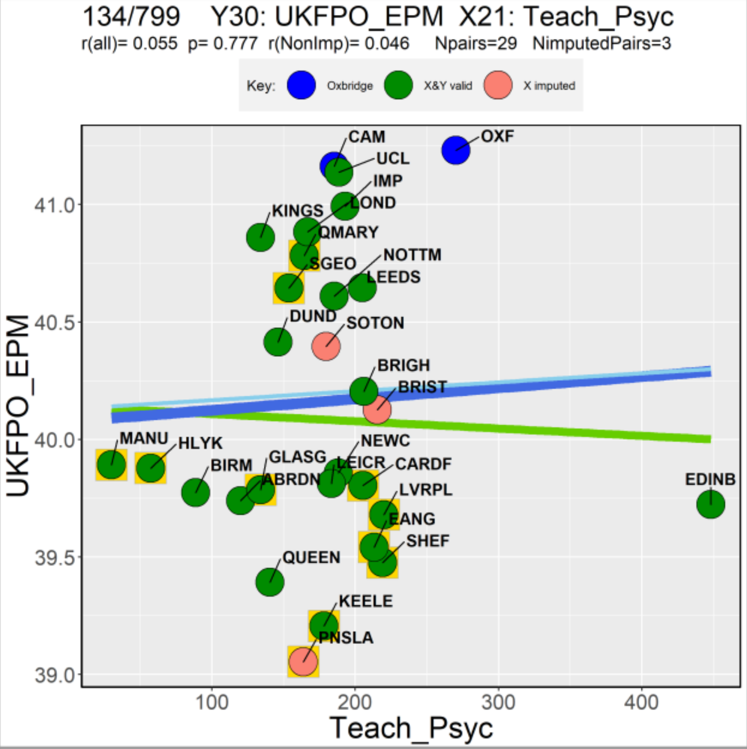
134/799 Y30: UKFPO_EPM X21: Teach_Psyc
r(all)= 0.055 p= 0.777 r(NonImp)= 0.046 Npairs=29 NimputedPairs=3
Key: Oxbridge X&Y valid X imputed
UKFPO_EPM
Teach_Psyc
CAM OXF UCL IMP KINGS LOND QMARY SGEO NOTTM LEEDS DUND SOTON BRIGH BRIST MANU HLYK NEWC GLASG LEICR CARDF BIRM ABRDN LVRPL EDINB EANG SHEF QUEEN KEELE PNSLA

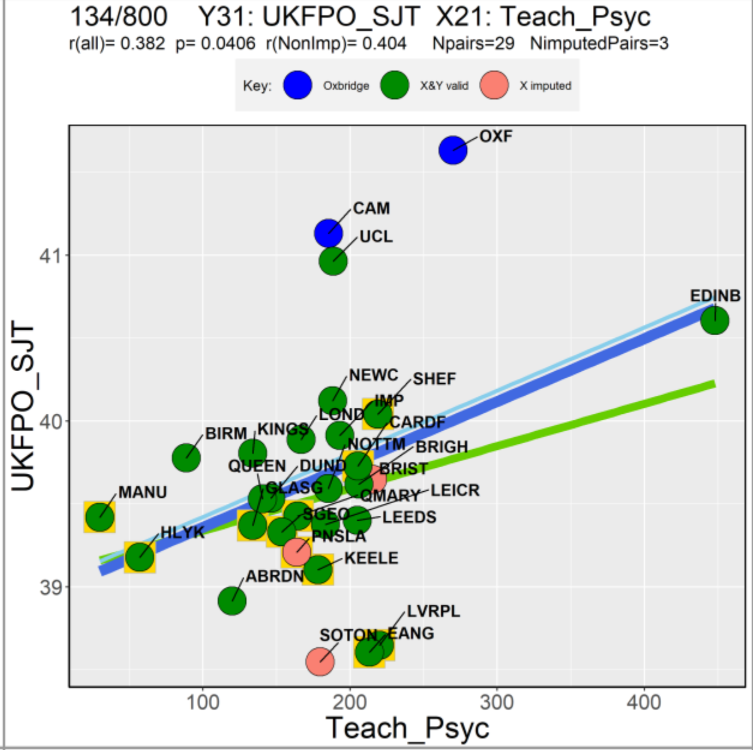
134/800 Y31: UKFPO_SJT X21: Teach_Psyc
r(all)= 0.382 p= 0.0406 r(NonImp)= 0.404 Npairs=29 NimputedPairs=3
Key: Oxbridge X&Y valid X imputed
UKFPO_SJT
Teach_Psyc
OXF CAM UCL EDINB NEWC SHEF IMP LOND CARDF BIRM KINGS NOTTM BRIGH QUEEN DUND BRIST MANU GLASG QMARY LEICR SGEO LEEDS HLYK PNSLA KEELE ABRDN LVRPL SOTON EANG

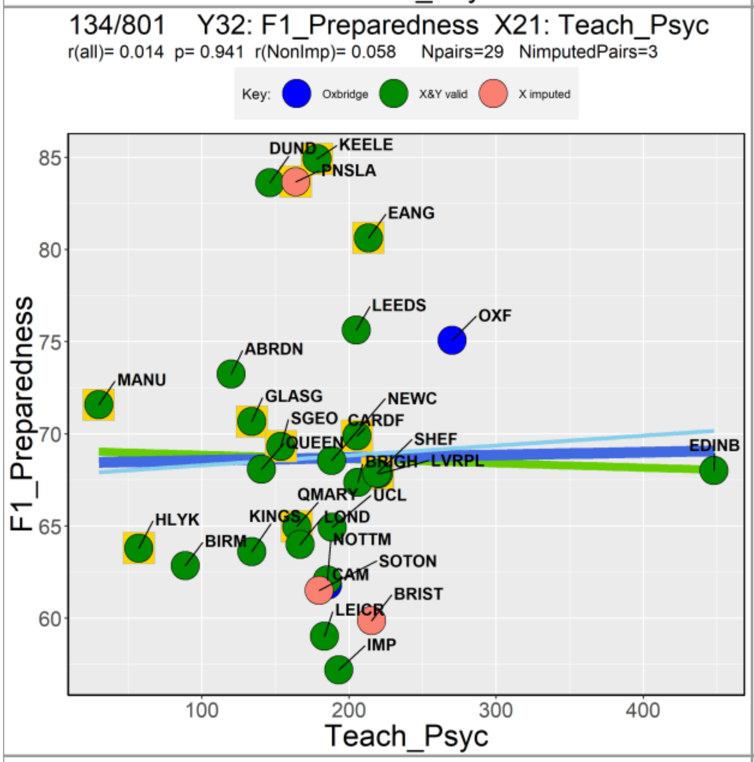
134/801 Y32: F1_Preparedness X21: Teach_Psyc
r(all)= 0.014 p= 0.941 r(NonImp)= 0.058 Npairs=29 NimputedPairs=3
Key: Oxbridge X&Y valid X imputed
F1_Preparedness
Teach_Psyc
DUND KEELE PNSLA EANG LEEDS OXF ABRDN MANU GLASG NEWC SGEO CARDF SHEF QUEEN EDINB BRIGH LVRPL QMARY UCL HLYK KINGS LOND BIRM NOTTM SOTON CAM BRIST LEICR IMP

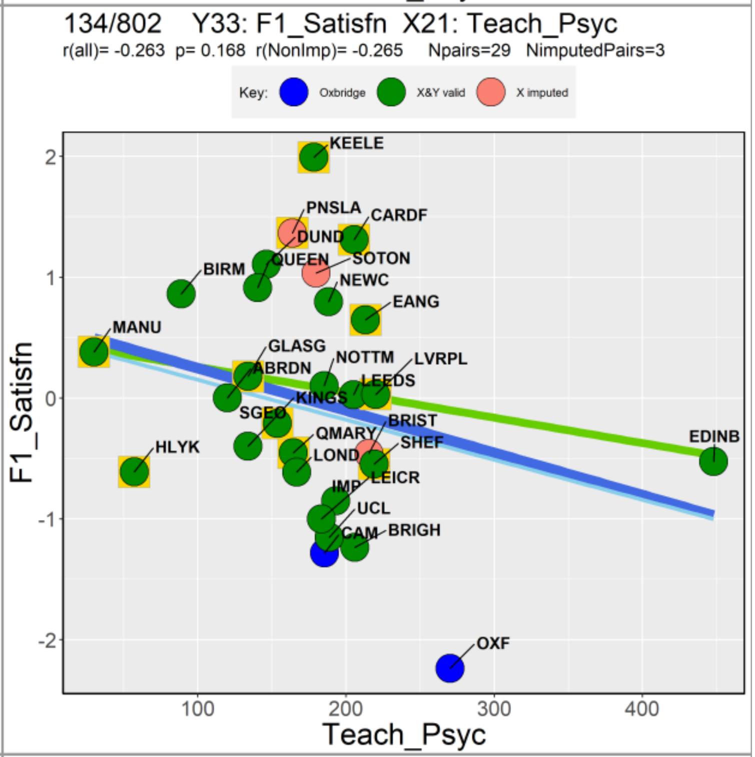
134/802 Y33: F1_Satisfn X21: Teach_Psyc
r(all)= -0.263 p= 0.168 r(NonImp)= -0.265 Npairs=29 NimputedPairs=3
Key: Oxbridge X&Y valid X imputed
F1_Satisfn
Teach_Psyc
KEELE PNSLA CARDF DUND BIRM QUEEN SOTON NEWC EANG MANU GLASG NOTTM LVRPL ABRDN LEEDS KINGS SGEO BRIST EDINB QMARY HLYK LOND SHEF IMP LEICR UCL CAM BRIGH OXF

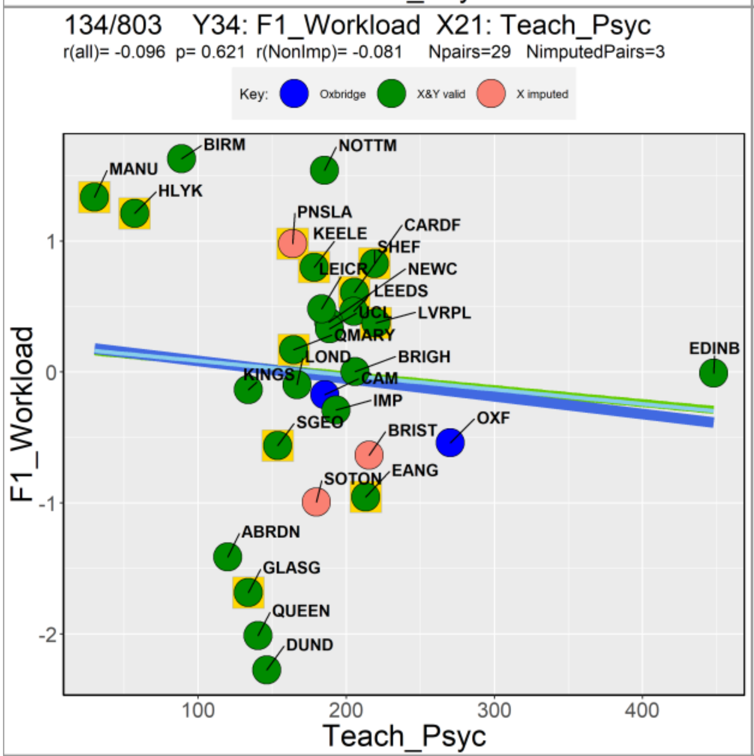
134/803 Y34: F1_Workload X21: Teach_Psyc
r(all)= -0.096 p= 0.621 r(NonImp)= -0.081 Npairs=29 NimputedPairs=3
Key: Oxbridge X&Y valid X imputed
F1_Workload
Teach_Psyc
BIRM NOTTM MANU HLYK PNSLA CARDF KEELE SHEF LEICR NEWC LEEDS UCL LVRPL QMARY EDINB LOND BRIGH KINGS CAM IMP SGEO BRIST OXF SOTON EANG ABRDN GLASG QUEEN DUND

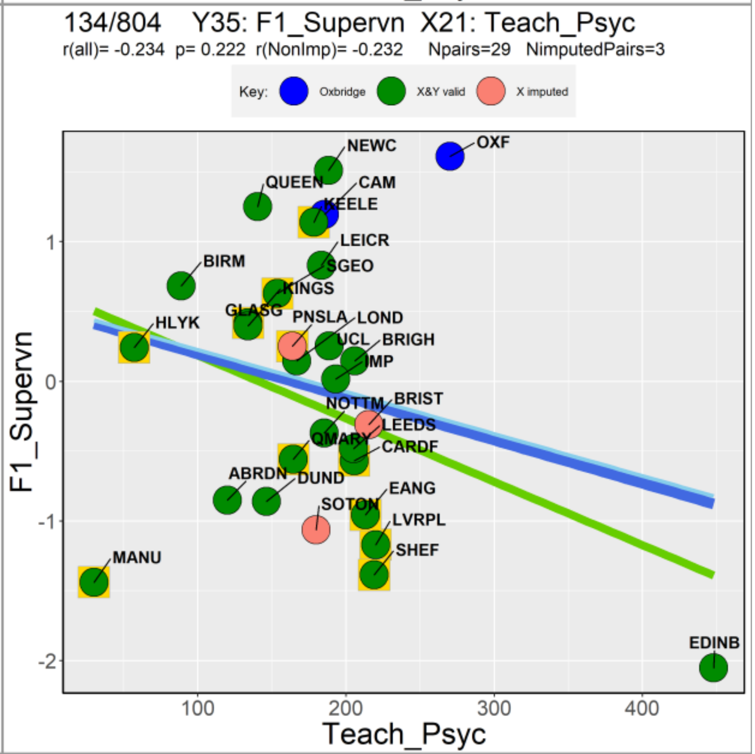
134/804 Y35: F1_Supervn X21: Teach_Psyc
r(all)= -0.234 p= 0.222 r(NonImp)= -0.232 Npairs=29 NimputedPairs=3
Key: Oxbridge X&Y valid X imputed
F1_Supervn
Teach_Psyc
NEWC OXF QUEEN CAM KEELE LEICR BIRM SGEO KINGS GLASG PNSLA LOND HLYK UCL BRIGH IMP NOTTM BRIST LEEDS QMARY CARDF ABRDN DUND EANG SOTON LVRPL SHEF MANU EDINB

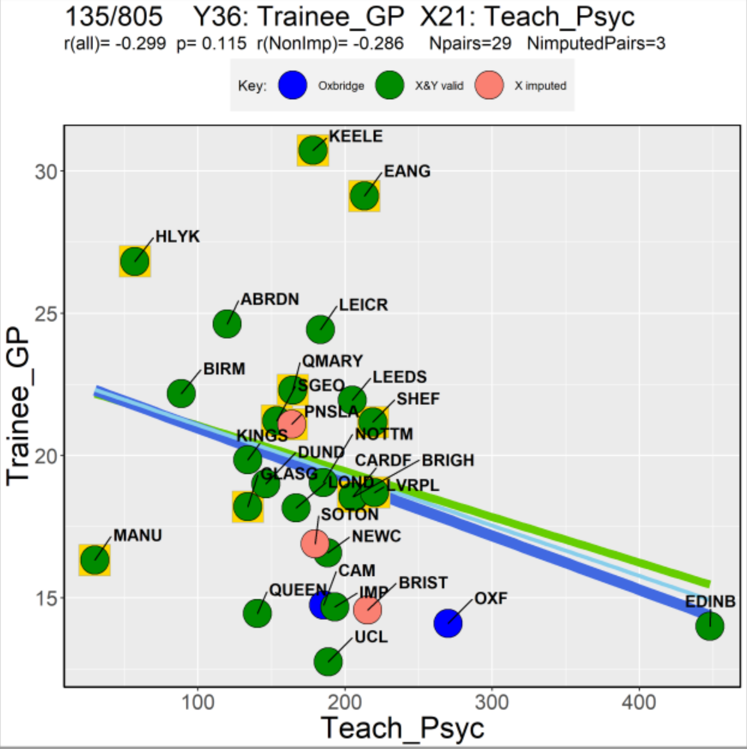
135/805 Y36: Trainee_GP X21: Teach_Psyc
r(all)= -0.299 p= 0.115 r(NonImp)= -0.286 Npairs=29 NimputedPairs=3
Key: Oxbridge X&Y valid X imputed
Trainee_GP
Teach_Psyc
KEELE
EANG
HLYK
ABRDN
LEICR
BIRM
QMARY
SGEO
LEEDS
PNSLA
SHEF
KINGS
NOTTM
DUND
CARDF
BRIGH
GLASG
LOND
LVRPL
SOTON
MANU
NEWC
CAM
QUEEN
IMP
BRIST
OXF
EDINB
UCL

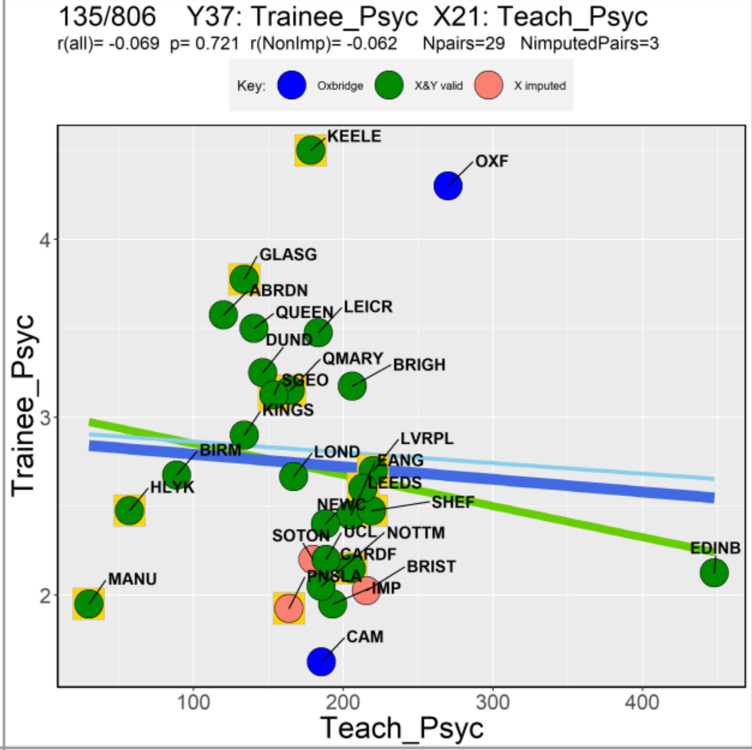
135/806 Y37: Trainee_Psyc X21: Teach_Psyc
r(all)= -0.069 p= 0.721 r(NonImp)= -0.062 Npairs=29 NimputedPairs=3
Key: Oxbridge X&Y valid X imputed
Trainee_Psyc
Teach_Psyc
KEELE
OXF
GLASG
ABRDN
QUEEN
LEICR
DUND
QMARY
BRIGH
SGEO
KINGS
BIRM
LOND
LVRPL
EANG
HLYK
LEEDS
NEWC
SHEF
SOTON
UCL
NOTTM
CARDF
BRIST
PNSLA
IMP
MANU
EDINB
CAM

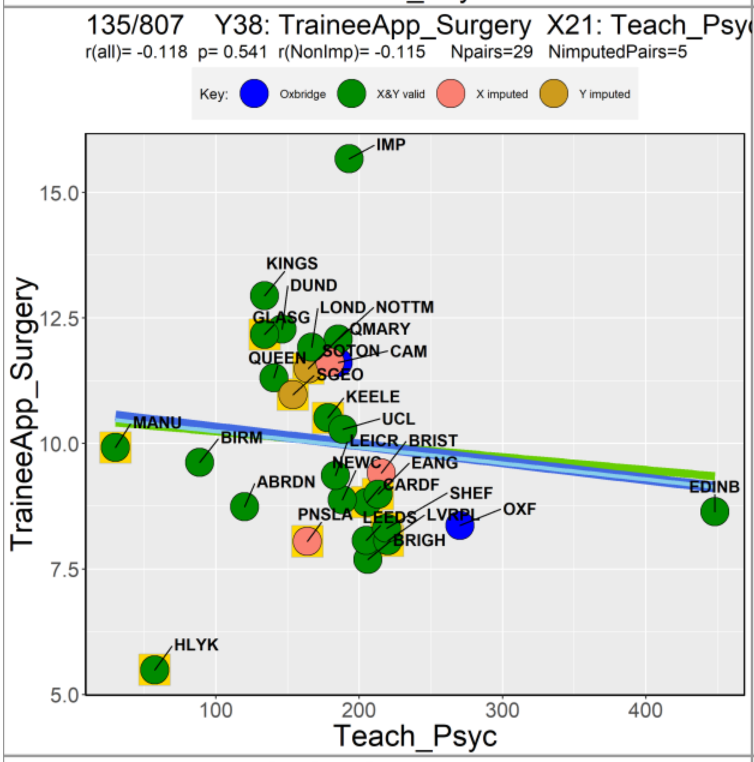
135/807 Y38: TraineeApp_Surgery X21: Teach_Psyc
r(all)= -0.118 p= 0.541 r(NonImp)= -0.115 Npairs=29 NimputedPairs=5
Key: Oxbridge X&Y valid X imputed Y imputed
TraineeApp_Surgery
Teach_Psyc
IMP
KINGS
DUND
LOND
NOTTM
GLASG
QMARY
QUEEN
SOTON
CAM
SGEO
KEELE
MANU
UCL
BIRM
LEICR
BRIST
NEWC
EANG
ABRDN
CARDF
SHEF
EDINB
PNSLA
LEEDS
LVRPL
OXF
BRIGH
HLYK

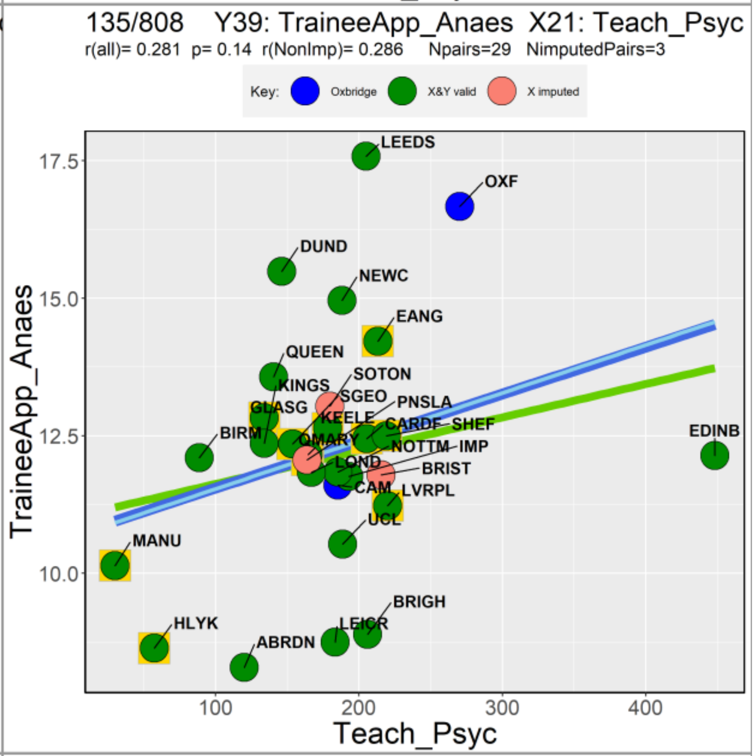
135/808 Y39: TraineeApp_Anaes X21: Teach_Psyc
r(all)= 0.281 p= 0.14 r(NonImp)= 0.286 Npairs=29 NimputedPairs=3
Key: Oxbridge X&Y valid X imputed
TraineeApp_Anaes
Teach_Psyc
LEEDS
OXF
DUND
NEWC
EANG
QUEEN
KINGS
SOTON
SGEO
GLASG
PNSLA
KEELE
CARDF
SHEF
BIRM
QMARY
NOTTM
IMP
EDINB
LOND
BRIST
CAM
LVRPL
UCL
MANU
BRIGH
HLYK
LEICR
ABRDN

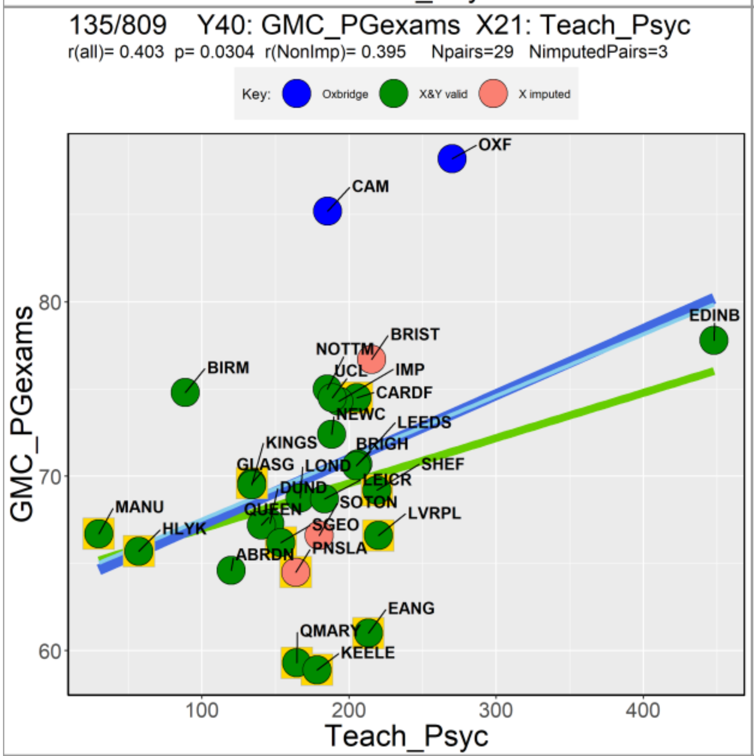
135/809 Y40: GMC_PGexams X21: Teach_Psyc
r(all)= 0.403 p= 0.0304 r(NonImp)= 0.395 Npairs=29 NimputedPairs=3
Key: Oxbridge X&Y valid X imputed
GMC_PGexams
Teach_Psyc
OXF
CAM
EDINB
NOTTM
BRIST
BIRM
UCL
IMP
CARDF
NEWC
LEEDS
KINGS
BRIGH
GLASG
LOND
SHEF
DUND
LEICR
QUEEN
SOTON
MANU
LVRPL
HLYK
SGEO
PNSLA
ABRDN
EANG
QMARY
KEELE

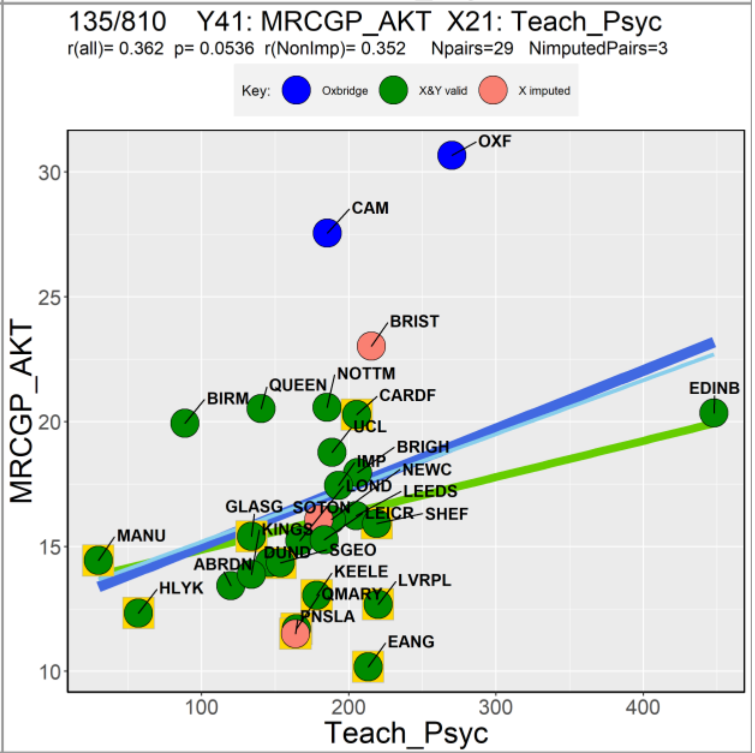
135/810 Y41: MRCGP_AKT X21: Teach_Psyc
r(all)= 0.362 p= 0.0536 r(NonImp)= 0.352 Npairs=29 NimputedPairs=3
Key: Oxbridge X&Y valid X imputed
MRCGP_AKT
Teach_Psyc
OXF
CAM
BRIST
QUEEN
NOTTM
BIRM
CARDF
EDINB
UCL
BRIGH
IMP
NEWC
LOND
LEEDS
GLASG
SOTON
LEICR
SHEF
KINGS
MANU
DUND
SGEO
ABRDN
KEELE
HLYK
QMARY
LVRPL
PNSLA
EANG

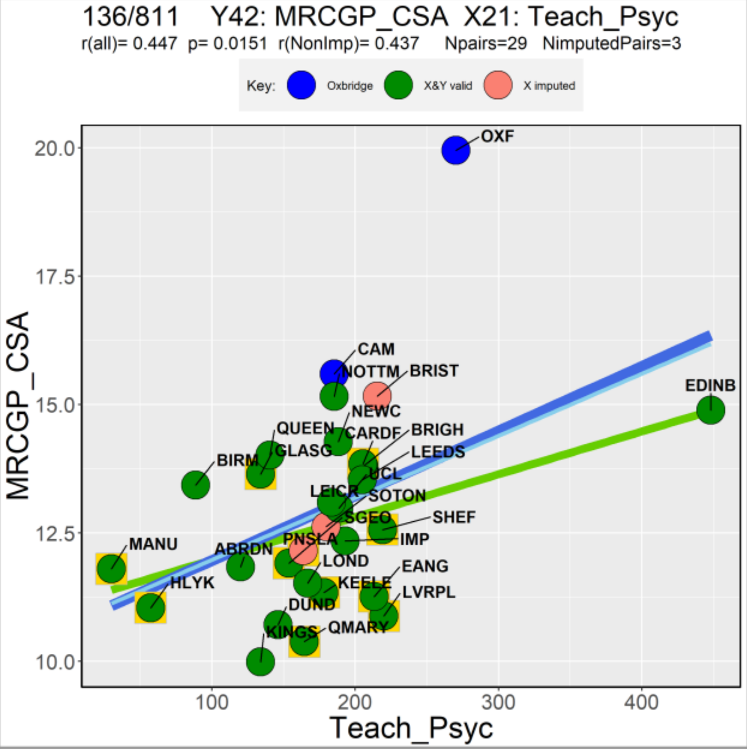
136/811 Y42: MRCGP_CSA X21: Teach_Psyc
r(all)= 0.447 p= 0.0151 r(NonImp)= 0.437 Npairs=29 NimputedPairs=3
Key: Oxbridge X&Y valid X imputed
MRCGP_CSA
Teach_Psyc

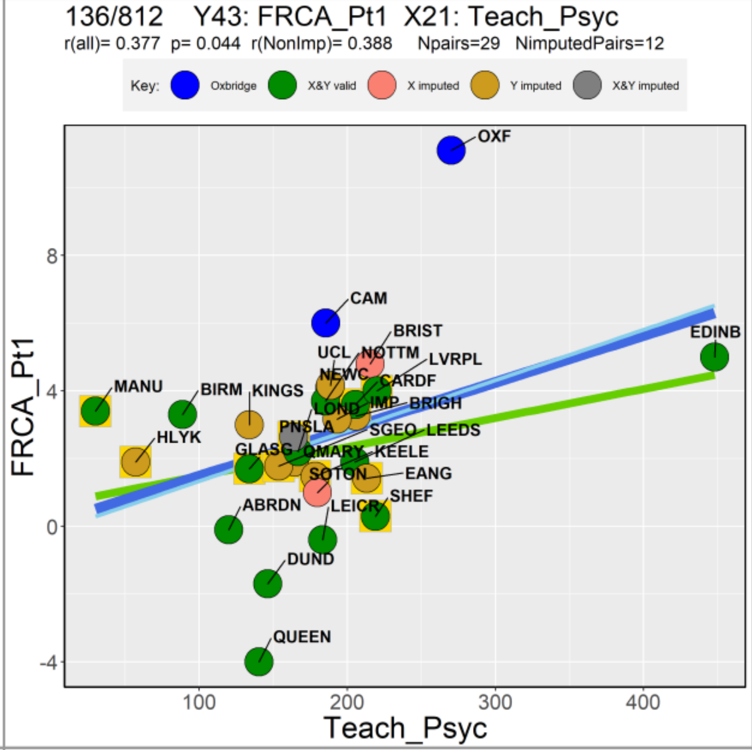
136/812 Y43: FRCA_Pt1 X21: Teach_Psyc
r(all)= 0.377 p= 0.044 r(NonImp)= 0.388 Npairs=29 NimputedPairs=12
Key: Oxbridge X&Y valid X imputed Y imputed X&Y imputed
FRCA_Pt1
Teach_Psyc

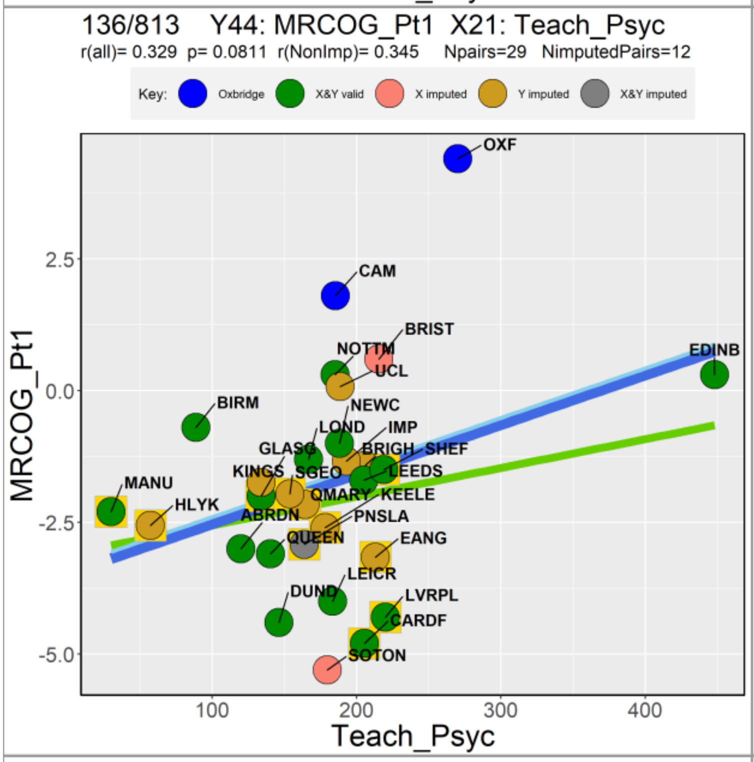
136/813 Y44: MRCOG_Pt1 X21: Teach_Psyc
r(all)= 0.329 p= 0.0811 r(NonImp)= 0.345 Npairs=29 NimputedPairs=12
Key: Oxbridge X&Y valid X imputed Y imputed X&Y imputed
MRCOG_Pt1
Teach_Psyc

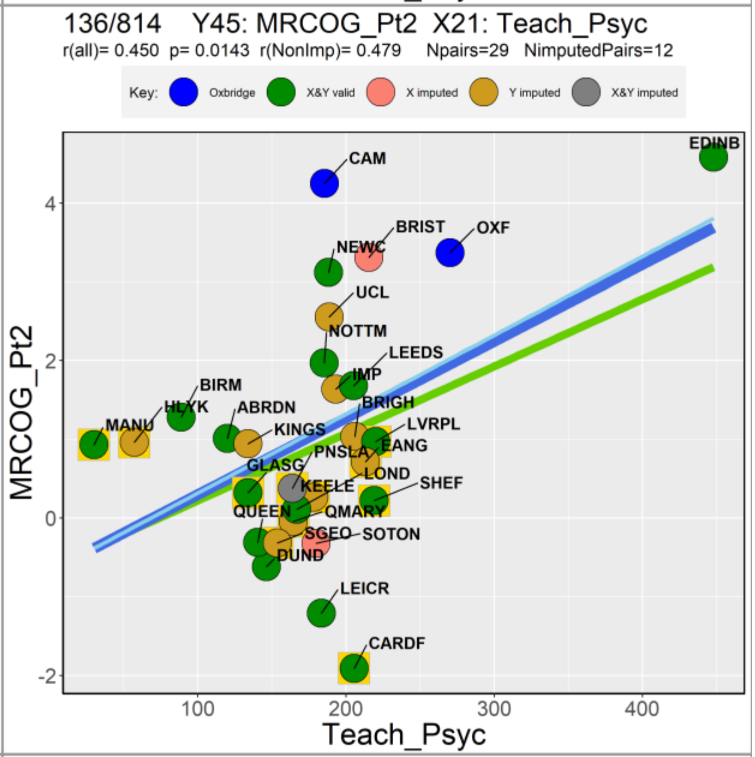
136/814 Y45: MRCOG_Pt2 X21: Teach_Psyc
r(all)= 0.450 p= 0.0143 r(NonImp)= 0.479 Npairs=29 NimputedPairs=12
Key: Oxbridge X&Y valid X imputed Y imputed X&Y imputed
MRCOG_Pt2
Teach_Psyc

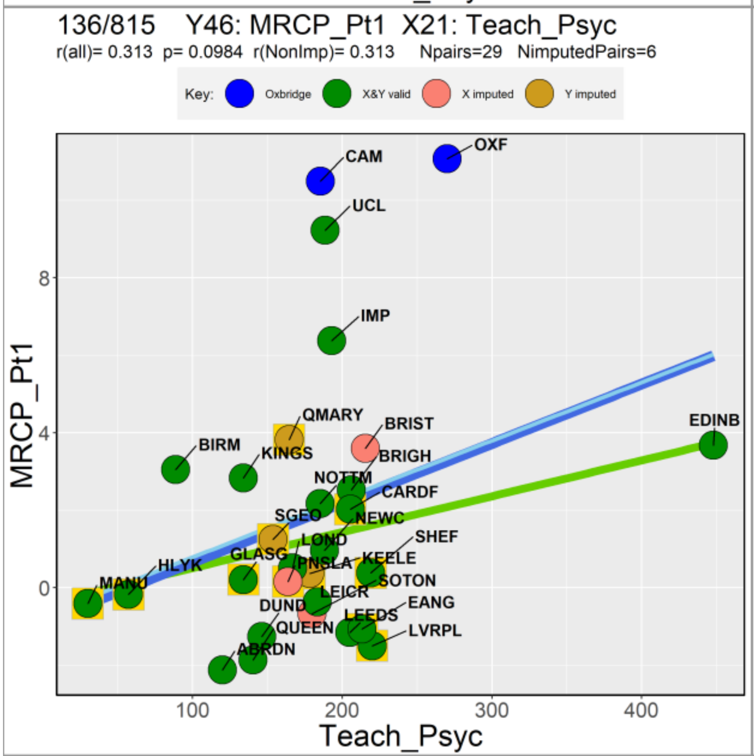
136/815 Y46: MRCP_Pt1 X21: Teach_Psyc
r(all)= 0.313 p= 0.0984 r(NonImp)= 0.313 Npairs=29 NimputedPairs=6
Key: Oxbridge X&Y valid X imputed Y imputed
MRCP_Pt1
Teach_Psyc

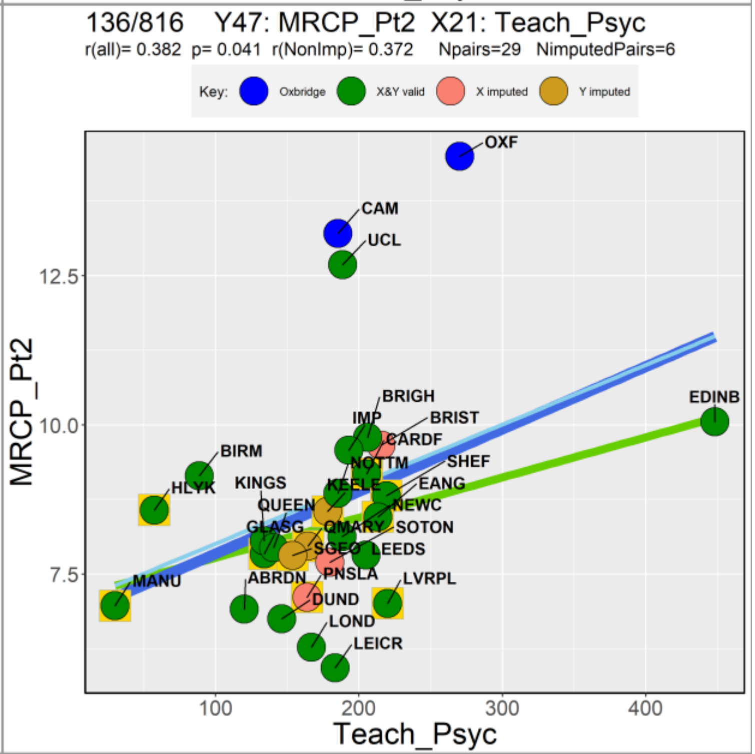
136/816 Y47: MRCP_Pt2 X21: Teach_Psyc
r(all)= 0.382 p= 0.041 r(NonImp)= 0.372 Npairs=29 NimputedPairs=6
Key: Oxbridge X&Y valid X imputed Y imputed
MRCP_Pt2
Teach_Psyc

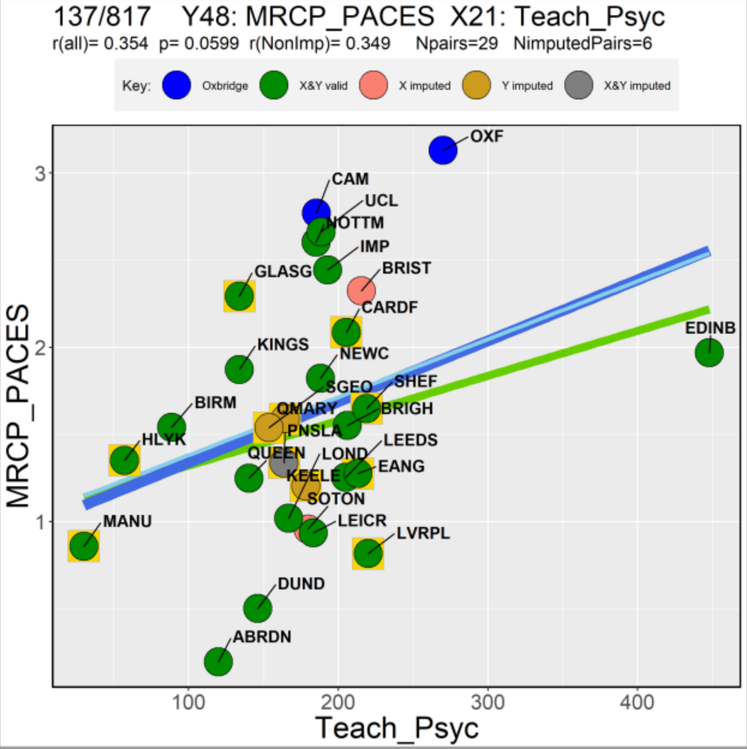
137/817 Y48: MRCP_PACES X21: Teach_Psyc
r(all)= 0.354 p= 0.0599 r(NonImp)= 0.349 Npairs=29 NimputedPairs=6
Key: Oxbridge X&Y valid X imputed Y imputed X&Y imputed
MRCP_PACES
Teach_Psyc
1
2
3
100
200
300
400
OXF
CAM
UCL
NOTTM
IMP
BRIST
GLASG
CARDF
EDINB
KINGS
NEWC
SGEO
SHEF
BIRM
QMARY
BRIGH
PNSLA
HLYK
LEEDS
QUEEN
LOND
KEELE
EANG
SOTON
MANU
LEICR
LVRPL
DUND
ABRDN

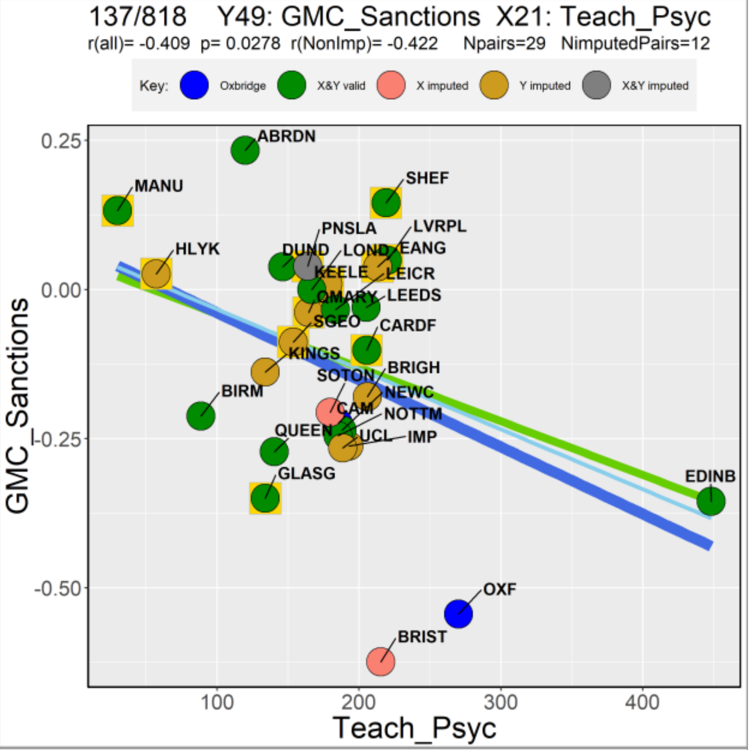
137/818 Y49: GMC_Sanctions X21: Teach_Psyc
r(all)= -0.409 p= 0.0278 r(NonImp)= -0.422 Npairs=29 NimputedPairs=12
Key: Oxbridge X&Y valid X imputed Y imputed X&Y imputed
GMC_Sanctions
Teach_Psyc
0.25
0.00
-0.25
-0.50
100
200
300
400
ABRDN
MANU
SHEF
PNSLA
LVRPL
HLYK
DUND
LOND
EANG
KEELE
LEICR
QMARY
LEEDS
SGEO
CARDF
KINGS
SOTON
BRIGH
BIRM
NEWC
CAM
NOTTM
QUEEN
UCL
IMP
GLASG
EDINB
OXF
BRIST

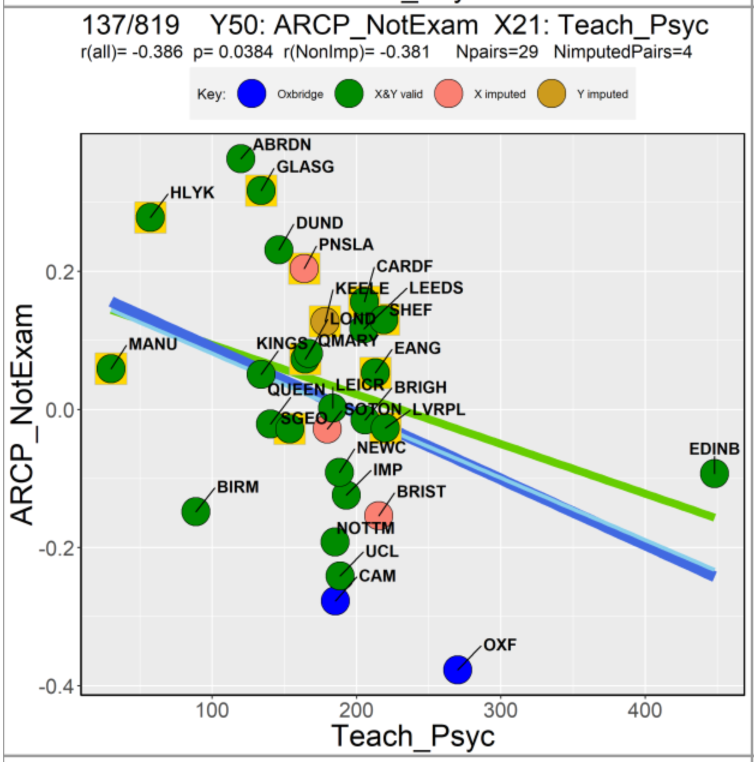
137/819 Y50: ARCP_NotExam X21: Teach_Psyc
r(all)= -0.386 p= 0.0384 r(NonImp)= -0.381 Npairs=29 NimputedPairs=4
Key: Oxbridge X&Y valid X imputed Y imputed
ARCP_NotExam
Teach_Psyc
0.2
0.0
-0.2
-0.4
100
200
300
400
ABRDN
GLASG
HLYK
DUND
PNSLA
CARDF
KEELE
LEEDS
SHEF
LOND
MANU
KINGS
QMARY
EANG
LEICR
BRIGH
QUEEN
SGEO
SOTON
LVRPL
EDINB
NEWC
IMP
BIRM
BRIST
NOTTM
UCL
CAM
OXF

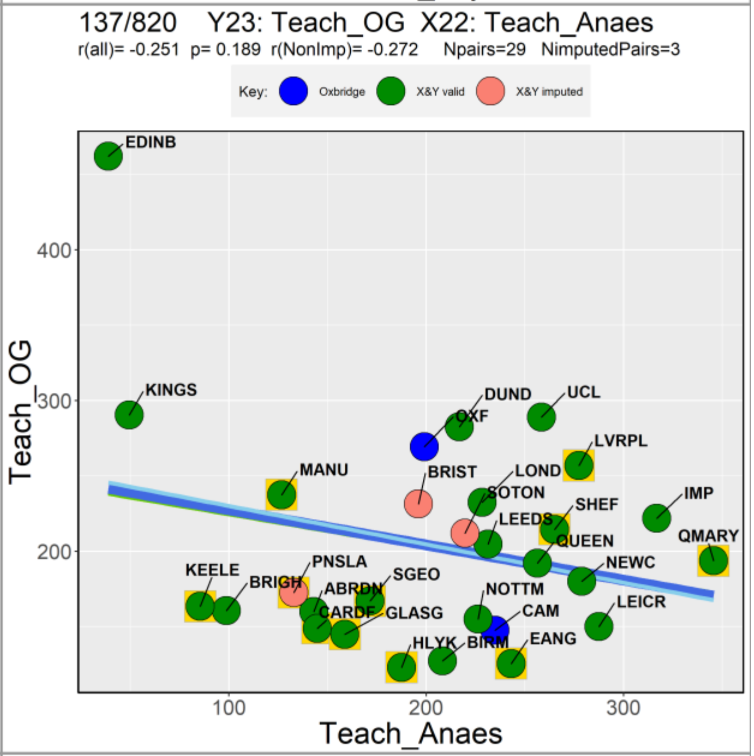
137/820 Y23: Teach_OG X22: Teach_Anaes
r(all)= -0.251 p= 0.189 r(NonImp)= -0.272 Npairs=29 NimputedPairs=3
Key: Oxbridge X&Y valid X&Y imputed
Teach_OG
Teach_Anaes
400
300
200
100
200
300
EDINB
KINGS
DUND
UCL
OXF
LVRPL
MANU
BRIST
LOND
SOTON
IMP
SHEF
LEEDS
QMARY
QUEEN
NEWC
KEELE
PNSLA
SGEO
BRIGH
ABRDN
NOTTM
CARDF
GLASG
CAM
LEICR
HLYK
BIRM
EANG

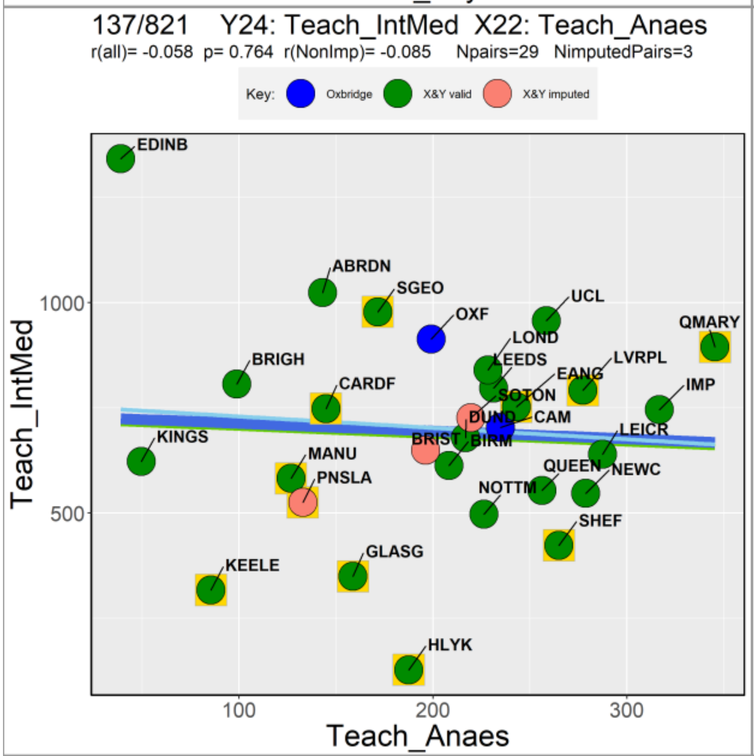
137/821 Y24: Teach_IntMed X22: Teach_Anaes
r(all)= -0.058 p= 0.764 r(NonImp)= -0.085 Npairs=29 NimputedPairs=3
Key: Oxbridge X&Y valid X&Y imputed
Teach_IntMed
Teach_Anaes
1000
500
100
200
300
EDINB
ABRDN
SGEO
UCL
OXF
QMARY
LOND
BRIGH
LEEDS
LVRPL
CARDF
EANG
IMP
SOTON
DUND
CAM
KINGS
BRIST
BIRM
LEICR
MANU
QUEEN
NEWC
PNSLA
NOTTM
SHEF
GLASG
KEELE
HLYK

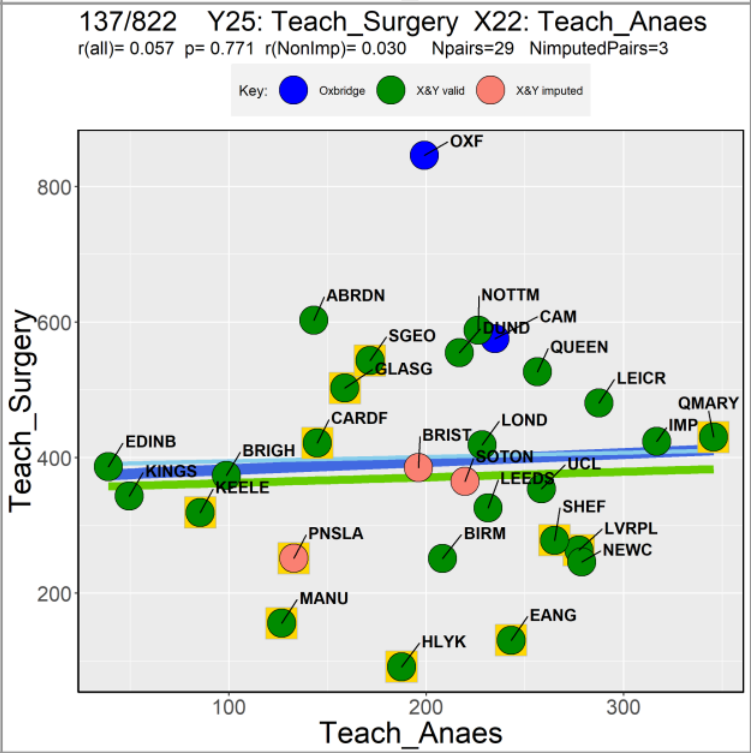
137/822 Y25: Teach_Surgery X22: Teach_Anaes
r(all)= 0.057 p= 0.771 r(NonImp)= 0.030 Npairs=29 NimputedPairs=3
Key: Oxbridge X&Y valid X&Y imputed
Teach_Surgery
Teach_Anaes
800
600
400
200
100
200
300
OXF
ABRDN
NOTTM
DUND
CAM
SGEO
QUEEN
GLASG
LEICR
QMARY
CARDF
LOND
IMP
EDINB
BRIGH
BRIST
KINGS
SOTON
UCL
KEELE
LEEDS
SHEF
PNSLA
BIRM
LVRPL
NEWC
MANU
EANG
HLYK

138/823 Y26: ExamTime X22: Teach_Anaes

r(all)= 0.147 p= 0.447 r(NonImp)= 0.151 Npairs=29 NimputedPairs=5

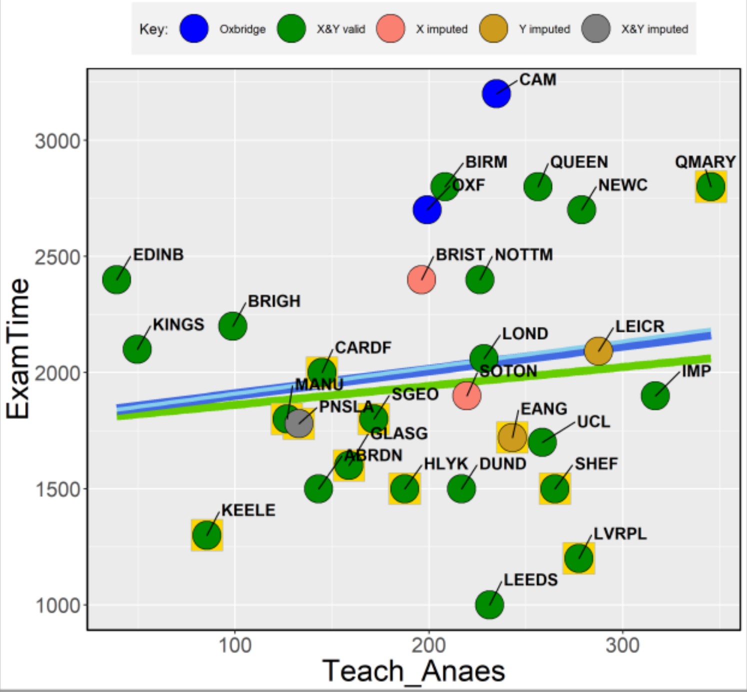

138/824 Y27: SelfRegLearn X22: Teach_Anaes

r(all)= -0.175 p= 0.364 r(NonImp)= -0.176 Npairs=29 NimputedPairs=3

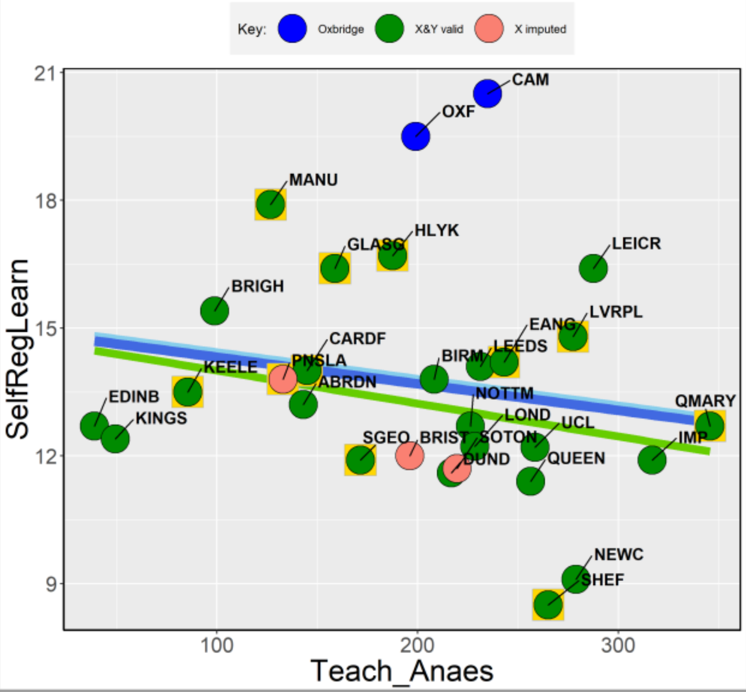

138/825 Y28: NSS_Satisfn X22: Teach_Anaes

r(all)= -0.030 p= 0.877 r(NonImp)= -0.012 Npairs=29 NimputedPairs=3

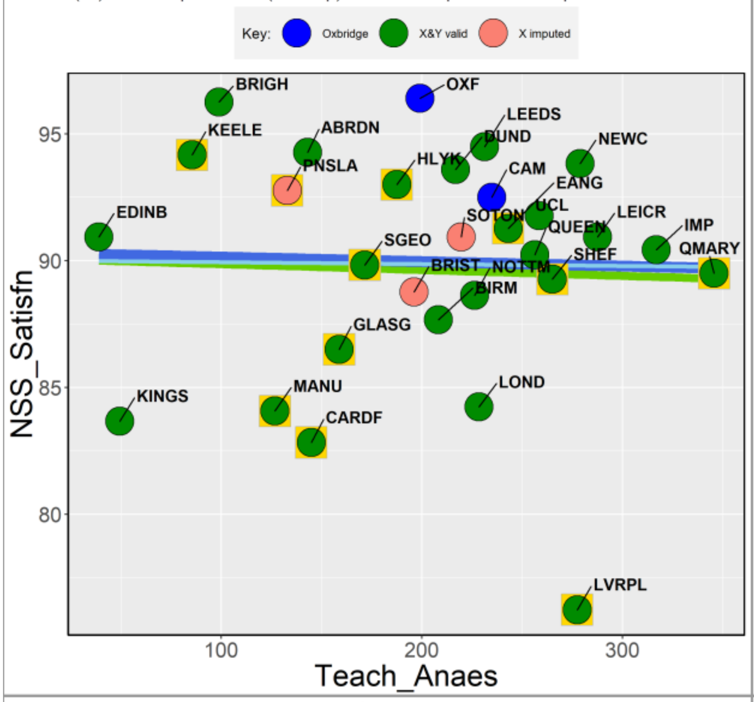

138/826 Y29: NSS_Feedback X22: Teach_Anaes

r(all)= 0.135 p= 0.486 r(NonImp)= 0.157 Npairs=29 NimputedPairs=3

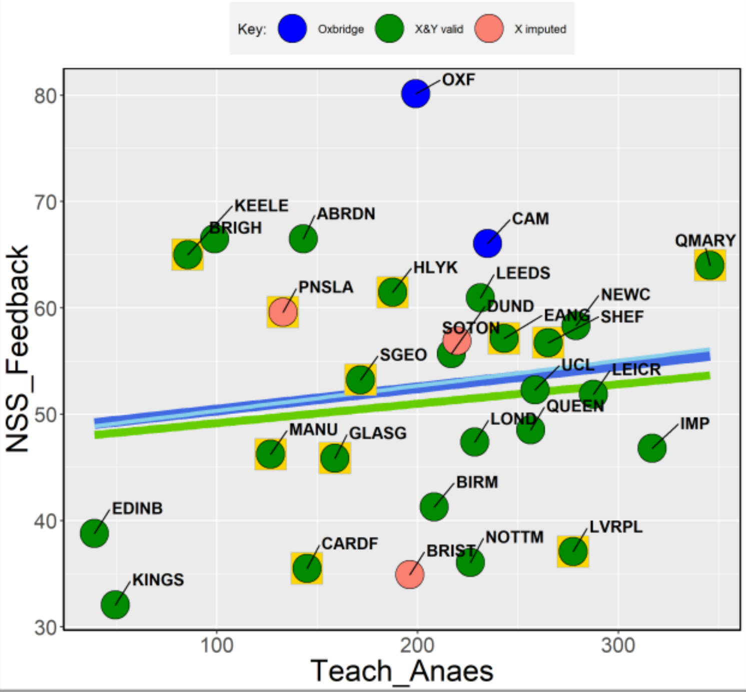

138/827 Y30: UKFPO_EPM X22: Teach_Anaes

r(all)= 0.254 p= 0.184 r(NonImp)= 0.209 Npairs=29 NimputedPairs=3

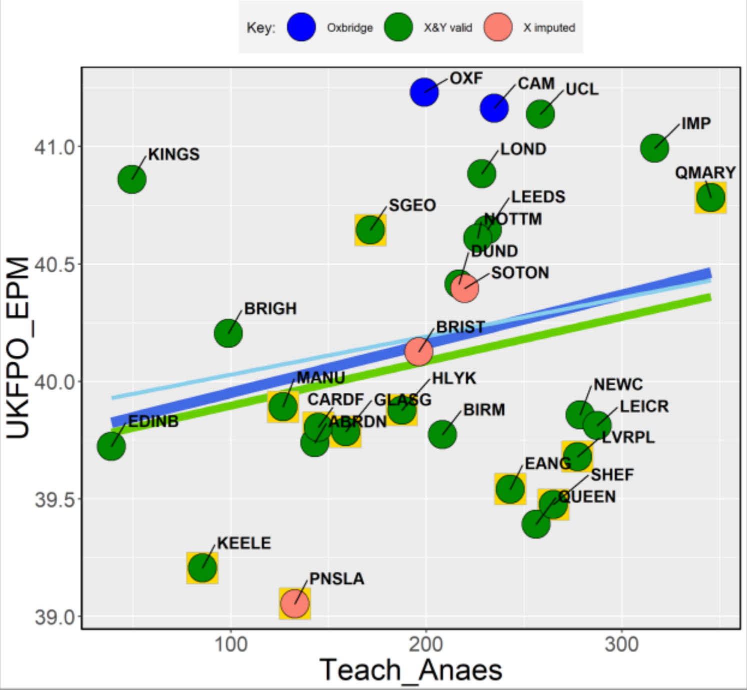

138/828 Y31: UKFPO_SJT X22: Teach_Anaes

r(all)= 0.005 p= 0.978 r(NonImp)= -0.004 Npairs=29 NimputedPairs=3

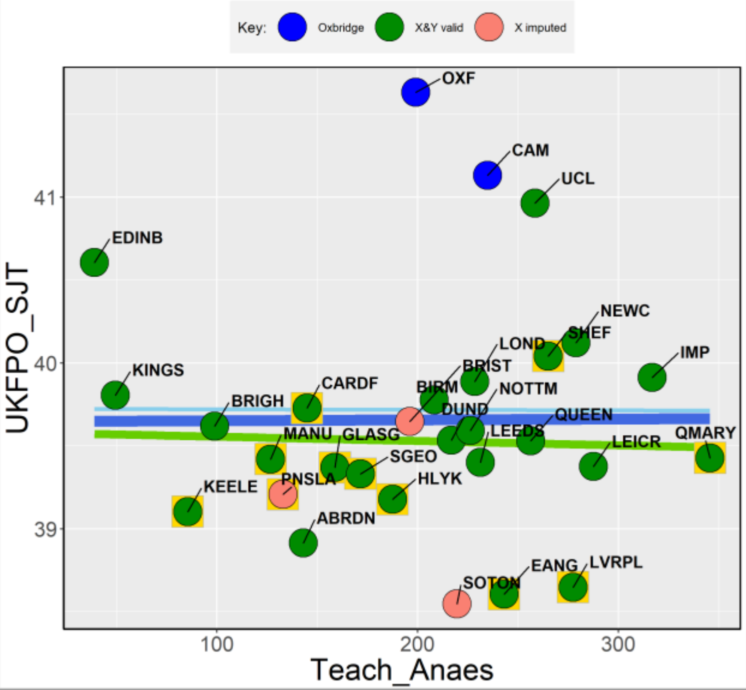

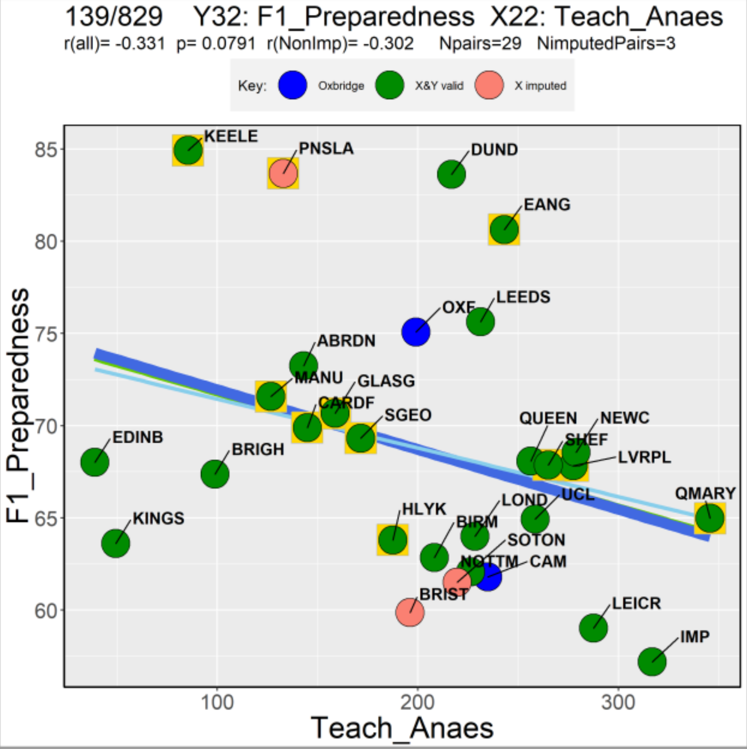
139/829 Y32: F1_Preparedness X22: Teach_Anaes
r(all)= -0.331 p= 0.0791 r(NonImp)= -0.302 Npairs=29 NimputedPairs=3
Key: Oxbridge X&Y valid X imputed
F1_Preparedness
Teach_Anaes
KEELE
PNSLA
DUND
EANG
LEEDS
OXF
ABRDN
MANU
GLASG
CARDF
SGEO
EDINB
BRIGH
QUEEN
NEWC
SHEF
LVRPL
QMARY
KINGS
HLYK
LOND
UCL
BIRM
SOTON
NOTTM
CAM
BRIST
LEICR
IMP

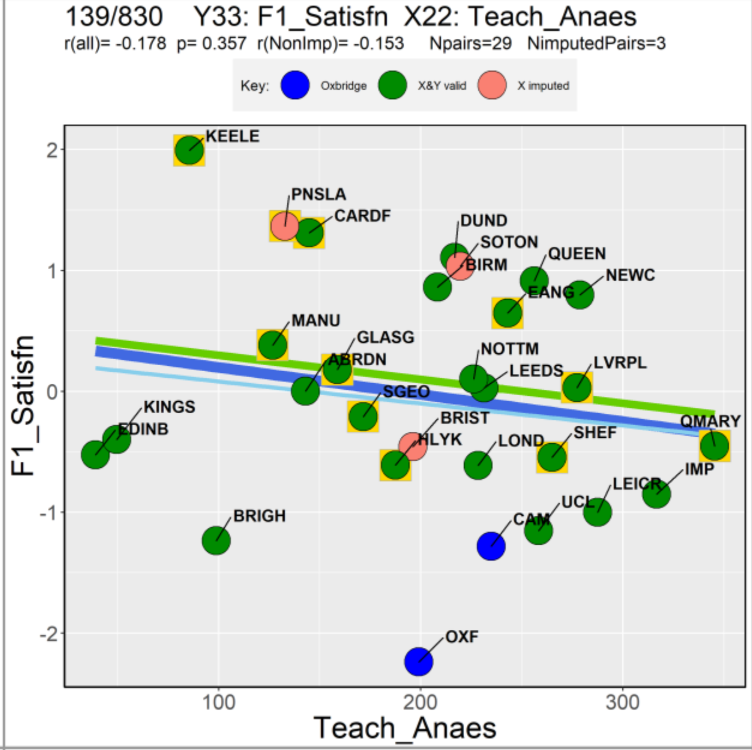
139/830 Y33: F1_Satisfn X22: Teach_Anaes
r(all)= -0.178 p= 0.357 r(NonImp)= -0.153 Npairs=29 NimputedPairs=3
Key: Oxbridge X&Y valid X imputed
F1_Satisfn
Teach_Anaes
KEELE
PNSLA
CARDF
DUND
SOTON
BIRM
QUEEN
NEWC
EANG
MANU
GLASG
ABRDN
NOTTM
LEEDS
LVRPL
KINGS
SGEO
EDINB
BRIST
HLYK
LOND
SHEF
QMARY
LEICR
IMP
UCL
CAM
BRIGH
OXF

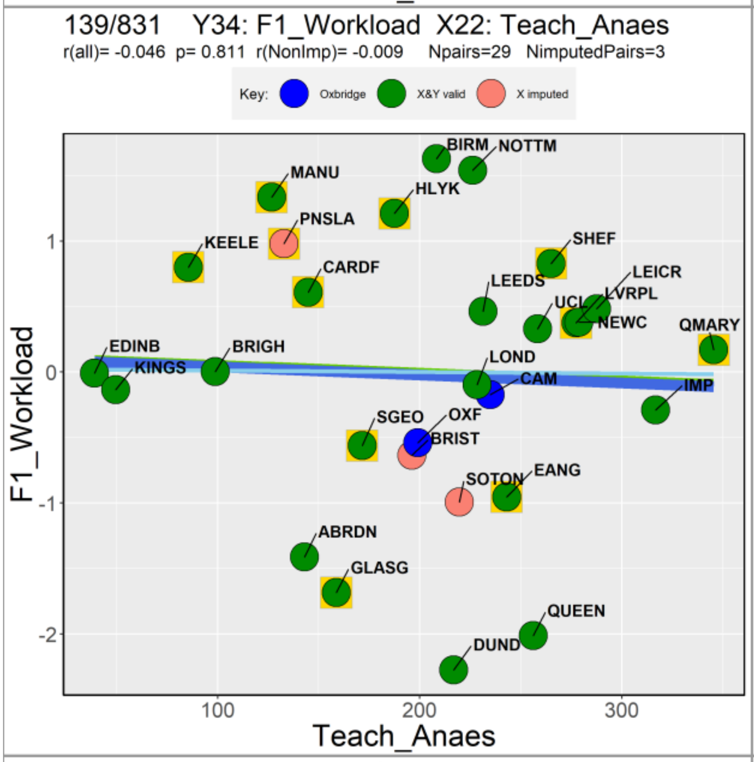
139/831 Y34: F1_Workload X22: Teach_Anaes
r(all)= -0.046 p= 0.811 r(NonImp)= -0.009 Npairs=29 NimputedPairs=3
Key: Oxbridge X&Y valid X imputed
F1_Workload
Teach_Anaes
BIRM
NOTTM
MANU
HLYK
PNSLA
KEELE
SHEF
CARDF
LEICR
LEEDS
LVRPL
UCL
NEWC
QMARY
EDINB
BRIGH
KINGS
LOND
CAM
IMP
SGEO
OXF
BRIST
SOTON
EANG
ABRDN
GLASG
QUEEN
DUND

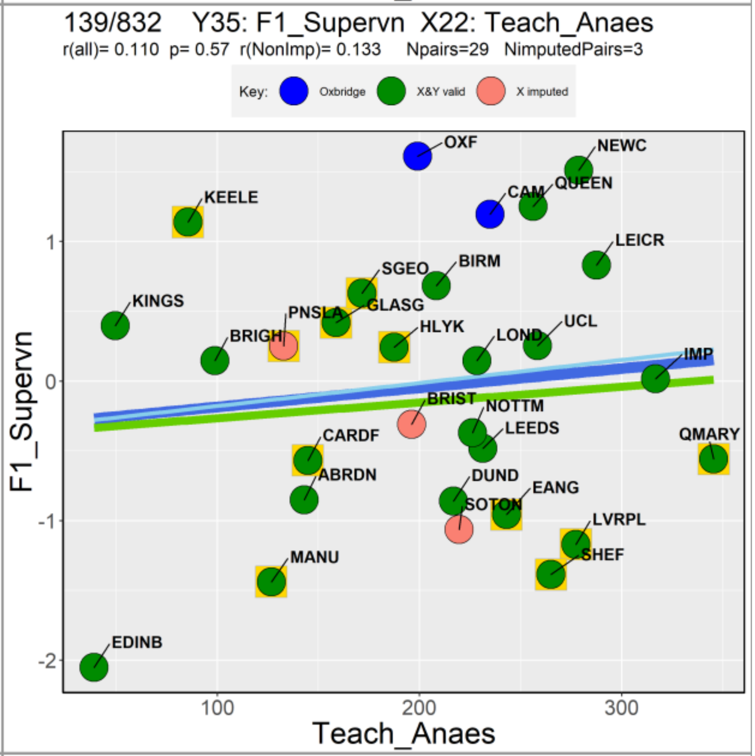
139/832 Y35: F1_Supervn X22: Teach_Anaes
r(all)= 0.110 p= 0.57 r(NonImp)= 0.133 Npairs=29 NimputedPairs=3
Key: Oxbridge X&Y valid X imputed
F1_Supervn
Teach_Anaes
OXF
NEWC
CAM
QUEEN
KEELE
LEICR
SGEO
BIRM
KINGS
PNSLA
GLASG
BRIGH
HLYK
LOND
UCL
IMP
BRIST
NOTTM
LEEDS
CARDF
QMARY
ABRDN
DUND
EANG
SOTON
LVRPL
SHEF
MANU
EDINB

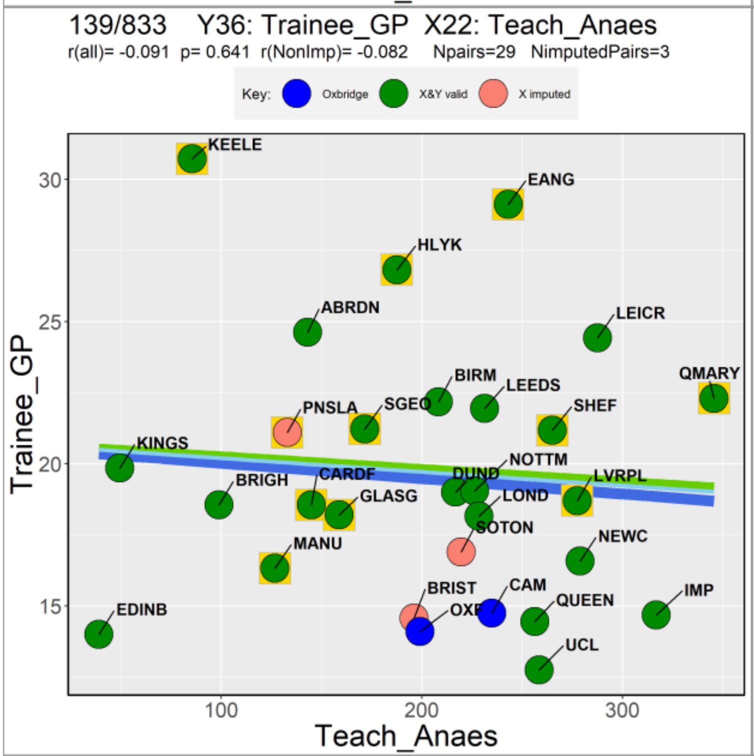
139/833 Y36: Trainee_GP X22: Teach_Anaes
r(all)= -0.091 p= 0.641 r(NonImp)= -0.082 Npairs=29 NimputedPairs=3
Key: Oxbridge X&Y valid X imputed
Trainee_GP
Teach_Anaes
KEELE
EANG
HLYK
ABRDN
LEICR
BIRM
LEEDS
QMARY
PNSLA
SGEO
SHEF
KINGS
NOTTM
DUND
LVRPL
BRIGH
CARDF
GLASG
LOND
SOTON
NEWC
MANU
BRIST
CAM
OXF
QUEEN
IMP
EDINB
UCL

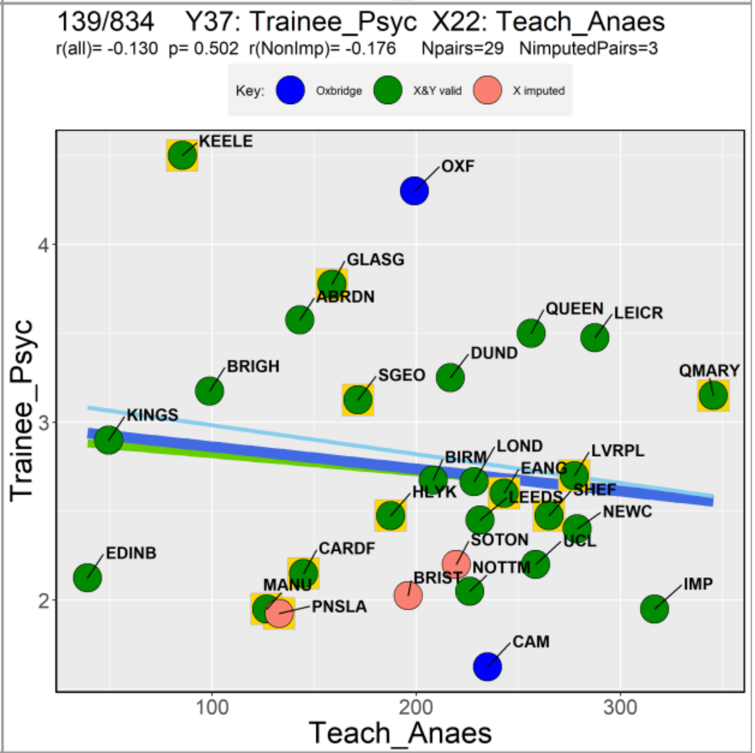
139/834 Y37: Trainee_Psyc X22: Teach_Anaes
r(all)= -0.130 p= 0.502 r(NonImp)= -0.176 Npairs=29 NimputedPairs=3
Key: Oxbridge X&Y valid X imputed
Trainee_Psyc
Teach_Anaes
KEELE
OXF
GLASG
ABRDN
QUEEN
LEICR
DUND
BRIGH
SGEO
QMARY
KINGS
BIRM
LOND
LVRPL
EANG
HLYK
LEEDS
SHEF
NEWC
SOTON
UCL
EDINB
CARDF
NOTTM
BRIST
MANU
PNSLA
IMP
CAM

140/835 Y38: TraineeApp_Surgery X22: Teach_Ana
r(all)= 0.195 p= 0.311 r(NonImp)= 0.131 Npairs=29 NimputedPairs=5

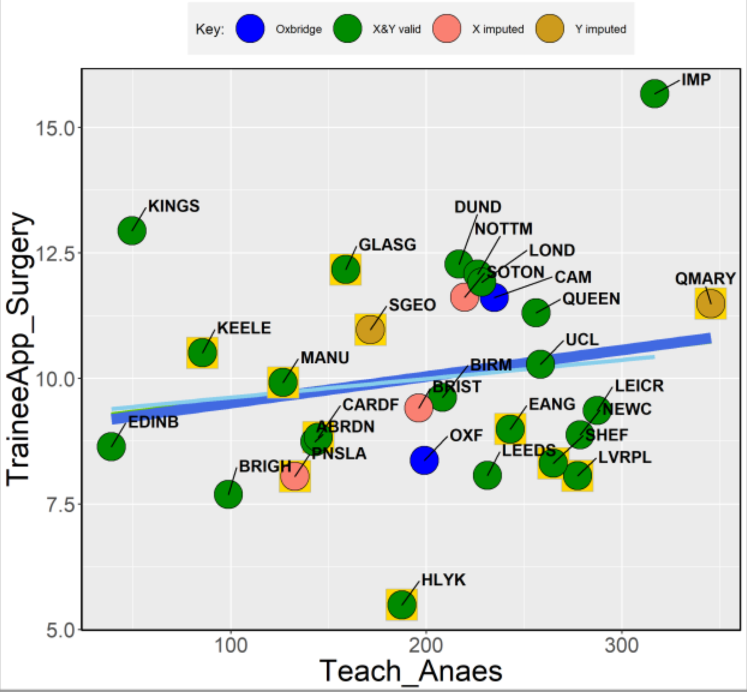

140/836 Y39: TraineeApp_Anaes X22: Teach_Anaes
r(all)= 0.154 p= 0.427 r(NonImp)= 0.150 Npairs=29 NimputedPairs=3

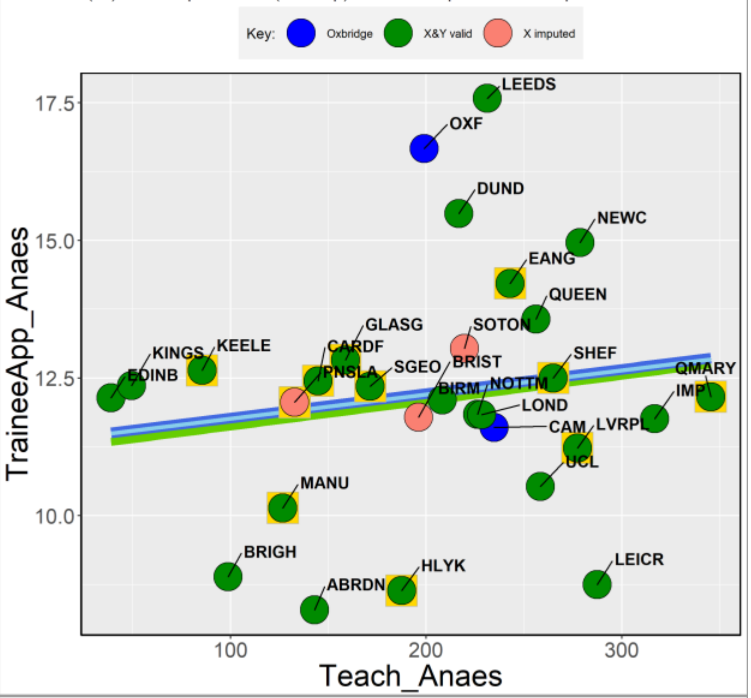

140/837 Y40: GMC_PGexams X22: Teach_Anaes
r(all)= -0.013 p= 0.947 r(NonImp)= -0.035 Npairs=29 NimputedPairs=3

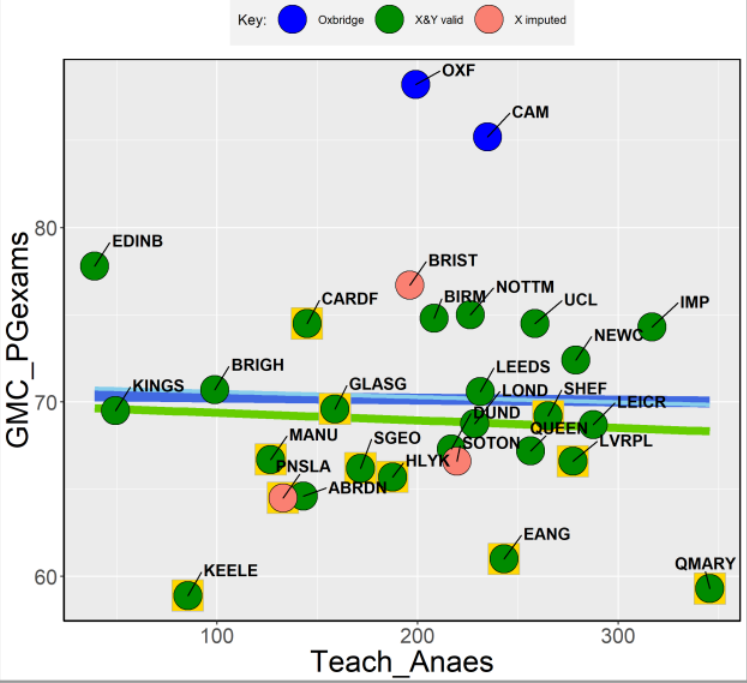

140/838 Y41: MRCGP_AKT X22: Teach_Anaes
r(all)= 0.005 p= 0.981 r(NonImp)= -0.030 Npairs=29 NimputedPairs=3

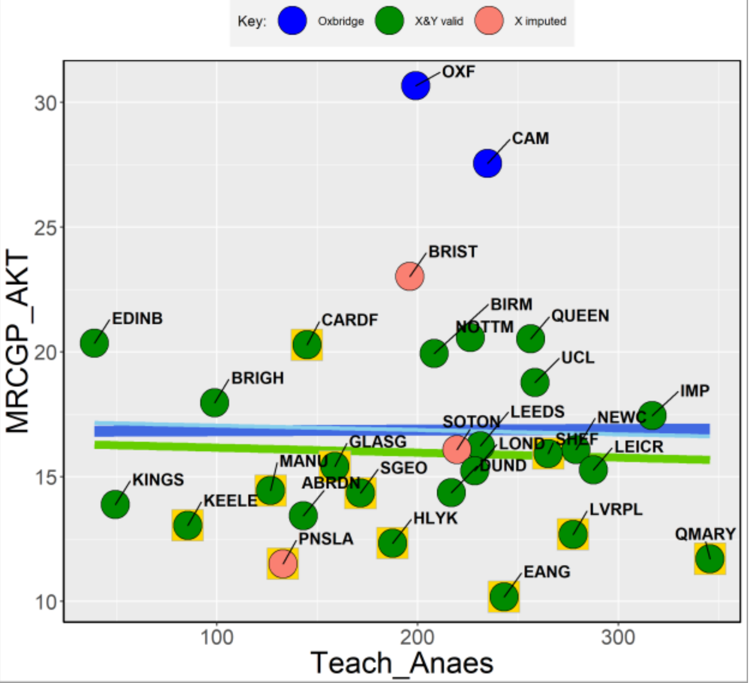

140/839 Y42: MRCGP_CSA X22: Teach_Anaes
r(all)= -0.010 p= 0.959 r(NonImp)= -0.019 Npairs=29 NimputedPairs=3

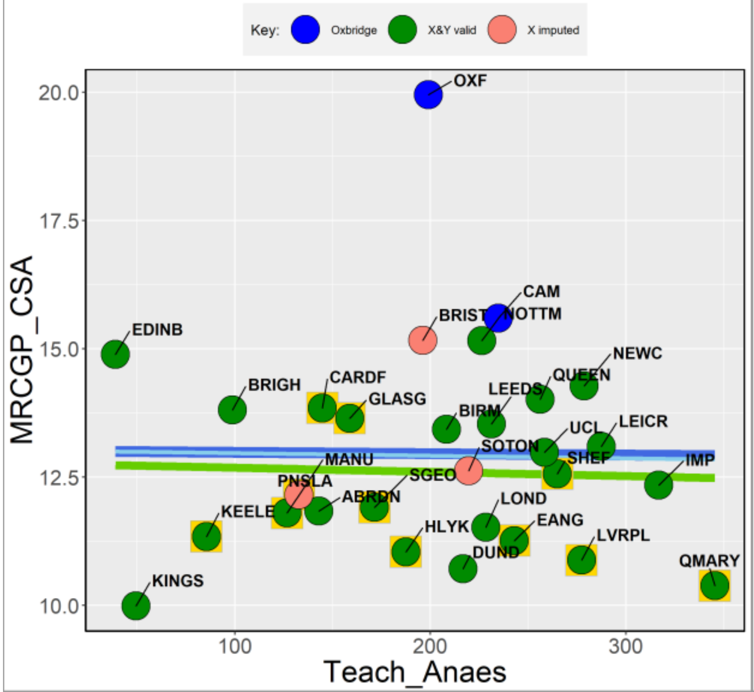

140/840 Y43: FRCA_Pt1 X22: Teach_Anaes
r(all)= -0.147 p= 0.448 r(NonImp)= -0.232 Npairs=29 NimputedPairs=12

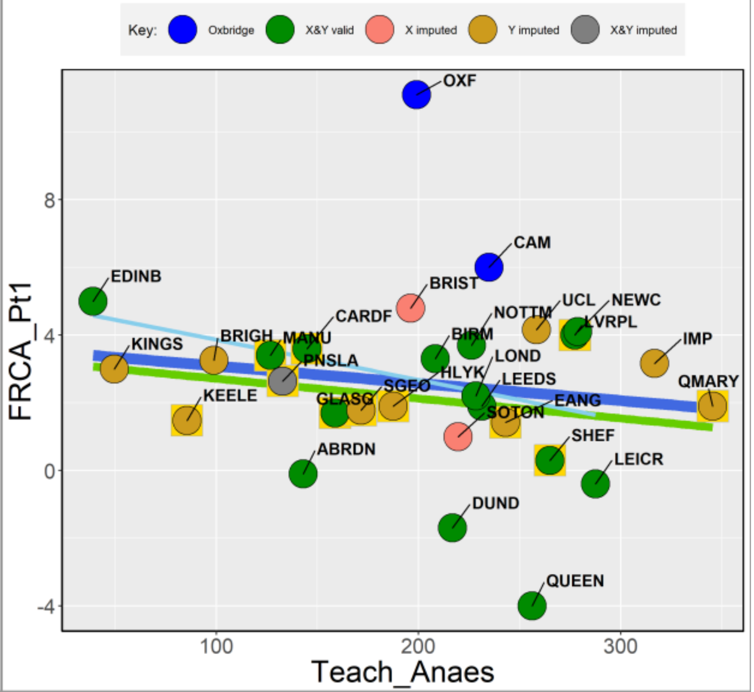

Supplement: Supplementary file 7 — Additional file 7. Graphs 631 to 840 (pages 106 to 140). [file 12916_2020_1572_MOESM7_ESM.pdf]
