# Supplementary material for: Exploring UK medical school differences: the MedDifs study of selection, teaching, student and F1 perceptions, postgraduate outcomes and fitness to practise
Source: BMC Med. 2020 May 14;18:136. doi: 10.1186/s12916-020-01572-3 (PMC7222458; doi:10.1186/s12916-020-01572-3)

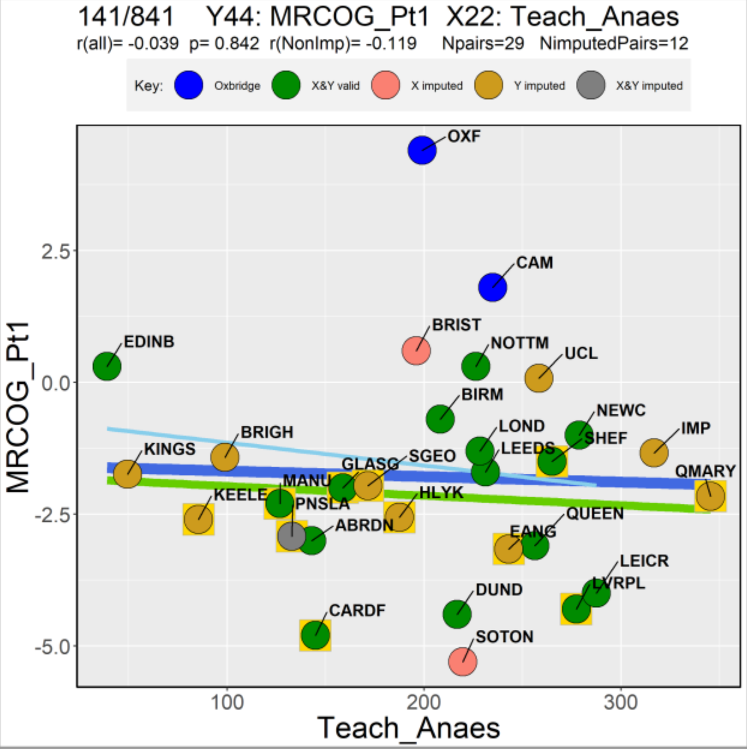
141/841 Y44: MRCOG_Pt1 X22: Teach_Anaes
r(all)= -0.039 p= 0.842 r(NonImp)= -0.119 Npairs=29 NimputedPairs=12
Key: Oxbridge X&Y valid X imputed Y imputed X&Y imputed
MRCOG_Pt1
Teach_Anaes

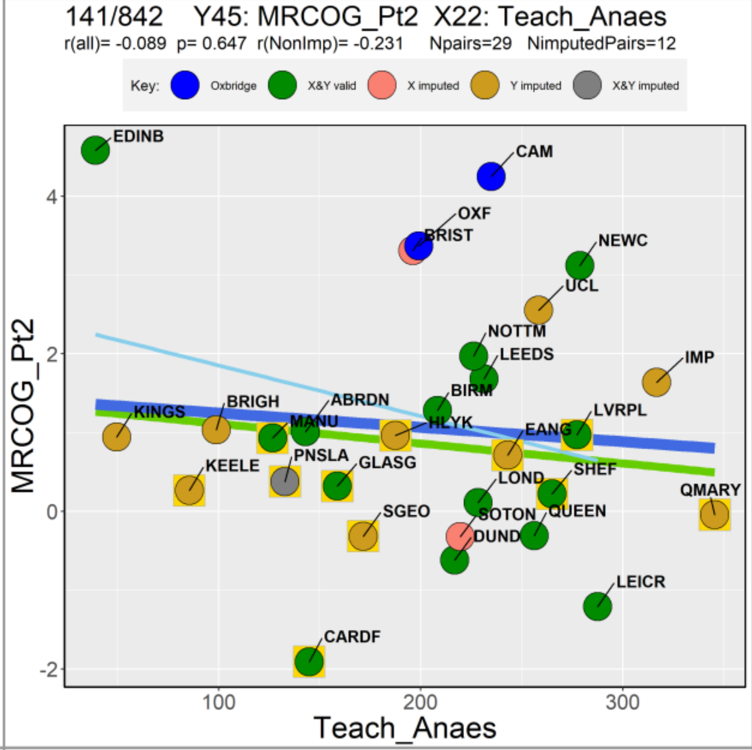
141/842 Y45: MRCOG_Pt2 X22: Teach_Anaes
r(all)= -0.089 p= 0.647 r(NonImp)= -0.231 Npairs=29 NimputedPairs=12
Key: Oxbridge X&Y valid X imputed Y imputed X&Y imputed
MRCOG_Pt2
Teach_Anaes

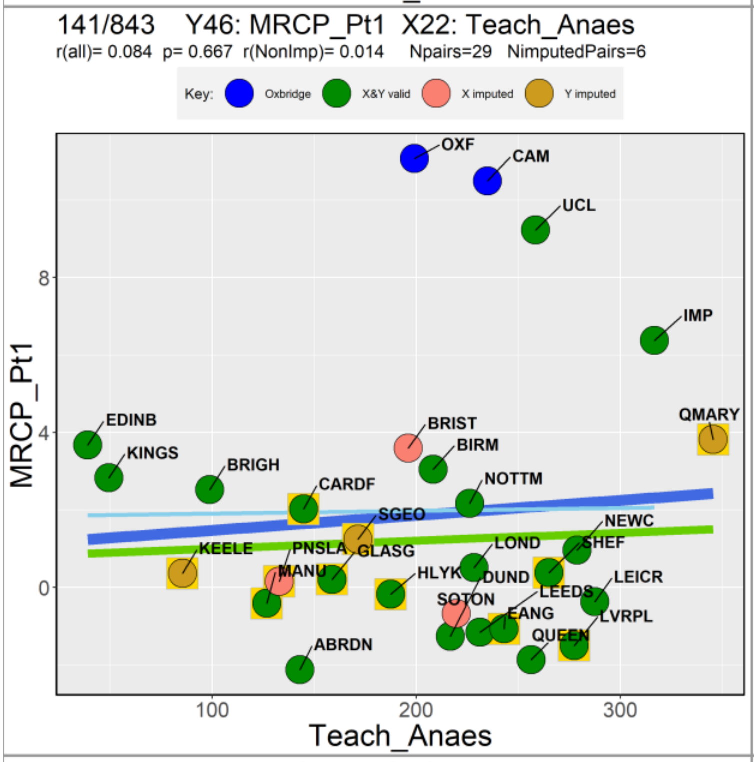
141/843 Y46: MRCP_Pt1 X22: Teach_Anaes
r(all)= 0.084 p= 0.667 r(NonImp)= 0.014 Npairs=29 NimputedPairs=6
Key: Oxbridge X&Y valid X imputed Y imputed
MRCP_Pt1
Teach_Anaes

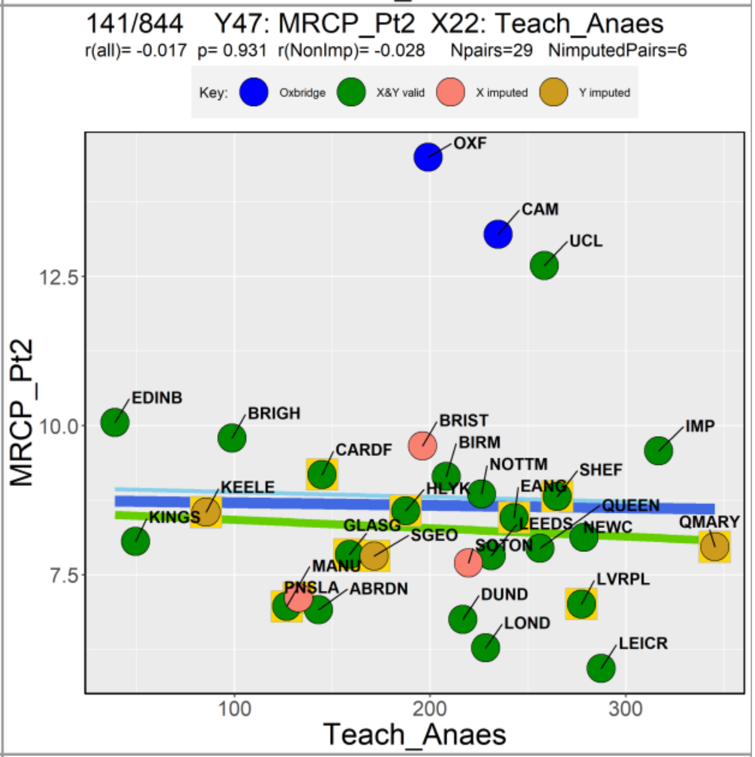
141/844 Y47: MRCP_Pt2 X22: Teach_Anaes
r(all)= -0.017 p= 0.931 r(NonImp)= -0.028 Npairs=29 NimputedPairs=6
Key: Oxbridge X&Y valid X imputed Y imputed
MRCP_Pt2
Teach_Anaes

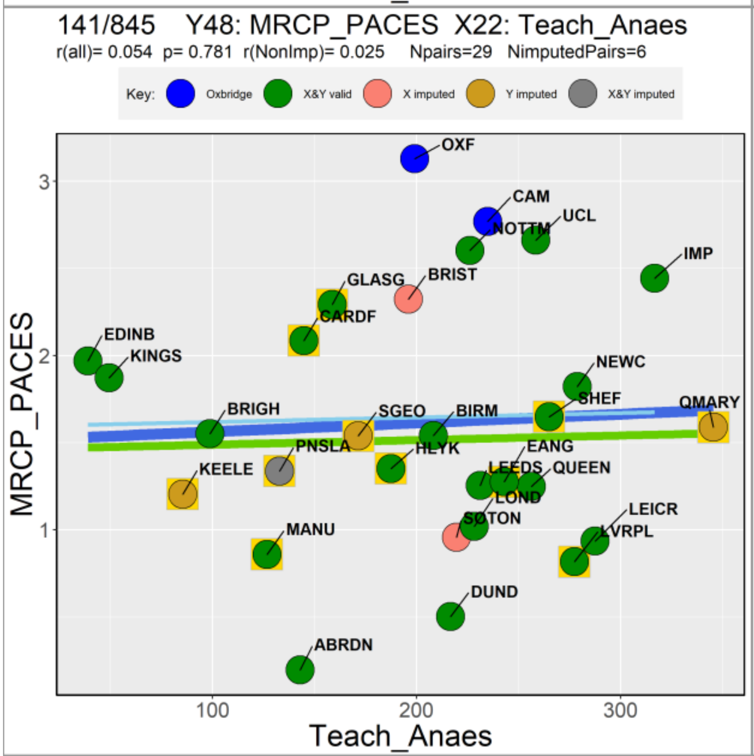
141/845 Y48: MRCP_PACES X22: Teach_Anaes
r(all)= 0.054 p= 0.781 r(NonImp)= 0.025 Npairs=29 NimputedPairs=6
Key: Oxbridge X&Y valid X imputed Y imputed X&Y imputed
MRCP_PACES
Teach_Anaes

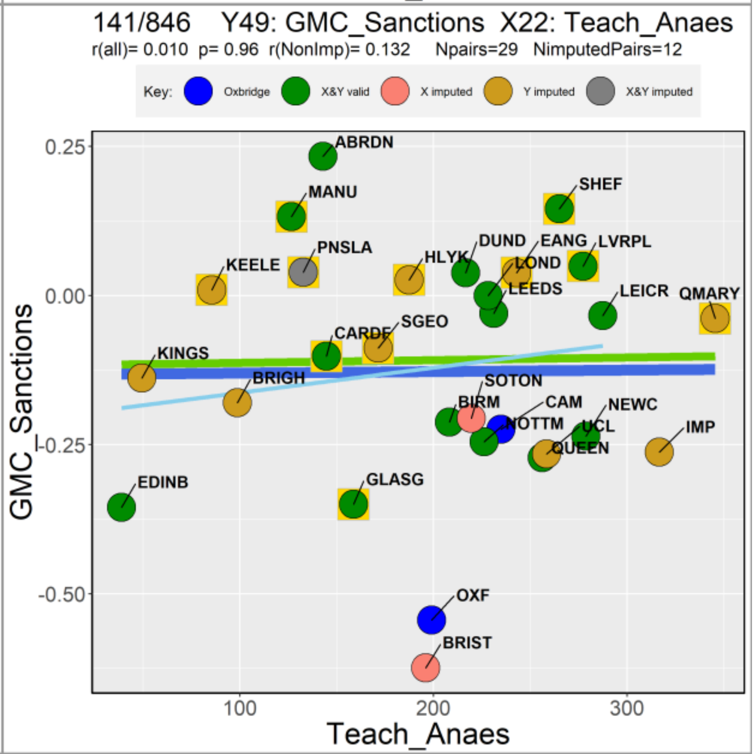
141/846 Y49: GMC_Sanctions X22: Teach_Anaes
r(all)= 0.010 p= 0.96 r(NonImp)= 0.132 Npairs=29 NimputedPairs=12
Key: Oxbridge X&Y valid X imputed Y imputed X&Y imputed
GMC_Sanctions
Teach_Anaes

142/847 Y50: ARCP_NotExam X22: Teach_Anaes
r(all)= -0.244 p= 0.203 r(NonImp)= -0.194 Npairs=29 NimputedPairs=4

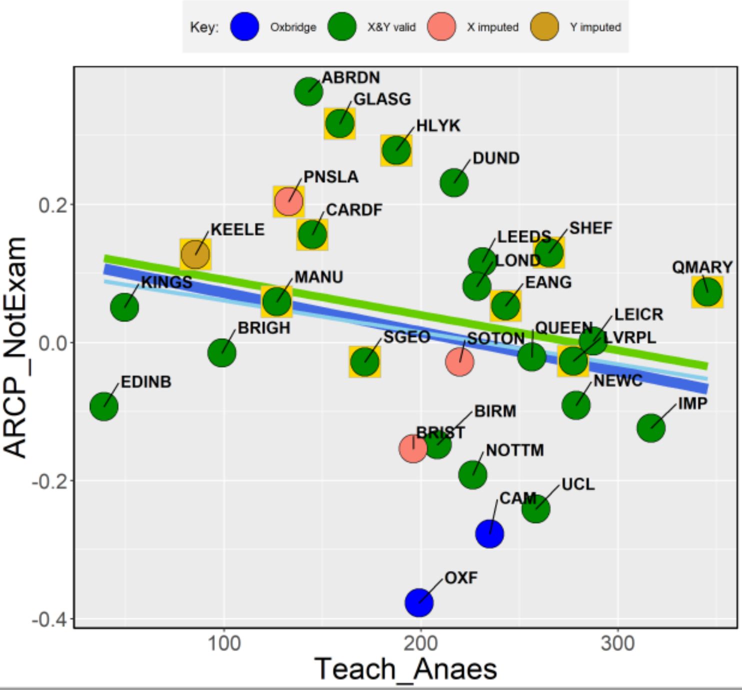

142/848 Y24: Teach_IntMed X23: Teach_OG
r(all)= 0.566 p= 0.00137 r(NonImp)= 0.567 Npairs=29 NimputedPairs=3

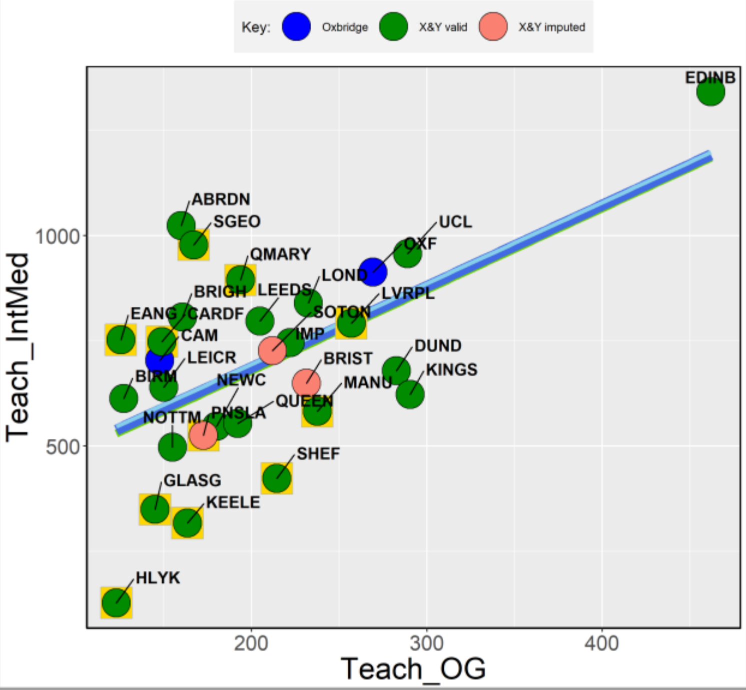

142/849 Y25: Teach_Surgery X23: Teach_OG
r(all)= 0.113 p= 0.56 r(NonImp)= 0.103 Npairs=29 NimputedPairs=3

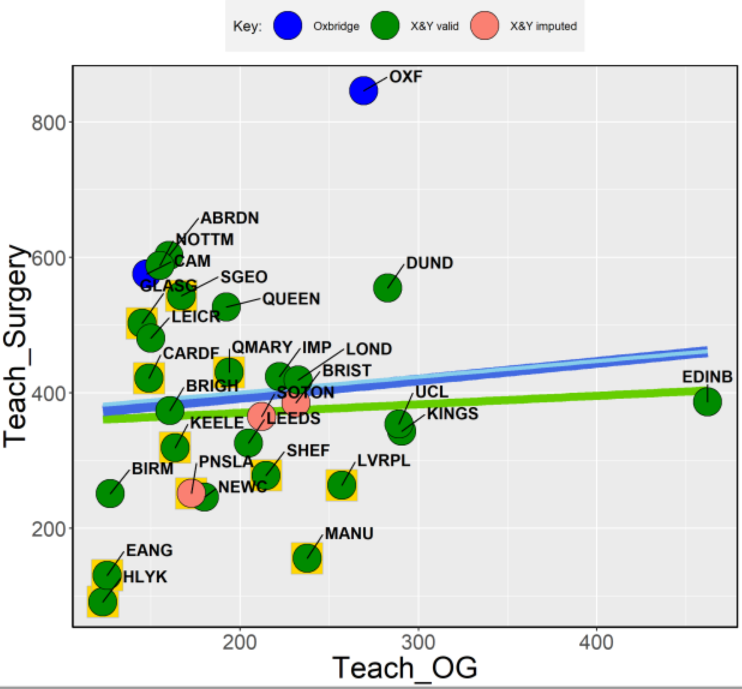

142/850 Y26: ExamTime X23: Teach_OG
r(all)= 0.001 p= 0.994 r(NonImp)= -0.034 Npairs=29 NimputedPairs=5

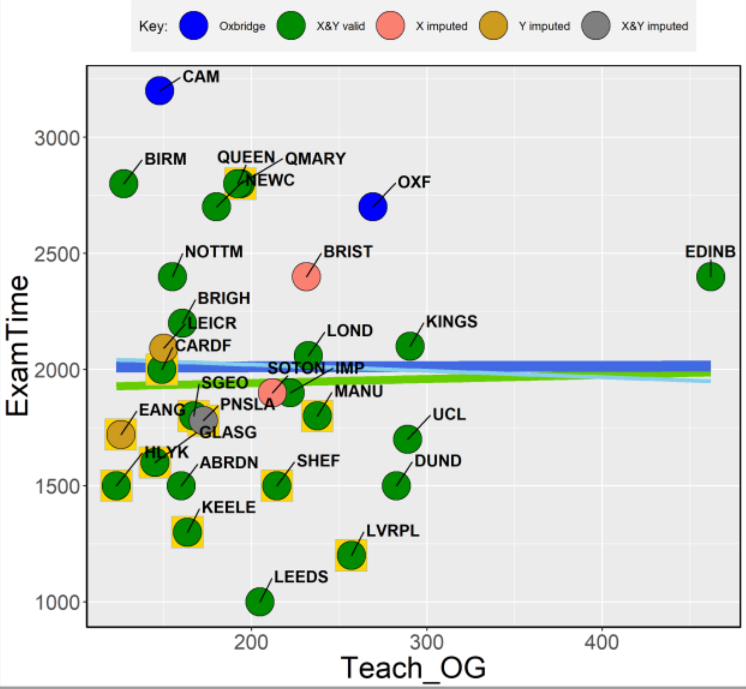

142/851 Y27: SelfRegLearn X23: Teach_OG
r(all)= -0.196 p= 0.307 r(NonImp)= -0.189 Npairs=29 NimputedPairs=3

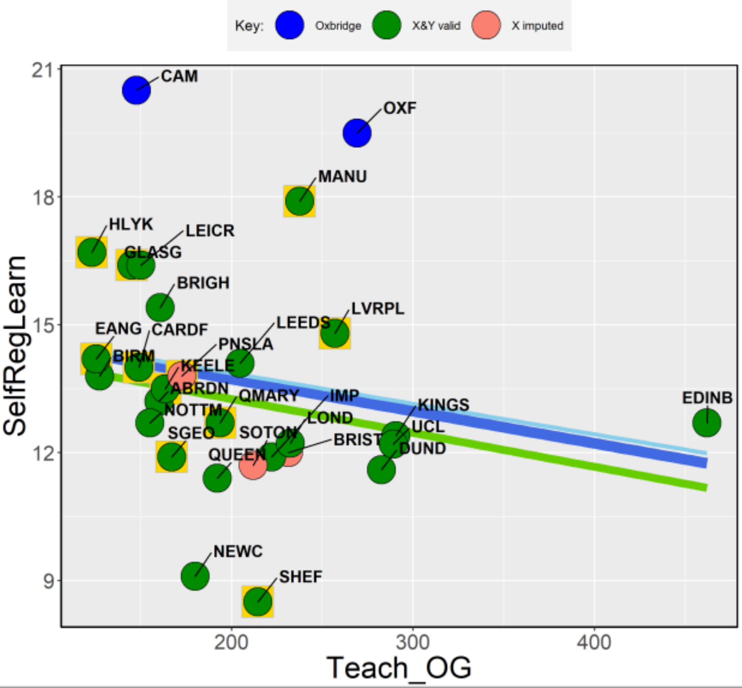

142/852 Y28: NSS_Satisfn X23: Teach_OG
r(all)= -0.119 p= 0.54 r(NonImp)= -0.108 Npairs=29 NimputedPairs=3

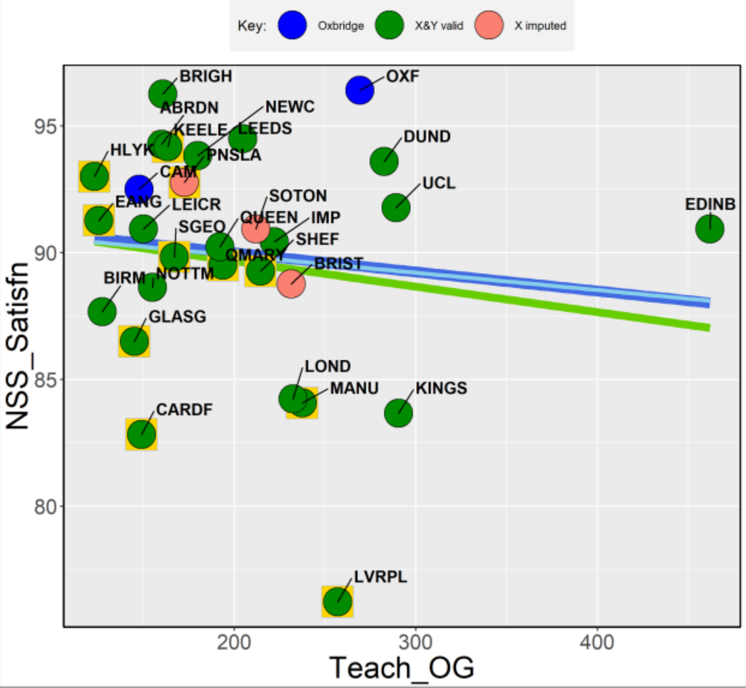

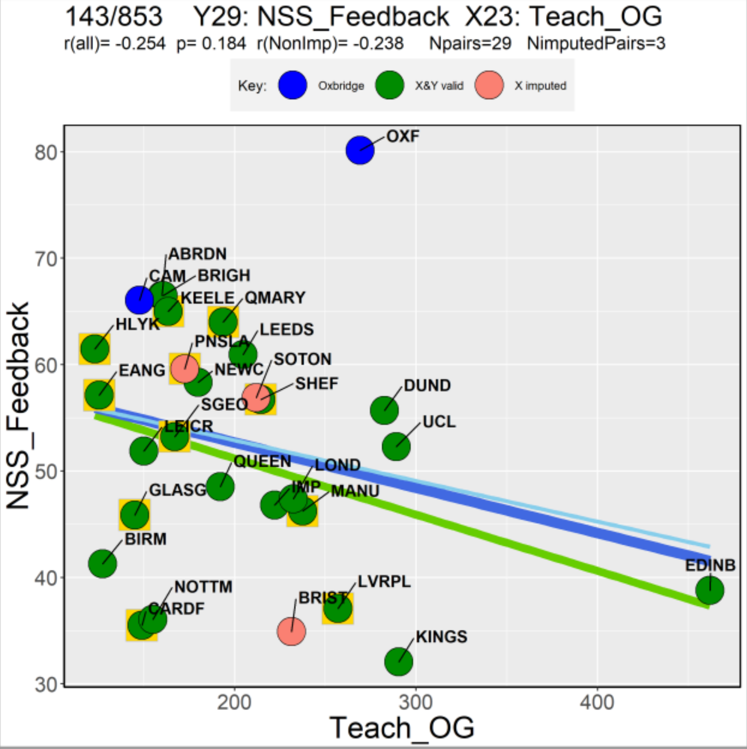
143/853 Y29: NSS_Feedback X23: Teach_OG
r(all)= -0.254 p= 0.184 r(NonImp)= -0.238 Npairs=29 NimputedPairs=3
Key: Oxbridge X&Y valid X imputed
NSS_Feedback
Teach_OG
80
70
60
50
40
30
200
300
400
OXF
ABRDN
CAM
BRIGH
KEELE
QMARY
HLYK
LEEDS
PNSLA
SOTON
EANG
NEWC
SHEF
DUND
SGEO
LEICR
UCL
QUEEN
LOND
GLASG
IMP
MANU
BIRM
EDINB
NOTTM
LVRPL
CARDF
BRIST
KINGS

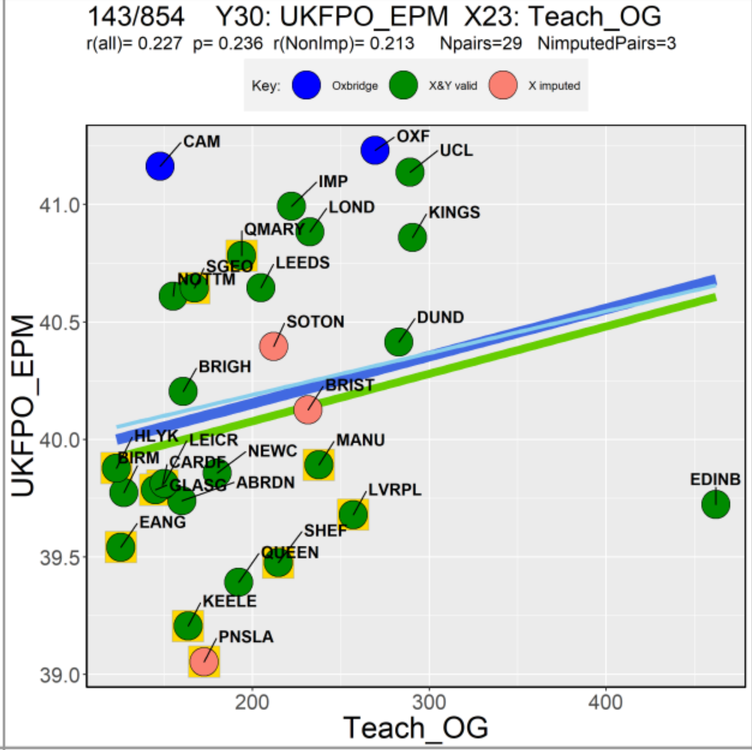
143/854 Y30: UKFPO_EPM X23: Teach_OG
r(all)= 0.227 p= 0.236 r(NonImp)= 0.213 Npairs=29 NimputedPairs=3
Key: Oxbridge X&Y valid X imputed
UKFPO_EPM
Teach_OG
41.0
40.5
40.0
39.5
39.0
200
300
400
CAM
OXF
UCL
IMP
LOND
KINGS
QMARY
SGEO
LEEDS
NOTTM
SOTON
DUND
BRIGH
BRIST
HLYK
LEICR
BIRM
CARDF
NEWC
MANU
GLASG
ABRDN
EDINB
LVRPL
EANG
SHEF
QUEEN
KEELE
PNSLA

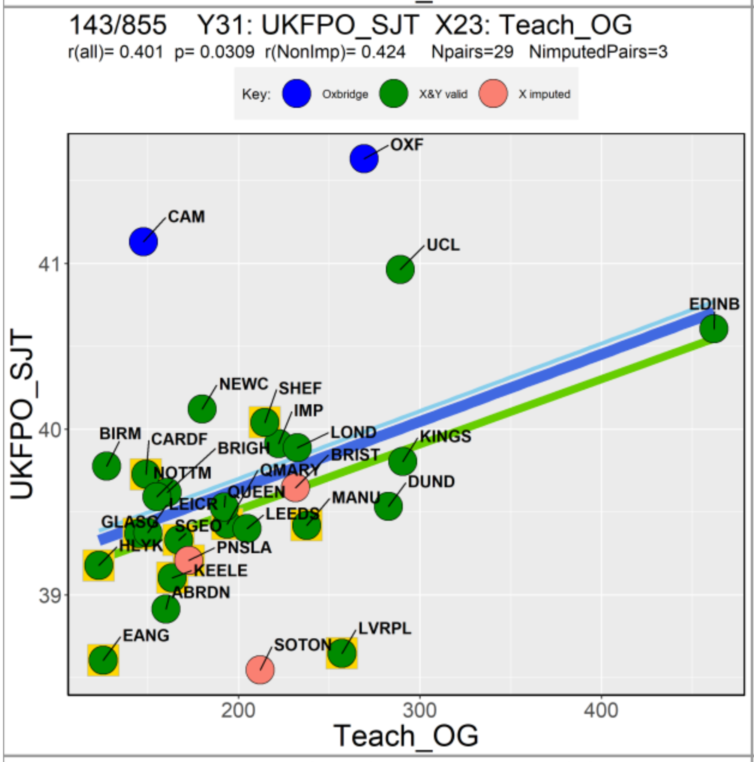
143/855 Y31: UKFPO_SJT X23: Teach_OG
r(all)= 0.401 p= 0.0309 r(NonImp)= 0.424 Npairs=29 NimputedPairs=3
Key: Oxbridge X&Y valid X imputed
UKFPO_SJT
Teach_OG
41
40
39
200
300
400
OXF
CAM
UCL
EDINB
NEWC
SHEF
IMP
BIRM
CARDF
LOND
KINGS
BRIGH
BRIST
NOTTM
QMARY
DUND
QUEEN
MANU
LEICR
GLASG
LEEDS
SGEO
HLYK
PNSLA
KEELE
ABRDN
EANG
LVRPL
SOTON

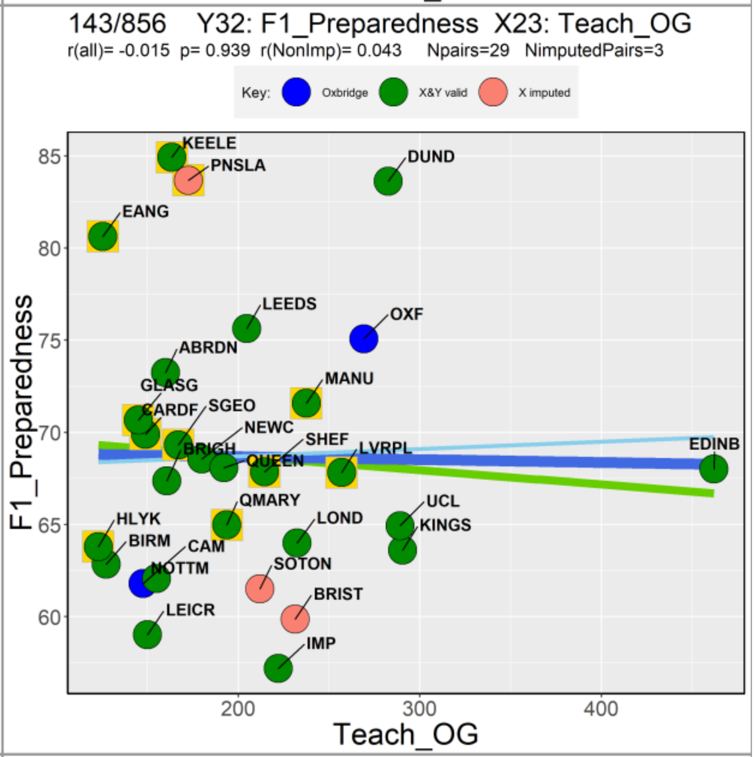
143/856 Y32: F1_Preparedness X23: Teach_OG
r(all)= -0.015 p= 0.939 r(NonImp)= 0.043 Npairs=29 NimputedPairs=3
Key: Oxbridge X&Y valid X imputed
F1_Preparedness
Teach_OG
85
80
75
70
65
60
200
300
400
KEELE
PNSLA
DUND
EANG
LEEDS
OXF
ABRDN
GLASG
MANU
CARDF
SGEO
NEWC
SHEF
EDINB
BRIGH
LVRPL
QUEEN
QMARY
UCL
HLYK
LOND
KINGS
BIRM
CAM
NOTTM
SOTON
BRIST
LEICR
IMP

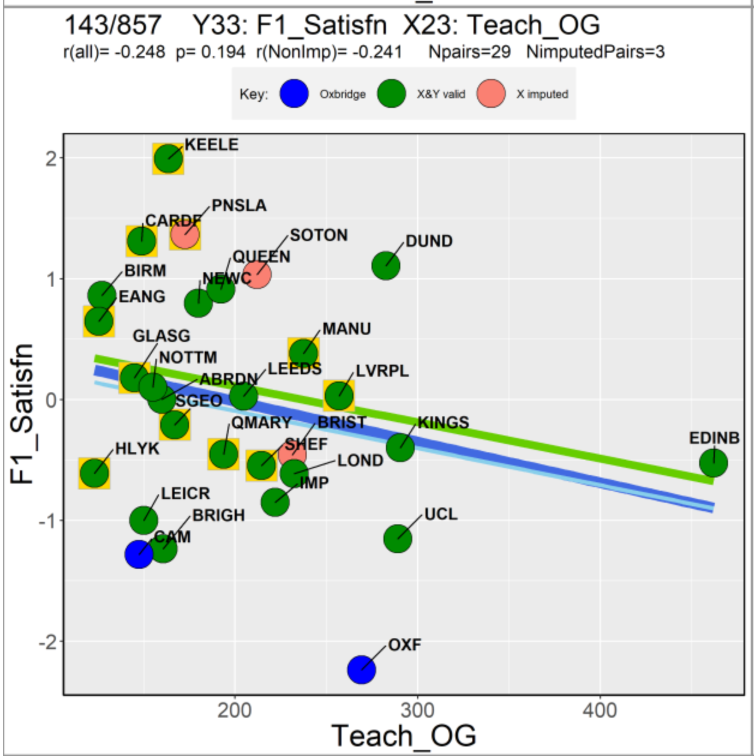
143/857 Y33: F1_Satisfn X23: Teach_OG
r(all)= -0.248 p= 0.194 r(NonImp)= -0.241 Npairs=29 NimputedPairs=3
Key: Oxbridge X&Y valid X imputed
F1_Satisfn
Teach_OG
2
1
0
-1
-2
200
300
400
KEELE
PNSLA
CARDF
SOTON
DUND
QUEEN
BIRM
NEWC
EANG
MANU
GLASG
NOTTM
LEEDS
LVRPL
ABRDN
SGEO
QMARY
BRIST
KINGS
EDINB
HLYK
SHEF
LOND
IMP
LEICR
BRIGH
UCL
CAM
OXF

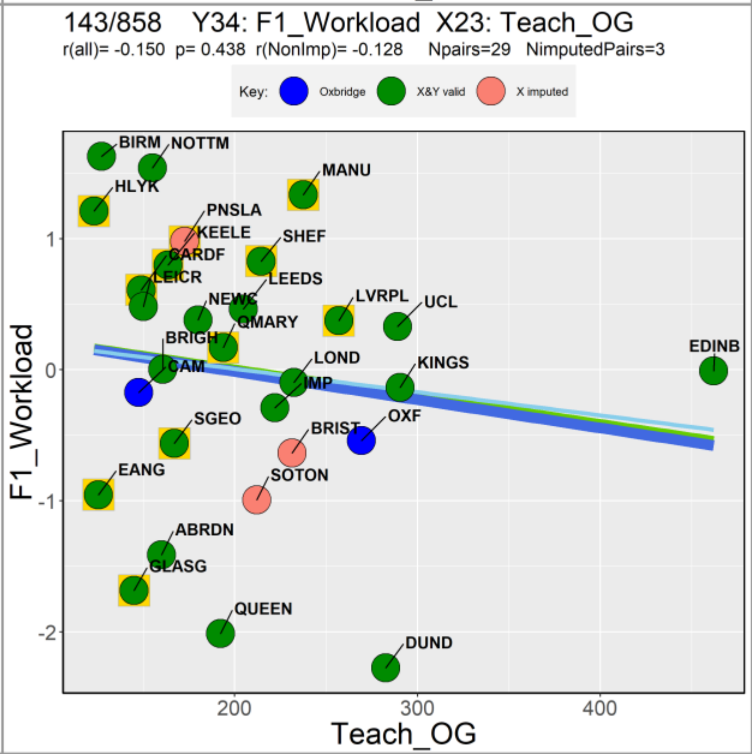
143/858 Y34: F1_Workload X23: Teach_OG
r(all)= -0.150 p= 0.438 r(NonImp)= -0.128 Npairs=29 NimputedPairs=3
Key: Oxbridge X&Y valid X imputed
F1_Workload
Teach_OG
1
0
-1
-2
200
300
400
BIRM
NOTTM
MANU
HLYK
PNSLA
KEELE
SHEF
CARDF
LEICR
LEEDS
NEWC
LVRPL
UCL
QMARY
BRIGH
EDINB
CAM
LOND
KINGS
IMP
SGEO
BRIST
OXF
EANG
SOTON
ABRDN
GLASG
QUEEN
DUND

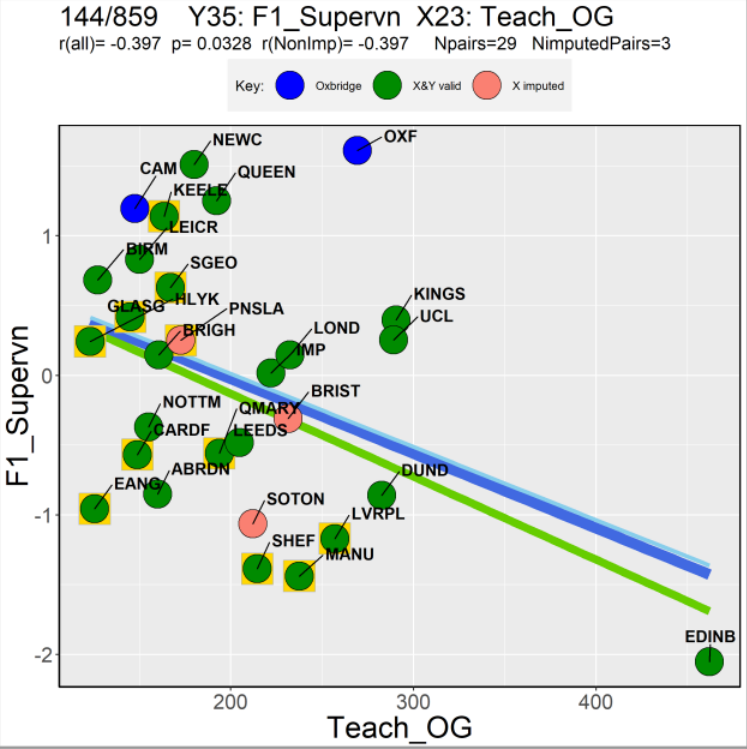
144/859 Y35: F1_Supervn X23: Teach_OG
r(all)= -0.397 p= 0.0328 r(NonImp)= -0.397 Npairs=29 NimputedPairs=3
Key: Oxbridge X&Y valid X imputed
F1_Supervn
Teach_OG

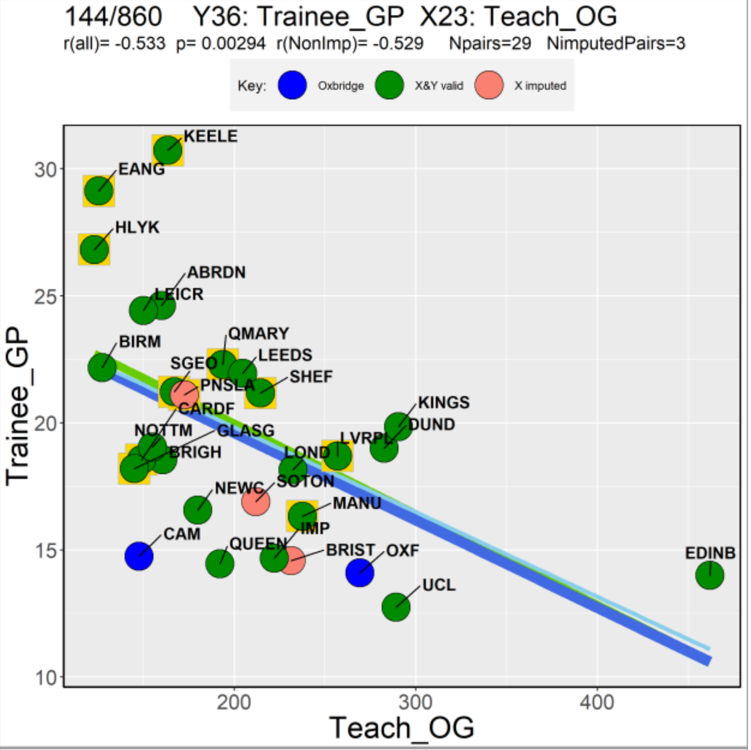
144/860 Y36: Trainee_GP X23: Teach_OG
r(all)= -0.533 p= 0.00294 r(NonImp)= -0.529 Npairs=29 NimputedPairs=3
Key: Oxbridge X&Y valid X imputed
Trainee_GP
Teach_OG

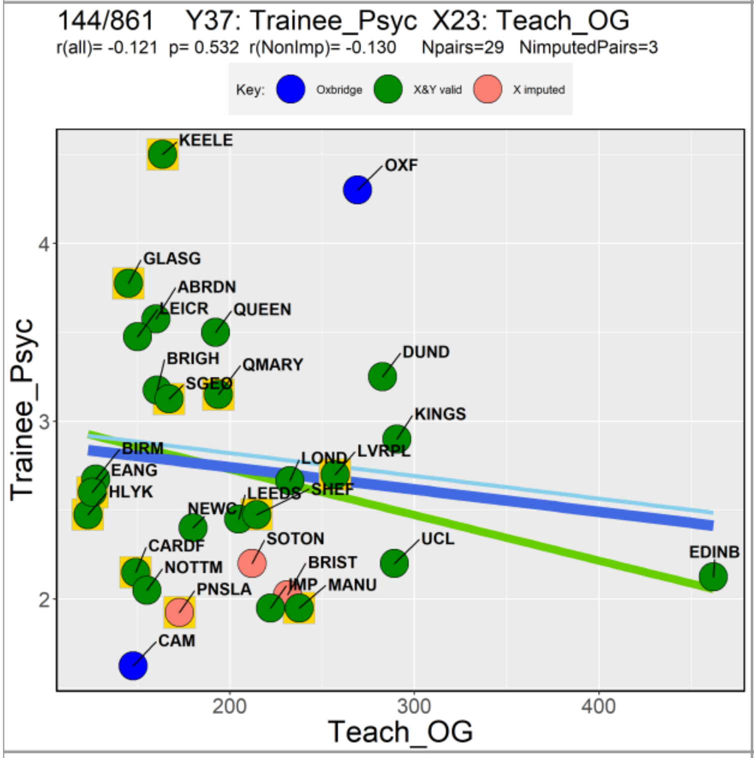
144/861 Y37: Trainee_Psyc X23: Teach_OG
r(all)= -0.121 p= 0.532 r(NonImp)= -0.130 Npairs=29 NimputedPairs=3
Key: Oxbridge X&Y valid X imputed
Trainee_Psyc
Teach_OG

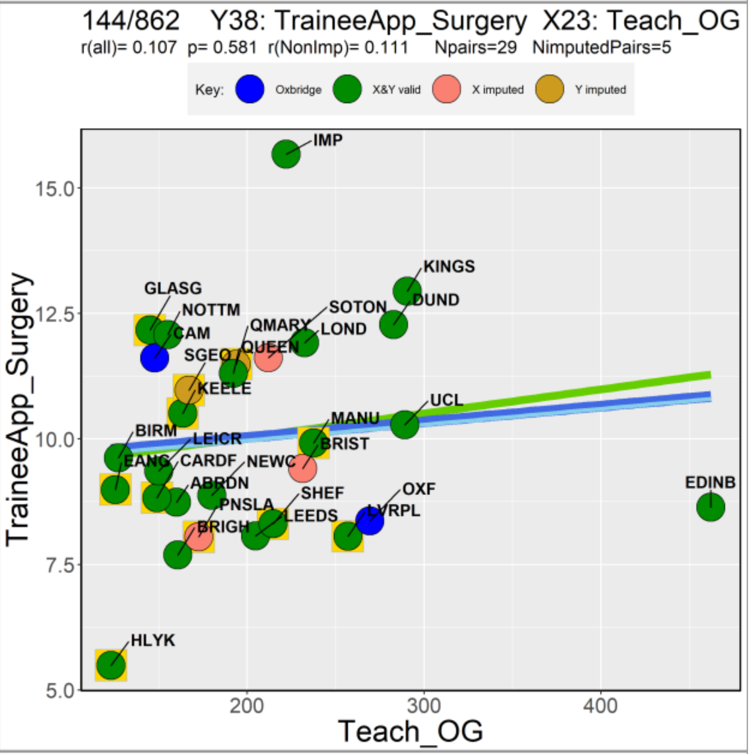
144/862 Y38: TraineeApp_Surgery X23: Teach_OG
r(all)= 0.107 p= 0.581 r(NonImp)= 0.111 Npairs=29 NimputedPairs=5
Key: Oxbridge X&Y valid X imputed Y imputed
TraineeApp_Surgery
Teach_OG

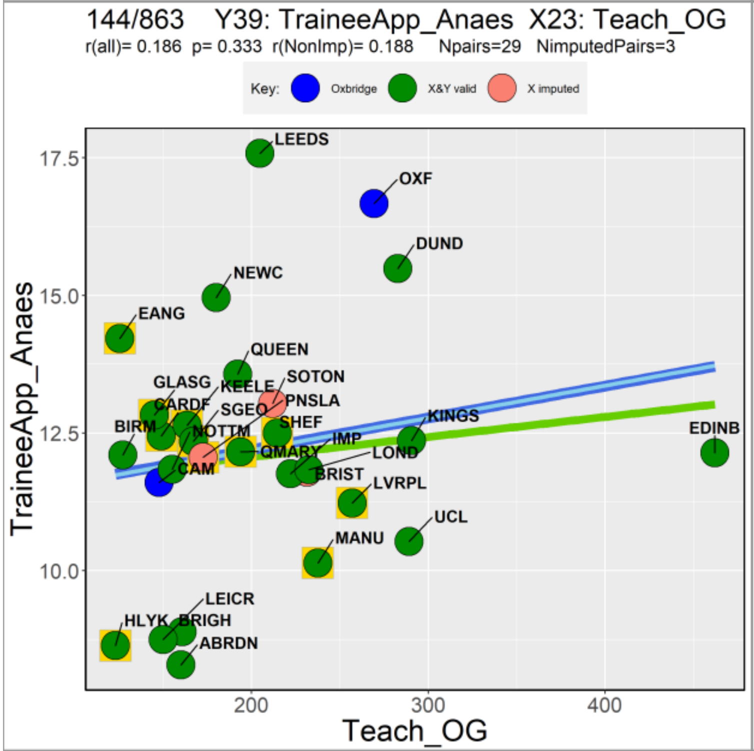
144/863 Y39: TraineeApp_Anaes X23: Teach_OG
r(all)= 0.186 p= 0.333 r(NonImp)= 0.188 Npairs=29 NimputedPairs=3
Key: Oxbridge X&Y valid X imputed
TraineeApp_Anaes
Teach_OG

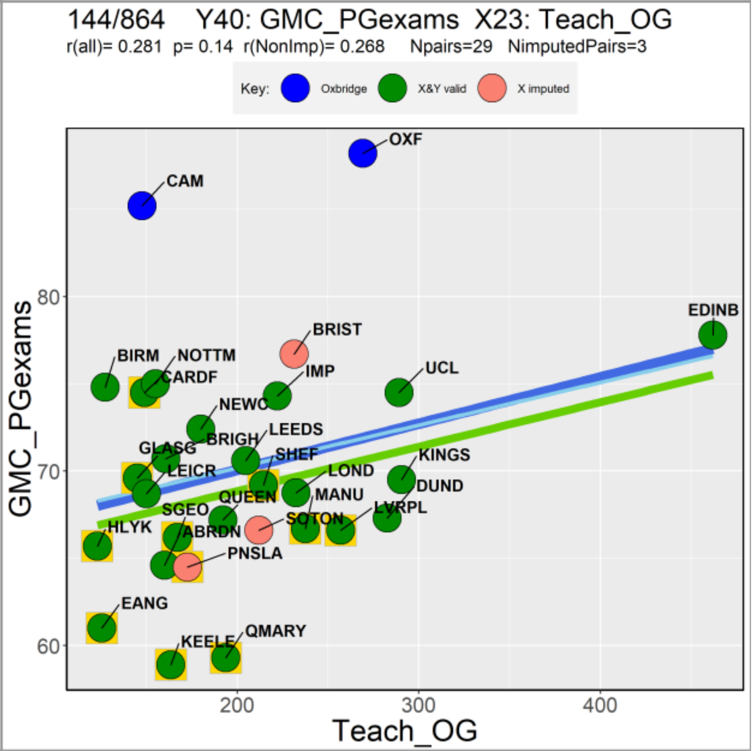
144/864 Y40: GMC_PGexams X23: Teach_OG
r(all)= 0.281 p= 0.14 r(NonImp)= 0.268 Npairs=29 NimputedPairs=3
Key: Oxbridge X&Y valid X imputed
GMC_PGexams
Teach_OG

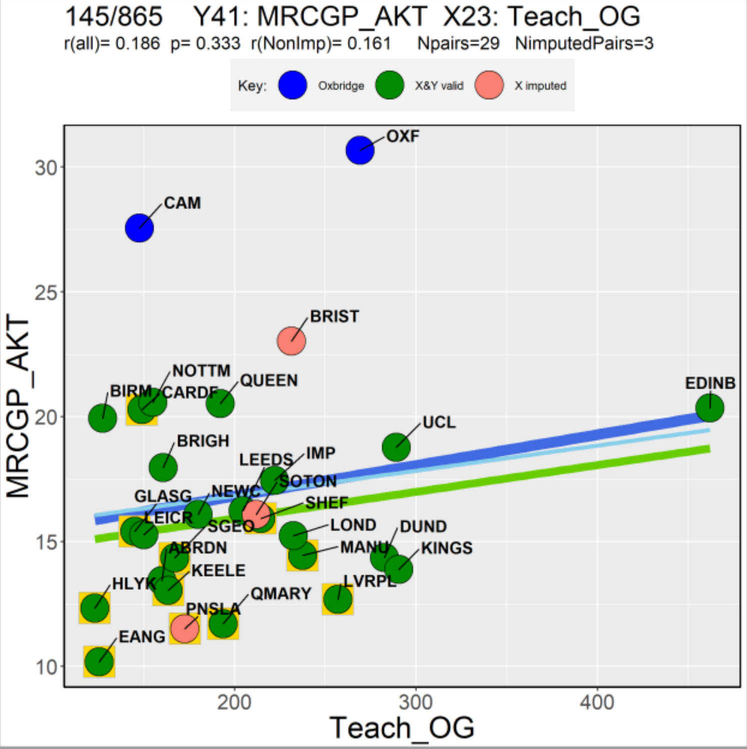
145/865 Y41: MRCGP_AKT X23: Teach_OG
r(all)= 0.186 p= 0.333 r(NonImp)= 0.161 Npairs=29 NimputedPairs=3
Key: Oxbridge X&Y valid X imputed
MRCGP_AKT
Teach_OG
OXF
CAM
BRIST
NOTTM
QUEEN
BIRM
CARDF
EDINB
UCL
BRIGH
LEEDS
IMP
SOTON
GLASG
NEWC
SHEF
LEICR
SGEO
LOND
DUND
ABRDN
MANU
KINGS
KEELE
HLYK
LVRPL
QMARY
PNSLA
EANG

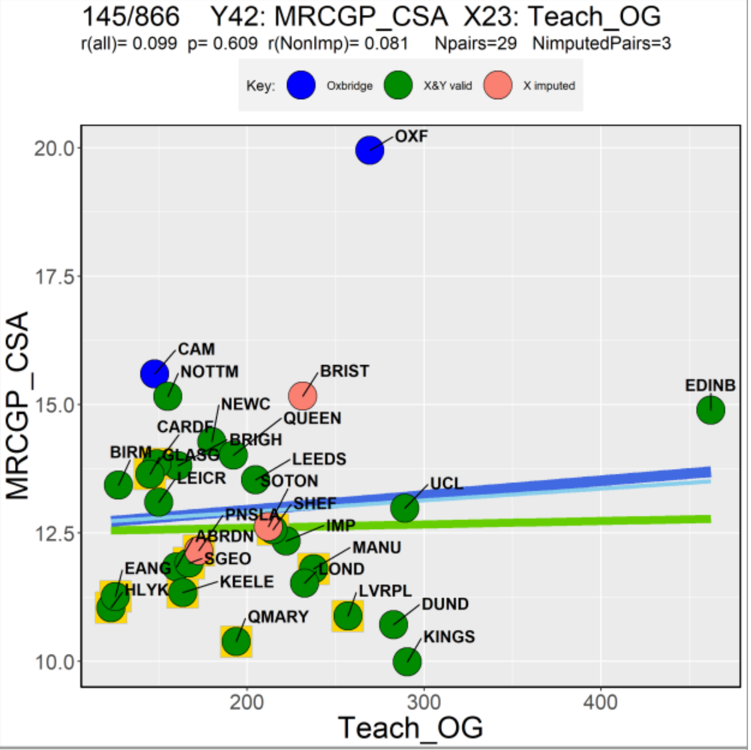
145/866 Y42: MRCGP_CSA X23: Teach_OG
r(all)= 0.099 p= 0.609 r(NonImp)= 0.081 Npairs=29 NimputedPairs=3
Key: Oxbridge X&Y valid X imputed
MRCGP_CSA
Teach_OG
OXF
CAM
NOTTM
BRIST
EDINB
NEWC
QUEEN
CARDF
BRIGH
BIRM
GLASG
LEEDS
LEICR
SOTON
UCL
SHEF
PNSLA
IMP
ABRDN
MANU
SGEO
EANG
LOND
HLYK
KEELE
LVRPL
DUND
QMARY
KINGS

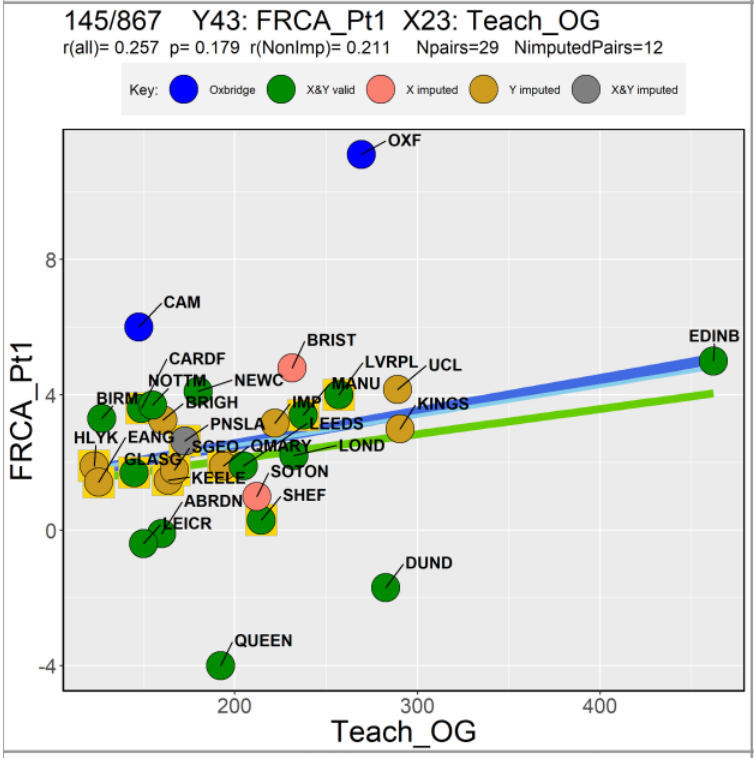
145/867 Y43: FRCA_Pt1 X23: Teach_OG
r(all)= 0.257 p= 0.179 r(NonImp)= 0.211 Npairs=29 NimputedPairs=12
Key: Oxbridge X&Y valid X imputed Y imputed X&Y imputed
FRCA_Pt1
Teach_OG
OXF
CAM
EDINB
BRIST
CARDF
LVRPL
UCL
NOTTM
NEWC
MANU
BIRM
BRIGH
IMP
KINGS
PNSLA
LEEDS
HLYK
EANG
GLASG
SGEO
QMARY
LOND
KEELE
SOTON
ABRDN
SHEF
LEICR
DUND
QUEEN

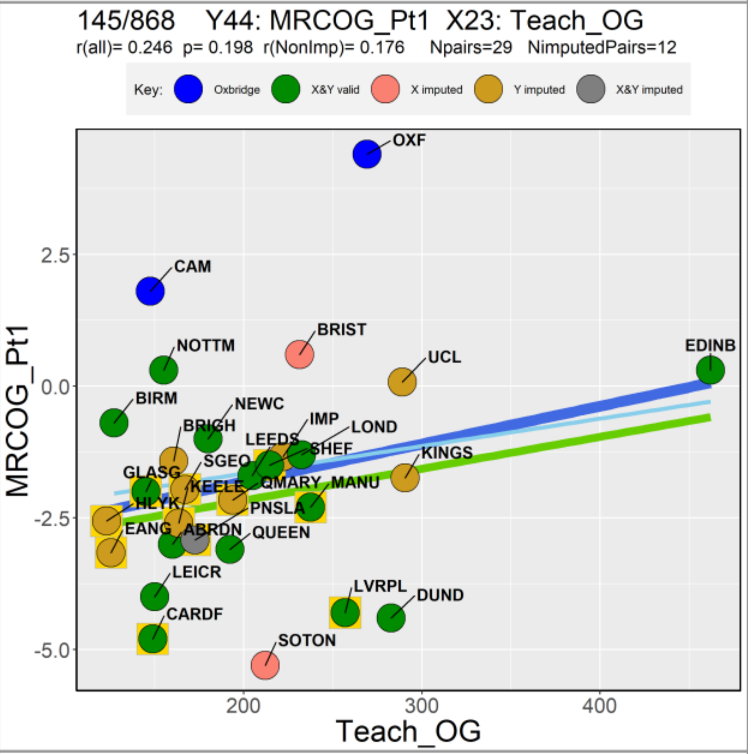
145/868 Y44: MRCOG_Pt1 X23: Teach_OG
r(all)= 0.246 p= 0.198 r(NonImp)= 0.176 Npairs=29 NimputedPairs=12
Key: Oxbridge X&Y valid X imputed Y imputed X&Y imputed
MRCOG_Pt1
Teach_OG
OXF
CAM
BRIST
NOTTM
UCL
EDINB
BIRM
NEWC
IMP
BRIGH
LOND
LEEDS
SHEF
SGEO
KINGS
GLASG
KEELE
QMARY
MANU
HLYK
PNSLA
EANG
ABRDN
QUEEN
LEICR
LVRPL
DUND
CARDF
SOTON

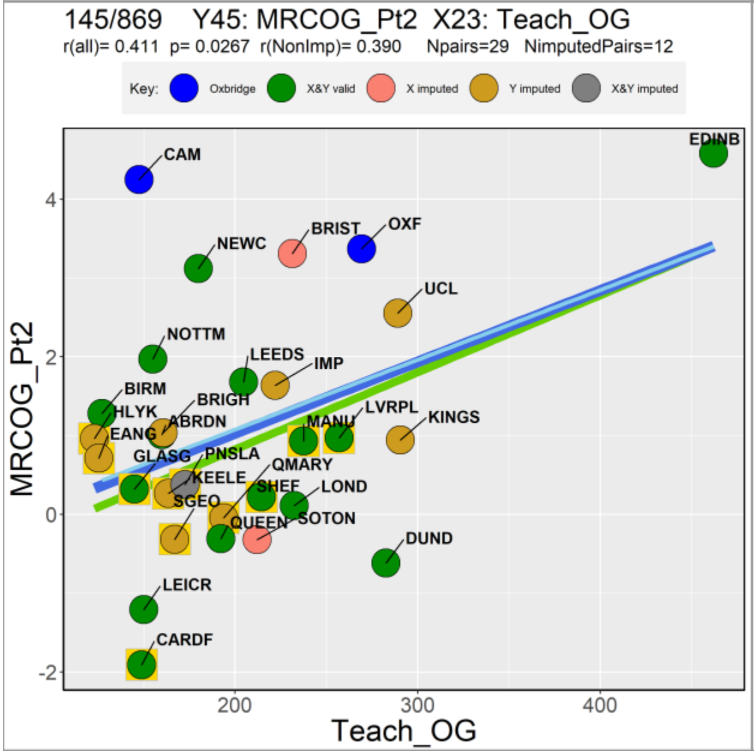
145/869 Y45: MRCOG_Pt2 X23: Teach_OG
r(all)= 0.411 p= 0.0267 r(NonImp)= 0.390 Npairs=29 NimputedPairs=12
Key: Oxbridge X&Y valid X imputed Y imputed X&Y imputed
MRCOG_Pt2
Teach_OG
EDINB
CAM
NEWC
BRIST
OXF
UCL
NOTTM
LEEDS
IMP
BIRM
BRIGH
HLYK
ABRDN
MANU
LVRPL
KINGS
EANG
GLASG
PNSLA
QMARY
KEELE
SHEF
LOND
SGEO
QUEEN
SOTON
DUND
LEICR
CARDF

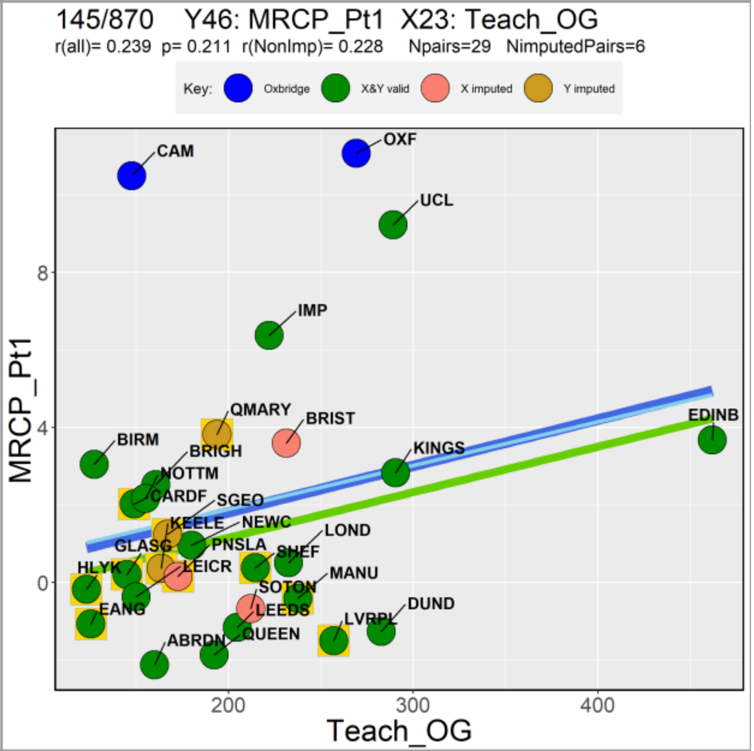
145/870 Y46: MRCP_Pt1 X23: Teach_OG
r(all)= 0.239 p= 0.211 r(NonImp)= 0.228 Npairs=29 NimputedPairs=6
Key: Oxbridge X&Y valid X imputed Y imputed
MRCP_Pt1
Teach_OG
OXF
CAM
UCL
IMP
QMARY
BRIST
EDINB
BIRM
BRIGH
KINGS
NOTTM
CARDF
SGEO
KEELE
NEWC
LOND
GLASG
PNSLA
SHEF
HLYK
LEICR
MANU
SOTON
EANG
LEEDS
DUND
LVRPL
ABRDN
QUEEN

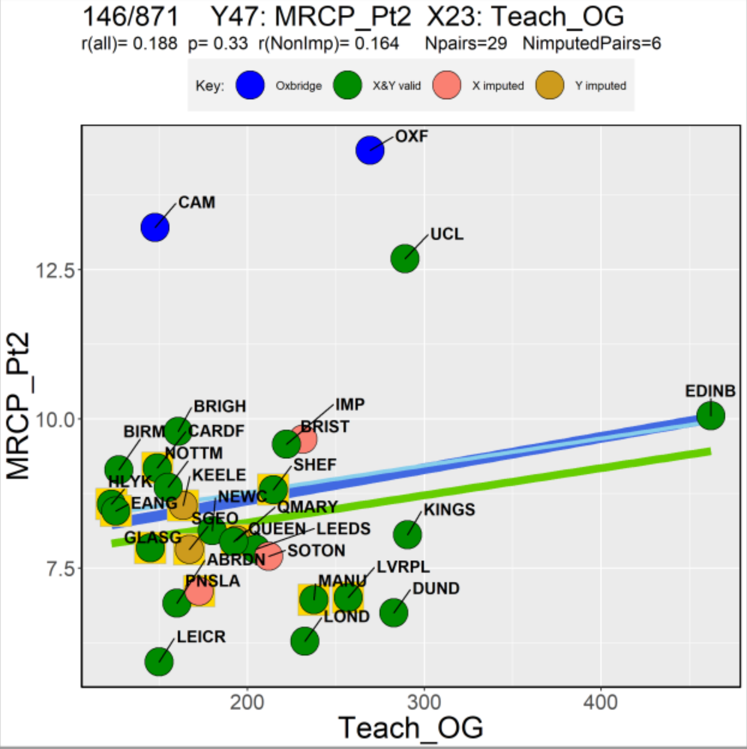
146/871 Y47: MRCP_Pt2 X23: Teach_OG
r(all)= 0.188 p= 0.33 r(NonImp)= 0.164 Npairs=29 NimputedPairs=6
Key: Oxbridge X&Y valid X imputed Y imputed
MRCP_Pt2
Teach_OG
12.5
10.0
7.5
200
300
400
OXF
CAM
UCL
EDINB
BRIGH
IMP
BIRM
CARDF
BRIST
NOTTM
SHEF
HLYK
KEELE
EANG
NEWC
QMARY
SGEO
QUEEN
LEEDS
KINGS
GLASG
SOTON
ABRDN
LVRPL
PNSLA
MANU
DUND
LOND
LEICR

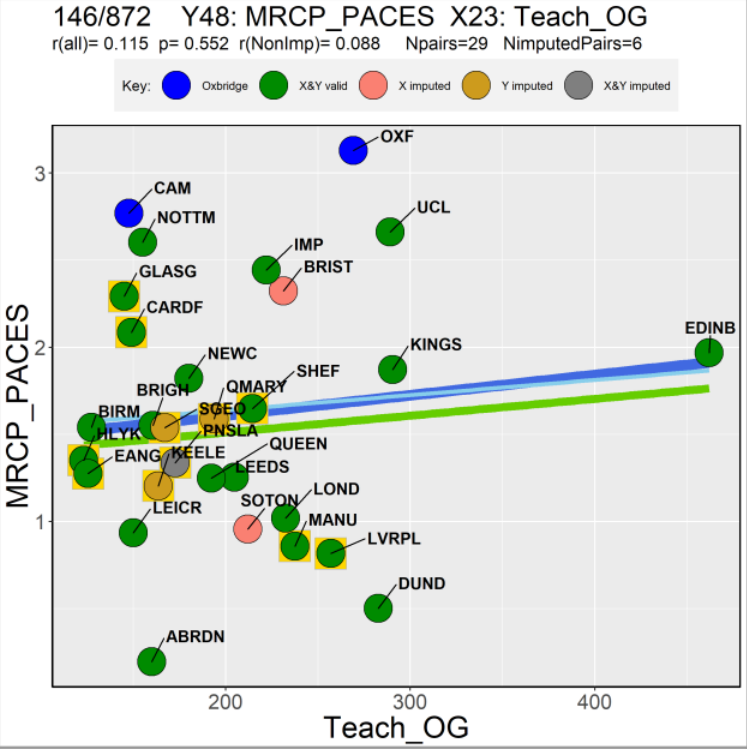
146/872 Y48: MRCP_PACES X23: Teach_OG
r(all)= 0.115 p= 0.552 r(NonImp)= 0.088 Npairs=29 NimputedPairs=6
Key: Oxbridge X&Y valid X imputed Y imputed X&Y imputed
MRCP_PACES
Teach_OG
3
2
1
200
300
400
OXF
CAM
NOTTM
UCL
IMP
GLASG
BRIST
CARDF
EDINB
NEWC
KINGS
SHEF
BRIGH
QMARY
BIRM
SGEO
HLYK
PNSLA
EANG
QUEEN
KEELE
LEEDS
LOND
SOTON
LEICR
MANU
LVRPL
DUND
ABRDN

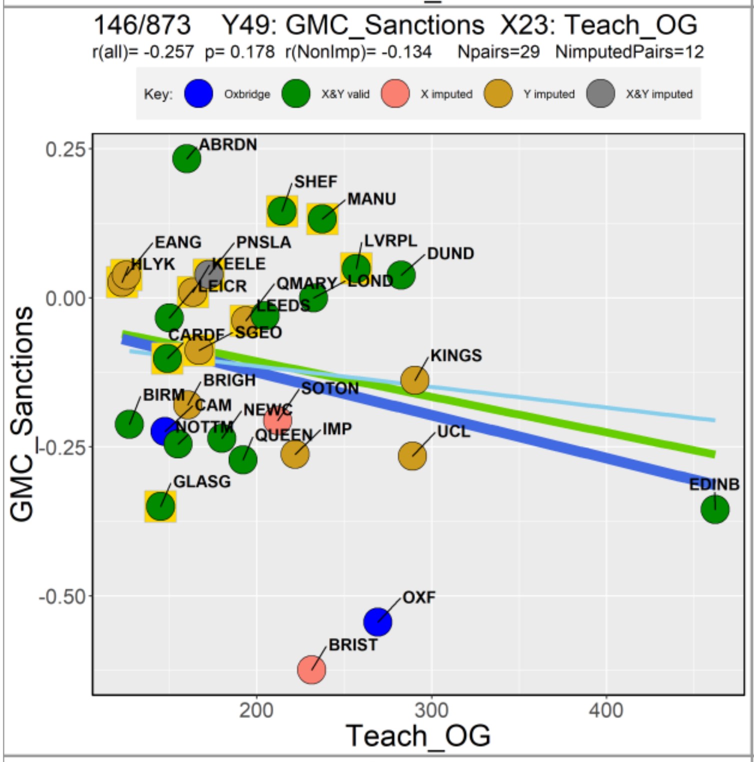
146/873 Y49: GMC_Sanctions X23: Teach_OG
r(all)= -0.257 p= 0.178 r(NonImp)= -0.134 Npairs=29 NimputedPairs=12
Key: Oxbridge X&Y valid X imputed Y imputed X&Y imputed
GMC_Sanctions
Teach_OG
0.25
0.00
-0.25
-0.50
200
300
400
ABRDN
SHEF
MANU
EANG
PNSLA
LVRPL
DUND
HLYK
KEELE
LEICR
QMARY
LOND
LEEDS
CARDF
SGEO
KINGS
BRIGH
SOTON
BIRM
CAM
NEWC
NOTTM
QUEEN
IMP
UCL
GLASG
EDINB
OXF
BRIST

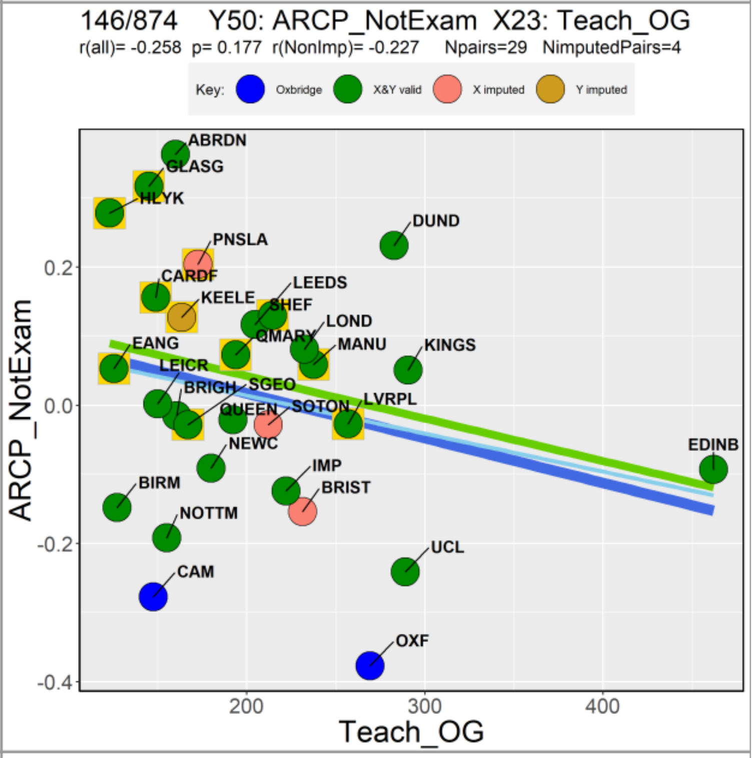
146/874 Y50: ARCP_NotExam X23: Teach_OG
r(all)= -0.258 p= 0.177 r(NonImp)= -0.227 Npairs=29 NimputedPairs=4
Key: Oxbridge X&Y valid X imputed Y imputed
ARCP_NotExam
Teach_OG
0.2
0.0
-0.2
-0.4
200
300
400
ABRDN
GLASG
HLYK
DUND
PNSLA
CARDF
LEEDS
KEELE
SHEF
LOND
QMARY
EANG
MANU
KINGS
LEICR
BRIGH
SGEO
QUEEN
SOTON
LVRPL
NEWC
EDINB
IMP
BIRM
BRIST
NOTTM
UCL
CAM
OXF

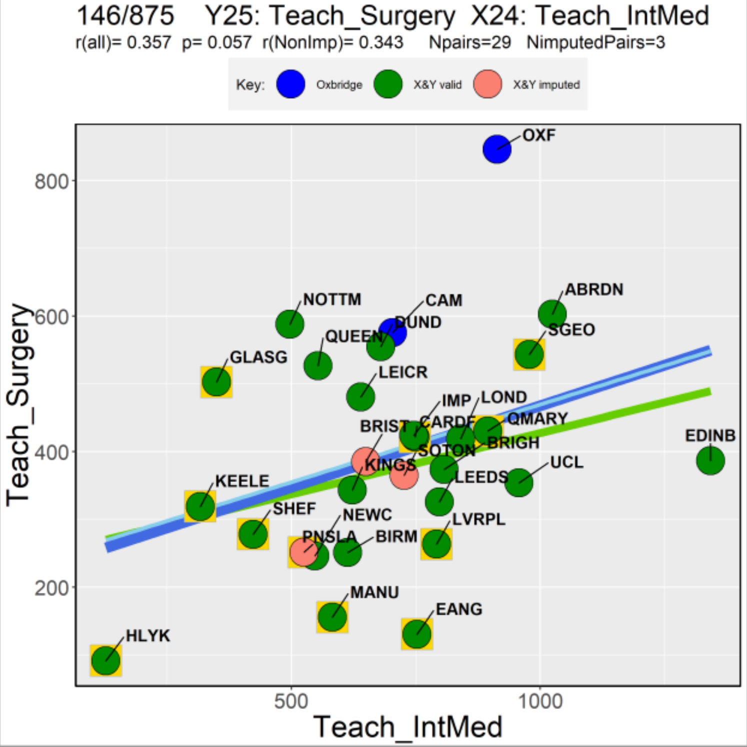
146/875 Y25: Teach_Surgery X24: Teach_IntMed
r(all)= 0.357 p= 0.057 r(NonImp)= 0.343 Npairs=29 NimputedPairs=3
Key: Oxbridge X&Y valid X&Y imputed
Teach_Surgery
Teach_IntMed
800
600
400
200
500
1000
OXF
NOTTM
CAM
ABRDN
QUEEN
DUND
SGEO
GLASG
LEICR
IMP
LOND
BRIST
CARDF
QMARY
EDINB
KINGS
BRIGH
SOTON
KEELE
LEEDS
UCL
SHEF
NEWC
LVRPL
PNSLA
BIRM
MANU
EANG
HLYK

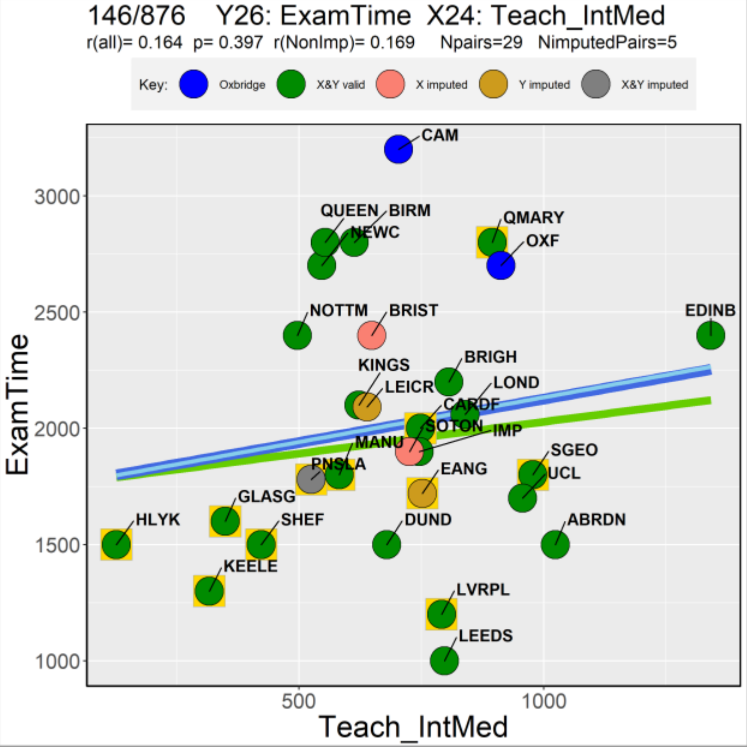
146/876 Y26: ExamTime X24: Teach_IntMed
r(all)= 0.164 p= 0.397 r(NonImp)= 0.169 Npairs=29 NimputedPairs=5
Key: Oxbridge X&Y valid X imputed Y imputed X&Y imputed
ExamTime
Teach_IntMed
3000
2500
2000
1500
1000
500
1000
CAM
QUEEN
BIRM
QMARY
NEWC
OXF
NOTTM
BRIST
EDINB
KINGS
BRIGH
LEICR
LOND
CARDF
SOTON
IMP
MANU
SGEO
PNSLA
EANG
UCL
GLASG
HLYK
SHEF
DUND
ABRDN
KEELE
LVRPL
LEEDS

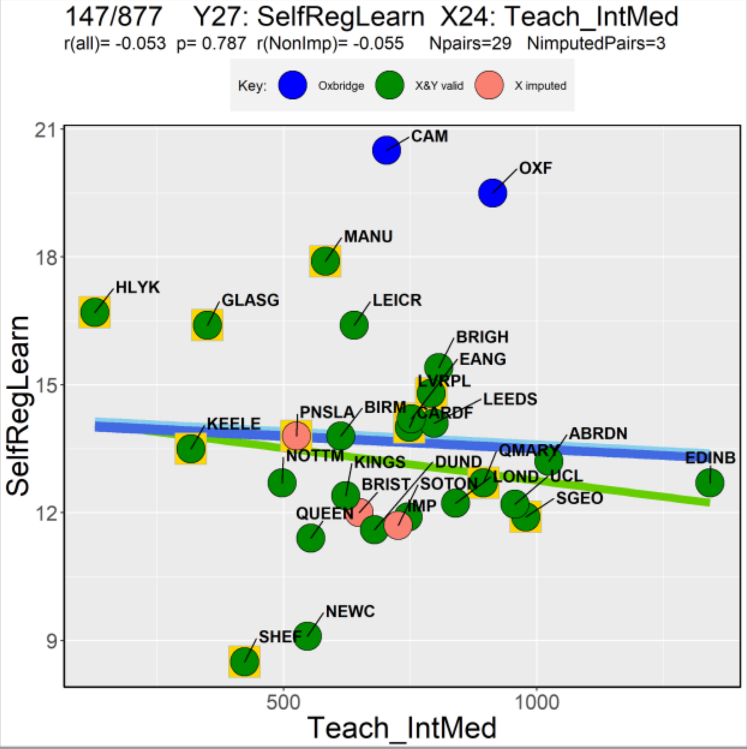
147/877 Y27: SelfRegLearn X24: Teach_IntMed
r(all)= -0.053 p= 0.787 r(NonImp)= -0.055 Npairs=29 NimputedPairs=3
Key: Oxbridge X&Y valid X imputed
SelfRegLearn
Teach_IntMed
CAM
OXF
MANU
HLYK
GLASG
LEICR
BRIGH
EANG
LVRPL
LEEDS
PNSLA
BIRM
CARDF
KEELE
ABRDN
NOTTM
KINGS
DUND
QMARY
EDINB
BRIST
SOTON
LOND
UCL
QUEEN
IMP
SGEO
NEWC
SHEF

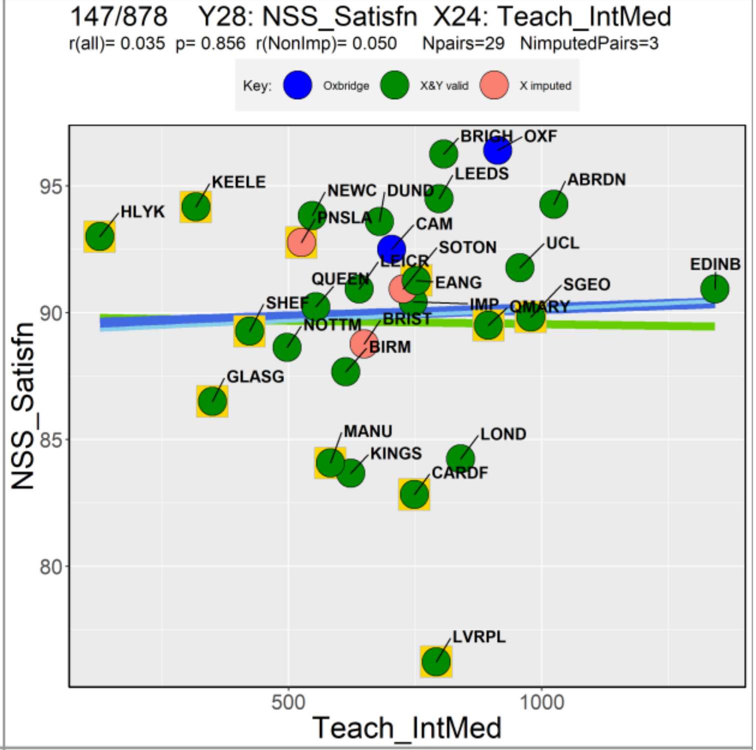
147/878 Y28: NSS_Satisfn X24: Teach_IntMed
r(all)= 0.035 p= 0.856 r(NonImp)= 0.050 Npairs=29 NimputedPairs=3
Key: Oxbridge X&Y valid X imputed
NSS_Satisfn
Teach_IntMed
BRIGH
OXF
KEELE
NEWC
DUND
LEEDS
ABRDN
HLYK
PNSLA
CAM
SOTON
UCL
LEICR
EDINB
QUEEN
EANG
SGEO
SHEF
IMP
QMARY
NOTTM
BRIST
BIRM
GLASG
MANU
LOND
KINGS
CARDF
LVRPL

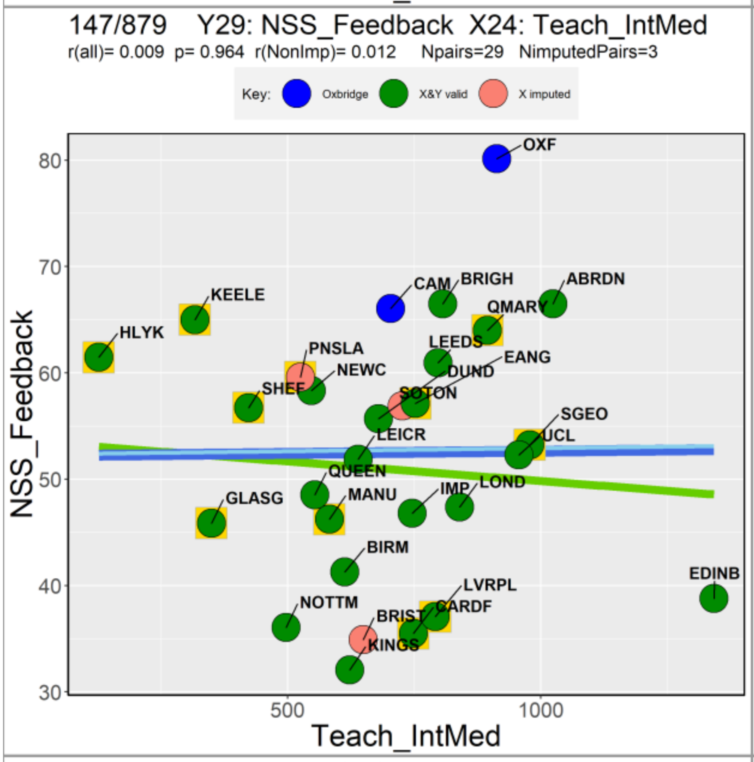
147/879 Y29: NSS_Feedback X24: Teach_IntMed
r(all)= 0.009 p= 0.964 r(NonImp)= 0.012 Npairs=29 NimputedPairs=3
Key: Oxbridge X&Y valid X imputed
NSS_Feedback
Teach_IntMed
OXF
CAM
BRIGH
ABRDN
KEELE
QMARY
HLYK
LEEDS
PNSLA
EANG
NEWC
DUND
SHEF
SOTON
SGEO
LEICR
UCL
QUEEN
GLASG
MANU
IMP
LOND
BIRM
EDINB
LVRPL
NOTTM
BRIST
CARDF
KINGS

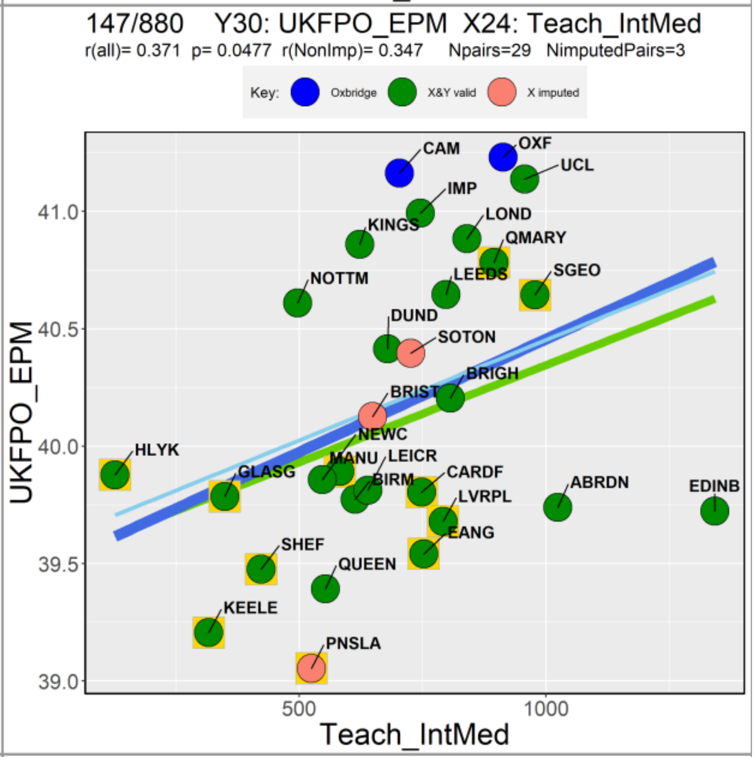
147/880 Y30: UKFPO_EPM X24: Teach_IntMed
r(all)= 0.371 p= 0.0477 r(NonImp)= 0.347 Npairs=29 NimputedPairs=3
Key: Oxbridge X&Y valid X imputed
UKFPO_EPM
Teach_IntMed
CAM
OXF
UCL
IMP
LOND
KINGS
QMARY
NOTTM
LEEDS
SGEO
DUND
SOTON
BRIGH
BRIST
NEWC
HLYK
MANU
LEICR
GLASG
CARDF
BIRM
LVRPL
ABRDN
EDINB
EANG
SHEF
QUEEN
KEELE
PNSLA

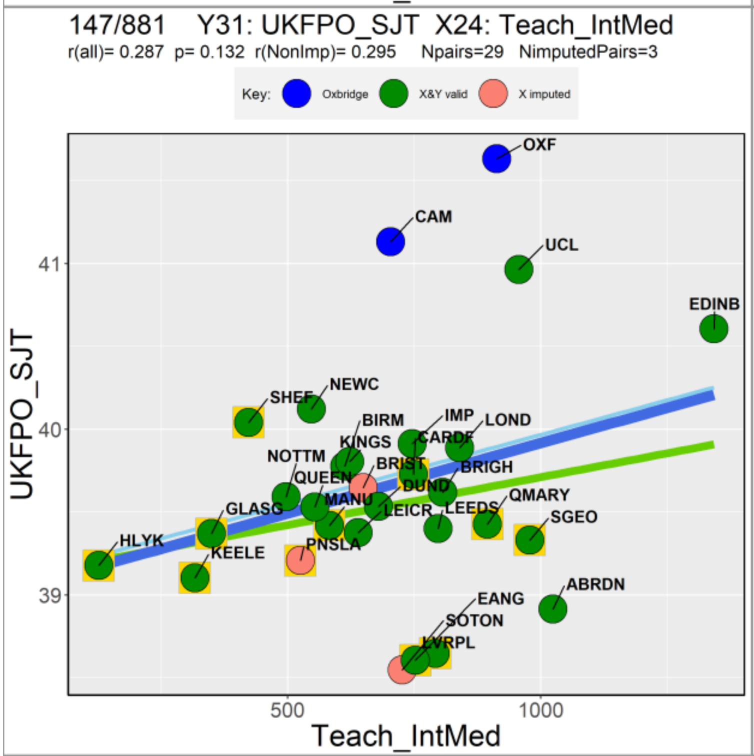
147/881 Y31: UKFPO_SJT X24: Teach_IntMed
r(all)= 0.287 p= 0.132 r(NonImp)= 0.295 Npairs=29 NimputedPairs=3
Key: Oxbridge X&Y valid X imputed
UKFPO_SJT
Teach_IntMed
OXF
CAM
UCL
EDINB
SHEF
NEWC
BIRM
IMP
LOND
KINGS
CARDF
NOTTM
BRIST
BRIGH
QUEEN
DUND
MANU
GLASG
LEICR
LEEDS
QMARY
SGEO
HLYK
KEELE
PNSLA
EANG
ABRDN
SOTON
LVRPL

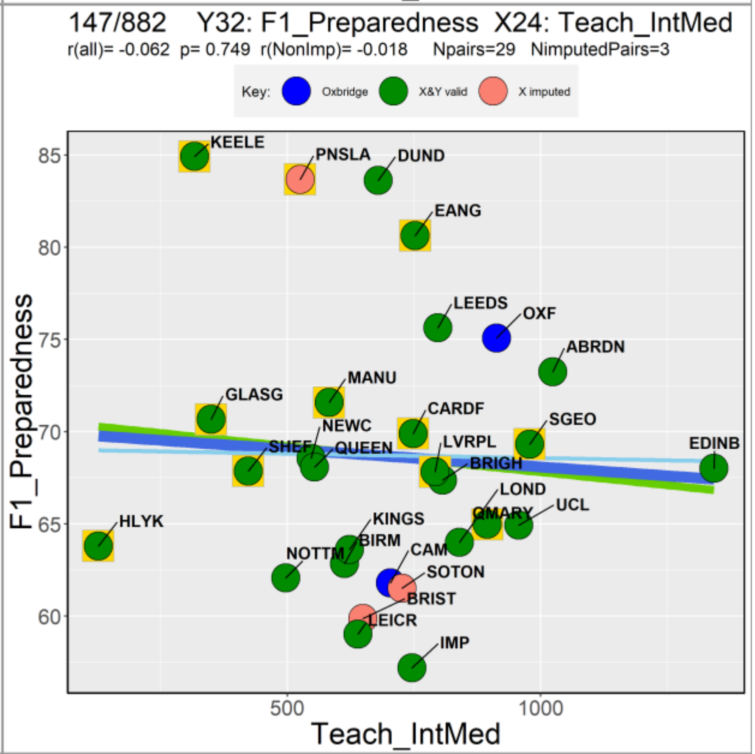
147/882 Y32: F1_Preparedness X24: Teach_IntMed
r(all)= -0.062 p= 0.749 r(NonImp)= -0.018 Npairs=29 NimputedPairs=3
Key: Oxbridge X&Y valid X imputed
F1_Preparedness
Teach_IntMed
KEELE
PNSLA
DUND
EANG
LEEDS
OXF
ABRDN
MANU
GLASG
CARDF
NEWC
SGEO
SHEF
QUEEN
LVRPL
EDINB
BRIGH
LOND
UCL
HLYK
KINGS
QMARY
BIRM
CAM
NOTTM
SOTON
BRIST
LEICR
IMP

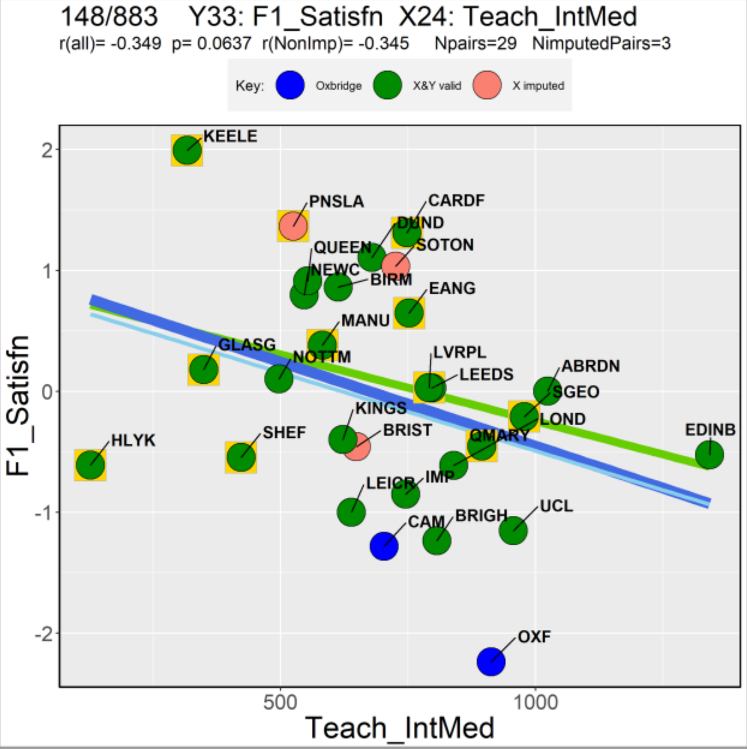
148/883 Y33: F1_Satisfn X24: Teach_IntMed
r(all)= -0.349 p= 0.0637 r(NonImp)= -0.345 Npairs=29 NimputedPairs=3
Key: Oxbridge X&Y valid X imputed
F1_Satisfn
Teach_IntMed
KEELE
PNSLA
CARDF
DUND
QUEEN
SOTON
NEWC
BIRM
EANG
MANU
GLASG
NOTTM
LVRPL
LEEDS
ABRDN
SGEO
KINGS
LOND
HLYK
SHEF
BRIST
QMARY
EDINB
LEICR
IMP
CAM
BRIGH
UCL
OXF

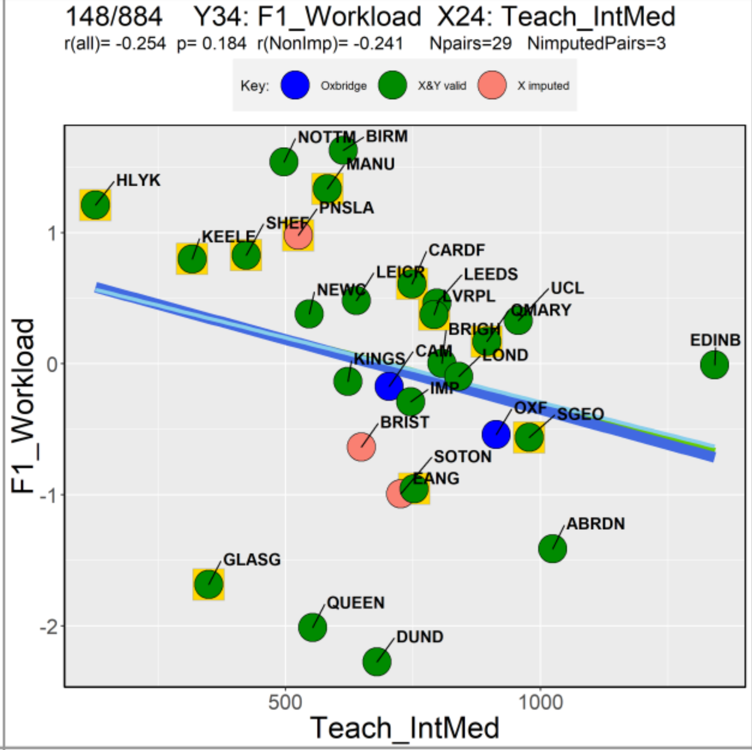
148/884 Y34: F1_Workload X24: Teach_IntMed
r(all)= -0.254 p= 0.184 r(NonImp)= -0.241 Npairs=29 NimputedPairs=3
Key: Oxbridge X&Y valid X imputed
F1_Workload
Teach_IntMed
NOTTM
BIRM
MANU
HLYK
PNSLA
KEELE
SHEF
CARDF
LEICR
LEEDS
NEWC
UCL
LVRPL
QMARY
BRIGH
EDINB
KINGS
CAM
LOND
IMP
OXF
SGEO
BRIST
SOTON
EANG
ABRDN
GLASG
QUEEN
DUND

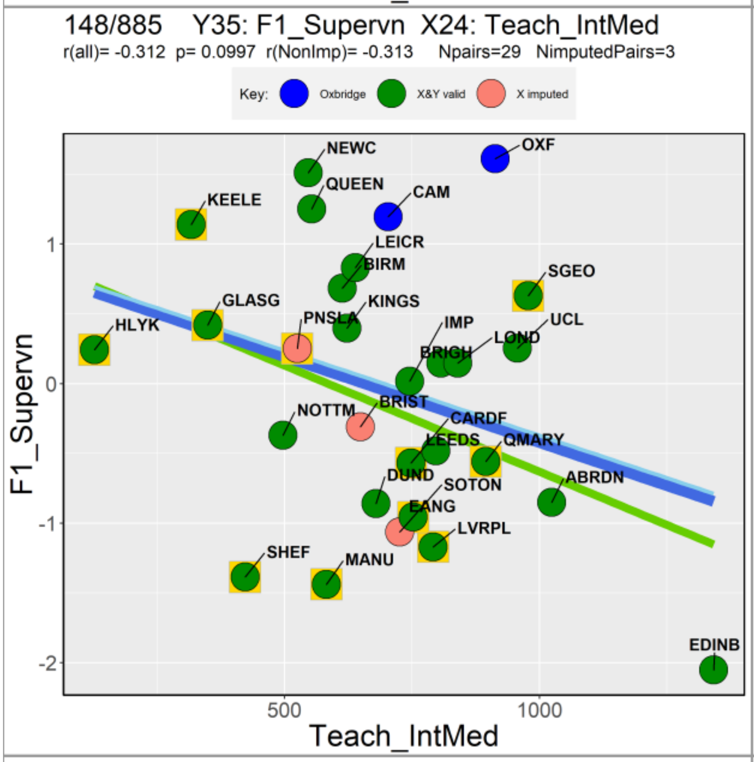
148/885 Y35: F1_Supervn X24: Teach_IntMed
r(all)= -0.312 p= 0.0997 r(NonImp)= -0.313 Npairs=29 NimputedPairs=3
Key: Oxbridge X&Y valid X imputed
F1_Supervn
Teach_IntMed
NEWC
OXF
QUEEN
CAM
KEELE
LEICR
BIRM
SGEO
GLASG
KINGS
HLYK
PNSLA
IMP
UCL
LOND
BRIGH
NOTTM
BRIST
CARDF
LEEDS
QMARY
DUND
SOTON
ABRDN
EANG
LVRPL
SHEF
MANU
EDINB

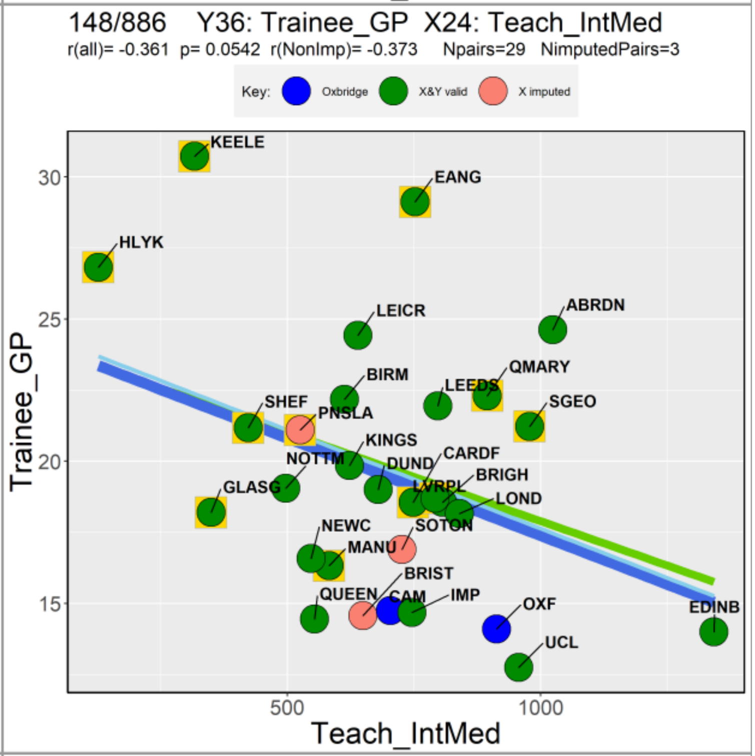
148/886 Y36: Trainee_GP X24: Teach_IntMed
r(all)= -0.361 p= 0.0542 r(NonImp)= -0.373 Npairs=29 NimputedPairs=3
Key: Oxbridge X&Y valid X imputed
Trainee_GP
Teach_IntMed
KEELE
EANG
HLYK
LEICR
ABRDN
QMARY
BIRM
LEEDS
SHEF
PNSLA
SGEO
KINGS
NOTTM
DUND
CARDF
GLASG
LVRPL
BRIGH
LOND
NEWC
SOTON
MANU
BRIST
QUEEN
CAM
IMP
OXF
EDINB
UCL

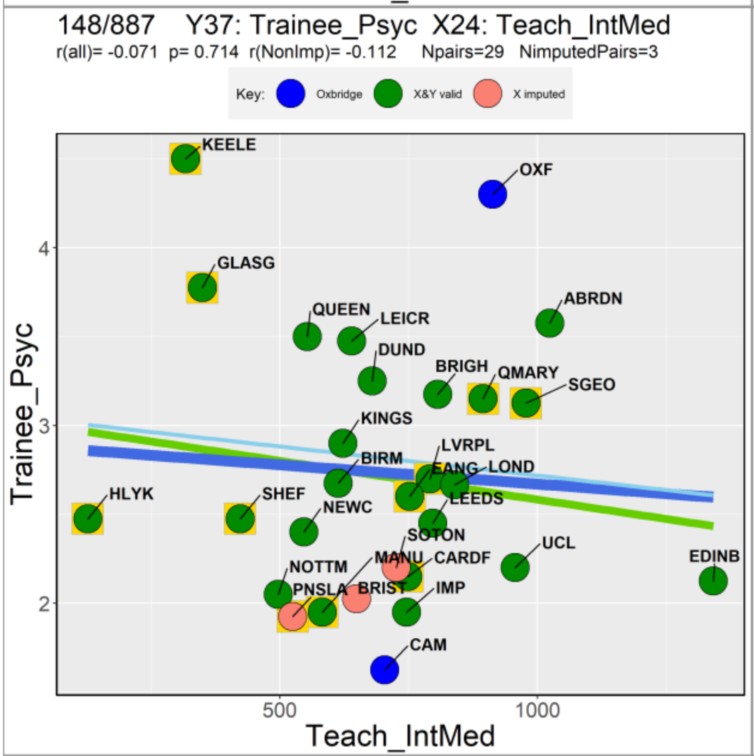
148/887 Y37: Trainee_Psyc X24: Teach_IntMed
r(all)= -0.071 p= 0.714 r(NonImp)= -0.112 Npairs=29 NimputedPairs=3
Key: Oxbridge X&Y valid X imputed
Trainee_Psyc
Teach_IntMed
KEELE
OXF
GLASG
QUEEN
LEICR
ABRDN
DUND
BRIGH
QMARY
SGEO
KINGS
LVRPL
BIRM
EANG
LOND
HLYK
SHEF
LEEDS
NEWC
SOTON
UCL
MANU
CARDF
EDINB
NOTTM
PNSLA
BRIST
IMP
CAM

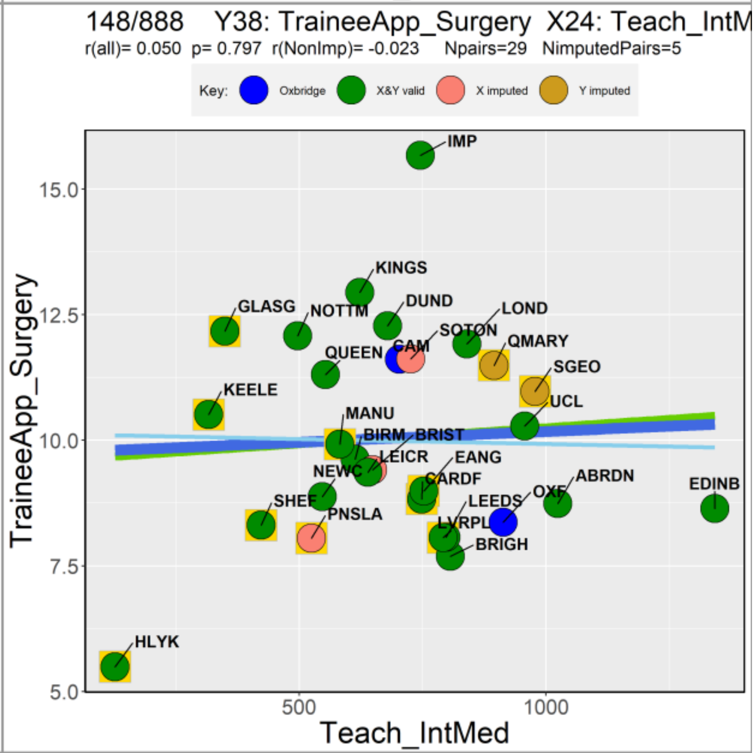
148/888 Y38: TraineeApp_Surgery X24: Teach_IntM
r(all)= 0.050 p= 0.797 r(NonImp)= -0.023 Npairs=29 NimputedPairs=5
Key: Oxbridge X&Y valid X imputed Y imputed
TraineeApp_Surgery
Teach_IntMed
IMP
KINGS
GLASG
NOTTM
DUND
LOND
SOTON
QUEEN
CAM
QMARY
SGEO
KEELE
UCL
MANU
BIRM
BRIST
LEICR
EANG
NEWC
CARDF
ABRDN
SHEF
LEEDS
OXF
EDINB
PNSLA
LVRPL
BRIGH
HLYK

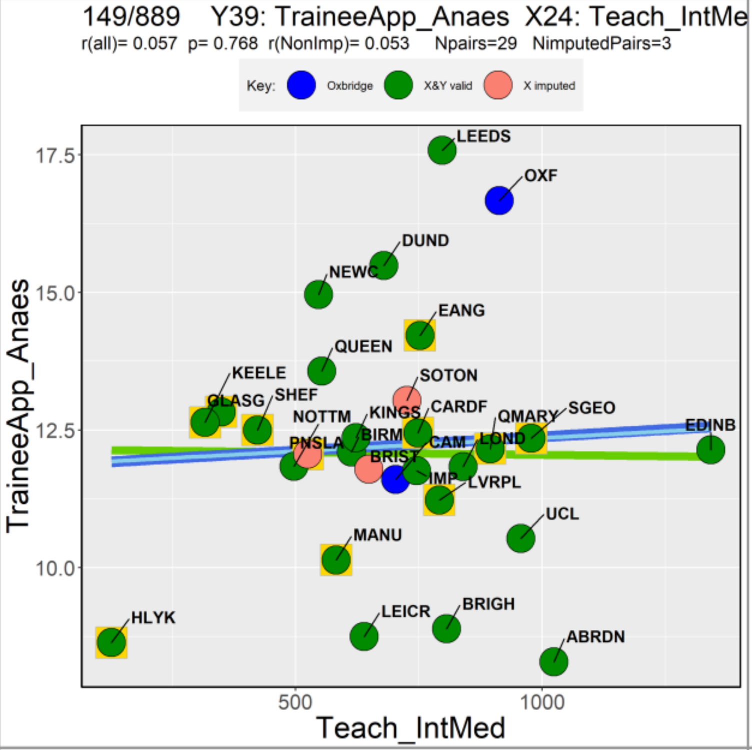
149/889 Y39: TraineeApp_Anaes X24: Teach_IntMe
r(all)= 0.057 p= 0.768 r(NonImp)= 0.053 Npairs=29 NimputedPairs=3
Key: Oxbridge X&Y valid X imputed
TraineeApp_Anaes
Teach_IntMed

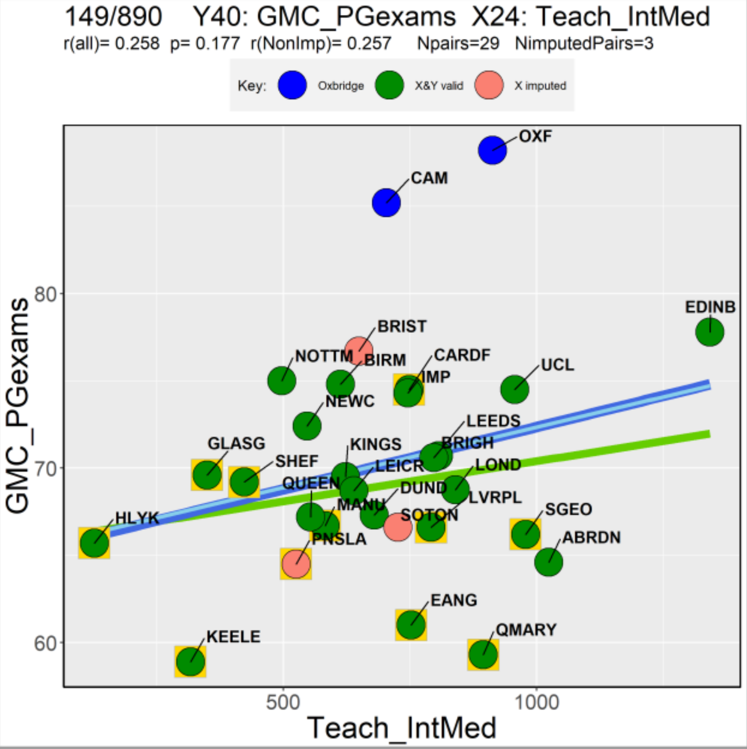
149/890 Y40: GMC_PGexams X24: Teach_IntMed
r(all)= 0.258 p= 0.177 r(NonImp)= 0.257 Npairs=29 NimputedPairs=3
Key: Oxbridge X&Y valid X imputed
GMC_PGexams
Teach_IntMed

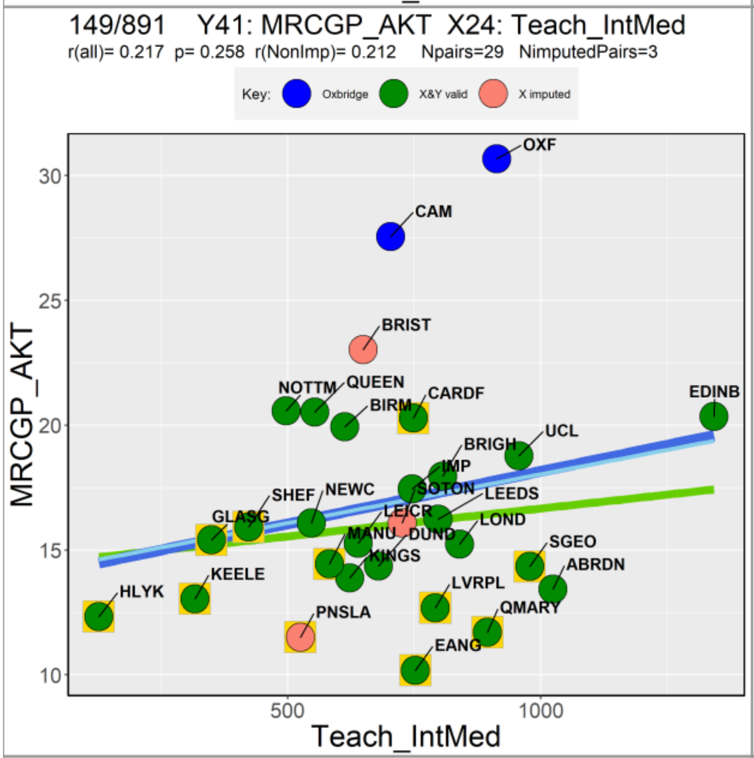
149/891 Y41: MRCGP_AKT X24: Teach_IntMed
r(all)= 0.217 p= 0.258 r(NonImp)= 0.212 Npairs=29 NimputedPairs=3
Key: Oxbridge X&Y valid X imputed
MRCGP_AKT
Teach_IntMed

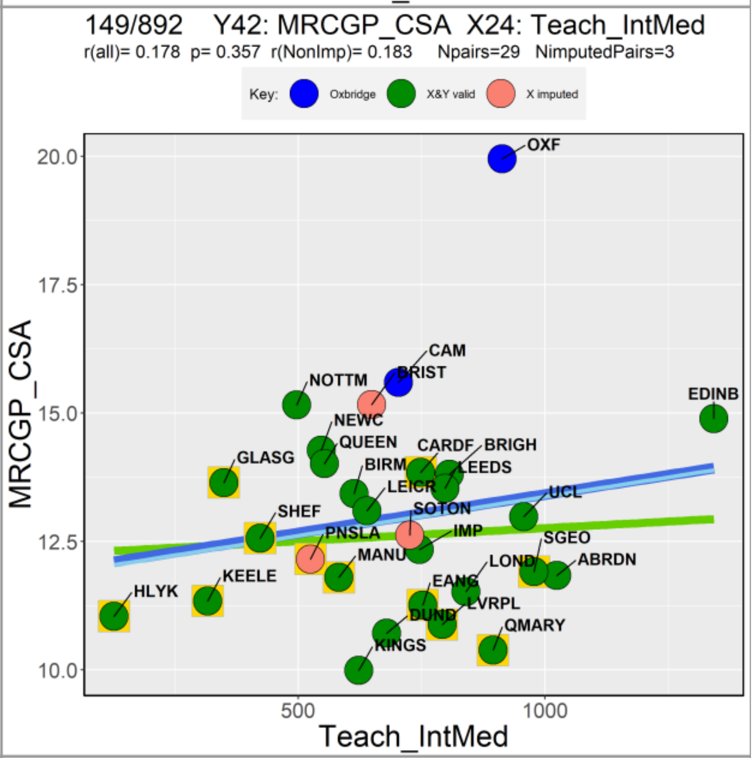
149/892 Y42: MRCGP_CSA X24: Teach_IntMed
r(all)= 0.178 p= 0.357 r(NonImp)= 0.183 Npairs=29 NimputedPairs=3
Key: Oxbridge X&Y valid X imputed
MRCGP_CSA
Teach_IntMed

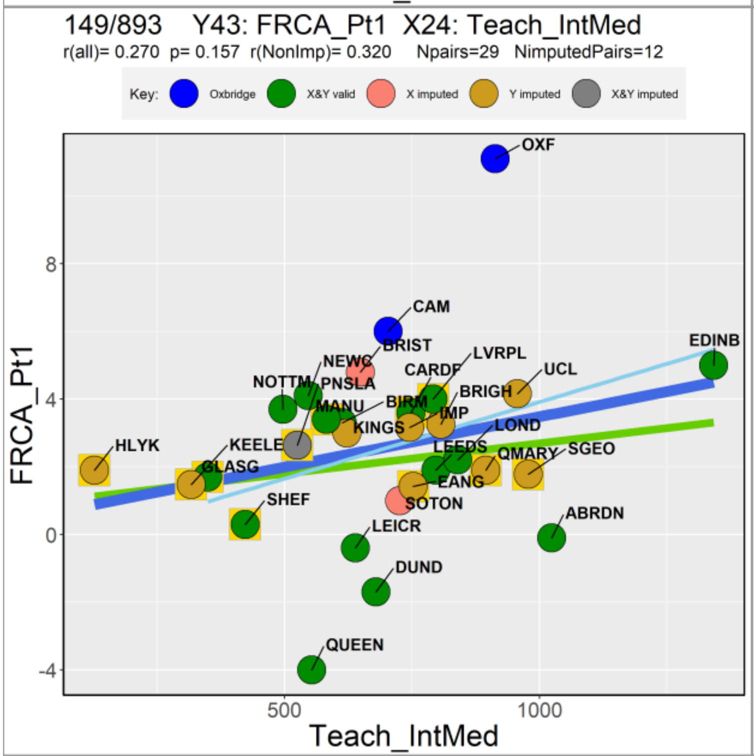
149/893 Y43: FRCA_Pt1 X24: Teach_IntMed
r(all)= 0.270 p= 0.157 r(NonImp)= 0.320 Npairs=29 NimputedPairs=12
Key: Oxbridge X&Y valid X imputed Y imputed X&Y imputed
FRCA_Pt1
Teach_IntMed

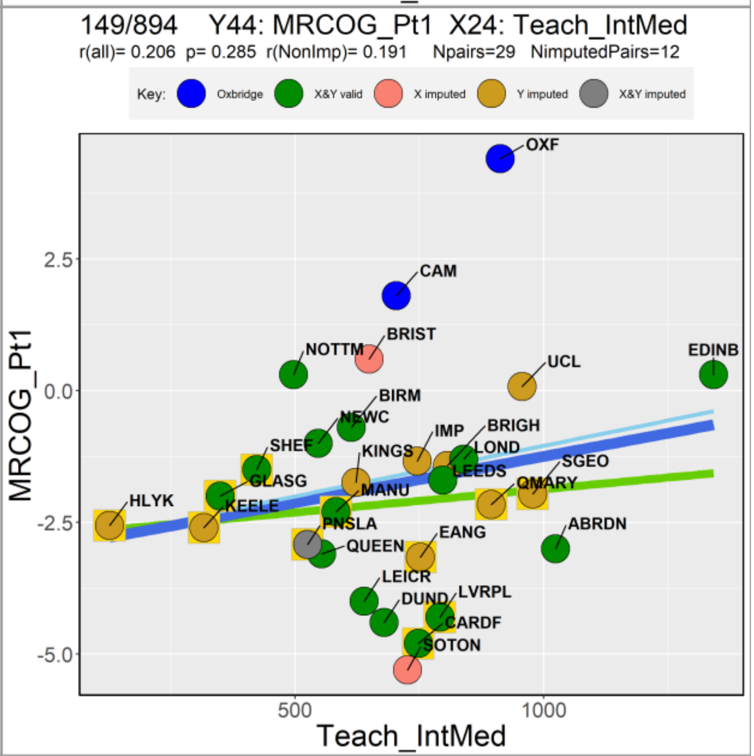
149/894 Y44: MRCOG_Pt1 X24: Teach_IntMed
r(all)= 0.206 p= 0.285 r(NonImp)= 0.191 Npairs=29 NimputedPairs=12
Key: Oxbridge X&Y valid X imputed Y imputed X&Y imputed
MRCOG_Pt1
Teach_IntMed

150/895 Y45: MRCOG_Pt2 X24: Teach_IntMed
r(all)= 0.287 p= 0.132 r(NonImp)= 0.409 Npairs=29 NimputedPairs=12

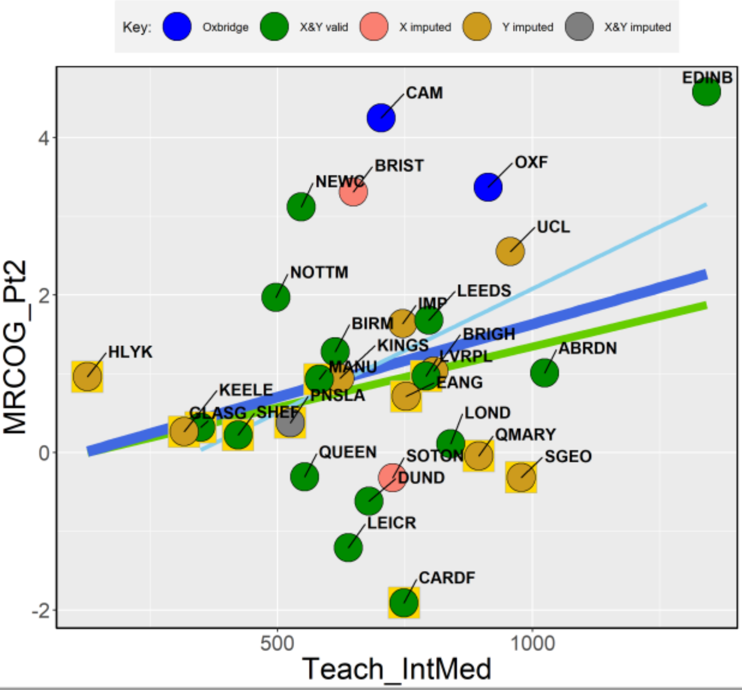

150/896 Y46: MRCP_Pt1 X24: Teach_IntMed
r(all)= 0.298 p= 0.116 r(NonImp)= 0.295 Npairs=29 NimputedPairs=6

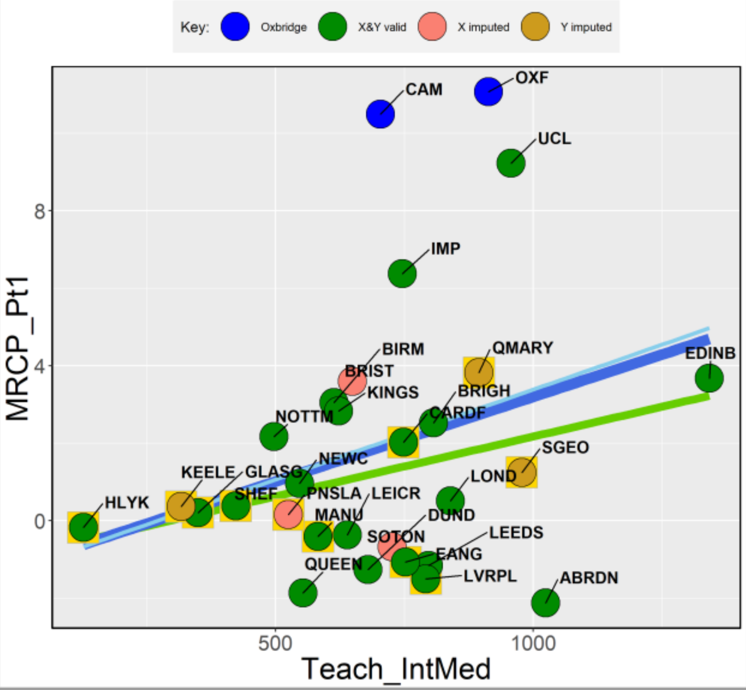

150/897 Y47: MRCP_Pt2 X24: Teach_IntMed
r(all)= 0.222 p= 0.248 r(NonImp)= 0.264 Npairs=29 NimputedPairs=6

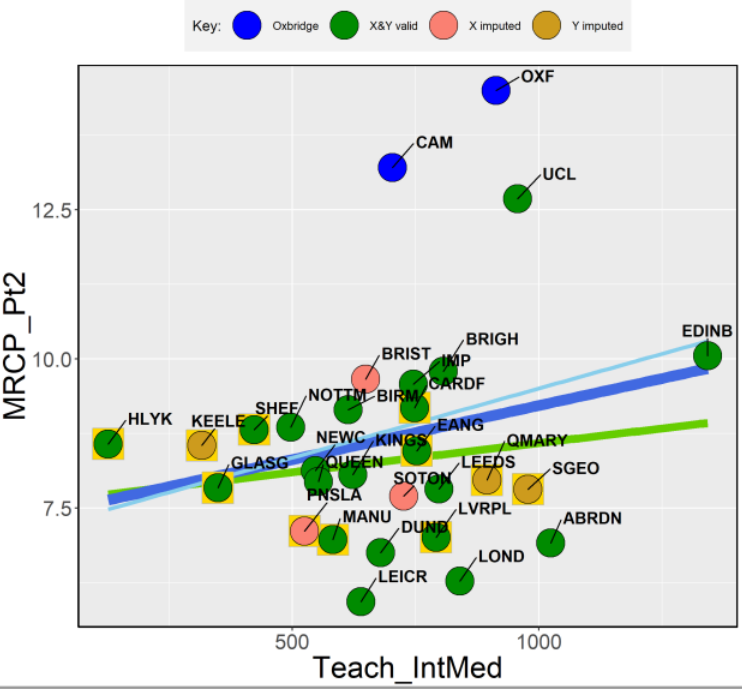

150/898 Y48: MRCP_PACES X24: Teach_IntMed
r(all)= 0.041 p= 0.831 r(NonImp)= 0.018 Npairs=29 NimputedPairs=6

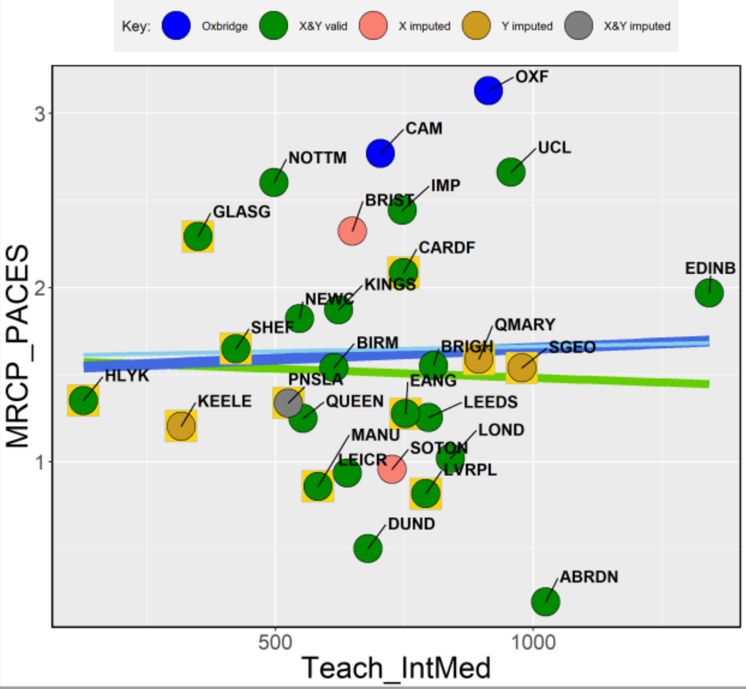

150/899 Y49: GMC_Sanctions X24: Teach_IntMed
r(all)= -0.148 p= 0.445 r(NonImp)= -0.054 Npairs=29 NimputedPairs=12

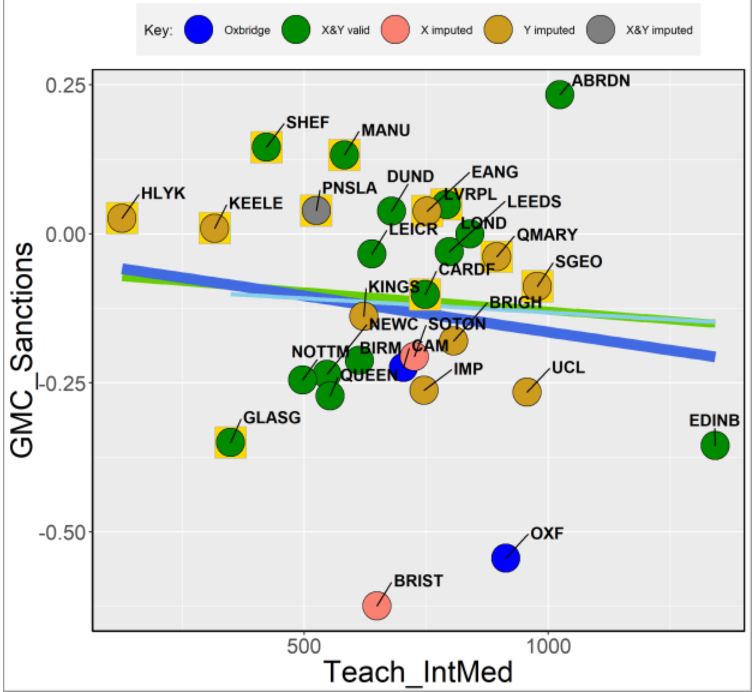

150/900 Y50: ARCP_NotExam X24: Teach_IntMed
r(all)= -0.320 p= 0.0903 r(NonImp)= -0.291 Npairs=29 NimputedPairs=4

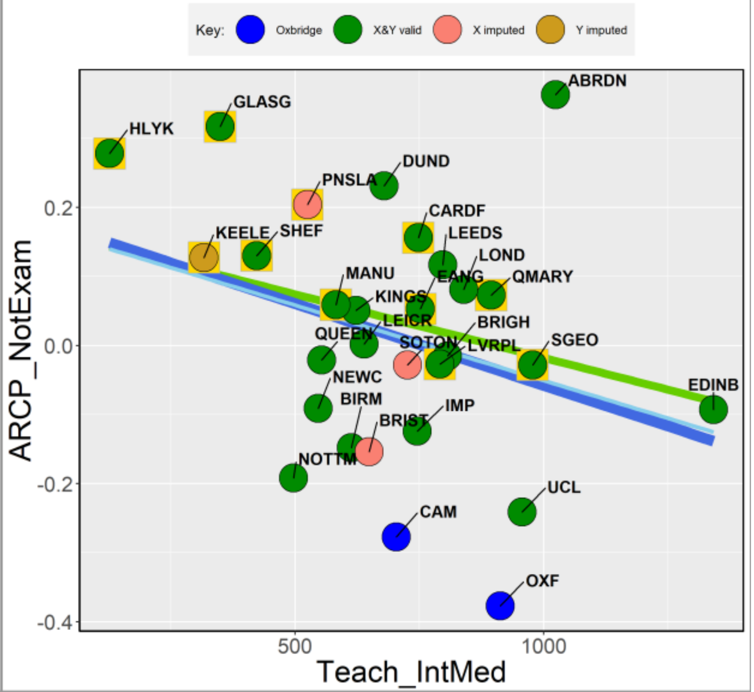

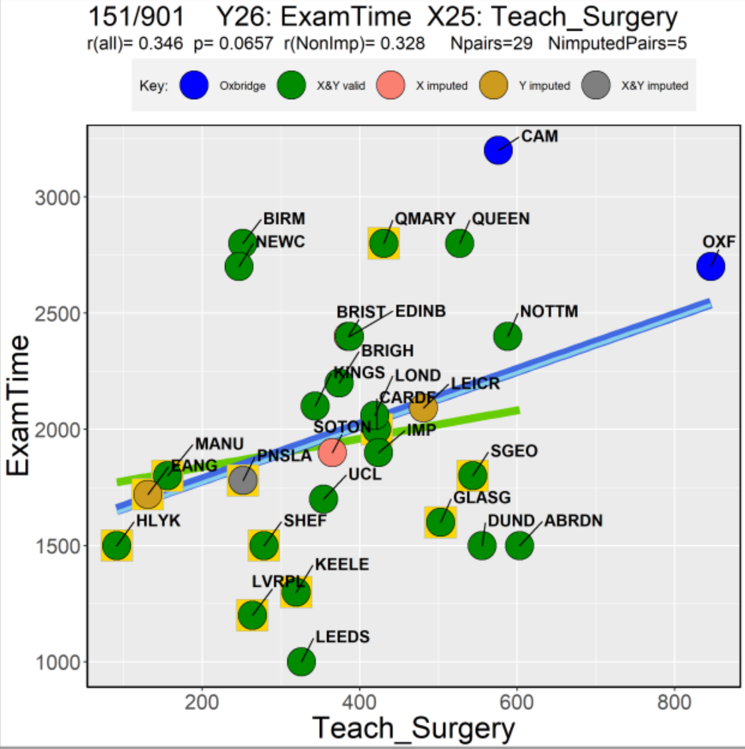
151/901 Y26: ExamTime X25: Teach_Surgery
r(all)= 0.346 p= 0.0657 r(NonImp)= 0.328 Npairs=29 NimputedPairs=5
Key: Oxbridge X&Y valid X imputed Y imputed X&Y imputed
ExamTime
Teach_Surgery
CAM
OXF
BIRM
NEWC
QMARY
QUEEN
BRIST
EDINB
NOTTM
BRIGH
KINGS
LOND
LEICR
CARDF
SOTON
IMP
MANU
EANG
PNSLA
SGEO
UCL
GLASG
HLYK
SHEF
DUND
ABRDN
KEELE
LVRPL
LEEDS

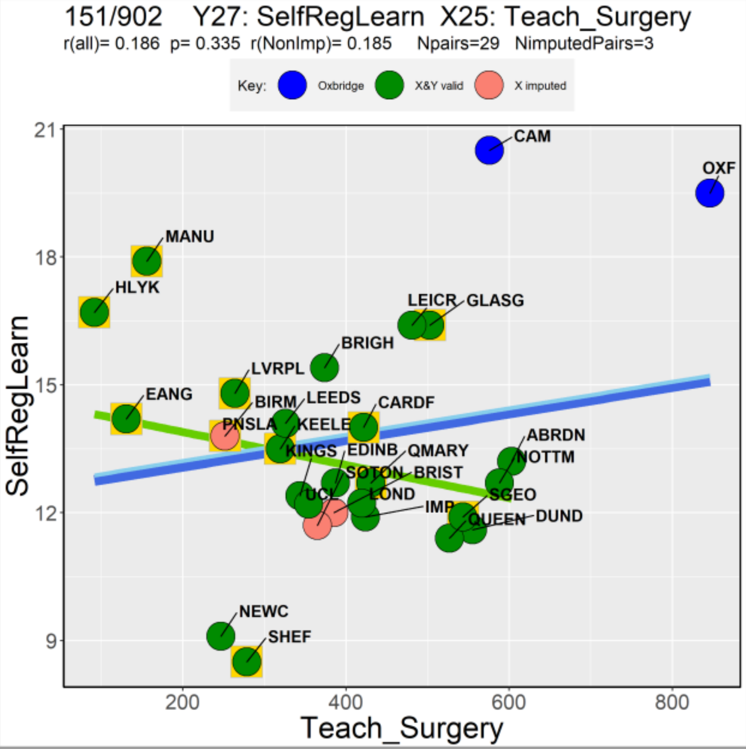
151/902 Y27: SelfRegLearn X25: Teach_Surgery
r(all)= 0.186 p= 0.335 r(NonImp)= 0.185 Npairs=29 NimputedPairs=3
Key: Oxbridge X&Y valid X imputed
SelfRegLearn
Teach_Surgery
CAM
OXF
MANU
HLYK
LEICR
GLASG
BRIGH
LVRPL
EANG
BIRM
LEEDS
CARDF
PNSLA
KEELE
ABRDN
KINGS
EDINB
QMARY
NOTTM
SOTON
BRIST
UCL
LOND
SGEO
IMP
QUEEN
DUND
NEWC
SHEF

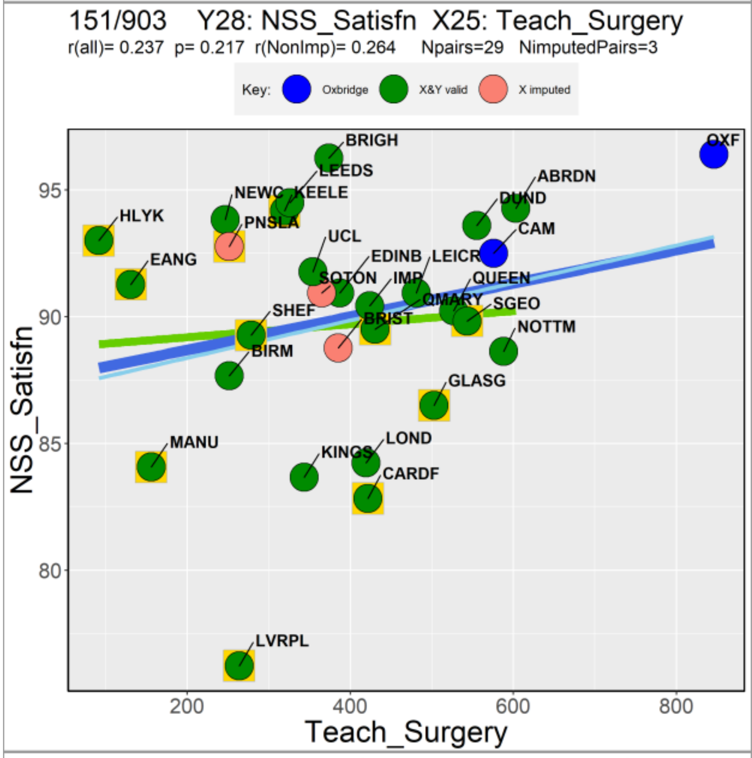
151/903 Y28: NSS_Satisfn X25: Teach_Surgery
r(all)= 0.237 p= 0.217 r(NonImp)= 0.264 Npairs=29 NimputedPairs=3
Key: Oxbridge X&Y valid X imputed
NSS_Satisfn
Teach_Surgery
OXF
BRIGH
LEEDS
ABRDN
NEWC
KEELE
DUND
HLYK
PNSLA
CAM
UCL
EANG
EDINB
LEICR
SOTON
IMP
QUEEN
QMARY
SGEO
SHEF
BRIST
NOTTM
BIRM
GLASG
MANU
LOND
KINGS
CARDF
LVRPL

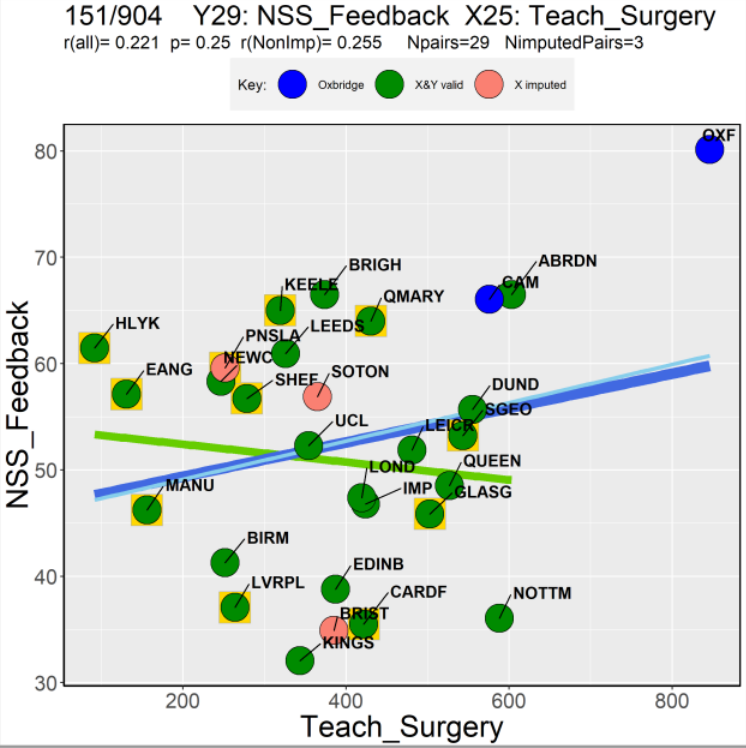
151/904 Y29: NSS_Feedback X25: Teach_Surgery
r(all)= 0.221 p= 0.25 r(NonImp)= 0.255 Npairs=29 NimputedPairs=3
Key: Oxbridge X&Y valid X imputed
NSS_Feedback
Teach_Surgery
OXF
ABRDN
BRIGH
KEELE
CAM
QMARY
HLYK
PNSLA
LEEDS
NEWC
EANG
SOTON
SHEF
DUND
SGEO
UCL
LEICR
QUEEN
LOND
MANU
IMP
GLASG
BIRM
EDINB
CARDF
LVRPL
NOTTM
BRIST
KINGS

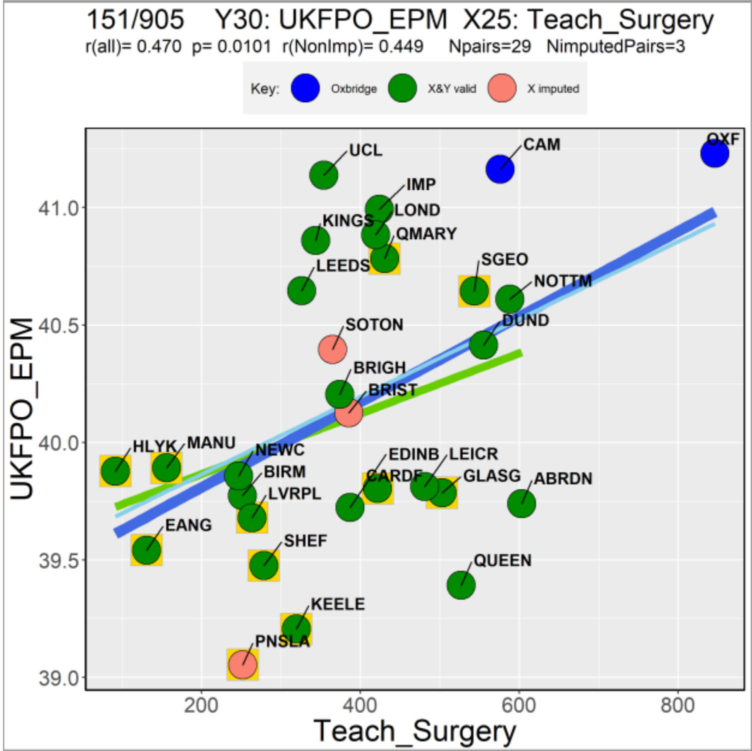
151/905 Y30: UKFPO_EPM X25: Teach_Surgery
r(all)= 0.470 p= 0.0101 r(NonImp)= 0.449 Npairs=29 NimputedPairs=3
Key: Oxbridge X&Y valid X imputed
UKFPO_EPM
Teach_Surgery
OXF
UCL
CAM
IMP
LOND
KINGS
QMARY
SGEO
LEEDS
NOTTM
SOTON
DUND
BRIGH
BRIST
HLYK
MANU
NEWC
EDINB
LEICR
BIRM
CARDF
GLASG
ABRDN
LVRPL
EANG
SHEF
QUEEN
KEELE
PNSLA

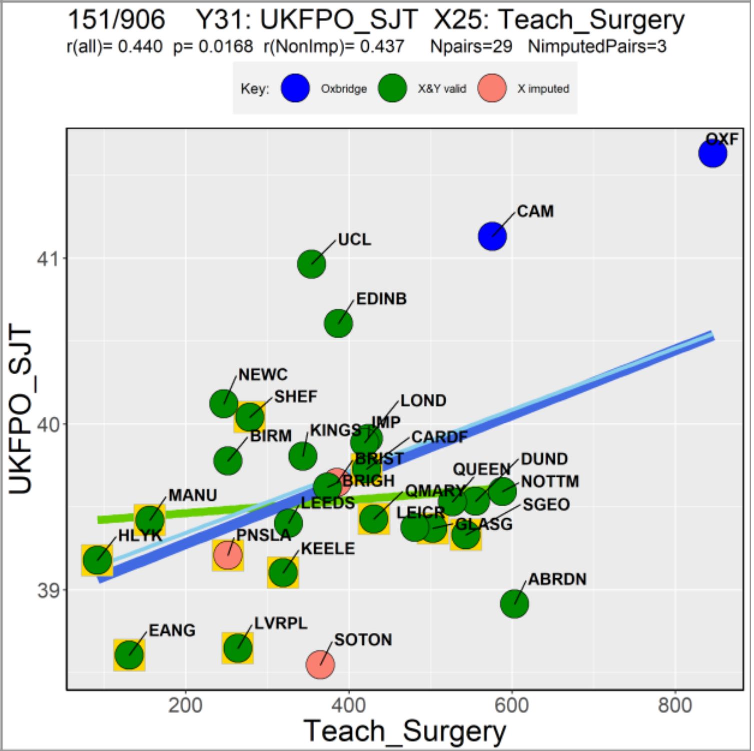
151/906 Y31: UKFPO_SJT X25: Teach_Surgery
r(all)= 0.440 p= 0.0168 r(NonImp)= 0.437 Npairs=29 NimputedPairs=3
Key: Oxbridge X&Y valid X imputed
UKFPO_SJT
Teach_Surgery
OXF
CAM
UCL
EDINB
NEWC
SHEF
LOND
KINGS
IMP
CARDF
BIRM
BRIST
DUND
QUEEN
NOTTM
BRIGH
MANU
QMARY
SGEO
LEEDS
LEICR
GLASG
HLYK
PNSLA
KEELE
ABRDN
EANG
LVRPL
SOTON

152/907 Y32: F1_Preparedness X25: Teach_Surgery
r(all)= -0.055 p= 0.778 r(NonImp)= -0.000 Npairs=29 NimputedPairs=3

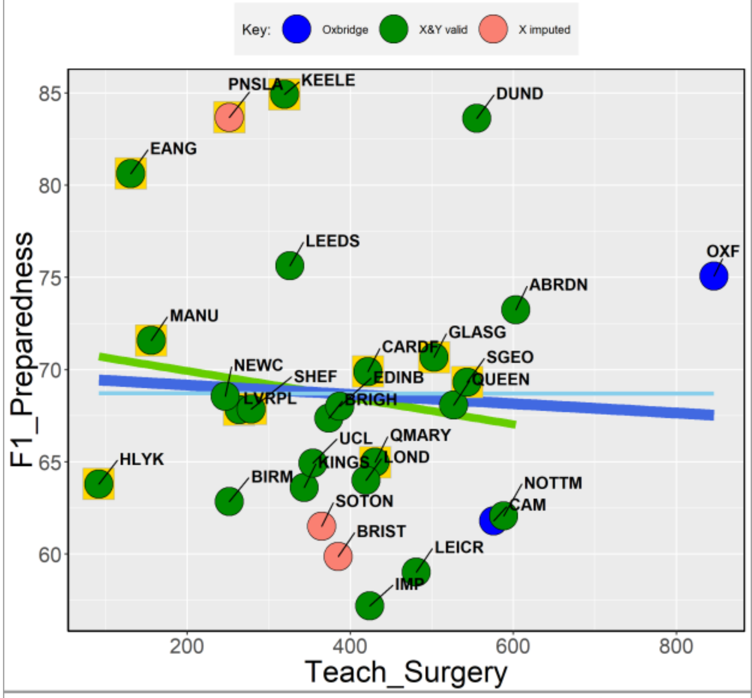

152/908 Y33: F1_Satisfn X25: Teach_Surgery
r(all)= -0.363 p= 0.0533 r(NonImp)= -0.335 Npairs=29 NimputedPairs=3

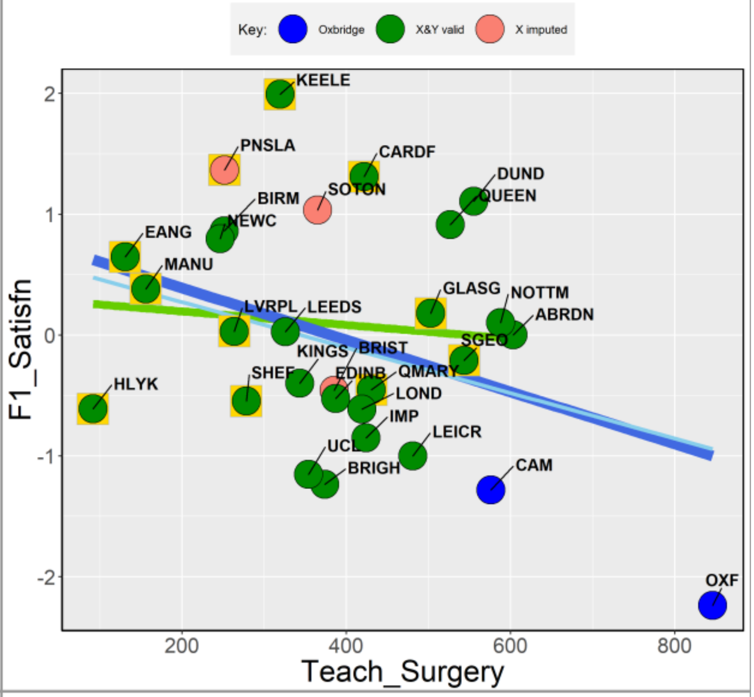

152/909 Y34: F1_Workload X25: Teach_Surgery
r(all)= -0.475 p= 0.00918 r(NonImp)= -0.479 Npairs=29 NimputedPairs=3

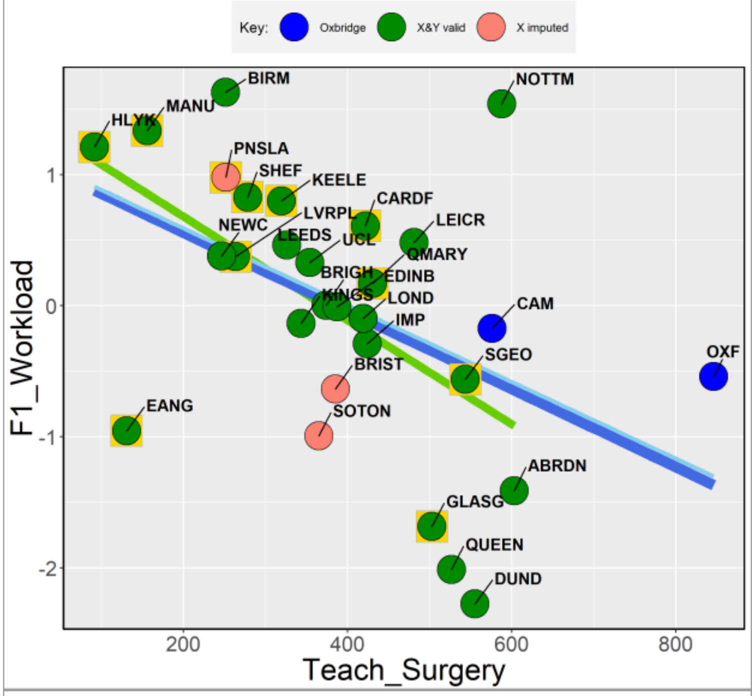

152/910 Y35: F1_Supervn X25: Teach_Surgery
r(all)= 0.312 p= 0.0999 r(NonImp)= 0.325 Npairs=29 NimputedPairs=3

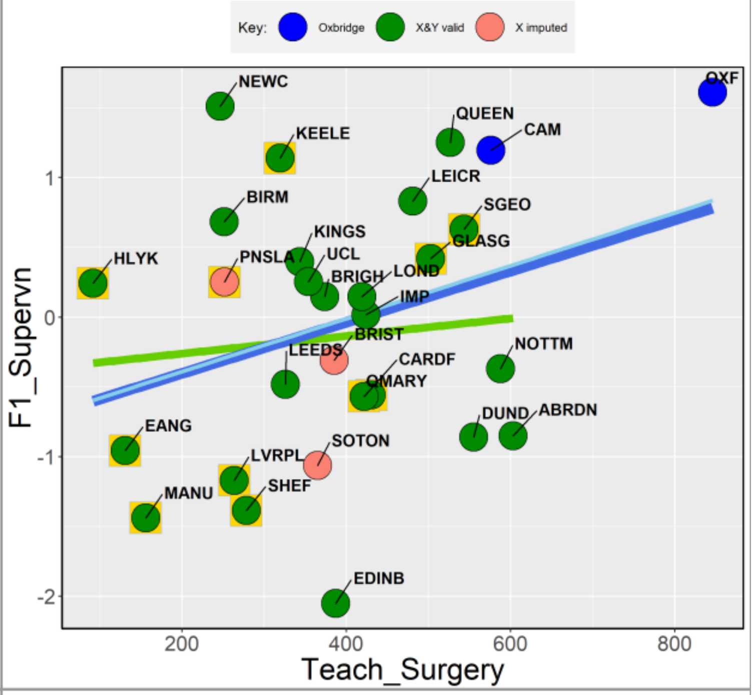

152/911 Y36: Trainee_GP X25: Teach_Surgery
r(all)= -0.361 p= 0.0546 r(NonImp)= -0.374 Npairs=29 NimputedPairs=3

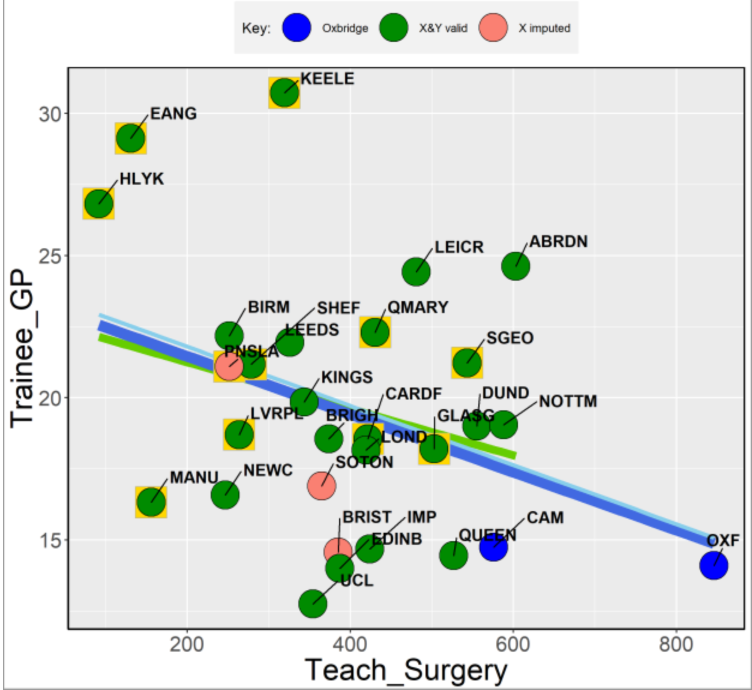

152/912 Y37: Trainee_Psyc X25: Teach_Surgery
r(all)= 0.412 p= 0.0263 r(NonImp)= 0.394 Npairs=29 NimputedPairs=3

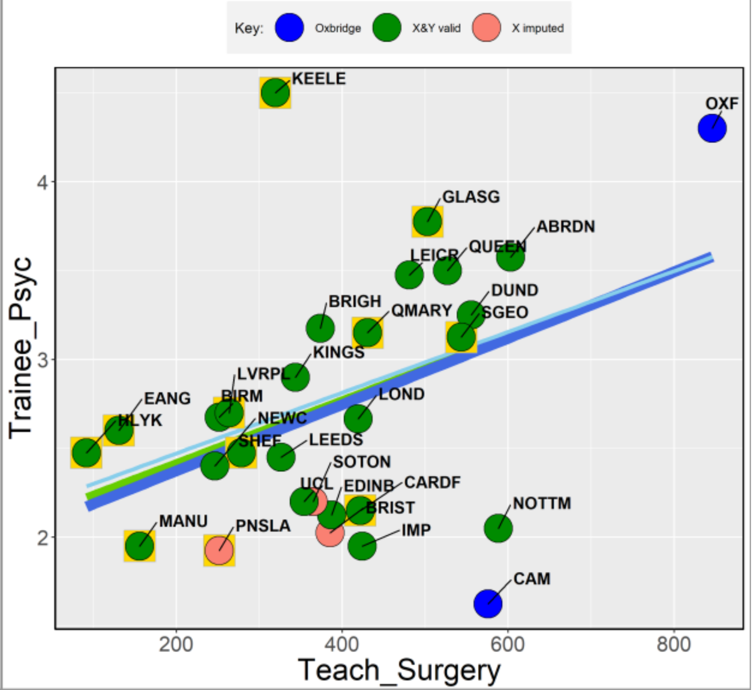

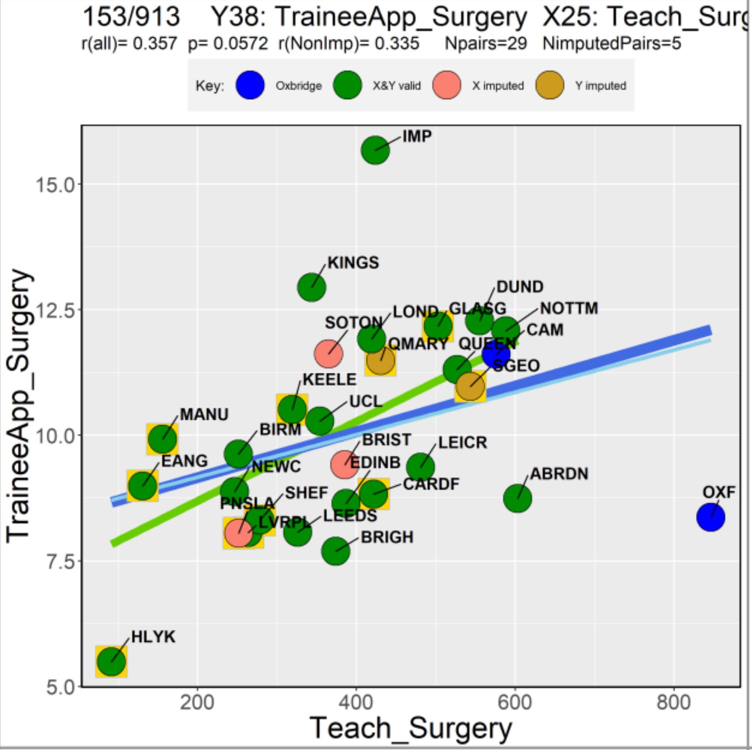
153/913 Y38: TraineeApp_Surgery X25: Teach_Surg
r(all)= 0.357 p= 0.0572 r(NonImp)= 0.335 Npairs=29 NimputedPairs=5
Key: Oxbridge X&Y valid X imputed Y imputed
TraineeApp_Surgery
Teach_Surgery

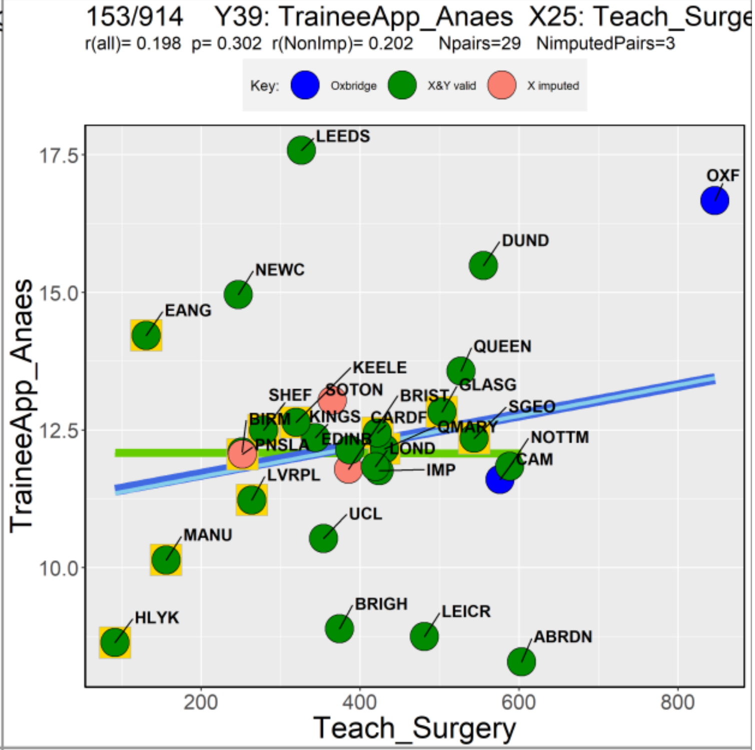
153/914 Y39: TraineeApp_Anaes X25: Teach_Surge
r(all)= 0.198 p= 0.302 r(NonImp)= 0.202 Npairs=29 NimputedPairs=3
Key: Oxbridge X&Y valid X imputed
TraineeApp_Anaes
Teach_Surgery

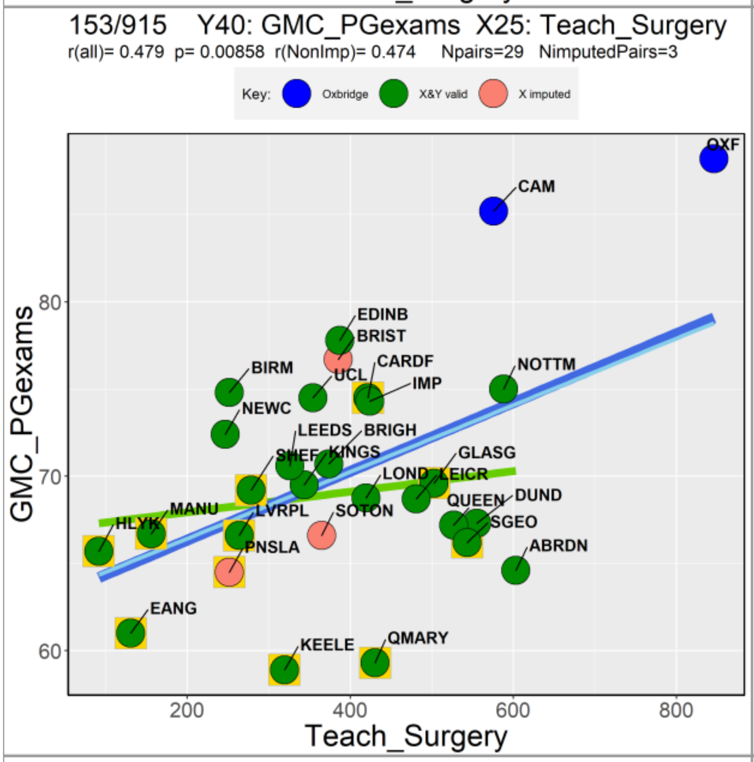
153/915 Y40: GMC_PGexams X25: Teach_Surgery
r(all)= 0.479 p= 0.00858 r(NonImp)= 0.474 Npairs=29 NimputedPairs=3
Key: Oxbridge X&Y valid X imputed
GMC_PGexams
Teach_Surgery

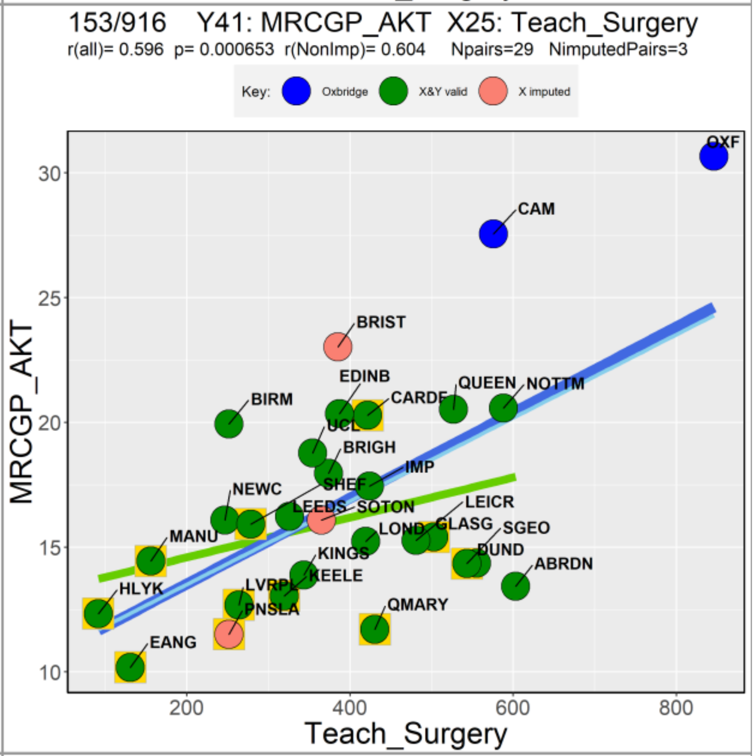
153/916 Y41: MRCGP_AKT X25: Teach_Surgery
r(all)= 0.596 p= 0.000653 r(NonImp)= 0.604 Npairs=29 NimputedPairs=3
Key: Oxbridge X&Y valid X imputed
MRCGP_AKT
Teach_Surgery

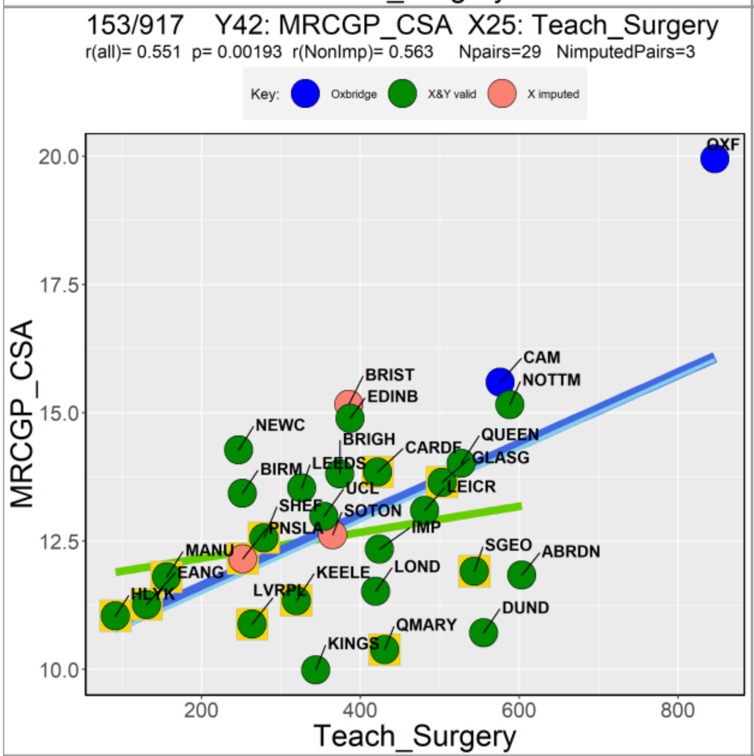
153/917 Y42: MRCGP_CSA X25: Teach_Surgery
r(all)= 0.551 p= 0.00193 r(NonImp)= 0.563 Npairs=29 NimputedPairs=3
Key: Oxbridge X&Y valid X imputed
MRCGP_CSA
Teach_Surgery

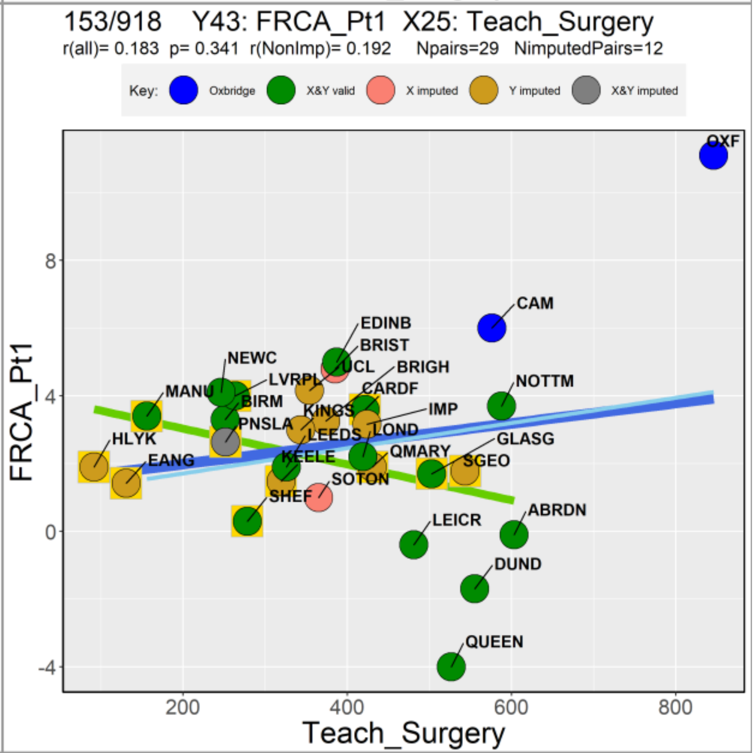
153/918 Y43: FRCA_Pt1 X25: Teach_Surgery
r(all)= 0.183 p= 0.341 r(NonImp)= 0.192 Npairs=29 NimputedPairs=12
Key: Oxbridge X&Y valid X imputed Y imputed X&Y imputed
FRCA_Pt1
Teach_Surgery

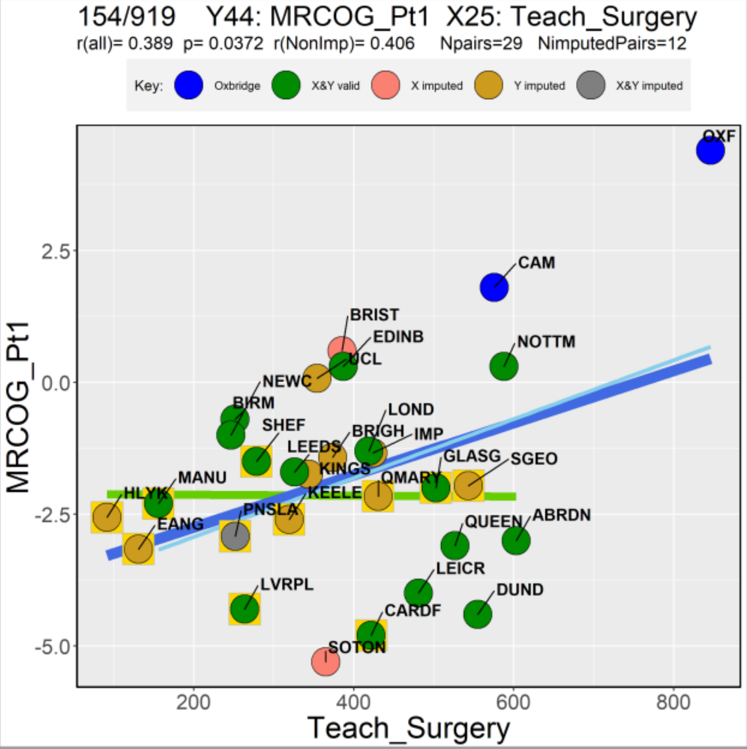
154/919 Y44: MRCOG_Pt1 X25: Teach_Surgery
r(all)= 0.389 p= 0.0372 r(NonImp)= 0.406 Npairs=29 NimputedPairs=12
Key: Oxbridge X&Y valid X imputed Y imputed X&Y imputed
MRCOG_Pt1
Teach_Surgery
2.5
0.0
-2.5
-5.0
200
400
600
800
OXF
CAM
BRIST
EDINB
NOTTM
UCL
NEWC
BIRM
SHEF
LOND
BRIGH
IMP
LEEDS
KINGS
GLASG
SGEO
MANU
QMARY
HLYK
KEELE
PNSLA
EANG
QUEEN
ABRDN
LEICR
LVRPL
DUND
CARDF
SOTON

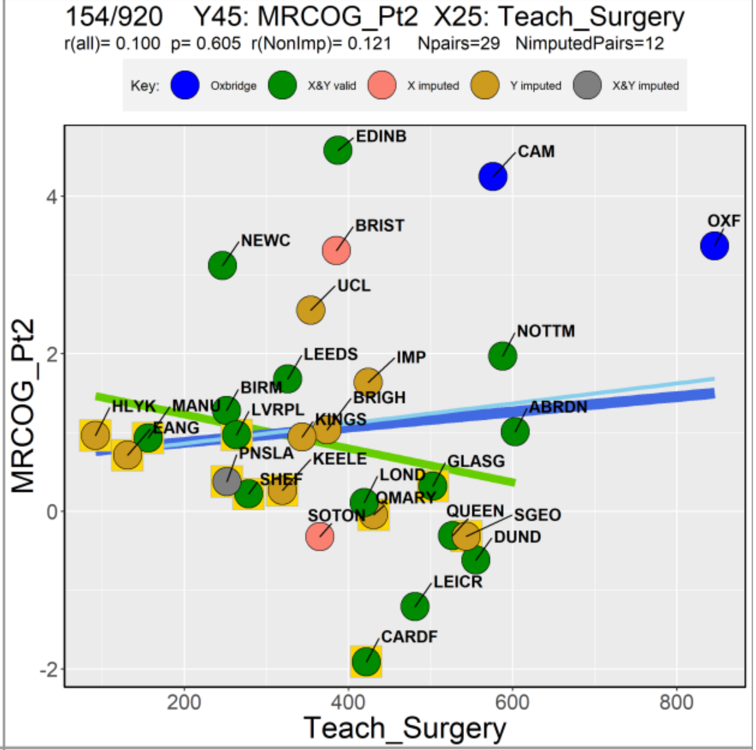
154/920 Y45: MRCOG_Pt2 X25: Teach_Surgery
r(all)= 0.100 p= 0.605 r(NonImp)= 0.121 Npairs=29 NimputedPairs=12
Key: Oxbridge X&Y valid X imputed Y imputed X&Y imputed
MRCOG_Pt2
Teach_Surgery
4
2
0
-2
200
400
600
800
EDINB
CAM
OXF
BRIST
NEWC
UCL
NOTTM
LEEDS
IMP
BIRM
BRIGH
HLYK
MANU
LVRPL
ABRDN
EANG
KINGS
PNSLA
KEELE
GLASG
LOND
SHEF
QMARY
SOTON
QUEEN
SGEO
DUND
LEICR
CARDF

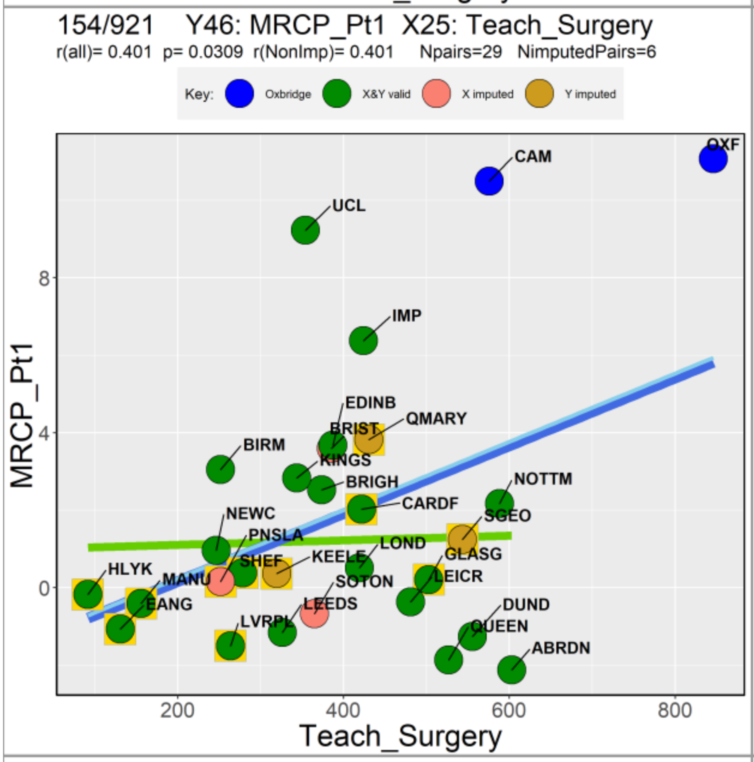
154/921 Y46: MRCP_Pt1 X25: Teach_Surgery
r(all)= 0.401 p= 0.0309 r(NonImp)= 0.401 Npairs=29 NimputedPairs=6
Key: Oxbridge X&Y valid X imputed Y imputed
MRCP_Pt1
Teach_Surgery
8
4
0
200
400
600
800
OXF
CAM
UCL
IMP
EDINB
BRIST
QMARY
BIRM
KINGS
BRIGH
NOTTM
CARDF
NEWC
SGEO
PNSLA
LOND
SHEF
KEELE
GLASG
HLYK
SOTON
MANU
LEICR
EANG
LEEDS
DUND
LVRPL
QUEEN
ABRDN

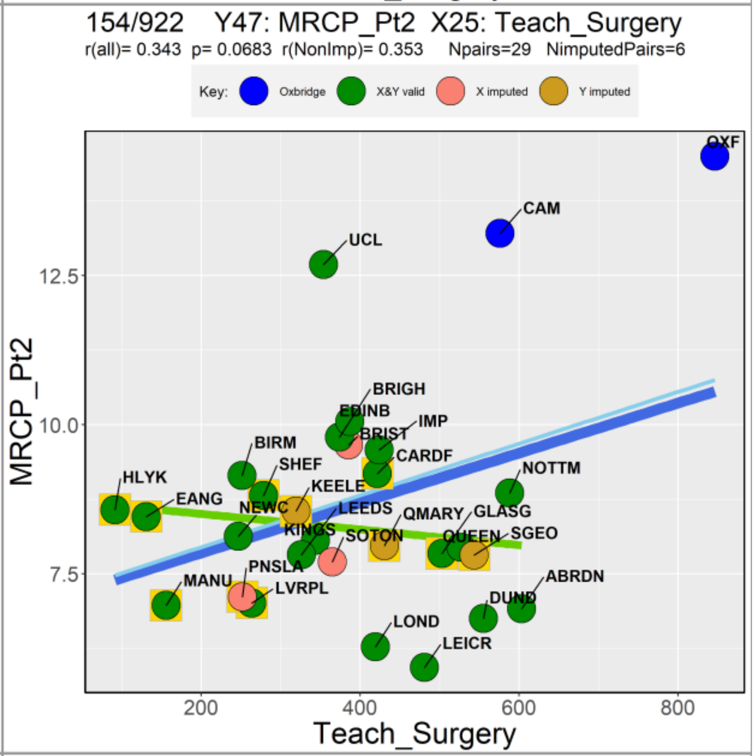
154/922 Y47: MRCP_Pt2 X25: Teach_Surgery
r(all)= 0.343 p= 0.0683 r(NonImp)= 0.353 Npairs=29 NimputedPairs=6
Key: Oxbridge X&Y valid X imputed Y imputed
MRCP_Pt2
Teach_Surgery
12.5
10.0
7.5
200
400
600
800
OXF
CAM
UCL
BRIGH
EDINB
BRIST
IMP
BIRM
CARDF
SHEF
HLYK
NOTTM
KEELE
EANG
NEWC
LEEDS
QMARY
GLASG
KINGS
SOTON
QUEEN
SGEO
PNSLA
MANU
LVRPL
ABRDN
DUND
LOND
LEICR

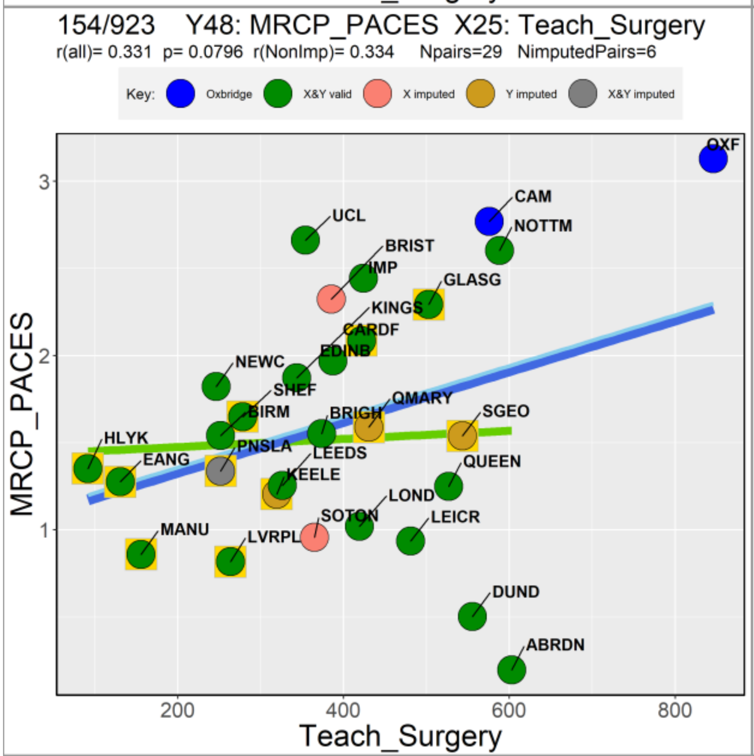
154/923 Y48: MRCP_PACES X25: Teach_Surgery
r(all)= 0.331 p= 0.0796 r(NonImp)= 0.334 Npairs=29 NimputedPairs=6
Key: Oxbridge X&Y valid X imputed Y imputed X&Y imputed
MRCP_PACES
Teach_Surgery
3
2
1
200
400
600
800
OXF
CAM
UCL
NOTTM
BRIST
IMP
GLASG
KINGS
CARDF
EDINB
NEWC
SHEF
QMARY
BIRM
BRIGH
SGEO
HLYK
PNSLA
EANG
LEEDS
QUEEN
KEELE
LOND
SOTON
LEICR
MANU
LVRPL
DUND
ABRDN

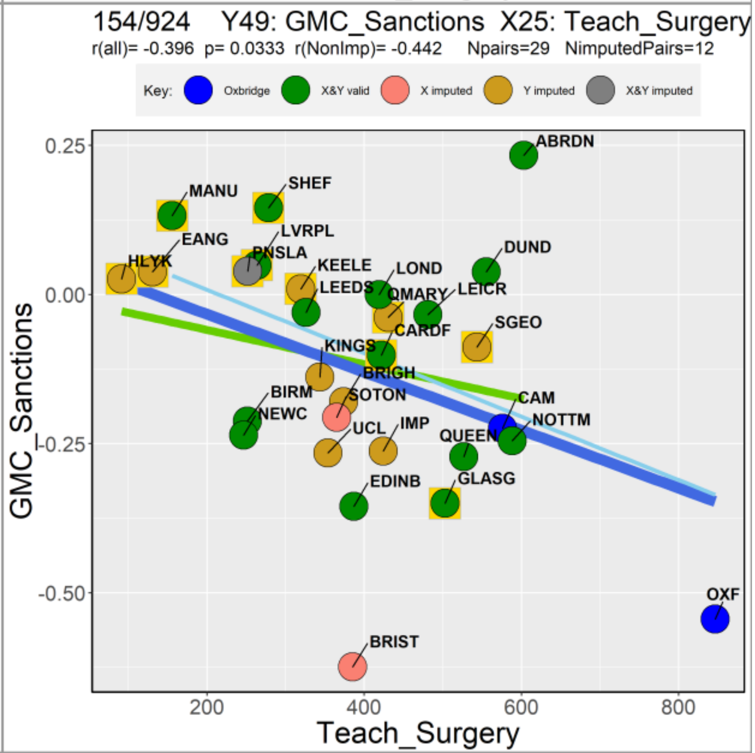
154/924 Y49: GMC_Sanctions X25: Teach_Surgery
r(all)= -0.396 p= 0.0333 r(NonImp)= -0.442 Npairs=29 NimputedPairs=12
Key: Oxbridge X&Y valid X imputed Y imputed X&Y imputed
GMC_Sanctions
Teach_Surgery
0.25
0.00
-0.25
-0.50
200
400
600
800
ABRDN
SHEF
MANU
LVRPL
EANG
DUND
PNSLA
HLYK
KEELE
LOND
LEEDS
QMARY
LEICR
CARDF
SGEO
KINGS
BRIGH
CAM
BIRM
SOTON
NEWC
NOTTM
UCL
IMP
QUEEN
EDINB
GLASG
OXF
BRIST

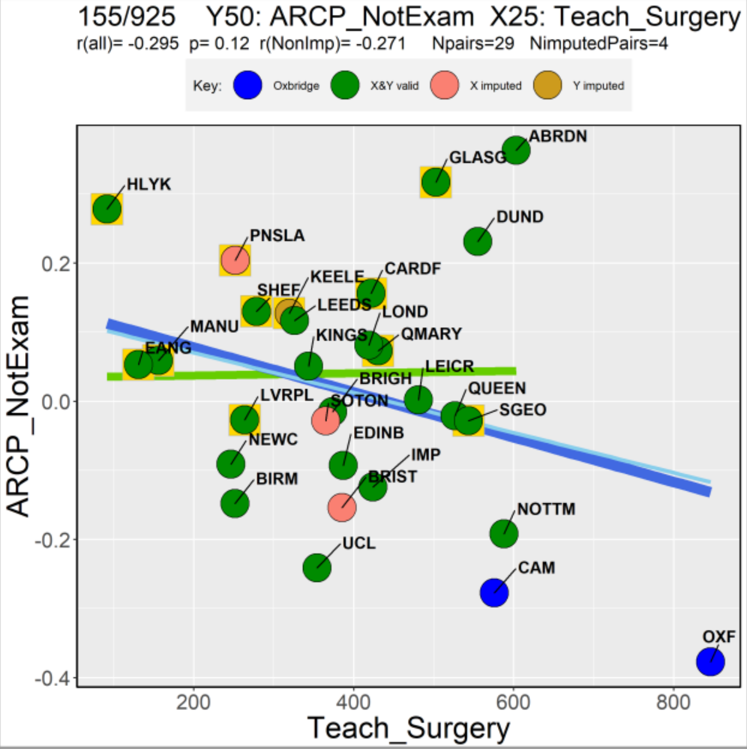
155/925 Y50: ARCP_NotExam X25: Teach_Surgery
r(all)= -0.295 p= 0.12 r(NonImp)= -0.271 Npairs=29 NimputedPairs=4
Key: Oxbridge X&Y valid X imputed Y imputed
ARCP_NotExam
Teach_Surgery
0.2
0.0
-0.2
-0.4
200
400
600
800
HLYK
PNSLA
GLASG
ABRDN
DUND
CARDF
KEELE
SHEF
LEEDS
LOND
MANU
KINGS
QMARY
EANG
LEICR
BRIGH
LVRPL
SOTON
QUEEN
SGEO
NEWC
EDINB
IMP
BIRM
BRIST
NOTTM
UCL
CAM
OXF

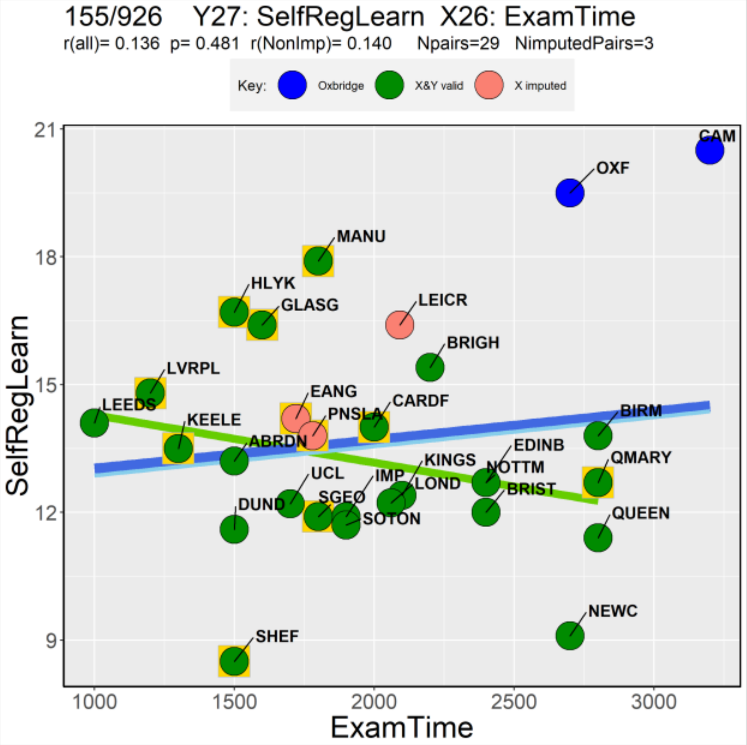
155/926 Y27: SelfRegLearn X26: ExamTime
r(all)= 0.136 p= 0.481 r(NonImp)= 0.140 Npairs=29 NimputedPairs=3
Key: Oxbridge X&Y valid X imputed
SelfRegLearn
ExamTime
21
18
15
12
9
1000
1500
2000
2500
3000
CAM
OXF
MANU
HLYK
GLASG
LEICR
BRIGH
LVRPL
EANG
LEEDS
PNSLA
CARDF
BIRM
KEELE
ABRDN
EDINB
KINGS
NOTTM
QMARY
UCL
IMP
LOND
BRIST
DUND
SGEO
SOTON
QUEEN
NEWC
SHEF

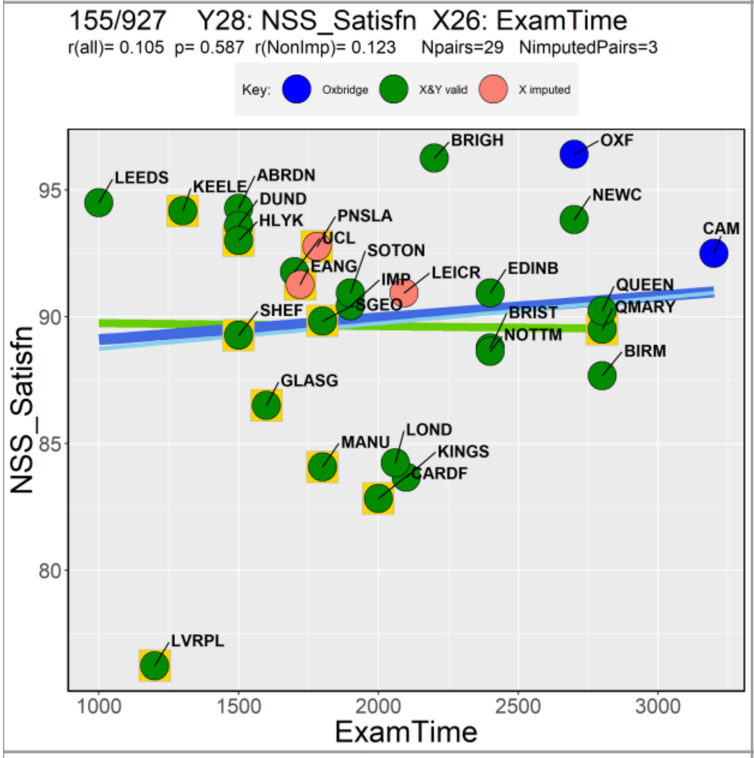
155/927 Y28: NSS_Satisfn X26: ExamTime
r(all)= 0.105 p= 0.587 r(NonImp)= 0.123 Npairs=29 NimputedPairs=3
Key: Oxbridge X&Y valid X imputed
NSS_Satisfn
ExamTime
95
90
85
80
1000
1500
2000
2500
3000
BRIGH
OXF
LEEDS
KEELE
ABRDN
DUND
NEWC
HLYK
PNSLA
CAM
UCL
SOTON
EANG
EDINB
IMP
LEICR
QUEEN
SGEO
QMARY
SHEF
BRIST
NOTTM
BIRM
GLASG
LOND
MANU
KINGS
CARDF
LVRPL

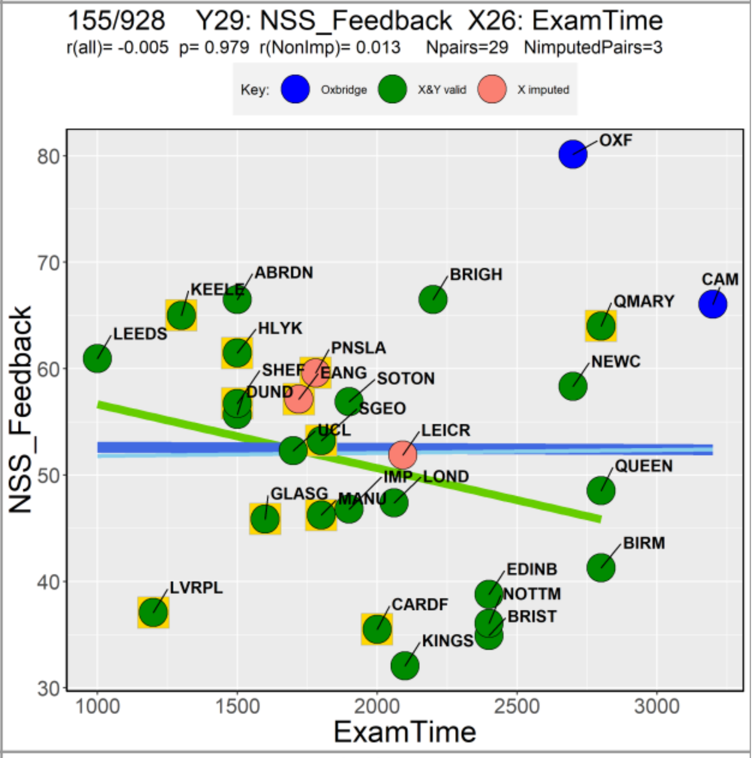
155/928 Y29: NSS_Feedback X26: ExamTime
r(all)= -0.005 p= 0.979 r(NonImp)= 0.013 Npairs=29 NimputedPairs=3
Key: Oxbridge X&Y valid X imputed
NSS_Feedback
ExamTime
80
70
60
50
40
30
1000
1500
2000
2500
3000
OXF
ABRDN
BRIGH
KEELE
CAM
QMARY
HLYK
LEEDS
PNSLA
SHEF
EANG
SOTON
QMARY
NEWC
DUND
SGEO
UCL
LEICR
GLASG
IMP
LOND
MANU
QUEEN
BIRM
EDINB
NOTTM
LVRPL
CARDF
BRIST
KINGS

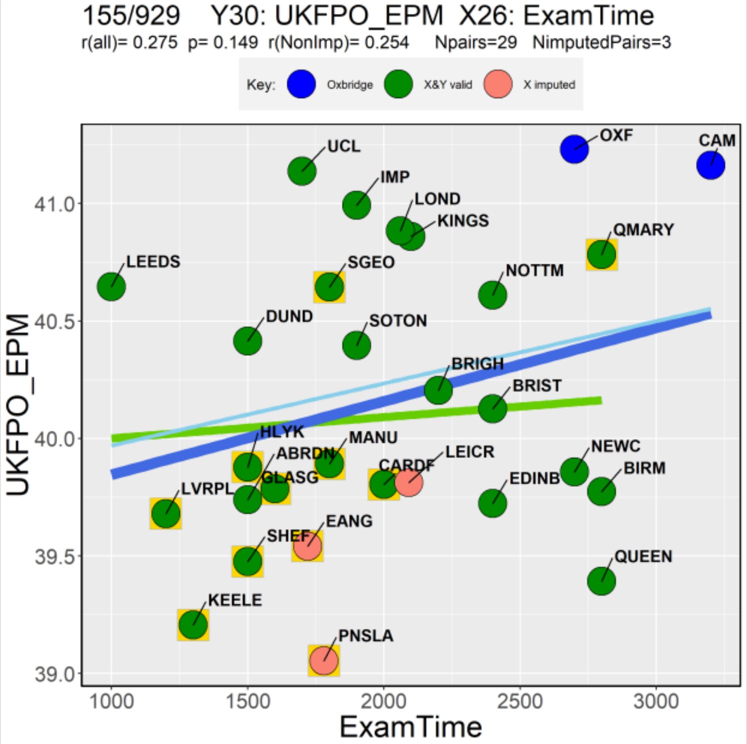
155/929 Y30: UKFPO_EPM X26: ExamTime
r(all)= 0.275 p= 0.149 r(NonImp)= 0.254 Npairs=29 NimputedPairs=3
Key: Oxbridge X&Y valid X imputed
UKFPO_EPM
ExamTime
41.0
40.5
40.0
39.5
39.0
1000
1500
2000
2500
3000
UCL
OXF
CAM
IMP
LOND
KINGS
QMARY
LEEDS
SGEO
NOTTM
DUND
SOTON
BRIGH
BRIST
HLYK
MANU
ABRDN
CARDF
LEICR
NEWC
GLASG
EDINB
BIRM
LVRPL
EANG
SHEF
QUEEN
KEELE
PNSLA

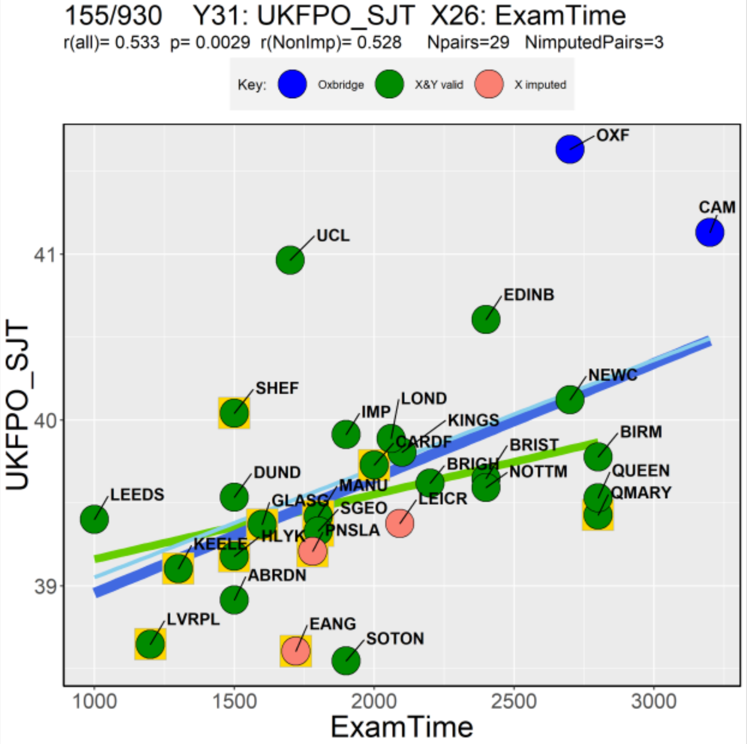
155/930 Y31: UKFPO_SJT X26: ExamTime
r(all)= 0.533 p= 0.0029 r(NonImp)= 0.528 Npairs=29 NimputedPairs=3
Key: Oxbridge X&Y valid X imputed
UKFPO_SJT
ExamTime
41
40
39
1000
1500
2000
2500
3000
OXF
CAM
UCL
EDINB
NEWC
SHEF
LOND
IMP
KINGS
CARDF
BIRM
BRIST
BRIGH
NOTTM
DUND
MANU
QUEEN
GLASG
LEICR
QMARY
LEEDS
SGEO
PNSLA
KEELE
HLYK
ABRDN
LVRPL
EANG
SOTON

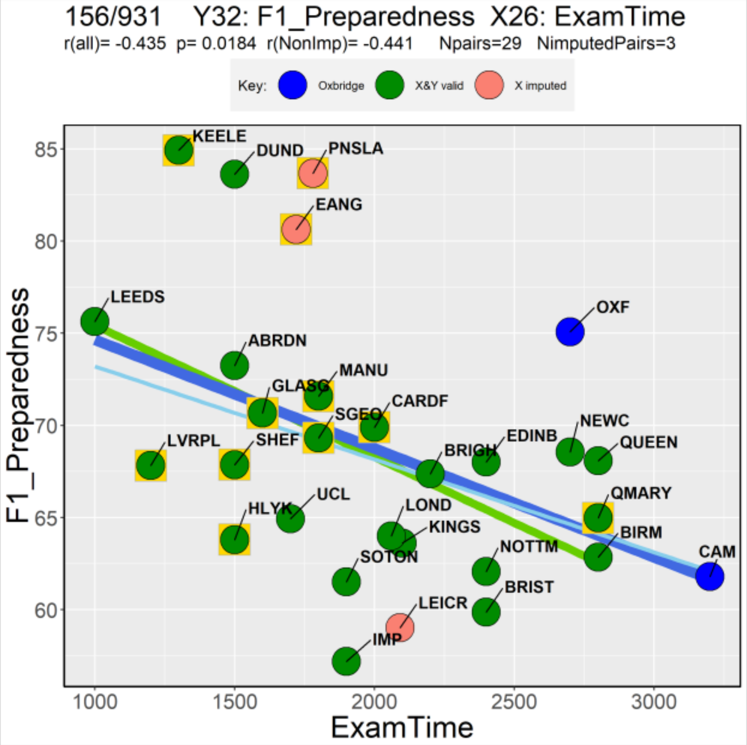
156/931 Y32: F1_Preparedness X26: ExamTime
r(all)= -0.435 p= 0.0184 r(NonImp)= -0.441 Npairs=29 NimputedPairs=3
Key: Oxbridge X&Y valid X imputed
F1_Preparedness
ExamTime

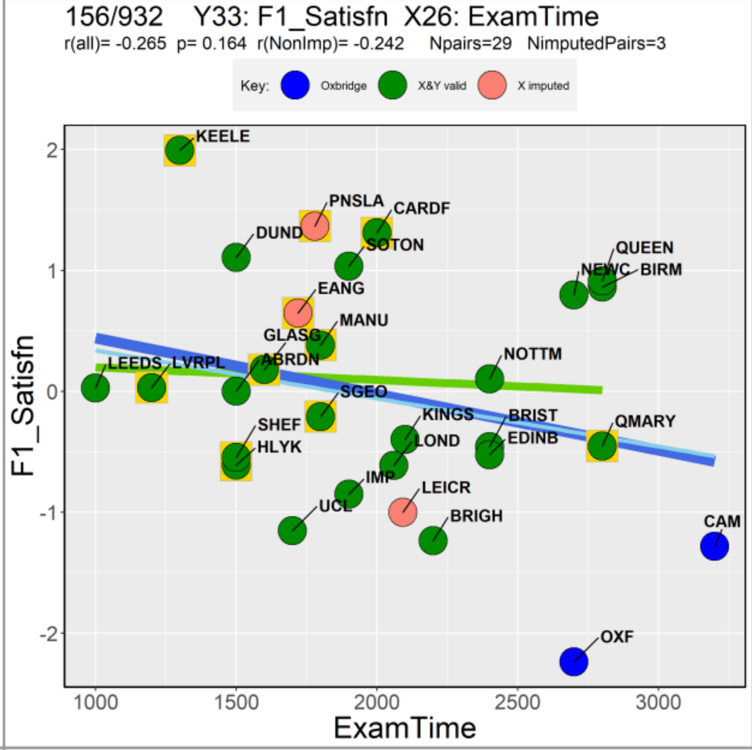
156/932 Y33: F1_Satisfn X26: ExamTime
r(all)= -0.265 p= 0.164 r(NonImp)= -0.242 Npairs=29 NimputedPairs=3
Key: Oxbridge X&Y valid X imputed
F1_Satisfn
ExamTime

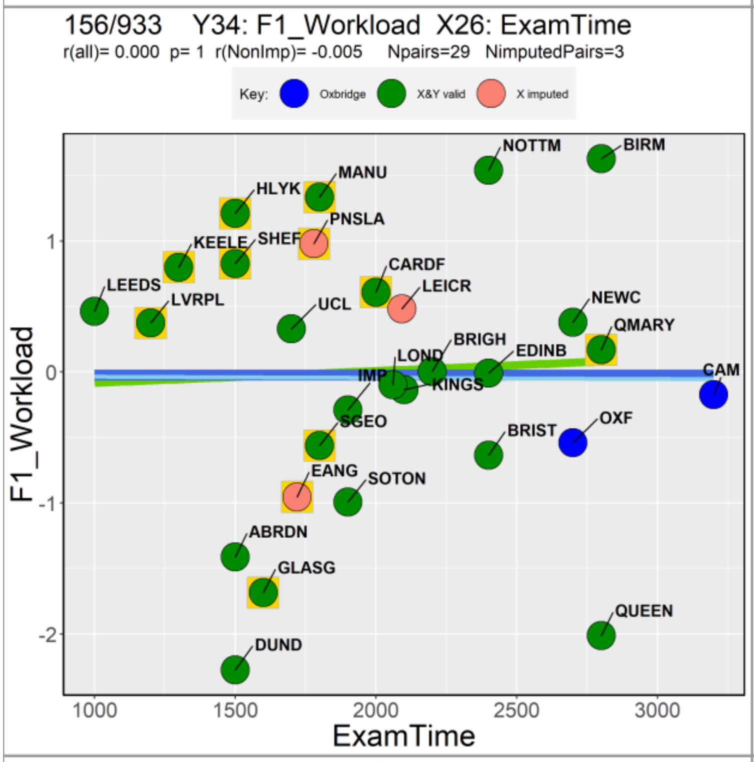
156/933 Y34: F1_Workload X26: ExamTime
r(all)= 0.000 p= 1 r(NonImp)= -0.005 Npairs=29 NimputedPairs=3
Key: Oxbridge X&Y valid X imputed
F1_Workload
ExamTime

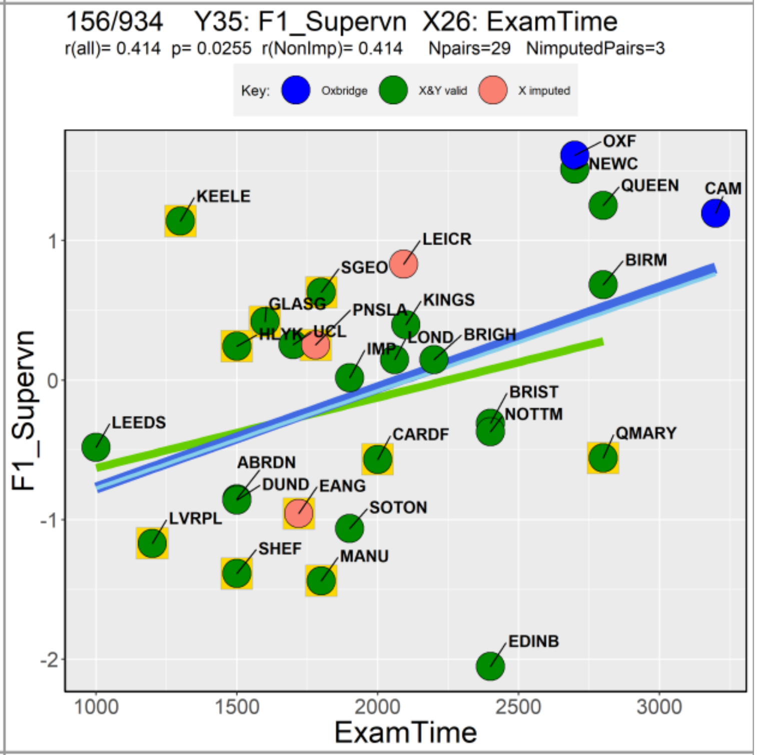
156/934 Y35: F1_Supervn X26: ExamTime
r(all)= 0.414 p= 0.0255 r(NonImp)= 0.414 Npairs=29 NimputedPairs=3
Key: Oxbridge X&Y valid X imputed
F1_Supervn
ExamTime

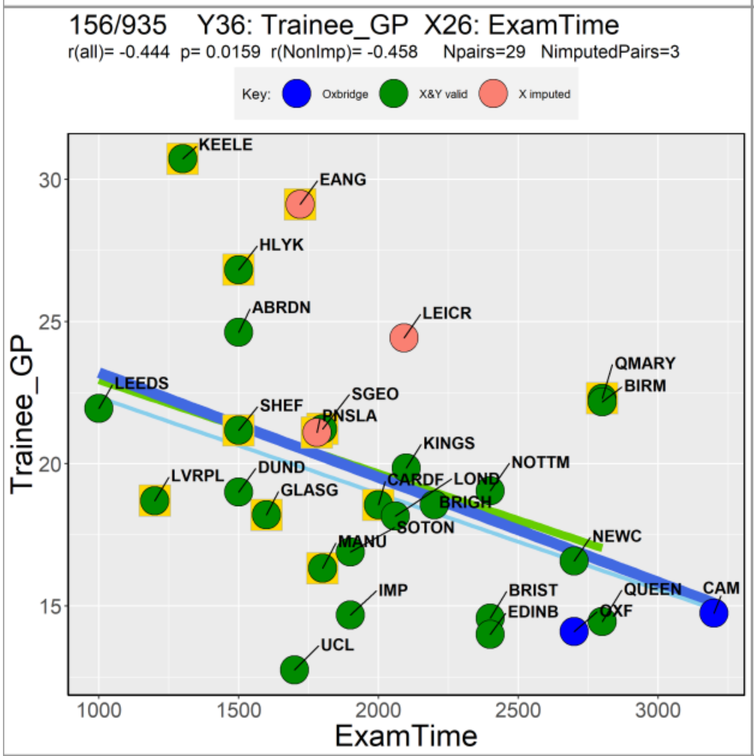
156/935 Y36: Trainee_GP X26: ExamTime
r(all)= -0.444 p= 0.0159 r(NonImp)= -0.458 Npairs=29 NimputedPairs=3
Key: Oxbridge X&Y valid X imputed
Trainee_GP
ExamTime

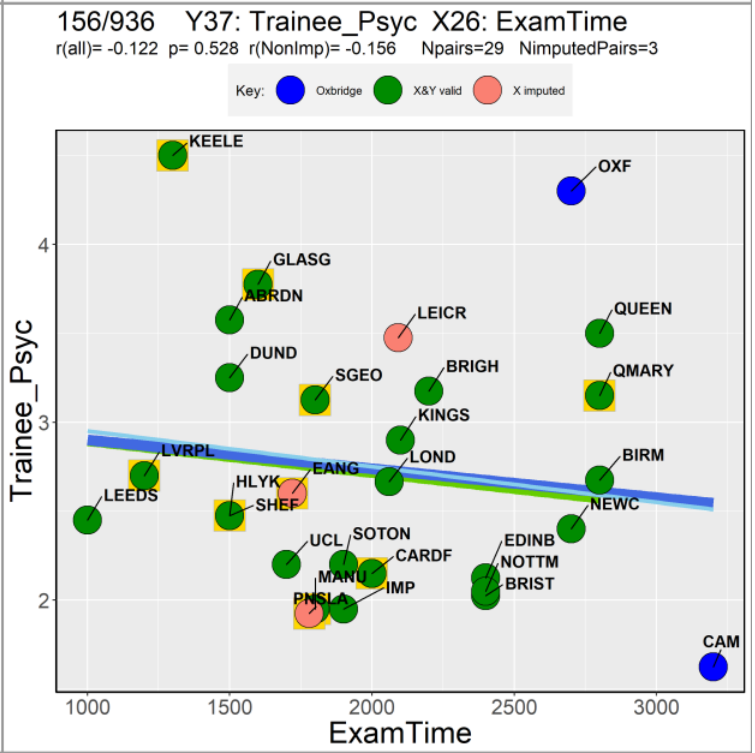
156/936 Y37: Trainee_Psyc X26: ExamTime
r(all)= -0.122 p= 0.528 r(NonImp)= -0.156 Npairs=29 NimputedPairs=3
Key: Oxbridge X&Y valid X imputed
Trainee_Psyc
ExamTime

157/937 Y38: TraineeApp_Surgery X26: ExamTime
r(all)= 0.202 p= 0.293 r(NonImp)= 0.164 Npairs=29 NimputedPairs=5

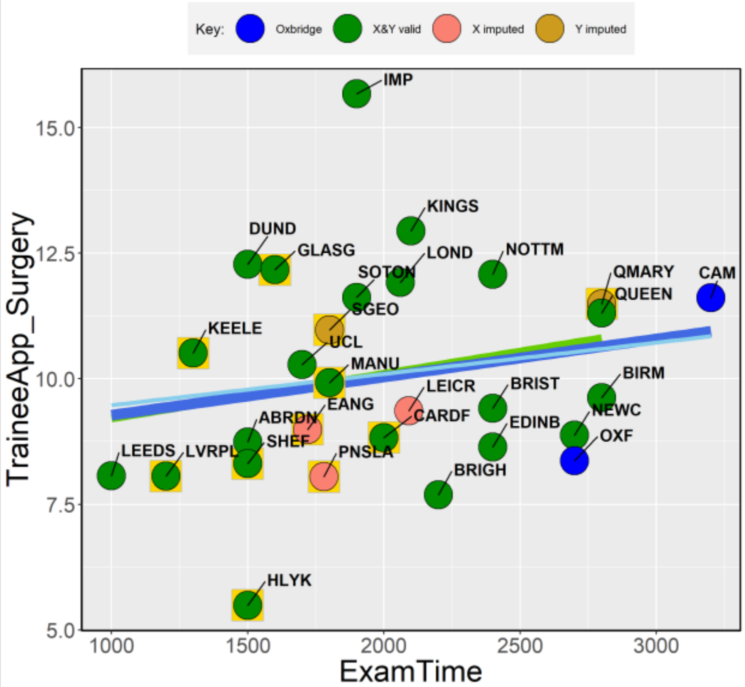

157/938 Y39: TraineeApp_Anaes X26: ExamTime
r(all)= 0.032 p= 0.869 r(NonImp)= 0.060 Npairs=29 NimputedPairs=3

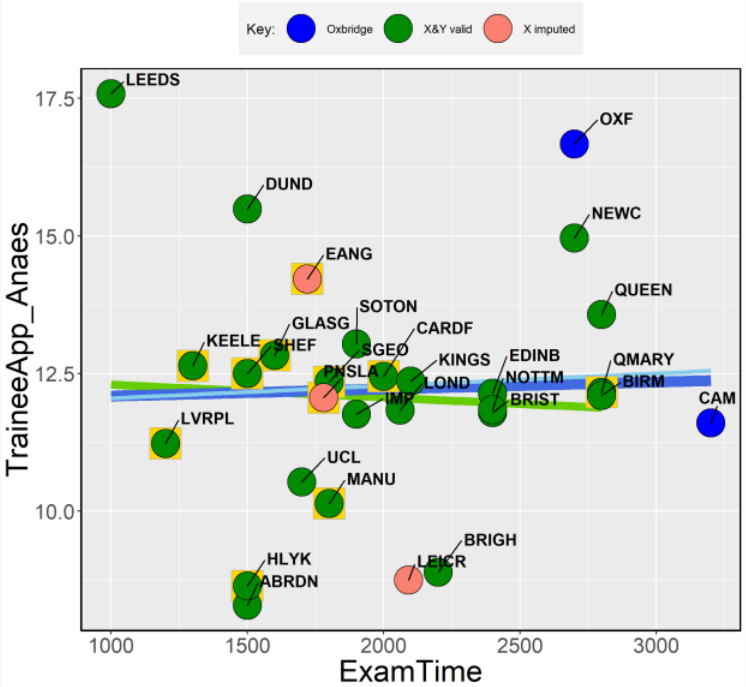

157/939 Y40: GMC_PGexams X26: ExamTime
r(all)= 0.522 p= 0.00365 r(NonImp)= 0.514 Npairs=29 NimputedPairs=3

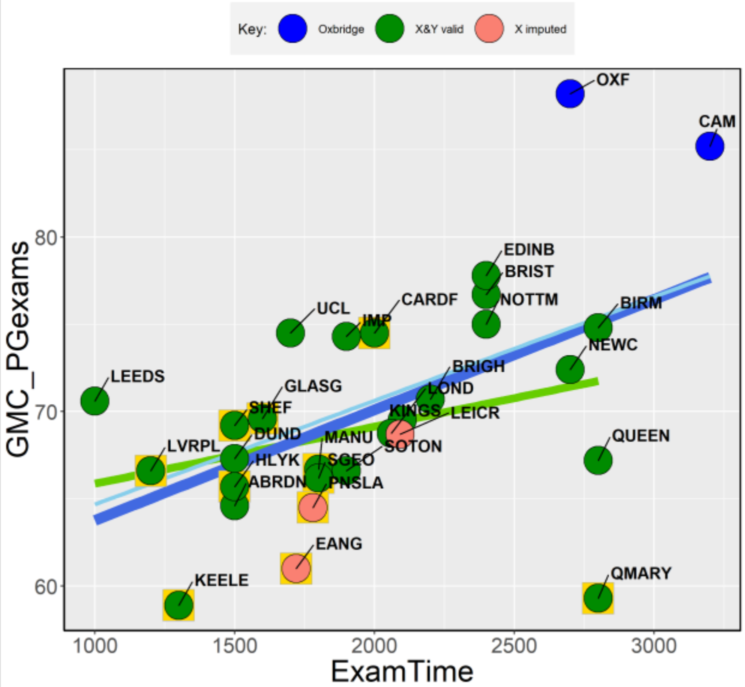

157/940 Y41: MRCGP_AKT X26: ExamTime
r(all)= 0.621 p= 0.000329 r(NonImp)= 0.624 Npairs=29 NimputedPairs=3

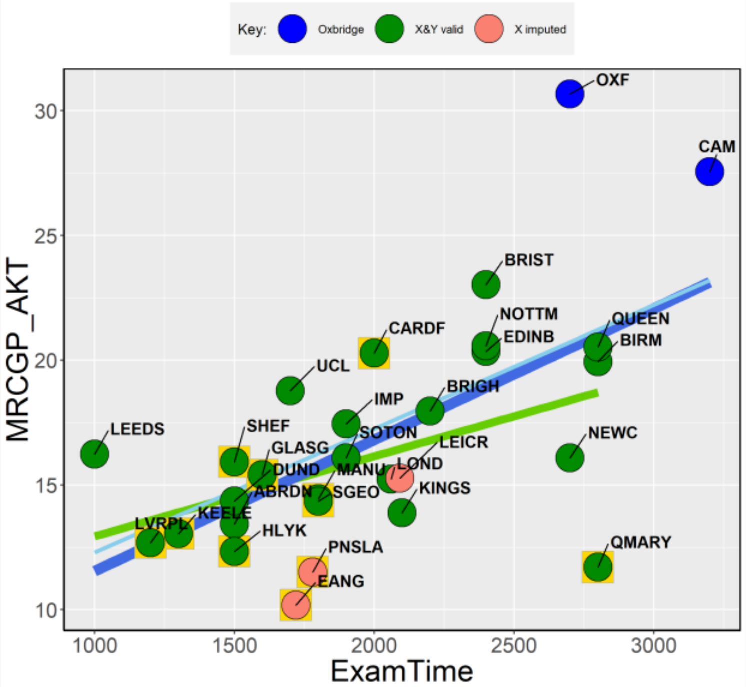

157/941 Y42: MRCGP_CSA X26: ExamTime
r(all)= 0.525 p= 0.00348 r(NonImp)= 0.514 Npairs=29 NimputedPairs=3

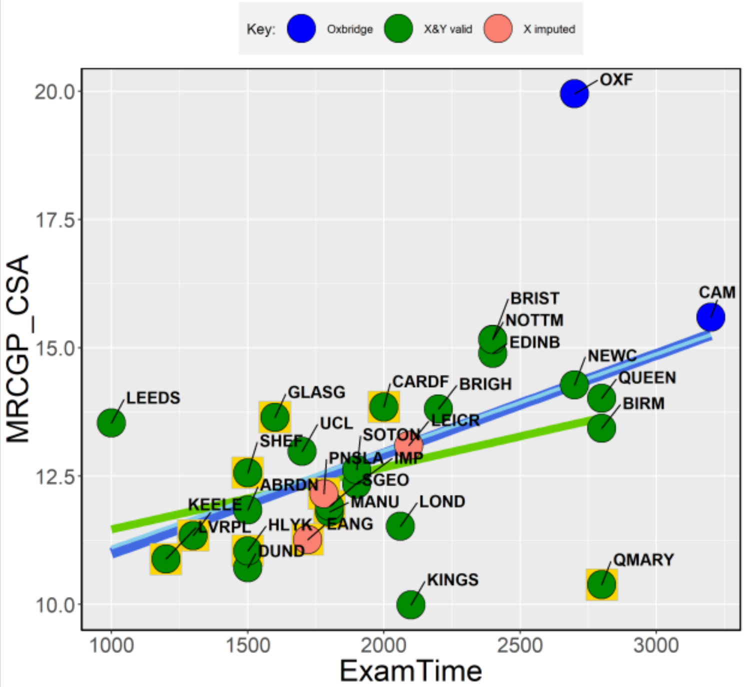

157/942 Y43: FRCA_Pt1 X26: ExamTime
r(all)= 0.334 p= 0.077 r(NonImp)= 0.373 Npairs=29 NimputedPairs=11

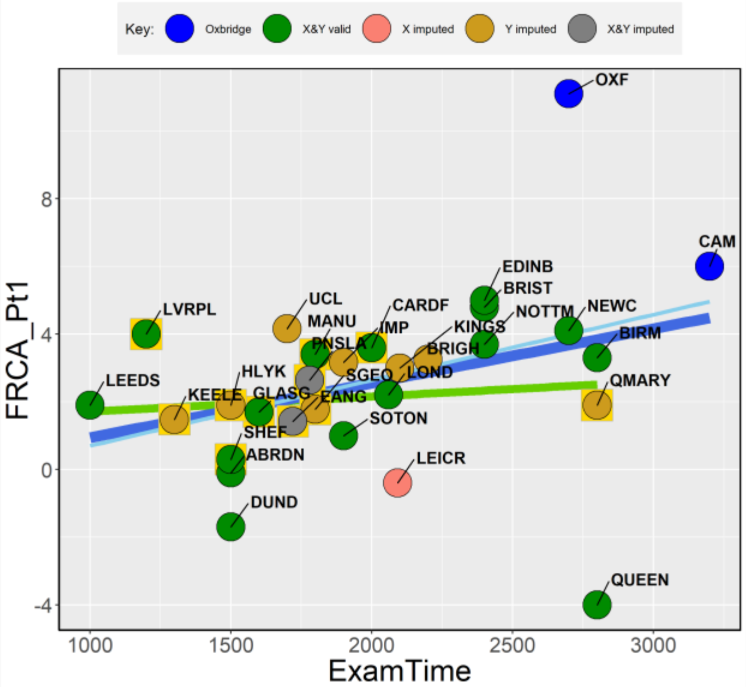

158/943 Y44: MRCOG_Pt1 X26: ExamTime
r(all)= 0.519 p= 0.00394 r(NonImp)= 0.589 Npairs=29 NimputedPairs=11

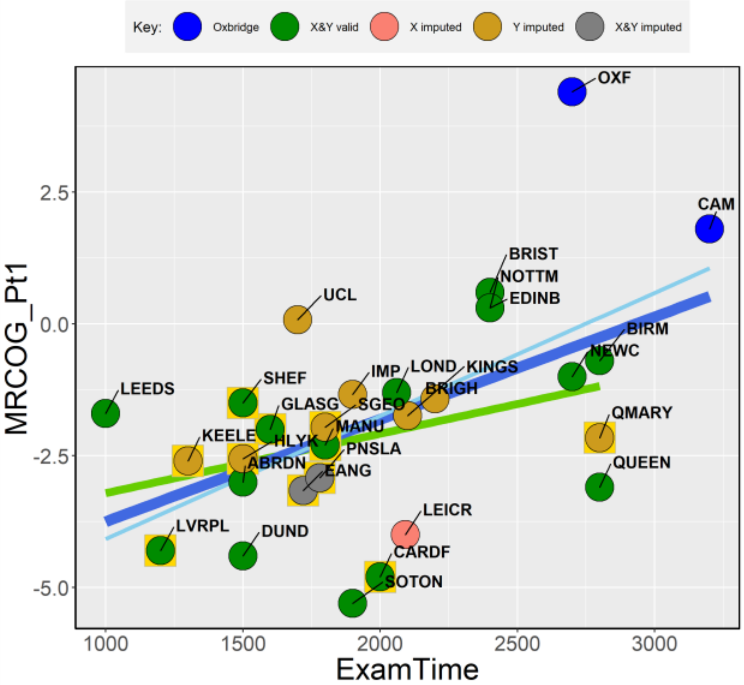

158/944 Y45: MRCOG_Pt2 X26: ExamTime
r(all)= 0.386 p= 0.0384 r(NonImp)= 0.491 Npairs=29 NimputedPairs=11

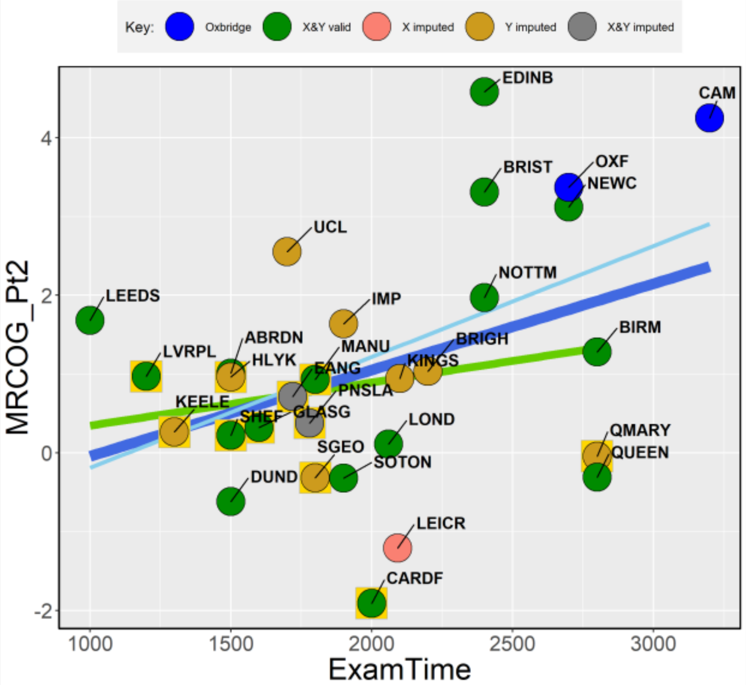

158/945 Y46: MRCP_Pt1 X26: ExamTime
r(all)= 0.538 p= 0.00261 r(NonImp)= 0.525 Npairs=29 NimputedPairs=6

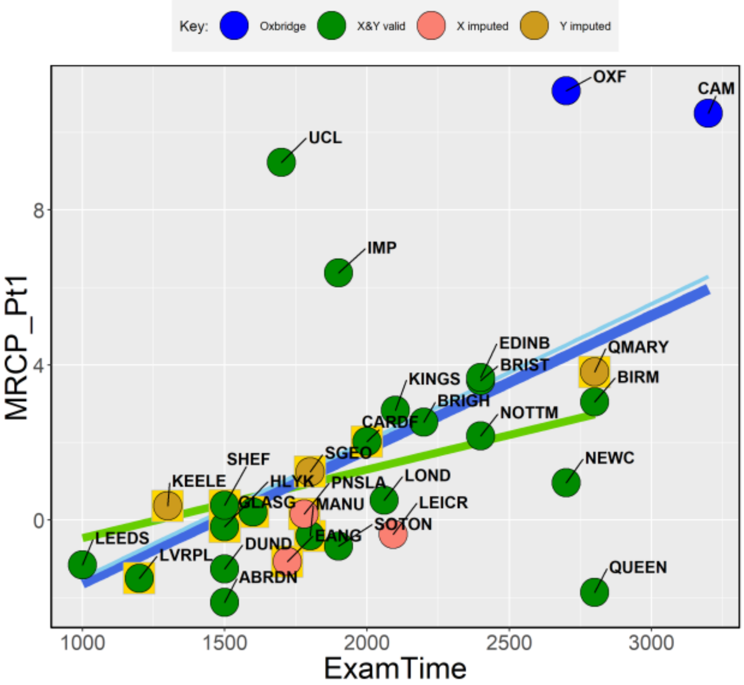

158/946 Y47: MRCP_Pt2 X26: ExamTime
r(all)= 0.437 p= 0.0178 r(NonImp)= 0.504 Npairs=29 NimputedPairs=6

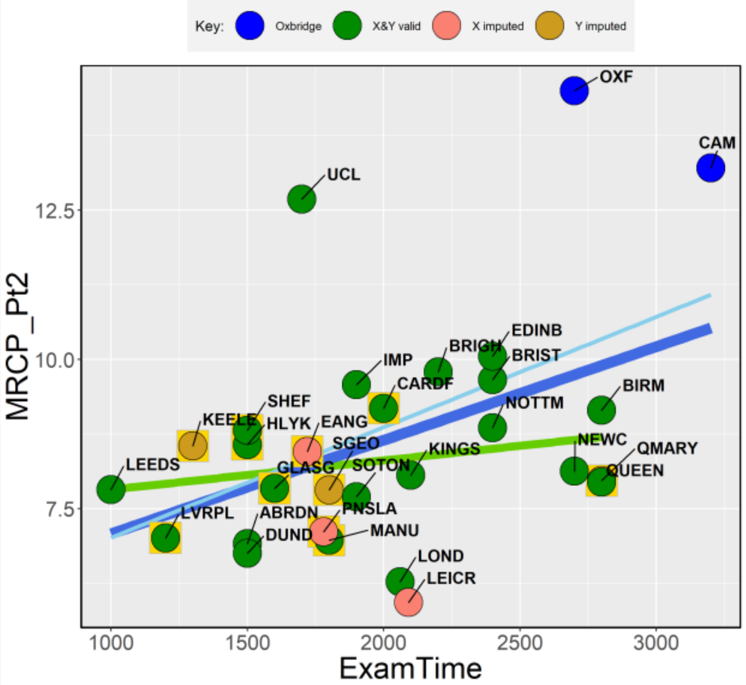

158/947 Y48: MRCP_PACES X26: ExamTime
r(all)= 0.486 p= 0.0075 r(NonImp)= 0.503 Npairs=29 NimputedPairs=6

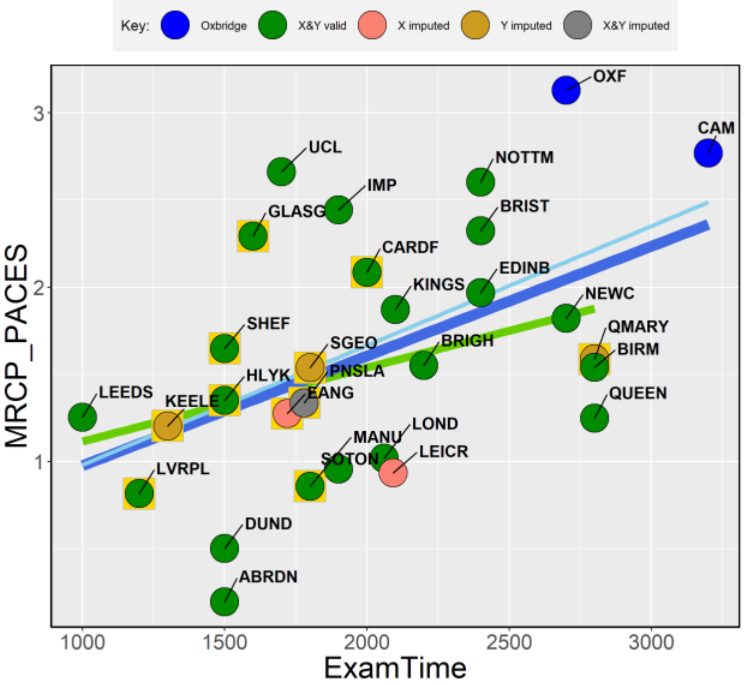

158/948 Y49: GMC_Sanctions X26: ExamTime
r(all)= -0.565 p= 0.0014 r(NonImp)= -0.621 Npairs=29 NimputedPairs=11

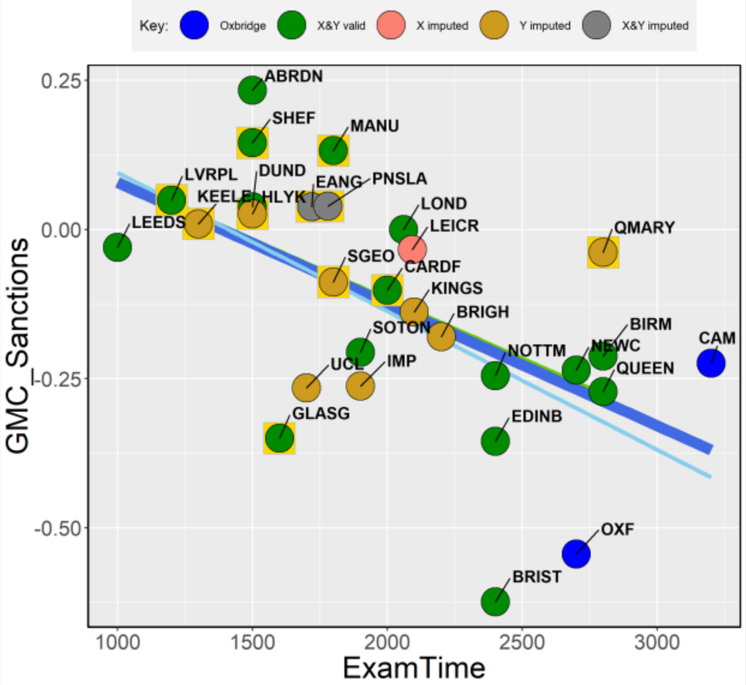

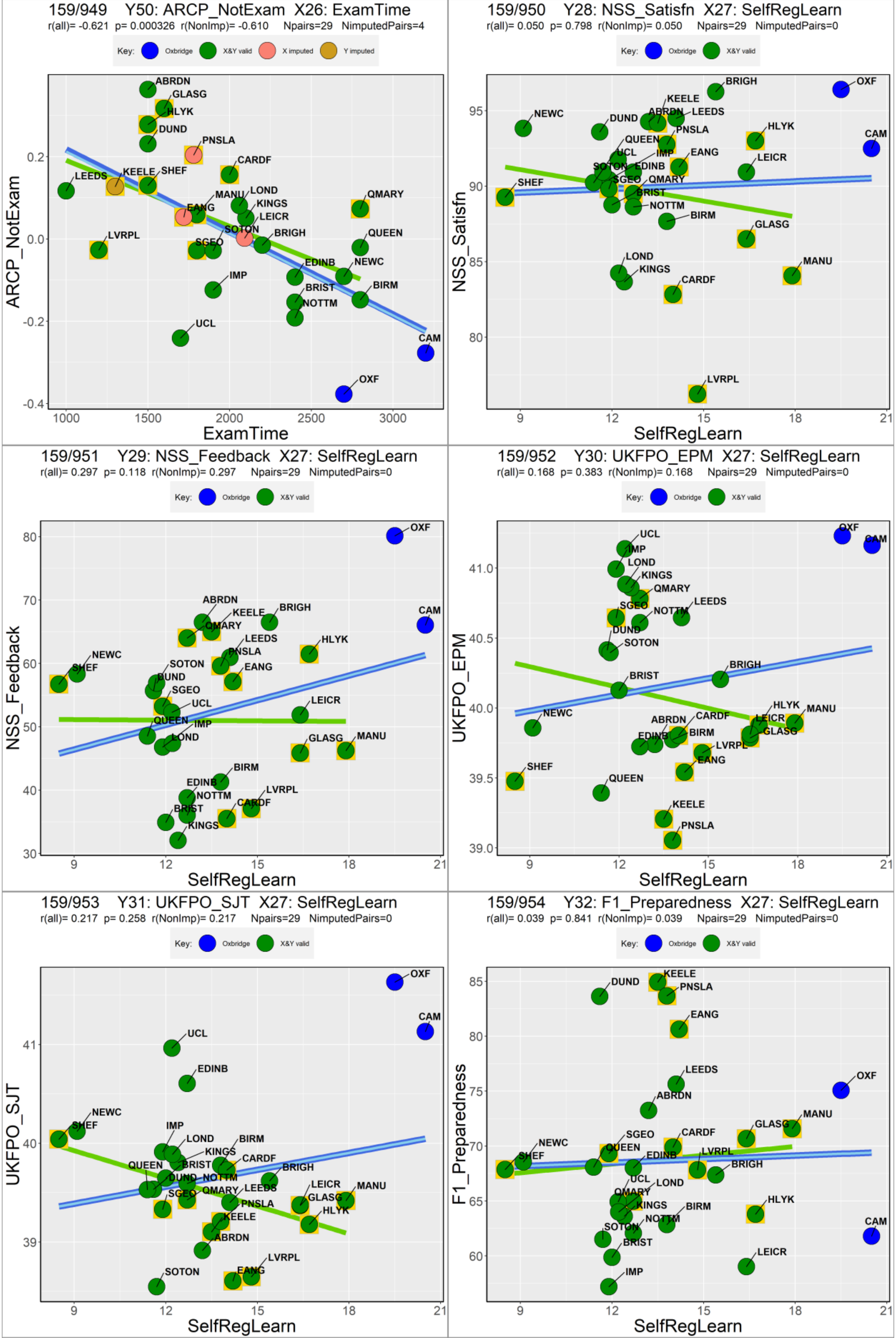
159/949 Y50: ARCP_NotExam X26: ExamTime
r(all)= -0.621 p= 0.000326 r(NonImp)= -0.610 Npairs=29 NimputedPairs=4
Key: Oxbridge X&Y valid X imputed Y imputed
ARCP_NotExam
ExamTime
159/950 Y28: NSS_Satisfn X27: SelfRegLearn
r(all)= 0.050 p= 0.798 r(NonImp)= 0.050 Npairs=29 NimputedPairs=0
Key: Oxbridge X&Y valid
NSS_Satisfn
SelfRegLearn
159/951 Y29: NSS_Feedback X27: SelfRegLearn
r(all)= 0.297 p= 0.118 r(NonImp)= 0.297 Npairs=29 NimputedPairs=0
Key: Oxbridge X&Y valid
NSS_Feedback
SelfRegLearn
159/952 Y30: UKFPO_EPM X27: SelfRegLearn
r(all)= 0.168 p= 0.383 r(NonImp)= 0.168 Npairs=29 NimputedPairs=0
Key: Oxbridge X&Y valid
UKFPO_EPM
SelfRegLearn
159/953 Y31: UKFPO_SJT X27: SelfRegLearn
r(all)= 0.217 p= 0.258 r(NonImp)= 0.217 Npairs=29 NimputedPairs=0
Key: Oxbridge X&Y valid
UKFPO_SJT
SelfRegLearn
159/954 Y32: F1_Preparedness X27: SelfRegLearn
r(all)= 0.039 p= 0.841 r(NonImp)= 0.039 Npairs=29 NimputedPairs=0
Key: Oxbridge X&Y valid
F1_Preparedness
SelfRegLearn

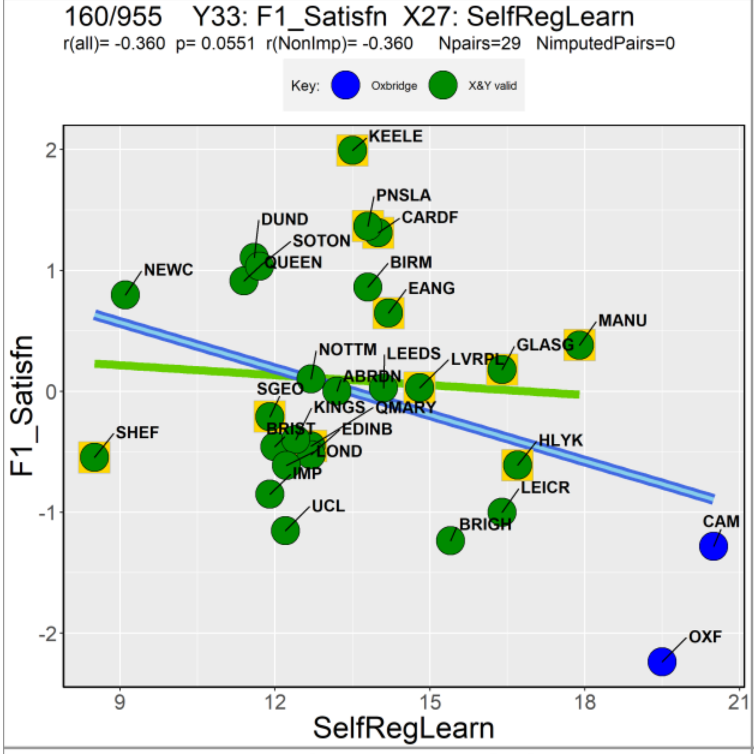
160/955 Y33: F1_Satisfn X27: SelfRegLearn
r(all)= -0.360 p= 0.0551 r(NonImp)= -0.360 Npairs=29 NimputedPairs=0
Key: Oxbridge X&Y valid
F1_Satisfn
SelfRegLearn
KEELE
PNSLA
CARDF
DUND
SOTON
QUEEN
NEWC
BIRM
EANG
MANU
NOTTM
LEEDS
LVRPL
GLASG
ABRDN
SGEO
KINGS
QMARY
BRIST
EDINB
LOND
IMP
SHEF
HLYK
LEICR
UCL
BRIGH
CAM
OXF

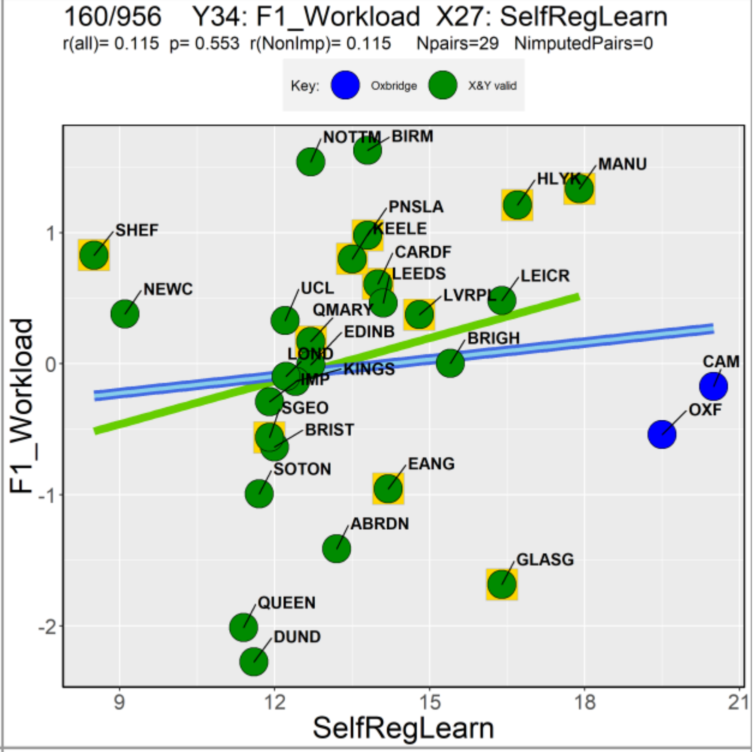
160/956 Y34: F1_Workload X27: SelfRegLearn
r(all)= 0.115 p= 0.553 r(NonImp)= 0.115 Npairs=29 NimputedPairs=0
Key: Oxbridge X&Y valid
F1_Workload
SelfRegLearn
NOTTM
BIRM
MANU
HLYK
PNSLA
KEELE
CARDF
LEEDS
SHEF
NEWC
UCL
LVRPL
LEICR
QMARY
EDINB
BRIGH
LOND
IMP
KINGS
SGEO
BRIST
SOTON
EANG
ABRDN
GLASG
QUEEN
DUND
CAM
OXF

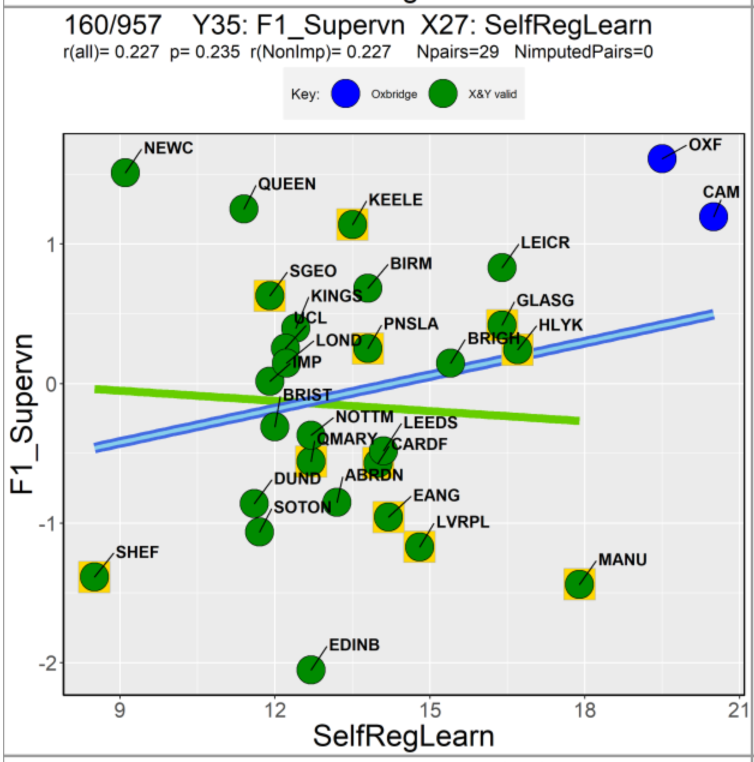
160/957 Y35: F1_Supervn X27: SelfRegLearn
r(all)= 0.227 p= 0.235 r(NonImp)= 0.227 Npairs=29 NimputedPairs=0
Key: Oxbridge X&Y valid
F1_Supervn
SelfRegLearn
NEWC
QUEEN
KEELE
OXF
CAM
LEICR
SGEO
BIRM
KINGS
GLASG
UCL
PNSLA
HLYK
LOND
BRIGH
IMP
BRIST
NOTTM
LEEDS
QMARY
CARDF
DUND
ABRDN
SOTON
EANG
LVRPL
SHEF
MANU
EDINB

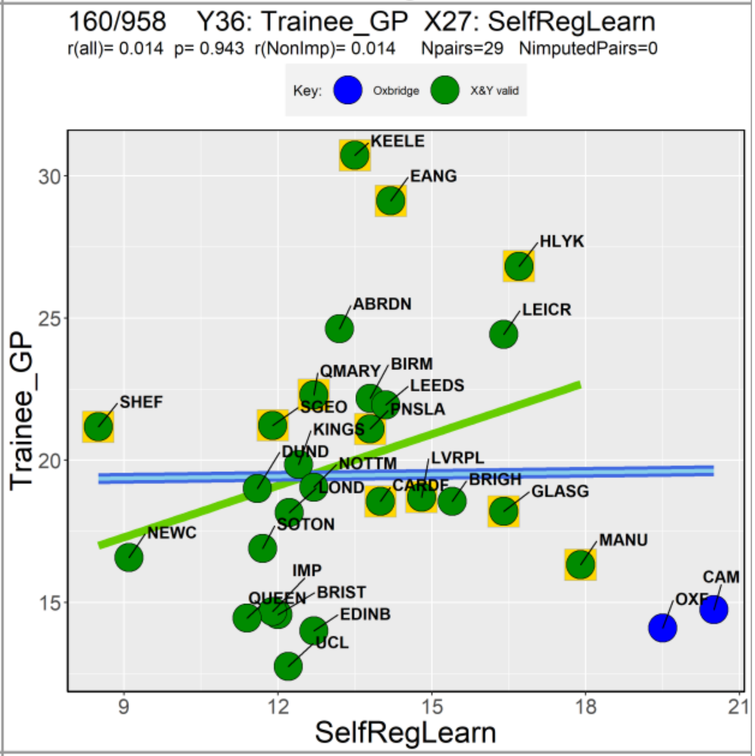
160/958 Y36: Trainee_GP X27: SelfRegLearn
r(all)= 0.014 p= 0.943 r(NonImp)= 0.014 Npairs=29 NimputedPairs=0
Key: Oxbridge X&Y valid
Trainee_GP
SelfRegLearn
KEELE
EANG
HLYK
ABRDN
LEICR
QMARY
BIRM
LEEDS
SHEF
SGEO
PNSLA
KINGS
DUND
NOTTM
LVRPL
LOND
CARDF
BRIGH
GLASG
SOTON
NEWC
MANU
IMP
QUEEN
BRIST
EDINB
UCL
CAM
OXF

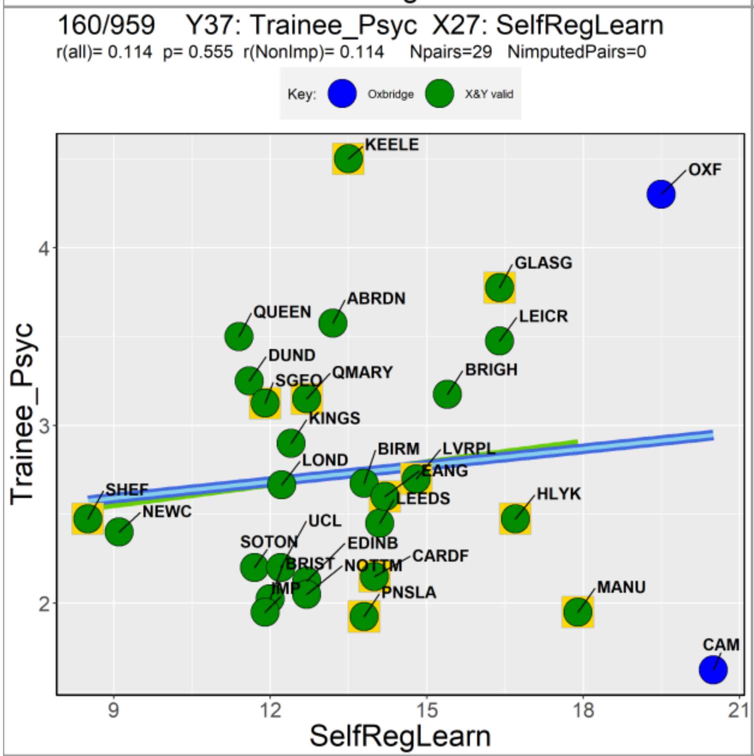
160/959 Y37: Trainee_Psyc X27: SelfRegLearn
r(all)= 0.114 p= 0.555 r(NonImp)= 0.114 Npairs=29 NimputedPairs=0
Key: Oxbridge X&Y valid
Trainee_Psyc
SelfRegLearn
KEELE
OXF
GLASG
QUEEN
ABRDN
LEICR
DUND
SGEO
QMARY
BRIGH
KINGS
LOND
BIRM
LVRPL
EANG
SHEF
LEEDS
NEWC
HLYK
UCL
SOTON
EDINB
BRIST
NOTTM
CARDF
IMP
PNSLA
MANU
CAM

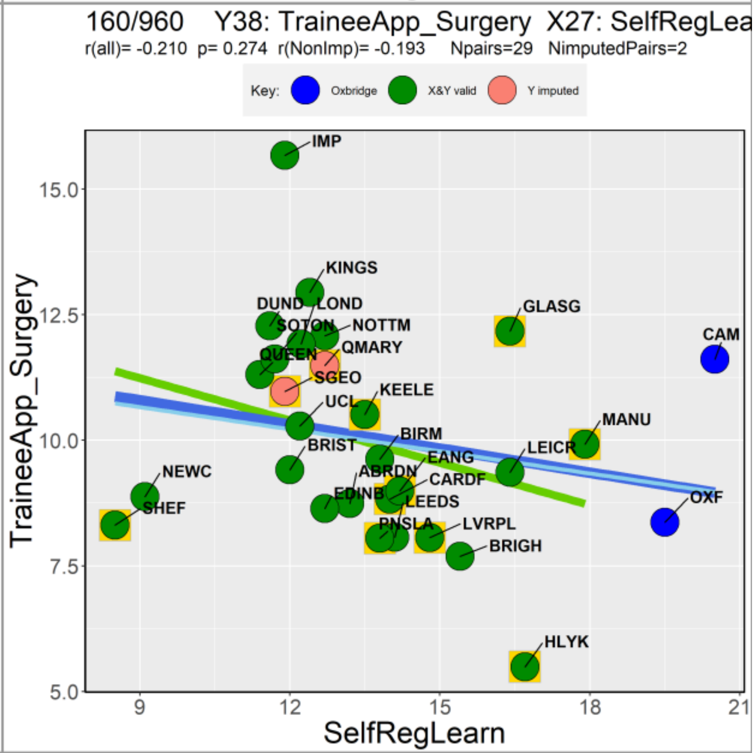
160/960 Y38: TraineeApp_Surgery X27: SelfRegLea
r(all)= -0.210 p= 0.274 r(NonImp)= -0.193 Npairs=29 NimputedPairs=2
Key: Oxbridge X&Y valid Y imputed
TraineeApp_Surgery
SelfRegLearn
IMP
KINGS
DUND
LOND
GLASG
SOTON
NOTTM
QMARY
QUEEN
CAM
SGEO
KEELE
UCL
MANU
BIRM
BRIST
LEICR
EANG
NEWC
ABRDN
CARDF
OXF
SHEF
EDINB
LEEDS
PNSLA
LVRPL
BRIGH
HLYK

161/961 Y39: TraineeApp_Anaes X27: SelfRegLearn
r(all)= -0.169 p= 0.381 r(NonImp)= -0.169 Npairs=29 NimputedPairs=0

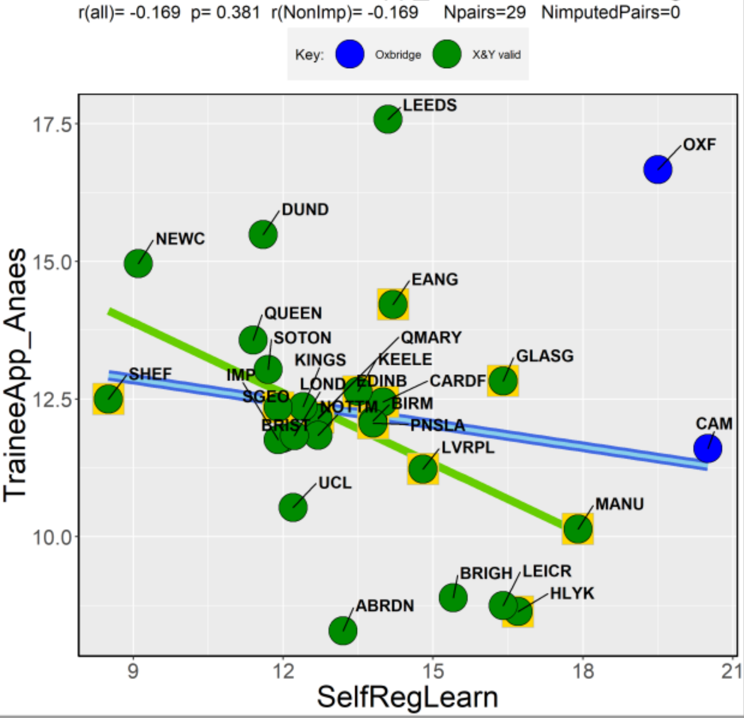

161/962 Y40: GMC_PGexams X27: SelfRegLearn
r(all)= 0.336 p= 0.0747 r(NonImp)= 0.336 Npairs=29 NimputedPairs=0

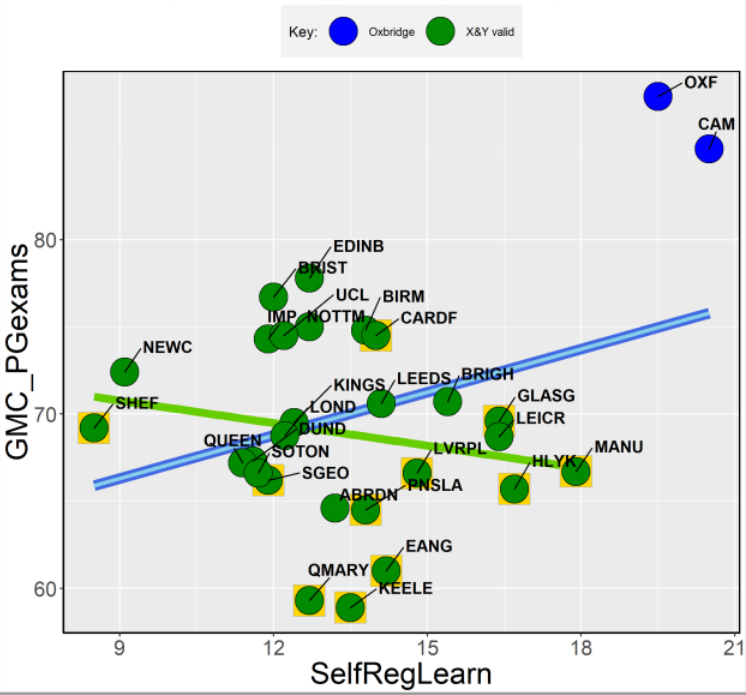

161/963 Y41: MRCGP_AKT X27: SelfRegLearn
r(all)= 0.335 p= 0.0753 r(NonImp)= 0.335 Npairs=29 NimputedPairs=0

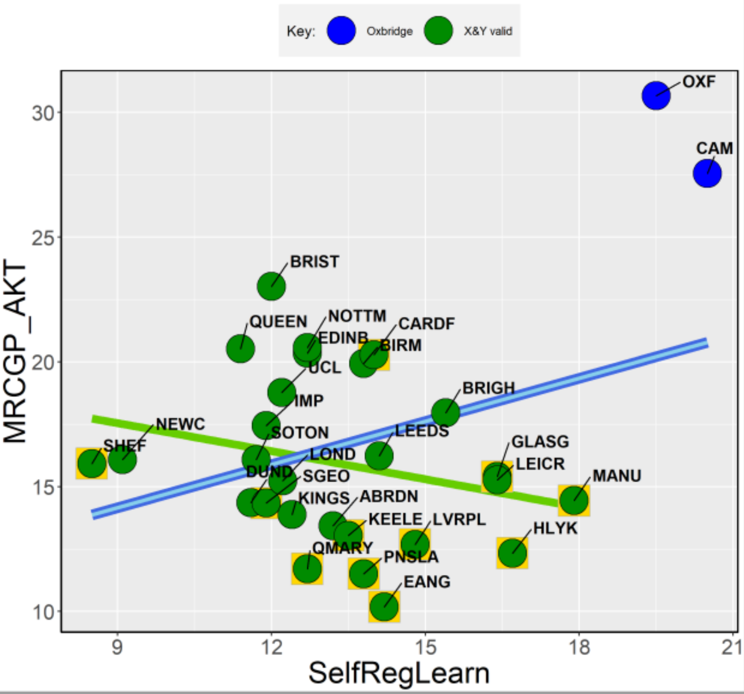

161/964 Y42: MRCGP_CSA X27: SelfRegLearn
r(all)= 0.339 p= 0.0721 r(NonImp)= 0.339 Npairs=29 NimputedPairs=0

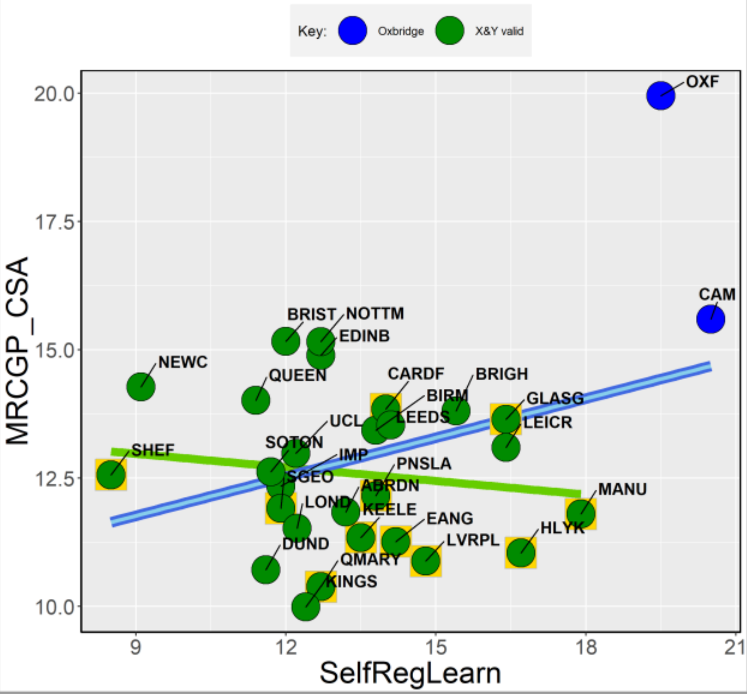

161/965 Y43: FRCA_Pt1 X27: SelfRegLearn
r(all)= 0.451 p= 0.014 r(NonImp)= 0.505 Npairs=29 NimputedPairs=10

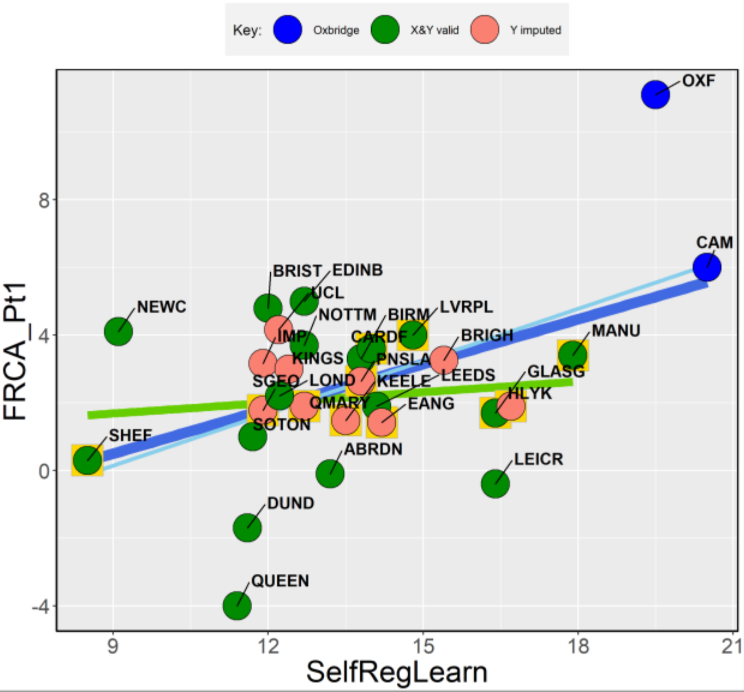

161/966 Y44: MRCOG_Pt1 X27: SelfRegLearn
r(all)= 0.286 p= 0.133 r(NonImp)= 0.351 Npairs=29 NimputedPairs=10

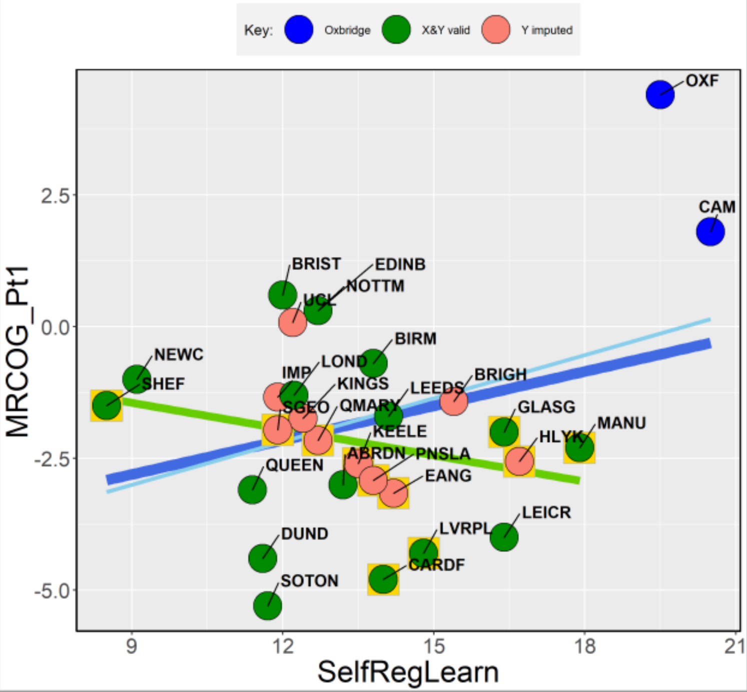

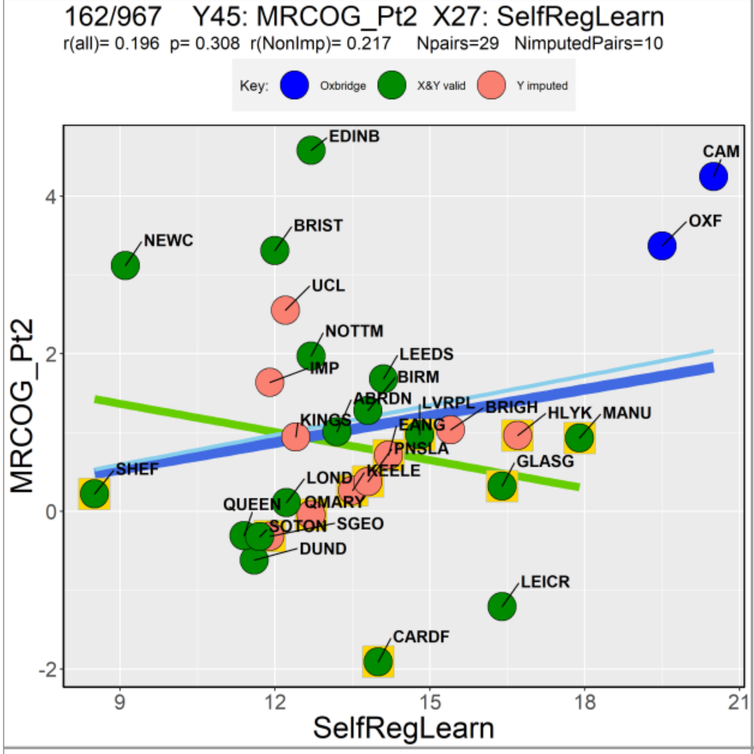
162/967 Y45: MRCOG_Pt2 X27: SelfRegLearn
r(all)= 0.196 p= 0.308 r(NonImp)= 0.217 Npairs=29 NimputedPairs=10
Key: Oxbridge X&Y valid Y imputed
MRCOG_Pt2
SelfRegLearn
EDINB CAM OXF NEWC BRIST UCL NOTTM LEEDS IMP BIRM ABRDN LVRPL BRIGH HLYK MANU KINGS EANG PNSLA GLASG SHEF LOND KEELE QUEEN QMARY SOTON SGEO DUND LEICR CARDF

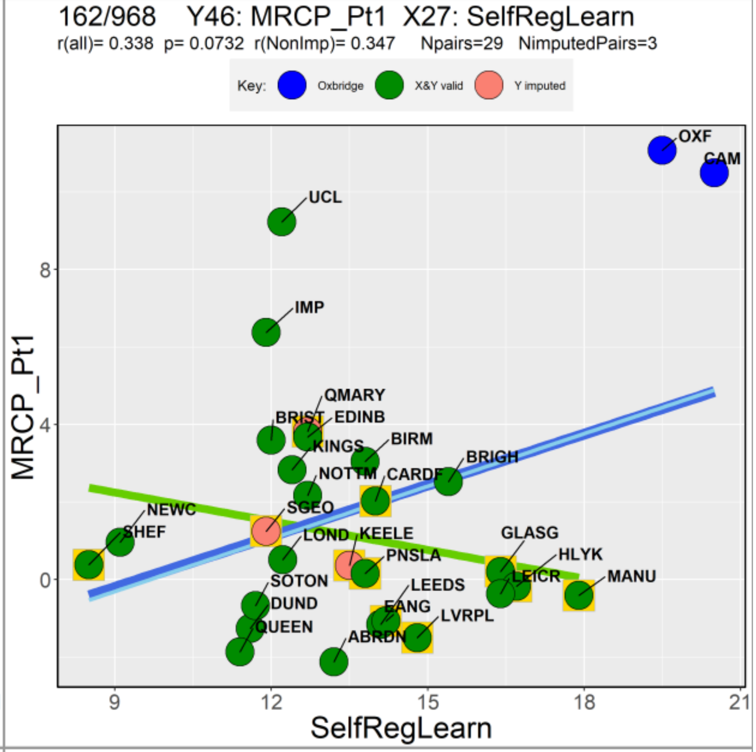
162/968 Y46: MRCP_Pt1 X27: SelfRegLearn
r(all)= 0.338 p= 0.0732 r(NonImp)= 0.347 Npairs=29 NimputedPairs=3
Key: Oxbridge X&Y valid Y imputed
MRCP_Pt1
SelfRegLearn
OXF CAM UCL IMP QMARY BRIST EDINB KINGS BIRM NOTTM CARDF BRIGH NEWC SGEO SHEF LOND KEELE GLASG PNSLA HLYK LEICR MANU SOTON LEEDS DUND EANG QUEEN LVRPL ABRDN

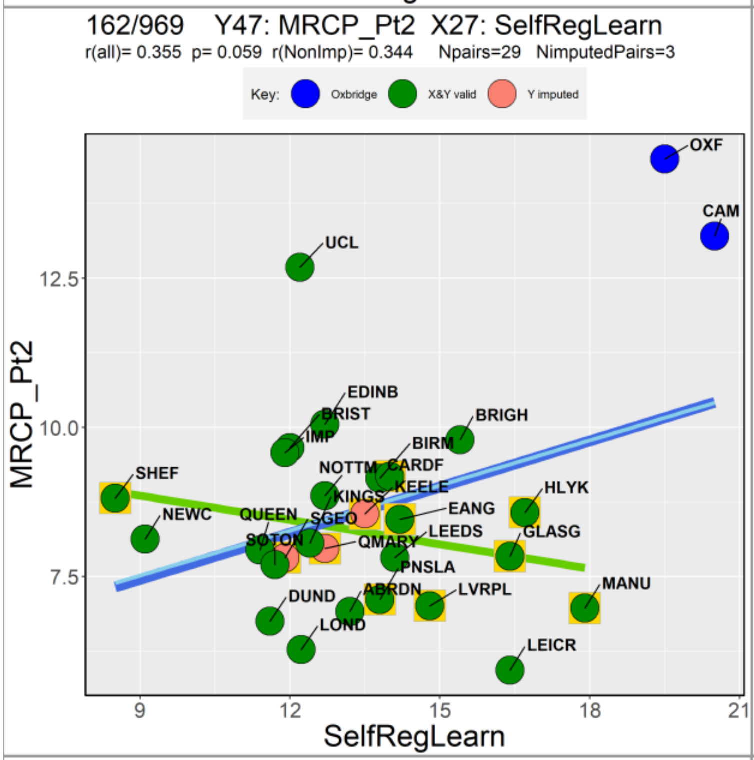
162/969 Y47: MRCP_Pt2 X27: SelfRegLearn
r(all)= 0.355 p= 0.059 r(NonImp)= 0.344 Npairs=29 NimputedPairs=3
Key: Oxbridge X&Y valid Y imputed
MRCP_Pt2
SelfRegLearn
OXF CAM UCL EDINB BRIST IMP BRIGH BIRM NOTTM CARDF KEELE KINGS SHEF HLYK NEWC QUEEN SGEO EANG SOTON QMARY LEEDS GLASG PNSLA DUND ABRDN LVRPL MANU LOND LEICR

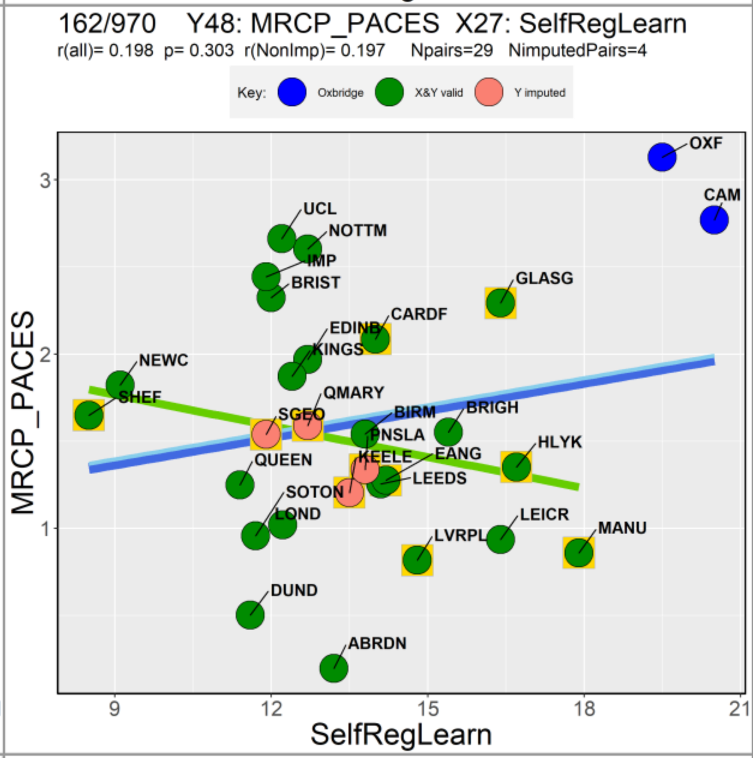
162/970 Y48: MRCP_PACES X27: SelfRegLearn
r(all)= 0.198 p= 0.303 r(NonImp)= 0.197 Npairs=29 NimputedPairs=4
Key: Oxbridge X&Y valid Y imputed
MRCP_PACES
SelfRegLearn
OXF CAM UCL NOTTM IMP BRIST GLASG CARDF EDINB KINGS NEWC SHEF QMARY SGEO BIRM BRIGH PNSLA HLYK QUEEN KEELE EANG LEEDS SOTON LOND LEICR LVRPL MANU DUND ABRDN

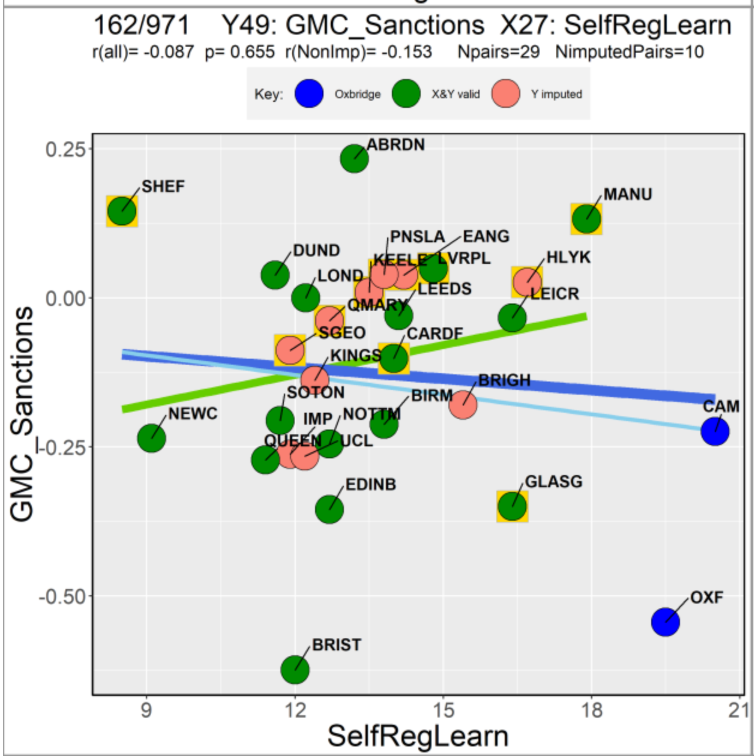
162/971 Y49: GMC_Sanctions X27: SelfRegLearn
r(all)= -0.087 p= 0.655 r(NonImp)= -0.153 Npairs=29 NimputedPairs=10
Key: Oxbridge X&Y valid Y imputed
GMC_Sanctions
SelfRegLearn
ABRDN SHEF MANU PNSLA EANG DUND KEELE LVRPL HLYK LOND LEEDS LEICR QMARY SGEO CARDF KINGS BRIGH SOTON BIRM NEWC IMP NOTTM CAM QUEEN UCL EDINB GLASG OXF BRIST

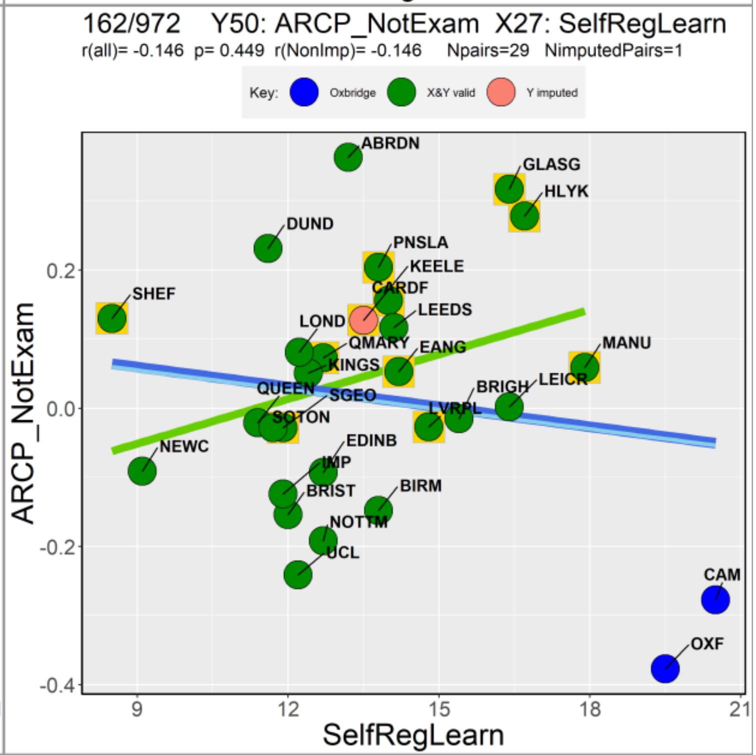
162/972 Y50: ARCP_NotExam X27: SelfRegLearn
r(all)= -0.146 p= 0.449 r(NonImp)= -0.146 Npairs=29 NimputedPairs=1
Key: Oxbridge X&Y valid Y imputed
ARCP_NotExam
SelfRegLearn
ABRDN GLASG HLYK DUND PNSLA KEELE SHEF CARDF LEEDS LOND QMARY EANG MANU KINGS BRIGH LEICR QUEEN SGEO SOTON LVRPL NEWC EDINB IMP BRIST BIRM NOTTM UCL CAM OXF

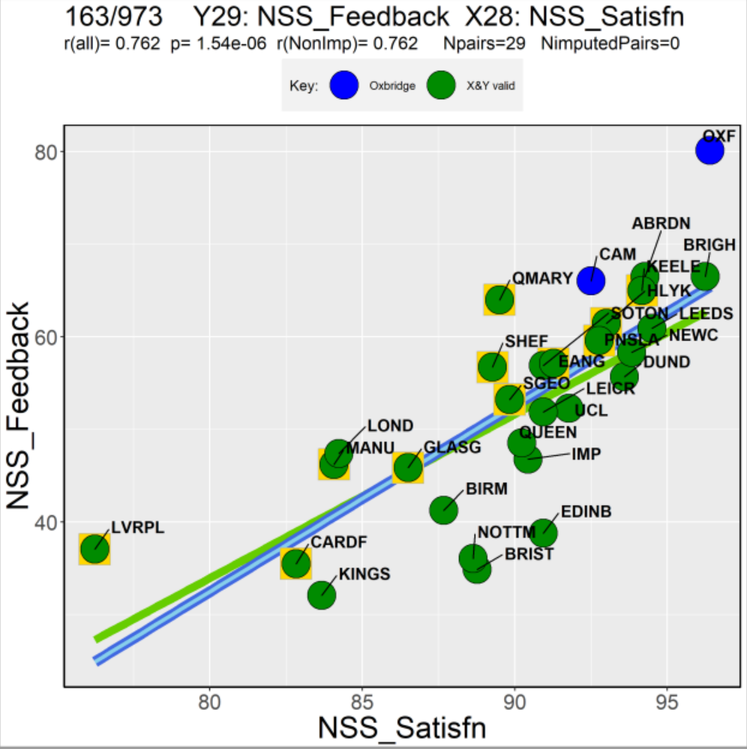
163/973 Y29: NSS_Feedback X28: NSS_Satisfn
r(all)= 0.762 p= 1.54e-06 r(NonImp)= 0.762 Npairs=29 NimputedPairs=0
Key: Oxbridge X&Y valid
NSS_Feedback
NSS_Satisfn
80
60
40
80
85
90
95
OXF
ABRDN
BRIGH
CAM
KEELE
QMARY
HLYK
SOTON
LEEDS
SHEF
PNSLA
NEWC
EANG
DUND
SGEO
LEICR
UCL
LOND
QUEEN
MANU
GLASG
IMP
BIRM
EDINB
LVRPL
NOTTM
CARDF
BRIST
KINGS

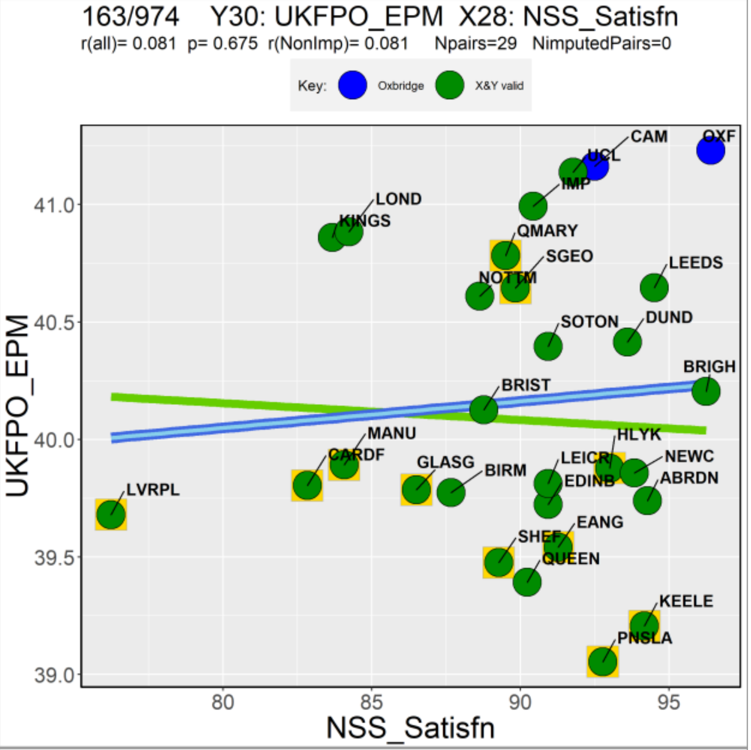
163/974 Y30: UKFPO_EPM X28: NSS_Satisfn
r(all)= 0.081 p= 0.675 r(NonImp)= 0.081 Npairs=29 NimputedPairs=0
Key: Oxbridge X&Y valid
UKFPO_EPM
NSS_Satisfn
41.0
40.5
40.0
39.5
39.0
80
85
90
95
CAM
OXF
UCL
IMP
LOND
KINGS
QMARY
SGEO
LEEDS
NOTTM
SOTON
DUND
BRIGH
BRIST
MANU
HLYK
CARDF
LEICR
NEWC
GLASG
BIRM
EDINB
ABRDN
LVRPL
EANG
SHEF
QUEEN
KEELE
PNSLA

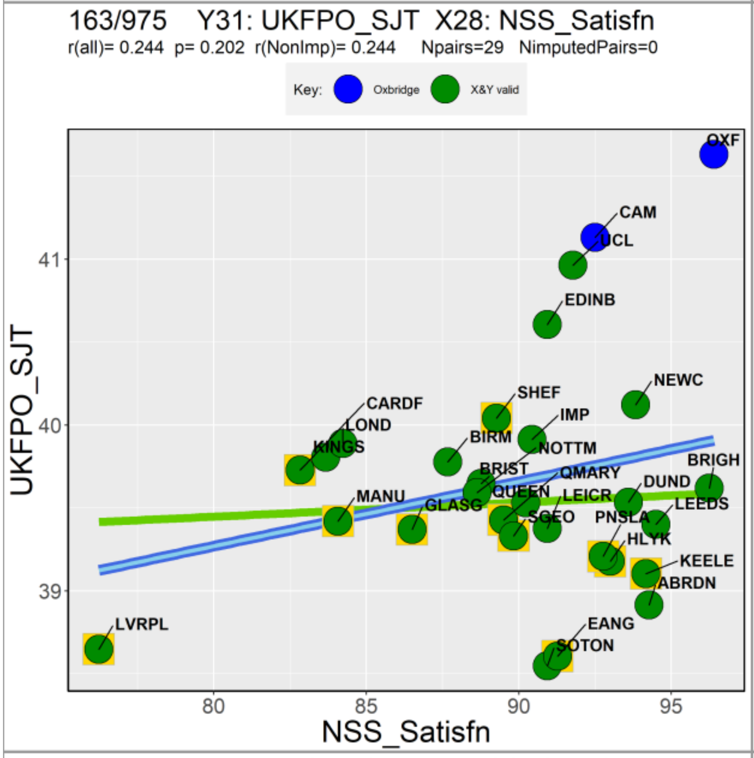
163/975 Y31: UKFPO_SJT X28: NSS_Satisfn
r(all)= 0.244 p= 0.202 r(NonImp)= 0.244 Npairs=29 NimputedPairs=0
Key: Oxbridge X&Y valid
UKFPO_SJT
NSS_Satisfn
41
40
39
80
85
90
95
OXF
CAM
UCL
EDINB
NEWC
SHEF
CARDF
IMP
LOND
BIRM
KINGS
NOTTM
BRIST
QMARY
BRIGH
DUND
QUEEN
MANU
LEICR
GLASG
LEEDS
SGEO
PNSLA
HLYK
KEELE
ABRDN
LVRPL
EANG
SOTON

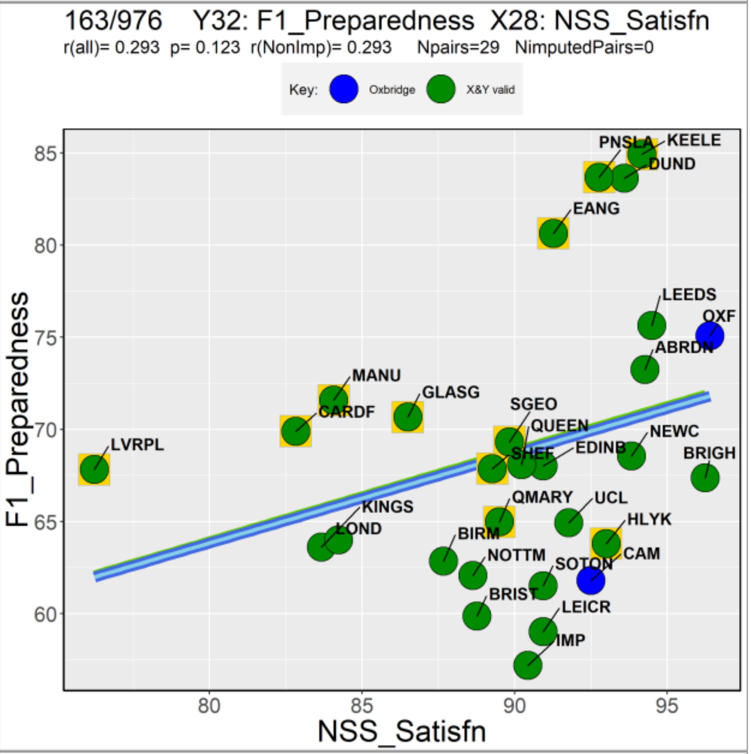
163/976 Y32: F1_Preparedness X28: NSS_Satisfn
r(all)= 0.293 p= 0.123 r(NonImp)= 0.293 Npairs=29 NimputedPairs=0
Key: Oxbridge X&Y valid
F1_Preparedness
NSS_Satisfn
85
80
75
70
65
60
80
85
90
95
PNSLA
KEELE
DUND
EANG
LEEDS
OXF
ABRDN
MANU
GLASG
SGEO
CARDF
QUEEN
NEWC
LVRPL
SHEF
EDINB
BRIGH
KINGS
QMARY
UCL
LOND
HLYK
BIRM
NOTTM
CAM
SOTON
BRIST
LEICR
IMP

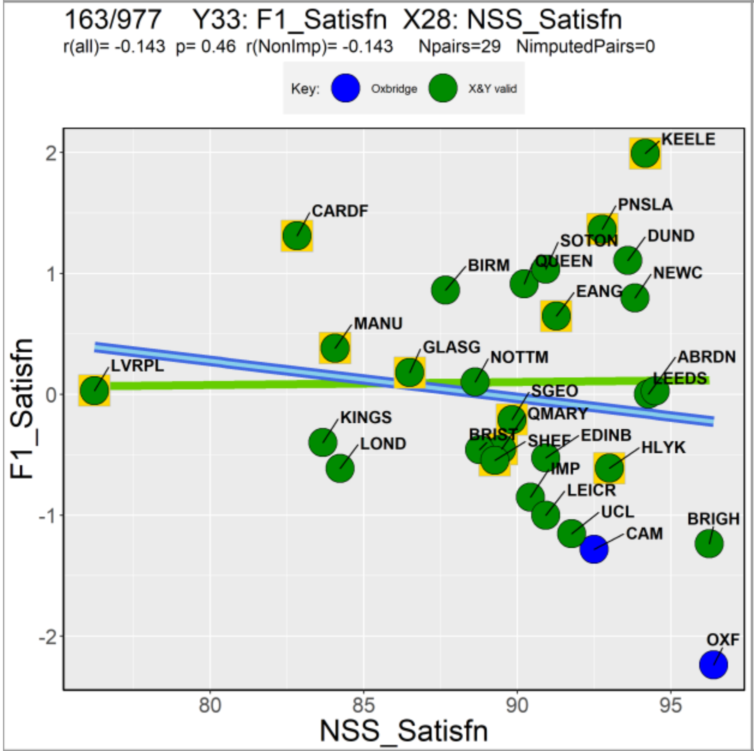
163/977 Y33: F1_Satisfn X28: NSS_Satisfn
r(all)= -0.143 p= 0.46 r(NonImp)= -0.143 Npairs=29 NimputedPairs=0
Key: Oxbridge X&Y valid
F1_Satisfn
NSS_Satisfn
2
1
0
-1
-2
80
85
90
95
KEELE
PNSLA
CARDF
SOTON
DUND
BIRM
QUEEN
NEWC
EANG
MANU
GLASG
NOTTM
ABRDN
LVRPL
LEEDS
SGEO
KINGS
QMARY
LOND
BRIST
SHEF
EDINB
HLYK
IMP
LEICR
UCL
CAM
BRIGH
OXF

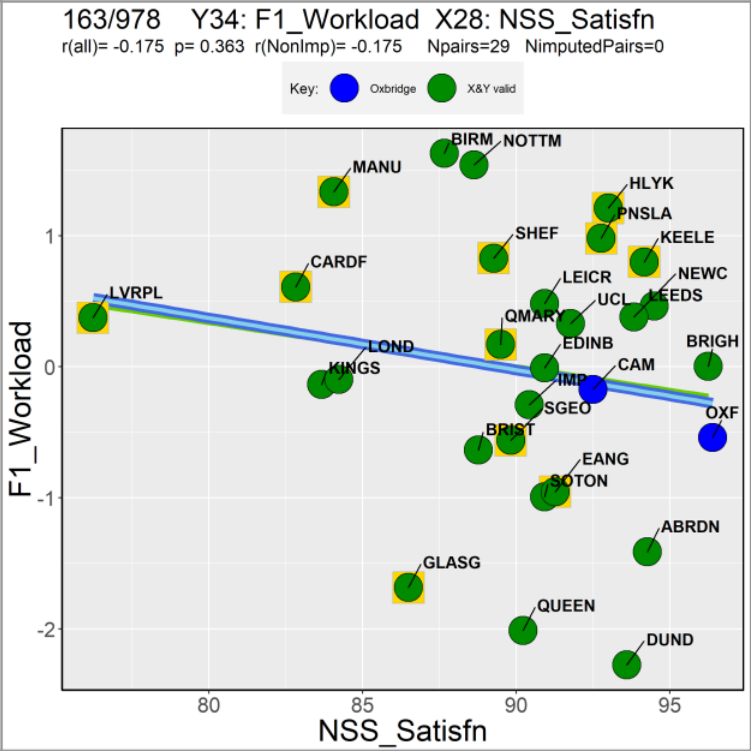
163/978 Y34: F1_Workload X28: NSS_Satisfn
r(all)= -0.175 p= 0.363 r(NonImp)= -0.175 Npairs=29 NimputedPairs=0
Key: Oxbridge X&Y valid
F1_Workload
NSS_Satisfn
1
0
-1
-2
80
85
90
95
BIRM
NOTTM
MANU
HLYK
PNSLA
SHEF
KEELE
CARDF
LEICR
NEWC
LVRPL
UCL
LEEDS
QMARY
EDINB
BRIGH
LOND
CAM
KINGS
IMP
SGEO
OXF
BRIST
EANG
SOTON
ABRDN
GLASG
QUEEN
DUND

164/979 Y35: F1_Supervn X28: NSS_Satisfn
r(all)= 0.325 p= 0.0849 r(NonImp)= 0.325 Npairs=29 NimputedPairs=0

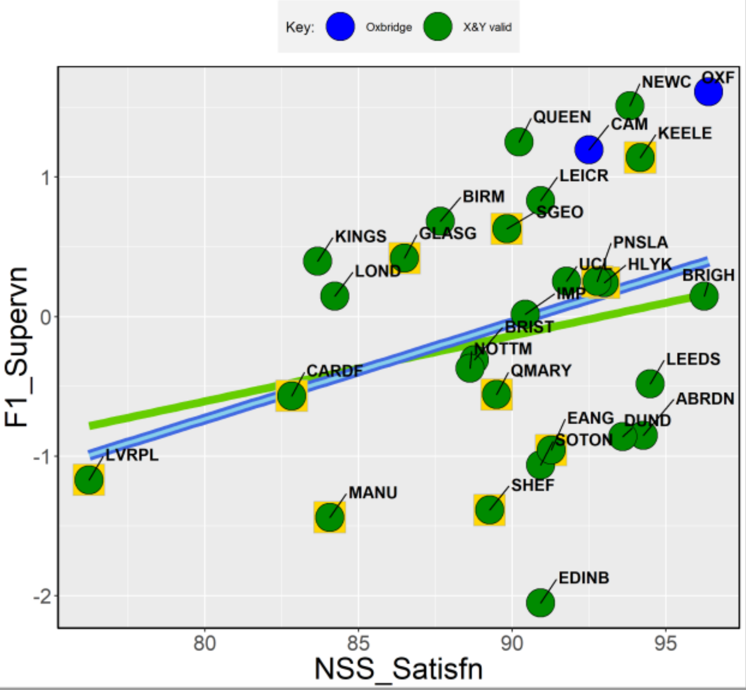

164/980 Y36: Trainee_GP X28: NSS_Satisfn
r(all)= 0.138 p= 0.477 r(NonImp)= 0.138 Npairs=29 NimputedPairs=0

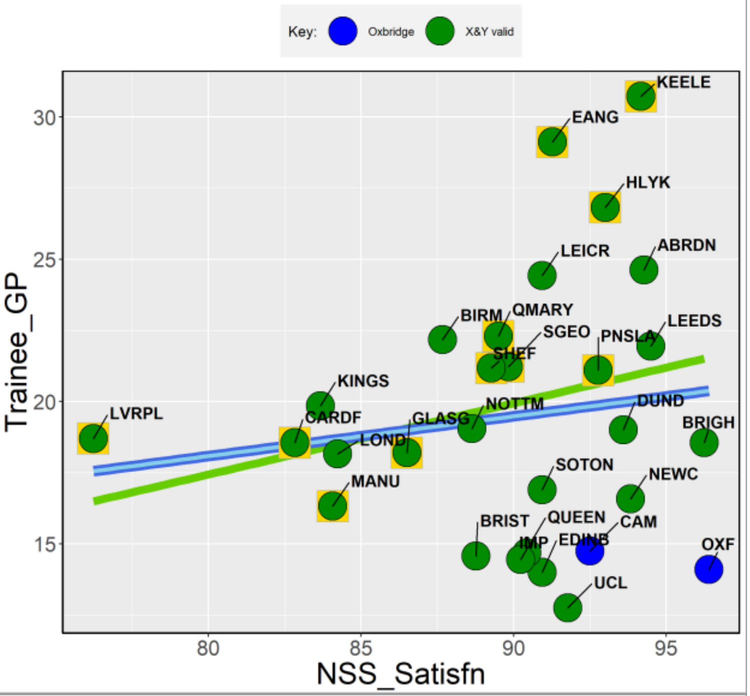

164/981 Y37: Trainee_Psyc X28: NSS_Satisfn
r(all)= 0.244 p= 0.203 r(NonImp)= 0.244 Npairs=29 NimputedPairs=0

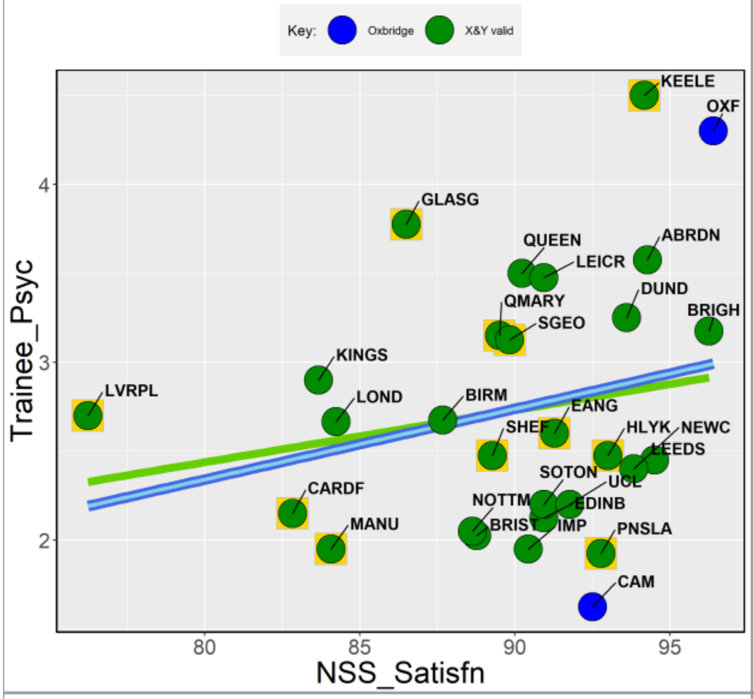

164/982 Y38: TraineeApp_Surgery X28: NSS_Satisf
r(all)= -0.188 p= 0.33 r(NonImp)= -0.187 Npairs=29 NimputedPairs=2

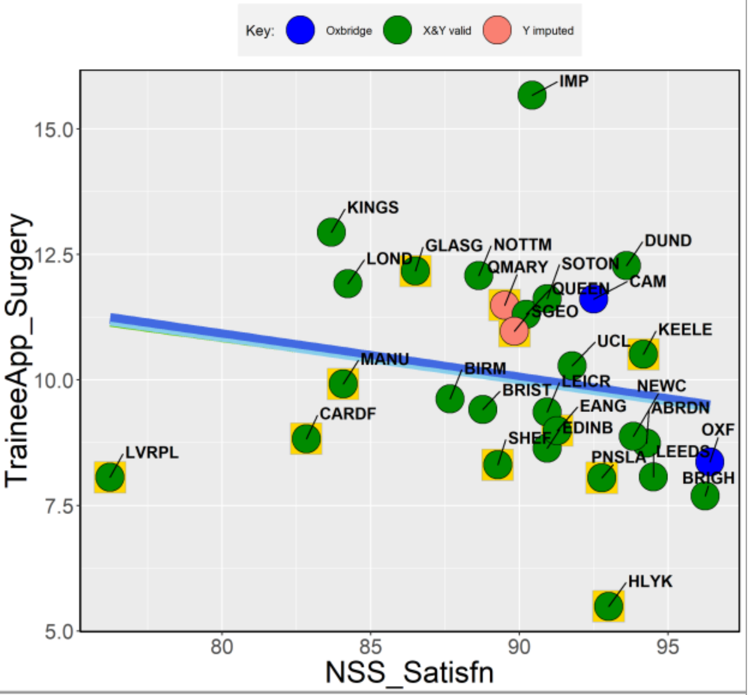

164/983 Y39: TraineeApp_Anaes X28: NSS_Satisfn
r(all)= 0.174 p= 0.366 r(NonImp)= 0.174 Npairs=29 NimputedPairs=0

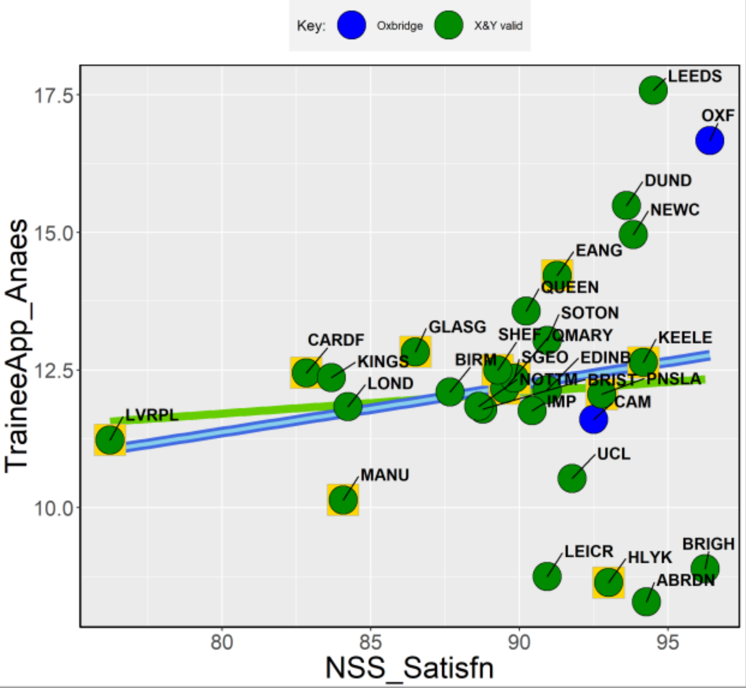

164/984 Y40: GMC_PGexams X28: NSS_Satisfn
r(all)= 0.108 p= 0.578 r(NonImp)= 0.108 Npairs=29 NimputedPairs=0

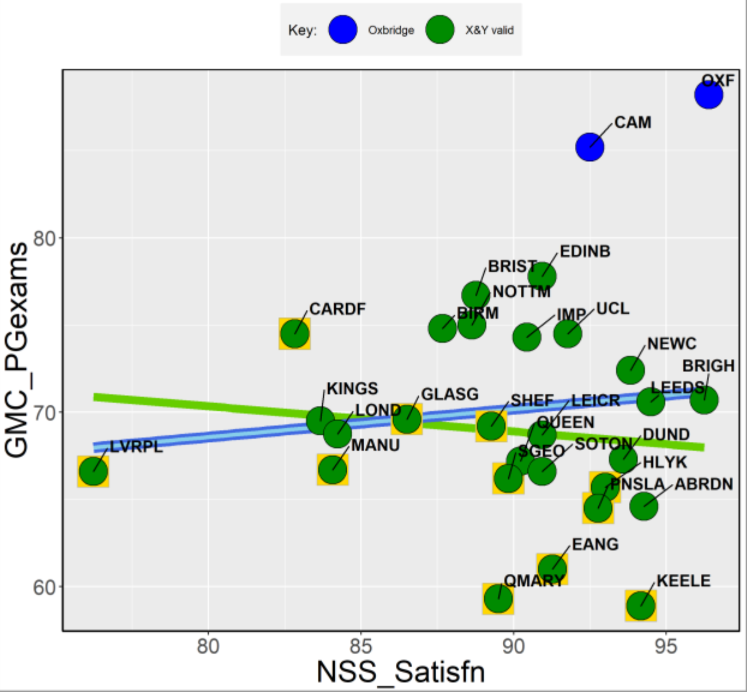

165/985 Y41: MRCGP_AKT X28: NSS_Satisfn
r(all)= 0.187 p= 0.331 r(NonImp)= 0.187 Npairs=29 NimputedPairs=0

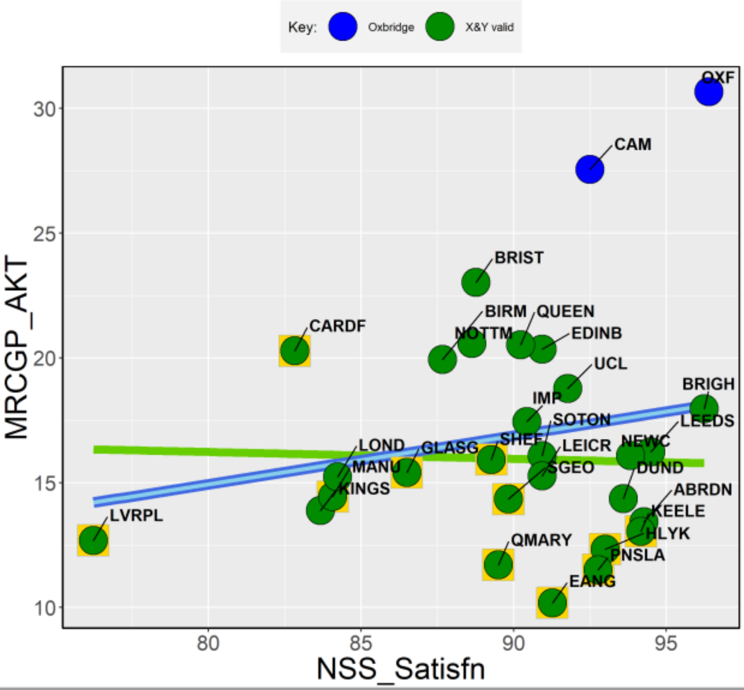

165/986 Y42: MRCGP_CSA X28: NSS_Satisfn
r(all)= 0.332 p= 0.0785 r(NonImp)= 0.332 Npairs=29 NimputedPairs=0

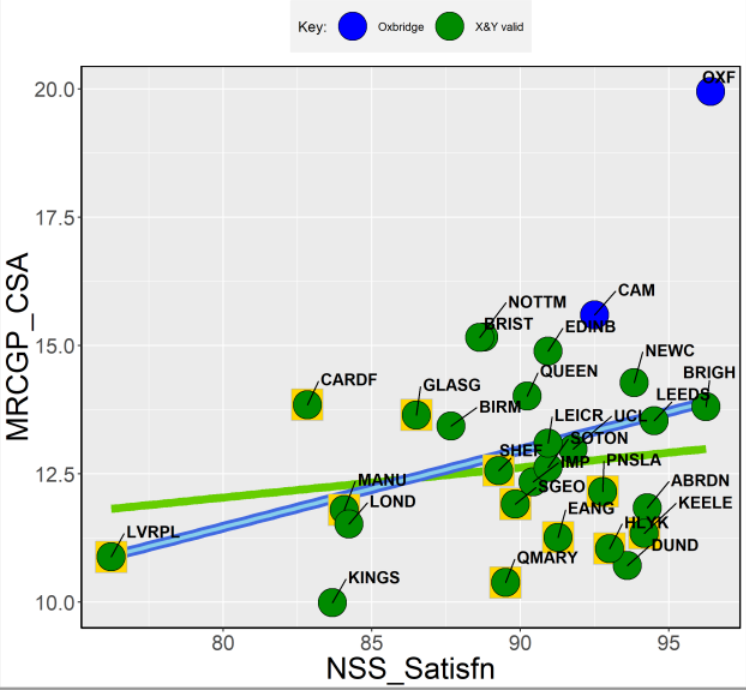

165/987 Y43: FRCA_Pt1 X28: NSS_Satisfn
r(all)= 0.006 p= 0.976 r(NonImp)= 0.019 Npairs=29 NimputedPairs=10

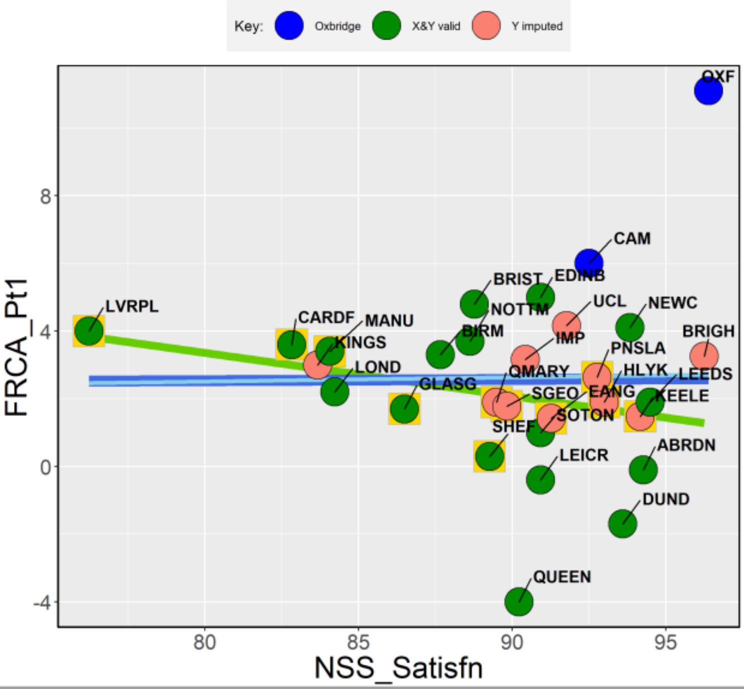

165/988 Y44: MRCOG_Pt1 X28: NSS_Satisfn
r(all)= 0.299 p= 0.116 r(NonImp)= 0.381 Npairs=29 NimputedPairs=10

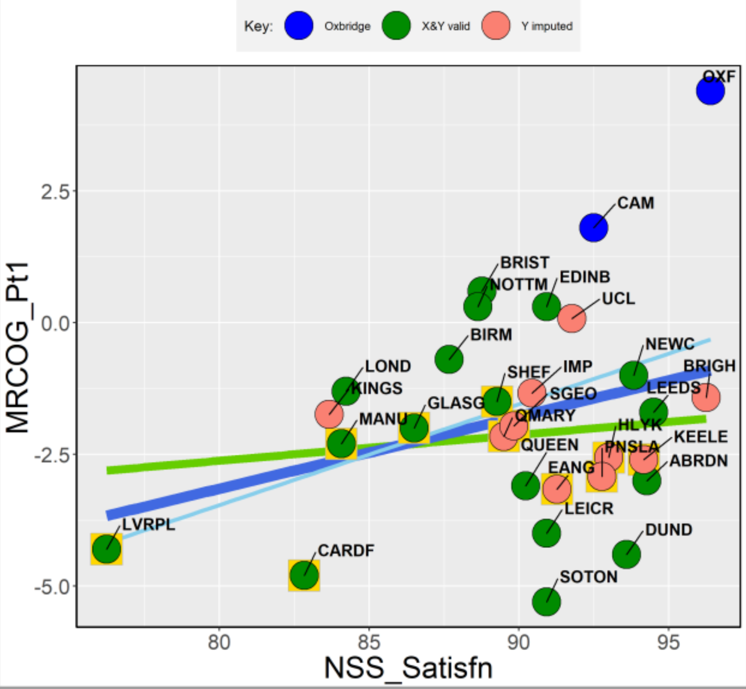

165/989 Y45: MRCOG_Pt2 X28: NSS_Satisfn
r(all)= 0.268 p= 0.161 r(NonImp)= 0.344 Npairs=29 NimputedPairs=10

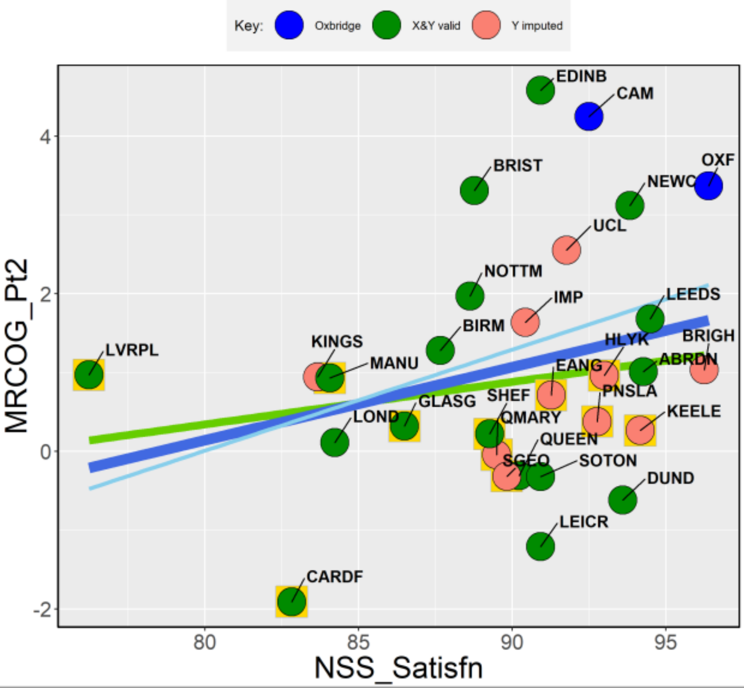

165/990 Y46: MRCP_Pt1 X28: NSS_Satisfn
r(all)= 0.205 p= 0.287 r(NonImp)= 0.227 Npairs=29 NimputedPairs=3

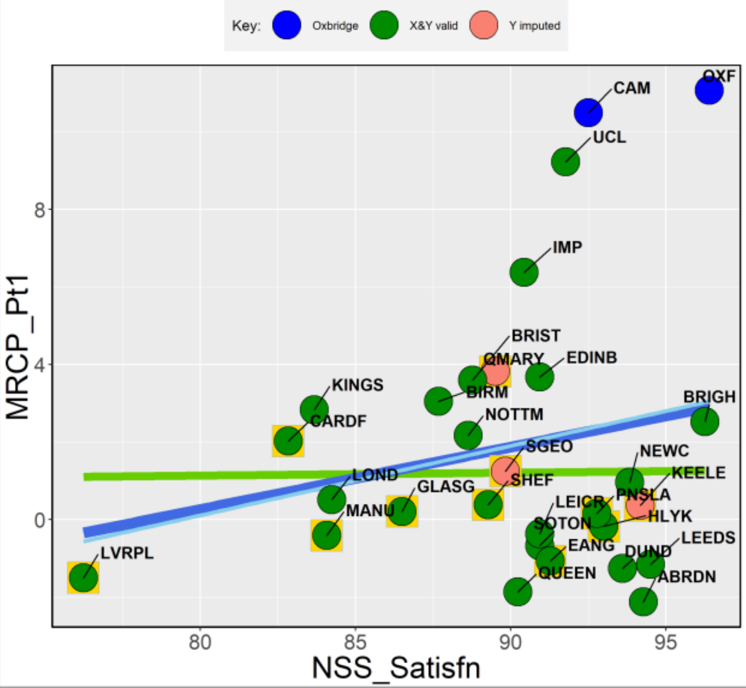

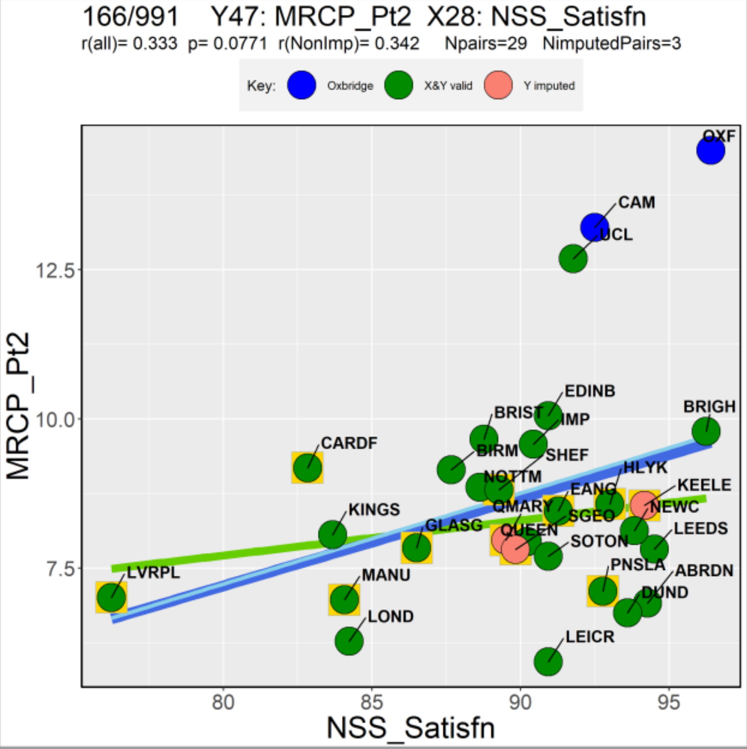
166/991 Y47: MRCP_Pt2 X28: NSS_Satisfn
r(all)= 0.333 p= 0.0771 r(NonImp)= 0.342 Npairs=29 NimputedPairs=3
Key: Oxbridge X&Y valid Y imputed
MRCP_Pt2
NSS_Satisfn
OXF
CAM
UCL
EDINB
BRIST
IMP
BRIGH
CARDF
BIRM
SHEF
HLYK
NOTTM
EANG
KEELE
KINGS
QMARY
NEWC
SGEO
GLASG
QUEEN
LEEDS
SOTON
LVRPL
PNSLA
ABRDN
MANU
DUND
LOND
LEICR

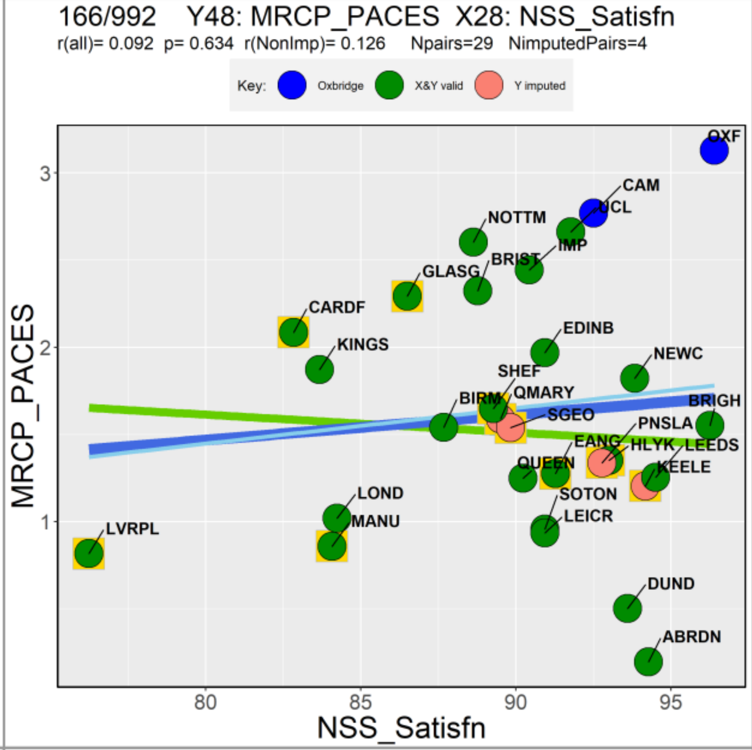
166/992 Y48: MRCP_PACES X28: NSS_Satisfn
r(all)= 0.092 p= 0.634 r(NonImp)= 0.126 Npairs=29 NimputedPairs=4
Key: Oxbridge X&Y valid Y imputed
MRCP_PACES
NSS_Satisfn
OXF
CAM
UCL
NOTTM
BRIST
IMP
GLASG
CARDF
EDINB
KINGS
NEWC
SHEF
BIRM
QMARY
BRIGH
SGEO
PNSLA
EANG
HLYK
LEEDS
QUEEN
KEELE
LOND
SOTON
MANU
LEICR
LVRPL
DUND
ABRDN

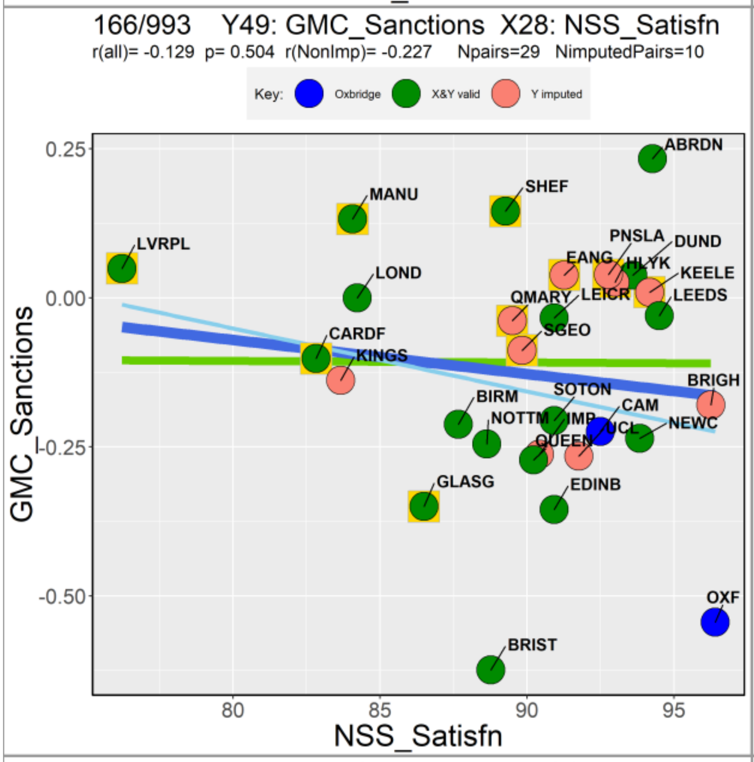
166/993 Y49: GMC_Sanctions X28: NSS_Satisfn
r(all)= -0.129 p= 0.504 r(NonImp)= -0.227 Npairs=29 NimputedPairs=10
Key: Oxbridge X&Y valid Y imputed
GMC_Sanctions
NSS_Satisfn
ABRDN
SHEF
MANU
LVRPL
PNSLA
DUND
EANG
HLYK
KEELE
LOND
QMARY
LEICR
LEEDS
SGEO
CARDF
KINGS
BRIGH
SOTON
BIRM
CAM
NOTTM
IMP
UCL
NEWC
QUEEN
GLASG
EDINB
OXF
BRIST

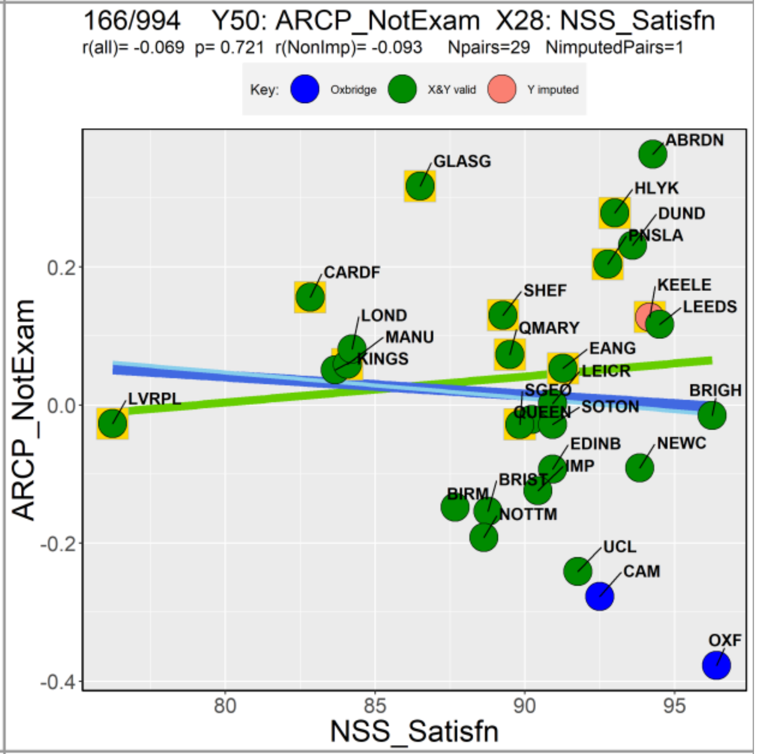
166/994 Y50: ARCP_NotExam X28: NSS_Satisfn
r(all)= -0.069 p= 0.721 r(NonImp)= -0.093 Npairs=29 NimputedPairs=1
Key: Oxbridge X&Y valid Y imputed
ARCP_NotExam
NSS_Satisfn
ABRDN
GLASG
HLYK
DUND
PNSLA
CARDF
KEELE
SHEF
LEEDS
LOND
QMARY
MANU
EANG
KINGS
LEICR
LVRPL
SGEO
BRIGH
SOTON
QUEEN
EDINB
NEWC
IMP
BRIST
BIRM
NOTTM
UCL
CAM
OXF

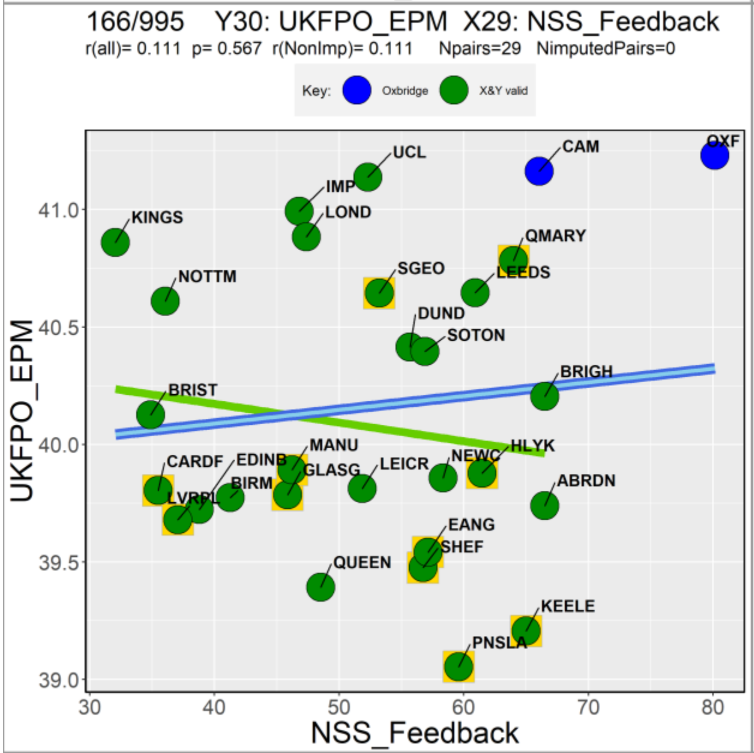
166/995 Y30: UKFPO_EPM X29: NSS_Feedback
r(all)= 0.111 p= 0.567 r(NonImp)= 0.111 Npairs=29 NimputedPairs=0
Key: Oxbridge X&Y valid
UKFPO_EPM
NSS_Feedback
OXF
CAM
UCL
IMP
KINGS
LOND
QMARY
NOTTM
SGEO
LEEDS
DUND
SOTON
BRIGH
BRIST
MANU
HLYK
CARDF
EDINB
GLASG
LEICR
NEWC
ABRDN
LVRPL
BIRM
EANG
SHEF
QUEEN
KEELE
PNSLA

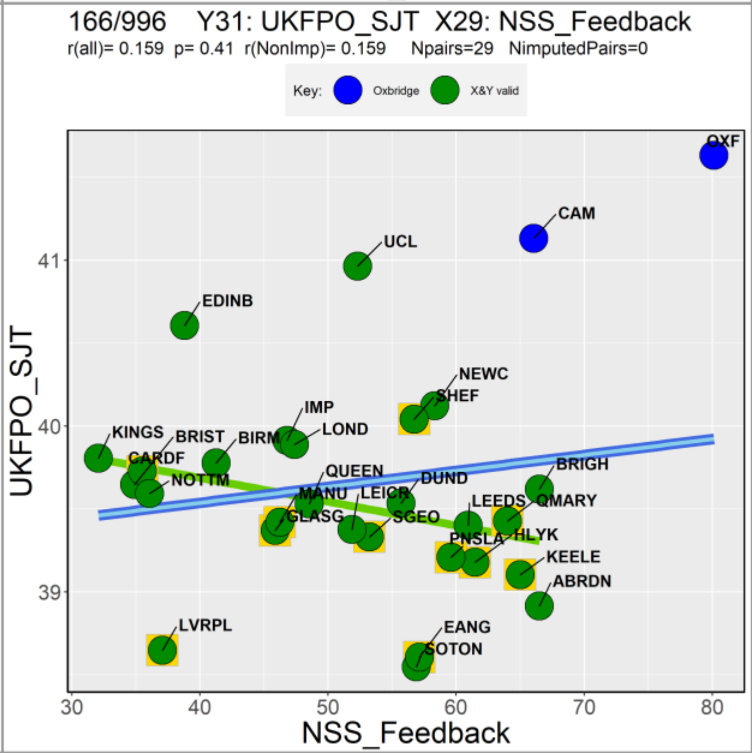
166/996 Y31: UKFPO_SJT X29: NSS_Feedback
r(all)= 0.159 p= 0.41 r(NonImp)= 0.159 Npairs=29 NimputedPairs=0
Key: Oxbridge X&Y valid
UKFPO_SJT
NSS_Feedback
OXF
CAM
UCL
EDINB
NEWC
SHEF
IMP
LOND
KINGS
BRIST
BIRM
CARDF
NOTTM
QUEEN
DUND
BRIGH
MANU
LEICR
LEEDS
QMARY
GLASG
SGEO
PNSLA
HLYK
KEELE
ABRDN
LVRPL
EANG
SOTON

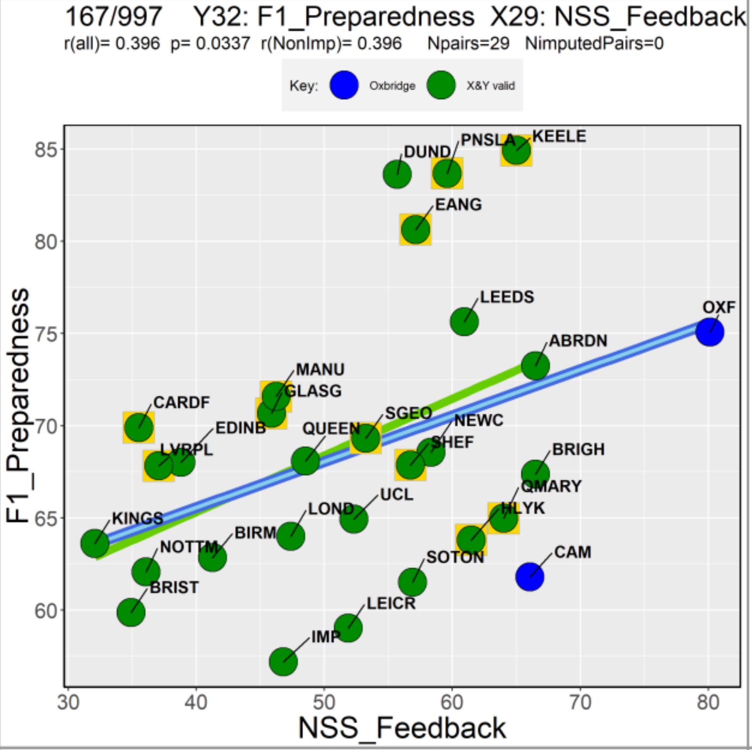
167/997 Y32: F1_Preparedness X29: NSS_Feedback
r(all)= 0.396 p= 0.0337 r(NonImp)= 0.396 Npairs=29 NimputedPairs=0
Key: Oxbridge X&Y valid
F1_Preparedness
NSS_Feedback
DUND PNSLA KEELE EANG LEEDS OXF ABRDN MANU GLASG CARDF SGEO EDINB QUEEN NEWC LVRPL SHEF BRIGH QMARY UCL KINGS LOND HLYK NOTTM BIRM SOTON CAM BRIST LEICR IMP

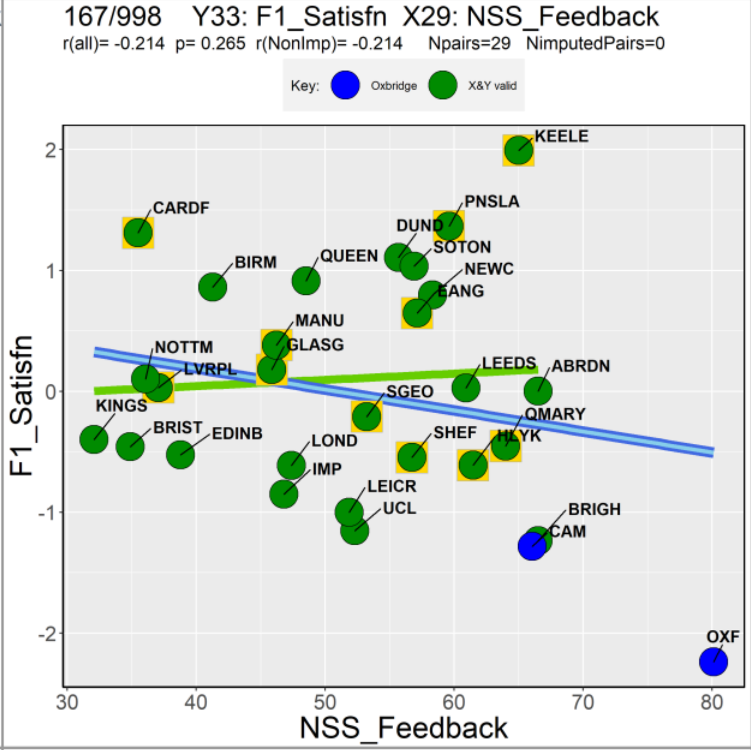
167/998 Y33: F1_Satisfn X29: NSS_Feedback
r(all)= -0.214 p= 0.265 r(NonImp)= -0.214 Npairs=29 NimputedPairs=0
Key: Oxbridge X&Y valid
F1_Satisfn
NSS_Feedback
KEELE CARDF PNSLA DUND SOTON BIRM QUEEN NEWC EANG MANU NOTTM GLASG LVRPL LEEDS ABRDN KINGS SGEO QMARY BRIST EDINB SHEF HLYK LOND IMP LEICR UCL BRIGH CAM OXF

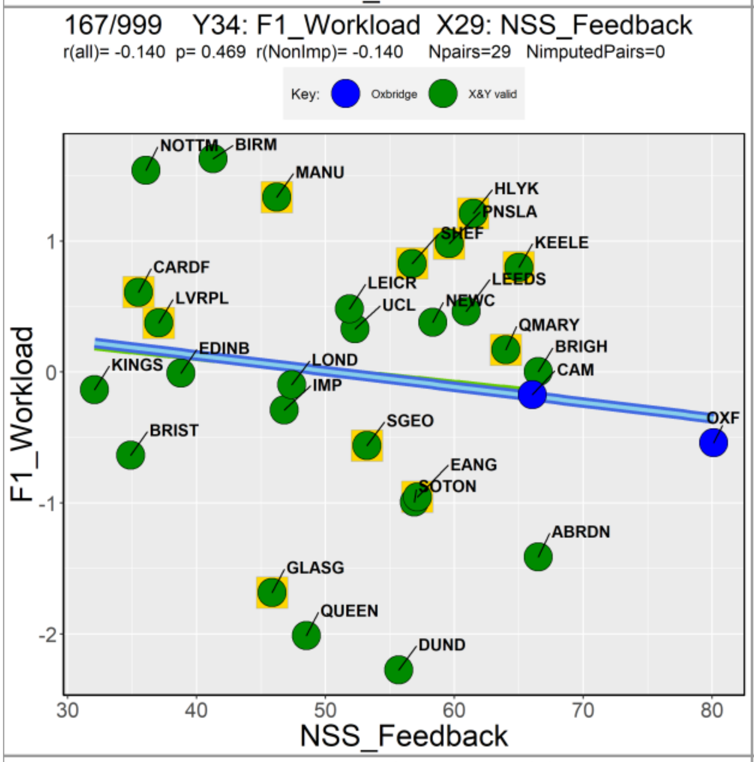
167/999 Y34: F1_Workload X29: NSS_Feedback
r(all)= -0.140 p= 0.469 r(NonImp)= -0.140 Npairs=29 NimputedPairs=0
Key: Oxbridge X&Y valid
F1_Workload
NSS_Feedback
NOTTM BIRM MANU HLYK PNSLA SHEF KEELE CARDF LEICR LEEDS LVRPL UCL NEWC QMARY EDINB BRIGH LOND CAM KINGS IMP OXF BRIST SGEO EANG SOTON ABRDN GLASG QUEEN DUND

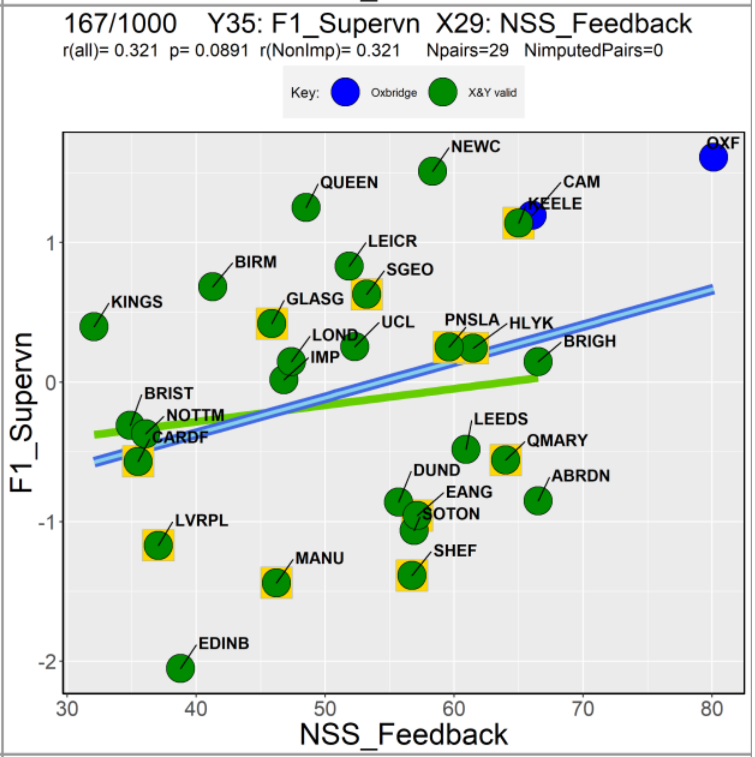
167/1000 Y35: F1_Supervn X29: NSS_Feedback
r(all)= 0.321 p= 0.0891 r(NonImp)= 0.321 Npairs=29 NimputedPairs=0
Key: Oxbridge X&Y valid
F1_Supervn
NSS_Feedback
OXF NEWC QUEEN CAM KEELE LEICR BIRM SGEO KINGS GLASG UCL PNSLA HLYK LOND IMP BRIGH BRIST NOTTM CARDF LEEDS QMARY DUND EANG ABRDN SOTON LVRPL SHEF MANU EDINB

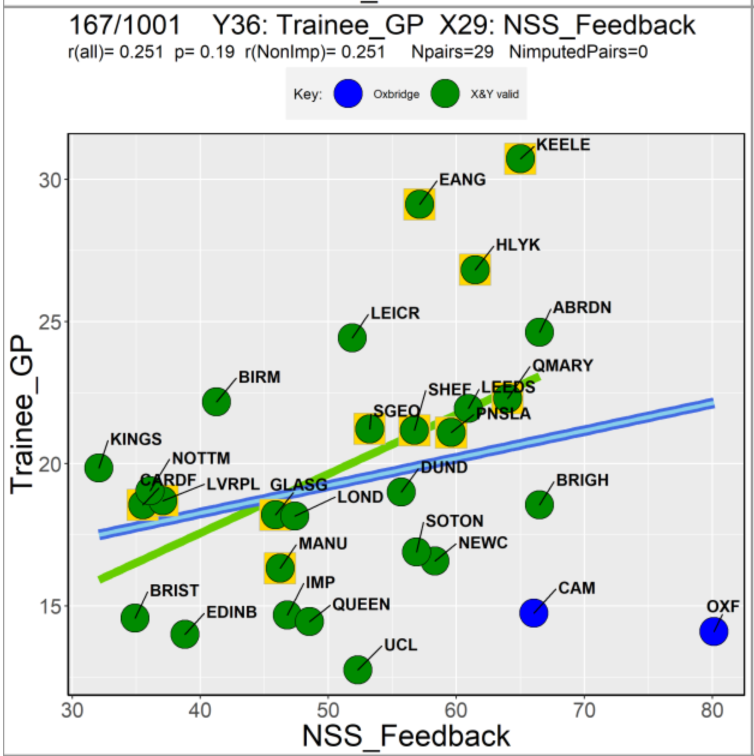
167/1001 Y36: Trainee_GP X29: NSS_Feedback
r(all)= 0.251 p= 0.19 r(NonImp)= 0.251 Npairs=29 NimputedPairs=0
Key: Oxbridge X&Y valid
Trainee_GP
NSS_Feedback
KEELE EANG HLYK LEICR ABRDN BIRM QMARY SHEF LEEDS SGEO PNSLA KINGS NOTTM DUND CARDF LVRPL GLASG LOND BRIGH SOTON MANU NEWC IMP CAM BRIST EDINB QUEEN OXF UCL

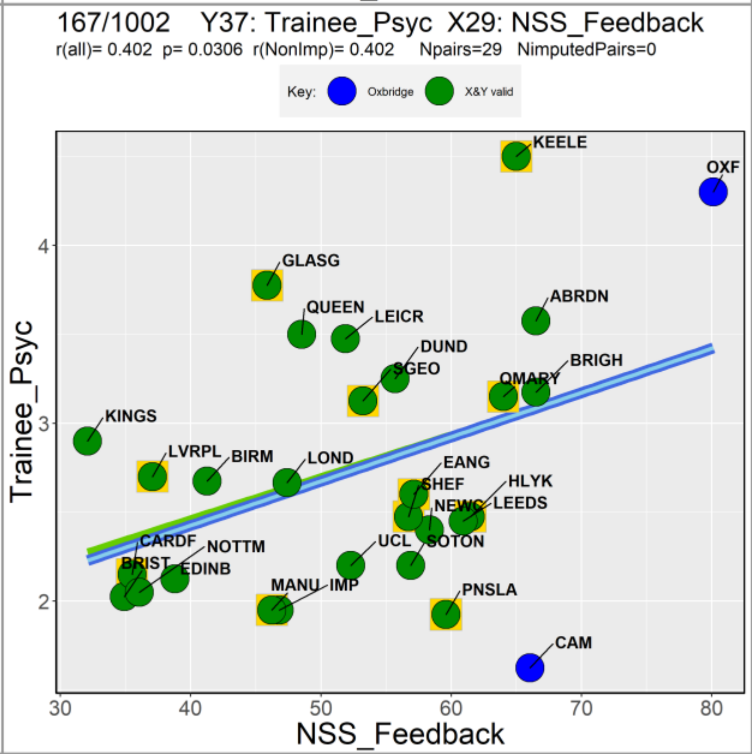
167/1002 Y37: Trainee_Psyc X29: NSS_Feedback
r(all)= 0.402 p= 0.0306 r(NonImp)= 0.402 Npairs=29 NimputedPairs=0
Key: Oxbridge X&Y valid
Trainee_Psyc
NSS_Feedback
KEELE OXF GLASG ABRDN QUEEN LEICR DUND SGEO BRIGH QMARY KINGS LVRPL BIRM LOND EANG SHEF HLYK NEWC LEEDS CARDF NOTTM BRIST EDINB UCL SOTON MANU IMP PNSLA CAM

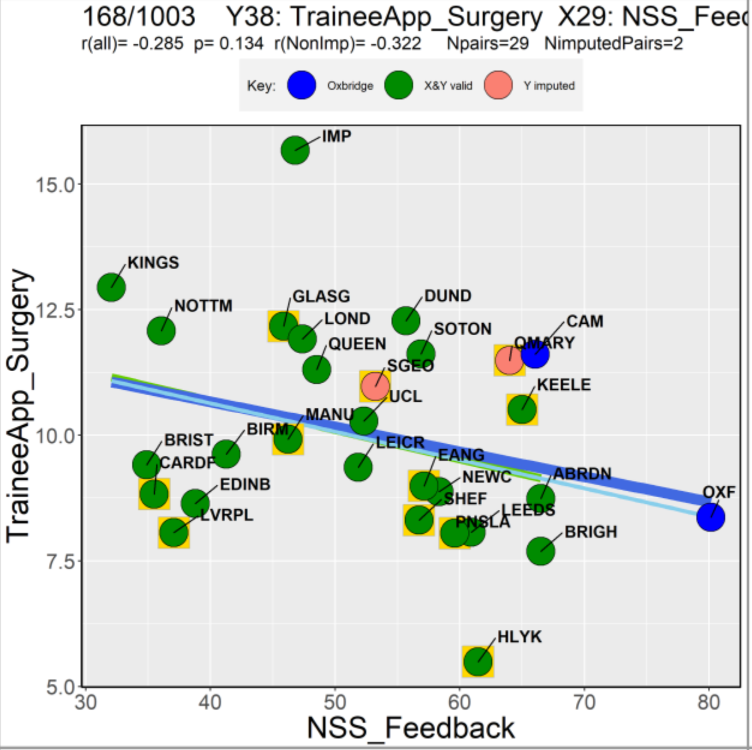
168/1003 Y38: TraineeApp_Surgery X29: NSS_Feed
r(all)= -0.285 p= 0.134 r(NonImp)= -0.322 Npairs=29 NimputedPairs=2
Key: Oxbridge X&Y valid Y imputed
TraineeApp_Surgery
NSS_Feedback

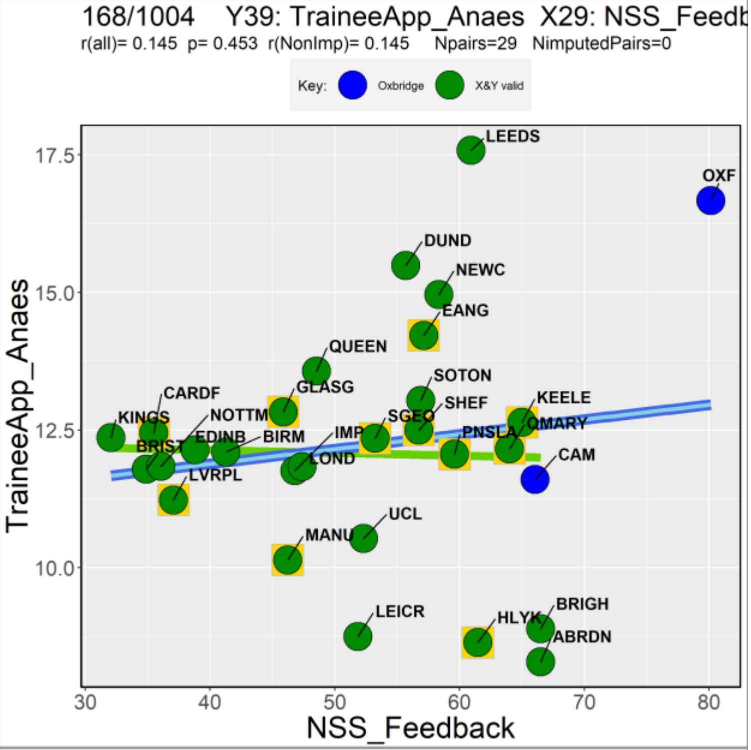
168/1004 Y39: TraineeApp_Anaes X29: NSS_Feedb
r(all)= 0.145 p= 0.453 r(NonImp)= 0.145 Npairs=29 NimputedPairs=0
Key: Oxbridge X&Y valid
TraineeApp_Anaes
NSS_Feedback

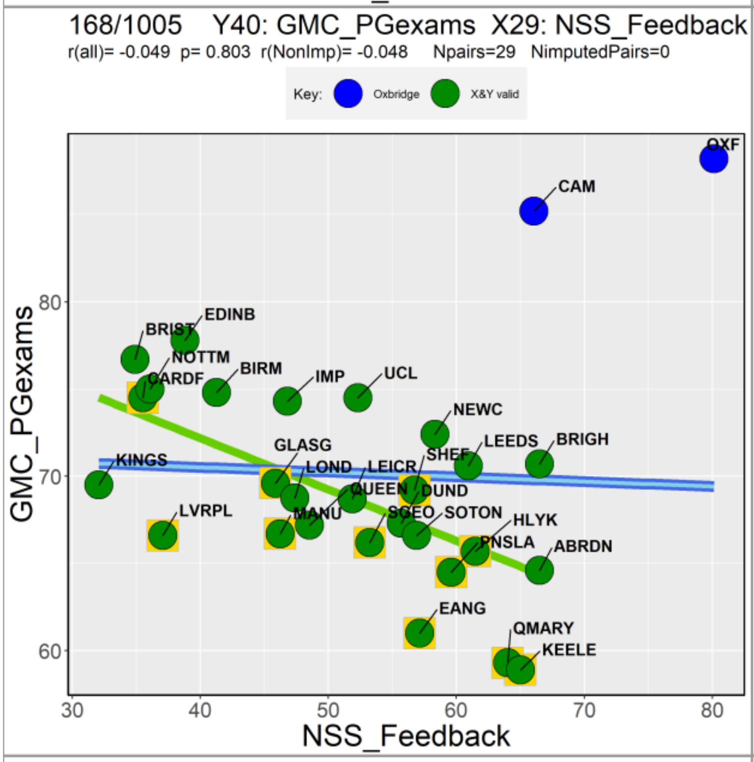
168/1005 Y40: GMC_PGexams X29: NSS_Feedback
r(all)= -0.049 p= 0.803 r(NonImp)= -0.048 Npairs=29 NimputedPairs=0
Key: Oxbridge X&Y valid
GMC_PGexams
NSS_Feedback

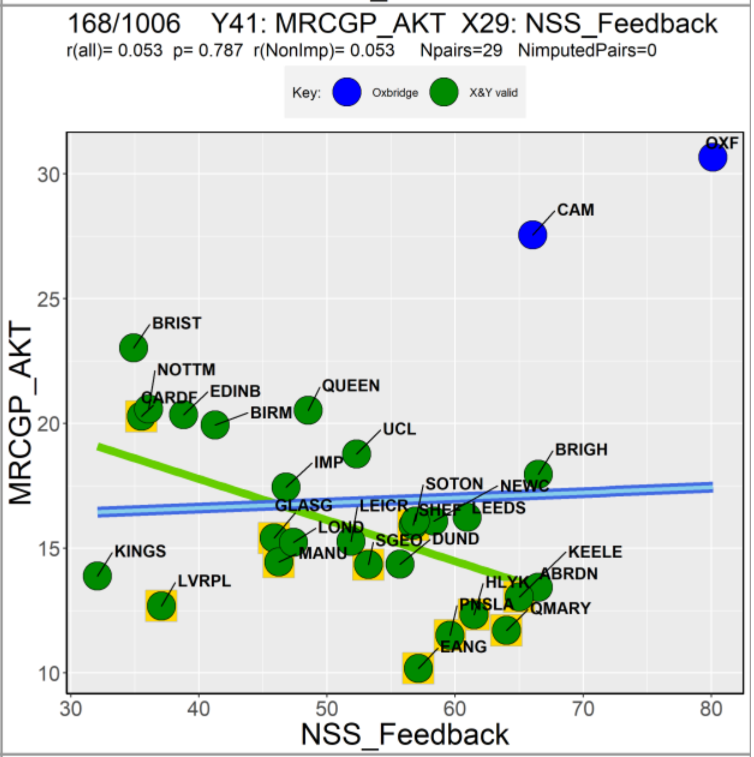
168/1006 Y41: MRCGP_AKT X29: NSS_Feedback
r(all)= 0.053 p= 0.787 r(NonImp)= 0.053 Npairs=29 NimputedPairs=0
Key: Oxbridge X&Y valid
MRCGP_AKT
NSS_Feedback

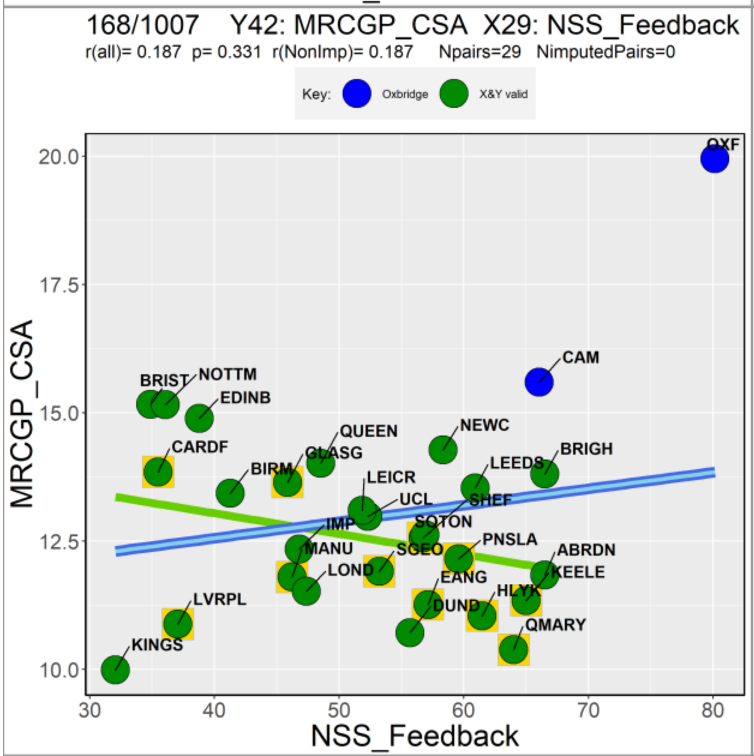
168/1007 Y42: MRCGP_CSA X29: NSS_Feedback
r(all)= 0.187 p= 0.331 r(NonImp)= 0.187 Npairs=29 NimputedPairs=0
Key: Oxbridge X&Y valid
MRCGP_CSA
NSS_Feedback

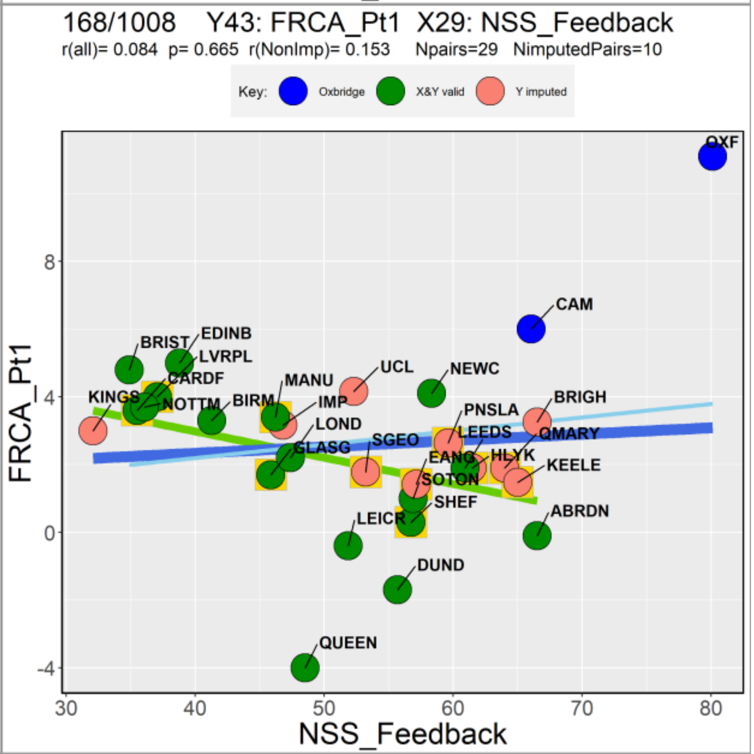
168/1008 Y43: FRCA_Pt1 X29: NSS_Feedback
r(all)= 0.084 p= 0.665 r(NonImp)= 0.153 Npairs=29 NimputedPairs=10
Key: Oxbridge X&Y valid Y imputed
FRCA_Pt1
NSS_Feedback

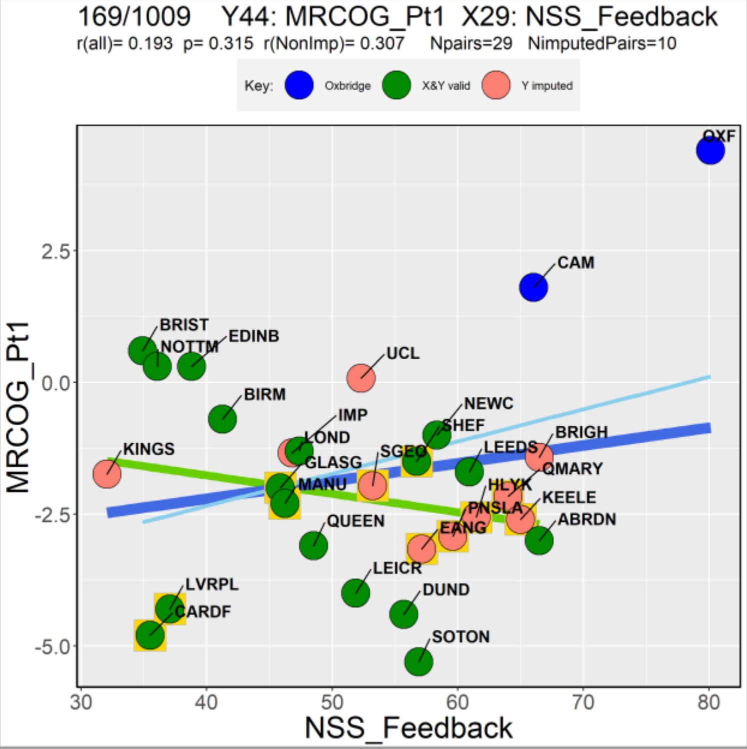
169/1009 Y44: MRCOG_Pt1 X29: NSS_Feedback
r(all)= 0.193 p= 0.315 r(NonImp)= 0.307 Npairs=29 NimputedPairs=10
Key: Oxbridge X&Y valid Y imputed
MRCOG_Pt1
NSS_Feedback

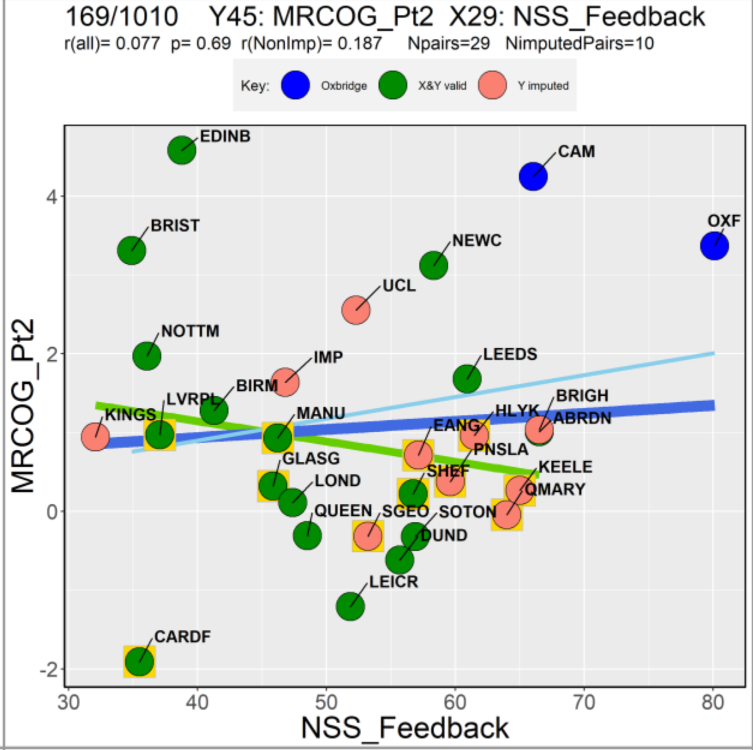
169/1010 Y45: MRCOG_Pt2 X29: NSS_Feedback
r(all)= 0.077 p= 0.69 r(NonImp)= 0.187 Npairs=29 NimputedPairs=10
Key: Oxbridge X&Y valid Y imputed
MRCOG_Pt2
NSS_Feedback

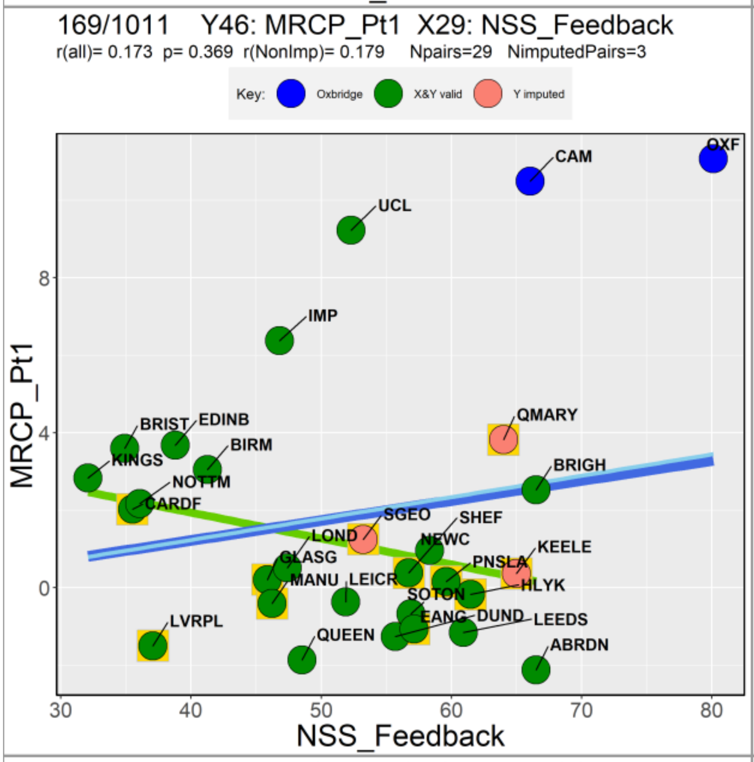
169/1011 Y46: MRCP_Pt1 X29: NSS_Feedback
r(all)= 0.173 p= 0.369 r(NonImp)= 0.179 Npairs=29 NimputedPairs=3
Key: Oxbridge X&Y valid Y imputed
MRCP_Pt1
NSS_Feedback

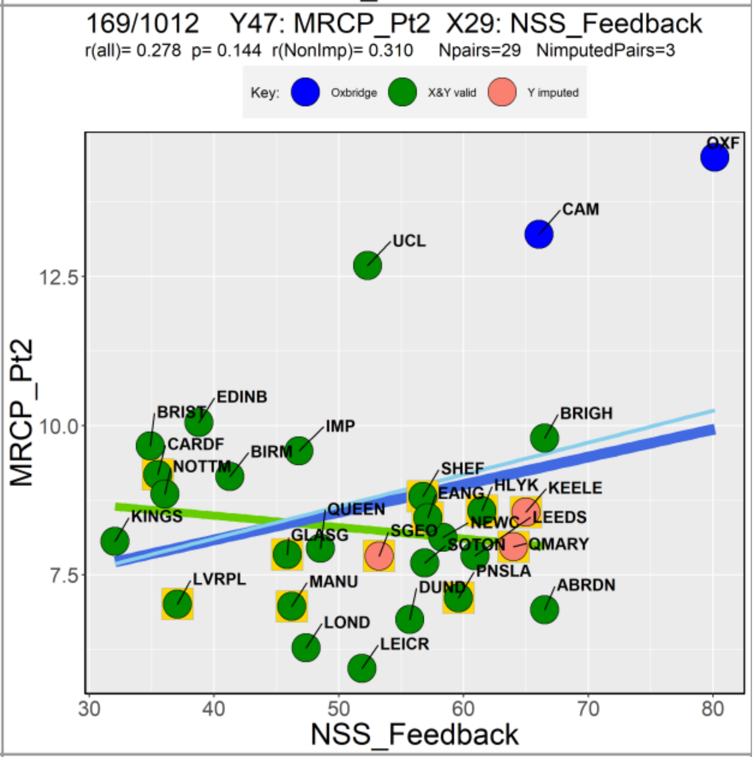
169/1012 Y47: MRCP_Pt2 X29: NSS_Feedback
r(all)= 0.278 p= 0.144 r(NonImp)= 0.310 Npairs=29 NimputedPairs=3
Key: Oxbridge X&Y valid Y imputed
MRCP_Pt2
NSS_Feedback

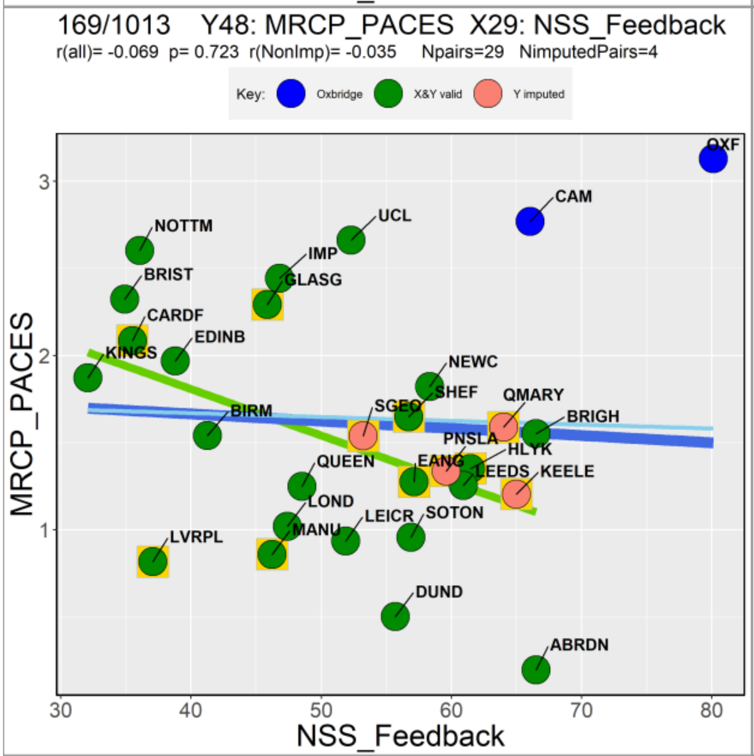
169/1013 Y48: MRCP_PACES X29: NSS_Feedback
r(all)= -0.069 p= 0.723 r(NonImp)= -0.035 Npairs=29 NimputedPairs=4
Key: Oxbridge X&Y valid Y imputed
MRCP_PACES
NSS_Feedback

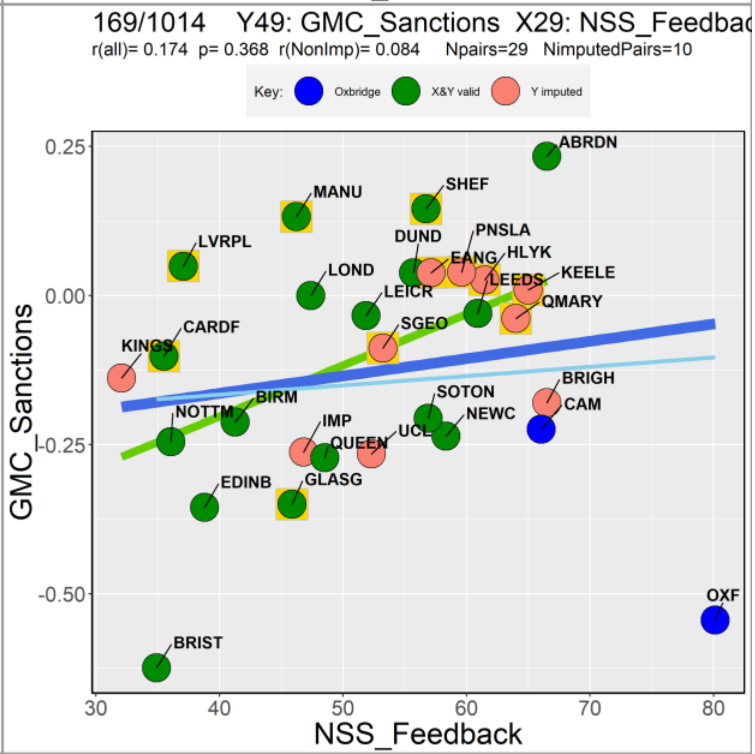
169/1014 Y49: GMC_Sanctions X29: NSS_Feedbac
r(all)= 0.174 p= 0.368 r(NonImp)= 0.084 Npairs=29 NimputedPairs=10
Key: Oxbridge X&Y valid Y imputed
GMC_Sanctions
NSS_Feedback

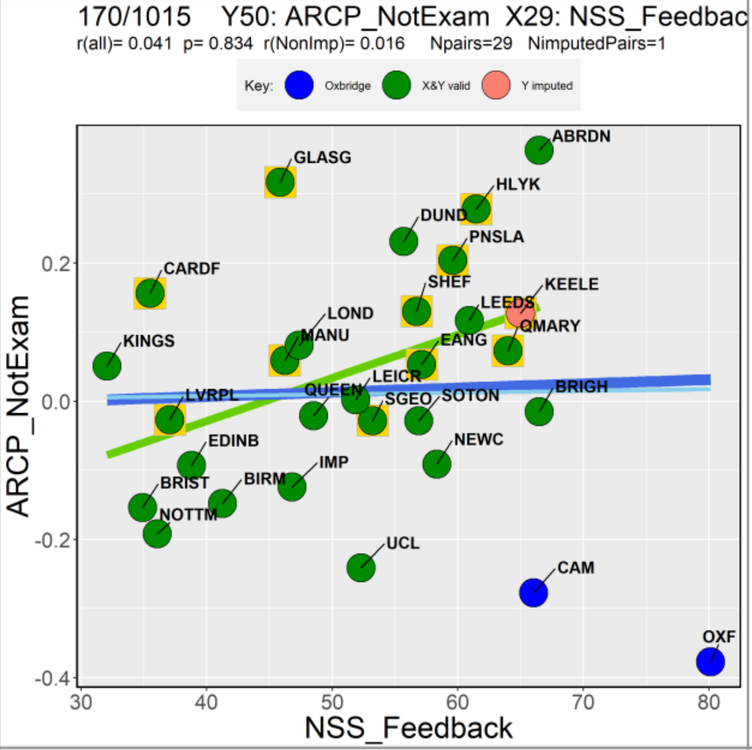
170/1015 Y50: ARCP_NotExam X29: NSS_Feedbac
r(all)= 0.041 p= 0.834 r(NonImp)= 0.016 Npairs=29 NimputedPairs=1
Key: Oxbridge X&Y valid Y imputed
ARCP_NotExam
NSS_Feedback

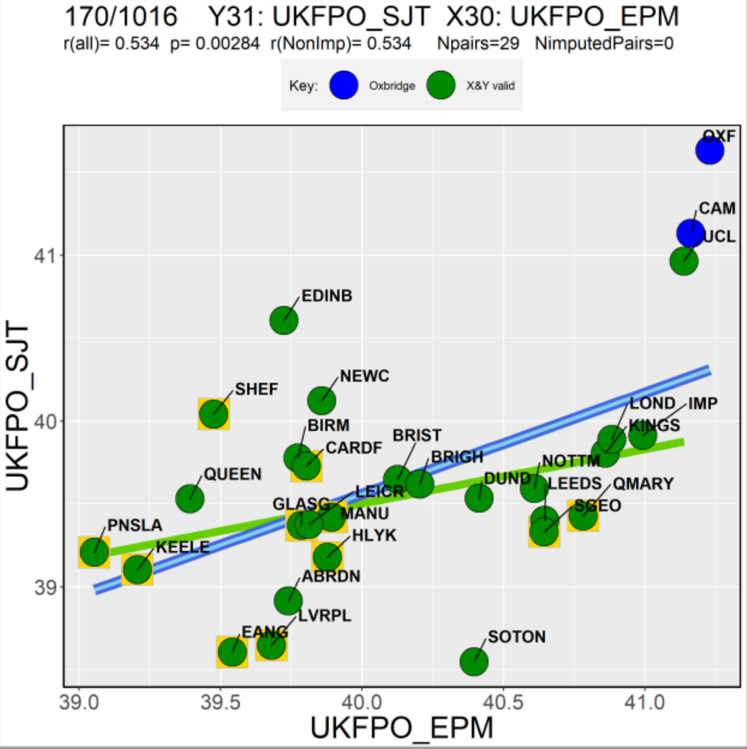
170/1016 Y31: UKFPO_SJT X30: UKFPO_EPM
r(all)= 0.534 p= 0.00284 r(NonImp)= 0.534 Npairs=29 NimputedPairs=0
Key: Oxbridge X&Y valid
UKFPO_SJT
UKFPO_EPM

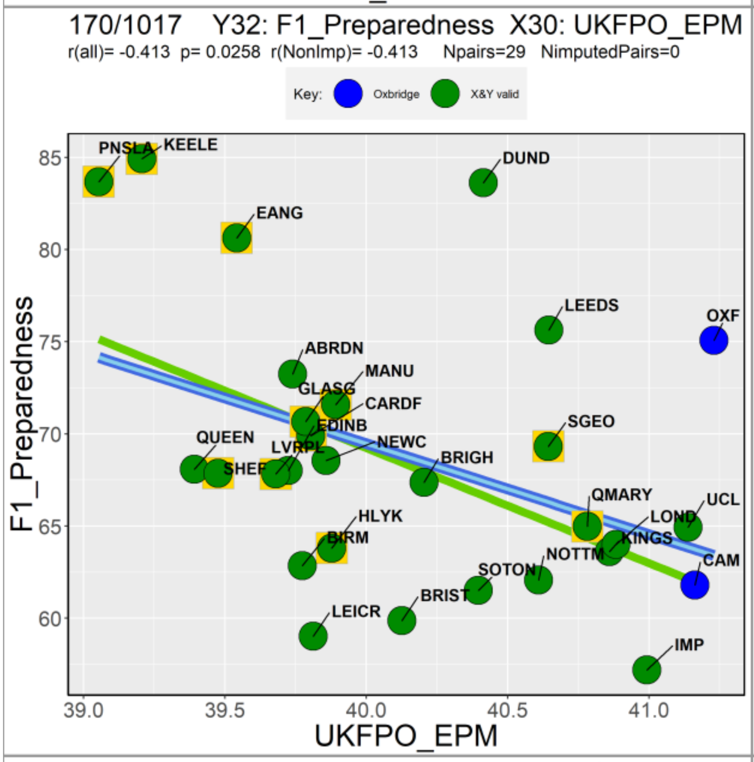
170/1017 Y32: F1_Preparedness X30: UKFPO_EPM
r(all)= -0.413 p= 0.0258 r(NonImp)= -0.413 Npairs=29 NimputedPairs=0
Key: Oxbridge X&Y valid
F1_Preparedness
UKFPO_EPM

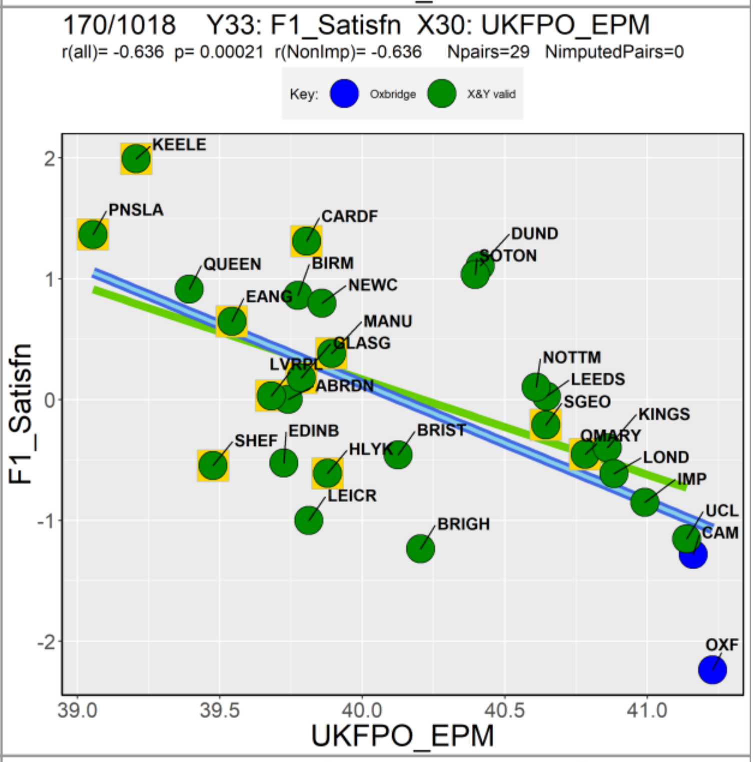
170/1018 Y33: F1_Satisfn X30: UKFPO_EPM
r(all)= -0.636 p= 0.00021 r(NonImp)= -0.636 Npairs=29 NimputedPairs=0
Key: Oxbridge X&Y valid
F1_Satisfn
UKFPO_EPM

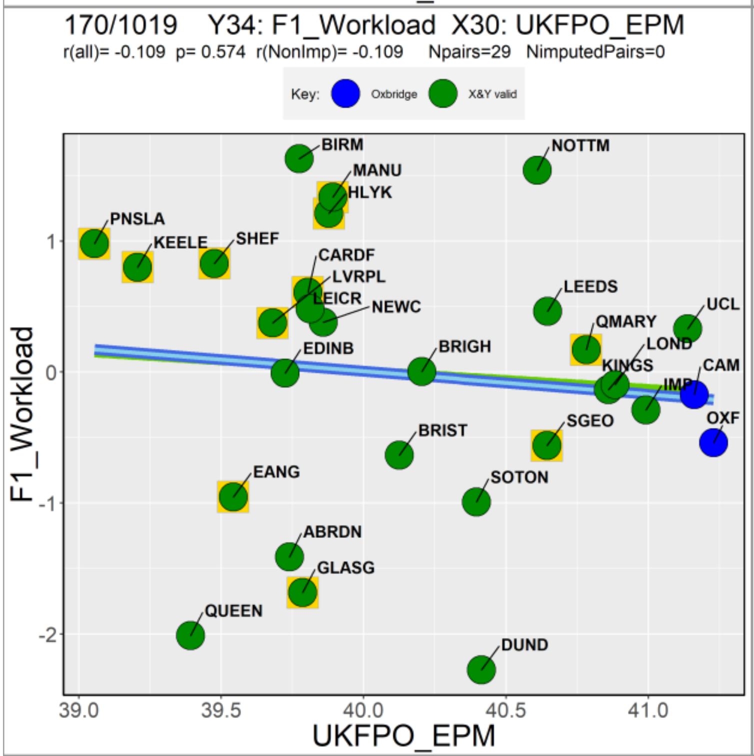
170/1019 Y34: F1_Workload X30: UKFPO_EPM
r(all)= -0.109 p= 0.574 r(NonImp)= -0.109 Npairs=29 NimputedPairs=0
Key: Oxbridge X&Y valid
F1_Workload
UKFPO_EPM

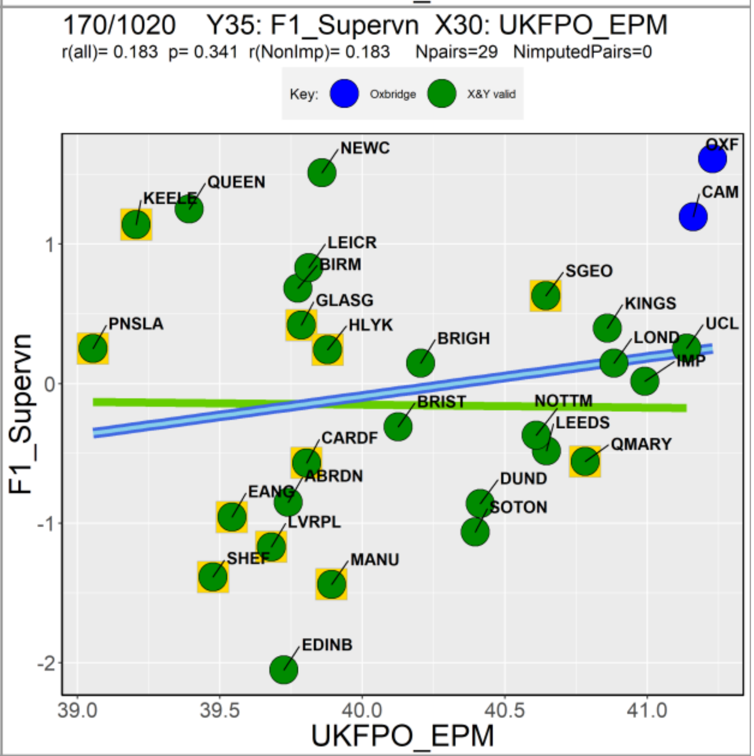
170/1020 Y35: F1_Supervn X30: UKFPO_EPM
r(all)= 0.183 p= 0.341 r(NonImp)= 0.183 Npairs=29 NimputedPairs=0
Key: Oxbridge X&Y valid
F1_Supervn
UKFPO_EPM

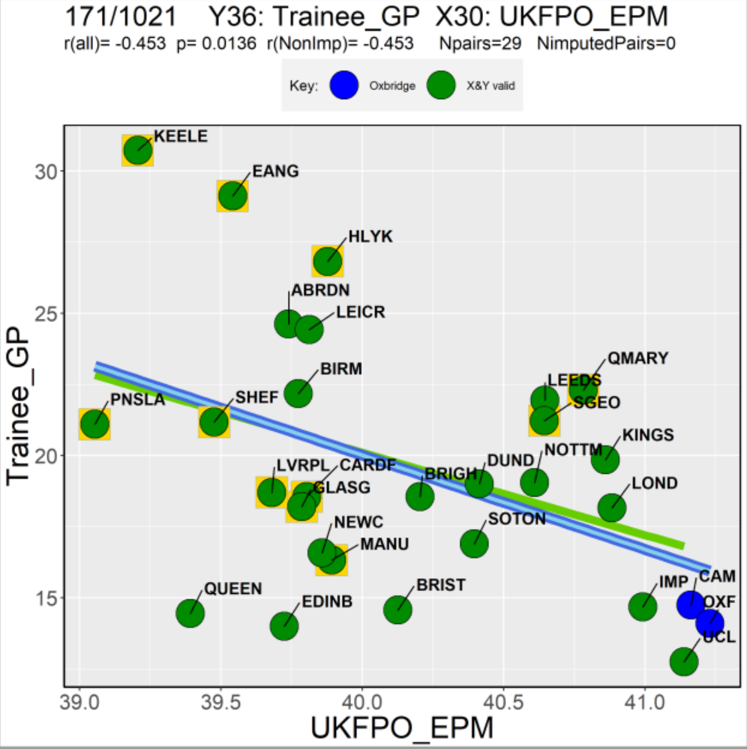
171/1021 Y36: Trainee_GP X30: UKFPO_EPM
r(all)= -0.453 p= 0.0136 r(NonImp)= -0.453 Npairs=29 NimputedPairs=0
Key: Oxbridge X&Y valid
Trainee_GP
UKFPO_EPM

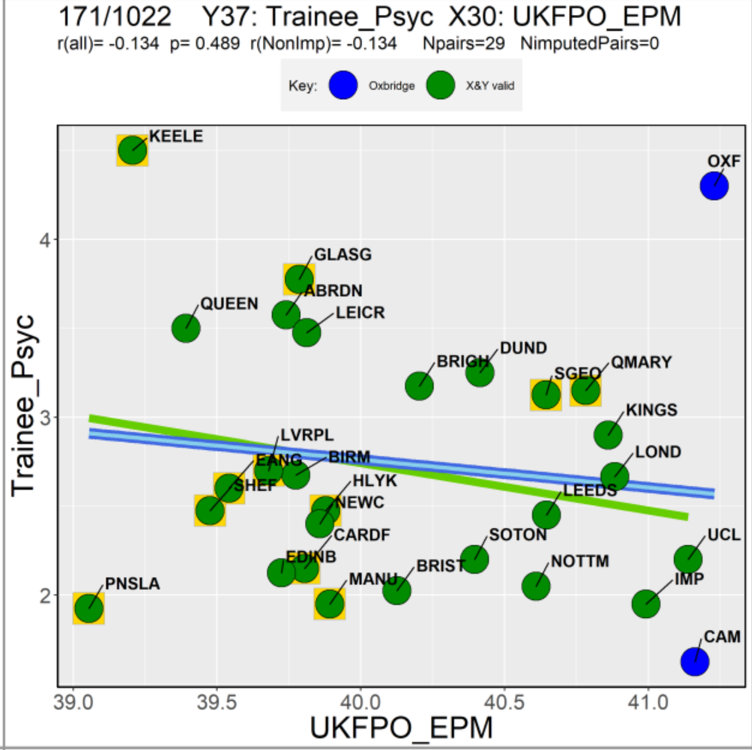
171/1022 Y37: Trainee_Psyc X30: UKFPO_EPM
r(all)= -0.134 p= 0.489 r(NonImp)= -0.134 Npairs=29 NimputedPairs=0
Key: Oxbridge X&Y valid
Trainee_Psyc
UKFPO_EPM

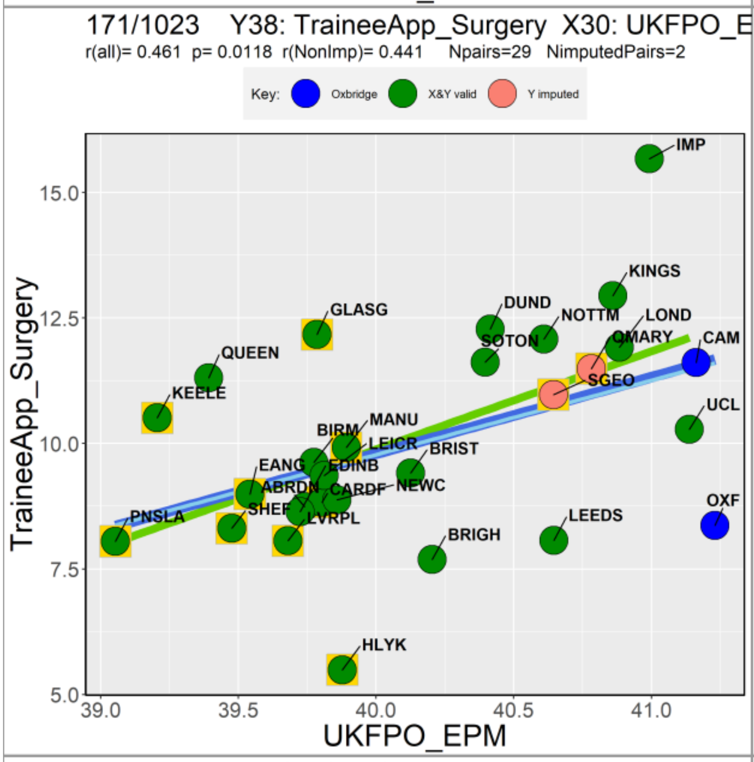
171/1023 Y38: TraineeApp_Surgery X30: UKFPO_E
r(all)= 0.461 p= 0.0118 r(NonImp)= 0.441 Npairs=29 NimputedPairs=2
Key: Oxbridge X&Y valid Y imputed
TraineeApp_Surgery
UKFPO_EPM

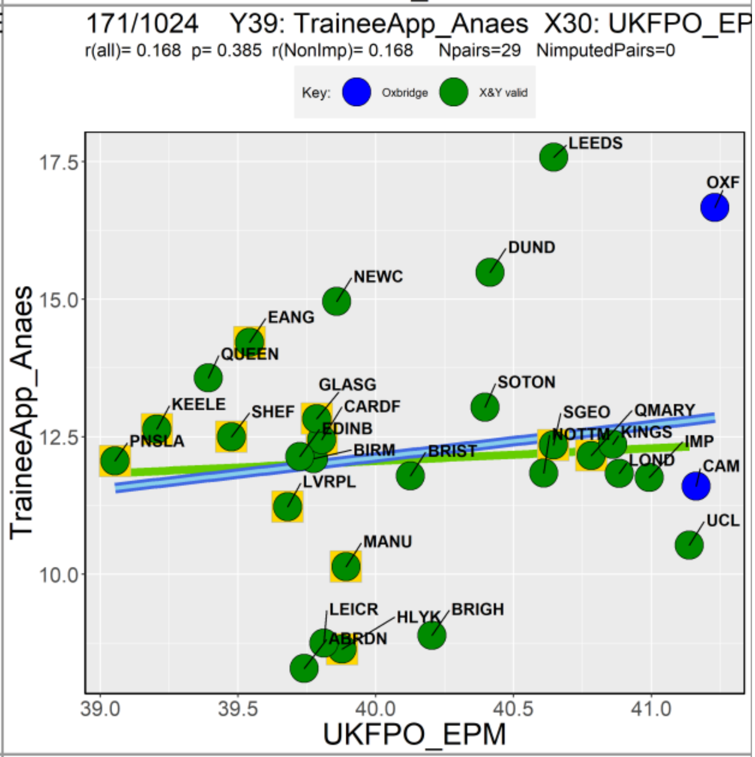
171/1024 Y39: TraineeApp_Anaes X30: UKFPO_EP
r(all)= 0.168 p= 0.385 r(NonImp)= 0.168 Npairs=29 NimputedPairs=0
Key: Oxbridge X&Y valid
TraineeApp_Anaes
UKFPO_EPM

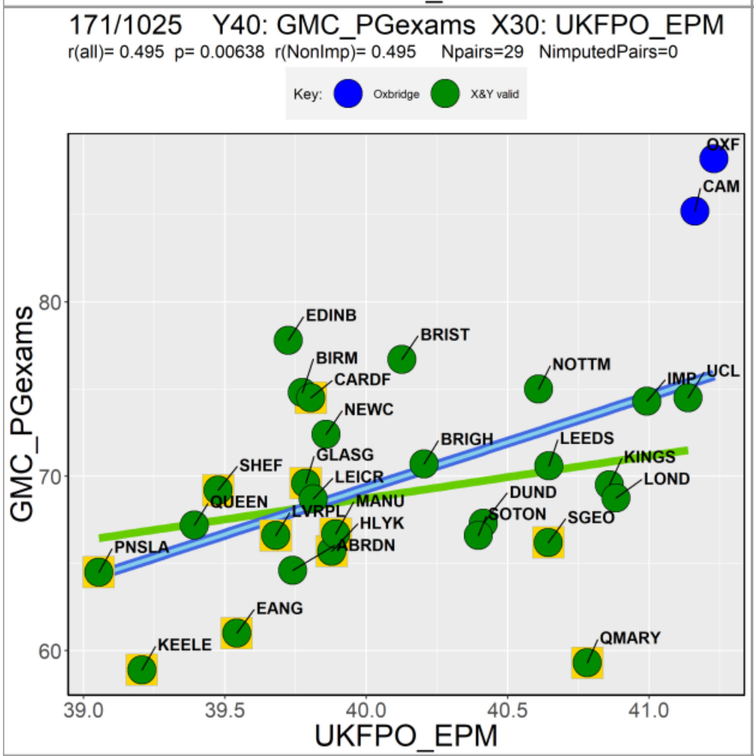
171/1025 Y40: GMC_PGexams X30: UKFPO_EPM
r(all)= 0.495 p= 0.00638 r(NonImp)= 0.495 Npairs=29 NimputedPairs=0
Key: Oxbridge X&Y valid
GMC_PGexams
UKFPO_EPM

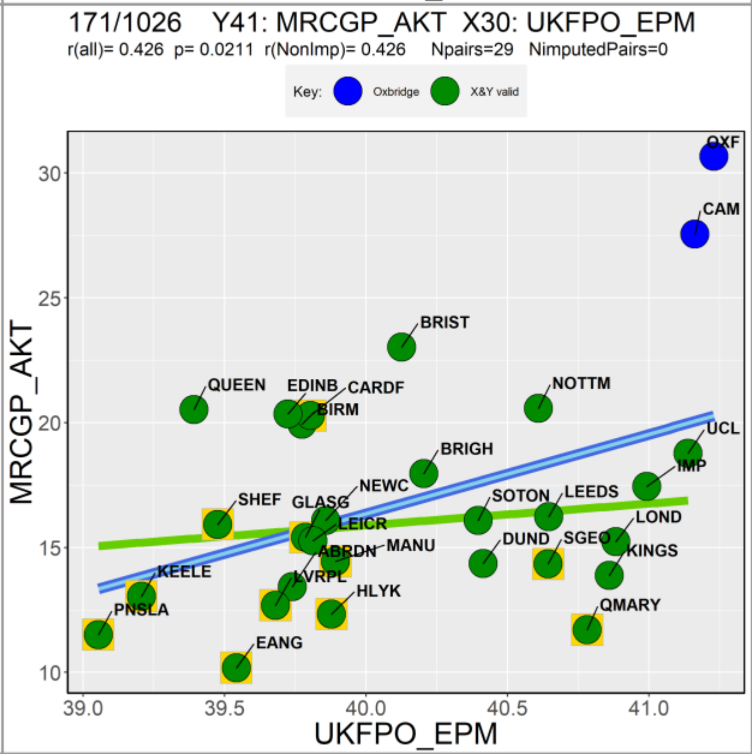
171/1026 Y41: MRCGP_AKT X30: UKFPO_EPM
r(all)= 0.426 p= 0.0211 r(NonImp)= 0.426 Npairs=29 NimputedPairs=0
Key: Oxbridge X&Y valid
MRCGP_AKT
UKFPO_EPM

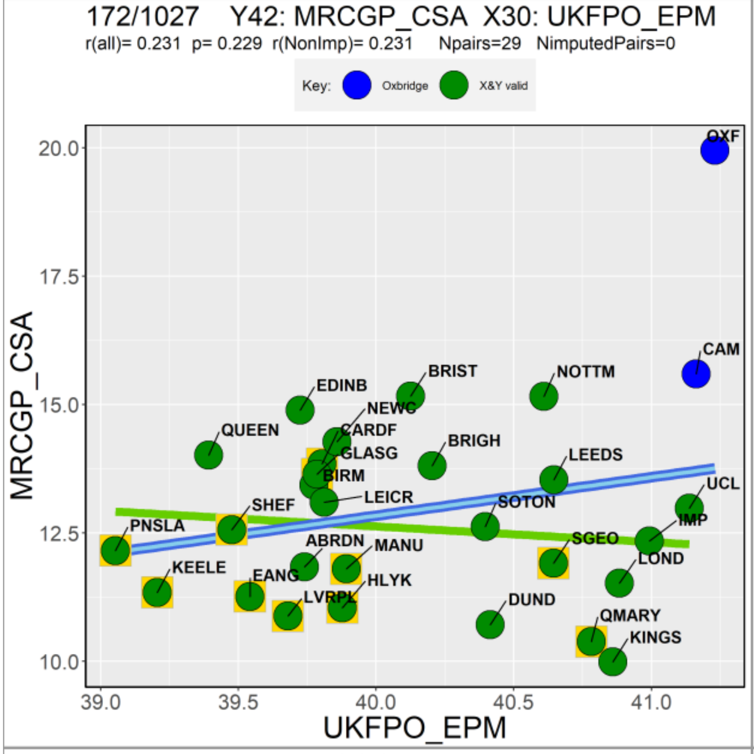
172/1027 Y42: MRCGP_CSA X30: UKFPO_EPM
r(all)= 0.231 p= 0.229 r(NonImp)= 0.231 Npairs=29 NimputedPairs=0
Key: Oxbridge X&Y valid
MRCGP_CSA
UKFPO_EPM

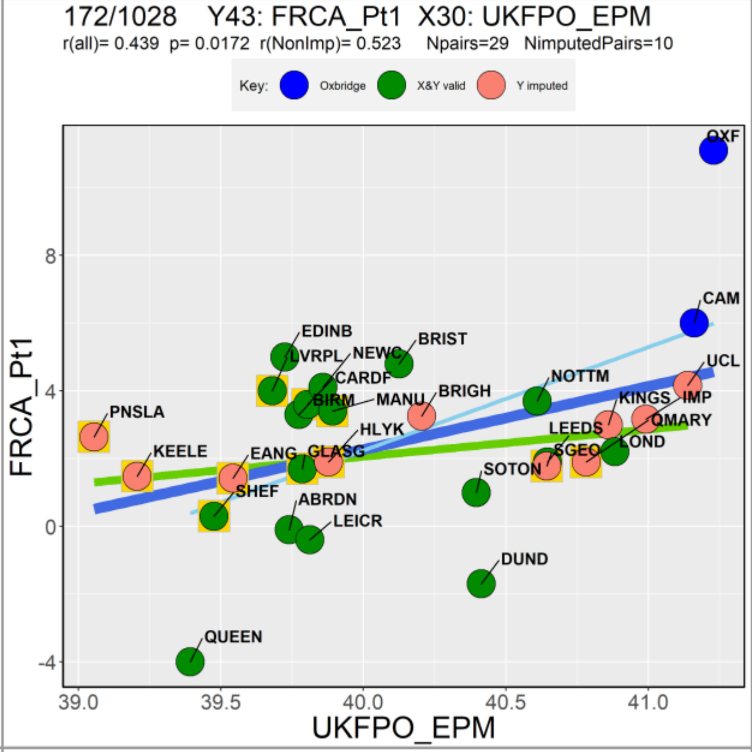
172/1028 Y43: FRCA_Pt1 X30: UKFPO_EPM
r(all)= 0.439 p= 0.0172 r(NonImp)= 0.523 Npairs=29 NimputedPairs=10
Key: Oxbridge X&Y valid Y imputed
FRCA_Pt1
UKFPO_EPM

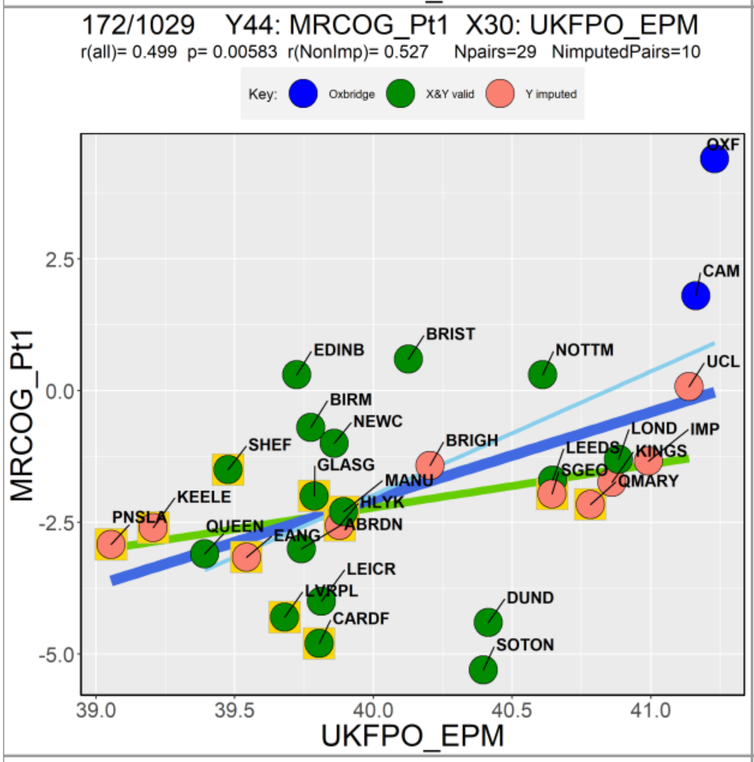
172/1029 Y44: MRCOG_Pt1 X30: UKFPO_EPM
r(all)= 0.499 p= 0.00583 r(NonImp)= 0.527 Npairs=29 NimputedPairs=10
Key: Oxbridge X&Y valid Y imputed
MRCOG_Pt1
UKFPO_EPM

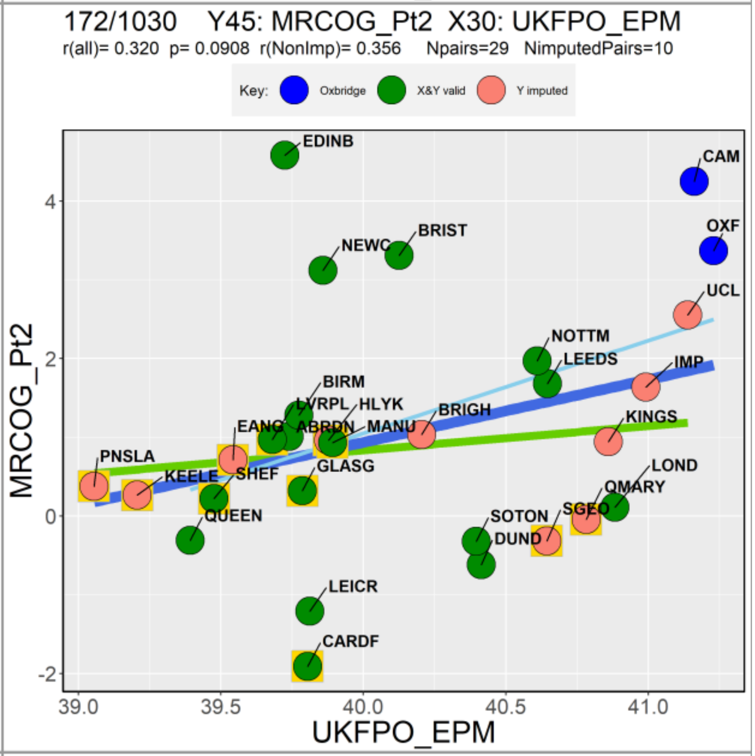
172/1030 Y45: MRCOG_Pt2 X30: UKFPO_EPM
r(all)= 0.320 p= 0.0908 r(NonImp)= 0.356 Npairs=29 NimputedPairs=10
Key: Oxbridge X&Y valid Y imputed
MRCOG_Pt2
UKFPO_EPM

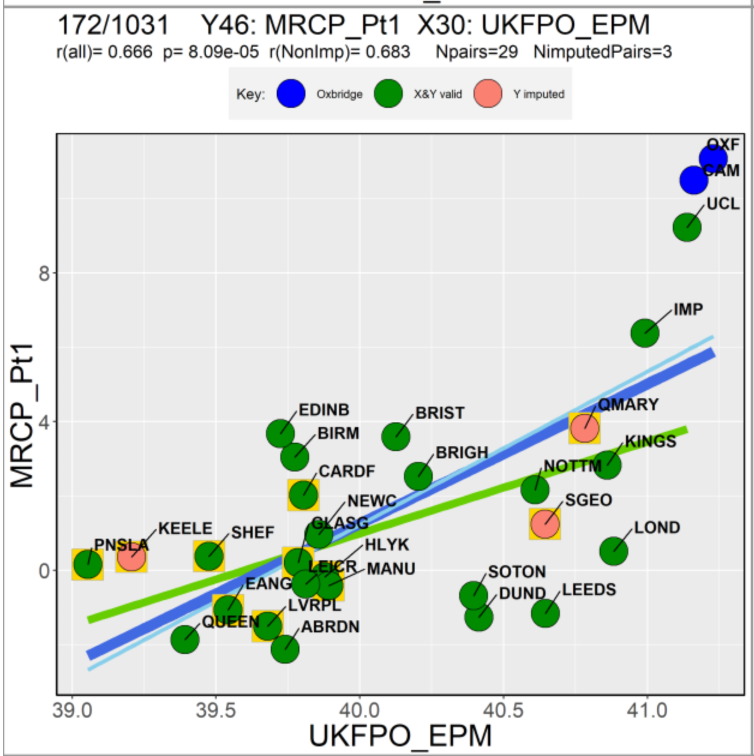
172/1031 Y46: MRCP_Pt1 X30: UKFPO_EPM
r(all)= 0.666 p= 8.09e-05 r(NonImp)= 0.683 Npairs=29 NimputedPairs=3
Key: Oxbridge X&Y valid Y imputed
MRCP_Pt1
UKFPO_EPM

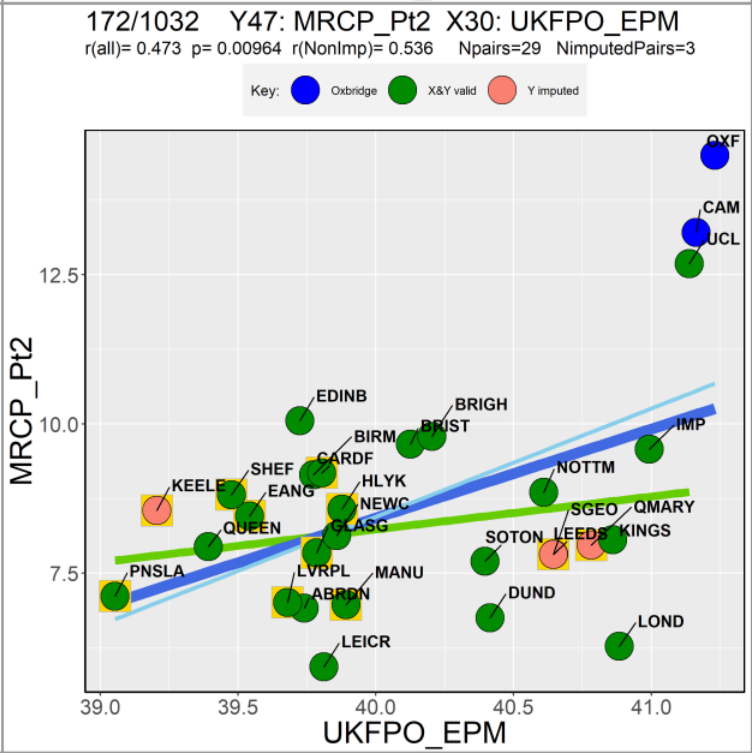
172/1032 Y47: MRCP_Pt2 X30: UKFPO_EPM
r(all)= 0.473 p= 0.00964 r(NonImp)= 0.536 Npairs=29 NimputedPairs=3
Key: Oxbridge X&Y valid Y imputed
MRCP_Pt2
UKFPO_EPM

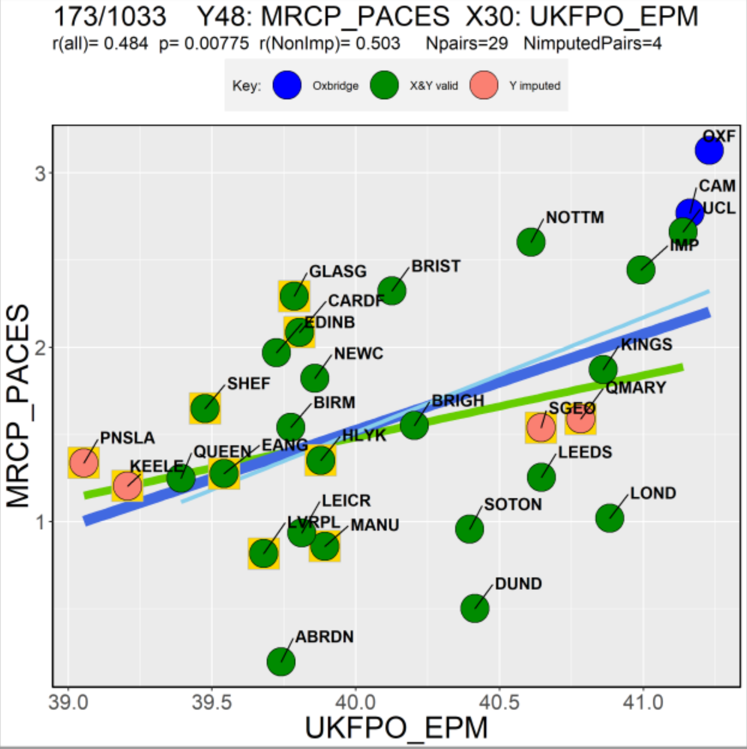
173/1033 Y48: MRCP_PACES X30: UKFPO_EPM
r(all)= 0.484 p= 0.00775 r(NonImp)= 0.503 Npairs=29 NimputedPairs=4
Key: Oxbridge X&Y valid Y imputed
MRCP_PACES
UKFPO_EPM

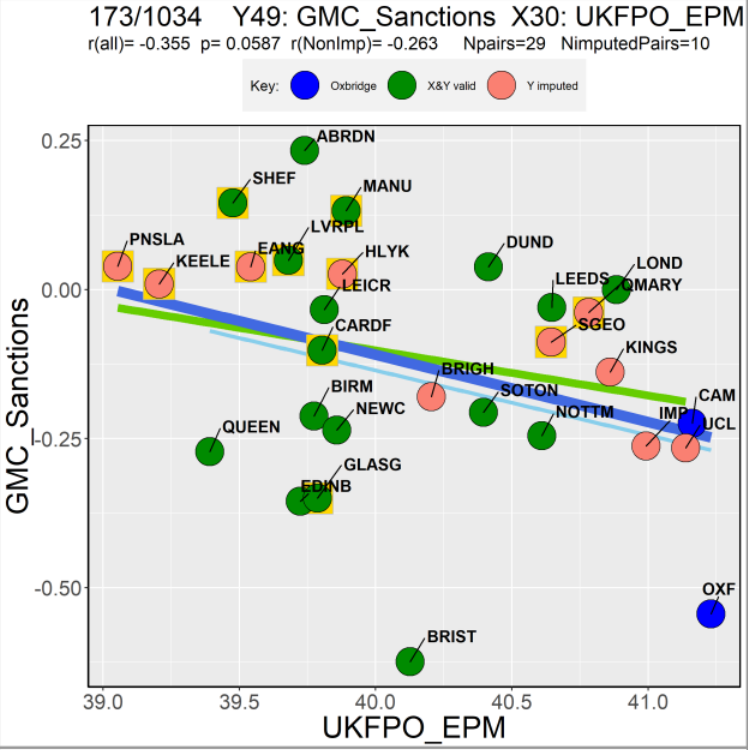
173/1034 Y49: GMC_Sanctions X30: UKFPO_EPM
r(all)= -0.355 p= 0.0587 r(NonImp)= -0.263 Npairs=29 NimputedPairs=10
Key: Oxbridge X&Y valid Y imputed
GMC_Sanctions
UKFPO_EPM

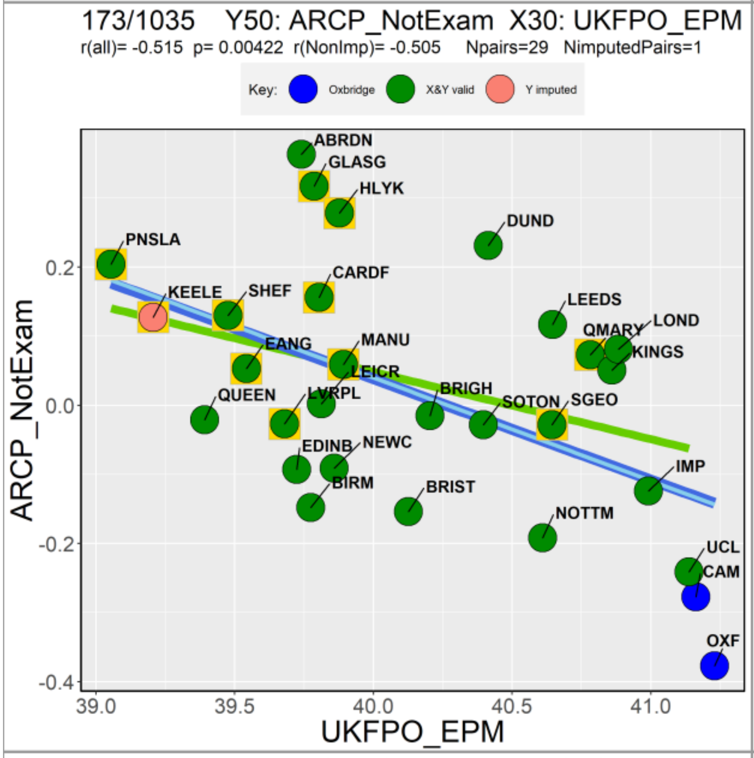
173/1035 Y50: ARCP_NotExam X30: UKFPO_EPM
r(all)= -0.515 p= 0.00422 r(NonImp)= -0.505 Npairs=29 NimputedPairs=1
Key: Oxbridge X&Y valid Y imputed
ARCP_NotExam
UKFPO_EPM

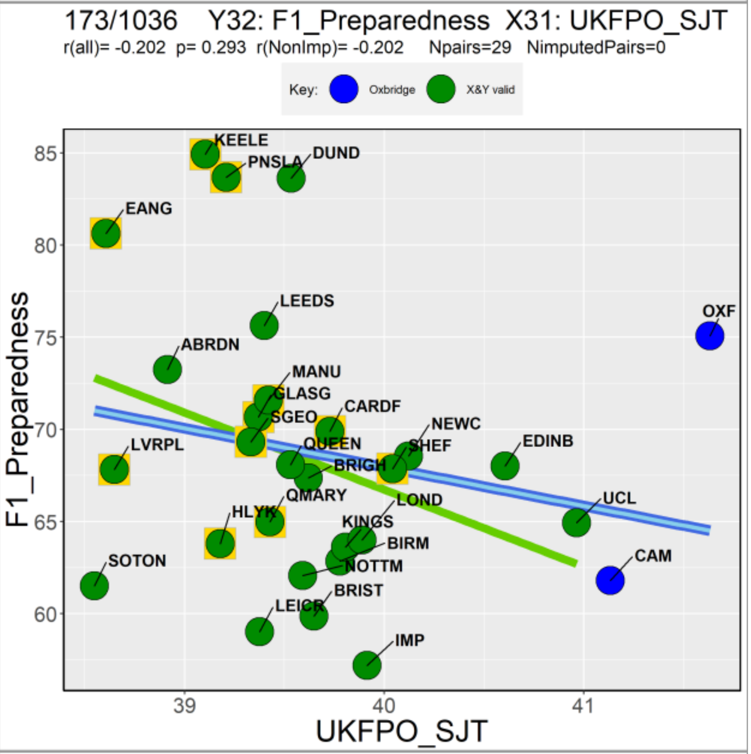
173/1036 Y32: F1_Preparedness X31: UKFPO_SJT
r(all)= -0.202 p= 0.293 r(NonImp)= -0.202 Npairs=29 NimputedPairs=0
Key: Oxbridge X&Y valid
F1_Preparedness
UKFPO_SJT

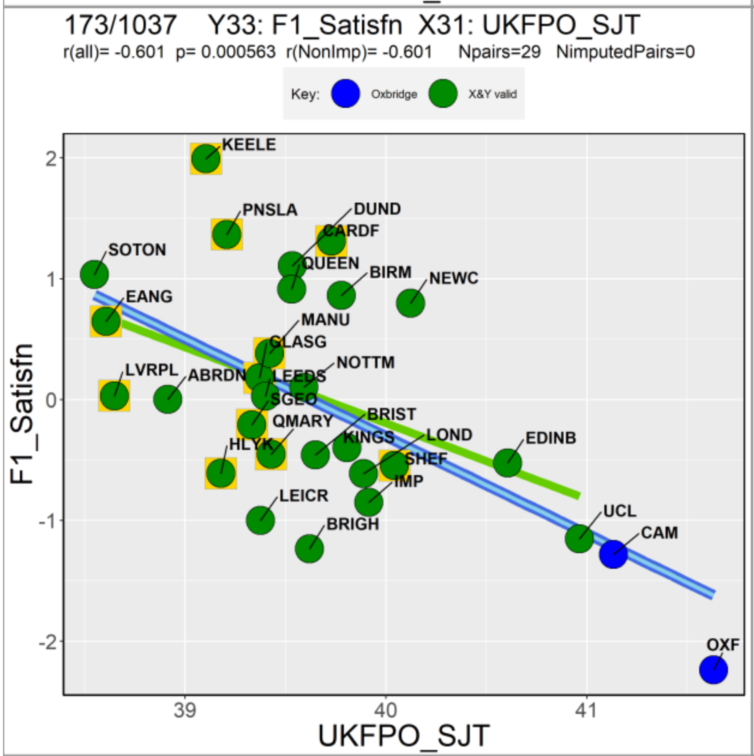
173/1037 Y33: F1_Satisfn X31: UKFPO_SJT
r(all)= -0.601 p= 0.000563 r(NonImp)= -0.601 Npairs=29 NimputedPairs=0
Key: Oxbridge X&Y valid
F1_Satisfn
UKFPO_SJT

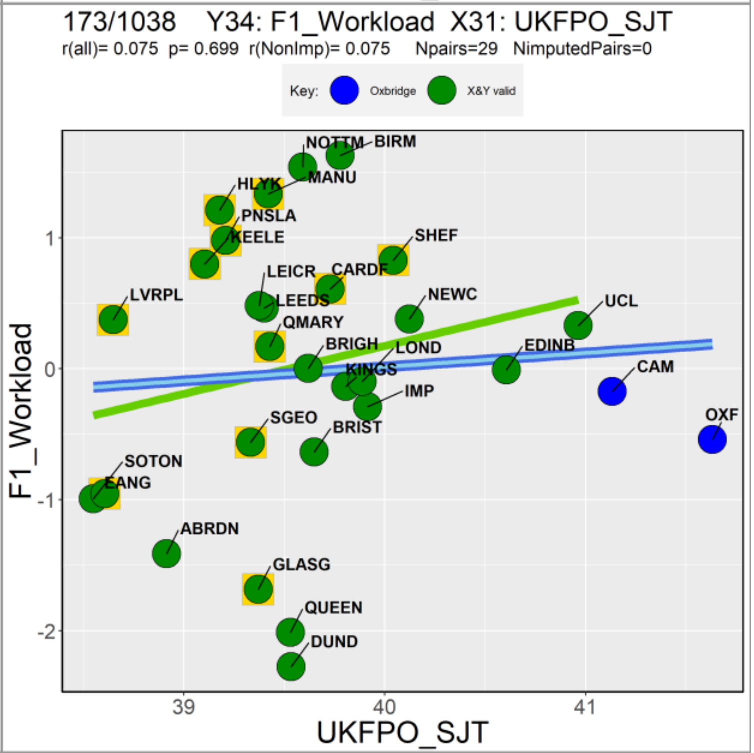
173/1038 Y34: F1_Workload X31: UKFPO_SJT
r(all)= 0.075 p= 0.699 r(NonImp)= 0.075 Npairs=29 NimputedPairs=0
Key: Oxbridge X&Y valid
F1_Workload
UKFPO_SJT

174/1039 Y35: F1_Supervn X31: UKFPO_SJT
r(all)= 0.355 p= 0.0586 r(NonImp)= 0.355 Npairs=29 NimputedPairs=0

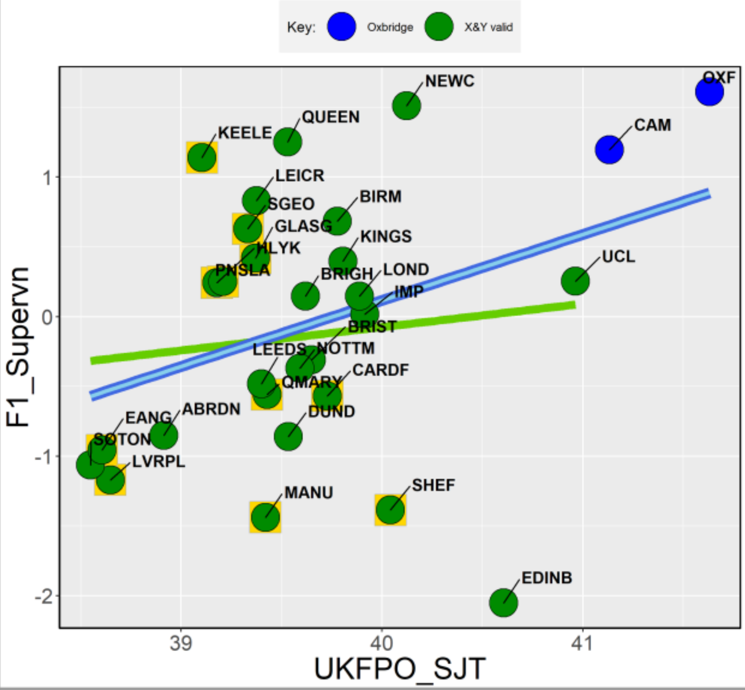

174/1040 Y36: Trainee_GP X31: UKFPO_SJT
r(all)= -0.608 p= 0.000473 r(NonImp)= -0.608 Npairs=29 NimputedPairs=0

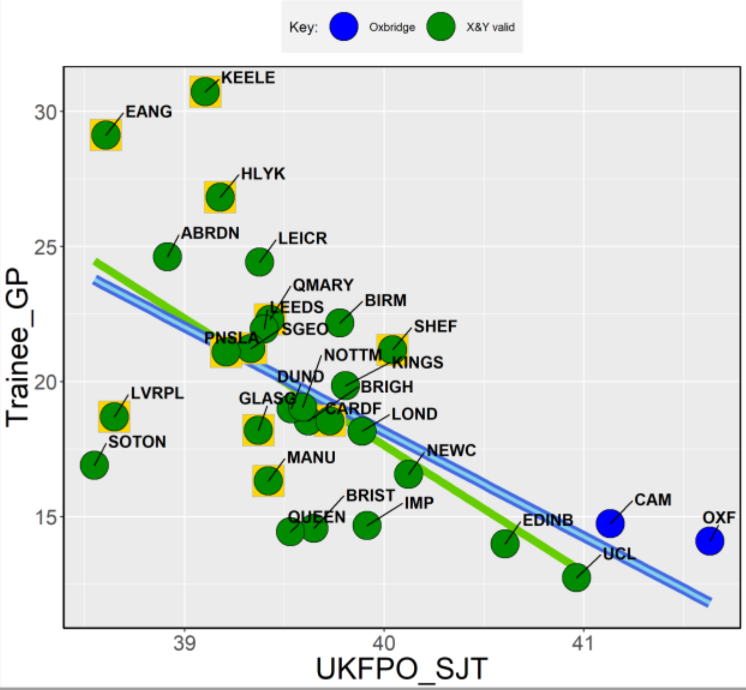

174/1041 Y37: Trainee_Psyc X31: UKFPO_SJT
r(all)= -0.086 p= 0.658 r(NonImp)= -0.086 Npairs=29 NimputedPairs=0

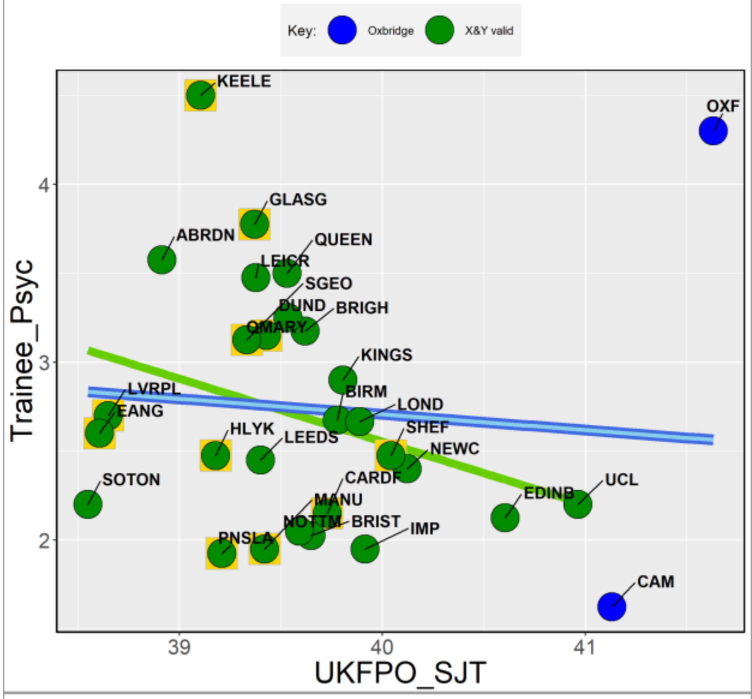

174/1042 Y38: TraineeApp_Surgery X31: UKFPO_S
r(all)= 0.075 p= 0.699 r(NonImp)= 0.093 Npairs=29 NimputedPairs=2

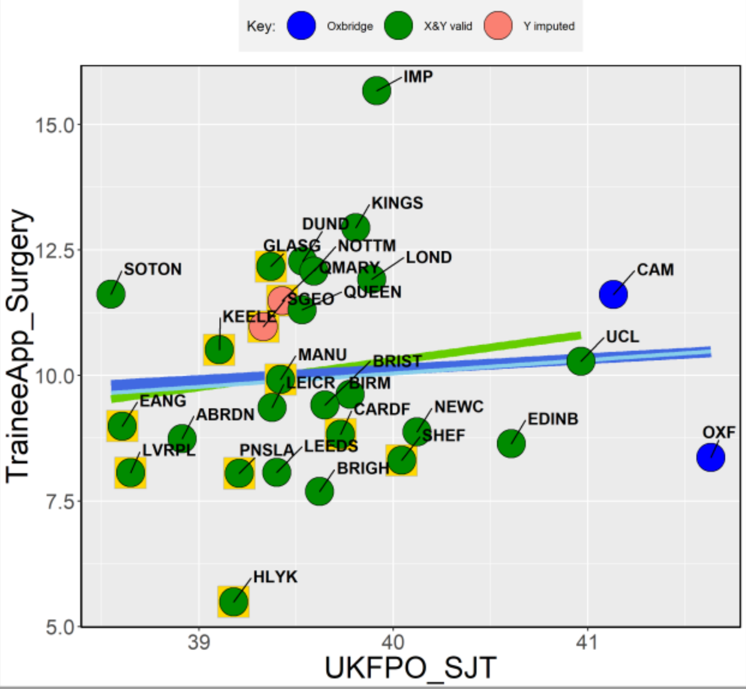

174/1043 Y39: TraineeApp_Anaes X31: UKFPO_SJ
r(all)= 0.198 p= 0.302 r(NonImp)= 0.198 Npairs=29 NimputedPairs=0

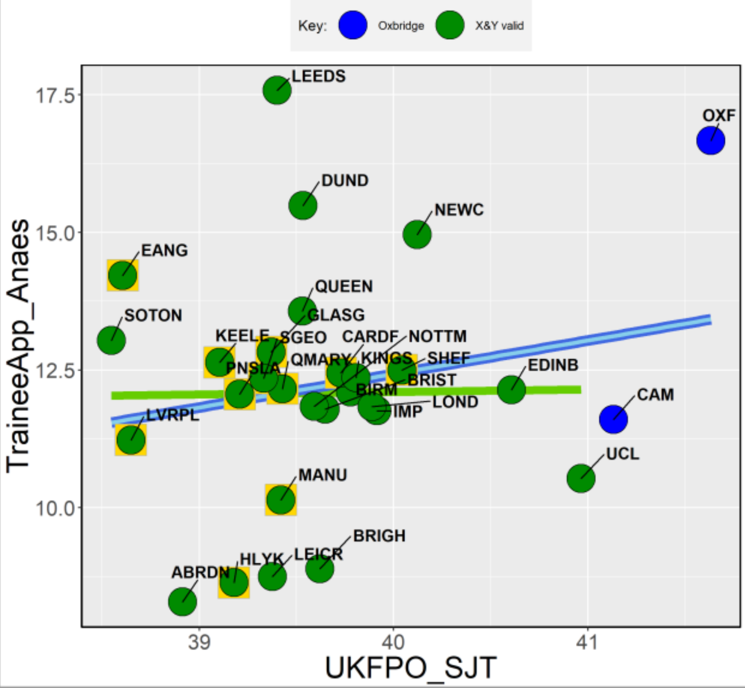

174/1044 Y40: GMC_PGexams X31: UKFPO_SJT
r(all)= 0.827 p= 3.15e-08 r(NonImp)= 0.827 Npairs=29 NimputedPairs=0

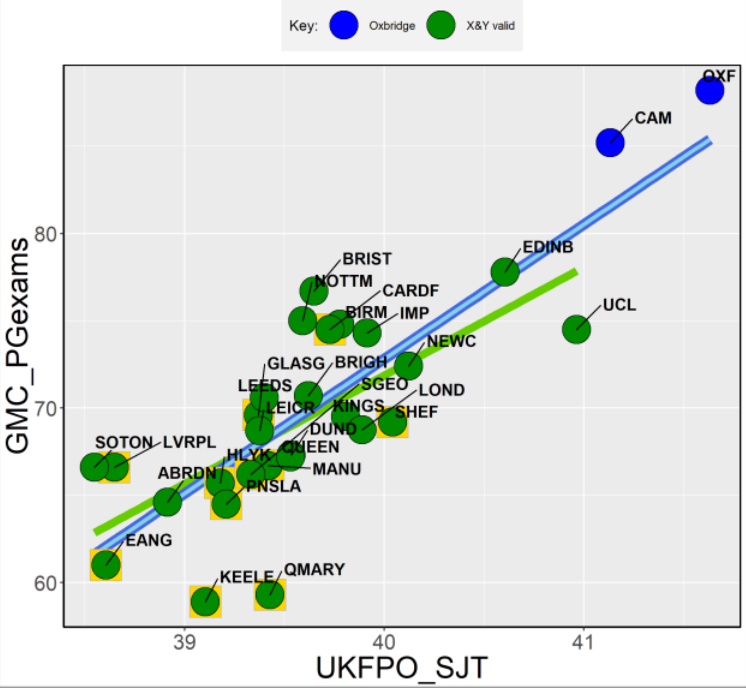

175/1045 Y41: MRCGP_AKT X31: UKFPO_SJT
r(all)= 0.776 p= 7.77e-07 r(NonImp)= 0.776 Npairs=29 NimputedPairs=0

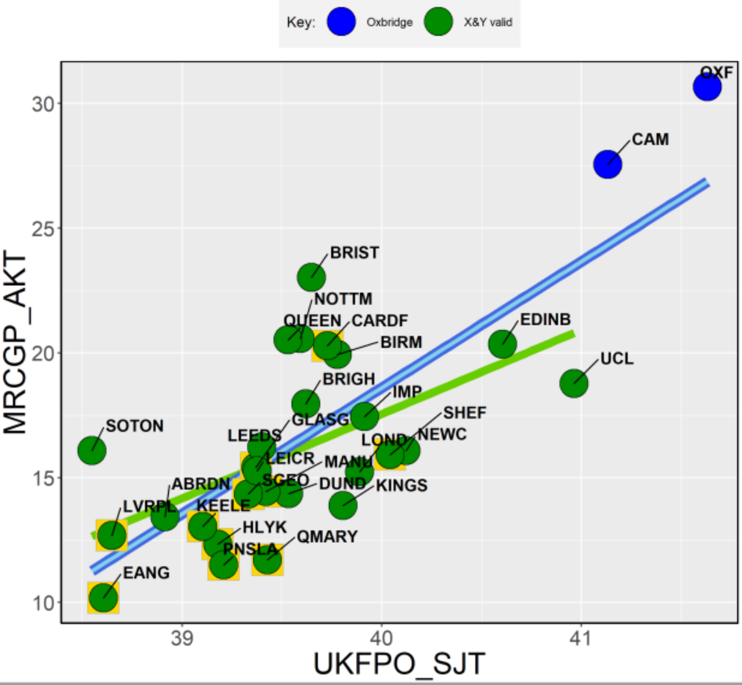

175/1046 Y42: MRCGP_CSA X31: UKFPO_SJT
r(all)= 0.669 p= 7.35e-05 r(NonImp)= 0.669 Npairs=29 NimputedPairs=0

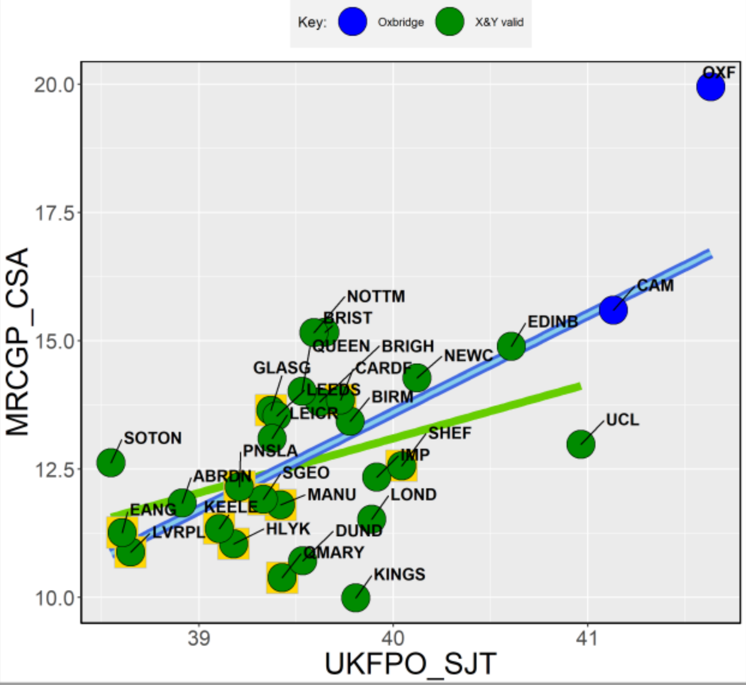

175/1047 Y43: FRCA_Pt1 X31: UKFPO_SJT
r(all)= 0.630 p= 0.000247 r(NonImp)= 0.644 Npairs=29 NimputedPairs=10

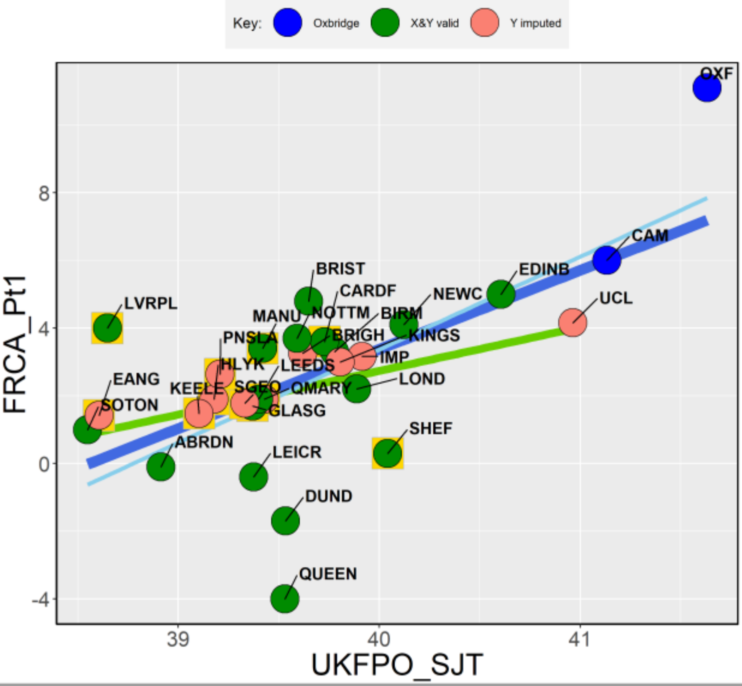

175/1048 Y44: MRCOG_Pt1 X31: UKFPO_SJT
r(all)= 0.821 p= 4.95e-08 r(NonImp)= 0.834 Npairs=29 NimputedPairs=10

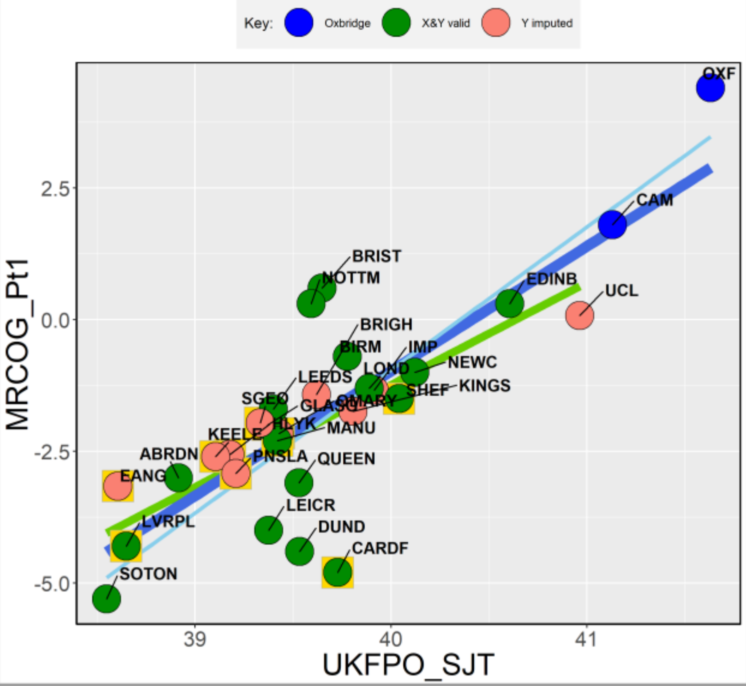

175/1049 Y45: MRCOG_Pt2 X31: UKFPO_SJT
r(all)= 0.623 p= 0.000309 r(NonImp)= 0.608 Npairs=29 NimputedPairs=10

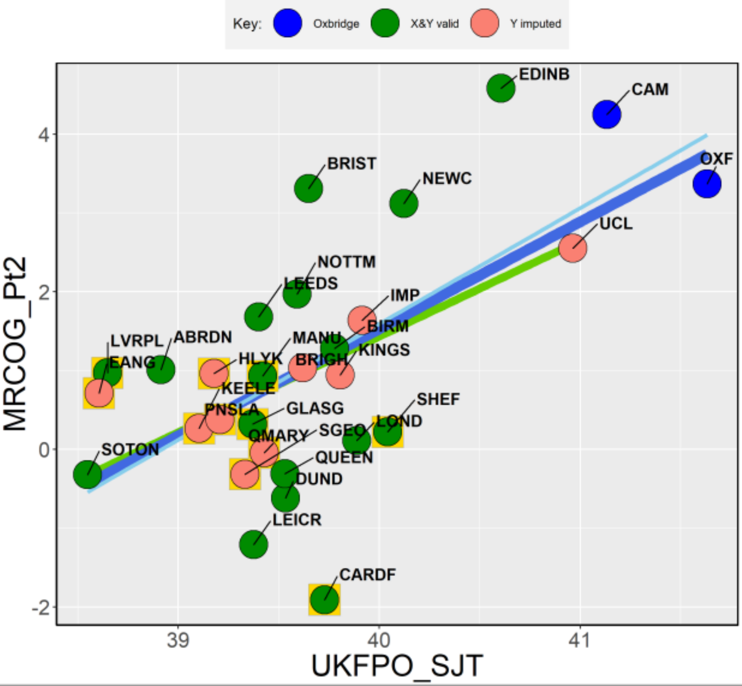

175/1050 Y46: MRCP_Pt1 X31: UKFPO_SJT
r(all)= 0.851 p= 4.96e-09 r(NonImp)= 0.866 Npairs=29 NimputedPairs=3

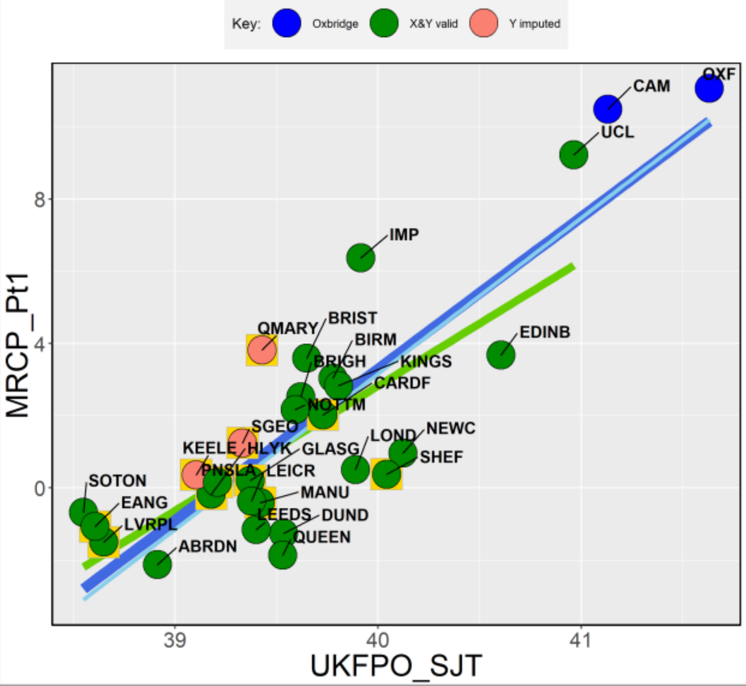

Supplement: Supplementary file 8 — Additional file 8. Graphs 841 to 1050 (pages 141 to 175). [file 12916_2020_1572_MOESM8_ESM.pdf]
