# Supplementary material for: Exploring UK medical school differences: the MedDifs study of selection, teaching, student and F1 perceptions, postgraduate outcomes and fitness to practise
Source: BMC Med. 2020 May 14;18:136. doi: 10.1186/s12916-020-01572-3 (PMC7222458; doi:10.1186/s12916-020-01572-3)
Supplement: Supplementary file 9 — Additional file 9. Graphs 1051 to 1225 (pages 176 to 205). [file 12916_2020_1572_MOESM9_ESM.pdf]

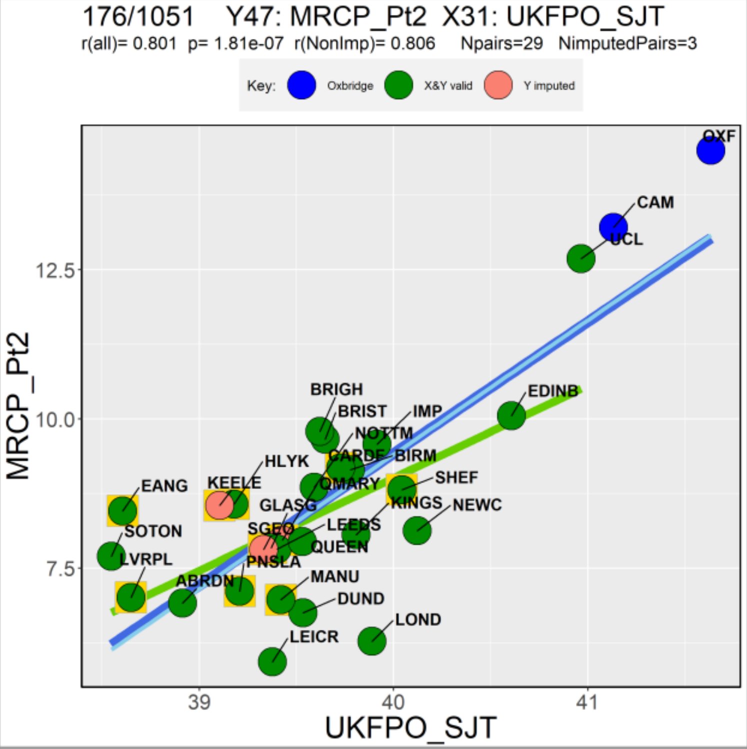
176/1051 Y47: MRCP_Pt2 X31: UKFPO_SJT
r(all)= 0.801 p= 1.81e-07 r(NonImp)= 0.806 Npairs=29 NimputedPairs=3
Key: Oxbridge X&Y valid Y imputed
MRCP_Pt2
UKFPO_SJT
12.5
10.0
7.5
39
40
41
OXF
CAM
UCL
EDINB
BRIGH
BRIST
IMP
NOTTM
HLYK
CARDF
BIRM
EANG
KEELE
QMARY
SHEF
GLASG
KINGS
NEWC
SOTON
SGEO
LEEDS
QUEEN
LVRPL
PNSLA
ABRDN
MANU
DUND
LOND
LEICR

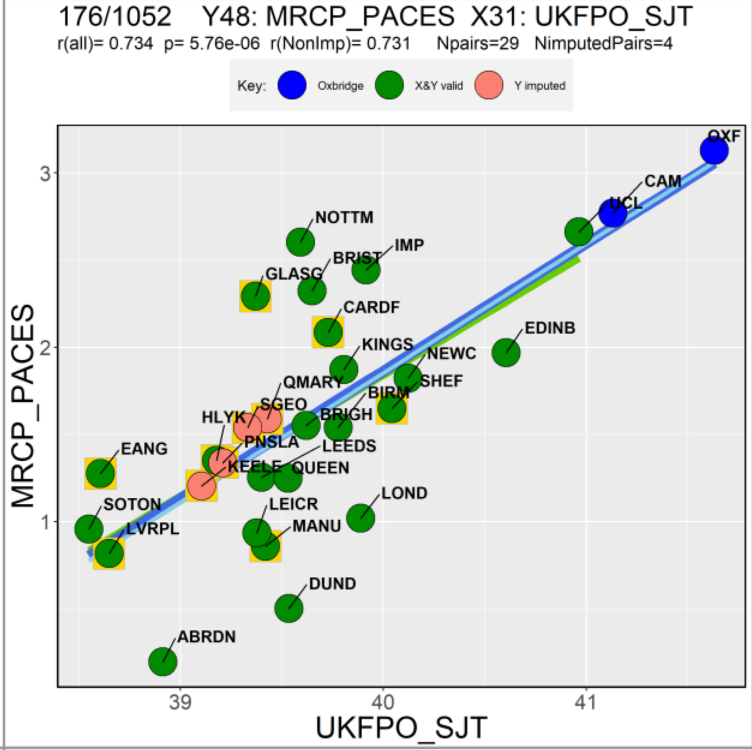
176/1052 Y48: MRCP_PACES X31: UKFPO_SJT
r(all)= 0.734 p= 5.76e-06 r(NonImp)= 0.731 Npairs=29 NimputedPairs=4
Key: Oxbridge X&Y valid Y imputed
MRCP_PACES
UKFPO_SJT
3
2
1
39
40
41
OXF
CAM
UCL
NOTTM
BRIST
IMP
GLASG
CARDF
EDINB
KINGS
NEWC
QMARY
SHEF
BIRM
SGEO
HLYK
BRIGH
PNSLA
LEEDS
EANG
KEELE
QUEEN
SOTON
LEICR
LOND
LVRPL
MANU
DUND
ABRDN

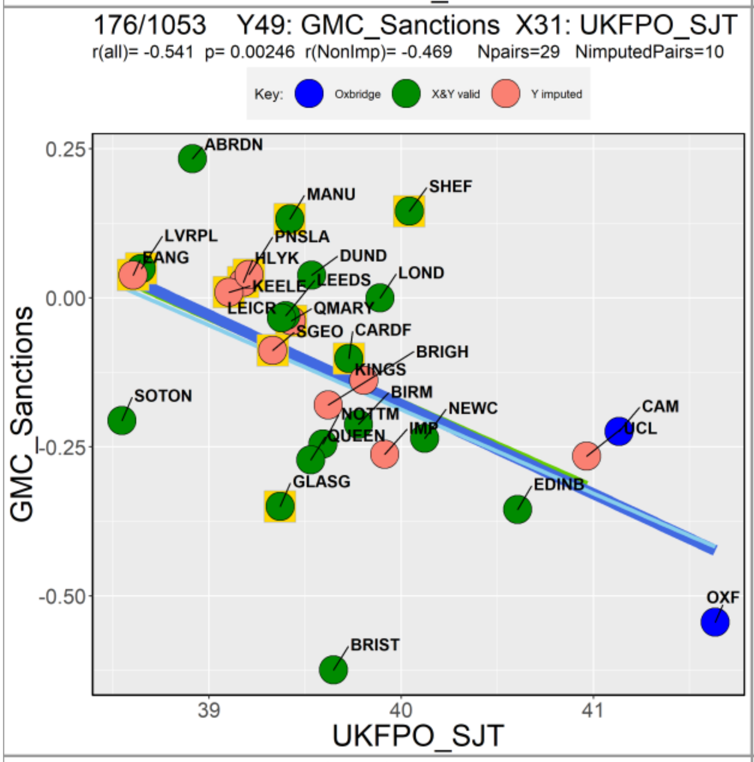
176/1053 Y49: GMC_Sanctions X31: UKFPO_SJT
r(all)= -0.541 p= 0.00246 r(NonImp)= -0.469 Npairs=29 NimputedPairs=10
Key: Oxbridge X&Y valid Y imputed
GMC_Sanctions
UKFPO_SJT
0.25
0.00
-0.25
-0.50
39
40
41
ABRDN
SHEF
MANU
LVRPL
PNSLA
EANG
HLYK
DUND
LEEDS
LOND
KEELE
LEICR
QMARY
SGEO
CARDF
BRIGH
KINGS
SOTON
BIRM
NEWC
CAM
NOTTM
UCL
QUEEN
IMP
GLASG
EDINB
OXF
BRIST

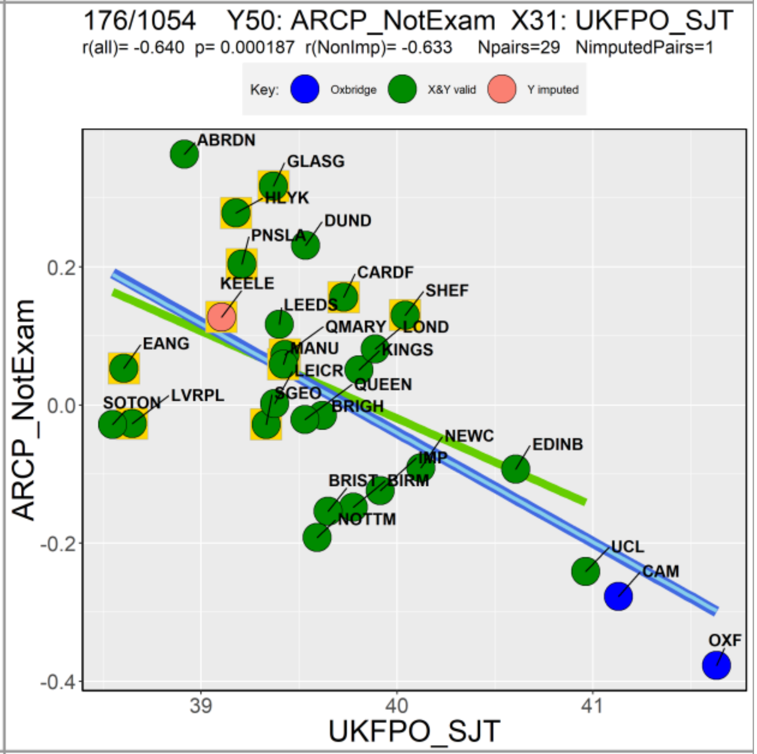
176/1054 Y50: ARCP_NotExam X31: UKFPO_SJT
r(all)= -0.640 p= 0.000187 r(NonImp)= -0.633 Npairs=29 NimputedPairs=1
Key: Oxbridge X&Y valid Y imputed
ARCP_NotExam
UKFPO_SJT
0.2
0.0
-0.2
-0.4
39
40
41
ABRDN
GLASG
HLYK
DUND
PNSLA
CARDF
SHEF
KEELE
LEEDS
QMARY
LOND
EANG
MANU
KINGS
LEICR
QUEEN
SOTON
LVRPL
SGEO
BRIGH
NEWC
EDINB
IMP
BRIST
BIRM
NOTTM
UCL
CAM
OXF

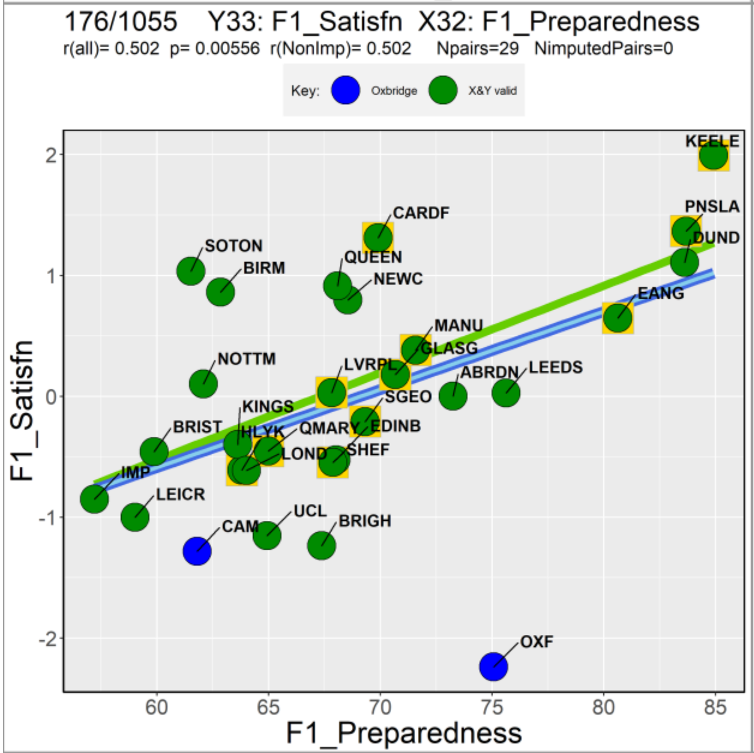
176/1055 Y33: F1_Satisfn X32: F1_Preparedness
r(all)= 0.502 p= 0.00556 r(NonImp)= 0.502 Npairs=29 NimputedPairs=0
Key: Oxbridge X&Y valid
F1_Satisfn
F1_Preparedness
2
1
0
-1
-2
60
65
70
75
80
85
KEELE
PNSLA
CARDF
DUND
SOTON
QUEEN
BIRM
NEWC
EANG
MANU
NOTTM
GLASG
LVRPL
ABRDN
LEEDS
SGEO
KINGS
BRIST
HLYK
QMARY
EDINB
LOND
SHEF
IMP
LEICR
UCL
BRIGH
CAM
OXF

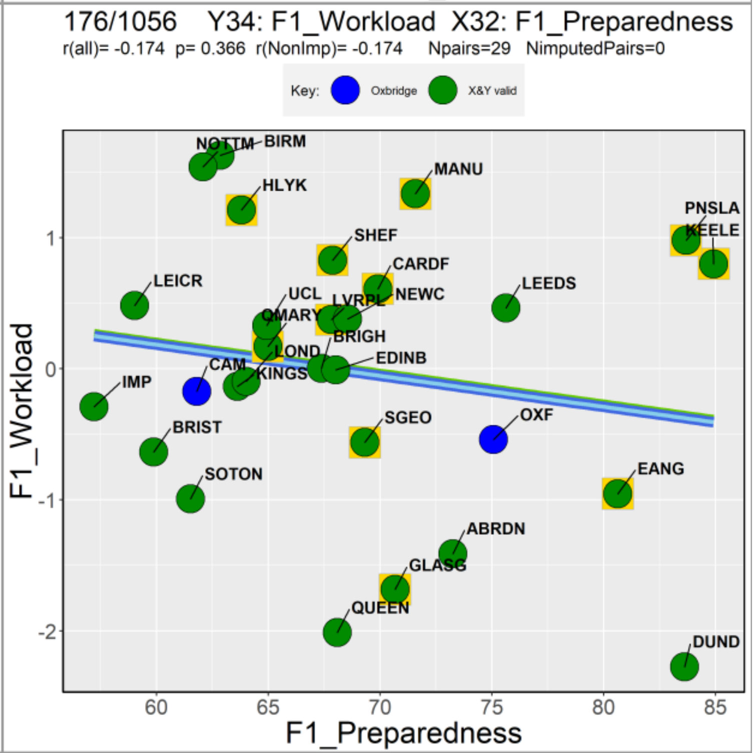
176/1056 Y34: F1_Workload X32: F1_Preparedness
r(all)= -0.174 p= 0.366 r(NonImp)= -0.174 Npairs=29 NimputedPairs=0
Key: Oxbridge X&Y valid
F1_Workload
F1_Preparedness
1
0
-1
-2
60
65
70
75
80
85
NOTTM
BIRM
MANU
HLYK
PNSLA
KEELE
SHEF
CARDF
LEICR
LEEDS
UCL
LVRPL
NEWC
QMARY
BRIGH
LOND
CAM
EDINB
KINGS
IMP
SGEO
OXF
BRIST
SOTON
EANG
ABRDN
GLASG
QUEEN
DUND

177/1057 Y35: F1_Supervn X32: F1_Preparedness
r(all)= -0.053 p= 0.783 r(NonImp)= -0.053 Npairs=29 NimputedPairs=0

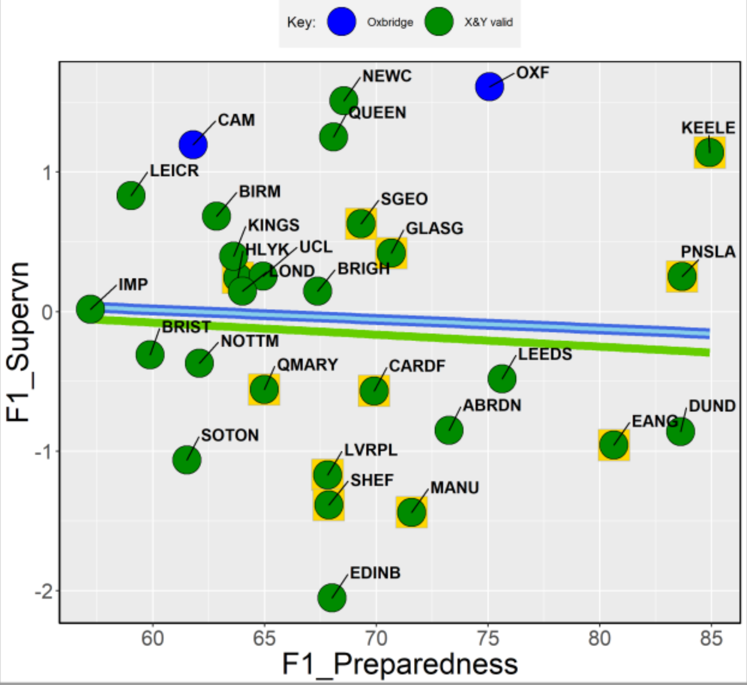

177/1058 Y36: Trainee_GP X32: F1_Preparedness
r(all)= 0.407 p= 0.0283 r(NonImp)= 0.407 Npairs=29 NimputedPairs=0

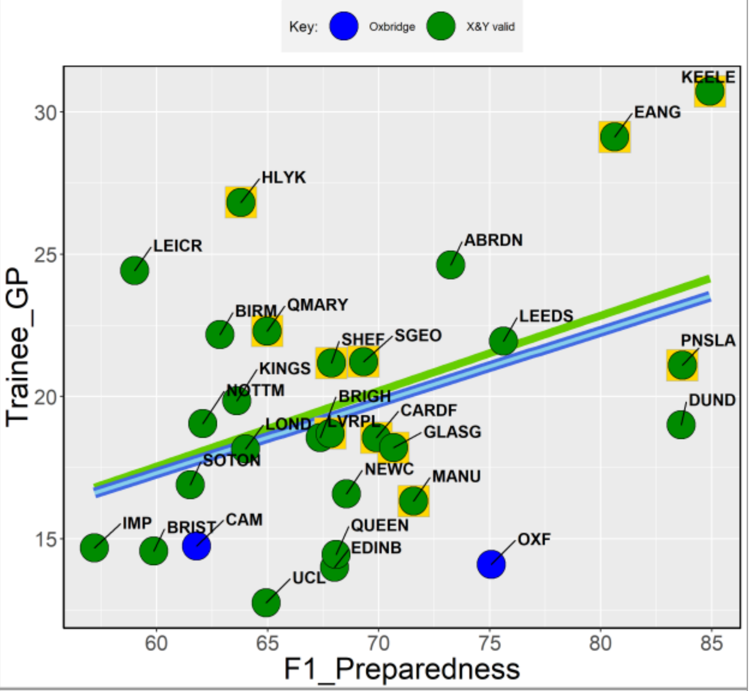

177/1059 Y37: Trainee_Psyc X32: F1_Preparedness
r(all)= 0.386 p= 0.0387 r(NonImp)= 0.386 Npairs=29 NimputedPairs=0

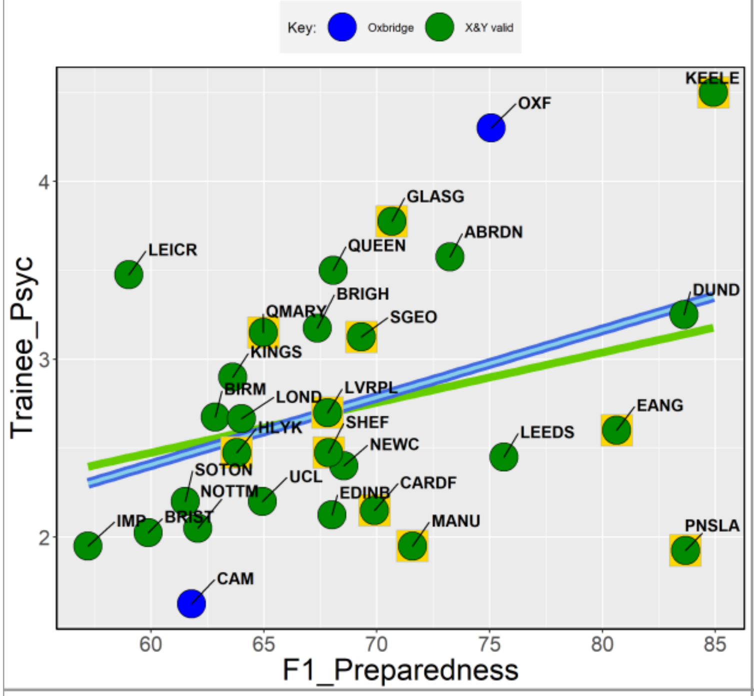

177/1060 Y38: TraineeApp_Surgery X32: F1_Prepar
r(all)= -0.267 p= 0.162 r(NonImp)= -0.260 Npairs=29 NimputedPairs=2

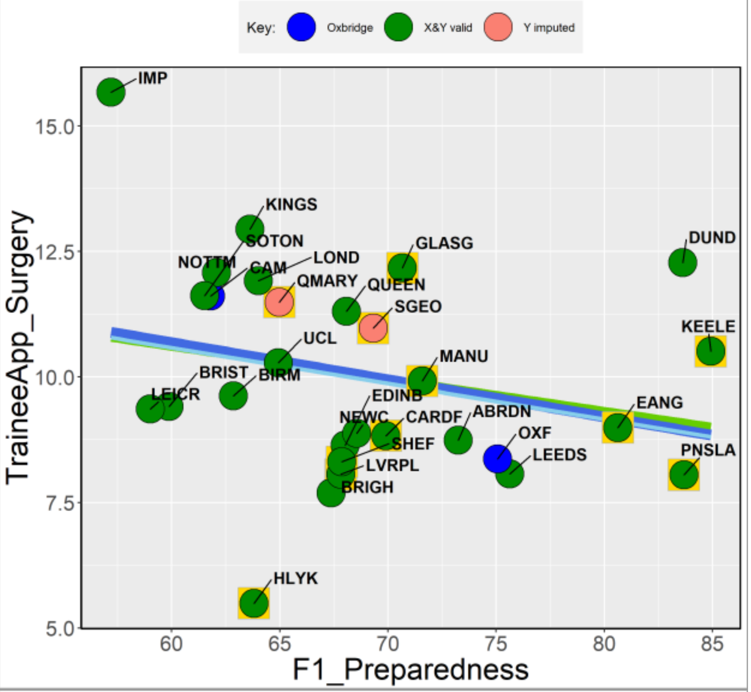

177/1061 Y39: TraineeApp_Anaes X32: F1_Prepare
r(all)= 0.422 p= 0.0224 r(NonImp)= 0.422 Npairs=29 NimputedPairs=0

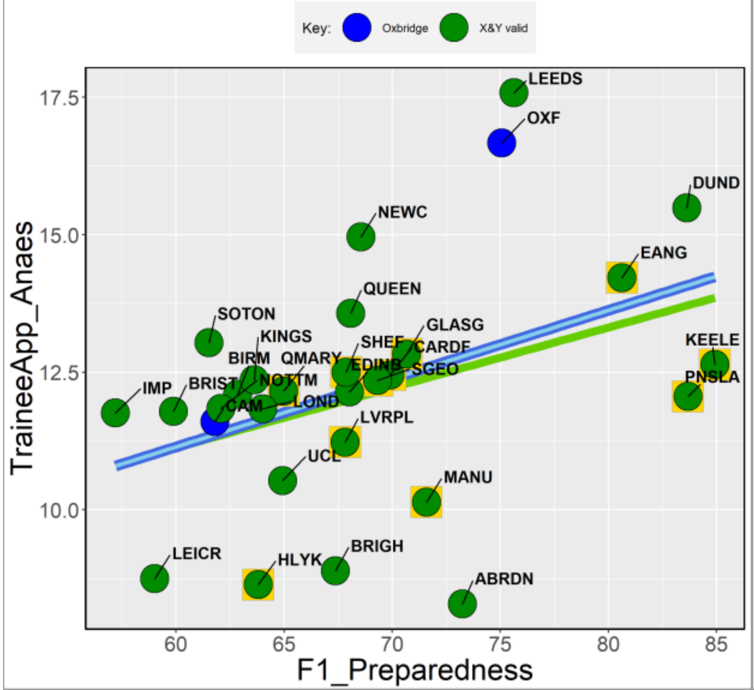

177/1062 Y40: GMC_PGexams X32: F1_Preparednes
r(all)= -0.365 p= 0.0518 r(NonImp)= -0.365 Npairs=29 NimputedPairs=0

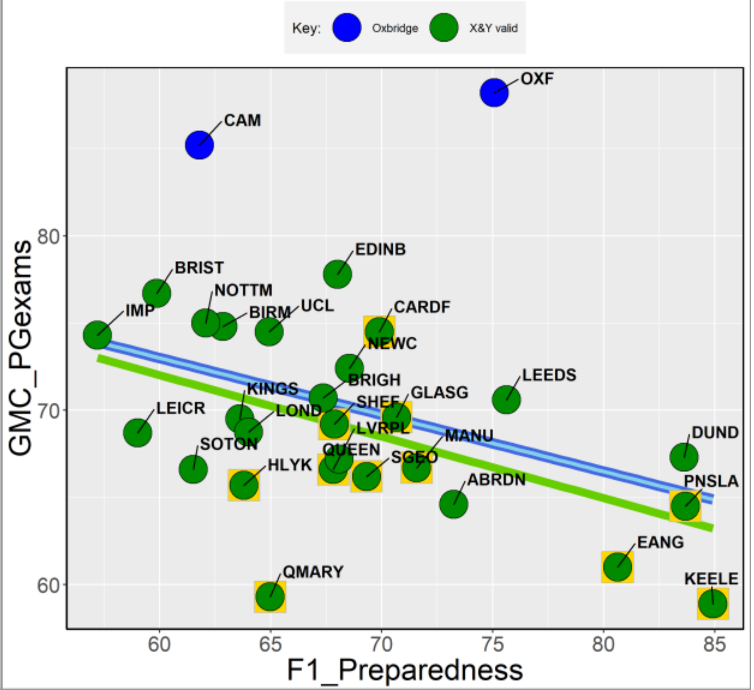

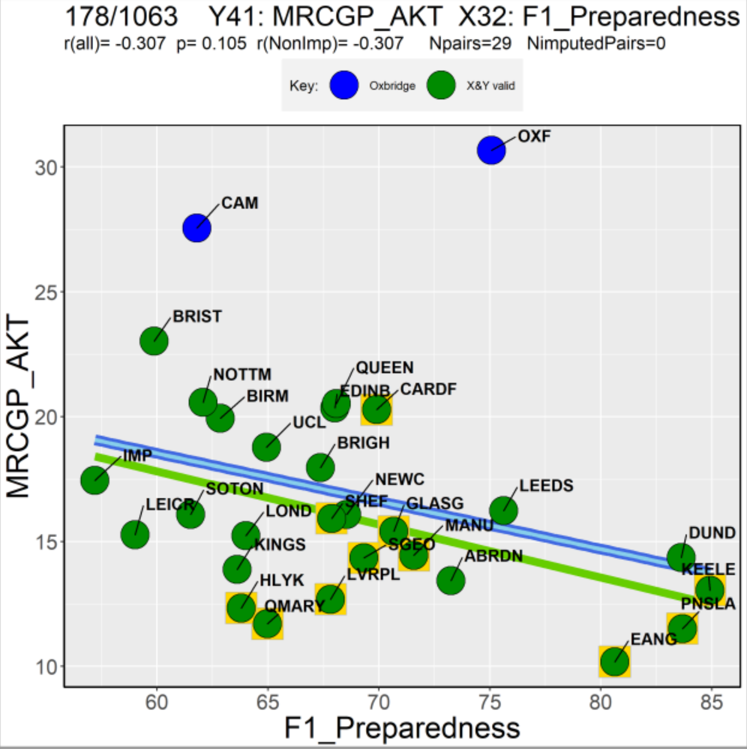
178/1063 Y41: MRCGP_AKT X32: F1_Preparedness
r(all)= -0.307 p= 0.105 r(NonImp)= -0.307 Npairs=29 NimputedPairs=0
Key: Oxbridge X&Y valid
MRCGP_AKT
F1_Preparedness
OXF CAM BRIST NOTTM BIRM QUEEN EDINB CARDF UCL BRIGH IMP NEWC LEEDS SOTON LEICR LOND SHEF GLASG MANU KINGS SGEO ABRDN LVRPL HLYK QMARY DUND KEELE PNSLA EANG
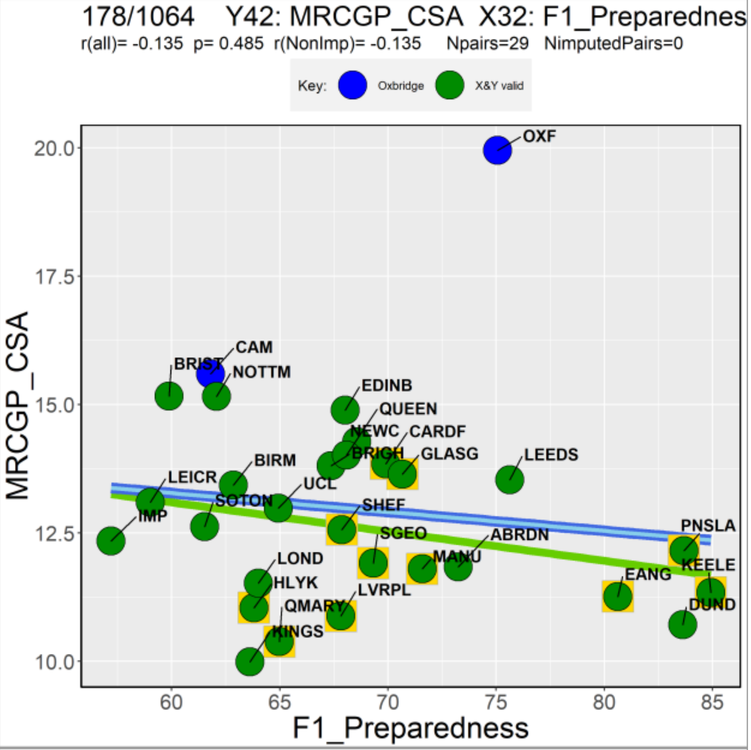
178/1064 Y42: MRCGP_CSA X32: F1_Preparednes
r(all)= -0.135 p= 0.485 r(NonImp)= -0.135 Npairs=29 NimputedPairs=0
Key: Oxbridge X&Y valid
MRCGP_CSA
F1_Preparedness
OXF CAM BRIST NOTTM EDINB QUEEN NEWC CARDF BRIGH GLASG BIRM LEEDS LEICR UCL IMP SOTON SHEF SGEO ABRDN PNSLA LOND MANU EANG KEELE HLYK LVRPL QMARY DUND KINGS
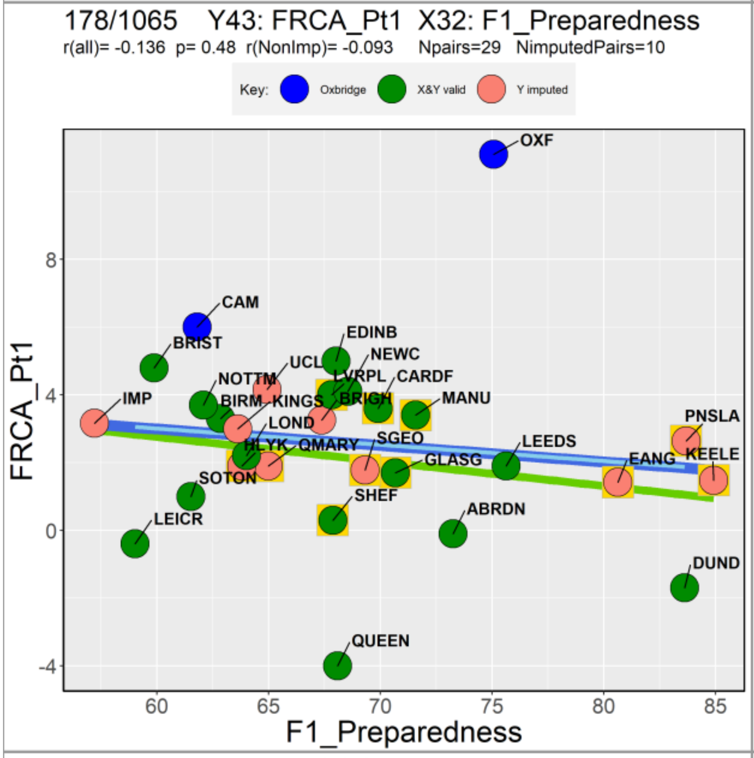
178/1065 Y43: FRCA_Pt1 X32: F1_Preparedness
r(all)= -0.136 p= 0.48 r(NonImp)= -0.093 Npairs=29 NimputedPairs=10
Key: Oxbridge X&Y valid Y imputed
FRCA_Pt1
F1_Preparedness
OXF CAM BRIST EDINB NEWC UCL LVRPL CARDF NOTTM IMP BIRM KINGS BRIGH MANU LOND PNSLA SGEO LEEDS HLYK QMARY GLASG EANG KEELE SOTON SHEF LEICR ABRDN DUND QUEEN
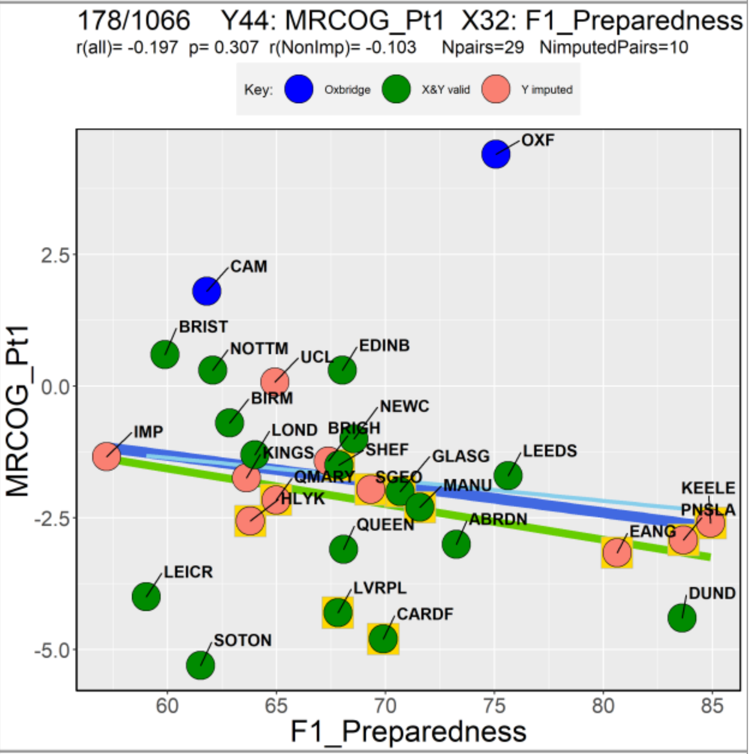
178/1066 Y44: MRCOG_Pt1 X32: F1_Preparedness
r(all)= -0.197 p= 0.307 r(NonImp)= -0.103 Npairs=29 NimputedPairs=10
Key: Oxbridge X&Y valid Y imputed
MRCOG_Pt1
F1_Preparedness
OXF CAM BRIST NOTTM UCL EDINB BIRM NEWC IMP LOND BRIGH SHEF KINGS GLASG LEEDS QMARY SGEO MANU HLYK KEELE PNSLA ABRDN EANG QUEEN LEICR LVRPL DUND CARDF SOTON
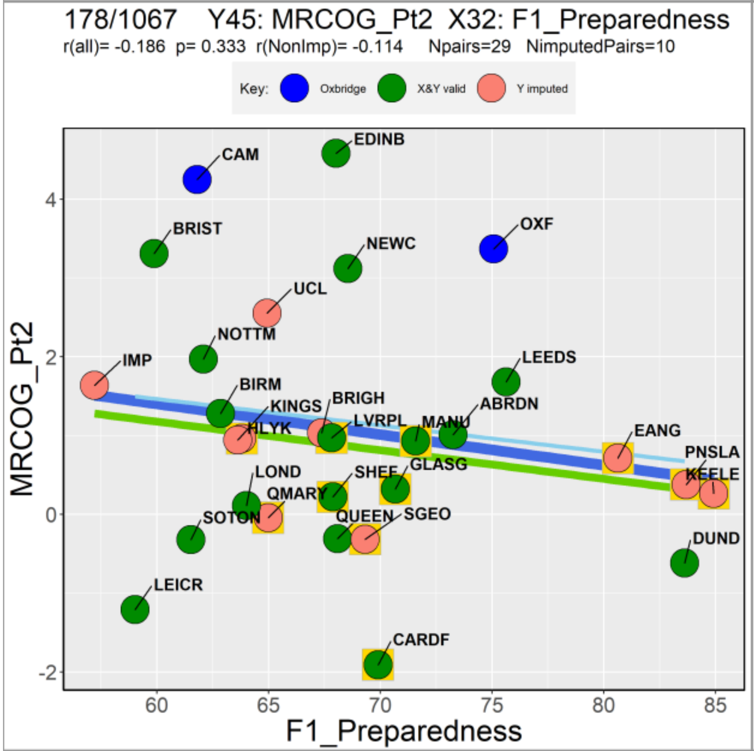
178/1067 Y45: MRCOG_Pt2 X32: F1_Preparedness
r(all)= -0.186 p= 0.333 r(NonImp)= -0.114 Npairs=29 NimputedPairs=10
Key: Oxbridge X&Y valid Y imputed
MRCOG_Pt2
F1_Preparedness
EDINB CAM BRIST NEWC OXF UCL NOTTM IMP LEEDS BIRM KINGS BRIGH ABRDN LVRPL MANU HLYK EANG PNSLA KEELE LOND SHEF GLASG QMARY SOTON QUEEN SGEO DUND LEICR CARDF
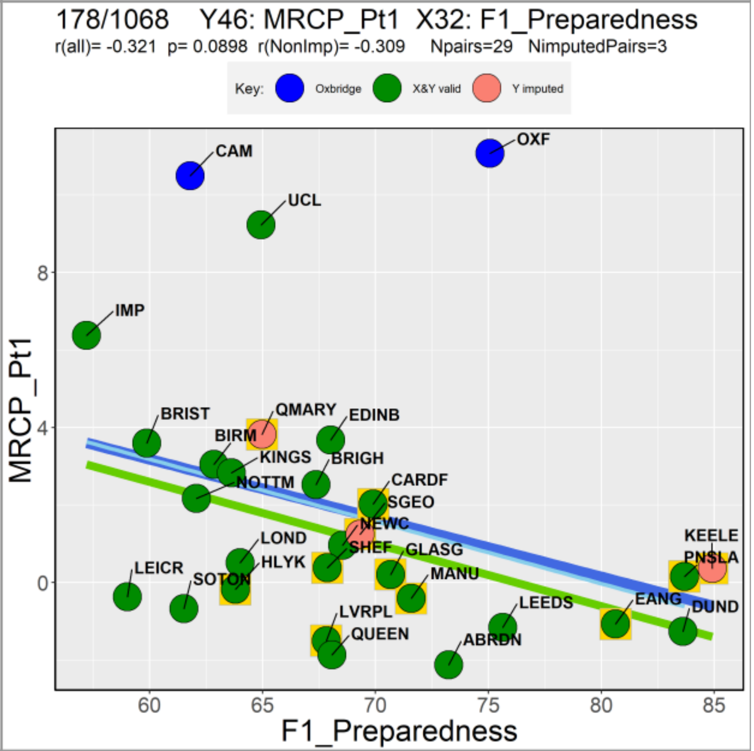
178/1068 Y46: MRCP_Pt1 X32: F1_Preparedness
r(all)= -0.321 p= 0.0898 r(NonImp)= -0.309 Npairs=29 NimputedPairs=3
Key: Oxbridge X&Y valid Y imputed
MRCP_Pt1
F1_Preparedness
OXF CAM UCL IMP QMARY EDINB BRIST BIRM KINGS BRIGH NOTTM CARDF SGEO NEWC LOND SHEF GLASG HLYK KEELE PNSLA LEICR SOTON MANU LEEDS EANG DUND LVRPL QUEEN ABRDN

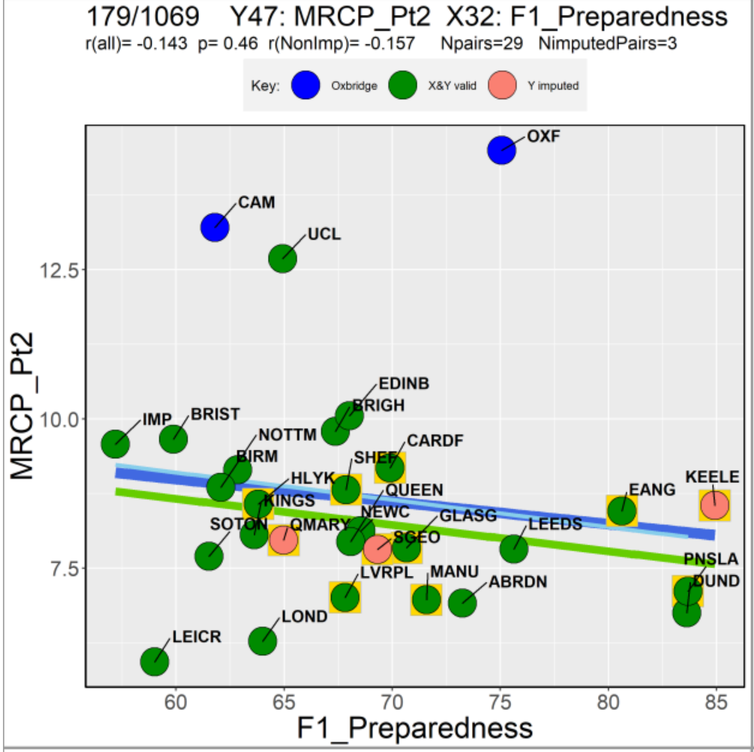
179/1069 Y47: MRCP_Pt2 X32: F1_Preparedness
r(all)= -0.143 p= 0.46 r(NonImp)= -0.157 Npairs=29 NimputedPairs=3
Key: Oxbridge X&Y valid Y imputed
MRCP_Pt2
F1_Preparedness

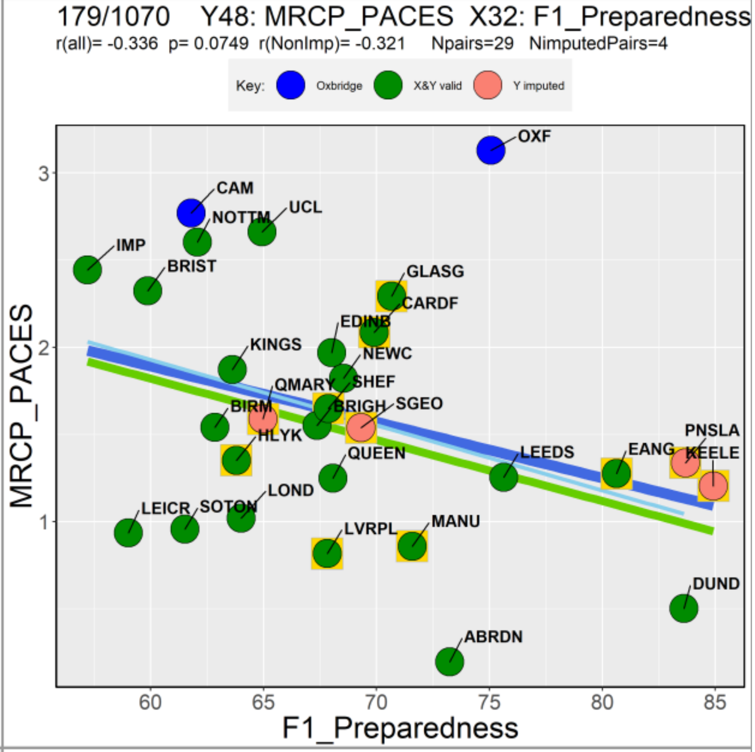
179/1070 Y48: MRCP_PACES X32: F1_Preparedness
r(all)= -0.336 p= 0.0749 r(NonImp)= -0.321 Npairs=29 NimputedPairs=4
Key: Oxbridge X&Y valid Y imputed
MRCP_PACES
F1_Preparedness

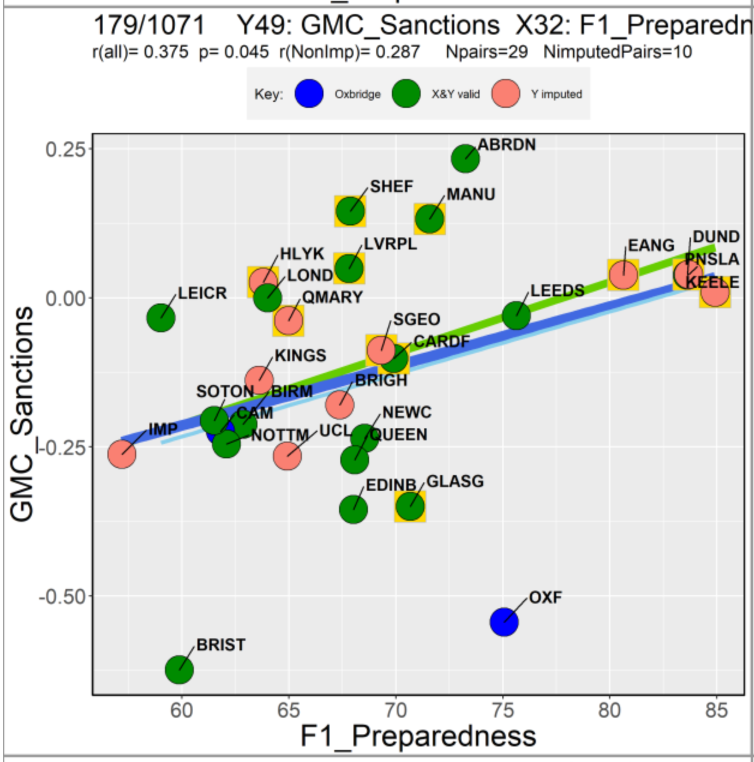
179/1071 Y49: GMC_Sanctions X32: F1_Preparedn
r(all)= 0.375 p= 0.045 r(NonImp)= 0.287 Npairs=29 NimputedPairs=10
Key: Oxbridge X&Y valid Y imputed
GMC_Sanctions
F1_Preparedness

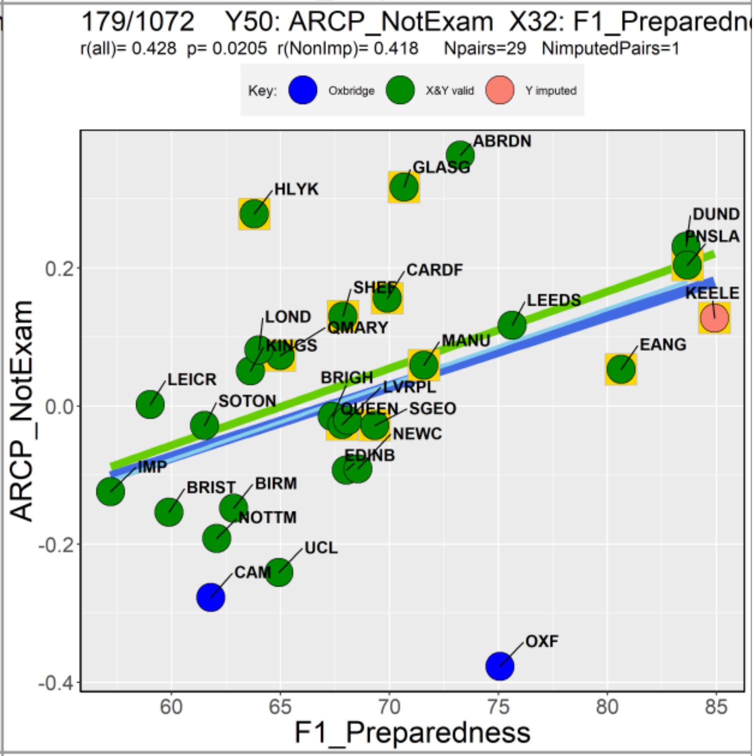
179/1072 Y50: ARCP_NotExam X32: F1_Preparedn
r(all)= 0.428 p= 0.0205 r(NonImp)= 0.418 Npairs=29 NimputedPairs=1
Key: Oxbridge X&Y valid Y imputed
ARCP_NotExam
F1_Preparedness

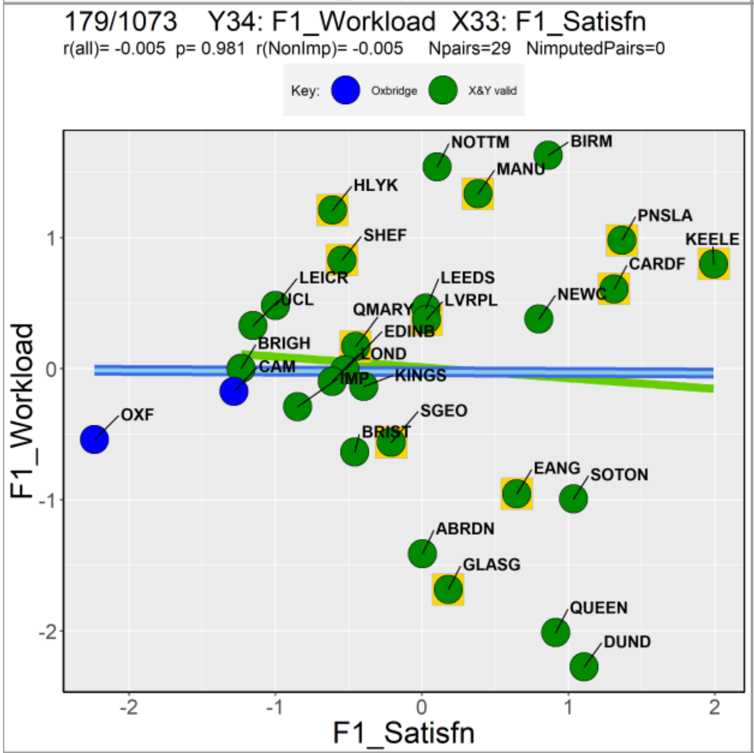
179/1073 Y34: F1_Workload X33: F1_Satisfn
r(all)= -0.005 p= 0.981 r(NonImp)= -0.005 Npairs=29 NimputedPairs=0
Key: Oxbridge X&Y valid
F1_Workload
F1_Satisfn

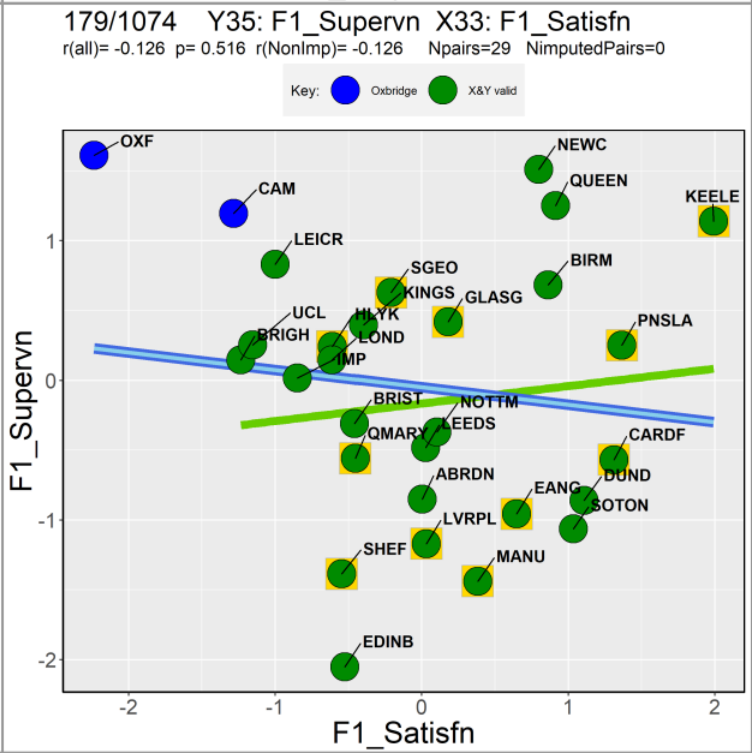
179/1074 Y35: F1_Supervn X33: F1_Satisfn
r(all)= -0.126 p= 0.516 r(NonImp)= -0.126 Npairs=29 NimputedPairs=0
Key: Oxbridge X&Y valid
F1_Supervn
F1_Satisfn

180/1075 Y36: Trainee_GP X33: F1_Satisfn
r(all)= 0.369 p= 0.0491 r(NonImp)= 0.369 Npairs=29 NimputedPairs=0

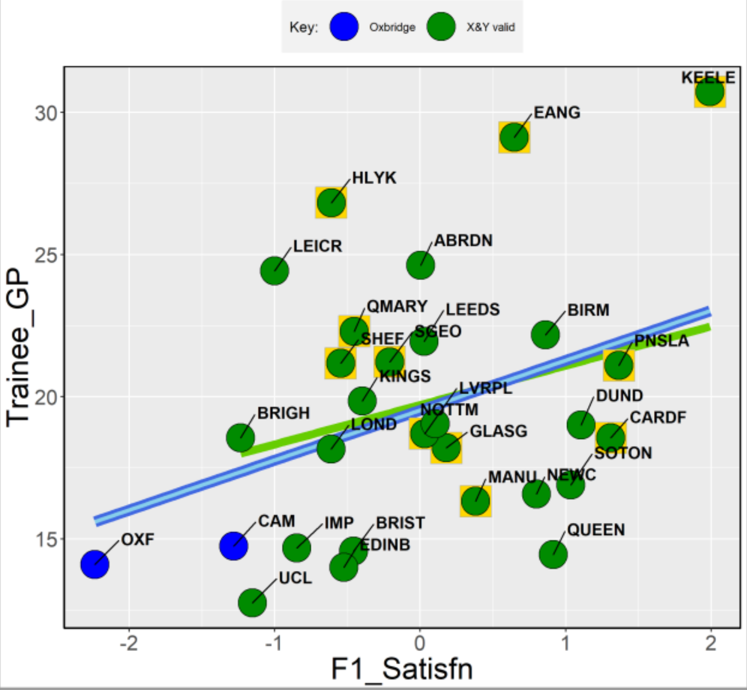

180/1076 Y37: Trainee_Psyc X33: F1_Satisfn
r(all)= 0.017 p= 0.929 r(NonImp)= 0.017 Npairs=29 NimputedPairs=0

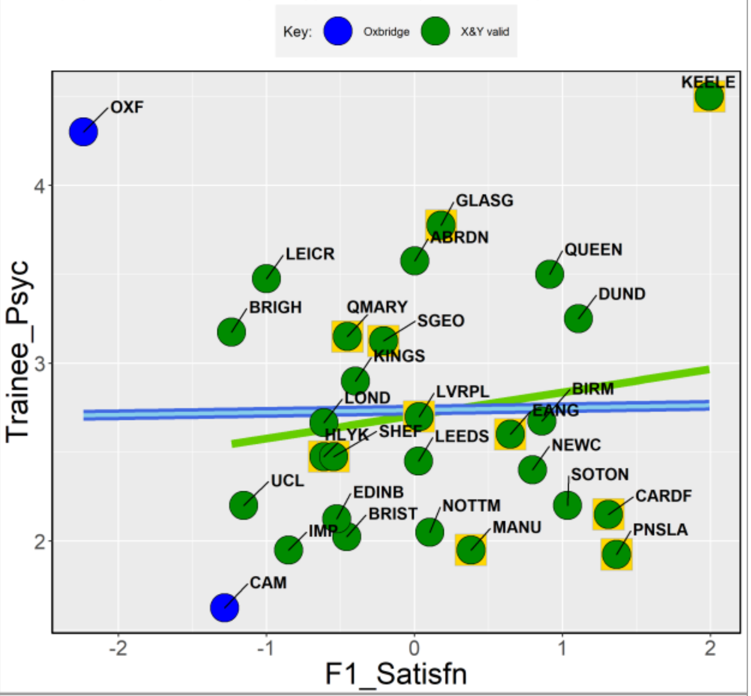

180/1077 Y38: TraineeApp_Surgery X33: F1_Satisfn
r(all)= 0.040 p= 0.838 r(NonImp)= 0.056 Npairs=29 NimputedPairs=2

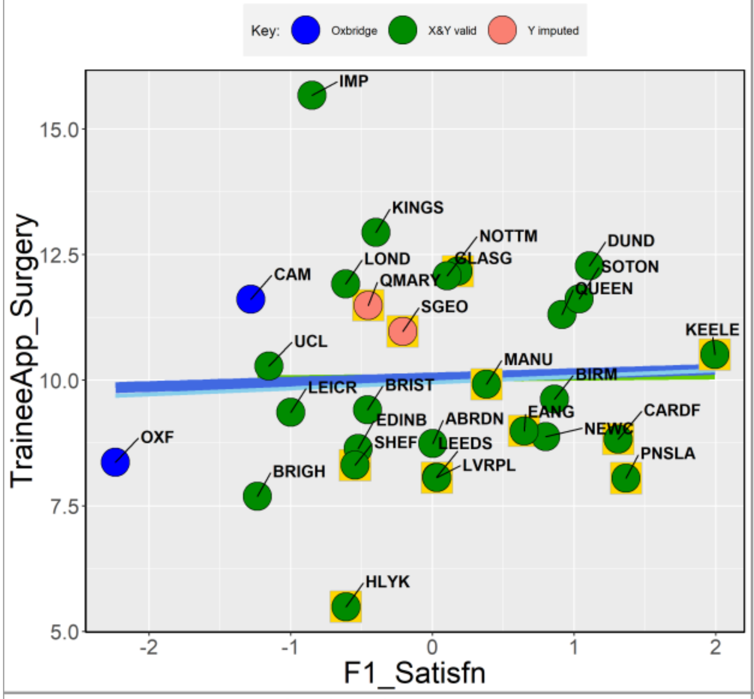

180/1078 Y39: TraineeApp_Anaes X33: F1_Satisfn
r(all)= 0.214 p= 0.265 r(NonImp)= 0.214 Npairs=29 NimputedPairs=0

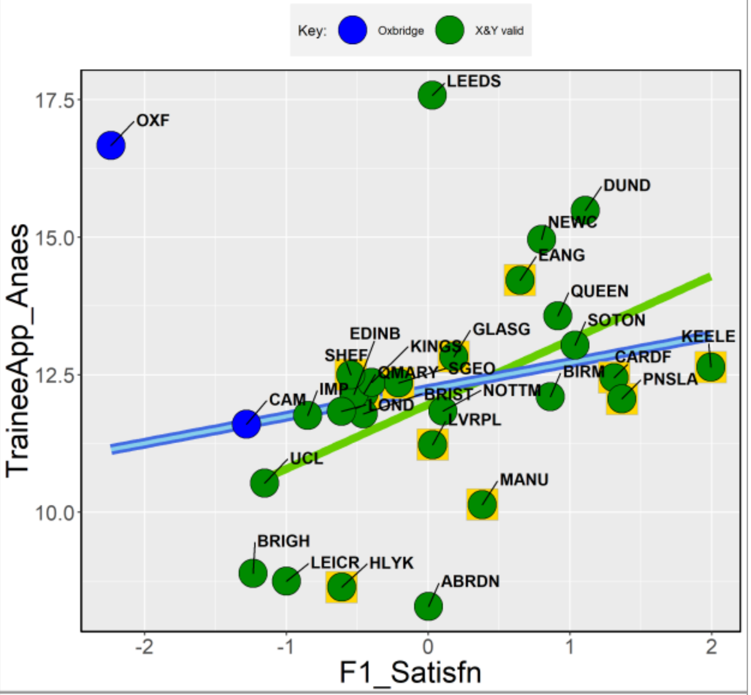

180/1079 Y40: GMC_PGexams X33: F1_Satisfn
r(all)= -0.555 p= 0.00179 r(NonImp)= -0.555 Npairs=29 NimputedPairs=0

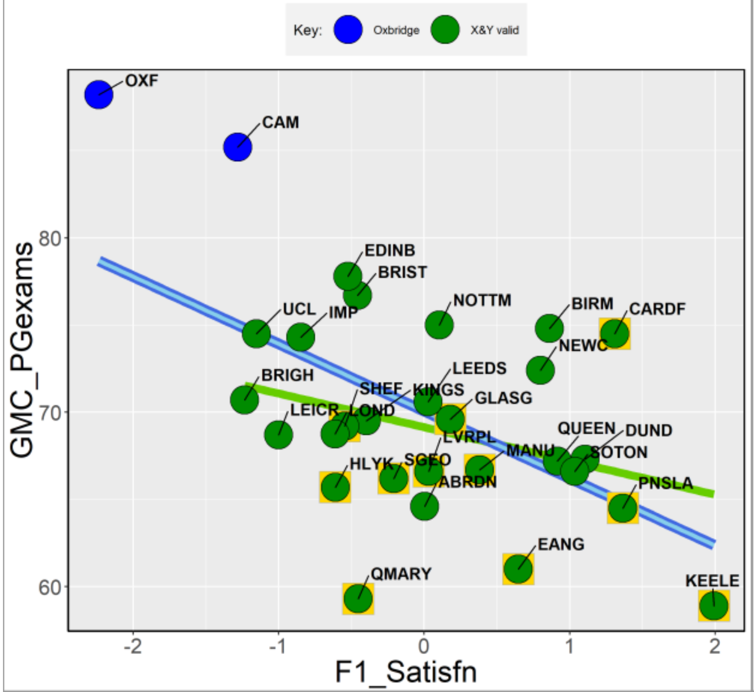

180/1080 Y41: MRCGP_AKT X33: F1_Satisfn
r(all)= -0.456 p= 0.0128 r(NonImp)= -0.456 Npairs=29 NimputedPairs=0

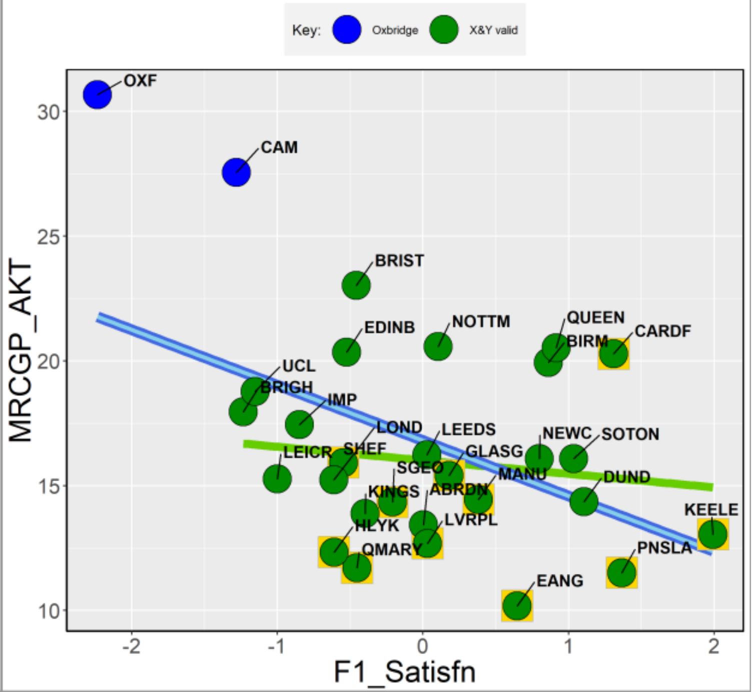

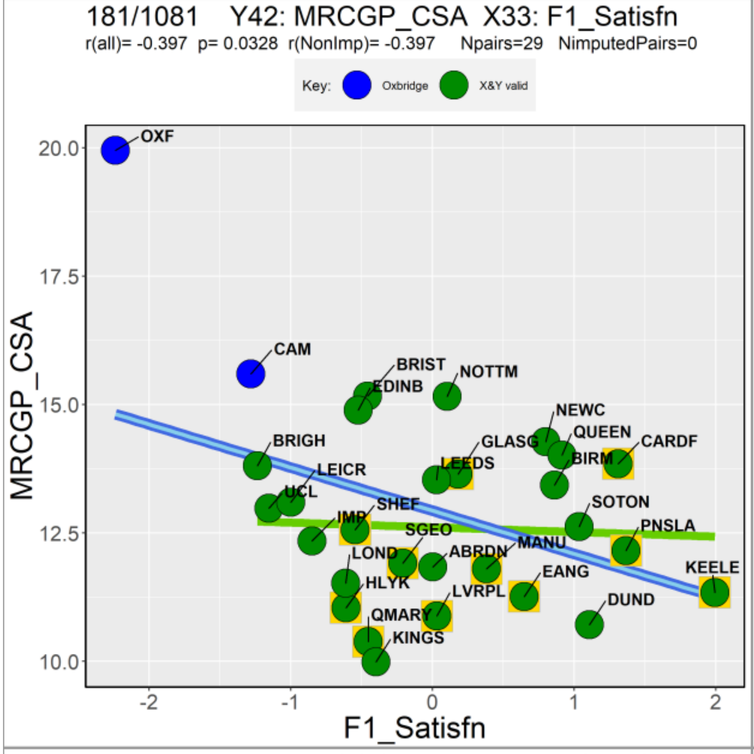
181/1081 Y42: MRCGP_CSA X33: F1_Satisfn
r(all)= -0.397 p= 0.0328 r(NonImp)= -0.397 Npairs=29 NimputedPairs=0
Key: Oxbridge X&Y valid
MRCGP_CSA
F1_Satisfn

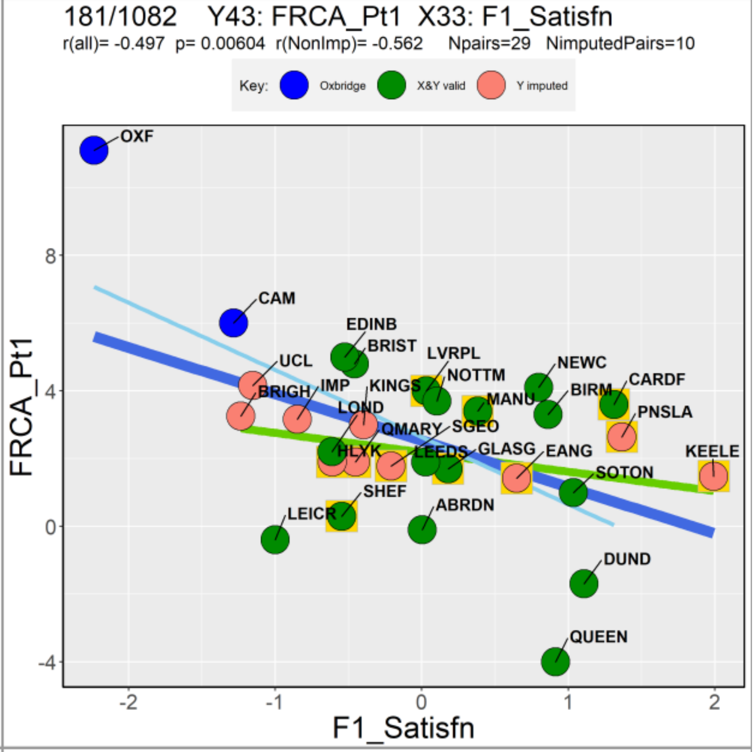
181/1082 Y43: FRCA_Pt1 X33: F1_Satisfn
r(all)= -0.497 p= 0.00604 r(NonImp)= -0.562 Npairs=29 NimputedPairs=10
Key: Oxbridge X&Y valid Y imputed
FRCA_Pt1
F1_Satisfn

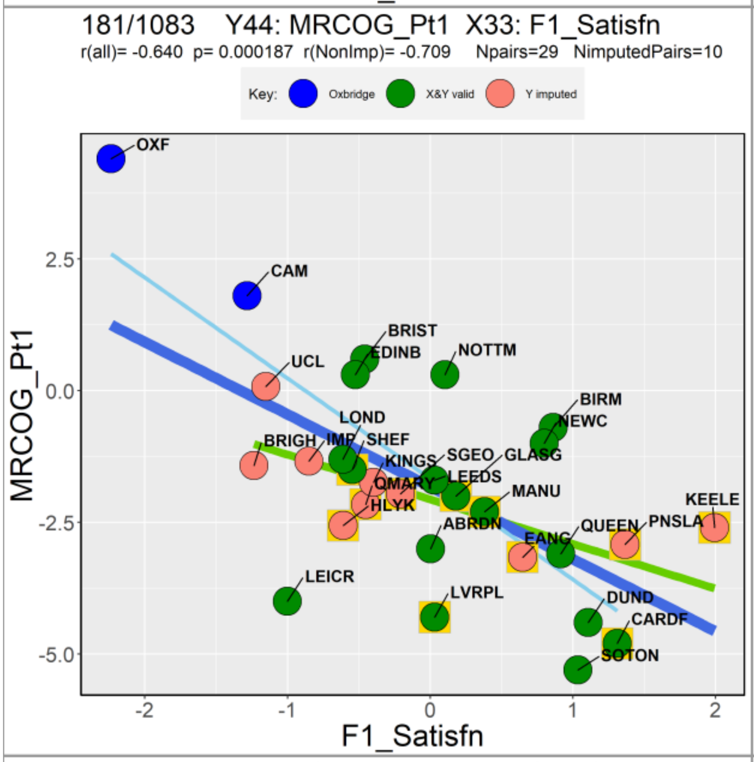
181/1083 Y44: MRCOG_Pt1 X33: F1_Satisfn
r(all)= -0.640 p= 0.000187 r(NonImp)= -0.709 Npairs=29 NimputedPairs=10
Key: Oxbridge X&Y valid Y imputed
MRCOG_Pt1
F1_Satisfn

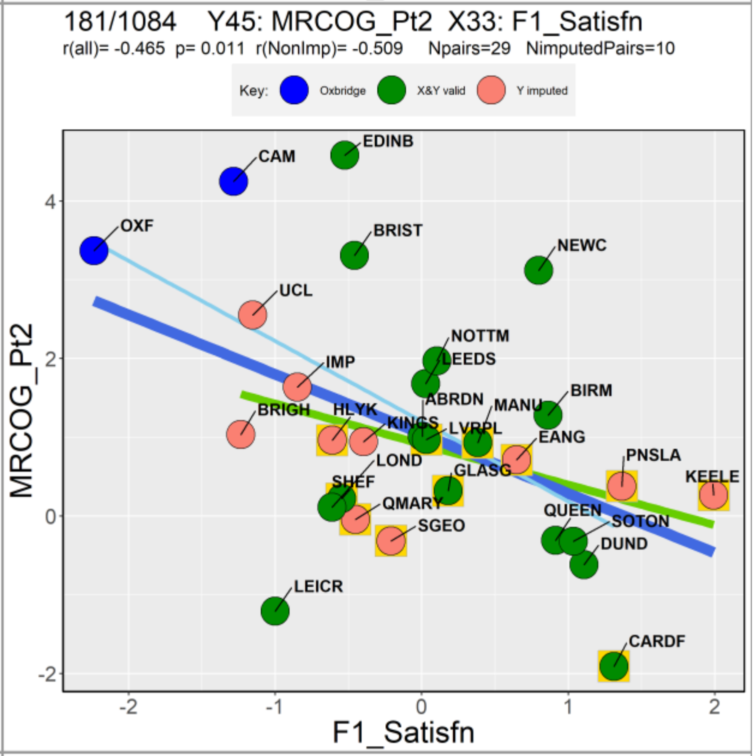
181/1084 Y45: MRCOG_Pt2 X33: F1_Satisfn
r(all)= -0.465 p= 0.011 r(NonImp)= -0.509 Npairs=29 NimputedPairs=10
Key: Oxbridge X&Y valid Y imputed
MRCOG_Pt2
F1_Satisfn

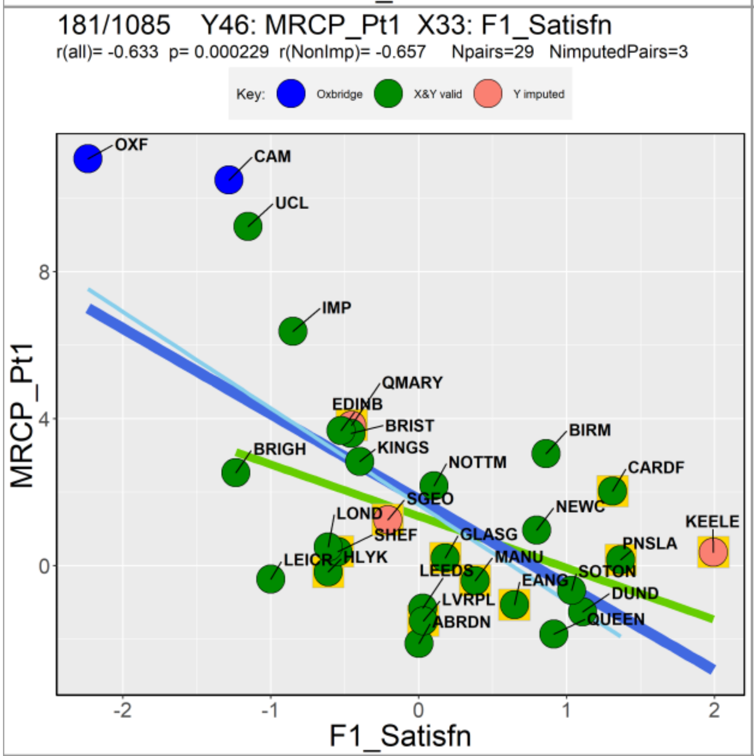
181/1085 Y46: MRCP_Pt1 X33: F1_Satisfn
r(all)= -0.633 p= 0.000229 r(NonImp)= -0.657 Npairs=29 NimputedPairs=3
Key: Oxbridge X&Y valid Y imputed
MRCP_Pt1
F1_Satisfn

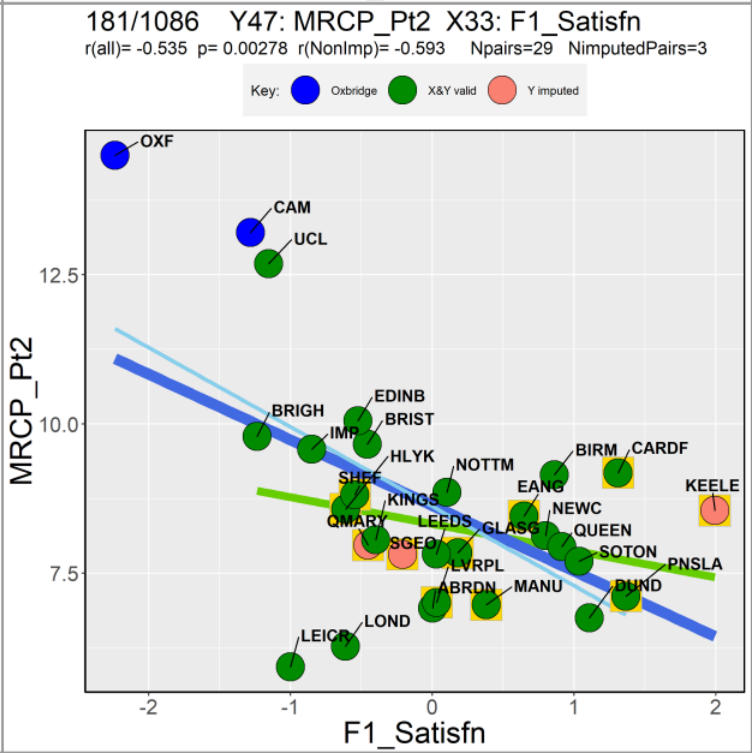
181/1086 Y47: MRCP_Pt2 X33: F1_Satisfn
r(all)= -0.535 p= 0.00278 r(NonImp)= -0.593 Npairs=29 NimputedPairs=3
Key: Oxbridge X&Y valid Y imputed
MRCP_Pt2
F1_Satisfn

182/1087 Y48: MRCP_PACES X33: F1_Satisfn

r(all)= -0.478 p= 0.00881 r(NonImp)= -0.480 Npairs=29 NimputedPairs=4

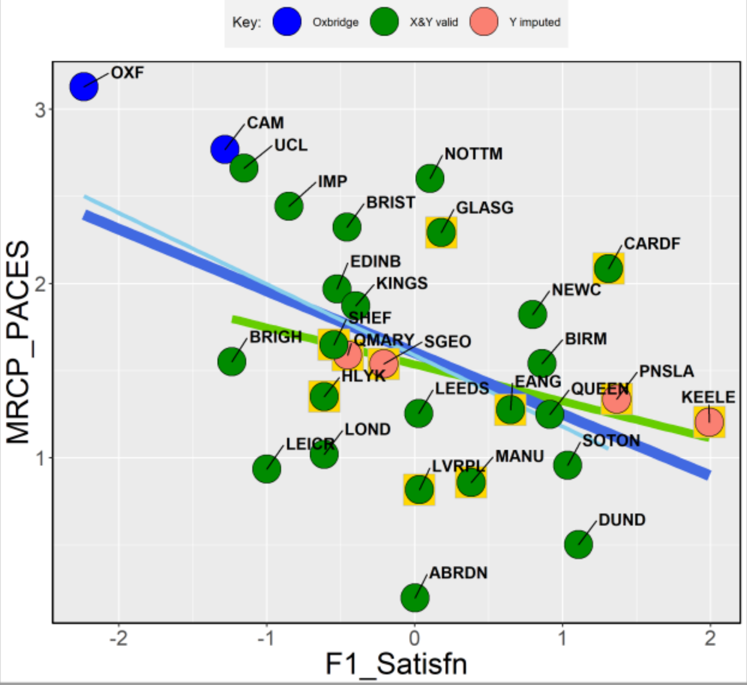

182/1088 Y49: GMC_Sanctions X33: F1_Satisfn

r(all)= 0.335 p= 0.0753 r(NonImp)= 0.259 Npairs=29 NimputedPairs=10

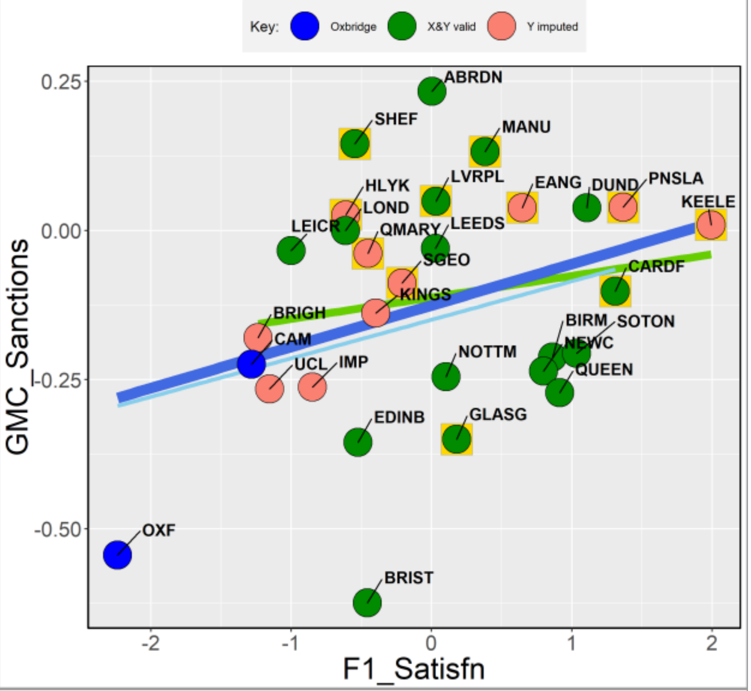

182/1089 Y50: ARCP_NotExam X33: F1_Satisfn

r(all)= 0.467 p= 0.0106 r(NonImp)= 0.460 Npairs=29 NimputedPairs=1

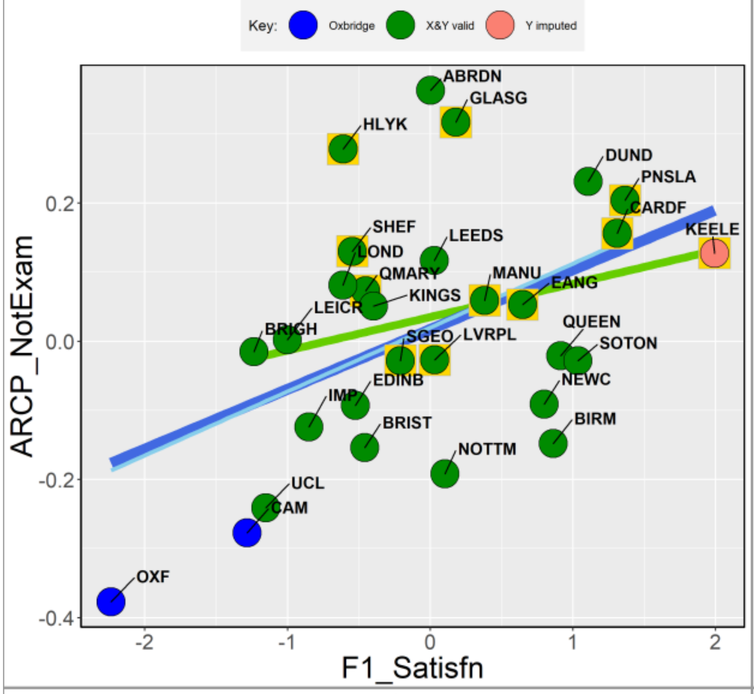

182/1090 Y35: F1_Supervn X34: F1_Workload

r(all)= -0.020 p= 0.918 r(NonImp)= -0.020 Npairs=29 NimputedPairs=0

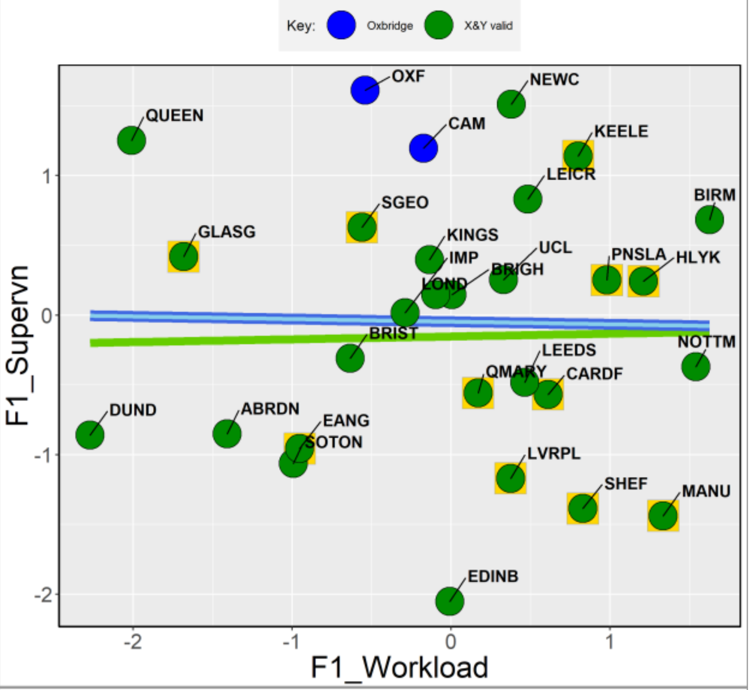

182/1091 Y36: Trainee_GP X34: F1_Workload

r(all)= 0.208 p= 0.278 r(NonImp)= 0.208 Npairs=29 NimputedPairs=0

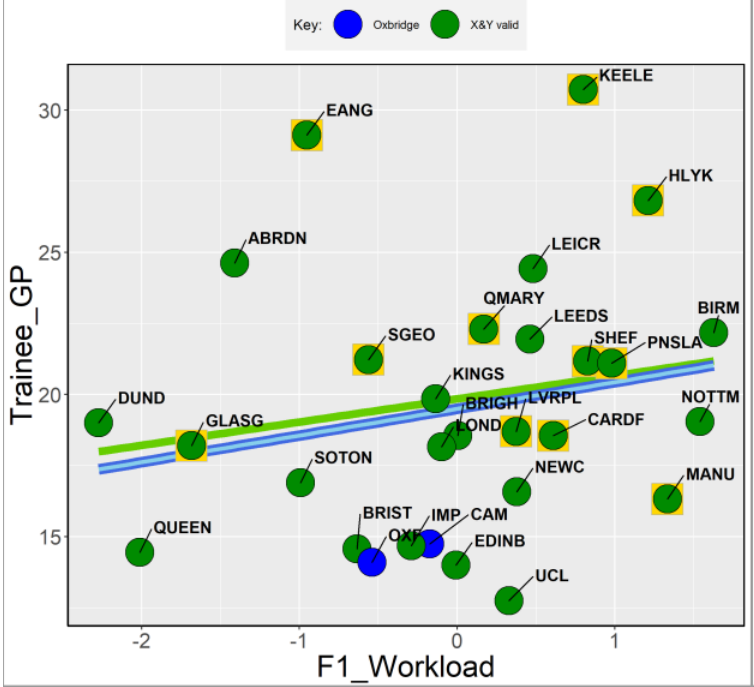

182/1092 Y37: Trainee_Psyc X34: F1_Workload

r(all)= -0.369 p= 0.0491 r(NonImp)= -0.369 Npairs=29 NimputedPairs=0

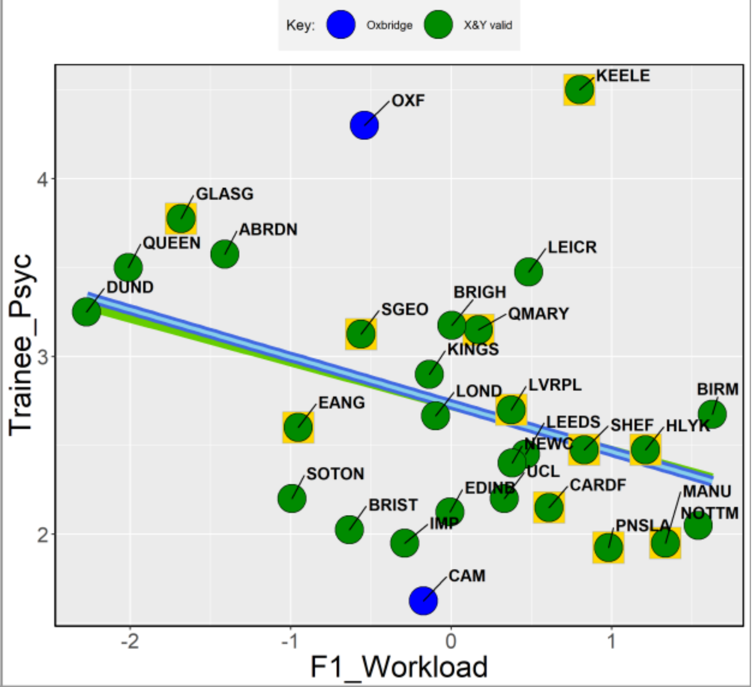

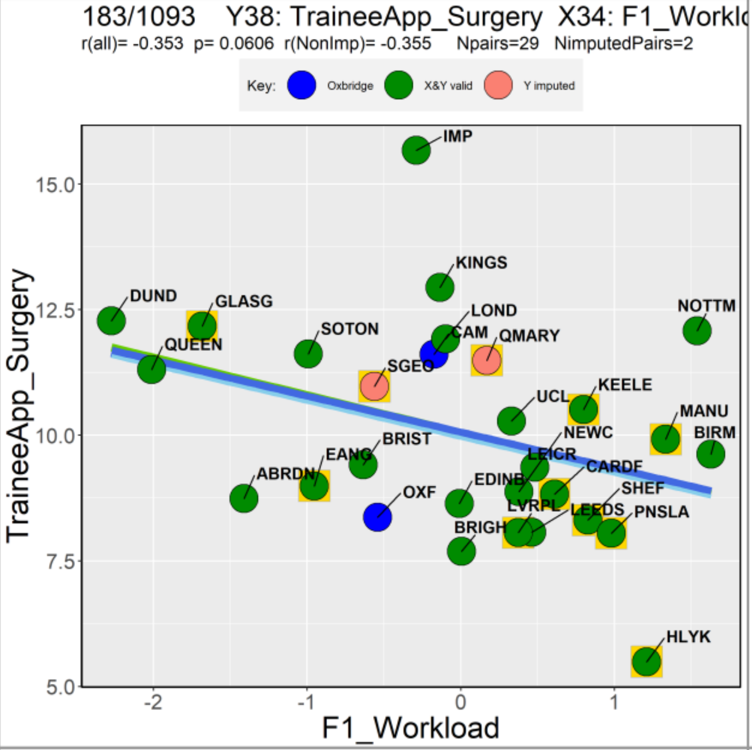
183/1093 Y38: TraineeApp_Surgery X34: F1_Workl
r(all)= -0.353 p= 0.0606 r(NonImp)= -0.355 Npairs=29 NimputedPairs=2
Key: Oxbridge X&Y valid Y imputed
TraineeApp_Surgery
F1_Workload

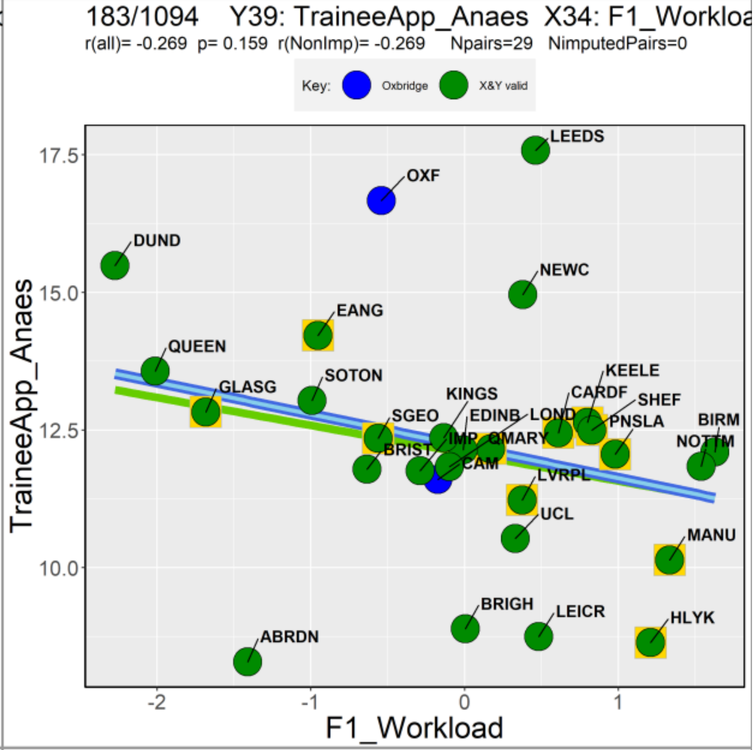
183/1094 Y39: TraineeApp_Anaes X34: F1_Workloa
r(all)= -0.269 p= 0.159 r(NonImp)= -0.269 Npairs=29 NimputedPairs=0
Key: Oxbridge X&Y valid
TraineeApp_Anaes
F1_Workload

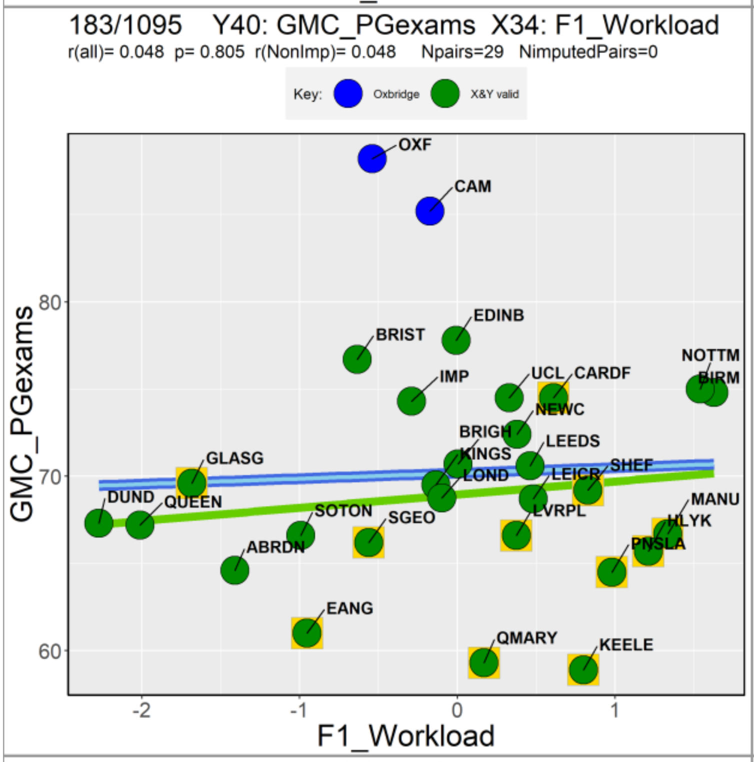
183/1095 Y40: GMC_PGexams X34: F1_Workload
r(all)= 0.048 p= 0.805 r(NonImp)= 0.048 Npairs=29 NimputedPairs=0
Key: Oxbridge X&Y valid
GMC_PGexams
F1_Workload

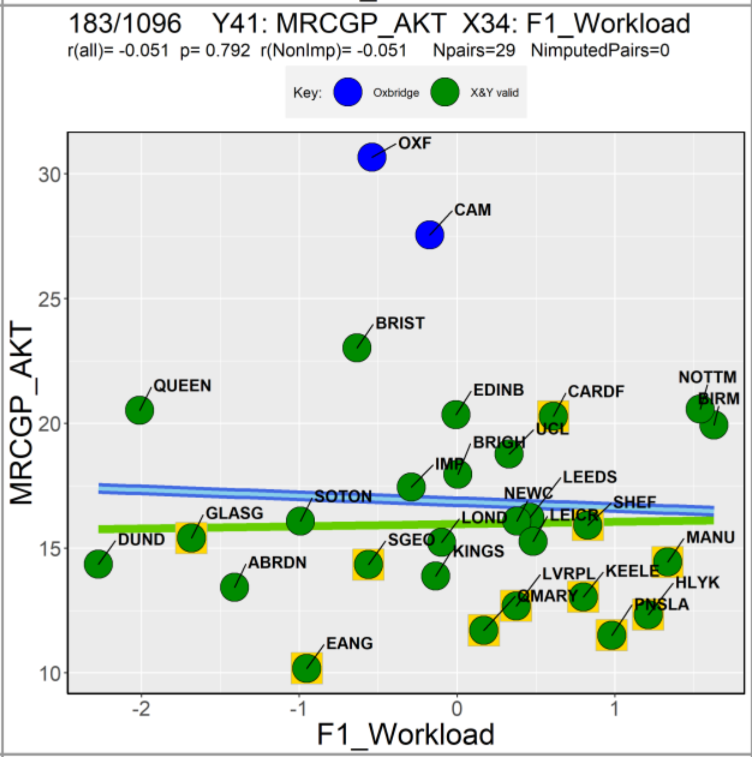
183/1096 Y41: MRCGP_AKT X34: F1_Workload
r(all)= -0.051 p= 0.792 r(NonImp)= -0.051 Npairs=29 NimputedPairs=0
Key: Oxbridge X&Y valid
MRCGP_AKT
F1_Workload

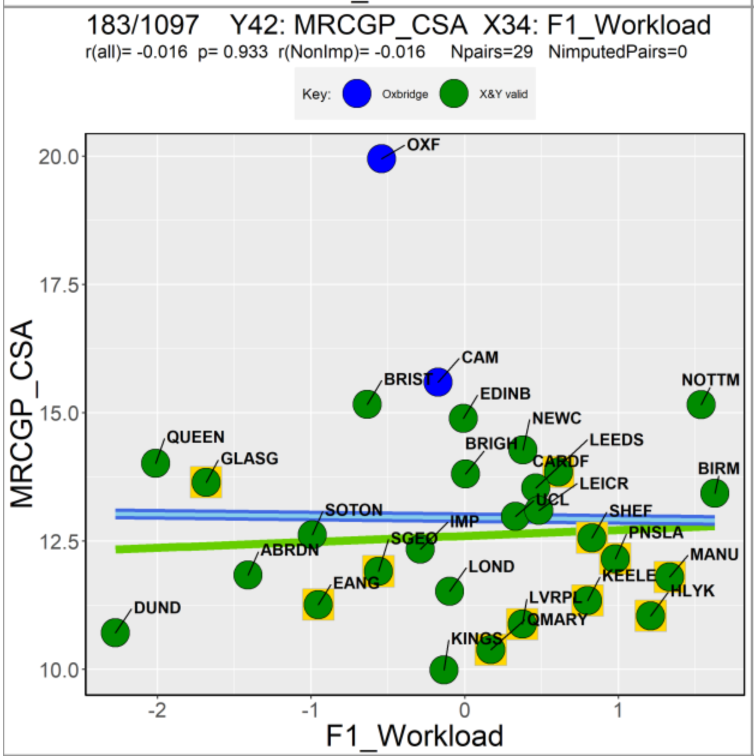
183/1097 Y42: MRCGP_CSA X34: F1_Workload
r(all)= -0.016 p= 0.933 r(NonImp)= -0.016 Npairs=29 NimputedPairs=0
Key: Oxbridge X&Y valid
MRCGP_CSA
F1_Workload

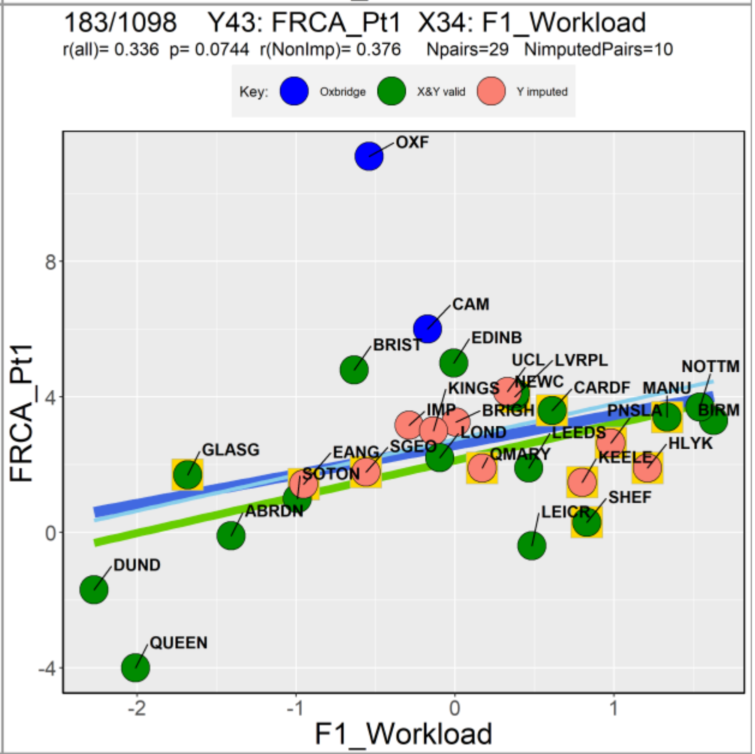
183/1098 Y43: FRCA_Pt1 X34: F1_Workload
r(all)= 0.336 p= 0.0744 r(NonImp)= 0.376 Npairs=29 NimputedPairs=10
Key: Oxbridge X&Y valid Y imputed
FRCA_Pt1
F1_Workload

184/1099 Y44: MRCOG_Pt1 X34: F1_Workload
r(all)= 0.149 p= 0.44 r(NonImp)= 0.191 Npairs=29 NimputedPairs=10

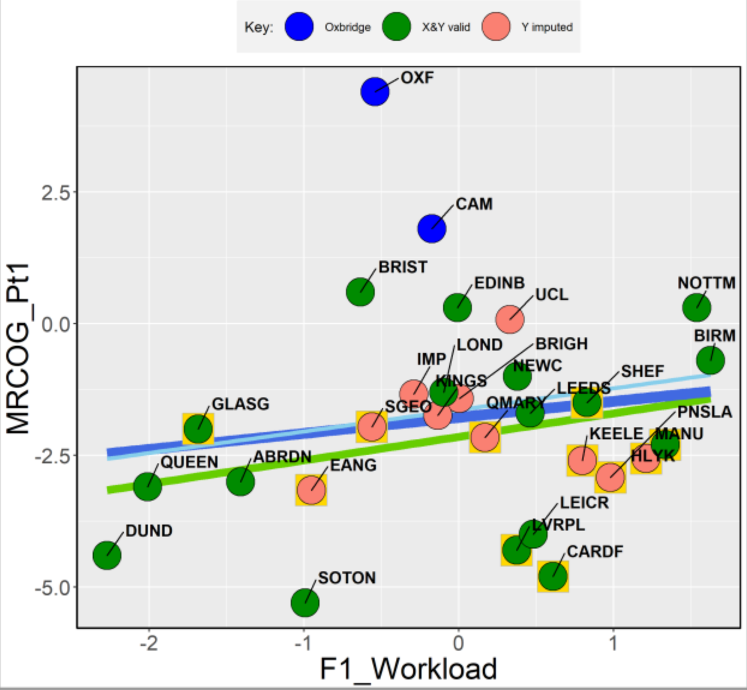

184/1100 Y45: MRCOG_Pt2 X34: F1_Workload
r(all)= 0.112 p= 0.564 r(NonImp)= 0.141 Npairs=29 NimputedPairs=10

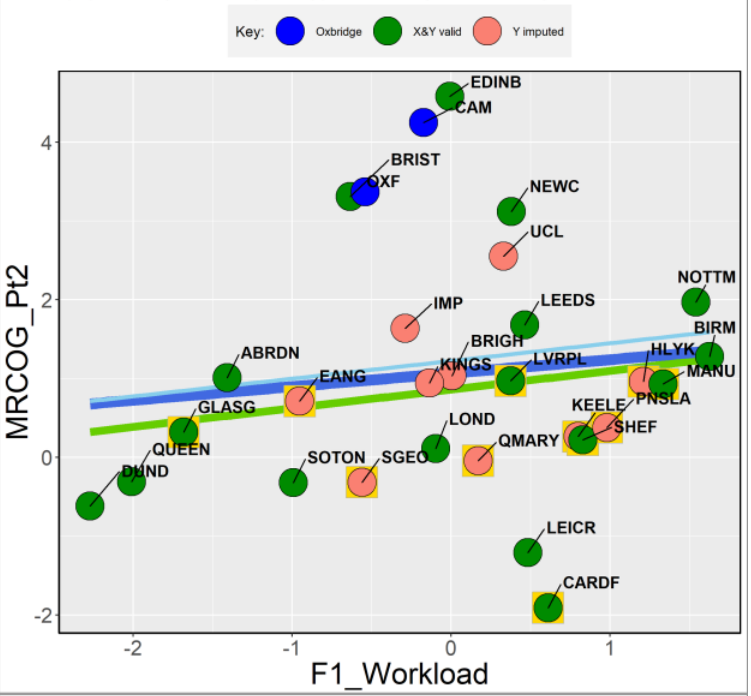

184/1101 Y46: MRCP_Pt1 X34: F1_Workload
r(all)= 0.113 p= 0.559 r(NonImp)= 0.122 Npairs=29 NimputedPairs=3

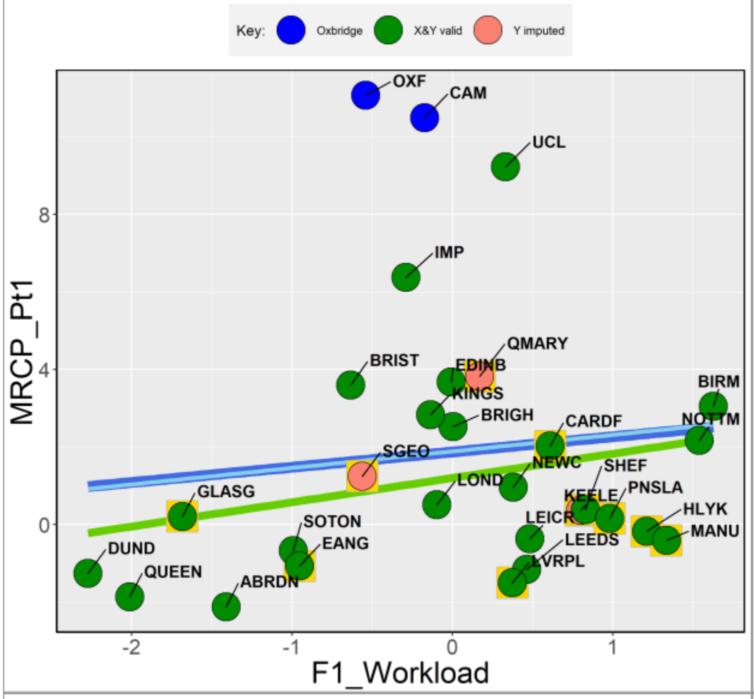

184/1102 Y47: MRCP_Pt2 X34: F1_Workload
r(all)= 0.053 p= 0.785 r(NonImp)= 0.051 Npairs=29 NimputedPairs=3

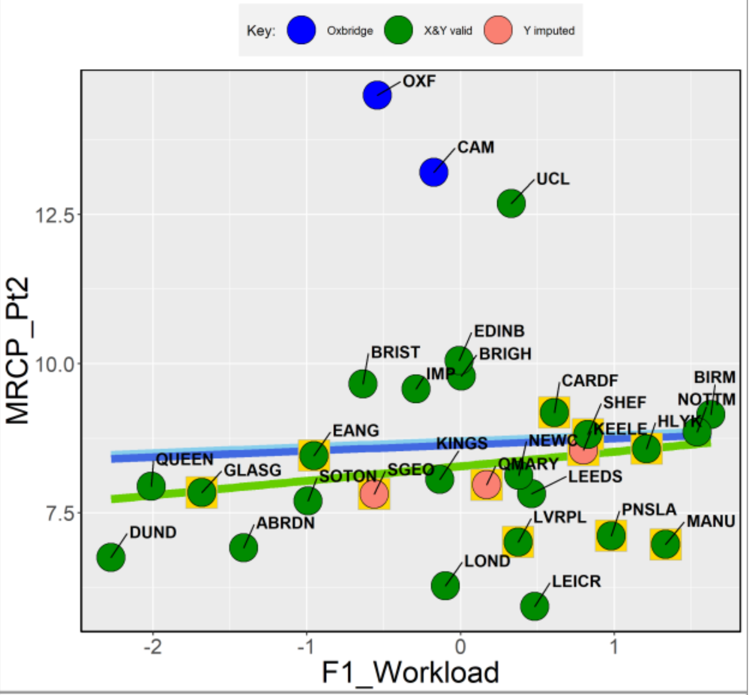

184/1103 Y48: MRCP_PACES X34: F1_Workload
r(all)= 0.142 p= 0.464 r(NonImp)= 0.181 Npairs=29 NimputedPairs=4

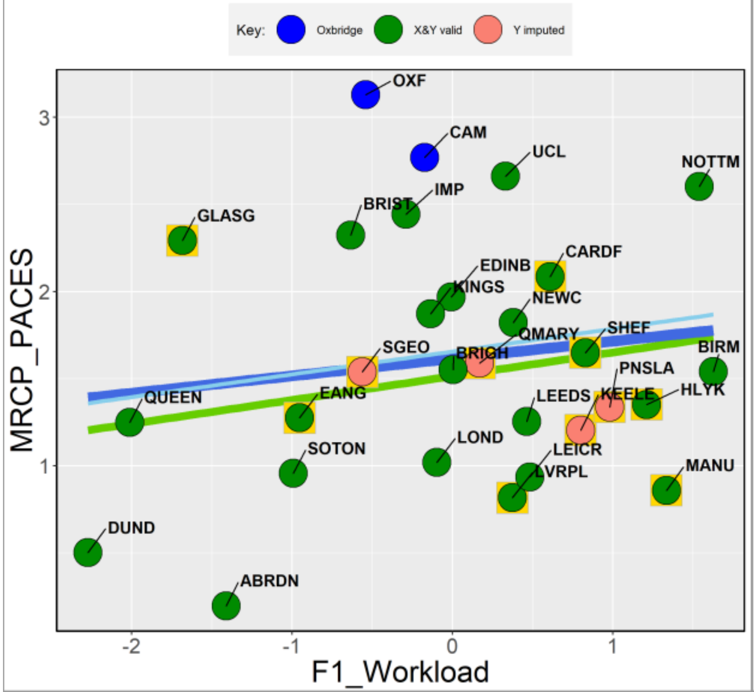

184/1104 Y49: GMC_Sanctions X34: F1_Workload
r(all)= 0.191 p= 0.321 r(NonImp)= 0.155 Npairs=29 NimputedPairs=10

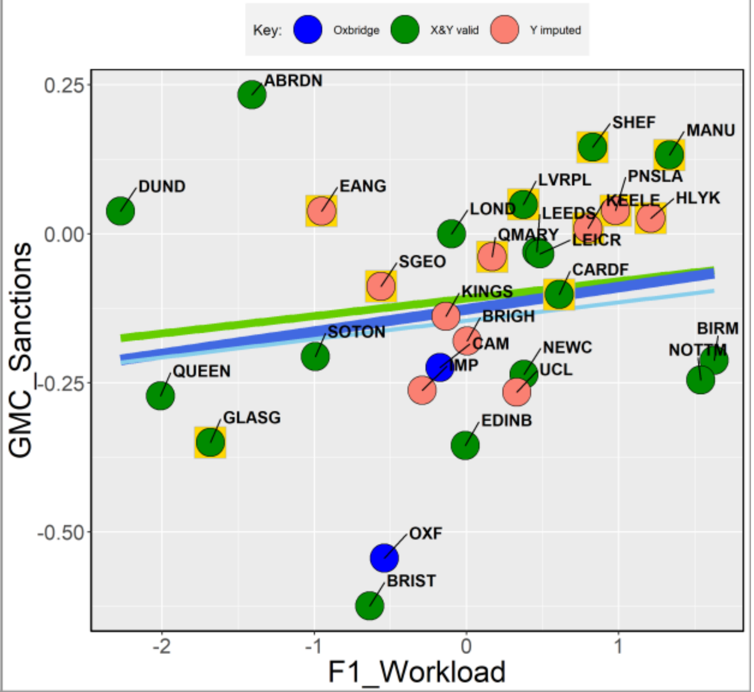

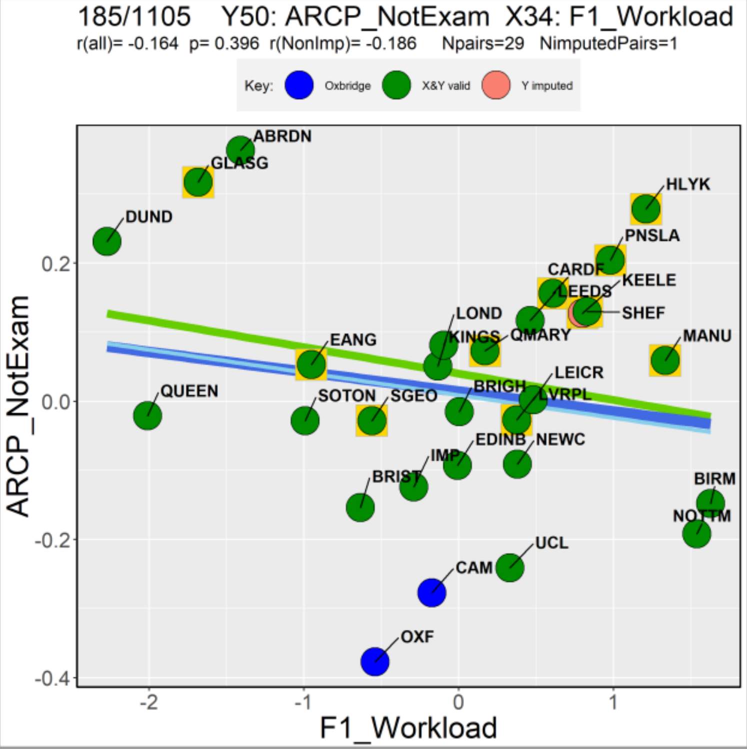
185/1105 Y50: ARCP_NotExam X34: F1_Workload
r(all)= -0.164 p= 0.396 r(NonImp)= -0.186 Npairs=29 NimputedPairs=1
Key: Oxbridge X&Y valid Y imputed
ARCP_NotExam
F1_Workload

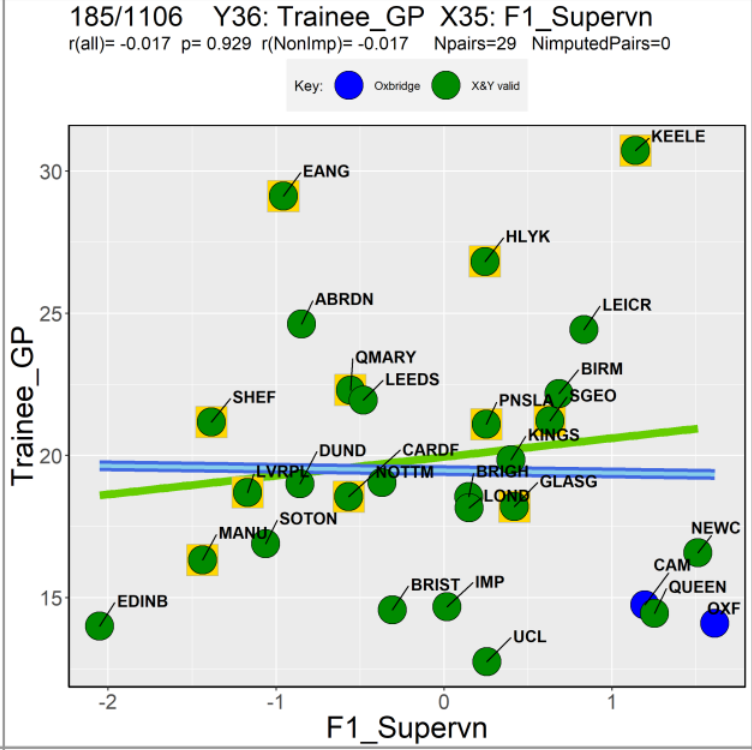
185/1106 Y36: Trainee_GP X35: F1_Supervn
r(all)= -0.017 p= 0.929 r(NonImp)= -0.017 Npairs=29 NimputedPairs=0
Key: Oxbridge X&Y valid
Trainee_GP
F1_Supervn

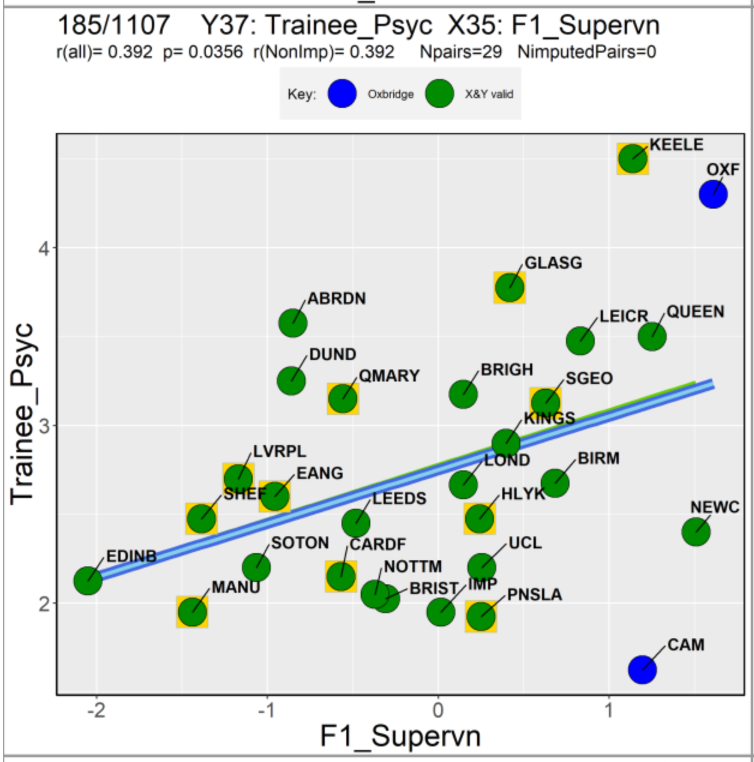
185/1107 Y37: Trainee_Psyc X35: F1_Supervn
r(all)= 0.392 p= 0.0356 r(NonImp)= 0.392 Npairs=29 NimputedPairs=0
Key: Oxbridge X&Y valid
Trainee_Psyc
F1_Supervn

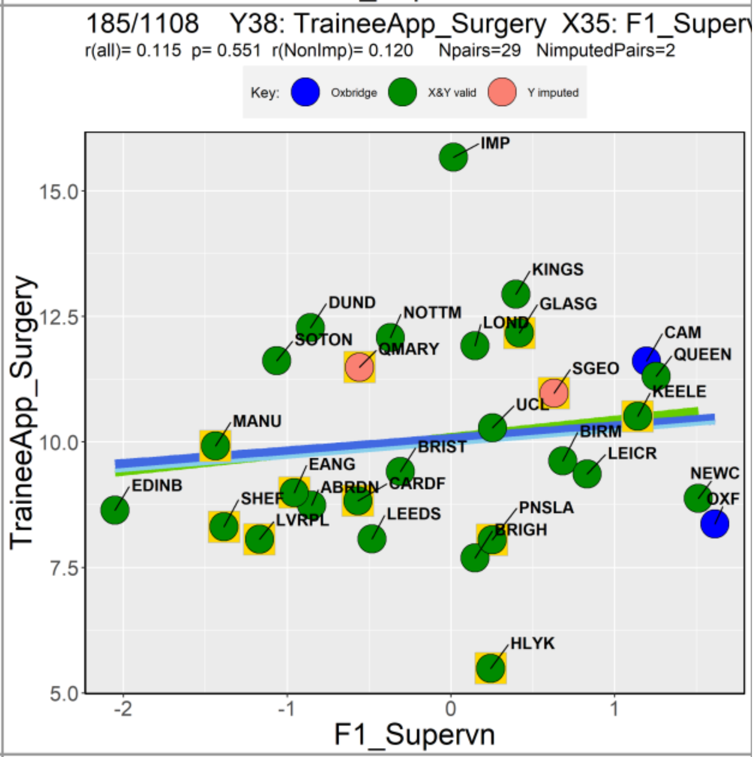
185/1108 Y38: TraineeApp_Surgery X35: F1_Superv
r(all)= 0.115 p= 0.551 r(NonImp)= 0.120 Npairs=29 NimputedPairs=2
Key: Oxbridge X&Y valid Y imputed
TraineeApp_Surgery
F1_Supervn

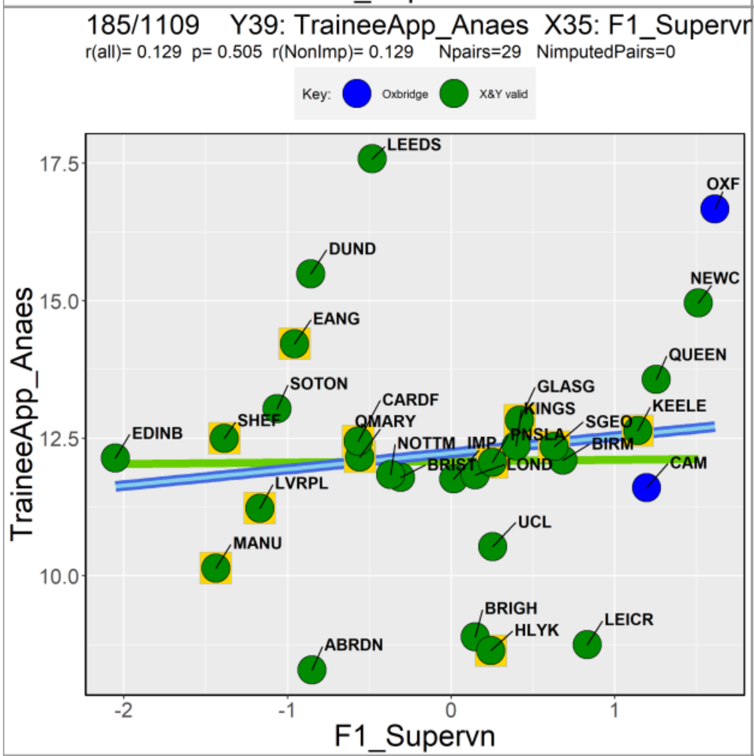
185/1109 Y39: TraineeApp_Anaes X35: F1_Supervn
r(all)= 0.129 p= 0.505 r(NonImp)= 0.129 Npairs=29 NimputedPairs=0
Key: Oxbridge X&Y valid
TraineeApp_Anaes
F1_Supervn

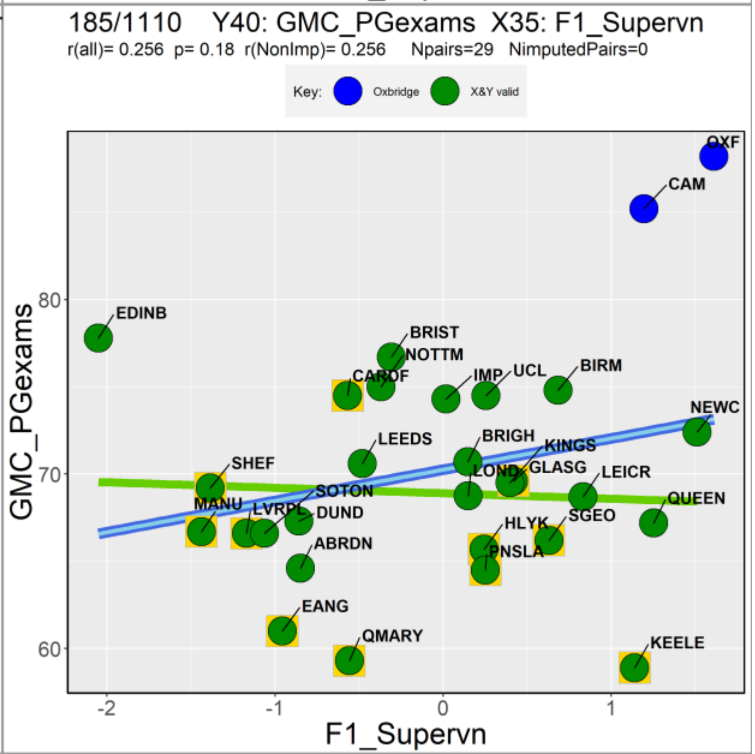
185/1110 Y40: GMC_PGexams X35: F1_Supervn
r(all)= 0.256 p= 0.18 r(NonImp)= 0.256 Npairs=29 NimputedPairs=0
Key: Oxbridge X&Y valid
GMC_PGexams
F1_Supervn

186/1111 Y41: MRCGP_AKT X35: F1_Supervn
r(all)= 0.339 p= 0.0718 r(NonImp)= 0.339 Npairs=29 NimputedPairs=0

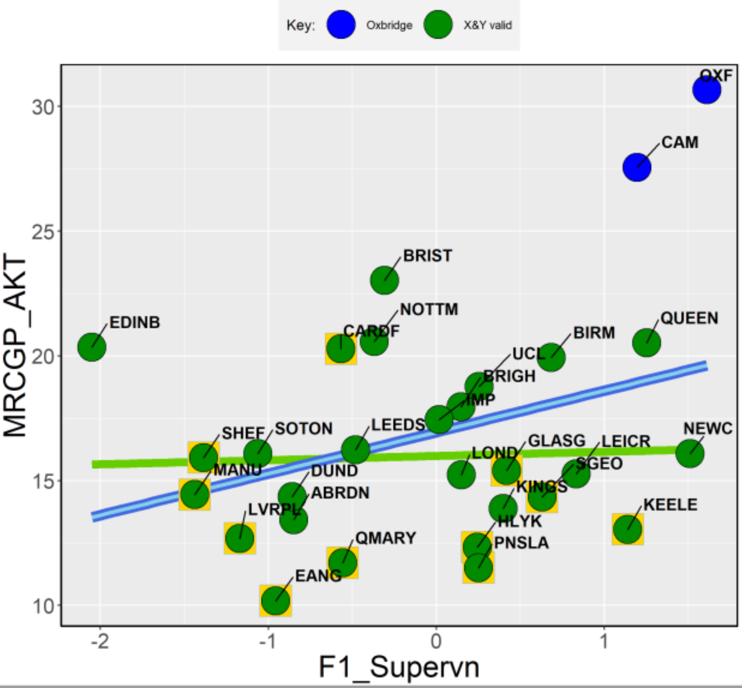

186/1112 Y42: MRCGP_CSA X35: F1_Supervn
r(all)= 0.345 p= 0.0666 r(NonImp)= 0.345 Npairs=29 NimputedPairs=0

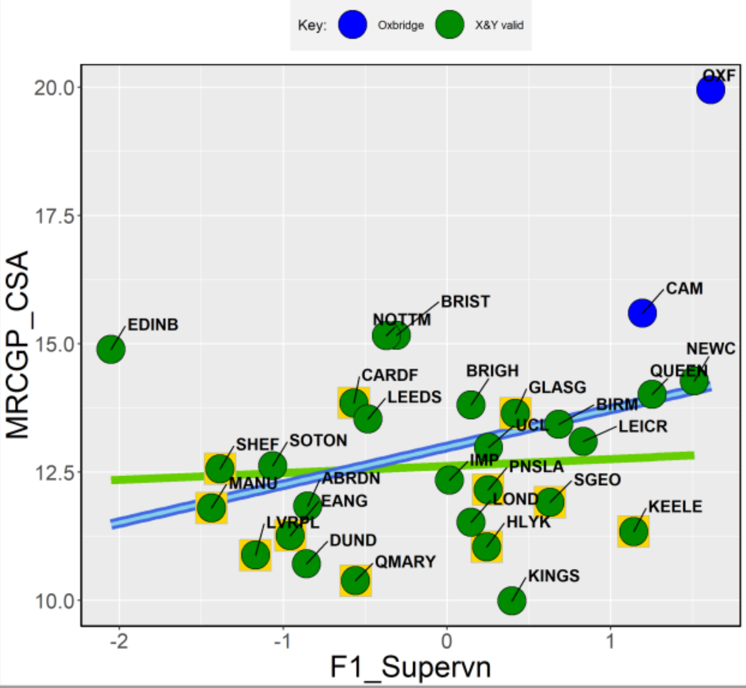

186/1113 Y43: FRCA_Pt1 X35: F1_Supervn
r(all)= 0.160 p= 0.406 r(NonImp)= 0.175 Npairs=29 NimputedPairs=10

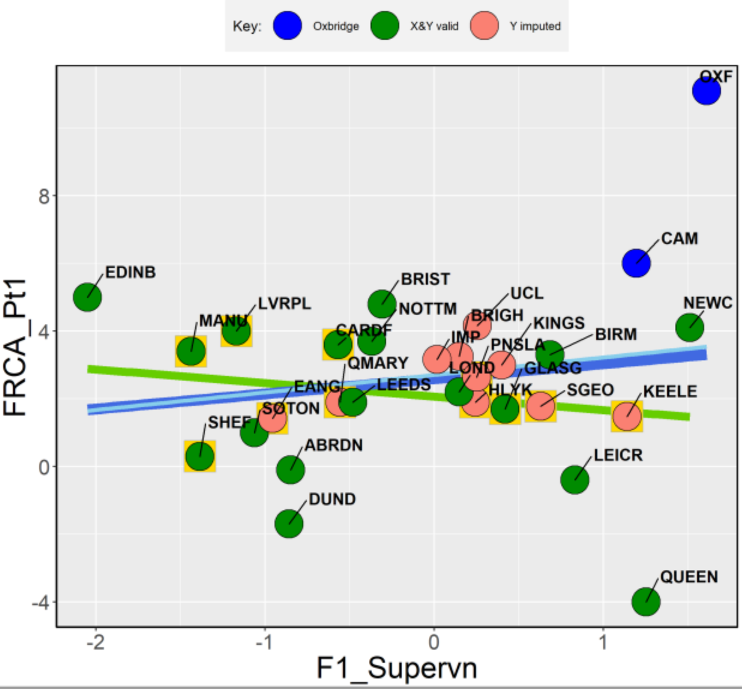

186/1114 Y44: MRCOG_Pt1 X35: F1_Supervn
r(all)= 0.353 p= 0.0602 r(NonImp)= 0.391 Npairs=29 NimputedPairs=10

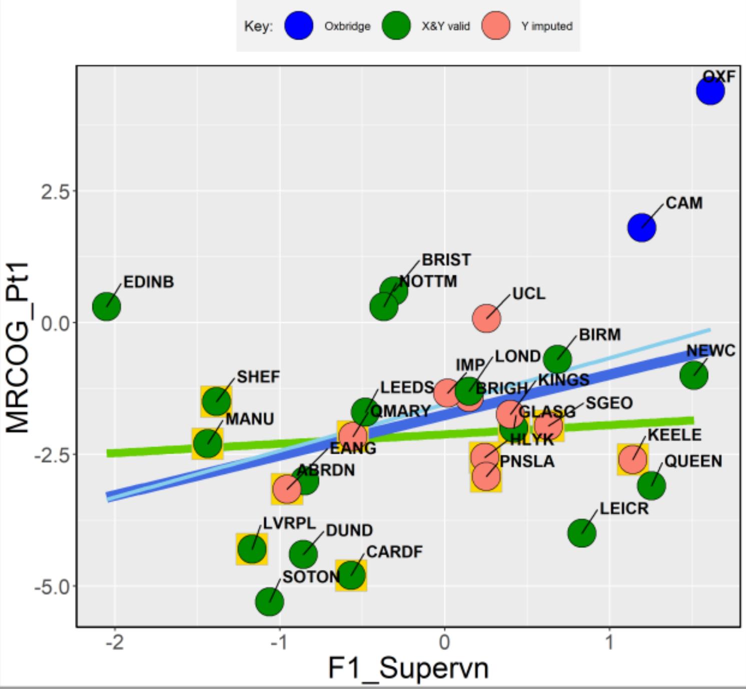

186/1115 Y45: MRCOG_Pt2 X35: F1_Supervn
r(all)= 0.087 p= 0.654 r(NonImp)= 0.129 Npairs=29 NimputedPairs=10

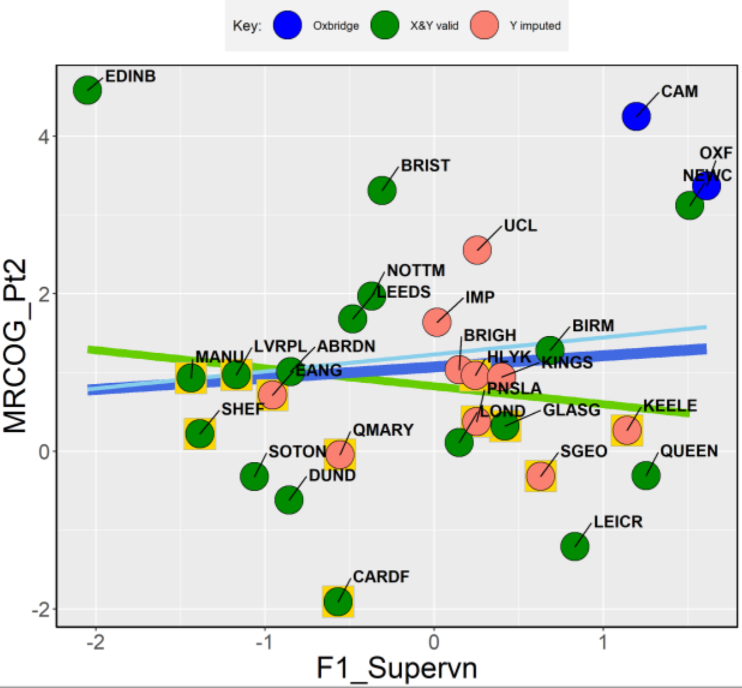

186/1116 Y46: MRCP_Pt1 X35: F1_Supervn
r(all)= 0.354 p= 0.0598 r(NonImp)= 0.410 Npairs=29 NimputedPairs=3

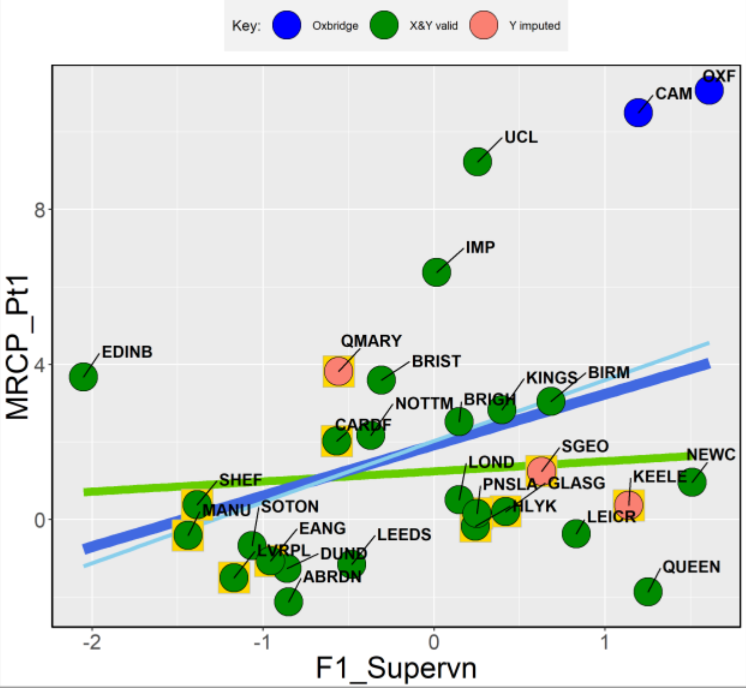

187/1117 Y47: MRCP_Pt2 X35: F1_Supervn
r(all)= 0.307 p= 0.105 r(NonImp)= 0.332 Npairs=29 NimputedPairs=3

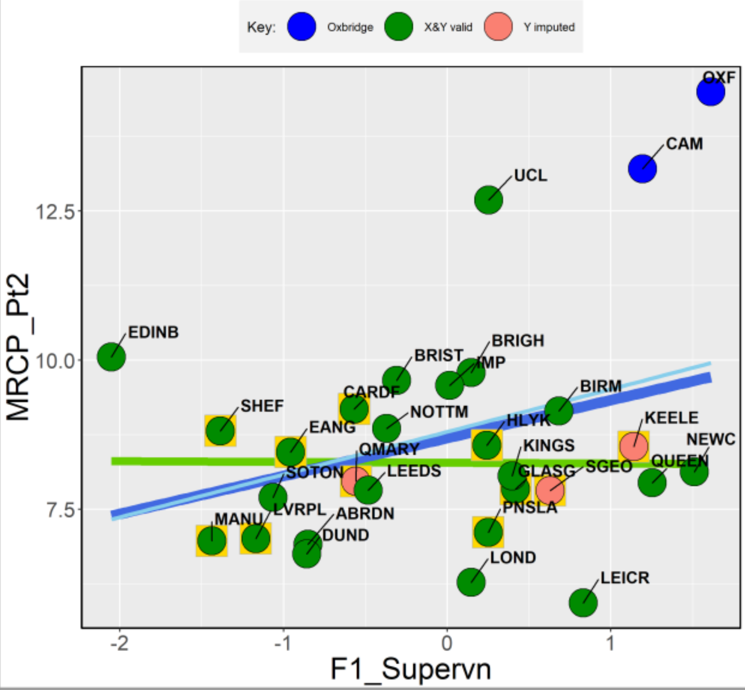

187/1118 Y48: MRCP_PACES X35: F1_Supervn
r(all)= 0.355 p= 0.0588 r(NonImp)= 0.413 Npairs=29 NimputedPairs=4

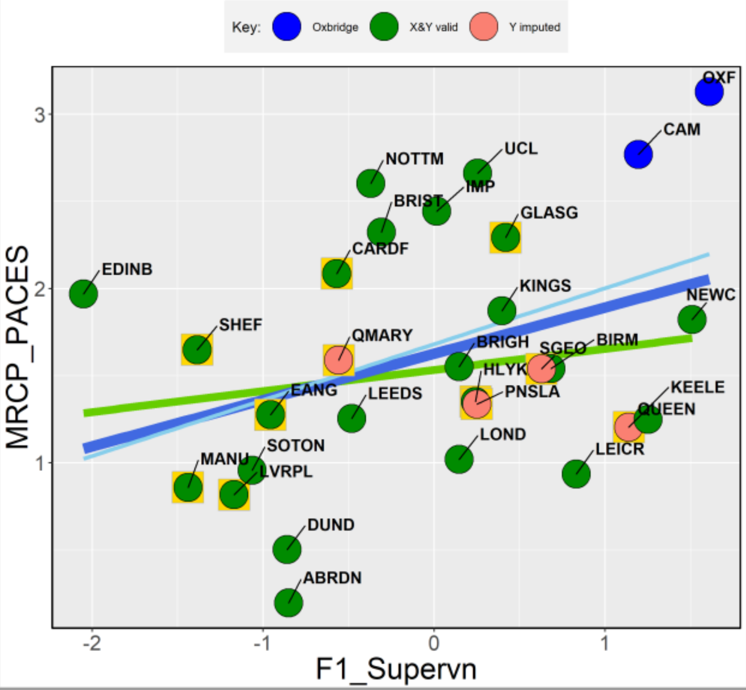

187/1119 Y49: GMC_Sanctions X35: F1_Supervn
r(all)= -0.360 p= 0.0553 r(NonImp)= -0.434 Npairs=29 NimputedPairs=10

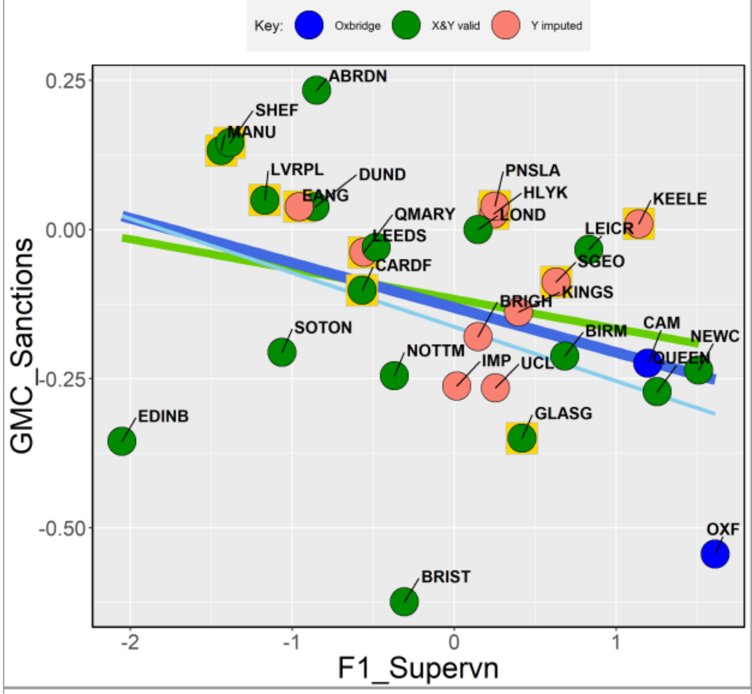

187/1120 Y50: ARCP_NotExam X35: F1_Supervn
r(all)= -0.311 p= 0.101 r(NonImp)= -0.353 Npairs=29 NimputedPairs=1

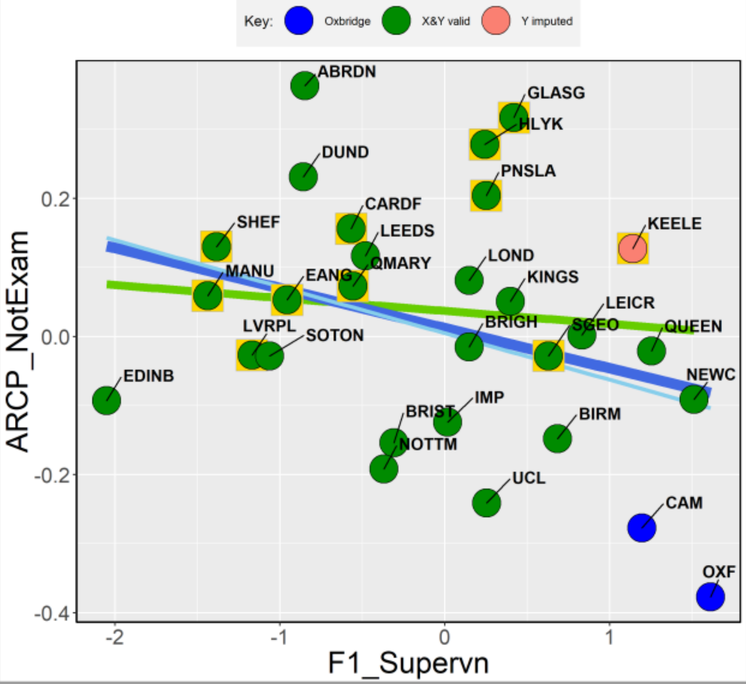

187/1121 Y37: Trainee_Psyc X36: Trainee_GP
r(all)= 0.372 p= 0.0468 r(NonImp)= 0.372 Npairs=29 NimputedPairs=0

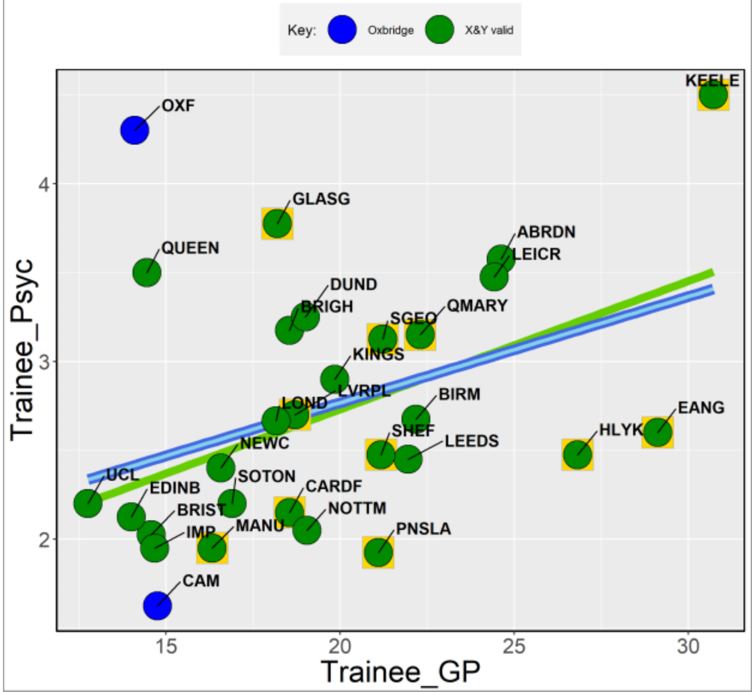

187/1122 Y38: TraineeApp_Surgery X36: Trainee_G

r(all)= -0.299 p= 0.115 r(NonImp)= -0.330 Npairs=29 NimputedPairs=2

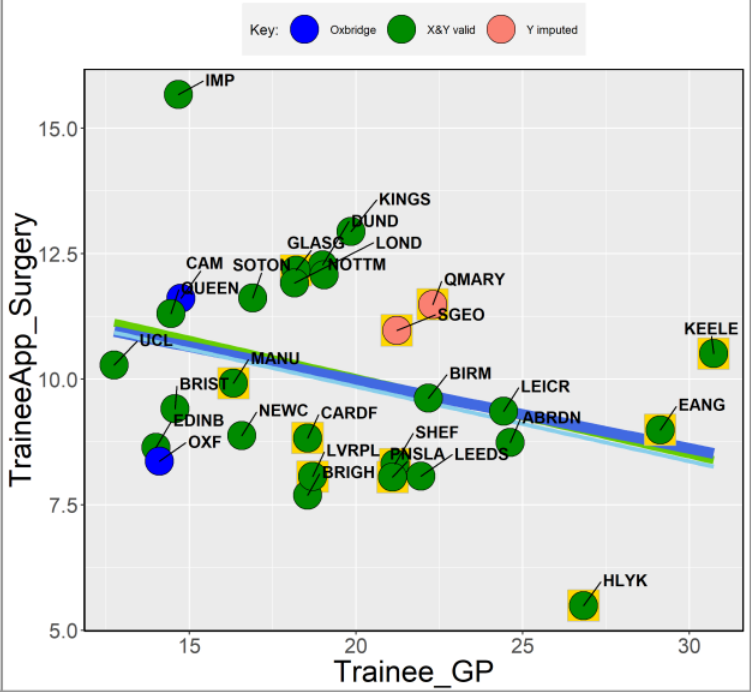

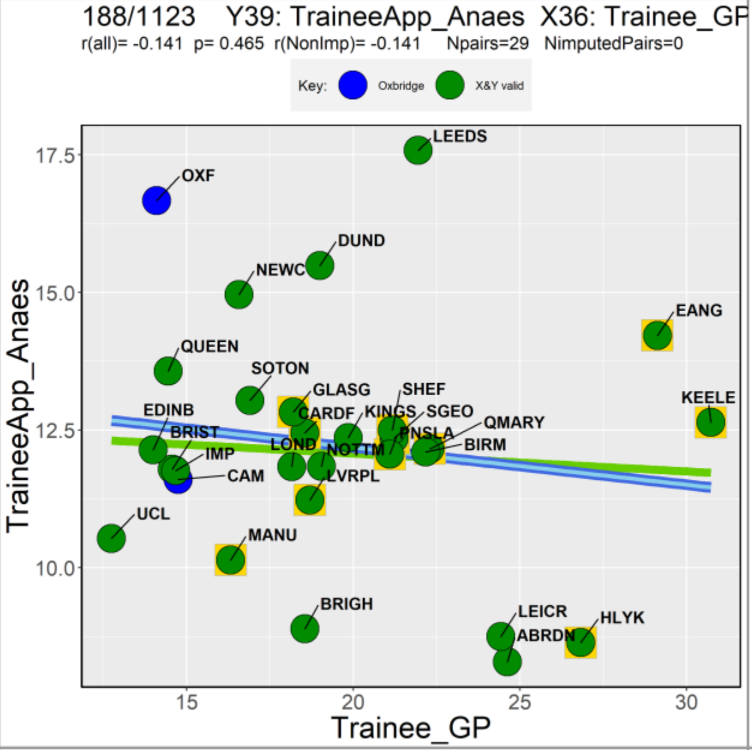
188/1123 Y39: TraineeApp_Anaes X36: Trainee_GP
r(all)= -0.141 p= 0.465 r(NonImp)= -0.141 Npairs=29 NimputedPairs=0
Key: Oxbridge X&Y valid
TraineeApp_Anaes
Trainee_GP
17.5
15.0
12.5
10.0
15
20
25
30
LEEDS
OXF
DUND
NEWC
EANG
QUEEN
SOTON
GLASG
SHEF
KEELE
EDINB
CARDF
KINGS
SGEO
BRIST
PNSLA
QMARY
LOND
NOTTM
BIRM
IMP
CAM
LVRPL
UCL
MANU
BRIGH
LEICR
HLYK
ABRDN
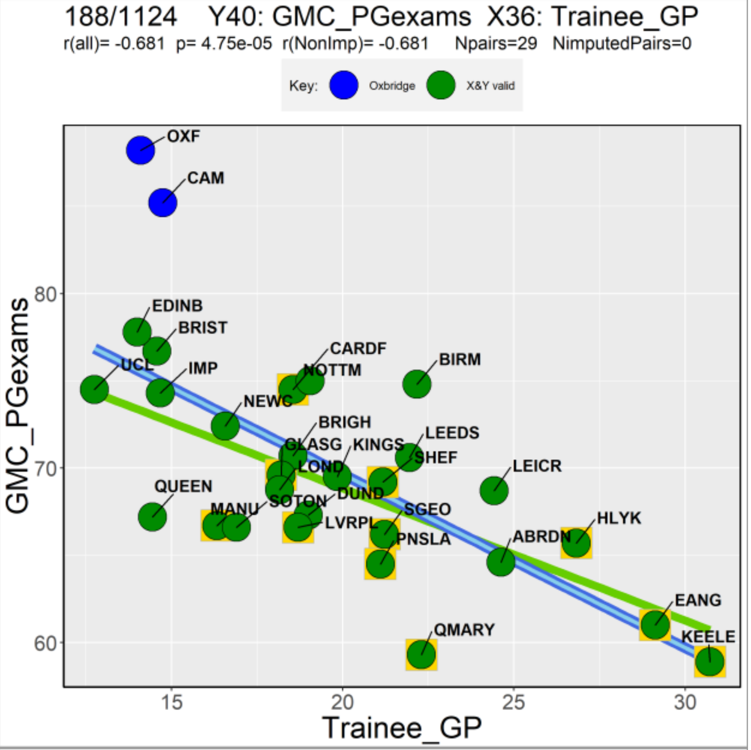
188/1124 Y40: GMC_PGexams X36: Trainee_GP
r(all)= -0.681 p= 4.75e-05 r(NonImp)= -0.681 Npairs=29 NimputedPairs=0
Key: Oxbridge X&Y valid
GMC_PGexams
Trainee_GP
80
70
60
15
20
25
30
OXF
CAM
EDINB
BRIST
CARDF
NOTTM
BIRM
UCL
IMP
NEWC
BRIGH
LEEDS
GLASG
KINGS
SHEF
LOND
LEICR
QUEEN
DUND
MANU
SOTON
SGEO
LVRPL
HLYK
PNSLA
ABRDN
EANG
QMARY
KEELE
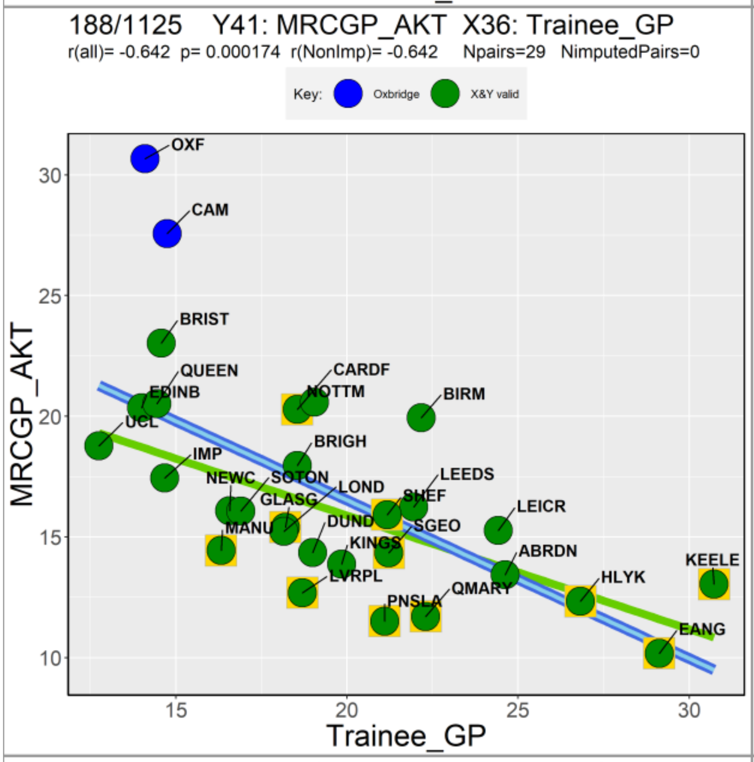
188/1125 Y41: MRCGP_AKT X36: Trainee_GP
r(all)= -0.642 p= 0.000174 r(NonImp)= -0.642 Npairs=29 NimputedPairs=0
Key: Oxbridge X&Y valid
MRCGP_AKT
Trainee_GP
30
25
20
15
10
15
20
25
30
OXF
CAM
BRIST
QUEEN
CARDF
EDINB
NOTTM
BIRM
UCL
BRIGH
IMP
LEEDS
NEWC
SOTON
LOND
GLASG
SHEF
LEICR
MANU
DUND
SGEO
KINGS
ABRDN
KEELE
LVRPL
HLYK
PNSLA
QMARY
EANG
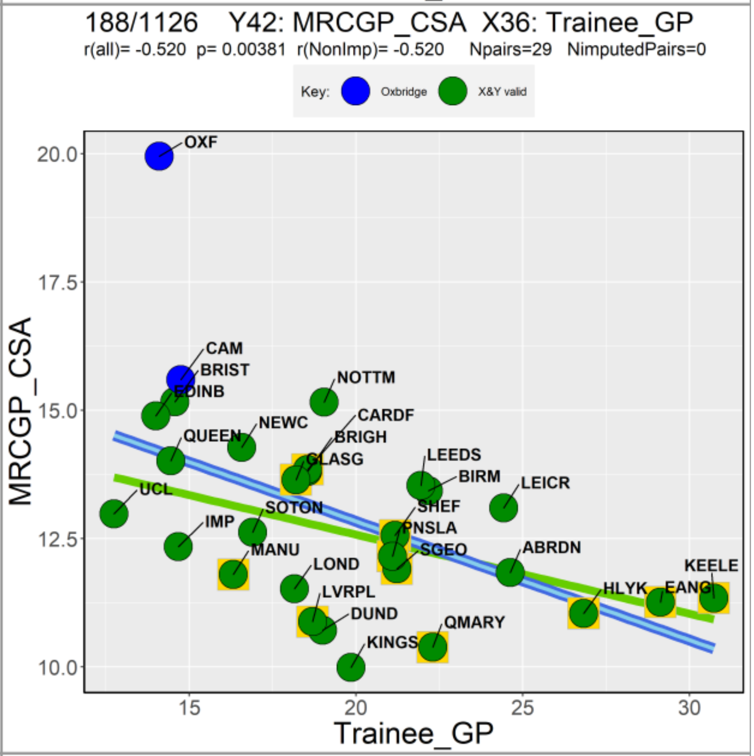
188/1126 Y42: MRCGP_CSA X36: Trainee_GP
r(all)= -0.520 p= 0.00381 r(NonImp)= -0.520 Npairs=29 NimputedPairs=0
Key: Oxbridge X&Y valid
MRCGP_CSA
Trainee_GP
20.0
17.5
15.0
12.5
10.0
15
20
25
30
OXF
CAM
BRIST
NOTTM
EDINB
CARDF
QUEEN
NEWC
BRIGH
GLASG
LEEDS
BIRM
UCL
LEICR
SOTON
SHEF
IMP
PNSLA
MANU
SGEO
ABRDN
LOND
KEELE
LVRPL
HLYK
EANG
DUND
QMARY
KINGS
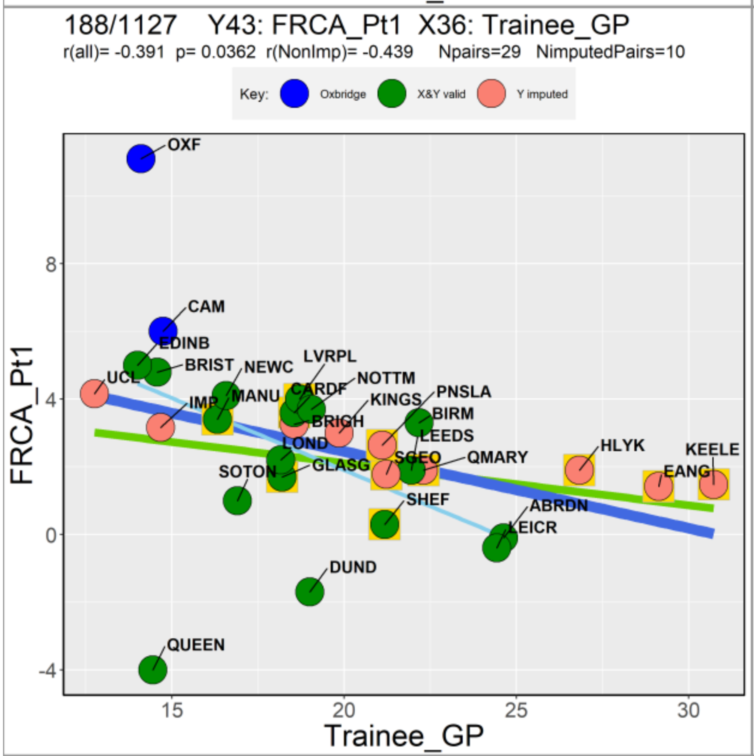
188/1127 Y43: FRCA_Pt1 X36: Trainee_GP
r(all)= -0.391 p= 0.0362 r(NonImp)= -0.439 Npairs=29 NimputedPairs=10
Key: Oxbridge X&Y valid Y imputed
FRCA_Pt1
Trainee_GP
8
4
0
-4
15
20
25
30
OXF
CAM
EDINB
BRIST
NEWC
LVRPL
UCL
NOTTM
MANU
CARDF
PNSLA
KINGS
IMP
BIRM
BRIGH
LEEDS
LOND
HLYK
KEELE
SGEO
QMARY
SOTON
GLASG
EANG
SHEF
ABRDN
LEICR
DUND
QUEEN
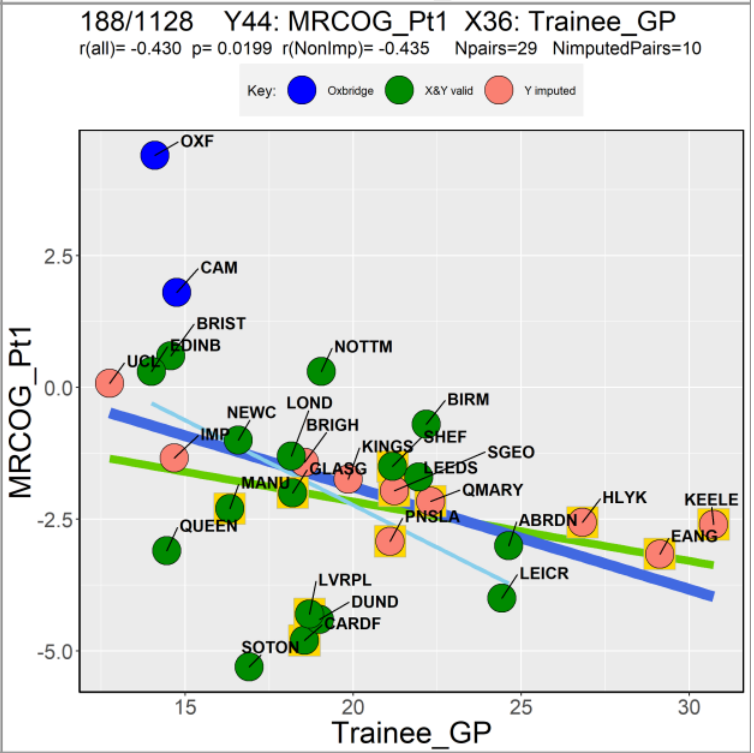
188/1128 Y44: MRCOG_Pt1 X36: Trainee_GP
r(all)= -0.430 p= 0.0199 r(NonImp)= -0.435 Npairs=29 NimputedPairs=10
Key: Oxbridge X&Y valid Y imputed
MRCOG_Pt1
Trainee_GP
2.5
0.0
-2.5
-5.0
15
20
25
30
OXF
CAM
BRIST
EDINB
NOTTM
UCL
BIRM
LOND
NEWC
BRIGH
SHEF
IMP
KINGS
SGEO
GLASG
LEEDS
MANU
QMARY
HLYK
KEELE
PNSLA
ABRDN
QUEEN
EANG
LEICR
LVRPL
DUND
CARDF
SOTON

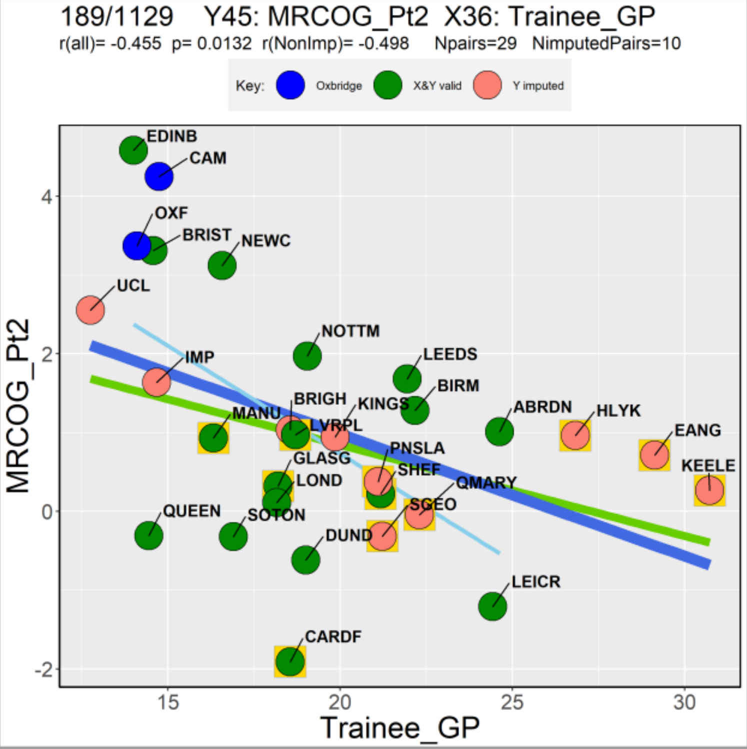
189/1129 Y45: MRCOG_Pt2 X36: Trainee_GP
r(all)= -0.455 p= 0.0132 r(NonImp)= -0.498 Npairs=29 NimputedPairs=10
Key: Oxbridge X&Y valid Y imputed
MRCOG_Pt2
Trainee_GP
EDINB
CAM
OXF
BRIST
NEWC
UCL
NOTTM
IMP
LEEDS
BIRM
ABRDN
HLYK
BRIGH
KINGS
MANU
LVRPL
EANG
KEELE
GLASG
PNSLA
SHEF
QMARY
LOND
SGEO
QUEEN
SOTON
DUND
LEICR
CARDF

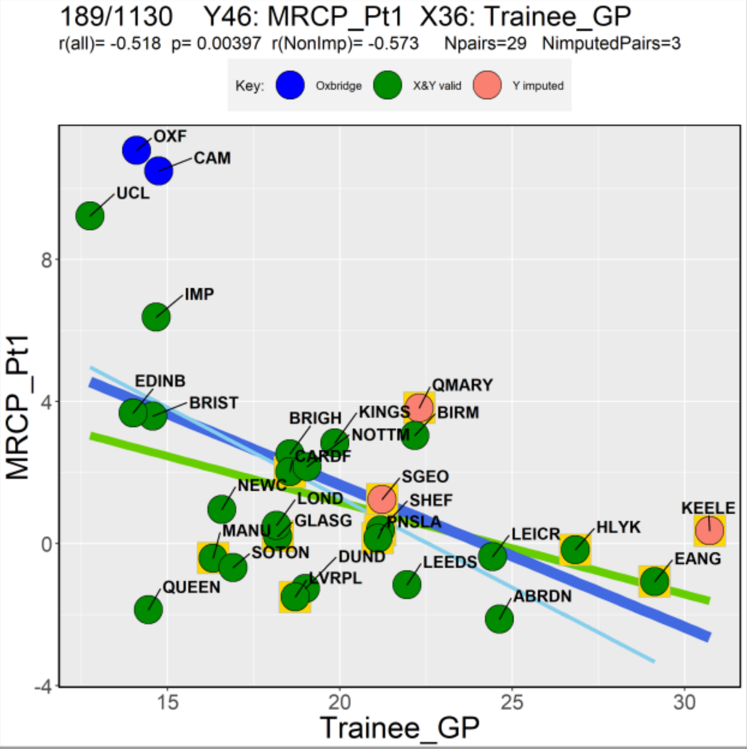
189/1130 Y46: MRCP_Pt1 X36: Trainee_GP
r(all)= -0.518 p= 0.00397 r(NonImp)= -0.573 Npairs=29 NimputedPairs=3
Key: Oxbridge X&Y valid Y imputed
MRCP_Pt1
Trainee_GP
OXF
CAM
UCL
IMP
EDINB
BRIST
QMARY
BRIGH
KINGS
BIRM
NOTTM
CARDF
SGEO
NEWC
LOND
SHEF
GLASG
PNSLA
MANU
SOTON
DUND
LEICR
HLYK
KEELE
EANG
LVRPL
LEEDS
QUEEN
ABRDN

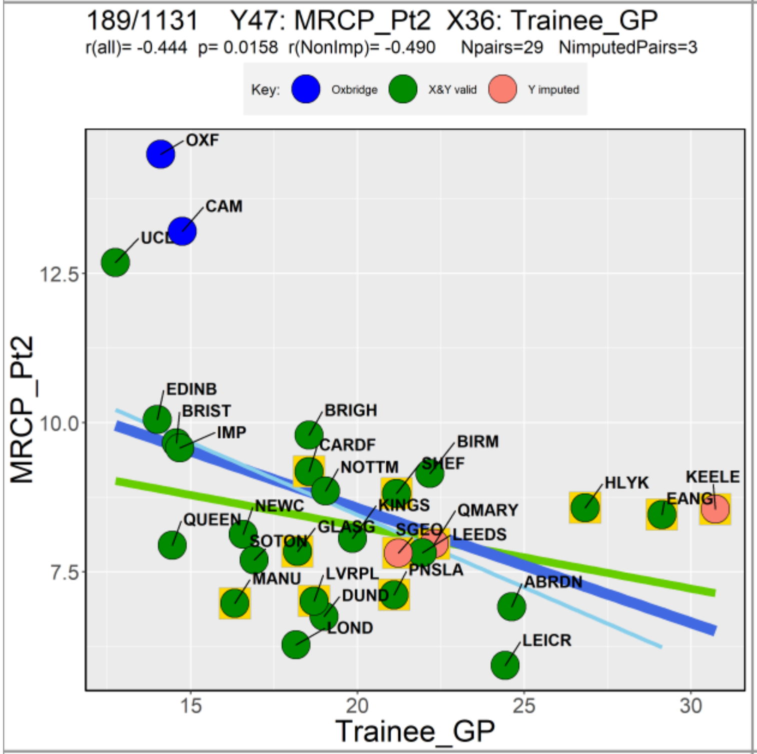
189/1131 Y47: MRCP_Pt2 X36: Trainee_GP
r(all)= -0.444 p= 0.0158 r(NonImp)= -0.490 Npairs=29 NimputedPairs=3
Key: Oxbridge X&Y valid Y imputed
MRCP_Pt2
Trainee_GP
OXF
CAM
UCL
EDINB
BRIST
IMP
BRIGH
BIRM
CARDF
NOTTM
SHEF
HLYK
KEELE
EANG
QUEEN
NEWC
KINGS
QMARY
SOTON
GLASG
SGEO
LEEDS
PNSLA
MANU
LVRPL
DUND
ABRDN
LOND
LEICR

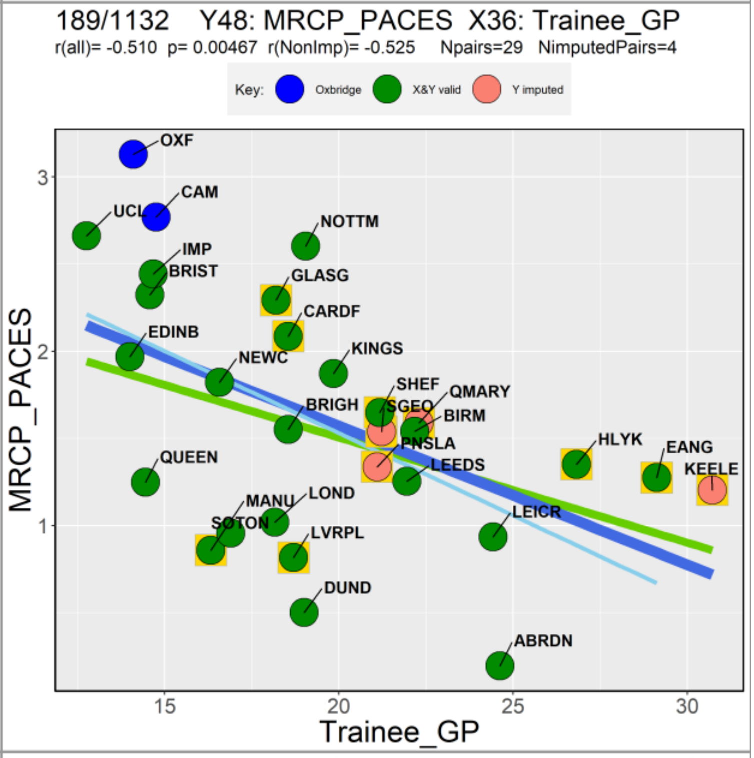
189/1132 Y48: MRCP_PACES X36: Trainee_GP
r(all)= -0.510 p= 0.00467 r(NonImp)= -0.525 Npairs=29 NimputedPairs=4
Key: Oxbridge X&Y valid Y imputed
MRCP_PACES
Trainee_GP
OXF
CAM
UCL
NOTTM
IMP
BRIST
GLASG
CARDF
EDINB
KINGS
NEWC
SHEF
QMARY
SGEO
BIRM
BRIGH
PNSLA
LEEDS
HLYK
EANG
KEELE
QUEEN
MANU
LOND
SOTON
LEICR
LVRPL
DUND
ABRDN

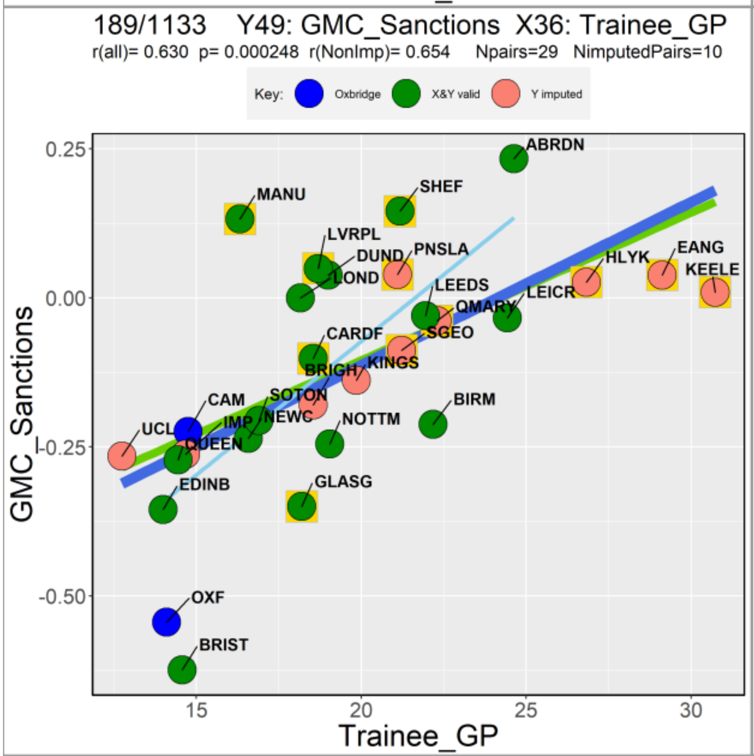
189/1133 Y49: GMC_Sanctions X36: Trainee_GP
r(all)= 0.630 p= 0.000248 r(NonImp)= 0.654 Npairs=29 NimputedPairs=10
Key: Oxbridge X&Y valid Y imputed
GMC_Sanctions
Trainee_GP
ABRDN
MANU
SHEF
LVRPL
DUND
PNSLA
EANG
HLYK
KEELE
LOND
LEEDS
LEICR
QMARY
CARDF
SGEO
BRIGH
KINGS
CAM
SOTON
BIRM
UCL
IMP
NEWC
NOTTM
QUEEN
EDINB
GLASG
OXF
BRIST

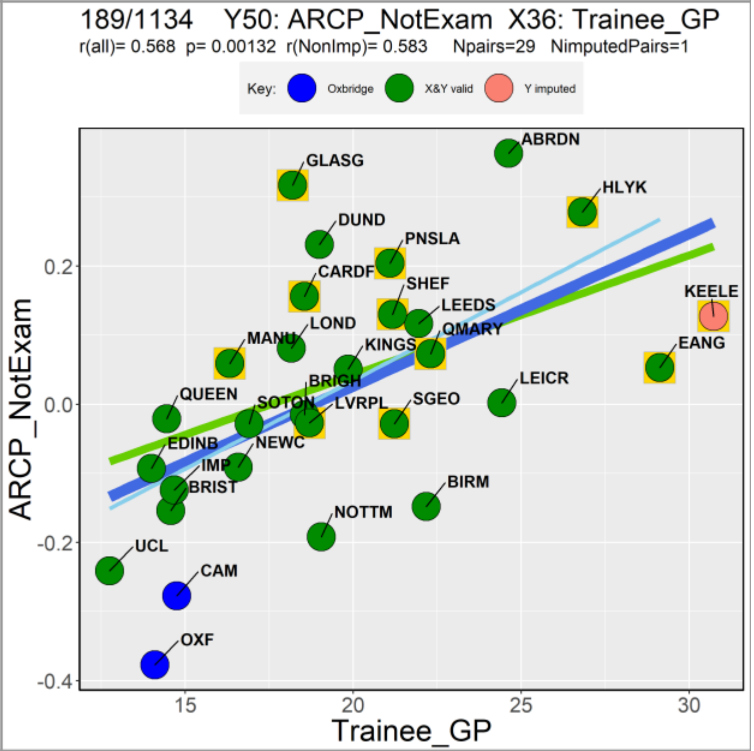
189/1134 Y50: ARCP_NotExam X36: Trainee_GP
r(all)= 0.568 p= 0.00132 r(NonImp)= 0.583 Npairs=29 NimputedPairs=1
Key: Oxbridge X&Y valid Y imputed
ARCP_NotExam
Trainee_GP
ABRDN
GLASG
HLYK
DUND
PNSLA
CARDF
SHEF
LEEDS
KEELE
LOND
MANU
QMARY
KINGS
EANG
BRIGH
LEICR
QUEEN
SOTON
LVRPL
SGEO
EDINB
NEWC
IMP
BRIST
BIRM
NOTTM
UCL
CAM
OXF

190/1135 Y38: TraineeApp_Surgery X37: Trainee_P

r(all)= -0.018 p= 0.926 r(NonImp)= -0.043 Npairs=29 NimputedPairs=2

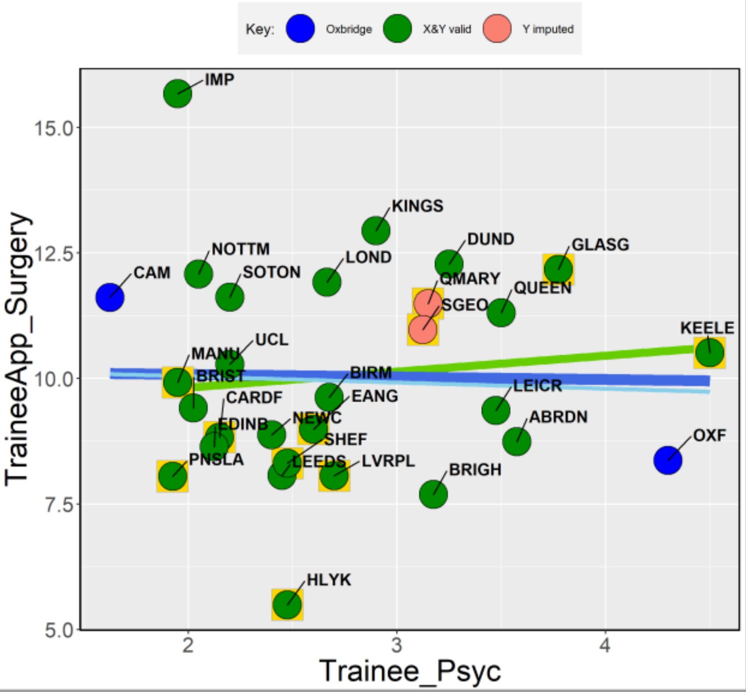

190/1136 Y39: TraineeApp_Anaes X37: Trainee_Ps

r(all)= 0.128 p= 0.51 r(NonImp)= 0.128 Npairs=29 NimputedPairs=0

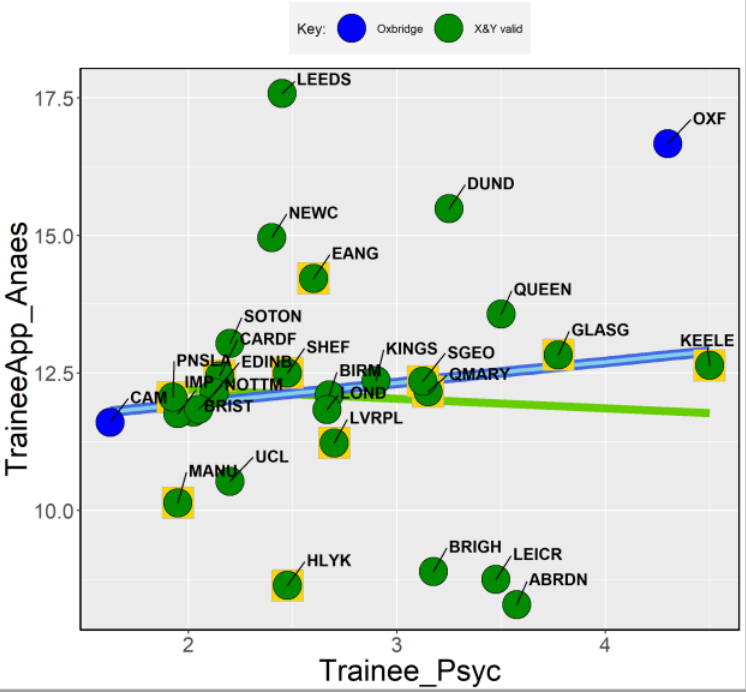

190/1137 Y40: GMC_PGexams X37: Trainee_Psyc

r(all)= -0.250 p= 0.192 r(NonImp)= -0.250 Npairs=29 NimputedPairs=0

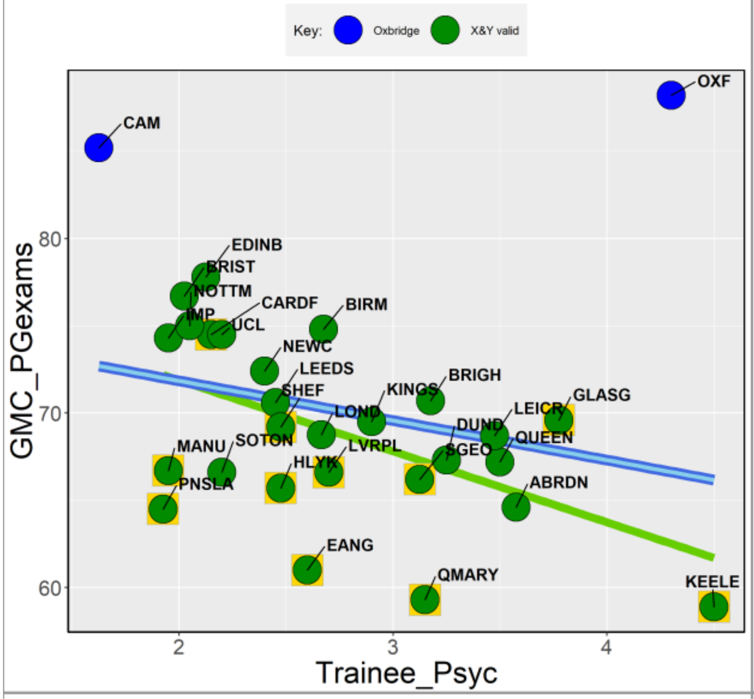

190/1138 Y41: MRCGP_AKT X37: Trainee_Psyc

r(all)= -0.076 p= 0.696 r(NonImp)= -0.076 Npairs=29 NimputedPairs=0

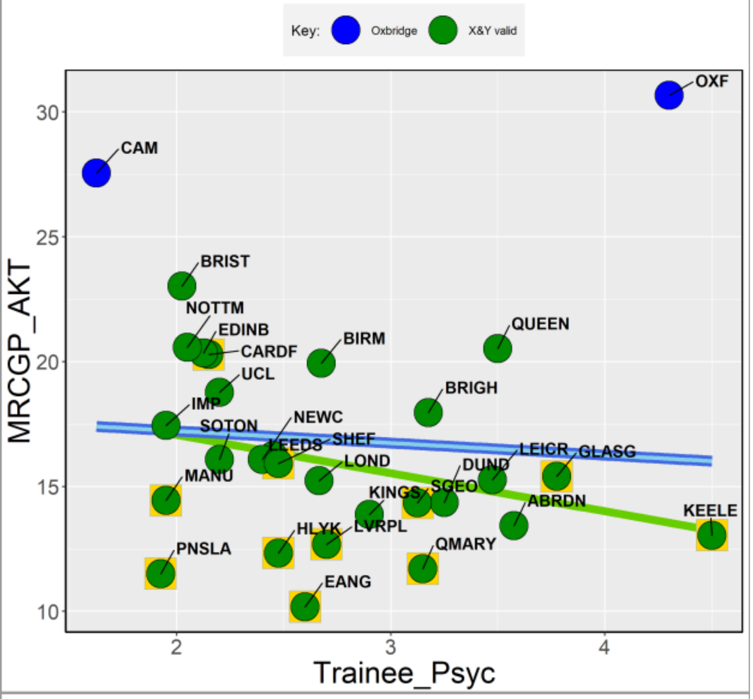

190/1139 Y42: MRCGP_CSA X37: Trainee_Psyc

r(all)= 0.022 p= 0.911 r(NonImp)= 0.022 Npairs=29 NimputedPairs=0

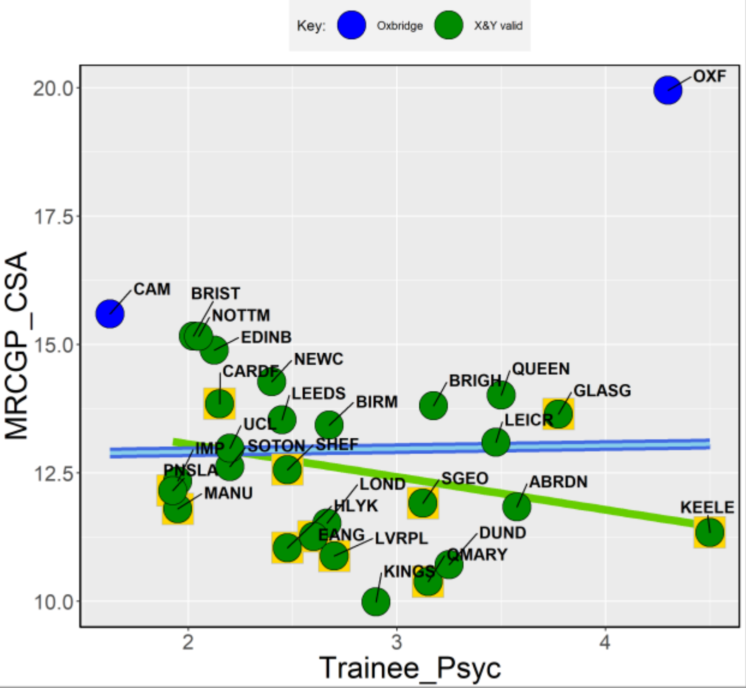

190/1140 Y43: FRCA_Pt1 X37: Trainee_Psyc

r(all)= -0.188 p= 0.328 r(NonImp)= -0.164 Npairs=29 NimputedPairs=10

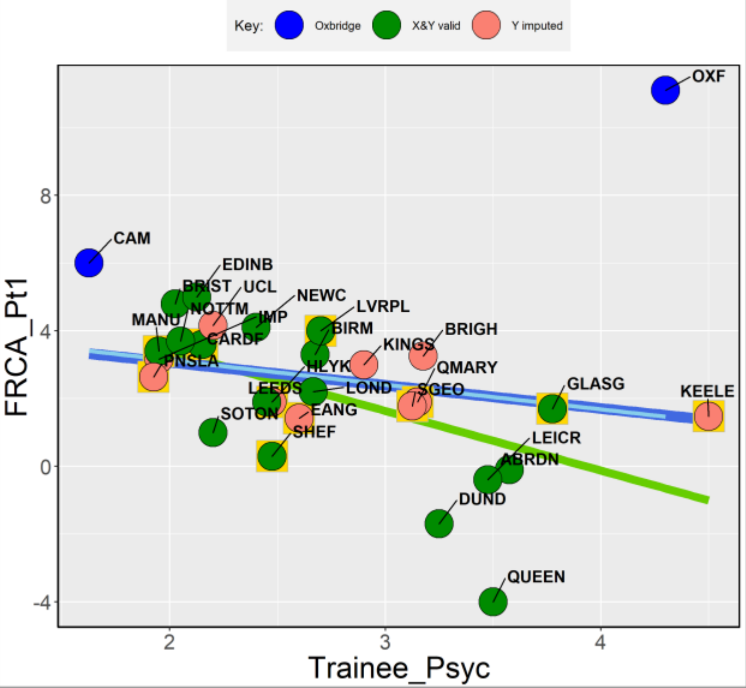

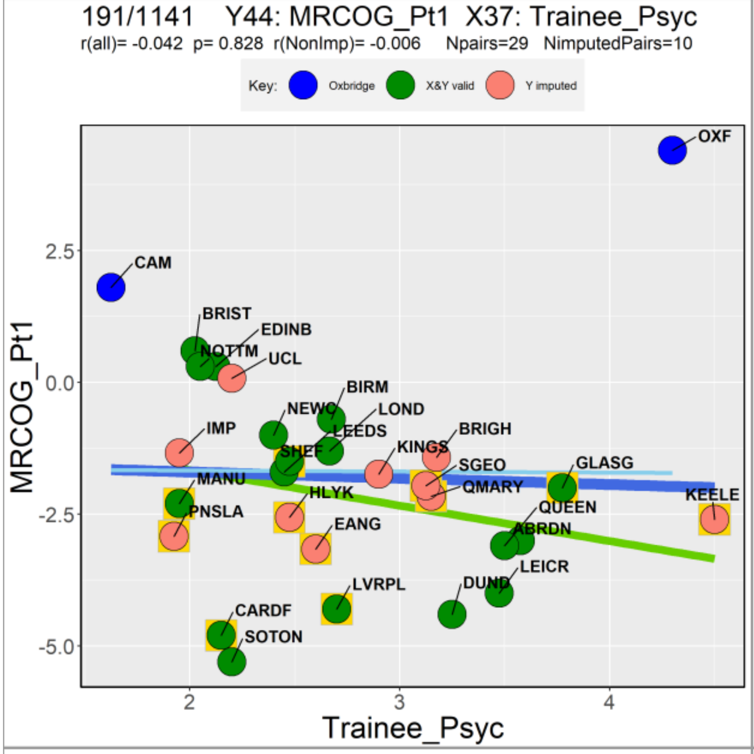
191/1141 Y44: MRCOG_Pt1 X37: Trainee_Psyc
r(all)= -0.042 p= 0.828 r(NonImp)= -0.006 Npairs=29 NimputedPairs=10
Key: Oxbridge X&Y valid Y imputed
MRCOG_Pt1
Trainee_Psyc
2.5
0.0
-2.5
-5.0
2
3
4
OXF
CAM
BRIST
EDINB
NOTTM
UCL
BIRM
NEWC
LOND
IMP
LEEDS
BRIGH
SHEF
KINGS
SGEO
GLASG
MANU
HLYK
QMARY
KEELE
PNSLA
QUEEN
EANG
ABRDN
LEICR
LVRPL
DUND
CARDF
SOTON

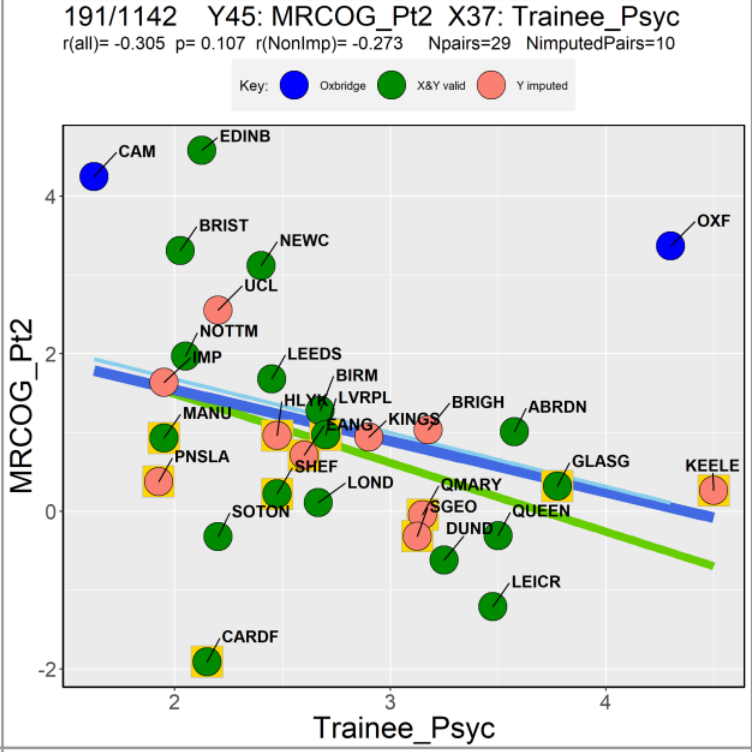
191/1142 Y45: MRCOG_Pt2 X37: Trainee_Psyc
r(all)= -0.305 p= 0.107 r(NonImp)= -0.273 Npairs=29 NimputedPairs=10
Key: Oxbridge X&Y valid Y imputed
MRCOG_Pt2
Trainee_Psyc
4
2
0
-2
2
3
4
EDINB
CAM
BRIST
NEWC
OXF
UCL
NOTTM
IMP
LEEDS
BIRM
HLYK
LVRPL
MANU
BRIGH
ABRDN
EANG
KINGS
PNSLA
SHEF
GLASG
KEELE
LOND
QMARY
SGEO
SOTON
QUEEN
DUND
LEICR
CARDF

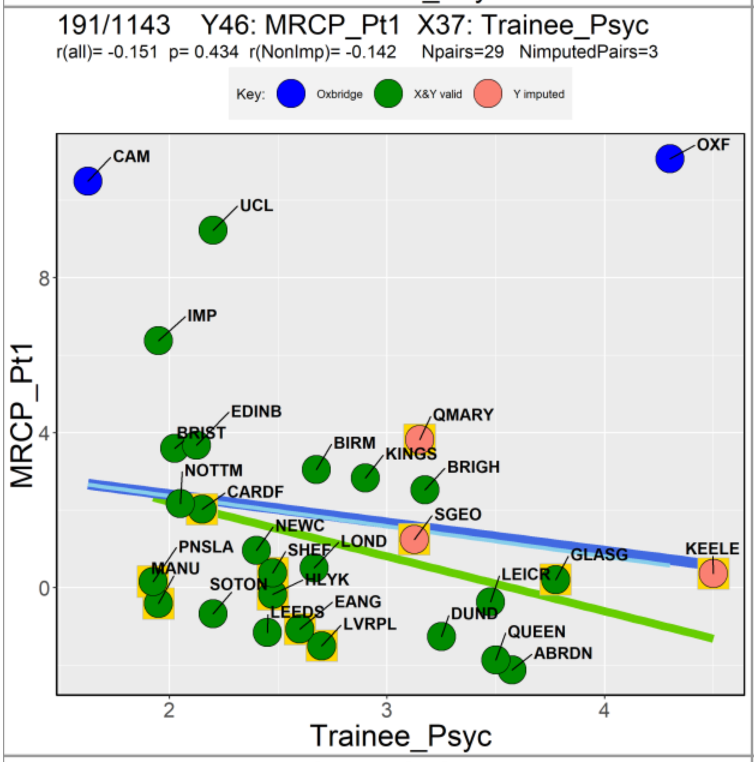
191/1143 Y46: MRCP_Pt1 X37: Trainee_Psyc
r(all)= -0.151 p= 0.434 r(NonImp)= -0.142 Npairs=29 NimputedPairs=3
Key: Oxbridge X&Y valid Y imputed
MRCP_Pt1
Trainee_Psyc
8
4
0
2
3
4
CAM
OXF
UCL
IMP
EDINB
QMARY
BRIST
BIRM
KINGS
NOTTM
BRIGH
CARDF
SGEO
NEWC
LOND
PNSLA
SHEF
GLASG
KEELE
MANU
LEICR
SOTON
HLYK
EANG
LEEDS
LVRPL
DUND
QUEEN
ABRDN

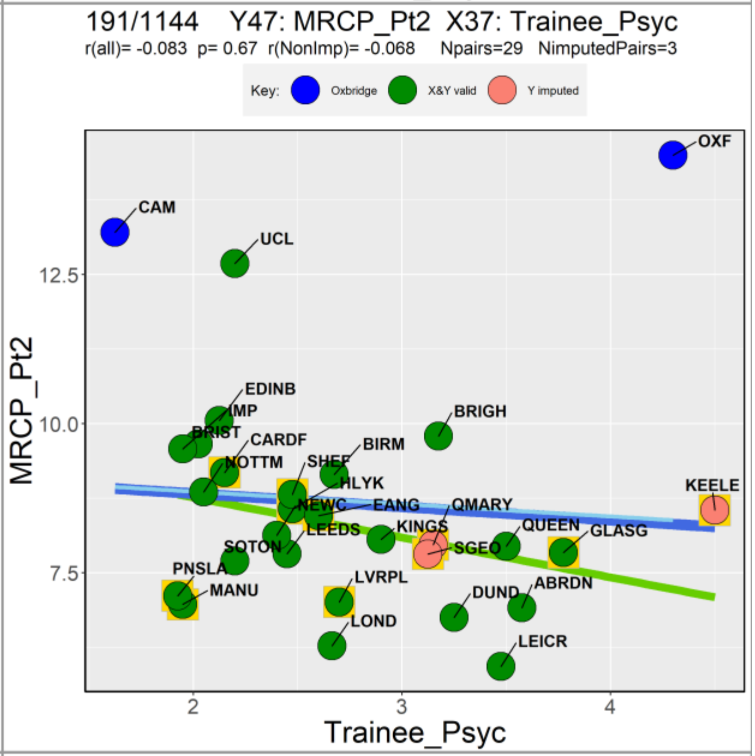
191/1144 Y47: MRCP_Pt2 X37: Trainee_Psyc
r(all)= -0.083 p= 0.67 r(NonImp)= -0.068 Npairs=29 NimputedPairs=3
Key: Oxbridge X&Y valid Y imputed
MRCP_Pt2
Trainee_Psyc
12.5
10.0
7.5
2
3
4
OXF
CAM
UCL
EDINB
IMP
BRIGH
BRIST
CARDF
BIRM
NOTTM
SHEF
HLYK
KEELE
NEWC
EANG
QMARY
LEEDS
QUEEN
GLASG
SOTON
SGEO
PNSLA
LVRPL
MANU
DUND
ABRDN
LOND
LEICR

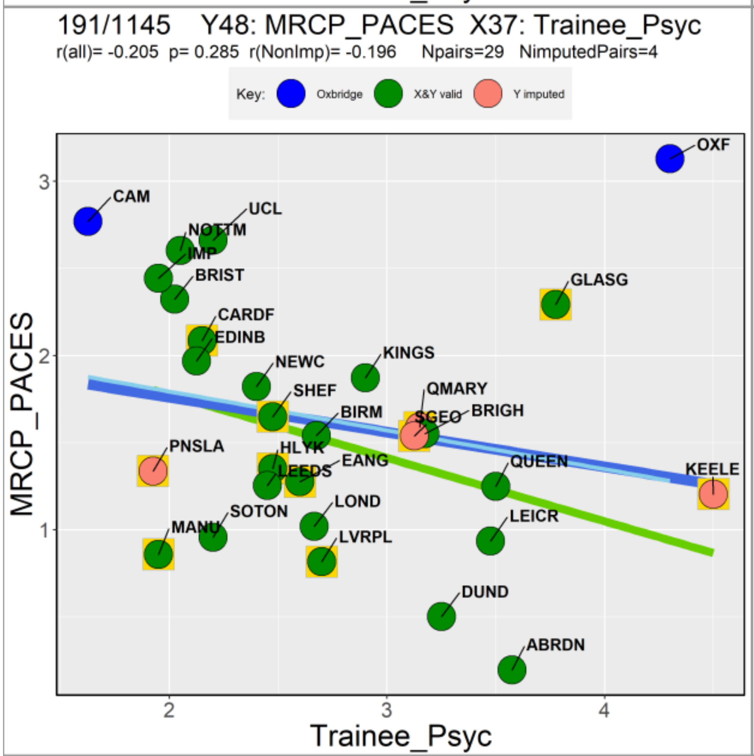
191/1145 Y48: MRCP_PACES X37: Trainee_Psyc
r(all)= -0.205 p= 0.285 r(NonImp)= -0.196 Npairs=29 NimputedPairs=4
Key: Oxbridge X&Y valid Y imputed
MRCP_PACES
Trainee_Psyc
3
2
1
2
3
4
OXF
CAM
UCL
NOTTM
IMP
BRIST
GLASG
CARDF
EDINB
KINGS
NEWC
SHEF
QMARY
BIRM
SGEO
BRIGH
PNSLA
HLYK
EANG
LEEDS
QUEEN
KEELE
LOND
SOTON
LEICR
MANU
LVRPL
DUND
ABRDN

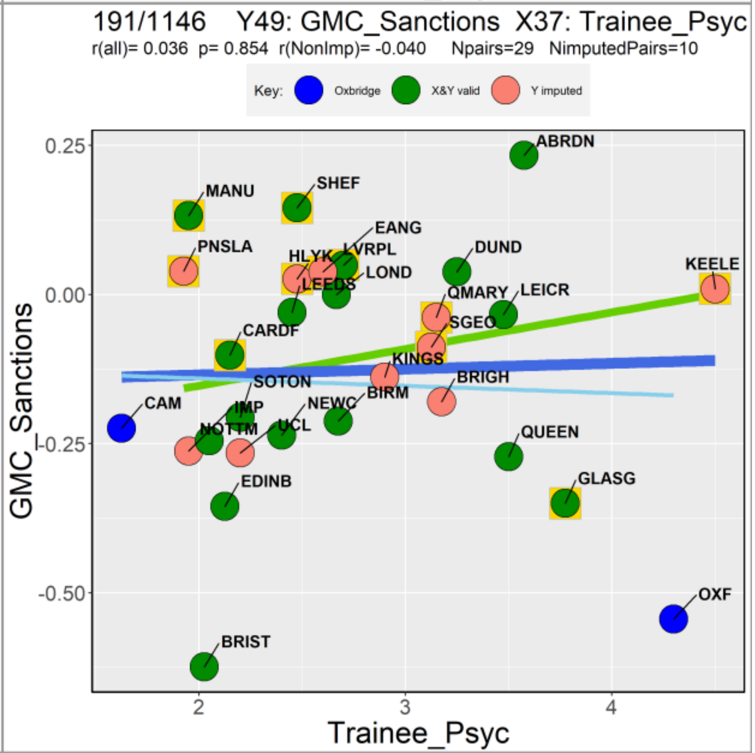
191/1146 Y49: GMC_Sanctions X37: Trainee_Psyc
r(all)= 0.036 p= 0.854 r(NonImp)= -0.040 Npairs=29 NimputedPairs=10
Key: Oxbridge X&Y valid Y imputed
GMC_Sanctions
Trainee_Psyc
0.25
0.00
-0.25
-0.50
2
3
4
ABRDN
MANU
SHEF
EANG
PNSLA
HLYK
LVRPL
DUND
LOND
KEELE
LEEDS
QMARY
LEICR
SGEO
CARDF
KINGS
SOTON
BRIGH
BIRM
CAM
IMP
NEWC
NOTTM
UCL
QUEEN
GLASG
EDINB
OXF
BRIST

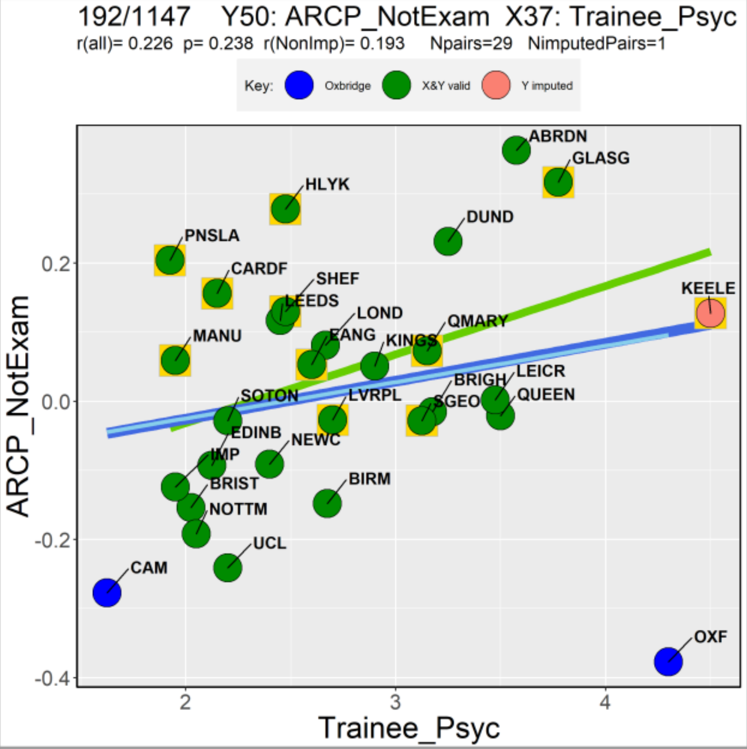
192/1147 Y50: ARCP_NotExam X37: Trainee_Psyc
r(all)= 0.226 p= 0.238 r(NonImp)= 0.193 Npairs=29 NimputedPairs=1
Key: Oxbridge X&Y valid Y imputed
ARCP_NotExam
Trainee_Psyc
ABRDN
GLASG
HLYK
DUND
PNSLA
CARDF
SHEF
LEEDS
LOND
QMARY
KEELE
MANU
EANG
KINGS
BRIGH
LEICR
SOTON
LVRPL
SGEO
QUEEN
EDINB
NEWC
IMP
BRIST
BIRM
NOTTM
UCL
CAM
OXF

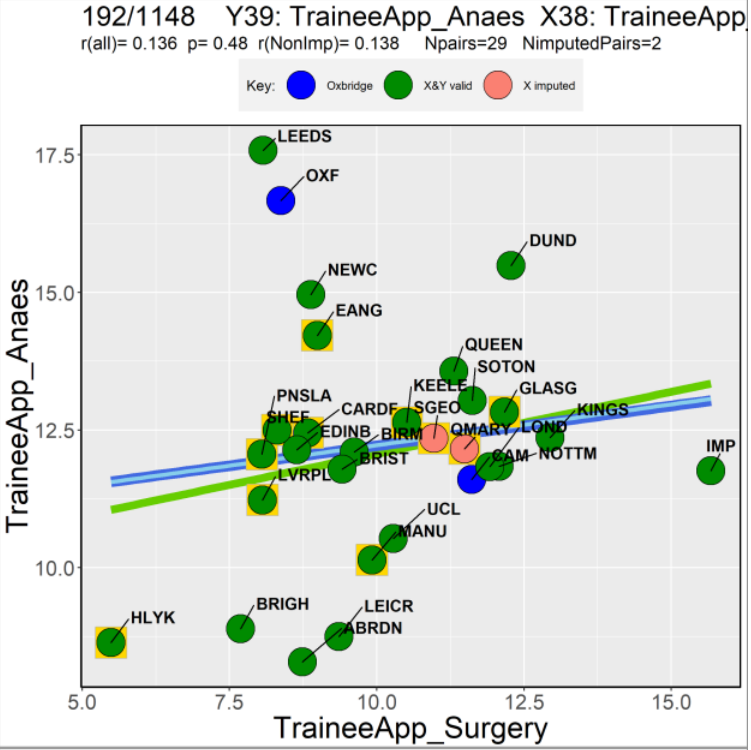
192/1148 Y39: TraineeApp_Anaes X38: TraineeApp_
r(all)= 0.136 p= 0.48 r(NonImp)= 0.138 Npairs=29 NimputedPairs=2
Key: Oxbridge X&Y valid X imputed
TraineeApp_Anaes
TraineeApp_Surgery
LEEDS
OXF
DUND
NEWC
EANG
QUEEN
SOTON
KEELE
PNSLA
GLASG
SGEO
KINGS
CARDF
SHEF
EDINB
BIRM
QMARY
LOND
CAM
NOTTM
IMP
BRIST
LVRPL
UCL
MANU
HLYK
BRIGH
LEICR
ABRDN

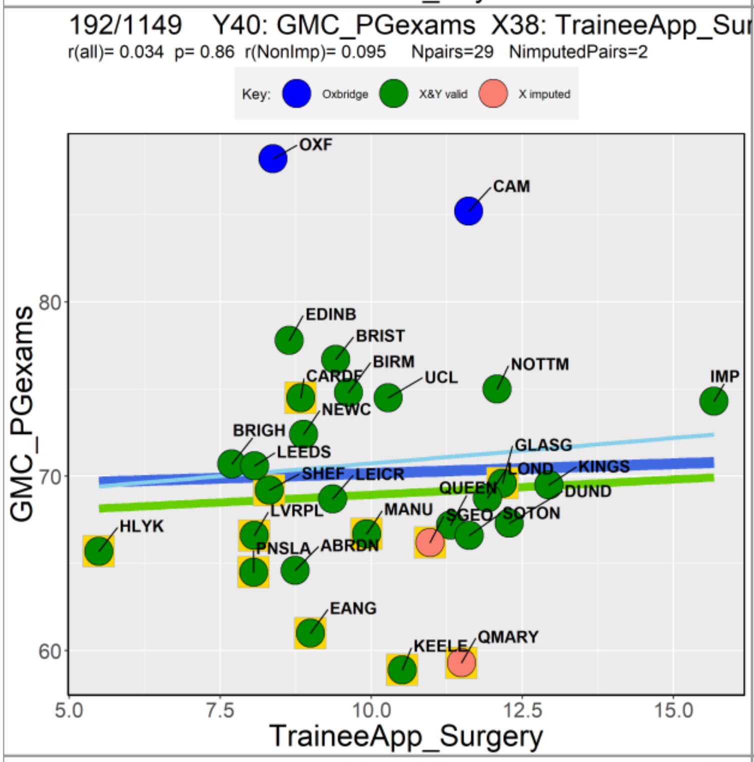
192/1149 Y40: GMC_PGexams X38: TraineeApp_Sur
r(all)= 0.034 p= 0.86 r(NonImp)= 0.095 Npairs=29 NimputedPairs=2
Key: Oxbridge X&Y valid X imputed
GMC_PGexams
TraineeApp_Surgery
OXF
CAM
EDINB
BRIST
BIRM
CARDF
UCL
NOTTM
IMP
NEWC
BRIGH
LEEDS
GLASG
SHEF
LEICR
LOND
KINGS
QUEEN
DUND
LVRPL
MANU
SGEO
SOTON
HLYK
PNSLA
ABRDN
EANG
KEELE
QMARY

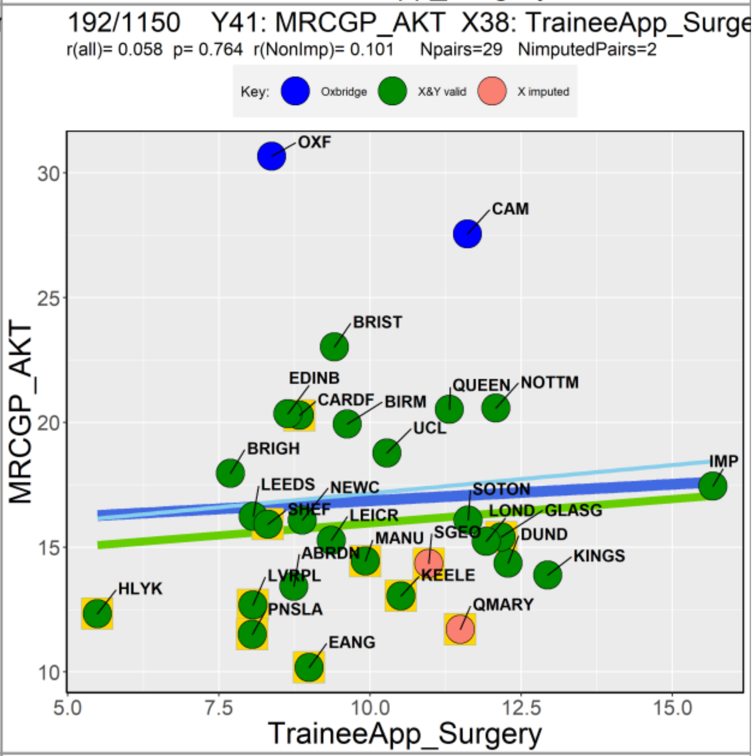
192/1150 Y41: MRCGP_AKT X38: TraineeApp_Surge
r(all)= 0.058 p= 0.764 r(NonImp)= 0.101 Npairs=29 NimputedPairs=2
Key: Oxbridge X&Y valid X imputed
MRCGP_AKT
TraineeApp_Surgery
OXF
CAM
BRIST
EDINB
CARDF
BIRM
QUEEN
NOTTM
UCL
BRIGH
LEEDS
NEWC
SOTON
IMP
SHEF
LEICR
LOND
GLASG
MANU
SGEO
DUND
ABRDN
KINGS
LVRPL
KEELE
HLYK
PNSLA
QMARY
EANG

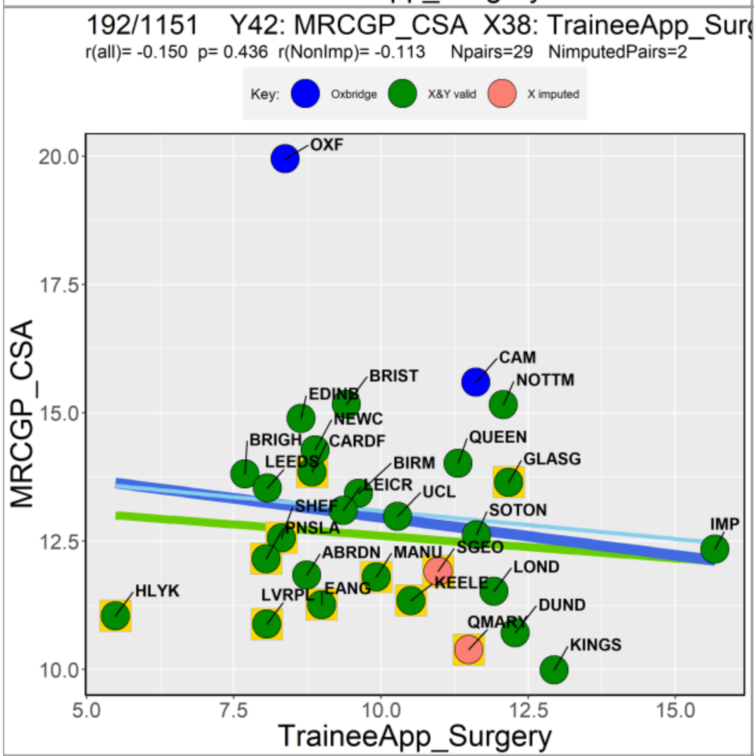
192/1151 Y42: MRCGP_CSA X38: TraineeApp_Surg
r(all)= -0.150 p= 0.436 r(NonImp)= -0.113 Npairs=29 NimputedPairs=2
Key: Oxbridge X&Y valid X imputed
MRCGP_CSA
TraineeApp_Surgery
OXF
CAM
BRIST
NOTTM
EDINB
NEWC
BRIGH
CARDF
QUEEN
LEEDS
BIRM
GLASG
SHEF
LEICR
UCL
SOTON
IMP
PNSLA
ABRDN
MANU
SGEO
LOND
KEELE
LVRPL
EANG
QMARY
DUND
HLYK
KINGS

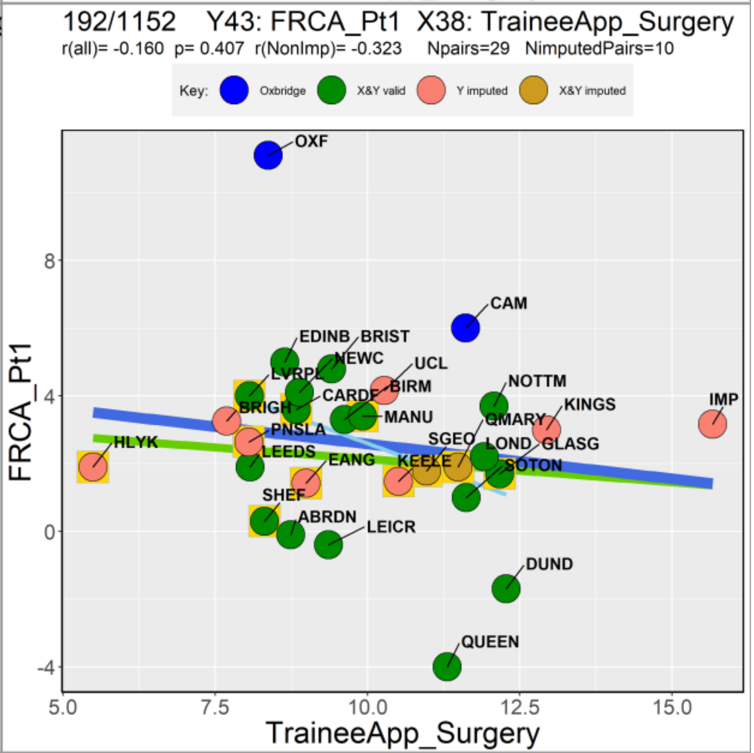
192/1152 Y43: FRCA_Pt1 X38: TraineeApp_Surgery
r(all)= -0.160 p= 0.407 r(NonImp)= -0.323 Npairs=29 NimputedPairs=10
Key: Oxbridge X&Y valid Y imputed X&Y imputed
FRCA_Pt1
TraineeApp_Surgery
OXF
CAM
EDINB
BRIST
LVRPL
NEWC
UCL
BIRM
NOTTM
CARDF
BRIGH
MANU
QMARY
KINGS
IMP
PNSLA
HLYK
SGEO
LOND
GLASG
LEEDS
EANG
KEELE
SOTON
SHEF
ABRDN
LEICR
DUND
QUEEN

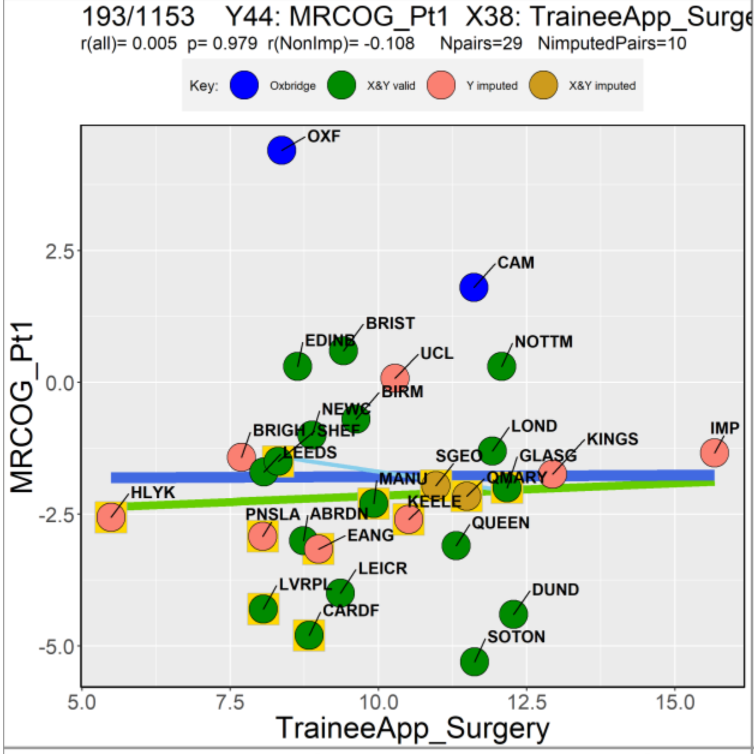
193/1153 Y44: MRCOG_Pt1 X38: TraineeApp_Surge
r(all)= 0.005 p= 0.979 r(NonImp)= -0.108 Npairs=29 NimputedPairs=10
Key: Oxbridge X&Y valid Y imputed X&Y imputed
OXF
CAM
BRIST
EDINB
UCL
NOTTM
BIRM
NEWC
BRIGH
SHEF
LOND
KINGS
IMP
LEEDS
SGEO
GLASG
MANU
QMARY
HLYK
KEELE
PNSLA
ABRDN
QUEEN
EANG
LEICR
LVRPL
DUND
CARDF
SOTON
MRCOG_Pt1
2.5
0.0
-2.5
-5.0
5.0
7.5
10.0
12.5
15.0
TraineeApp_Surgery

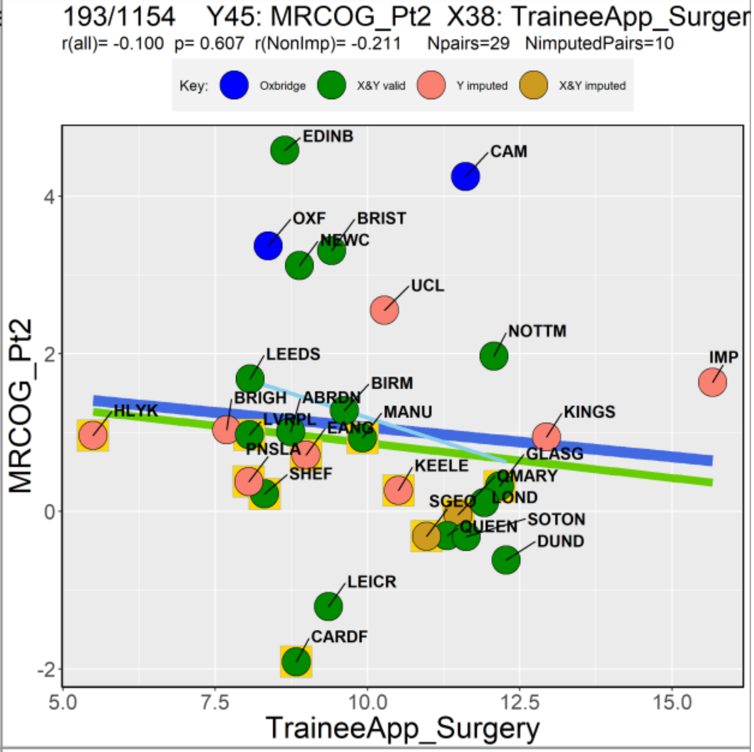
193/1154 Y45: MRCOG_Pt2 X38: TraineeApp_Surger
r(all)= -0.100 p= 0.607 r(NonImp)= -0.211 Npairs=29 NimputedPairs=10
Key: Oxbridge X&Y valid Y imputed X&Y imputed
EDINB
CAM
OXF
BRIST
NEWC
UCL
NOTTM
LEEDS
IMP
BIRM
HLYK
BRIGH
ABRDN
MANU
KINGS
LVRPL
EANG
PNSLA
GLASG
KEELE
SHEF
QMARY
SGEO
LOND
QUEEN
SOTON
DUND
LEICR
CARDF
MRCOG_Pt2
4
2
0
-2
5.0
7.5
10.0
12.5
15.0
TraineeApp_Surgery

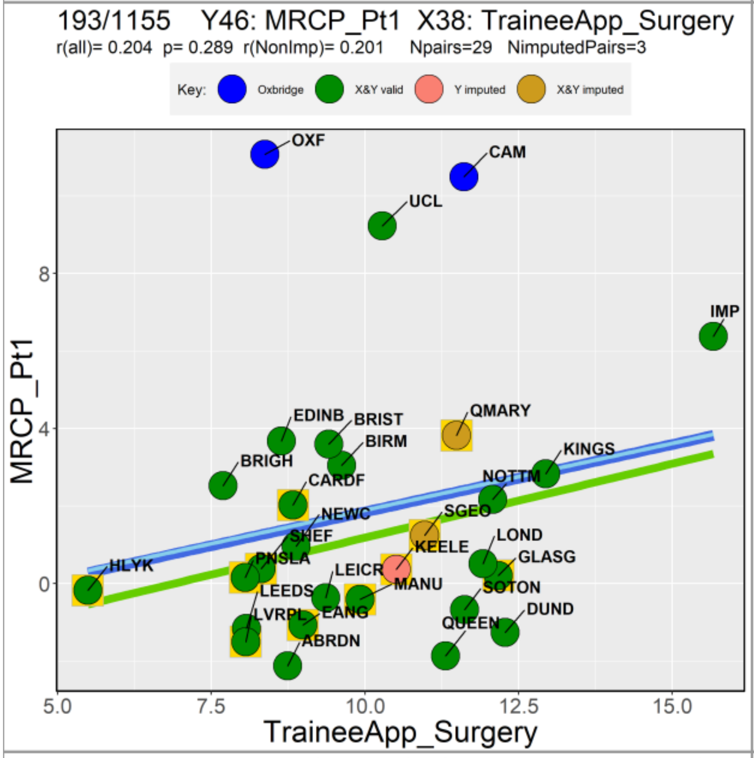
193/1155 Y46: MRCP_Pt1 X38: TraineeApp_Surgery
r(all)= 0.204 p= 0.289 r(NonImp)= 0.201 Npairs=29 NimputedPairs=3
Key: Oxbridge X&Y valid Y imputed X&Y imputed
OXF
CAM
UCL
IMP
EDINB
BRIST
QMARY
BIRM
KINGS
BRIGH
CARDF
NOTTM
NEWC
SGEO
SHEF
LOND
KEELE
HLYK
PNSLA
GLASG
LEICR
LEEDS
MANU
SOTON
LVRPL
EANG
QUEEN
DUND
ABRDN
MRCP_Pt1
8
4
0
5.0
7.5
10.0
12.5
15.0
TraineeApp_Surgery

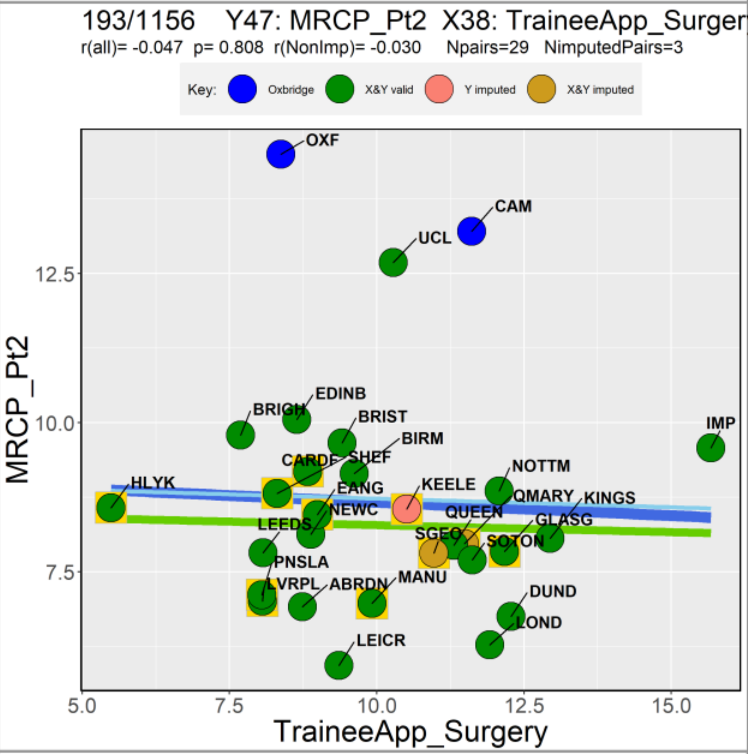
193/1156 Y47: MRCP_Pt2 X38: TraineeApp_Surger
r(all)= -0.047 p= 0.808 r(NonImp)= -0.030 Npairs=29 NimputedPairs=3
Key: Oxbridge X&Y valid Y imputed X&Y imputed
OXF
CAM
UCL
EDINB
BRIGH
BRIST
BIRM
IMP
CARDF
SHEF
NOTTM
HLYK
EANG
KEELE
NEWC
QUEEN
QMARY
KINGS
LEEDS
GLASG
SGEO
SOTON
PNSLA
LVRPL
ABRDN
MANU
DUND
LOND
LEICR
MRCP_Pt2
12.5
10.0
7.5
5.0
7.5
10.0
12.5
15.0
TraineeApp_Surgery

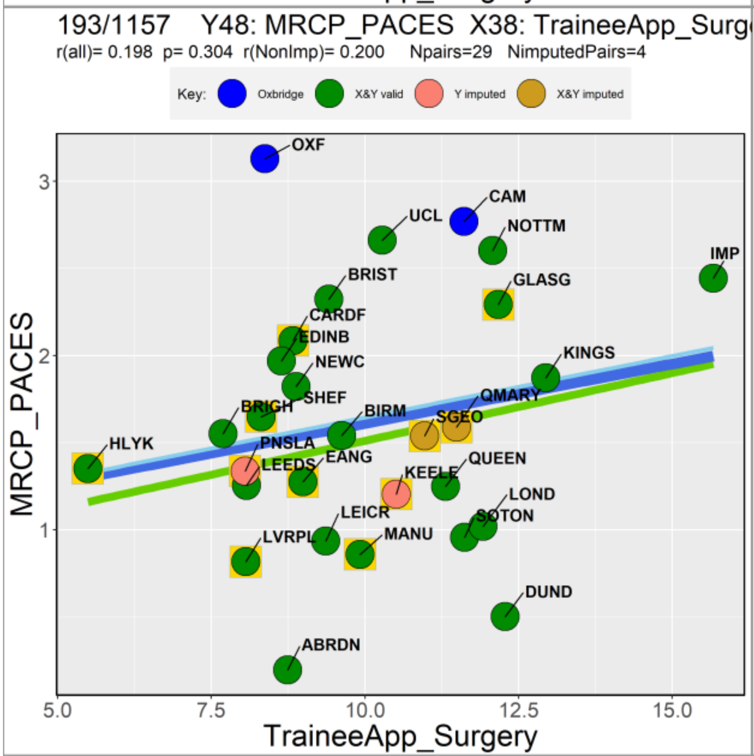
193/1157 Y48: MRCP_PACES X38: TraineeApp_Surg
r(all)= 0.198 p= 0.304 r(NonImp)= 0.200 Npairs=29 NimputedPairs=4
Key: Oxbridge X&Y valid Y imputed X&Y imputed
OXF
CAM
UCL
NOTTM
IMP
BRIST
GLASG
CARDF
EDINB
NEWC
KINGS
SHEF
QMARY
BRIGH
BIRM
SGEO
HLYK
PNSLA
EANG
QUEEN
LEEDS
KEELE
LOND
SOTON
LEICR
LVRPL
MANU
DUND
ABRDN
MRCP_PACES
3
2
1
5.0
7.5
10.0
12.5
15.0
TraineeApp_Surgery

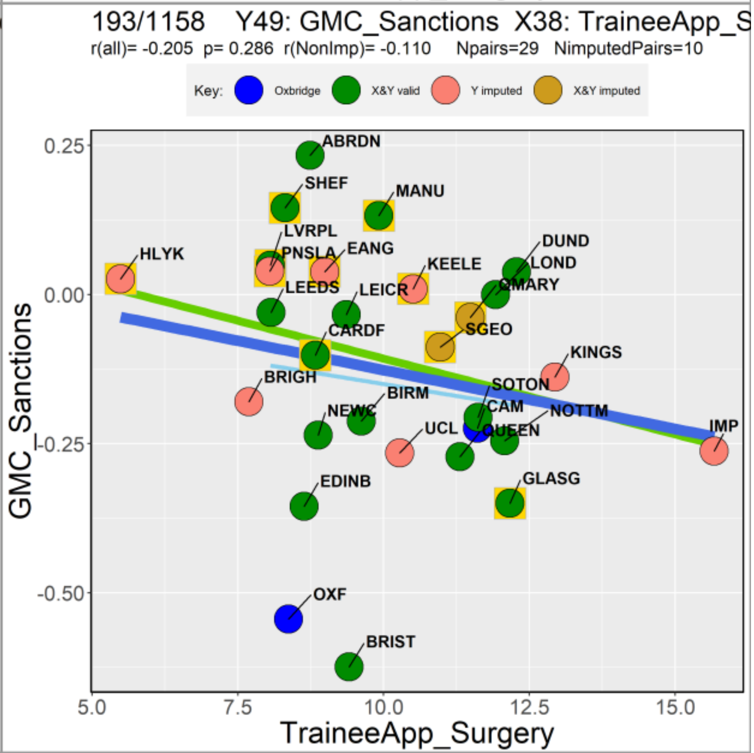
193/1158 Y49: GMC_Sanctions X38: TraineeApp_S
r(all)= -0.205 p= 0.286 r(NonImp)= -0.110 Npairs=29 NimputedPairs=10
Key: Oxbridge X&Y valid Y imputed X&Y imputed
ABRDN
SHEF
MANU
LVRPL
DUND
HLYK
PNSLA
EANG
LOND
KEELE
QMARY
LEEDS
LEICR
CARDF
SGEO
KINGS
BRIGH
SOTON
BIRM
CAM
NEWC
NOTTM
UCL
QUEEN
IMP
EDINB
GLASG
OXF
BRIST
GMC_Sanctions
0.25
0.00
-0.25
-0.50
5.0
7.5
10.0
12.5
15.0
TraineeApp_Surgery

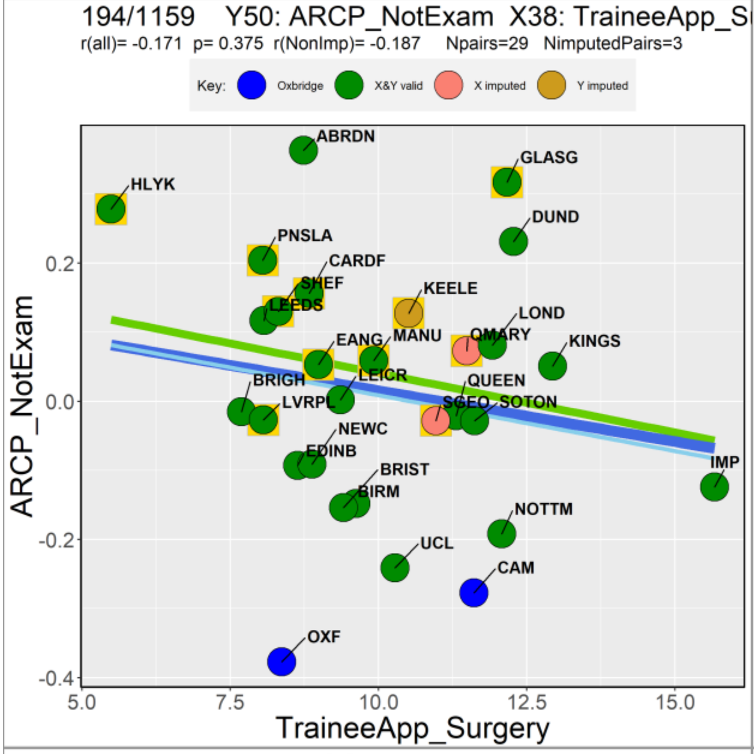
194/1159 Y50: ARCP_NotExam X38: TraineeApp_S
r(all)= -0.171 p= 0.375 r(NonImp)= -0.187 Npairs=29 NimputedPairs=3
Key: Oxbridge X&Y valid X imputed Y imputed
ARCP_NotExam
TraineeApp_Surgery
ABRDN
GLASG
HLYK
DUND
PNSLA
CARDF
SHEF
KEELE
LEEDS
LOND
EANG
MANU
QMARY
KINGS
BRIGH
LEICR
QUEEN
LVRPL
SGEO
SOTON
NEWC
EDINB
BRIST
BIRM
IMP
NOTTM
UCL
CAM
OXF

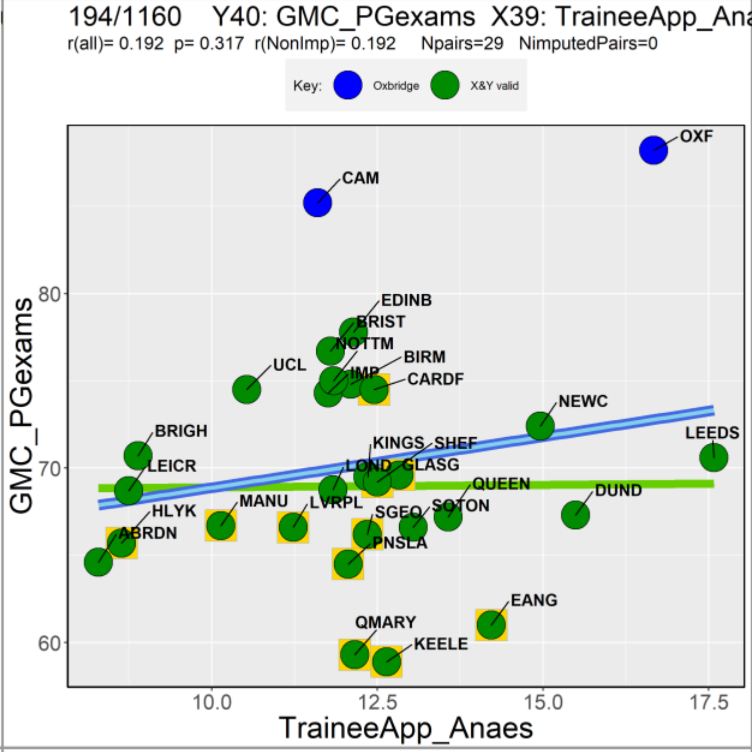
194/1160 Y40: GMC_PGexams X39: TraineeApp_Ana
r(all)= 0.192 p= 0.317 r(NonImp)= 0.192 Npairs=29 NimputedPairs=0
Key: Oxbridge X&Y valid
GMC_PGexams
TraineeApp_Anaes
OXF
CAM
EDINB
BRIST
NOTTM
UCL
BIRM
IMP
CARDF
NEWC
BRIGH
LEEDS
KINGS
SHEF
LEICR
LOND
GLASG
QUEEN
DUND
HLYK
MANU
LVRPL
SGEO
SOTON
ABRDN
PNSLA
EANG
QMARY
KEELE

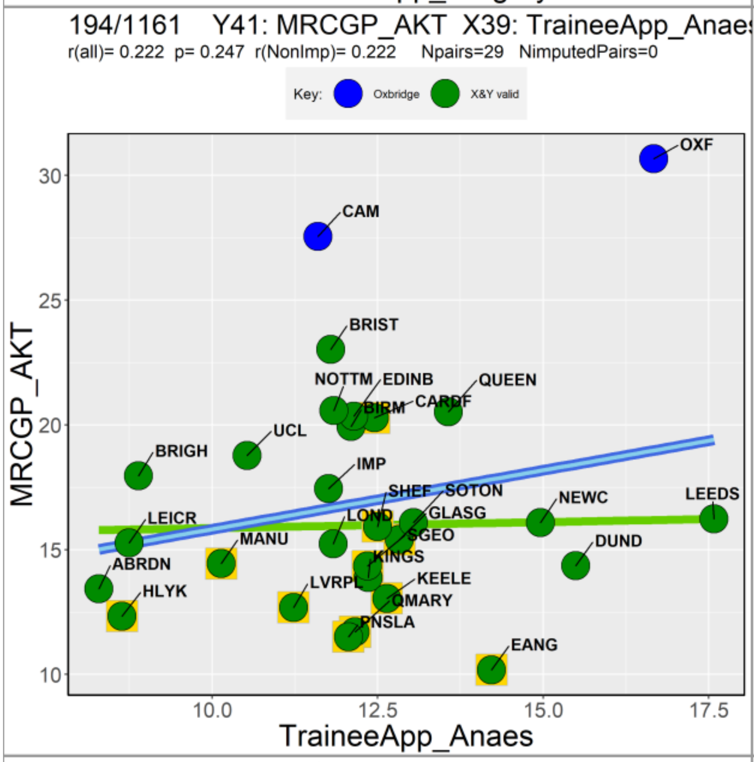
194/1161 Y41: MRCGP_AKT X39: TraineeApp_Anae
r(all)= 0.222 p= 0.247 r(NonImp)= 0.222 Npairs=29 NimputedPairs=0
Key: Oxbridge X&Y valid
MRCGP_AKT
TraineeApp_Anaes
OXF
CAM
BRIST
NOTTM
EDINB
QUEEN
BIRM
CARDF
UCL
BRIGH
IMP
SHEF
SOTON
NEWC
LEEDS
LEICR
LOND
GLASG
MANU
SGEO
DUND
ABRDN
KINGS
KEELE
HLYK
LVRPL
QMARY
PNSLA
EANG

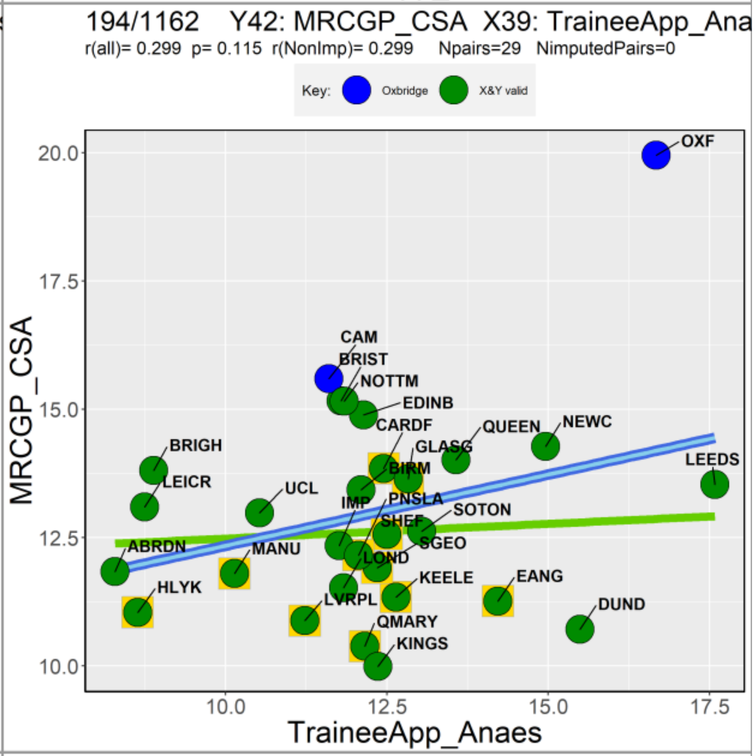
194/1162 Y42: MRCGP_CSA X39: TraineeApp_Ana
r(all)= 0.299 p= 0.115 r(NonImp)= 0.299 Npairs=29 NimputedPairs=0
Key: Oxbridge X&Y valid
MRCGP_CSA
TraineeApp_Anaes
OXF
CAM
BRIST
NOTTM
EDINB
CARDF
QUEEN
NEWC
BRIGH
GLASG
LEEDS
LEICR
BIRM
UCL
IMP
PNSLA
SOTON
SHEF
ABRDN
MANU
SGEO
LOND
HLYK
KEELE
EANG
LVRPL
DUND
QMARY
KINGS

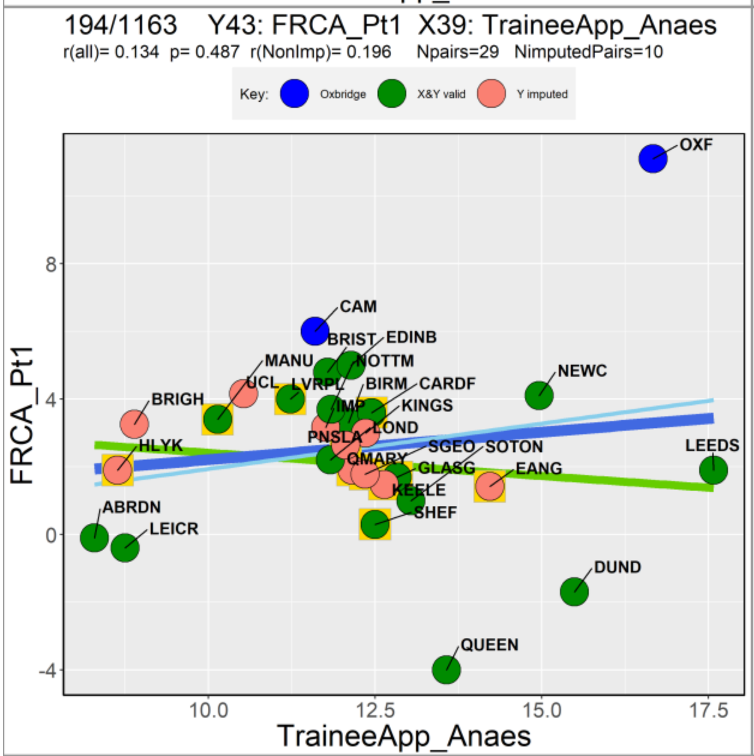
194/1163 Y43: FRCA_Pt1 X39: TraineeApp_Anaes
r(all)= 0.134 p= 0.487 r(NonImp)= 0.196 Npairs=29 NimputedPairs=10
Key: Oxbridge X&Y valid Y imputed
FRCA_Pt1
TraineeApp_Anaes
OXF
CAM
BRIST
EDINB
MANU
NOTTM
NEWC
UCL
LVRPL
BIRM
CARDF
BRIGH
IMP
KINGS
LOND
PNSLA
HLYK
SGEO
SOTON
LEEDS
QMARY
GLASG
EANG
KEELE
SHEF
ABRDN
LEICR
DUND
QUEEN

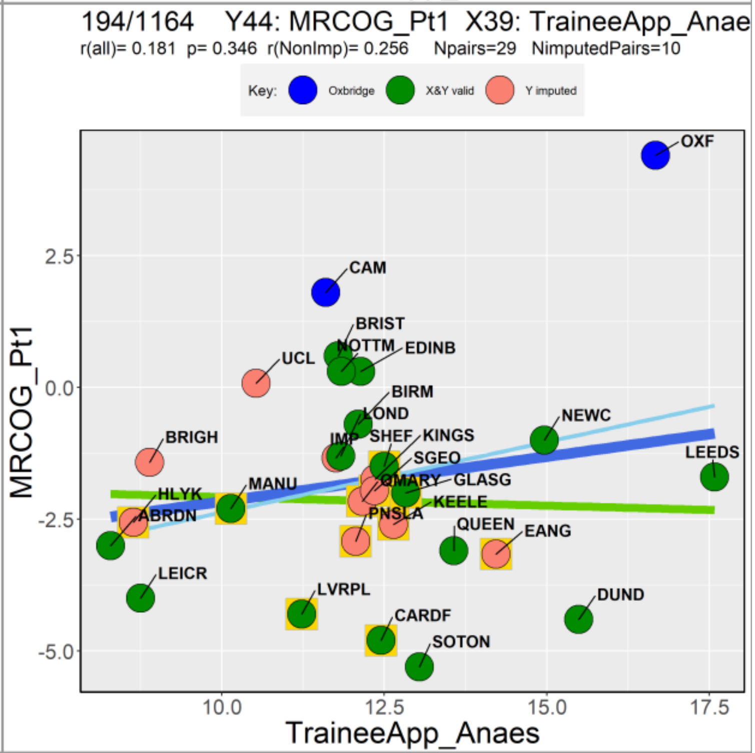
194/1164 Y44: MRCOG_Pt1 X39: TraineeApp_Anae
r(all)= 0.181 p= 0.346 r(NonImp)= 0.256 Npairs=29 NimputedPairs=10
Key: Oxbridge X&Y valid Y imputed
MRCOG_Pt1
TraineeApp_Anaes
OXF
CAM
BRIST
UCL
NOTTM
EDINB
BIRM
LOND
NEWC
BRIGH
IMP
SHEF
KINGS
SGEO
LEEDS
QMARY
GLASG
HLYK
MANU
KEELE
ABRDN
PNSLA
QUEEN
EANG
LEICR
LVRPL
DUND
CARDF
SOTON

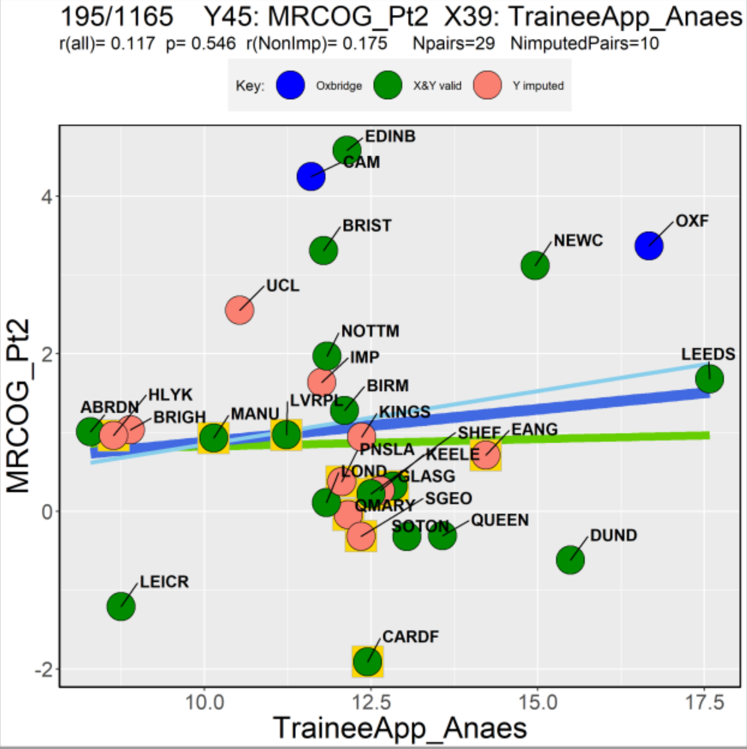
195/1165 Y45: MRCOG_Pt2 X39: TraineeApp_Anaes
r(all)= 0.117 p= 0.546 r(NonImp)= 0.175 Npairs=29 NimputedPairs=10
Key: Oxbridge X&Y valid Y imputed
MRCOG_Pt2
TraineeApp_Anaes
EDINB
CAM
BRIST
OXF
NEWC
UCL
NOTTM
IMP
LEEDS
BIRM
ABRDN
HLYK
BRIGH
MANU
LVRPL
KINGS
SHEF
EANG
PNSLA
KEELE
LOND
GLASG
SGEO
QMARY
SOTON
QUEEN
DUND
LEICR
CARDF

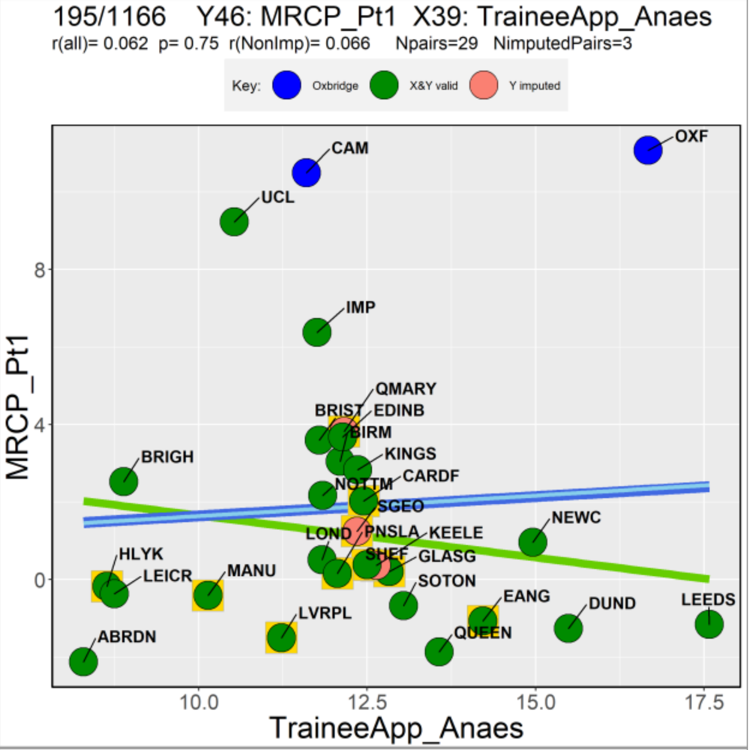
195/1166 Y46: MRCP_Pt1 X39: TraineeApp_Anaes
r(all)= 0.062 p= 0.75 r(NonImp)= 0.066 Npairs=29 NimputedPairs=3
Key: Oxbridge X&Y valid Y imputed
MRCP_Pt1
TraineeApp_Anaes
CAM
OXF
UCL
IMP
QMARY
BRIST
EDINB
BIRM
KINGS
BRIGH
NOTTM
CARDF
SGEO
NEWC
LOND
PNSLA
KEELE
HLYK
SHEF
GLASG
LEICR
MANU
SOTON
EANG
DUND
LEEDS
LVRPL
QUEEN
ABRDN

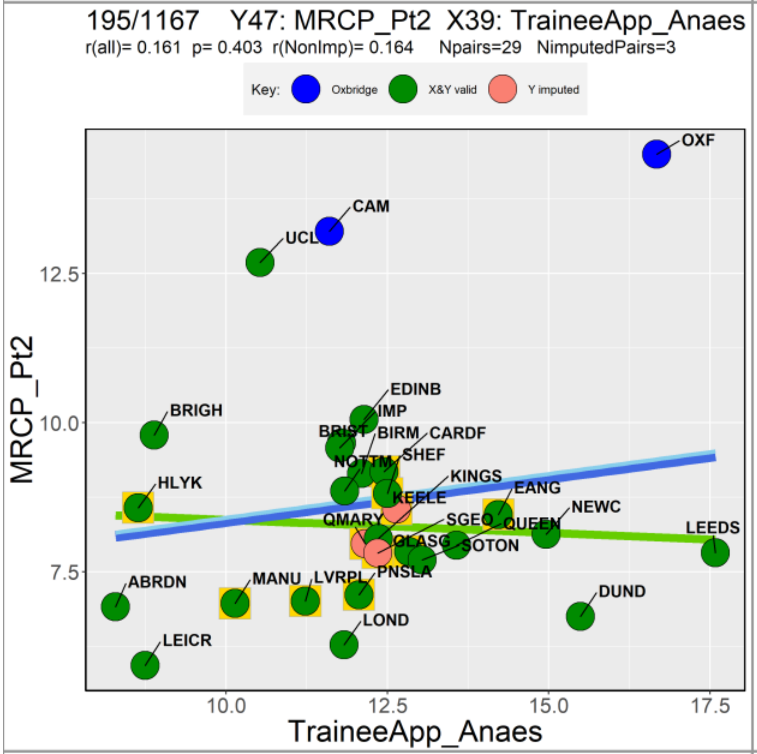
195/1167 Y47: MRCP_Pt2 X39: TraineeApp_Anaes
r(all)= 0.161 p= 0.403 r(NonImp)= 0.164 Npairs=29 NimputedPairs=3
Key: Oxbridge X&Y valid Y imputed
MRCP_Pt2
TraineeApp_Anaes
OXF
CAM
UCL
EDINB
IMP
BRIGH
BRIST
BIRM
CARDF
NOTTM
SHEF
KINGS
HLYK
EANG
KEELE
NEWC
QMARY
SGEO
QUEEN
GLASG
SOTON
LEEDS
PNSLA
ABRDN
MANU
LVRPL
DUND
LOND
LEICR

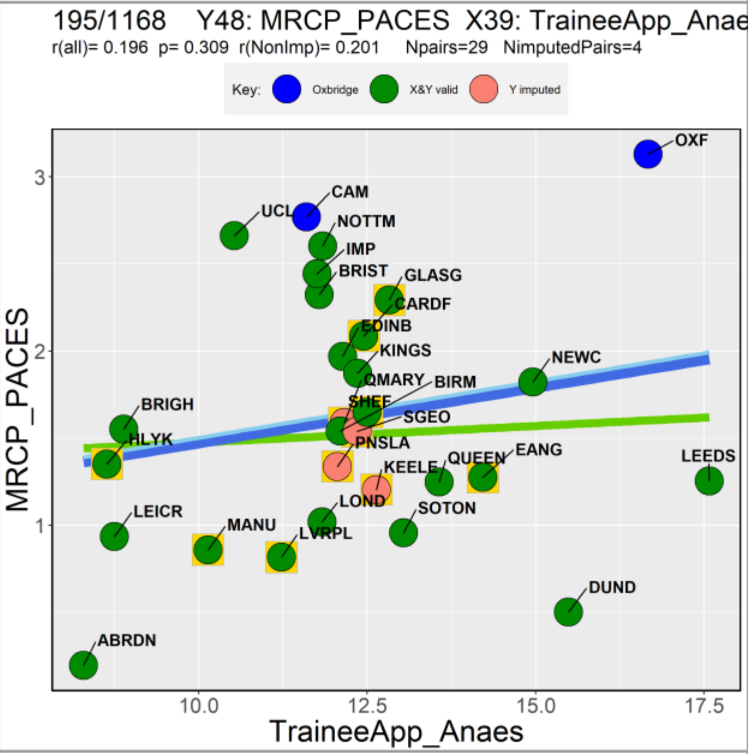
195/1168 Y48: MRCP_PACES X39: TraineeApp_Anae
r(all)= 0.196 p= 0.309 r(NonImp)= 0.201 Npairs=29 NimputedPairs=4
Key: Oxbridge X&Y valid Y imputed
MRCP_PACES
TraineeApp_Anaes
OXF
CAM
UCL
NOTTM
IMP
BRIST
GLASG
CARDF
EDINB
KINGS
NEWC
QMARY
BIRM
BRIGH
SHEF
SGEO
HLYK
PNSLA
QUEEN
EANG
KEELE
LEEDS
LOND
SOTON
LEICR
MANU
LVRPL
DUND
ABRDN

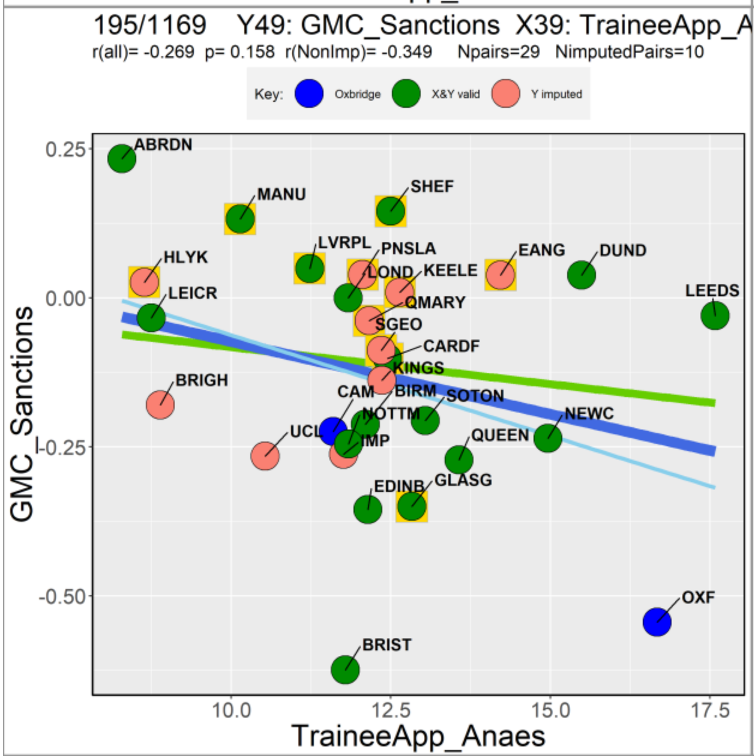
195/1169 Y49: GMC_Sanctions X39: TraineeApp_A
r(all)= -0.269 p= 0.158 r(NonImp)= -0.349 Npairs=29 NimputedPairs=10
Key: Oxbridge X&Y valid Y imputed
GMC_Sanctions
TraineeApp_Anaes
ABRDN
MANU
SHEF
LVRPL
PNSLA
HLYK
LOND
KEELE
EANG
DUND
LEICR
QMARY
LEEDS
SGEO
CARDF
KINGS
BRIGH
CAM
BIRM
SOTON
NOTTM
NEWC
UCL
IMP
QUEEN
EDINB
GLASG
OXF
BRIST

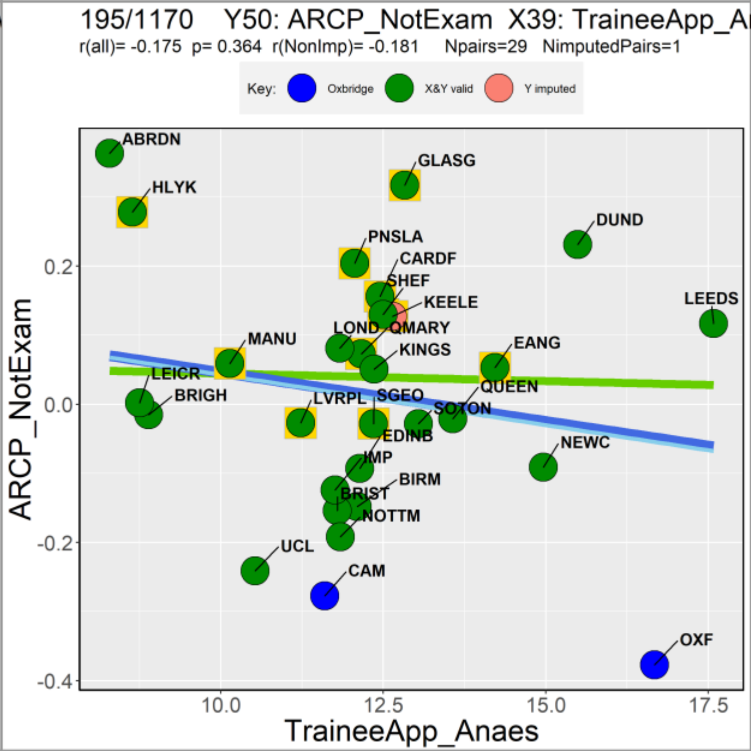
195/1170 Y50: ARCP_NotExam X39: TraineeApp_A
r(all)= -0.175 p= 0.364 r(NonImp)= -0.181 Npairs=29 NimputedPairs=1
Key: Oxbridge X&Y valid Y imputed
ARCP_NotExam
TraineeApp_Anaes
ABRDN
GLASG
HLYK
DUND
PNSLA
CARDF
SHEF
KEELE
LEEDS
LOND
QMARY
MANU
KINGS
EANG
LEICR
BRIGH
LVRPL
SGEO
QUEEN
SOTON
EDINB
NEWC
IMP
BIRM
BRIST
NOTTM
UCL
CAM
OXF

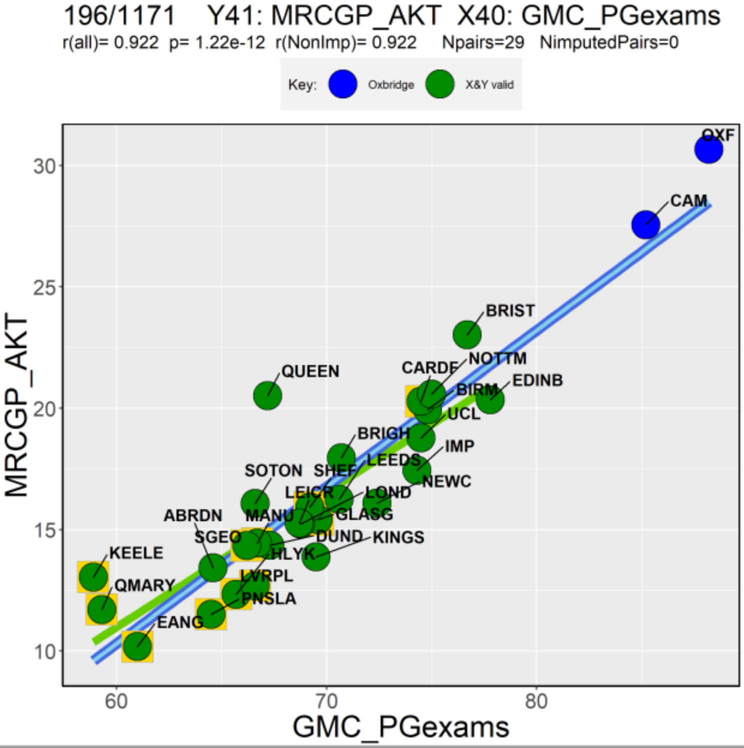
196/1171 Y41: MRCGP_AKT X40: GMC_PGexams
r(all)= 0.922 p= 1.22e-12 r(NonImp)= 0.922 Npairs=29 NimputedPairs=0
Key: Oxbridge X&Y valid
MRCGP_AKT
GMC_PGexams
OXF CAM BRIST QUEEN CARDF NOTTM BIRM EDINB UCL BRIGH IMP LEEDS SOTON SHEF NEWC LEICR LOND ABRDN MANU GLASG SGEO DUND KINGS HLYK KEELE LVRPL PNSLA QMARY EANG

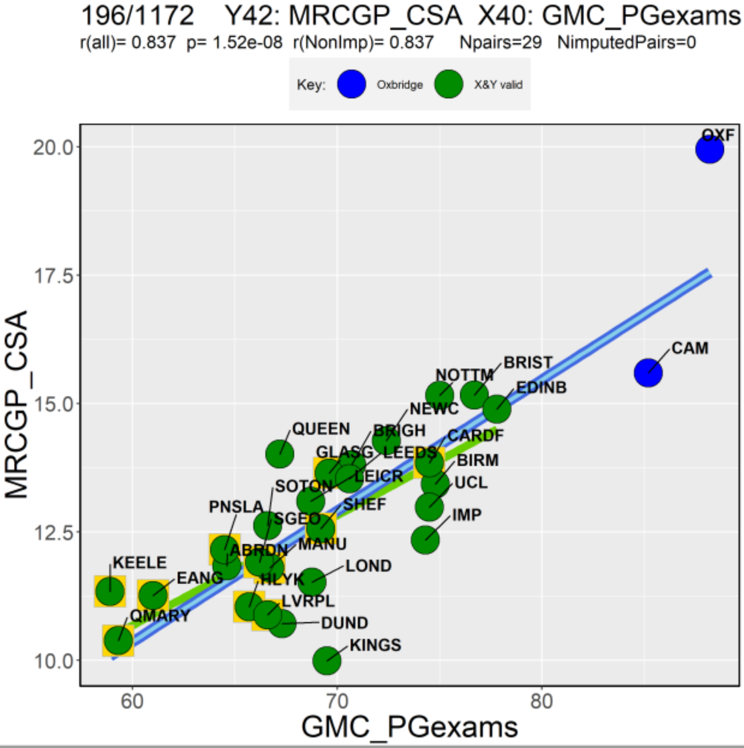
196/1172 Y42: MRCGP_CSA X40: GMC_PGexams
r(all)= 0.837 p= 1.52e-08 r(NonImp)= 0.837 Npairs=29 NimputedPairs=0
Key: Oxbridge X&Y valid
MRCGP_CSA
GMC_PGexams
OXF CAM NOTTM BRIST EDINB NEWC QUEEN BRIGH CARDF GLASG LEEDS BIRM LEICR UCL SOTON SHEF IMP PNSLA SGEO MANU ABRDN KEELE EANG HLYK LOND LVRPL QMARY DUND KINGS

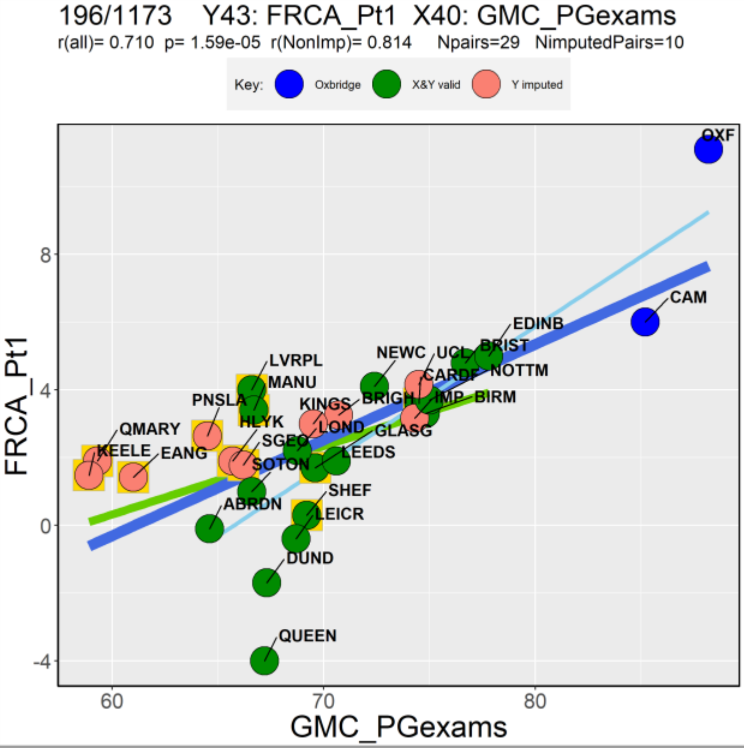
196/1173 Y43: FRCA_Pt1 X40: GMC_PGexams
r(all)= 0.710 p= 1.59e-05 r(NonImp)= 0.814 Npairs=29 NimputedPairs=10
Key: Oxbridge X&Y valid Y imputed
FRCA_Pt1
GMC_PGexams
OXF CAM EDINB BRIST NEWC UCL CARDF NOTTM LVRPL MANU KINGS BRIGH IMP BIRM PNSLA HLYK LOND GLASG QMARY SGEO KEELE EANG SOTON LEEDS SHEF ABRDN LEICR DUND QUEEN

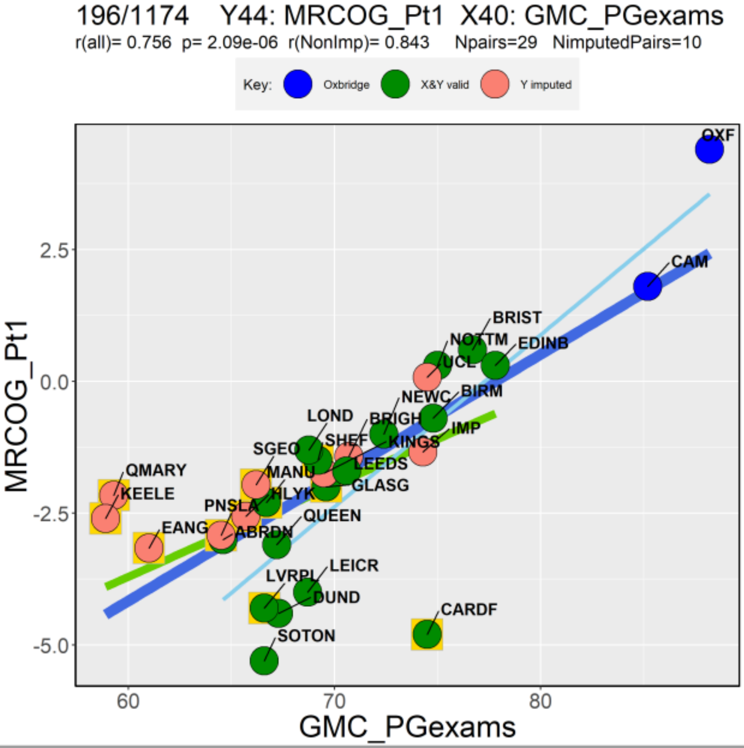
196/1174 Y44: MRCOG_Pt1 X40: GMC_PGexams
r(all)= 0.756 p= 2.09e-06 r(NonImp)= 0.843 Npairs=29 NimputedPairs=10
Key: Oxbridge X&Y valid Y imputed
MRCOG_Pt1
GMC_PGexams
OXF CAM BRIST NOTTM EDINB UCL NEWC BIRM LOND BRIGH IMP SGEO SHEF KINGS MANU LEEDS QMARY HLYK GLASG KEELE PNSLA EANG ABRDN QUEEN LEICR LVRPL DUND CARDF SOTON

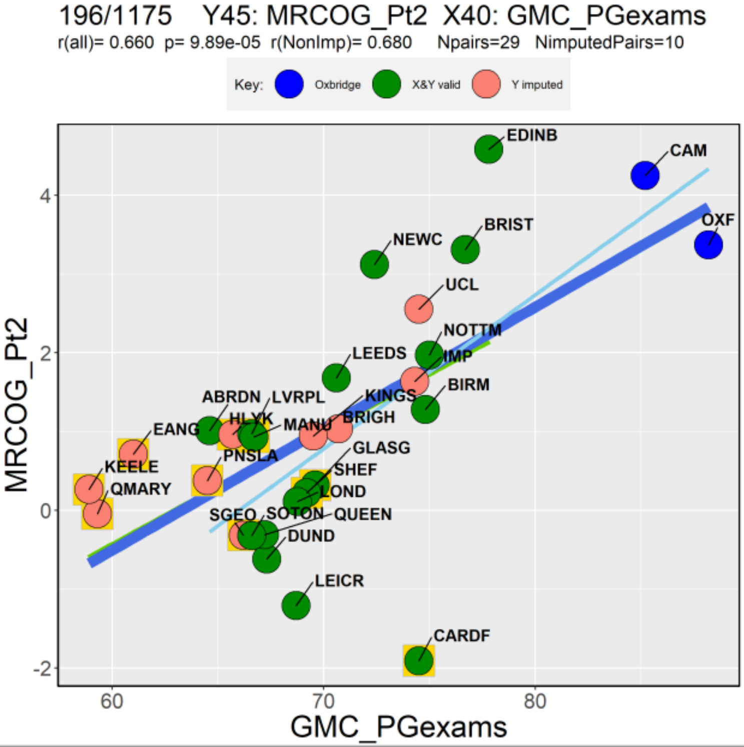
196/1175 Y45: MRCOG_Pt2 X40: GMC_PGexams
r(all)= 0.660 p= 9.89e-05 r(NonImp)= 0.680 Npairs=29 NimputedPairs=10
Key: Oxbridge X&Y valid Y imputed
MRCOG_Pt2
GMC_PGexams
EDINB CAM OXF BRIST NEWC UCL NOTTM LEEDS IMP BIRM ABRDN LVRPL KINGS EANG HLYK MANU BRIGH GLASG PNSLA SHEF KEELE LOND QMARY SGEO SOTON QUEEN DUND LEICR CARDF

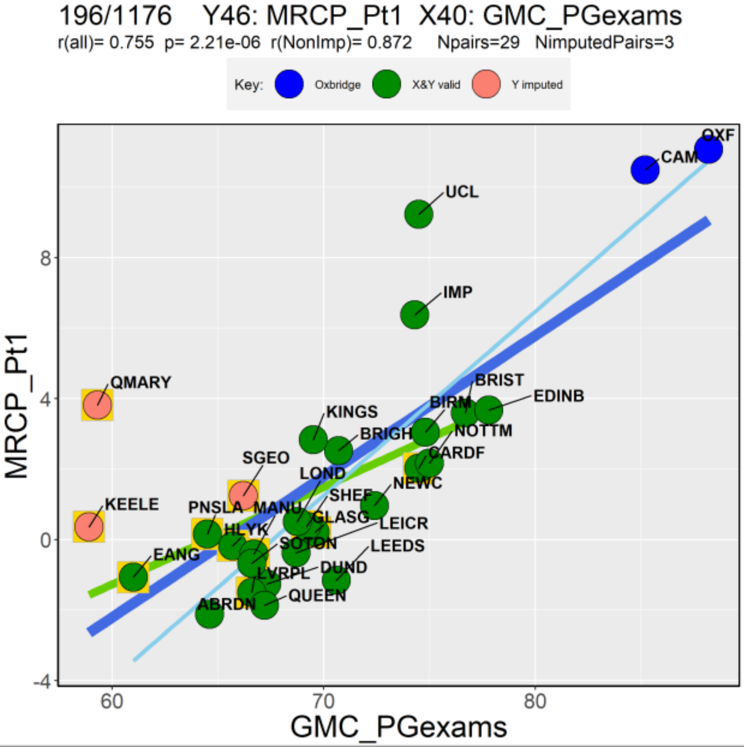
196/1176 Y46: MRCP_Pt1 X40: GMC_PGexams
r(all)= 0.755 p= 2.21e-06 r(NonImp)= 0.872 Npairs=29 NimputedPairs=3
Key: Oxbridge X&Y valid Y imputed
MRCP_Pt1
GMC_PGexams
OXF CAM UCL IMP QMARY BRIST EDINB BIRM KINGS BRIGH NOTTM CARDF SGEO NEWC LOND SHEF KEELE PNSLA MANU GLASG LEICR HLYK SOTON LEEDS EANG LVRPL DUND ABRDN QUEEN

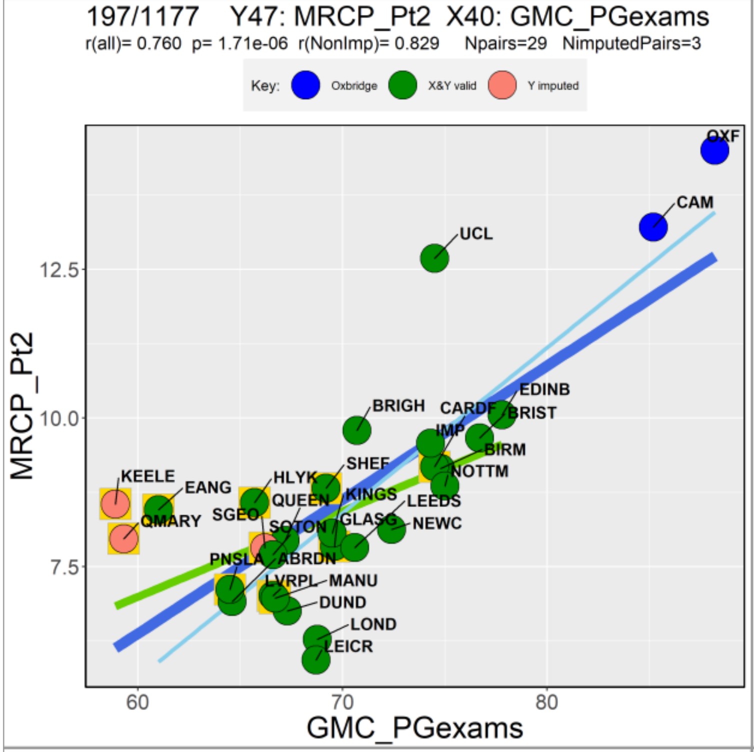
197/1177 Y47: MRCP_Pt2 X40: GMC_PGexams
r(all)= 0.760 p= 1.71e-06 r(NonImp)= 0.829 Npairs=29 NimputedPairs=3
Key: Oxbridge X&Y valid Y imputed
MRCP_Pt2
GMC_PGexams

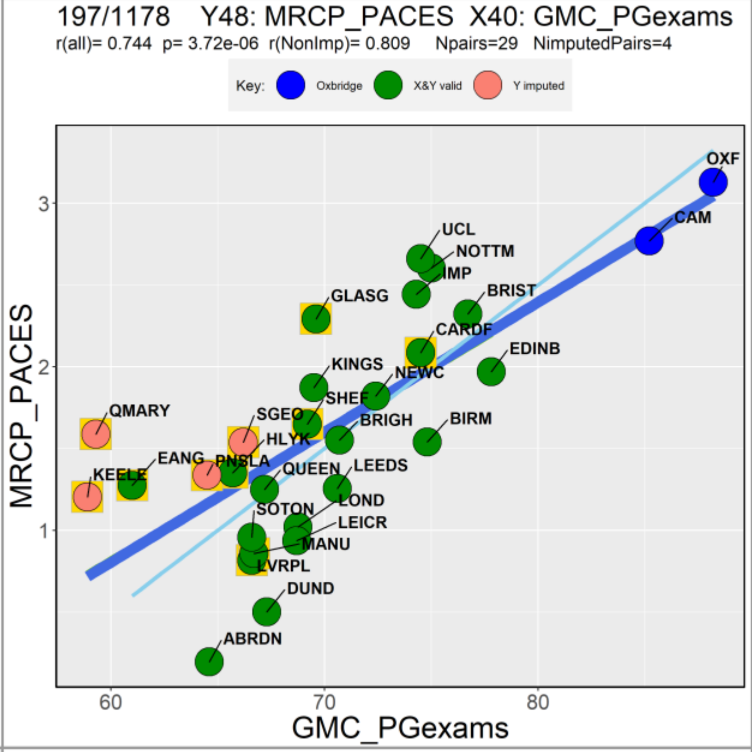
197/1178 Y48: MRCP_PACES X40: GMC_PGexams
r(all)= 0.744 p= 3.72e-06 r(NonImp)= 0.809 Npairs=29 NimputedPairs=4
Key: Oxbridge X&Y valid Y imputed
MRCP_PACES
GMC_PGexams

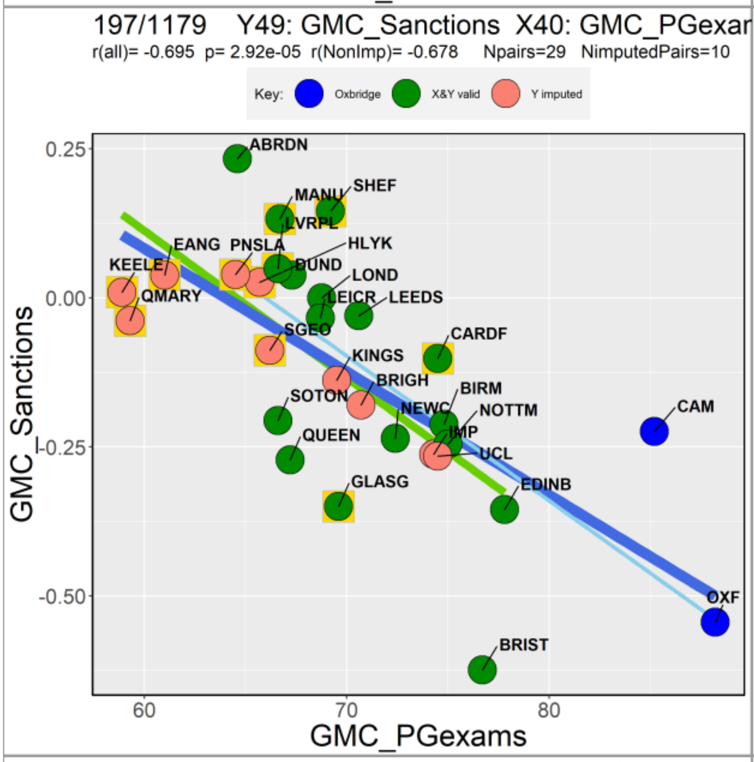
197/1179 Y49: GMC_Sanctions X40: GMC_PGexar
r(all)= -0.695 p= 2.92e-05 r(NonImp)= -0.678 Npairs=29 NimputedPairs=10
Key: Oxbridge X&Y valid Y imputed
GMC_Sanctions
GMC_PGexams

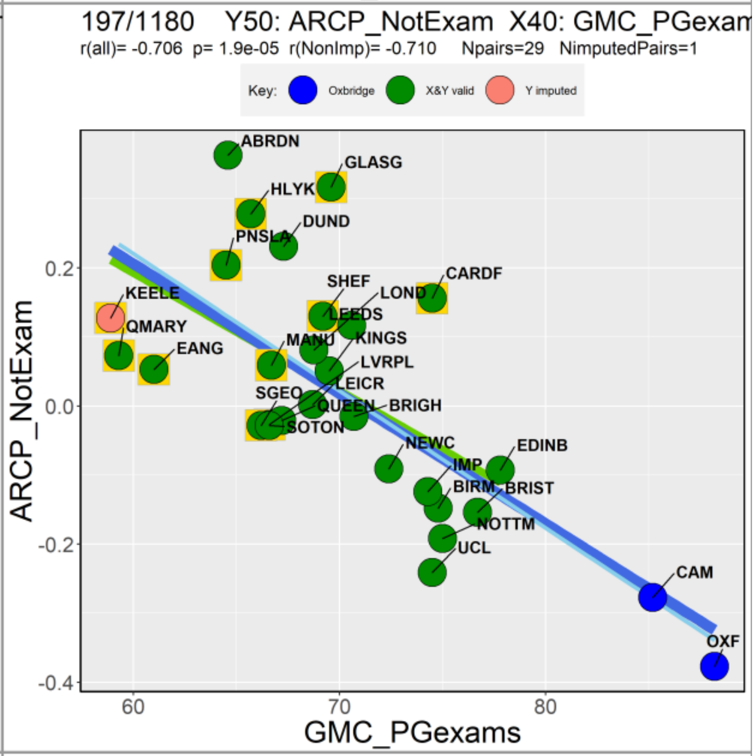
197/1180 Y50: ARCP_NotExam X40: GMC_PGexam
r(all)= -0.706 p= 1.9e-05 r(NonImp)= -0.710 Npairs=29 NimputedPairs=1
Key: Oxbridge X&Y valid Y imputed
ARCP_NotExam
GMC_PGexams

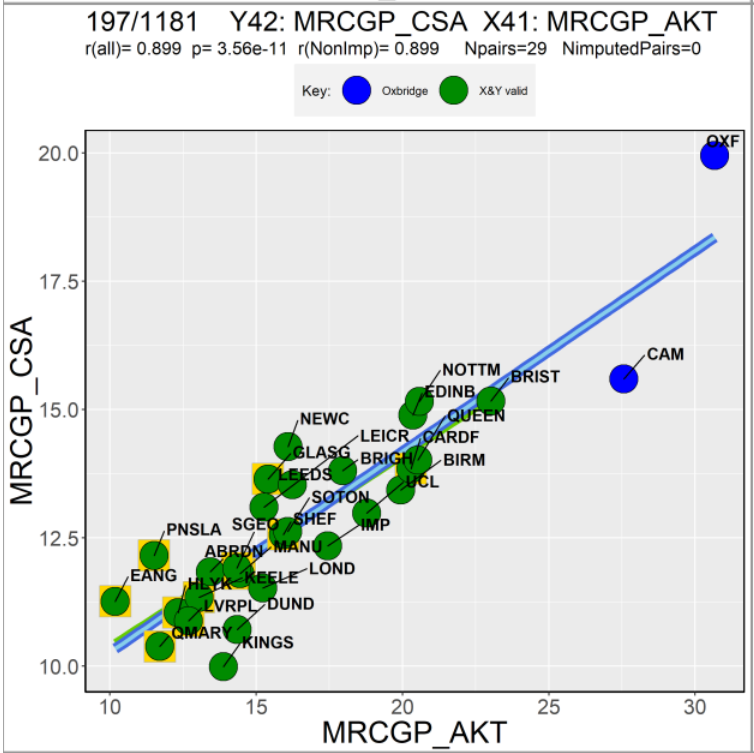
197/1181 Y42: MRCGP_CSA X41: MRCGP_AKT
r(all)= 0.899 p= 3.56e-11 r(NonImp)= 0.899 Npairs=29 NimputedPairs=0
Key: Oxbridge X&Y valid
MRCGP_CSA
MRCGP_AKT

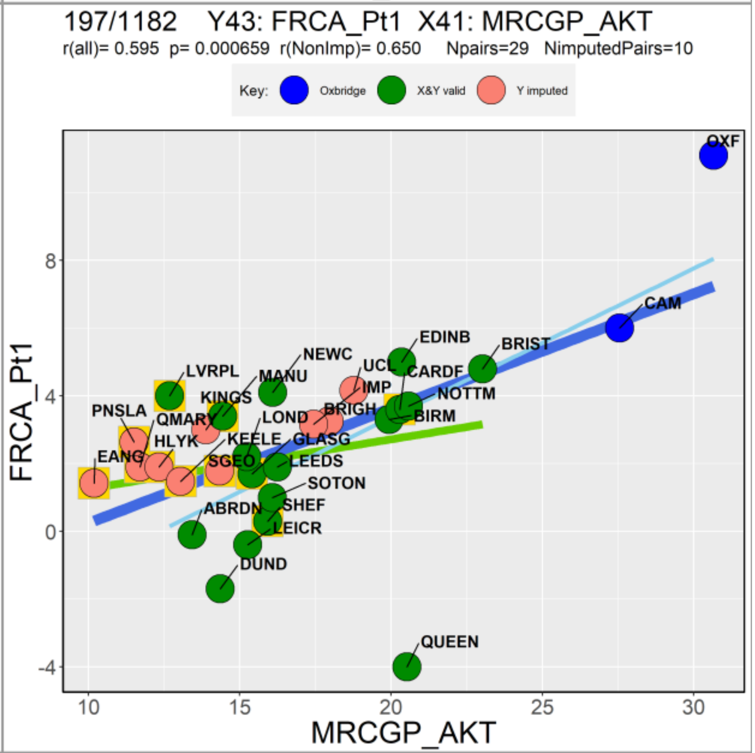
197/1182 Y43: FRCA_Pt1 X41: MRCGP_AKT
r(all)= 0.595 p= 0.000659 r(NonImp)= 0.650 Npairs=29 NimputedPairs=10
Key: Oxbridge X&Y valid Y imputed
FRCA_Pt1
MRCGP_AKT

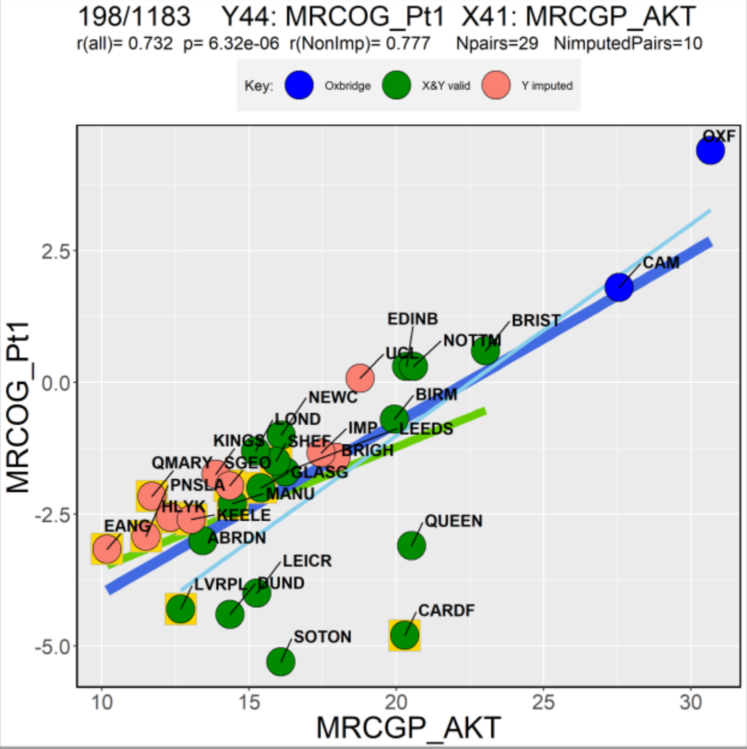
198/1183 Y44: MRCOG_Pt1 X41: MRCGP_AKT
r(all)= 0.732 p= 6.32e-06 r(NonImp)= 0.777 Npairs=29 NimputedPairs=10
Key: Oxbridge X&Y valid Y imputed
MRCOG_Pt1
MRCGP_AKT

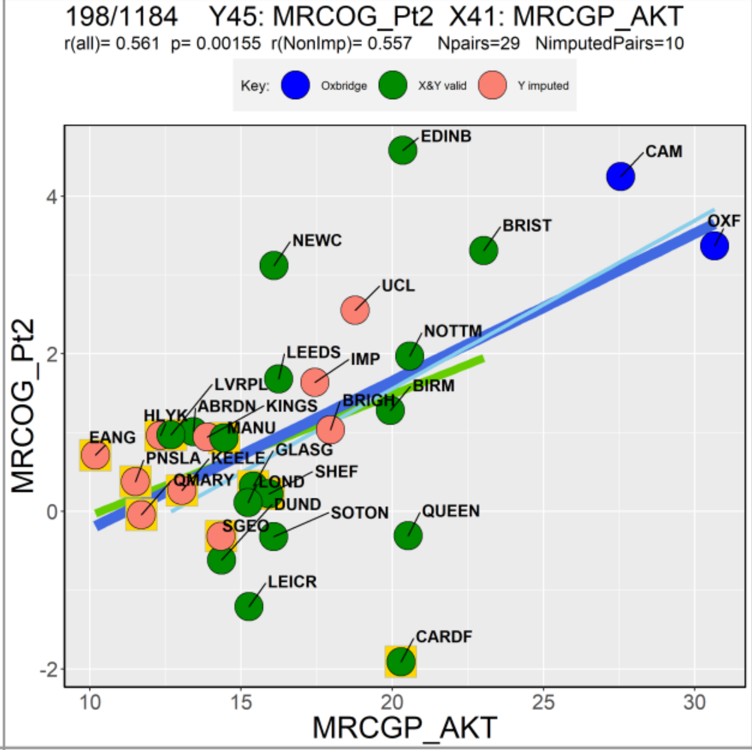
198/1184 Y45: MRCOG_Pt2 X41: MRCGP_AKT
r(all)= 0.561 p= 0.00155 r(NonImp)= 0.557 Npairs=29 NimputedPairs=10
Key: Oxbridge X&Y valid Y imputed
MRCOG_Pt2
MRCGP_AKT

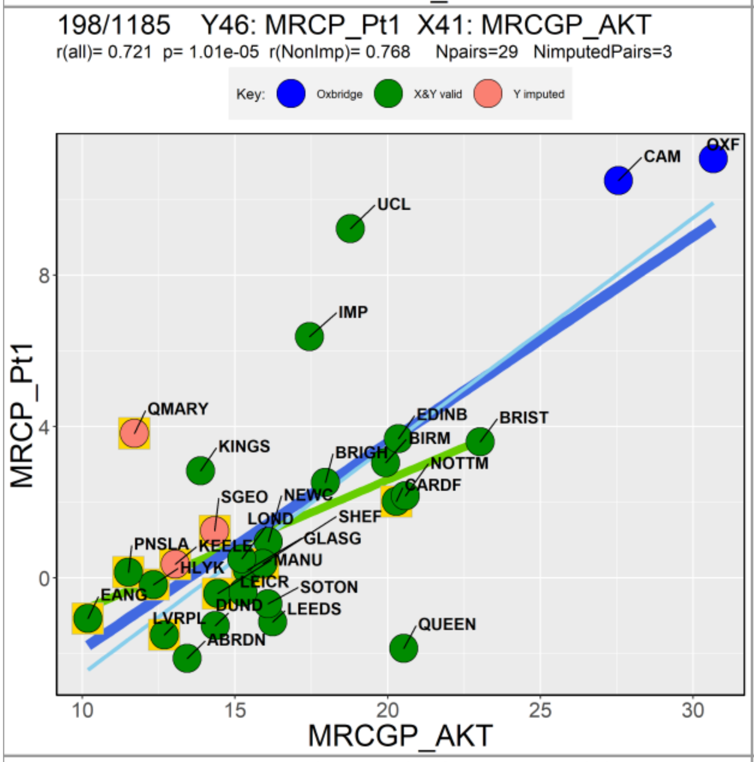
198/1185 Y46: MRCP_Pt1 X41: MRCGP_AKT
r(all)= 0.721 p= 1.01e-05 r(NonImp)= 0.768 Npairs=29 NimputedPairs=3
Key: Oxbridge X&Y valid Y imputed
MRCP_Pt1
MRCGP_AKT

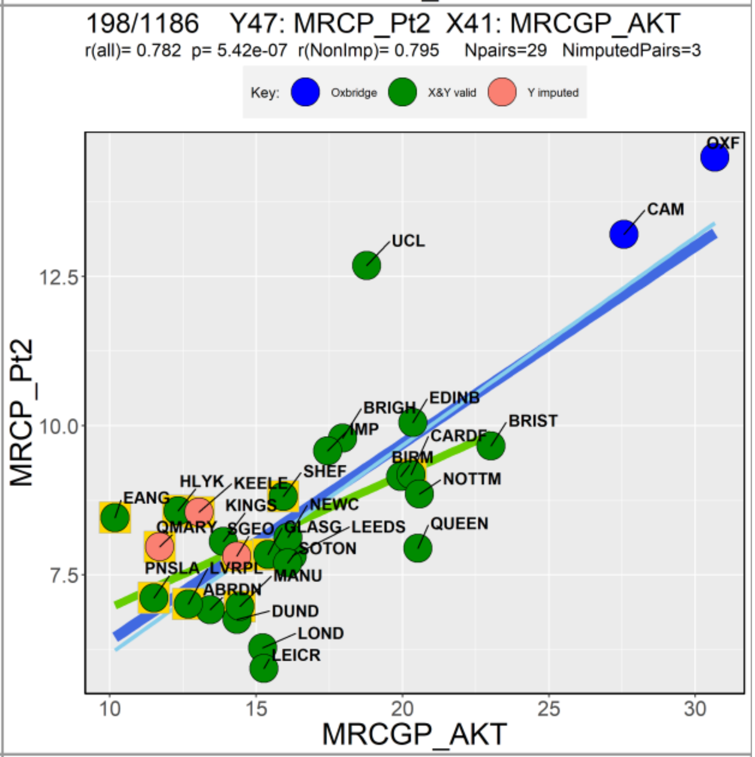
198/1186 Y47: MRCP_Pt2 X41: MRCGP_AKT
r(all)= 0.782 p= 5.42e-07 r(NonImp)= 0.795 Npairs=29 NimputedPairs=3
Key: Oxbridge X&Y valid Y imputed
MRCP_Pt2
MRCGP_AKT

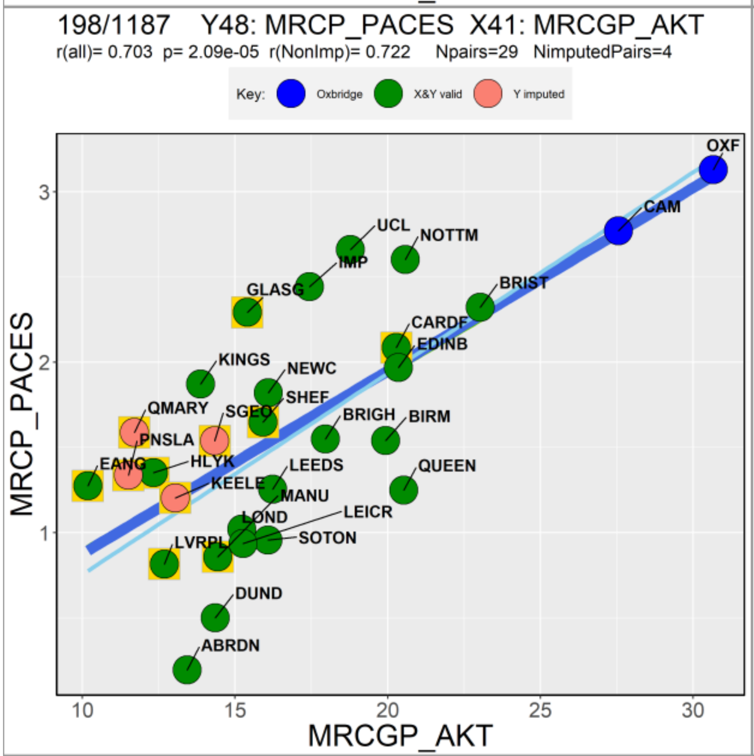
198/1187 Y48: MRCP_PACES X41: MRCGP_AKT
r(all)= 0.703 p= 2.09e-05 r(NonImp)= 0.722 Npairs=29 NimputedPairs=4
Key: Oxbridge X&Y valid Y imputed
MRCP_PACES
MRCGP_AKT

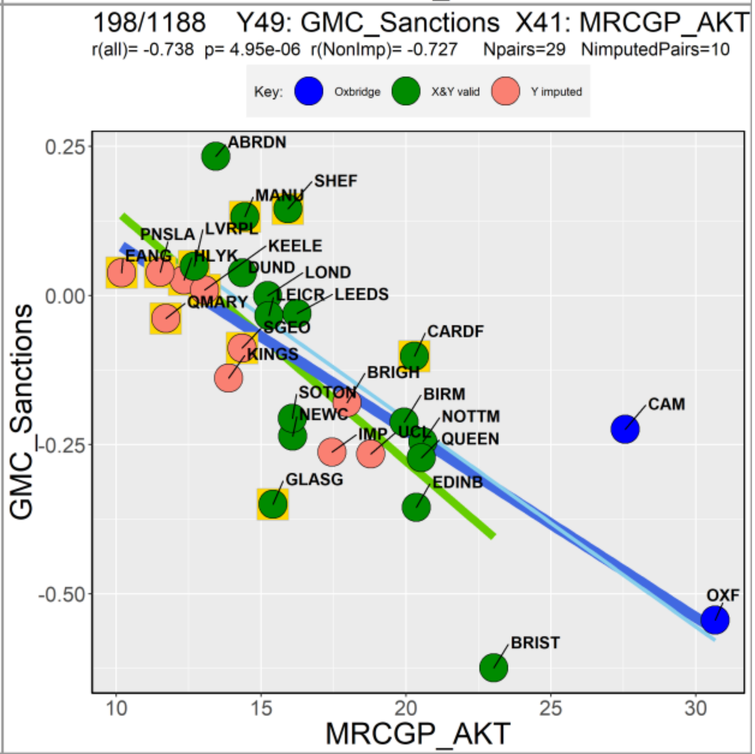
198/1188 Y49: GMC_Sanctions X41: MRCGP_AKT
r(all)= -0.738 p= 4.95e-06 r(NonImp)= -0.727 Npairs=29 NimputedPairs=10
Key: Oxbridge X&Y valid Y imputed
GMC_Sanctions
MRCGP_AKT

199/1189 Y50: ARCP_NotExam X41: MRCGP_AKT
r(all)= -0.728 p= 7.69e-06 r(NonImp)= -0.723 Npairs=29 NimputedPairs=1

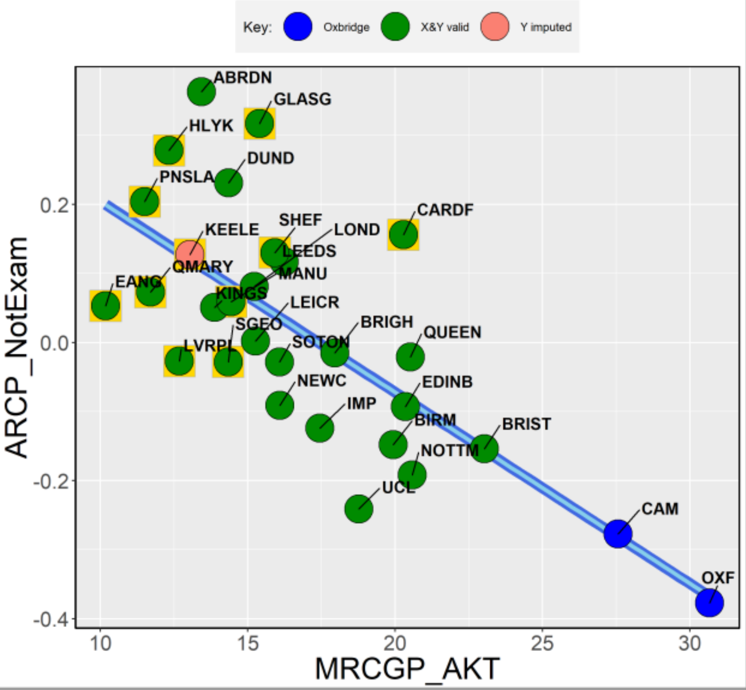

199/1190 Y43: FRCA_Pt1 X42: MRCGP_CSA
r(all)= 0.614 p= 0.000394 r(NonImp)= 0.694 Npairs=29 NimputedPairs=10

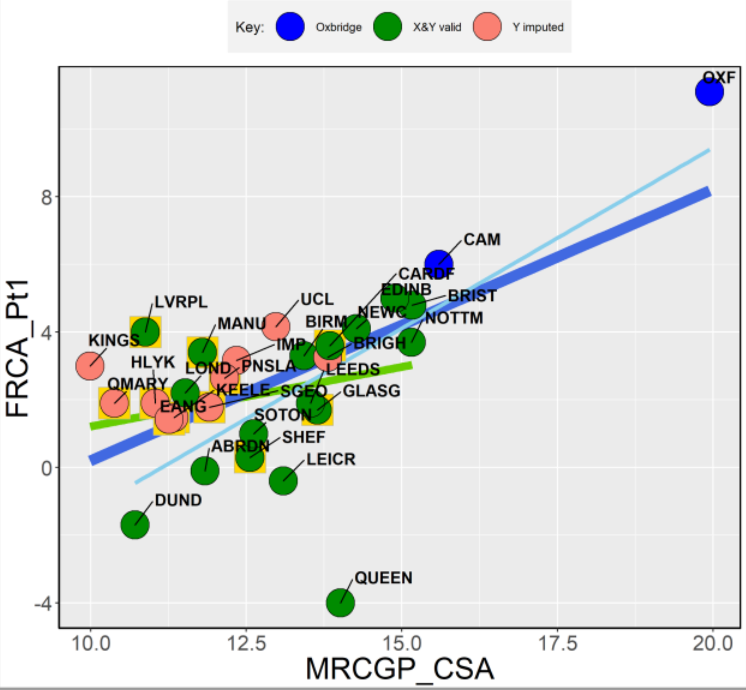

199/1191 Y44: MRCOG_Pt1 X42: MRCGP_CSA
r(all)= 0.717 p= 1.19e-05 r(NonImp)= 0.805 Npairs=29 NimputedPairs=10

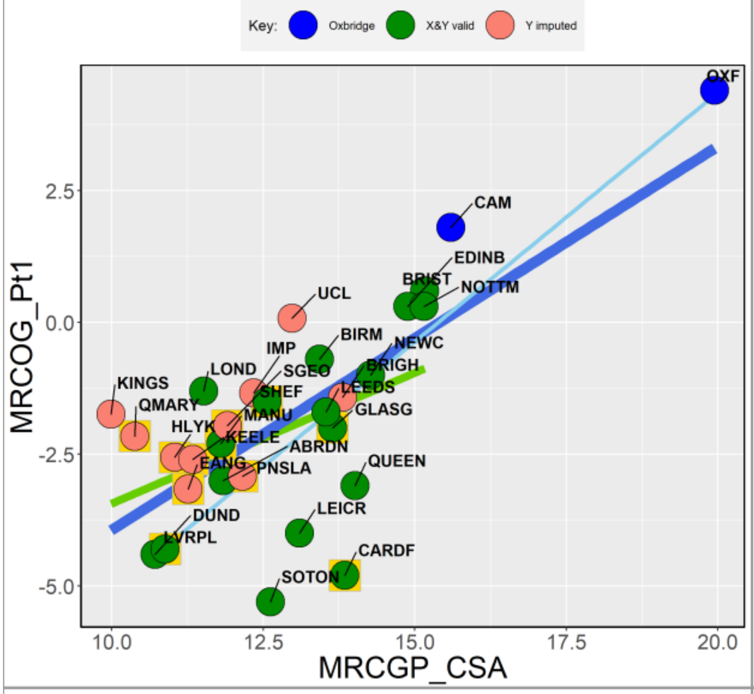

199/1192 Y45: MRCOG_Pt2 X42: MRCGP_CSA
r(all)= 0.561 p= 0.00155 r(NonImp)= 0.590 Npairs=29 NimputedPairs=10

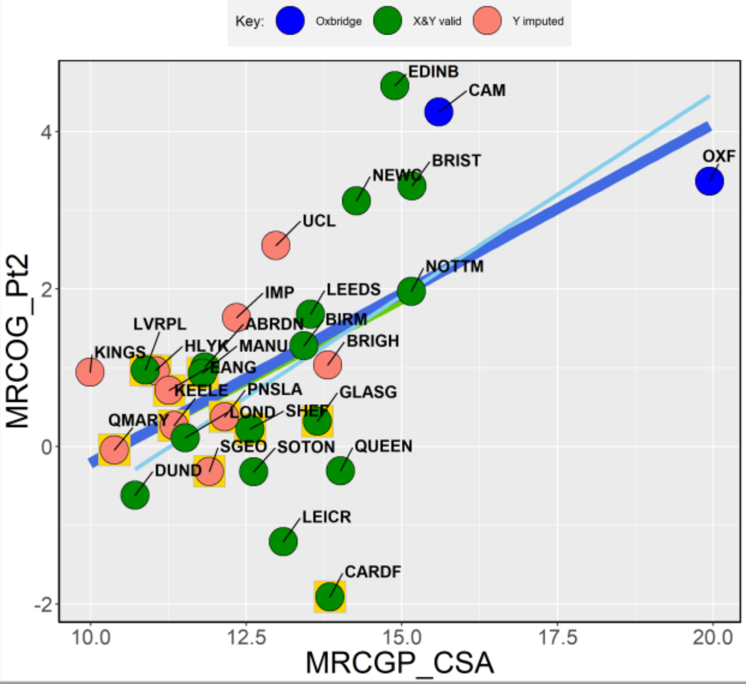

199/1193 Y46: MRCP_Pt1 X42: MRCGP_CSA
r(all)= 0.574 p= 0.00114 r(NonImp)= 0.620 Npairs=29 NimputedPairs=3

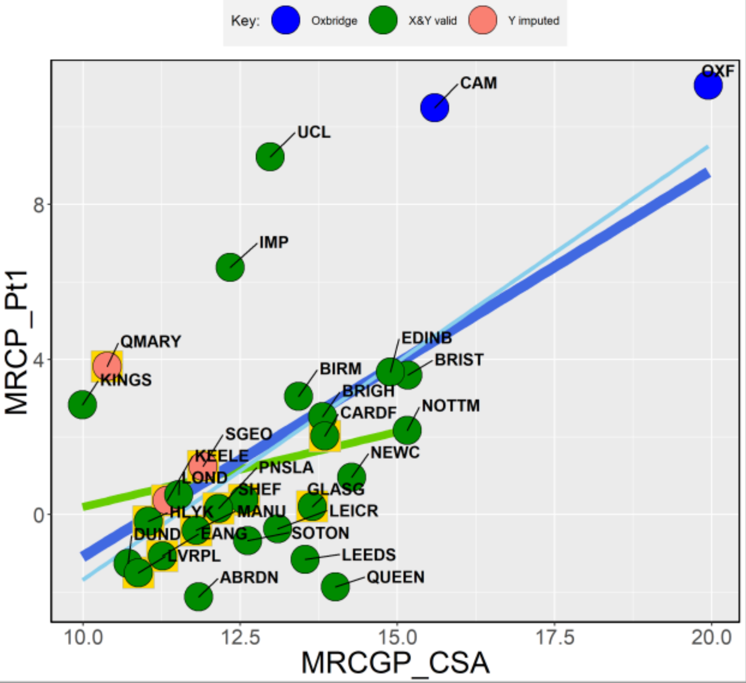

199/1194 Y47: MRCP_Pt2 X42: MRCGP_CSA
r(all)= 0.695 p= 2.85e-05 r(NonImp)= 0.706 Npairs=29 NimputedPairs=3

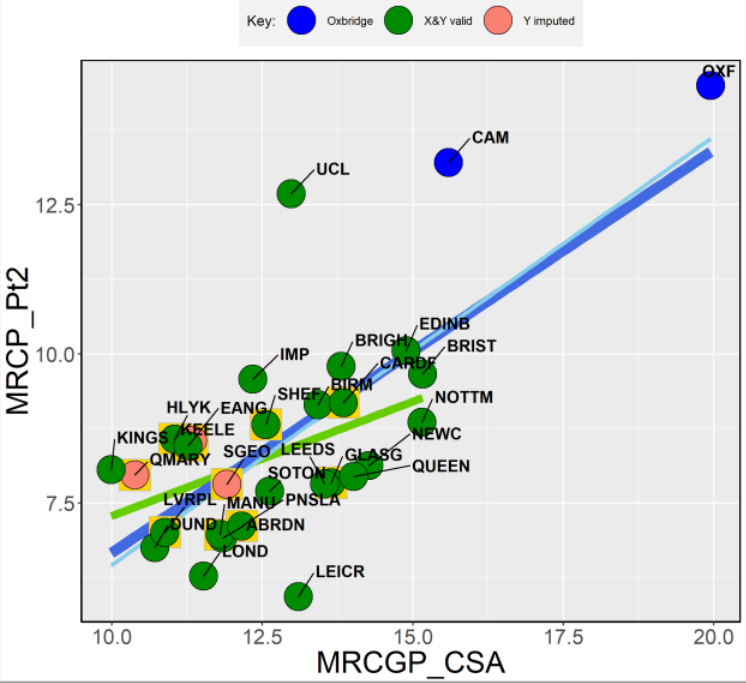

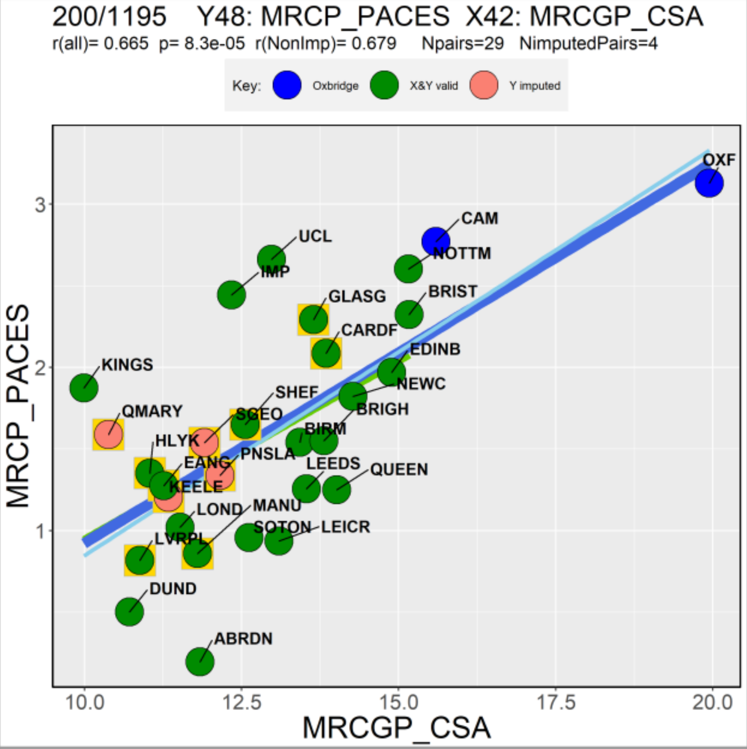
200/1195 Y48: MRCP_PACES X42: MRCGP_CSA
r(all)= 0.665 p= 8.3e-05 r(NonImp)= 0.679 Npairs=29 NimputedPairs=4
Key: Oxbridge X&Y valid Y imputed
MRCP_PACES
MRCGP_CSA

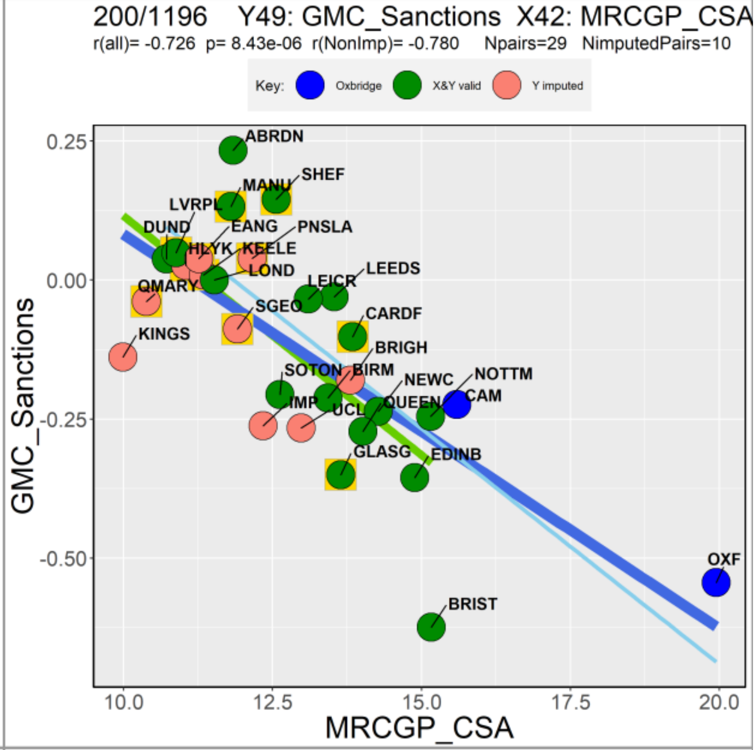
200/1196 Y49: GMC_Sanctions X42: MRCGP_CSA
r(all)= -0.726 p= 8.43e-06 r(NonImp)= -0.780 Npairs=29 NimputedPairs=10
Key: Oxbridge X&Y valid Y imputed
GMC_Sanctions
MRCGP_CSA

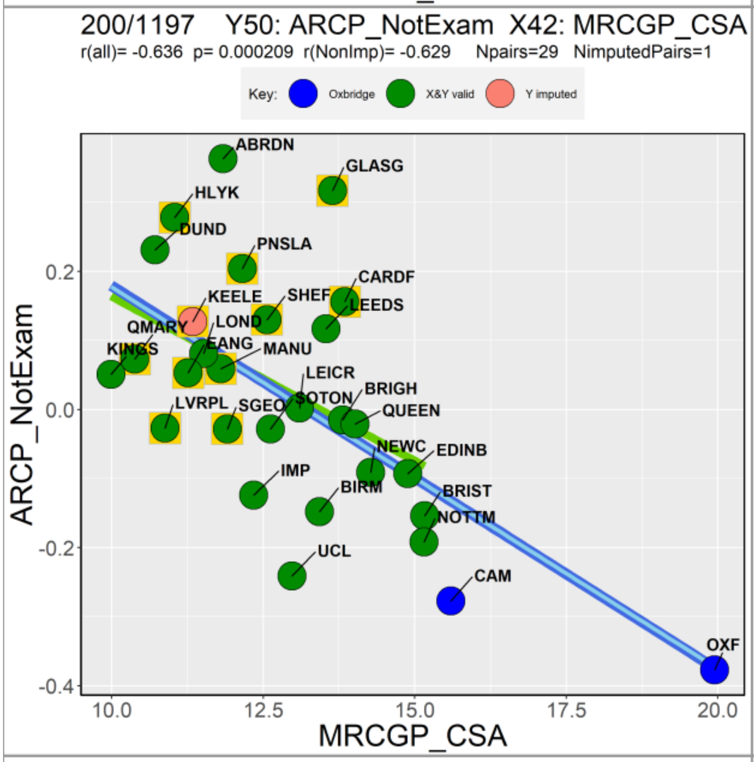
200/1197 Y50: ARCP_NotExam X42: MRCGP_CSA
r(all)= -0.636 p= 0.000209 r(NonImp)= -0.629 Npairs=29 NimputedPairs=1
Key: Oxbridge X&Y valid Y imputed
ARCP_NotExam
MRCGP_CSA

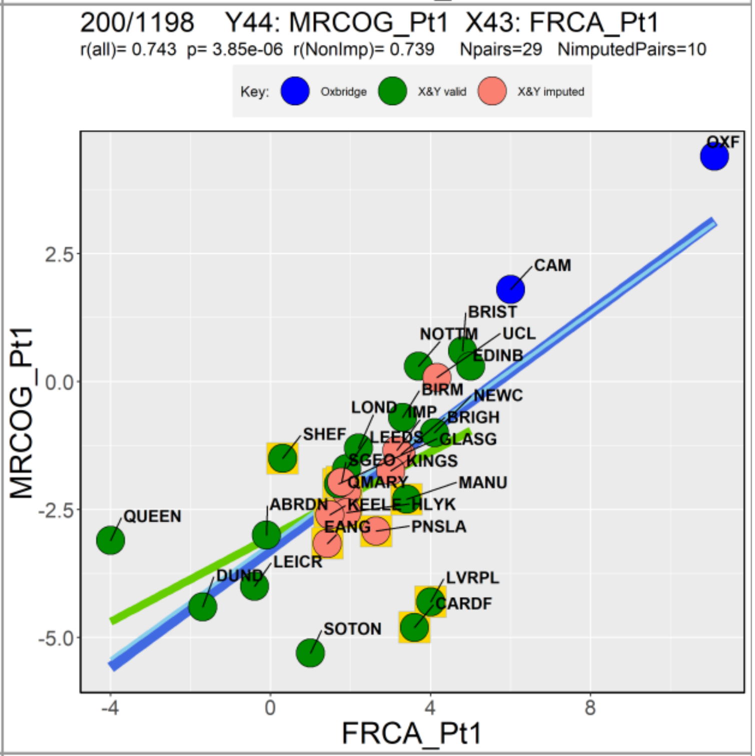
200/1198 Y44: MRCOG_Pt1 X43: FRCA_Pt1
r(all)= 0.743 p= 3.85e-06 r(NonImp)= 0.739 Npairs=29 NimputedPairs=10
Key: Oxbridge X&Y valid X&Y imputed
MRCOG_Pt1
FRCA_Pt1

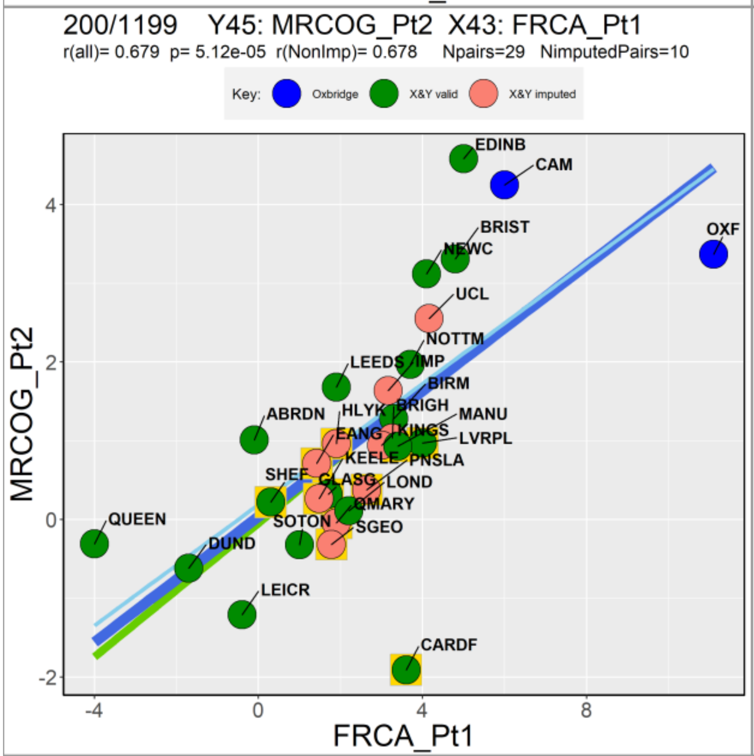
200/1199 Y45: MRCOG_Pt2 X43: FRCA_Pt1
r(all)= 0.679 p= 5.12e-05 r(NonImp)= 0.678 Npairs=29 NimputedPairs=10
Key: Oxbridge X&Y valid X&Y imputed
MRCOG_Pt2
FRCA_Pt1

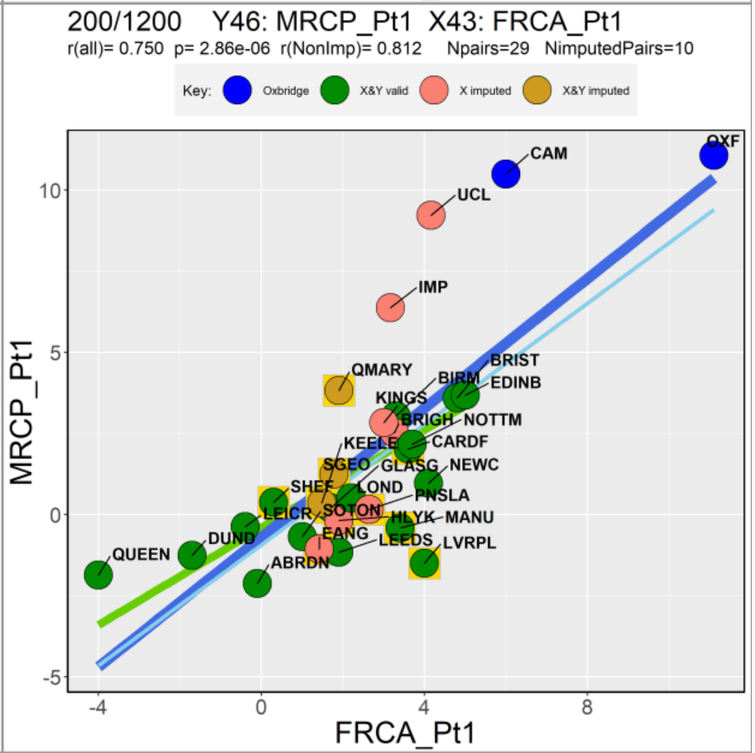
200/1200 Y46: MRCP_Pt1 X43: FRCA_Pt1
r(all)= 0.750 p= 2.86e-06 r(NonImp)= 0.812 Npairs=29 NimputedPairs=10
Key: Oxbridge X&Y valid X imputed X&Y imputed
MRCP_Pt1
FRCA_Pt1

201/1201 Y47: MRCP_Pt2 X43: FRCA_Pt1

r(all)= 0.718 p= 1.14e-05 r(NonImp)= 0.754 Npairs=29 NimputedPairs=10

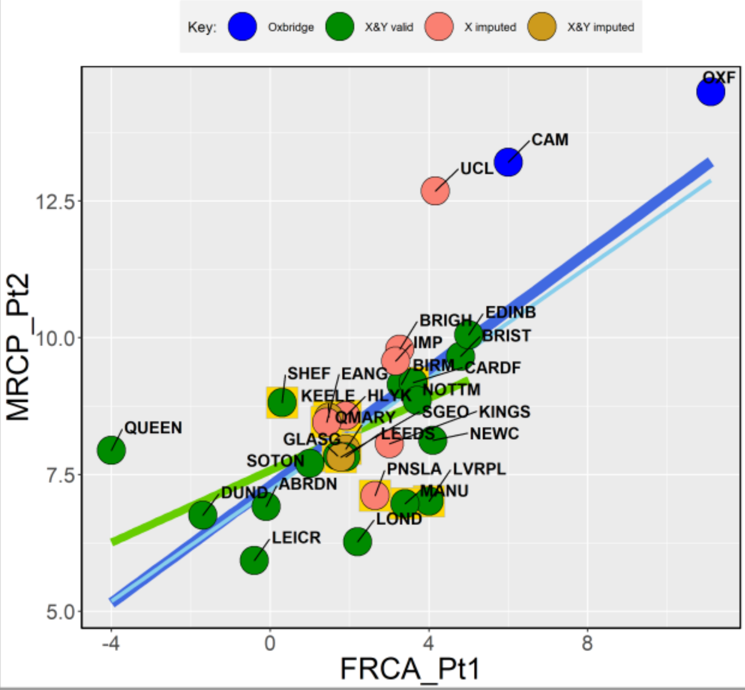

201/1202 Y48: MRCP_PACES X43: FRCA_Pt1

r(all)= 0.689 p= 3.59e-05 r(NonImp)= 0.700 Npairs=29 NimputedPairs=10

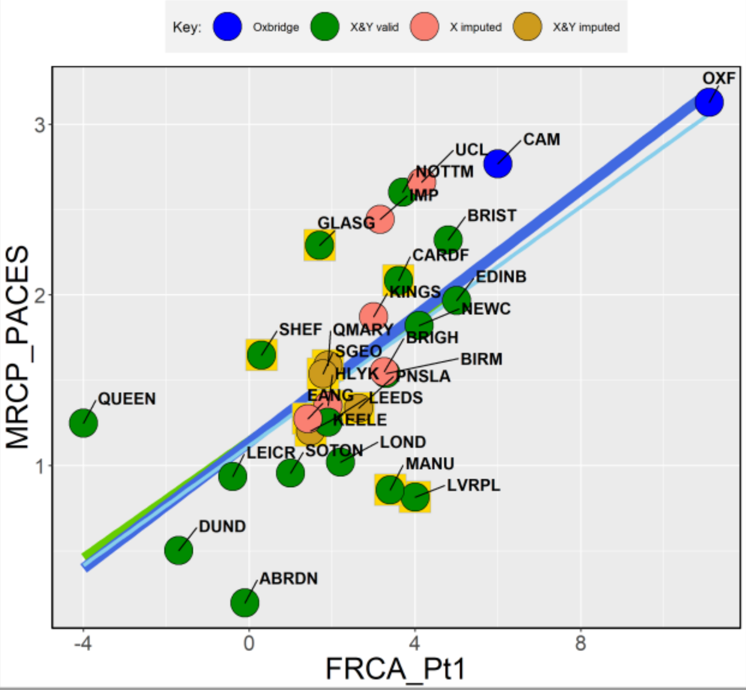

201/1203 Y49: GMC_Sanctions X43: FRCA_Pt1

r(all)= -0.521 p= 0.00375 r(NonImp)= -0.508 Npairs=29 NimputedPairs=10

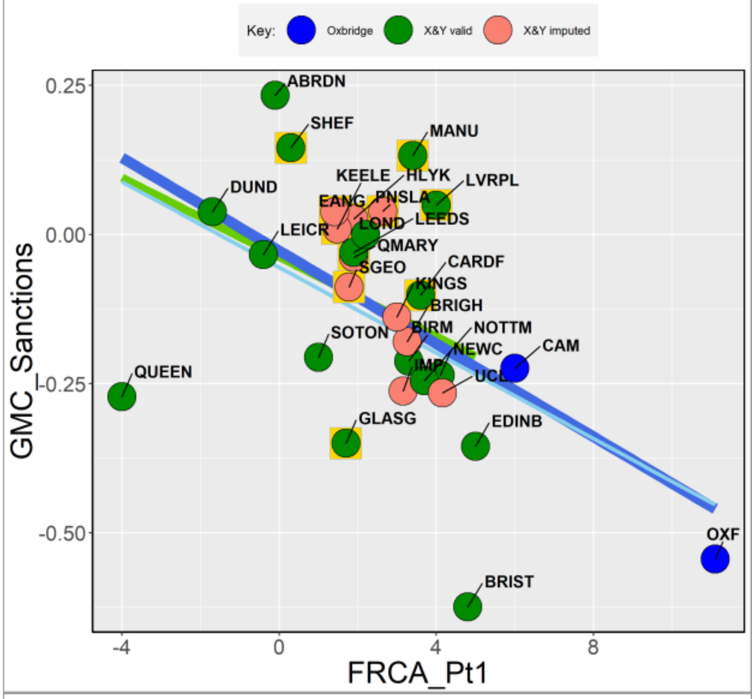

201/1204 Y50: ARCP_NotExam X43: FRCA_Pt1

r(all)= -0.631 p= 0.000245 r(NonImp)= -0.664 Npairs=29 NimputedPairs=10

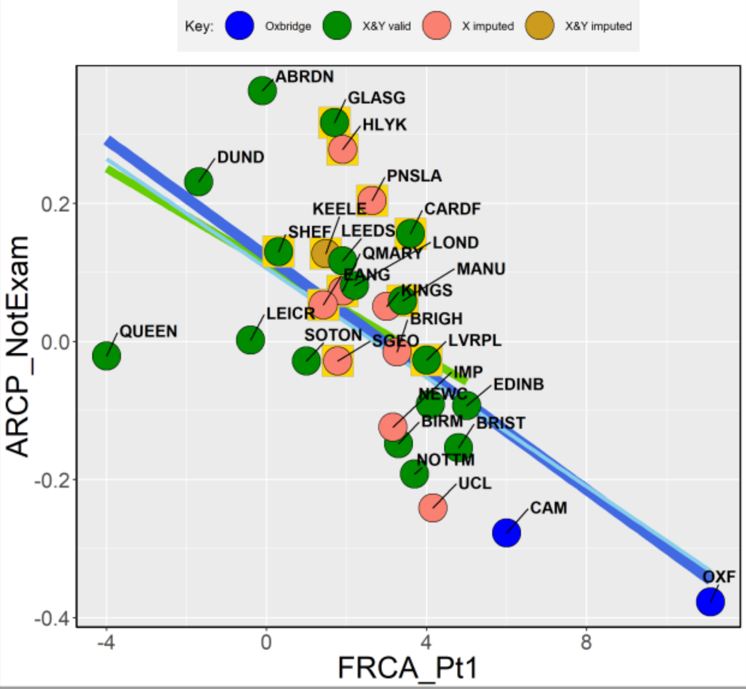

201/1205 Y45: MRCOG_Pt2 X44: MRCOG_Pt1

r(all)= 0.807 p= 1.2e-07 r(NonImp)= 0.815 Npairs=29 NimputedPairs=10

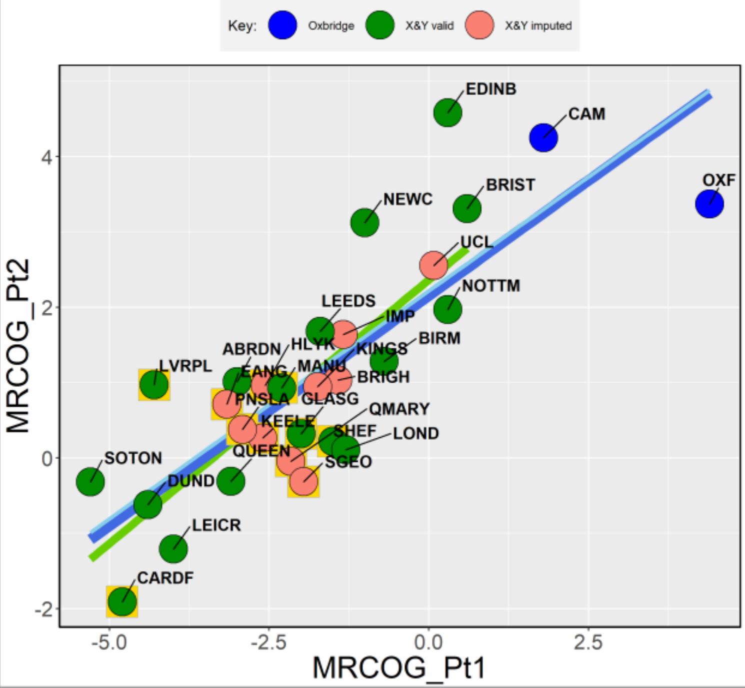

201/1206 Y46: MRCP_Pt1 X44: MRCOG_Pt1

r(all)= 0.790 p= 3.44e-07 r(NonImp)= 0.830 Npairs=29 NimputedPairs=10

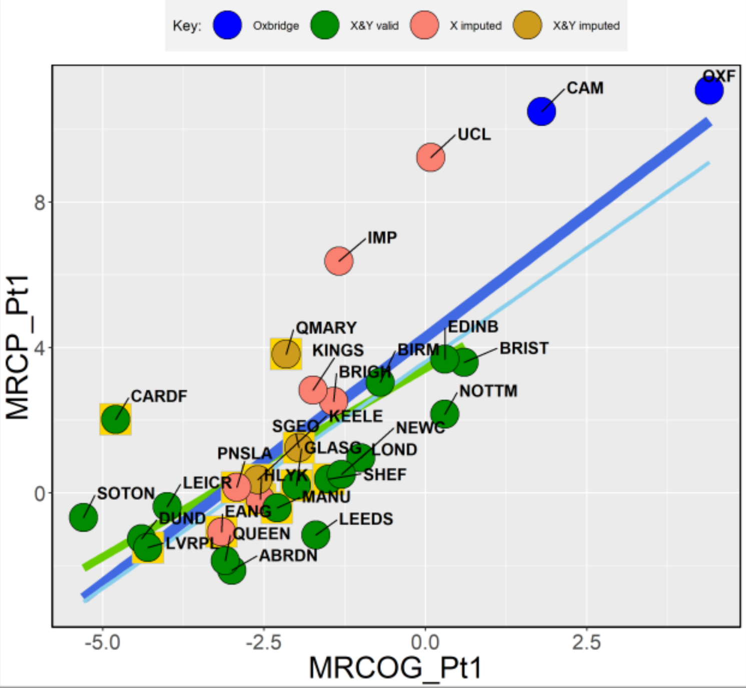

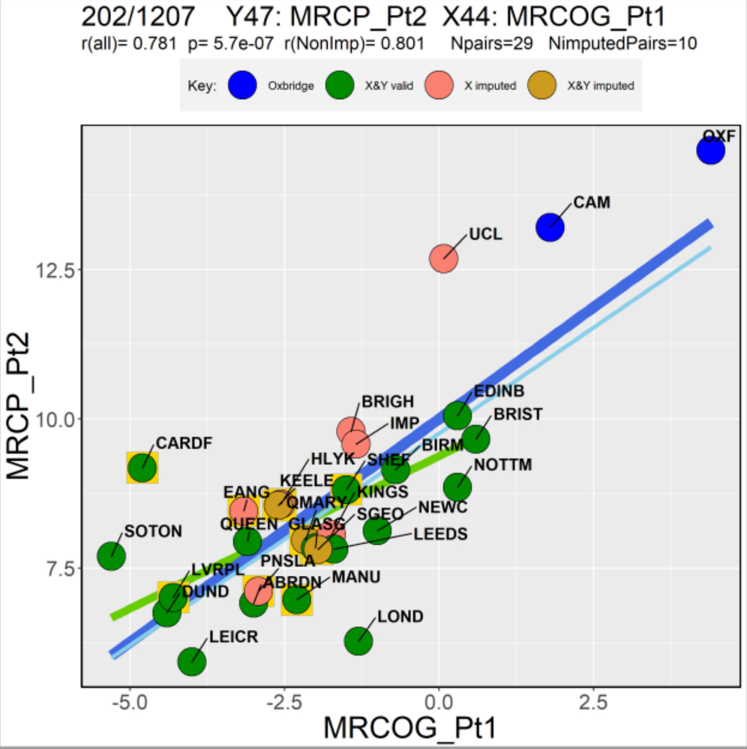
202/1207 Y47: MRCP_Pt2 X44: MRCOG_Pt1
r(all)= 0.781 p= 5.7e-07 r(NonImp)= 0.801 Npairs=29 NimputedPairs=10
Key: Oxbridge X&Y valid X imputed X&Y imputed
MRCP_Pt2
MRCOG_Pt1

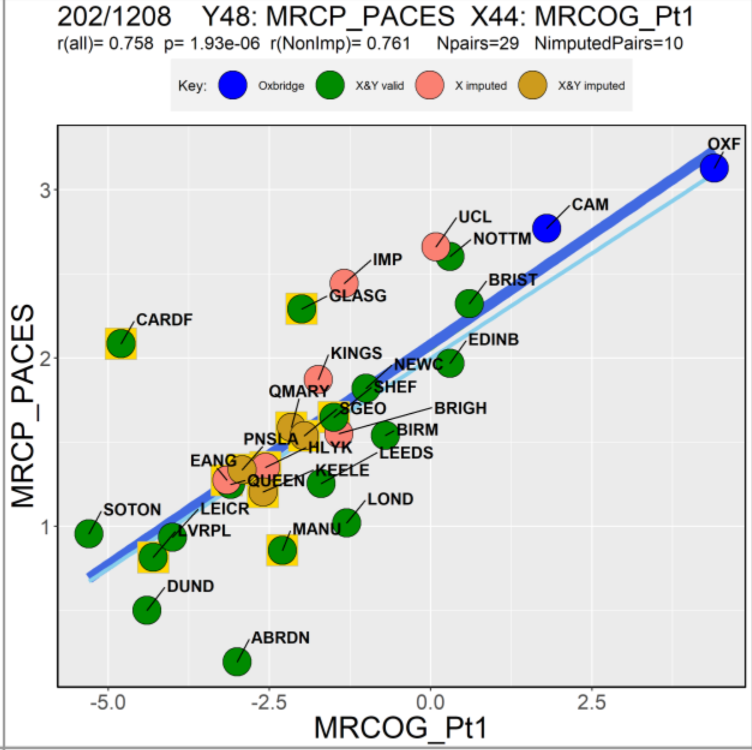
202/1208 Y48: MRCP_PACES X44: MRCOG_Pt1
r(all)= 0.758 p= 1.93e-06 r(NonImp)= 0.761 Npairs=29 NimputedPairs=10
Key: Oxbridge X&Y valid X imputed X&Y imputed
MRCP_PACES
MRCOG_Pt1

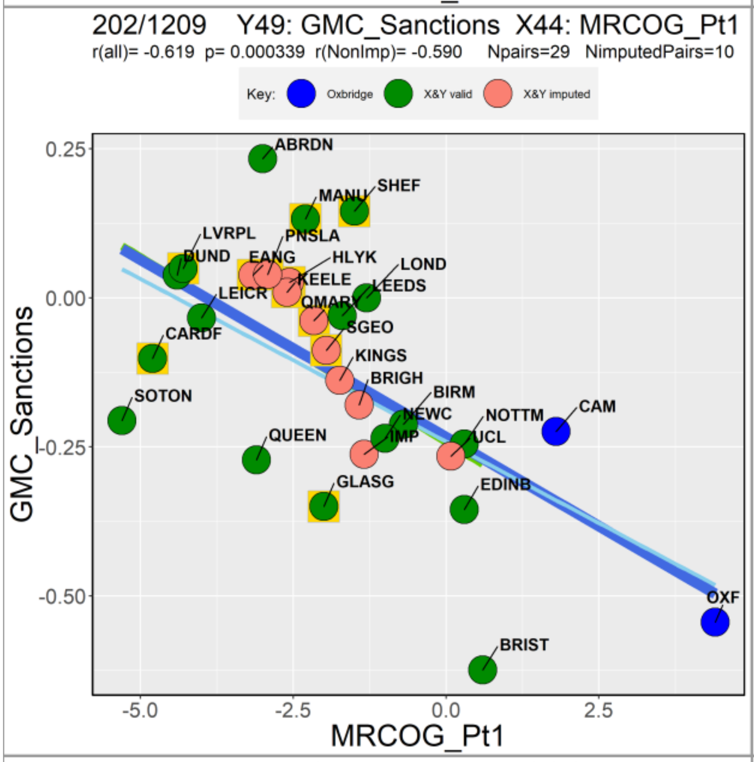
202/1209 Y49: GMC_Sanctions X44: MRCOG_Pt1
r(all)= -0.619 p= 0.000339 r(NonImp)= -0.590 Npairs=29 NimputedPairs=10
Key: Oxbridge X&Y valid X&Y imputed
GMC_Sanctions
MRCOG_Pt1

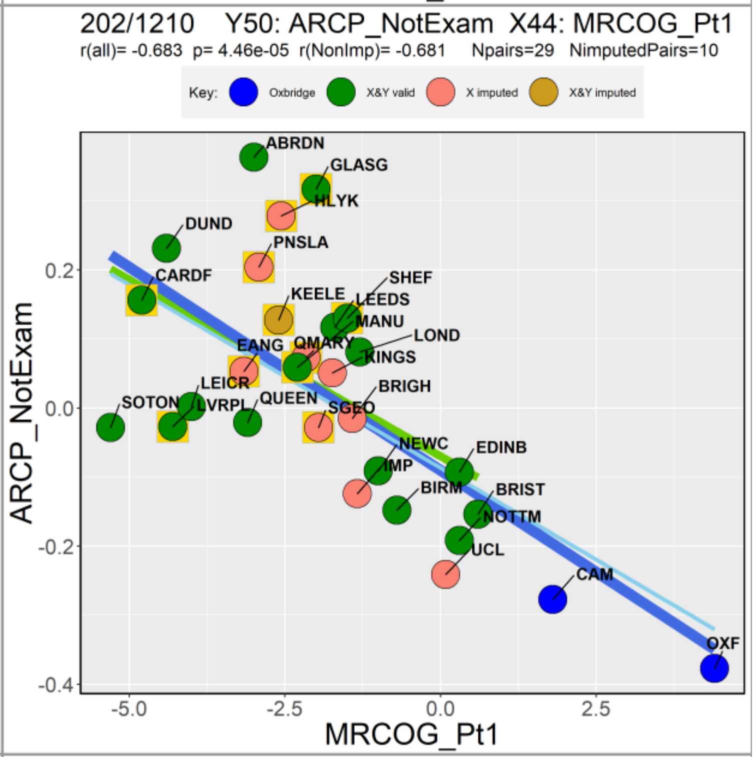
202/1210 Y50: ARCP_NotExam X44: MRCOG_Pt1
r(all)= -0.683 p= 4.46e-05 r(NonImp)= -0.681 Npairs=29 NimputedPairs=10
Key: Oxbridge X&Y valid X imputed X&Y imputed
ARCP_NotExam
MRCOG_Pt1

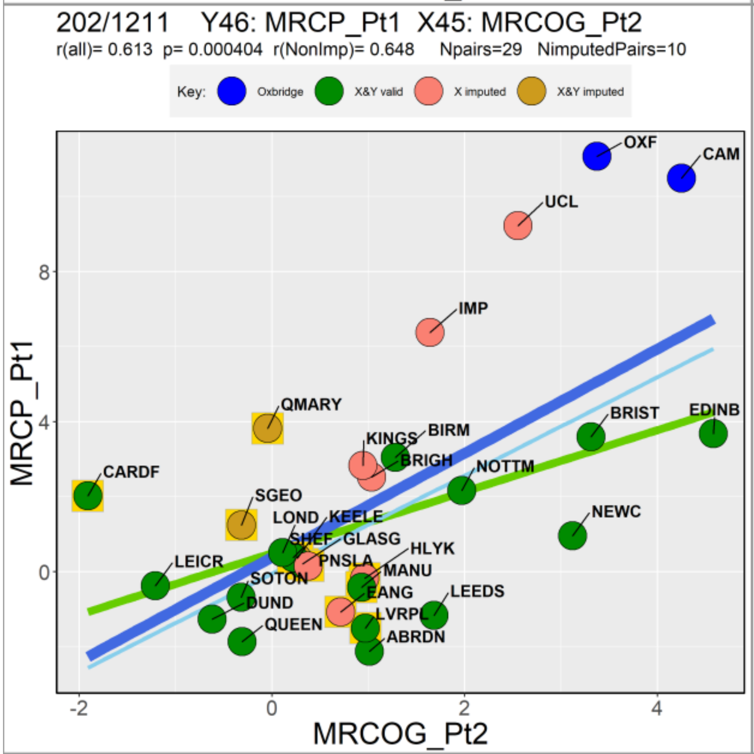
202/1211 Y46: MRCP_Pt1 X45: MRCOG_Pt2
r(all)= 0.613 p= 0.000404 r(NonImp)= 0.648 Npairs=29 NimputedPairs=10
Key: Oxbridge X&Y valid X imputed X&Y imputed
MRCP_Pt1
MRCOG_Pt2

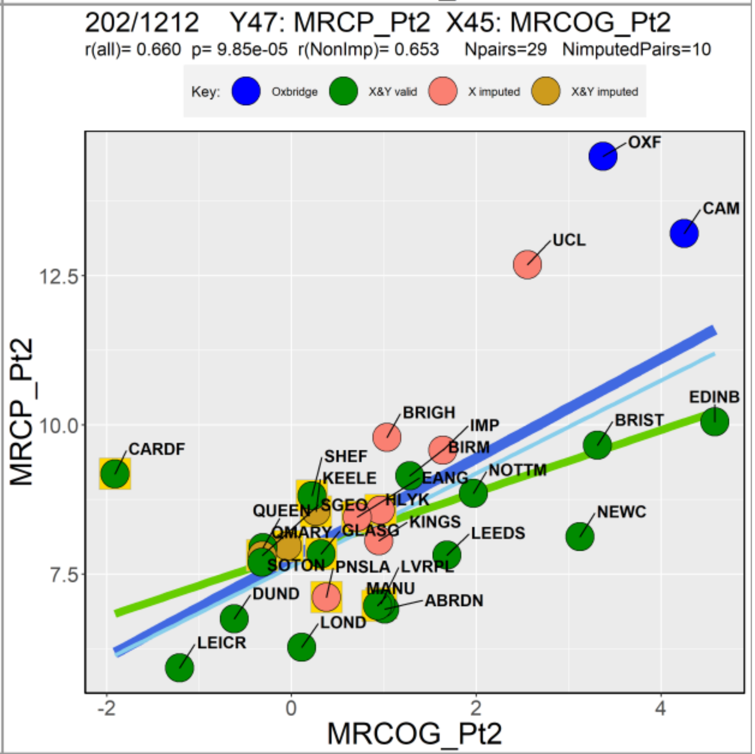
202/1212 Y47: MRCP_Pt2 X45: MRCOG_Pt2
r(all)= 0.660 p= 9.85e-05 r(NonImp)= 0.653 Npairs=29 NimputedPairs=10
Key: Oxbridge X&Y valid X imputed X&Y imputed
MRCP_Pt2
MRCOG_Pt2

203/1213 Y48: MRCP_PACES X45: MRCOG_Pt2
r(all)= 0.566 p= 0.00138 r(NonImp)= 0.554 Npairs=29 NimputedPairs=10

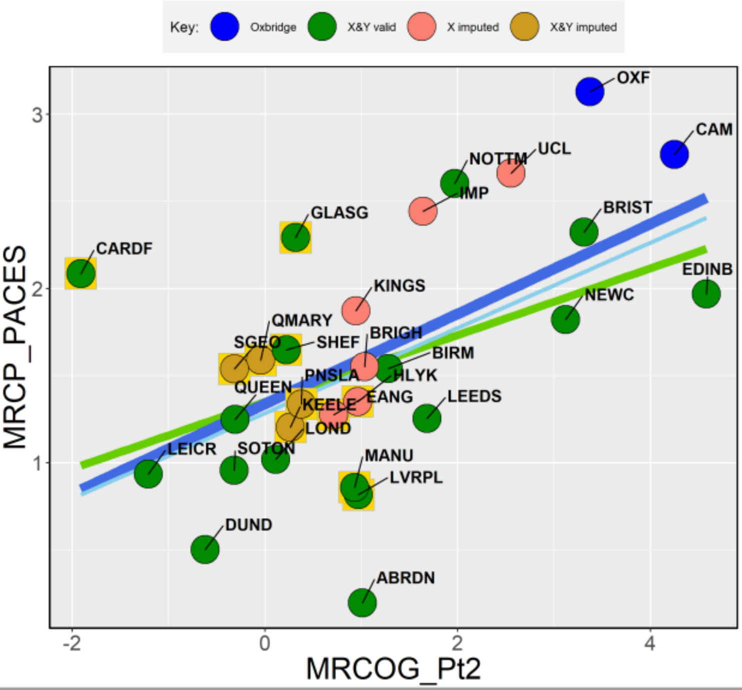

203/1214 Y49: GMC_Sanctions X45: MRCOG_Pt2
r(all)= -0.544 p= 0.00227 r(NonImp)= -0.518 Npairs=29 NimputedPairs=10

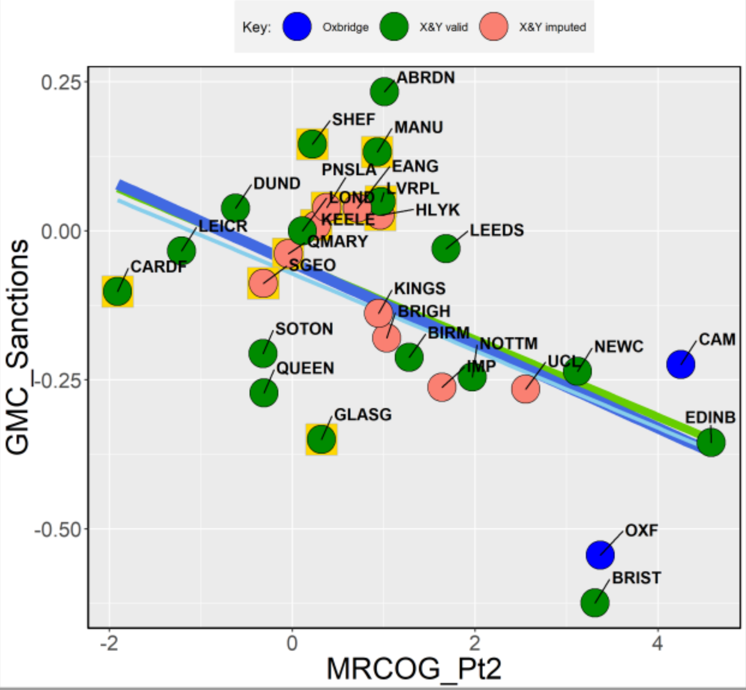

203/1215 Y50: ARCP_NotExam X45: MRCOG_Pt2
r(all)= -0.612 p= 0.000422 r(NonImp)= -0.624 Npairs=29 NimputedPairs=10

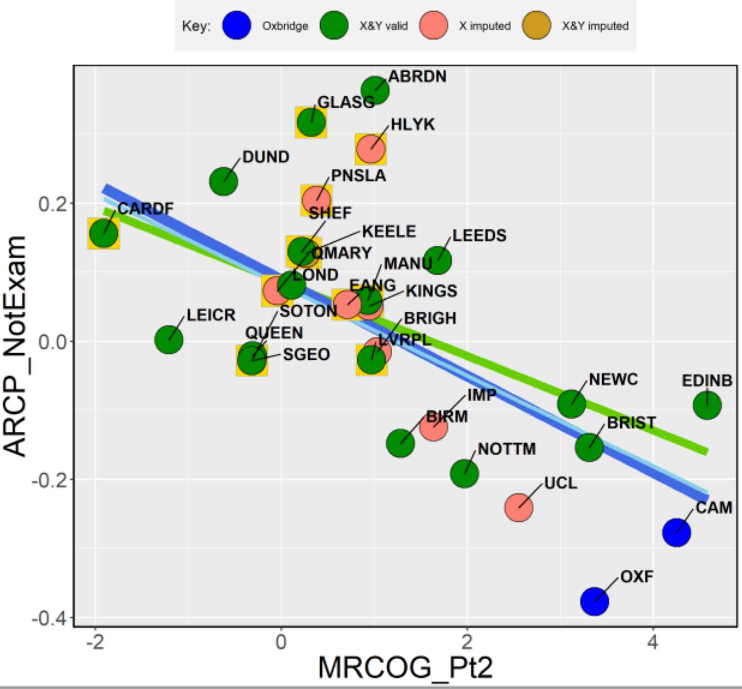

203/1216 Y47: MRCP_Pt2 X46: MRCP_Pt1
r(all)= 0.899 p= 3.38e-11 r(NonImp)= 0.917 Npairs=29 NimputedPairs=3

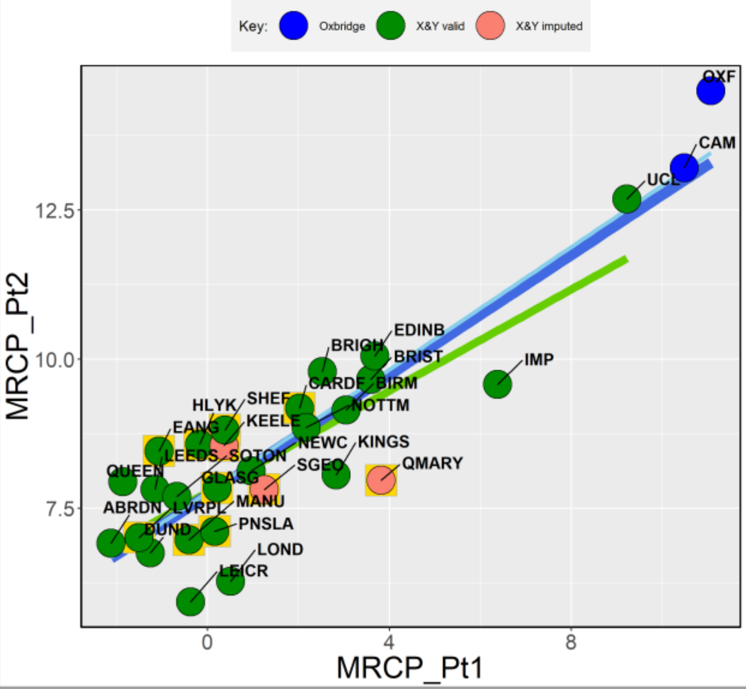

203/1217 Y48: MRCP_PACES X46: MRCP_Pt1
r(all)= 0.837 p= 1.58e-08 r(NonImp)= 0.840 Npairs=29 NimputedPairs=4

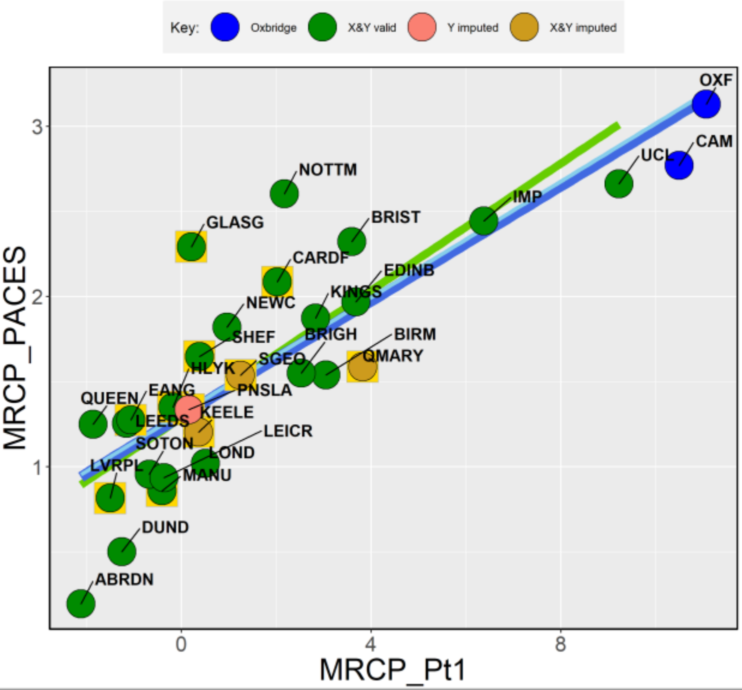

203/1218 Y49: GMC_Sanctions X46: MRCP_Pt1
r(all)= -0.606 p= 0.000492 r(NonImp)= -0.601 Npairs=29 NimputedPairs=10

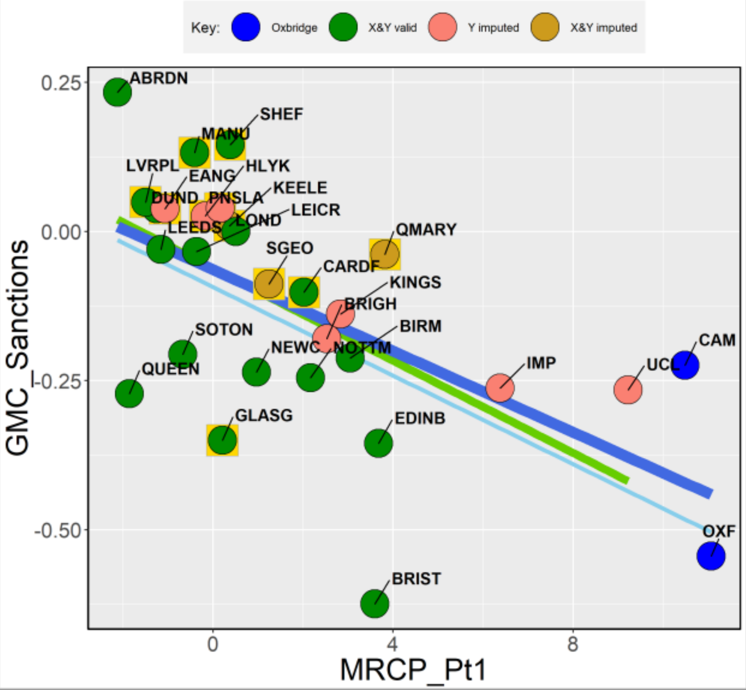

204/1219 Y50: ARCP_NotExam X46: MRCP_Pt1

r(all)= -0.743 p= 3.86e-06 r(NonImp)= -0.757 Npairs=29 NimputedPairs=3

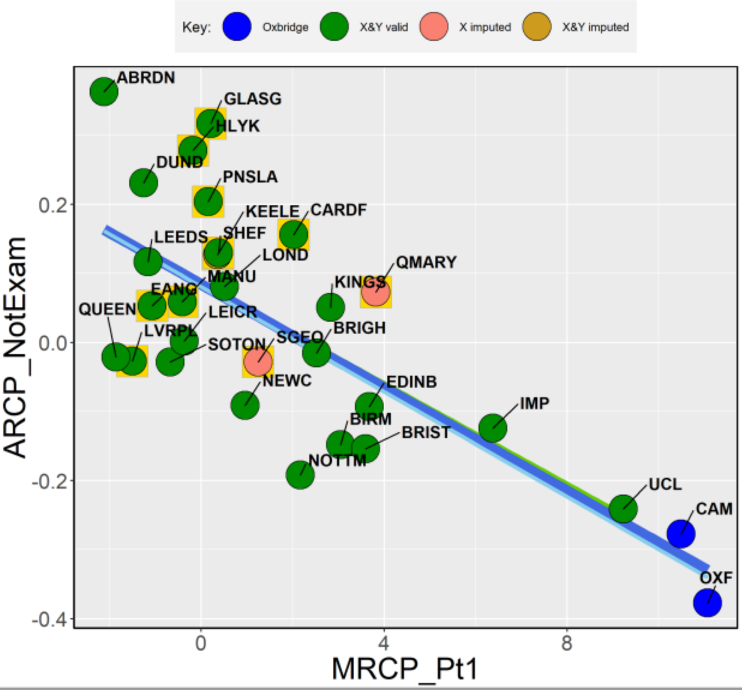

204/1220 Y48: MRCP_PACES X47: MRCP_Pt2

r(all)= 0.815 p= 7.44e-08 r(NonImp)= 0.821 Npairs=29 NimputedPairs=4

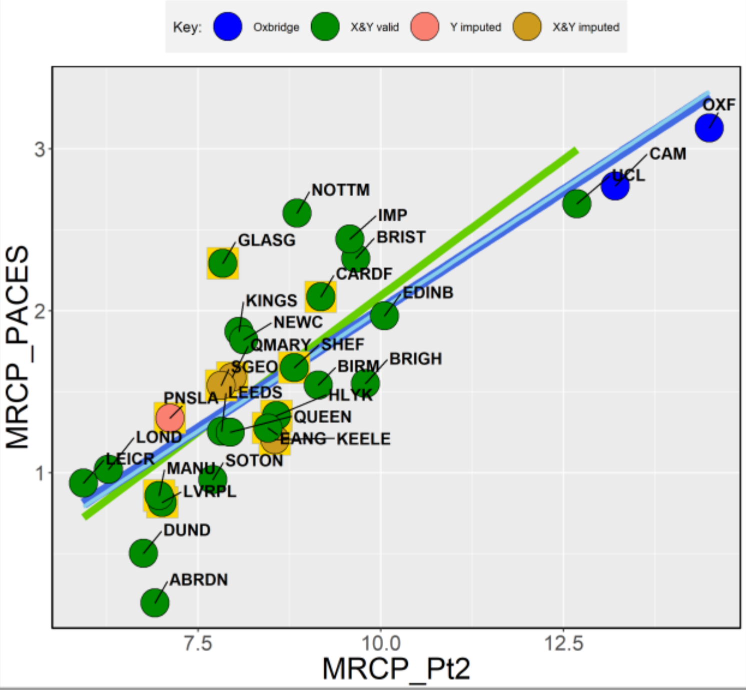

204/1221 Y49: GMC_Sanctions X47: MRCP_Pt2

r(all)= -0.614 p= 0.000394 r(NonImp)= -0.622 Npairs=29 NimputedPairs=10

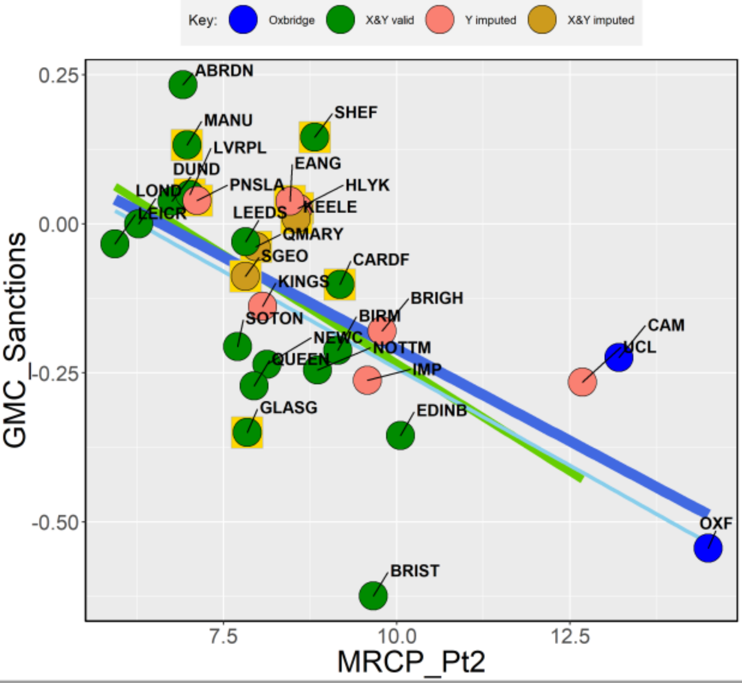

204/1222 Y50: ARCP_NotExam X47: MRCP_Pt2

r(all)= -0.700 p= 2.41e-05 r(NonImp)= -0.709 Npairs=29 NimputedPairs=3

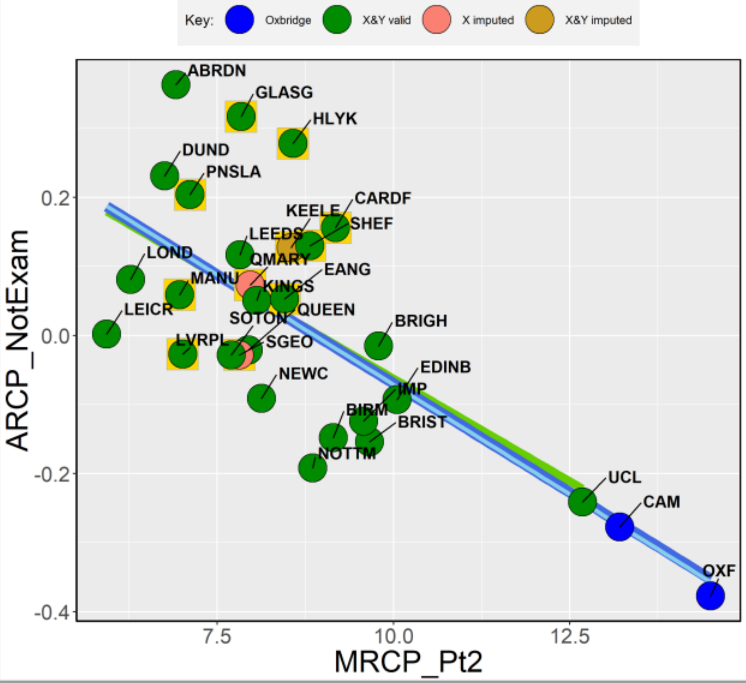

204/1223 Y49: GMC_Sanctions X48: MRCP_PACE

r(all)= -0.757 p= 1.97e-06 r(NonImp)= -0.761 Npairs=29 NimputedPairs=10

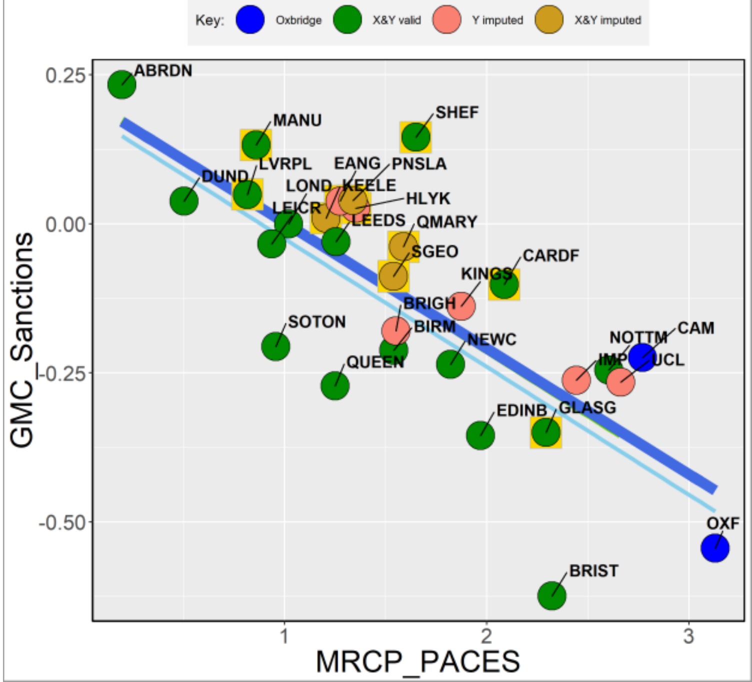

204/1224 Y50: ARCP_NotExam X48: MRCP_PACES

r(all)= -0.659 p= 1e-04 r(NonImp)= -0.657 Npairs=29 NimputedPairs=4

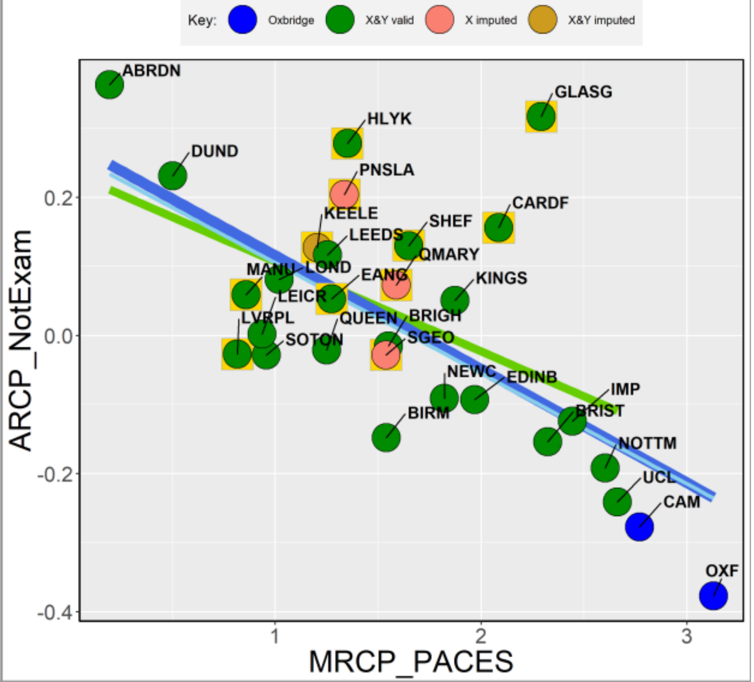

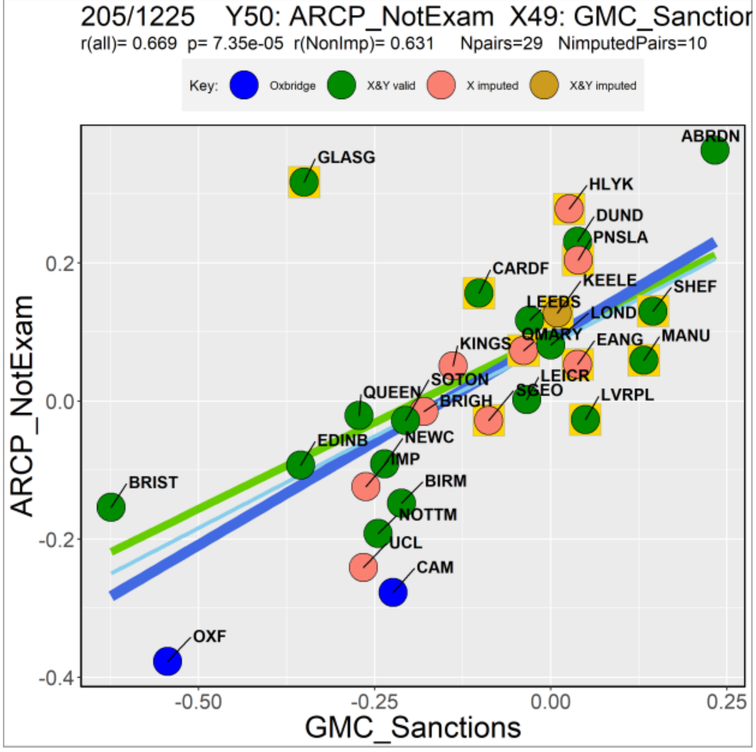

205/1225 Y50: ARCP_NotExam X49: GMC_Sanctior
r(all)= 0.669 p= 7.35e-05 r(NonImp)= 0.631 Npairs=29 NimputedPairs=10
Key: Oxbridge X&Y valid X imputed X&Y imputed
ARCP_NotExam
GMC_Sanctions
ABRDN
GLASG
HLYK
DUND
PNSLA
CARDF
KEELE
SHEF
LEEDS
LOND
QMARY
EANG
MANU
KINGS
SOTON
LEICR
QUEEN
BRIGH
SGEO
LVRPL
NEWC
EDINB
IMP
BRIST
BIRM
NOTTM
UCL
CAM
OXF
0.2
0.0
-0.2
-0.4
-0.50
-0.25
0.00
0.25
